# Exploring the potential of Huangqin Tang in breast cancer treatment using network pharmacological analysis and experimental verification

**DOI:** 10.1186/s12906-024-04523-0

**Published:** 2024-06-07

**Authors:** Ziqiao Zhao, Yongxia Zhu, Fangyi Long, Yu Ma, Qing Tang, Ting Wang

**Affiliations:** 1 School of Pharmacy, Chengdu University of Traditional Chinese Medicine, No.1166 Liutai Avenue, Chengdu, 611137 China; 2 Department of Clinical Research, Sichuan Clinical Research Center for Cancer, Sichuan Cancer Hospital & Institute, Sichuan Cancer Center, Affiliated Cancer Hospital of University of Electronic Science and Technology of China, Chengdu, 610041 China; 3 Laboratory Medicine Center, Sichuan Provincial Maternity and Child Health Care Hospital, Affiliated Women’s and Children’s Hospital of Chengdu Medical College, Chengdu Medical College, Chengdu, 610041 China; 4 School of Life Science and Engineering, Southwest Jiaotong University, Chengdu, 610031 China; 5 Department of Pharmacy, Sichuan Clinical Research Center for Cancer, Sichuan Cancer Hospital & Institute, Sichuan Cancer Center, Affiliated Cancer Hospital of University of Electronic Science and Technology of China, Chengdu, China

**Keywords:** Huangqin tang, Traditional chinese medicine, Breast cancer, Network pharmacology, Mechanism, Molecular docking

## Abstract

**Aims of this study:**

This study aims to investigate the potential of Huangqin Tang (HQT), a traditional Chinese medicine formulation, in the treatment of breast cancer (BC) through a comprehensive approach integrating network pharmacology, molecular docking, and experimental validation.

**Methods:**

Chemical composition and target information of HQT were collected using the Traditional Chinese Medicine Systems Pharmacology Database and Analysis Platform (TCMSP). Disease-related target genes were obtained from the GeneCards database. Network pharmacological analysis, including construction of compound-disease-target networks and protein-protein interaction networks, was performed. Molecular docking simulations were conducted to evaluate the binding affinity between HQT components and key targets. Experimental validation was carried out using cell viability assays, clone formation assays, flow cytometry, Western blotting, and pathway analysis.

**Results:**

A total of 210 candidate targets were identified. Network analysis revealed STAT3, AKT1, MAPK3 etc. as central targets. Enrichment analysis suggested HQT may exert anti-tumor effects through regulating lipid metabolism and inflammation related pathways. Molecular docking showed that the key compounds baicalein, wogonin, kaempferol and quercetin all bound effectively to MAPK1. The binding of baicalein to IL6 and naringenin to TNF-α was also relatively stable. The experimental results demonstrated that HQT effectively inhibited the proliferation of breast cancer cells, with IC50 values of 2.334 mg/mL and 1.749 mg/mL in MCF-7 cells at 24 h and 48 h, and IC50 values of 1.286 mg/mL and 1.496 mg/mL in MDA-MB-231 cells at 24 h and 48 h, respectively. Furthermore, HQT induced cell cycle arrest at the G2/M phase in breast cancer cells and downregulated the expression of related proteins including CDK1, Cyclin B1, CDK2, and Cyclin E. Additionally, HQT promoted apoptosis in breast cancer cells by upregulating the expression of Bak and CC-3, while downregulating the expression of Bcl-2. Notably, HQT also exhibited regulatory effects on the HIF-1 signaling pathway.

**Conclusions:**

This study provides insights into the potential multi-component and multi-target mechanisms of HQT against BC, suggesting it may achieve therapeutic effects through regulating inflammatory response and cancer-related pathways via the identified active compounds and targets. The findings highlight the importance of integrating traditional medicine with modern approaches for the development of novel cancer therapies.

**Supplementary Information:**

The online version contains supplementary material available at 10.1186/s12906-024-04523-0.

## Introduction

Breast cancer (BC) is the leading cause of cancer-related deaths among women worldwide and the most prevalent type of cancer. According to the International Agency for Research on Cancer (IARC) of the World Health Organization, the number of new BC cases in 2020 was 2.26 million, surpassing lung cancer for the first time and becoming the world’s top cancer [1]. China, in particular, faces a significant burden of BC, with approximately 420,000 new cases and nearly 120,000 deaths in 2020. Current treatment options for BC include local treatments such as surgery and radiotherapy, as well as systemic treatments like chemotherapy, endocrine therapy, biotherapy, and targeted therapy [2]. These treatment modalities have shown some efficacy. However, chemotherapy [3] and radiotherapy [4] may cause damage to normal cells, leading to side effects such as nausea, hair loss, fatigue, and immune suppression. Additionally, long-term use of certain drugs may induce drug resistance in tumor cells, reducing the effectiveness of treatment [5]. Even after completing treatment, some patients may still face the possibility of recurrence, especially in advanced stages or with distant metastasis [6]. Therefore, there is an urgent need to discover new effective treatment methods. Traditional Chinese Medicine (TCM) is often considered a gentler treatment approach, helping to alleviate side effects associated with Western medicine. Moreover, TCM emphasizes evidence-based treatment and focuses on holistic patient management, not only targeting the tumor itself but also regulating the patient’s constitution, aiming to improve their quality of life and physical resilience [7]. Therefore, we chose to explore the perspective of traditional Chinese medicine and adopt TCM therapies as adjunctive treatment modalities to alleviate patient discomfort and improve their quality of life.

Huangqin Tang (HQT) is derived from Zhang Zhongjing’s “Shang-Han-Lun” and consists of four herbs: *Scutellaria baicalensis* Georgi (Huangqin, HQ), *Glycyrrhiza uralensis* Fisch. (Gancao, GC), *Paeonia lactiflora* Pall. (Baishao, BS), *Ziziphus jujuba* Mill. (Dazao, DZ). Modern studies have demonstrated that HQT possesses antibacterial, anti-inflammatory, and analgesic properties [8]. In recent years, the anti-tumor effects of HQT have garnered attention [9–11]. Flavonoids found in HQ, such as baicalein and wogonin, exhibit therapeutic effects on BC. Baicalein notably downregulates the expression of p-AKT, p-mTOR, NF-κB, and p-IκB while enhancing IκB expression in both MCF-7 and MDA-MB-231 cells. Furthermore, it reduces the ratios of p-AKT/AKT and p-mTOR/mTOR [12]. Wogonin induces apoptosis in breast cancer cells by activating ERK and caspases, and it is associated with the inhibition of the PI3K/Akt/survivin signaling pathway in MCF-7 cells [13]. Paeoniflorin in BS inhibits the proliferation and invasion of breast cancer cells by suppressing the Notch-1 signaling pathway [14]. Additionally, glycyrrhizinic acid in GC decreases breast cancer cell activity and induces cell death by activating NADPH oxidase and inducible nitric oxide synthase, reducing glutathione and glutathione peroxidase levels, and promoting the production of reactive oxygen species and reactive nitrogen species [15]. Moreover, jujube polysaccharide has shown inhibitory effects on various cancer cells [16]. Considering these findings, we pose the question: Can HQT effectively treat BC, and if so, what are its potential mechanisms of action?

The concept of network pharmacology was first introduced by Professor Hopkins of the University of Dundee, UK in 2007 [17]. Network pharmacology surpasses the limitations of traditional single-target thinking and provides a new approach to drug discovery for complex, multi-target diseases. Its multi-component, multi-target, multi-pathway, and multi-functionality research strategy aligns with the holistic concept of evidence-based TCM treatment. Network pharmacology has gained prominence in TCM research in recent years, as it enables systematic analysis to predict and explain the effects of drugs. In this study, we aim to employ a combination of network pharmacology, molecular docking, and experimental validation to investigate the mechanisms of action of HQT in the treatment of BC. The findings will contribute to the theoretical basis for the clinical application of HQT.

## Materials and methods

### Network pharmacological analysis

#### Collection of chemical composition and target information of HQT

The chemical components of each herb in HQT were obtained through the Traditional Chinese Medicine Systems Pharmacology Database and Analysis Platform (TCMSP, https://old.tcmsp-e.com/tcmsp.php). The potential active ingredients in the herbs of HQT were identified based on drug-like (DL) and oral bioavailability (OB). OB represents the percentage of an administered oral drug dose that reaches systemic circulation and produces the drug’s intended effect. It reflects the convergence of Absorption, Distribution, Metabolism, and Excretion (ADME) processes, serving as a critical indicator of the drug-like properties of the active molecule. Thus, an OB ≥ 30% was employed as the criterion for selecting candidate active molecules within HQT. The average DL score in the DrugBank database was found to be 0.18. Hence, compounds with a DL ≥ 0.18 were chosen as candidates for further study. Target information of the active ingredients of HQT was collected from the TCMSP database and corrected using the Uniprot database (https://www.uniprot.org/).

#### Acquisition of disease targets

Disease-related target genes for BC were collected from the GeneCards database (https://www.genecards.org/) using “breast cancer” as the keyword.

#### Prediction of candidate targets of HQT during BC treatment

The HQT active ingredient targets and BC targets were imported into VENNY2.1 (https://bioinfogp.cnb.csic.es/tools/venny/) to identify overlapping targets, which represent candidate targets of HQT for the treatment of BC.

#### Construction of “Compound-Disease-Target” network

The active compounds in HQT and the potential targets were imported into Cytoscape 3.7.0 software to construct a network of “Compound-Disease-Target” interactions. The network was visualized using Cytoscape software and analyzed using Network Analyzer to identify the key components of HQT for the treatment of BC.

#### GO and KEGG pathway enrichment analyses

The potential targets were imported into the Metascape database (https://metascape.org/gp/index.html#/main/step1) for Gene Ontology (GO) and Kyoto Encyclopedia of Genes and Genomes (KEGG) pathway enrichment analysis. The analysis aimed to demonstrate the main biological processes and potential molecular mechanisms of HQT against BC.

#### Construction of PPI networks

The potential targets were imported into the STRING database (https://cn.string-db.org/) to construct a protein-protein interaction (PPI) network. The organism was set as Homo sapiens. In order to enhance the reliability of the data and minimize the occurrence of false positives, a minimum required interaction score of 0.94 was used, and disconnected nodes were hidden in the network. Cytoscape 3.7.0 software was used for network topology analysis, screening core targets, and visualize them.

#### Construction of the network of the main pathways and targets of HQT treatment of BC

Cytoscape 3.7.0 software was used to build “Pathway-target” network diagrams, facilitating the analysis of the connection between drugs and diseases.

### Molecular docking

Chemical structures of drugs were obtained from the TCMSP database in PDB format, and 3D structures of targets were downloaded from the Alphafold database (https://alphafold.ebi.ac.uk/). AutoDock Tools 1.5.7 was used for pre-processing of proteins and small molecules, including detection of torsional bonds. Molecular docking was performed, and the binding activity between the component and the target was evaluated based on the binding energy. PyMOL software was used for analysis and mapping.

### Experimental validation

#### Reagents

Fetal bovine serum (FBS, GEMINI, 900–1080) was purchased from Sichuan Longyu Technology Co. Cell Counting Kit (CCK-8, AbMole, M4839) and dulbecco’s modified eagle medium (DMEM, GIBCO, C11965500BT) were purchased from Chengdu Radiometer Biotechnology Co. Annexin V-FITC/PI Apoptosis Detection Kit (KGA512) was purchased from Sichuan Sailanbo Technology Co. SDS-PAGE Sample Loading Buffer (5X) (BL502B), BCA Protein Assay Kit (BL521A) were purchased from Beijing Labgic Technology Co., Ltd. Oriscience Supersensitive ECL Kit (723J111) was purchased from Oriscience Biotechnology Co., Ltd. Disposable cell culture dishes (230713-961BF), 15 mL centrifuge tubes (231004-6058-1AA), 50 mL centrifuge tubes (230916-4060-AA) were purchased from Sichuan Bojeri Biotechnology Co. Six-well plate (803006) and 96-well plate (803096) were purchased from Suzhou Saipu Biotechnology Co. CDK1-specific polyclonal antibody (19532-1-AP) and Cyclin B1 polyclonal antibody (28603-1-AP) were purchased from Wuhan Sanying Biotechnology Co. Ltd.Bcl-2 (D17C4) Rabbit mAb (3498 S), Bak (D4E4) Rabbit mAb (12105T), Cleaved Caspase-3 (Asp175) Antibody (CC-3, 9661 S) from Cell Signaling Technology.

#### Preparation of HQT extract

The four herbs required for the preparation of HQT were purchased from Sichuan Fuxitang Pharmaceutical Co Ltd (Sichuan, China). In accordance with the formulation described in the “Shang-Han-Lun” and subsequent studies on the prescription, the ratio of HQ: BS: GC: DZ is 3:2:2:2, with water serving as the solvent. The herbs were soaked in pure water at a ratio of 1:10 times for 30 min. They were then boiled for 1 h, and the liquid was poured out. Next, water was added at a ratio of 1:8 and boiled for 40 min, after which the liquid was poured out again. The two aqueous extracts were mixed and filtered through gauze. The supernatant was concentrated to 50 mL using a rotary evaporator. The supernatant was then frozen at −80 °C overnight. The freeze-dried product was obtained using a freeze-dryer, yielding 18.78 g of lyophilized powder. For the in vitro experiments, the HQT powder was dissolved in cell culture medium at the desired concentrations.

#### Cell culture

The MCF-7 and MDA-MB-231 human breast cancer cell lines were purchased from ATCC and cultured in DMEM medium supplemented with 10% fetal bovine serum, 1% penicillin, and 1% streptomycin. The cells were incubated at 37 °C in a humidified atmosphere of 5% CO_2_.

#### CCK-8 assay

Logarithmically grown MCF-7 and MDA-MB-231 cells were collected and seeded into 96-well plates at a density of 4 × 10^4^ cells/mL. After cell attachment, various concentrations of HQT solution (0.5, 1, 2, 3, 4, 6, and 8 mg/mL) were added with three replicate wells per group. After incubation for 24 h and 48 h, respectively, the 96-well plates were removed, and CCK-8 solution was added to each well. The incubation was continued at 37 °C for 3–4 h. The plates were then incubated at 37 °C for 3–4 hours. Following incubation, the absorbance was measured at 490 nm using an enzyme marker. The optimal intervention time and concentration of HQT for subsequent experiments were determined based on cell viability.

#### Clone formation

Logarithmically grown MCF-7 and MDA-MB-231 cells were collected and seeded into six-well plates at a density of 300 cells per well. After cell attachment, different concentrations of HQT solution (0.25, 0.5, 1, 1.5, and 2 mg/mL) were added to the treatment groups, and the solution was changed every three days. After approximately 10–14 days, clones were formed. The supernatant was discarded, and the cells were washed twice with phosphate-buffered saline (PBS). The cells were fixed with 75% ethanol, stained with crystal violet for 10–15 min, and then rinsed twice with PBS. Photographs of the clones were taken, and the clone formation rate was calculated.

#### Flow cytometry

Logarithmically grown MCF-7 and MDA-MB-231 cells were seeded into six-well plates. After cell attachment, the treatment groups were treated with different concentrations of HQT solution (0.5, 1, 1.5, and 2 mg/mL) for 48 h. The cells were then collected by trypsin digestion. A portion of the cells was stained for apoptosis using the Annexin V-FITC/PI kit according to the instructions. Another portion of cells was fixed with ethanol overnight and stained with propidium iodide (PI) to analyze the cell cycle. The results were analyzed using a flow cytometer.

#### Western blot

Logarithmically grown MCF-7 and MDA-MB-231 cells were seeded into six-well plates. After cell attachment, the treatment groups were treated with different concentrations of HQT solution (0.5, 1, 1.5, and 2 mg/mL) for 48 h. The cells were then collected, lysed with RIPA buffer using sonication, and the total protein was extracted. The protein concentration was determined using a BCA kit. Protein samples were mixed with 5× loading buffer at a 1:4 ratio and denatured at 100 °C for 10 min. The proteins were separated using SDS-PAGE, transferred to PVDF membranes, and blocked with 5% skimmed milk. The membranes were incubated overnight at 4 °C with primary antibodies. After washing with TBST, the membranes were incubated with corresponding secondary antibodies for 1 h. Enhanced chemiluminescence (ECL) reagent was used to visualize the protein bands. The band density was measured and quantified using an image analysis system and ImageJ software.

### Statistical analysis

All data were statistically analyzed using GraphPad Prism 9 software. The results were expressed as mean ± standard deviation (x ± s). The t-test was used to compare the means between two groups, the rank sum test was used for rank sum data, and ANOVA was used to compare the means of multiple groups. A p-value of less than 0.05 was considered statistically significant.

## Results

### Network pharmacological analysis

#### Collection of HQT chemical composition

After screening the active ingredients of HQT in the TCMSP database with OB ≥ 30% and DL ≥ 0.18, a total of 170 active ingredients were obtained. Among them, 36 belonged to HQ, 92 belonged to GC, 13 belonged to BS, and 29 belonged to DZ. The specific ingredients of the drug are detailed in the supplemental document.

#### Collection of compound targets and disease-related genes

**Fig. 1:**
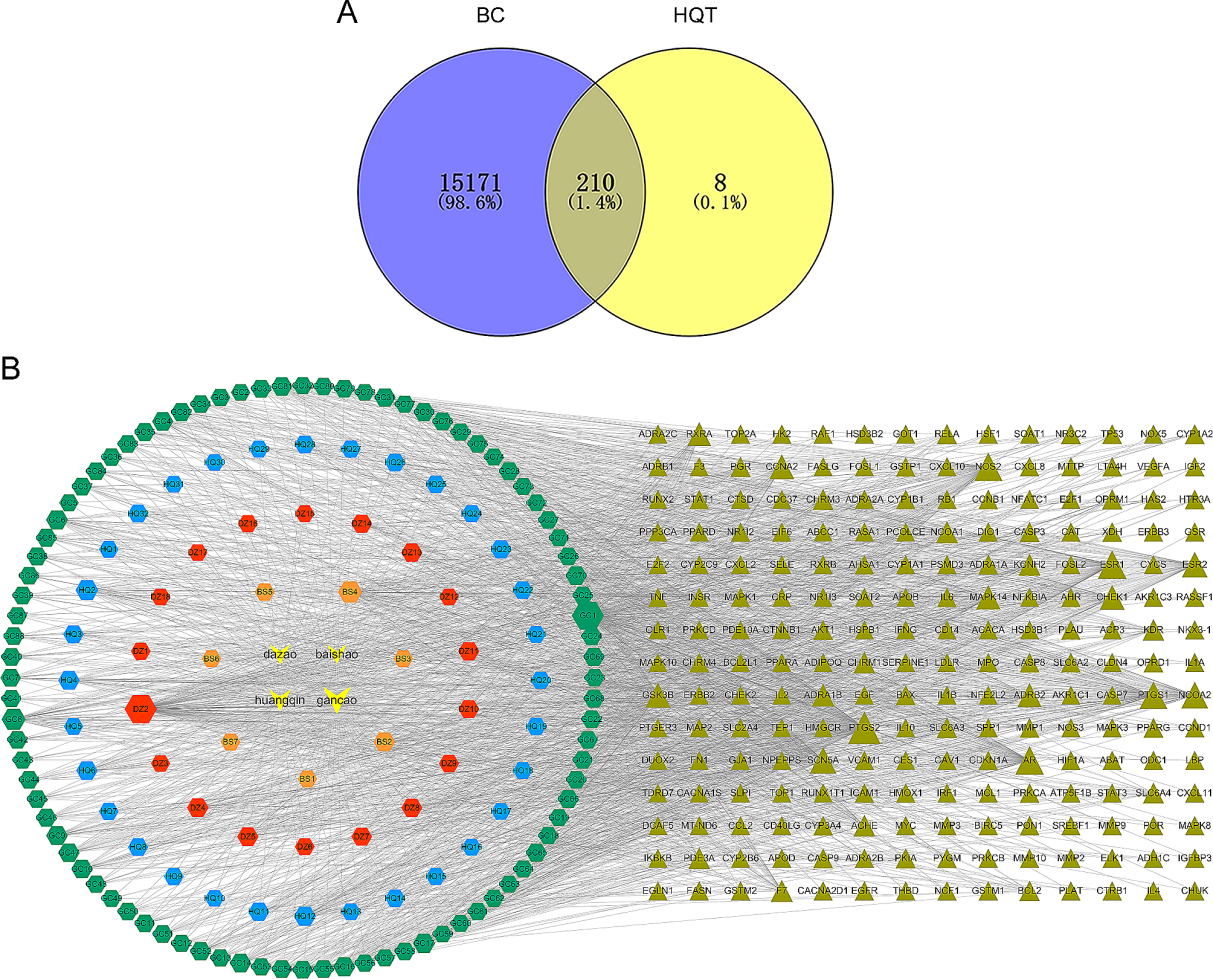
(**A**) Venn diagram illustrates the overlap between the active ingredients of Huangqin Tang (HQT) and the common targets associated with breast cancer (BC). (**B**) Compound-Disease-Target network, where grass green triangles represent the shared targets between the drug and the disease. Additionally, the orange, red, blue, and green hexagons indicate the active ingredients related to BS, DZ, HQ, and GC, respectively

We collected the targets of 170 active compounds in HQT from the TCMSP database. After integrating UniProt database entries and removing duplicates, we obtained 1869 targets. Additionally, we screened 15,381 target genes associated with BC from the GeneCards database. By comparing the active compound targets and disease targets, we identified 210 overlapping targets, which were considered the core genes for further analysis (Fig. 1A).

#### Analysis of “Compound-Disease-Target” network

To understand the main components through which HQT treats BC, we visualized an active Compound-Disease-Target network using Cytoscape 3.7.0 (Fig. 1B). The network consisted of 349 nodes and 1960 edges. We identified the top 10 active ingredients based on their degree values, including quercetin, kaempferol, wogonin, naringenin, and others (Table 1). These compounds were considered potential bioactive compounds for HQT against BC.

**Table 1:** The top 10 active ingredients identified in the “Compound-Disease-Target” network

TCMSP ID	Molecule name	OB (%)	DL	Degree	Source
MOL000098	quercetin	46.43	0.28	123	DZ, GC,
MOL000422	kaempferol	41.88	0.24	47	BS, GC,
MOL000173	wogonin	30.68	0.23	35	HQ
MOL004328	naringenin	59.29	0.21	29	GC
MOL002714	baicalein	33.52	0.21	28	HQ
MOL000392	formononetin	69.67	0.21	27	GC,
MOL003896	7-Methoxy-2-methyl isoflavone	42.56	0.20	27	GC
MOL000497	licochalcone a	40.79	0.29	25	GC
MOL000358	beta-sitosterol	36.91	0.75	24	BS, DZ, HQ
MOL004978	2-[(3R)-8,8-dimethyl-3,4-dihydro-2 H-pyrano[6,5-f]chromen-3-yl]-5-methoxyphenol	36.21	0.52	23	GC

#### GO and KEGG enrichment analysis

**Fig. 2:**
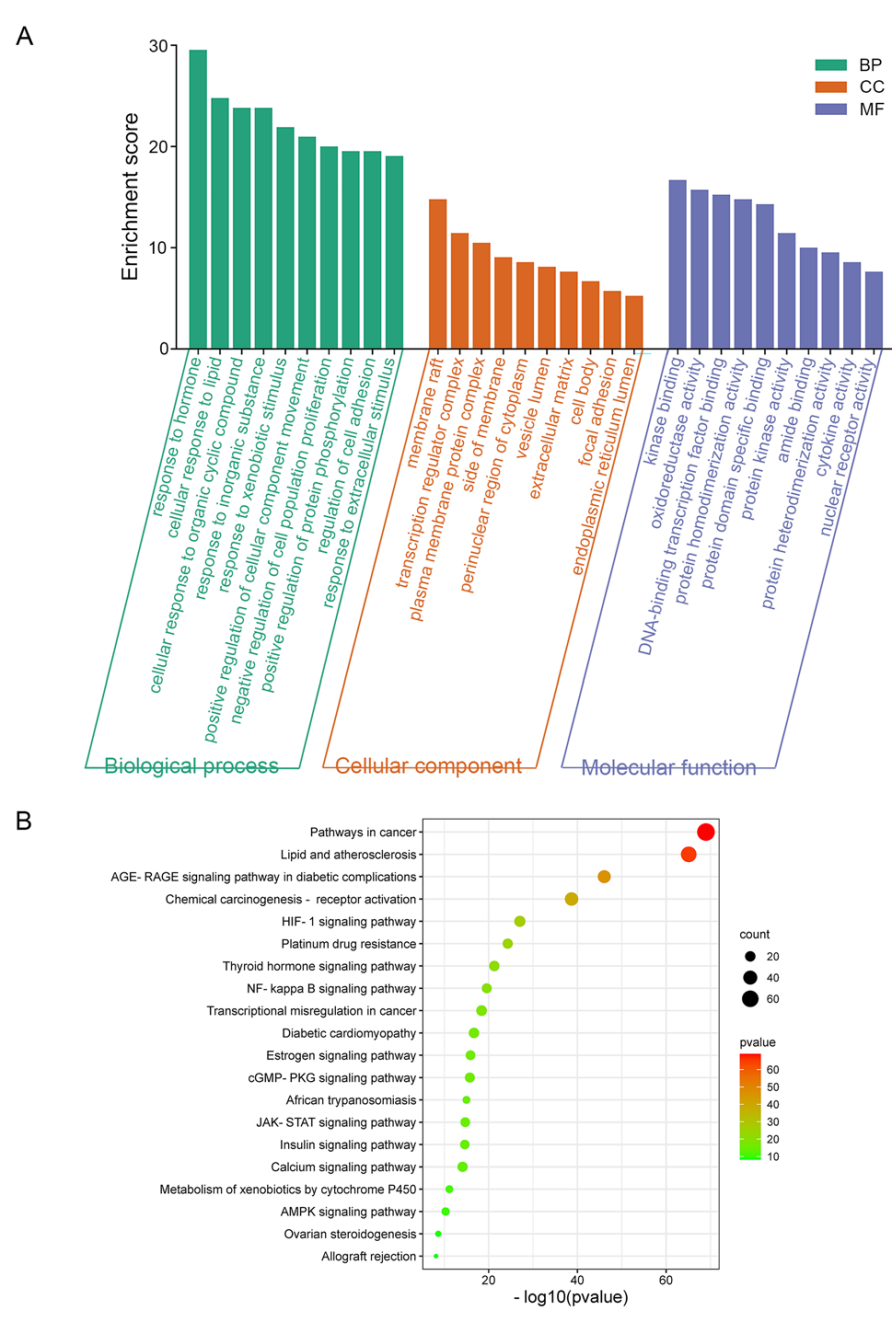
The results of functional analysis of common targets between HQT and breast cancer. (**A**) shows the Gene Ontology (GO) functional analysis, highlighting the enriched biological processes, cellular components, and molecular functions associated with the common targets. (**B**) presents the Kyoto Encyclopedia of Genes and Genomes (KEGG) pathway enrichment analysis, revealing the pathways that are significantly enriched among the common targets

To uncover the functions of HQT in treating BC, we performed GO and KEGG enrichment analyses on the common targets. The top 10 significantly enriched terms in the biological process (BP), cellular component (CC), and molecular function (MF) categories are displayed (Fig. 2A). The results showed that the main BP terms included response to lipopolysaccharide, response to bacterial origin molecules, response to oxidative stress, reactive oxygen species metabolic process, and response to hypoxia. The main CC terms included membrane raft, membrane microdomain, caveola, vesicle lumen, and presynaptic membrane. The main MF terms included nuclear receptor activity, ligand-activated transcription factor activity, RNA polymerase II-specific DNA-binding transcription factor binding, heme binding, and cytokine receptor binding.

Additionally, we conducted KEGG pathway enrichment analysis on the 210 common targets. A total of 263 pathways were identified, and the top 10 signaling pathways were selected based on count value and p-value for visualization (Fig. 2B). The results showed that the targets were mainly enriched in pathways such as lipid and atherosclerosis, AGE-RAGE signaling pathway in diabetic complications, hepatitis B, prostate cancer, and IL-17 signaling pathway.

#### PPI network analysis

**Fig. 3:**
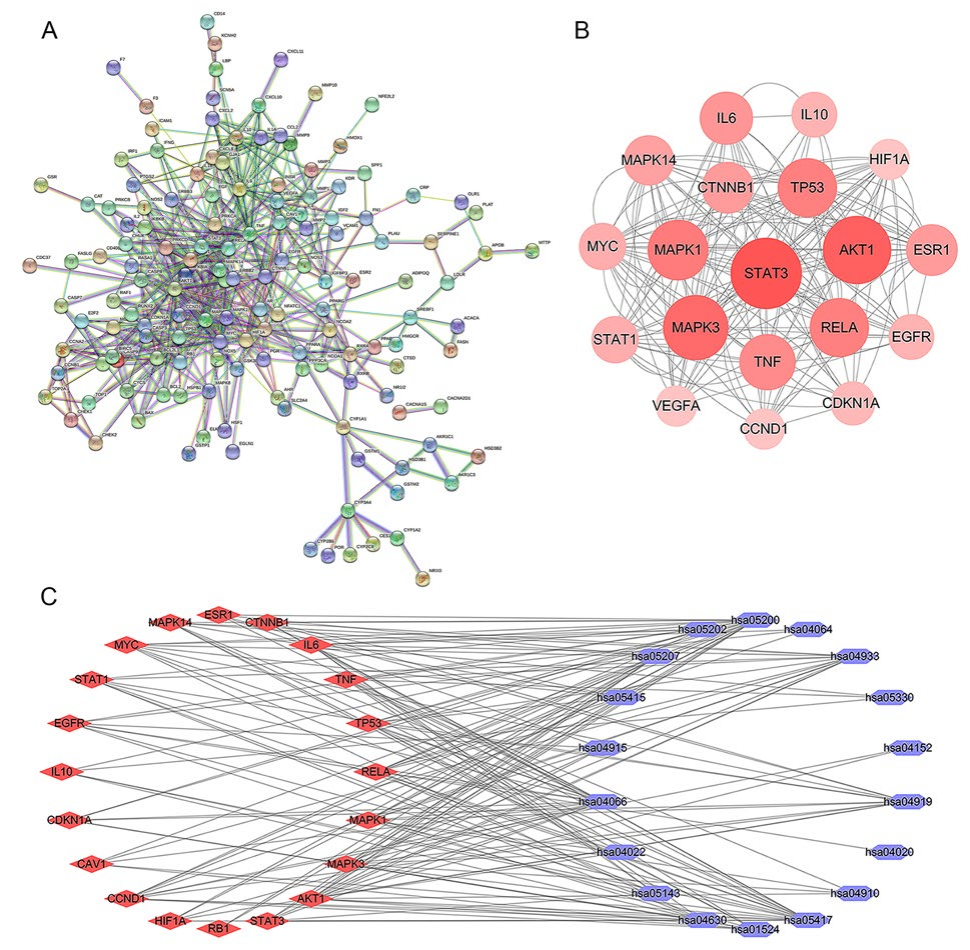
The protein-protein interaction (PPI) networks and the visualization of core targets associated with drug-disease common targets. (**A**) The PPI network of drug-disease common targets is presented, highlighting the interactions between proteins. (**B**) A visual representation of the core targets within the PPI network. (**C**) The top 20 pathways that were analyzed for KEGG enrichment and the top 20 targets from the PPI network, resulting in the Pathway-Target network

Using the STRING database, we constructed a PPI network (Fig. 3A). The network was analyzed and ranked based on degree value (Fig. 3B). The top 10 core targets were identified as STAT3, AKT1, MAPK3, MAPK1, RELA, TP53, TNF, IL6, ESR1, and CTNNB1. These targets could be considered the major action targets of HQT in BC treatment, indicating that HQT acts through multiple potential targets.

#### “Pathway-Target” network analysis

We performed a network analysis between the top 20 pathways from KEGG enrichment analysis and the top 20 targets from the PPI network (Fig. 3C). The results showed that the targets were mainly enriched in “Pathways in cancer”, followed by the “HIF-1 signaling pathway”. This suggests that HQT may exert its anti-breast cancer effect through the HIF-1 signaling pathway, providing a reference for further experiments (Table 2).

**Table 2:** The results of the “Pathway-Target” network analysis

Pathway	Degree
hsa05200: Pathways in cancer	20
hsa04066: HIF-1 signaling pathway	18
hsa05417: Lipid and atherosclerosis	17
hsa04933: AGE-RAGE signaling pathway in diabetic complications	13
hsa05207: Chemical carcinogenesis - receptor activation	12
hsa04919: Thyroid hormone signaling pathway	11
hsa01524: Platinum drug resistance	9
hsa04630: JAK-STAT signaling pathway	9
hsa05415: Diabetic cardiomyopathy	7
hsa05143: African trypanosomiasis	6

### Molecular docking

**Fig. 4:**
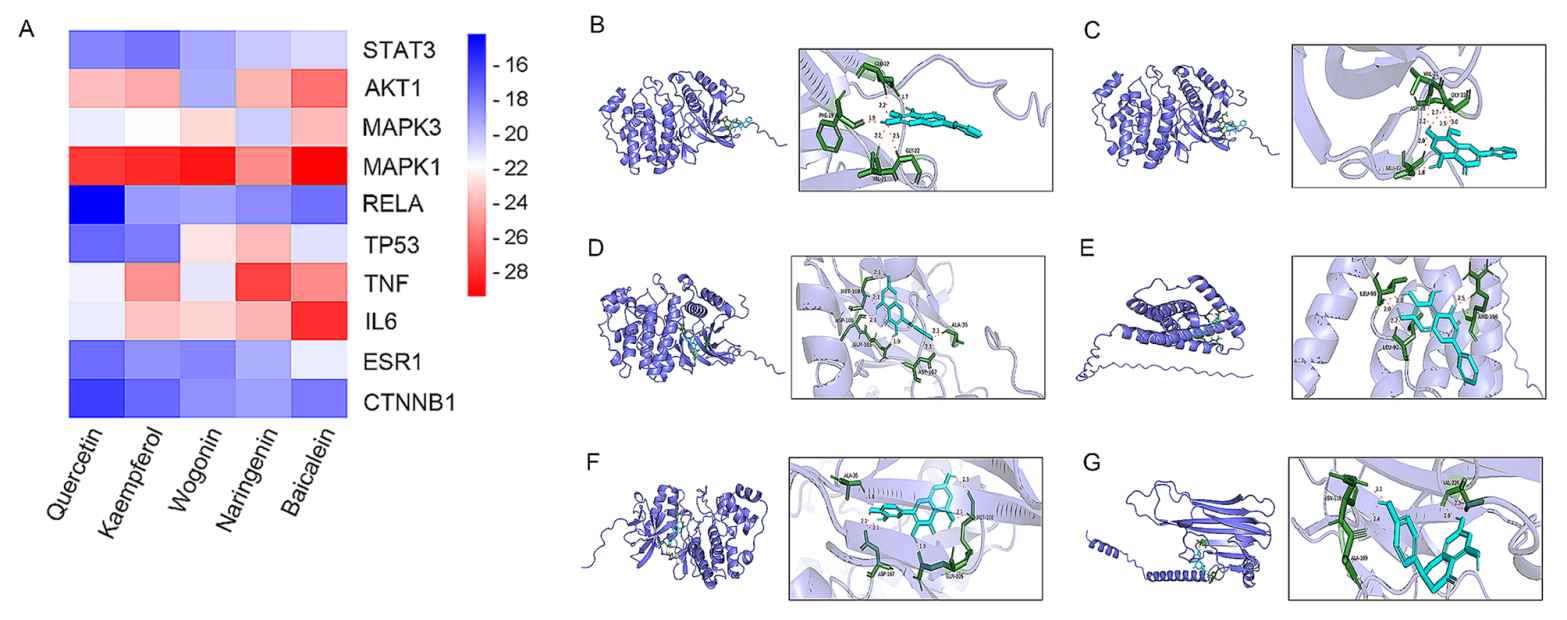
The results of molecular docking analysis of key components to the core target, along with the calculation of binding energy. (**A**) The binding energy is color-coded, with lower values appearing more red. (**B**) to (**G**) provide detailed visualizations of the binding patterns between small molecules and target proteins. On the left side of each set, the overall binding pattern is depicted, with the small molecule shown in cyan and the target protein in purple. On the right side, a zoomed-in view highlights the specific interactions, with hydrogen bonds represented by dotted lines and residue names indicated. Specifically, (**B**) illustrates the docking mode of baicalein and MAPK1, (**C**) shows the docking mode of wogonin and MAPK1, (**D**) presents the docking mode of kaempferol and MAPK1, (**E**) displays the docking mode of baicalein and IL6, (**F**) showcases the docking mode of quercetin and MAPK1, and (**G**) demonstrates the docking mode of naringenin and TNF-α.

In this study, the top 10 core targets were subjected to molecular docking with key compounds such as quercetin, kaempferol, wogonin, naringenin, and baicalein (Fig. 4A) (Table 3). The binding energy was calculated to assess the complementarity between the compound and the protein, with lower binding energy indicating higher stability. Among them, the binding energies of baicalein, wogonin, kaempferol, and quercetin with MAPK1 were − 29.5 kJ/mol, −28.7 kJ/mol, −28.1 kJ/mol, and − 27.7 kJ/mol, respectively (Fig. 4B–F). The binding energy of baicalein with IL6 was − 28.0 kJ/mol (Fig. 4E), and the binding energy of naringenin with TNF-α was − 27.4 kJ/mol (Fig. 4G). These results indicate that the components can bind to the active sites of the targets. We selected the best combinations with the highest stability and visualized them, representing small molecules and proteins with better binding abilities.

**Table 3:** The binding energy values obtained from molecular docking analysis

Targets\Binding Energy (kJ/mol)	Compound				
	quercetin	kaempferol	wogonin	naringenin	baicalein
STAT3	−18.37	−17.91	−19.33	−20.21	−20.71
AKT1	−23.72	−23.72	−19.46	−24.10	−26.11
MAPK3	−21.25	−21.92	−23.01	−20.46	−23.89
MAPK1	−27.66	−28.12	−28.74	−25.23	−29.46
RELA	−14.27	−18.83	−19.25	−18.45	−17.74
TP53	−17.32	−17.95	−22.72	−23.89	−20.96
TNF	−21.55	−25.06	−21.21	−27.45	−25.27
IL6	−21.30	−23.60	−23.22	−24.10	−28.03
ESR1	−17.53	−18.54	−18.33	−19.50	−21.25
CTNNB1	−16.15	−17.45	−18.62	−19.00	−17.95

### Experimental validation

#### Inhibition of proliferation in human breast cancer cells by HQT

**Fig. 5:**
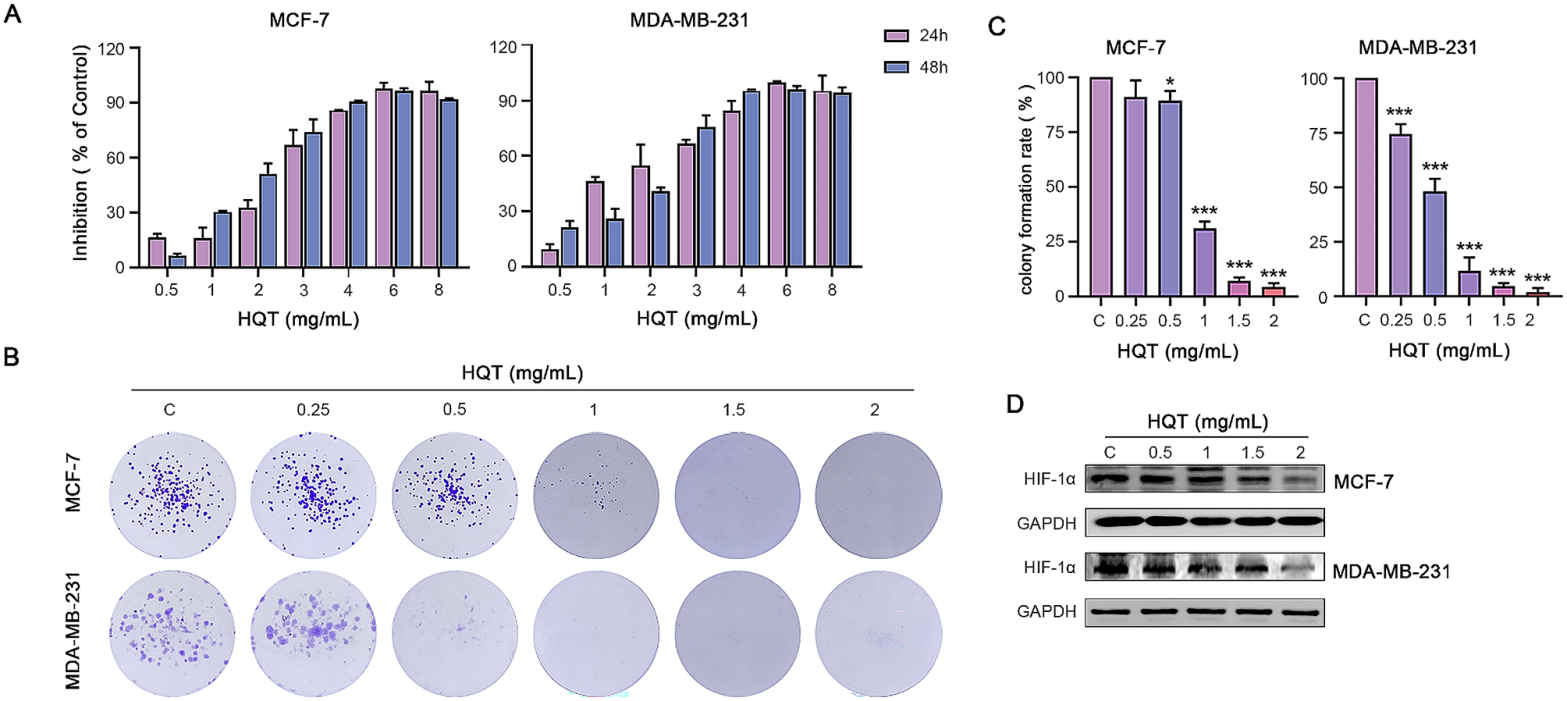
The inhibitory effects of HQT on the proliferation of human breast cancer cells. (**A**) The cell viability of breast cancer cells treated with various concentrations of HQT for 24 and 48 h is assessed using the CCK-8 assay. (**B**) The inhibitory effects of different HQT concentrations on the clone formation ability of breast cancer cells, while the corresponding clone formation rate is presented in (**C**). (**D**) The expression of HIF-1α protein in breast cancer cells treated with two different types of drugs

To assess the impact of HQT on human breast cancer cells, we conducted a cell viability assay using the CCK-8 method on MCF-7 and MDA-MB-231 cells treated with various concentrations of HQT (Fig. 5A). The results demonstrated a dose-dependent inhibition of breast cancer cell proliferation by HQT. The IC50 values for MCF-7 cells were 2.334 mg/mL at 24 h and 1.749 mg/mL at 48 h, while the IC50 values for MDA-MB-231 cells were 1.286 mg/mL at 24 h and 1.496 mg/mL at 48 h. Furthermore, HQT exhibited a significant inhibitory effect on the clonogenic ability of individual breast cancer cells in a dose-dependent manner (Fig. 5B-C).

#### Induction of cell cycle arrest in human breast cancer cells by HQT

**Fig. 6:**
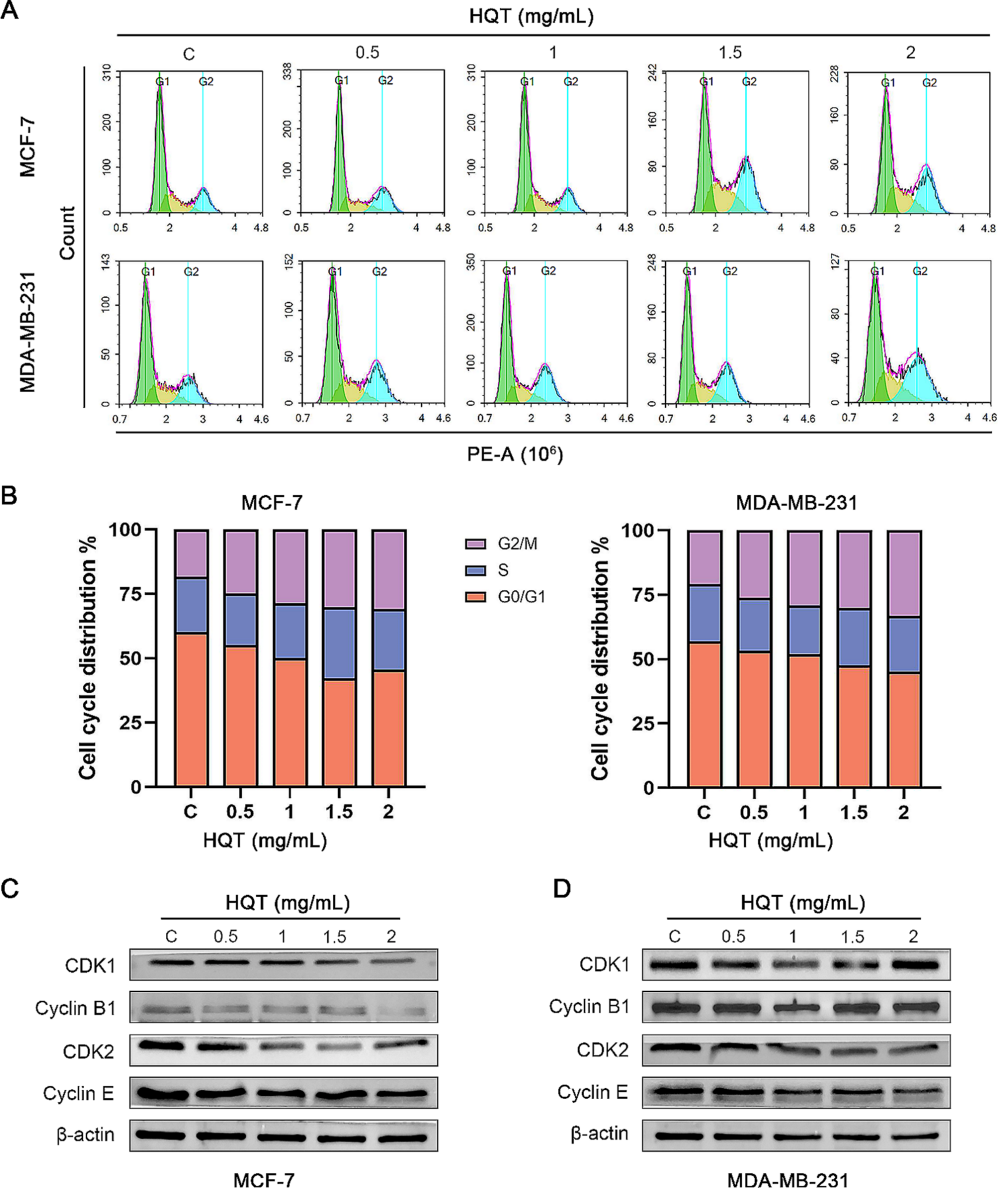
The effect of HQT on the cell cycle of breast cancer cells. (**A**) and (**B**) depict the cell cycle analysis using flow cytometry with the propidium iodide (PI) method. (**C**) and (**D**) present the protein expression levels of G2/M phase-associated proteins, including CDK1, Cyclin B1, CDK2, and Cyclin E

Using flow cytometry, we analyzed the cell cycle of breast cancer cells stained with PI (Fig. 6A-B). Additionally, we examined the expression of cell cycle-related proteins through Western blot analysis (Fig. 6C-D). The results revealed that HQT induced cell cycle arrest in breast cancer cells, specifically arresting them in the G2/M phase. After treating MCF-7 and MDA-MB-231 cells with different concentrations of HQT for 48 h, the expression levels of CDK1, Cyclin B1, CDK2, and Cyclin E were reduced compared to the control group. These findings indicated that HQT induces cell cycle arrest in BC.

#### Promotion of apoptosis in human breast cancer cells by HQT

**Fig. 7:**
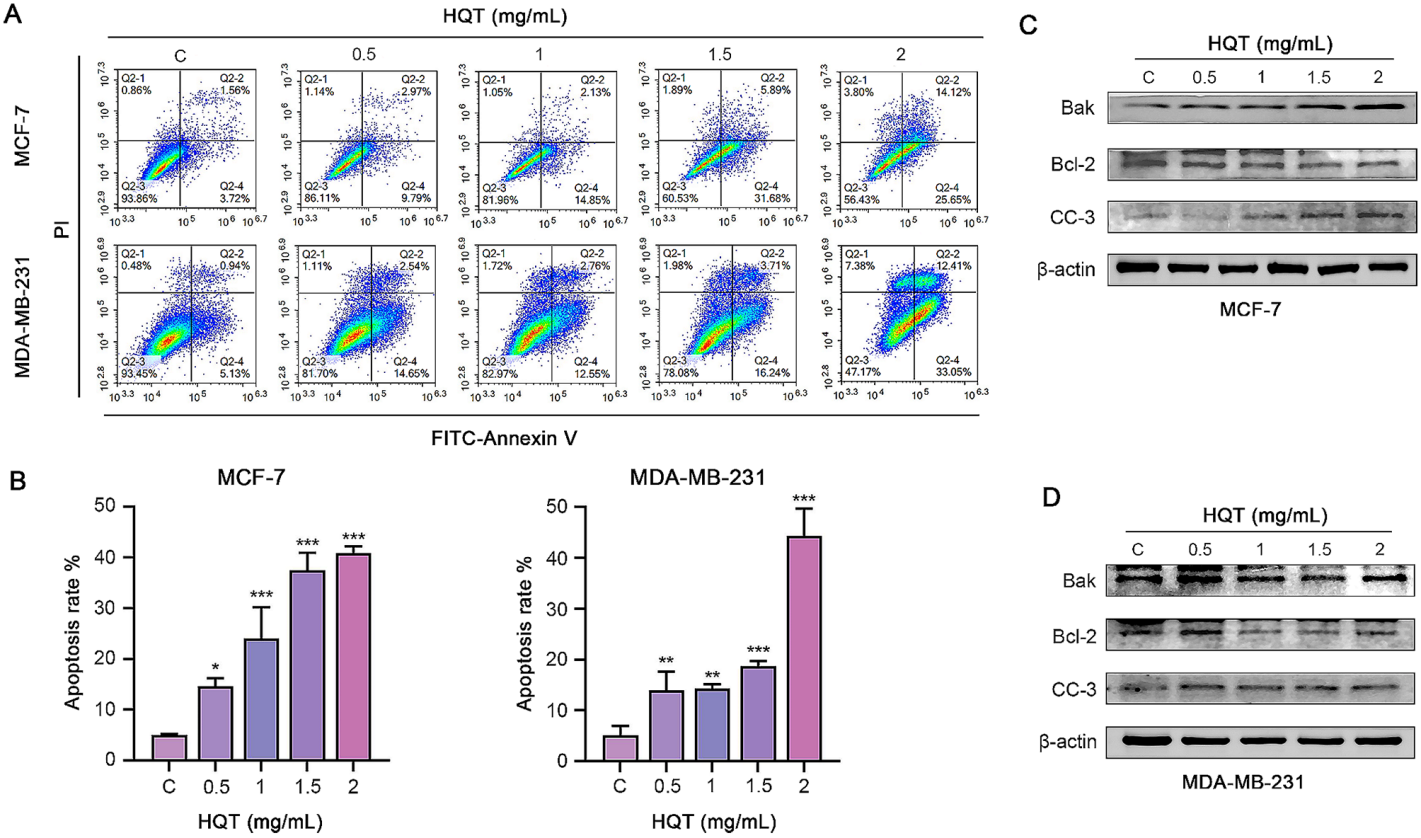
The effect of HQT on apoptosis in breast cancer cells. (**A**) and (**B**) show the detection of apoptosis rate using flow cytometry with the Annexin V-FITC/PI method. (**C**) and (**D**) exhibit the protein expression levels of apoptosis-related proteins, including Bak, Bcl-2, CC-3, and β-actin

Apoptosis was assessed using flow cytometry after staining breast cancer cells with Annexin V-FITC/PI (Fig. 7A-B). Additionally, the expression of apoptosis-related proteins was examined through Western blot analysis (Fig. 7C-D). The results demonstrated that HQT treatment at different concentrations for 48 h significantly promoted apoptosis in MCF-7 and MDA-MB-231 cells compared to the control group. Moreover, the expression levels of Bak and CC-3 proteins were significantly increased, while the expression level of Bcl-2 was decreased. These results indicated that HQT can promote apoptosis in breast cancer cells.

#### Anti-breast cancer effects of HQT through the HIF-1 signaling pathway

The expression of HIF-1α in the HIF-1 signaling pathway was evaluated using Western blot analysis (Fig. 5D). The results showed that treatment of MDA-MB-231 and MCF-7 cells with different concentrations of HQT for 48 h significantly reduced the expression of HIF-1α protein compared to the control group. This suggests that HQT exerts its anti-breast cancer effects, at least in part, through the modulation of the HIF-1 signaling pathway.

## Discussion

The potential mechanisms of HQT in treating BC were investigated in this study using a network pharmacology-based approach. The analysis identified several active compounds and candidate targets of HQT against BC, revealing a multi-targeted approach consistent with the holistic principles of TCM. The network and enrichment analysis results suggested that HQT may achieve its anti-tumor effects by modulating multiple biological processes and signaling pathways, including those related to lipid metabolism, inflammation, and hypoxia. Compounds such as quercetin, kaempferol, and baicalein were found to have good binding abilities with central targets like STAT3, AKT1, and MAPK1/3, and may contribute to the effects of HQT. Studies have shown that quercetin can enhance the sensitivity of cancer cells to cell drug resistance by inhibiting PI3K/AKT/mTOR and MAPK signaling pathways [18]. Molecular docking results provided further evidence of the binding interactions between active compounds and targets, supporting the network analysis predictions. In comparison to current treatments, HQT exhibits a multifaceted impact on breast cancer cells, targeting multiple pathways and receptors, and holds the advantage of producing fewer toxic side effects due to its natural origin. However, additional experiments such as site-directed mutagenesis and molecular dynamics simulations are needed to validate the specific binding sites and interaction forces.

The experimental validation conducted in this study confirmed the in silico predictions, demonstrating that HQT can inhibit breast cancer cell proliferation, induce cell cycle arrest, promote apoptosis, and modulate the HIF-1 signaling pathway. These findings are consistent with previous studies on the anti-tumor activities of HQT components, highlighting the relevance of traditional herbal formulations in modern cancer therapy. The observed effects of HQT on cell cycle progression and apoptosis suggest its potential as a cytostatic and cytotoxic agent against breast cancer cells. Furthermore, the modulation of the HIF-1 signaling pathway by HQT suggests its involvement in tumor microenvironment regulation, angiogenesis, and metastasis, providing additional avenues for therapeutic intervention [19].

A key finding of this study is that HQT may exert its anti-breast cancer effects by regulating the HIF-1 signaling pathway. The HIF-1 pathway plays a central role in tumor adaptation to hypoxia and is closely associated with cancer progression, invasion, metastasis, and therapeutic resistance [20]. The observed downregulation of HIF-1α protein expression following HQT treatment suggests its potential to promote HIF-1α degradation, thereby inhibiting the HIF-1 pathway. Research has demonstrated that among breast cancer cells, MDA-MB-231 cells exhibit the highest expression of HIF-1α, and its inhibition can deactivate Bcl-2 function, trigger the cascade reaction of cysteine proteases, and induce apoptosis [21]. Moreover, active compounds found in HQT, such as quercetin [22] and kaempferol [23], have been shown to impede the accumulation of HIF-1α under hypoxic conditions, implying a potential direct interaction between these compounds and HIF-1α. Further studies are needed to elucidate the precise mechanisms by which HQT modulates HIF-1 signaling and its downstream effects in breast cancer cells, which could provide potential targets for therapeutic intervention and synergistic combination strategies with conventional treatments.

Despite the promising findings, several limitations should be acknowledged. First, the study primarily focused on in vitro experiments, and further in vivo studies are needed to validate the efficacy and safety of HQT in animal models and clinical trials. Second, the molecular mechanisms revealed in this study are still not comprehensive and in-depth. Additionally, the complex nature of TCM formulations poses challenges in elucidating the precise mechanisms of action and identifying the active components responsible for therapeutic effects.

## Conclusion

**Fig. 8:**
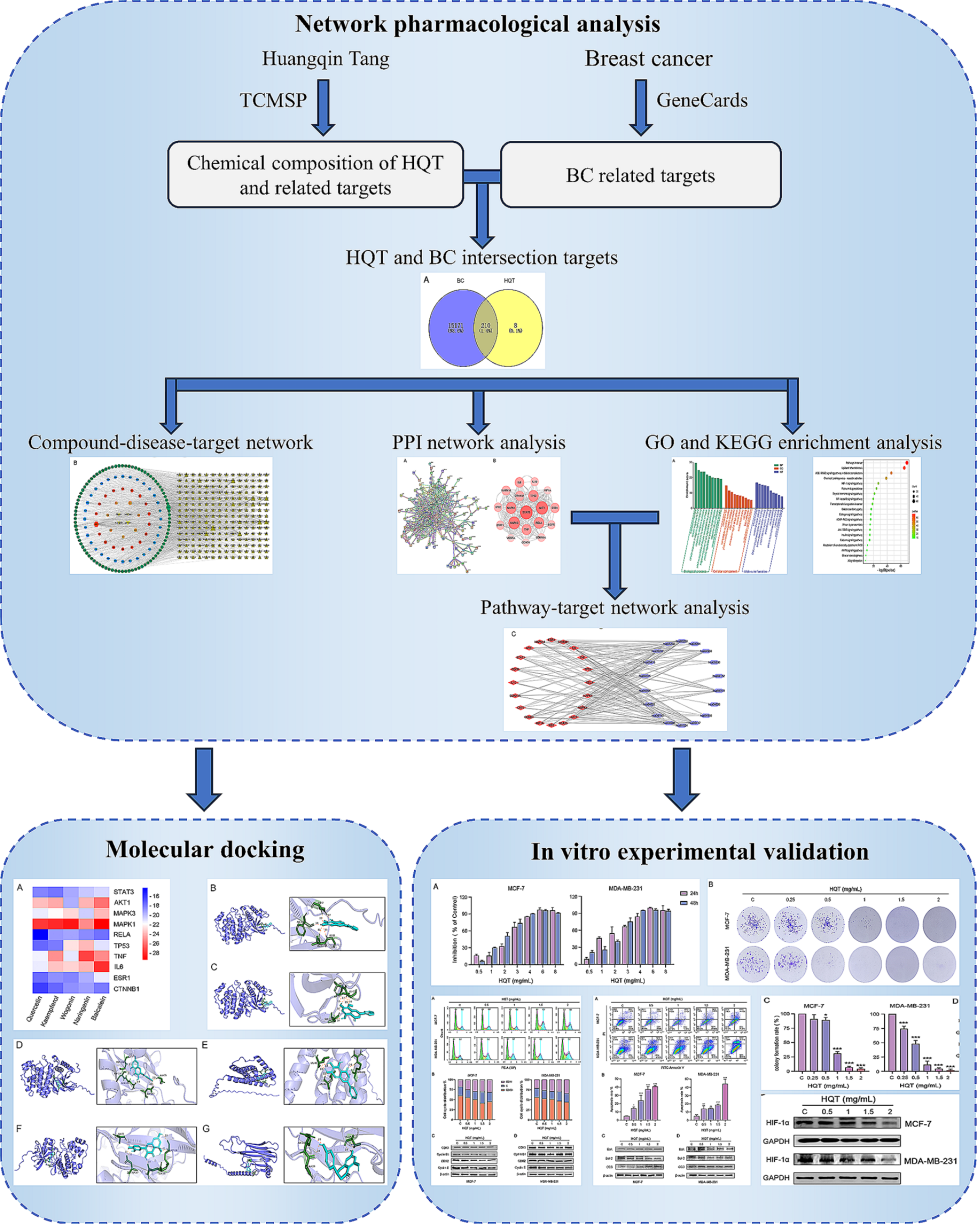
The flowchart of this study

In conclusion, the network pharmacology-based approach employed in this study elucidated the potential mechanisms of HQT in treating BC. Figure 8 depicts the flowchart of this study. These findings demonstrated that HQT exerts its effects through multiple targets and pathways associated with BC, aligning with the holistic principles of TCM. Experimental validation supported the in silico predictions, highlighting HQT’s ability to inhibit cell proliferation, induce cell cycle arrest, promote apoptosis, and modulate the HIF-1 signaling pathway. These results provide valuable insights into the therapeutic potential of HQT in BC treatment, suggesting its multi-targeted approach and modulation of the tumor microenvironment. However, further in vivo studies and comprehensive investigations are necessary to validate these findings and uncover the precise molecular mechanisms underlying HQT’s effects. Nonetheless, this study underscores the significance of traditional herbal formulations like HQT in modern cancer therapy, offering new avenues for therapeutic intervention and potential synergistic combinations with conventional treatments.

### Electronic supplementary material

Below is the link to the electronic supplementary material.

Supplementary Material 1

Supplementary Material 2

Supplementary Material 3

Supplementary Material 4

Supplementary Material 5

## Data Availability

The data generated in this study are available from the corresponding author upon request.

## Abbreviations

HQT: Huangqin Tang
TCMSP: Traditional Chinese Medicine Systems Pharmacology
BC: Breast cancer
IARC: International Agency for Research on Cancer
HQ: Huangqin
BS: Baishao
GC: Gancao
DZ: Dazao
DL: Drug-like
OB: Oral bioavailability
GO: Gene Ontology
KEGG: Kyoto Encyclopedia of Genes and Genomes
PPI: Protein-protein interaction
CCK-8: Cell Counting Kit
DMEM: Dulbecco’s modified eagle medium
CC-3: Cleaved Caspase-3
PI: Propidium iodide
BP: Biological process
CC: Cellular component
MF: Molecular function
