# Supplementary material for: Exploring the potential of Huangqin Tang in breast cancer treatment using network pharmacological analysis and experimental verification
Source: BMC Complement Med Ther. 2024 Jun 7;24:221. doi: 10.1186/s12906-024-04523-0 (PMC11161988; doi:10.1186/s12906-024-04523-0)
Supplement: Supplementary file 1 — Supplementary Material 1 [file 12906_2024_4523_MOESM1_ESM.pdf]

# Information about Huangqin active ingredient

| Latin binomial nomenclature name | Chinese name | Mol ID | Molecule Name | MW | OB (%) | Caco-2 | BBB | DL |
|---|---|---|---|---|---|---|---|---|
| *Scutellaria baicalensis* Georgi | Huangqin | MOL001689 | acacetin | 284.28 | 34.97 | 0.67 | -0.05 | 0.24 |
| *Scutellaria baicalensis* Georgi | Huangqin | MOL000173 | wogonin | 284.28 | 30.68 | 0.79 | 0.04 | 0.23 |
| *Scutellaria baicalensis* Georgi | Huangqin | MOL000228 | (2R)-7-hydroxy-5-methoxy-2-phenylchroman-4-one | 270.3 | 55.23 | 0.87 | 0.26 | 0.2 |
| *Scutellaria baicalensis* Georgi | Huangqin | MOL002714 | baicalein | 270.25 | 33.52 | 0.63 | -0.05 | 0.21 |
| *Scutellaria baicalensis* Georgi | Huangqin | MOL002908 | 5,8,2'-Trihydroxy-7-methoxyflavone | 300.28 | 37.01 | 0.76 | -0.07 | 0.27 |
| *Scutellaria baicalensis* Georgi | Huangqin | MOL002909 | 5,7,2,5-tetrahydroxy-8,6-dimethoxyflavone | 376.34 | 33.82 | 0.35 | -0.59 | 0.45 |
| *Scutellaria baicalensis* Georgi | Huangqin | MOL002910 | Carthamidin | 288.27 | 41.15 | 0.16 | -0.42 | 0.24 |
| *Scutellaria baicalensis* Georgi | Huangqin | MOL002911 | 2,6,2',4'-tetrahydroxy-6'-methoxychaleone | 302.3 | 69.04 | -0.07 | -0.32 | 0.22 |
| *Scutellaria baicalensis* Georgi | Huangqin | MOL002913 | Dihydrobaicalin_qt | 272.27 | 40.04 | 0.56 | 0.18 | 0.21 |
| *Scutellaria baicalensis* Georgi | Huangqin | MOL002914 | Eriodyctiol (flavanone) | 288.27 | 41.35 | 0.05 | -0.66 | 0.24 |
| *Scutellaria baicalensis* Georgi | Huangqin | MOL002915 | Salvigenin | 328.34 | 49.07 | 0.86 | -0.03 | 0.33 |
| *Scutellaria baicalensis* Georgi | Huangqin | MOL002917 | 5,2',6'-Trihydroxy-7,8-dimethoxyflavone | 330.31 | 45.05 | 0.48 | -0.11 | 0.33 |
| *Scutellaria baicalensis* Georgi | Huangqin | MOL002925 | 5,7,2',6'-Tetrahydroxyflavone | 286.25 | 37.01 | 0.18 | -0.56 | 0.24 |
| *Scutellaria baicalensis* Georgi | Huangqin | MOL002926 | dihydrooroxylin A | 286.3 | 38.72 | 0.71 | 0.03 | 0.23 |
| *Scutellaria baicalensis* Georgi | Huangqin | MOL002927 | Skullcapflavone II | 374.37 | 69.51 | 0.68 | -0.07 | 0.44 |
| *Scutellaria baicalensis* Georgi | Huangqin | MOL002928 | oroxylin a | 284.28 | 41.37 | 0.76 | 0.13 | 0.23 |
| *Scutellaria baicalensis* Georgi | Huangqin | MOL002932 | Panicolin | 314.31 | 76.26 | 0.84 | 0.31 | 0.29 |
| *Scutellaria baicalensis* Georgi | Huangqin | MOL002933 | 5,7,4'-Trihydroxy-8-methoxyflavone | 300.28 | 36.56 | 0.46 | -0.4 | 0.27 |
| *Scutellaria baicalensis* Georgi | Huangqin | MOL002934 | NEOBAICALEIN | 374.37 | 104.34 | 0.74 | -0.19 | 0.44 |
| *Scutellaria baicalensis* Georgi | Huangqin | MOL002937 | DIHYDROOROXYLIN | 286.3 | 66.06 | 0.67 | 0.13 | 0.23 |
| *Scutellaria baicalensis* Georgi | Huangqin | MOL000358 | beta-sitosterol | 414.79 | 36.91 | 1.32 | 0.99 | 0.75 |
| *Scutellaria baicalensis* Georgi | Huangqin | MOL000359 | sitosterol | 414.79 | 36.91 | 1.32 | 0.87 | 0.75 |
| *Scutellaria baicalensis* Georgi | Huangqin | MOL000525 | Norwogonin | 270.25 | 39.4 | 0.6 | -0.17 | 0.21 |
| *Scutellaria baicalensis* Georgi | Huangqin | MOL000552 | 5,2'-Dihydroxy-6,7,8-trimethoxyflavone | 344.34 | 31.71 | 0.93 | 0 | 0.35 |
| *Scutellaria baicalensis* Georgi | Huangqin | MOL000073 | ent-Epicatechin | 290.29 | 48.96 | 0.02 | -0.64 | 0.24 |
| *Scutellaria baicalensis* Georgi | Huangqin | MOL000449 | Stigmasterol | 412.77 | 43.83 | 1.44 | 1 | 0.76 |
| *Scutellaria baicalensis* Georgi | Huangqin | MOL001458 | coptisine | 320.34 | 30.67 | 1.21 | 0.32 | 0.86 |
| *Scutellaria baicalensis* Georgi | Huangqin | MOL001490 | bis[(2S)-2-ethylhexyl] benzene-1,2-dicarboxylate | 390.62 | 43.59 | 0.98 | 0.68 | 0.35 |
| *Scutellaria baicalensis* Georgi | Huangqin | MOL001506 | Supraene | 410.8 | 33.55 | 2.08 | 1.73 | 0.42 |
| *Scutellaria baicalensis* Georgi | Huangqin | MOL002879 | Diop | 390.62 | 43.59 | 0.79 | 0.26 | 0.39 |
| *Scutellaria baicalensis* Georgi | Huangqin | MOL002897 | epiberberine | 336.39 | 43.09 | 1.17 | 0.4 | 0.78 |
| *Scutellaria baicalensis* Georgi | Huangqin | MOL008206 | Moslosooflavone | 298.31 | 44.09 | 1.01 | 0.54 | 0.25 |
| *Scutellaria baicalensis* Georgi | Huangqin | MOL010415 | 11,13-Eicosadienoic acid, methyl ester | 322.59 | 39.28 | 1.46 | 1.24 | 0.23 |
| *Scutellaria baicalensis* Georgi | Huangqin | MOL012245 | 5,7,4'-trihydroxy-6-methoxyflavanone | 302.3 | 36.63 | 0.43 | -0.32 | 0.27 |
| *Scutellaria baicalensis* Georgi | Huangqin | MOL012246 | 5,7,4'-trihydroxy-8-methoxyflavanone | 302.3 | 74.24 | 0.37 | -0.43 | 0.26 |
| *Scutellaria baicalensis* Georgi | Huangqin | MOL012266 | rivularin | 344.34 | 37.94 | 0.65 | -0.13 | 0.37 |

# Information about Baishao active ingredient

| Latin binomial nomenclature name | Chinese name | Mol ID | Molecule Name | MW | OB (%) | Caco-2 | BBB | DL |
|---|---|---|---|---|---|---|---|---|
| *Paeonia lactiflora* Pall. | Baishao | MOL001910 | 11alpha,12alpha-epoxy-3beta-23-dihydroxy-30-norolean-20-en-28,12beta-olide | 470.71 | 64.77 | 0.09 | -0.59 | 0.38 |
| *Paeonia lactiflora* Pall. | Baishao | MOL001918 | paeoniflorgenone | 318.35 | 87.59 | -0.09 | -0.56 | 0.37 |
| *Paeonia lactiflora* Pall. | Baishao | MOL001919 | (3S,5R,8R,9R,10S,14S)-3,17-dihydroxy-4,4,8,10,14-pentamethyl-2,3,5,6,7,9-hexahydro-1H-cyclopenta[a]phenanthrene-15,16-dione | 358.52 | 43.56 | 0 | -0.41 | 0.53 |
| *Paeonia lactiflora* Pall. | Baishao | MOL001921 | Lactiflorin | 462.49 | 49.12 | -1.13 | -1.76 | 0.8 |
| *Paeonia lactiflora* Pall. | Baishao | MOL001924 | paeoniflorin | 480.51 | 53.87 | -1.47 | -1.86 | 0.79 |
| *Paeonia lactiflora* Pall. | Baishao | MOL001925 | paeoniflorin_qt | 318.35 | 68.18 | -0.34 | -0.73 | 0.4 |
| *Paeonia lactiflora* Pall. | Baishao | MOL001928 | albiflorin_qt | 318.35 | 66.64 | -0.49 | -0.88 | 0.33 |
| *Paeonia lactiflora* Pall. | Baishao | MOL001930 | benzoyl paeoniflorin | 584.62 | 31.27 | -0.69 | -1.24 | 0.75 |
| *Paeonia lactiflora* Pall. | Baishao | MOL000211 | Mairin | 456.78 | 55.38 | 0.73 | 0.22 | 0.78 |
| *Paeonia lactiflora* Pall. | Baishao | MOL000358 | beta-sitosterol | 414.79 | 36.91 | 1.32 | 0.99 | 0.75 |
| *Paeonia lactiflora* Pall. | Baishao | MOL000359 | sitosterol | 414.79 | 36.91 | 1.32 | 0.87 | 0.75 |
| *Paeonia lactiflora* Pall. | Baishao | MOL000422 | kaempferol | 286.25 | 41.88 | 0.26 | -0.55 | 0.24 |
| *Paeonia lactiflora* Pall. | Baishao | MOL000492 | (+)-catechin | 290.29 | 54.83 | -0.03 | -0.73 | 0.24 |

## Information about Gancao active ingredient

| Latin binomial nomenclature name | Chinese name | Mol ID | Molecule Name | MW | OB (%) | Caco-2 | BBB | DL |
|---|---|---|---|---|---|---|---|---|
| *Glycyrrhiza uralensis* Fisch. | Gancao | MOL004806 | euchrenone | 406.56 | 30.29 | 1.09 | 0.39 | 0.57 |
| *Glycyrrhiza uralensis* Fisch. | Gancao | MOL004864 | 5,7-dihydroxy-3-(4-methoxyphenyl)-8-(3-methylbut-2-enyl)chromone | 352.41 | 30.49 | 0.9 | 0.21 | 0.41 |
| *Glycyrrhiza uralensis* Fisch. | Gancao | MOL004985 | icos-5-enoic acid | 310.58 | 30.7 | 1.22 | 1.09 | 0.2 |
| *Glycyrrhiza uralensis* Fisch. | Gancao | MOL004996 | gadelaidic acid | 310.58 | 30.7 | 1.2 | 0.94 | 0.2 |
| *Glycyrrhiza uralensis* Fisch. | Gancao | MOL004805 | (2S)-2-[4-hydroxy-3-(3-methylbut-2-enyl)phenyl]-8,8-dimethyl-2,3-dihydropyrano[2,3-f]chromen-4-one | 390.51 | 31.79 | 1 | 0.25 | 0.72 |
| *Glycyrrhiza uralensis* Fisch. | Gancao | MOL004814 | Isotrifoliol | 298.26 | 31.94 | 0.53 | -0.25 | 0.42 |
| *Glycyrrhiza uralensis* Fisch. | Gancao | MOL004833 | Phaseolinisoflavan | 324.4 | 32.01 | 1.01 | 0.46 | 0.45 |
| *Glycyrrhiza uralensis* Fisch. | Gancao | MOL004988 | Kanzonol F | 420.54 | 32.47 | 1.18 | 0.56 | 0.89 |
| *Glycyrrhiza uralensis* Fisch. | Gancao | MOL001792 | DFV | 256.27 | 32.76 | 0.51 | -0.29 | 0.18 |
| *Glycyrrhiza uralensis* Fisch. | Gancao | MOL004860 | licorice glycoside E | 693.71 | 32.89 | -2.06 | -2.8 | 0.27 |
| *Glycyrrhiza uralensis* Fisch. | Gancao | MOL004882 | Licocoumarone | 340.4 | 33.21 | 0.84 | 0.06 | 0.36 |
| *Glycyrrhiza uralensis* Fisch. | Gancao | MOL004905 | 3,22-Dihydroxy-11-oxo-delta(12)-oleanene-27-alpha-methoxycarbonyl-29-oic acid | 512.75 | 34.32 | -0.06 | -0.75 | 0.55 |
| *Glycyrrhiza uralensis* Fisch. | Gancao | MOL004935 | Sigmoidin-B | 356.4 | 34.88 | 0.42 | -0.41 | 0.41 |
| *Glycyrrhiza uralensis* Fisch. | Gancao | MOL004978 | 2-[(3R)-8,8-dimethyl-3,4-dihydro-2H-pyrano[6,5-f]chromen-3-yl]-5-methoxyphenol | 338.43 | 36.21 | 1.12 | 0.61 | 0.52 |
| *Glycyrrhiza uralensis* Fisch. | Gancao | MOL004945 | (2S)-7-hydroxy-2-(4-hydroxyphenyl)-8-(3-methylbut-2-enyl)chroman-4-one | 324.4 | 36.57 | 0.72 | -0.04 | 0.32 |
| *Glycyrrhiza uralensis* Fisch. | Gancao | MOL000359 | sitosterol | 414.79 | 36.91 | 1.32 | 0.87 | 0.75 |
| *Glycyrrhiza uralensis* Fisch. | Gancao | MOL004917 | glycyroside | 562.57 | 37.25 | -1.58 | -2.56 | 0.79 |
| *Glycyrrhiza uralensis* Fisch. | Gancao | MOL004957 | HMO | 268.28 | 38.37 | 0.79 | 0.25 | 0.21 |
| *Glycyrrhiza uralensis* Fisch. | Gancao | MOL004991 | 7-Acetoxy-2-methylisoflavone | 294.32 | 38.92 | 0.74 | 0.16 | 0.26 |
| *Glycyrrhiza uralensis* Fisch. | Gancao | MOL004884 | Licoisoflavone B | 352.36 | 38.93 | 0.46 | -0.18 | 0.55 |
| *Glycyrrhiza uralensis* Fisch. | Gancao | MOL004989 | 6-prenylated eriodictyol | 356.4 | 39.22 | 0.4 | -0.29 | 0.41 |
| *Glycyrrhiza uralensis* Fisch. | Gancao | MOL004815 | (E)-1-(2,4-dihydroxyphenyl)-3-(2,2-dimethylchromen-6-yl)prop-2-en-1-one | 322.38 | 39.62 | 0.66 | -0.12 | 0.35 |
| *Glycyrrhiza uralensis* Fisch. | Gancao | MOL004980 | Inflacoumarin A | 322.38 | 39.71 | 0.73 | -0.24 | 0.33 |
| *Glycyrrhiza uralensis* Fisch. | Gancao | MOL000497 | licochalcone a | 338.43 | 40.79 | 0.82 | -0.21 | 0.29 |
| *Glycyrrhiza uralensis* Fisch. | Gancao | MOL004924 | (-)-Medicocarpin | 432.46 | 40.99 | -0.6 | -1.34 | 0.95 |
| *Glycyrrhiza uralensis* Fisch. | Gancao | MOL005013 | 18α-hydroxyglycyrrhetic acid | 486.76 | 41.16 | -0.29 | -0.78 | 0.71 |
| *Glycyrrhiza uralensis* Fisch. | Gancao | MOL005008 | Glycyrrhiza flavonol A | 370.38 | 41.28 | -0.09 | -0.81 | 0.6 |
| *Glycyrrhiza uralensis* Fisch. | Gancao | MOL004883 | Licoisoflavone | 354.38 | 41.61 | 0.37 | -0.27 | 0.42 |
| *Glycyrrhiza uralensis* Fisch. | Gancao | MOL000422 | kaempferol | 286.25 | 41.88 | 0.26 | -0.55 | 0.24 |
| *Glycyrrhiza uralensis* Fisch. | Gancao | MOL003896 | 7-Methoxy-2-methyl isoflavone | 266.31 | 42.56 | 1.16 | 0.56 | 0.2 |
| *Glycyrrhiza uralensis* Fisch. | Gancao | MOL004915 | Eurycarpin A | 338.38 | 43.28 | 0.43 | -0.06 | 0.37 |
| *Glycyrrhiza uralensis* Fisch. | Gancao | MOL004966 | 3'-Hydroxy-4'-O-Methylglabridin | 354.43 | 43.71 | 1 | 0.73 | 0.57 |
| *Glycyrrhiza uralensis* Fisch. | Gancao | MOL004866 | 2-(3,4-dihydroxyphenyl)-5,7-dihydroxy-6-(3-methylbut-2-enyl)chromone | 354.38 | 44.15 | 0.48 | -0.28 | 0.41 |
| *Glycyrrhiza uralensis* Fisch. | Gancao | MOL004948 | Isoglycyrol | 366.39 | 44.7 | 0.91 | 0.05 | 0.84 |
| *Glycyrrhiza uralensis* Fisch. | Gancao | MOL004828 | Glepidotin A | 338.38 | 44.72 | 0.79 | 0.06 | 0.35 |
| *Glycyrrhiza uralensis* Fisch. | Gancao | MOL004949 | Isolicoflavonol | 354.38 | 45.17 | 0.54 | -0.42 | 0.42 |
| *Glycyrrhiza uralensis* Fisch. | Gancao | MOL004811 | Glyasperin C | 356.45 | 45.56 | 0.71 | 0.07 | 0.4 |
| *Glycyrrhiza uralensis* Fisch. | Gancao | MOL004974 | 3'-Methoxyglabridin | 354.43 | 46.16 | 0.94 | 0.47 | 0.57 |
| *Glycyrrhiza uralensis* Fisch. | Gancao | MOL004911 | Glabrene | 322.38 | 46.27 | 0.99 | 0.04 | 0.44 |
| *Glycyrrhiza uralensis* Fisch. | Gancao | MOL004898 | (E)-3-[3,4-dihydroxy-5-(3-methylbut-2-enyl)phenyl]-1-(2,4-dihydroxyphenyl)prop-2-en-1-one | 340.4 | 46.27 | 0.41 | -0.4 | 0.31 |
| *Glycyrrhiza uralensis* Fisch. | Gancao | MOL000098 | quercetin | 302.25 | 46.43 | 0.05 | -0.77 | 0.28 |
| *Glycyrrhiza uralensis* Fisch. | Gancao | MOL004961 | Quercetin der. | 330.31 | 46.45 | 0.39 | -0.44 | 0.33 |
| *Glycyrrhiza uralensis* Fisch. | Gancao | MOL000417 | Calycosin | 284.28 | 47.75 | 0.52 | -0.43 | 0.24 |
| *Glycyrrhiza uralensis* Fisch. | Gancao | MOL004913 | 1,3-dihydroxy-9-methoxy-6-benzofurano[3,2-c]chromenone | 298.26 | 48.14 | 0.48 | -0.19 | 0.43 |
| *Glycyrrhiza uralensis* Fisch. | Gancao | MOL004827 | Semilicoisoflavone B | 352.36 | 48.78 | 0.45 | -0.33 | 0.55 |
| *Glycyrrhiza uralensis* Fisch. | Gancao | MOL004857 | Gancaonin B | 368.41 | 48.79 | 0.58 | -0.1 | 0.45 |
| *Glycyrrhiza uralensis* Fisch. | Gancao | MOL002565 | Medicarpin | 270.3 | 49.22 | 1 | 0.53 | 0.34 |
| *Glycyrrhiza uralensis* Fisch. | Gancao | MOL004848 | licochalcone G | 354.43 | 49.25 | 0.64 | -0.04 | 0.32 |
| *Glycyrrhiza uralensis* Fisch. | Gancao | MOL000354 | isorhamnetin | 316.28 | 49.6 | 0.31 | -0.54 | 0.31 |
| *Glycyrrhiza uralensis* Fisch. | Gancao | MOL005016 | Odoratin | 314.31 | 49.95 | 0.42 | -0.24 | 0.3 |
| *Glycyrrhiza uralensis* Fisch. | Gancao | MOL005001 | Gancaonin H | 420.49 | 50.1 | 0.6 | -0.14 | 0.78 |
| *Glycyrrhiza uralensis* Fisch. | Gancao | MOL004820 | kanzonols W | 336.36 | 50.48 | 0.63 | 0.04 | 0.52 |
| *Glycyrrhiza uralensis* Fisch. | Gancao | MOL000239 | Jaranol | 314.31 | 50.83 | 0.61 | -0.22 | 0.29 |
| *Glycyrrhiza uralensis* Fisch. | Gancao | MOL004856 | Gancaonin A | 352.41 | 51.08 | 0.8 | 0.13 | 0.4 |
| *Glycyrrhiza uralensis* Fisch. | Gancao | MOL003656 | Lupiwighteone | 338.38 | 51.64 | 0.68 | -0.23 | 0.37 |
| *Glycyrrhiza uralensis* Fisch. | Gancao | MOL004885 | licoisoflavanone | 354.38 | 52.47 | 0.39 | -0.22 | 0.54 |
| *Glycyrrhiza uralensis* Fisch. | Gancao | MOL004912 | Glabrone | 336.36 | 52.51 | 0.59 | -0.11 | 0.5 |
| *Glycyrrhiza uralensis* Fisch. | Gancao | MOL004879 | Glycyrin | 382.44 | 52.61 | 0.59 | -0.13 | 0.47 |
| *Glycyrrhiza uralensis* Fisch. | Gancao | MOL004910 | Glabranin | 324.4 | 52.9 | 0.97 | 0.31 | 0.31 |
| *Glycyrrhiza uralensis* Fisch. | Gancao | MOL004908 | Glabridin | 324.4 | 53.25 | 0.97 | 0.36 | 0.47 |
| *Glycyrrhiza uralensis* Fisch. | Gancao | MOL004993 | 8-prenylated eriodictyol | 356.4 | 53.79 | 0.43 | -0.44 | 0.4 |
| *Glycyrrhiza uralensis* Fisch. | Gancao | MOL005020 | dehydroglyasperins C | 340.4 | 53.82 | 0.68 | -0.12 | 0.37 |
| *Glycyrrhiza uralensis* Fisch. | Gancao | MOL005018 | Xambioona | 388.49 | 54.85 | 1.09 | 0.52 | 0.87 |
| *Glycyrrhiza uralensis* Fisch. | Gancao | MOL000211 | Mairin | 456.78 | 55.38 | 0.73 | 0.22 | 0.78 |
| *Glycyrrhiza uralensis* Fisch. | Gancao | MOL005012 | Licoagroisoflavone | 336.36 | 57.28 | 0.71 | 0.09 | 0.49 |
| *Glycyrrhiza uralensis* Fisch. | Gancao | MOL004838 | 8-(6-hydroxy-2-benzofuranyl)-2,2-dimethyl-5-chromenol | 308.35 | 58.44 | 1 | 0.34 | 0.38 |
| *Glycyrrhiza uralensis* Fisch. | Gancao | MOL005003 | Licoagrocarpin | 338.43 | 58.81 | 1.23 | 0.61 | 0.58 |
| *Glycyrrhiza uralensis* Fisch. | Gancao | MOL004328 | naringenin | 272.27 | 59.29 | 0.28 | -0.37 | 0.21 |
| *Glycyrrhiza uralensis* Fisch. | Gancao | MOL004849 | 3-(2,4-dihydroxyphenyl)-8-(1,1-dimethylprop-2-enyl)-7-hydroxy-5-methoxy-coumarin | 368.41 | 59.62 | 0.4 | -0.23 | 0.43 |

## Information about Gancao active ingredient

| Latin binomial nomenclature name | Chinese name | Mol ID | Molecule Name | MW | OB (%) | Caco-2 | BBB | DL |
|---|---|---|---|---|---|---|---|---|
| *Glycyrrhiza uralensis* Fisch. | Gancao | MOL004824 | (2S)-6-(2,4-dihydroxyphenyl)-2-(2-hydroxypropan-2-yl)-4-methoxy-2,3-dihydrofuro[3,2-g]chromen-7-one | 384.41 | 60.25 | 0 | -0.76 | 0.63 |
| *Glycyrrhiza uralensis* Fisch. | Gancao | MOL005000 | Gancaonin G | 352.41 | 60.44 | 0.78 | 0.23 | 0.39 |
| *Glycyrrhiza uralensis* Fisch. | Gancao | MOL004907 | Glyzaglabrin | 298.26 | 61.07 | 0.34 | -0.2 | 0.35 |
| *Glycyrrhiza uralensis* Fisch. | Gancao | MOL004835 | Glypallichalcone | 284.33 | 61.6 | 0.76 | 0.23 | 0.19 |
| *Glycyrrhiza uralensis* Fisch. | Gancao | MOL004914 | 1,3-dihydroxy-8,9-dimethoxy-6-benzofurano[3,2-c]chromenone | 328.29 | 62.9 | 0.4 | -0.34 | 0.53 |
| *Glycyrrhiza uralensis* Fisch. | Gancao | MOL004855 | Licoricone | 382.44 | 63.58 | 0.53 | -0.14 | 0.47 |
| *Glycyrrhiza uralensis* Fisch. | Gancao | MOL004829 | Glepidotin B | 340.4 | 64.46 | 0.46 | -0.09 | 0.34 |
| *Glycyrrhiza uralensis* Fisch. | Gancao | MOL004808 | glyasperin B | 370.43 | 65.22 | 0.47 | -0.09 | 0.44 |
| *Glycyrrhiza uralensis* Fisch. | Gancao | MOL004903 | liquiritin | 418.43 | 65.69 | -1.06 | -1.93 | 0.74 |
| *Glycyrrhiza uralensis* Fisch. | Gancao | MOL004863 | 3-(3,4-dihydroxyphenyl)-5,7-dihydroxy-8-(3-methylbut-2-enyl)chromone | 354.38 | 66.37 | 0.52 | -0.13 | 0.41 |
| *Glycyrrhiza uralensis* Fisch. | Gancao | MOL000392 | formononetin | 268.28 | 69.67 | 0.78 | 0.02 | 0.21 |
| *Glycyrrhiza uralensis* Fisch. | Gancao | MOL004959 | 1-Methoxyphaseollidin | 354.43 | 69.98 | 1.01 | 0.48 | 0.64 |
| *Glycyrrhiza uralensis* Fisch. | Gancao | MOL004941 | (2R)-7-hydroxy-2-(4-hydroxyphenyl)chroman-4-one | 256.27 | 71.12 | 0.41 | -0.25 | 0.18 |
| *Glycyrrhiza uralensis* Fisch. | Gancao | MOL005007 | Glyasperins M | 368.41 | 72.67 | 0.49 | -0.04 | 0.59 |
| *Glycyrrhiza uralensis* Fisch. | Gancao | MOL000500 | Vestitol | 272.32 | 74.66 | 0.86 | 0.3 | 0.21 |
| *Glycyrrhiza uralensis* Fisch. | Gancao | MOL001484 | Inermine | 284.28 | 75.18 | 0.89 | 0.4 | 0.54 |
| *Glycyrrhiza uralensis* Fisch. | Gancao | MOL004810 | glyasperin F | 354.38 | 75.84 | 0.43 | -0.15 | 0.54 |
| *Glycyrrhiza uralensis* Fisch. | Gancao | MOL004841 | Licochalcone B | 286.3 | 76.76 | 0.47 | -0.46 | 0.19 |
| *Glycyrrhiza uralensis* Fisch. | Gancao | MOL005017 | Phaseol | 336.36 | 78.77 | 0.76 | -0.06 | 0.58 |
| *Glycyrrhiza uralensis* Fisch. | Gancao | MOL004891 | shinpterocarpin | 322.38 | 80.3 | 1.1 | 0.68 | 0.73 |
| *Glycyrrhiza uralensis* Fisch. | Gancao | MOL004904 | licopyranocoumarin | 384.41 | 80.36 | 0.13 | -0.62 | 0.65 |
| *Glycyrrhiza uralensis* Fisch. | Gancao | MOL004990 | 7,2',4'-trihydroxy－5-methoxy-3－arylcoumarin | 300.28 | 83.71 | 0.24 | -0.59 | 0.27 |
| *Glycyrrhiza uralensis* Fisch. | Gancao | MOL002311 | Glycyrol | 366.39 | 90.78 | 0.71 | -0.2 | 0.67 |

## Information about Dazao active ingredient

| Latin binomial nomenclature name | Chinese name | Mol ID | Molecule Name | MW | OB (%) | Caco-2 | BBB | DL |
|---|---|---|---|---|---|---|---|---|
| *Ziziphus jujuba* Mill. | Dazao | MOL001522 | (S)-Coclaurine | 285.37 | 42.35 | 0.7 | 0.06 | 0.24 |
| *Ziziphus jujuba* Mill. | Dazao | MOL000096 | (-)-catechin | 290.29 | 49.68 | -0.03 | -0.78 | 0.24 |
| *Ziziphus jujuba* Mill. | Dazao | MOL000492 | (+)-catechin | 290.29 | 54.83 | -0.03 | -0.73 | 0.24 |
| *Ziziphus jujuba* Mill. | Dazao | MOL008647 | Moupinamide | 313.38 | 86.71 | 0.55 | -0.51 | 0.26 |
| *Ziziphus jujuba* Mill. | Dazao | MOL000098 | quercetin | 302.25 | 46.43 | 0.05 | -0.77 | 0.28 |
| *Ziziphus jujuba* Mill. | Dazao | MOL012921 | stepharine | 297.38 | 31.55 | 0.64 | 0.17 | 0.33 |
| *Ziziphus jujuba* Mill. | Dazao | MOL012976 | coumestrol | 268.23 | 32.49 | 0.55 | -0.48 | 0.34 |
| *Ziziphus jujuba* Mill. | Dazao | MOL007213 | Nuciferin | 295.41 | 34.43 | 1.22 | 0.83 | 0.4 |
| *Ziziphus jujuba* Mill. | Dazao | MOL012992 | Mauritine D | 342.46 | 89.13 | 0.59 | 0.62 | 0.45 |
| *Ziziphus jujuba* Mill. | Dazao | MOL000627 | Stepholidine | 327.41 | 33.11 | 0.83 | 0.29 | 0.54 |
| *Ziziphus jujuba* Mill. | Dazao | MOL000783 | Protoporphyrin | 562.72 | 30.86 | 0.67 | -0.63 | 0.56 |
| *Ziziphus jujuba* Mill. | Dazao | MOL002773 | beta-carotene | 536.96 | 37.18 | 2.25 | 1.52 | 0.58 |
| *Ziziphus jujuba* Mill. | Dazao | MOL012940 | Spiradine A | 311.46 | 113.52 | 0.29 | 0.14 | 0.61 |
| *Ziziphus jujuba* Mill. | Dazao | MOL012989 | Jujuboside C_qt | 472.78 | 40.26 | 0.37 | -0.43 | 0.62 |
| *Ziziphus jujuba* Mill. | Dazao | MOL012961 | jujuboside A_qt | 472.78 | 36.67 | 0.54 | -0.05 | 0.62 |
| *Ziziphus jujuba* Mill. | Dazao | MOL012946 | zizyphus saponin I_qt | 472.78 | 32.69 | 0.42 | -0.32 | 0.62 |
| *Ziziphus jujuba* Mill. | Dazao | MOL003410 | Ziziphin_qt | 472.78 | 66.95 | 0.49 | -0.12 | 0.62 |
| *Ziziphus jujuba* Mill. | Dazao | MOL005360 | malkangunin | 432.56 | 57.71 | 0.22 | -0.17 | 0.63 |
| *Ziziphus jujuba* Mill. | Dazao | MOL012986 | Jujubasaponin V_qt | 472.78 | 36.99 | 0.38 | -0.51 | 0.63 |
| *Ziziphus jujuba* Mill. | Dazao | MOL000358 | beta-sitosterol | 414.79 | 36.91 | 1.32 | 0.99 | 0.75 |
| *Ziziphus jujuba* Mill. | Dazao | MOL000449 | Stigmasterol | 412.77 | 43.83 | 1.44 | 1 | 0.76 |
| *Ziziphus jujuba* Mill. | Dazao | MOL004350 | Ruvoside_qt | 390.57 | 36.12 | -0.31 | -1.13 | 0.76 |
| *Ziziphus jujuba* Mill. | Dazao | MOL008034 | 21302-79-4 | 486.76 | 73.52 | -0.05 | -0.54 | 0.77 |
| *Ziziphus jujuba* Mill. | Dazao | MOL000211 | Mairin | 456.78 | 55.38 | 0.73 | 0.22 | 0.78 |
| *Ziziphus jujuba* Mill. | Dazao | MOL001454 | berberine | 336.39 | 36.86 | 1.24 | 0.57 | 0.78 |
| *Ziziphus jujuba* Mill. | Dazao | MOL013357 | (3S,6R,8S,9S,10R,13R,14S,17R)-17-[(1R,4R)-4-ethyl-1,5-dimethylhexyl]-10,13-dimethyl-2,3,6,7,8,9,11,12,14,15,16,17-dodecahydro-1H-cyclopenta[a]phenanthrene-3,6-diol | 430.79 | 34.37 | 0.82 | 0.31 | 0.78 |
| *Ziziphus jujuba* Mill. | Dazao | MOL012980 | Daechuine S6 | 548.75 | 46.48 | 0.38 | 0.02 | 0.79 |
| *Ziziphus jujuba* Mill. | Dazao | MOL000787 | Fumarine | 353.4 | 59.26 | 0.56 | -0.13 | 0.83 |
| *Ziziphus jujuba* Mill. | Dazao | MOL012981 | Daechuine S7 | 514.74 | 44.82 | 0.46 | 0.12 | 0.83 |
