# Supplementary material for: Exploring the potential of Huangqin Tang in breast cancer treatment using network pharmacological analysis and experimental verification
Source: BMC Complement Med Ther. 2024 Jun 7;24:221. doi: 10.1186/s12906-024-04523-0 (PMC11161988; doi:10.1186/s12906-024-04523-0)
Supplement: Supplementary file 2 — Supplementary Material 2 [file 12906_2024_4523_MOESM2_ESM.pdf]

Targets related to Huangqin

MolId	MolName	Target	Symbol
MOL000073	ent-Epicatechin	Estrogen receptor	ESR1
MOL000073	ent-Epicatechin	Prostaglandin G/H synthase 1	PTGS1
MOL000073	ent-Epicatechin	Prostaglandin G/H synthase 2	PTGS2
MOL000173	wogonin	Beta-2 adrenergic receptor	ADRB2
MOL000173	wogonin	Activator of 90 kDa heat shock protein ATPase homolog 1	AHSA1
MOL000173	wogonin	RAC-alpha serine/threonine-protein kinase	AKT1
MOL000173	wogonin	Androgen receptor	AR
MOL000173	wogonin	Apoptosis regulator BAX	BAX
MOL000173	wogonin	Apoptosis regulator Bcl-2	BCL2
MOL000173	wogonin	Caspase-3	CASP3
MOL000173	wogonin	Caspase-9	CASP9
MOL000173	wogonin	C-C motif chemokine 2	CCL2
MOL000173	wogonin	G1/S-specific cyclin-D1	CCND1
MOL000173	wogonin	Cyclin-dependent kinase inhibitor 1	CDKN1A
MOL000173	wogonin	Serine/threonine-protein kinase Chk1	CHEK1
MOL000173	wogonin	Interleukin-8	CXCL8
MOL000173	wogonin	Eukaryotic translation initiation factor 6	EIF6
MOL000173	wogonin	Estrogen receptor	ESR1
MOL000173	wogonin	Fibronectin	FN1
MOL000173	wogonin	Gamma-aminobutyric acid receptor subunit alpha-1	GABRA1
MOL000173	wogonin	Glycogen synthase kinase-3 beta	GSK3B
MOL000173	wogonin	Interleukin-6	IL6
MOL000173	wogonin	Vascular endothelial growth factor receptor 2	KDR
MOL000173	wogonin	Mitogen-activated protein kinase 14	MAPK14
MOL000173	wogonin	Induced myeloid leukemia cell differentiation protein Mcl-1	MCL1
MOL000173	wogonin	Interstitial collagenase	MMP1
MOL000173	wogonin	Nitric oxide synthase, inducible	NOS2
MOL000173	wogonin	CGMP-inhibited 3',5'-cyclic phosphodiesterase A	PDE3A
MOL000173	wogonin	Protein kinase C delta type	PRKCD
MOL000173	wogonin	Prostaglandin E2 receptor EP3 subtype	PTGER3
MOL000173	wogonin	Prostaglandin G/H synthase 1	PTGS1
MOL000173	wogonin	Prostaglandin G/H synthase 2	PTGS2
MOL000173	wogonin	Transcription factor p65	RELA
MOL000173	wogonin	Retinoic acid receptor RXR-alpha	RXRA
MOL000173	wogonin	Sodium channel protein type 5 subunit alpha	SCN5A
MOL000173	wogonin	Telomerase protein component 1	TEP1
MOL000173	wogonin	Tumor necrosis factor	TNF
MOL000173	wogonin	Cellular tumor antigen p53	TP53
MOL000228	(2R)-7-hydroxy-5-methoxy-2-phenylchroman-4-one	Alpha-1A adrenergic receptor	ADRA1A
MOL000228	(2R)-7-hydroxy-5-methoxy-2-phenylchroman-4-one	Alpha-1B adrenergic receptor	ADRA1B
MOL000228	(2R)-7-hydroxy-5-methoxy-2-phenylchroman-4-one	Beta-2 adrenergic receptor	ADRB2
MOL000228	(2R)-7-hydroxy-5-methoxy-2-phenylchroman-4-one	Muscarinic acetylcholine receptor M1	CHRM1
MOL000228	(2R)-7-hydroxy-5-methoxy-2-phenylchroman-4-one	Muscarinic acetylcholine receptor M3	CHRM3
MOL000228	(2R)-7-hydroxy-5-methoxy-2-phenylchroman-4-one	Estrogen receptor	ESR1
MOL000228	(2R)-7-hydroxy-5-methoxy-2-phenylchroman-4-one	Gamma-aminobutyric acid receptor subunit alpha-1	GABRA1
MOL000228	(2R)-7-hydroxy-5-methoxy-2-phenylchroman-4-one	CGMP-inhibited 3',5'-cyclic phosphodiesterase A	PDE3A
MOL000228	(2R)-7-hydroxy-5-methoxy-2-phenylchroman-4-one	cAMP-dependent protein kinase inhibitor alpha	PKIA
MOL000228	(2R)-7-hydroxy-5-methoxy-2-phenylchroman-4-one	Prostaglandin G/H synthase 1	PTGS1
MOL000228	(2R)-7-hydroxy-5-methoxy-2-phenylchroman-4-one	Prostaglandin G/H synthase 2	PTGS2
MOL000228	(2R)-7-hydroxy-5-methoxy-2-phenylchroman-4-one	Retinoic acid receptor RXR-alpha	RXRA
MOL000228	(2R)-7-hydroxy-5-methoxy-2-phenylchroman-4-one	Sodium channel protein type 5 subunit alpha	SCN5A
MOL000228	(2R)-7-hydroxy-5-methoxy-2-phenylchroman-4-one	Sodium-dependent dopamine transporter	SLC6A3
MOL000228	(2R)-7-hydroxy-5-methoxy-2-phenylchroman-4-one	Sodium-dependent serotonin transporter	SLC6A4
MOL000358	beta-sitosterol	Alpha-1A adrenergic receptor	ADRA1A
MOL000358	beta-sitosterol	Alpha-1B adrenergic receptor	ADRA1B
MOL000358	beta-sitosterol	Beta-2 adrenergic receptor	ADRB2
MOL000358	beta-sitosterol	Apoptosis regulator BAX	BAX
MOL000358	beta-sitosterol	Apoptosis regulator Bcl-2	BCL2
MOL000358	beta-sitosterol	Caspase-3	CASP3
MOL000358	beta-sitosterol	Caspase-8	CASP8
MOL000358	beta-sitosterol	Caspase-9	CASP9
MOL000358	beta-sitosterol	Muscarinic acetylcholine receptor M1	CHRM1
MOL000358	beta-sitosterol	Muscarinic acetylcholine receptor M2	CHRM2
MOL000358	beta-sitosterol	Muscarinic acetylcholine receptor M3	CHRM3
MOL000358	beta-sitosterol	Muscarinic acetylcholine receptor M4	CHRM4
MOL000358	beta-sitosterol	Neuronal acetylcholine receptor subunit alpha-2	CHRNA2
MOL000358	beta-sitosterol	Gamma-aminobutyric acid receptor subunit alpha-1	GABRA1
MOL000358	beta-sitosterol	Potassium voltage-gated channel subfamily H member 2	KCNH2
MOL000358	beta-sitosterol	Microtubule-associated protein 2	MAP2
MOL000358	beta-sitosterol	Nuclear receptor coactivator 2	NCOA2
MOL000358	beta-sitosterol	Mu-type opioid receptor	OPRM1
MOL000358	beta-sitosterol	CGMP-inhibited 3',5'-cyclic phosphodiesterase A	PDE3A
MOL000358	beta-sitosterol	Progesterone receptor	PGR
MOL000358	beta-sitosterol	Serum paraoxonase/arylesterase 1	PON1
MOL000358	beta-sitosterol	Protein kinase C alpha type	PRKCA
MOL000358	beta-sitosterol	Prostaglandin G/H synthase 1	PTGS1
MOL000358	beta-sitosterol	Prostaglandin G/H synthase 2	PTGS2
MOL000358	beta-sitosterol	Sodium channel protein type 5 subunit alpha	SCN5A
MOL000358	beta-sitosterol	Sodium-dependent serotonin transporter	SLC6A4
MOL000359	sitosterol	Nuclear receptor coactivator 2	NCOA2
MOL000359	sitosterol	Mineralocorticoid receptor	NR3C2
MOL000359	sitosterol	Progesterone receptor	PGR
MOL000449	Stigmasterol	Alcohol dehydrogenase 1C	ADH1C
MOL000449	Stigmasterol	Alpha-1A adrenergic receptor	ADRA1A
MOL000449	Stigmasterol	Alpha-1B adrenergic receptor	ADRA1B
MOL000449	Stigmasterol	Alpha-2A adrenergic receptor	ADRA2A
MOL000449	Stigmasterol	Beta-1 adrenergic receptor	ADRB1
MOL000449	Stigmasterol	Beta-2 adrenergic receptor	ADRB2
MOL000449	Stigmasterol	Muscarinic acetylcholine receptor M1	CHRM1
MOL000449	Stigmasterol	Muscarinic acetylcholine receptor M2	CHRM2
MOL000449	Stigmasterol	Muscarinic acetylcholine receptor M3	CHRM3
MOL000449	Stigmasterol	Chymotrypsinogen B	CTRB1

Targets related to Huangqin

MolId	MolName	Target	Symbol
MOL000449	Stigmasterol	Gamma-aminobutyric acid receptor subunit alpha-1	GABRA1
MOL000449	Stigmasterol	Leukotriene A-4 hydrolase	LTA4H
MOL000449	Stigmasterol	Nuclear receptor coactivator 1	NCOA1
MOL000449	Stigmasterol	Nuclear receptor coactivator 2	NCOA2
MOL000449	Stigmasterol	Mineralocorticoid receptor	NR3C2
MOL000449	Stigmasterol	Progesterone receptor	PGR
MOL000449	Stigmasterol	Urokinase-type plasminogen activator	PLAU
MOL000449	Stigmasterol	Prostaglandin G/H synthase 1	PTGS1
MOL000449	Stigmasterol	Prostaglandin G/H synthase 2	PTGS2
MOL000449	Stigmasterol	Retinoic acid receptor RXR-alpha	RXRA
MOL000449	Stigmasterol	Sodium channel protein type 5 subunit alpha	SCN5A
MOL000449	Stigmasterol	Sodium-dependent noradrenaline transporter	SLC6A2
MOL000449	Stigmasterol	Sodium-dependent dopamine transporter	SLC6A3
MOL000525	Norwogonin	Androgen receptor	AR
MOL000525	Norwogonin	Serine/threonine-protein kinase Chk1	CHEK1
MOL000525	Norwogonin	Nitric oxide synthase, inducible	NOS2
MOL000525	Norwogonin	CGMP-inhibited 3',5'-cyclic phosphodiesterase A	PDE3A
MOL000525	Norwogonin	Prostaglandin G/H synthase 1	PTGS1
MOL000525	Norwogonin	Prostaglandin G/H synthase 2	PTGS2
MOL000552	5,2'-Dihydroxy-6,7,8-trimethoxyflavone	Androgen receptor	AR
MOL000552	5,2'-Dihydroxy-6,7,8-trimethoxyflavone	Estrogen receptor beta	ESR2
MOL000552	5,2'-Dihydroxy-6,7,8-trimethoxyflavone	Coagulation factor VII	F7
MOL000552	5,2'-Dihydroxy-6,7,8-trimethoxyflavone	Potassium voltage-gated channel subfamily H member 2	KCNH2
MOL000552	5,2'-Dihydroxy-6,7,8-trimethoxyflavone	Vascular endothelial growth factor receptor 2	KDR
MOL000552	5,2'-Dihydroxy-6,7,8-trimethoxyflavone	Nuclear receptor coactivator 1	NCOA1
MOL000552	5,2'-Dihydroxy-6,7,8-trimethoxyflavone	Nuclear receptor coactivator 2	NCOA2
MOL000552	5,2'-Dihydroxy-6,7,8-trimethoxyflavone	Nitric oxide synthase, inducible	NOS2
MOL000552	5,2'-Dihydroxy-6,7,8-trimethoxyflavone	Prostaglandin G/H synthase 1	PTGS1
MOL000552	5,2'-Dihydroxy-6,7,8-trimethoxyflavone	Prostaglandin G/H synthase 2	PTGS2
MOL000552	5,2'-Dihydroxy-6,7,8-trimethoxyflavone	Sodium channel protein type 5 subunit alpha	SCN5A
MOL001458	coptisine	Androgen receptor	AR
MOL001458	coptisine	Estrogen receptor	ESR1
MOL001458	coptisine	Potassium voltage-gated channel subfamily H member 2	KCNH2
MOL001458	coptisine	Nitric oxide synthase, inducible	NOS2
MOL001458	coptisine	Prostaglandin G/H synthase 1	PTGS1
MOL001458	coptisine	Prostaglandin G/H synthase 2	PTGS2
MOL001458	coptisine	Sodium channel protein type 5 subunit alpha	SCN5A
MOL001490	bis[(2S)-2-ethylhexyl] benzene-1,2-dicarboxylate	Sodium channel protein type 5 subunit alpha	SCN5A
MOL001689	acacetin	Beta-2 adrenergic receptor	ADRB2
MOL001689	acacetin	Androgen receptor	AR
MOL001689	acacetin	Apoptosis regulator BAX	BAX
MOL001689	acacetin	Apoptosis regulator Bcl-2	BCL2
MOL001689	acacetin	Caspase-3	CASP3
MOL001689	acacetin	Caspase-8	CASP8
MOL001689	acacetin	Cyclin-dependent kinase inhibitor 1	CDKN1A
MOL001689	acacetin	Serine/threonine-protein kinase Chk1	CHEK1
MOL001689	acacetin	Tumor necrosis factor ligand superfamily member 6	FASLG
MOL001689	acacetin	Fatty acid synthase	FASN
MOL001689	acacetin	Nuclear receptor coactivator 1	NCOA1
MOL001689	acacetin	Nuclear receptor coactivator 2	NCOA2
MOL001689	acacetin	Nitric oxide synthase, inducible	NOS2
MOL001689	acacetin	CGMP-inhibited 3',5'-cyclic phosphodiesterase A	PDE3A
MOL001689	acacetin	Prostaglandin G/H synthase 1	PTGS1
MOL001689	acacetin	Prostaglandin G/H synthase 2	PTGS2
MOL001689	acacetin	Transcription factor p65	RELA
MOL001689	acacetin	Cellular tumor antigen p53	TP53
MOL002714	baicalein	Aryl hydrocarbon receptor	AHR
MOL002714	baicalein	RAC-alpha serine/threonine-protein kinase	AKT1
MOL002714	baicalein	Apolipoprotein D	APOD
MOL002714	baicalein	Androgen receptor	AR
MOL002714	baicalein	Apoptosis regulator BAX	BAX
MOL002714	baicalein	Apoptosis regulator Bcl-2	BCL2
MOL002714	baicalein	Caspase-3	CASP3
MOL002714	baicalein	G2/mitotic-specific cyclin-B1	CCNB1
MOL002714	baicalein	Cytochrome c	CYCS
MOL002714	baicalein	Egl nine homolog 1	EGLN1
MOL002714	baicalein	Fos-related antigen 1	FOSL1
MOL002714	baicalein	Fos-related antigen 2	FOSL2
MOL002714	baicalein	Hypoxia-inducible factor 1-alpha	HIF1A
MOL002714	baicalein	Insulin-like growth factor II	IGF2
MOL002714	baicalein	Matrix metalloproteinase-9	MMP9
MOL002714	baicalein	Myeloperoxidase	MPO
MOL002714	baicalein	Nuclear receptor coactivator 1	NCOA1
MOL002714	baicalein	Nuclear receptor coactivator 2	NCOA2
MOL002714	baicalein	Nuclear factor of activated T-cells, cytoplasmic 1	NFATC1
MOL002714	baicalein	NADPH oxidase 5	NOX5
MOL002714	baicalein	CGMP-inhibited 3',5'-cyclic phosphodiesterase A	PDE3A
MOL002714	baicalein	Prostaglandin G/H synthase 1	PTGS1
MOL002714	baicalein	Prostaglandin G/H synthase 2	PTGS2
MOL002714	baicalein	Transcription factor p65	RELA
MOL002714	baicalein	Tudor domain-containing protein 7	TDRD7
MOL002714	baicalein	Cellular tumor antigen p53	TP53
MOL002714	baicalein	Vascular endothelial growth factor A	VEGFA
MOL002879	Diop	Beta-2 adrenergic receptor	ADRB2
MOL002879	Diop	Muscarinic acetylcholine receptor M3	CHRM3
MOL002879	Diop	Sodium channel protein type 5 subunit alpha	SCN5A
MOL002897	epiberberine	Androgen receptor	AR
MOL002897	epiberberine	Estrogen receptor	ESR1
MOL002897	epiberberine	Potassium voltage-gated channel subfamily H member 2	KCNH2
MOL002897	epiberberine	Nuclear receptor coactivator 2	NCOA2
MOL002897	epiberberine	Nitric oxide synthase, inducible	NOS2
MOL002897	epiberberine	cAMP and cAMP-inhibited cGMP 3',5'-cyclic phosphodiesterase 10A	PDE10A

Targets related to Huangqin

MolId	MolName	Target	Symbol
MOL002897	epiberberine	Prostaglandin G/H synthase 2	PTGS2
MOL002897	epiberberine	Retinoic acid receptor RXR-alpha	RXRA
MOL002909	5,7,2,5-tetrahydroxy-8,6-dimethoxyflavone	Androgen receptor	AR
MOL002909	5,7,2,5-tetrahydroxy-8,6-dimethoxyflavone	Nuclear receptor coactivator 2	NCOA2
MOL002909	5,7,2,5-tetrahydroxy-8,6-dimethoxyflavone	Nitric oxide synthase, inducible	NOS2
MOL002909	5,7,2,5-tetrahydroxy-8,6-dimethoxyflavone	Prostaglandin G/H synthase 2	PTGS2
MOL002909	5,7,2,5-tetrahydroxy-8,6-dimethoxyflavone	Glycogen phosphorylase, muscle form	PYGM
MOL002910	Carthamidin	Prostaglandin G/H synthase 1	PTGS1
MOL002910	Carthamidin	Prostaglandin G/H synthase 2	PTGS2
MOL002913	Dihydrobaicalin_qt	Prostaglandin G/H synthase 1	PTGS1
MOL002913	Dihydrobaicalin_qt	Prostaglandin G/H synthase 2	PTGS2
MOL002914	Eriodyctiol (flavanone)	Nuclear receptor coactivator 2	NCOA2
MOL002914	Eriodyctiol (flavanone)	Prostaglandin G/H synthase 1	PTGS1
MOL002914	Eriodyctiol (flavanone)	Prostaglandin G/H synthase 2	PTGS2
MOL002914	Eriodyctiol (flavanone)	Glycogen phosphorylase, muscle form	PYGM
MOL002915	Salvigenin	Acetylcholinesterase	ACHE
MOL002915	Salvigenin	Alpha-1B adrenergic receptor	ADRA1B
MOL002915	Salvigenin	Beta-2 adrenergic receptor	ADRB2
MOL002915	Salvigenin	Coagulation factor VII	F7
MOL002915	Salvigenin	Nuclear receptor coactivator 2	NCOA2
MOL002915	Salvigenin	Nitric oxide synthase, inducible	NOS2
MOL002915	Salvigenin	Prostaglandin G/H synthase 1	PTGS1
MOL002915	Salvigenin	Prostaglandin G/H synthase 2	PTGS2
MOL002915	Salvigenin	Retinoic acid receptor RXR-alpha	RXRA
MOL002915	Salvigenin	Sodium channel protein type 5 subunit alpha	SCN5A
MOL002917	5,2',6'-Trihydroxy-7,8-dimethoxyflavone	Androgen receptor	AR
MOL002917	5,2',6'-Trihydroxy-7,8-dimethoxyflavone	Serine/threonine-protein kinase Chk1	CHEK1
MOL002917	5,2',6'-Trihydroxy-7,8-dimethoxyflavone	Estrogen receptor beta	ESR2
MOL002917	5,2',6'-Trihydroxy-7,8-dimethoxyflavone	Nuclear receptor coactivator 2	NCOA2
MOL002917	5,2',6'-Trihydroxy-7,8-dimethoxyflavone	Nitric oxide synthase, inducible	NOS2
MOL002917	5,2',6'-Trihydroxy-7,8-dimethoxyflavone	Prostaglandin G/H synthase 1	PTGS1
MOL002917	5,2',6'-Trihydroxy-7,8-dimethoxyflavone	Prostaglandin G/H synthase 2	PTGS2
MOL002917	5,2',6'-Trihydroxy-7,8-dimethoxyflavone	Sodium channel protein type 5 subunit alpha	SCN5A
MOL002925	5,7,2',6'-Tetrahydroxyflavone	Androgen receptor	AR
MOL002925	5,7,2',6'-Tetrahydroxyflavone	Prostaglandin G/H synthase 1	PTGS1
MOL002925	5,7,2',6'-Tetrahydroxyflavone	Prostaglandin G/H synthase 2	PTGS2
MOL002927	Skullcapflavone II	Androgen receptor	AR
MOL002927	Skullcapflavone II	Voltage-dependent calcium channel subunit alpha-2/delta-1	CACNA2D1
MOL002927	Skullcapflavone II	Coagulation factor VII	F7
MOL002927	Skullcapflavone II	Potassium voltage-gated channel subfamily H member 2	KCNH2
MOL002927	Skullcapflavone II	Vascular endothelial growth factor receptor 2	KDR
MOL002927	Skullcapflavone II	Nuclear receptor coactivator 1	NCOA1
MOL002927	Skullcapflavone II	Nuclear receptor coactivator 2	NCOA2
MOL002927	Skullcapflavone II	Nitric oxide synthase, inducible	NOS2
MOL002927	Skullcapflavone II	Prostaglandin G/H synthase 1	PTGS1
MOL002927	Skullcapflavone II	Prostaglandin G/H synthase 2	PTGS2
MOL002927	Skullcapflavone II	Sodium channel protein type 5 subunit alpha	SCN5A
MOL002928	oroxylin a	Alpha-1B adrenergic receptor	ADRA1B
MOL002928	oroxylin a	Beta-2 adrenergic receptor	ADRB2
MOL002928	oroxylin a	Androgen receptor	AR
MOL002928	oroxylin a	Apoptosis regulator Bcl-2	BCL2
MOL002928	oroxylin a	Caspase-3	CASP3
MOL002928	oroxylin a	G2/mitotic-specific cyclin-B1	CCNB1
MOL002928	oroxylin a	Cytochrome P450 1A2	CYP1A2
MOL002928	oroxylin a	Cytochrome P450 2C9	CYP2C9
MOL002928	oroxylin a	Interleukin-6	IL6
MOL002928	oroxylin a	Nuclear receptor coactivator 1	NCOA1
MOL002928	oroxylin a	Nuclear receptor coactivator 2	NCOA2
MOL002928	oroxylin a	Nitric oxide synthase, inducible	NOS2
MOL002928	oroxylin a	CGMP-inhibited 3',5'-cyclic phosphodiesterase A	PDE3A
MOL002928	oroxylin a	cAMP-dependent protein kinase inhibitor alpha	PKIA
MOL002928	oroxylin a	Prostaglandin G/H synthase 1	PTGS1
MOL002928	oroxylin a	Prostaglandin G/H synthase 2	PTGS2
MOL002928	oroxylin a	Retinoic acid receptor RXR-alpha	RXRA
MOL002928	oroxylin a	Sodium channel protein type 5 subunit alpha	SCN5A
MOL002932	Panicolin	Androgen receptor	AR
MOL002932	Panicolin	Serine/threonine-protein kinase Chk1	CHEK1
MOL002932	Panicolin	Estrogen receptor beta	ESR2
MOL002932	Panicolin	Nuclear receptor coactivator 1	NCOA1
MOL002932	Panicolin	Nitric oxide synthase, inducible	NOS2
MOL002932	Panicolin	Prostaglandin G/H synthase 1	PTGS1
MOL002932	Panicolin	Prostaglandin G/H synthase 2	PTGS2
MOL002932	Panicolin	Sodium channel protein type 5 subunit alpha	SCN5A
MOL002933	5,7,4'-Trihydroxy-8-methoxyflavone	Androgen receptor	AR
MOL002933	5,7,4'-Trihydroxy-8-methoxyflavone	Serine/threonine-protein kinase Chk1	CHEK1
MOL002933	5,7,4'-Trihydroxy-8-methoxyflavone	Estrogen receptor	ESR1
MOL002933	5,7,4'-Trihydroxy-8-methoxyflavone	Glycogen synthase kinase-3 beta	GSK3B
MOL002933	5,7,4'-Trihydroxy-8-methoxyflavone	Mitogen-activated protein kinase 14	MAPK14
MOL002933	5,7,4'-Trihydroxy-8-methoxyflavone	Nuclear receptor coactivator 2	NCOA2
MOL002933	5,7,4'-Trihydroxy-8-methoxyflavone	Nitric oxide synthase, inducible	NOS2
MOL002933	5,7,4'-Trihydroxy-8-methoxyflavone	Prostaglandin G/H synthase 1	PTGS1
MOL002933	5,7,4'-Trihydroxy-8-methoxyflavone	Prostaglandin G/H synthase 2	PTGS2
MOL002933	5,7,4'-Trihydroxy-8-methoxyflavone	Glycogen phosphorylase, muscle form	PYGM
MOL002934	NEOBAICALEIN	Androgen receptor	AR
MOL002934	NEOBAICALEIN	Serine/threonine-protein kinase Chk1	CHEK1
MOL002934	NEOBAICALEIN	Estrogen receptor	ESR1
MOL002934	NEOBAICALEIN	Estrogen receptor beta	ESR2
MOL002934	NEOBAICALEIN	Coagulation factor VII	F7
MOL002934	NEOBAICALEIN	Glycogen synthase kinase-3 beta	GSK3B
MOL002934	NEOBAICALEIN	Potassium voltage-gated channel subfamily H member 2	KCNH2
MOL002934	NEOBAICALEIN	Nuclear receptor coactivator 2	NCOA2
MOL002934	NEOBAICALEIN	Nitric oxide synthase, inducible	NOS2

Targets related to Huangqin

MolId	MolName	Target	Symbol
MOL002934	NEOBAICALEIN	Prostaglandin G/H synthase 2	PTGS2
MOL002934	NEOBAICALEIN	Glycogen phosphorylase, muscle form	PYGM
MOL002934	NEOBAICALEIN	Sodium channel protein type 5 subunit alpha	SCN5A
MOL002937	DIHYDROOROXYLIN	Alpha-1B adrenergic receptor	ADRA1B
MOL002937	DIHYDROOROXYLIN	Beta-2 adrenergic receptor	ADRB2
MOL002937	DIHYDROOROXYLIN	Nuclear receptor coactivator 1	NCOA1
MOL002937	DIHYDROOROXYLIN	CGMP-inhibited 3',5'-cyclic phosphodiesterase A	PDE3A
MOL002937	DIHYDROOROXYLIN	Prostaglandin G/H synthase 1	PTGS1
MOL002937	DIHYDROOROXYLIN	Prostaglandin G/H synthase 2	PTGS2
MOL002937	DIHYDROOROXYLIN	Retinoic acid receptor RXR-alpha	RXRA
MOL002937	DIHYDROOROXYLIN	Sodium channel protein type 5 subunit alpha	SCN5A
MOL008206	Moslosooflavone	Alpha-1B adrenergic receptor	ADRA1B
MOL008206	Moslosooflavone	Beta-2 adrenergic receptor	ADRB2
MOL008206	Moslosooflavone	Androgen receptor	AR
MOL008206	Moslosooflavone	Serine/threonine-protein kinase Chk1	CHEK1
MOL008206	Moslosooflavone	Estrogen receptor beta	ESR2
MOL008206	Moslosooflavone	Gamma-aminobutyric acid receptor subunit alpha-1	GABRA1
MOL008206	Moslosooflavone	Glycogen synthase kinase-3 beta	GSK3B
MOL008206	Moslosooflavone	Mitogen-activated protein kinase 14	MAPK14
MOL008206	Moslosooflavone	Nuclear receptor coactivator 1	NCOA1
MOL008206	Moslosooflavone	Nitric oxide synthase, inducible	NOS2
MOL008206	Moslosooflavone	Prostaglandin G/H synthase 1	PTGS1
MOL008206	Moslosooflavone	Prostaglandin G/H synthase 2	PTGS2
MOL008206	Moslosooflavone	Retinoic acid receptor RXR-alpha	RXRA
MOL008206	Moslosooflavone	Sodium channel protein type 5 subunit alpha	SCN5A
MOL010415	11,13-Eicosadienoic acid, methyl ester	Nuclear receptor coactivator 2	NCOA2
MOL012245	5,7,4'-trihydroxy-6-methoxyflavanone	Prostaglandin G/H synthase 1	PTGS1
MOL012245	5,7,4'-trihydroxy-6-methoxyflavanone	Prostaglandin G/H synthase 2	PTGS2
MOL012246	5,7,4'-trihydroxy-8-methoxyflavanone	Prostaglandin G/H synthase 1	PTGS1
MOL012246	5,7,4'-trihydroxy-8-methoxyflavanone	Prostaglandin G/H synthase 2	PTGS2
MOL012266	rivularin	Androgen receptor	AR
MOL012266	rivularin	Estrogen receptor beta	ESR2
MOL012266	rivularin	Coagulation factor VII	F7
MOL012266	rivularin	Potassium voltage-gated channel subfamily H member 2	KCNH2
MOL012266	rivularin	Vascular endothelial growth factor receptor 2	KDR
MOL012266	rivularin	Nuclear receptor coactivator 1	NCOA1
MOL012266	rivularin	Nuclear receptor coactivator 2	NCOA2
MOL012266	rivularin	Nitric oxide synthase, inducible	NOS2
MOL012266	rivularin	Prostaglandin G/H synthase 1	PTGS1
MOL012266	rivularin	Prostaglandin G/H synthase 2	PTGS2
MOL012266	rivularin	Retinoic acid receptor RXR-alpha	RXRA
MOL012266	rivularin	Sodium channel protein type 5 subunit alpha	SCN5A

Targets related to Baishao

MolId	MolName	Target	Symbol
MOL000211	Mairin	Progesterone receptor	PGR
MOL000358	beta-sitosterol	Alpha-1A adrenergic receptor	ADRA1A
MOL000358	beta-sitosterol	Alpha-1B adrenergic receptor	ADRA1B
MOL000358	beta-sitosterol	Beta-2 adrenergic receptor	ADRB2
MOL000358	beta-sitosterol	Apoptosis regulator BAX	BAX
MOL000358	beta-sitosterol	Apoptosis regulator Bcl-2	BCL2
MOL000358	beta-sitosterol	Caspase-3	CASP3
MOL000358	beta-sitosterol	Caspase-8	CASP8
MOL000358	beta-sitosterol	Caspase-9	CASP9
MOL000358	beta-sitosterol	Muscarinic acetylcholine receptor M1	CHRM1
MOL000358	beta-sitosterol	Muscarinic acetylcholine receptor M2	CHRM2
MOL000358	beta-sitosterol	Muscarinic acetylcholine receptor M3	CHRM3
MOL000358	beta-sitosterol	Muscarinic acetylcholine receptor M4	CHRM4
MOL000358	beta-sitosterol	Neuronal acetylcholine receptor subunit alpha-2	CHRNA2
MOL000358	beta-sitosterol	Gamma-aminobutyric acid receptor subunit alpha-1	GABRA1
MOL000358	beta-sitosterol	Potassium voltage-gated channel subfamily H member 2	KCNH2
MOL000358	beta-sitosterol	Microtubule-associated protein 2	MAP2
MOL000358	beta-sitosterol	Nuclear receptor coactivator 2	NCOA2
MOL000358	beta-sitosterol	Mu-type opioid receptor	OPRM1
MOL000358	beta-sitosterol	CGMP-inhibited 3',5'-cyclic phosphodiesterase A	PDE3A
MOL000358	beta-sitosterol	Progesterone receptor	PGR
MOL000358	beta-sitosterol	Serum paraoxonase/arylesterase 1	PON1
MOL000358	beta-sitosterol	Protein kinase C alpha type	PRKCA
MOL000358	beta-sitosterol	Prostaglandin G/H synthase 1	PTGS1
MOL000358	beta-sitosterol	Prostaglandin G/H synthase 2	PTGS2
MOL000358	beta-sitosterol	Sodium channel protein type 5 subunit alpha	SCN5A
MOL000358	beta-sitosterol	Sodium-dependent serotonin transporter	SLC6A4
MOL000359	sitosterol	Nuclear receptor coactivator 2	NCOA2
MOL000359	sitosterol	Mineralocorticoid receptor	NR3C2
MOL000359	sitosterol	Progesterone receptor	PGR
MOL000422	kaempferol	Acetylcholinesterase	ACHE
MOL000422	kaempferol	Alpha-1B adrenergic receptor	ADRA1B
MOL000422	kaempferol	Aryl hydrocarbon receptor	AHR
MOL000422	kaempferol	Activator of 90 kDa heat shock protein ATPase homolog 1	AHSA1
MOL000422	kaempferol	Aldo-keto reductase family 1 member C3	AKR1C3
MOL000422	kaempferol	RAC-alpha serine/threonine-protein kinase	AKT1
MOL000422	kaempferol	Androgen receptor	AR
MOL000422	kaempferol	Apoptosis regulator BAX	BAX
MOL000422	kaempferol	Apoptosis regulator Bcl-2	BCL2
MOL000422	kaempferol	Caspase-3	CASP3
MOL000422	kaempferol	Muscarinic acetylcholine receptor M1	CHRM1
MOL000422	kaempferol	Muscarinic acetylcholine receptor M2	CHRM2
MOL000422	kaempferol	Cytochrome P450 1A1	CYP1A1
MOL000422	kaempferol	Cytochrome P450 1A2	CYP1A2
MOL000422	kaempferol	Cytochrome P450 1B1	CYP1B1
MOL000422	kaempferol	Cytochrome P450 3A4	CYP3A4
MOL000422	kaempferol	Type I iodothyronine deiodinase	DIO1
MOL000422	kaempferol	Coagulation factor VII	F7
MOL000422	kaempferol	Gamma-aminobutyric acid receptor subunit alpha-1	GABRA1
MOL000422	kaempferol	Glutathione S-transferase Mu 1	GSTM1
MOL000422	kaempferol	Glutathione S-transferase Mu 2	GSTM2
MOL000422	kaempferol	Glutathione S-transferase P	GSTP1
MOL000422	kaempferol	Hyaluronan synthase 2	HAS2
MOL000422	kaempferol	Heme oxygenase 1	HMOX1
MOL000422	kaempferol	Intercellular adhesion molecule 1	ICAM1
MOL000422	kaempferol	Inhibitor of nuclear factor kappa-B kinase subunit beta	IKBKB
MOL000422	kaempferol	Insulin receptor	INSR
MOL000422	kaempferol	Mitogen-activated protein kinase 8	MAPK8
MOL000422	kaempferol	Interstitial collagenase	MMP1
MOL000422	kaempferol	Nuclear receptor coactivator 2	NCOA2
MOL000422	kaempferol	Nitric oxide synthase, inducible	NOS2
MOL000422	kaempferol	Nuclear receptor subfamily 1 group I member 2	NR1I2
MOL000422	kaempferol	Nuclear receptor subfamily 1 group I member 3	NR1I3
MOL000422	kaempferol	Progesterone receptor	PGR
MOL000422	kaempferol	Peroxisome proliferator-activated receptor gamma	PPARG
MOL000422	kaempferol	Serine/threonine-protein phosphatase 2B catalytic subunit alpha isoform	PPP3CA
MOL000422	kaempferol	26S proteasome non-ATPase regulatory subunit 3	PSMD3
MOL000422	kaempferol	Prostaglandin G/H synthase 1	PTGS1
MOL000422	kaempferol	Prostaglandin G/H synthase 2	PTGS2
MOL000422	kaempferol	Transcription factor p65	RELA
MOL000422	kaempferol	E-selectin	SELE
MOL000422	kaempferol	Solute carrier family 2, facilitated glucose transporter member 4	SLC2A4
MOL000422	kaempferol	Sodium-dependent noradrenaline transporter	SLC6A2
MOL000422	kaempferol	Antileukoproteinase	SLPI
MOL000422	kaempferol	Signal transducer and activator of transcription 1-alpha/beta	STAT1
MOL000422	kaempferol	Tumor necrosis factor	TNF
MOL000422	kaempferol	Vascular cell adhesion protein 1	VCAM1
MOL000422	kaempferol	Xanthine dehydrogenase/oxidase	XDH
MOL000492	(+)-catechin	Catalase	CAT
MOL000492	(+)-catechin	Estrogen receptor	ESR1
MOL000492	(+)-catechin	Hyaluronan synthase 2	HAS2
MOL000492	(+)-catechin	Nuclear receptor coactivator 2	NCOA2
MOL000492	(+)-catechin	Prostaglandin G/H synthase 1	PTGS1
MOL000492	(+)-catechin	Prostaglandin G/H synthase 2	PTGS2
MOL000492	(+)-catechin	Retinoic acid receptor RXR-alpha	RXRA
MOL001918	paeoniflorgenone	Gamma-aminobutyric acid receptor subunit alpha-1	GABRA1
MOL001919	(3S,5R,8R,9R,10S,14S)-3,17-dihydroxy-4,4,8,10,14-pentamethyl-2,3,5,6,7,9-hexahydro-1H-cyclopenta[a]phenanthrene-15,16-dione	Mineralocorticoid receptor	NR3C2
MOL001919	(3S,5R,8R,9R,10S,14S)-3,17-dihydroxy-4,4,8,10,14-pentamethyl-2,3,5,6,7,9-hexahydro-1H-cyclopenta[a]phenanthrene-15,16-dione	Progesterone receptor	PGR
MOL001924	paeoniflorin	Monocyte differentiation antigen CD14	CD14
MOL001924	paeoniflorin	Interleukin-6	IL6

Targets related to Baishao

MolId	MolName	Target	Symbol
MOL001924	paeoniflorin	Lipopolysaccharide-binding protein	LBP
MOL001924	paeoniflorin	Tumor necrosis factor	TNF

Targets related to Gancao

MolId	MolName	Target	Symbol
MOL000098	quercetin	Acetyl-CoA carboxylase 1	ACACA
MOL000098	quercetin	Acetylcholinesterase	ACHE
MOL000098	quercetin	Prostatic acid phosphatase	ACP3
MOL000098	quercetin	Beta-2 adrenergic receptor	ADRB2
MOL000098	quercetin	Aryl hydrocarbon receptor	AHR
MOL000098	quercetin	Activator of 90 kDa heat shock protein ATPase homolog 1	AHSA1
MOL000098	quercetin	RAC-alpha serine/threonine-protein kinase	AKT1
MOL000098	quercetin	Androgen receptor	AR
MOL000098	quercetin	Apoptosis regulator BAX	BAX
MOL000098	quercetin	Apoptosis regulator Bcl-2	BCL2
MOL000098	quercetin	Bcl-2-like protein 1	BCL2L1
MOL000098	quercetin	Baculoviral IAP repeat-containing protein 5	BIRC5
MOL000098	quercetin	Caspase-3	CASP3
MOL000098	quercetin	Caspase-8	CASP8
MOL000098	quercetin	Caspase-9	CASP9
MOL000098	quercetin	Caveolin-1	CAV1
MOL000098	quercetin	C-C motif chemokine 2	CCL2
MOL000098	quercetin	G2/mitotic-specific cyclin-B1	CCNB1
MOL000098	quercetin	G1/S-specific cyclin-D1	CCND1
MOL000098	quercetin	CD40 ligand	CD40LG
MOL000098	quercetin	Cyclin-dependent kinase inhibitor 1	CDKN1A
MOL000098	quercetin	Serine/threonine-protein kinase Chk2	CHEK2
MOL000098	quercetin	Inhibitor of nuclear factor kappa-B kinase subunit alpha	CHUK
MOL000098	quercetin	Claudin-4	CLDN4
MOL000098	quercetin	C-reactive protein	CRP
MOL000098	quercetin	Cathepsin D	CTSD
MOL000098	quercetin	C-X-C motif chemokine 10	CXCL10
MOL000098	quercetin	C-X-C motif chemokine 11	CXCL11
MOL000098	quercetin	C-X-C motif chemokine 2	CXCL2
MOL000098	quercetin	Interleukin-8	CXCL8
MOL000098	quercetin	Cytochrome P450 1A1	CYP1A1
MOL000098	quercetin	Cytochrome P450 1A2	CYP1A2
MOL000098	quercetin	Cytochrome P450 1B1	CYP1B1
MOL000098	quercetin	Cytochrome P450 3A4	CYP3A4
MOL000098	quercetin	DDB1- and CUL4-associated factor 5	DCAF5
MOL000098	quercetin	Type I iodothyronine deiodinase	DIO1
MOL000098	quercetin	Dual oxidase 2	DUOX2
MOL000098	quercetin	Transcription factor E2F1	E2F1
MOL000098	quercetin	Transcription factor E2F2	E2F2
MOL000098	quercetin	Pro-epidermal growth factor	EGF
MOL000098	quercetin	Epidermal growth factor receptor	EGFR
MOL000098	quercetin	Eukaryotic translation initiation factor 6	EIF6
MOL000098	quercetin	ETS domain-containing protein Elk-1	ELK1
MOL000098	quercetin	Receptor tyrosine-protein kinase erbB-2	ERBB2
MOL000098	quercetin	Receptor tyrosine-protein kinase erbB-3	ERBB3
MOL000098	quercetin	Tissue factor	F3
MOL000098	quercetin	Coagulation factor VII	F7
MOL000098	quercetin	Gamma-aminobutyric acid receptor subunit alpha-1	GABRA1
MOL000098	quercetin	Gap junction alpha-1 protein	GJA1
MOL000098	quercetin	Glutathione S-transferase Mu 1	GSTM1
MOL000098	quercetin	Glutathione S-transferase Mu 2	GSTM2
MOL000098	quercetin	Glutathione S-transferase P	GSTP1
MOL000098	quercetin	Hyaluronan synthase 2	HAS2
MOL000098	quercetin	Hypoxia-inducible factor 1-alpha	HIF1A
MOL000098	quercetin	Hexokinase-2	HK2
MOL000098	quercetin	Heme oxygenase 1	HMOX1
MOL000098	quercetin	Heat shock factor protein 1	HSF1
MOL000098	quercetin	Heat shock protein beta-1	HSPB1
MOL000098	quercetin	Intercellular adhesion molecule 1	ICAM1
MOL000098	quercetin	Interferon gamma	IFNG
MOL000098	quercetin	Insulin-like growth factor II	IGF2
MOL000098	quercetin	Insulin-like growth factor-binding protein 3	IGFBP3
MOL000098	quercetin	Interleukin-10	IL10
MOL000098	quercetin	Interleukin-1 alpha	IL1A
MOL000098	quercetin	Interleukin-1 beta	IL1B
MOL000098	quercetin	Interleukin-2	IL2
MOL000098	quercetin	Interleukin-6	IL6
MOL000098	quercetin	Insulin receptor	INSR
MOL000098	quercetin	Interferon regulatory factor 1	IRF1
MOL000098	quercetin	Potassium voltage-gated channel subfamily H member 2	KCNH2
MOL000098	quercetin	Mitogen-activated protein kinase 1	MAPK1
MOL000098	quercetin	Interstitial collagenase	MMP1
MOL000098	quercetin	72 kDa type IV collagenase	MMP2
MOL000098	quercetin	Stromelysin-1	MMP3
MOL000098	quercetin	Matrix metalloproteinase-9	MMP9
MOL000098	quercetin	Myeloperoxidase	MPO
MOL000098	quercetin	Myc proto-oncogene protein	MYC
MOL000098	quercetin	Neutrophil cytosol factor 1	NCF1
MOL000098	quercetin	Nuclear receptor coactivator 2	NCOA2
MOL000098	quercetin	Nuclear factor erythroid 2-related factor 2	NFE2L2
MOL000098	quercetin	NF-kappa-B inhibitor alpha	NFKBIA
MOL000098	quercetin	Homeobox protein Nkx-3.1	NKX3-1
MOL000098	quercetin	Nitric oxide synthase, endothelial	NOS3
MOL000098	quercetin	Puromycin-sensitive aminopeptidase	NPEPPS
MOL000098	quercetin	Nuclear receptor subfamily 1 group I member 2	NR1I2
MOL000098	quercetin	Nuclear receptor subfamily 1 group I member 3	NR1I3
MOL000098	quercetin	Ornithine decarboxylase	ODC1
MOL000098	quercetin	Procollagen C-endopeptidase enhancer 1	PCOLCE
MOL000098	quercetin	Tissue-type plasminogen activator	PLAT
MOL000098	quercetin	Urokinase-type plasminogen activator	PLAU
MOL000098	quercetin	Serum paraoxonase/arylesterase 1	PON1
MOL000098	quercetin	NADPH--cytochrome P450 reductase	POR

Targets related to Gancao

MolId	MolName	Target	Symbol
MOL000098	quercetin	Peroxisome proliferator-activated receptor alpha	PPARA
MOL000098	quercetin	Peroxisome proliferator-activated receptor delta	PPARD
MOL000098	quercetin	Peroxisome proliferator-activated receptor gamma	PPARG
MOL000098	quercetin	Protein kinase C alpha type	PRKCA
MOL000098	quercetin	Protein kinase C beta type	PRKCB
MOL000098	quercetin	26S proteasome non-ATPase regulatory subunit 3	PSMD3
MOL000098	quercetin	Prostaglandin E2 receptor EP3 subtype	PTGER3
MOL000098	quercetin	Prostaglandin G/H synthase 1	PTGS1
MOL000098	quercetin	Prostaglandin G/H synthase 2	PTGS2
MOL000098	quercetin	RAF proto-oncogene serine/threonine-protein kinase	RAF1
MOL000098	quercetin	Ras GTPase-activating protein 1	RASA1
MOL000098	quercetin	Ras association domain-containing protein 1	RASSF1
MOL000098	quercetin	Retinoblastoma-associated protein	RB1
MOL000098	quercetin	Transcription factor p65	RELA
MOL000098	quercetin	Protein CBFA2T1	RUNX1T1
MOL000098	quercetin	Runt-related transcription factor 2	RUNX2
MOL000098	quercetin	Retinoic acid receptor RXR-alpha	RXRA
MOL000098	quercetin	Sodium channel protein type 5 subunit alpha	SCN5A
MOL000098	quercetin	E-selectin	SELE
MOL000098	quercetin	Plasminogen activator inhibitor 1	SERPINE1
MOL000098	quercetin	Solute carrier family 2, facilitated glucose transporter member 4	SLC2A4
MOL000098	quercetin	Osteopontin	SPP1
MOL000098	quercetin	Signal transducer and activator of transcription 1-alpha/beta	STAT1
MOL000098	quercetin	Thrombomodulin	THBD
MOL000098	quercetin	Tumor necrosis factor	TNF
MOL000098	quercetin	DNA topoisomerase 1	TOP1
MOL000098	quercetin	DNA topoisomerase 2-alpha	TOP2A
MOL000098	quercetin	Cellular tumor antigen p53	TP53
MOL000098	quercetin	Vascular cell adhesion protein 1	VCAM1
MOL000098	quercetin	Vascular endothelial growth factor A	VEGFA
MOL000098	quercetin	Xanthine dehydrogenase/oxidase	XDH
MOL000211	Mairin	Progesterone receptor	PGR
MOL000239	Jaranol	Androgen receptor	AR
MOL000239	Jaranol	Serine/threonine-protein kinase Chk1	CHEK1
MOL000239	Jaranol	Estrogen receptor beta	ESR2
MOL000239	Jaranol	Nuclear receptor coactivator 2	NCOA2
MOL000239	Jaranol	Nitric oxide synthase, inducible	NOS2
MOL000239	Jaranol	Prostaglandin G/H synthase 1	PTGS1
MOL000239	Jaranol	Prostaglandin G/H synthase 2	PTGS2
MOL000239	Jaranol	Sodium channel protein type 5 subunit alpha	SCN5A
MOL000354	isorhamnetin	Acetylcholinesterase	ACHE
MOL000354	isorhamnetin	Androgen receptor	AR
MOL000354	isorhamnetin	Cyclin-A2	CCNA2
MOL000354	isorhamnetin	Serine/threonine-protein kinase Chk1	CHEK1
MOL000354	isorhamnetin	Estrogen receptor	ESR1
MOL000354	isorhamnetin	Estrogen receptor beta	ESR2
MOL000354	isorhamnetin	Coagulation factor VII	F7
MOL000354	isorhamnetin	Gamma-aminobutyric acid receptor subunit alpha-1	GABRA1
MOL000354	isorhamnetin	Glutamate receptor 2	GRIA2
MOL000354	isorhamnetin	Glycogen synthase kinase-3 beta	GSK3B
MOL000354	isorhamnetin	Mitogen-activated protein kinase 14	MAPK14
MOL000354	isorhamnetin	Neutrophil cytosol factor 1	NCF1
MOL000354	isorhamnetin	Nuclear receptor coactivator 1	NCOA1
MOL000354	isorhamnetin	Nuclear receptor coactivator 2	NCOA2
MOL000354	isorhamnetin	Nitric oxide synthase, inducible	NOS2
MOL000354	isorhamnetin	Oxidized low-density lipoprotein receptor 1	OLR1
MOL000354	isorhamnetin	Prostaglandin G/H synthase 1	PTGS1
MOL000354	isorhamnetin	Prostaglandin G/H synthase 2	PTGS2
MOL000354	isorhamnetin	Glycogen phosphorylase, muscle form	PYGM
MOL000354	isorhamnetin	Transcription factor p65	RELA
MOL000354	isorhamnetin	Xanthine dehydrogenase/oxidase	XDH
MOL000359	sitosterol	Nuclear receptor coactivator 2	NCOA2
MOL000359	sitosterol	Mineralocorticoid receptor	NR3C2
MOL000359	sitosterol	Progesterone receptor	PGR
MOL000392	formononetin	Acetylcholinesterase	ACHE
MOL000392	formononetin	Alpha-1A adrenergic receptor	ADRA1A
MOL000392	formononetin	Beta-2 adrenergic receptor	ADRB2
MOL000392	formononetin	Androgen receptor	AR
MOL000392	formononetin	ATP synthase subunit beta, mitochondrial	ATP5F1B
MOL000392	formononetin	Cyclin-A2	CCNA2
MOL000392	formononetin	Serine/threonine-protein kinase Chk1	CHEK1
MOL000392	formononetin	Muscarinic acetylcholine receptor M1	CHRM1
MOL000392	formononetin	Estrogen receptor	ESR1
MOL000392	formononetin	Estrogen receptor beta	ESR2
MOL000392	formononetin	Glycogen synthase kinase-3 beta	GSK3B
MOL000392	formononetin	3 beta-hydroxysteroid dehydrogenase/Delta 5-->4-isomerase type 1	HSD3B1
MOL000392	formononetin	3 beta-hydroxysteroid dehydrogenase/Delta 5-->4-isomerase type 2	HSD3B2
MOL000392	formononetin	Interleukin-4	IL4
MOL000392	formononetin	Mitogen-activated protein kinase 14	MAPK14
MOL000392	formononetin	NADH-ubiquinone oxidoreductase chain 6	MT-ND6
MOL000392	formononetin	Nitric oxide synthase, inducible	NOS2
MOL000392	formononetin	CGMP-inhibited 3',5'-cyclic phosphodiesterase A	PDE3A
MOL000392	formononetin	cAMP-dependent protein kinase inhibitor alpha	PKIA
MOL000392	formononetin	Peroxisome proliferator-activated receptor gamma	PPARG
MOL000392	formononetin	Prostaglandin G/H synthase 1	PTGS1
MOL000392	formononetin	Prostaglandin G/H synthase 2	PTGS2
MOL000392	formononetin	Retinoic acid receptor RXR-alpha	RXRA
MOL000392	formononetin	Sodium-dependent dopamine transporter	SLC6A3
MOL000392	formononetin	Sodium-dependent serotonin transporter	SLC6A4
MOL000417	Calycosin	Beta-2 adrenergic receptor	ADRB2
MOL000417	Calycosin	Androgen receptor	AR
MOL000417	Calycosin	Cyclin-A2	CCNA2

Targets related to Gancao

MolId	MolName	Target	Symbol
MOL000417	Calycosin	Serine/threonine-protein kinase Chk1	CHEK1
MOL000417	Calycosin	Estrogen receptor	ESR1
MOL000417	Calycosin	Estrogen receptor beta	ESR2
MOL000417	Calycosin	Glycogen synthase kinase-3 beta	GSK3B
MOL000417	Calycosin	Mitogen-activated protein kinase 14	MAPK14
MOL000417	Calycosin	Nuclear receptor coactivator 2	NCOA2
MOL000417	Calycosin	Nitric oxide synthase, inducible	NOS2
MOL000417	Calycosin	CGMP-inhibited 3',5'-cyclic phosphodiesterase A	PDE3A
MOL000417	Calycosin	Prostaglandin G/H synthase 1	PTGS1
MOL000417	Calycosin	Prostaglandin G/H synthase 2	PTGS2
MOL000417	Calycosin	Retinoic acid receptor RXR-alpha	RXRA
MOL000422	kaempferol	Acetylcholinesterase	ACHE
MOL000422	kaempferol	Alpha-1B adrenergic receptor	ADRA1B
MOL000422	kaempferol	Aryl hydrocarbon receptor	AHR
MOL000422	kaempferol	Activator of 90 kDa heat shock protein ATPase homolog 1	AHSA1
MOL000422	kaempferol	Aldo-keto reductase family 1 member C3	AKR1C3
MOL000422	kaempferol	RAC-alpha serine/threonine-protein kinase	AKT1
MOL000422	kaempferol	Androgen receptor	AR
MOL000422	kaempferol	Apoptosis regulator BAX	BAX
MOL000422	kaempferol	Apoptosis regulator Bcl-2	BCL2
MOL000422	kaempferol	Caspase-3	CASP3
MOL000422	kaempferol	Muscarinic acetylcholine receptor M1	CHRM1
MOL000422	kaempferol	Muscarinic acetylcholine receptor M2	CHRM2
MOL000422	kaempferol	Cytochrome P450 1A1	CYP1A1
MOL000422	kaempferol	Cytochrome P450 1A2	CYP1A2
MOL000422	kaempferol	Cytochrome P450 1B1	CYP1B1
MOL000422	kaempferol	Cytochrome P450 3A4	CYP3A4
MOL000422	kaempferol	Type I iodothyronine deiodinase	DIO1
MOL000422	kaempferol	Coagulation factor VII	F7
MOL000422	kaempferol	Gamma-aminobutyric acid receptor subunit alpha-1	GABRA1
MOL000422	kaempferol	Glutathione S-transferase Mu 1	GSTM1
MOL000422	kaempferol	Glutathione S-transferase Mu 2	GSTM2
MOL000422	kaempferol	Glutathione S-transferase P	GSTP1
MOL000422	kaempferol	Hyaluronan synthase 2	HAS2
MOL000422	kaempferol	Heme oxygenase 1	HMOX1
MOL000422	kaempferol	Intercellular adhesion molecule 1	ICAM1
MOL000422	kaempferol	Inhibitor of nuclear factor kappa-B kinase subunit beta	IKBKB
MOL000422	kaempferol	Insulin receptor	INSR
MOL000422	kaempferol	Mitogen-activated protein kinase 8	MAPK8
MOL000422	kaempferol	Interstitial collagenase	MMP1
MOL000422	kaempferol	Nuclear receptor coactivator 2	NCOA2
MOL000422	kaempferol	Nitric oxide synthase, inducible	NOS2
MOL000422	kaempferol	Nuclear receptor subfamily 1 group I member 2	NR1I2
MOL000422	kaempferol	Nuclear receptor subfamily 1 group I member 3	NR1I3
MOL000422	kaempferol	Progesterone receptor	PGR
MOL000422	kaempferol	Peroxisome proliferator-activated receptor gamma	PPARG
MOL000422	kaempferol	Serine/threonine-protein phosphatase 2B catalytic subunit alpha isoform	PPP3CA
MOL000422	kaempferol	26S proteasome non-ATPase regulatory subunit 3	PSMD3
MOL000422	kaempferol	Prostaglandin G/H synthase 1	PTGS1
MOL000422	kaempferol	Prostaglandin G/H synthase 2	PTGS2
MOL000422	kaempferol	Transcription factor p65	RELA
MOL000422	kaempferol	E-selectin	SELE
MOL000422	kaempferol	Solute carrier family 2, facilitated glucose transporter member 4	SLC2A4
MOL000422	kaempferol	Sodium-dependent noradrenaline transporter	SLC6A2
MOL000422	kaempferol	Antileukoproteinase	SLPI
MOL000422	kaempferol	Signal transducer and activator of transcription 1-alpha/beta	STAT1
MOL000422	kaempferol	Tumor necrosis factor	TNF
MOL000422	kaempferol	Vascular cell adhesion protein 1	VCAM1
MOL000422	kaempferol	Xanthine dehydrogenase/oxidase	XDH
MOL000497	licochalcone a	Alpha-1B adrenergic receptor	ADRA1B
MOL000497	licochalcone a	Beta-2 adrenergic receptor	ADRB2
MOL000497	licochalcone a	Androgen receptor	AR
MOL000497	licochalcone a	Apoptosis regulator Bcl-2	BCL2
MOL000497	licochalcone a	Cyclin-A2	CCNA2
MOL000497	licochalcone a	G1/S-specific cyclin-D1	CCND1
MOL000497	licochalcone a	Serine/threonine-protein kinase Chk1	CHEK1
MOL000497	licochalcone a	Muscarinic acetylcholine receptor M1	CHRM1
MOL000497	licochalcone a	Eukaryotic translation initiation factor 6	EIF6
MOL000497	licochalcone a	Estrogen receptor	ESR1
MOL000497	licochalcone a	Estrogen receptor beta	ESR2
MOL000497	licochalcone a	Fos-related antigen 2	FOSL2
MOL000497	licochalcone a	Glycogen synthase kinase-3 beta	GSK3B
MOL000497	licochalcone a	Mitogen-activated protein kinase 1	MAPK1
MOL000497	licochalcone a	Mitogen-activated protein kinase 14	MAPK14
MOL000497	licochalcone a	Nuclear receptor coactivator 2	NCOA2
MOL000497	licochalcone a	Nitric oxide synthase, inducible	NOS2
MOL000497	licochalcone a	Prostaglandin G/H synthase 1	PTGS1
MOL000497	licochalcone a	Prostaglandin G/H synthase 2	PTGS2
MOL000497	licochalcone a	Retinoblastoma-associated protein	RB1
MOL000497	licochalcone a	Transcription factor p65	RELA
MOL000497	licochalcone a	Sodium channel protein type 5 subunit alpha	SCN5A
MOL000497	licochalcone a	Sodium-dependent dopamine transporter	SLC6A3
MOL000497	licochalcone a	Signal transducer and activator of transcription 3	STAT3
MOL000500	Vestitol	Alpha-1A adrenergic receptor	ADRA1A
MOL000500	Vestitol	Alpha-1B adrenergic receptor	ADRA1B
MOL000500	Vestitol	Beta-2 adrenergic receptor	ADRB2
MOL000500	Vestitol	Androgen receptor	AR
MOL000500	Vestitol	Cyclin-A2	CCNA2
MOL000500	Vestitol	Serine/threonine-protein kinase Chk1	CHEK1
MOL000500	Vestitol	Muscarinic acetylcholine receptor M1	CHRM1
MOL000500	Vestitol	Muscarinic acetylcholine receptor M4	CHRM4
MOL000500	Vestitol	Estrogen receptor	ESR1

Targets related to Gancao

MolId	MolName	Target	Symbol
MOL000500	Vestitol	Estrogen receptor beta	ESR2
MOL000500	Vestitol	Glycogen synthase kinase-3 beta	GSK3B
MOL000500	Vestitol	Mitogen-activated protein kinase 14	MAPK14
MOL000500	Vestitol	Nitric oxide synthase, inducible	NOS2
MOL000500	Vestitol	CGMP-inhibited 3',5'-cyclic phosphodiesterase A	PDE3A
MOL000500	Vestitol	cAMP-dependent protein kinase inhibitor alpha	PKIA
MOL000500	Vestitol	Prostaglandin G/H synthase 1	PTGS1
MOL000500	Vestitol	Prostaglandin G/H synthase 2	PTGS2
MOL000500	Vestitol	Retinoic acid receptor RXR-alpha	RXRA
MOL000500	Vestitol	Sodium channel protein type 5 subunit alpha	SCN5A
MOL000500	Vestitol	Sodium-dependent dopamine transporter	SLC6A3
MOL000500	Vestitol	Sodium-dependent serotonin transporter	SLC6A4
MOL001484	Inermine	Alpha-1B adrenergic receptor	ADRA1B
MOL001484	Inermine	Alpha-1D adrenergic receptor	ADRA1D
MOL001484	Inermine	Beta-2 adrenergic receptor	ADRB2
MOL001484	Inermine	Muscarinic acetylcholine receptor M1	CHRM1
MOL001484	Inermine	Muscarinic acetylcholine receptor M3	CHRM3
MOL001484	Inermine	5-hydroxytryptamine receptor 3A	HTR3A
MOL001484	Inermine	Mu-type opioid receptor	OPRM1
MOL001484	Inermine	Prostaglandin G/H synthase 1	PTGS1
MOL001484	Inermine	Prostaglandin G/H synthase 2	PTGS2
MOL001484	Inermine	Retinoic acid receptor RXR-alpha	RXRA
MOL001484	Inermine	Sodium channel protein type 5 subunit alpha	SCN5A
MOL001792	DFV	Beta-2 adrenergic receptor	ADRB2
MOL001792	DFV	Estrogen receptor	ESR1
MOL001792	DFV	cAMP-dependent protein kinase inhibitor alpha	PKIA
MOL001792	DFV	Prostaglandin G/H synthase 1	PTGS1
MOL001792	DFV	Prostaglandin G/H synthase 2	PTGS2
MOL001792	DFV	Retinoic acid receptor RXR-alpha	RXRA
MOL001792	DFV	Sodium-dependent serotonin transporter	SLC6A4
MOL002311	Glycyrol	Cyclin-A2	CCNA2
MOL002311	Glycyrol	Serine/threonine-protein kinase Chk1	CHEK1
MOL002311	Glycyrol	Estrogen receptor	ESR1
MOL002311	Glycyrol	Glycogen synthase kinase-3 beta	GSK3B
MOL002311	Glycyrol	Vascular endothelial growth factor receptor 2	KDR
MOL002311	Glycyrol	Mitogen-activated protein kinase 14	MAPK14
MOL002311	Glycyrol	Nitric oxide synthase, inducible	NOS2
MOL002311	Glycyrol	Prostaglandin G/H synthase 2	PTGS2
MOL002565	Medicarpin	Alpha-1A adrenergic receptor	ADRA1A
MOL002565	Medicarpin	Alpha-1B adrenergic receptor	ADRA1B
MOL002565	Medicarpin	Alpha-1D adrenergic receptor	ADRA1D
MOL002565	Medicarpin	Beta-2 adrenergic receptor	ADRB2
MOL002565	Medicarpin	Cyclin-A2	CCNA2
MOL002565	Medicarpin	Muscarinic acetylcholine receptor M1	CHRM1
MOL002565	Medicarpin	Muscarinic acetylcholine receptor M2	CHRM2
MOL002565	Medicarpin	Muscarinic acetylcholine receptor M3	CHRM3
MOL002565	Medicarpin	Muscarinic acetylcholine receptor M4	CHRM4
MOL002565	Medicarpin	Muscarinic acetylcholine receptor M5	CHRM5
MOL002565	Medicarpin	Estrogen receptor	ESR1
MOL002565	Medicarpin	Estrogen receptor beta	ESR2
MOL002565	Medicarpin	Mitogen-activated protein kinase 10	MAPK10
MOL002565	Medicarpin	Nitric oxide synthase, inducible	NOS2
MOL002565	Medicarpin	Delta-type opioid receptor	OPRD1
MOL002565	Medicarpin	Mu-type opioid receptor	OPRM1
MOL002565	Medicarpin	CGMP-inhibited 3',5'-cyclic phosphodiesterase A	PDE3A
MOL002565	Medicarpin	Prostaglandin G/H synthase 1	PTGS1
MOL002565	Medicarpin	Prostaglandin G/H synthase 2	PTGS2
MOL002565	Medicarpin	Retinoic acid receptor RXR-alpha	RXRA
MOL002565	Medicarpin	Sodium channel protein type 5 subunit alpha	SCN5A
MOL002565	Medicarpin	Sodium-dependent dopamine transporter	SLC6A3
MOL002565	Medicarpin	Sodium-dependent serotonin transporter	SLC6A4
MOL003656	Lupiwighteone	Androgen receptor	AR
MOL003656	Lupiwighteone	Cyclin-A2	CCNA2
MOL003656	Lupiwighteone	Serine/threonine-protein kinase Chk1	CHEK1
MOL003656	Lupiwighteone	Estrogen receptor	ESR1
MOL003656	Lupiwighteone	Estrogen receptor beta	ESR2
MOL003656	Lupiwighteone	Glycogen synthase kinase-3 beta	GSK3B
MOL003656	Lupiwighteone	Mitogen-activated protein kinase 14	MAPK14
MOL003656	Lupiwighteone	Nuclear receptor coactivator 2	NCOA2
MOL003656	Lupiwighteone	Nitric oxide synthase, inducible	NOS2
MOL003656	Lupiwighteone	Prostaglandin G/H synthase 2	PTGS2
MOL003656	Lupiwighteone	Sodium channel protein type 5 subunit alpha	SCN5A
MOL003896	7-Methoxy-2-methyl isoflavone	Acetylcholinesterase	ACHE
MOL003896	7-Methoxy-2-methyl isoflavone	Alpha-1B adrenergic receptor	ADRA1B
MOL003896	7-Methoxy-2-methyl isoflavone	Alpha-1D adrenergic receptor	ADRA1D
MOL003896	7-Methoxy-2-methyl isoflavone	Beta-1 adrenergic receptor	ADRB1
MOL003896	7-Methoxy-2-methyl isoflavone	Beta-2 adrenergic receptor	ADRB2
MOL003896	7-Methoxy-2-methyl isoflavone	Androgen receptor	AR
MOL003896	7-Methoxy-2-methyl isoflavone	Cyclin-A2	CCNA2
MOL003896	7-Methoxy-2-methyl isoflavone	Serine/threonine-protein kinase Chk1	CHEK1
MOL003896	7-Methoxy-2-methyl isoflavone	Muscarinic acetylcholine receptor M1	CHRM1
MOL003896	7-Methoxy-2-methyl isoflavone	Muscarinic acetylcholine receptor M3	CHRM3
MOL003896	7-Methoxy-2-methyl isoflavone	Muscarinic acetylcholine receptor M5	CHRM5
MOL003896	7-Methoxy-2-methyl isoflavone	Estrogen receptor	ESR1
MOL003896	7-Methoxy-2-methyl isoflavone	Estrogen receptor beta	ESR2
MOL003896	7-Methoxy-2-methyl isoflavone	Gamma-aminobutyric acid receptor subunit alpha-1	GABRA1
MOL003896	7-Methoxy-2-methyl isoflavone	Glycogen synthase kinase-3 beta	GSK3B
MOL003896	7-Methoxy-2-methyl isoflavone	Leukotriene A-4 hydrolase	LTA4H
MOL003896	7-Methoxy-2-methyl isoflavone	Mitogen-activated protein kinase 14	MAPK14
MOL003896	7-Methoxy-2-methyl isoflavone	Nuclear receptor coactivator 1	NCOA1
MOL003896	7-Methoxy-2-methyl isoflavone	Nuclear receptor coactivator 2	NCOA2
MOL003896	7-Methoxy-2-methyl isoflavone	Nitric oxide synthase, inducible	NOS2

Targets related to Gancao

MolId	MolName	Target	Symbol
MOL003896	7-Methoxy-2-methyl isoflavone	Mu-type opioid receptor	OPRM1
MOL003896	7-Methoxy-2-methyl isoflavone	CGMP-inhibited 3',5'-cyclic phosphodiesterase A	PDE3A
MOL003896	7-Methoxy-2-methyl isoflavone	cAMP-dependent protein kinase inhibitor alpha	PKIA
MOL003896	7-Methoxy-2-methyl isoflavone	Prostaglandin G/H synthase 1	PTGS1
MOL003896	7-Methoxy-2-methyl isoflavone	Prostaglandin G/H synthase 2	PTGS2
MOL003896	7-Methoxy-2-methyl isoflavone	Retinoic acid receptor RXR-alpha	RXRA
MOL003896	7-Methoxy-2-methyl isoflavone	Sodium channel protein type 5 subunit alpha	SCN5A
MOL003896	7-Methoxy-2-methyl isoflavone	Sodium-dependent dopamine transporter	SLC6A3
MOL003896	7-Methoxy-2-methyl isoflavone	Sodium-dependent serotonin transporter	SLC6A4
MOL004328	naringenin	4-aminobutyrate aminotransferase, mitochondrial	ABAT
MOL004328	naringenin	Multidrug resistance-associated protein 1	ABCC1
MOL004328	naringenin	Adiponectin	ADIPOQ
MOL004328	naringenin	Aldo-keto reductase family 1 member C1	AKR1C1
MOL004328	naringenin	RAC-alpha serine/threonine-protein kinase	AKT1
MOL004328	naringenin	Apolipoprotein B-100	APOB
MOL004328	naringenin	Apoptosis regulator Bcl-2	BCL2
MOL004328	naringenin	Caspase-3	CASP3
MOL004328	naringenin	Catalase	CAT
MOL004328	naringenin	Liver carboxylesterase 1	CES1
MOL004328	naringenin	Estrogen receptor	ESR1
MOL004328	naringenin	Fatty acid synthase	FASN
MOL004328	naringenin	Aspartate aminotransferase, cytoplasmic	GOT1
MOL004328	naringenin	Glutathione reductase, mitochondrial	GSR
MOL004328	naringenin	Glutathione S-transferase P	GSTP1
MOL004328	naringenin	3-hydroxy-3-methylglutaryl-coenzyme A reductase	HMGCR
MOL004328	naringenin	Low-density lipoprotein receptor	LDLR
MOL004328	naringenin	Mitogen-activated protein kinase 1	MAPK1
MOL004328	naringenin	Mitogen-activated protein kinase 3	MAPK3
MOL004328	naringenin	Microsomal triglyceride transfer protein large subunit	MTTP
MOL004328	naringenin	Phospholipase B1, membrane-associated	PLB1
MOL004328	naringenin	Peroxisome proliferator-activated receptor alpha	PPARA
MOL004328	naringenin	Peroxisome proliferator-activated receptor gamma	PPARG
MOL004328	naringenin	Prostaglandin G/H synthase 1	PTGS1
MOL004328	naringenin	Prostaglandin G/H synthase 2	PTGS2
MOL004328	naringenin	Transcription factor p65	RELA
MOL004328	naringenin	Sterol O-acyltransferase 1	SOAT1
MOL004328	naringenin	Sterol O-acyltransferase 2	SOAT2
MOL004328	naringenin	Sterol regulatory element-binding protein 1	SREBF1
MOL004805	(2S)-2-[4-hydroxy-3-(3-methylbut-2-enyl)phenyl]-8,8-dimethyl-2,3-dihydropyrano[2,3-f]chromen-4-one	Androgen receptor	AR
MOL004805	(2S)-2-[4-hydroxy-3-(3-methylbut-2-enyl)phenyl]-8,8-dimethyl-2,3-dihydropyrano[2,3-f]chromen-4-one	Estrogen receptor	ESR1
MOL004805	(2S)-2-[4-hydroxy-3-(3-methylbut-2-enyl)phenyl]-8,8-dimethyl-2,3-dihydropyrano[2,3-f]chromen-4-one	Estrogen receptor beta	ESR2
MOL004805	(2S)-2-[4-hydroxy-3-(3-methylbut-2-enyl)phenyl]-8,8-dimethyl-2,3-dihydropyrano[2,3-f]chromen-4-one	Glycogen synthase kinase-3 beta	GSK3B
MOL004805	(2S)-2-[4-hydroxy-3-(3-methylbut-2-enyl)phenyl]-8,8-dimethyl-2,3-dihydropyrano[2,3-f]chromen-4-one	Potassium voltage-gated channel subfamily H member 2	KCNH2
MOL004805	(2S)-2-[4-hydroxy-3-(3-methylbut-2-enyl)phenyl]-8,8-dimethyl-2,3-dihydropyrano[2,3-f]chromen-4-one	Mitogen-activated protein kinase 14	MAPK14
MOL004805	(2S)-2-[4-hydroxy-3-(3-methylbut-2-enyl)phenyl]-8,8-dimethyl-2,3-dihydropyrano[2,3-f]chromen-4-one	Nitric oxide synthase, inducible	NOS2
MOL004805	(2S)-2-[4-hydroxy-3-(3-methylbut-2-enyl)phenyl]-8,8-dimethyl-2,3-dihydropyrano[2,3-f]chromen-4-one	Prostaglandin G/H synthase 2	PTGS2
MOL004806	euchrenone	Estrogen receptor	ESR1
MOL004806	euchrenone	Estrogen receptor beta	ESR2
MOL004806	euchrenone	Potassium voltage-gated channel subfamily H member 2	KCNH2
MOL004806	euchrenone	Nitric oxide synthase, inducible	NOS2
MOL004806	euchrenone	Coagulation factor Xa	PTGS2
MOL004806	euchrenone	Sodium channel protein type 5 subunit alpha	SCN5A
MOL004808	glyasperin B	Acetylcholinesterase	ACHE
MOL004808	glyasperin B	Androgen receptor	AR
MOL004808	glyasperin B	Cyclin-A2	CCNA2
MOL004808	glyasperin B	Estrogen receptor	ESR1
MOL004808	glyasperin B	Estrogen receptor beta	ESR2
MOL004808	glyasperin B	Coagulation factor VII	F7
MOL004808	glyasperin B	Glycogen synthase kinase-3 beta	GSK3B
MOL004808	glyasperin B	Vascular endothelial growth factor receptor 2	KDR
MOL004808	glyasperin B	Nuclear receptor coactivator 2	NCOA2
MOL004808	glyasperin B	Nitric oxide synthase, inducible	NOS2
MOL004808	glyasperin B	Prostaglandin G/H synthase 2	PTGS2
MOL004810	glyasperin F	Androgen receptor	AR
MOL004810	glyasperin F	Cyclin-A2	CCNA2
MOL004810	glyasperin F	Estrogen receptor	ESR1
MOL004810	glyasperin F	Estrogen receptor beta	ESR2
MOL004810	glyasperin F	Glycogen synthase kinase-3 beta	GSK3B
MOL004810	glyasperin F	Mitogen-activated protein kinase 14	MAPK14
MOL004810	glyasperin F	Nitric oxide synthase, inducible	NOS2
MOL004810	glyasperin F	Prostaglandin G/H synthase 1	PTGS1
MOL004810	glyasperin F	Prostaglandin G/H synthase 2	PTGS2
MOL004810	glyasperin F	Sodium channel protein type 5 subunit alpha	SCN5A
MOL004811	Glyasperin C	Acetylcholinesterase	ACHE
MOL004811	Glyasperin C	Androgen receptor	AR
MOL004811	Glyasperin C	Cyclin-A2	CCNA2
MOL004811	Glyasperin C	Serine/threonine-protein kinase Chk1	CHEK1
MOL004811	Glyasperin C	Estrogen receptor	ESR1
MOL004811	Glyasperin C	Estrogen receptor beta	ESR2
MOL004811	Glyasperin C	Glycogen synthase kinase-3 beta	GSK3B
MOL004811	Glyasperin C	Potassium voltage-gated channel subfamily H member 2	KCNH2
MOL004811	Glyasperin C	Mitogen-activated protein kinase 14	MAPK14
MOL004811	Glyasperin C	Nuclear receptor coactivator 2	NCOA2
MOL004811	Glyasperin C	Nitric oxide synthase, inducible	NOS2

Targets related to Gancao

MolId	MolName	Target	Symbol
MOL004811	Glyasperin C	Prostaglandin G/H synthase 2	PTGS2
MOL004811	Glyasperin C	Retinoic acid receptor RXR-alpha	RXRA
MOL004811	Glyasperin C	Sodium channel protein type 5 subunit alpha	SCN5A
MOL004814	Isotrifoliol	Androgen receptor	AR
MOL004814	Isotrifoliol	Cyclin-A2	CCNA2
MOL004814	Isotrifoliol	Serine/threonine-protein kinase Chk1	CHEK1
MOL004814	Isotrifoliol	Estrogen receptor	ESR1
MOL004814	Isotrifoliol	Estrogen receptor beta	ESR2
MOL004814	Isotrifoliol	Glycogen synthase kinase-3 beta	GSK3B
MOL004814	Isotrifoliol	Mitogen-activated protein kinase 14	MAPK14
MOL004814	Isotrifoliol	Nitric oxide synthase, inducible	NOS2
MOL004814	Isotrifoliol	Prostaglandin G/H synthase 2	PTGS2
MOL004815	(E)-1-(2,4-dihydroxyphenyl)-3-(2,2-dimethylchromen-6-yl)prop-2-en-1-one	Alpha-1B adrenergic receptor	ADRA1B
MOL004815	(E)-1-(2,4-dihydroxyphenyl)-3-(2,2-dimethylchromen-6-yl)prop-2-en-1-one	Androgen receptor	AR
MOL004815	(E)-1-(2,4-dihydroxyphenyl)-3-(2,2-dimethylchromen-6-yl)prop-2-en-1-one	Cyclin-A2	CCNA2
MOL004815	(E)-1-(2,4-dihydroxyphenyl)-3-(2,2-dimethylchromen-6-yl)prop-2-en-1-one	Serine/threonine-protein kinase Chk1	CHEK1
MOL004815	(E)-1-(2,4-dihydroxyphenyl)-3-(2,2-dimethylchromen-6-yl)prop-2-en-1-one	Estrogen receptor	ESR1
MOL004815	(E)-1-(2,4-dihydroxyphenyl)-3-(2,2-dimethylchromen-6-yl)prop-2-en-1-one	Estrogen receptor beta	ESR2
MOL004815	(E)-1-(2,4-dihydroxyphenyl)-3-(2,2-dimethylchromen-6-yl)prop-2-en-1-one	Glycogen synthase kinase-3 beta	GSK3B
MOL004815	(E)-1-(2,4-dihydroxyphenyl)-3-(2,2-dimethylchromen-6-yl)prop-2-en-1-one	Mitogen-activated protein kinase 14	MAPK14
MOL004815	(E)-1-(2,4-dihydroxyphenyl)-3-(2,2-dimethylchromen-6-yl)prop-2-en-1-one	Nuclear receptor coactivator 2	NCOA2
MOL004815	(E)-1-(2,4-dihydroxyphenyl)-3-(2,2-dimethylchromen-6-yl)prop-2-en-1-one	Nitric oxide synthase, inducible	NOS2
MOL004815	(E)-1-(2,4-dihydroxyphenyl)-3-(2,2-dimethylchromen-6-yl)prop-2-en-1-one	Prostaglandin G/H synthase 1	PTGS1
MOL004815	(E)-1-(2,4-dihydroxyphenyl)-3-(2,2-dimethylchromen-6-yl)prop-2-en-1-one	Prostaglandin G/H synthase 2	PTGS2
MOL004815	(E)-1-(2,4-dihydroxyphenyl)-3-(2,2-dimethylchromen-6-yl)prop-2-en-1-one	Retinoic acid receptor RXR-alpha	RXRA
MOL004815	(E)-1-(2,4-dihydroxyphenyl)-3-(2,2-dimethylchromen-6-yl)prop-2-en-1-one	Sodium channel protein type 5 subunit alpha	SCN5A
MOL004820	kanzonols W	Androgen receptor	AR
MOL004820	kanzonols W	Cyclin-A2	CCNA2
MOL004820	kanzonols W	Serine/threonine-protein kinase Chk1	CHEK1
MOL004820	kanzonols W	Estrogen receptor	ESR1
MOL004820	kanzonols W	Estrogen receptor beta	ESR2
MOL004820	kanzonols W	Glycogen synthase kinase-3 beta	GSK3B
MOL004820	kanzonols W	Mitogen-activated protein kinase 14	MAPK14
MOL004820	kanzonols W	Nuclear receptor coactivator 1	NCOA1
MOL004820	kanzonols W	Nuclear receptor coactivator 2	NCOA2
MOL004820	kanzonols W	Nitric oxide synthase, inducible	NOS2
MOL004820	kanzonols W	Prostaglandin G/H synthase 1	PTGS1
MOL004820	kanzonols W	Prostaglandin G/H synthase 2	PTGS2
MOL004820	kanzonols W	Retinoic acid receptor RXR-alpha	RXRA
MOL004820	kanzonols W	Sodium channel protein type 5 subunit alpha	SCN5A
MOL004824	(2S)-6-(2,4-dihydroxyphenyl)-2-(2-hydroxypropan-2-yl)-4-methoxy-2,3-dihydrofuro[3,2-g]chromen-7-one	Acetylcholinesterase	ACHE
MOL004824	(2S)-6-(2,4-dihydroxyphenyl)-2-(2-hydroxypropan-2-yl)-4-methoxy-2,3-dihydrofuro[3,2-g]chromen-7-one	Androgen receptor	AR
MOL004824	(2S)-6-(2,4-dihydroxyphenyl)-2-(2-hydroxypropan-2-yl)-4-methoxy-2,3-dihydrofuro[3,2-g]chromen-7-one	Cyclin-A2	CCNA2
MOL004824	(2S)-6-(2,4-dihydroxyphenyl)-2-(2-hydroxypropan-2-yl)-4-methoxy-2,3-dihydrofuro[3,2-g]chromen-7-one	Serine/threonine-protein kinase Chk1	CHEK1
MOL004824	(2S)-6-(2,4-dihydroxyphenyl)-2-(2-hydroxypropan-2-yl)-4-methoxy-2,3-dihydrofuro[3,2-g]chromen-7-one	Estrogen receptor	ESR1
MOL004824	(2S)-6-(2,4-dihydroxyphenyl)-2-(2-hydroxypropan-2-yl)-4-methoxy-2,3-dihydrofuro[3,2-g]chromen-7-one	Estrogen receptor beta	ESR2
MOL004824	(2S)-6-(2,4-dihydroxyphenyl)-2-(2-hydroxypropan-2-yl)-4-methoxy-2,3-dihydrofuro[3,2-g]chromen-7-one	Coagulation factor VII	F7
MOL004824	(2S)-6-(2,4-dihydroxyphenyl)-2-(2-hydroxypropan-2-yl)-4-methoxy-2,3-dihydrofuro[3,2-g]chromen-7-one	Glycogen synthase kinase-3 beta	GSK3B
MOL004824	(2S)-6-(2,4-dihydroxyphenyl)-2-(2-hydroxypropan-2-yl)-4-methoxy-2,3-dihydrofuro[3,2-g]chromen-7-one	Vascular endothelial growth factor receptor 2	KDR
MOL004824	(2S)-6-(2,4-dihydroxyphenyl)-2-(2-hydroxypropan-2-yl)-4-methoxy-2,3-dihydrofuro[3,2-g]chromen-7-one	Mitogen-activated protein kinase 14	MAPK14
MOL004824	(2S)-6-(2,4-dihydroxyphenyl)-2-(2-hydroxypropan-2-yl)-4-methoxy-2,3-dihydrofuro[3,2-g]chromen-7-one	Nitric oxide synthase, inducible	NOS2
MOL004824	(2S)-6-(2,4-dihydroxyphenyl)-2-(2-hydroxypropan-2-yl)-4-methoxy-2,3-dihydrofuro[3,2-g]chromen-7-one	Prostaglandin G/H synthase 2	PTGS2
MOL004827	Semilicoisoflavone B	Acetylcholinesterase	ACHE
MOL004827	Semilicoisoflavone B	Androgen receptor	AR
MOL004827	Semilicoisoflavone B	Serine/threonine-protein kinase Chk1	CHEK1
MOL004827	Semilicoisoflavone B	Estrogen receptor	ESR1
MOL004827	Semilicoisoflavone B	Coagulation factor VII	F7
MOL004827	Semilicoisoflavone B	Glycogen synthase kinase-3 beta	GSK3B
MOL004827	Semilicoisoflavone B	Nitric oxide synthase, inducible	NOS2
MOL004827	Semilicoisoflavone B	Prostaglandin G/H synthase 2	PTGS2
MOL004827	Semilicoisoflavone B	Sodium channel protein type 5 subunit alpha	SCN5A
MOL004828	Glepidotin A	Androgen receptor	AR
MOL004828	Glepidotin A	Cyclin-A2	CCNA2
MOL004828	Glepidotin A	Serine/threonine-protein kinase Chk1	CHEK1
MOL004828	Glepidotin A	Estrogen receptor	ESR1
MOL004828	Glepidotin A	Coagulation factor VII	F7
MOL004828	Glepidotin A	Glycogen synthase kinase-3 beta	GSK3B
MOL004828	Glepidotin A	Vascular endothelial growth factor receptor 2	KDR
MOL004828	Glepidotin A	Mitogen-activated protein kinase 14	MAPK14
MOL004828	Glepidotin A	Nitric oxide synthase, inducible	NOS2
MOL004828	Glepidotin A	CGMP-inhibited 3',5'-cyclic phosphodiesterase A	PDE3A
MOL004828	Glepidotin A	Prostaglandin G/H synthase 1	PTGS1
MOL004828	Glepidotin A	Prostaglandin G/H synthase 2	PTGS2
MOL004828	Glepidotin A	Retinoic acid receptor RXR-alpha	RXRA
MOL004828	Glepidotin A	Sodium channel protein type 5 subunit alpha	SCN5A
MOL004829	Glepidotin B	Alpha-1B adrenergic receptor	ADRA1B
MOL004829	Glepidotin B	Estrogen receptor	ESR1
MOL004829	Glepidotin B	Coagulation factor VII	F7
MOL004829	Glepidotin B	Nuclear receptor coactivator 1	NCOA1
MOL004829	Glepidotin B	CGMP-inhibited 3',5'-cyclic phosphodiesterase A	PDE3A

Targets related to Gancao

MolId	MolName	Target	Symbol
MOL004829	Glepidotin B	Prostaglandin G/H synthase 1	PTGS1
MOL004829	Glepidotin B	Prostaglandin G/H synthase 2	PTGS2
MOL004829	Glepidotin B	Retinoic acid receptor RXR-alpha	RXRA
MOL004829	Glepidotin B	Sodium channel protein type 5 subunit alpha	SCN5A
MOL004833	Phaseolinisoflavan	Acetylcholinesterase	ACHE
MOL004833	Phaseolinisoflavan	Alpha-1B adrenergic receptor	ADRA1B
MOL004833	Phaseolinisoflavan	Beta-2 adrenergic receptor	ADRB2
MOL004833	Phaseolinisoflavan	Androgen receptor	AR
MOL004833	Phaseolinisoflavan	Cyclin-A2	CCNA2
MOL004833	Phaseolinisoflavan	Serine/threonine-protein kinase Chk1	CHEK1
MOL004833	Phaseolinisoflavan	Muscarinic acetylcholine receptor M1	CHRM1
MOL004833	Phaseolinisoflavan	Estrogen receptor	ESR1
MOL004833	Phaseolinisoflavan	Estrogen receptor beta	ESR2
MOL004833	Phaseolinisoflavan	Glycogen synthase kinase-3 beta	GSK3B
MOL004833	Phaseolinisoflavan	Mitogen-activated protein kinase 14	MAPK14
MOL004833	Phaseolinisoflavan	Nuclear receptor coactivator 1	NCOA1
MOL004833	Phaseolinisoflavan	Nitric oxide synthase, inducible	NOS2
MOL004833	Phaseolinisoflavan	Prostaglandin G/H synthase 2	PTGS2
MOL004833	Phaseolinisoflavan	Retinoic acid receptor RXR-alpha	RXRA
MOL004833	Phaseolinisoflavan	Sodium channel protein type 5 subunit alpha	SCN5A
MOL004835	Glypallichalcone	Alpha-1B adrenergic receptor	ADRA1B
MOL004835	Glypallichalcone	Beta-2 adrenergic receptor	ADRB2
MOL004835	Glypallichalcone	Androgen receptor	AR
MOL004835	Glypallichalcone	Cyclin-A2	CCNA2
MOL004835	Glypallichalcone	Serine/threonine-protein kinase Chk1	CHEK1
MOL004835	Glypallichalcone	Muscarinic acetylcholine receptor M1	CHRM1
MOL004835	Glypallichalcone	Estrogen receptor	ESR1
MOL004835	Glypallichalcone	Estrogen receptor beta	ESR2
MOL004835	Glypallichalcone	Glycogen synthase kinase-3 beta	GSK3B
MOL004835	Glypallichalcone	Leukotriene A-4 hydrolase	LTA4H
MOL004835	Glypallichalcone	Mitogen-activated protein kinase 14	MAPK14
MOL004835	Glypallichalcone	Nuclear receptor coactivator 1	NCOA1
MOL004835	Glypallichalcone	Nitric oxide synthase, inducible	NOS2
MOL004835	Glypallichalcone	CGMP-inhibited 3',5'-cyclic phosphodiesterase A	PDE3A
MOL004835	Glypallichalcone	cAMP-dependent protein kinase inhibitor alpha	PKIA
MOL004835	Glypallichalcone	Prostaglandin G/H synthase 1	PTGS1
MOL004835	Glypallichalcone	Prostaglandin G/H synthase 2	PTGS2
MOL004835	Glypallichalcone	Sodium channel protein type 5 subunit alpha	SCN5A
MOL004835	Glypallichalcone	Sodium-dependent dopamine transporter	SLC6A3
MOL004835	Glypallichalcone	Sodium-dependent serotonin transporter	SLC6A4
MOL004838	8-(6-hydroxy-2-benzofuranyl)-2,2-dimethyl-5-chromenol	Estrogen receptor	ESR1
MOL004838	8-(6-hydroxy-2-benzofuranyl)-2,2-dimethyl-5-chromenol	Nitric oxide synthase, inducible	NOS2
MOL004838	8-(6-hydroxy-2-benzofuranyl)-2,2-dimethyl-5-chromenol	Prostaglandin G/H synthase 2	PTGS2
MOL004838	8-(6-hydroxy-2-benzofuranyl)-2,2-dimethyl-5-chromenol	Retinoic acid receptor RXR-alpha	RXRA
MOL004841	Licochalcone B	Beta-2 adrenergic receptor	ADRB2
MOL004841	Licochalcone B	Androgen receptor	AR
MOL004841	Licochalcone B	Cyclin-A2	CCNA2
MOL004841	Licochalcone B	Serine/threonine-protein kinase Chk1	CHEK1
MOL004841	Licochalcone B	Estrogen receptor	ESR1
MOL004841	Licochalcone B	Estrogen receptor beta	ESR2
MOL004841	Licochalcone B	Glycogen synthase kinase-3 beta	GSK3B
MOL004841	Licochalcone B	Mitogen-activated protein kinase 14	MAPK14
MOL004841	Licochalcone B	Nitric oxide synthase, inducible	NOS2
MOL004841	Licochalcone B	CGMP-inhibited 3',5'-cyclic phosphodiesterase A	PDE3A
MOL004841	Licochalcone B	Prostaglandin G/H synthase 1	PTGS1
MOL004841	Licochalcone B	Prostaglandin G/H synthase 2	PTGS2
MOL004848	licochalcone G	Androgen receptor	AR
MOL004848	licochalcone G	Cyclin-A2	CCNA2
MOL004848	licochalcone G	Estrogen receptor	ESR1
MOL004848	licochalcone G	Estrogen receptor beta	ESR2
MOL004848	licochalcone G	Glycogen synthase kinase-3 beta	GSK3B
MOL004848	licochalcone G	Vascular endothelial growth factor receptor 2	KDR
MOL004848	licochalcone G	Mitogen-activated protein kinase 14	MAPK14
MOL004848	licochalcone G	Nuclear receptor coactivator 2	NCOA2
MOL004848	licochalcone G	Nitric oxide synthase, inducible	NOS2
MOL004848	licochalcone G	Prostaglandin G/H synthase 2	PTGS2
MOL004849	3-(2,4-dihydroxyphenyl)-8-(1,1-dimethylprop-2-enyl)-7-hydroxy-5-methoxy-coumarin	Androgen receptor	AR
MOL004849	3-(2,4-dihydroxyphenyl)-8-(1,1-dimethylprop-2-enyl)-7-hydroxy-5-methoxy-coumarin	Serine/threonine-protein kinase Chk1	CHEK1
MOL004849	3-(2,4-dihydroxyphenyl)-8-(1,1-dimethylprop-2-enyl)-7-hydroxy-5-methoxy-coumarin	Estrogen receptor	ESR1
MOL004849	3-(2,4-dihydroxyphenyl)-8-(1,1-dimethylprop-2-enyl)-7-hydroxy-5-methoxy-coumarin	Estrogen receptor beta	ESR2
MOL004849	3-(2,4-dihydroxyphenyl)-8-(1,1-dimethylprop-2-enyl)-7-hydroxy-5-methoxy-coumarin	Coagulation factor VII	F7
MOL004849	3-(2,4-dihydroxyphenyl)-8-(1,1-dimethylprop-2-enyl)-7-hydroxy-5-methoxy-coumarin	Glycogen synthase kinase-3 beta	GSK3B
MOL004849	3-(2,4-dihydroxyphenyl)-8-(1,1-dimethylprop-2-enyl)-7-hydroxy-5-methoxy-coumarin	Potassium voltage-gated channel subfamily H member 2	KCNH2
MOL004849	3-(2,4-dihydroxyphenyl)-8-(1,1-dimethylprop-2-enyl)-7-hydroxy-5-methoxy-coumarin	Vascular endothelial growth factor receptor 2	KDR
MOL004849	3-(2,4-dihydroxyphenyl)-8-(1,1-dimethylprop-2-enyl)-7-hydroxy-5-methoxy-coumarin	Mitogen-activated protein kinase 14	MAPK14
MOL004849	3-(2,4-dihydroxyphenyl)-8-(1,1-dimethylprop-2-enyl)-7-hydroxy-5-methoxy-coumarin	Nuclear receptor coactivator 1	NCOA1
MOL004849	3-(2,4-dihydroxyphenyl)-8-(1,1-dimethylprop-2-enyl)-7-hydroxy-5-methoxy-coumarin	Nuclear receptor coactivator 2	NCOA2
MOL004849	3-(2,4-dihydroxyphenyl)-8-(1,1-dimethylprop-2-enyl)-7-hydroxy-5-methoxy-coumarin	Nitric oxide synthase, inducible	NOS2
MOL004849	3-(2,4-dihydroxyphenyl)-8-(1,1-dimethylprop-2-enyl)-7-hydroxy-5-methoxy-coumarin	Prostaglandin G/H synthase 2	PTGS2

Targets related to Gancao

MolId	MolName	Target	Symbol
MOL004855	Licoricone	Androgen receptor	AR
MOL004855	Licoricone	Serine/threonine-protein kinase Chk1	CHEK1
MOL004855	Licoricone	Estrogen receptor	ESR1
MOL004855	Licoricone	Potassium voltage-gated channel subfamily H member 2	KCNH2
MOL004855	Licoricone	Vascular endothelial growth factor receptor 2	KDR
MOL004855	Licoricone	Nuclear receptor coactivator 2	NCOA2
MOL004855	Licoricone	Nitric oxide synthase, inducible	NOS2
MOL004855	Licoricone	Prostaglandin G/H synthase 2	PTGS2
MOL004856	Gancaonin A	Acetylcholinesterase	ACHE
MOL004856	Gancaonin A	Androgen receptor	AR
MOL004856	Gancaonin A	Cyclin-A2	CCNA2
MOL004856	Gancaonin A	Serine/threonine-protein kinase Chk1	CHEK1
MOL004856	Gancaonin A	Estrogen receptor	ESR1
MOL004856	Gancaonin A	Estrogen receptor beta	ESR2
MOL004856	Gancaonin A	Glycogen synthase kinase-3 beta	GSK3B
MOL004856	Gancaonin A	Nuclear receptor coactivator 2	NCOA2
MOL004856	Gancaonin A	Nitric oxide synthase, inducible	NOS2
MOL004856	Gancaonin A	Prostaglandin G/H synthase 2	PTGS2
MOL004856	Gancaonin A	Sodium channel protein type 5 subunit alpha	SCN5A
MOL004857	Gancaonin B	Alpha-1B adrenergic receptor	ADRA1B
MOL004857	Gancaonin B	Beta-2 adrenergic receptor	ADRB2
MOL004857	Gancaonin B	Androgen receptor	AR
MOL004857	Gancaonin B	Cyclin-A2	CCNA2
MOL004857	Gancaonin B	Serine/threonine-protein kinase Chk1	CHEK1
MOL004857	Gancaonin B	Estrogen receptor	ESR1
MOL004857	Gancaonin B	Estrogen receptor beta	ESR2
MOL004857	Gancaonin B	Coagulation factor VII	F7
MOL004857	Gancaonin B	Glycogen synthase kinase-3 beta	GSK3B
MOL004857	Gancaonin B	Vascular endothelial growth factor receptor 2	KDR
MOL004857	Gancaonin B	Nuclear receptor coactivator 2	NCOA2
MOL004857	Gancaonin B	Nitric oxide synthase, inducible	NOS2
MOL004857	Gancaonin B	Prostaglandin G/H synthase 2	PTGS2
MOL004863	3-(3,4-dihydroxyphenyl)-5,7-dihydroxy-8-(3-methylbut-2-enyl)chromone	Androgen receptor	AR
MOL004863	3-(3,4-dihydroxyphenyl)-5,7-dihydroxy-8-(3-methylbut-2-enyl)chromone	Cyclin-A2	CCNA2
MOL004863	3-(3,4-dihydroxyphenyl)-5,7-dihydroxy-8-(3-methylbut-2-enyl)chromone	Serine/threonine-protein kinase Chk1	CHEK1
MOL004863	3-(3,4-dihydroxyphenyl)-5,7-dihydroxy-8-(3-methylbut-2-enyl)chromone	Estrogen receptor	ESR1
MOL004863	3-(3,4-dihydroxyphenyl)-5,7-dihydroxy-8-(3-methylbut-2-enyl)chromone	Glycogen synthase kinase-3 beta	GSK3B
MOL004863	3-(3,4-dihydroxyphenyl)-5,7-dihydroxy-8-(3-methylbut-2-enyl)chromone	Mitogen-activated protein kinase 14	MAPK14
MOL004863	3-(3,4-dihydroxyphenyl)-5,7-dihydroxy-8-(3-methylbut-2-enyl)chromone	Nuclear receptor coactivator 2	NCOA2
MOL004863	3-(3,4-dihydroxyphenyl)-5,7-dihydroxy-8-(3-methylbut-2-enyl)chromone	Nitric oxide synthase, inducible	NOS2
MOL004863	3-(3,4-dihydroxyphenyl)-5,7-dihydroxy-8-(3-methylbut-2-enyl)chromone	Prostaglandin G/H synthase 2	PTGS2
MOL004864	5,7-dihydroxy-3-(4-methoxyphenyl)-8-(3-methylbut-2-enyl)chromone	Androgen receptor	AR
MOL004864	5,7-dihydroxy-3-(4-methoxyphenyl)-8-(3-methylbut-2-enyl)chromone	Cyclin-A2	CCNA2
MOL004864	5,7-dihydroxy-3-(4-methoxyphenyl)-8-(3-methylbut-2-enyl)chromone	Serine/threonine-protein kinase Chk1	CHEK1
MOL004864	5,7-dihydroxy-3-(4-methoxyphenyl)-8-(3-methylbut-2-enyl)chromone	Estrogen receptor	ESR1
MOL004864	5,7-dihydroxy-3-(4-methoxyphenyl)-8-(3-methylbut-2-enyl)chromone	Estrogen receptor beta	ESR2
MOL004864	5,7-dihydroxy-3-(4-methoxyphenyl)-8-(3-methylbut-2-enyl)chromone	Glycogen synthase kinase-3 beta	GSK3B
MOL004864	5,7-dihydroxy-3-(4-methoxyphenyl)-8-(3-methylbut-2-enyl)chromone	Potassium voltage-gated channel subfamily H member 2	KCNH2
MOL004864	5,7-dihydroxy-3-(4-methoxyphenyl)-8-(3-methylbut-2-enyl)chromone	Mitogen-activated protein kinase 14	MAPK14
MOL004864	5,7-dihydroxy-3-(4-methoxyphenyl)-8-(3-methylbut-2-enyl)chromone	Nuclear receptor coactivator 2	NCOA2
MOL004864	5,7-dihydroxy-3-(4-methoxyphenyl)-8-(3-methylbut-2-enyl)chromone	Nitric oxide synthase, inducible	NOS2
MOL004864	5,7-dihydroxy-3-(4-methoxyphenyl)-8-(3-methylbut-2-enyl)chromone	Prostaglandin G/H synthase 2	PTGS2
MOL004866	2-(3,4-dihydroxyphenyl)-5,7-dihydroxy-6-(3-methylbut-2-enyl)chromone	Beta-2 adrenergic receptor	ADRB2
MOL004866	2-(3,4-dihydroxyphenyl)-5,7-dihydroxy-6-(3-methylbut-2-enyl)chromone	Androgen receptor	AR
MOL004866	2-(3,4-dihydroxyphenyl)-5,7-dihydroxy-6-(3-methylbut-2-enyl)chromone	Cyclin-A2	CCNA2
MOL004866	2-(3,4-dihydroxyphenyl)-5,7-dihydroxy-6-(3-methylbut-2-enyl)chromone	Serine/threonine-protein kinase Chk1	CHEK1
MOL004866	2-(3,4-dihydroxyphenyl)-5,7-dihydroxy-6-(3-methylbut-2-enyl)chromone	Coagulation factor VII	F7
MOL004866	2-(3,4-dihydroxyphenyl)-5,7-dihydroxy-6-(3-methylbut-2-enyl)chromone	Prostaglandin G/H synthase 2	PTGS2
MOL004866	2-(3,4-dihydroxyphenyl)-5,7-dihydroxy-6-(3-methylbut-2-enyl)chromone	Sodium channel protein type 5 subunit alpha	SCN5A
MOL004879	Glycyrin	Androgen receptor	AR
MOL004879	Glycyrin	Serine/threonine-protein kinase Chk1	CHEK1
MOL004879	Glycyrin	Estrogen receptor	ESR1
MOL004879	Glycyrin	Estrogen receptor beta	ESR2
MOL004879	Glycyrin	Potassium voltage-gated channel subfamily H member 2	KCNH2
MOL004879	Glycyrin	Vascular endothelial growth factor receptor 2	KDR
MOL004879	Glycyrin	Nuclear receptor coactivator 2	NCOA2
MOL004879	Glycyrin	Nitric oxide synthase, inducible	NOS2
MOL004879	Glycyrin	Prostaglandin G/H synthase 2	PTGS2
MOL004882	Licocoumarone	Androgen receptor	AR
MOL004882	Licocoumarone	Cyclin-A2	CCNA2
MOL004882	Licocoumarone	Estrogen receptor	ESR1
MOL004882	Licocoumarone	Estrogen receptor beta	ESR2
MOL004882	Licocoumarone	Glycogen synthase kinase-3 beta	GSK3B
MOL004883	Licoisoflavone	Androgen receptor	AR
MOL004883	Licoisoflavone	Cyclin-A2	CCNA2
MOL004883	Licoisoflavone	Serine/threonine-protein kinase Chk1	CHEK1
MOL004883	Licoisoflavone	Estrogen receptor	ESR1
MOL004883	Licoisoflavone	Vascular endothelial growth factor receptor 2	KDR
MOL004883	Licoisoflavone	Mitogen-activated protein kinase 14	MAPK14
MOL004883	Licoisoflavone	Nuclear receptor coactivator 2	NCOA2
MOL004883	Licoisoflavone	Nitric oxide synthase, inducible	NOS2
MOL004883	Licoisoflavone	Prostaglandin G/H synthase 2	PTGS2
MOL004884	Licoisoflavone B	Acetylcholinesterase	ACHE
MOL004884	Licoisoflavone B	Androgen receptor	AR
MOL004884	Licoisoflavone B	Cyclin-A2	CCNA2
MOL004884	Licoisoflavone B	Serine/threonine-protein kinase Chk1	CHEK1
MOL004884	Licoisoflavone B	Estrogen receptor	ESR1
MOL004884	Licoisoflavone B	Estrogen receptor beta	ESR2
MOL004884	Licoisoflavone B	Glycogen synthase kinase-3 beta	GSK3B
MOL004884	Licoisoflavone B	Nitric oxide synthase, inducible	NOS2
MOL004884	Licoisoflavone B	Prostaglandin G/H synthase 2	PTGS2
MOL004885	licoisoflavanone	Acetylcholinesterase	ACHE

Targets related to Gancao

MolId	MolName	Target	Symbol
MOL004885	licoisoflavanone	Androgen receptor	AR
MOL004885	licoisoflavanone	Cyclin-A2	CCNA2
MOL004885	licoisoflavanone	Estrogen receptor	ESR1
MOL004885	licoisoflavanone	Estrogen receptor beta	ESR2
MOL004885	licoisoflavanone	Coagulation factor VII	F7
MOL004885	licoisoflavanone	Glycogen synthase kinase-3 beta	GSK3B
MOL004885	licoisoflavanone	Nuclear receptor coactivator 1	NCOA1
MOL004885	licoisoflavanone	Nitric oxide synthase, inducible	NOS2
MOL004885	licoisoflavanone	Prostaglandin G/H synthase 1	PTGS1
MOL004885	licoisoflavanone	Prostaglandin G/H synthase 2	PTGS2
MOL004885	licoisoflavanone	Sodium channel protein type 5 subunit alpha	SCN5A
MOL004891	shinpterocarpin	Alpha-1B adrenergic receptor	ADRA1B
MOL004891	shinpterocarpin	Alpha-1D adrenergic receptor	ADRA1D
MOL004891	shinpterocarpin	Beta-2 adrenergic receptor	ADRB2
MOL004891	shinpterocarpin	Androgen receptor	AR
MOL004891	shinpterocarpin	Cyclin-A2	CCNA2
MOL004891	shinpterocarpin	Muscarinic acetylcholine receptor M1	CHRM1
MOL004891	shinpterocarpin	Muscarinic acetylcholine receptor M3	CHRM3
MOL004891	shinpterocarpin	Estrogen receptor	ESR1
MOL004891	shinpterocarpin	Estrogen receptor beta	ESR2
MOL004891	shinpterocarpin	Glycogen synthase kinase-3 beta	GSK3B
MOL004891	shinpterocarpin	5-hydroxytryptamine receptor 3A	HTR3A
MOL004891	shinpterocarpin	Potassium voltage-gated channel subfamily H member 2	KCNH2
MOL004891	shinpterocarpin	Mitogen-activated protein kinase 14	MAPK14
MOL004891	shinpterocarpin	Nuclear receptor coactivator 1	NCOA1
MOL004891	shinpterocarpin	Nitric oxide synthase, inducible	NOS2
MOL004891	shinpterocarpin	Delta-type opioid receptor	OPRD1
MOL004891	shinpterocarpin	Mu-type opioid receptor	OPRM1
MOL004891	shinpterocarpin	Prostaglandin G/H synthase 1	PTGS1
MOL004891	shinpterocarpin	Prostaglandin G/H synthase 2	PTGS2
MOL004891	shinpterocarpin	Retinoic acid receptor RXR-alpha	RXRA
MOL004891	shinpterocarpin	Retinoic acid receptor RXR-beta	RXRB
MOL004891	shinpterocarpin	Sodium channel protein type 5 subunit alpha	SCN5A
MOL004898	(E)-3-[3,4-dihydroxy-5-(3-methylbut-2-enyl)phenyl]-1-(2,4-	Androgen receptor	AR
MOL004898	(E)-3-[3,4-dihydroxy-5-(3-methylbut-2-enyl)phenyl]-1-(2,4-	Cyclin-A2	CCNA2
MOL004898	(E)-3-[3,4-dihydroxy-5-(3-methylbut-2-enyl)phenyl]-1-(2,4-	Estrogen receptor	ESR1
MOL004898	(E)-3-[3,4-dihydroxy-5-(3-methylbut-2-enyl)phenyl]-1-(2,4-	Glycogen synthase kinase-3 beta	GSK3B
MOL004898	(E)-3-[3,4-dihydroxy-5-(3-methylbut-2-enyl)phenyl]-1-(2,4-	Mitogen-activated protein kinase 14	MAPK14
MOL004898	(E)-3-[3,4-dihydroxy-5-(3-methylbut-2-enyl)phenyl]-1-(2,4-	Nuclear receptor coactivator 2	NCOA2
MOL004898	(E)-3-[3,4-dihydroxy-5-(3-methylbut-2-enyl)phenyl]-1-(2,4-	Prostaglandin G/H synthase 2	PTGS2
MOL004903	liquiritin	Coagulation factor VII	F7
MOL004903	liquiritin	Vascular endothelial growth factor receptor 2	KDR
MOL004903	liquiritin	Prostaglandin G/H synthase 2	PTGS2
MOL004904	licopyranocoumarin	Acetylcholinesterase	ACHE
MOL004904	licopyranocoumarin	Androgen receptor	AR
MOL004904	licopyranocoumarin	Cyclin-A2	CCNA2
MOL004904	licopyranocoumarin	Estrogen receptor	ESR1
MOL004904	licopyranocoumarin	Coagulation factor VII	F7
MOL004904	licopyranocoumarin	Vascular endothelial growth factor receptor 2	KDR
MOL004904	licopyranocoumarin	Nitric oxide synthase, inducible	NOS2
MOL004904	licopyranocoumarin	Prostaglandin G/H synthase 2	PTGS2
MOL004907	Glyzaglabrin	Androgen receptor	AR
MOL004907	Glyzaglabrin	Cyclin-A2	CCNA2
MOL004907	Glyzaglabrin	Serine/threonine-protein kinase Chk1	CHEK1
MOL004907	Glyzaglabrin	Estrogen receptor	ESR1
MOL004907	Glyzaglabrin	Estrogen receptor beta	ESR2
MOL004907	Glyzaglabrin	Glycogen synthase kinase-3 beta	GSK3B
MOL004907	Glyzaglabrin	Mitogen-activated protein kinase 14	MAPK14
MOL004907	Glyzaglabrin	Nitric oxide synthase, inducible	NOS2
MOL004907	Glyzaglabrin	Prostaglandin G/H synthase 1	PTGS1
MOL004907	Glyzaglabrin	Prostaglandin G/H synthase 2	PTGS2
MOL004908	Glabridin	Acetylcholinesterase	ACHE
MOL004908	Glabridin	Alpha-1B adrenergic receptor	ADRA1B
MOL004908	Glabridin	Beta-2 adrenergic receptor	ADRB2
MOL004908	Glabridin	Androgen receptor	AR
MOL004908	Glabridin	Cyclin-A2	CCNA2
MOL004908	Glabridin	Serine/threonine-protein kinase Chk1	CHEK1
MOL004908	Glabridin	Muscarinic acetylcholine receptor M1	CHRM1
MOL004908	Glabridin	Estrogen receptor	ESR1
MOL004908	Glabridin	Estrogen receptor beta	ESR2
MOL004908	Glabridin	Glycogen synthase kinase-3 beta	GSK3B
MOL004908	Glabridin	Mitogen-activated protein kinase 14	MAPK14
MOL004908	Glabridin	Nuclear receptor coactivator 1	NCOA1
MOL004908	Glabridin	Nuclear receptor coactivator 2	NCOA2
MOL004908	Glabridin	Nitric oxide synthase, inducible	NOS2
MOL004908	Glabridin	Prostaglandin G/H synthase 2	PTGS2
MOL004908	Glabridin	Retinoic acid receptor RXR-alpha	RXRA
MOL004908	Glabridin	Retinoic acid receptor RXR-beta	RXRB
MOL004908	Glabridin	Sodium channel protein type 5 subunit alpha	SCN5A
MOL004910	Glabranin	Estrogen receptor	ESR1
MOL004910	Glabranin	Nitric oxide synthase, inducible	NOS2
MOL004910	Glabranin	CGMP-inhibited 3',5'-cyclic phosphodiesterase A	PDE3A
MOL004910	Glabranin	Prostaglandin G/H synthase 1	PTGS1
MOL004910	Glabranin	Prostaglandin G/H synthase 2	PTGS2
MOL004910	Glabranin	Sodium channel protein type 5 subunit alpha	SCN5A
MOL004911	Glabrene	Beta-2 adrenergic receptor	ADRB2
MOL004911	Glabrene	Androgen receptor	AR
MOL004911	Glabrene	Estrogen receptor	ESR1
MOL004911	Glabrene	Estrogen receptor beta	ESR2
MOL004911	Glabrene	Glycogen synthase kinase-3 beta	GSK3B
MOL004911	Glabrene	Mitogen-activated protein kinase 14	MAPK14
MOL004911	Glabrene	Nuclear receptor coactivator 2	NCOA2

Targets related to Gancao

MolId	MolName	Target	Symbol
MOL004911	Glabrene	Nitric oxide synthase, inducible	NOS2
MOL004911	Glabrene	Prostaglandin G/H synthase 1	PTGS1
MOL004911	Glabrene	Prostaglandin G/H synthase 2	PTGS2
MOL004911	Glabrene	Retinoic acid receptor RXR-alpha	RXRA
MOL004911	Glabrene	Sodium channel protein type 5 subunit alpha	SCN5A
MOL004912	Glabrone	Acetylcholinesterase	ACHE
MOL004912	Glabrone	Androgen receptor	AR
MOL004912	Glabrone	Cyclin-A2	CCNA2
MOL004912	Glabrone	Serine/threonine-protein kinase Chk1	CHEK1
MOL004912	Glabrone	Estrogen receptor	ESR1
MOL004912	Glabrone	Estrogen receptor beta	ESR2
MOL004912	Glabrone	Glycogen synthase kinase-3 beta	GSK3B
MOL004912	Glabrone	Mitogen-activated protein kinase 14	MAPK14
MOL004912	Glabrone	Nitric oxide synthase, inducible	NOS2
MOL004912	Glabrone	Prostaglandin G/H synthase 1	PTGS1
MOL004912	Glabrone	Prostaglandin G/H synthase 2	PTGS2
MOL004912	Glabrone	Retinoic acid receptor RXR-alpha	RXRA
MOL004912	Glabrone	Sodium channel protein type 5 subunit alpha	SCN5A
MOL004913	1,3-dihydroxy-9-methoxy-6-benzofurano[3,2-c]chromenone	Cyclin-A2	CCNA2
MOL004913	1,3-dihydroxy-9-methoxy-6-benzofurano[3,2-c]chromenone	Serine/threonine-protein kinase Chk1	CHEK1
MOL004913	1,3-dihydroxy-9-methoxy-6-benzofurano[3,2-c]chromenone	Estrogen receptor	ESR1
MOL004913	1,3-dihydroxy-9-methoxy-6-benzofurano[3,2-c]chromenone	Estrogen receptor beta	ESR2
MOL004913	1,3-dihydroxy-9-methoxy-6-benzofurano[3,2-c]chromenone	Glycogen synthase kinase-3 beta	GSK3B
MOL004913	1,3-dihydroxy-9-methoxy-6-benzofurano[3,2-c]chromenone	Mitogen-activated protein kinase 14	MAPK14
MOL004914	1,3-dihydroxy-8,9-dimethoxy-6-benzofurano[3,2-c]chromenone	Androgen receptor	AR
MOL004914	1,3-dihydroxy-8,9-dimethoxy-6-benzofurano[3,2-c]chromenone	Serine/threonine-protein kinase Chk1	CHEK1
MOL004914	1,3-dihydroxy-8,9-dimethoxy-6-benzofurano[3,2-c]chromenone	Estrogen receptor	ESR1
MOL004914	1,3-dihydroxy-8,9-dimethoxy-6-benzofurano[3,2-c]chromenone	Glycogen synthase kinase-3 beta	GSK3B
MOL004914	1,3-dihydroxy-8,9-dimethoxy-6-benzofurano[3,2-c]chromenone	Mitogen-activated protein kinase 14	MAPK14
MOL004915	Eurycarpin A	Androgen receptor	AR
MOL004915	Eurycarpin A	Cyclin-A2	CCNA2
MOL004915	Eurycarpin A	Serine/threonine-protein kinase Chk1	CHEK1
MOL004915	Eurycarpin A	Estrogen receptor	ESR1
MOL004915	Eurycarpin A	Estrogen receptor beta	ESR2
MOL004915	Eurycarpin A	Glycogen synthase kinase-3 beta	GSK3B
MOL004915	Eurycarpin A	Mitogen-activated protein kinase 14	MAPK14
MOL004915	Eurycarpin A	Nitric oxide synthase, inducible	NOS2
MOL004915	Eurycarpin A	Prostaglandin G/H synthase 2	PTGS2
MOL004915	Eurycarpin A	Sodium channel protein type 5 subunit alpha	SCN5A
MOL004924	(-)-Medicocarpin	Acetylcholinesterase	ACHE
MOL004924	(-)-Medicocarpin	Prostaglandin G/H synthase 2	PTGS2
MOL004935	Sigmoidin-B	Estrogen receptor	ESR1
MOL004935	Sigmoidin-B	Vascular endothelial growth factor receptor 2	KDR
MOL004935	Sigmoidin-B	Prostaglandin G/H synthase 2	PTGS2
MOL004941	(2R)-7-hydroxy-2-(4-hydroxyphenyl)chroman-4-one	Beta-2 adrenergic receptor	ADRB2
MOL004941	(2R)-7-hydroxy-2-(4-hydroxyphenyl)chroman-4-one	Estrogen receptor	ESR1
MOL004941	(2R)-7-hydroxy-2-(4-hydroxyphenyl)chroman-4-one	Gamma-aminobutyric acid receptor subunit alpha-1	GABRA1
MOL004941	(2R)-7-hydroxy-2-(4-hydroxyphenyl)chroman-4-one	CGMP-inhibited 3',5'-cyclic phosphodiesterase A	PDE3A
MOL004941	(2R)-7-hydroxy-2-(4-hydroxyphenyl)chroman-4-one	cAMP-dependent protein kinase inhibitor alpha	PKIA
MOL004941	(2R)-7-hydroxy-2-(4-hydroxyphenyl)chroman-4-one	Prostaglandin G/H synthase 1	PTGS1
MOL004941	(2R)-7-hydroxy-2-(4-hydroxyphenyl)chroman-4-one	Prostaglandin G/H synthase 2	PTGS2
MOL004941	(2R)-7-hydroxy-2-(4-hydroxyphenyl)chroman-4-one	Retinoic acid receptor RXR-alpha	RXRA
MOL004941	(2R)-7-hydroxy-2-(4-hydroxyphenyl)chroman-4-one	Sodium-dependent serotonin transporter	SLC6A4
MOL004945	(2S)-7-hydroxy-2-(4-hydroxyphenyl)-8-(3-methylbut-2-enyl)chroman-4-one	Alpha-1B adrenergic receptor	ADRA1B
MOL004945	(2S)-7-hydroxy-2-(4-hydroxyphenyl)-8-(3-methylbut-2-enyl)chroman-4-one	Beta-2 adrenergic receptor	ADRB2
MOL004945	(2S)-7-hydroxy-2-(4-hydroxyphenyl)-8-(3-methylbut-2-enyl)chroman-4-one	Estrogen receptor	ESR1
MOL004945	(2S)-7-hydroxy-2-(4-hydroxyphenyl)-8-(3-methylbut-2-enyl)chroman-4-one	Estrogen receptor beta	ESR2
MOL004945	(2S)-7-hydroxy-2-(4-hydroxyphenyl)-8-(3-methylbut-2-enyl)chroman-4-one	Nitric oxide synthase, inducible	NOS2
MOL004945	(2S)-7-hydroxy-2-(4-hydroxyphenyl)-8-(3-methylbut-2-enyl)chroman-4-one	CGMP-inhibited 3',5'-cyclic phosphodiesterase A	PDE3A
MOL004945	(2S)-7-hydroxy-2-(4-hydroxyphenyl)-8-(3-methylbut-2-enyl)chroman-4-one	Prostaglandin G/H synthase 1	PTGS1
MOL004945	(2S)-7-hydroxy-2-(4-hydroxyphenyl)-8-(3-methylbut-2-enyl)chroman-4-one	Prostaglandin G/H synthase 2	PTGS2
MOL004945	(2S)-7-hydroxy-2-(4-hydroxyphenyl)-8-(3-methylbut-2-enyl)chroman-4-one	Sodium channel protein type 5 subunit alpha	SCN5A
MOL004948	Isoglycyrol	Androgen receptor	AR
MOL004948	Isoglycyrol	Estrogen receptor	ESR1
MOL004948	Isoglycyrol	Glycogen synthase kinase-3 beta	GSK3B
MOL004948	Isoglycyrol	Nitric oxide synthase, inducible	NOS2
MOL004948	Isoglycyrol	Prostaglandin G/H synthase 2	PTGS2
MOL004949	Isolicoflavonol	Androgen receptor	AR
MOL004949	Isolicoflavonol	Cyclin-A2	CCNA2
MOL004949	Isolicoflavonol	Estrogen receptor	ESR1
MOL004949	Isolicoflavonol	Glycogen synthase kinase-3 beta	GSK3B
MOL004949	Isolicoflavonol	Nuclear receptor coactivator 2	NCOA2
MOL004949	Isolicoflavonol	Nitric oxide synthase, inducible	NOS2
MOL004949	Isolicoflavonol	Prostaglandin G/H synthase 2	PTGS2
MOL004957	HMO	Beta-2 adrenergic receptor	ADRB2
MOL004957	HMO	Androgen receptor	AR
MOL004957	HMO	Cyclin-A2	CCNA2
MOL004957	HMO	Serine/threonine-protein kinase Chk1	CHEK1
MOL004957	HMO	Muscarinic acetylcholine receptor M1	CHRM1
MOL004957	HMO	Estrogen receptor	ESR1
MOL004957	HMO	Estrogen receptor beta	ESR2
MOL004957	HMO	Glycogen synthase kinase-3 beta	GSK3B
MOL004957	HMO	Mitogen-activated protein kinase 14	MAPK14
MOL004957	HMO	Nitric oxide synthase, inducible	NOS2
MOL004957	HMO	CGMP-inhibited 3',5'-cyclic phosphodiesterase A	PDE3A
MOL004957	HMO	cAMP-dependent protein kinase inhibitor alpha	PKIA
MOL004957	HMO	Prostaglandin G/H synthase 1	PTGS1
MOL004957	HMO	Prostaglandin G/H synthase 2	PTGS2
MOL004957	HMO	Retinoic acid receptor RXR-alpha	RXRA
MOL004957	HMO	Sodium channel protein type 5 subunit alpha	SCN5A
MOL004957	HMO	Sodium-dependent dopamine transporter	SLC6A3
MOL004957	HMO	Sodium-dependent serotonin transporter	SLC6A4

Targets related to Gancao

MolId	MolName	Target	Symbol
MOL004959	1-Methoxyphaseollidin	Alpha-1B adrenergic receptor	ADRA1B
MOL004959	1-Methoxyphaseollidin	Alpha-1D adrenergic receptor	ADRA1D
MOL004959	1-Methoxyphaseollidin	Beta-2 adrenergic receptor	ADRB2
MOL004959	1-Methoxyphaseollidin	Androgen receptor	AR
MOL004959	1-Methoxyphaseollidin	Cyclin-A2	CCNA2
MOL004959	1-Methoxyphaseollidin	Estrogen receptor	ESR1
MOL004959	1-Methoxyphaseollidin	Estrogen receptor beta	ESR2
MOL004959	1-Methoxyphaseollidin	Glycogen synthase kinase-3 beta	GSK3B
MOL004959	1-Methoxyphaseollidin	Potassium voltage-gated channel subfamily H member 2	KCNH2
MOL004959	1-Methoxyphaseollidin	Vascular endothelial growth factor receptor 2	KDR
MOL004959	1-Methoxyphaseollidin	Mitogen-activated protein kinase 14	MAPK14
MOL004959	1-Methoxyphaseollidin	Nuclear receptor coactivator 1	NCOA1
MOL004959	1-Methoxyphaseollidin	Nuclear receptor coactivator 2	NCOA2
MOL004959	1-Methoxyphaseollidin	Nitric oxide synthase, inducible	NOS2
MOL004959	1-Methoxyphaseollidin	Prostaglandin G/H synthase 1	PTGS1
MOL004959	1-Methoxyphaseollidin	Prostaglandin G/H synthase 2	PTGS2
MOL004959	1-Methoxyphaseollidin	Retinoic acid receptor RXR-alpha	RXRA
MOL004959	1-Methoxyphaseollidin	Sodium channel protein type 5 subunit alpha	SCN5A
MOL004961	Quercetin der.	Androgen receptor	AR
MOL004961	Quercetin der.	Estrogen receptor	ESR1
MOL004961	Quercetin der.	Estrogen receptor beta	ESR2
MOL004961	Quercetin der.	Glycogen synthase kinase-3 beta	GSK3B
MOL004961	Quercetin der.	Mitogen-activated protein kinase 14	MAPK14
MOL004961	Quercetin der.	Nuclear receptor coactivator 2	NCOA2
MOL004961	Quercetin der.	Nitric oxide synthase, inducible	NOS2
MOL004961	Quercetin der.	Prostaglandin G/H synthase 1	PTGS1
MOL004961	Quercetin der.	Prostaglandin G/H synthase 2	PTGS2
MOL004961	Quercetin der.	Sodium channel protein type 5 subunit alpha	SCN5A
MOL004966	3'-Hydroxy-4'-O-Methylglabridin	Alpha-1B adrenergic receptor	ADRA1B
MOL004966	3'-Hydroxy-4'-O-Methylglabridin	Beta-2 adrenergic receptor	ADRB2
MOL004966	3'-Hydroxy-4'-O-Methylglabridin	Androgen receptor	AR
MOL004966	3'-Hydroxy-4'-O-Methylglabridin	Cyclin-A2	CCNA2
MOL004966	3'-Hydroxy-4'-O-Methylglabridin	Serine/threonine-protein kinase Chk1	CHEK1
MOL004966	3'-Hydroxy-4'-O-Methylglabridin	Estrogen receptor	ESR1
MOL004966	3'-Hydroxy-4'-O-Methylglabridin	Estrogen receptor beta	ESR2
MOL004966	3'-Hydroxy-4'-O-Methylglabridin	Coagulation factor VII	F7
MOL004966	3'-Hydroxy-4'-O-Methylglabridin	Glycogen synthase kinase-3 beta	GSK3B
MOL004966	3'-Hydroxy-4'-O-Methylglabridin	Potassium voltage-gated channel subfamily H member 2	KCNH2
MOL004966	3'-Hydroxy-4'-O-Methylglabridin	Vascular endothelial growth factor receptor 2	KDR
MOL004966	3'-Hydroxy-4'-O-Methylglabridin	Mitogen-activated protein kinase 14	MAPK14
MOL004966	3'-Hydroxy-4'-O-Methylglabridin	Nuclear receptor coactivator 1	NCOA1
MOL004966	3'-Hydroxy-4'-O-Methylglabridin	Nuclear receptor coactivator 2	NCOA2
MOL004966	3'-Hydroxy-4'-O-Methylglabridin	Nitric oxide synthase, inducible	NOS2
MOL004966	3'-Hydroxy-4'-O-Methylglabridin	Prostaglandin G/H synthase 1	PTGS1
MOL004966	3'-Hydroxy-4'-O-Methylglabridin	Prostaglandin G/H synthase 2	PTGS2
MOL004966	3'-Hydroxy-4'-O-Methylglabridin	Sodium channel protein type 5 subunit alpha	SCN5A
MOL004974	3'-Methoxyglabridin	Acetylcholinesterase	ACHE
MOL004974	3'-Methoxyglabridin	Alpha-1B adrenergic receptor	ADRA1B
MOL004974	3'-Methoxyglabridin	Beta-2 adrenergic receptor	ADRB2
MOL004974	3'-Methoxyglabridin	Androgen receptor	AR
MOL004974	3'-Methoxyglabridin	Cyclin-A2	CCNA2
MOL004974	3'-Methoxyglabridin	Serine/threonine-protein kinase Chk1	CHEK1
MOL004974	3'-Methoxyglabridin	Estrogen receptor	ESR1
MOL004974	3'-Methoxyglabridin	Estrogen receptor beta	ESR2
MOL004974	3'-Methoxyglabridin	Coagulation factor VII	F7
MOL004974	3'-Methoxyglabridin	Glycogen synthase kinase-3 beta	GSK3B
MOL004974	3'-Methoxyglabridin	Potassium voltage-gated channel subfamily H member 2	KCNH2
MOL004974	3'-Methoxyglabridin	Mitogen-activated protein kinase 14	MAPK14
MOL004974	3'-Methoxyglabridin	Nuclear receptor coactivator 1	NCOA1
MOL004974	3'-Methoxyglabridin	Nuclear receptor coactivator 2	NCOA2
MOL004974	3'-Methoxyglabridin	Nitric oxide synthase, inducible	NOS2
MOL004974	3'-Methoxyglabridin	Prostaglandin G/H synthase 1	PTGS1
MOL004974	3'-Methoxyglabridin	Prostaglandin G/H synthase 2	PTGS2
MOL004974	3'-Methoxyglabridin	Retinoic acid receptor RXR-alpha	RXRA
MOL004974	3'-Methoxyglabridin	Sodium channel protein type 5 subunit alpha	SCN5A
MOL004978	2-[(3R)-8,8-dimethyl-3,4-dihydro-2H-pyrano[6,5-f]chromen-3-yl]-5-	Acetylcholinesterase	ACHE
MOL004978	2-[(3R)-8,8-dimethyl-3,4-dihydro-2H-pyrano[6,5-f]chromen-3-yl]-5-	Alpha-1B adrenergic receptor	ADRA1B
MOL004978	2-[(3R)-8,8-dimethyl-3,4-dihydro-2H-pyrano[6,5-f]chromen-3-yl]-5-	Beta-2 adrenergic receptor	ADRB2
MOL004978	2-[(3R)-8,8-dimethyl-3,4-dihydro-2H-pyrano[6,5-f]chromen-3-yl]-5-	Androgen receptor	AR
MOL004978	2-[(3R)-8,8-dimethyl-3,4-dihydro-2H-pyrano[6,5-f]chromen-3-yl]-5-	Cyclin-A2	CCNA2
MOL004978	2-[(3R)-8,8-dimethyl-3,4-dihydro-2H-pyrano[6,5-f]chromen-3-yl]-5-	Serine/threonine-protein kinase Chk1	CHEK1
MOL004978	2-[(3R)-8,8-dimethyl-3,4-dihydro-2H-pyrano[6,5-f]chromen-3-yl]-5-	Muscarinic acetylcholine receptor M1	CHRM1
MOL004978	2-[(3R)-8,8-dimethyl-3,4-dihydro-2H-pyrano[6,5-f]chromen-3-yl]-5-	Muscarinic acetylcholine receptor M3	CHRM3
MOL004978	2-[(3R)-8,8-dimethyl-3,4-dihydro-2H-pyrano[6,5-f]chromen-3-yl]-5-	Estrogen receptor	ESR1
MOL004978	2-[(3R)-8,8-dimethyl-3,4-dihydro-2H-pyrano[6,5-f]chromen-3-yl]-5-	Estrogen receptor beta	ESR2
MOL004978	2-[(3R)-8,8-dimethyl-3,4-dihydro-2H-pyrano[6,5-f]chromen-3-yl]-5-	Glycogen synthase kinase-3 beta	GSK3B
MOL004978	2-[(3R)-8,8-dimethyl-3,4-dihydro-2H-pyrano[6,5-f]chromen-3-yl]-5-	Potassium voltage-gated channel subfamily H member 2	KCNH2
MOL004978	2-[(3R)-8,8-dimethyl-3,4-dihydro-2H-pyrano[6,5-f]chromen-3-yl]-5-	Mitogen-activated protein kinase 14	MAPK14
MOL004978	2-[(3R)-8,8-dimethyl-3,4-dihydro-2H-pyrano[6,5-f]chromen-3-yl]-5-	Nuclear receptor coactivator 1	NCOA1
MOL004978	2-[(3R)-8,8-dimethyl-3,4-dihydro-2H-pyrano[6,5-f]chromen-3-yl]-5-	Nuclear receptor coactivator 2	NCOA2
MOL004978	2-[(3R)-8,8-dimethyl-3,4-dihydro-2H-pyrano[6,5-f]chromen-3-yl]-5-	Nitric oxide synthase, inducible	NOS2
MOL004978	2-[(3R)-8,8-dimethyl-3,4-dihydro-2H-pyrano[6,5-f]chromen-3-yl]-5-	Prostaglandin G/H synthase 1	PTGS1
MOL004978	2-[(3R)-8,8-dimethyl-3,4-dihydro-2H-pyrano[6,5-f]chromen-3-yl]-5-	Prostaglandin G/H synthase 2	PTGS2
MOL004978	2-[(3R)-8,8-dimethyl-3,4-dihydro-2H-pyrano[6,5-f]chromen-3-yl]-5-	Retinoic acid receptor RXR-alpha	RXRA
MOL004978	2-[(3R)-8,8-dimethyl-3,4-dihydro-2H-pyrano[6,5-f]chromen-3-yl]-5-	Retinoic acid receptor RXR-beta	RXRB
MOL004978	2-[(3R)-8,8-dimethyl-3,4-dihydro-2H-pyrano[6,5-f]chromen-3-yl]-5-	Sodium channel protein type 5 subunit alpha	SCN5A
MOL004978	2-[(3R)-8,8-dimethyl-3,4-dihydro-2H-pyrano[6,5-f]chromen-3-yl]-5-	Sodium-dependent dopamine transporter	SLC6A3
MOL004980	Inflacoumarin A	Beta-2 adrenergic receptor	ADRB2
MOL004980	Inflacoumarin A	Androgen receptor	AR
MOL004980	Inflacoumarin A	Estrogen receptor	ESR1
MOL004980	Inflacoumarin A	Nuclear receptor coactivator 2	NCOA2
MOL004980	Inflacoumarin A	Prostaglandin G/H synthase 1	PTGS1

Targets related to Gancao

MolId	MolName	Target	Symbol
MOL004980	Inflacoumarin A	Prostaglandin G/H synthase 2	PTGS2
MOL004980	Inflacoumarin A	Sodium channel protein type 5 subunit alpha	SCN5A
MOL004985	icos-5-enoic acid	Nuclear receptor coactivator 2	NCOA2
MOL004988	Kanzonol F	Androgen receptor	AR
MOL004988	Kanzonol F	Estrogen receptor	ESR1
MOL004988	Kanzonol F	Estrogen receptor beta	ESR2
MOL004988	Kanzonol F	Nuclear receptor coactivator 2	NCOA2
MOL004988	Kanzonol F	Prostaglandin G/H synthase 2	PTGS2
MOL004989	6-prenylated eriodictyol	Estrogen receptor	ESR1
MOL004989	6-prenylated eriodictyol	Coagulation factor VII	F7
MOL004989	6-prenylated eriodictyol	Nitric oxide synthase, inducible	NOS2
MOL004989	6-prenylated eriodictyol	Prostaglandin G/H synthase 2	PTGS2
MOL004989	6-prenylated eriodictyol	Sodium channel protein type 5 subunit alpha	SCN5A
MOL004990	7,2',4'-trihydroxy－5-methoxy-3－arylcoumarin	Androgen receptor	AR
MOL004990	7,2',4'-trihydroxy－5-methoxy-3－arylcoumarin	Serine/threonine-protein kinase Chk1	CHEK1
MOL004990	7,2',4'-trihydroxy－5-methoxy-3－arylcoumarin	Estrogen receptor	ESR1
MOL004990	7,2',4'-trihydroxy－5-methoxy-3－arylcoumarin	Estrogen receptor beta	ESR2
MOL004990	7,2',4'-trihydroxy－5-methoxy-3－arylcoumarin	Glycogen synthase kinase-3 beta	GSK3B
MOL004990	7,2',4'-trihydroxy－5-methoxy-3－arylcoumarin	Mitogen-activated protein kinase 14	MAPK14
MOL004990	7,2',4'-trihydroxy－5-methoxy-3－arylcoumarin	Nitric oxide synthase, inducible	NOS2
MOL004990	7,2',4'-trihydroxy－5-methoxy-3－arylcoumarin	Prostaglandin G/H synthase 1	PTGS1
MOL004990	7,2',4'-trihydroxy－5-methoxy-3－arylcoumarin	Prostaglandin G/H synthase 2	PTGS2
MOL004991	7-Acetoxy-2-methylisoflavone	Acetylcholinesterase	ACHE
MOL004991	7-Acetoxy-2-methylisoflavone	Alpha-1B adrenergic receptor	ADRA1B
MOL004991	7-Acetoxy-2-methylisoflavone	Alpha-1D adrenergic receptor	ADRA1D
MOL004991	7-Acetoxy-2-methylisoflavone	Beta-2 adrenergic receptor	ADRB2
MOL004991	7-Acetoxy-2-methylisoflavone	Androgen receptor	AR
MOL004991	7-Acetoxy-2-methylisoflavone	Serine/threonine-protein kinase Chk1	CHEK1
MOL004991	7-Acetoxy-2-methylisoflavone	Estrogen receptor	ESR1
MOL004991	7-Acetoxy-2-methylisoflavone	Gamma-aminobutyric acid receptor subunit alpha-1	GABRA1
MOL004991	7-Acetoxy-2-methylisoflavone	Glycogen synthase kinase-3 beta	GSK3B
MOL004991	7-Acetoxy-2-methylisoflavone	Mitogen-activated protein kinase 14	MAPK14
MOL004991	7-Acetoxy-2-methylisoflavone	Nuclear receptor coactivator 2	NCOA2
MOL004991	7-Acetoxy-2-methylisoflavone	Nitric oxide synthase, inducible	NOS2
MOL004991	7-Acetoxy-2-methylisoflavone	CGMP-inhibited 3',5'-cyclic phosphodiesterase A	PDE3A
MOL004991	7-Acetoxy-2-methylisoflavone	Prostaglandin G/H synthase 1	PTGS1
MOL004991	7-Acetoxy-2-methylisoflavone	Prostaglandin G/H synthase 2	PTGS2
MOL004991	7-Acetoxy-2-methylisoflavone	Retinoic acid receptor RXR-alpha	RXRA
MOL004991	7-Acetoxy-2-methylisoflavone	Sodium channel protein type 5 subunit alpha	SCN5A
MOL004993	8-prenylated eriodictyol	Estrogen receptor	ESR1
MOL004993	8-prenylated eriodictyol	Coagulation factor VII	F7
MOL004993	8-prenylated eriodictyol	Nuclear receptor coactivator 1	NCOA1
MOL004993	8-prenylated eriodictyol	Prostaglandin G/H synthase 2	PTGS2
MOL004993	8-prenylated eriodictyol	Sodium channel protein type 5 subunit alpha	SCN5A
MOL004996	gadelaidic acid	Nuclear receptor coactivator 2	NCOA2
MOL005000	Gancaonin G	Androgen receptor	AR
MOL005000	Gancaonin G	Cyclin-A2	CCNA2
MOL005000	Gancaonin G	Serine/threonine-protein kinase Chk1	CHEK1
MOL005000	Gancaonin G	Estrogen receptor	ESR1
MOL005000	Gancaonin G	Estrogen receptor beta	ESR2
MOL005000	Gancaonin G	Glycogen synthase kinase-3 beta	GSK3B
MOL005000	Gancaonin G	Mitogen-activated protein kinase 14	MAPK14
MOL005000	Gancaonin G	Nuclear receptor coactivator 2	NCOA2
MOL005000	Gancaonin G	Nitric oxide synthase, inducible	NOS2
MOL005000	Gancaonin G	Prostaglandin G/H synthase 2	PTGS2
MOL005001	Gancaonin H	Androgen receptor	AR
MOL005001	Gancaonin H	Cyclin-A2	CCNA2
MOL005001	Gancaonin H	Estrogen receptor	ESR1
MOL005001	Gancaonin H	Vascular endothelial growth factor receptor 2	KDR
MOL005001	Gancaonin H	Nuclear receptor coactivator 2	NCOA2
MOL005001	Gancaonin H	Prostaglandin G/H synthase 2	PTGS2
MOL005003	Licoagrocarpin	Acetylcholinesterase	ACHE
MOL005003	Licoagrocarpin	Alpha-1B adrenergic receptor	ADRA1B
MOL005003	Licoagrocarpin	Beta-2 adrenergic receptor	ADRB2
MOL005003	Licoagrocarpin	Androgen receptor	AR
MOL005003	Licoagrocarpin	Cyclin-A2	CCNA2
MOL005003	Licoagrocarpin	Muscarinic acetylcholine receptor M1	CHRM1
MOL005003	Licoagrocarpin	Muscarinic acetylcholine receptor M3	CHRM3
MOL005003	Licoagrocarpin	Muscarinic acetylcholine receptor M5	CHRM5
MOL005003	Licoagrocarpin	Estrogen receptor	ESR1
MOL005003	Licoagrocarpin	Estrogen receptor beta	ESR2
MOL005003	Licoagrocarpin	Glycogen synthase kinase-3 beta	GSK3B
MOL005003	Licoagrocarpin	Potassium voltage-gated channel subfamily H member 2	KCNH2
MOL005003	Licoagrocarpin	Mitogen-activated protein kinase 14	MAPK14
MOL005003	Licoagrocarpin	Nuclear receptor coactivator 2	NCOA2
MOL005003	Licoagrocarpin	Nitric oxide synthase, inducible	NOS2
MOL005003	Licoagrocarpin	Prostaglandin G/H synthase 1	PTGS1
MOL005003	Licoagrocarpin	Prostaglandin G/H synthase 2	PTGS2
MOL005003	Licoagrocarpin	Retinoic acid receptor RXR-alpha	RXRA
MOL005003	Licoagrocarpin	Retinoic acid receptor RXR-beta	RXRB
MOL005003	Licoagrocarpin	Sodium channel protein type 5 subunit alpha	SCN5A
MOL005007	Glyasperins M	Acetylcholinesterase	ACHE
MOL005007	Glyasperins M	Androgen receptor	AR
MOL005007	Glyasperins M	Cyclin-A2	CCNA2
MOL005007	Glyasperins M	Estrogen receptor	ESR1
MOL005007	Glyasperins M	Estrogen receptor beta	ESR2
MOL005007	Glyasperins M	Coagulation factor VII	F7
MOL005007	Glyasperins M	Glycogen synthase kinase-3 beta	GSK3B
MOL005007	Glyasperins M	Potassium voltage-gated channel subfamily H member 2	KCNH2
MOL005007	Glyasperins M	Vascular endothelial growth factor receptor 2	KDR
MOL005007	Glyasperins M	Nuclear receptor coactivator 1	NCOA1
MOL005007	Glyasperins M	Nuclear receptor coactivator 2	NCOA2

Targets related to Gancao

MolId	MolName	Target	Symbol
MOL005007	Glyasperins M	Nitric oxide synthase, inducible	NOS2
MOL005007	Glyasperins M	Prostaglandin G/H synthase 1	PTGS1
MOL005007	Glyasperins M	Prostaglandin G/H synthase 2	PTGS2
MOL005007	Glyasperins M	Sodium channel protein type 5 subunit alpha	SCN5A
MOL005008	Glycyrrhiza flavonol A	Acetylcholinesterase	ACHE
MOL005008	Glycyrrhiza flavonol A	Androgen receptor	AR
MOL005008	Glycyrrhiza flavonol A	Cyclin-A2	CCNA2
MOL005008	Glycyrrhiza flavonol A	Estrogen receptor	ESR1
MOL005008	Glycyrrhiza flavonol A	Estrogen receptor beta	ESR2
MOL005008	Glycyrrhiza flavonol A	Coagulation factor VII	F7
MOL005008	Glycyrrhiza flavonol A	Glycogen synthase kinase-3 beta	GSK3B
MOL005008	Glycyrrhiza flavonol A	Nitric oxide synthase, inducible	NOS2
MOL005008	Glycyrrhiza flavonol A	Prostaglandin G/H synthase 2	PTGS2
MOL005012	Licoagroisoflavone	Androgen receptor	AR
MOL005012	Licoagroisoflavone	Cyclin-A2	CCNA2
MOL005012	Licoagroisoflavone	Serine/threonine-protein kinase Chk1	CHEK1
MOL005012	Licoagroisoflavone	Estrogen receptor	ESR1
MOL005012	Licoagroisoflavone	Estrogen receptor beta	ESR2
MOL005012	Licoagroisoflavone	Glycogen synthase kinase-3 beta	GSK3B
MOL005012	Licoagroisoflavone	Mitogen-activated protein kinase 14	MAPK14
MOL005012	Licoagroisoflavone	Nitric oxide synthase, inducible	NOS2
MOL005012	Licoagroisoflavone	Prostaglandin G/H synthase 2	PTGS2
MOL005012	Licoagroisoflavone	Sodium channel protein type 5 subunit alpha	SCN5A
MOL005016	Odoratin	Androgen receptor	AR
MOL005016	Odoratin	Cyclin-A2	CCNA2
MOL005016	Odoratin	Serine/threonine-protein kinase Chk1	CHEK1
MOL005016	Odoratin	Estrogen receptor	ESR1
MOL005016	Odoratin	Estrogen receptor beta	ESR2
MOL005016	Odoratin	Glycogen synthase kinase-3 beta	GSK3B
MOL005016	Odoratin	Mitogen-activated protein kinase 14	MAPK14
MOL005016	Odoratin	Nuclear receptor coactivator 2	NCOA2
MOL005016	Odoratin	Nitric oxide synthase, inducible	NOS2
MOL005016	Odoratin	Prostaglandin G/H synthase 1	PTGS1
MOL005016	Odoratin	Prostaglandin G/H synthase 2	PTGS2
MOL005016	Odoratin	Retinoic acid receptor RXR-alpha	RXRA
MOL005016	Odoratin	Sodium channel protein type 5 subunit alpha	SCN5A
MOL005017	Phaseol	Androgen receptor	AR
MOL005017	Phaseol	Cyclin-A2	CCNA2
MOL005017	Phaseol	Serine/threonine-protein kinase Chk1	CHEK1
MOL005017	Phaseol	Estrogen receptor	ESR1
MOL005017	Phaseol	Glycogen synthase kinase-3 beta	GSK3B
MOL005017	Phaseol	Vascular endothelial growth factor receptor 2	KDR
MOL005017	Phaseol	Mitogen-activated protein kinase 14	MAPK14
MOL005017	Phaseol	Prostaglandin G/H synthase 2	PTGS2
MOL005018	Xambioona	Estrogen receptor	ESR1
MOL005018	Xambioona	Estrogen receptor beta	ESR2
MOL005018	Xambioona	Nuclear receptor coactivator 2	NCOA2
MOL005018	Xambioona	Nitric oxide synthase, inducible	NOS2
MOL005018	Xambioona	Prostaglandin G/H synthase 2	PTGS2
MOL005020	dehydroglyasperins C	Beta-2 adrenergic receptor	ADRB2
MOL005020	dehydroglyasperins C	Androgen receptor	AR
MOL005020	dehydroglyasperins C	Cyclin-A2	CCNA2
MOL005020	dehydroglyasperins C	Serine/threonine-protein kinase Chk1	CHEK1
MOL005020	dehydroglyasperins C	Estrogen receptor	ESR1
MOL005020	dehydroglyasperins C	Estrogen receptor beta	ESR2
MOL005020	dehydroglyasperins C	Mitogen-activated protein kinase 14	MAPK14
MOL005020	dehydroglyasperins C	Nuclear receptor coactivator 2	NCOA2
MOL005020	dehydroglyasperins C	Nitric oxide synthase, inducible	NOS2
MOL005020	dehydroglyasperins C	Prostaglandin G/H synthase 2	PTGS2
MOL005020	dehydroglyasperins C	Sodium channel protein type 5 subunit alpha	SCN5A

Targets related to Dazao

MolId	MolName	Target	Symbol
MOL000098	quercetin	Acetyl-CoA carboxylase 1	ACACA
MOL000098	quercetin	Acetylcholinesterase	ACHE
MOL007213	Nuciferin	Acetylcholinesterase	ACHE
MOL000098	quercetin	Prostatic acid phosphatase	ACP3
MOL000449	Stigmasterol	Alcohol dehydrogenase 1C	ADH1C
MOL001522	(S)-Coclaurine	Alpha-1A adrenergic receptor	ADRA1A
MOL012921	stepharine	Alpha-1A adrenergic receptor	ADRA1A
MOL007213	Nuciferin	Alpha-1A adrenergic receptor	ADRA1A
MOL000358	beta-sitosterol	Alpha-1A adrenergic receptor	ADRA1A
MOL000449	Stigmasterol	Alpha-1A adrenergic receptor	ADRA1A
MOL001522	(S)-Coclaurine	Alpha-1B adrenergic receptor	ADRA1B
MOL012921	stepharine	Alpha-1B adrenergic receptor	ADRA1B
MOL007213	Nuciferin	Alpha-1B adrenergic receptor	ADRA1B
MOL000627	Stepholidine	Alpha-1B adrenergic receptor	ADRA1B
MOL000358	beta-sitosterol	Alpha-1B adrenergic receptor	ADRA1B
MOL000449	Stigmasterol	Alpha-1B adrenergic receptor	ADRA1B
MOL000787	Fumarine	Alpha-1B adrenergic receptor	ADRA1B
MOL001522	(S)-Coclaurine	Alpha-1D adrenergic receptor	ADRA1D
MOL007213	Nuciferin	Alpha-1D adrenergic receptor	ADRA1D
MOL000627	Stepholidine	Alpha-1D adrenergic receptor	ADRA1D
MOL000787	Fumarine	Alpha-1D adrenergic receptor	ADRA1D
MOL001522	(S)-Coclaurine	Alpha-2A adrenergic receptor	ADRA2A
MOL000449	Stigmasterol	Alpha-2A adrenergic receptor	ADRA2A
MOL012921	stepharine	Alpha-2B adrenergic receptor	ADRA2B
MOL007213	Nuciferin	Alpha-2B adrenergic receptor	ADRA2B
MOL000627	Stepholidine	Alpha-2B adrenergic receptor	ADRA2B
MOL001522	(S)-Coclaurine	Alpha-2C adrenergic receptor	ADRA2C
MOL000627	Stepholidine	Alpha-2C adrenergic receptor	ADRA2C
MOL000449	Stigmasterol	Beta-1 adrenergic receptor	ADRB1
MOL001522	(S)-Coclaurine	Beta-2 adrenergic receptor	ADRB2
MOL012921	stepharine	Beta-2 adrenergic receptor	ADRB2
MOL000098	quercetin	Beta-2 adrenergic receptor	ADRB2
MOL007213	Nuciferin	Beta-2 adrenergic receptor	ADRB2
MOL000627	Stepholidine	Beta-2 adrenergic receptor	ADRB2
MOL000358	beta-sitosterol	Beta-2 adrenergic receptor	ADRB2
MOL000449	Stigmasterol	Beta-2 adrenergic receptor	ADRB2
MOL001454	berberine	Beta-2 adrenergic receptor	ADRB2
MOL000787	Fumarine	Beta-2 adrenergic receptor	ADRB2
MOL000098	quercetin	Aryl hydrocarbon receptor	AHR
MOL000098	quercetin	Activator of 90 kDa heat shock protein ATPase homolog 1	AHSA1
MOL000098	quercetin	RAC-alpha serine/threonine-protein kinase	AKT1
MOL002773	beta-carotene	RAC-alpha serine/threonine-protein kinase	AKT1
MOL000098	quercetin	Androgen receptor	AR
MOL007213	Nuciferin	Androgen receptor	AR
MOL001454	berberine	Androgen receptor	AR
MOL000098	quercetin	Apoptosis regulator BAX	BAX
MOL000358	beta-sitosterol	Apoptosis regulator BAX	BAX
MOL000098	quercetin	Apoptosis regulator Bcl-2	BCL2
MOL002773	beta-carotene	Apoptosis regulator Bcl-2	BCL2
MOL000358	beta-sitosterol	Apoptosis regulator Bcl-2	BCL2
MOL000098	quercetin	Bcl-2-like protein 1	BCL2L1
MOL000098	quercetin	Baculoviral IAP repeat-containing protein 5	BIRC5
MOL000787	Fumarine	Voltage-dependent L-type calcium channel subunit alpha-1S	CACNA1S
MOL000098	quercetin	Caspase-3	CASP3
MOL002773	beta-carotene	Caspase-3	CASP3
MOL000358	beta-sitosterol	Caspase-3	CASP3
MOL002773	beta-carotene	Caspase-7	CASP7
MOL000098	quercetin	Caspase-8	CASP8
MOL002773	beta-carotene	Caspase-8	CASP8
MOL000358	beta-sitosterol	Caspase-8	CASP8
MOL000098	quercetin	Caspase-9	CASP9
MOL002773	beta-carotene	Caspase-9	CASP9
MOL000358	beta-sitosterol	Caspase-9	CASP9
MOL000492	(+)-catechin	Catalase	CAT
MOL000098	quercetin	Caveolin-1	CAV1
MOL002773	beta-carotene	Caveolin-1	CAV1
MOL000098	quercetin	C-C motif chemokine 2	CCL2
MOL000098	quercetin	G2/mitotic-specific cyclin-B1	CCNB1
MOL000098	quercetin	G1/S-specific cyclin-D1	CCND1
MOL000098	quercetin	CD40 ligand	CD40LG
MOL012976	coumestrol	Hsp90 co-chaperone Cdc37	CDC37
MOL000098	quercetin	Cyclin-dependent kinase inhibitor 1	CDKN1A
MOL000098	quercetin	Serine/threonine-protein kinase Chk2	CHEK2
MOL001522	(S)-Coclaurine	Muscarinic acetylcholine receptor M1	CHRM1
MOL012921	stepharine	Muscarinic acetylcholine receptor M1	CHRM1
MOL007213	Nuciferin	Muscarinic acetylcholine receptor M1	CHRM1
MOL000627	Stepholidine	Muscarinic acetylcholine receptor M1	CHRM1
MOL000358	beta-sitosterol	Muscarinic acetylcholine receptor M1	CHRM1
MOL000449	Stigmasterol	Muscarinic acetylcholine receptor M1	CHRM1
MOL000787	Fumarine	Muscarinic acetylcholine receptor M1	CHRM1
MOL012921	stepharine	Muscarinic acetylcholine receptor M2	CHRM2
MOL000627	Stepholidine	Muscarinic acetylcholine receptor M2	CHRM2
MOL000358	beta-sitosterol	Muscarinic acetylcholine receptor M2	CHRM2
MOL000449	Stigmasterol	Muscarinic acetylcholine receptor M2	CHRM2
MOL001522	(S)-Coclaurine	Muscarinic acetylcholine receptor M3	CHRM3
MOL012921	stepharine	Muscarinic acetylcholine receptor M3	CHRM3
MOL007213	Nuciferin	Muscarinic acetylcholine receptor M3	CHRM3
MOL000627	Stepholidine	Muscarinic acetylcholine receptor M3	CHRM3
MOL000358	beta-sitosterol	Muscarinic acetylcholine receptor M3	CHRM3
MOL000449	Stigmasterol	Muscarinic acetylcholine receptor M3	CHRM3
MOL000787	Fumarine	Muscarinic acetylcholine receptor M3	CHRM3
MOL012921	stepharine	Muscarinic acetylcholine receptor M4	CHRM4

Targets related to Dazao

MolId	MolName	Target	Symbol
MOL007213	Nuciferin	Muscarinic acetylcholine receptor M4	CHRM4
MOL000627	Stepholidine	Muscarinic acetylcholine receptor M4	CHRM4
MOL000358	beta-sitosterol	Muscarinic acetylcholine receptor M4	CHRM4
MOL000787	Fumarine	Muscarinic acetylcholine receptor M4	CHRM4
MOL012921	stepharine	Muscarinic acetylcholine receptor M5	CHRM5
MOL007213	Nuciferin	Muscarinic acetylcholine receptor M5	CHRM5
MOL000627	Stepholidine	Muscarinic acetylcholine receptor M5	CHRM5
MOL000787	Fumarine	Muscarinic acetylcholine receptor M5	CHRM5
MOL012921	stepharine	Neuronal acetylcholine receptor subunit alpha-2	CHRNA2
MOL007213	Nuciferin	Neuronal acetylcholine receptor subunit alpha-2	CHRNA2
MOL000358	beta-sitosterol	Neuronal acetylcholine receptor subunit alpha-2	CHRNA2
MOL000098	quercetin	Inhibitor of nuclear factor kappa-B kinase subunit alpha	CHUK
MOL000098	quercetin	Claudin-4	CLDN4
MOL000098	quercetin	C-reactive protein	CRP
MOL002773	beta-carotene	Catenin beta-1	CTNNB1
MOL000449	Stigmasterol	Chymotrypsinogen B	CTRB1
MOL000098	quercetin	Cathepsin D	CTSD
MOL000098	quercetin	C-X-C motif chemokine 10	CXCL10
MOL000098	quercetin	C-X-C motif chemokine 11	CXCL11
MOL000098	quercetin	C-X-C motif chemokine 2	CXCL2
MOL000098	quercetin	Interleukin-8	CXCL8
MOL000098	quercetin	Cytochrome P450 1A1	CYP1A1
MOL000098	quercetin	Cytochrome P450 1A2	CYP1A2
MOL002773	beta-carotene	Cytochrome P450 1A2	CYP1A2
MOL000098	quercetin	Cytochrome P450 1B1	CYP1B1
MOL012976	coumestrol	Cytochrome P450 2B6	CYP2B6
MOL000098	quercetin	Cytochrome P450 3A4	CYP3A4
MOL012976	coumestrol	Cytochrome P450 3A4	CYP3A4
MOL002773	beta-carotene	Cytochrome P450 3A4	CYP3A4
MOL000098	quercetin	DDB1- and CUL4-associated factor 5	DCAF5
MOL000098	quercetin	Type I iodothyronine deiodinase	DIO1
MOL000098	quercetin	Dual oxidase 2	DUOX2
MOL000098	quercetin	Transcription factor E2F1	E2F1
MOL000098	quercetin	Transcription factor E2F2	E2F2
MOL000098	quercetin	Pro-epidermal growth factor	EGF
MOL000098	quercetin	Epidermal growth factor receptor	EGFR
MOL000098	quercetin	Eukaryotic translation initiation factor 6	EIF6
MOL000098	quercetin	ETS domain-containing protein Elk-1	ELK1
MOL000098	quercetin	Receptor tyrosine-protein kinase erbB-2	ERBB2
MOL000098	quercetin	Receptor tyrosine-protein kinase erbB-3	ERBB3
MOL000096	(-)-catechin	Estrogen receptor	ESR1
MOL000492	(+)-catechin	Estrogen receptor	ESR1
MOL001454	berberine	Estrogen receptor	ESR1
MOL000098	quercetin	Tissue factor	F3
MOL002773	beta-carotene	Tissue factor	F3
MOL000098	quercetin	Coagulation factor VII	F7
MOL000787	Fumarine	Coagulation factor VII	F7
MOL000096	(-)-catechin	Fatty acid synthase	FASN
MOL012921	stepharine	Gamma-aminobutyric acid receptor subunit alpha-1	GABRA1
MOL000098	quercetin	Gamma-aminobutyric acid receptor subunit alpha-1	GABRA1
MOL007213	Nuciferin	Gamma-aminobutyric acid receptor subunit alpha-1	GABRA1
MOL000358	beta-sitosterol	Gamma-aminobutyric acid receptor subunit alpha-1	GABRA1
MOL000449	Stigmasterol	Gamma-aminobutyric acid receptor subunit alpha-1	GABRA1
MOL000098	quercetin	Gap junction alpha-1 protein	GJA1
MOL002773	beta-carotene	Gap junction alpha-1 protein	GJA1
MOL000098	quercetin	Glutathione S-transferase Mu 1	GSTM1
MOL000098	quercetin	Glutathione S-transferase Mu 2	GSTM2
MOL000098	quercetin	Glutathione S-transferase P	GSTP1
MOL000492	(+)-catechin	Hyaluronan synthase 2	HAS2
MOL000098	quercetin	Hyaluronan synthase 2	HAS2
MOL000098	quercetin	Hypoxia-inducible factor 1-alpha	HIF1A
MOL000098	quercetin	Hexokinase-2	HK2
MOL000098	quercetin	Heme oxygenase 1	HMOX1
MOL002773	beta-carotene	Heme oxygenase 1	HMOX1
MOL000098	quercetin	Heat shock factor protein 1	HSF1
MOL000098	quercetin	Heat shock protein beta-1	HSPB1
MOL012921	stepharine	5-hydroxytryptamine receptor 3A	HTR3A
MOL007213	Nuciferin	5-hydroxytryptamine receptor 3A	HTR3A
MOL000787	Fumarine	5-hydroxytryptamine receptor 3A	HTR3A
MOL000098	quercetin	Intercellular adhesion molecule 1	ICAM1
MOL000098	quercetin	Interferon gamma	IFNG
MOL000098	quercetin	Insulin-like growth factor II	IGF2
MOL000098	quercetin	Insulin-like growth factor-binding protein 3	IGFBP3
MOL000098	quercetin	Interleukin-10	IL10
MOL000098	quercetin	Interleukin-1 alpha	IL1A
MOL000098	quercetin	Interleukin-1 beta	IL1B
MOL000098	quercetin	Interleukin-2	IL2
MOL000098	quercetin	Interleukin-6	IL6
MOL000098	quercetin	Insulin receptor	INSR
MOL000098	quercetin	Interferon regulatory factor 1	IRF1
MOL000098	quercetin	Potassium voltage-gated channel subfamily H member 2	KCNH2
MOL000627	Stepholidine	Potassium voltage-gated channel subfamily H member 2	KCNH2
MOL000358	beta-sitosterol	Potassium voltage-gated channel subfamily H member 2	KCNH2
MOL001454	berberine	Potassium voltage-gated channel subfamily H member 2	KCNH2
MOL000787	Fumarine	Potassium voltage-gated channel subfamily H member 2	KCNH2
MOL000787	Fumarine	Vascular endothelial growth factor receptor 2	KDR
MOL000096	(-)-catechin	Krueppel-like factor 7	KLF7
MOL000449	Stigmasterol	Leukotriene A-4 hydrolase	LTA4H
MOL000358	beta-sitosterol	Microtubule-associated protein 2	MAP2
MOL000098	quercetin	Mitogen-activated protein kinase 1	MAPK1
MOL000098	quercetin	Interstitial collagenase	MMP1
MOL002773	beta-carotene	Interstitial collagenase	MMP1

Targets related to Dazao

MolId	MolName	Target	Symbol
MOL002773	beta-carotene	Stromelysin-2	MMP10
MOL000098	quercetin	72 kDa type IV collagenase	MMP2
MOL002773	beta-carotene	72 kDa type IV collagenase	MMP2
MOL000098	quercetin	Stromelysin-1	MMP3
MOL000098	quercetin	Matrix metalloproteinase-9	MMP9
MOL000098	quercetin	Myeloperoxidase	MPO
MOL000098	quercetin	Myc proto-oncogene protein	MYC
MOL002773	beta-carotene	Myc proto-oncogene protein	MYC
MOL000098	quercetin	Neutrophil cytosol factor 1	NCF1
MOL000449	Stigmasterol	Nuclear receptor coactivator 1	NCOA1
MOL004350	Ruvoside_qt	Nuclear receptor coactivator 1	NCOA1
MOL001522	(S)-Coclaurine	Nuclear receptor coactivator 2	NCOA2
MOL000096	(-)-catechin	Nuclear receptor coactivator 2	NCOA2
MOL000492	(+)-catechin	Nuclear receptor coactivator 2	NCOA2
MOL000098	quercetin	Nuclear receptor coactivator 2	NCOA2
MOL000358	beta-sitosterol	Nuclear receptor coactivator 2	NCOA2
MOL000449	Stigmasterol	Nuclear receptor coactivator 2	NCOA2
MOL001454	berberine	Nuclear receptor coactivator 2	NCOA2
MOL000098	quercetin	Nuclear factor erythroid 2-related factor 2	NFE2L2
MOL000098	quercetin	NF-kappa-B inhibitor alpha	NFKBIA
MOL000098	quercetin	Homeobox protein Nkx-3.1	NKX3-1
MOL001454	berberine	Nitric oxide synthase, inducible	NOS2
MOL000098	quercetin	Nitric oxide synthase, endothelial	NOS3
MOL000098	quercetin	Puromycin-sensitive aminopeptidase	NPEPPS
MOL000098	quercetin	Nuclear receptor subfamily 1 group I member 2	NR1I2
MOL012976	coumestrol	Nuclear receptor subfamily 1 group I member 2	NR1I2
MOL000098	quercetin	Nuclear receptor subfamily 1 group I member 3	NR1I3
MOL012946	zizyphus saponin I_qt	Mineralocorticoid receptor	NR3C2
MOL012986	Jujubasaponin V_qt	Mineralocorticoid receptor	NR3C2
MOL000449	Stigmasterol	Mineralocorticoid receptor	NR3C2
MOL004350	Ruvoside_qt	Mineralocorticoid receptor	NR3C2
MOL000098	quercetin	Ornithine decarboxylase	ODC1
MOL012921	stepharine	Delta-type opioid receptor	OPRD1
MOL007213	Nuciferin	Delta-type opioid receptor	OPRD1
MOL000627	Stepholidine	Delta-type opioid receptor	OPRD1
MOL000787	Fumarine	Delta-type opioid receptor	OPRD1
MOL001522	(S)-Coclaurine	Mu-type opioid receptor	OPRM1
MOL012921	stepharine	Mu-type opioid receptor	OPRM1
MOL007213	Nuciferin	Mu-type opioid receptor	OPRM1
MOL000627	Stepholidine	Mu-type opioid receptor	OPRM1
MOL000358	beta-sitosterol	Mu-type opioid receptor	OPRM1
MOL000787	Fumarine	Mu-type opioid receptor	OPRM1
MOL000098	quercetin	Procollagen C-endopeptidase enhancer 1	PCOLCE
MOL001454	berberine	cAMP and cAMP-inhibited cGMP 3',5'-cyclic phosphodiesterase 10A	PDE10A
MOL001522	(S)-Coclaurine	CGMP-inhibited 3',5'-cyclic phosphodiesterase A	PDE3A
MOL000358	beta-sitosterol	CGMP-inhibited 3',5'-cyclic phosphodiesterase A	PDE3A
MOL000787	Fumarine	CGMP-inhibited 3',5'-cyclic phosphodiesterase A	PDE3A
MOL000358	beta-sitosterol	Progesterone receptor	PGR
MOL000449	Stigmasterol	Progesterone receptor	PGR
MOL000211	Mairin	Progesterone receptor	PGR
MOL000098	quercetin	Tissue-type plasminogen activator	PLAT
MOL000098	quercetin	Urokinase-type plasminogen activator	PLAU
MOL000449	Stigmasterol	Urokinase-type plasminogen activator	PLAU
MOL000098	quercetin	Serum paraoxonase/arylesterase 1	PON1
MOL000358	beta-sitosterol	Serum paraoxonase/arylesterase 1	PON1
MOL000098	quercetin	NADPH--cytochrome P450 reductase	POR
MOL000098	quercetin	Peroxisome proliferator-activated receptor alpha	PPARA
MOL000098	quercetin	Peroxisome proliferator-activated receptor delta	PPARD
MOL000096	(-)-catechin	Peroxisome proliferator-activated receptor gamma	PPARG
MOL000098	quercetin	Peroxisome proliferator-activated receptor gamma	PPARG
MOL000098	quercetin	Protein kinase C alpha type	PRKCA
MOL000358	beta-sitosterol	Protein kinase C alpha type	PRKCA
MOL000098	quercetin	Protein kinase C beta type	PRKCB
MOL000098	quercetin	26S proteasome non-ATPase regulatory subunit 3	PSMD3
MOL000098	quercetin	Prostaglandin E2 receptor EP3 subtype	PTGER3
MOL001522	(S)-Coclaurine	Prostaglandin G/H synthase 1	PTGS1
MOL000096	(-)-catechin	Prostaglandin G/H synthase 1	PTGS1
MOL000492	(+)-catechin	Prostaglandin G/H synthase 1	PTGS1
MOL012921	stepharine	Prostaglandin G/H synthase 1	PTGS1
MOL000098	quercetin	Prostaglandin G/H synthase 1	PTGS1
MOL007213	Nuciferin	Prostaglandin G/H synthase 1	PTGS1
MOL000627	Stepholidine	Prostaglandin G/H synthase 1	PTGS1
MOL000358	beta-sitosterol	Prostaglandin G/H synthase 1	PTGS1
MOL000449	Stigmasterol	Prostaglandin G/H synthase 1	PTGS1
MOL001454	berberine	Prostaglandin G/H synthase 1	PTGS1
MOL000787	Fumarine	Prostaglandin G/H synthase 1	PTGS1
MOL001522	(S)-Coclaurine	Prostaglandin G/H synthase 2	PTGS2
MOL000096	(-)-catechin	Prostaglandin G/H synthase 2	PTGS2
MOL000492	(+)-catechin	Prostaglandin G/H synthase 2	PTGS2
MOL012921	stepharine	Prostaglandin G/H synthase 2	PTGS2
MOL000098	quercetin	Prostaglandin G/H synthase 2	PTGS2
MOL012976	coumestrol	Prostaglandin G/H synthase 2	PTGS2
MOL007213	Nuciferin	Prostaglandin G/H synthase 2	PTGS2
MOL012992	Mauritine D	Prostaglandin G/H synthase 2	PTGS2
MOL000627	Stepholidine	Prostaglandin G/H synthase 2	PTGS2
MOL002773	beta-carotene	Prostaglandin G/H synthase 2	PTGS2
MOL000358	beta-sitosterol	Prostaglandin G/H synthase 2	PTGS2
MOL000449	Stigmasterol	Prostaglandin G/H synthase 2	PTGS2
MOL001454	berberine	Prostaglandin G/H synthase 2	PTGS2
MOL000787	Fumarine	Prostaglandin G/H synthase 2	PTGS2
MOL000098	quercetin	RAF proto-oncogene serine/threonine-protein kinase	RAF1
MOL000098	quercetin	Ras GTPase-activating protein 1	RASA1

Targets related to Dazao

MolId	MolName	Target	Symbol
MOL000098	quercetin	Ras association domain-containing protein 1	RASSF1
MOL000098	quercetin	Retinoblastoma-associated protein	RB1
MOL000098	quercetin	Transcription factor p65	RELA
MOL000098	quercetin	Protein CBFA2T1	RUNX1T1
MOL000098	quercetin	Runt-related transcription factor 2	RUNX2
MOL001522	(S)-Coclaurine	Retinoic acid receptor RXR-alpha	RXRA
MOL000492	(+)-catechin	Retinoic acid receptor RXR-alpha	RXRA
MOL012921	stepharine	Retinoic acid receptor RXR-alpha	RXRA
MOL000098	quercetin	Retinoic acid receptor RXR-alpha	RXRA
MOL007213	Nuciferin	Retinoic acid receptor RXR-alpha	RXRA
MOL000627	Stepholidine	Retinoic acid receptor RXR-alpha	RXRA
MOL000449	Stigmasterol	Retinoic acid receptor RXR-alpha	RXRA
MOL001454	berberine	Retinoic acid receptor RXR-alpha	RXRA
MOL001522	(S)-Coclaurine	Sodium channel protein type 5 subunit alpha	SCN5A
MOL012921	stepharine	Sodium channel protein type 5 subunit alpha	SCN5A
MOL000098	quercetin	Sodium channel protein type 5 subunit alpha	SCN5A
MOL007213	Nuciferin	Sodium channel protein type 5 subunit alpha	SCN5A
MOL012992	Mauritine D	Sodium channel protein type 5 subunit alpha	SCN5A
MOL000627	Stepholidine	Sodium channel protein type 5 subunit alpha	SCN5A
MOL000358	beta-sitosterol	Sodium channel protein type 5 subunit alpha	SCN5A
MOL000449	Stigmasterol	Sodium channel protein type 5 subunit alpha	SCN5A
MOL001454	berberine	Sodium channel protein type 5 subunit alpha	SCN5A
MOL000787	Fumarine	Sodium channel protein type 5 subunit alpha	SCN5A
MOL000098	quercetin	E-selectin	SELE
MOL000098	quercetin	Plasminogen activator inhibitor 1	SERPINE1
MOL000098	quercetin	Solute carrier family 2, facilitated glucose transporter member 4	SLC2A4
MOL012921	stepharine	Sodium-dependent noradrenaline transporter	SLC6A2
MOL007213	Nuciferin	Sodium-dependent noradrenaline transporter	SLC6A2
MOL000449	Stigmasterol	Sodium-dependent noradrenaline transporter	SLC6A2
MOL001522	(S)-Coclaurine	Sodium-dependent dopamine transporter	SLC6A3
MOL012921	stepharine	Sodium-dependent dopamine transporter	SLC6A3
MOL007213	Nuciferin	Sodium-dependent dopamine transporter	SLC6A3
MOL000627	Stepholidine	Sodium-dependent dopamine transporter	SLC6A3
MOL000449	Stigmasterol	Sodium-dependent dopamine transporter	SLC6A3
MOL000787	Fumarine	Sodium-dependent dopamine transporter	SLC6A3
MOL001522	(S)-Coclaurine	Sodium-dependent serotonin transporter	SLC6A4
MOL012921	stepharine	Sodium-dependent serotonin transporter	SLC6A4
MOL007213	Nuciferin	Sodium-dependent serotonin transporter	SLC6A4
MOL000627	Stepholidine	Sodium-dependent serotonin transporter	SLC6A4
MOL000358	beta-sitosterol	Sodium-dependent serotonin transporter	SLC6A4
MOL000787	Fumarine	Sodium-dependent serotonin transporter	SLC6A4
MOL000098	quercetin	Osteopontin	SPP1
MOL000098	quercetin	Signal transducer and activator of transcription 1-alpha/beta	STAT1
MOL000098	quercetin	Thrombomodulin	THBD
MOL000098	quercetin	Tumor necrosis factor	TNF
MOL000098	quercetin	DNA topoisomerase 1	TOP1
MOL000098	quercetin	DNA topoisomerase 2-alpha	TOP2A
MOL000098	quercetin	Cellular tumor antigen p53	TP53
MOL000098	quercetin	Vascular cell adhesion protein 1	VCAM1
MOL000098	quercetin	Vascular endothelial growth factor A	VEGFA
MOL002773	beta-carotene	Vascular endothelial growth factor A	VEGFA
MOL000098	quercetin	Xanthine dehydrogenase/oxidase	XDH
