# Supplementary material for: Exploring the potential of Huangqin Tang in breast cancer treatment using network pharmacological analysis and experimental verification
Source: BMC Complement Med Ther. 2024 Jun 7;24:221. doi: 10.1186/s12906-024-04523-0 (PMC11161988; doi:10.1186/s12906-024-04523-0)
Supplement: Supplementary file 3 — Supplementary Material 3 [file 12906_2024_4523_MOESM3_ESM.pdf]

## Breast cancer-related targets

| Gene Symbol | Description | Category | Gifts | GC Id | Relevance score |
|---|---|---|---|---|---|
| BRCA2 | BRCA2 DNA Repair Associated | Protein Coding | 43 | GC13P032315 | 401.1433716 |
| BRCA1 | BRCA1 DNA Repair Associated | Protein Coding | 45 | GC17M043044 | 394.7716064 |
| TP53 | Tumor Protein P53 | Protein Coding | 47 | GC17M007661 | 245.7268066 |
| PALB2 | Partner And Localizer Of BRCA2 | Protein Coding | 39 | GC16M023603 | 219.8490906 |
| CHEK2 | Checkpoint Kinase 2 | Protein Coding | 47 | GC22M028687 | 219.7015381 |
| ATM | ATM Serine/Threonine Kinase | Protein Coding | 47 | GC11P108222 | 210.1045532 |
| CDH1 | Cadherin 1 | Protein Coding | 44 | GC16P068737 | 200.7112732 |
| BRIP1 | BRCA1 Interacting Helicase 1 | Protein Coding | 43 | GC17M061679 | 195.986969 |
| PTEN | Phosphatase And Tensin Homolog | Protein Coding | 47 | GC10P091563 | 183.807373 |
| MSH2 | MutS Homolog 2 | Protein Coding | 43 | GC02P047403 | 181.7635498 |
| MSH6 | MutS Homolog 6 | Protein Coding | 44 | GC02P047695 | 181.4637909 |
| MLH1 | MutL Homolog 1 | Protein Coding | 43 | GC03P036993 | 176.2012024 |
| ERBB2 | Erb-B2 Receptor Tyrosine Kinase 2 | Protein Coding | 48 | GC17P039687 | 173.9440308 |
| BARD1 | BRCA1 Associated RING Domain 1 | Protein Coding | 42 | GC02M214725 | 172.3237457 |
| APC | APC Regulator Of WNT Signaling Pathway | Protein Coding | 43 | GC05P112707 | 167.759079 |
| RAD51D | RAD51 Paralog D | Protein Coding | 35 | GC17M035092 | 158.8196716 |
| PIK3CA | Phosphatidylinositol-4,5-Bisphosphate 3-Kinase Catalytic Subunit Alpha | Protein Coding | 47 | GC03P179148 | 158.1931915 |
| NBN | Nibrin | Protein Coding | 42 | GC08M089933 | 153.7332001 |
| PMS2 | PMS1 Homolog 2, Mismatch Repair System Component | Protein Coding | 43 | GC07M005973 | 152.2571411 |
| KRAS | KRAS Proto-Oncogene, GTPase | Protein Coding | 46 | GC12M025204 | 148.1534424 |
| RAD51C | RAD51 Paralog C | Protein Coding | 39 | GC17P058692 | 146.0561371 |
| ESR1 | Estrogen Receptor 1 | Protein Coding | 47 | GC06P151656 | 144.0557404 |
| AKT1 | AKT Serine/Threonine Kinase 1 | Protein Coding | 47 | GC14M104769 | 139.3179321 |
| RAD51L3-RFFL | RAD51L3-RFFL Readthrough | RNA Gene | 10 | GC17M035009 | 136.6577759 |
| C11orf65 | Chromosome 11 Open Reading Frame 65 | Protein Coding | 29 | GC11M108308 | 134.5608215 |
| MUTYH | MutY DNA Glycosylase | Protein Coding | 41 | GC01M045329 | 132.6644897 |
| EGFR | Epidermal Growth Factor Receptor | Protein Coding | 48 | GC07P055019 | 131.9008179 |
| RAD50 | RAD50 Double Strand Break Repair Protein | Protein Coding | 44 | GC05P132556 | 126.6927185 |
| MIR21 | MicroRNA 21 | RNA Gene | 23 | GC17P059841 | 125.9242096 |
| MIR34A | MicroRNA 34a | RNA Gene | 22 | GC01M009151 | 112.6822891 |
| MRE11 | MRE11 Homolog, Double Strand Break Repair Nuclease | Protein Coding | 39 | GC11M095921 | 112.6645966 |
| RET | Ret Proto-Oncogene | Protein Coding | 48 | GC10P043152 | 110.0509644 |
| CDKN2A | Cyclin Dependent Kinase Inhibitor 2A | Protein Coding | 45 | GC09M021967 | 108.188446 |
| CASP8 | Caspase 8 | Protein Coding | 46 | GC02P201233 | 107.48806 |
| MIR221 | MicroRNA 221 | RNA Gene | 19 | GC0XM045746 | 107.4586639 |
| RAD51 | RAD51 Recombinase | Protein Coding | 47 | GC15P040694 | 106.8693695 |
| MIR17 | MicroRNA 17 | RNA Gene | 21 | GC13P091350 | 103.9813843 |
| POLE | DNA Polymerase Epsilon, Catalytic Subunit | Protein Coding | 44 | GC12M132679 | 102.1390305 |
| MIR200B | MicroRNA 200b | RNA Gene | 19 | GC01P001167 | 99.641716 |
| MIR146A | MicroRNA 146a | RNA Gene | 21 | GC05P160485 | 99.43452454 |
| MIR222 | MicroRNA 222 | RNA Gene | 20 | GC0XM045747 | 99.30436707 |
| MIR200C | MicroRNA 200c | RNA Gene | 21 | GC12P019855 | 99.09066772 |
| STK11 | Serine/Threonine Kinase 11 | Protein Coding | 44 | GC19P001177 | 97.45901489 |
| MIR205 | MicroRNA 205 | RNA Gene | 21 | GC01P209432 | 95.82650757 |
| MIR141 | MicroRNA 141 | RNA Gene | 21 | GC12P019856 | 95.2509613 |
| MIR125A | MicroRNA 125a | RNA Gene | 22 | GC19P064198 | 94.94804382 |
| MIR20A | MicroRNA 20a | RNA Gene | 19 | GC13P091544 | 94.40834808 |
| MIR126 | MicroRNA 126 | RNA Gene | 21 | GC09P136670 | 94.12482452 |
| CTNNB1 | Catenin Beta 1 | Protein Coding | 47 | GC03P041236 | 93.84712219 |
| MIR200A | MicroRNA 200a | RNA Gene | 20 | GC01P003591 | 93.25384521 |
| BRAF | B-Raf Proto-Oncogene, Serine/Threonine Kinase | Protein Coding | 47 | GC07M140732 | 93.14012146 |
| SMAD4 | SMAD Family Member 4 | Protein Coding | 45 | GC18P051028 | 92.32136536 |
| MIR155 | MicroRNA 155 | RNA Gene | 21 | GC21P025573 | 92.12945557 |
| XRCC2 | X-Ray Repair Cross Complementing 2 | Protein Coding | 37 | GC07M152644 | 91.04722595 |
| POLD1 | DNA Polymerase Delta 1, Catalytic Subunit | Protein Coding | 41 | GC19P064129 | 90.75523376 |
| FANCC | FA Complementation Group C | Protein Coding | 43 | GC09M095099 | 90.25719452 |
| MIR31 | MicroRNA 31 | RNA Gene | 20 | GC09M021736 | 89.66468811 |
| MIR27A | MicroRNA 27a | RNA Gene | 22 | GC19M014439 | 89.15814209 |
| XRCC3 | X-Ray Repair Cross Complementing 3 | Protein Coding | 38 | GC14M103697 | 88.9382782 |
| MIR127 | MicroRNA 127 | RNA Gene | 20 | GC14P109204 | 88.24166107 |
| AXIN2 | Axin 2 | Protein Coding | 43 | GC17M065528 | 86.38704681 |
| AR | Androgen Receptor | Protein Coding | 47 | GC0XP067544 | 86.35150909 |
| MIR210 | MicroRNA 210 | RNA Gene | 20 | GC11M002880 | 85.11364746 |
| MET | MET Proto-Oncogene, Receptor Tyrosine Kinase | Protein Coding | 48 | GC07P116672 | 85.00702667 |
| MIR10B | MicroRNA 10b | RNA Gene | 21 | GC02P176150 | 82.84472656 |
| PPM1D | Protein Phosphatase, Mg2+/Mn2+ Dependent 1D | Protein Coding | 43 | GC17P060600 | 81.91273499 |
| NF1 | Neurofibromin 1 | Protein Coding | 43 | GC17P031094 | 81.14781952 |
| CCND1 | Cyclin D1 | Protein Coding | 46 | GC11P069641 | 81.09307098 |
| FANCM | FA Complementation Group M | Protein Coding | 38 | GC14P045135 | 80.53436279 |
| SLC22A18 | Solute Carrier Family 22 Member 18 | Protein Coding | 38 | GC11P002899 | 80.31819153 |
| RB1 | RB Transcriptional Corepressor 1 | Protein Coding | 42 | GC13P048303 | 80.24233246 |
| ABRAXAS1 | Abraxas 1, BRCA1 A Complex Subunit | Protein Coding | 29 | GC04M083460 | 80.10180664 |
| FGFR2 | Fibroblast Growth Factor Receptor 2 | Protein Coding | 48 | GC10M121478 | 79.43762207 |
| MIR451A | MicroRNA 451a | RNA Gene | 19 | GC17M028861 | 79.3785553 |
| MIR182 | MicroRNA 182 | RNA Gene | 20 | GC07M129770 | 79.10193634 |
| MIR96 | MicroRNA 96 | RNA Gene | 22 | GC07M129774 | 78.39910889 |
| MIR429 | MicroRNA 429 | RNA Gene | 18 | GC01P003593 | 78.38942719 |
| KLLN | Killin, P53 Regulated DNA Replication Inhibitor | Protein Coding | 26 | GC10M087860 | 78.36975861 |
| PHB1 | Prohibitin 1 | Protein Coding | 35 | GC17M049406 | 77.81019592 |
| RAD54L | RAD54 Like | Protein Coding | 42 | GC01P046313 | 77.51517487 |
| MIR30E | MicroRNA 30e | RNA Gene | 21 | GC01P040754 | 77.29302979 |
| MIR373 | MicroRNA 373 | RNA Gene | 18 | GC19P064310 | 75.79141235 |
| CTNNA1 | Catenin Alpha 1 | Protein Coding | 42 | GC05P138613 | 75.65458679 |
| MIR335 | MicroRNA 335 | RNA Gene | 19 | GC07P130496 | 75.61483765 |
| MIR204 | MicroRNA 204 | RNA Gene | 22 | GC09M070809 | 74.84855652 |
| CDC73 | Cell Division Cycle 73 | Protein Coding | 40 | GC01P193121 | 74.34609985 |
| MIR499A | MicroRNA 499a | RNA Gene | 21 | GC20P034990 | 73.43591309 |
| DICER1 | Dicer 1, Ribonuclease III | Protein Coding | 43 | GC14M095086 | 73.02610779 |
| RB1CC1 | RB1 Inducible Coiled-Coil 1 | Protein Coding | 37 | GC08M052622 | 72.83372498 |
| MIR145 | MicroRNA 145 | RNA Gene | 21 | GC05P149430 | 72.62837982 |
| MIR146B | MicroRNA 146b | RNA Gene | 20 | GC10P102436 | 72.16836548 |
| HMMR | Hyaluronan Mediated Motility Receptor | Protein Coding | 38 | GC05P163480 | 72.01972961 |

| | | | | | |
|---|---|---|---|---|---|
| MIR206 | MicroRNA 206 | RNA Gene | 20 | GC06P052144 | 71.63612366 |
| EPCAM | Epithelial Cell Adhesion Molecule | Protein Coding | 43 | GC02P047345 | 70.74287415 |
| MDM2 | MDM2 Proto-Oncogene | Protein Coding | 48 | GC12P068808 | 69.68603516 |
| MIR199B | MicroRNA 199b | RNA Gene | 19 | GC09M128244 | 69.33805084 |
| MIR196A2 | MicroRNA 196a-2 | RNA Gene | 22 | GC12P054973 | 69.04385376 |
| FGFR3 | Fibroblast Growth Factor Receptor 3 | Protein Coding | 48 | GC04P001795 | 69.00141144 |
| MIR193B | MicroRNA 193b | RNA Gene | 20 | GC16P014307 | 67.68727875 |
| HRAS | HRas Proto-Oncogene, GTPase | Protein Coding | 47 | GC11M002875 | 67.53601837 |
| TGFBR2 | Transforming Growth Factor Beta Receptor 2 | Protein Coding | 46 | GC03P030623 | 67.21146393 |
| BLM | BLM RecQ Like Helicase | Protein Coding | 44 | GC15P090717 | 66.89498138 |
| MIR128-1 | MicroRNA 128-1 | RNA Gene | 18 | GC02P135665 | 66.0189743 |
| NQO2 | N-Ribosyldihydronicotinamide:Quinone Reductase 2 | Protein Coding | 40 | GC06P003199 | 66.00312805 |
| NRAS | NRAS Proto-Oncogene, GTPase | Protein Coding | 44 | GC01M114704 | 65.34151459 |
| HERC2 | HECT And RLD Domain Containing E3 Ubiquitin Protein Ligase 2 | Protein Coding | 41 | GC15M028111 | 64.46363831 |
| TERT | Telomerase Reverse Transcriptase | Protein Coding | 46 | GC05M001253 | 64.28440857 |
| PTCH1 | Patched 1 | Protein Coding | 44 | GC09M095442 | 63.98075867 |
| MIR520C | MicroRNA 520c | RNA Gene | 18 | GC19P064303 | 63.20370102 |
| BSCL2 | BSCL2 Lipid Droplet Biogenesis Associated, Seipin | Protein Coding | 38 | GC11M087134 | 62.66215515 |
| MIR143 | MicroRNA 143 | RNA Gene | 23 | GC05P149410 | 62.38846207 |
| MIR502 | MicroRNA 502 | RNA Gene | 16 | GC0XP050014 | 62.2041893 |
| MIR9-3 | MicroRNA 9-3 | RNA Gene | 18 | GC15P089368 | 62.16215515 |
| BAP1 | BRCA1 Associated Protein 1 | Protein Coding | 43 | GC03M052401 | 61.69193268 |
| ALK | ALK Receptor Tyrosine Kinase | Protein Coding | 47 | GC02M029190 | 61.22890091 |
| GNG3 | G Protein Subunit Gamma 3 | Protein Coding | 34 | GC11P062707 | 61.22453308 |
| HNRNPUL2-BSCL2 | HNRNPUL2-BSCL2 Readthrough (NMD Candidate) | RNA Gene | 15 | GC11M087135 | 60.80251312 |
| MIR661 | MicroRNA 661 | RNA Gene | 17 | GC08M143945 | 60.7243309 |
| MIR510 | MicroRNA 510 | RNA Gene | 15 | GC0XM147272 | 60.4108963 |
| MLH3 | MutL Homolog 3 | Protein Coding | 38 | GC14M075013 | 59.73960114 |
| BMPR1A | Bone Morphogenetic Protein Receptor Type 1A | Protein Coding | 45 | GC10P091494 | 59.69776917 |
| CDK4 | Cyclin Dependent Kinase 4 | Protein Coding | 47 | GC12M057743 | 59.65292358 |
| CDKN1B | Cyclin Dependent Kinase Inhibitor 1B | Protein Coding | 42 | GC12P020070 | 59.58003998 |
| BAX | BCL2 Associated X, Apoptosis Regulator | Protein Coding | 43 | GC19P048954 | 58.51321793 |
| SMARCA4 | SWI/SNF Related, Matrix Associated, Actin Dependent Regulator Of Chromatin, Subfamily A, Member 4 | Protein Coding | 44 | GC19P010932 | 58.46540451 |
| MIR203A | MicroRNA 203a | RNA Gene | 19 | GC14P109273 | 58.19491577 |
| KIT | KIT Proto-Oncogene, Receptor Tyrosine Kinase | Protein Coding | 47 | GC04P054657 | 57.71308899 |
| MYC | MYC Proto-Oncogene, BHLH Transcription Factor | Protein Coding | 46 | GC08P127735 | 57.21255875 |
| TSC1 | TSC Complex Subunit 1 | Protein Coding | 42 | GC09M132891 | 57.04977798 |
| FASLG | Fas Ligand | Protein Coding | 42 | GC01P172628 | 56.60876465 |
| MSH3 | MutS Homolog 3 | Protein Coding | 39 | GC05P080654 | 56.44869995 |
| HOXB13 | Homeobox B13 | Protein Coding | 38 | GC17M048725 | 55.6555481 |
| MIR34C | MicroRNA 34c | RNA Gene | 21 | GC11P111696 | 55.31592941 |
| AURKA | Aurora Kinase A | Protein Coding | 44 | GC20M056370 | 55.09529114 |
| PGR | Progesterone Receptor | Protein Coding | 44 | GC11M101030 | 54.01527405 |
| SRC | SRC Proto-Oncogene, Non-Receptor Tyrosine Kinase | Protein Coding | 46 | GC20P037344 | 53.82036972 |
| CYP19A1 | Cytochrome P450 Family 19 Subfamily A Member 1 | Protein Coding | 44 | GC15M051208 | 53.370327 |
| BCL2 | BCL2 Apoptosis Regulator | Protein Coding | 45 | GC18M063123 | 53.24340057 |
| MIR15A | MicroRNA 15a | RNA Gene | 17 | GC13M050049 | 53.21144485 |
| IGF2 | Insulin Like Growth Factor 2 | Protein Coding | 43 | GC11M003030 | 52.94005585 |
| TSC2 | TSC Complex Subunit 2 | Protein Coding | 44 | GC16P010714 | 52.7201767 |
| VEGFA | Vascular Endothelial Growth Factor A | Protein Coding | 43 | GC06P043770 | 52.31933594 |
| MAP2K1 | Mitogen-Activated Protein Kinase Kinase 1 | Protein Coding | 48 | GC15P066386 | 52.30592346 |
| IL1B | Interleukin 1 Beta | Protein Coding | 42 | GC02M112829 | 51.7790184 |
| MIR34B | MicroRNA 34b | RNA Gene | 21 | GC11P111704 | 51.36093521 |
| PPARG | Peroxisome Proliferator Activated Receptor Gamma | Protein Coding | 47 | GC03P012287 | 51.33750534 |
| EP300 | E1A Binding Protein P300 | Protein Coding | 46 | GC22P041091 | 50.88924408 |
| SDHB | Succinate Dehydrogenase Complex Iron Sulfur Subunit B | Protein Coding | 43 | GC01M017952 | 50.5535965 |
| MIRLET7D | MicroRNA Let-7d | RNA Gene | 21 | GC09P094178 | 50.18736267 |
| PIK3R1 | Phosphoinositide-3-Kinase Regulatory Subunit 1 | Protein Coding | 46 | GC05P068215 | 49.96168137 |
| STAT3 | Signal Transducer And Activator Of Transcription 3 | Protein Coding | 47 | GC17M042313 | 49.47840881 |
| MUC1 | Mucin 1, Cell Surface Associated | Protein Coding | 44 | GC01M155185 | 49.23511505 |
| MIRLET7C | MicroRNA Let-7c | RNA Gene | 21 | GC21P016551 | 49.17221069 |
| CDKN1A | Cyclin Dependent Kinase Inhibitor 1A | Protein Coding | 43 | GC06P080534 | 48.89817047 |
| MAP3K1 | Mitogen-Activated Protein Kinase Kinase Kinase 1 | Protein Coding | 44 | GC05P056815 | 48.88804245 |
| ESR2 | Estrogen Receptor 2 | Protein Coding | 44 | GC14M064084 | 48.58745575 |
| PMS1 | PMS1 Homolog 1, Mismatch Repair System Component | Protein Coding | 38 | GC02P189784 | 47.97454834 |
| MTOR | Mechanistic Target Of Rapamycin Kinase | Protein Coding | 48 | GC01M011106 | 47.92563248 |
| IL6 | Interleukin 6 | Protein Coding | 44 | GC07P022725 | 47.20645905 |
| MIR93 | MicroRNA 93 | RNA Gene | 21 | GC07M101711 | 47.1968689 |
| FGFR1 | Fibroblast Growth Factor Receptor 1 | Protein Coding | 49 | GC08M038400 | 46.98979187 |
| MIR18A | MicroRNA 18a | RNA Gene | 18 | GC13P091547 | 46.77330017 |
| MIR106B | MicroRNA 106b | RNA Gene | 21 | GC07M101712 | 46.47862625 |
| FLCN | Folliculin | Protein Coding | 38 | GC17M017206 | 46.29430771 |
| ERCC2 | ERCC Excision Repair 2, TFIIH Core Complex Helicase Subunit | Protein Coding | 43 | GC19M045349 | 46.27495575 |
| FBXW7 | F-Box And WD Repeat Domain Containing 7 | Protein Coding | 40 | GC04M152321 | 46.23443222 |
| TGFB1 | Transforming Growth Factor Beta 1 | Protein Coding | 46 | GC19M041301 | 45.93596649 |
| TNF | Tumor Necrosis Factor | Protein Coding | 46 | GC06P080353 | 45.84078217 |
| MIR214 | MicroRNA 214 | RNA Gene | 21 | GC01M172234 | 44.82743073 |
| PRKN | Parkin RBR E3 Ubiquitin Protein Ligase | Protein Coding | 37 | GC06M161348 | 44.7983284 |
| KLF6 | Kruppel Like Factor 6 | Protein Coding | 38 | GC10M003779 | 44.76128387 |
| ZFHX3 | Zinc Finger Homeobox 3 | Protein Coding | 37 | GC16M072782 | 44.55838013 |
| MIR29C | MicroRNA 29c | RNA Gene | 17 | GC01M207802 | 44.47867584 |
| MEN1 | Menin 1 | Protein Coding | 41 | GC11M064803 | 44.26059341 |
| MIR223 | MicroRNA 223 | RNA Gene | 21 | GC0XP066018 | 44.24494553 |
| PRLR | Prolactin Receptor | Protein Coding | 43 | GC05M035048 | 44.08259201 |
| TP63 | Tumor Protein P63 | Protein Coding | 43 | GC03P189598 | 44.02439117 |
| EGF | Epidermal Growth Factor | Protein Coding | 46 | GC04P109912 | 43.91867065 |
| ATR | ATR Serine/Threonine Kinase | Protein Coding | 47 | GC03M142449 | 43.50870514 |
| MIR29A | MicroRNA 29a | RNA Gene | 21 | GC07M130876 | 43.42752075 |
| IRF1 | Interferon Regulatory Factor 1 | Protein Coding | 42 | GC05M132440 | 42.70999146 |
| MIRLET7G | MicroRNA Let-7g | RNA Gene | 19 | GC03M052268 | 42.46589661 |
| SDHD | Succinate Dehydrogenase Complex Subunit D | Protein Coding | 39 | GC11P112087 | 42.36323166 |
| IGF1 | Insulin Like Growth Factor 1 | Protein Coding | 43 | GC12M102395 | 42.33262634 |
| LRRC56 | Leucine Rich Repeat Containing 56 | Protein Coding | 31 | GC11P000518 | 42.22078705 |
| ABCG2 | ATP Binding Cassette Subfamily G Member 2 (Junior Blood Group) | Protein Coding | 45 | GC04M088090 | 42.12527466 |

| | | | | | |
|---|---|---|---|---|---|
| PALLD | Palladin, Cytoskeletal Associated Protein | Protein Coding | 39 | GC04P168497 | 41.97382736 |
| BUB1B | BUB1 Mitotic Checkpoint Serine/Threonine Kinase B | Protein Coding | 44 | GC15P040161 | 41.93107605 |
| PTGS2 | Prostaglandin-Endoperoxide Synthase 2 | Protein Coding | 44 | GC01M186640 | 41.8691864 |
| MIR19A | MicroRNA 19a | RNA Gene | 19 | GC13P091543 | 41.6067276 |
| WWOX | WW Domain Containing Oxidoreductase | Protein Coding | 42 | GC16P078099 | 41.57515717 |
| ERCC6 | ERCC Excision Repair 6, Chromatin Remodeling Factor | Protein Coding | 42 | GC10M049454 | 41.48934174 |
| MMP2 | Matrix Metallopeptidase 2 | Protein Coding | 47 | GC16P055390 | 41.23122787 |
| BCAR4 | Breast Cancer Anti-Estrogen Resistance 4 | RNA Gene | 17 | GC16M012188 | 41.0978241 |
| HIF1A | Hypoxia Inducible Factor 1 Subunit Alpha | Protein Coding | 44 | GC14P061695 | 40.98484421 |
| MSR1 | Macrophage Scavenger Receptor 1 | Protein Coding | 41 | GC08M016107 | 40.92393494 |
| RRAS2 | RAS Related 2 | Protein Coding | 43 | GC11M014299 | 40.72749329 |
| DCC | DCC Netrin 1 Receptor | Protein Coding | 42 | GC18P052340 | 40.69026184 |
| MMP9 | Matrix Metallopeptidase 9 | Protein Coding | 48 | GC20P046008 | 40.43242645 |
| EPHB2 | EPH Receptor B2 | Protein Coding | 46 | GC01P022710 | 40.36902618 |
| GSTM1 | Glutathione S-Transferase Mu 1 | Protein Coding | 37 | GC01P109687 | 40.35544342 |
| FH | Fumarate Hydratase | Protein Coding | 41 | GC01M241499 | 40.01113892 |
| GSTP1 | Glutathione S-Transferase Pi 1 | Protein Coding | 45 | GC11P067583 | 40.01038361 |
| CD274 | CD274 Molecule | Protein Coding | 39 | GC09P005450 | 39.80286407 |
| CASP3 | Caspase 3 | Protein Coding | 44 | GC04M184627 | 39.78097534 |
| MAPK1 | Mitogen-Activated Protein Kinase 1 | Protein Coding | 46 | GC22M021759 | 39.73882294 |
| ABCB1 | ATP Binding Cassette Subfamily B Member 1 | Protein Coding | 46 | GC07M087504 | 39.67247009 |
| CXCR4 | C-X-C Motif Chemokine Receptor 4 | Protein Coding | 46 | GC02M136114 | 39.18053818 |
| PTPN11 | Protein Tyrosine Phosphatase Non-Receptor Type 11 | Protein Coding | 47 | GC12P112418 | 39.11302185 |
| IGF1R | Insulin Like Growth Factor 1 Receptor | Protein Coding | 48 | GC15P098648 | 39.00255966 |
| MIR122 | MicroRNA 122 | RNA Gene | 21 | GC18P058451 | 38.99209976 |
| MIR128-2 | MicroRNA 128-2 | RNA Gene | 20 | GC03P035750 | 38.92622375 |
| FAS | Fas Cell Surface Death Receptor | Protein Coding | 44 | GC10P091581 | 38.91827393 |
| TWIST1 | Twist Family BHLH Transcription Factor 1 | Protein Coding | 41 | GC07M019020 | 38.57237244 |
| XRCC1 | X-Ray Repair Cross Complementing 1 | Protein Coding | 39 | GC19M043543 | 38.51759338 |
| RNASEL | Ribonuclease L | Protein Coding | 40 | GC01M182573 | 38.36544037 |
| MKI67 | Marker Of Proliferation Ki-67 | Protein Coding | 39 | GC10M128096 | 38.27606201 |
| RBBP8 | RB Binding Protein 8, Endonuclease | Protein Coding | 39 | GC18P022798 | 38.23240662 |
| PBOV1 | Prostate And Breast Cancer Overexpressed 1 | Protein Coding | 25 | GC06M138215 | 38.23005676 |
| NOTCH1 | Notch Receptor 1 | Protein Coding | 46 | GC09M137427 | 38.18323898 |
| MIR98 | MicroRNA 98 | RNA Gene | 18 | GC0XM053631 | 38.16069412 |
| ERBB3 | Erb-B2 Receptor Tyrosine Kinase 3 | Protein Coding | 48 | GC12P057156 | 38.14984894 |
| CDKN3 | Cyclin Dependent Kinase Inhibitor 3 | Protein Coding | 39 | GC14P054398 | 37.98990631 |
| FHIT | Fragile Histidine Triad Diadenosine Triphosphatase | Protein Coding | 39 | GC03M059747 | 37.95461655 |
| MIR25 | MicroRNA 25 | RNA Gene | 19 | GC07M100093 | 37.88374329 |
| MIR148A | MicroRNA 148a | RNA Gene | 19 | GC07M025993 | 37.86405563 |
| GNAS | GNAS Complex Locus | Protein Coding | 46 | GC20P058839 | 37.83966827 |
| MIRLET7A3 | MicroRNA Let-7a-3 | RNA Gene | 19 | GC22P046112 | 37.80378342 |
| MIR23B | MicroRNA 23b | RNA Gene | 19 | GC09P095085 | 37.7926712 |
| PDGFRA | Platelet Derived Growth Factor Receptor Alpha | Protein Coding | 48 | GC04P054229 | 37.75664902 |
| TFF1 | Trefoil Factor 1 | Protein Coding | 38 | GC21M042362 | 37.7088089 |
| IDH1 | Isocitrate Dehydrogenase (NADP(+)) 1 | Protein Coding | 47 | GC02M208236 | 37.634758 |
| BIRC5 | Baculoviral IAP Repeat Containing 5 | Protein Coding | 41 | GC17P078214 | 37.55888367 |
| MIR183 | MicroRNA 183 | RNA Gene | 18 | GC07M129822 | 37.52091599 |
| JAK2 | Janus Kinase 2 | Protein Coding | 47 | GC09P004985 | 37.51981354 |
| VHL | Von Hippel-Lindau Tumor Suppressor | Protein Coding | 41 | GC03P012251 | 37.20381165 |
| HNF1B | HNF1 Homeobox B | Protein Coding | 38 | GC17M037686 | 37.17376328 |
| EZH2 | Enhancer Of Zeste 2 Polycomb Repressive Complex 2 Subunit | Protein Coding | 48 | GC07M148807 | 37.12316895 |
| SRD5A2 | Steroid 5 Alpha-Reductase 2 | Protein Coding | 39 | GC02M031522 | 37.06009293 |
| SOX9 | SRY-Box Transcription Factor 9 | Protein Coding | 41 | GC17P072121 | 37.0076828 |
| MIRLET7I | MicroRNA Let-7i | RNA Gene | 20 | GC12P062606 | 36.91242599 |
| MAP3K6 | Mitogen-Activated Protein Kinase Kinase Kinase 6 | Protein Coding | 38 | GC01M027354 | 36.88957214 |
| PARP1 | Poly(ADP-Ribose) Polymerase 1 | Protein Coding | 44 | GC01M226360 | 36.74614716 |
| ERCC1 | ERCC Excision Repair 1, Endonuclease Non-Catalytic Subunit | Protein Coding | 42 | GC19M063945 | 36.66645813 |
| SMAD7 | SMAD Family Member 7 | Protein Coding | 42 | GC18M048919 | 36.60212708 |
| MTHFR | Methylenetetrahydrofolate Reductase | Protein Coding | 43 | GC01M011785 | 36.54724884 |
| ROS1 | ROS Proto-Oncogene 1, Receptor Tyrosine Kinase | Protein Coding | 42 | GC06M117287 | 36.52880096 |
| MIR192 | MicroRNA 192 | RNA Gene | 21 | GC11M064891 | 36.36956787 |
| MIR16-1 | MicroRNA 16-1 | RNA Gene | 20 | GC13M050048 | 36.36207581 |
| CYP1A1 | Cytochrome P450 Family 1 Subfamily A Member 1 | Protein Coding | 43 | GC15M074719 | 36.30873871 |
| EGFR-AS1 | EGFR Antisense RNA 1 | RNA Gene | 15 | GC07M055179 | 36.27468109 |
| JUN | Jun Proto-Oncogene, AP-1 Transcription Factor Subunit | Protein Coding | 43 | GC01M058780 | 36.14318848 |
| MIR195 | MicroRNA 195 | RNA Gene | 19 | GC17M007018 | 36.03092194 |
| MIRLET7B | MicroRNA Let-7b | RNA Gene | 19 | GC22P046119 | 35.97008514 |
| PLAU | Plasminogen Activator, Urokinase | Protein Coding | 47 | GC10P073909 | 35.89250183 |
| CD44 | CD44 Molecule (Indian Blood Group) | Protein Coding | 42 | GC11P035139 | 35.88093185 |
| DROSHA | Drosha Ribonuclease III | Protein Coding | 38 | GC05M031401 | 35.84576797 |
| TP73 | Tumor Protein P73 | Protein Coding | 42 | GC01P003652 | 35.83202744 |
| KLK3 | Kallikrein Related Peptidase 3 | Protein Coding | 42 | GC19P050854 | 35.5982666 |
| MIR140 | MicroRNA 140 | RNA Gene | 22 | GC16P069934 | 35.5513382 |
| TOP2A | DNA Topoisomerase II Alpha | Protein Coding | 46 | GC17M040388 | 35.52558517 |
| MIR185 | MicroRNA 185 | RNA Gene | 21 | GC22P034421 | 35.31600952 |
| TNFRSF10B | TNF Receptor Superfamily Member 10b | Protein Coding | 44 | GC08M023020 | 35.3078537 |
| IL10 | Interleukin 10 | Protein Coding | 42 | GC01M206767 | 35.23885345 |
| NCOA3 | Nuclear Receptor Coactivator 3 | Protein Coding | 42 | GC20P047501 | 35.17263031 |
| H19 | H19 Imprinted Maternally Expressed Transcript | RNA Gene | 27 | GC11M001995 | 35.15089417 |
| PPP2R1B | Protein Phosphatase 2 Scaffold Subunit Abeta | Protein Coding | 42 | GC11M111695 | 35.08364868 |
| IGFBP3 | Insulin Like Growth Factor Binding Protein 3 | Protein Coding | 41 | GC07M045912 | 35.0742836 |
| CXCL8 | C-X-C Motif Chemokine Ligand 8 | Protein Coding | 35 | GC04P073740 | 34.92607117 |
| TYMS | Thymidylate Synthetase | Protein Coding | 43 | GC18P000657 | 34.89962006 |
| UCA1 | Urothelial Cancer Associated 1 | RNA Gene | 22 | GC19P015828 | 34.89933014 |
| CTSD | Cathepsin D | Protein Coding | 47 | GC11M001752 | 34.87359619 |
| SMARCB1 | SWI/SNF Related, Matrix Associated, Actin Dependent Regulator Of Chromatin, Subfamily B, Member 1 | Protein Coding | 40 | GC22P023786 | 34.77357483 |
| ELAC2 | ElaC Ribonuclease Z 2 | Protein Coding | 38 | GC17M012991 | 34.63188553 |
| CASP10 | Caspase 10 | Protein Coding | 43 | GC02P201182 | 34.49826813 |
| MIR107 | MicroRNA 107 | RNA Gene | 19 | GC10M089600 | 34.46872711 |
| MIR483 | MicroRNA 483 | RNA Gene | 18 | GC11M003033 | 34.37498474 |
| NFKB1 | Nuclear Factor Kappa B Subunit 1 | Protein Coding | 46 | GC04P102501 | 34.20308304 |
| BCAR1 | BCAR1 Scaffold Protein, Cas Family Member | Protein Coding | 39 | GC16M075228 | 34.08821106 |
| CDK1 | Cyclin Dependent Kinase 1 | Protein Coding | 40 | GC10P060772 | 34.02873611 |

| | | | | | |
|---|---|---|---|---|---|
| KDR | Kinase Insert Domain Receptor | Protein Coding | 48 | GC04M055078 | 34.01868439 |
| MIR486-1 | MicroRNA 486-1 | RNA Gene | 17 | GC08M041660 | 34.01860046 |
| DLC1 | DLC1 Rho GTPase Activating Protein | Protein Coding | 40 | GC08M013083 | 33.92012787 |
| KRT19 | Keratin 19 | Protein Coding | 40 | GC17M041523 | 33.87438202 |
| BCL2L1 | BCL2 Like 1 | Protein Coding | 42 | GC20M031664 | 33.86715317 |
| MIRLET7A1 | MicroRNA Let-7a-1 | RNA Gene | 19 | GC09P094175 | 33.81129456 |
| RAF1 | Raf-1 Proto-Oncogene, Serine/Threonine Kinase | Protein Coding | 48 | GC03M012583 | 33.74996948 |
| CTLA4 | Cytotoxic T-Lymphocyte Associated Protein 4 | Protein Coding | 43 | GC02P203867 | 33.71294022 |
| SMAD3 | SMAD Family Member 3 | Protein Coding | 45 | GC15P067063 | 33.622509 |
| AOPEP | Aminopeptidase O (Putative) | Protein Coding | 32 | GC09P094728 | 33.6046257 |
| RIPK1 | Receptor Interacting Serine/Threonine Kinase 1 | Protein Coding | 43 | GC06P003227 | 33.57828522 |
| BCAR3 | BCAR3 Adaptor Protein, NSP Family Member | Protein Coding | 35 | GC01M093561 | 33.53579712 |
| SOD2 | Superoxide Dismutase 2 | Protein Coding | 45 | GC06M159669 | 33.52492523 |
| TGFA | Transforming Growth Factor Alpha | Protein Coding | 41 | GC02M070447 | 33.51522827 |
| IGF2R | Insulin Like Growth Factor 2 Receptor | Protein Coding | 43 | GC06P159969 | 33.49101639 |
| CYP17A1 | Cytochrome P450 Family 17 Subfamily A Member 1 | Protein Coding | 44 | GC10M102830 | 33.47998428 |
| TGFBR1 | Transforming Growth Factor Beta Receptor 1 | Protein Coding | 47 | GC09P099104 | 33.28454971 |
| MIR181A1 | MicroRNA 181a-1 | RNA Gene | 18 | GC01M198860 | 33.22990036 |
| MT-CYB | Mitochondrially Encoded Cytochrome B | Protein Coding | 28 | GCMTP014749 | 33.11027527 |
| MSMB | Microseminoprotein Beta | Protein Coding | 37 | GC10M046033 | 33.02718735 |
| SDHC | Succinate Dehydrogenase Complex Subunit C | Protein Coding | 39 | GC01P161314 | 32.94676208 |
| POLK | DNA Polymerase Kappa | Protein Coding | 38 | GC05P075511 | 32.9258728 |
| AXIN1 | Axin 1 | Protein Coding | 42 | GC16M000287 | 32.91665649 |
| POT1 | Protection Of Telomeres 1 | Protein Coding | 38 | GC07M124822 | 32.88488007 |
| FGF2 | Fibroblast Growth Factor 2 | Protein Coding | 41 | GC04P122826 | 32.82780075 |
| MIR130A | MicroRNA 130a | RNA Gene | 20 | GC11P057641 | 32.82397842 |
| RABL3 | RAB, Member Of RAS Oncogene Family Like 3 | Protein Coding | 31 | GC03M120686 | 32.8081131 |
| MIR22 | MicroRNA 22 | RNA Gene | 20 | GC17M001713 | 32.68249512 |
| OVCAS1 | Ovarian Cancer, Epithelial, Susceptibility To | Genetic Locus | 2 | GC03U900587 | 32.58191299 |
| PRKAR1A | Protein Kinase CAMP-Dependent Type I Regulatory Subunit Alpha | Protein Coding | 46 | GC17P069011 | 32.55380249 |
| MIR100 | MicroRNA 100 | RNA Gene | 20 | GC11M122152 | 32.47828674 |
| MIR215 | MicroRNA 215 | RNA Gene | 19 | GC01M220117 | 32.42900467 |
| PDGFRB | Platelet Derived Growth Factor Receptor Beta | Protein Coding | 48 | GC05M150113 | 32.42289352 |
| CASP9 | Caspase 9 | Protein Coding | 42 | GC01M015491 | 32.38999939 |
| MMP1 | Matrix Metallopeptidase 1 | Protein Coding | 46 | GC11M102810 | 32.21751404 |
| BUB1 | BUB1 Mitotic Checkpoint Serine/Threonine Kinase | Protein Coding | 43 | GC02M110637 | 32.19268036 |
| RASSF1 | Ras Association Domain Family Member 1 | Protein Coding | 39 | GC03M050329 | 32.18911362 |
| MIR150 | MicroRNA 150 | RNA Gene | 21 | GC19M049500 | 32.17944717 |
| CEACAM5 | CEA Cell Adhesion Molecule 5 | Protein Coding | 39 | GC19P063709 | 32.15687561 |
| CDK2 | Cyclin Dependent Kinase 2 | Protein Coding | 46 | GC12P055966 | 32.09918594 |
| KRT7 | Keratin 7 | Protein Coding | 38 | GC12P052232 | 32.09382248 |
| OGG1 | 8-Oxoguanine DNA Glycosylase | Protein Coding | 42 | GC03P012204 | 32.0537262 |
| CDK6 | Cyclin Dependent Kinase 6 | Protein Coding | 47 | GC07M092604 | 32.04198456 |
| MGMT | O-6-Methylguanine-DNA Methyltransferase | Protein Coding | 46 | GC10P129467 | 31.91819382 |
| VEGFC | Vascular Endothelial Growth Factor C | Protein Coding | 42 | GC04M176683 | 31.87919807 |
| ERBB4 | Erb-B2 Receptor Tyrosine Kinase 4 | Protein Coding | 48 | GC02M211375 | 31.85335541 |
| SP1 | Sp1 Transcription Factor | Protein Coding | 40 | GC12P053380 | 31.84454727 |
| CAV1 | Caveolin 1 | Protein Coding | 43 | GC07P116524 | 31.8378067 |
| IL1RN | Interleukin 1 Receptor Antagonist | Protein Coding | 43 | GC02P121641 | 31.8354454 |
| CYP1B1 | Cytochrome P450 Family 1 Subfamily B Member 1 | Protein Coding | 43 | GC02M038066 | 31.82231331 |
| ARID1A | AT-Rich Interaction Domain 1A | Protein Coding | 39 | GC01P026693 | 31.76452637 |
| ABCC1 | ATP Binding Cassette Subfamily C Member 1 | Protein Coding | 44 | GC16P015949 | 31.75717926 |
| MMP14 | Matrix Metallopeptidase 14 | Protein Coding | 46 | GC14P031737 | 31.72519493 |
| MAD1L1 | Mitotic Arrest Deficient 1 Like 1 | Protein Coding | 41 | GC07M001815 | 31.70090485 |
| ING1 | Inhibitor Of Growth Family Member 1 | Protein Coding | 38 | GC13P110712 | 31.69920349 |
| RHOA | Ras Homolog Family Member A | Protein Coding | 42 | GC03M049359 | 31.62714767 |
| RELA | RELA Proto-Oncogene, NF-KB Subunit | Protein Coding | 46 | GC11M065653 | 31.62259674 |
| TNFSF10 | TNF Superfamily Member 10 | Protein Coding | 39 | GC03M172505 | 31.6050663 |
| MIR137 | MicroRNA 137 | RNA Gene | 20 | GC01M098046 | 31.59239769 |
| DNMT1 | DNA Methyltransferase 1 | Protein Coding | 46 | GC19M010133 | 31.4969368 |
| E2F1 | E2F Transcription Factor 1 | Protein Coding | 39 | GC20M033675 | 31.45635605 |
| MIR342 | MicroRNA 342 | RNA Gene | 19 | GC14P100109 | 31.41335678 |
| CCAT2 | Colon Cancer Associated Transcript 2 | RNA Gene | 14 | GC08P127400 | 31.36375618 |
| FZD3 | Frizzled Class Receptor 3 | Protein Coding | 39 | GC08P028494 | 31.34211349 |
| MIR133B | MicroRNA 133b | RNA Gene | 21 | GC06P052148 | 31.31232834 |
| SNAI2 | Snail Family Transcriptional Repressor 2 | Protein Coding | 41 | GC08M048917 | 31.27379417 |
| BCL10 | BCL10 Immune Signaling Adaptor | Protein Coding | 39 | GC01M085265 | 31.25199509 |
| WRAP53 | WD Repeat Containing Antisense To TP53 | Protein Coding | 37 | GC17P010890 | 31.23377609 |
| AKT2 | AKT Serine/Threonine Kinase 2 | Protein Coding | 48 | GC19M040230 | 31.23197937 |
| DDB2 | Damage Specific DNA Binding Protein 2 | Protein Coding | 41 | GC11P047237 | 31.18631554 |
| CXCL12 | C-X-C Motif Chemokine Ligand 12 | Protein Coding | 39 | GC10M044294 | 31.18107033 |
| FANCE | FA Complementation Group E | Protein Coding | 37 | GC06P080513 | 31.10591888 |
| PTK2 | Protein Tyrosine Kinase 2 | Protein Coding | 42 | GC08M140657 | 31.09601593 |
| LEP | Leptin | Protein Coding | 42 | GC07P128241 | 31.01536942 |
| IFNG | Interferon Gamma | Protein Coding | 43 | GC12M068154 | 31.00134277 |
| ABL1 | ABL Proto-Oncogene 1, Non-Receptor Tyrosine Kinase | Protein Coding | 47 | GC09P130713 | 30.93636322 |
| GREB1 | Growth Regulating Estrogen Receptor Binding 1 | Protein Coding | 32 | GC02P011482 | 30.93161774 |
| MIR10A | MicroRNA 10a | RNA Gene | 21 | GC17M048579 | 30.88305092 |
| ERCC4 | ERCC Excision Repair 4, Endonuclease Catalytic Subunit | Protein Coding | 41 | GC16P013920 | 30.83530998 |
| FN1 | Fibronectin 1 | Protein Coding | 44 | GC02M215360 | 30.83052063 |
| MIRLET7E | MicroRNA Let-7e | RNA Gene | 21 | GC19P064197 | 30.82695389 |
| PCNA | Proliferating Cell Nuclear Antigen | Protein Coding | 45 | GC20M005114 | 30.81447983 |
| SPP1 | Secreted Phosphoprotein 1 | Protein Coding | 41 | GC04P087975 | 30.72299767 |
| NAT2 | N-Acetyltransferase 2 | Protein Coding | 38 | GC08P018391 | 30.70347404 |
| HOTAIR | HOX Transcript Antisense RNA | RNA Gene | 24 | GC12M053962 | 30.6686039 |
| SNCG | Synuclein Gamma | Protein Coding | 36 | GC10P091496 | 30.65664291 |
| CSF3 | Colony Stimulating Factor 3 | Protein Coding | 38 | GC17P040015 | 30.60936737 |
| MAPK3 | Mitogen-Activated Protein Kinase 3 | Protein Coding | 43 | GC16M036472 | 30.60839272 |
| PDCD1 | Programmed Cell Death 1 | Protein Coding | 42 | GC02M241849 | 30.51808167 |
| IL2 | Interleukin 2 | Protein Coding | 42 | GC04M122451 | 30.46707726 |
| CHEK1 | Checkpoint Kinase 1 | Protein Coding | 44 | GC11P125625 | 30.41320419 |
| CCAT1 | Colon Cancer Associated Transcript 1 | RNA Gene | 14 | GC08M127207 | 30.4025116 |
| ACTB | Actin Beta | Protein Coding | 44 | GC07M005527 | 30.38458633 |
| FLT1 | Fms Related Receptor Tyrosine Kinase 1 | Protein Coding | 46 | GC13M028300 | 30.38428497 |
| STAT1 | Signal Transducer And Activator Of Transcription 1 | Protein Coding | 47 | GC02M190908 | 30.35177422 |

| | | | | | |
|---|---|---|---|---|---|
| VDR | Vitamin D Receptor | Protein Coding | 46 | GC12M047841 | 30.32495689 |
| MAPK8 | Mitogen-Activated Protein Kinase 8 | Protein Coding | 44 | GC10P048306 | 30.21482468 |
| MCC | MCC Regulator Of WNT Signaling Pathway | Protein Coding | 36 | GC05M113022 | 30.19978142 |
| SDHA | Succinate Dehydrogenase Complex Flavoprotein Subunit A | Protein Coding | 42 | GC05P000208 | 30.15283203 |
| CYP2A6 | Cytochrome P450 Family 2 Subfamily A Member 6 | Protein Coding | 43 | GC19M040843 | 30.1366272 |
| MIR15B | MicroRNA 15b | RNA Gene | 18 | GC03P160404 | 30.11198425 |
| HGF | Hepatocyte Growth Factor | Protein Coding | 47 | GC07M081699 | 30.0896225 |
| BRMS1 | BRMS1 Transcriptional Repressor And Anoikis Regulator | Protein Coding | 34 | GC11M087325 | 30.06300163 |
| GLI1 | GLI Family Zinc Finger 1 | Protein Coding | 42 | GC12P057460 | 30.03839684 |
| MALAT1 | Metastasis Associated Lung Adenocarcinoma Transcript 1 | RNA Gene | 23 | GC11P069543 | 30.0164814 |
| NQO1 | NAD(P)H Quinone Dehydrogenase 1 | Protein Coding | 43 | GC16M069706 | 29.99095917 |
| MIR191 | MicroRNA 191 | RNA Gene | 22 | GC03M051014 | 29.98549652 |
| KRT5 | Keratin 5 | Protein Coding | 42 | GC12M052514 | 29.94244003 |
| CCNA2 | Cyclin A2 | Protein Coding | 40 | GC04M121816 | 29.80787659 |
| PLAUR | Plasminogen Activator, Urokinase Receptor | Protein Coding | 39 | GC19M043646 | 29.6759243 |
| XIAP | X-Linked Inhibitor Of Apoptosis | Protein Coding | 43 | GC0XP123859 | 29.62684631 |
| CASC2 | Cancer Susceptibility 2 | RNA Gene | 23 | GC10P118046 | 29.61973953 |
| NTHL1 | Nth Like DNA Glycosylase 1 | Protein Coding | 40 | GC16M006688 | 29.60526276 |
| NRG1 | Neuregulin 1 | Protein Coding | 42 | GC08P031639 | 29.51873589 |
| TYMP | Thymidine Phosphorylase | Protein Coding | 42 | GC22M050525 | 29.5028038 |
| WT1 | WT1 Transcription Factor | Protein Coding | 44 | GC11M032365 | 29.49803925 |
| OPCML | Opioid Binding Protein/Cell Adhesion Molecule Like | Protein Coding | 39 | GC11M132405 | 29.42129898 |
| KRT18 | Keratin 18 | Protein Coding | 43 | GC12P052948 | 29.35758781 |
| TLR2 | Toll Like Receptor 2 | Protein Coding | 46 | GC04P153684 | 29.32722282 |
| CCNB1 | Cyclin B1 | Protein Coding | 41 | GC05P069167 | 29.29060745 |
| KRT8 | Keratin 8 | Protein Coding | 42 | GC12M052897 | 29.25564384 |
| CYCS | Cytochrome C, Somatic | Protein Coding | 43 | GC07M025118 | 29.24186325 |
| CTNNA2 | Catenin Alpha 2 | Protein Coding | 38 | GC02P079185 | 29.23801994 |
| SUFU | SUFU Negative Regulator Of Hedgehog Signaling | Protein Coding | 38 | GC10P102503 | 29.20854568 |
| SNAI1 | Snail Family Transcriptional Repressor 1 | Protein Coding | 41 | GC20P049982 | 29.14424133 |
| MIR372 | MicroRNA 372 | RNA Gene | 17 | GC19P064309 | 29.12555504 |
| CDKN2B | Cyclin Dependent Kinase Inhibitor 2B | Protein Coding | 42 | GC09M022002 | 29.10299301 |
| FLT4 | Fms Related Receptor Tyrosine Kinase 4 | Protein Coding | 46 | GC05M180607 | 29.09859467 |
| PTHLH | Parathyroid Hormone Like Hormone | Protein Coding | 42 | GC12M027959 | 29.09220314 |
| ZEB1 | Zinc Finger E-Box Binding Homeobox 1 | Protein Coding | 43 | GC10P031318 | 29.07288742 |
| CCNE1 | Cyclin E1 | Protein Coding | 43 | GC19P029811 | 29.0675087 |
| NF2 | NF2, Moesin-Ezrin-Radixin Like (MERLIN) Tumor Suppressor | Protein Coding | 43 | GC22P029603 | 29.05142593 |
| GPER1 | G Protein-Coupled Estrogen Receptor 1 | Protein Coding | 36 | GC07P001966 | 29.03760147 |
| FANCD2 | FA Complementation Group D2 | Protein Coding | 42 | GC03P010026 | 29.02500916 |
| HSP90AA1 | Heat Shock Protein 90 Alpha Family Class A Member 1 | Protein Coding | 43 | GC14M102080 | 28.99308777 |
| MEG3 | Maternally Expressed 3 | RNA Gene | 26 | GC14P109506 | 28.76644135 |
| CCND2 | Cyclin D2 | Protein Coding | 43 | GC12P019785 | 28.75583649 |
| BMP6 | Bone Morphogenetic Protein 6 | Protein Coding | 39 | GC06P007726 | 28.75172806 |
| SMAD2 | SMAD Family Member 2 | Protein Coding | 44 | GC18M047809 | 28.75091171 |
| MIR331 | MicroRNA 331 | RNA Gene | 18 | GC12P095308 | 28.74568748 |
| DPYD | Dihydropyrimidine Dehydrogenase | Protein Coding | 47 | GC01M097015 | 28.74181557 |
| NTRK1 | Neurotrophic Receptor Tyrosine Kinase 1 | Protein Coding | 46 | GC01P156815 | 28.6730423 |
| LCP1 | Lymphocyte Cytosolic Protein 1 | Protein Coding | 39 | GC13M046132 | 28.67034912 |
| GALNT12 | Polypeptide N-Acetylgalactosaminyltransferase 12 | Protein Coding | 36 | GC09P098807 | 28.6023922 |
| ITGB1 | Integrin Subunit Beta 1 | Protein Coding | 45 | GC10M032993 | 28.56315994 |
| NKX2-1 | NK2 Homeobox 1 | Protein Coding | 41 | GC14M036516 | 28.53691101 |
| HSPB1 | Heat Shock Protein Family B (Small) Member 1 | Protein Coding | 46 | GC07P076302 | 28.52564621 |
| MIR30D | MicroRNA 30d | RNA Gene | 18 | GC08M134804 | 28.51168823 |
| SOX2 | SRY-Box Transcription Factor 2 | Protein Coding | 40 | GC03P181711 | 28.50999451 |
| MIR181A2 | MicroRNA 181a-2 | RNA Gene | 19 | GC09P124692 | 28.38028717 |
| BCAS1 | Brain Enriched Myelin Associated Protein 1 | Protein Coding | 34 | GC20M053936 | 28.33684158 |
| FGF8 | Fibroblast Growth Factor 8 | Protein Coding | 42 | GC10M101770 | 28.33544159 |
| FOS | Fos Proto-Oncogene, AP-1 Transcription Factor Subunit | Protein Coding | 46 | GC14P075278 | 28.29618835 |
| HDAC1 | Histone Deacetylase 1 | Protein Coding | 44 | GC01P032292 | 28.25901413 |
| MMP7 | Matrix Metallopeptidase 7 | Protein Coding | 43 | GC11M102425 | 28.24265671 |
| MXI1 | MAX Interactor 1, Dimerization Protein | Protein Coding | 37 | GC10P110208 | 28.22741318 |
| PRKCA | Protein Kinase C Alpha | Protein Coding | 45 | GC17P066302 | 28.21786118 |
| GATA3 | GATA Binding Protein 3 | Protein Coding | 44 | GC10P008045 | 28.18485641 |
| MCL1 | MCL1 Apoptosis Regulator, BCL2 Family Member | Protein Coding | 42 | GC01M151623 | 28.13262939 |
| MIR106A | MicroRNA 106a | RNA Gene | 18 | GC0XM134304 | 28.11656189 |
| BCAS4 | Breast Carcinoma Amplified Sequence 4 | Protein Coding | 31 | GC20P050794 | 28.11225128 |
| MIR197 | MicroRNA 197 | RNA Gene | 20 | GC01P109549 | 28.08683586 |
| PLK1 | Polo Like Kinase 1 | Protein Coding | 44 | GC16P024198 | 28.04352188 |
| MAP3K8 | Mitogen-Activated Protein Kinase Kinase Kinase 8 | Protein Coding | 40 | GC10P030500 | 28.00346565 |
| INS | Insulin | Protein Coding | 42 | GC11M002159 | 28.00036049 |
| PVT1 | Pvt1 Oncogene | RNA Gene | 25 | GC08P127853 | 27.96901512 |
| COMT | Catechol-O-Methyltransferase | Protein Coding | 46 | GC22P019941 | 27.96066475 |
| RAC1 | Rac Family Small GTPase 1 | Protein Coding | 44 | GC07P006377 | 27.94117737 |
| HFE | Homeostatic Iron Regulator | Protein Coding | 40 | GC06P026087 | 27.92778587 |
| MAPK14 | Mitogen-Activated Protein Kinase 14 | Protein Coding | 46 | GC06P080526 | 27.90375328 |
| AREG | Amphiregulin | Protein Coding | 38 | GC04P074445 | 27.88951492 |
| KRT20 | Keratin 20 | Protein Coding | 36 | GC17M040875 | 27.80789566 |
| NME1 | NME/NM23 Nucleoside Diphosphate Kinase 1 | Protein Coding | 41 | GC17P053581 | 27.73793983 |
| GSK3B | Glycogen Synthase Kinase 3 Beta | Protein Coding | 44 | GC03M119821 | 27.70258331 |
| AKT3 | AKT Serine/Threonine Kinase 3 | Protein Coding | 48 | GC01M243488 | 27.69615555 |
| FGFR4 | Fibroblast Growth Factor Receptor 4 | Protein Coding | 46 | GC05P177086 | 27.66818237 |
| TIMP1 | TIMP Metallopeptidase Inhibitor 1 | Protein Coding | 39 | GC0XP047583 | 27.65037155 |
| THBS1 | Thrombospondin 1 | Protein Coding | 40 | GC15P039581 | 27.64279938 |
| SLC2A1 | Solute Carrier Family 2 Member 1 | Protein Coding | 47 | GC01M042925 | 27.63510132 |
| CSF2 | Colony Stimulating Factor 2 | Protein Coding | 38 | GC05P132073 | 27.60110855 |
| VIM | Vimentin | Protein Coding | 45 | GC10P017227 | 27.56327438 |
| SEC23B | SEC23 Homolog B, COPII Coat Complex Component | Protein Coding | 38 | GC20P018507 | 27.53382874 |
| PRKACA | Protein Kinase CAMP-Activated Catalytic Subunit Alpha | Protein Coding | 46 | GC19M014453 | 27.53321838 |
| CCL2 | C-C Motif Chemokine Ligand 2 | Protein Coding | 43 | GC17P034255 | 27.512146 |
| IRS1 | Insulin Receptor Substrate 1 | Protein Coding | 42 | GC02M226731 | 27.47458267 |
| PIK3CG | Phosphatidylinositol-4,5-Bisphosphate 3-Kinase Catalytic Subunit Gamma | Protein Coding | 43 | GC07P106865 | 27.45630455 |
| RNF43 | Ring Finger Protein 43 | Protein Coding | 37 | GC17M058352 | 27.43715858 |
| RPS6KB1 | Ribosomal Protein S6 Kinase B1 | Protein Coding | 43 | GC17P059893 | 27.42234039 |
| RARB | Retinoic Acid Receptor Beta | Protein Coding | 44 | GC03P024689 | 27.34637451 |
| MIR26A1 | MicroRNA 26a-1 | RNA Gene | 19 | GC03P037969 | 27.29957962 |

| | | | | | |
|---|---|---|---|---|---|
| PRKCD | Protein Kinase C Delta | Protein Coding | 48 | GC03P053156 | 27.28242874 |
| FASN | Fatty Acid Synthase | Protein Coding | 44 | GC17M082078 | 27.2719059 |
| SERPINA3 | Serpin Family A Member 3 | Protein Coding | 39 | GC14P094612 | 27.23696899 |
| AIP | Aryl Hydrocarbon Receptor Interacting Protein | Protein Coding | 39 | GC11P067468 | 27.16529083 |
| GNRH1 | Gonadotropin Releasing Hormone 1 | Protein Coding | 38 | GC08M025419 | 27.16119957 |
| PRL | Prolactin | Protein Coding | 38 | GC06M022287 | 27.13305473 |
| CDH2 | Cadherin 2 | Protein Coding | 47 | GC18M027950 | 27.1148262 |
| SERPINB5 | Serpin Family B Member 5 | Protein Coding | 38 | GC18P063476 | 27.10267639 |
| DNMT3B | DNA Methyltransferase 3 Beta | Protein Coding | 46 | GC20P032762 | 27.07818031 |
| TIMP3 | TIMP Metallopeptidase Inhibitor 3 | Protein Coding | 40 | GC22P035094 | 27.06597519 |
| TCF7L2 | Transcription Factor 7 Like 2 | Protein Coding | 42 | GC10P112950 | 27.04433632 |
| MXRA5 | Matrix Remodeling Associated 5 | Protein Coding | 30 | GC0XM003308 | 27.03765678 |
| PLK2 | Polo Like Kinase 2 | Protein Coding | 39 | GC05M058453 | 27.03683472 |
| SERPINE1 | Serpin Family E Member 1 | Protein Coding | 44 | GC07P101127 | 27.0235405 |
| CDKN2B-AS1 | CDKN2B Antisense RNA 1 | RNA Gene | 21 | GC09P021994 | 26.96277428 |
| CYP2D6 | Cytochrome P450 Family 2 Subfamily D Member 6 | Protein Coding | 43 | GC22M042126 | 26.93946838 |
| TIMP2 | TIMP Metallopeptidase Inhibitor 2 | Protein Coding | 39 | GC17M078852 | 26.93515778 |
| FOXP3 | Forkhead Box P3 | Protein Coding | 42 | GC0XM049250 | 26.92794418 |
| MTUS1 | Microtubule Associated Scaffold Protein 1 | Protein Coding | 32 | GC08M017643 | 26.77080917 |
| CREB1 | CAMP Responsive Element Binding Protein 1 | Protein Coding | 44 | GC02P207529 | 26.76957703 |
| FOXO1 | Forkhead Box O1 | Protein Coding | 44 | GC13M040555 | 26.72414398 |
| TGFB2 | Transforming Growth Factor Beta 2 | Protein Coding | 46 | GC01P218345 | 26.69112587 |
| BMI1 | BMI1 Proto-Oncogene, Polycomb Ring Finger | Protein Coding | 38 | GC10P022326 | 26.65481567 |
| RAD54B | RAD54 Homolog B | Protein Coding | 37 | GC08M094371 | 26.62254715 |
| TOP1 | DNA Topoisomerase I | Protein Coding | 44 | GC20P041028 | 26.60899734 |
| ETV6 | ETS Variant Transcription Factor 6 | Protein Coding | 42 | GC12P011649 | 26.56079483 |
| GAS5 | Growth Arrest Specific 5 | RNA Gene | 23 | GC01M173947 | 26.54757118 |
| MIR181B1 | MicroRNA 181b-1 | RNA Gene | 19 | GC01M198858 | 26.50969315 |
| PDGFRL | Platelet Derived Growth Factor Receptor Like | Protein Coding | 39 | GC08P017576 | 26.49794579 |
| SKP2 | S-Phase Kinase Associated Protein 2 | Protein Coding | 39 | GC05P036151 | 26.48945618 |
| KLK10 | Kallikrein Related Peptidase 10 | Protein Coding | 36 | GC19M051012 | 26.45414734 |
| HERPUD1 | Homocysteine Inducible ER Protein With Ubiquitin Like Domain 1 | Protein Coding | 35 | GC16P056932 | 26.35380173 |
| PTPRJ | Protein Tyrosine Phosphatase Receptor Type J | Protein Coding | 42 | GC11P048002 | 26.35068703 |
| PLA2G2A | Phospholipase A2 Group IIA | Protein Coding | 42 | GC01M019975 | 26.34031296 |
| MIR142 | MicroRNA 142 | RNA Gene | 20 | GC17M058331 | 26.33836365 |
| MT-CO1 | Mitochondrially Encoded Cytochrome C Oxidase I | Protein Coding | 30 | GCMTP005906 | 26.3370533 |
| FOXA1 | Forkhead Box A1 | Protein Coding | 38 | GC14M037589 | 26.2552948 |
| NFE2L2 | NFE2 Like BZIP Transcription Factor 2 | Protein Coding | 45 | GC02M177227 | 26.2510376 |
| BCAS3 | BCAS3 Microtubule Associated Cell Migration Factor | Protein Coding | 34 | GC17P060678 | 26.24861908 |
| IFI27 | Interferon Alpha Inducible Protein 27 | Protein Coding | 35 | GC14P094104 | 26.22068596 |
| BAK1 | BCL2 Antagonist/Killer 1 | Protein Coding | 39 | GC06M033572 | 26.19687653 |
| HSPA5 | Heat Shock Protein Family A (Hsp70) Member 5 | Protein Coding | 42 | GC09M125234 | 26.15777969 |
| MIR30A | MicroRNA 30a | RNA Gene | 20 | GC06M071403 | 26.0380764 |
| CYP3A4 | Cytochrome P450 Family 3 Subfamily A Member 4 | Protein Coding | 45 | GC07M099759 | 25.925457 |
| PAK1 | P21 (RAC1) Activated Kinase 1 | Protein Coding | 43 | GC11M087708 | 25.91454315 |
| RARA | Retinoic Acid Receptor Alpha | Protein Coding | 44 | GC17P040309 | 25.89664841 |
| PDGFB | Platelet Derived Growth Factor Subunit B | Protein Coding | 44 | GC22M056581 | 25.86737061 |
| CCL5 | C-C Motif Chemokine Ligand 5 | Protein Coding | 38 | GC17M035871 | 25.86343384 |
| KRT14 | Keratin 14 | Protein Coding | 42 | GC17M041582 | 25.83489227 |
| BRMS1L | BRMS1 Like Transcriptional Repressor | Protein Coding | 31 | GC14P035844 | 25.81051254 |
| CTAG1B | Cancer/Testis Antigen 1B | Protein Coding | 28 | GC0XM154617 | 25.74474907 |
| INSR | Insulin Receptor | Protein Coding | 48 | GC19M007112 | 25.7070446 |
| MIR199A1 | MicroRNA 199a-1 | RNA Gene | 18 | GC19M010817 | 25.61145592 |
| SHBG | Sex Hormone Binding Globulin | Protein Coding | 37 | GC17P007613 | 25.59272003 |
| KISS1 | KiSS-1 Metastasis Suppressor | Protein Coding | 38 | GC01M204190 | 25.58344841 |
| POU5F1 | POU Class 5 Homeobox 1 | Protein Coding | 40 | GC06M063557 | 25.54187393 |
| MIR449A | MicroRNA 449a | RNA Gene | 19 | GC05M055171 | 25.51858902 |
| TNFRSF10A | TNF Receptor Superfamily Member 10a | Protein Coding | 40 | GC08M023190 | 25.51244926 |
| ALDH1A1 | Aldehyde Dehydrogenase 1 Family Member A1 | Protein Coding | 42 | GC09M072900 | 25.49980545 |
| PGBD3 | PiggyBac Transposable Element Derived 3 | Protein Coding | 23 | GC10M049517 | 25.49766731 |
| CD24 | CD24 Molecule | Protein Coding | 33 | GC06M106969 | 25.49732399 |
| FOXM1 | Forkhead Box M1 | Protein Coding | 39 | GC12M002857 | 25.44319916 |
| CCND3 | Cyclin D3 | Protein Coding | 42 | GC06M041934 | 25.43967438 |
| DHFR | Dihydrofolate Reductase | Protein Coding | 43 | GC05M080626 | 25.39957809 |
| MDM4 | MDM4 Regulator Of P53 | Protein Coding | 39 | GC01P204516 | 25.35268402 |
| MTA1 | Metastasis Associated 1 | Protein Coding | 38 | GC14P105419 | 25.33300209 |
| BCAS2 | BCAS2 Pre-MRNA Processing Factor | Protein Coding | 33 | GC01M114567 | 25.31023979 |
| NFKBIA | NFKB Inhibitor Alpha | Protein Coding | 45 | GC14M035401 | 25.31022263 |
| IDH2 | Isocitrate Dehydrogenase (NADP(+)) 2 | Protein Coding | 47 | GC15M090083 | 25.2878685 |
| DMD | Dystrophin | Protein Coding | 42 | GC0XM031097 | 25.27186012 |
| FOXO3 | Forkhead Box O3 | Protein Coding | 40 | GC06P108559 | 25.26641464 |
| SHC1 | SHC Adaptor Protein 1 | Protein Coding | 39 | GC01M154962 | 25.22486496 |
| MIR23A | MicroRNA 23a | RNA Gene | 19 | GC19M014440 | 25.18861198 |
| CTSB | Cathepsin B | Protein Coding | 47 | GC08M011842 | 25.17530632 |
| ETS1 | ETS Proto-Oncogene 1, Transcription Factor | Protein Coding | 42 | GC11M128458 | 25.17008018 |
| CREBBP | CREB Binding Protein | Protein Coding | 47 | GC16M006859 | 25.12180328 |
| IL4 | Interleukin 4 | Protein Coding | 40 | GC05P132673 | 25.10796547 |
| AGR3 | Anterior Gradient 3, Protein Disulphide Isomerase Family Member | Protein Coding | 33 | GC07M016854 | 25.05806923 |
| PIP | Prolactin Induced Protein | Protein Coding | 35 | GC07P143132 | 25.05438995 |
| AHR | Aryl Hydrocarbon Receptor | Protein Coding | 44 | GC07P016916 | 25.0350914 |
| GREM1 | Gremlin 1, DAN Family BMP Antagonist | Protein Coding | 38 | GC15P039592 | 25.0193119 |
| SOS1 | SOS Ras/Rac Guanine Nucleotide Exchange Factor 1 | Protein Coding | 43 | GC02M039021 | 24.9997406 |
| LGALS3 | Galectin 3 | Protein Coding | 40 | GC14P055124 | 24.95547104 |
| MITF | Melanocyte Inducing Transcription Factor | Protein Coding | 43 | GC03P069788 | 24.90100288 |
| MIR320A | MicroRNA 320a | RNA Gene | 20 | GC08M022265 | 24.89441681 |
| GJA1 | Gap Junction Protein Alpha 1 | Protein Coding | 45 | GC06P121436 | 24.82389069 |
| CA9 | Carbonic Anhydrase 9 | Protein Coding | 41 | GC09P035673 | 24.79990196 |
| VEGFD | Vascular Endothelial Growth Factor D | Protein Coding | 32 | GC0XM015345 | 24.79761124 |
| BMP4 | Bone Morphogenetic Protein 4 | Protein Coding | 44 | GC14M053949 | 24.76821899 |
| BMP2 | Bone Morphogenetic Protein 2 | Protein Coding | 43 | GC20P006767 | 24.71787071 |
| HSPA4 | Heat Shock Protein Family A (Hsp70) Member 4 | Protein Coding | 37 | GC05P133051 | 24.71755409 |
| CASP7 | Caspase 7 | Protein Coding | 44 | GC10P113679 | 24.67348099 |
| TSG101 | Tumor Susceptibility 101 | Protein Coding | 37 | GC11M018468 | 24.64730835 |
| CTNND1 | Catenin Delta 1 | Protein Coding | 41 | GC11P058110 | 24.63897705 |
| MIR152 | MicroRNA 152 | RNA Gene | 21 | GC17M048037 | 24.63198471 |

| | | | | | | |
|---|---|---|---|---|---|---|
| CLDN7 | Claudin 7 | Protein Coding | 36 | GC17M007259 | 24.62231827 |
| NEAT1 | Nuclear Paraspeckle Assembly Transcript 1 | RNA Gene | 23 | GC11P069538 | 24.62109375 |
| WRN | WRN RecQ Like Helicase | Protein Coding | 41 | GC08P031033 | 24.62023354 |
| H2AC18 | H2A Clustered Histone 18 | Protein Coding | 26 | GC01M151582 | 24.61821747 |
| NRP1 | Neuropilin 1 | Protein Coding | 42 | GC10M033177 | 24.60615158 |
| HSD17B1 | Hydroxysteroid 17-Beta Dehydrogenase 1 | Protein Coding | 38 | GC17P053168 | 24.5866394 |
| LEF1 | Lymphoid Enhancer Binding Factor 1 | Protein Coding | 43 | GC04M108047 | 24.56596375 |
| SYP | Synaptophysin | Protein Coding | 40 | GC0XM049187 | 24.55997467 |
| NOS2 | Nitric Oxide Synthase 2 | Protein Coding | 44 | GC17M027756 | 24.52831268 |
| ITGA6 | Integrin Subunit Alpha 6 | Protein Coding | 44 | GC02P172427 | 24.48069191 |
| CDH3 | Cadherin 3 | Protein Coding | 43 | GC16P068637 | 24.39672089 |
| TACC1 | Transforming Acidic Coiled-Coil Containing Protein 1 | Protein Coding | 36 | GC08P038728 | 24.39215851 |
| PTPN12 | Protein Tyrosine Phosphatase Non-Receptor Type 12 | Protein Coding | 43 | GC07P077537 | 24.36424828 |
| TNFSF11 | TNF Superfamily Member 11 | Protein Coding | 44 | GC13P042562 | 24.36182022 |
| CCR6 | C-C Motif Chemokine Receptor 6 | Protein Coding | 39 | GC06P167111 | 24.33859634 |
| E2F3 | E2F Transcription Factor 3 | Protein Coding | 37 | GC06P020402 | 24.33098221 |
| AXL | AXL Receptor Tyrosine Kinase | Protein Coding | 47 | GC19P041219 | 24.30341339 |
| SETD2 | SET Domain Containing 2, Histone Lysine Methyltransferase | Protein Coding | 42 | GC03M047033 | 24.29980087 |
| YBX1 | Y-Box Binding Protein 1 | Protein Coding | 34 | GC01P042682 | 24.27790451 |
| HDAC9 | Histone Deacetylase 9 | Protein Coding | 43 | GC07P018086 | 24.26727295 |
| MIR9-1 | MicroRNA 9-1 | RNA Gene | 19 | GC01M156420 | 24.24159622 |
| LZTS1 | Leucine Zipper Tumor Suppressor 1 | Protein Coding | 35 | GC08M020246 | 24.22284508 |
| BID | BH3 Interacting Domain Death Agonist | Protein Coding | 39 | GC22M017734 | 24.18830299 |
| GSTT1 | Glutathione S-Transferase Theta 1 | Protein Coding | 30 | GC22Mi00270 | 24.18209839 |
| MYB | MYB Proto-Oncogene, Transcription Factor | Protein Coding | 44 | GC06P135180 | 24.16569519 |
| ENG | Endoglin | Protein Coding | 43 | GC09M127815 | 24.13657379 |
| S100A4 | S100 Calcium Binding Protein A4 | Protein Coding | 40 | GC01M153543 | 24.11841202 |
| SPARC | Secreted Protein Acidic And Cysteine Rich | Protein Coding | 45 | GC05M151661 | 24.11801529 |
| DNMT3A | DNA Methyltransferase 3 Alpha | Protein Coding | 46 | GC02M025228 | 24.10664558 |
| RUNX1 | RUNX Family Transcription Factor 1 | Protein Coding | 43 | GC21M034787 | 24.07907486 |
| RAD51B | RAD51 Paralog B | Protein Coding | 33 | GC14P067819 | 24.06201744 |
| JAK1 | Janus Kinase 1 | Protein Coding | 48 | GC01M064833 | 24.04997826 |
| NOTCH3 | Notch Receptor 3 | Protein Coding | 45 | GC19M015159 | 24.01713562 |
| FGF1 | Fibroblast Growth Factor 1 | Protein Coding | 42 | GC05M142555 | 24.01569557 |
| TFAP2A | Transcription Factor AP-2 Alpha | Protein Coding | 42 | GC06M010393 | 23.98719025 |
| NTRK3 | Neurotrophic Receptor Tyrosine Kinase 3 | Protein Coding | 47 | GC15M087859 | 23.98606873 |
| MUC16 | Mucin 16, Cell Surface Associated | Protein Coding | 34 | GC19M008848 | 23.93789291 |
| RECQL | RecQ Like Helicase | Protein Coding | 38 | GC12M021468 | 23.92467117 |
| ANKRD30A | Ankyrin Repeat Domain 30A | Protein Coding | 32 | GC10P037134 | 23.89482498 |
| KLF5 | Kruppel Like Factor 5 | Protein Coding | 40 | GC13P073054 | 23.85230255 |
| STAT5A | Signal Transducer And Activator Of Transcription 5A | Protein Coding | 42 | GC17P042287 | 23.83333588 |
| XPC | XPC Complex Subunit, DNA Damage Recognition And Repair Factor | Protein Coding | 42 | GC03M020208 | 23.82931519 |
| BSG | Basigin (Ok Blood Group) | Protein Coding | 39 | GC19P000571 | 23.81731033 |
| HNF1A | HNF1 Homeobox A | Protein Coding | 41 | GC12P125851 | 23.78863525 |
| PTK6 | Protein Tyrosine Kinase 6 | Protein Coding | 43 | GC20M063528 | 23.76307678 |
| MIR196B | MicroRNA 196b | RNA Gene | 20 | GC07M027549 | 23.74722099 |
| GADD45A | Growth Arrest And DNA Damage Inducible Alpha | Protein Coding | 41 | GC01P067685 | 23.72103691 |
| BCL2L11 | BCL2 Like 11 | Protein Coding | 39 | GC02P111119 | 23.71827316 |
| ABCC2 | ATP Binding Cassette Subfamily C Member 2 | Protein Coding | 43 | GC10P099782 | 23.70552063 |
| MMP3 | Matrix Metallopeptidase 3 | Protein Coding | 45 | GC11M102835 | 23.67292595 |
| LOX | Lysyl Oxidase | Protein Coding | 42 | GC05M122063 | 23.67038155 |
| SLMAP | Sarcolemma Associated Protein | Protein Coding | 35 | GC03P057866 | 23.64458084 |
| EPHA2 | EPH Receptor A2 | Protein Coding | 47 | GC01M016124 | 23.5968895 |
| ICAM1 | Intercellular Adhesion Molecule 1 | Protein Coding | 45 | GC19P010388 | 23.5436058 |
| EZR | Ezrin | Protein Coding | 40 | GC06M158765 | 23.51797104 |
| KCNQ1OT1 | KCNQ1 Opposite Strand/Antisense Transcript 1 | RNA Gene | 26 | GC11M003051 | 23.48705673 |
| ITGB3 | Integrin Subunit Beta 3 | Protein Coding | 46 | GC17P053399 | 23.47439957 |
| DKK1 | Dickkopf WNT Signaling Pathway Inhibitor 1 | Protein Coding | 42 | GC10P052314 | 23.45531082 |
| MIR224 | MicroRNA 224 | RNA Gene | 17 | GC0XM151958 | 23.4430027 |
| WNT5A | Wnt Family Member 5A | Protein Coding | 44 | GC03M055465 | 23.43409729 |
| ST14 | ST14 Transmembrane Serine Protease Matriptase | Protein Coding | 44 | GC11P130159 | 23.40706253 |
| TOX3 | TOX High Mobility Group Box Family Member 3 | Protein Coding | 34 | GC16M052447 | 23.38296509 |
| CLU | Clusterin | Protein Coding | 42 | GC08M027596 | 23.27770233 |
| MYCN | MYCN Proto-Oncogene, BHLH Transcription Factor | Protein Coding | 42 | GC02P015949 | 23.27653122 |
| GDF15 | Growth Differentiation Factor 15 | Protein Coding | 37 | GC19P063124 | 23.23722076 |
| XPA | XPA, DNA Damage Recognition And Repair Factor | Protein Coding | 41 | GC09M097654 | 23.16616631 |
| BAD | BCL2 Associated Agonist Of Cell Death | Protein Coding | 40 | GC11M087193 | 23.15785217 |
| IGFBP2 | Insulin Like Growth Factor Binding Protein 2 | Protein Coding | 39 | GC02P216632 | 23.14467621 |
| LEPR | Leptin Receptor | Protein Coding | 45 | GC01P065421 | 23.1301384 |
| SIRT1 | Sirtuin 1 | Protein Coding | 43 | GC10P067884 | 23.12195778 |
| HMGB1 | High Mobility Group Box 1 | Protein Coding | 40 | GC13M030456 | 23.07859421 |
| TERC | Telomerase RNA Component | RNA Gene | 28 | GC03M169765 | 23.05017471 |
| CTAG2 | Cancer/Testis Antigen 2 | Protein Coding | 31 | GC0XM154651 | 23.04781914 |
| APEX1 | Apurinic/Apyrimidinic Endodeoxyribonuclease 1 | Protein Coding | 39 | GC14P020455 | 23.04666138 |
| STAT5B | Signal Transducer And Activator Of Transcription 5B | Protein Coding | 44 | GC17M042199 | 23.04293633 |
| HDAC4 | Histone Deacetylase 4 | Protein Coding | 46 | GC02M239048 | 23.01915741 |
| ENO2 | Enolase 2 | Protein Coding | 42 | GC12P006913 | 23.01528358 |
| TH2LCRR | T Helper Type 2 Locus Control Region Associated RNA | RNA Gene | 13 | GC05M132631 | 23.01083183 |
| ETV4 | ETS Variant Transcription Factor 4 | Protein Coding | 38 | GC17M043527 | 22.95975494 |
| SRA1 | Steroid Receptor RNA Activator 1 | Protein Coding | 34 | GC05M140537 | 22.94770432 |
| BECN1 | Beclin 1 | Protein Coding | 40 | GC17M042810 | 22.93272209 |
| CDC25A | Cell Division Cycle 25A | Protein Coding | 42 | GC03M048173 | 22.91950226 |
| GAPDH | Glyceraldehyde-3-Phosphate Dehydrogenase | Protein Coding | 43 | GC12P019827 | 22.87062073 |
| KLF4 | Kruppel Like Factor 4 | Protein Coding | 40 | GC09M107484 | 22.86532593 |
| XIST | X Inactive Specific Transcript | RNA Gene | 24 | GC0XM073820 | 22.86347389 |
| STS | Steroid Sulfatase | Protein Coding | 42 | GC0XP007146 | 22.85566902 |
| GMNN | Geminin DNA Replication Inhibitor | Protein Coding | 40 | GC06P024779 | 22.82967377 |
| CSF1 | Colony Stimulating Factor 1 | Protein Coding | 39 | GC01P109911 | 22.82462692 |
| PKHD1 | PKHD1 Ciliary IPT Domain Containing Fibrocystin/Polyductin | Protein Coding | 34 | GC06M063956 | 22.81482506 |
| NOTCH2 | Notch Receptor 2 | Protein Coding | 45 | GC01M119911 | 22.80892563 |
| HLA-G | Major Histocompatibility Complex, Class I, G | Protein Coding | 40 | GC06P080301 | 22.80549431 |
| MMP11 | Matrix Metallopeptidase 11 | Protein Coding | 40 | GC22P023768 | 22.78707504 |
| ANXA2 | Annexin A2 | Protein Coding | 43 | GC15M060347 | 22.78664017 |
| CCNG1 | Cyclin G1 | Protein Coding | 36 | GC05P163438 | 22.7814064 |
| ITGAV | Integrin Subunit Alpha V | Protein Coding | 42 | GC02P186589 | 22.72253418 |

| | | | | | |
|---|---|---|---|---|---|
| ELAVL1 | ELAV Like RNA Binding Protein 1 | Protein Coding | 37 | GC19M007958 | 22.69292259 |
| XBP1 | X-Box Binding Protein 1 | Protein Coding | 40 | GC22M028794 | 22.64292908 |
| ECT2 | Epithelial Cell Transforming 2 | Protein Coding | 36 | GC03P172750 | 22.64261627 |
| PIK3R2 | Phosphoinositide-3-Kinase Regulatory Subunit 2 | Protein Coding | 44 | GC19P018153 | 22.62488556 |
| CDC42 | Cell Division Cycle 42 | Protein Coding | 46 | GC01P022292 | 22.61886215 |
| HBEGF | Heparin Binding EGF Like Growth Factor | Protein Coding | 38 | GC05M140332 | 22.60394669 |
| MIR375 | MicroRNA 375 | RNA Gene | 20 | GC02M219001 | 22.58062744 |
| WNT1 | Wnt Family Member 1 | Protein Coding | 40 | GC12P049266 | 22.56712914 |
| MTDH | Metadherin | Protein Coding | 35 | GC08P097643 | 22.55761719 |
| ANXA1 | Annexin A1 | Protein Coding | 43 | GC09P073151 | 22.54302979 |
| SMARCE1 | SWI/SNF Related, Matrix Associated, Actin Dependent Regulator Of Chromatin, Subfamily E, Member 1 | Protein Coding | 40 | GC17M040624 | 22.52911568 |
| CSF1R | Colony Stimulating Factor 1 Receptor | Protein Coding | 46 | GC05M150053 | 22.51791382 |
| IL17A | Interleukin 17A | Protein Coding | 39 | GC06P052186 | 22.50838089 |
| NCOR1 | Nuclear Receptor Corepressor 1 | Protein Coding | 39 | GC17M016029 | 22.49794197 |
| IGFBP1 | Insulin Like Growth Factor Binding Protein 1 | Protein Coding | 38 | GC07P047629 | 22.49330521 |
| PDPN | Podoplanin | Protein Coding | 36 | GC01P013583 | 22.49186325 |
| GPX1 | Glutathione Peroxidase 1 | Protein Coding | 42 | GC03M051039 | 22.48787689 |
| MUCL1 | Mucin Like 1 | Protein Coding | 29 | GC12P054830 | 22.46976471 |
| ERCC5 | ERCC Excision Repair 5, Endonuclease | Protein Coding | 40 | GC13P102845 | 22.42357635 |
| YAP1 | Yes1 Associated Transcriptional Regulator | Protein Coding | 42 | GC11P102110 | 22.41731644 |
| CBFB | Core-Binding Factor Subunit Beta | Protein Coding | 40 | GC16P067063 | 22.40891075 |
| LOC107303340 | 3p25 Von Hippel-Lindau Tumor Suppressor, E3 Ubiquitin Protein Ligase Alu-Mediated Recombination Region | Biological Region | 3 | GC03P012257 | 22.40472221 |
| FGF3 | Fibroblast Growth Factor 3 | Protein Coding | 40 | GC11M087469 | 22.38846397 |
| SOX4 | SRY-Box Transcription Factor 4 | Protein Coding | 39 | GC06P021593 | 22.38321877 |
| PRKD1 | Protein Kinase D1 | Protein Coding | 44 | GC14M029576 | 22.38010025 |
| FGF7 | Fibroblast Growth Factor 7 | Protein Coding | 37 | GC15P049423 | 22.37401962 |
| SF3B1 | Splicing Factor 3b Subunit 1 | Protein Coding | 40 | GC02M197401 | 22.35390472 |
| MAP2K2 | Mitogen-Activated Protein Kinase Kinase 2 | Protein Coding | 47 | GC19M004090 | 22.32479477 |
| FSCN1 | Fascin Actin-Bundling Protein 1 | Protein Coding | 38 | GC07P005592 | 22.31712723 |
| TUG1 | Taurine Up-Regulated 1 | Protein Coding | 21 | GC22P030969 | 22.30545807 |
| SPRY4-IT1 | SPRY4 Intronic Transcript 1 | RNA Gene | 15 | GC05M142318 | 22.29983521 |
| EREG | Epiregulin | Protein Coding | 38 | GC04P074366 | 22.29301071 |
| LINC-ROR | Long Intergenic Non-Protein Coding RNA, Regulator Of Reprogramming | RNA Gene | 15 | GC18M057054 | 22.26299095 |
| FAN1 | FANCD2 And FANCI Associated Nuclease 1 | Protein Coding | 33 | GC15P039471 | 22.25084114 |
| NAT1 | N-Acetyltransferase 1 | Protein Coding | 40 | GC08P018179 | 22.21837807 |
| EGR1 | Early Growth Response 1 | Protein Coding | 42 | GC05P138465 | 22.2094841 |
| MIR149 | MicroRNA 149 | RNA Gene | 21 | GC02P240456 | 22.20506668 |
| FGF17 | Fibroblast Growth Factor 17 | Protein Coding | 40 | GC08P022042 | 22.16321182 |
| KITLG | KIT Ligand | Protein Coding | 40 | GC12M088492 | 22.13928986 |
| LDHA | Lactate Dehydrogenase A | Protein Coding | 46 | GC11P018394 | 22.11331558 |
| TEK | TEK Receptor Tyrosine Kinase | Protein Coding | 46 | GC09P027109 | 22.10095978 |
| CCR7 | C-C Motif Chemokine Receptor 7 | Protein Coding | 39 | GC17M041604 | 22.0666523 |
| TUBB | Tubulin Beta Class I | Protein Coding | 44 | GC06P080331 | 22.02997208 |
| GRB2 | Growth Factor Receptor Bound Protein 2 | Protein Coding | 42 | GC17M075318 | 22.00289345 |
| PCAT1 | Prostate Cancer Associated Transcript 1 | RNA Gene | 17 | GC08P126553 | 21.99112701 |
| PML | PML Nuclear Body Scaffold | Protein Coding | 41 | GC15P073994 | 21.9660778 |
| AMER1 | APC Membrane Recruitment Protein 1 | Protein Coding | 32 | GC0XM064185 | 21.95251274 |
| ENO1 | Enolase 1 | Protein Coding | 42 | GC01M008861 | 21.91492081 |
| EPHB4 | EPH Receptor B4 | Protein Coding | 47 | GC07M101787 | 21.90023041 |
| PIK3CB | Phosphatidylinositol-4,5-Bisphosphate 3-Kinase Catalytic Subunit Beta | Protein Coding | 43 | GC03M138652 | 21.89281464 |
| EPSTI1 | Epithelial Stromal Interaction 1 | Protein Coding | 31 | GC13M042886 | 21.86988068 |
| SULT1A1 | Sulfotransferase Family 1A Member 1 | Protein Coding | 38 | GC16M036286 | 21.80263901 |
| PPP2R1A | Protein Phosphatase 2 Scaffold Subunit Aalpha | Protein Coding | 42 | GC19P064211 | 21.79536057 |
| NR1H2 | Nuclear Receptor Subfamily 1 Group H Member 2 | Protein Coding | 42 | GC19P050329 | 21.76020432 |
| WNT3 | Wnt Family Member 3 | Protein Coding | 42 | GC17M046762 | 21.74909592 |
| CYP1A2 | Cytochrome P450 Family 1 Subfamily A Member 2 | Protein Coding | 42 | GC15P074748 | 21.74905586 |
| CDC25C | Cell Division Cycle 25C | Protein Coding | 43 | GC05M138285 | 21.7339859 |
| CTTN | Cortactin | Protein Coding | 38 | GC11P070398 | 21.72363853 |
| MVP | Major Vault Protein | Protein Coding | 36 | GC16P040192 | 21.70938873 |
| BDNF | Brain Derived Neurotrophic Factor | Protein Coding | 43 | GC11M027654 | 21.70882225 |
| ARID4B | AT-Rich Interaction Domain 4B | Protein Coding | 32 | GC01M235133 | 21.70111084 |
| ACVR1B | Activin A Receptor Type 1B | Protein Coding | 42 | GC12P051951 | 21.69612312 |
| PXN | Paxillin | Protein Coding | 40 | GC12M120210 | 21.67106438 |
| LGALS1 | Galectin 1 | Protein Coding | 38 | GC22P037675 | 21.66576385 |
| CHUK | Component Of Inhibitor Of Nuclear Factor Kappa B Kinase Complex | Protein Coding | 46 | GC10M100188 | 21.66268539 |
| PTK2B | Protein Tyrosine Kinase 2 Beta | Protein Coding | 44 | GC08P027311 | 21.6385994 |
| MT-ND1 | Mitochondrially Encoded NADH:Ubiquinone Oxidoreductase Core Subunit 1 | Protein Coding | 28 | GCMTP003309 | 21.63749695 |
| XRCC6 | X-Ray Repair Cross Complementing 6 | Protein Coding | 40 | GC22P041622 | 21.6338768 |
| STMN1 | Stathmin 1 | Protein Coding | 39 | GC01M025884 | 21.58908081 |
| DRAIC | Downregulated RNA In Cancer, Inhibitor Of Cell Invasion And Migration | RNA Gene | 14 | GC15P114665 | 21.57039642 |
| XRCC5 | X-Ray Repair Cross Complementing 5 | Protein Coding | 39 | GC02P216107 | 21.51106262 |
| MAX | MYC Associated Factor X | Protein Coding | 41 | GC14M065087 | 21.46364594 |
| MAGED2 | MAGE Family Member D2 | Protein Coding | 36 | GC0XP054807 | 21.44956398 |
| LASP1 | LIM And SH3 Protein 1 | Protein Coding | 38 | GC17P038869 | 21.41111565 |
| MMP13 | Matrix Metallopeptidase 13 | Protein Coding | 46 | GC11M102942 | 21.40993881 |
| LOC111589215 | BRCA1 Promoter Region | Biological Region | 2 | GC17P053873 | 21.35683441 |
| ODC1 | Ornithine Decarboxylase 1 | Protein Coding | 43 | GC02M010432 | 21.34293938 |
| CLCA2 | Chloride Channel Accessory 2 | Protein Coding | 37 | GC01P086424 | 21.28835487 |
| IL2RA | Interleukin 2 Receptor Subunit Alpha | Protein Coding | 44 | GC10M006010 | 21.27949333 |
| EIF4E | Eukaryotic Translation Initiation Factor 4E | Protein Coding | 43 | GC04M098879 | 21.24403763 |
| SPDEF | SAM Pointed Domain Containing ETS Transcription Factor | Protein Coding | 34 | GC06M063744 | 21.23665428 |
| IL3 | Interleukin 3 | Protein Coding | 38 | GC05P132060 | 21.22320938 |
| CTCF | CCCTC-Binding Factor | Protein Coding | 41 | GC16P067563 | 21.1882782 |
| FGF4 | Fibroblast Growth Factor 4 | Protein Coding | 38 | GC11M087468 | 21.18525696 |
| DLEC1 | DLEC1 Cilia And Flagella Associated Protein | Protein Coding | 33 | GC03P038038 | 21.17919922 |
| MME | Membrane Metalloendopeptidase | Protein Coding | 45 | GC03P155024 | 21.13768005 |
| PLCG1 | Phospholipase C Gamma 1 | Protein Coding | 43 | GC20P041136 | 21.13362885 |
| FADD | Fas Associated Via Death Domain | Protein Coding | 41 | GC11P070203 | 21.1182251 |
| SCGB2A2 | Secretoglobin Family 2A Member 2 | Protein Coding | 34 | GC11P062269 | 21.08686256 |
| AURKB | Aurora Kinase B | Protein Coding | 44 | GC17M010281 | 21.07863998 |
| MIR24-2 | MicroRNA 24-2 | RNA Gene | 20 | GC19M014438 | 21.06535721 |
| NFKB2 | Nuclear Factor Kappa B Subunit 2 | Protein Coding | 46 | GC10P102394 | 21.05595207 |
| DKC1 | Dyskerin Pseudouridine Synthase 1 | Protein Coding | 41 | GC0XP154762 | 21.03688049 |

| | | | | | |
|---|---|---|---|---|---|
| NR3C1 | Nuclear Receptor Subfamily 3 Group C Member 1 | Protein Coding | 44 | GC05M143277 | 21.03507614 |
| RUNX2 | RUNX Family Transcription Factor 2 | Protein Coding | 42 | GC06P080616 | 21.0174675 |
| REST | RE1 Silencing Transcription Factor | Protein Coding | 39 | GC04P056907 | 20.95795059 |
| HMGA1 | High Mobility Group AT-Hook 1 | Protein Coding | 41 | GC06P080476 | 20.93239594 |
| WNT10B | Wnt Family Member 10B | Protein Coding | 43 | GC12M048965 | 20.93025589 |
| ID1 | Inhibitor Of DNA Binding 1, HLH Protein | Protein Coding | 37 | GC20P031605 | 20.92852974 |
| ATAD2 | ATPase Family AAA Domain Containing 2 | Protein Coding | 35 | GC08M123319 | 20.90577698 |
| E2F2 | E2F Transcription Factor 2 | Protein Coding | 39 | GC01M023643 | 20.90186691 |
| ABCC11 | ATP Binding Cassette Subfamily C Member 11 | Protein Coding | 39 | GC16M048166 | 20.90099716 |
| PIK3CD | Phosphatidylinositol-4,5-Bisphosphate 3-Kinase Catalytic Subunit Delta | Protein Coding | 48 | GC01P009629 | 20.87484741 |
| MYO1B | Myosin IB | Protein Coding | 34 | GC02P191246 | 20.84028625 |
| ITGA3 | Integrin Subunit Alpha 3 | Protein Coding | 43 | GC17P050055 | 20.81884003 |
| NCOA1 | Nuclear Receptor Coactivator 1 | Protein Coding | 40 | GC02P024492 | 20.81238556 |
| PTTG1 | PTTG1 Regulator Of Sister Chromatid Separation, Securin | Protein Coding | 37 | GC05P160422 | 20.78310013 |
| CSNK2A1 | Casein Kinase 2 Alpha 1 | Protein Coding | 46 | GC20M000472 | 20.77484703 |
| HDAC2 | Histone Deacetylase 2 | Protein Coding | 46 | GC06M113933 | 20.76770782 |
| CHGA | Chromogranin A | Protein Coding | 40 | GC14P092929 | 20.75844193 |
| BAG1 | BAG Cochaperone 1 | Protein Coding | 37 | GC09M033245 | 20.73601532 |
| RHOB | Ras Homolog Family Member B | Protein Coding | 39 | GC02P020447 | 20.72170258 |
| TCIM | Transcriptional And Immune Response Regulator | Protein Coding | 27 | GC08P040153 | 20.68027115 |
| AMPH | Amphiphysin | Protein Coding | 39 | GC07M039038 | 20.65888214 |
| PROM1 | Prominin 1 | Protein Coding | 41 | GC04M015965 | 20.6450119 |
| NPM1 | Nucleophosmin 1 | Protein Coding | 44 | GC05P171387 | 20.64380455 |
| SYK | Spleen Associated Tyrosine Kinase | Protein Coding | 45 | GC09P091929 | 20.64081383 |
| ERCC3 | ERCC Excision Repair 3, TFIIH Core Complex Helicase Subunit | Protein Coding | 43 | GC02M127257 | 20.59679794 |
| KDM5B | Lysine Demethylase 5B | Protein Coding | 39 | GC01M202696 | 20.57290268 |
| PSCA | Prostate Stem Cell Antigen | Protein Coding | 36 | GC08P142670 | 20.5410862 |
| MIR125B1 | MicroRNA 125b-1 | RNA Gene | 20 | GC11M122100 | 20.53294945 |
| NCOA6 | Nuclear Receptor Coactivator 6 | Protein Coding | 35 | GC20M034809 | 20.43571854 |
| LRP6 | LDL Receptor Related Protein 6 | Protein Coding | 43 | GC12M020470 | 20.43091965 |
| RELB | RELB Proto-Oncogene, NF-KB Subunit | Protein Coding | 40 | GC19P063849 | 20.40668297 |
| ZFAS1 | ZNFX1 Antisense RNA 1 | RNA Gene | 19 | GC20P049276 | 20.395401 |
| HOTTIP | HOXA Distal Transcript Antisense RNA | RNA Gene | 22 | GC07P027198 | 20.37471962 |
| ALB | Albumin | Protein Coding | 44 | GC04P073397 | 20.35723686 |
| EPHA3 | EPH Receptor A3 | Protein Coding | 41 | GC03P089077 | 20.33510208 |
| FOXC1 | Forkhead Box C1 | Protein Coding | 38 | GC06P001610 | 20.32966042 |
| TBX3 | T-Box Transcription Factor 3 | Protein Coding | 39 | GC12M114670 | 20.30895615 |
| CEBPB | CCAAT Enhancer Binding Protein Beta | Protein Coding | 39 | GC20P050190 | 20.30425453 |
| NCOR2 | Nuclear Receptor Corepressor 2 | Protein Coding | 39 | GC12M124324 | 20.30354309 |
| CD34 | CD34 Molecule | Protein Coding | 39 | GC01M207880 | 20.25569153 |
| TXN | Thioredoxin | Protein Coding | 40 | GC09M110243 | 20.25430679 |
| CCN2 | Cellular Communication Network Factor 2 | Protein Coding | 37 | GC06M131948 | 20.21788788 |
| MIR29B2 | MicroRNA 29b-2 | RNA Gene | 18 | GC01M207806 | 20.21170998 |
| JAG1 | Jagged Canonical Notch Ligand 1 | Protein Coding | 45 | GC20M010637 | 20.14658737 |
| CCN1 | Cellular Communication Network Factor 1 | Protein Coding | 30 | GC01P085581 | 20.13774109 |
| LCN2 | Lipocalin 2 | Protein Coding | 40 | GC09P128149 | 20.1201973 |
| ROBO1 | Roundabout Guidance Receptor 1 | Protein Coding | 40 | GC03M078597 | 20.10822487 |
| PTENP1 | Phosphatase And Tensin Homolog Pseudogene 1 | Pseudogene | 19 | GC09M033673 | 20.10479546 |
| TRIM24 | Tripartite Motif Containing 24 | Protein Coding | 38 | GC07P138460 | 20.09247971 |
| TH2-LCR | Th2 Cytokine Locus Control Region | Biological Region | 2 | GC05P132629 | 20.08927917 |
| GRN | Granulin Precursor | Protein Coding | 43 | GC17P044345 | 20.06860352 |
| RAD52 | RAD52 Homolog, DNA Repair Protein | Protein Coding | 37 | GC12M000912 | 20.06746674 |
| RRAS | RAS Related | Protein Coding | 38 | GC19M049635 | 20.06158257 |
| KIAA0100 | KIAA0100 | Protein Coding | 31 | GC17M028614 | 20.05493546 |
| BRCA3 | Breast Cancer 3 | Genetic Locus | 2 | GC13U990062 | 20.03203964 |
| CDK7 | Cyclin Dependent Kinase 7 | Protein Coding | 40 | GC05P069363 | 20.02819061 |
| ITGB4 | Integrin Subunit Beta 4 | Protein Coding | 44 | GC17P075721 | 20.02033615 |
| IRS2 | Insulin Receptor Substrate 2 | Protein Coding | 40 | GC13M109752 | 19.98576736 |
| PIN1 | Peptidylprolyl Cis/Trans Isomerase, NIMA-Interacting 1 | Protein Coding | 42 | GC19P009835 | 19.96759796 |
| TMEM71 | Transmembrane Protein 71 | Protein Coding | 28 | GC08M132685 | 19.96492577 |
| PRMT7 | Protein Arginine Methyltransferase 7 | Protein Coding | 40 | GC16P068476 | 19.92862701 |
| ARAF | A-Raf Proto-Oncogene, Serine/Threonine Kinase | Protein Coding | 40 | GC0XP047562 | 19.91977692 |
| FOXE1 | Forkhead Box E1 | Protein Coding | 38 | GC09P097853 | 19.90639877 |
| IGFBP5 | Insulin Like Growth Factor Binding Protein 5 | Protein Coding | 38 | GC02M216672 | 19.89786911 |
| TGFB3 | Transforming Growth Factor Beta 3 | Protein Coding | 44 | GC14M075958 | 19.89543152 |
| TIMELESS | Timeless Circadian Regulator | Protein Coding | 34 | GC12M056416 | 19.89212608 |
| RHOBTB2 | Rho Related BTB Domain Containing 2 | Protein Coding | 36 | GC08P022987 | 19.88140869 |
| PIK3R3 | Phosphoinositide-3-Kinase Regulatory Subunit 3 | Protein Coding | 39 | GC01M046041 | 19.87586594 |
| MIR193A | MicroRNA 193a | RNA Gene | 18 | GC17P031559 | 19.85993767 |
| ALCAM | Activated Leukocyte Cell Adhesion Molecule | Protein Coding | 39 | GC03P105366 | 19.85331345 |
| CYP24A1 | Cytochrome P450 Family 24 Subfamily A Member 1 | Protein Coding | 42 | GC20M054153 | 19.84703255 |
| RBFOX1 | RNA Binding Fox-1 Homolog 1 | Protein Coding | 34 | GC16P010919 | 19.82649422 |
| HMOX1 | Heme Oxygenase 1 | Protein Coding | 46 | GC22P035380 | 19.82488251 |
| SPHK1 | Sphingosine Kinase 1 | Protein Coding | 42 | GC17P076376 | 19.80980682 |
| HEATR6 | HEAT Repeat Containing 6 | Protein Coding | 28 | GC17M060041 | 19.79496574 |
| H2AX | H2A.X Variant Histone | Protein Coding | 34 | GC11M119175 | 19.73423386 |
| APOD | Apolipoprotein D | Protein Coding | 39 | GC03M195568 | 19.72924423 |
| MAPK10 | Mitogen-Activated Protein Kinase 10 | Protein Coding | 45 | GC04M085990 | 19.70976639 |
| KIF15 | Kinesin Family Member 15 | Protein Coding | 32 | GC03P046354 | 19.69162178 |
| CASC22 | Cancer Susceptibility 22 | RNA Gene | 14 | GC16P052258 | 19.68000412 |
| MIR16-2 | MicroRNA 16-2 | RNA Gene | 18 | GC03P160413 | 19.64684677 |
| PTPN3 | Protein Tyrosine Phosphatase Non-Receptor Type 3 | Protein Coding | 40 | GC09M109375 | 19.62616158 |
| CEMIP | Cell Migration Inducing Hyaluronidase 1 | Protein Coding | 31 | GC15P080779 | 19.61081696 |
| CUL1 | Cullin 1 | Protein Coding | 39 | GC07P148697 | 19.5867672 |
| PTPRF | Protein Tyrosine Phosphatase Receptor Type F | Protein Coding | 44 | GC01P043527 | 19.56941986 |
| ST18 | ST18 C2H2C-Type Zinc Finger Transcription Factor | Protein Coding | 34 | GC08M052110 | 19.56557655 |
| BCYRN1 | Brain Cytoplasmic RNA 1 | RNA Gene | 18 | GC02P047331 | 19.56534386 |
| IDO1 | Indoleamine 2,3-Dioxygenase 1 | Protein Coding | 40 | GC08P039891 | 19.50487137 |
| UHRF1 | Ubiquitin Like With PHD And Ring Finger Domains 1 | Protein Coding | 34 | GC19P004945 | 19.4912529 |
| DANCR | Differentiation Antagonizing Non-Protein Coding RNA | RNA Gene | 21 | GC04P052712 | 19.48783112 |
| CCAR2 | Cell Cycle And Apoptosis Regulator 2 | Protein Coding | 32 | GC08P022604 | 19.47841454 |
| CEP85L | Centrosomal Protein 85 Like | Protein Coding | 31 | GC06M118460 | 19.4678154 |
| RHOC | Ras Homolog Family Member C | Protein Coding | 36 | GC01M112701 | 19.45646286 |
| AKR1C3 | Aldo-Keto Reductase Family 1 Member C3 | Protein Coding | 42 | GC10P005035 | 19.45618439 |
| ROCK1 | Rho Associated Coiled-Coil Containing Protein Kinase 1 | Protein Coding | 44 | GC18M020946 | 19.4516983 |

| | | | | | |
|---|---|---|---|---|---|
| PELP1 | Proline, Glutamate And Leucine Rich Protein 1 | Protein Coding | 35 | GC17M004669 | 19.43302536 |
| DDR1 | Discoidin Domain Receptor Tyrosine Kinase 1 | Protein Coding | 42 | GC06P080334 | 19.43065262 |
| TCERG1 | Transcription Elongation Regulator 1 | Protein Coding | 35 | GC05P146447 | 19.35414886 |
| S100A6 | S100 Calcium Binding Protein A6 | Protein Coding | 38 | GC01M153545 | 19.34940338 |
| HSPA8 | Heat Shock Protein Family A (Hsp70) Member 8 | Protein Coding | 43 | GC11M123057 | 19.28698158 |
| GNRHR | Gonadotropin Releasing Hormone Receptor | Protein Coding | 44 | GC04M067737 | 19.26511383 |
| WNT3A | Wnt Family Member 3A | Protein Coding | 42 | GC01P229182 | 19.25158882 |
| EMSY | EMSY Transcriptional Repressor, BRCA2 Interacting | Protein Coding | 31 | GC11P077687 | 19.23917198 |
| NGFR | Nerve Growth Factor Receptor | Protein Coding | 41 | GC17P049495 | 19.22298431 |
| HLA-A | Major Histocompatibility Complex, Class I, A | Protein Coding | 42 | GC06P080311 | 19.2229538 |
| TFAP2C | Transcription Factor AP-2 Gamma | Protein Coding | 37 | GC20P056629 | 19.21789551 |
| HABP2 | Hyaluronan Binding Protein 2 | Protein Coding | 42 | GC10P113550 | 19.20987701 |
| BTK | Bruton Tyrosine Kinase | Protein Coding | 47 | GC0XM101349 | 19.18532562 |
| DIRC3 | Disrupted In Renal Carcinoma 3 | RNA Gene | 22 | GC02M217284 | 19.17963409 |
| CCNH | Cyclin H | Protein Coding | 40 | GC05M087311 | 19.17157173 |
| ELF3 | E74 Like ETS Transcription Factor 3 | Protein Coding | 36 | GC01P202007 | 19.13749313 |
| MIR181C | MicroRNA 181c | RNA Gene | 19 | GC19P014141 | 19.0856514 |
| SSTR2 | Somatostatin Receptor 2 | Protein Coding | 43 | GC17P073165 | 19.0596962 |
| BIRC3 | Baculoviral IAP Repeat Containing 3 | Protein Coding | 42 | GC11P102317 | 19.04791451 |
| TLR4 | Toll Like Receptor 4 | Protein Coding | 46 | GC09P117704 | 19.04549026 |
| BEX2 | Brain Expressed X-Linked 2 | Protein Coding | 30 | GC0XM103309 | 18.99990845 |
| MST1R | Macrophage Stimulating 1 Receptor | Protein Coding | 44 | GC03M051072 | 18.98189354 |
| TPD52 | Tumor Protein D52 | Protein Coding | 38 | GC08M079920 | 18.95630836 |
| PTPRG | Protein Tyrosine Phosphatase Receptor Type G | Protein Coding | 40 | GC03P061561 | 18.94031525 |
| CYTOR | Cytoskeleton Regulator RNA | RNA Gene | 18 | GC02P088013 | 18.92629433 |
| AGR2 | Anterior Gradient 2, Protein Disulphide Isomerase Family Member | Protein Coding | 35 | GC07M017036 | 18.91368484 |
| CFLAR | CASP8 And FADD Like Apoptosis Regulator | Protein Coding | 40 | GC02P201117 | 18.88399315 |
| PSEN2 | Presenilin 2 | Protein Coding | 44 | GC01P226870 | 18.87109566 |
| CCNE2 | Cyclin E2 | Protein Coding | 37 | GC08M094879 | 18.85843277 |
| DUSP1 | Dual Specificity Phosphatase 1 | Protein Coding | 42 | GC05M172768 | 18.83199501 |
| LNCR3 | Lung Cancer Susceptibility 3 | Genetic Locus | 2 | GC05U901116 | 18.83064461 |
| EIF3H | Eukaryotic Translation Initiation Factor 3 Subunit H | Protein Coding | 35 | GC08M116642 | 18.81959152 |
| INS-IGF2 | INS-IGF2 Readthrough | Protein Coding | 26 | GC11M003032 | 18.81957817 |
| MYLK | Myosin Light Chain Kinase | Protein Coding | 46 | GC03M123610 | 18.81845093 |
| CRP | C-Reactive Protein | Protein Coding | 41 | GC01M159726 | 18.81835938 |
| MINPP1 | Multiple Inositol-Polyphosphate Phosphatase 1 | Protein Coding | 41 | GC10P087504 | 18.7885437 |
| CDKN2C | Cyclin Dependent Kinase Inhibitor 2C | Protein Coding | 40 | GC01P050960 | 18.75035095 |
| HDAC6 | Histone Deacetylase 6 | Protein Coding | 47 | GC0XP048801 | 18.7425766 |
| SOCS3 | Suppressor Of Cytokine Signaling 3 | Protein Coding | 38 | GC17M078356 | 18.71607971 |
| MACROD1 | Mono-ADP Ribosylhydrolase 1 | Protein Coding | 30 | GC11M063998 | 18.70090485 |
| NGF | Nerve Growth Factor | Protein Coding | 44 | GC01M115285 | 18.69524384 |
| RECQL4 | RecQ Like Helicase 4 | Protein Coding | 38 | GC08M145324 | 18.69186592 |
| EPOR | Erythropoietin Receptor | Protein Coding | 43 | GC19M011377 | 18.68988419 |
| PCA3 | Prostate Cancer Associated 3 | RNA Gene | 22 | GC09P076691 | 18.6891346 |
| TNFRSF11B | TNF Receptor Superfamily Member 11b | Protein Coding | 43 | GC08M118923 | 18.68125916 |
| HNRNPK | Heterogeneous Nuclear Ribonucleoprotein K | Protein Coding | 39 | GC09M092476 | 18.6680069 |
| S100A2 | S100 Calcium Binding Protein A2 | Protein Coding | 36 | GC01M153561 | 18.62583923 |
| FBLN1 | Fibulin 1 | Protein Coding | 41 | GC22P045502 | 18.61326599 |
| MIR101-1 | MicroRNA 101-1 | RNA Gene | 19 | GC01M065058 | 18.60000038 |
| MIR497 | MicroRNA 497 | RNA Gene | 18 | GC17M007022 | 18.59696007 |
| UGT1A1 | UDP Glucuronosyltransferase Family 1 Member A1 | Protein Coding | 45 | GC02P233760 | 18.59664154 |
| MC1R | Melanocortin 1 Receptor | Protein Coding | 42 | GC16P089912 | 18.57043839 |
| TNFRSF11A | TNF Receptor Superfamily Member 11a | Protein Coding | 43 | GC18P062325 | 18.55318832 |
| BPIFA4P | BPI Fold Containing Family A Member 4, Pseudogene | Pseudogene | 16 | GC20P033193 | 18.52480507 |
| MIAT | Myocardial Infarction Associated Transcript | RNA Gene | 23 | GC22P026646 | 18.52258682 |
| FBN1 | Fibrillin 1 | Protein Coding | 42 | GC15M048408 | 18.52067947 |
| HMGCR | 3-Hydroxy-3-Methylglutaryl-CoA Reductase | Protein Coding | 41 | GC05P075336 | 18.50590897 |
| WNT6 | Wnt Family Member 6 | Protein Coding | 38 | GC02P218859 | 18.49902725 |
| CASP2 | Caspase 2 | Protein Coding | 43 | GC07P148003 | 18.48085403 |
| ERGIC3 | ERGIC And Golgi 3 | Protein Coding | 32 | GC20P035542 | 18.47565842 |
| ATF1 | Activating Transcription Factor 1 | Protein Coding | 40 | GC12P050763 | 18.46192169 |
| SNHG16 | Small Nucleolar RNA Host Gene 16 | RNA Gene | 18 | GC17P077027 | 18.45203018 |
| KDM4B | Lysine Demethylase 4B | Protein Coding | 42 | GC19P004969 | 18.44433212 |
| MCM2 | Minichromosome Maintenance Complex Component 2 | Protein Coding | 42 | GC03P127598 | 18.44353485 |
| NOTCH4 | Notch Receptor 4 | Protein Coding | 41 | GC06M063639 | 18.4356308 |
| OLA1 | Obg Like ATPase 1 | Protein Coding | 38 | GC02M174072 | 18.42562103 |
| LRP5 | LDL Receptor Related Protein 5 | Protein Coding | 44 | GC11P068298 | 18.41506004 |
| MUC5AC | Mucin 5AC, Oligomeric Mucus/Gel-Forming | Protein Coding | 36 | GC11P001698 | 18.3980751 |
| RHBDF2 | Rhomboid 5 Homolog 2 | Protein Coding | 39 | GC17M076470 | 18.38126183 |
| LMNA | Lamin A/C | Protein Coding | 44 | GC01P156082 | 18.3703022 |
| MUC4 | Mucin 4, Cell Surface Associated | Protein Coding | 36 | GC03M195746 | 18.35702896 |
| RAD21 | RAD21 Cohesin Complex Component | Protein Coding | 40 | GC08M116846 | 18.35100555 |
| MED12 | Mediator Complex Subunit 12 | Protein Coding | 39 | GC0XP071118 | 18.34406471 |
| CDX2 | Caudal Type Homeobox 2 | Protein Coding | 40 | GC13M027962 | 18.33543777 |
| AFP | Alpha Fetoprotein | Protein Coding | 42 | GC04P073431 | 18.33345413 |
| NCOA2 | Nuclear Receptor Coactivator 2 | Protein Coding | 40 | GC08M070109 | 18.32098961 |
| CSNK1A1 | Casein Kinase 1 Alpha 1 | Protein Coding | 43 | GC05M149492 | 18.32029724 |
| CDKN1C | Cyclin Dependent Kinase Inhibitor 1C | Protein Coding | 42 | GC11M003101 | 18.308218 |
| FOSL1 | FOS Like 1, AP-1 Transcription Factor Subunit | Protein Coding | 40 | GC11M087302 | 18.29872513 |
| IBSP | Integrin Binding Sialoprotein | Protein Coding | 36 | GC04P087799 | 18.28928757 |
| GHRH | Growth Hormone Releasing Hormone | Protein Coding | 34 | GC20M037251 | 18.27490616 |
| NTRK2 | Neurotrophic Receptor Tyrosine Kinase 2 | Protein Coding | 48 | GC09P084668 | 18.2670536 |
| LYVE1 | Lymphatic Vessel Endothelial Hyaluronan Receptor 1 | Protein Coding | 39 | GC11M011031 | 18.25503922 |
| ZEB2 | Zinc Finger E-Box Binding Homeobox 2 | Protein Coding | 42 | GC02M144384 | 18.2491188 |
| PDGFA | Platelet Derived Growth Factor Subunit A | Protein Coding | 39 | GC07M000497 | 18.22327614 |
| GH1 | Growth Hormone 1 | Protein Coding | 39 | GC17M063917 | 18.22081757 |
| SOX2-OT | SOX2 Overlapping Transcript | RNA Gene | 22 | GC03P180989 | 18.22036362 |
| DVL1 | Dishevelled Segment Polarity Protein 1 | Protein Coding | 42 | GC01M001335 | 18.22001648 |
| SULT1E1 | Sulfotransferase Family 1E Member 1 | Protein Coding | 39 | GC04M069841 | 18.19126129 |
| WWTR1 | WW Domain Containing Transcription Regulator 1 | Protein Coding | 36 | GC03M149517 | 18.17959213 |
| ESRRA | Estrogen Related Receptor Alpha | Protein Coding | 43 | GC11P064305 | 18.17102242 |
| IL6ST | Interleukin 6 Cytokine Family Signal Transducer | Protein Coding | 43 | GC05M055935 | 18.15406799 |
| SCGB1D2 | Secretoglobin Family 1D Member 2 | Protein Coding | 29 | GC11P062260 | 18.14400482 |
| MAP2K5 | Mitogen-Activated Protein Kinase Kinase 5 | Protein Coding | 41 | GC15P114658 | 18.12463379 |
| MAP2K3 | Mitogen-Activated Protein Kinase Kinase 3 | Protein Coding | 43 | GC17P052376 | 18.10329437 |

| | | | | | |
|---|---|---|---|---|---|
| SKP1 | S-Phase Kinase Associated Protein 1 | Protein Coding | 38 | GC05M134148 | 18.10222244 |
| CCDC170 | Coiled-Coil Domain Containing 170 | Protein Coding | 29 | GC06P151494 | 18.08868408 |
| MT-CO2 | Mitochondrially Encoded Cytochrome C Oxidase II | Protein Coding | 31 | GCMTP007587 | 18.08392715 |
| PRDM14 | PR/SET Domain 14 | Protein Coding | 35 | GC08M070051 | 18.05027771 |
| HLA-DQB1 | Major Histocompatibility Complex, Class II, DQ Beta 1 | Protein Coding | 39 | GC06M063664 | 18.04472351 |
| GRB7 | Growth Factor Receptor Bound Protein 7 | Protein Coding | 39 | GC17P053040 | 18.0304985 |
| IL11 | Interleukin 11 | Protein Coding | 36 | GC19M055364 | 18.02048492 |
| RINT1 | RAD50 Interactor 1 | Protein Coding | 33 | GC07P105532 | 18.0049324 |
| ELK1 | ETS Transcription Factor ELK1 | Protein Coding | 38 | GC0XM047635 | 17.99117279 |
| EPAS1 | Endothelial PAS Domain Protein 1 | Protein Coding | 44 | GC02P046293 | 17.98836517 |
| CRNN | Cornulin | Protein Coding | 32 | GC01M152381 | 17.98598099 |
| TCF7L1 | Transcription Factor 7 Like 1 | Protein Coding | 37 | GC02P085133 | 17.97861099 |
| SMAD6 | SMAD Family Member 6 | Protein Coding | 42 | GC15P066702 | 17.94721222 |
| YY1 | YY1 Transcription Factor | Protein Coding | 43 | GC14P100238 | 17.90467453 |
| MAPK12 | Mitogen-Activated Protein Kinase 12 | Protein Coding | 42 | GC22M056245 | 17.90167236 |
| PRDM2 | PR/SET Domain 2 | Protein Coding | 36 | GC01P013885 | 17.89762306 |
| CYP2E1 | Cytochrome P450 Family 2 Subfamily E Member 1 | Protein Coding | 40 | GC10P133520 | 17.89702415 |
| FANCG | FA Complementation Group G | Protein Coding | 38 | GC09M035073 | 17.8916378 |
| PANDAR | Promoter Of CDKN1A Antisense DNA Damage Activated RNA | RNA Gene | 13 | GC06M036673 | 17.88931465 |
| TCF7 | Transcription Factor 7 | Protein Coding | 39 | GC05P134114 | 17.8702774 |
| RNF6 | Ring Finger Protein 6 | Protein Coding | 34 | GC13M026132 | 17.85298538 |
| HOXA11-AS | HOXA11 Antisense RNA | RNA Gene | 21 | GC07P027184 | 17.85241127 |
| WNT2 | Wnt Family Member 2 | Protein Coding | 39 | GC07M117301 | 17.85004807 |
| BRCATA | Breast Cancer, 11;22 Translocation Associated | Genetic Locus | 2 | GC00U936827 | 17.83192635 |
| ANXA5 | Annexin A5 | Protein Coding | 41 | GC04M121667 | 17.80329895 |
| AKAP13 | A-Kinase Anchoring Protein 13 | Protein Coding | 39 | GC15P115167 | 17.79949188 |
| BMP7 | Bone Morphogenetic Protein 7 | Protein Coding | 40 | GC20M057168 | 17.79684448 |
| SPINK1 | Serine Peptidase Inhibitor Kazal Type 1 | Protein Coding | 39 | GC05M147825 | 17.78667068 |
| USF3 | Upstream Transcription Factor Family Member 3 | Protein Coding | 27 | GC03M113649 | 17.77613449 |
| NET1 | Neuroepithelial Cell Transforming 1 | Protein Coding | 38 | GC10P005444 | 17.77310371 |
| TG | Thyroglobulin | Protein Coding | 40 | GC08P132866 | 17.76802444 |
| PIAS1 | Protein Inhibitor Of Activated STAT 1 | Protein Coding | 40 | GC15P068054 | 17.73630142 |
| LUCAT1 | Lung Cancer Associated Transcript 1 | RNA Gene | 15 | GC05M091054 | 17.73524857 |
| MIR196A1 | MicroRNA 196a-1 | RNA Gene | 21 | GC17M048632 | 17.73219872 |
| LSP1 | Lymphocyte Specific Protein 1 | Protein Coding | 37 | GC11P001852 | 17.73200035 |
| CRNDE | Colorectal Neoplasia Differentially Expressed | RNA Gene | 19 | GC16M054845 | 17.70620918 |
| PPARA | Peroxisome Proliferator Activated Receptor Alpha | Protein Coding | 43 | GC22P046150 | 17.70164871 |
| CXCL10 | C-X-C Motif Chemokine Ligand 10 | Protein Coding | 39 | GC04M076021 | 17.6777935 |
| MAPK9 | Mitogen-Activated Protein Kinase 9 | Protein Coding | 43 | GC05M180249 | 17.67017365 |
| LIMK1 | LIM Domain Kinase 1 | Protein Coding | 44 | GC07P074082 | 17.65626335 |
| AKIP1 | A-Kinase Interacting Protein 1 | Protein Coding | 30 | GC11P008911 | 17.62635422 |
| PTP4A1 | Protein Tyrosine Phosphatase 4A1 | Protein Coding | 37 | GC06P080813 | 17.62378311 |
| TRIM28 | Tripartite Motif Containing 28 | Protein Coding | 38 | GC19P058544 | 17.60984802 |
| CSNK1D | Casein Kinase 1 Delta | Protein Coding | 44 | GC17M082239 | 17.60846901 |
| CAGE1 | Cancer Antigen 1 | Protein Coding | 31 | GC06M007326 | 17.57520676 |
| IGFBP6 | Insulin Like Growth Factor Binding Protein 6 | Protein Coding | 38 | GC12P053097 | 17.5314064 |
| MIR29B1 | MicroRNA 29b-1 | RNA Gene | 19 | GC07M130877 | 17.49835968 |
| ERG | ETS Transcription Factor ERG | Protein Coding | 40 | GC21M038367 | 17.47910118 |
| GRP | Gastrin Releasing Peptide | Protein Coding | 36 | GC18P059220 | 17.47583008 |
| MAD2L1 | Mitotic Arrest Deficient 2 Like 1 | Protein Coding | 40 | GC04M120055 | 17.47412491 |
| SFN | Stratifin | Protein Coding | 40 | GC01P027589 | 17.4733448 |
| NOVA1 | NOVA Alternative Splicing Regulator 1 | Protein Coding | 36 | GC14M026443 | 17.46785545 |
| CBL | Cbl Proto-Oncogene | Protein Coding | 46 | GC11P119206 | 17.46219635 |
| EFNA1 | Ephrin A1 | Protein Coding | 38 | GC01P155127 | 17.46183395 |
| FZD7 | Frizzled Class Receptor 7 | Protein Coding | 40 | GC02P202034 | 17.4375267 |
| ATRX | ATRX Chromatin Remodeler | Protein Coding | 42 | GC0XM077504 | 17.38416862 |
| RUNX3 | RUNX Family Transcription Factor 3 | Protein Coding | 39 | GC01M024899 | 17.37887764 |
| TDGF1 | Teratocarcinoma-Derived Growth Factor 1 | Protein Coding | 39 | GC03P046706 | 17.37639618 |
| FLT3 | Fms Related Receptor Tyrosine Kinase 3 | Protein Coding | 47 | GC13M028003 | 17.37231636 |
| ECM1 | Extracellular Matrix Protein 1 | Protein Coding | 41 | GC01P150508 | 17.36907196 |
| LRATD2 | LRAT Domain Containing 2 | Protein Coding | 27 | GC08M131533 | 17.35455132 |
| RAB25 | RAB25, Member RAS Oncogene Family | Protein Coding | 35 | GC01P156061 | 17.34250641 |
| NCAM1 | Neural Cell Adhesion Molecule 1 | Protein Coding | 42 | GC11P112961 | 17.33922195 |
| NEK2 | NIMA Related Kinase 2 | Protein Coding | 46 | GC01M211658 | 17.33416176 |
| BCL6 | BCL6 Transcription Repressor | Protein Coding | 42 | GC03M187721 | 17.32787704 |
| SLC9A3R1 | SLC9A3 Regulator 1 | Protein Coding | 40 | GC17P074749 | 17.32424164 |
| DLL4 | Delta Like Canonical Notch Ligand 4 | Protein Coding | 41 | GC15P040929 | 17.30464554 |
| LIFR | LIF Receptor Subunit Alpha | Protein Coding | 42 | GC05M038475 | 17.30018234 |
| TRIM37 | Tripartite Motif Containing 37 | Protein Coding | 37 | GC17M058982 | 17.29492569 |
| MIR31HG | MIR31 Host Gene | RNA Gene | 18 | GC09M021439 | 17.27435112 |
| MIR424 | MicroRNA 424 | RNA Gene | 18 | GC0XM134689 | 17.27360535 |
| SEMA3A | Semaphorin 3A | Protein Coding | 41 | GC07M083955 | 17.24454498 |
| BMPR2 | Bone Morphogenetic Protein Receptor Type 2 | Protein Coding | 45 | GC02P202376 | 17.24151039 |
| ANGPT2 | Angiopoietin 2 | Protein Coding | 42 | GC08M006499 | 17.23828888 |
| CALR | Calreticulin | Protein Coding | 46 | GC19P012938 | 17.21016693 |
| GPC3 | Glypican 3 | Protein Coding | 40 | GC0XM133535 | 17.20160866 |
| PRNCR1 | Prostate Cancer Associated Non-Coding RNA 1 | RNA Gene | 13 | GC08P127079 | 17.1833744 |
| POLQ | DNA Polymerase Theta | Protein Coding | 36 | GC03M121431 | 17.17251205 |
| CCN4 | Cellular Communication Network Factor 4 | Protein Coding | 31 | GC08P133192 | 17.15476227 |
| NRP2 | Neuropilin 2 | Protein Coding | 40 | GC02P205681 | 17.14358902 |
| HMGA2 | High Mobility Group AT-Hook 2 | Protein Coding | 40 | GC12P065824 | 17.14279938 |
| WEE1 | WEE1 G2 Checkpoint Kinase | Protein Coding | 41 | GC11P009573 | 17.1422081 |
| ZMYND8 | Zinc Finger MYND-Type Containing 8 | Protein Coding | 35 | GC20M047209 | 17.13962173 |
| S100A14 | S100 Calcium Binding Protein A14 | Protein Coding | 34 | GC01M153614 | 17.13676262 |
| PYHIN1 | Pyrin And HIN Domain Family Member 1 | Protein Coding | 33 | GC01P158900 | 17.13423538 |
| IFI16 | Interferon Gamma Inducible Protein 16 | Protein Coding | 38 | GC01P158969 | 17.13312721 |
| CDC27 | Cell Division Cycle 27 | Protein Coding | 36 | GC17M047117 | 17.1166153 |
| RBBP4 | RB Binding Protein 4, Chromatin Remodeling Factor | Protein Coding | 38 | GC01P032651 | 17.11579514 |
| HDAC5 | Histone Deacetylase 5 | Protein Coding | 43 | GC17M044076 | 17.09334564 |
| MAP2K4 | Mitogen-Activated Protein Kinase Kinase 4 | Protein Coding | 41 | GC17P012020 | 17.05929565 |
| FGF10 | Fibroblast Growth Factor 10 | Protein Coding | 42 | GC05M044340 | 17.05342484 |
| LNCRNA-ATB | LncRNA Activated By TGF-Beta | RNA Gene | 6 | GC14P031990 | 17.04117393 |
| S100A7 | S100 Calcium Binding Protein A7 | Protein Coding | 36 | GC01M153457 | 17.04098892 |
| TPM3 | Tropomyosin 3 | Protein Coding | 42 | GC01M154127 | 17.03959274 |
| RBX1 | Ring-Box 1 | Protein Coding | 38 | GC22P040951 | 17.02783775 |

| | | | | | |
|---|---|---|---|---|---|
| PRKD3 | Protein Kinase D3 | Protein Coding | 40 | GC02M037251 | 17.02088547 |
| CASC9 | Cancer Susceptibility 9 | RNA Gene | 15 | GC08M075132 | 17.01703644 |
| WNT7B | Wnt Family Member 7B | Protein Coding | 39 | GC22M045920 | 17.01536942 |
| EDNRA | Endothelin Receptor Type A | Protein Coding | 44 | GC04P147480 | 16.97464752 |
| SHH | Sonic Hedgehog Signaling Molecule | Protein Coding | 45 | GC07M155799 | 16.93344688 |
| ADH1B | Alcohol Dehydrogenase 1B (Class I), Beta Polypeptide | Protein Coding | 40 | GC04M099304 | 16.9194603 |
| PCAT6 | Prostate Cancer Associated Transcript 6 | RNA Gene | 14 | GC01P202810 | 16.91083527 |
| TP53BP1 | Tumor Protein P53 Binding Protein 1 | Protein Coding | 39 | GC15M043403 | 16.89426422 |
| PKM | Pyruvate Kinase M1/2 | Protein Coding | 40 | GC15M072199 | 16.89070702 |
| ACTN4 | Actinin Alpha 4 | Protein Coding | 42 | GC19P038647 | 16.89019012 |
| XPO1 | Exportin 1 | Protein Coding | 42 | GC02M061445 | 16.88855553 |
| SUSD2 | Sushi Domain Containing 2 | Protein Coding | 34 | GC22P024181 | 16.88442612 |
| BMP10 | Bone Morphogenetic Protein 10 | Protein Coding | 36 | GC02M068865 | 16.8741684 |
| ENSG00000215409 | Pseudogene Similar To Part Of Breast Cancer Antigen NY-BR-1 (NY-BR-1) | Pseudogene | 2 | GC10M027331 | 16.87351418 |
| ENSG00000230087 | Amphiphysin (Stiff-Man Syndrome With Breast Cancer 128kDa Autoantigen) (AMPH) Pseudogene | Pseudogene | 2 | GC11P005355 | 16.87351418 |
| ZNF365 | Zinc Finger Protein 365 | Protein Coding | 35 | GC10P062374 | 16.86443138 |
| MAPK7 | Mitogen-Activated Protein Kinase 7 | Protein Coding | 43 | GC17P052291 | 16.85609245 |
| MIR139 | MicroRNA 139 | RNA Gene | 19 | GC11M072615 | 16.81558418 |
| MIR296 | MicroRNA 296 | RNA Gene | 17 | GC20M058817 | 16.81295204 |
| STIM1 | Stromal Interaction Molecule 1 | Protein Coding | 43 | GC11P003855 | 16.81233788 |
| NR5A1 | Nuclear Receptor Subfamily 5 Group A Member 1 | Protein Coding | 45 | GC09M124481 | 16.79512787 |
| ALDH2 | Aldehyde Dehydrogenase 2 Family Member | Protein Coding | 46 | GC12P111766 | 16.79121399 |
| NR3C2 | Nuclear Receptor Subfamily 3 Group C Member 2 | Protein Coding | 42 | GC04M148078 | 16.78235817 |
| EWSR1 | EWS RNA Binding Protein 1 | Protein Coding | 39 | GC22P034936 | 16.76190376 |
| NORAD | Non-Coding RNA Activated By DNA Damage | RNA Gene | 15 | GC20M036234 | 16.72091484 |
| PTPA | Protein Phosphatase 2 Phosphatase Activator | Protein Coding | 34 | GC09P129111 | 16.69462585 |
| LINC00511 | Long Intergenic Non-Protein Coding RNA 511 | RNA Gene | 17 | GC17M072323 | 16.69207764 |
| AFAP1 | Actin Filament Associated Protein 1 | Protein Coding | 34 | GC04M007758 | 16.68624687 |
| CASC8 | Cancer Susceptibility 8 | RNA Gene | 15 | GC08M131407 | 16.68105507 |
| SGO1 | Shugoshin 1 | Protein Coding | 33 | GC03M020338 | 16.65383148 |
| CSNK2A2 | Casein Kinase 2 Alpha 2 | Protein Coding | 42 | GC16M058157 | 16.6466713 |
| ITGB3BP | Integrin Subunit Beta 3 Binding Protein | Protein Coding | 36 | GC01M063440 | 16.64251137 |
| THBS4 | Thrombospondin 4 | Protein Coding | 39 | GC05P079991 | 16.63389587 |
| SDHAF2 | Succinate Dehydrogenase Complex Assembly Factor 2 | Protein Coding | 36 | GC11P061430 | 16.62304497 |
| CDC25B | Cell Division Cycle 25B | Protein Coding | 41 | GC20P003998 | 16.62296677 |
| RALA | RAS Like Proto-Oncogene A | Protein Coding | 41 | GC07P039622 | 16.60293961 |
| EBAG9 | Estrogen Receptor Binding Site Associated Antigen 9 | Protein Coding | 34 | GC08P109536 | 16.59480858 |
| STARD13 | StAR Related Lipid Transfer Domain Containing 13 | Protein Coding | 36 | GC13M033103 | 16.58321571 |
| ABCA1 | ATP Binding Cassette Subfamily A Member 1 | Protein Coding | 43 | GC09M104781 | 16.57593346 |
| ZMIZ1 | Zinc Finger MIZ-Type Containing 1 | Protein Coding | 38 | GC10P079068 | 16.55551147 |
| S100A8 | S100 Calcium Binding Protein A8 | Protein Coding | 38 | GC01M153391 | 16.54747772 |
| SPRY4 | Sprouty RTK Signaling Antagonist 4 | Protein Coding | 39 | GC05M142310 | 16.54101181 |
| NT5E | 5'-Nucleotidase Ecto | Protein Coding | 46 | GC06P085449 | 16.53310394 |
| KDM4C | Lysine Demethylase 4C | Protein Coding | 38 | GC09P006720 | 16.52943611 |
| SRGAP1 | SLIT-ROBO Rho GTPase Activating Protein 1 | Protein Coding | 39 | GC12P063844 | 16.51960754 |
| ELOC | Elongin C | Protein Coding | 31 | GC08M073939 | 16.50382996 |
| PAX5 | Paired Box 5 | Protein Coding | 42 | GC09M036828 | 16.49632263 |
| IKBKE | Inhibitor Of Nuclear Factor Kappa B Kinase Subunit Epsilon | Protein Coding | 40 | GC01P206470 | 16.47996902 |
| ZFP36 | ZFP36 Ring Finger Protein | Protein Coding | 36 | GC19P039406 | 16.46621323 |
| PES1 | Pescadillo Ribosomal Biogenesis Factor 1 | Protein Coding | 34 | GC22M030576 | 16.46377754 |
| CSNK2B | Casein Kinase 2 Beta | Protein Coding | 42 | GC06P080357 | 16.44605255 |
| LOXL2 | Lysyl Oxidase Like 2 | Protein Coding | 42 | GC08M023296 | 16.44304276 |
| SNHG15 | Small Nucleolar RNA Host Gene 15 | RNA Gene | 21 | GC07M044983 | 16.43582535 |
| FZD8 | Frizzled Class Receptor 8 | Protein Coding | 39 | GC10M035638 | 16.39558029 |
| HIPK2 | Homeodomain Interacting Protein Kinase 2 | Protein Coding | 40 | GC07M139561 | 16.36944199 |
| PTPRC | Protein Tyrosine Phosphatase Receptor Type C | Protein Coding | 46 | GC01P198607 | 16.36679077 |
| TET2 | Tet Methylcytosine Dioxygenase 2 | Protein Coding | 40 | GC04P105145 | 16.36140823 |
| TP53COR1 | Tumor Protein P53 Pathway Corepressor 1 | RNA Gene | 8 | GC06U903133 | 16.35841179 |
| TPM1 | Tropomyosin 1 | Protein Coding | 43 | GC15P117505 | 16.35078049 |
| SLC39A1 | Solute Carrier Family 39 Member 1 | Protein Coding | 35 | GC01M153960 | 16.34622955 |
| MSLN | Mesothelin | Protein Coding | 37 | GC16P010651 | 16.3230896 |
| SQSTM1 | Sequestosome 1 | Protein Coding | 43 | GC05P179806 | 16.30486107 |
| FAM3C | FAM3 Metabolism Regulating Signaling Molecule C | Protein Coding | 35 | GC07M121349 | 16.30118179 |
| COL1A1 | Collagen Type I Alpha 1 Chain | Protein Coding | 45 | GC17M050183 | 16.29727364 |
| CSK | C-Terminal Src Kinase | Protein Coding | 42 | GC15P074782 | 16.28391266 |
| DLL1 | Delta Like Canonical Notch Ligand 1 | Protein Coding | 43 | GC06M170282 | 16.28027153 |
| MIR328 | MicroRNA 328 | RNA Gene | 19 | GC16M067203 | 16.27647972 |
| SAFB | Scaffold Attachment Factor B | Protein Coding | 35 | GC19P005623 | 16.27070808 |
| ENAH | ENAH Actin Regulator | Protein Coding | 37 | GC01M225486 | 16.26879501 |
| SLC39A6 | Solute Carrier Family 39 Member 6 | Protein Coding | 34 | GC18M036108 | 16.26856041 |
| CT83 | Cancer/Testis Antigen 83 | Protein Coding | 25 | GC0XM116461 | 16.24165535 |
| BLID | BH3-Like Motif Containing, Cell Death Inducer | Protein Coding | 28 | GC11M122115 | 16.2386322 |
| ARHGEF2 | Rho/Rac Guanine Nucleotide Exchange Factor 2 | Protein Coding | 41 | GC01M155946 | 16.22152328 |
| CALML4 | Calmodulin Like 4 | Protein Coding | 30 | GC15M068190 | 16.20362854 |
| ABI1 | Abl Interactor 1 | Protein Coding | 38 | GC10M026746 | 16.20173073 |
| P3H2 | Prolyl 3-Hydroxylase 2 | Protein Coding | 33 | GC03M189956 | 16.19100189 |
| LOC106721785 | BRCA2 Promoter/Silencer Region | Biological Region | 2 | GC13P032314 | 16.18345261 |
| FGF19 | Fibroblast Growth Factor 19 | Protein Coding | 39 | GC11M087464 | 16.16620827 |
| LSM1 | LSM1 Homolog, MRNA Degradation Associated | Protein Coding | 34 | GC08M038163 | 16.16596222 |
| SF3B2 | Splicing Factor 3b Subunit 2 | Protein Coding | 35 | GC11P066050 | 16.16465378 |
| FOLH1 | Folate Hydrolase 1 | Protein Coding | 42 | GC11M086914 | 16.15577698 |
| BACH1 | BTB Domain And CNC Homolog 1 | Protein Coding | 38 | GC21P029194 | 16.15313721 |
| WNT11 | Wnt Family Member 11 | Protein Coding | 40 | GC11M076186 | 16.13951302 |
| CST6 | Cystatin E/M | Protein Coding | 36 | GC11P069585 | 16.10393715 |
| PTN | Pleiotrophin | Protein Coding | 38 | GC07M137227 | 16.09283257 |
| SST | Somatostatin | Protein Coding | 37 | GC03M187668 | 16.08607864 |
| PRMT2 | Protein Arginine Methyltransferase 2 | Protein Coding | 36 | GC21P046635 | 16.08511734 |
| DVL2 | Dishevelled Segment Polarity Protein 2 | Protein Coding | 40 | GC17M007225 | 16.08255196 |
| CDC6 | Cell Division Cycle 6 | Protein Coding | 40 | GC17P040287 | 16.06809616 |
| HES1 | Hes Family BHLH Transcription Factor 1 | Protein Coding | 38 | GC03P194136 | 16.04419708 |
| CD28 | CD28 Molecule | Protein Coding | 42 | GC02P203706 | 16.01874924 |
| DAPK1 | Death Associated Protein Kinase 1 | Protein Coding | 44 | GC09P087497 | 15.9864502 |
| KMT2D | Lysine Methyltransferase 2D | Protein Coding | 38 | GC12M049018 | 15.98254108 |
| NR1I2 | Nuclear Receptor Subfamily 1 Group I Member 2 | Protein Coding | 41 | GC03P119780 | 15.97760773 |

| | | | | | |
|---|---|---|---|---|---|
| CD82 | CD82 Molecule | Protein Coding | 38 | GC11P044586 | 15.97494888 |
| DUSP3 | Dual Specificity Phosphatase 3 | Protein Coding | 40 | GC17M043766 | 15.95990086 |
| CERNA2 | Competing Endogenous LncRNA 2 For MicroRNA Let-7b | RNA Gene | 11 | GC10M084169 | 15.93496323 |
| EGOT | Eosinophil Granule Ontogeny Transcript | RNA Gene | 20 | GC03M004790 | 15.92107391 |
| SMO | Smoothened, Frizzled Class Receptor | Protein Coding | 44 | GC07P131679 | 15.91210556 |
| RYBP | RING1 And YY1 Binding Protein | Protein Coding | 36 | GC03M072371 | 15.90075302 |
| PPP1R14C | Protein Phosphatase 1 Regulatory Inhibitor Subunit 14C | Protein Coding | 34 | GC06P150143 | 15.89127731 |
| EHBP1 | EH Domain Binding Protein 1 | Protein Coding | 34 | GC02P062673 | 15.85427189 |
| TNFRSF1B | TNF Receptor Superfamily Member 1B | Protein Coding | 42 | GC01P012169 | 15.85061455 |
| ARFGEF3 | ARFGEF Family Member 3 | Protein Coding | 27 | GC06P138161 | 15.83289528 |
| FANCA | FA Complementation Group A | Protein Coding | 43 | GC16M089810 | 15.83119011 |
| INPPL1 | Inositol Polyphosphate Phosphatase Like 1 | Protein Coding | 44 | GC11P072223 | 15.82464027 |
| MIR202 | MicroRNA 202 | RNA Gene | 19 | GC10M133247 | 15.81293488 |
| KSR1 | Kinase Suppressor Of Ras 1 | Protein Coding | 38 | GC17P027456 | 15.78530788 |
| PCAT29 | Prostate Cancer Associated Transcript 29 | RNA Gene | 15 | GC15P115999 | 15.77013206 |
| CD36 | CD36 Molecule | Protein Coding | 43 | GC07P080369 | 15.76759148 |
| MIR124-3 | MicroRNA 124-3 | RNA Gene | 17 | GC20P063319 | 15.7643404 |
| PSMB5 | Proteasome 20S Subunit Beta 5 | Protein Coding | 39 | GC14M023016 | 15.76242828 |
| PECAM1 | Platelet And Endothelial Cell Adhesion Molecule 1 | Protein Coding | 36 | GC17M064319 | 15.74936867 |
| ESRRG | Estrogen Related Receptor Gamma | Protein Coding | 41 | GC01M216503 | 15.74637699 |
| FGF6 | Fibroblast Growth Factor 6 | Protein Coding | 37 | GC12M005112 | 15.73463821 |
| CKS1B | CDC28 Protein Kinase Regulatory Subunit 1B | Protein Coding | 36 | GC01P154974 | 15.73328018 |
| KLK13 | Kallikrein Related Peptidase 13 | Protein Coding | 36 | GC19M064227 | 15.72052002 |
| WNT5B | Wnt Family Member 5B | Protein Coding | 40 | GC12P001529 | 15.71951199 |
| PAX8 | Paired Box 8 | Protein Coding | 40 | GC02M113215 | 15.71911335 |
| CHD7 | Chromodomain Helicase DNA Binding Protein 7 | Protein Coding | 41 | GC08P060678 | 15.71481705 |
| GPC6 | Glypican 6 | Protein Coding | 40 | GC13P093226 | 15.70747757 |
| MAP2K6 | Mitogen-Activated Protein Kinase Kinase 6 | Protein Coding | 41 | GC17P069414 | 15.70016098 |
| JUP | Junction Plakoglobin | Protein Coding | 43 | GC17M041754 | 15.6970892 |
| FZR1 | Fizzy And Cell Division Cycle 20 Related 1 | Protein Coding | 37 | GC19P003506 | 15.68624496 |
| ROR2 | Receptor Tyrosine Kinase Like Orphan Receptor 2 | Protein Coding | 43 | GC09M092696 | 15.67702866 |
| UGDH | UDP-Glucose 6-Dehydrogenase | Protein Coding | 40 | GC04M039502 | 15.66829205 |
| B2M | Beta-2-Microglobulin | Protein Coding | 43 | GC15P044711 | 15.66143799 |
| FGF9 | Fibroblast Growth Factor 9 | Protein Coding | 41 | GC13P021671 | 15.66102982 |
| TRIP13 | Thyroid Hormone Receptor Interactor 13 | Protein Coding | 37 | GC05P000892 | 15.64871788 |
| PRKCQ | Protein Kinase C Theta | Protein Coding | 44 | GC10M006393 | 15.64853668 |
| HLA-B | Major Histocompatibility Complex, Class I, B | Protein Coding | 42 | GC06M063563 | 15.63983536 |
| SRRM3 | Serine/Arginine Repetitive Matrix 3 | Protein Coding | 28 | GC07P076201 | 15.61271667 |
| ANKRD17 | Ankyrin Repeat Domain 17 | Protein Coding | 35 | GC04M073074 | 15.61095619 |
| NNT-AS1 | NNT Antisense RNA 1 | RNA Gene | 14 | GC05M043989 | 15.61005211 |
| JAG2 | Jagged Canonical Notch Ligand 2 | Protein Coding | 39 | GC14M105140 | 15.60602283 |
| FZD1 | Frizzled Class Receptor 1 | Protein Coding | 42 | GC07P091264 | 15.59750366 |
| EIF4EBP1 | Eukaryotic Translation Initiation Factor 4E Binding Protein 1 | Protein Coding | 41 | GC08P038415 | 15.59385204 |
| BCDIN3D | BCDIN3 Domain Containing RNA Methyltransferase | Protein Coding | 31 | GC12M049839 | 15.59355259 |
| HPR | Haptoglobin-Related Protein | Protein Coding | 36 | GC16P072097 | 15.57952881 |
| COLCA2 | Colorectal Cancer Associated 2 | Protein Coding | 22 | GC11P111298 | 15.57478428 |
| CLOCK | Clock Circadian Regulator | Protein Coding | 39 | GC04M055427 | 15.55622673 |
| CA2 | Carbonic Anhydrase 2 | Protein Coding | 46 | GC08P085463 | 15.55531502 |
| HLA-DRB1 | Major Histocompatibility Complex, Class II, DR Beta 1 | Protein Coding | 42 | GC06M032578 | 15.55094051 |
| IGFBP4 | Insulin Like Growth Factor Binding Protein 4 | Protein Coding | 38 | GC17P040443 | 15.54673767 |
| SMAD1 | SMAD Family Member 1 | Protein Coding | 38 | GC04P145481 | 15.54111671 |
| KLF17 | Kruppel Like Factor 17 | Protein Coding | 34 | GC01P044048 | 15.53993225 |
| UIMC1 | Ubiquitin Interaction Motif Containing 1 | Protein Coding | 36 | GC05M176905 | 15.53562546 |
| ZNF703 | Zinc Finger Protein 703 | Protein Coding | 32 | GC08P037695 | 15.53136635 |
| SNHG7 | Small Nucleolar RNA Host Gene 7 | RNA Gene | 20 | GC09M137441 | 15.52927399 |
| ROCK2 | Rho Associated Coiled-Coil Containing Protein Kinase 2 | Protein Coding | 42 | GC02M011297 | 15.51844215 |
| HOXA-AS2 | HOXA Cluster Antisense RNA 2 | RNA Gene | 18 | GC07P027107 | 15.5171051 |
| ADIPOQ | Adiponectin, C1Q And Collagen Domain Containing | Protein Coding | 40 | GC03P186842 | 15.51100159 |
| PFN1 | Profilin 1 | Protein Coding | 42 | GC17M004945 | 15.49862003 |
| SPHK2 | Sphingosine Kinase 2 | Protein Coding | 39 | GC19P048619 | 15.49289322 |
| LINC00472 | Long Intergenic Non-Protein Coding RNA 472 | RNA Gene | 21 | GC06M071344 | 15.48509216 |
| MRC2 | Mannose Receptor C Type 2 | Protein Coding | 37 | GC17P062627 | 15.48212814 |
| IL7 | Interleukin 7 | Protein Coding | 39 | GC08M078689 | 15.43098354 |
| PRSS50 | Serine Protease 50 | Protein Coding | 30 | GC03M046712 | 15.42353439 |
| IFNA2 | Interferon Alpha 2 | Protein Coding | 38 | GC09M021384 | 15.41861153 |
| POSTN | Periostin | Protein Coding | 39 | GC13M037562 | 15.39550209 |
| PSMD6 | Proteasome 26S Subunit, Non-ATPase 6 | Protein Coding | 35 | GC03M063973 | 15.37109089 |
| CCNB2 | Cyclin B2 | Protein Coding | 39 | GC15P059105 | 15.36382294 |
| GRM1 | Glutamate Metabotropic Receptor 1 | Protein Coding | 46 | GC06P145973 | 15.36192799 |
| FSHR | Follicle Stimulating Hormone Receptor | Protein Coding | 44 | GC02M048953 | 15.34901047 |
| RPS20 | Ribosomal Protein S20 | Protein Coding | 39 | GC08M056067 | 15.34603977 |
| GPATCH2 | G-Patch Domain Containing 2 | Protein Coding | 30 | GC01M217426 | 15.33502007 |
| HULC | Hepatocellular Carcinoma Up-Regulated Long Non-Coding RNA | RNA Gene | 21 | GC06P008438 | 15.32021999 |
| HSPB8 | Heat Shock Protein Family B (Small) Member 8 | Protein Coding | 40 | GC12P119173 | 15.31597519 |
| PDLIM2 | PDZ And LIM Domain 2 | Protein Coding | 34 | GC08P022578 | 15.31382847 |
| SFRP1 | Secreted Frizzled Related Protein 1 | Protein Coding | 39 | GC08M041262 | 15.304842 |
| MIR32 | MicroRNA 32 | RNA Gene | 20 | GC09M109046 | 15.30354691 |
| FZD6 | Frizzled Class Receptor 6 | Protein Coding | 42 | GC08P103298 | 15.29839993 |
| KLK6 | Kallikrein Related Peptidase 6 | Protein Coding | 39 | GC19M050958 | 15.27448082 |
| ILK | Integrin Linked Kinase | Protein Coding | 41 | GC11P006604 | 15.25836563 |
| NUS1 | NUS1 Dehydrodolichyl Diphosphate Synthase Subunit | Protein Coding | 35 | GC06P117675 | 15.25811958 |
| GATA2 | GATA Binding Protein 2 | Protein Coding | 43 | GC03M128479 | 15.25410366 |
| PEA15 | Proliferation And Apoptosis Adaptor Protein 15 | Protein Coding | 38 | GC01P160205 | 15.25200748 |
| CUL3 | Cullin 3 | Protein Coding | 42 | GC02M224470 | 15.25135612 |
| RHNO1 | RAD9-HUS1-RAD1 Interacting Nuclear Orphan 1 | Protein Coding | 26 | GC12P002876 | 15.25109291 |
| CTAG1A | Cancer/Testis Antigen 1A | Protein Coding | 25 | GC0XP154585 | 15.24217224 |
| PSMA1 | Proteasome 20S Subunit Alpha 1 | Protein Coding | 38 | GC11M014505 | 15.20546722 |
| ST8SIA1 | ST8 Alpha-N-Acetyl-Neuraminide Alpha-2,8-Sialyltransferase 1 | Protein Coding | 35 | GC12M022063 | 15.17290115 |
| MCM7 | Minichromosome Maintenance Complex Component 7 | Protein Coding | 39 | GC07M100092 | 15.16850471 |
| NDRG1 | N-Myc Downstream Regulated 1 | Protein Coding | 40 | GC08M133237 | 15.16379929 |
| CDH13 | Cadherin 13 | Protein Coding | 40 | GC16P082626 | 15.15027237 |
| SCHLAP1 | SWI/SNF Complex Antagonist Associated With Prostate Cancer 1 | RNA Gene | 14 | GC02P180511 | 15.14886379 |
| MATK | Megakaryocyte-Associated Tyrosine Kinase | Protein Coding | 40 | GC19M003777 | 15.1460247 |
| NSD1 | Nuclear Receptor Binding SET Domain Protein 1 | Protein Coding | 39 | GC05P177134 | 15.14315224 |
| TOE1 | Target Of EGR1, Exonuclease | Protein Coding | 35 | GC01P045339 | 15.13434315 |

| | | | | | |
|---|---|---|---|---|---|
| GSE1 | Gse1 Coiled-Coil Protein | Protein Coding | 30 | GC16P085171 | 15.13055515 |
| CLDN4 | Claudin 4 | Protein Coding | 36 | GC07P073799 | 15.1293335 |
| NFIB | Nuclear Factor I B | Protein Coding | 39 | GC09M014077 | 15.12880611 |
| KEAP1 | Kelch Like ECH Associated Protein 1 | Protein Coding | 43 | GC19M010486 | 15.12071133 |
| SOX10 | SRY-Box Transcription Factor 10 | Protein Coding | 42 | GC22M057110 | 15.12004089 |
| RAP1A | RAP1A, Member Of RAS Oncogene Family | Protein Coding | 40 | GC01P111542 | 15.11334038 |
| CUEDC2 | CUE Domain Containing 2 | Protein Coding | 33 | GC10M102424 | 15.11082458 |
| NBAT1 | Neuroblastoma Associated Transcript 1 | RNA Gene | 15 | GC06M022137 | 15.10030937 |
| WASF3 | WASP Family Member 3 | Protein Coding | 36 | GC13P026557 | 15.09800911 |
| HIF1A-AS2 | HIF1A Antisense RNA 2 | RNA Gene | 15 | GC14M061747 | 15.09792709 |
| GACAT2 | Gastric Cancer Associated Transcript 2 | RNA Gene | 14 | GC18M008695 | 15.09209824 |
| POMC | Proopiomelanocortin | Protein Coding | 43 | GC02M025160 | 15.09052086 |
| VWA5A | Von Willebrand Factor A Domain Containing 5A | Protein Coding | 30 | GC11P124115 | 15.08930016 |
| U2AF1 | U2 Small Nuclear RNA Auxiliary Factor 1 | Protein Coding | 39 | GC21M043092 | 15.0776825 |
| MED1 | Mediator Complex Subunit 1 | Protein Coding | 36 | GC17M039404 | 15.04854774 |
| RPS6KB2 | Ribosomal Protein S6 Kinase B2 | Protein Coding | 42 | GC11P067428 | 15.02357578 |
| MIR99A | MicroRNA 99a | RNA Gene | 21 | GC21P016539 | 15.02351952 |
| TP73-AS1 | TP73 Antisense RNA 1 | RNA Gene | 21 | GC01M005619 | 15.01540756 |
| MORC2 | MORC Family CW-Type Zinc Finger 2 | Protein Coding | 36 | GC22M030925 | 14.99629593 |
| MIR181B2 | MicroRNA 181b-2 | RNA Gene | 18 | GC09P124693 | 14.97835922 |
| HPGD | 15-Hydroxyprostaglandin Dehydrogenase | Protein Coding | 42 | GC04M174490 | 14.97284126 |
| ADH1C | Alcohol Dehydrogenase 1C (Class I), Gamma Polypeptide | Protein Coding | 37 | GC04M099336 | 14.9610672 |
| NKILA | NF-KappaB Interacting LncRNA | RNA Gene | 12 | GC20P057711 | 14.9592762 |
| CADM1 | Cell Adhesion Molecule 1 | Protein Coding | 39 | GC11M115169 | 14.95804024 |
| PCAT7 | Prostate Cancer Associated Transcript 7 | RNA Gene | 13 | GC09P094555 | 14.94949913 |
| CALCA | Calcitonin Related Polypeptide Alpha | Protein Coding | 42 | GC11M014945 | 14.94063187 |
| FOSL2 | FOS Like 2, AP-1 Transcription Factor Subunit | Protein Coding | 37 | GC02P028392 | 14.93556786 |
| FGF5 | Fibroblast Growth Factor 5 | Protein Coding | 39 | GC04P080266 | 14.91353416 |
| APOE | Apolipoprotein E | Protein Coding | 45 | GC19P063844 | 14.9046917 |
| ARRDC3 | Arrestin Domain Containing 3 | Protein Coding | 32 | GC05M091368 | 14.88325119 |
| PPP1R1B | Protein Phosphatase 1 Regulatory Inhibitor Subunit 1B | Protein Coding | 38 | GC17P039626 | 14.87669754 |
| IL1A | Interleukin 1 Alpha | Protein Coding | 39 | GC02M112773 | 14.87326241 |
| WDR11 | WD Repeat Domain 11 | Protein Coding | 35 | GC10P120851 | 14.84602642 |
| GACAT3 | Gastric Cancer Associated Transcript 3 | RNA Gene | 14 | GC02P016050 | 14.82136154 |
| EDN1 | Endothelin 1 | Protein Coding | 43 | GC06P012256 | 14.81613922 |
| CD40 | CD40 Molecule | Protein Coding | 43 | GC20P046118 | 14.79821205 |
| MMP17 | Matrix Metallopeptidase 17 | Protein Coding | 40 | GC12P131828 | 14.78830338 |
| CSN2 | Casein Beta | Protein Coding | 30 | GC04M069955 | 14.76107216 |
| FANCI | FA Complementation Group I | Protein Coding | 37 | GC15P089243 | 14.75641823 |
| PBXIP1 | PBX Homeobox Interacting Protein 1 | Protein Coding | 32 | GC01M154944 | 14.75266647 |
| NANOG | Nanog Homeobox | Protein Coding | 35 | GC12P007787 | 14.74932194 |
| FZD5 | Frizzled Class Receptor 5 | Protein Coding | 42 | GC02M207762 | 14.71254921 |
| SRY | Sex Determining Region Y | Protein Coding | 33 | GC0YM002698 | 14.7042942 |
| GPR161 | G Protein-Coupled Receptor 161 | Protein Coding | 38 | GC01M168080 | 14.70228577 |
| RRM2 | Ribonucleotide Reductase Regulatory Subunit M2 | Protein Coding | 42 | GC02P010123 | 14.69666004 |
| TRERF1 | Transcriptional Regulating Factor 1 | Protein Coding | 34 | GC06M042224 | 14.68091869 |
| KLC1 | Kinesin Light Chain 1 | Protein Coding | 36 | GC14P109264 | 14.66840076 |
| ABCC12 | ATP Binding Cassette Subfamily C Member 12 | Protein Coding | 35 | GC16M048081 | 14.64628601 |
| FABP4 | Fatty Acid Binding Protein 4 | Protein Coding | 40 | GC08M081478 | 14.63455772 |
| AREL1 | Apoptosis Resistant E3 Ubiquitin Protein Ligase 1 | Protein Coding | 31 | GC14M074653 | 14.60674953 |
| PIGR | Polymeric Immunoglobulin Receptor | Protein Coding | 36 | GC01M206928 | 14.60256386 |
| TFPI | Tissue Factor Pathway Inhibitor | Protein Coding | 40 | GC02M187464 | 14.58492374 |
| LOC110806263 | TERT 5' Regulatory Region | Biological Region | 3 | GC05P001294 | 14.5654459 |
| STRADA | STE20 Related Adaptor Alpha | Protein Coding | 39 | GC17M063683 | 14.5646801 |
| LIN28B | Lin-28 Homolog B | Protein Coding | 37 | GC06P104949 | 14.55437279 |
| APOBEC3G | Apolipoprotein B MRNA Editing Enzyme Catalytic Subunit 3G | Protein Coding | 36 | GC22P039160 | 14.5519371 |
| CYP17A1-AS1 | CYP17A1 Antisense RNA 1 | RNA Gene | 13 | GC10P102747 | 14.54947472 |
| TCF4 | Transcription Factor 4 | Protein Coding | 42 | GC18M055222 | 14.5494194 |
| CT45A10 | Cancer/Testis Antigen Family 45 Member A10 | Protein Coding | 17 | GC0XM135881 | 14.54715538 |
| NR0B1 | Nuclear Receptor Subfamily 0 Group B Member 1 | Protein Coding | 42 | GC0XM030304 | 14.53892326 |
| WT1-AS | WT1 Antisense RNA | RNA Gene | 24 | GC11P032434 | 14.53635788 |
| KDM1A | Lysine Demethylase 1A | Protein Coding | 43 | GC01P023019 | 14.53343391 |
| RABL6 | RAB, Member RAS Oncogene Family Like 6 | Protein Coding | 30 | GC09P136807 | 14.52038956 |
| HOXA5 | Homeobox A5 | Protein Coding | 37 | GC07M027542 | 14.51919746 |
| MAGEA1 | MAGE Family Member A1 | Protein Coding | 34 | GC0XP153179 | 14.51650429 |
| HPSE | Heparanase | Protein Coding | 40 | GC04M083292 | 14.51037693 |
| FABP3 | Fatty Acid Binding Protein 3 | Protein Coding | 39 | GC01M031365 | 14.50899506 |
| MIRLET7F1 | MicroRNA Let-7f-1 | RNA Gene | 18 | GC09P094275 | 14.50312233 |
| USP15 | Ubiquitin Specific Peptidase 15 | Protein Coding | 41 | GC12P062260 | 14.49831963 |
| NOS3 | Nitric Oxide Synthase 3 | Protein Coding | 46 | GC07P150990 | 14.49471283 |
| MT-TT | Mitochondrially Encoded TRNA-Thr (ACN) | RNA Gene | 14 | GCMTP015890 | 14.48304749 |
| TLR9 | Toll Like Receptor 9 | Protein Coding | 41 | GC03M052222 | 14.47827435 |
| BIRC2 | Baculoviral IAP Repeat Containing 2 | Protein Coding | 40 | GC11P102347 | 14.46945667 |
| TAB1 | TGF-Beta Activated Kinase 1 (MAP3K7) Binding Protein 1 | Protein Coding | 37 | GC22P039419 | 14.44584465 |
| PTGS1 | Prostaglandin-Endoperoxide Synthase 1 | Protein Coding | 42 | GC09P122370 | 14.42102337 |
| TPM2 | Tropomyosin 2 | Protein Coding | 41 | GC09M035672 | 14.40954399 |
| RANBP9 | RAN Binding Protein 9 | Protein Coding | 37 | GC06M013621 | 14.40802956 |
| LIG4 | DNA Ligase 4 | Protein Coding | 43 | GC13M108207 | 14.40085506 |
| LHCGR | Luteinizing Hormone/Choriogonadotropin Receptor | Protein Coding | 42 | GC02M048686 | 14.3932209 |
| TPX2 | TPX2 Microtubule Nucleation Factor | Protein Coding | 37 | GC20P031739 | 14.39019203 |
| IFNA1 | Interferon Alpha 1 | Protein Coding | 38 | GC09P021585 | 14.36728477 |
| SELENOP | Selenoprotein P | Protein Coding | 30 | GC05M042800 | 14.34594154 |
| GAST | Gastrin | Protein Coding | 35 | GC17P041712 | 14.34404755 |
| SAFB2 | Scaffold Attachment Factor B2 | Protein Coding | 34 | GC19M005587 | 14.33753395 |
| IRAIN | IGF1R Antisense Imprinted Non-Protein Coding RNA | RNA Gene | 14 | GC15M098645 | 14.29806709 |
| FGF13 | Fibroblast Growth Factor 13 | Protein Coding | 38 | GC0XM138615 | 14.29644203 |
| SERPINA1 | Serpin Family A Member 1 | Protein Coding | 44 | GC14M094376 | 14.28387737 |
| KDM3B | Lysine Demethylase 3B | Protein Coding | 39 | GC05P138352 | 14.280653 |
| CASC15 | Cancer Susceptibility 15 | RNA Gene | 18 | GC06P021669 | 14.27688789 |
| ALDOA | Aldolase, Fructose-Bisphosphate A | Protein Coding | 43 | GC16P030064 | 14.27668095 |
| PHLDA2 | Pleckstrin Homology Like Domain Family A Member 2 | Protein Coding | 33 | GC11M002928 | 14.27345848 |
| PRSS1 | Serine Protease 1 | Protein Coding | 42 | GC07P147973 | 14.24200439 |
| MIR423 | MicroRNA 423 | RNA Gene | 19 | GC17P030117 | 14.23503304 |
| SCGB3A1 | Secretoglobin Family 3A Member 1 | Protein Coding | 31 | GC05M180590 | 14.2114172 |
| CDH5 | Cadherin 5 | Protein Coding | 42 | GC16P066366 | 14.21076393 |

| | | | | | |
|---|---|---|---|---|---|
| CETN3 | Centrin 3 | Protein Coding | 35 | GC05M090392 | 14.21004581 |
| MTAP | Methylthioadenosine Phosphorylase | Protein Coding | 43 | GC09P021792 | 14.20419312 |
| MAGEA4 | MAGE Family Member A4 | Protein Coding | 34 | GC0XP151912 | 14.20407486 |
| MIR663A | MicroRNA 663a | RNA Gene | 16 | GC20M026189 | 14.20243073 |
| RECK | Reversion Inducing Cysteine Rich Protein With Kazal Motifs | Protein Coding | 36 | GC09P036036 | 14.1889925 |
| MAGEA3 | MAGE Family Member A3 | Protein Coding | 34 | GC0XP152698 | 14.18388462 |
| CCKBR | Cholecystokinin B Receptor | Protein Coding | 42 | GC11P006259 | 14.17389393 |
| TPD52L2 | TPD52 Like 2 | Protein Coding | 34 | GC20P063865 | 14.16788387 |
| RNF146 | Ring Finger Protein 146 | Protein Coding | 33 | GC06P127266 | 14.155797 |
| ETV1 | ETS Variant Transcription Factor 1 | Protein Coding | 42 | GC07M013891 | 14.12547588 |
| CLPTM1L | CLPTM1 Like | Protein Coding | 34 | GC05M001317 | 14.12442589 |
| WWC1 | WW And C2 Domain Containing 1 | Protein Coding | 36 | GC05P168291 | 14.114851 |
| PRC1 | Protein Regulator Of Cytokinesis 1 | Protein Coding | 35 | GC15M090966 | 14.10525131 |
| CXCR2 | C-X-C Motif Chemokine Receptor 2 | Protein Coding | 44 | GC02P218125 | 14.09226131 |
| APPBP2 | Amyloid Beta Precursor Protein Binding Protein 2 | Protein Coding | 32 | GC17M060443 | 14.08628654 |
| DDR2 | Discoidin Domain Receptor Tyrosine Kinase 2 | Protein Coding | 46 | GC01P162631 | 14.07775497 |
| DLEU1 | Deleted In Lymphocytic Leukemia 1 | RNA Gene | 27 | GC13P050119 | 14.06702042 |
| IKBKB | Inhibitor Of Nuclear Factor Kappa B Kinase Subunit Beta | Protein Coding | 47 | GC08P042271 | 14.06311893 |
| LMTK3 | Lemur Tyrosine Kinase 3 | Protein Coding | 32 | GC19M048485 | 14.06121826 |
| MAPK6 | Mitogen-Activated Protein Kinase 6 | Protein Coding | 40 | GC15P051952 | 14.05555725 |
| LYPD3 | LY6/PLAUR Domain Containing 3 | Protein Coding | 33 | GC19M043460 | 14.05420399 |
| CXCL1 | C-X-C Motif Chemokine Ligand 1 | Protein Coding | 37 | GC04P073869 | 14.03589058 |
| FAP | Fibroblast Activation Protein Alpha | Protein Coding | 41 | GC02M162170 | 14.01812363 |
| DIABLO | Diablo IAP-Binding Mitochondrial Protein | Protein Coding | 42 | GC12M122208 | 14.01550961 |
| CD4 | CD4 Molecule | Protein Coding | 44 | GC12P006786 | 14.01195335 |
| POLH | DNA Polymerase Eta | Protein Coding | 44 | GC06P043576 | 14.00205898 |
| EIF3A | Eukaryotic Translation Initiation Factor 3 Subunit A | Protein Coding | 38 | GC10M119034 | 13.98945332 |
| MNX1-AS1 | MNX1 Antisense RNA 1 (Head To Head) | RNA Gene | 16 | GC07P157010 | 13.96966934 |
| TKT | Transketolase | Protein Coding | 41 | GC03M053224 | 13.96899033 |
| SVEP1 | Sushi, Von Willebrand Factor Type A, EGF And Pentraxin Domain Containing 1 | Protein Coding | 33 | GC09M110365 | 13.9613781 |
| GLI2 | GLI Family Zinc Finger 2 | Protein Coding | 43 | GC02P120735 | 13.94918633 |
| KISS1R | KISS1 Receptor | Protein Coding | 43 | GC19P002496 | 13.94180107 |
| RASGRP3 | RAS Guanyl Releasing Protein 3 | Protein Coding | 38 | GC02P033436 | 13.93423271 |
| TYR | Tyrosinase | Protein Coding | 45 | GC11P089177 | 13.92597961 |
| EPB41L4A-DT | EPB41L4A Divergent Transcript | RNA Gene | 15 | GC05P112421 | 13.9185133 |
| HBP1 | HMG-Box Transcription Factor 1 | Protein Coding | 34 | GC07P107168 | 13.91519165 |
| PPP5C | Protein Phosphatase 5 Catalytic Subunit | Protein Coding | 40 | GC19P063894 | 13.91337585 |
| CERK | Ceramide Kinase | Protein Coding | 36 | GC22M046684 | 13.91195107 |
| BANP | BTG3 Associated Nuclear Protein | Protein Coding | 35 | GC16P087949 | 13.90884686 |
| UBE2T | Ubiquitin Conjugating Enzyme E2 T | Protein Coding | 38 | GC01M202332 | 13.89500713 |
| KLK9 | Kallikrein Related Peptidase 9 | Protein Coding | 32 | GC19M051002 | 13.89460564 |
| KIAA1671 | KIAA1671 | Protein Coding | 26 | GC22P034780 | 13.89123917 |
| ADAM11 | ADAM Metallopeptidase Domain 11 | Protein Coding | 36 | GC17P053241 | 13.88733864 |
| AIF1L | Allograft Inflammatory Factor 1 Like | Protein Coding | 31 | GC09P131185 | 13.8804493 |
| COX7A2L | Cytochrome C Oxidase Subunit 7A2 Like | Protein Coding | 36 | GC02M042312 | 13.87498665 |
| DUSP6 | Dual Specificity Phosphatase 6 | Protein Coding | 44 | GC12M089347 | 13.84714127 |
| HIC1 | HIC ZBTB Transcriptional Repressor 1 | Protein Coding | 37 | GC17P002054 | 13.84281349 |
| FANCF | FA Complementation Group F | Protein Coding | 36 | GC11M022600 | 13.83585548 |
| FAM83D | Family With Sequence Similarity 83 Member D | Protein Coding | 32 | GC20P038926 | 13.82216263 |
| MACC1 | MET Transcriptional Regulator MACC1 | Protein Coding | 33 | GC07M020140 | 13.81853867 |
| NOP16 | NOP16 Nucleolar Protein | Protein Coding | 28 | GC05M176383 | 13.79957962 |
| CASC16 | Cancer Susceptibility 16 | RNA Gene | 14 | GC16M052587 | 13.7901392 |
| PCID2 | PCI Domain Containing 2 | Protein Coding | 29 | GC13M113167 | 13.78055 |
| PLK3 | Polo Like Kinase 3 | Protein Coding | 40 | GC01P044799 | 13.77657318 |
| CITED4 | Cbp/P300 Interacting Transactivator With Glu/Asp Rich Carboxy-Terminal Domain 4 | Protein Coding | 30 | GC01M040861 | 13.77336121 |
| RNF11 | Ring Finger Protein 11 | Protein Coding | 33 | GC01P051236 | 13.77109337 |
| TRADD | TNFRSF1A Associated Via Death Domain | Protein Coding | 38 | GC16M067154 | 13.71925545 |
| MCM4 | Minichromosome Maintenance Complex Component 4 | Protein Coding | 42 | GC08P047965 | 13.71891308 |
| MUC2 | Mucin 2, Oligomeric Mucus/Gel-Forming | Protein Coding | 36 | GC11P001074 | 13.71017456 |
| LINC00052 | Long Intergenic Non-Protein Coding RNA 52 | RNA Gene | 18 | GC15P087576 | 13.69445801 |
| GREB1L | GREB1 Like Retinoic Acid Receptor Coactivator | Protein Coding | 31 | GC18P021242 | 13.68567944 |
| CCDC26 | CCDC26 Long Non-Coding RNA | RNA Gene | 23 | GC08M131430 | 13.66417408 |
| RERG | RAS Like Estrogen Regulated Growth Inhibitor | Protein Coding | 34 | GC12M020519 | 13.64552879 |
| SETBP1 | SET Binding Protein 1 | Protein Coding | 36 | GC18P044680 | 13.63828373 |
| CASC11 | Cancer Susceptibility 11 | RNA Gene | 14 | GC08M131419 | 13.61813831 |
| SMC4 | Structural Maintenance Of Chromosomes 4 | Protein Coding | 36 | GC03P160399 | 13.61676788 |
| PDCD4 | Programmed Cell Death 4 | Protein Coding | 38 | GC10P110871 | 13.59399796 |
| EPHX1 | Epoxide Hydrolase 1 | Protein Coding | 40 | GC01P225810 | 13.59269905 |
| LINP1 | LncRNA In Non-Homologous End Joining Pathway 1 | RNA Gene | 13 | GC10P006738 | 13.58720779 |
| PAXIP1 | PAX Interacting Protein 1 | Protein Coding | 34 | GC07M154943 | 13.58651733 |
| PLA2G10 | Phospholipase A2 Group X | Protein Coding | 39 | GC16M014672 | 13.56683922 |
| DSCAM-AS1 | DSCAM Antisense RNA 1 | RNA Gene | 15 | GC21P040383 | 13.54967117 |
| RLIM | Ring Finger Protein, LIM Domain Interacting | Protein Coding | 35 | GC0XM074685 | 13.54940796 |
| TPD52L1 | TPD52 Like 1 | Protein Coding | 35 | GC06P125119 | 13.54172897 |
| F3 | Coagulation Factor III, Tissue Factor | Protein Coding | 41 | GC01M094580 | 13.53842735 |
| CFTR | CF Transmembrane Conductance Regulator | Protein Coding | 46 | GC07P117287 | 13.51272678 |
| MIF | Macrophage Migration Inhibitory Factor | Protein Coding | 43 | GC22P023894 | 13.51159477 |
| MIR30B | MicroRNA 30b | RNA Gene | 20 | GC08M134800 | 13.51027393 |
| ITGA5 | Integrin Subunit Alpha 5 | Protein Coding | 44 | GC12M054846 | 13.50313473 |
| PROX1 | Prospero Homeobox 1 | Protein Coding | 39 | GC01P213983 | 13.48035049 |
| MT-TE | Mitochondrially Encoded TRNA-Glu (GAA/G) | RNA Gene | 11 | GCMTM014676 | 13.46931267 |
| WNT4 | Wnt Family Member 4 | Protein Coding | 42 | GC01M022190 | 13.46073246 |
| PAWR | Pro-Apoptotic WT1 Regulator | Protein Coding | 36 | GC12M079574 | 13.4571991 |
| MAPK4 | Mitogen-Activated Protein Kinase 4 | Protein Coding | 39 | GC18P050564 | 13.44702911 |
| MPO | Myeloperoxidase | Protein Coding | 46 | GC17M058269 | 13.44246674 |
| MIR144 | MicroRNA 144 | RNA Gene | 19 | GC17M034587 | 13.42802143 |
| ALOX5 | Arachidonate 5-Lipoxygenase | Protein Coding | 44 | GC10P045374 | 13.40931606 |
| HEYL | Hes Related Family BHLH Transcription Factor With YRPW Motif Like | Protein Coding | 35 | GC01M039623 | 13.40849876 |
| MIR24-1 | MicroRNA 24-1 | RNA Gene | 19 | GC09P095086 | 13.37183857 |
| GNA11 | G Protein Subunit Alpha 11 | Protein Coding | 43 | GC19P003094 | 13.3356638 |
| NOD2 | Nucleotide Binding Oligomerization Domain Containing 2 | Protein Coding | 43 | GC16P050693 | 13.32748318 |
| LINC01116 | Long Intergenic Non-Protein Coding RNA 1116 | RNA Gene | 15 | GC02M176629 | 13.27373981 |
| CASR | Calcium Sensing Receptor | Protein Coding | 46 | GC03P122183 | 13.27145195 |
| MIR124-1 | MicroRNA 124-1 | RNA Gene | 20 | GC08M009903 | 13.25398636 |

| | | | | | |
|---|---|---|---|---|---|
| MTR | 5-Methyltetrahydrofolate-Homocysteine Methyltransferase | Protein Coding | 43 | GC01P236795 | 13.24990273 |
| ACTC1 | Actin Alpha Cardiac Muscle 1 | Protein Coding | 39 | GC15M034790 | 13.24750805 |
| EXO1 | Exonuclease 1 | Protein Coding | 39 | GC01P241847 | 13.22682285 |
| CEACAM3 | CEA Cell Adhesion Molecule 3 | Protein Coding | 39 | GC19P041796 | 13.22066116 |
| LINC01234 | Long Intergenic Non-Protein Coding RNA 1234 | RNA Gene | 15 | GC12M113584 | 13.21916199 |
| CCNT2 | Cyclin T2 | Protein Coding | 34 | GC02P134918 | 13.21852589 |
| MUC6 | Mucin 6, Oligomeric Mucus/Gel-Forming | Protein Coding | 35 | GC11M001012 | 13.21110821 |
| TUNAR | TCL1 Upstream Neural Differentiation-Associated RNA | RNA Gene | 17 | GC14P095876 | 13.20963669 |
| MIR136 | MicroRNA 136 | RNA Gene | 19 | GC14P109207 | 13.20204926 |
| MIR532 | MicroRNA 532 | RNA Gene | 17 | GC0XP050564 | 13.18967724 |
| IL13 | Interleukin 13 | Protein Coding | 40 | GC05P132656 | 13.17861176 |
| BCR | BCR Activator Of RhoGEF And GTPase | Protein Coding | 46 | GC22P023179 | 13.14716721 |
| HK2 | Hexokinase 2 | Protein Coding | 42 | GC02P074833 | 13.11887932 |
| LINC00628 | Long Intergenic Non-Protein Coding RNA 628 | RNA Gene | 14 | GC01M204494 | 13.11081886 |
| SRD5A1 | Steroid 5 Alpha-Reductase 1 | Protein Coding | 39 | GC05P006633 | 13.10195637 |
| MIR181D | MicroRNA 181d | RNA Gene | 17 | GC19P013874 | 13.08912849 |
| HSD17B3 | Hydroxysteroid 17-Beta Dehydrogenase 3 | Protein Coding | 43 | GC09M096255 | 13.05603981 |
| AFAP1-AS1 | AFAP1 Antisense RNA 1 | RNA Gene | 17 | GC04P007756 | 13.04243469 |
| APAF1 | Apoptotic Peptidase Activating Factor 1 | Protein Coding | 42 | GC12P098645 | 13.02818012 |
| PCAT18 | Prostate Cancer Associated Transcript 18 | RNA Gene | 19 | GC18M026957 | 13.01960373 |
| PRKDC | Protein Kinase, DNA-Activated, Catalytic Subunit | Protein Coding | 45 | GC08M047773 | 12.99580574 |
| PLG | Plasminogen | Protein Coding | 44 | GC06P160702 | 12.95958424 |
| GATA4 | GATA Binding Protein 4 | Protein Coding | 43 | GC08P011676 | 12.95115852 |
| SEMA4A | Semaphorin 4A | Protein Coding | 40 | GC01P156147 | 12.94712257 |
| MYD88 | MYD88 Innate Immune Signal Transduction Adaptor | Protein Coding | 44 | GC03P038139 | 12.94666672 |
| GSN | Gelsolin | Protein Coding | 43 | GC09P121201 | 12.92563725 |
| RMST | Rhabdomyosarcoma 2 Associated Transcript | RNA Gene | 21 | GC12P097431 | 12.92510033 |
| DPP10-AS1 | DPP10 Antisense RNA 1 | RNA Gene | 14 | GC02M115130 | 12.9145689 |
| IKBKG | Inhibitor Of Nuclear Factor Kappa B Kinase Regulatory Subunit Gamma | Protein Coding | 43 | GC0XP154541 | 12.89281273 |
| PCAT19 | Prostate Cancer Associated Transcript 19 | RNA Gene | 15 | GC19M063827 | 12.89021015 |
| GHR | Growth Hormone Receptor | Protein Coding | 41 | GC05P042429 | 12.88380432 |
| CAT | Catalase | Protein Coding | 46 | GC11P034460 | 12.84266281 |
| WIF1 | WNT Inhibitory Factor 1 | Protein Coding | 39 | GC12M065050 | 12.83358574 |
| PDPK1 | 3-Phosphoinositide Dependent Protein Kinase 1 | Protein Coding | 44 | GC16P002537 | 12.83327103 |
| KLK4 | Kallikrein Related Peptidase 4 | Protein Coding | 42 | GC19M064215 | 12.82184982 |
| NTPCR | Nucleoside-Triphosphatase, Cancer-Related | Protein Coding | 31 | GC01P232950 | 12.79775429 |
| MAGEC2 | MAGE Family Member C2 | Protein Coding | 31 | GC0XM142202 | 12.76898003 |
| CHD1 | Chromodomain Helicase DNA Binding Protein 1 | Protein Coding | 41 | GC05M098853 | 12.76213169 |
| GNAQ | G Protein Subunit Alpha Q | Protein Coding | 44 | GC09M077716 | 12.75560284 |
| LINC00901 | Long Intergenic Non-Protein Coding RNA 901 | RNA Gene | 14 | GC03P116911 | 12.7320919 |
| DELEC1 | Deleted In Esophageal Cancer 1 | RNA Gene | 22 | GC09P118628 | 12.72446442 |
| CIB1 | Calcium And Integrin Binding 1 | Protein Coding | 37 | GC15M090229 | 12.72350121 |
| ZNF667-AS1 | ZNF667 Antisense RNA 1 (Head To Head) | RNA Gene | 14 | GC19P056477 | 12.71245098 |
| NES | Nestin | Protein Coding | 36 | GC01M156668 | 12.68545151 |
| MIR92A1 | MicroRNA 92a-1 | RNA Gene | 19 | GC13P091546 | 12.67134666 |
| DPH1 | Diphthamide Biosynthesis 1 | Protein Coding | 36 | GC17P002030 | 12.61865616 |
| PSG2 | Pregnancy Specific Beta-1-Glycoprotein 2 | Protein Coding | 31 | GC19M043064 | 12.61271095 |
| CALB2 | Calbindin 2 | Protein Coding | 36 | GC16P071358 | 12.5868721 |
| TUSC3 | Tumor Suppressor Candidate 3 | Protein Coding | 38 | GC08P015417 | 12.58126926 |
| PEBP1 | Phosphatidylethanolamine Binding Protein 1 | Protein Coding | 40 | GC12P118135 | 12.56643295 |
| SULF1 | Sulfatase 1 | Protein Coding | 38 | GC08P069466 | 12.55926895 |
| PHOX2B | Paired Like Homeobox 2B | Protein Coding | 39 | GC04M041746 | 12.55103588 |
| GATA1 | GATA Binding Protein 1 | Protein Coding | 42 | GC0XP048786 | 12.54952431 |
| MIR27B | MicroRNA 27b | RNA Gene | 20 | GC09P095097 | 12.53798866 |
| FOXC2-AS1 | FOXC2 Antisense RNA 1 | RNA Gene | 14 | GC16M086566 | 12.52828121 |
| TNFRSF1A | TNF Receptor Superfamily Member 1A | Protein Coding | 43 | GC12M006328 | 12.52678108 |
| OR3A4P | Olfactory Receptor Family 3 Subfamily A Member 4 Pseudogene | Pseudogene | 17 | GC17P004216 | 12.50252247 |
| MAGI2-AS3 | MAGI2 Antisense RNA 3 | RNA Gene | 16 | GC07P079452 | 12.50218201 |
| SLC30A2 | Solute Carrier Family 30 Member 2 | Protein Coding | 38 | GC01M026048 | 12.50208092 |
| TTK | TTK Protein Kinase | Protein Coding | 42 | GC06P080003 | 12.48594666 |
| FEZF1 | FEZ Family Zinc Finger 1 | Protein Coding | 36 | GC07M122301 | 12.48482513 |
| COL18A1 | Collagen Type XVIII Alpha 1 Chain | Protein Coding | 42 | GC21P045405 | 12.46337318 |
| IL24 | Interleukin 24 | Protein Coding | 36 | GC01P206897 | 12.45887375 |
| MIRLET7F2 | MicroRNA Let-7f-2 | RNA Gene | 17 | GC0XM053632 | 12.45741653 |
| ITGA2 | Integrin Subunit Alpha 2 | Protein Coding | 42 | GC05P052989 | 12.45201015 |
| THPO | Thrombopoietin | Protein Coding | 41 | GC03M184371 | 12.4462204 |
| LPP | LIM Domain Containing Preferred Translocation Partner In Lipoma | Protein Coding | 39 | GC03P188153 | 12.43464756 |
| LINC00958 | Long Intergenic Non-Protein Coding RNA 958 | RNA Gene | 16 | GC11M012877 | 12.42680931 |
| CLDND1 | Claudin Domain Containing 1 | Protein Coding | 34 | GC03M098497 | 12.42365456 |
| TGM2 | Transglutaminase 2 | Protein Coding | 43 | GC20M038127 | 12.41977596 |
| DSP | Desmoplakin | Protein Coding | 45 | GC06P007541 | 12.40049744 |
| E2F4 | E2F Transcription Factor 4 | Protein Coding | 40 | GC16P067192 | 12.39485359 |
| TWIST2 | Twist Family BHLH Transcription Factor 2 | Protein Coding | 38 | GC02P238848 | 12.38992882 |
| SASH1 | SAM And SH3 Domain Containing 1 | Protein Coding | 36 | GC06P148193 | 12.3885107 |
| ERVH48-1 | Endogenous Retrovirus Group 48 Member 1 | Protein Coding | 14 | GC21M042916 | 12.34320736 |
| TRPS1 | Transcriptional Repressor GATA Binding 1 | Protein Coding | 42 | GC08M115408 | 12.33615208 |
| LINC00993 | Long Intergenic Non-Protein Coding RNA 993 | RNA Gene | 13 | GC10P037249 | 12.31470871 |
| CT45A1 | Cancer/Testis Antigen Family 45 Member A1 | Protein Coding | 25 | GC0XP135713 | 12.2712841 |
| LINC00574 | Long Intergenic Non-Protein Coding RNA 574 | RNA Gene | 18 | GC06P169790 | 12.26000786 |
| MIR324 | MicroRNA 324 | RNA Gene | 19 | GC17M007223 | 12.25569916 |
| DMBT1 | Deleted In Malignant Brain Tumors 1 | Protein Coding | 36 | GC10P122560 | 12.25550079 |
| HLA-DQA1 | Major Histocompatibility Complex, Class II, DQ Alpha 1 | Protein Coding | 39 | GC06P080389 | 12.25419807 |
| TMPRSS2 | Transmembrane Serine Protease 2 | Protein Coding | 42 | GC21M041464 | 12.23864555 |
| IGF2BP3 | Insulin Like Growth Factor 2 MRNA Binding Protein 3 | Protein Coding | 37 | GC07M023316 | 12.23616219 |
| BLCAP | BLCAP Apoptosis Inducing Factor | Protein Coding | 30 | GC20M037492 | 12.23604393 |
| SNHG1 | Small Nucleolar RNA Host Gene 1 | RNA Gene | 20 | GC11M087140 | 12.22555733 |
| LINC00160 | Long Intergenic Non-Protein Coding RNA 160 | RNA Gene | 15 | GC21M034723 | 12.21450806 |
| SSX2 | SSX Family Member 2 | Protein Coding | 32 | GC0XM052696 | 12.18889618 |
| PRKAA1 | Protein Kinase AMP-Activated Catalytic Subunit Alpha 1 | Protein Coding | 42 | GC05M040759 | 12.1858139 |
| F2R | Coagulation Factor II Thrombin Receptor | Protein Coding | 42 | GC05P076716 | 12.13517857 |
| EXT2 | Exostosin Glycosyltransferase 2 | Protein Coding | 41 | GC11P044095 | 12.12925243 |
| VTN | Vitronectin | Protein Coding | 39 | GC17M034571 | 12.12610435 |
| MIR338 | MicroRNA 338 | RNA Gene | 17 | GC17M081126 | 12.10579681 |
| MANCR | Mitotically Associated Long Non Coding RNA | RNA Gene | 14 | GC10M004577 | 12.10517883 |

| | | | | | |
|---|---|---|---|---|---|
| YWHAE | Tyrosine 3-Monooxygenase/Tryptophan 5-Monooxygenase Activation Protein Epsilon | Protein Coding | 44 | GC17M002805 | 12.10151863 |
| TJP1 | Tight Junction Protein 1 | Protein Coding | 39 | GC15M029699 | 12.10029984 |
| DAB2IP | DAB2 Interacting Protein | Protein Coding | 36 | GC09P121566 | 12.09435272 |
| MIR370 | MicroRNA 370 | RNA Gene | 20 | GC14P109205 | 12.08888435 |
| EFNA3 | Ephrin A3 | Protein Coding | 40 | GC01P155078 | 12.0854435 |
| IL6R | Interleukin 6 Receptor | Protein Coding | 43 | GC01P154405 | 12.08146381 |
| SERPINB2 | Serpin Family B Member 2 | Protein Coding | 39 | GC18P063871 | 12.06433105 |
| SCAI | Suppressor Of Cancer Cell Invasion | Protein Coding | 30 | GC09M124942 | 12.06031322 |
| GPR68 | G Protein-Coupled Receptor 68 | Protein Coding | 39 | GC14M091232 | 12.04379177 |
| INHA | Inhibin Subunit Alpha | Protein Coding | 38 | GC02P219569 | 12.03967476 |
| CLDN3 | Claudin 3 | Protein Coding | 36 | GC07M073768 | 12.03190517 |
| ING3 | Inhibitor Of Growth Family Member 3 | Protein Coding | 35 | GC07P120950 | 12.02739525 |
| AMACR | Alpha-Methylacyl-CoA Racemase | Protein Coding | 42 | GC05M033986 | 12.02719498 |
| IL4R | Interleukin 4 Receptor | Protein Coding | 43 | GC16P027823 | 12.02694225 |
| PTGER4 | Prostaglandin E Receptor 4 | Protein Coding | 42 | GC05P040679 | 12.02544594 |
| TCHP | Trichoplein Keratin Filament Binding | Protein Coding | 31 | GC12P109900 | 12.00960732 |
| CD9 | CD9 Molecule | Protein Coding | 38 | GC12P019798 | 12.0072422 |
| STAT6 | Signal Transducer And Activator Of Transcription 6 | Protein Coding | 45 | GC12M057095 | 12.00217152 |
| PBRM1 | Polybromo 1 | Protein Coding | 42 | GC03M052545 | 11.99911022 |
| CT47A1 | Cancer/Testis Antigen Family 47 Member A1 | Protein Coding | 20 | GC0XM120982 | 11.99689007 |
| CT47A11 | Cancer/Testis Antigen Family 47 Member A11 | Protein Coding | 19 | GC0XM120933 | 11.99689007 |
| CT47A2 | Cancer/Testis Antigen Family 47 Member A2 | Protein Coding | 18 | GC0XM120977 | 11.99689007 |
| CT47A3 | Cancer/Testis Antigen Family 47 Member A3 | Protein Coding | 18 | GC0XM120973 | 11.99689007 |
| CT47A4 | Cancer/Testis Antigen Family 47 Member A4 | Protein Coding | 17 | GC0XM120967 | 11.99689007 |
| DCK | Deoxycytidine Kinase | Protein Coding | 39 | GC04P070992 | 11.99345112 |
| FOXCUT | FOXC1 Upstream Transcript | RNA Gene | 13 | GC06P001604 | 11.98203468 |
| MIR2052HG | MIR2052 Host Gene | RNA Gene | 13 | GC08P074599 | 11.96315002 |
| WNT2B | Wnt Family Member 2B | Protein Coding | 39 | GC01P112466 | 11.96142769 |
| IL18 | Interleukin 18 | Protein Coding | 38 | GC11M112143 | 11.95485783 |
| LINC01016 | Long Intergenic Non-Protein Coding RNA 1016 | RNA Gene | 13 | GC06M064509 | 11.9448719 |
| ACTA2 | Actin Alpha 2, Smooth Muscle | Protein Coding | 42 | GC10M088935 | 11.92643356 |
| LINC00339 | Long Intergenic Non-Protein Coding RNA 339 | RNA Gene | 19 | GC01P022291 | 11.90677166 |
| CXCL14 | C-X-C Motif Chemokine Ligand 14 | Protein Coding | 33 | GC05M135617 | 11.90055656 |
| BLACAT1 | BLACAT1 Overlapping LEMD1 Locus | Protein Coding | 13 | GC01M205434 | 11.89445972 |
| HP | Haptoglobin | Protein Coding | 41 | GC16P072089 | 11.89039707 |
| MELK | Maternal Embryonic Leucine Zipper Kinase | Protein Coding | 42 | GC09P036572 | 11.88916111 |
| APC2 | APC Regulator Of WNT Signaling Pathway 2 | Protein Coding | 35 | GC19P002601 | 11.87971401 |
| HAVCR2 | Hepatitis A Virus Cellular Receptor 2 | Protein Coding | 40 | GC05M157063 | 11.87728691 |
| MT-ND4L | Mitochondrially Encoded NADH:Ubiquinone Oxidoreductase Core Subunit 4L | Protein Coding | 27 | GCMTP010472 | 11.87348461 |
| ANGPT1 | Angiopoietin 1 | Protein Coding | 41 | GC08M107246 | 11.86969757 |
| AGER | Advanced Glycosylation End-Product Specific Receptor | Protein Coding | 40 | GC06M032180 | 11.85542107 |
| RACK1 | Receptor For Activated C Kinase 1 | Protein Coding | 35 | GC05M181718 | 11.84411049 |
| LINC00520 | Long Intergenic Non-Protein Coding RNA 520 | RNA Gene | 17 | GC14M055781 | 11.83802509 |
| LCAL1 | Lung Cancer Associated LncRNA 1 | RNA Gene | 13 | GC06M079274 | 11.83492279 |
| GCRG224 | Gastric Cancer-Related Gene GCRG224 | RNA Gene | 5 | GC11U900303 | 11.82816124 |
| GNB1 | G Protein Subunit Beta 1 | Protein Coding | 40 | GC01M001785 | 11.81784916 |
| RTEL1 | Regulator Of Telomere Elongation Helicase 1 | Protein Coding | 36 | GC20P063658 | 11.80848312 |
| SLC19A1 | Solute Carrier Family 19 Member 1 | Protein Coding | 42 | GC21M045493 | 11.80480862 |
| BGLAP | Bone Gamma-Carboxyglutamate Protein | Protein Coding | 38 | GC01P156242 | 11.79110336 |
| SUZ12 | SUZ12 Polycomb Repressive Complex 2 Subunit | Protein Coding | 36 | GC17P031937 | 11.78575516 |
| FGF14-AS2 | FGF14 Antisense RNA 2 | RNA Gene | 13 | GC13P102394 | 11.76859474 |
| CDH17 | Cadherin 17 | Protein Coding | 38 | GC08M094127 | 11.7575922 |
| LINC01671 | Long Intergenic Non-Protein Coding RNA 1671 | RNA Gene | 11 | GC21M042579 | 11.75360489 |
| WFDC2 | WAP Four-Disulfide Core Domain 2 | Protein Coding | 34 | GC20P045469 | 11.74337578 |
| ICOSLG | Inducible T Cell Costimulator Ligand | Protein Coding | 36 | GC21M044222 | 11.73582363 |
| SLC5A8 | Solute Carrier Family 5 Member 8 | Protein Coding | 38 | GC12M101155 | 11.72155952 |
| LINC00598 | Long Intergenic Non-Protein Coding RNA 598 | RNA Gene | 16 | GC13M040101 | 11.71750641 |
| ACP3 | Acid Phosphatase 3 | Protein Coding | 34 | GC03P133888 | 11.71273899 |
| KLRK1 | Killer Cell Lectin Like Receptor K1 | Protein Coding | 36 | GC12M020442 | 11.71116638 |
| LINC01089 | Long Intergenic Non-Protein Coding RNA 1089 | RNA Gene | 15 | GC12M122153 | 11.71029854 |
| LINC02099 | Long Intergenic Non-Protein Coding RNA 2099 | RNA Gene | 13 | GC08P029749 | 11.70664024 |
| TFRC | Transferrin Receptor | Protein Coding | 43 | GC03M196027 | 11.69912243 |
| RRM1 | Ribonucleotide Reductase Catalytic Subunit M1 | Protein Coding | 43 | GC11P004115 | 11.69363022 |
| REV3L | REV3 Like, DNA Directed Polymerase Zeta Catalytic Subunit | Protein Coding | 40 | GC06M111299 | 11.68137074 |
| IRF4 | Interferon Regulatory Factor 4 | Protein Coding | 39 | GC06P000391 | 11.68078804 |
| ACE | Angiotensin I Converting Enzyme | Protein Coding | 46 | GC17P063477 | 11.67822075 |
| PPP2R2A | Protein Phosphatase 2 Regulatory Subunit Balpha | Protein Coding | 39 | GC08P026292 | 11.65181065 |
| TNFRSF10D | TNF Receptor Superfamily Member 10d | Protein Coding | 37 | GC08M023135 | 11.62259769 |
| MPL | MPL Proto-Oncogene, Thrombopoietin Receptor | Protein Coding | 43 | GC01P043337 | 11.61585426 |
| NUMA1 | Nuclear Mitotic Apparatus Protein 1 | Protein Coding | 39 | GC11M072002 | 11.61077213 |
| PEG10 | Paternally Expressed 10 | Protein Coding | 36 | GC07P094656 | 11.60687256 |
| PTPN13 | Protein Tyrosine Phosphatase Non-Receptor Type 13 | Protein Coding | 39 | GC04P086594 | 11.59993744 |
| ST8SIA6-AS1 | ST8SIA6 Antisense RNA 1 | RNA Gene | 15 | GC10P017386 | 11.59401321 |
| NCOA4 | Nuclear Receptor Coactivator 4 | Protein Coding | 35 | GC10M046005 | 11.59100056 |
| BNIP3 | BCL2 Interacting Protein 3 | Protein Coding | 36 | GC10M131966 | 11.57838821 |
| LAMC2 | Laminin Subunit Gamma 2 | Protein Coding | 42 | GC01P183186 | 11.57792473 |
| GRPR | Gastrin Releasing Peptide Receptor | Protein Coding | 41 | GC0XP016141 | 11.57540798 |
| CIP2A | Cellular Inhibitor Of PP2A | Protein Coding | 28 | GC03M108545 | 11.573843 |
| NTS | Neurotensin | Protein Coding | 36 | GC12P085876 | 11.57373905 |
| CT47A12 | Cancer/Testis Antigen Family 47 Member A12 | Protein Coding | 17 | GC0XM120878 | 11.5658102 |
| WNT7A | Wnt Family Member 7A | Protein Coding | 44 | GC03M020205 | 11.55428314 |
| ABCC3 | ATP Binding Cassette Subfamily C Member 3 | Protein Coding | 42 | GC17P050634 | 11.54902649 |
| CD8A | CD8a Molecule | Protein Coding | 41 | GC02M086784 | 11.54111862 |
| MCM3 | Minichromosome Maintenance Complex Component 3 | Protein Coding | 40 | GC06M052264 | 11.52628517 |
| MIR132 | MicroRNA 132 | RNA Gene | 21 | GC17M002049 | 11.5158596 |
| TMEFF2 | Transmembrane Protein With EGF Like And Two Follistatin Like Domains 2 | Protein Coding | 35 | GC02M191950 | 11.51202202 |
| BRD4 | Bromodomain Containing 4 | Protein Coding | 43 | GC19M015236 | 11.50477028 |
| RASSF1-AS1 | RASSF1 Antisense RNA 1 | RNA Gene | 13 | GC03P050337 | 11.50370789 |
| MYCL | MYCL Proto-Oncogene, BHLH Transcription Factor | Protein Coding | 33 | GC01M039895 | 11.50231743 |
| BRINP1 | BMP/Retinoic Acid Inducible Neural Specific 1 | Protein Coding | 29 | GC09M119153 | 11.50191307 |
| XAGE1B | X Antigen Family Member 1B | Protein Coding | 23 | GC0XM052513 | 11.50107384 |
| POLR1C | RNA Polymerase I And III Subunit C | Protein Coding | 38 | GC06P080601 | 11.50085258 |
| SAT1 | Spermidine/Spermine N1-Acetyltransferase 1 | Protein Coding | 39 | GC0XP023784 | 11.48377037 |
| FLI1 | Fli-1 Proto-Oncogene, ETS Transcription Factor | Protein Coding | 43 | GC11P128686 | 11.48249912 |

| | | | | | |
|---|---|---|---|---|---|
| MEFV | MEFV Innate Immuity Regulator, Pyrin | Protein Coding | 39 | GC16M006828 | 11.48224258 |
| RMRP | RNA Component Of Mitochondrial RNA Processing Endoribonuclease | RNA Gene | 25 | GC09M035655 | 11.47088432 |
| ASXL1 | ASXL Transcriptional Regulator 1 | Protein Coding | 39 | GC20P032619 | 11.46371651 |
| HMMR-AS1 | HMMR Antisense RNA 1 | RNA Gene | 13 | GC05M163482 | 11.44519329 |
| PTPRG-AS1 | PTPRG Antisense RNA 1 | RNA Gene | 15 | GC03M062248 | 11.43828583 |
| PRKCE | Protein Kinase C Epsilon | Protein Coding | 43 | GC02P045651 | 11.42713547 |
| CD40LG | CD40 Ligand | Protein Coding | 43 | GC0XP136649 | 11.41770172 |
| GACAT1 | Gastric Cancer Associated Transcript 1 | RNA Gene | 11 | GC02P107754 | 11.40932941 |
| ATF3 | Activating Transcription Factor 3 | Protein Coding | 39 | GC01P212565 | 11.40731907 |
| S100A9 | S100 Calcium Binding Protein A9 | Protein Coding | 38 | GC01P153357 | 11.39629555 |
| SELENBP1 | Selenium Binding Protein 1 | Protein Coding | 39 | GC01M151364 | 11.38510132 |
| DPP4 | Dipeptidyl Peptidase 4 | Protein Coding | 45 | GC02M161992 | 11.36928272 |
| SLX4 | SLX4 Structure-Specific Endonuclease Subunit | Protein Coding | 33 | GC16M006853 | 11.35855675 |
| HSD3B2 | Hydroxy-Delta-5-Steroid Dehydrogenase, 3 Beta- And Steroid Delta-Isomerase 2 | Protein Coding | 42 | GC01P119414 | 11.35796356 |
| SELE | Selectin E | Protein Coding | 40 | GC01M169722 | 11.35318279 |
| SOCS1 | Suppressor Of Cytokine Signaling 1 | Protein Coding | 39 | GC16M011896 | 11.34368038 |
| COL7A1 | Collagen Type VII Alpha 1 Chain | Protein Coding | 40 | GC03M048564 | 11.30991364 |
| KCNK15-AS1 | KCNK15 And WISP2 Antisense RNA 1 | RNA Gene | 14 | GC20M044694 | 11.29810333 |
| FUT3 | Fucosyltransferase 3 (Lewis Blood Group) | Protein Coding | 38 | GC19M005843 | 11.29724693 |
| CEACAM6 | CEA Cell Adhesion Molecule 6 | Protein Coding | 38 | GC19P041750 | 11.29466152 |
| STXBP5-AS1 | STXBP5 Antisense RNA 1 | RNA Gene | 16 | GC06M146841 | 11.29236603 |
| IGKV@ | Immunoglobulin Kappa Variable Cluster | Gene Cluster | 7 | GC02U990059 | 11.29060173 |
| GSTM3 | Glutathione S-Transferase Mu 3 | Protein Coding | 42 | GC01M109733 | 11.29001236 |
| CT47A10 | Cancer/Testis Antigen Family 47 Member A10 | Protein Coding | 17 | GC0XM120938 | 11.2884779 |
| RASGRP1 | RAS Guanyl Releasing Protein 1 | Protein Coding | 43 | GC15M038488 | 11.28076744 |
| ADAM12 | ADAM Metallopeptidase Domain 12 | Protein Coding | 42 | GC10M126012 | 11.26085949 |
| IL15 | Interleukin 15 | Protein Coding | 38 | GC04P141636 | 11.25342178 |
| XAGE1A | X Antigen Family Member 1A | Protein Coding | 22 | GC0XP052496 | 11.23593712 |
| TNFRSF8 | TNF Receptor Superfamily Member 8 | Protein Coding | 38 | GC01P012063 | 11.23283482 |
| GZMB | Granzyme B | Protein Coding | 42 | GC14M024630 | 11.23042202 |
| CD79A | CD79a Molecule | Protein Coding | 42 | GC19P041877 | 11.2289772 |
| HSPD1 | Heat Shock Protein Family D (Hsp60) Member 1 | Protein Coding | 42 | GC02M197486 | 11.22833633 |
| CDH23 | Cadherin Related 23 | Protein Coding | 41 | GC10P071396 | 11.2275753 |
| TNFRSF10C | TNF Receptor Superfamily Member 10c | Protein Coding | 36 | GC08P023102 | 11.21905708 |
| CHRNA5 | Cholinergic Receptor Nicotinic Alpha 5 Subunit | Protein Coding | 39 | GC15P078565 | 11.21387291 |
| PMAIP1 | Phorbol-12-Myristate-13-Acetate-Induced Protein 1 | Protein Coding | 37 | GC18P059899 | 11.1931057 |
| NECTIN4 | Nectin Cell Adhesion Molecule 4 | Protein Coding | 38 | GC01M161071 | 11.17905045 |
| ACD | ACD Shelterin Complex Subunit And Telomerase Recruitment Factor | Protein Coding | 39 | GC16M067658 | 11.17371941 |
| LZTR1 | Leucine Zipper Like Transcription Regulator 1 | Protein Coding | 39 | GC22P034492 | 11.17369843 |
| RAD23B | RAD23 Homolog B, Nucleotide Excision Repair Protein | Protein Coding | 40 | GC09P107283 | 11.17202854 |
| MIR361 | MicroRNA 361 | RNA Gene | 17 | GC0XM085903 | 11.16649914 |
| SDC1 | Syndecan 1 | Protein Coding | 38 | GC02M020200 | 11.16090202 |
| RXRA | Retinoid X Receptor Alpha | Protein Coding | 44 | GC09P134317 | 11.15608311 |
| KLK2 | Kallikrein Related Peptidase 2 | Protein Coding | 40 | GC19P050861 | 11.15271187 |
| NAMPT | Nicotinamide Phosphoribosyltransferase | Protein Coding | 42 | GC07M106248 | 11.15127468 |
| ALPP | Alkaline Phosphatase, Placental | Protein Coding | 40 | GC02P232378 | 11.14883804 |
| LINC00636 | Long Intergenic Non-Protein Coding RNA 636 | RNA Gene | 16 | GC03P107883 | 11.1405983 |
| SLC5A5 | Solute Carrier Family 5 Member 5 | Protein Coding | 41 | GC19P063114 | 11.11849785 |
| UCHL1 | Ubiquitin C-Terminal Hydrolase L1 | Protein Coding | 46 | GC04P041256 | 11.11829567 |
| PINK1-AS | PINK1 Antisense RNA | RNA Gene | 13 | GC01M020642 | 11.095047 |
| TFF3 | Trefoil Factor 3 | Protein Coding | 36 | GC21M042311 | 11.09024906 |
| CCNA1 | Cyclin A1 | Protein Coding | 38 | GC13P036431 | 11.08589363 |
| LINC02130 | Long Intergenic Non-Protein Coding RNA 2130 | RNA Gene | 10 | GC16M014365 | 11.08538437 |
| NBR2 | Neighbor Of BRCA1 LncRNA 2 | RNA Gene | 23 | GC17P043125 | 11.07209492 |
| ADARB2-AS1 | ADARB2 Antisense RNA 1 | RNA Gene | 18 | GC10P001526 | 11.05913353 |
| PTPN1 | Protein Tyrosine Phosphatase Non-Receptor Type 1 | Protein Coding | 45 | GC20P050510 | 11.04842758 |
| FANCB | FA Complementation Group B | Protein Coding | 36 | GC0XM014690 | 11.04742241 |
| MIR376A1 | MicroRNA 376a-1 | RNA Gene | 15 | GC14P109523 | 11.04189301 |
| PNPLA6 | Patatin Like Phospholipase Domain Containing 6 | Protein Coding | 40 | GC19P007534 | 11.03908539 |
| UGT1A9 | UDP Glucuronosyltransferase Family 1 Member A9 | Protein Coding | 41 | GC02P233671 | 11.03643322 |
| FCGR2A | Fc Gamma Receptor IIa | Protein Coding | 42 | GC01P161505 | 11.03387642 |
| EPO | Erythropoietin | Protein Coding | 39 | GC07P100720 | 11.0190506 |
| PDE11A | Phosphodiesterase 11A | Protein Coding | 42 | GC02M177624 | 11.01567554 |
| LINC01405 | Long Intergenic Non-Protein Coding RNA 1405 | RNA Gene | 14 | GC12P110936 | 11.0124836 |
| KLF3-AS1 | KLF3 Antisense RNA 1 | RNA Gene | 15 | GC04M038594 | 10.99450874 |
| PDCD4-AS1 | PDCD4 Antisense RNA 1 | RNA Gene | 15 | GC10M110868 | 10.99450874 |
| PKD1P6-NPIPP1 | PKD1P6-NPIPP1 Readthrough | Pseudogene | 7 | GC16M015629 | 10.99450874 |
| STUB1 | STIP1 Homology And U-Box Containing Protein 1 | Protein Coding | 40 | GC16P010649 | 10.98151398 |
| STK4 | Serine/Threonine Kinase 4 | Protein Coding | 44 | GC20P044966 | 10.97748089 |
| PRKCI | Protein Kinase C Iota | Protein Coding | 43 | GC03P170222 | 10.9738903 |
| DLK1 | Delta Like Non-Canonical Notch Ligand 1 | Protein Coding | 39 | GC14P109323 | 10.97360516 |
| G6PD | Glucose-6-Phosphate Dehydrogenase | Protein Coding | 44 | GC0XM154531 | 10.97328377 |
| HAGLR | HOXD Antisense Growth-Associated Long Non-Coding RNA | RNA Gene | 16 | GC02M176173 | 10.97290039 |
| GHET1 | Gastric Carcinoma Proliferation Enhancing Transcript 1 | RNA Gene | 12 | GC07P149226 | 10.96163082 |
| COL14A1 | Collagen Type XIV Alpha 1 Chain | Protein Coding | 39 | GC08P120101 | 10.95485497 |
| EED | Embryonic Ectoderm Development | Protein Coding | 38 | GC11P086244 | 10.93264866 |
| MIR377 | MicroRNA 377 | RNA Gene | 18 | GC14P109527 | 10.91257286 |
| CEBPA | CCAAT Enhancer Binding Protein Alpha | Protein Coding | 42 | GC19M033299 | 10.90619183 |
| CCR5 | C-C Motif Chemokine Receptor 5 | Protein Coding | 43 | GC03P046460 | 10.89753151 |
| HSF1 | Heat Shock Transcription Factor 1 | Protein Coding | 40 | GC08P144291 | 10.89424419 |
| HPN | Hepsin | Protein Coding | 40 | GC19P035040 | 10.89377308 |
| CDT1 | Chromatin Licensing And DNA Replication Factor 1 | Protein Coding | 39 | GC16P088803 | 10.88372803 |
| ALOX12 | Arachidonate 12-Lipoxygenase, 12S Type | Protein Coding | 40 | GC17P006995 | 10.87243843 |
| ANTXR1 | ANTXR Cell Adhesion Molecule 1 | Protein Coding | 41 | GC02P068977 | 10.86787415 |
| CYP3A5 | Cytochrome P450 Family 3 Subfamily A Member 5 | Protein Coding | 42 | GC07M099648 | 10.86646938 |
| NDUFA13 | NADH:Ubiquinone Oxidoreductase Subunit A13 | Protein Coding | 39 | GC19P019515 | 10.86333752 |
| URI1 | URI1 Prefoldin Like Chaperone | Protein Coding | 34 | GC19P029923 | 10.86257267 |
| CYP27B1 | Cytochrome P450 Family 27 Subfamily B Member 1 | Protein Coding | 43 | GC12M057757 | 10.86166096 |
| CHRNA3 | Cholinergic Receptor Nicotinic Alpha 3 Subunit | Protein Coding | 42 | GC15M081612 | 10.85614586 |
| MIR212 | MicroRNA 212 | RNA Gene | 19 | GC17M002050 | 10.85304451 |
| PAX3 | Paired Box 3 | Protein Coding | 42 | GC02M222199 | 10.84366989 |
| HNF4A | Hepatocyte Nuclear Factor 4 Alpha | Protein Coding | 45 | GC20P044355 | 10.84228039 |
| CTSK | Cathepsin K | Protein Coding | 46 | GC01M151665 | 10.83043575 |
| FRGCA | FOXM1-Regulated, Gastric Cancer Associated | RNA Gene | 13 | GC21M043141 | 10.80983925 |
| S100P | S100 Calcium Binding Protein P | Protein Coding | 35 | GC04P006700 | 10.80376244 |

| | | | | | |
|---|---|---|---|---|---|
| KMT2A | Lysine Methyltransferase 2A | Protein Coding | 42 | GC11P118436 | 10.79390907 |
| PON1 | Paraoxonase 1 | Protein Coding | 43 | GC07M095297 | 10.79350853 |
| TSHR | Thyroid Stimulating Hormone Receptor | Protein Coding | 42 | GC14P080954 | 10.79029655 |
| JADRR | JADE1 Adjacent Regulatory RNA | RNA Gene | 12 | GC04P128768 | 10.78995705 |
| CT47B1 | Cancer/Testis Antigen Family 47 Member B1 | Protein Coding | 21 | GC0XM120872 | 10.77082157 |
| FSHB | Follicle Stimulating Hormone Subunit Beta | Protein Coding | 41 | GC11P030210 | 10.7568531 |
| BRCD1 | Cancer, Familial, With In Vitro Radioresistance | Genetic Locus | 2 | GC00U930878 | 10.74044228 |
| MIR330 | MicroRNA 330 | RNA Gene | 19 | GC19M063960 | 10.72936916 |
| EFNB1 | Ephrin B1 | Protein Coding | 40 | GC0XP068828 | 10.7289362 |
| SMARCA2 | SWI/SNF Related, Matrix Associated, Actin Dependent Regulator Of Chromatin, Subfamily A, Member 2 | Protein Coding | 45 | GC09P001980 | 10.72741795 |
| PLAG1 | PLAG1 Zinc Finger | Protein Coding | 37 | GC08M056262 | 10.72724724 |
| H3-3A | H3.3 Histone A | Protein Coding | 32 | GC01P226062 | 10.7068634 |
| MECP2 | Methyl-CpG Binding Protein 2 | Protein Coding | 40 | GC0XM154021 | 10.70056152 |
| SOD1 | Superoxide Dismutase 1 | Protein Coding | 46 | GC21P031659 | 10.68558502 |
| WNT10A | Wnt Family Member 10A | Protein Coding | 41 | GC02P218897 | 10.68076324 |
| HLA-C | Major Histocompatibility Complex, Class I, C | Protein Coding | 40 | GC06M063562 | 10.6640873 |
| BBC3 | BCL2 Binding Component 3 | Protein Coding | 35 | GC19M047220 | 10.66202164 |
| EEF1A1 | Eukaryotic Translation Elongation Factor 1 Alpha 1 | Protein Coding | 38 | GC06M073515 | 10.65463257 |
| CXCR5 | C-X-C Motif Chemokine Receptor 5 | Protein Coding | 38 | GC11P118912 | 10.65219975 |
| CYP2C19 | Cytochrome P450 Family 2 Subfamily C Member 19 | Protein Coding | 43 | GC10P094762 | 10.64422035 |
| S100B | S100 Calcium Binding Protein B | Protein Coding | 41 | GC21M050462 | 10.63768768 |
| DES | Desmin | Protein Coding | 42 | GC02P219418 | 10.63216972 |
| MIR489 | MicroRNA 489 | RNA Gene | 17 | GC07M093483 | 10.63026619 |
| TRAF6 | TNF Receptor Associated Factor 6 | Protein Coding | 41 | GC11M036467 | 10.62987328 |
| CTBP1 | C-Terminal Binding Protein 1 | Protein Coding | 43 | GC04M001211 | 10.6293335 |
| SIX1 | SIX Homeobox 1 | Protein Coding | 38 | GC14M060643 | 10.60629272 |
| KLK7 | Kallikrein Related Peptidase 7 | Protein Coding | 37 | GC19M064219 | 10.58427429 |
| TGIF1 | TGFB Induced Factor Homeobox 1 | Protein Coding | 40 | GC18P003411 | 10.56820107 |
| PRMT1 | Protein Arginine Methyltransferase 1 | Protein Coding | 42 | GC19P049675 | 10.56499672 |
| LIF | LIF Interleukin 6 Family Cytokine | Protein Coding | 37 | GC22M030240 | 10.55941963 |
| MST1 | Macrophage Stimulating 1 | Protein Coding | 42 | GC03M049683 | 10.55604553 |
| BUB3 | BUB3 Mitotic Checkpoint Protein | Protein Coding | 42 | GC10P123154 | 10.54922295 |
| CDC45 | Cell Division Cycle 45 | Protein Coding | 39 | GC22P019479 | 10.54558945 |
| KLK15 | Kallikrein Related Peptidase 15 | Protein Coding | 38 | GC19M050825 | 10.53915215 |
| SPINT2 | Serine Peptidase Inhibitor, Kunitz Type 2 | Protein Coding | 39 | GC19P038244 | 10.53481197 |
| GLI3 | GLI Family Zinc Finger 3 | Protein Coding | 43 | GC07M041960 | 10.52157211 |
| F2 | Coagulation Factor II, Thrombin | Protein Coding | 45 | GC11P046720 | 10.52120399 |
| NUP214 | Nucleoporin 214 | Protein Coding | 40 | GC09P131125 | 10.51358604 |
| MIR455 | MicroRNA 455 | RNA Gene | 18 | GC09P114209 | 10.51231766 |
| PROKR2 | Prokineticin Receptor 2 | Protein Coding | 40 | GC20M005398 | 10.49474335 |
| RHEB | Ras Homolog, MTORC1 Binding | Protein Coding | 43 | GC07M151466 | 10.49046993 |
| BANCR | BRAF-Activated Non-Protein Coding RNA | RNA Gene | 14 | GC09M069296 | 10.48083115 |
| VWF | Von Willebrand Factor | Protein Coding | 44 | GC12M005917 | 10.4691143 |
| SLC45A3 | Solute Carrier Family 45 Member 3 | Protein Coding | 35 | GC01M205626 | 10.46377373 |
| PTH | Parathyroid Hormone | Protein Coding | 41 | GC11M013492 | 10.45938015 |
| RPL5 | Ribosomal Protein L5 | Protein Coding | 42 | GC01P092832 | 10.44496441 |
| BRDT | Bromodomain Testis Associated | Protein Coding | 39 | GC01P091949 | 10.43723774 |
| HNF1A-AS1 | HNF1A Antisense RNA 1 | RNA Gene | 18 | GC12M122123 | 10.42777252 |
| ANPEP | Alanyl Aminopeptidase, Membrane | Protein Coding | 45 | GC15M089784 | 10.41776466 |
| CXCR1 | C-X-C Motif Chemokine Receptor 1 | Protein Coding | 40 | GC02M218162 | 10.40941048 |
| ARID1B | AT-Rich Interaction Domain 1B | Protein Coding | 39 | GC06P156777 | 10.40667152 |
| LTA | Lymphotoxin Alpha | Protein Coding | 39 | GC06P080351 | 10.40600395 |
| VTCN1 | V-Set Domain Containing T Cell Activation Inhibitor 1 | Protein Coding | 36 | GC01M117143 | 10.40581703 |
| CCL20 | C-C Motif Chemokine Ligand 20 | Protein Coding | 38 | GC02P227838 | 10.40441036 |
| TINCR | TINCR Ubiquitin Domain Containing | Protein Coding | 21 | GC19M005558 | 10.39974022 |
| SEPTIN9 | Septin 9 | Protein Coding | 34 | GC17P077282 | 10.37986946 |
| TRB | T Cell Receptor Beta Locus | Protein Coding | 18 | GC07P148232 | 10.35383606 |
| JAK3 | Janus Kinase 3 | Protein Coding | 46 | GC19M017824 | 10.35362053 |
| AMH | Anti-Mullerian Hormone | Protein Coding | 40 | GC19P002659 | 10.3523283 |
| EIF2AK2 | Eukaryotic Translation Initiation Factor 2 Alpha Kinase 2 | Protein Coding | 42 | GC02M037099 | 10.34971428 |
| RCVRN | Recoverin | Protein Coding | 34 | GC17M009896 | 10.34880924 |
| DDX5 | DEAD-Box Helicase 5 | Protein Coding | 41 | GC17M064498 | 10.33832932 |
| SRARP | Steroid Receptor Associated And Regulated Protein | Protein Coding | 25 | GC01P016017 | 10.32624435 |
| MMP12 | Matrix Metallopeptidase 12 | Protein Coding | 42 | GC11M102862 | 10.31071568 |
| LAMB3 | Laminin Subunit Beta 3 | Protein Coding | 41 | GC01M209614 | 10.30629158 |
| FENDRR | FOXF1 Adjacent Non-Coding Developmental Regulatory RNA | RNA Gene | 18 | GC16M086511 | 10.30206776 |
| MYOD1 | Myogenic Differentiation 1 | Protein Coding | 42 | GC11P017741 | 10.30164528 |
| MDK | Midkine | Protein Coding | 39 | GC11P046380 | 10.29126835 |
| CT47A6 | Cancer/Testis Antigen Family 47 Member A6 | Protein Coding | 18 | GC0XM120958 | 10.28496933 |
| CT47A7 | Cancer/Testis Antigen Family 47 Member A7 | Protein Coding | 17 | GC0XM120953 | 10.28496933 |
| CT47A8 | Cancer/Testis Antigen Family 47 Member A8 | Protein Coding | 16 | GC0XM120956 | 10.28496933 |
| CT47A9 | Cancer/Testis Antigen Family 47 Member A9 | Protein Coding | 16 | GC0XM120943 | 10.28496933 |
| MIR378A | MicroRNA 378a | RNA Gene | 19 | GC05P149732 | 10.28274345 |
| MIR133A1 | MicroRNA 133a-1 | RNA Gene | 17 | GC18M024066 | 10.27169228 |
| NDNF | Neuron Derived Neurotrophic Factor | Protein Coding | 32 | GC04M121029 | 10.26914883 |
| CDCP1 | CUB Domain Containing Protein 1 | Protein Coding | 35 | GC03M045082 | 10.26864529 |
| BIRC7 | Baculoviral IAP Repeat Containing 7 | Protein Coding | 37 | GC20P063235 | 10.26357937 |
| VIP | Vasoactive Intestinal Peptide | Protein Coding | 40 | GC06P152750 | 10.2586174 |
| KDM6A | Lysine Demethylase 6A | Protein Coding | 42 | GC0XP044873 | 10.25433826 |
| GJB2 | Gap Junction Protein Beta 2 | Protein Coding | 42 | GC13M020187 | 10.24948692 |
| LINC00673 | Long Intergenic Non-Protein Coding RNA 673 | RNA Gene | 16 | GC17M072290 | 10.24325752 |
| TRPM8 | Transient Receptor Potential Cation Channel Subfamily M Member 8 | Protein Coding | 40 | GC02P233917 | 10.24299908 |
| ANXA3 | Annexin A3 | Protein Coding | 38 | GC04P078551 | 10.23254585 |
| SPIN1 | Spindlin 1 | Protein Coding | 34 | GC09P088388 | 10.23174572 |
| AMHR2 | Anti-Mullerian Hormone Receptor Type 2 | Protein Coding | 43 | GC12P053423 | 10.22660255 |
| LDOC1 | LDOC1 Regulator Of NFKB Signaling | Protein Coding | 30 | GC0XM141111 | 10.22485924 |
| SLC29A1 | Solute Carrier Family 29 Member 1 (Augustine Blood Group) | Protein Coding | 43 | GC06P044219 | 10.22039986 |
| LTF | Lactotransferrin | Protein Coding | 40 | GC03M046435 | 10.21703911 |
| SATB1 | SATB Homeobox 1 | Protein Coding | 39 | GC03M020313 | 10.21182728 |
| MECOM | MDS1 And EVI1 Complex Locus | Protein Coding | 43 | GC03M169083 | 10.20923042 |
| AAGAB | Alpha And Gamma Adaptin Binding Protein | Protein Coding | 35 | GC15M067200 | 10.19901848 |
| RETN | Resistin | Protein Coding | 38 | GC19P007669 | 10.19530392 |
| PROK2 | Prokineticin 2 | Protein Coding | 39 | GC03M071771 | 10.1918087 |
| TLR3 | Toll Like Receptor 3 | Protein Coding | 46 | GC04P186059 | 10.18852806 |

| | | | | | |
|---|---|---|---|---|---|
| FST | Follistatin | Protein Coding | 42 | GC05P053480 | 10.18486404 |
| CHI3L1 | Chitinase 3 Like 1 | Protein Coding | 40 | GC01M203148 | 10.17993355 |
| SPINT1 | Serine Peptidase Inhibitor, Kunitz Type 1 | Protein Coding | 38 | GC15P040844 | 10.17933273 |
| KRT17 | Keratin 17 | Protein Coding | 40 | GC17M041619 | 10.17346859 |
| TK1 | Thymidine Kinase 1 | Protein Coding | 40 | GC17M078175 | 10.16794205 |
| CXCR3 | C-X-C Motif Chemokine Receptor 3 | Protein Coding | 39 | GC0XM071615 | 10.15562248 |
| DNAJC21 | DnaJ Heat Shock Protein Family (Hsp40) Member C21 | Protein Coding | 31 | GC05P034929 | 10.15156364 |
| LGALS3BP | Galectin 3 Binding Protein | Protein Coding | 38 | GC17M078971 | 10.12028599 |
| SREBF1 | Sterol Regulatory Element Binding Transcription Factor 1 | Protein Coding | 41 | GC17M017810 | 10.11593056 |
| VAV3 | Vav Guanine Nucleotide Exchange Factor 3 | Protein Coding | 39 | GC01M107571 | 10.1111908 |
| CD80 | CD80 Molecule | Protein Coding | 39 | GC03M119524 | 10.08664036 |
| PCM1 | Pericentriolar Material 1 | Protein Coding | 38 | GC08P017922 | 10.08517647 |
| MIR186 | MicroRNA 186 | RNA Gene | 19 | GC01M071067 | 10.08221054 |
| MIEN1 | Migration And Invasion Enhancer 1 | Protein Coding | 30 | GC17M039728 | 10.07753658 |
| MLLT10 | MLLT10 Histone Lysine Methyltransferase DOT1L Cofactor | Protein Coding | 37 | GC10P021524 | 10.0651722 |
| PIM1 | Pim-1 Proto-Oncogene, Serine/Threonine Kinase | Protein Coding | 44 | GC06P080542 | 10.06083012 |
| EXT1 | Exostosin Glycosyltransferase 1 | Protein Coding | 44 | GC08M117798 | 10.05168533 |
| JUND | JunD Proto-Oncogene, AP-1 Transcription Factor Subunit | Protein Coding | 38 | GC19M018279 | 10.04964542 |
| SFRP4 | Secreted Frizzled Related Protein 4 | Protein Coding | 39 | GC07M037912 | 10.03925228 |
| IFNB1 | Interferon Beta 1 | Protein Coding | 40 | GC09M021077 | 10.0385437 |
| MIR211 | MicroRNA 211 | RNA Gene | 19 | GC15M031065 | 10.03775501 |
| SOX3 | SRY-Box Transcription Factor 3 | Protein Coding | 40 | GC0XM140502 | 10.03391838 |
| TINF2 | TERF1 Interacting Nuclear Factor 2 | Protein Coding | 37 | GC14M024263 | 10.03002834 |
| MIR129-1 | MicroRNA 129-1 | RNA Gene | 18 | GC07P128207 | 10.02946854 |
| E2F5 | E2F Transcription Factor 5 | Protein Coding | 36 | GC08P085177 | 10.01382351 |
| GFAP | Glial Fibrillary Acidic Protein | Protein Coding | 42 | GC17M044905 | 10.00907707 |
| NKX3-1 | NK3 Homeobox 1 | Protein Coding | 36 | GC08M023678 | 10.00010967 |
| CT47A5 | Cancer/Testis Antigen Family 47 Member A5 | Protein Coding | 17 | GC0XM120963 | 9.997548103 |
| CEP57 | Centrosomal Protein 57 | Protein Coding | 38 | GC11P095789 | 9.99530983 |
| SIRT3 | Sirtuin 3 | Protein Coding | 42 | GC11M000215 | 9.991583824 |
| DKK3 | Dickkopf WNT Signaling Pathway Inhibitor 3 | Protein Coding | 36 | GC11M011962 | 9.982379913 |
| CBR3-AS1 | CBR3 Antisense RNA 1 | RNA Gene | 17 | GC21M036131 | 9.981344223 |
| KCTD1 | Potassium Channel Tetramerization Domain Containing 1 | Protein Coding | 36 | GC18M026454 | 9.976007462 |
| VCAM1 | Vascular Cell Adhesion Molecule 1 | Protein Coding | 40 | GC01P100719 | 9.971172333 |
| SNHG12 | Small Nucleolar RNA Host Gene 12 | RNA Gene | 22 | GC01M028578 | 9.970745087 |
| MIR345 | MicroRNA 345 | RNA Gene | 19 | GC14P100307 | 9.960330009 |
| PAX7 | Paired Box 7 | Protein Coding | 41 | GC01P018631 | 9.957790375 |
| SLPI | Secretory Leukocyte Peptidase Inhibitor | Protein Coding | 36 | GC20M045252 | 9.93667984 |
| HSPA1A | Heat Shock Protein Family A (Hsp70) Member 1A | Protein Coding | 40 | GC06P080375 | 9.936233521 |
| FEN1 | Flap Structure-Specific Endonuclease 1 | Protein Coding | 41 | GC11P061793 | 9.914281845 |
| TNFAIP3 | TNF Alpha Induced Protein 3 | Protein Coding | 43 | GC06P137866 | 9.91222477 |
| TTN | Titin | Protein Coding | 42 | GC02M178525 | 9.907056808 |
| TCF3 | Transcription Factor 3 | Protein Coding | 40 | GC19M001609 | 9.903303146 |
| IL17RD | Interleukin 17 Receptor D | Protein Coding | 39 | GC03M057089 | 9.892499924 |
| CYP11A1 | Cytochrome P450 Family 11 Subfamily A Member 1 | Protein Coding | 44 | GC15M074337 | 9.886548996 |
| LGR5 | Leucine Rich Repeat Containing G Protein-Coupled Receptor 5 | Protein Coding | 39 | GC12P071439 | 9.876918793 |
| HNRNPA2B1 | Heterogeneous Nuclear Ribonucleoprotein A2/B1 | Protein Coding | 41 | GC07M026174 | 9.871905327 |
| RIOX2 | Ribosomal Oxygenase 2 | Protein Coding | 30 | GC03M097942 | 9.855597496 |
| MIR151A | MicroRNA 151a | RNA Gene | 18 | GC08M140734 | 9.854032516 |
| UGT2B15 | UDP Glucuronosyltransferase Family 2 Member B15 | Protein Coding | 40 | GC04M068646 | 9.851693153 |
| TNFRSF6B | TNF Receptor Superfamily Member 6b | Protein Coding | 38 | GC20P063696 | 9.84562397 |
| RPS19 | Ribosomal Protein S19 | Protein Coding | 43 | GC19P063712 | 9.837179184 |
| RPL15 | Ribosomal Protein L15 | Protein Coding | 39 | GC03P023916 | 9.830208778 |
| ADAM17 | ADAM Metallopeptidase Domain 17 | Protein Coding | 46 | GC02M009488 | 9.826047897 |
| MIR184 | MicroRNA 184 | RNA Gene | 22 | GC15P079209 | 9.807071686 |
| ANOS1 | Anosmin 1 | Protein Coding | 32 | GC0XM008528 | 9.781079292 |
| DDX3X | DEAD-Box Helicase 3 X-Linked | Protein Coding | 41 | GC0XP041333 | 9.77253437 |
| GHRL | Ghrelin And Obestatin Prepropeptide | Protein Coding | 39 | GC03M010285 | 9.771641731 |
| ENPP2 | Ectonucleotide Pyrophosphatase/Phosphodiesterase 2 | Protein Coding | 40 | GC08M119556 | 9.762169838 |
| MIR216A | MicroRNA 216a | RNA Gene | 19 | GC02M055988 | 9.759872437 |
| UBE3A | Ubiquitin Protein Ligase E3A | Protein Coding | 42 | GC15M025333 | 9.753992081 |
| CDK12 | Cyclin Dependent Kinase 12 | Protein Coding | 39 | GC17P039461 | 9.75143528 |
| AZGP1 | Alpha-2-Glycoprotein 1, Zinc-Binding | Protein Coding | 40 | GC07M099967 | 9.746153831 |
| MLANA | Melan-A | Protein Coding | 35 | GC09P006430 | 9.73884201 |
| USP7 | Ubiquitin Specific Peptidase 7 | Protein Coding | 44 | GC16M008892 | 9.737298965 |
| DMRT1 | Doublesex And Mab-3 Related Transcription Factor 1 | Protein Coding | 38 | GC09P000831 | 9.728261948 |
| CEACAM7 | CEA Cell Adhesion Molecule 7 | Protein Coding | 34 | GC19M041673 | 9.72517395 |
| RPSA | Ribosomal Protein SA | Protein Coding | 40 | GC03P039406 | 9.715783119 |
| PTCH2 | Patched 2 | Protein Coding | 40 | GC01M044819 | 9.709133148 |
| LTO1 | LTO1 Maturation Factor Of ABCE1 | Protein Coding | 27 | GC11M087462 | 9.702992439 |
| CDK8 | Cyclin Dependent Kinase 8 | Protein Coding | 43 | GC13P026254 | 9.684274673 |
| DCN | Decorin | Protein Coding | 42 | GC12M091140 | 9.683216095 |
| ACP1 | Acid Phosphatase 1 | Protein Coding | 39 | GC02P000322 | 9.682781219 |
| PHLPP1 | PH Domain And Leucine Rich Repeat Protein Phosphatase 1 | Protein Coding | 34 | GC18P062715 | 9.679347038 |
| DDIT3 | DNA Damage Inducible Transcript 3 | Protein Coding | 40 | GC12M057516 | 9.67855835 |
| HJURP | Holliday Junction Recognition Protein | Protein Coding | 32 | GC02M233834 | 9.67787075 |
| SCGB2A1 | Secretoglobin Family 2A Member 1 | Protein Coding | 32 | GC11P062227 | 9.675074577 |
| TFPI2 | Tissue Factor Pathway Inhibitor 2 | Protein Coding | 38 | GC07M093885 | 9.664085388 |
| PPARGC1A | PPARG Coactivator 1 Alpha | Protein Coding | 41 | GC04M023755 | 9.656104088 |
| PGF | Placental Growth Factor | Protein Coding | 38 | GC14M074941 | 9.640915871 |
| PPARD | Peroxisome Proliferator Activated Receptor Delta | Protein Coding | 42 | GC06P080509 | 9.637161255 |
| USP9X | Ubiquitin Specific Peptidase 9 X-Linked | Protein Coding | 43 | GC0XP041085 | 9.613857269 |
| POU5F1B | POU Class 5 Homeobox 1B | Protein Coding | 31 | GC08P127322 | 9.603224754 |
| EPHA7 | EPH Receptor A7 | Protein Coding | 43 | GC06M093240 | 9.60114193 |
| MYBL1 | MYB Proto-Oncogene Like 1 | Protein Coding | 36 | GC08M066562 | 9.600944519 |
| CASP1 | Caspase 1 | Protein Coding | 44 | GC11M105025 | 9.599045753 |
| PRDX1 | Peroxiredoxin 1 | Protein Coding | 43 | GC01M045511 | 9.590107918 |
| LALBA | Lactalbumin Alpha | Protein Coding | 36 | GC12M048567 | 9.584739685 |
| P2RX7 | Purinergic Receptor P2X 7 | Protein Coding | 42 | GC12P125862 | 9.581642151 |
| H3C2 | H3 Clustered Histone 2 | Protein Coding | 28 | GC06M026032 | 9.580028534 |
| MIR138-1 | MicroRNA 138-1 | RNA Gene | 19 | GC03P044115 | 9.570368767 |
| KLK5 | Kallikrein Related Peptidase 5 | Protein Coding | 38 | GC19M050943 | 9.570217133 |
| TGFBR3 | Transforming Growth Factor Beta Receptor 3 | Protein Coding | 42 | GC01M091680 | 9.554558754 |
| SERPINB3 | Serpin Family B Member 3 | Protein Coding | 37 | GC18M063655 | 9.554258347 |
| CSNK1E | Casein Kinase 1 Epsilon | Protein Coding | 42 | GC22M056268 | 9.542160988 |

| | | | | | |
|---|---|---|---|---|---|
| EML4 | EMAP Like 4 | Protein Coding | 34 | GC02P042169 | 9.5313797 |
| CD19 | CD19 Molecule | Protein Coding | 44 | GC16P040100 | 9.527823448 |
| IFITM1 | Interferon Induced Transmembrane Protein 1 | Protein Coding | 36 | GC11P000313 | 9.517112732 |
| TACR3 | Tachykinin Receptor 3 | Protein Coding | 45 | GC04M103586 | 9.515699387 |
| PRKCB | Protein Kinase C Beta | Protein Coding | 42 | GC16P024204 | 9.50825119 |
| CASC17 | Cancer Susceptibility 17 | RNA Gene | 12 | GC17M070886 | 9.506593704 |
| AKR1B10 | Aldo-Keto Reductase Family 1 Member B10 | Protein Coding | 39 | GC07P134527 | 9.498260498 |
| FILIP1L | Filamin A Interacting Protein 1 Like | Protein Coding | 31 | GC03M099830 | 9.494078636 |
| MRPS22 | Mitochondrial Ribosomal Protein S22 | Protein Coding | 38 | GC03P139005 | 9.48543644 |
| TRAF4 | TNF Receptor Associated Factor 4 | Protein Coding | 38 | GC17P052500 | 9.477065086 |
| ADAM10 | ADAM Metallopeptidase Domain 10 | Protein Coding | 47 | GC15M058588 | 9.471644402 |
| FANCL | FA Complementation Group L | Protein Coding | 40 | GC02M058127 | 9.470293999 |
| MIR26B | MicroRNA 26b | RNA Gene | 21 | GC02P218402 | 9.469479561 |
| CDH11 | Cadherin 11 | Protein Coding | 43 | GC16M064943 | 9.468236923 |
| OLFM4 | Olfactomedin 4 | Protein Coding | 35 | GC13P053028 | 9.467037201 |
| TRAF7 | TNF Receptor Associated Factor 7 | Protein Coding | 38 | GC16P010727 | 9.463910103 |
| CNC2 | Carney Complex Type 2, Multiple Neoplasia And Lentiginosis | Genetic Locus | 3 | GC02U990267 | 9.451519966 |
| MIR574 | MicroRNA 574 | RNA Gene | 19 | GC04P038938 | 9.450434685 |
| NUP107 | Nucleoporin 107 | Protein Coding | 38 | GC12P068686 | 9.447508812 |
| PAX6 | Paired Box 6 | Protein Coding | 43 | GC11M031784 | 9.446169853 |
| DVL3 | Dishevelled Segment Polarity Protein 3 | Protein Coding | 40 | GC03P184155 | 9.443800926 |
| TRPV6 | Transient Receptor Potential Cation Channel Subfamily V Member 6 | Protein Coding | 42 | GC07M142871 | 9.44009304 |
| CTSL | Cathepsin L | Protein Coding | 42 | GC09P087725 | 9.438058853 |
| RICTOR | RPTOR Independent Companion Of MTOR Complex 2 | Protein Coding | 39 | GC05M038937 | 9.437280655 |
| THRB | Thyroid Hormone Receptor Beta | Protein Coding | 44 | GC03M024117 | 9.427206039 |
| MIR103A1 | MicroRNA 103a-1 | RNA Gene | 17 | GC05M168560 | 9.418956757 |
| SPEN | Spen Family Transcriptional Repressor | Protein Coding | 37 | GC01P015848 | 9.413984299 |
| UBE2C | Ubiquitin Conjugating Enzyme E2 C | Protein Coding | 41 | GC20P045812 | 9.409215927 |
| KAT5 | Lysine Acetyltransferase 5 | Protein Coding | 43 | GC11P065711 | 9.40860939 |
| EDNRB | Endothelin Receptor Type B | Protein Coding | 44 | GC13M077895 | 9.392467499 |
| HSD3B1 | Hydroxy-Delta-5-Steroid Dehydrogenase, 3 Beta- And Steroid Delta-Isomerase 1 | Protein Coding | 39 | GC01P119507 | 9.388690948 |
| ARL11 | ADP Ribosylation Factor Like GTPase 11 | Protein Coding | 34 | GC13P049628 | 9.379831314 |
| TNC | Tenascin C | Protein Coding | 44 | GC09M115019 | 9.379747391 |
| MBL2 | Mannose Binding Lectin 2 | Protein Coding | 42 | GC10M052760 | 9.379002571 |
| RPL11 | Ribosomal Protein L11 | Protein Coding | 42 | GC01P023691 | 9.37898159 |
| MIR675 | MicroRNA 675 | RNA Gene | 18 | GC11M003020 | 9.378212929 |
| DDX41 | DEAD-Box Helicase 41 | Protein Coding | 40 | GC05M177511 | 9.372455597 |
| LINC00261 | Long Intergenic Non-Protein Coding RNA 261 | RNA Gene | 18 | GC20M022547 | 9.367228508 |
| MIR503 | MicroRNA 503 | RNA Gene | 18 | GC0XM134688 | 9.367090225 |
| HS6ST1 | Heparan Sulfate 6-O-Sulfotransferase 1 | Protein Coding | 38 | GC02M128236 | 9.361598015 |
| OSM | Oncostatin M | Protein Coding | 38 | GC22M030262 | 9.359079361 |
| TFE3 | Transcription Factor Binding To IGHM Enhancer 3 | Protein Coding | 39 | GC0XM049028 | 9.3516922 |
| MIR485 | MicroRNA 485 | RNA Gene | 17 | GC14P109537 | 9.351510048 |
| RXRB | Retinoid X Receptor Beta | Protein Coding | 42 | GC06M033193 | 9.349264145 |
| ARSH | Arylsulfatase Family Member H | Protein Coding | 30 | GC0XP003006 | 9.346508026 |
| FUS | FUS RNA Binding Protein | Protein Coding | 40 | GC16P031180 | 9.341115952 |
| TRAF2 | TNF Receptor Associated Factor 2 | Protein Coding | 39 | GC09P136881 | 9.338370323 |
| MIR30C1 | MicroRNA 30c-1 | RNA Gene | 21 | GC01P040757 | 9.299893379 |
| CT55 | Cancer/Testis Antigen 55 | Protein Coding | 24 | GC0XM135156 | 9.290295601 |
| CEACAM1 | CEA Cell Adhesion Molecule 1 | Protein Coding | 39 | GC19M042507 | 9.288199425 |
| ADAR | Adenosine Deaminase RNA Specific | Protein Coding | 40 | GC01M154582 | 9.282993317 |
| STEAP2 | STEAP2 Metalloreductase | Protein Coding | 36 | GC07P090167 | 9.282959938 |
| MFAP5 | Microfibril Associated Protein 5 | Protein Coding | 38 | GC12M008637 | 9.272526741 |
| CLDN1 | Claudin 1 | Protein Coding | 41 | GC03M190305 | 9.267248154 |
| HESX1 | HESX Homeobox 1 | Protein Coding | 36 | GC03M057207 | 9.264079094 |
| HSP90B1 | Heat Shock Protein 90 Beta Family Member 1 | Protein Coding | 41 | GC12P103930 | 9.262907028 |
| CASC3 | CASC3 Exon Junction Complex Subunit | Protein Coding | 31 | GC17P040140 | 9.253604889 |
| ORC1 | Origin Recognition Complex Subunit 1 | Protein Coding | 39 | GC01M052372 | 9.250904083 |
| DYRK1A | Dual Specificity Tyrosine Phosphorylation Regulated Kinase 1A | Protein Coding | 45 | GC21P037365 | 9.236177444 |
| CCAR1 | Cell Division Cycle And Apoptosis Regulator 1 | Protein Coding | 34 | GC10P068721 | 9.222732544 |
| LRP1B | LDL Receptor Related Protein 1B | Protein Coding | 38 | GC02M140231 | 9.222730637 |
| COL4A3 | Collagen Type IV Alpha 3 Chain | Protein Coding | 40 | GC02P227164 | 9.213723183 |
| HPRT1 | Hypoxanthine Phosphoribosyltransferase 1 | Protein Coding | 42 | GC0XP134460 | 9.209970474 |
| UGT1A6 | UDP Glucuronosyltransferase Family 1 Member A6 | Protein Coding | 39 | GC02P233691 | 9.199277878 |
| FES | FES Proto-Oncogene, Tyrosine Kinase | Protein Coding | 43 | GC15P090883 | 9.19788456 |
| UGT1A7 | UDP Glucuronosyltransferase Family 1 Member A7 | Protein Coding | 36 | GC02P233681 | 9.19669342 |
| INSL3 | Insulin Like 3 | Protein Coding | 36 | GC19M017816 | 9.192266464 |
| MIR92A2 | MicroRNA 92a-2 | RNA Gene | 16 | GC0XM134300 | 9.190658569 |
| MTHFD1 | Methylenetetrahydrofolate Dehydrogenase, Cyclohydrolase And Formyltetrahydrofolate Synthetase 1 | Protein Coding | 40 | GC14P064388 | 9.187606812 |
| HYAL1 | Hyaluronidase 1 | Protein Coding | 42 | GC03M050299 | 9.18389225 |
| CYP2C9 | Cytochrome P450 Family 2 Subfamily C Member 9 | Protein Coding | 43 | GC10P094938 | 9.18384552 |
| IRX5 | Iroquois Homeobox 5 | Protein Coding | 36 | GC16P054930 | 9.177159309 |
| RAN | RAN, Member RAS Oncogene Family | Protein Coding | 39 | GC12P130871 | 9.172432899 |
| LAMC1 | Laminin Subunit Gamma 1 | Protein Coding | 39 | GC01P182992 | 9.161676407 |
| NBR1 | NBR1 Autophagy Cargo Receptor | Protein Coding | 34 | GC17P043170 | 9.161296844 |
| LPL | Lipoprotein Lipase | Protein Coding | 45 | GC08P019901 | 9.158999443 |
| F2RL1 | F2R Like Trypsin Receptor 1 | Protein Coding | 40 | GC05P076818 | 9.15054512 |
| ADAM9 | ADAM Metallopeptidase Domain 9 | Protein Coding | 44 | GC08P038996 | 9.150297165 |
| POLG | DNA Polymerase Gamma, Catalytic Subunit | Protein Coding | 43 | GC15M089798 | 9.146867752 |
| HNRNPA1 | Heterogeneous Nuclear Ribonucleoprotein A1 | Protein Coding | 41 | GC12P054280 | 9.144291878 |
| INSL6 | Insulin Like 6 | Protein Coding | 31 | GC09M004991 | 9.144182205 |
| CGA | Glycoprotein Hormones, Alpha Polypeptide | Protein Coding | 39 | GC06M087085 | 9.138792992 |
| FLNA | Filamin A | Protein Coding | 43 | GC0XM154348 | 9.135349274 |
| ADA | Adenosine Deaminase | Protein Coding | 45 | GC20M044620 | 9.124372482 |
| OVCA2 | OVCA2 Serine Hydrolase Domain Containing | Protein Coding | 30 | GC17P002041 | 9.113300323 |
| MFN2 | Mitofusin 2 | Protein Coding | 43 | GC01P011980 | 9.112897873 |
| ETS2 | ETS Proto-Oncogene 2, Transcription Factor | Protein Coding | 39 | GC21P038805 | 9.105821609 |
| TIAM1 | TIAM Rac1 Associated GEF 1 | Protein Coding | 40 | GC21M031118 | 9.099892616 |
| ERCC8 | ERCC Excision Repair 8, CSA Ubiquitin Ligase Complex Subunit | Protein Coding | 38 | GC05M060890 | 9.094571114 |
| TUBA1A | Tubulin Alpha 1a | Protein Coding | 43 | GC12M049184 | 9.093615532 |
| PROS1 | Protein S | Protein Coding | 43 | GC03M093873 | 9.090623856 |
| CAVIN3 | Caveolae Associated Protein 3 | Protein Coding | 29 | GC11M006326 | 9.086593628 |
| TBL1XR1 | TBL1X Receptor 1 | Protein Coding | 40 | GC03M177019 | 9.080383301 |
| OCA2 | OCA2 Melanosomal Transmembrane Protein | Protein Coding | 39 | GC15M027754 | 9.079816818 |

| | | | | | |
|---|---|---|---|---|---|
| EI24 | EI24 Autophagy Associated Transmembrane Protein | Protein Coding | 31 | GC11P125570 | 9.078710556 |
| FOXP1 | Forkhead Box P1 | Protein Coding | 40 | GC03M070926 | 9.068447113 |
| KMT2C | Lysine Methyltransferase 2C | Protein Coding | 38 | GC07M152134 | 9.067909241 |
| NNMT | Nicotinamide N-Methyltransferase | Protein Coding | 39 | GC11P114257 | 9.066348076 |
| RARG | Retinoic Acid Receptor Gamma | Protein Coding | 43 | GC12M053210 | 9.052555084 |
| PTH1R | Parathyroid Hormone 1 Receptor | Protein Coding | 44 | GC03P046877 | 9.043348312 |
| MAML2 | Mastermind Like Transcriptional Coactivator 2 | Protein Coding | 34 | GC11M095976 | 9.04172802 |
| PKD1 | Polycystin 1, Transient Receptor Potential Channel Interacting | Protein Coding | 42 | GC16M006689 | 9.041329384 |
| CP | Ceruloplasmin | Protein Coding | 44 | GC03M149162 | 9.03947258 |
| TP53BP2 | Tumor Protein P53 Binding Protein 2 | Protein Coding | 38 | GC01M223779 | 9.038502693 |
| FOXC2 | Forkhead Box C2 | Protein Coding | 39 | GC16P086577 | 9.022426605 |
| PRC1-AS1 | PRC1 Antisense RNA 1 | RNA Gene | 12 | GC15P090966 | 9.005273819 |
| MIR542 | MicroRNA 542 | RNA Gene | 17 | GC0XM134638 | 9.004257202 |
| CCL3 | C-C Motif Chemokine Ligand 3 | Protein Coding | 36 | GC17M036088 | 9.004132271 |
| SEMA3B | Semaphorin 3B | Protein Coding | 35 | GC03P050267 | 9.000377655 |
| HELLS | Helicase, Lymphoid Specific | Protein Coding | 41 | GC10P094501 | 8.999078751 |
| MIR130B | MicroRNA 130b | RNA Gene | 18 | GC22P035397 | 8.996931076 |
| LIMD1 | LIM Domain Containing 1 | Protein Coding | 35 | GC03P045555 | 8.992010117 |
| PDK1 | Pyruvate Dehydrogenase Kinase 1 | Protein Coding | 42 | GC02P172555 | 8.974936485 |
| SNHG20 | Small Nucleolar RNA Host Gene 20 | RNA Gene | 17 | GC17P077087 | 8.973701477 |
| ASCL1 | Achaete-Scute Family BHLH Transcription Factor 1 | Protein Coding | 42 | GC12P102957 | 8.971804619 |
| PCGEM1 | PCGEM1 Prostate-Specific Transcript | RNA Gene | 19 | GC02P192749 | 8.964727402 |
| LCK | LCK Proto-Oncogene, Src Family Tyrosine Kinase | Protein Coding | 47 | GC01P032251 | 8.954182625 |
| TACC3 | Transforming Acidic Coiled-Coil Containing Protein 3 | Protein Coding | 39 | GC04P001723 | 8.951259613 |
| MIR301A | MicroRNA 301a | RNA Gene | 19 | GC17M059151 | 8.948696136 |
| RBM6 | RNA Binding Motif Protein 6 | Protein Coding | 32 | GC03P049940 | 8.944576263 |
| ANO1 | Anoctamin 1 | Protein Coding | 38 | GC11P069986 | 8.942378998 |
| IQGAP1 | IQ Motif Containing GTPase Activating Protein 1 | Protein Coding | 38 | GC15P090388 | 8.934682846 |
| PRF1 | Perforin 1 | Protein Coding | 41 | GC10M070597 | 8.933930397 |
| MCAM | Melanoma Cell Adhesion Molecule | Protein Coding | 38 | GC11M119308 | 8.93362236 |
| HOXB9 | Homeobox B9 | Protein Coding | 36 | GC17M048621 | 8.929315567 |
| SPAG9 | Sperm Associated Antigen 9 | Protein Coding | 36 | GC17M050962 | 8.925408363 |
| ZFTA | Zinc Finger Translocation Associated | Protein Coding | 23 | GC11M089330 | 8.925235748 |
| GDNF | Glial Cell Derived Neurotrophic Factor | Protein Coding | 43 | GC05M037812 | 8.924492836 |
| RPS27 | Ribosomal Protein S27 | Protein Coding | 38 | GC01P153991 | 8.923172951 |
| F5 | Coagulation Factor V | Protein Coding | 42 | GC01M169511 | 8.921940804 |
| CD86 | CD86 Molecule | Protein Coding | 39 | GC03P122055 | 8.913783073 |
| ARNT | Aryl Hydrocarbon Receptor Nuclear Translocator | Protein Coding | 39 | GC01M150809 | 8.902933121 |
| ENPP1 | Ectonucleotide Pyrophosphatase/Phosphodiesterase 1 | Protein Coding | 44 | GC06P131808 | 8.899607658 |
| MYH11 | Myosin Heavy Chain 11 | Protein Coding | 42 | GC16M015704 | 8.893497467 |
| CCL4 | C-C Motif Chemokine Ligand 4 | Protein Coding | 36 | GC17P036103 | 8.892075539 |
| PSMC3IP | PSMC3 Interacting Protein | Protein Coding | 35 | GC17M042572 | 8.891090393 |
| ATP7A | ATPase Copper Transporting Alpha | Protein Coding | 42 | GC0XP077986 | 8.887724876 |
| CHKA | Choline Kinase Alpha | Protein Coding | 38 | GC11M068052 | 8.886825562 |
| KLK8 | Kallikrein Related Peptidase 8 | Protein Coding | 38 | GC19M050996 | 8.885210991 |
| MIR148B | MicroRNA 148b | RNA Gene | 19 | GC12P054337 | 8.882761002 |
| HLTF | Helicase Like Transcription Factor | Protein Coding | 35 | GC03M149030 | 8.876318932 |
| CXADR | CXADR Ig-Like Cell Adhesion Molecule | Protein Coding | 39 | GC21P017513 | 8.875923157 |
| MIR218-1 | MicroRNA 218-1 | RNA Gene | 17 | GC04P020835 | 8.866272926 |
| USP8 | Ubiquitin Specific Peptidase 8 | Protein Coding | 43 | GC15P050424 | 8.843986511 |
| IGF2-AS | IGF2 Antisense RNA | RNA Gene | 21 | GC11P002140 | 8.833913803 |
| FZD4 | Frizzled Class Receptor 4 | Protein Coding | 45 | GC11M086945 | 8.831658363 |
| OCIAD1 | OCIA Domain Containing 1 | Protein Coding | 32 | GC04P048805 | 8.830644608 |
| VRK1 | VRK Serine/Threonine Kinase 1 | Protein Coding | 44 | GC14P096797 | 8.815446854 |
| PAX9 | Paired Box 9 | Protein Coding | 40 | GC14P036657 | 8.811857224 |
| SLC34A2 | Solute Carrier Family 34 Member 2 | Protein Coding | 42 | GC04P025657 | 8.804777145 |
| DAB2 | DAB Adaptor Protein 2 | Protein Coding | 38 | GC05M039371 | 8.802600861 |
| COPS5 | COP9 Signalosome Subunit 5 | Protein Coding | 38 | GC08M067043 | 8.789813995 |
| CD59 | CD59 Molecule (CD59 Blood Group) | Protein Coding | 41 | GC11M033704 | 8.785261154 |
| PSAP | Prosaposin | Protein Coding | 43 | GC10M071816 | 8.773633957 |
| CDX1 | Caudal Type Homeobox 1 | Protein Coding | 35 | GC05P150166 | 8.757270813 |
| CRABP2 | Cellular Retinoic Acid Binding Protein 2 | Protein Coding | 39 | GC01M156701 | 8.757127762 |
| POU1F1 | POU Class 1 Homeobox 1 | Protein Coding | 38 | GC03M087259 | 8.756778717 |
| CDC20 | Cell Division Cycle 20 | Protein Coding | 39 | GC01P043358 | 8.75614357 |
| MED19 | Mediator Complex Subunit 19 | Protein Coding | 30 | GC11M057703 | 8.747873306 |
| HOPX | HOP Homeobox | Protein Coding | 32 | GC04M056647 | 8.742555618 |
| POLB | DNA Polymerase Beta | Protein Coding | 40 | GC08P042338 | 8.737288475 |
| TBK1 | TANK Binding Kinase 1 | Protein Coding | 44 | GC12P064451 | 8.733880043 |
| RHO | Rhodopsin | Protein Coding | 43 | GC03P133835 | 8.732405663 |
| ADRB2 | Adrenoceptor Beta 2 | Protein Coding | 44 | GC05P148825 | 8.727593422 |
| MIR125B2 | MicroRNA 125b-2 | RNA Gene | 20 | GC21P016590 | 8.725459099 |
| MB | Myoglobin | Protein Coding | 39 | GC22M035606 | 8.723925591 |
| NAB2 | NGFI-A Binding Protein 2 | Protein Coding | 37 | GC12P057240 | 8.721805573 |
| LCOR | Ligand Dependent Nuclear Receptor Corepressor | Protein Coding | 33 | GC10P096832 | 8.72177124 |
| PICALM | Phosphatidylinositol Binding Clathrin Assembly Protein | Protein Coding | 40 | GC11M085957 | 8.718426704 |
| SSTR1 | Somatostatin Receptor 1 | Protein Coding | 41 | GC14P038207 | 8.717670441 |
| TLR5 | Toll Like Receptor 5 | Protein Coding | 42 | GC01M223749 | 8.711174011 |
| GGT1 | Gamma-Glutamyltransferase 1 | Protein Coding | 45 | GC22P024583 | 8.708693504 |
| SFRP2 | Secreted Frizzled Related Protein 2 | Protein Coding | 36 | GC04M153780 | 8.703526497 |
| TRAF3 | TNF Receptor Associated Factor 3 | Protein Coding | 42 | GC14P109258 | 8.70087719 |
| FOLR1 | Folate Receptor Alpha | Protein Coding | 43 | GC11P072190 | 8.699690819 |
| INTS6 | Integrator Complex Subunit 6 | Protein Coding | 34 | GC13M051354 | 8.699655533 |
| PARN | Poly(A)-Specific Ribonuclease | Protein Coding | 40 | GC16M014435 | 8.697388649 |
| AGK | Acylglycerol Kinase | Protein Coding | 37 | GC07P141551 | 8.694363594 |
| CYP11B1 | Cytochrome P450 Family 11 Subfamily B Member 1 | Protein Coding | 43 | GC08M142872 | 8.647417068 |
| FKBP5 | FKBP Prolyl Isomerase 5 | Protein Coding | 42 | GC06M063751 | 8.646590233 |
| CD47 | CD47 Molecule | Protein Coding | 38 | GC03M108043 | 8.643940926 |
| CYP21A2 | Cytochrome P450 Family 21 Subfamily A Member 2 | Protein Coding | 40 | GC06P080382 | 8.639393806 |
| LPAR1 | Lysophosphatidic Acid Receptor 1 | Protein Coding | 41 | GC09M110873 | 8.639312744 |
| CALD1 | Caldesmon 1 | Protein Coding | 38 | GC07P134744 | 8.638440132 |
| ELANE | Elastase, Neutrophil Expressed | Protein Coding | 44 | GC19P002492 | 8.633953094 |
| AKR1C1 | Aldo-Keto Reductase Family 1 Member C1 | Protein Coding | 38 | GC10P004963 | 8.631681442 |
| RPS10 | Ribosomal Protein S10 | Protein Coding | 39 | GC06M063743 | 8.628076553 |
| ADAM15 | ADAM Metallopeptidase Domain 15 | Protein Coding | 39 | GC01P155050 | 8.624294281 |
| DHH | Desert Hedgehog Signaling Molecule | Protein Coding | 36 | GC12M049405 | 8.618766785 |

| | | | | | |
|---|---|---|---|---|---|
| PTP4A3 | Protein Tyrosine Phosphatase 4A3 | Protein Coding | 36 | GC08P141391 | 8.615849495 |
| ORC4 | Origin Recognition Complex Subunit 4 | Protein Coding | 37 | GC02M147930 | 8.607527733 |
| CCK | Cholecystokinin | Protein Coding | 38 | GC03M042274 | 8.606467247 |
| LNC-LBCS | LncRNA Bladder And Prostate Cancer Suppressor, HnRNPK Interacting | RNA Gene | 13 | GC06M019322 | 8.605313301 |
| KAT2B | Lysine Acetyltransferase 2B | Protein Coding | 43 | GC03P020043 | 8.602098465 |
| OXT | Oxytocin/Neurophysin I Prepropeptide | Protein Coding | 36 | GC20P003068 | 8.598249435 |
| GKN1 | Gastrokine 1 | Protein Coding | 35 | GC02P068974 | 8.596264839 |
| CASP6 | Caspase 6 | Protein Coding | 43 | GC04M109688 | 8.590427399 |
| SPANXD | SPANX Family Member D | Protein Coding | 27 | GC0XM141697 | 8.584287643 |
| TUSC7 | Tumor Suppressor Candidate 7 | RNA Gene | 15 | GC03P116647 | 8.569551468 |
| COL11A1 | Collagen Type XI Alpha 1 Chain | Protein Coding | 39 | GC01M102876 | 8.569464684 |
| CDA | Cytidine Deaminase | Protein Coding | 39 | GC01P020588 | 8.567551613 |
| ITGA9 | Integrin Subunit Alpha 9 | Protein Coding | 39 | GC03P037468 | 8.56256485 |
| TPO | Thyroid Peroxidase | Protein Coding | 44 | GC02P001374 | 8.559534073 |
| SH2B3 | SH2B Adaptor Protein 3 | Protein Coding | 42 | GC12P111405 | 8.55785656 |
| NTN1 | Netrin 1 | Protein Coding | 40 | GC17P009021 | 8.547605515 |
| MIR584 | MicroRNA 584 | RNA Gene | 18 | GC05M149062 | 8.544075966 |
| CSE1L | Chromosome Segregation 1 Like | Protein Coding | 35 | GC20P049046 | 8.541090965 |
| MAF | MAF BZIP Transcription Factor | Protein Coding | 41 | GC16M079204 | 8.539208412 |
| TTR | Transthyretin | Protein Coding | 43 | GC18P031557 | 8.538974762 |
| SYNE1 | Spectrin Repeat Containing Nuclear Envelope Protein 1 | Protein Coding | 38 | GC06M152121 | 8.537500381 |
| TERF2IP | TERF2 Interacting Protein | Protein Coding | 38 | GC16P075647 | 8.536913872 |
| ATP7B | ATPase Copper Transporting Beta | Protein Coding | 43 | GC13M051930 | 8.533514977 |
| CD68 | CD68 Molecule | Protein Coding | 35 | GC17P007579 | 8.531797409 |
| PAEP | Progestagen Associated Endometrial Protein | Protein Coding | 36 | GC09P135561 | 8.531728745 |
| G6PC1 | Glucose-6-Phosphatase Catalytic Subunit 1 | Protein Coding | 35 | GC17P053646 | 8.50770092 |
| CTBP2 | C-Terminal Binding Protein 2 | Protein Coding | 39 | GC10M124984 | 8.4980793 |
| TFDP1 | Transcription Factor Dp-1 | Protein Coding | 39 | GC13P113584 | 8.493275642 |
| LATS2 | Large Tumor Suppressor Kinase 2 | Protein Coding | 39 | GC13M020973 | 8.489191055 |
| DMTF1 | Cyclin D Binding Myb Like Transcription Factor 1 | Protein Coding | 33 | GC07P087155 | 8.486495972 |
| SLC16A7 | Solute Carrier Family 16 Member 7 | Protein Coding | 39 | GC12P059596 | 8.485321045 |
| PICART1 | P53 Inducible Cancer Associated RNA Transcript 1 | RNA Gene | 12 | GC17M050051 | 8.484124184 |
| MIR198 | MicroRNA 198 | RNA Gene | 15 | GC03M120395 | 8.481820107 |
| NOG | Noggin | Protein Coding | 42 | GC17P056593 | 8.481548309 |
| EEF1A2 | Eukaryotic Translation Elongation Factor 1 Alpha 2 | Protein Coding | 41 | GC20M063488 | 8.478683472 |
| CYLD | CYLD Lysine 63 Deubiquitinase | Protein Coding | 44 | GC16P050742 | 8.476044655 |
| SOX17 | SRY-Box Transcription Factor 17 | Protein Coding | 38 | GC08P054457 | 8.475682259 |
| VEGFB | Vascular Endothelial Growth Factor B | Protein Coding | 39 | GC11P064234 | 8.473708153 |
| LINC01133 | Long Intergenic Non-Protein Coding RNA 1133 | RNA Gene | 14 | GC01P159959 | 8.458810806 |
| SHMT1 | Serine Hydroxymethyltransferase 1 | Protein Coding | 40 | GC17M025184 | 8.451393127 |
| ACACA | Acetyl-CoA Carboxylase Alpha | Protein Coding | 44 | GC17M037084 | 8.448218346 |
| MKRN3 | Makorin Ring Finger Protein 3 | Protein Coding | 35 | GC15P039986 | 8.446946144 |
| IGFBP7 | Insulin Like Growth Factor Binding Protein 7 | Protein Coding | 40 | GC04M057030 | 8.445827484 |
| SETDB1 | SET Domain Bifurcated Histone Lysine Methyltransferase 1 | Protein Coding | 37 | GC01P150926 | 8.444070816 |
| ID2 | Inhibitor Of DNA Binding 2 | Protein Coding | 39 | GC02P008678 | 8.441373825 |
| SNHG5 | Small Nucleolar RNA Host Gene 5 | RNA Gene | 21 | GC06M085650 | 8.440238953 |
| EPB41L3 | Erythrocyte Membrane Protein Band 4.1 Like 3 | Protein Coding | 38 | GC18M005382 | 8.438591003 |
| KLF10 | Kruppel Like Factor 10 | Protein Coding | 38 | GC08M102648 | 8.435813904 |
| CBX2 | Chromobox 2 | Protein Coding | 38 | GC17P079778 | 8.431921005 |
| COL4A2 | Collagen Type IV Alpha 2 Chain | Protein Coding | 41 | GC13P110305 | 8.429306984 |
| MIR498 | MicroRNA 498 | RNA Gene | 15 | GC19P064294 | 8.420687675 |
| MIR495 | MicroRNA 495 | RNA Gene | 17 | GC14P109542 | 8.414082527 |
| ZNRD1ASP | Zinc Ribbon Domain Containing 1 Antisense, Pseudogene | Pseudogene | 15 | GC06M065586 | 8.413659096 |
| TUSC2 | Tumor Suppressor 2, Mitochondrial Calcium Regulator | Protein Coding | 30 | GC03M051105 | 8.404269218 |
| TACSTD2 | Tumor Associated Calcium Signal Transducer 2 | Protein Coding | 40 | GC01M058575 | 8.394278526 |
| IST1 | IST1 Factor Associated With ESCRT-III | Protein Coding | 33 | GC16P071946 | 8.383882523 |
| CDK5 | Cyclin Dependent Kinase 5 | Protein Coding | 47 | GC07M151053 | 8.381698608 |
| RASA1 | RAS P21 Protein Activator 1 | Protein Coding | 42 | GC05P087267 | 8.37669754 |
| COL4A1 | Collagen Type IV Alpha 1 Chain | Protein Coding | 43 | GC13M110148 | 8.375640869 |
| COX5A | Cytochrome C Oxidase Subunit 5A | Protein Coding | 39 | GC15M074919 | 8.370617867 |
| MYH9 | Myosin Heavy Chain 9 | Protein Coding | 44 | GC22M036281 | 8.366202354 |
| FCGR3A | Fc Gamma Receptor IIIa | Protein Coding | 41 | GC01M161541 | 8.366199493 |
| SLC35C2 | Solute Carrier Family 35 Member C2 | Protein Coding | 34 | GC20M046345 | 8.35853672 |
| THY1 | Thy-1 Cell Surface Antigen | Protein Coding | 38 | GC11M119417 | 8.356651306 |
| RBP1 | Retinol Binding Protein 1 | Protein Coding | 35 | GC03M139517 | 8.342266083 |
| USP28 | Ubiquitin Specific Peptidase 28 | Protein Coding | 35 | GC11M113797 | 8.337003708 |
| ORC6 | Origin Recognition Complex Subunit 6 | Protein Coding | 35 | GC16P046689 | 8.335323334 |
| CD46 | CD46 Molecule | Protein Coding | 42 | GC01P207752 | 8.329788208 |
| SRSF2 | Serine And Arginine Rich Splicing Factor 2 | Protein Coding | 36 | GC17M076734 | 8.323200226 |
| MSMP | Microseminoprotein, Prostate Associated | Protein Coding | 26 | GC09M035753 | 8.321789742 |
| CLIC1 | Chloride Intracellular Channel 1 | Protein Coding | 37 | GC06M063619 | 8.318582535 |
| CFL1 | Cofilin 1 | Protein Coding | 40 | GC11M065823 | 8.317251205 |
| TES | Testin LIM Domain Protein | Protein Coding | 36 | GC07P116210 | 8.31593895 |
| TMC8 | Transmembrane Channel Like 8 | Protein Coding | 36 | GC17P078130 | 8.311098099 |
| ACY1 | Aminoacylase 1 | Protein Coding | 41 | GC03P051983 | 8.294885635 |
| MIR326 | MicroRNA 326 | RNA Gene | 21 | GC11M075335 | 8.29289341 |
| AIFM1 | Apoptosis Inducing Factor Mitochondria Associated 1 | Protein Coding | 43 | GC0XM130129 | 8.291812897 |
| MIR494 | MicroRNA 494 | RNA Gene | 17 | GC14P109541 | 8.291791916 |
| TNS4 | Tensin 4 | Protein Coding | 34 | GC17M040475 | 8.291723251 |
| CR2 | Complement C3d Receptor 2 | Protein Coding | 42 | GC01P207454 | 8.291435242 |
| UMPS | Uridine Monophosphate Synthetase | Protein Coding | 40 | GC03P124730 | 8.290545464 |
| TMPRSS4 | Transmembrane Serine Protease 4 | Protein Coding | 35 | GC11P118077 | 8.289912224 |
| GPC1 | Glypican 1 | Protein Coding | 39 | GC02P240435 | 8.28884697 |
| RBL2 | RB Transcriptional Corepressor Like 2 | Protein Coding | 39 | GC16P053433 | 8.281250954 |
| RPS24 | Ribosomal Protein S24 | Protein Coding | 38 | GC10P078033 | 8.276674271 |
| SPOP | Speckle Type BTB/POZ Protein | Protein Coding | 38 | GC17M049598 | 8.275783539 |
| ANAPC1 | Anaphase Promoting Complex Subunit 1 | Protein Coding | 36 | GC02M111611 | 8.273554802 |
| TUBB3 | Tubulin Beta 3 Class III | Protein Coding | 43 | GC16P091197 | 8.268832207 |
| PPP1CB | Protein Phosphatase 1 Catalytic Subunit Beta | Protein Coding | 41 | GC02P028752 | 8.260857582 |
| PLK4 | Polo Like Kinase 4 | Protein Coding | 43 | GC04P127880 | 8.257152557 |
| MIR491 | MicroRNA 491 | RNA Gene | 19 | GC09P020716 | 8.257005692 |
| CGB5 | Chorionic Gonadotropin Subunit Beta 5 | Protein Coding | 32 | GC19P049043 | 8.251669884 |
| MIR4435-2HG | MIR4435-2 Host Gene | RNA Gene | 18 | GC02M111194 | 8.245985985 |
| NEDD4 | NEDD4 E3 Ubiquitin Protein Ligase | Protein Coding | 42 | GC15M055826 | 8.24590683 |
| STAG3 | Stromal Antigen 3 | Protein Coding | 38 | GC07P100177 | 8.241994858 |

| | | | | | |
|---|---|---|---|---|---|
| NLRP3 | NLR Family Pyrin Domain Containing 3 | Protein Coding | 42 | GC01P247415 | 8.231641769 |
| MIR340 | MicroRNA 340 | RNA Gene | 18 | GC05M180015 | 8.229107857 |
| FLRT3 | Fibronectin Leucine Rich Transmembrane Protein 3 | Protein Coding | 40 | GC20M014322 | 8.227434158 |
| WDR77 | WD Repeat Domain 77 | Protein Coding | 35 | GC01M111439 | 8.22722435 |
| H4-16 | H4 Histone 16 | Protein Coding | 30 | GC12M020508 | 8.215572357 |
| SOS2 | SOS Ras/Rho Guanine Nucleotide Exchange Factor 2 | Protein Coding | 40 | GC14M050117 | 8.213692665 |
| FGA | Fibrinogen Alpha Chain | Protein Coding | 43 | GC04M154583 | 8.210090637 |
| BTRC | Beta-Transducin Repeat Containing E3 Ubiquitin Protein Ligase | Protein Coding | 41 | GC10P101354 | 8.210012436 |
| XRCC4 | X-Ray Repair Cross Complementing 4 | Protein Coding | 39 | GC05P083077 | 8.208645821 |
| PORCN | Porcupine O-Acyltransferase | Protein Coding | 37 | GC0XP050497 | 8.205005646 |
| MIR425 | MicroRNA 425 | RNA Gene | 19 | GC03M051007 | 8.204485893 |
| BUB1B-PAK6 | BUB1B-PAK6 Readthrough | Protein Coding | 15 | GC15P040257 | 8.204462051 |
| KIF20B | Kinesin Family Member 20B | Protein Coding | 34 | GC10P089701 | 8.203108788 |
| IL7R | Interleukin 7 Receptor | Protein Coding | 41 | GC05P035852 | 8.197796822 |
| PLAT | Plasminogen Activator, Tissue Type | Protein Coding | 44 | GC08M042174 | 8.196699142 |
| GCLC | Glutamate-Cysteine Ligase Catalytic Subunit | Protein Coding | 39 | GC06M053497 | 8.189954758 |
| LPAR3 | Lysophosphatidic Acid Receptor 3 | Protein Coding | 38 | GC01M084811 | 8.188606262 |
| STAT4 | Signal Transducer And Activator Of Transcription 4 | Protein Coding | 42 | GC02M191029 | 8.184428215 |
| PMEPA1 | Prostate Transmembrane Protein, Androgen Induced 1 | Protein Coding | 34 | GC20M057648 | 8.18178463 |
| SLC6A3 | Solute Carrier Family 6 Member 3 | Protein Coding | 45 | GC05M001392 | 8.180887222 |
| MYOG | Myogenin | Protein Coding | 37 | GC01M203083 | 8.178733826 |
| DUXAP9 | Double Homeobox A Pseudogene 9 | Pseudogene | 7 | GC14P031387 | 8.178582191 |
| GSDME | Gasdermin E | Protein Coding | 32 | GC07M024699 | 8.177684784 |
| LUC7L2 | LUC7 Like 2, Pre-MRNA Splicing Factor | Protein Coding | 32 | GC07P139344 | 8.176976204 |
| TFF2 | Trefoil Factor 2 | Protein Coding | 37 | GC21M042346 | 8.174559593 |
| MTRR | 5-Methyltetrahydrofolate-Homocysteine Methyltransferase Reductase | Protein Coding | 39 | GC05P007851 | 8.173557281 |
| PER1 | Period Circadian Regulator 1 | Protein Coding | 37 | GC17M010272 | 8.172294617 |
| PAK4 | P21 (RAC1) Activated Kinase 4 | Protein Coding | 43 | GC19P039125 | 8.169309616 |
| AGO2 | Argonaute RISC Catalytic Component 2 | Protein Coding | 36 | GC08M140522 | 8.165401459 |
| MAK | Male Germ Cell Associated Kinase | Protein Coding | 39 | GC06M010762 | 8.1646595 |
| CCR3 | C-C Motif Chemokine Receptor 3 | Protein Coding | 40 | GC03P046404 | 8.160037041 |
| HDGF | Heparin Binding Growth Factor | Protein Coding | 36 | GC01M156897 | 8.158230782 |
| CCL11 | C-C Motif Chemokine Ligand 11 | Protein Coding | 40 | GC17P034285 | 8.157835007 |
| WWP2 | WW Domain Containing E3 Ubiquitin Protein Ligase 2 | Protein Coding | 40 | GC16P069796 | 8.145466805 |
| CYB5A | Cytochrome B5 Type A | Protein Coding | 39 | GC18M074250 | 8.144385338 |
| OTX2 | Orthodenticle Homeobox 2 | Protein Coding | 41 | GC14M056799 | 8.121442795 |
| LYN | LYN Proto-Oncogene, Src Family Tyrosine Kinase | Protein Coding | 43 | GC08P055879 | 8.11356163 |
| EHMT2 | Euchromatic Histone Lysine Methyltransferase 2 | Protein Coding | 40 | GC06M031879 | 8.112826347 |
| GOLM2 | Golgi Membrane Protein 2 | Protein Coding | 27 | GC15P044289 | 8.111813545 |
| GADD45G | Growth Arrest And DNA Damage Inducible Gamma | Protein Coding | 36 | GC09P089605 | 8.107494354 |
| SELP | Selectin P | Protein Coding | 41 | GC01M169558 | 8.105730057 |
| LIN28A | Lin-28 Homolog A | Protein Coding | 35 | GC01P026410 | 8.102386475 |
| LY6K | Lymphocyte Antigen 6 Family Member K | Protein Coding | 32 | GC08P142700 | 8.097428322 |
| RPS29 | Ribosomal Protein S29 | Protein Coding | 36 | GC14M049570 | 8.094874382 |
| TAGLN | Transgelin | Protein Coding | 39 | GC11P117199 | 8.094395638 |
| PRAME | PRAME Nuclear Receptor Transcriptional Regulator | Protein Coding | 34 | GC22M022547 | 8.090416908 |
| SEZ6L2 | Seizure Related 6 Homolog Like 2 | Protein Coding | 34 | GC16M036452 | 8.077397346 |
| DIRAS3 | DIRAS Family GTPase 3 | Protein Coding | 29 | GC01M068045 | 8.073729515 |
| AKR1C2 | Aldo-Keto Reductase Family 1 Member C2 | Protein Coding | 41 | GC10M004987 | 8.07178688 |
| EDA | Ectodysplasin A | Protein Coding | 38 | GC0XP069618 | 8.071718216 |
| ITGA2B | Integrin Subunit Alpha 2b | Protein Coding | 46 | GC17M044863 | 8.069505692 |
| ACVR1 | Activin A Receptor Type 1 | Protein Coding | 46 | GC02M157736 | 8.064027786 |
| KIF1B | Kinesin Family Member 1B | Protein Coding | 40 | GC01P010210 | 8.051817894 |
| RYR1 | Ryanodine Receptor 1 | Protein Coding | 42 | GC19P063535 | 8.047586441 |
| PHLPP2 | PH Domain And Leucine Rich Repeat Protein Phosphatase 2 | Protein Coding | 36 | GC16M071637 | 8.047444344 |
| MIR28 | MicroRNA 28 | RNA Gene | 19 | GC03P188688 | 8.045030594 |
| DHX37 | DEAH-Box Helicase 37 | Protein Coding | 34 | GC12M124946 | 8.041837692 |
| SH3GL1 | SH3 Domain Containing GRB2 Like 1, Endophilin A2 | Protein Coding | 38 | GC19M004360 | 8.041248322 |
| SCUBE3 | Signal Peptide, CUB Domain And EGF Like Domain Containing 3 | Protein Coding | 36 | GC06P080508 | 8.041119576 |
| CENPF | Centromere Protein F | Protein Coding | 39 | GC01P214603 | 8.030546188 |
| CBLL1 | Cbl Proto-Oncogene Like 1 | Protein Coding | 33 | GC07P107743 | 8.021663666 |
| CD55 | CD55 Molecule (Cromer Blood Group) | Protein Coding | 42 | GC01P207321 | 8.017427444 |
| SDCCAG8 | SHH Signaling And Ciliogenesis Regulator SDCCAG8 | Protein Coding | 38 | GC01P243255 | 8.015824318 |
| PRKAA2 | Protein Kinase AMP-Activated Catalytic Subunit Alpha 2 | Protein Coding | 46 | GC01P056645 | 8.013944626 |
| EPHA5 | EPH Receptor A5 | Protein Coding | 42 | GC04M065319 | 8.013108253 |
| KLK11 | Kallikrein Related Peptidase 11 | Protein Coding | 37 | GC19M064223 | 8.012651443 |
| PODXL | Podocalyxin Like | Protein Coding | 39 | GC07M131500 | 8.012415886 |
| IL1R1 | Interleukin 1 Receptor Type 1 | Protein Coding | 40 | GC02P102136 | 8.011564255 |
| CD164 | CD164 Molecule | Protein Coding | 38 | GC06M109366 | 8.007498741 |
| RTEL1-TNFRSF6B | RTEL1-TNFRSF6B Readthrough (NMD Candidate) | RNA Gene | 15 | GC20P063657 | 8.007157326 |
| RNY1 | RNA, Ro60-Associated Y1 | RNA Gene | 15 | GC07M148987 | 7.996426582 |
| RNY3 | RNA, Ro60-Associated Y3 | RNA Gene | 14 | GC07P149006 | 7.996426582 |
| EPHB6 | EPH Receptor B6 | Protein Coding | 41 | GC07P147996 | 7.995657921 |
| MADD | MAP Kinase Activating Death Domain | Protein Coding | 39 | GC11P047290 | 7.989716053 |
| CUL5 | Cullin 5 | Protein Coding | 39 | GC11P108008 | 7.984937668 |
| GAEC1 | Gene Amplified In Esophageal Cancer 1 | RNA Gene | 4 | GC07U901831 | 7.984894276 |
| AIMP2 | Aminoacyl TRNA Synthetase Complex Interacting Multifunctional Protein 2 | Protein Coding | 37 | GC07P006016 | 7.981208324 |
| S100A1 | S100 Calcium Binding Protein A1 | Protein Coding | 36 | GC01P153627 | 7.979939461 |
| AGAP2-AS1 | AGAP2 Antisense RNA 1 | RNA Gene | 13 | GC12P057726 | 7.976515293 |
| VCP | Valosin Containing Protein | Protein Coding | 44 | GC09M035056 | 7.971859932 |
| PBK | PDZ Binding Kinase | Protein Coding | 38 | GC08M027809 | 7.963516235 |
| LBR | Lamin B Receptor | Protein Coding | 42 | GC01M225401 | 7.961688042 |
| AGTR1 | Angiotensin II Receptor Type 1 | Protein Coding | 45 | GC03P148697 | 7.955394268 |
| ZEB2-AS1 | ZEB2 Antisense RNA 1 | RNA Gene | 19 | GC02P144519 | 7.946087837 |
| MFGE8 | Milk Fat Globule EGF And Factor V/VIII Domain Containing | Protein Coding | 39 | GC15M088898 | 7.942759514 |
| PRPF4 | Pre-MRNA Processing Factor 4 | Protein Coding | 38 | GC09P113275 | 7.940806389 |
| FUT4 | Fucosyltransferase 4 | Protein Coding | 33 | GC11P094544 | 7.938540459 |
| MIR135B | MicroRNA 135b | RNA Gene | 20 | GC01M205448 | 7.935585499 |
| ECRG4 | ECRG4 Augurin Precursor | Protein Coding | 26 | GC02P106063 | 7.931233883 |
| SMC1A | Structural Maintenance Of Chromosomes 1A | Protein Coding | 40 | GC0XM053374 | 7.929465294 |
| MIR135A1 | MicroRNA 135a-1 | RNA Gene | 19 | GC03M052296 | 7.92750597 |
| SERBP1 | SERPINE1 MRNA Binding Protein 1 | Protein Coding | 31 | GC01M067407 | 7.926814556 |
| MIR17HG | MiR-17-92a-1 Cluster Host Gene | RNA Gene | 25 | GC13P091347 | 7.920260429 |
| DNTT | DNA Nucleotidylexotransferase | Protein Coding | 37 | GC10P096304 | 7.918721676 |
| TNS1 | Tensin 1 | Protein Coding | 35 | GC02M217799 | 7.918542862 |

| | | | | | |
|---|---|---|---|---|---|
| COL2A1 | Collagen Type II Alpha 1 Chain | Protein Coding | 43 | GC12M047972 | 7.910278797 |
| CDYL2 | Chromodomain Y Like 2 | Protein Coding | 32 | GC16M080598 | 7.909247875 |
| PTPRH | Protein Tyrosine Phosphatase Receptor Type H | Protein Coding | 36 | GC19M055181 | 7.90616703 |
| ABCB4 | ATP Binding Cassette Subfamily B Member 4 | Protein Coding | 43 | GC07M087401 | 7.903206348 |
| TLR7 | Toll Like Receptor 7 | Protein Coding | 43 | GC0XP012867 | 7.900792599 |
| RPL13 | Ribosomal Protein L13 | Protein Coding | 38 | GC16P091179 | 7.894107819 |
| L1CAM | L1 Cell Adhesion Molecule | Protein Coding | 42 | GC0XM153864 | 7.886521816 |
| GAB1 | GRB2 Associated Binding Protein 1 | Protein Coding | 40 | GC04P143336 | 7.886449337 |
| MIR22HG | MIR22 Host Gene | RNA Gene | 25 | GC17M002869 | 7.883399487 |
| NPC1 | NPC Intracellular Cholesterol Transporter 1 | Protein Coding | 44 | GC18M023506 | 7.882177353 |
| ACHE | Acetylcholinesterase (Cartwright Blood Group) | Protein Coding | 41 | GC07M100889 | 7.874952316 |
| TSPY1 | Testis Specific Protein Y-Linked 1 | Protein Coding | 30 | GC0YP009469 | 7.873265266 |
| LIG1 | DNA Ligase 1 | Protein Coding | 41 | GC19M048115 | 7.872554779 |
| URGCP | Upregulator Of Cell Proliferation | Protein Coding | 31 | GC07M043876 | 7.868072987 |
| GPNMB | Glycoprotein Nmb | Protein Coding | 40 | GC07P023238 | 7.859582424 |
| HNRNPU | Heterogeneous Nuclear Ribonucleoprotein U | Protein Coding | 38 | GC01M244844 | 7.856520653 |
| MIR381 | MicroRNA 381 | RNA Gene | 18 | GC14P109530 | 7.853264332 |
| SRPK1 | SRSF Protein Kinase 1 | Protein Coding | 41 | GC06M063756 | 7.844410896 |
| PER2 | Period Circadian Regulator 2 | Protein Coding | 39 | GC02M238244 | 7.843056202 |
| FOXQ1 | Forkhead Box Q1 | Protein Coding | 31 | GC06P001312 | 7.842067719 |
| ZNF217 | Zinc Finger Protein 217 | Protein Coding | 35 | GC20M053567 | 7.840907574 |
| DEK | DEK Proto-Oncogene | Protein Coding | 37 | GC06M018224 | 7.825214386 |
| REL | REL Proto-Oncogene, NF-KB Subunit | Protein Coding | 42 | GC02P060881 | 7.818760872 |
| MAP3K7 | Mitogen-Activated Protein Kinase Kinase Kinase 7 | Protein Coding | 46 | GC06M090513 | 7.817889214 |
| RXRG | Retinoid X Receptor Gamma | Protein Coding | 39 | GC01M165401 | 7.816710472 |
| MIR154 | MicroRNA 154 | RNA Gene | 19 | GC14P109513 | 7.814015388 |
| TIE1 | Tyrosine Kinase With Immunoglobulin Like And EGF Like Domains 1 | Protein Coding | 39 | GC01P043300 | 7.813913345 |
| SLIT2 | Slit Guidance Ligand 2 | Protein Coding | 40 | GC04P020287 | 7.812903404 |
| CCN6 | Cellular Communication Network Factor 6 | Protein Coding | 33 | GC06P112053 | 7.805732727 |
| GSK3A | Glycogen Synthase Kinase 3 Alpha | Protein Coding | 43 | GC19M063852 | 7.804412842 |
| PPHLN1 | Periphilin 1 | Protein Coding | 32 | GC12P042238 | 7.804193974 |
| MAPT | Microtubule Associated Protein Tau | Protein Coding | 44 | GC17P045894 | 7.802229881 |
| CXCL5 | C-X-C Motif Chemokine Ligand 5 | Protein Coding | 36 | GC04M073995 | 7.798790932 |
| ACSL4 | Acyl-CoA Synthetase Long Chain Family Member 4 | Protein Coding | 39 | GC0XM109624 | 7.798552513 |
| PRUNE2 | Prune Homolog 2 With BCH Domain | Protein Coding | 34 | GC09M076611 | 7.797206879 |
| MIR605 | MicroRNA 605 | RNA Gene | 17 | GC10P051299 | 7.791265965 |
| LCT | Lactase | Protein Coding | 38 | GC02M135787 | 7.791226864 |
| BMP15 | Bone Morphogenetic Protein 15 | Protein Coding | 36 | GC0XP050910 | 7.786548615 |
| MPLKIP | M-Phase Specific PLK1 Interacting Protein | Protein Coding | 32 | GC07M040126 | 7.78249073 |
| MIR219A1 | MicroRNA 219a-1 | RNA Gene | 19 | GC06P033207 | 7.781878471 |
| MMP26 | Matrix Metallopeptidase 26 | Protein Coding | 34 | GC11P004706 | 7.781708241 |
| SMCHD1 | Structural Maintenance Of Chromosomes Flexible Hinge Domain Containing 1 | Protein Coding | 36 | GC18P002649 | 7.77633667 |
| SALL4 | Spalt Like Transcription Factor 4 | Protein Coding | 39 | GC20M051784 | 7.775586128 |
| GPRC5A | G Protein-Coupled Receptor Class C Group 5 Member A | Protein Coding | 36 | GC12P020084 | 7.769866467 |
| CGB3 | Chorionic Gonadotropin Subunit Beta 3 | Protein Coding | 30 | GC19M064092 | 7.767601013 |
| RALBP1 | RalA Binding Protein 1 | Protein Coding | 40 | GC18P009465 | 7.765699863 |
| HBB | Hemoglobin Subunit Beta | Protein Coding | 41 | GC11M006315 | 7.765445709 |
| PRDX3 | Peroxiredoxin 3 | Protein Coding | 39 | GC10M119167 | 7.763619423 |
| LETMD1 | LETM1 Domain Containing 1 | Protein Coding | 32 | GC12P051047 | 7.763396263 |
| MALT1 | MALT1 Paracaspase | Protein Coding | 43 | GC18P058671 | 7.762928963 |
| ZNF268 | Zinc Finger Protein 268 | Protein Coding | 33 | GC12P133181 | 7.762480259 |
| CD2 | CD2 Molecule | Protein Coding | 39 | GC01P116754 | 7.757265568 |
| DCLK1 | Doublecortin Like Kinase 1 | Protein Coding | 40 | GC13M035768 | 7.747893333 |
| FEZF1-AS1 | FEZF1 Antisense RNA 1 | RNA Gene | 15 | GC07P122303 | 7.746499062 |
| ID4 | Inhibitor Of DNA Binding 4, HLH Protein | Protein Coding | 35 | GC06P019837 | 7.746448517 |
| LOC107982234 | WT1/WT1-AS Bi-Directional Promoter Region | Biological Region | 3 | GC11P032430 | 7.744631767 |
| NUDT1 | Nudix Hydrolase 1 | Protein Coding | 38 | GC07P002242 | 7.742958546 |
| TLR1 | Toll Like Receptor 1 | Protein Coding | 42 | GC04M038793 | 7.740073204 |
| FOXA2 | Forkhead Box A2 | Protein Coding | 40 | GC20M022581 | 7.738650322 |
| GNRH2 | Gonadotropin Releasing Hormone 2 | Protein Coding | 32 | GC20P003976 | 7.732384205 |
| PLAGL1 | PLAG1 Like Zinc Finger 1 | Protein Coding | 39 | GC06M143940 | 7.72542429 |
| MIR33A | MicroRNA 33a | RNA Gene | 19 | GC22P041900 | 7.719690323 |
| PAK2 | P21 (RAC1) Activated Kinase 2 | Protein Coding | 41 | GC03P196739 | 7.719367504 |
| NPTN-IT1 | NPTN Intronic Transcript 1 | RNA Gene | 13 | GC15M073566 | 7.717943668 |
| RPS15 | Ribosomal Protein S15 | Protein Coding | 35 | GC19P001438 | 7.716480255 |
| SLFN11 | Schlafen Family Member 11 | Protein Coding | 31 | GC17M035350 | 7.710017204 |
| MN1 | MN1 Proto-Oncogene, Transcriptional Regulator | Protein Coding | 38 | GC22M027748 | 7.707823753 |
| DOCK8 | Dedicator Of Cytokinesis 8 | Protein Coding | 40 | GC09P000214 | 7.707288742 |
| USF2 | Upstream Transcription Factor 2, C-Fos Interacting | Protein Coding | 35 | GC19P035268 | 7.706807613 |
| STC1 | Stanniocalcin 1 | Protein Coding | 36 | GC08M023841 | 7.700612068 |
| GPC4 | Glypican 4 | Protein Coding | 40 | GC0XM133300 | 7.696077347 |
| ZFP36L1 | ZFP36 Ring Finger Protein Like 1 | Protein Coding | 38 | GC14M068787 | 7.692462444 |
| TAC1 | Tachykinin Precursor 1 | Protein Coding | 38 | GC07P097731 | 7.688979149 |
| SPTAN1 | Spectrin Alpha, Non-Erythrocytic 1 | Protein Coding | 43 | GC09P128552 | 7.686974525 |
| SAA1 | Serum Amyloid A1 | Protein Coding | 37 | GC11P018479 | 7.681398392 |
| LOC109504725 | Androgen Receptor Repeat Instability Region | Biological Region | 3 | GC0XP067546 | 7.67496109 |
| MIR339 | MicroRNA 339 | RNA Gene | 20 | GC07M001022 | 7.674228191 |
| BIVM-ERCC5 | BIVM-ERCC5 Readthrough | Protein Coding | 16 | GC13P102814 | 7.673055649 |
| NUMB | NUMB Endocytic Adaptor Protein | Protein Coding | 41 | GC14M073275 | 7.67234993 |
| MIR134 | MicroRNA 134 | RNA Gene | 18 | GC14P109512 | 7.671107292 |
| NUPR1 | Nuclear Protein 1, Transcriptional Regulator | Protein Coding | 31 | GC16M028532 | 7.670063019 |
| MMP15 | Matrix Metallopeptidase 15 | Protein Coding | 42 | GC16P058025 | 7.668417454 |
| ZEB1-AS1 | ZEB1 Antisense RNA 1 | RNA Gene | 17 | GC10M031166 | 7.667812347 |
| COL1A2 | Collagen Type I Alpha 2 Chain | Protein Coding | 43 | GC07P094394 | 7.666914463 |
| ZAP70 | Zeta Chain Of T Cell Receptor Associated Protein Kinase 70 | Protein Coding | 46 | GC02P097742 | 7.656892776 |
| CPA1 | Carboxypeptidase A1 | Protein Coding | 41 | GC07P130380 | 7.65231514 |
| WNT9A | Wnt Family Member 9A | Protein Coding | 38 | GC01M227920 | 7.651197433 |
| GUSB | Glucuronidase Beta | Protein Coding | 43 | GC07M065960 | 7.648701668 |
| ZNF350 | Zinc Finger Protein 350 | Protein Coding | 34 | GC19M064270 | 7.648450375 |
| MIR7-1 | MicroRNA 7-1 | RNA Gene | 17 | GC09M092477 | 7.640860081 |
| SLC22A16 | Solute Carrier Family 22 Member 16 | Protein Coding | 34 | GC06M110424 | 7.640432835 |
| IGH | Immunoglobulin Heavy Locus | Protein Coding | 17 | GC14M112400 | 7.639221191 |
| SOCAR | Serous Ovarian Cancer Associated RNA | RNA Gene | 10 | GC02P122424 | 7.639127254 |
| ALKBH3 | AlkB Homolog 3, Alpha-Ketoglutarate Dependent Dioxygenase | Protein Coding | 34 | GC11P043902 | 7.63828373 |
| MAP1LC3A | Microtubule Associated Protein 1 Light Chain 3 Alpha | Protein Coding | 37 | GC20P034546 | 7.635397911 |

| | | | | | |
|---|---|---|---|---|---|
| MYBL2 | MYB Proto-Oncogene Like 2 | Protein Coding | 38 | GC20P043667 | 7.618069649 |
| CLDN18 | Claudin 18 | Protein Coding | 38 | GC03P137998 | 7.611573219 |
| ACRBP | Acrosin Binding Protein | Protein Coding | 30 | GC12M006638 | 7.610981941 |
| IL5 | Interleukin 5 | Protein Coding | 40 | GC05M132541 | 7.607557297 |
| SMC3 | Structural Maintenance Of Chromosomes 3 | Protein Coding | 40 | GC10P110567 | 7.602720261 |
| NUTM1 | NUT Midline Carcinoma Family Member 1 | Protein Coding | 31 | GC15P034343 | 7.589437962 |
| AVP | Arginine Vasopressin | Protein Coding | 41 | GC20M003082 | 7.586884975 |
| H1-0 | H1.0 Linker Histone | Protein Coding | 31 | GC22P038367 | 7.584803104 |
| RBPJ | Recombination Signal Binding Protein For Immunoglobulin Kappa J Region | Protein Coding | 42 | GC04P026165 | 7.584605217 |
| ST20 | Suppressor Of Tumorigenicity 20 | RNA Gene | 23 | GC15M079898 | 7.584421158 |
| NACC1 | Nucleus Accumbens Associated 1 | Protein Coding | 37 | GC19P013701 | 7.580021858 |
| NHP2 | NHP2 Ribonucleoprotein | Protein Coding | 39 | GC05M178149 | 7.579558849 |
| NOX1 | NADPH Oxidase 1 | Protein Coding | 38 | GC0XM100843 | 7.579484463 |
| TRAF1 | TNF Receptor Associated Factor 1 | Protein Coding | 38 | GC09M120902 | 7.564995289 |
| RBL1 | RB Transcriptional Corepressor Like 1 | Protein Coding | 37 | GC20M036996 | 7.564521313 |
| TSPAN31 | Tetraspanin 31 | Protein Coding | 35 | GC12P057738 | 7.564138412 |
| CSF3R | Colony Stimulating Factor 3 Receptor | Protein Coding | 42 | GC01M036466 | 7.562031746 |
| CAMK2N1 | Calcium/Calmodulin Dependent Protein Kinase II Inhibitor 1 | Protein Coding | 29 | GC01M020482 | 7.556292057 |
| TFG | Trafficking From ER To Golgi Regulator | Protein Coding | 40 | GC03P100709 | 7.554090977 |
| NTSR1 | Neurotensin Receptor 1 | Protein Coding | 39 | GC20P062708 | 7.550009727 |
| CLDN6 | Claudin 6 | Protein Coding | 36 | GC16M003014 | 7.547427177 |
| GTF2H5 | General Transcription Factor IIH Subunit 5 | Protein Coding | 35 | GC06P158168 | 7.543177605 |
| REG4 | Regenerating Family Member 4 | Protein Coding | 35 | GC01M119794 | 7.539782047 |
| PSAT1 | Phosphoserine Aminotransferase 1 | Protein Coding | 42 | GC09P078297 | 7.538953781 |
| EEF2 | Eukaryotic Translation Elongation Factor 2 | Protein Coding | 43 | GC19M003976 | 7.534496307 |
| MAD2L2 | Mitotic Arrest Deficient 2 Like 2 | Protein Coding | 39 | GC01M011674 | 7.533975601 |
| UGT2B17 | UDP Glucuronosyltransferase Family 2 Member B17 | Protein Coding | 38 | GC04M068537 | 7.529019356 |
| PRKACB | Protein Kinase CAMP-Activated Catalytic Subunit Beta | Protein Coding | 44 | GC01P084078 | 7.528017998 |
| VDAC1 | Voltage Dependent Anion Channel 1 | Protein Coding | 42 | GC05M133975 | 7.526082993 |
| KRT13 | Keratin 13 | Protein Coding | 39 | GC17M041500 | 7.519154549 |
| THEMIS2 | Thymocyte Selection Associated Family Member 2 | Protein Coding | 30 | GC01P027872 | 7.511286736 |
| EFNB3 | Ephrin B3 | Protein Coding | 38 | GC17P010893 | 7.510596752 |
| SFTPA1 | Surfactant Protein A1 | Protein Coding | 39 | GC10P092345 | 7.503427982 |
| TPM4 | Tropomyosin 4 | Protein Coding | 35 | GC19P063078 | 7.497777939 |
| TBC1D3 | TBC1 Domain Family Member 3 | Protein Coding | 24 | GC17M038181 | 7.490194321 |
| IL32 | Interleukin 32 | Protein Coding | 34 | GC16P010759 | 7.487534046 |
| PXDN | Peroxidasin | Protein Coding | 40 | GC02M001635 | 7.484786987 |
| CAMP | Cathelicidin Antimicrobial Peptide | Protein Coding | 36 | GC03P048865 | 7.482841969 |
| CD276 | CD276 Molecule | Protein Coding | 37 | GC15P073683 | 7.482778549 |
| FHL2 | Four And A Half LIM Domains 2 | Protein Coding | 42 | GC02M105357 | 7.48017931 |
| RBM5 | RNA Binding Motif Protein 5 | Protein Coding | 35 | GC03P050296 | 7.477570534 |
| TF | Transferrin | Protein Coding | 44 | GC03P133912 | 7.476698399 |
| IKZF1 | IKAROS Family Zinc Finger 1 | Protein Coding | 43 | GC07P050303 | 7.475067139 |
| API5 | Apoptosis Inhibitor 5 | Protein Coding | 34 | GC11P043311 | 7.474962234 |
| RNH1 | Ribonuclease/Angiogenin Inhibitor 1 | Protein Coding | 34 | GC11M002876 | 7.472899914 |
| RPS6KA2 | Ribosomal Protein S6 Kinase A2 | Protein Coding | 41 | GC06M166409 | 7.465917587 |
| NIN | Ninein | Protein Coding | 39 | GC14M050719 | 7.462252617 |
| GADD45B | Growth Arrest And DNA Damage Inducible Beta | Protein Coding | 35 | GC19P002476 | 7.462242126 |
| PIWIL1 | Piwi Like RNA-Mediated Gene Silencing 1 | Protein Coding | 37 | GC12P130337 | 7.460118771 |
| NONO | Non-POU Domain Containing Octamer Binding | Protein Coding | 40 | GC0XP071255 | 7.45116806 |
| LAMA5 | Laminin Subunit Alpha 5 | Protein Coding | 39 | GC20M062307 | 7.450139523 |
| BPIFA1 | BPI Fold Containing Family A Member 1 | Protein Coding | 32 | GC20P033235 | 7.44878006 |
| IGF2BP2 | Insulin Like Growth Factor 2 MRNA Binding Protein 2 | Protein Coding | 40 | GC03M185643 | 7.44797039 |
| FDPS | Farnesyl Diphosphate Synthase | Protein Coding | 41 | GC01P155308 | 7.443861485 |
| KNL1 | Kinetochore Scaffold 1 | Protein Coding | 34 | GC15P040595 | 7.432815552 |
| SATB2 | SATB Homeobox 2 | Protein Coding | 40 | GC02M199269 | 7.43227911 |
| XDH | Xanthine Dehydrogenase | Protein Coding | 42 | GC02M031334 | 7.432105064 |
| KRT15 | Keratin 15 | Protein Coding | 35 | GC17M041513 | 7.431824684 |
| RIOX1 | Ribosomal Oxygenase 1 | Protein Coding | 24 | GC14P073492 | 7.431280613 |
| PCLAF | PCNA Clamp Associated Factor | Protein Coding | 29 | GC15M081142 | 7.424714565 |
| AKAP3 | A-Kinase Anchoring Protein 3 | Protein Coding | 32 | GC12M005129 | 7.411458969 |
| COL4A5 | Collagen Type IV Alpha 5 Chain | Protein Coding | 40 | GC0XP108439 | 7.407982349 |
| SNHG6 | Small Nucleolar RNA Host Gene 6 | RNA Gene | 18 | GC08M066921 | 7.4050951 |
| HEPACAM | Hepatic And Glial Cell Adhesion Molecule | Protein Coding | 36 | GC11M124919 | 7.399581432 |
| ACP5 | Acid Phosphatase 5, Tartrate Resistant | Protein Coding | 42 | GC19M011574 | 7.395531654 |
| SERPINF1 | Serpin Family F Member 1 | Protein Coding | 40 | GC17P001761 | 7.395174026 |
| NOP10 | NOP10 Ribonucleoprotein | Protein Coding | 36 | GC15M034341 | 7.39516449 |
| INHBA | Inhibin Subunit Beta A | Protein Coding | 40 | GC07M041668 | 7.381458282 |
| ATF2 | Activating Transcription Factor 2 | Protein Coding | 41 | GC02M175072 | 7.381289482 |
| PRDX2 | Peroxiredoxin 2 | Protein Coding | 41 | GC19M012796 | 7.379261971 |
| RPS6KA5 | Ribosomal Protein S6 Kinase A5 | Protein Coding | 40 | GC14M090847 | 7.377964973 |
| LINC-PINT | Long Intergenic Non-Protein Coding RNA, P53 Induced Transcript | RNA Gene | 17 | GC07M130986 | 7.377702236 |
| PASD1 | PAS Domain Containing Repressor 1 | Protein Coding | 26 | GC0XP151563 | 7.377668858 |
| IL13RA2 | Interleukin 13 Receptor Subunit Alpha 2 | Protein Coding | 35 | GC0XM115003 | 7.376926899 |
| NSD2 | Nuclear Receptor Binding SET Domain Protein 2 | Protein Coding | 35 | GC04P001872 | 7.376135826 |
| SPA17 | Sperm Autoantigenic Protein 17 | Protein Coding | 33 | GC11P124673 | 7.36950922 |
| TRPV4 | Transient Receptor Potential Cation Channel Subfamily V Member 4 | Protein Coding | 44 | GC12M109783 | 7.369147778 |
| LINC00858 | Long Intergenic Non-Protein Coding RNA 858 | RNA Gene | 14 | GC10P091469 | 7.362873554 |
| NIPBL | NIPBL Cohesin Loading Factor | Protein Coding | 38 | GC05P036876 | 7.362674713 |
| IKZF3 | IKAROS Family Zinc Finger 3 | Protein Coding | 38 | GC17M041541 | 7.35936594 |
| BTC | Betacellulin | Protein Coding | 39 | GC04M074744 | 7.358966827 |
| ADNP | Activity Dependent Neuroprotector Homeobox | Protein Coding | 39 | GC20M050888 | 7.350583076 |
| TMPRSS3 | Transmembrane Serine Protease 3 | Protein Coding | 39 | GC21M042371 | 7.344798088 |
| PIK3C3 | Phosphatidylinositol 3-Kinase Catalytic Subunit Type 3 | Protein Coding | 44 | GC18P041955 | 7.34400177 |
| RASA2 | RAS P21 Protein Activator 2 | Protein Coding | 35 | GC03P141487 | 7.341365814 |
| ABCA3 | ATP Binding Cassette Subfamily A Member 3 | Protein Coding | 44 | GC16M002275 | 7.340417862 |
| SPTBN1 | Spectrin Beta, Non-Erythrocytic 1 | Protein Coding | 41 | GC02P054456 | 7.336458683 |
| MIA2 | MIA SH3 Domain ER Export Factor 2 | Protein Coding | 34 | GC14P039230 | 7.33498764 |
| CEP290 | Centrosomal Protein 290 | Protein Coding | 36 | GC12M088049 | 7.331502914 |
| MYO18B | Myosin XVIIIB | Protein Coding | 34 | GC22P025742 | 7.328091621 |
| PIAS3 | Protein Inhibitor Of Activated STAT 3 | Protein Coding | 36 | GC01M145848 | 7.323560238 |
| HDAC3 | Histone Deacetylase 3 | Protein Coding | 44 | GC05M141583 | 7.321789742 |
| BMX | BMX Non-Receptor Tyrosine Kinase | Protein Coding | 40 | GC0XP015392 | 7.319500446 |
| TGFB1I1 | Transforming Growth Factor Beta 1 Induced Transcript 1 | Protein Coding | 37 | GC16P040322 | 7.317483902 |
| SYT7 | Synaptotagmin 7 | Protein Coding | 35 | GC11M061513 | 7.314953804 |

| | | | | | |
|---|---|---|---|---|---|
| ERN1 | Endoplasmic Reticulum To Nucleus Signaling 1 | Protein Coding | 40 | GC17M064039 | 7.310089588 |
| PVR | PVR Cell Adhesion Molecule | Protein Coding | 40 | GC19P063832 | 7.30938673 |
| PTGER2 | Prostaglandin E Receptor 2 | Protein Coding | 43 | GC14P052314 | 7.308784962 |
| GKN2 | Gastrokine 2 | Protein Coding | 32 | GC02M068945 | 7.308350563 |
| RSF1 | Remodeling And Spacing Factor 1 | Protein Coding | 32 | GC11M087713 | 7.30552578 |
| RCN1 | Reticulocalbin 1 | Protein Coding | 36 | GC11P032090 | 7.304420471 |
| CBR1 | Carbonyl Reductase 1 | Protein Coding | 40 | GC21P036069 | 7.304409027 |
| FGL1 | Fibrinogen Like 1 | Protein Coding | 36 | GC08M017864 | 7.298895359 |
| NLK | Nemo Like Kinase | Protein Coding | 39 | GC17P052456 | 7.298551083 |
| NMB | Neuromedin B | Protein Coding | 36 | GC15M084655 | 7.29846096 |
| TLE1 | TLE Family Member 1, Transcriptional Corepressor | Protein Coding | 39 | GC09M081583 | 7.293656349 |
| TSPO | Translocator Protein | Protein Coding | 40 | GC22P043151 | 7.29338789 |
| NAA15 | N-Alpha-Acetyltransferase 15, NatA Auxiliary Subunit | Protein Coding | 35 | GC04P139301 | 7.292694569 |
| TPR | Translocated Promoter Region, Nuclear Basket Protein | Protein Coding | 39 | GC01M186319 | 7.288656235 |
| CUX1 | Cut Like Homeobox 1 | Protein Coding | 40 | GC07P101815 | 7.27427721 |
| MS4A1 | Membrane Spanning 4-Domains A1 | Protein Coding | 42 | GC11P060507 | 7.272031784 |
| VIPR1 | Vasoactive Intestinal Peptide Receptor 1 | Protein Coding | 41 | GC03P042490 | 7.266154766 |
| EHMT1 | Euchromatic Histone Lysine Methyltransferase 1 | Protein Coding | 40 | GC09P137618 | 7.258368492 |
| MSN | Moesin | Protein Coding | 42 | GC0XP065588 | 7.257109642 |
| TIA1 | TIA1 Cytotoxic Granule Associated RNA Binding Protein | Protein Coding | 39 | GC02M070209 | 7.256054878 |
| KL | Klotho | Protein Coding | 42 | GC13P033016 | 7.255009651 |
| APOA1 | Apolipoprotein A1 | Protein Coding | 43 | GC11M116835 | 7.253983974 |
| PROP1 | PROP Paired-Like Homeobox 1 | Protein Coding | 37 | GC05M177992 | 7.243697166 |
| CCDC6 | Coiled-Coil Domain Containing 6 | Protein Coding | 35 | GC10M059788 | 7.242576122 |
| TPBG | Trophoblast Glycoprotein | Protein Coding | 38 | GC06P083237 | 7.237730026 |
| FMR1 | Fragile X Messenger Ribonucleoprotein 1 | Protein Coding | 39 | GC0XP147933 | 7.236457348 |
| CLEC3B | C-Type Lectin Domain Family 3 Member B | Protein Coding | 36 | GC03P046362 | 7.22979641 |
| ELN | Elastin | Protein Coding | 40 | GC07P074027 | 7.227454185 |
| APOB | Apolipoprotein B | Protein Coding | 42 | GC02M020956 | 7.220458031 |
| SULF2 | Sulfatase 2 | Protein Coding | 36 | GC20M047656 | 7.214310646 |
| RUVBL1 | RuvB Like AAA ATPase 1 | Protein Coding | 39 | GC03M128064 | 7.212322712 |
| FOXK2 | Forkhead Box K2 | Protein Coding | 34 | GC17P082519 | 7.210268974 |
| GALT | Galactose-1-Phosphate Uridylyltransferase | Protein Coding | 42 | GC09P040090 | 7.209493637 |
| CCN5 | Cellular Communication Network Factor 5 | Protein Coding | 29 | GC20P044715 | 7.205046177 |
| ARRB1 | Arrestin Beta 1 | Protein Coding | 39 | GC11M087632 | 7.203659058 |
| ELAVL4 | ELAV Like RNA Binding Protein 4 | Protein Coding | 36 | GC01P050025 | 7.201319695 |
| MIR590 | MicroRNA 590 | RNA Gene | 19 | GC07P074191 | 7.201209545 |
| FOXL2 | Forkhead Box L2 | Protein Coding | 38 | GC03M138944 | 7.200375557 |
| ADAMTSL1 | ADAMTS Like 1 | Protein Coding | 38 | GC09P017906 | 7.200142384 |
| CD81 | CD81 Molecule | Protein Coding | 40 | GC11P002516 | 7.19979763 |
| RPA1 | Replication Protein A1 | Protein Coding | 40 | GC17P001829 | 7.198969364 |
| CD63 | CD63 Molecule | Protein Coding | 38 | GC12M055725 | 7.193204403 |
| ALX4 | ALX Homeobox 4 | Protein Coding | 36 | GC11M044238 | 7.180965424 |
| CREB3L4 | CAMP Responsive Element Binding Protein 3 Like 4 | Protein Coding | 33 | GC01P153967 | 7.16311264 |
| ITGAE | Integrin Subunit Alpha E | Protein Coding | 36 | GC17M004111 | 7.15927124 |
| ABCB11 | ATP Binding Cassette Subfamily B Member 11 | Protein Coding | 43 | GC02M168922 | 7.158433914 |
| MIR376C | MicroRNA 376c | RNA Gene | 17 | GC14P109526 | 7.157770157 |
| MMP10 | Matrix Metallopeptidase 10 | Protein Coding | 41 | GC11M102770 | 7.156535149 |
| FTO | FTO Alpha-Ketoglutarate Dependent Dioxygenase | Protein Coding | 41 | GC16P053811 | 7.150333405 |
| WNT16 | Wnt Family Member 16 | Protein Coding | 38 | GC07P121325 | 7.147491932 |
| ALOX15B | Arachidonate 15-Lipoxygenase Type B | Protein Coding | 37 | GC17P008039 | 7.146059513 |
| MIR432 | MicroRNA 432 | RNA Gene | 17 | GC14P109206 | 7.145867348 |
| PIK3R4 | Phosphoinositide-3-Kinase Regulatory Subunit 4 | Protein Coding | 41 | GC03M130678 | 7.145700455 |
| RAC3 | Rac Family Small GTPase 3 | Protein Coding | 40 | GC17P082031 | 7.141115189 |
| UBE2V1 | Ubiquitin Conjugating Enzyme E2 V1 | Protein Coding | 36 | GC20M050082 | 7.13978672 |
| PENK | Proenkephalin | Protein Coding | 34 | GC08M056436 | 7.135622978 |
| FLNC | Filamin C | Protein Coding | 41 | GC07P128830 | 7.134456635 |
| GPR101 | G Protein-Coupled Receptor 101 | Protein Coding | 35 | GC0XM137030 | 7.127487183 |
| PDE8B | Phosphodiesterase 8B | Protein Coding | 42 | GC05P077180 | 7.118765831 |
| MIR452 | MicroRNA 452 | RNA Gene | 15 | GC0XM151959 | 7.118038654 |
| DKK2 | Dickkopf WNT Signaling Pathway Inhibitor 2 | Protein Coding | 38 | GC04M106921 | 7.115264416 |
| SCGB1A1 | Secretoglobin Family 1A Member 1 | Protein Coding | 36 | GC11P062405 | 7.110078812 |
| TGIF2 | TGFB Induced Factor Homeobox 2 | Protein Coding | 35 | GC20P036573 | 7.107996464 |
| FER1L4 | Fer-1 Like Family Member 4 (Pseudogene) | Pseudogene | 20 | GC20M035558 | 7.106350422 |
| SFTPD | Surfactant Protein D | Protein Coding | 39 | GC10M079937 | 7.09766531 |
| RARS1 | Arginyl-TRNA Synthetase 1 | Protein Coding | 35 | GC05P168487 | 7.096086502 |
| SGK1 | Serum/Glucocorticoid Regulated Kinase 1 | Protein Coding | 43 | GC06M134169 | 7.095646858 |
| SEMA3F | Semaphorin 3F | Protein Coding | 38 | GC03P050309 | 7.089884758 |
| EIF5A2 | Eukaryotic Translation Initiation Factor 5A2 | Protein Coding | 35 | GC03M170888 | 7.085554123 |
| MYH8 | Myosin Heavy Chain 8 | Protein Coding | 36 | GC17M010390 | 7.083518028 |
| ASNS | Asparagine Synthetase (Glutamine-Hydrolyzing) | Protein Coding | 42 | GC07M097854 | 7.081029892 |
| KCNQ1 | Potassium Voltage-Gated Channel Subfamily Q Member 1 | Protein Coding | 44 | GC11P002444 | 7.07830143 |
| FMNL2 | Formin Like 2 | Protein Coding | 34 | GC02P152335 | 7.078093529 |
| LINC01194 | Long Intergenic Non-Protein Coding RNA 1194 | RNA Gene | 16 | GC05P012578 | 7.073698044 |
| NAPSA | Napsin A Aspartic Peptidase | Protein Coding | 36 | GC19M064188 | 7.071383476 |
| MIR337 | MicroRNA 337 | RNA Gene | 19 | GC14P109521 | 7.070581913 |
| MIR365A | MicroRNA 365a | RNA Gene | 18 | GC16P014309 | 7.069812775 |
| MIR490 | MicroRNA 490 | RNA Gene | 17 | GC07P136903 | 7.068764687 |
| F2RL3 | F2R Like Thrombin Or Trypsin Receptor 3 | Protein Coding | 41 | GC19P016888 | 7.067370892 |
| RASSF5 | Ras Association Domain Family Member 5 | Protein Coding | 35 | GC01P206507 | 7.06733799 |
| CCBE1 | Collagen And Calcium Binding EGF Domains 1 | Protein Coding | 38 | GC18M059430 | 7.064353466 |
| TMPRSS11A | Transmembrane Serine Protease 11A | Protein Coding | 34 | GC04M067909 | 7.060410023 |
| IREB2 | Iron Responsive Element Binding Protein 2 | Protein Coding | 40 | GC15P078437 | 7.060392857 |
| WDCP | WD Repeat And Coiled Coil Containing | Protein Coding | 26 | GC02M024032 | 7.0603652 |
| WAS | WASP Actin Nucleation Promoting Factor | Protein Coding | 43 | GC0XP048676 | 7.059365273 |
| UGT1A | UDP Glucuronosyltransferase Family 1 Member A Complex Locus | Uncategorized | 11 | GC02P233592 | 7.058786392 |
| CD5 | CD5 Molecule | Protein Coding | 37 | GC11P061114 | 7.055550098 |
| LPAR2 | Lysophosphatidic Acid Receptor 2 | Protein Coding | 39 | GC19M019624 | 7.055465698 |
| PDX1 | Pancreatic And Duodenal Homeobox 1 | Protein Coding | 42 | GC13P027921 | 7.05422163 |
| PRSS8 | Serine Protease 8 | Protein Coding | 41 | GC16M036582 | 7.048728943 |
| CHAC1 | ChaC Glutathione Specific Gamma-Glutamylcyclotransferase 1 | Protein Coding | 32 | GC15P040942 | 7.046084404 |
| HIC2 | HIC ZBTB Transcriptional Repressor 2 | Protein Coding | 32 | GC22P034521 | 7.041343689 |
| KRT1 | Keratin 1 | Protein Coding | 42 | GC12M052674 | 7.039627552 |
| SCT | Secretin | Protein Coding | 32 | GC11M000626 | 7.03875494 |
| TACC2 | Transforming Acidic Coiled-Coil Containing Protein 2 | Protein Coding | 34 | GC10P121989 | 7.036678791 |

| | | | | | |
|---|---|---|---|---|---|
| MBD4 | Methyl-CpG Binding Domain 4, DNA Glycosylase | Protein Coding | 38 | GC03M129430 | 7.032867908 |
| FAM98A | Family With Sequence Similarity 98 Member A | Protein Coding | 30 | GC02M033532 | 7.031746864 |
| UBC | Ubiquitin C | Protein Coding | 37 | GC12M124911 | 7.030749321 |
| CLDN2 | Claudin 2 | Protein Coding | 38 | GC0XP106900 | 7.028675556 |
| JUNB | JunB Proto-Oncogene, AP-1 Transcription Factor Subunit | Protein Coding | 37 | GC19P012791 | 7.021186829 |
| TOB1 | Transducer Of ERBB2, 1 | Protein Coding | 36 | GC17M050862 | 7.019906998 |
| PRCC | Proline Rich Mitotic Checkpoint Control Factor | Protein Coding | 33 | GC01P156750 | 7.018567085 |
| MRTFA | Myocardin Related Transcription Factor A | Protein Coding | 33 | GC22M056576 | 7.010437965 |
| FCGR2B | Fc Gamma Receptor IIb | Protein Coding | 42 | GC01P161667 | 7.006106377 |
| DKK4 | Dickkopf WNT Signaling Pathway Inhibitor 4 | Protein Coding | 36 | GC08M042373 | 7.001217842 |
| FGF23 | Fibroblast Growth Factor 23 | Protein Coding | 41 | GC12M004368 | 6.99947834 |
| WNT9B | Wnt Family Member 9B | Protein Coding | 40 | GC17P046833 | 6.998509407 |
| CHD8 | Chromodomain Helicase DNA Binding Protein 8 | Protein Coding | 36 | GC14M021385 | 6.997776985 |
| PTPN22 | Protein Tyrosine Phosphatase Non-Receptor Type 22 | Protein Coding | 44 | GC01M113813 | 6.9949193 |
| EFEMP1 | EGF Containing Fibulin Extracellular Matrix Protein 1 | Protein Coding | 40 | GC02M055865 | 6.99402523 |
| FOXD2-AS1 | FOXD2 Adjacent Opposite Strand RNA 1 | RNA Gene | 15 | GC01M047432 | 6.988849163 |
| TTC4 | Tetratricopeptide Repeat Domain 4 | Protein Coding | 31 | GC01P054715 | 6.987241745 |
| GPR19 | G Protein-Coupled Receptor 19 | Protein Coding | 32 | GC12M020472 | 6.986150265 |
| TBXT | T-Box Transcription Factor T | Protein Coding | 34 | GC06M166158 | 6.984223366 |
| NCR1 | Natural Cytotoxicity Triggering Receptor 1 | Protein Coding | 36 | GC19P054906 | 6.980146408 |
| ACKR3 | Atypical Chemokine Receptor 3 | Protein Coding | 37 | GC02P236537 | 6.972281933 |
| YES1 | YES Proto-Oncogene 1, Src Family Tyrosine Kinase | Protein Coding | 43 | GC18M000721 | 6.968968391 |
| RARRES1 | Retinoic Acid Receptor Responder 1 | Protein Coding | 37 | GC03M158696 | 6.967569351 |
| CTC1 | CST Telomere Replication Complex Component 1 | Protein Coding | 31 | GC17M010292 | 6.961246967 |
| CPT2 | Carnitine Palmitoyltransferase 2 | Protein Coding | 44 | GC01P053196 | 6.961130142 |
| FZD10 | Frizzled Class Receptor 10 | Protein Coding | 40 | GC12P130162 | 6.956719398 |
| STEAP3 | STEAP3 Metalloreductase | Protein Coding | 40 | GC02P119222 | 6.955551147 |
| ZBTB16 | Zinc Finger And BTB Domain Containing 16 | Protein Coding | 40 | GC11P114059 | 6.955020905 |
| MDH2 | Malate Dehydrogenase 2 | Protein Coding | 43 | GC07P076048 | 6.953613281 |
| MIR95 | MicroRNA 95 | RNA Gene | 17 | GC04M008007 | 6.946261406 |
| ACVR2A | Activin A Receptor Type 2A | Protein Coding | 40 | GC02P147844 | 6.945469856 |
| SEC14L2 | SEC14 Like Lipid Binding 2 | Protein Coding | 36 | GC22P030396 | 6.936919689 |
| TXNDC15 | Thioredoxin Domain Containing 15 | Protein Coding | 34 | GC05P134873 | 6.929222584 |
| ANG | Angiogenin | Protein Coding | 40 | GC14P031538 | 6.922673702 |
| GPX3 | Glutathione Peroxidase 3 | Protein Coding | 38 | GC05P150997 | 6.922120571 |
| RGCC | Regulator Of Cell Cycle | Protein Coding | 31 | GC13P041457 | 6.91986084 |
| IFNAR1 | Interferon Alpha And Beta Receptor Subunit 1 | Protein Coding | 41 | GC21P033324 | 6.917791367 |
| SMARCA5 | SWI/SNF Related, Matrix Associated, Actin Dependent Regulator Of Chromatin, Subfamily A, Member 5 | Protein Coding | 37 | GC04P143513 | 6.916371346 |
| RIPK2 | Receptor Interacting Serine/Threonine Kinase 2 | Protein Coding | 40 | GC08P089795 | 6.908596516 |
| GPT | Glutamic--Pyruvic Transaminase | Protein Coding | 37 | GC08P144502 | 6.90734148 |
| PLCB4 | Phospholipase C Beta 4 | Protein Coding | 42 | GC20P009024 | 6.905785084 |
| SEMA4F | Ssemaphorin 4F | Protein Coding | 35 | GC02P074654 | 6.90559721 |
| CKB | Creatine Kinase B | Protein Coding | 40 | GC14M103519 | 6.901136875 |
| CCDC141 | Coiled-Coil Domain Containing 141 | Protein Coding | 31 | GC02M178829 | 6.894455433 |
| VRK2 | VRK Serine/Threonine Kinase 2 | Protein Coding | 38 | GC02P057907 | 6.892698288 |
| ARMC5 | Armadillo Repeat Containing 5 | Protein Coding | 32 | GC16P040320 | 6.890628815 |
| NSMF | NMDA Receptor Synaptonuclear Signaling And Neuronal Migration Factor | Protein Coding | 34 | GC09M137447 | 6.880100727 |
| IRF5 | Interferon Regulatory Factor 5 | Protein Coding | 42 | GC07P128937 | 6.871278286 |
| CLDN23 | Claudin 23 | Protein Coding | 32 | GC08P008701 | 6.870542526 |
| ASPSCR1 | ASPSCR1 Tether For SLC2A4, UBX Domain Containing | Protein Coding | 37 | GC17P081976 | 6.868377209 |
| TAC3 | Tachykinin Precursor 3 | Protein Coding | 40 | GC12M057009 | 6.858230591 |
| GHRHR | Growth Hormone Releasing Hormone Receptor | Protein Coding | 41 | GC07P030938 | 6.855569839 |
| AMBP | Alpha-1-Microglobulin/Bikunin Precursor | Protein Coding | 39 | GC09M114060 | 6.855216503 |
| CD247 | CD247 Molecule | Protein Coding | 44 | GC01M167399 | 6.853652 |
| CHIC2 | Cysteine Rich Hydrophobic Domain 2 | Protein Coding | 34 | GC04M053996 | 6.849542618 |
| ATP8B1 | ATPase Phospholipid Transporting 8B1 | Protein Coding | 36 | GC18M057646 | 6.843447685 |
| FURIN | Furin, Paired Basic Amino Acid Cleaving Enzyme | Protein Coding | 41 | GC15P090868 | 6.841121197 |
| PAX2 | Paired Box 2 | Protein Coding | 42 | GC10P100735 | 6.839229584 |
| CRYAA | Crystallin Alpha A | Protein Coding | 41 | GC21P043169 | 6.836739063 |
| POR | Cytochrome P450 Oxidoreductase | Protein Coding | 45 | GC07P075899 | 6.825927734 |
| ATF7IP | Activating Transcription Factor 7 Interacting Protein | Protein Coding | 32 | GC12P014365 | 6.81163311 |
| STC2 | Stanniocalcin 2 | Protein Coding | 38 | GC05M173314 | 6.809667587 |
| SCAP | SREBF Chaperone | Protein Coding | 37 | GC03M047413 | 6.80687809 |
| H19-ICR | H19/IGF2 Imprinting Control Region | Biological Region | 6 | GC11P002009 | 6.804301262 |
| BAG3 | BAG Cochaperone 3 | Protein Coding | 39 | GC10P119651 | 6.801129818 |
| MIR190A | MicroRNA 190a | RNA Gene | 19 | GC15P115964 | 6.800111771 |
| AKT1S1 | AKT1 Substrate 1 | Protein Coding | 34 | GC19M049869 | 6.798726559 |
| FLG | Filaggrin | Protein Coding | 36 | GC01M152274 | 6.794706821 |
| MRAS | Muscle RAS Oncogene Homolog | Protein Coding | 41 | GC03P138347 | 6.794578552 |
| KCNH1 | Potassium Voltage-Gated Channel Subfamily H Member 1 | Protein Coding | 43 | GC01M210678 | 6.78627491 |
| PDCD1LG2 | Programmed Cell Death 1 Ligand 2 | Protein Coding | 37 | GC09P005510 | 6.784667015 |
| SH3KBP1 | SH3 Domain Containing Kinase Binding Protein 1 | Protein Coding | 36 | GC0XM019552 | 6.784503937 |
| WNT8B | Wnt Family Member 8B | Protein Coding | 38 | GC10P100463 | 6.780493736 |
| TP53TG1 | TP53 Target 1 | RNA Gene | 22 | GC07M087325 | 6.777688503 |
| RBBP6 | RB Binding Protein 6, Ubiquitin Ligase | Protein Coding | 35 | GC16P024537 | 6.776799679 |
| CD22 | CD22 Molecule | Protein Coding | 41 | GC19P035319 | 6.773788929 |
| HSP90AB1 | Heat Shock Protein 90 Alpha Family Class B Member 1 | Protein Coding | 42 | GC06P044246 | 6.772764206 |
| PLS3 | Plastin 3 | Protein Coding | 38 | GC0XP115560 | 6.76968956 |
| GIPC1 | GIPC PDZ Domain Containing Family Member 1 | Protein Coding | 39 | GC19M014479 | 6.765088558 |
| PLCE1 | Phospholipase C Epsilon 1 | Protein Coding | 39 | GC10P093993 | 6.764855862 |
| ACTG1 | Actin Gamma 1 | Protein Coding | 44 | GC17M081509 | 6.763878822 |
| PRPF31 | Pre-MRNA Processing Factor 31 | Protein Coding | 39 | GC19P064320 | 6.762674332 |
| SNCA | Synuclein Alpha | Protein Coding | 45 | GC04M089724 | 6.761163712 |
| PPP2CA | Protein Phosphatase 2 Catalytic Subunit Alpha | Protein Coding | 44 | GC05M134194 | 6.75977993 |
| MAP3K2 | Mitogen-Activated Protein Kinase Kinase Kinase 2 | Protein Coding | 40 | GC02M127298 | 6.75357151 |
| SERPINC1 | Serpin Family C Member 1 | Protein Coding | 43 | GC01M174525 | 6.753114223 |
| MIR218-2 | MicroRNA 218-2 | RNA Gene | 19 | GC05M168768 | 6.752580166 |
| METTL13 | Methyltransferase 13, EEF1A Lysine And N-Terminal Methyltransferase | Protein Coding | 34 | GC01P171781 | 6.752519608 |
| RPS27A | Ribosomal Protein S27a | Protein Coding | 37 | GC02P055231 | 6.75114727 |
| IGHG1 | Immunoglobulin Heavy Constant Gamma 1 (G1m Marker) | Protein Coding | 29 | GC14M105736 | 6.747807503 |
| RPL26 | Ribosomal Protein L26 | Protein Coding | 37 | GC17M008377 | 6.740792274 |
| RANBP2 | RAN Binding Protein 2 | Protein Coding | 42 | GC02P108719 | 6.738179684 |
| FIP1L1 | Factor Interacting With PAPOLA And CPSF1 | Protein Coding | 35 | GC04P053422 | 6.724611282 |
| FUT2 | Fucosyltransferase 2 | Protein Coding | 40 | GC19P048695 | 6.723986626 |

| | | | | | |
|---|---|---|---|---|---|
| EDAR | Ectodysplasin A Receptor | Protein Coding | 39 | GC02M108894 | 6.722870827 |
| MYHAS | Myosin Heavy Chain Gene Cluster Antisense RNA | RNA Gene | 13 | GC17P010383 | 6.716319084 |
| HTR2A | 5-Hydroxytryptamine Receptor 2A | Protein Coding | 43 | GC13M046831 | 6.714083672 |
| RPS26 | Ribosomal Protein S26 | Protein Coding | 37 | GC12P056043 | 6.713833809 |
| DDIAS | DNA Damage Induced Apoptosis Suppressor | Protein Coding | 27 | GC11P082899 | 6.71284914 |
| CLK2 | CDC Like Kinase 2 | Protein Coding | 38 | GC01M155262 | 6.708558083 |
| SOX5 | SRY-Box Transcription Factor 5 | Protein Coding | 41 | GC12M023529 | 6.702252388 |
| RSPO1 | R-Spondin 1 | Protein Coding | 39 | GC01M037612 | 6.700962067 |
| BCOR | BCL6 Corepressor | Protein Coding | 38 | GC0XM040049 | 6.699620724 |
| SCN5A | Sodium Voltage-Gated Channel Alpha Subunit 5 | Protein Coding | 46 | GC03M038549 | 6.698981285 |
| STRAP | Serine/Threonine Kinase Receptor Associated Protein | Protein Coding | 32 | GC12P015882 | 6.697734833 |
| USB1 | U6 SnRNA Biogenesis Phosphodiesterase 1 | Protein Coding | 32 | GC16P057999 | 6.685684204 |
| CACYBP | Calcyclin Binding Protein | Protein Coding | 34 | GC01P175001 | 6.683596611 |
| DGCR5 | DiGeorge Syndrome Critical Region Gene 5 | RNA Gene | 19 | GC22P034383 | 6.682974815 |
| GAS8-AS1 | GAS8 Antisense RNA 1 | RNA Gene | 20 | GC16M090028 | 6.682449818 |
| NODAL | Nodal Growth Differentiation Factor | Protein Coding | 40 | GC10M070431 | 6.674561501 |
| TMEM97 | Transmembrane Protein 97 | Protein Coding | 32 | GC17P028319 | 6.67126894 |
| SEMA4D | Semaphorin 4D | Protein Coding | 42 | GC09M089360 | 6.667284012 |
| MIR608 | MicroRNA 608 | RNA Gene | 17 | GC10P100974 | 6.665950298 |
| MIRLET7A2 | MicroRNA Let-7a-2 | RNA Gene | 21 | GC11M122146 | 6.663298607 |
| HOXB2 | Homeobox B2 | Protein Coding | 35 | GC17M048540 | 6.661716938 |
| CRTC1 | CREB Regulated Transcription Coactivator 1 | Protein Coding | 37 | GC19P063129 | 6.658808708 |
| MIR346 | MicroRNA 346 | RNA Gene | 19 | GC10M086264 | 6.65846777 |
| ASPH | Aspartate Beta-Hydroxylase | Protein Coding | 39 | GC08M061500 | 6.656579494 |
| HDAC8 | Histone Deacetylase 8 | Protein Coding | 42 | GC0XM072329 | 6.656334877 |
| MIR129-2 | MicroRNA 129-2 | RNA Gene | 18 | GC11P043759 | 6.653608322 |
| PON2 | Paraoxonase 2 | Protein Coding | 40 | GC07M095404 | 6.650808334 |
| SLC7A5 | Solute Carrier Family 7 Member 5 | Protein Coding | 40 | GC16M087830 | 6.645465851 |
| MSI1 | Musashi RNA Binding Protein 1 | Protein Coding | 36 | GC12M120341 | 6.645336628 |
| KRT10 | Keratin 10 | Protein Coding | 39 | GC17M040818 | 6.641278267 |
| HIF1A-AS1 | HIF1A Antisense RNA 1 | RNA Gene | 15 | GC14M061681 | 6.636276245 |
| RUFY3 | RUN And FYVE Domain Containing 3 | Protein Coding | 31 | GC04P070704 | 6.634941578 |
| CTNNBIP1 | Catenin Beta Interacting Protein 1 | Protein Coding | 36 | GC01M009848 | 6.634362698 |
| ASAP1-IT1 | ASAP1 Intronic Transcript 1 | RNA Gene | 14 | GC08M130295 | 6.630529404 |
| SBDS | SBDS Ribosome Maturation Factor | Protein Coding | 37 | GC07M066987 | 6.630229473 |
| MAPRE1 | Microtubule Associated Protein RP/EB Family Member 1 | Protein Coding | 39 | GC20P032819 | 6.623738289 |
| CAPN9 | Calpain 9 | Protein Coding | 38 | GC01P230747 | 6.617971897 |
| HOXA11 | Homeobox A11 | Protein Coding | 39 | GC07M027548 | 6.615291595 |
| GSR | Glutathione-Disulfide Reductase | Protein Coding | 44 | GC08M030678 | 6.613465309 |
| LRP1 | LDL Receptor Related Protein 1 | Protein Coding | 43 | GC12P057128 | 6.609636307 |
| STEAP4 | STEAP4 Metalloreductase | Protein Coding | 36 | GC07M088381 | 6.608587742 |
| STAR | Steroidogenic Acute Regulatory Protein | Protein Coding | 41 | GC08M038145 | 6.608400345 |
| FPGS | Folylpolyglutamate Synthase | Protein Coding | 38 | GC09P127794 | 6.608267307 |
| HOXA9 | Homeobox A9 | Protein Coding | 37 | GC07M027162 | 6.603521347 |
| FBXO5 | F-Box Protein 5 | Protein Coding | 32 | GC06M152970 | 6.602089882 |
| POLR1H | RNA Polymerase I Subunit H | Protein Coding | 30 | GC06P083247 | 6.600543499 |
| SPN | Sialophorin | Protein Coding | 35 | GC16P029662 | 6.598854065 |
| IL12RB1 | Interleukin 12 Receptor Subunit Beta 1 | Protein Coding | 39 | GC19M018058 | 6.589980125 |
| RPL27 | Ribosomal Protein L27 | Protein Coding | 38 | GC17P042998 | 6.588600159 |
| LAMA1 | Laminin Subunit Alpha 1 | Protein Coding | 42 | GC18M006941 | 6.587780952 |
| PALS1 | Protein Associated With LIN7 1, MAGUK P55 Family Member | Protein Coding | 31 | GC14P067240 | 6.584627151 |
| LOC107303338 | 3p25 FANCD2 Alu-Mediated Recombination Region | Biological Region | 3 | GC03P012234 | 6.582858086 |
| GPX2 | Glutathione Peroxidase 2 | Protein Coding | 39 | GC14M064939 | 6.580753326 |
| CUBN | Cubilin | Protein Coding | 42 | GC10M016824 | 6.579249382 |
| CD226 | CD226 Molecule | Protein Coding | 38 | GC18M069831 | 6.578291893 |
| PTRH2 | Peptidyl-TRNA Hydrolase 2 | Protein Coding | 38 | GC17M059674 | 6.57806015 |
| ESRRB | Estrogen Related Receptor Beta | Protein Coding | 44 | GC14P076310 | 6.577076912 |
| SMARCAL1 | SWI/SNF Related, Matrix Associated, Actin Dependent Regulator Of Chromatin, Subfamily A Like 1 | Protein Coding | 39 | GC02P216412 | 6.574502468 |
| TRPM2-AS | TRPM2 Antisense RNA | RNA Gene | 13 | GC21M044414 | 6.572144508 |
| SGK3 | Serum/Glucocorticoid Regulated Kinase Family Member 3 | Protein Coding | 36 | GC08P066712 | 6.57076931 |
| EXOC2 | Exocyst Complex Component 2 | Protein Coding | 35 | GC06M000485 | 6.568500042 |
| HSPB2 | Heat Shock Protein Family B (Small) Member 2 | Protein Coding | 35 | GC11P111913 | 6.563469887 |
| PGAP3 | Post-GPI Attachment To Proteins Phospholipase 3 | Protein Coding | 34 | GC17M041535 | 6.563118458 |
| MICA | MHC Class I Polypeptide-Related Sequence A | Protein Coding | 35 | GC06P031399 | 6.56109333 |
| PDGFD | Platelet Derived Growth Factor D | Protein Coding | 38 | GC11M103907 | 6.556510448 |
| TBX5 | T-Box Transcription Factor 5 | Protein Coding | 42 | GC12M114353 | 6.555223942 |
| CRH | Corticotropin Releasing Hormone | Protein Coding | 39 | GC08M066176 | 6.554546356 |
| YWHAZ | Tyrosine 3-Monooxygenase/Tryptophan 5-Monooxygenase Activation Protein Zeta | Protein Coding | 42 | GC08M100917 | 6.550893307 |
| PDZD2 | PDZ Domain Containing 2 | Protein Coding | 34 | GC05P031639 | 6.549399853 |
| LINC01772 | Long Intergenic Non-Protein Coding RNA 1772 | RNA Gene | 12 | GC01P017689 | 6.540667534 |
| MIR199A2 | MicroRNA 199a-2 | RNA Gene | 19 | GC01M172235 | 6.539485931 |
| DAXX | Death Domain Associated Protein | Protein Coding | 38 | GC06M033318 | 6.526224136 |
| LINC00460 | Long Intergenic Non-Protein Coding RNA 460 | RNA Gene | 16 | GC13P106376 | 6.518608093 |
| PHF13 | PHD Finger Protein 13 | Protein Coding | 29 | GC01P006613 | 6.515906811 |
| EPHA1 | EPH Receptor A1 | Protein Coding | 42 | GC07M143390 | 6.515879631 |
| BCL9 | BCL9 Transcription Coactivator | Protein Coding | 37 | GC01P147541 | 6.514362335 |
| ANGPTL4 | Angiopoietin Like 4 | Protein Coding | 40 | GC19P008363 | 6.512877464 |
| TP53AIP1 | Tumor Protein P53 Regulated Apoptosis Inducing Protein 1 | Protein Coding | 34 | GC11M128934 | 6.51093626 |
| CDK2AP1 | Cyclin Dependent Kinase 2 Associated Protein 1 | Protein Coding | 32 | GC12M123250 | 6.510234833 |
| ADRB3 | Adrenoceptor Beta 3 | Protein Coding | 42 | GC08M037962 | 6.506612778 |
| MAGEC1 | MAGE Family Member C1 | Protein Coding | 31 | GC0XP141905 | 6.50625515 |
| LGALS4 | Galectin 4 | Protein Coding | 34 | GC19M063769 | 6.503265381 |
| RPS14 | Ribosomal Protein S14 | Protein Coding | 38 | GC05M150443 | 6.494033813 |
| NUP98 | Nucleoporin 98 And 96 Precursor | Protein Coding | 39 | GC11M003671 | 6.49320507 |
| SPAAR | Small Regulatory Polypeptide Of Amino Acid Response | Protein Coding | 14 | GC09P040175 | 6.490172863 |
| ACTG2 | Actin Gamma 2, Smooth Muscle | Protein Coding | 40 | GC02P073892 | 6.48871994 |
| IQANK1 | IQ Motif And Ankyrin Repeat Containing 1 | Protein Coding | 16 | GC08P144134 | 6.481222153 |
| RBM38 | RNA Binding Motif Protein 38 | Protein Coding | 34 | GC20P057391 | 6.479429245 |
| DPYSL5 | Dihydropyrimidinase Like 5 | Protein Coding | 36 | GC02P026847 | 6.478979111 |
| SCGB1D1 | Secretoglobin Family 1D Member 1 | Protein Coding | 25 | GC11P062208 | 6.476221085 |
| TNXB | Tenascin XB | Protein Coding | 40 | GC06M063627 | 6.476042747 |
| CD27 | CD27 Molecule | Protein Coding | 41 | GC12P019809 | 6.473137856 |
| PTBP1 | Polypyrimidine Tract Binding Protein 1 | Protein Coding | 38 | GC19P000797 | 6.468858719 |
| BMP8B | Bone Morphogenetic Protein 8b | Protein Coding | 35 | GC01M039757 | 6.465702057 |

| | | | | | |
|---|---|---|---|---|---|
| ALDH9A1 | Aldehyde Dehydrogenase 9 Family Member A1 | Protein Coding | 38 | GC01M165678 | 6.465052605 |
| LMNB2 | Lamin B2 | Protein Coding | 39 | GC19M004979 | 6.462639332 |
| TMCO1 | Transmembrane And Coiled-Coil Domains 1 | Protein Coding | 35 | GC01M165724 | 6.46018219 |
| FBXO24 | F-Box Protein 24 | Protein Coding | 31 | GC07P100982 | 6.458434105 |
| TERF2 | Telomeric Repeat Binding Factor 2 | Protein Coding | 36 | GC16M069355 | 6.456626892 |
| CYGB | Cytoglobin | Protein Coding | 34 | GC17M076527 | 6.45366621 |
| CD1A | CD1a Molecule | Protein Coding | 37 | GC01P158255 | 6.453397751 |
| B3GALT5 | Beta-1,3-Galactosyltransferase 5 | Protein Coding | 36 | GC21P039556 | 6.451593876 |
| RAPH1 | Ras Association (RalGDS/AF-6) And Pleckstrin Homology Domains 1 | Protein Coding | 34 | GC02M203394 | 6.449193001 |
| LOC110485084 | BRCA1 Intronic Recombination Region | Biological Region | 2 | GC17P053868 | 6.443403244 |
| THBD | Thrombomodulin | Protein Coding | 40 | GC20M023026 | 6.442495823 |
| CNDP2 | Carnosine Dipeptidase 2 | Protein Coding | 38 | GC18P074495 | 6.438565254 |
| CUL4B | Cullin 4B | Protein Coding | 38 | GC0XM120524 | 6.436822891 |
| PGK1 | Phosphoglycerate Kinase 1 | Protein Coding | 43 | GC0XP077987 | 6.436230659 |
| NPRL2 | NPR2 Like, GATOR1 Complex Subunit | Protein Coding | 35 | GC03M051111 | 6.435521126 |
| PRKG1 | Protein Kinase CGMP-Dependent 1 | Protein Coding | 45 | GC10P050991 | 6.434970379 |
| RPL35A | Ribosomal Protein L35a | Protein Coding | 38 | GC03P197949 | 6.434396744 |
| RAC2 | Rac Family Small GTPase 2 | Protein Coding | 46 | GC22M037227 | 6.428068161 |
| MC2R | Melanocortin 2 Receptor | Protein Coding | 43 | GC18M024023 | 6.427660942 |
| RPL18 | Ribosomal Protein L18 | Protein Coding | 39 | GC19M048615 | 6.426617146 |
| ITGA4 | Integrin Subunit Alpha 4 | Protein Coding | 43 | GC02P181456 | 6.419884682 |
| IL2RB | Interleukin 2 Receptor Subunit Beta | Protein Coding | 43 | GC22M037125 | 6.41898632 |
| LAMB1 | Laminin Subunit Beta 1 | Protein Coding | 44 | GC07M107923 | 6.416069984 |
| ESM1 | Endothelial Cell Specific Molecule 1 | Protein Coding | 35 | GC05M054977 | 6.415393829 |
| SOX11 | SRY-Box Transcription Factor 11 | Protein Coding | 37 | GC02P005703 | 6.413240433 |
| ATP2C1 | ATPase Secretory Pathway Ca2+ Transporting 1 | Protein Coding | 41 | GC03P130850 | 6.412360191 |
| UBE2K | Ubiquitin Conjugating Enzyme E2 K | Protein Coding | 35 | GC04P039700 | 6.411015511 |
| VCAN | Versican | Protein Coding | 43 | GC05P083471 | 6.409799576 |
| MMP8 | Matrix Metallopeptidase 8 | Protein Coding | 43 | GC11M102617 | 6.406680107 |
| GCM2 | Glial Cells Missing Transcription Factor 2 | Protein Coding | 36 | GC06M010873 | 6.399789333 |
| IGF2BP1 | Insulin Like Growth Factor 2 MRNA Binding Protein 1 | Protein Coding | 36 | GC17P048997 | 6.396470547 |
| SFTPC | Surfactant Protein C | Protein Coding | 38 | GC08P022156 | 6.396307945 |
| EIF1AX | Eukaryotic Translation Initiation Factor 1A X-Linked | Protein Coding | 35 | GC0XM020124 | 6.389025688 |
| CBS | Cystathionine Beta-Synthase | Protein Coding | 44 | GC21M043053 | 6.385053635 |
| MIR217 | MicroRNA 217 | RNA Gene | 19 | GC02M055982 | 6.377476692 |
| EPHA4 | EPH Receptor A4 | Protein Coding | 44 | GC02M221418 | 6.376830101 |
| CHD1L | Chromodomain Helicase DNA Binding Protein 1 Like | Protein Coding | 38 | GC01P147804 | 6.376507282 |
| RPS15A | Ribosomal Protein S15a | Protein Coding | 38 | GC16M018781 | 6.374907494 |
| MAGT1 | Magnesium Transporter 1 | Protein Coding | 36 | GC0XM077989 | 6.373488903 |
| CASP5 | Caspase 5 | Protein Coding | 39 | GC11M104995 | 6.373095512 |
| MAPK13 | Mitogen-Activated Protein Kinase 13 | Protein Coding | 43 | GC06P080527 | 6.370292664 |
| ARHGAP5 | Rho GTPase Activating Protein 5 | Protein Coding | 38 | GC14P032081 | 6.370249748 |
| PRODH | Proline Dehydrogenase 1 | Protein Coding | 42 | GC22M018912 | 6.369342327 |
| SNAPC5 | Small Nuclear RNA Activating Complex Polypeptide 5 | Protein Coding | 32 | GC15M081218 | 6.364650249 |
| DYRK1B | Dual Specificity Tyrosine Phosphorylation Regulated Kinase 1B | Protein Coding | 43 | GC19M039825 | 6.361574173 |
| HAMP | Hepcidin Antimicrobial Peptide | Protein Coding | 39 | GC19P063447 | 6.360945702 |
| ABCA4 | ATP Binding Cassette Subfamily A Member 4 | Protein Coding | 42 | GC01M093992 | 6.36042881 |
| NEK8 | NIMA Related Kinase 8 | Protein Coding | 37 | GC17P028725 | 6.358286381 |
| PFAS | Phosphoribosylformylglycinamidine Synthase | Protein Coding | 39 | GC17P008247 | 6.349948883 |
| ALOX15 | Arachidonate 15-Lipoxygenase | Protein Coding | 40 | GC17M004630 | 6.347595215 |
| SOX30 | SRY-Box Transcription Factor 30 | Protein Coding | 34 | GC05M157624 | 6.346601009 |
| ADA2 | Adenosine Deaminase 2 | Protein Coding | 33 | GC22M017192 | 6.346243382 |
| GATA6 | GATA Binding Protein 6 | Protein Coding | 42 | GC18P022169 | 6.343825817 |
| EIF2AK3 | Eukaryotic Translation Initiation Factor 2 Alpha Kinase 3 | Protein Coding | 43 | GC02M088556 | 6.342834473 |
| PLXND1 | Plexin D1 | Protein Coding | 38 | GC03M129555 | 6.335511208 |
| CHD4 | Chromodomain Helicase DNA Binding Protein 4 | Protein Coding | 40 | GC12M006570 | 6.332089424 |
| RPS6KA3 | Ribosomal Protein S6 Kinase A3 | Protein Coding | 47 | GC0XM020149 | 6.31340313 |
| COL3A1 | Collagen Type III Alpha 1 Chain | Protein Coding | 42 | GC02P188974 | 6.309891701 |
| ROR1 | Receptor Tyrosine Kinase Like Orphan Receptor 1 | Protein Coding | 42 | GC01P063774 | 6.309112072 |
| H4C1 | H4 Clustered Histone 1 | Protein Coding | 30 | GC06P079874 | 6.307826042 |
| PSMB8 | Proteasome 20S Subunit Beta 8 | Protein Coding | 45 | GC06M032840 | 6.30601263 |
| UGT1A8 | UDP Glucuronosyltransferase Family 1 Member A8 | Protein Coding | 35 | GC02P233618 | 6.305190086 |
| AKAP9 | A-Kinase Anchoring Protein 9 | Protein Coding | 40 | GC07P091940 | 6.303757668 |
| RALB | RAS Like Proto-Oncogene B | Protein Coding | 39 | GC02P120240 | 6.302879333 |
| RAB27A | RAB27A, Member RAS Oncogene Family | Protein Coding | 44 | GC15M055202 | 6.301903725 |
| SLC39A4 | Solute Carrier Family 39 Member 4 | Protein Coding | 38 | GC08M144409 | 6.300786495 |
| TYK2 | Tyrosine Kinase 2 | Protein Coding | 47 | GC19M010350 | 6.300185204 |
| PKP1 | Plakophilin 1 | Protein Coding | 38 | GC01P201283 | 6.29805088 |
| AQP1 | Aquaporin 1 (Colton Blood Group) | Protein Coding | 42 | GC07P030911 | 6.297172546 |
| SERPINB4 | Serpin Family B Member 4 | Protein Coding | 32 | GC18M063637 | 6.292399883 |
| MSX1 | Msh Homeobox 1 | Protein Coding | 41 | GC04P004861 | 6.29077816 |
| OCIAD2 | OCIA Domain Containing 2 | Protein Coding | 32 | GC04M048887 | 6.290686607 |
| MIR422A | MicroRNA 422a | RNA Gene | 16 | GC15M063870 | 6.290610313 |
| RPS17 | Ribosomal Protein S17 | Protein Coding | 38 | GC15M082536 | 6.289994717 |
| CD38 | CD38 Molecule | Protein Coding | 41 | GC04P017742 | 6.289700508 |
| PRSS21 | Serine Protease 21 | Protein Coding | 38 | GC16P010749 | 6.289362431 |
| EDARADD | EDAR Associated Death Domain | Protein Coding | 35 | GC01P236348 | 6.287091255 |
| BIRC6 | Baculoviral IAP Repeat Containing 6 | Protein Coding | 38 | GC02P032509 | 6.285939217 |
| IL16 | Interleukin 16 | Protein Coding | 36 | GC15P081159 | 6.28391695 |
| NEK3 | NIMA Related Kinase 3 | Protein Coding | 37 | GC13M052132 | 6.282181263 |
| GEN1 | GEN1 Holliday Junction 5' Flap Endonuclease | Protein Coding | 35 | GC02P017754 | 6.27959156 |
| FAM215A | Family With Sequence Similarity 215 Member A | RNA Gene | 19 | GC17P043917 | 6.278765202 |
| IL33 | Interleukin 33 | Protein Coding | 34 | GC09P006440 | 6.278607368 |
| MICB | MHC Class I Polypeptide-Related Sequence B | Protein Coding | 37 | GC06P080348 | 6.274959564 |
| CXCL13 | C-X-C Motif Chemokine Ligand 13 | Protein Coding | 36 | GC04P077511 | 6.271755695 |
| DUXAP10 | Double Homeobox A Pseudogene 10 | Pseudogene | 10 | GC14M019281 | 6.266233444 |
| CTBP1-AS | CTBP1 Antisense RNA | RNA Gene | 13 | GC04P001210 | 6.26315403 |
| CACNA1G | Calcium Voltage-Gated Channel Subunit Alpha1 G | Protein Coding | 44 | GC17P053571 | 6.26262188 |
| RSPO2 | R-Spondin 2 | Protein Coding | 39 | GC08M107899 | 6.261182785 |
| MUC5B | Mucin 5B, Oligomeric Mucus/Gel-Forming | Protein Coding | 38 | GC11P001703 | 6.258310318 |
| ITGAM | Integrin Subunit Alpha M | Protein Coding | 43 | GC16P040304 | 6.254763603 |
| ITGA11 | Integrin Subunit Alpha 11 | Protein Coding | 39 | GC15M068296 | 6.254402637 |
| NMBR | Neuromedin B Receptor | Protein Coding | 38 | GC06M142059 | 6.254154205 |
| GNB3 | G Protein Subunit Beta 3 | Protein Coding | 42 | GC12P006839 | 6.252852917 |
| ELAPOR1 | Endosome-Lysosome Associated Apoptosis And Autophagy Regulator 1 | Protein Coding | 27 | GC01P109181 | 6.250524044 |

| | | | | | |
|---|---|---|---|---|---|
| BGN | Biglycan | Protein Coding | 39 | GC0XP153494 | 6.242666721 |
| DUS2 | Dihydrouridine Synthase 2 | Protein Coding | 32 | GC16P067987 | 6.241945267 |
| JAZF1 | JAZF Zinc Finger 1 | Protein Coding | 34 | GC07M027830 | 6.241295338 |
| TERF1 | Telomeric Repeat Binding Factor 1 | Protein Coding | 37 | GC08P073003 | 6.23955822 |
| ZFPM2 | Zinc Finger Protein, FOG Family Member 2 | Protein Coding | 38 | GC08P104590 | 6.239160538 |
| NR4A1 | Nuclear Receptor Subfamily 4 Group A Member 1 | Protein Coding | 42 | GC12P052022 | 6.235080242 |
| ISG15 | ISG15 Ubiquitin Like Modifier | Protein Coding | 40 | GC01P001001 | 6.233696938 |
| C3 | Complement C3 | Protein Coding | 43 | GC19M006677 | 6.232787132 |
| VCL | Vinculin | Protein Coding | 42 | GC10P073995 | 6.230985641 |
| PHB2 | Prohibitin 2 | Protein Coding | 37 | GC12M006965 | 6.222862244 |
| USP33 | Ubiquitin Specific Peptidase 33 | Protein Coding | 35 | GC01M077695 | 6.219772816 |
| NDC80 | NDC80 Kinetochore Complex Component | Protein Coding | 36 | GC18P002571 | 6.218140125 |
| NCL | Nucleolin | Protein Coding | 37 | GC02M231453 | 6.217961311 |
| FAM98B | Family With Sequence Similarity 98 Member B | Protein Coding | 28 | GC15P038454 | 6.217858791 |
| SKIV2L | Ski2 Like RNA Helicase | Protein Coding | 39 | GC06P080379 | 6.217169762 |
| CHRNB4 | Cholinergic Receptor Nicotinic Beta 4 Subunit | Protein Coding | 39 | GC15M081615 | 6.216554642 |
| TCF12 | Transcription Factor 12 | Protein Coding | 42 | GC15P056918 | 6.213757515 |
| OCLN | Occludin | Protein Coding | 39 | GC05P069492 | 6.211278439 |
| H3C1 | H3 Clustered Histone 1 | Protein Coding | 31 | GC06P079873 | 6.208534718 |
| MIR625 | MicroRNA 625 | RNA Gene | 15 | GC14P065471 | 6.208465576 |
| MIR515-1 | MicroRNA 515-1 | RNA Gene | 15 | GC19P053679 | 6.20702076 |
| BACE1-AS | BACE1 Antisense RNA | RNA Gene | 14 | GC11P117348 | 6.203927994 |
| LMO1 | LIM Domain Only 1 | Protein Coding | 38 | GC11M008224 | 6.20381546 |
| TUBG1 | Tubulin Gamma 1 | Protein Coding | 42 | GC17P042609 | 6.201536179 |
| TNK2 | Tyrosine Kinase Non Receptor 2 | Protein Coding | 41 | GC03M195863 | 6.200665474 |
| SLC35E3 | Solute Carrier Family 35 Member E3 | Protein Coding | 30 | GC12P068746 | 6.198534012 |
| GRIN2B | Glutamate Ionotropic Receptor NMDA Type Subunit 2B | Protein Coding | 46 | GC12M013437 | 6.195726395 |
| MUC12 | Mucin 12, Cell Surface Associated | Protein Coding | 30 | GC07P100969 | 6.195412159 |
| ASS1 | Argininosuccinate Synthase 1 | Protein Coding | 43 | GC09P130444 | 6.195309639 |
| CRX | Cone-Rod Homeobox | Protein Coding | 38 | GC19P047819 | 6.193824768 |
| ABCC4 | ATP Binding Cassette Subfamily C Member 4 | Protein Coding | 42 | GC13M095019 | 6.190705299 |
| S100A11 | S100 Calcium Binding Protein A11 | Protein Coding | 38 | GC01M152032 | 6.188381195 |
| DNAH8 | Dynein Axonemal Heavy Chain 8 | Protein Coding | 34 | GC06P080553 | 6.186742783 |
| DRD2 | Dopamine Receptor D2 | Protein Coding | 45 | GC11M113409 | 6.182799339 |
| FAT4 | FAT Atypical Cadherin 4 | Protein Coding | 36 | GC04P125315 | 6.181786537 |
| SFRP5 | Secreted Frizzled Related Protein 5 | Protein Coding | 35 | GC10M097766 | 6.179321289 |
| TYRP1 | Tyrosinase Related Protein 1 | Protein Coding | 42 | GC09P012683 | 6.17912674 |
| CYP2A13 | Cytochrome P450 Family 2 Subfamily A Member 13 | Protein Coding | 39 | GC19P041088 | 6.177695751 |
| RPTOR | Regulatory Associated Protein Of MTOR Complex 1 | Protein Coding | 38 | GC17P080544 | 6.175000191 |
| MAP3K20 | Mitogen-Activated Protein Kinase Kinase Kinase 20 | Protein Coding | 39 | GC02P173076 | 6.174898624 |
| IL12A | Interleukin 12A | Protein Coding | 39 | GC03P159988 | 6.172174931 |
| LRBA | LPS Responsive Beige-Like Anchor Protein | Protein Coding | 37 | GC04M150264 | 6.171669483 |
| HSPG2 | Heparan Sulfate Proteoglycan 2 | Protein Coding | 43 | GC01M021822 | 6.171652794 |
| KIF14 | Kinesin Family Member 14 | Protein Coding | 36 | GC01M200551 | 6.159959793 |
| PRAC2 | PRAC2 Small Nuclear Protein | Protein Coding | 18 | GC17P048720 | 6.15749836 |
| PHF6 | PHD Finger Protein 6 | Protein Coding | 36 | GC0XP134373 | 6.149852753 |
| BMPR1B | Bone Morphogenetic Protein Receptor Type 1B | Protein Coding | 46 | GC04P094757 | 6.145461082 |
| TNFSF12 | TNF Superfamily Member 12 | Protein Coding | 38 | GC17P010881 | 6.142782211 |
| NUDT6 | Nudix Hydrolase 6 | Protein Coding | 34 | GC04M122888 | 6.141020775 |
| CD163 | CD163 Molecule | Protein Coding | 39 | GC12M007715 | 6.140457153 |
| GPSM2 | G Protein Signaling Modulator 2 | Protein Coding | 38 | GC01P108875 | 6.140080452 |
| PKIB | CAMP-Dependent Protein Kinase Inhibitor Beta | Protein Coding | 34 | GC06P122472 | 6.139672279 |
| REG1A | Regenerating Family Member 1 Alpha | Protein Coding | 36 | GC02P079120 | 6.138768196 |
| SLC6A4 | Solute Carrier Family 6 Member 4 | Protein Coding | 43 | GC17M030194 | 6.138628006 |
| RAG2 | Recombination Activating 2 | Protein Coding | 38 | GC11M036575 | 6.127038956 |
| LLGL1 | LLGL Scribble Cell Polarity Complex Component 1 | Protein Coding | 36 | GC17P018225 | 6.126463413 |
| SLC16A4 | Solute Carrier Family 16 Member 4 | Protein Coding | 35 | GC01M110362 | 6.121709824 |
| ZFYVE26 | Zinc Finger FYVE-Type Containing 26 | Protein Coding | 35 | GC14M067727 | 6.119197845 |
| KMT2B | Lysine Methyltransferase 2B | Protein Coding | 35 | GC19P063458 | 6.116823196 |
| ZNRF3 | Zinc And Ring Finger 3 | Protein Coding | 32 | GC22P028883 | 6.108936787 |
| CPA4 | Carboxypeptidase A4 | Protein Coding | 38 | GC07P130293 | 6.108080864 |
| IGSF8 | Immunoglobulin Superfamily Member 8 | Protein Coding | 35 | GC01M160061 | 6.107866764 |
| TCF15 | Transcription Factor 15 | Protein Coding | 30 | GC20M000603 | 6.105771065 |
| GUCY2C | Guanylate Cyclase 2C | Protein Coding | 41 | GC12M014612 | 6.101464748 |
| NRIP1 | Nuclear Receptor Interacting Protein 1 | Protein Coding | 39 | GC21M014961 | 6.100701332 |
| PYY | Peptide YY | Protein Coding | 39 | GC17M043952 | 6.093086243 |
| ZNF224 | Zinc Finger Protein 224 | Protein Coding | 34 | GC19P044094 | 6.088912964 |
| THBS3 | Thrombospondin 3 | Protein Coding | 37 | GC01M155195 | 6.087760448 |
| COX6C | Cytochrome C Oxidase Subunit 6C | Protein Coding | 35 | GC08M099899 | 6.085790634 |
| MGAT4A | Alpha-1,3-Mannosyl-Glycoprotein 4-Beta-N-Acetylglucosaminyltransferase A | Protein Coding | 35 | GC02M098619 | 6.083592415 |
| CD70 | CD70 Molecule | Protein Coding | 38 | GC19M006638 | 6.082832336 |
| BCL11A | BAF Chromatin Remodeling Complex Subunit BCL11A | Protein Coding | 39 | GC02M060451 | 6.081205845 |
| ZKSCAN1 | Zinc Finger With KRAB And SCAN Domains 1 | Protein Coding | 33 | GC07P100015 | 6.075387478 |
| HACE1 | HECT Domain And Ankyrin Repeat Containing E3 Ubiquitin Protein Ligase 1 | Protein Coding | 39 | GC06M104728 | 6.074854374 |
| RND2 | Rho Family GTPase 2 | Protein Coding | 32 | GC17P053180 | 6.074775696 |
| BNC1 | Basonuclin 1 | Protein Coding | 32 | GC15M083255 | 6.063268661 |
| CD99 | CD99 Molecule (Xg Blood Group) | Protein Coding | 35 | GC0XP002691 | 6.062849522 |
| LINC00312 | Long Intergenic Non-Protein Coding RNA 312 | RNA Gene | 18 | GC03P008571 | 6.050906181 |
| TBX3-AS1 | TBX3 Antisense RNA 1 | RNA Gene | 12 | GC12P114761 | 6.04972887 |
| FOXP2 | Forkhead Box P2 | Protein Coding | 39 | GC07P114086 | 6.049264908 |
| PPP2R5E | Protein Phosphatase 2 Regulatory Subunit B'Epsilon | Protein Coding | 36 | GC14M063371 | 6.049160004 |
| PYCARD | PYD And CARD Domain Containing | Protein Coding | 37 | GC16M031201 | 6.045487404 |
| SAPCD2 | Suppressor APC Domain Containing 2 | Protein Coding | 27 | GC09M137062 | 6.040593147 |
| PLEKHA8 | Pleckstrin Homology Domain Containing A8 | Protein Coding | 32 | GC07P030027 | 6.030469418 |
| FAM210B | Family With Sequence Similarity 210 Member B | Protein Coding | 28 | GC20P056358 | 6.030200958 |
| ZFP36L2 | ZFP36 Ring Finger Protein Like 2 | Protein Coding | 34 | GC02M043184 | 6.029278755 |
| NSUN2 | NOP2/Sun RNA Methyltransferase 2 | Protein Coding | 38 | GC05M006599 | 6.022280693 |
| CTCFL | CCCTC-Binding Factor Like | Protein Coding | 36 | GC20M057495 | 6.021527767 |
| SLC16A1 | Solute Carrier Family 16 Member 1 | Protein Coding | 43 | GC01M112967 | 6.018291473 |
| SSTR5 | Somatostatin Receptor 5 | Protein Coding | 40 | GC16P001072 | 6.016859055 |
| FUT6 | Fucosyltransferase 6 | Protein Coding | 38 | GC19M005830 | 6.016310692 |
| PLA2G4A | Phospholipase A2 Group IVA | Protein Coding | 44 | GC01P186798 | 6.012000084 |
| CCR2 | C-C Motif Chemokine Receptor 2 | Protein Coding | 40 | GC03P046443 | 6.011921883 |
| PATE1 | Prostate And Testis Expressed 1 | Protein Coding | 27 | GC11P125746 | 6.01054287 |
| ADM | Adrenomedullin | Protein Coding | 40 | GC11P010304 | 6.009471893 |

| | | | | | |
|---|---|---|---|---|---|
| RPS6 | Ribosomal Protein S6 | Protein Coding | 38 | GC09M019375 | 6.006762028 |
| NDFIP1 | Nedd4 Family Interacting Protein 1 | Protein Coding | 34 | GC05P142108 | 6.001320839 |
| REEP5 | Receptor Accessory Protein 5 | Protein Coding | 36 | GC05M112876 | 6.001241207 |
| HOXA13 | Homeobox A13 | Protein Coding | 39 | GC07M027552 | 6.000123024 |
| CBFA2T3 | CBFA2/RUNX1 Partner Transcriptional Co-Repressor 3 | Protein Coding | 38 | GC16M088874 | 5.999666214 |
| SAG | S-Antigen Visual Arrestin | Protein Coding | 40 | GC02P233377 | 5.994582176 |
| SNHG14 | Small Nucleolar RNA Host Gene 14 | RNA Gene | 16 | GC15P039149 | 5.992725372 |
| EAF2 | ELL Associated Factor 2 | Protein Coding | 34 | GC03P121835 | 5.992137432 |
| CLTC | Clathrin Heavy Chain | Protein Coding | 41 | GC17P059619 | 5.991381645 |
| UNC93A | Unc-93 Homolog A | Protein Coding | 31 | GC06P167271 | 5.989351273 |
| TAP1 | Transporter 1, ATP Binding Cassette Subfamily B Member | Protein Coding | 42 | GC06M063671 | 5.98559618 |
| RNF14 | Ring Finger Protein 14 | Protein Coding | 37 | GC05P141958 | 5.983480453 |
| DERPC | DERPC Proline And Glycine Rich Nuclear Protein | Protein Coding | 11 | GC16M069118 | 5.981781006 |
| HTRA3 | HtrA Serine Peptidase 3 | Protein Coding | 36 | GC04P008269 | 5.980751991 |
| AHCY | Adenosylhomocysteinase | Protein Coding | 44 | GC20M034771 | 5.980639935 |
| KRT4 | Keratin 4 | Protein Coding | 39 | GC12M052806 | 5.980185509 |
| ARHGEF26 | Rho Guanine Nucleotide Exchange Factor 26 | Protein Coding | 31 | GC03P154124 | 5.979557514 |
| UBA7 | Ubiquitin Like Modifier Activating Enzyme 7 | Protein Coding | 36 | GC03M049805 | 5.978804588 |
| PRMT5 | Protein Arginine Methyltransferase 5 | Protein Coding | 39 | GC14M022920 | 5.976576805 |
| LOC107988030 | MITF-M Promoter Region | Biological Region | 2 | GC03P069934 | 5.975605488 |
| LAMTOR5 | Late Endosomal/Lysosomal Adaptor, MAPK And MTOR Activator 5 | Protein Coding | 31 | GC01M110401 | 5.975244045 |
| PPP1R3A | Protein Phosphatase 1 Regulatory Subunit 3A | Protein Coding | 38 | GC07M113876 | 5.973248482 |
| METTL3 | Methyltransferase 3, N6-Adenosine-Methyltransferase Complex Catalytic Subunit | Protein Coding | 36 | GC14M021498 | 5.972707272 |
| RPS28 | Ribosomal Protein S28 | Protein Coding | 35 | GC19P008383 | 5.97147274 |
| MAGEA11 | MAGE Family Member A11 | Protein Coding | 33 | GC0XP149688 | 5.970114708 |
| ST6GAL1 | ST6 Beta-Galactoside Alpha-2,6-Sialyltransferase 1 | Protein Coding | 39 | GC03P186930 | 5.96816349 |
| TNFRSF12A | TNF Receptor Superfamily Member 12A | Protein Coding | 38 | GC16P003018 | 5.966627121 |
| TNFSF15 | TNF Superfamily Member 15 | Protein Coding | 39 | GC09M114784 | 5.962240219 |
| NOP14 | NOP14 Nucleolar Protein | Protein Coding | 31 | GC04M002939 | 5.95992136 |
| MIR508 | MicroRNA 508 | RNA Gene | 16 | GC0XM147236 | 5.959515572 |
| SEMA3E | Semaphorin 3E | Protein Coding | 39 | GC07M083363 | 5.955442905 |
| AOC3 | Amine Oxidase Copper Containing 3 | Protein Coding | 40 | GC17P042851 | 5.955434799 |
| HEIH | Hepatocellular Carcinoma Up-Regulated EZH2-Associated Long Non-Coding RNA | RNA Gene | 15 | GC05M181695 | 5.951296329 |
| MRPL23 | Mitochondrial Ribosomal Protein L23 | Protein Coding | 32 | GC11P001948 | 5.950028419 |
| LIG3 | DNA Ligase 3 | Protein Coding | 42 | GC17P034980 | 5.947631359 |
| MSI2 | Musashi RNA Binding Protein 2 | Protein Coding | 36 | GC17P057255 | 5.946894646 |
| SPANXC | SPANX Family Member C | Protein Coding | 30 | GC0XM141241 | 5.941615582 |
| OR4C5 | Olfactory Receptor Family 4 Subfamily C Member 5 | Protein Coding | 21 | GC11M086899 | 5.940908432 |
| SIK3 | SIK Family Kinase 3 | Protein Coding | 38 | GC11M116843 | 5.934942245 |
| F8 | Coagulation Factor VIII | Protein Coding | 42 | GC0XM154835 | 5.930419922 |
| GTF2I | General Transcription Factor IIi | Protein Coding | 37 | GC07P074838 | 5.930212498 |
| GSTA1 | Glutathione S-Transferase Alpha 1 | Protein Coding | 38 | GC06M052791 | 5.927490711 |
| CACNA2D2 | Calcium Voltage-Gated Channel Auxiliary Subunit Alpha2delta 2 | Protein Coding | 38 | GC03M051113 | 5.927276134 |
| HAND2-AS1 | HAND2 Antisense RNA 1 | RNA Gene | 19 | GC04P173527 | 5.927055359 |
| CHMP2A | Charged Multivesicular Body Protein 2A | Protein Coding | 34 | GC19M058551 | 5.922774792 |
| SNHG3 | Small Nucleolar RNA Host Gene 3 | RNA Gene | 18 | GC01P028506 | 5.920217991 |
| GNPTAB | N-Acetylglucosamine-1-Phosphate Transferase Subunits Alpha And Beta | Protein Coding | 38 | GC12M101745 | 5.919965744 |
| AGAP2 | ArfGAP With GTPase Domain, Ankyrin Repeat And PH Domain 2 | Protein Coding | 36 | GC12M057723 | 5.919190407 |
| ARG1 | Arginase 1 | Protein Coding | 44 | GC06P131473 | 5.914470673 |
| MTA3 | Metastasis Associated 1 Family Member 3 | Protein Coding | 37 | GC02P042494 | 5.913146973 |
| DMTN | Dematin Actin Binding Protein | Protein Coding | 32 | GC08P022048 | 5.912426949 |
| KDM5A | Lysine Demethylase 5A | Protein Coding | 38 | GC12M000280 | 5.910520554 |
| TRIM33 | Tripartite Motif Containing 33 | Protein Coding | 39 | GC01M114392 | 5.909314156 |
| IAPP | Islet Amyloid Polypeptide | Protein Coding | 38 | GC12P021354 | 5.9057827 |
| ITIH5 | Inter-Alpha-Trypsin Inhibitor Heavy Chain 5 | Protein Coding | 34 | GC10M007559 | 5.902494431 |
| F13A1 | Coagulation Factor XIII A Chain | Protein Coding | 42 | GC06M006144 | 5.902433395 |
| TBX1 | T-Box Transcription Factor 1 | Protein Coding | 38 | GC22P034407 | 5.897610664 |
| NR0B2 | Nuclear Receptor Subfamily 0 Group B Member 2 | Protein Coding | 40 | GC01M027086 | 5.897177696 |
| BARHL1 | BarH Like Homeobox 1 | Protein Coding | 31 | GC09P132582 | 5.895665169 |
| PLXNA1 | Plexin A1 | Protein Coding | 38 | GC03P126988 | 5.893672466 |
| AIM2 | Absent In Melanoma 2 | Protein Coding | 37 | GC01M159062 | 5.893177509 |
| PTPRK | Protein Tyrosine Phosphatase Receptor Type K | Protein Coding | 40 | GC06M127949 | 5.892382622 |
| TARS1 | Threonyl-TRNA Synthetase 1 | Protein Coding | 35 | GC05P033441 | 5.886509418 |
| PLIN2 | Perilipin 2 | Protein Coding | 37 | GC09M019163 | 5.884376049 |
| LAMA4 | Laminin Subunit Alpha 4 | Protein Coding | 41 | GC06M112107 | 5.883970261 |
| CHD5 | Chromodomain Helicase DNA Binding Protein 5 | Protein Coding | 37 | GC01M006104 | 5.883575916 |
| NUAK1 | NUAK Family Kinase 1 | Protein Coding | 40 | GC12M106063 | 5.882536888 |
| UBD | Ubiquitin D | Protein Coding | 35 | GC06M063475 | 5.881072998 |
| MIR1207 | MicroRNA 1207 | RNA Gene | 15 | GC08P128049 | 5.880541801 |
| SDC2 | Syndecan 2 | Protein Coding | 40 | GC08P096496 | 5.877084255 |
| LINC00963 | Long Intergenic Non-Protein Coding RNA 963 | RNA Gene | 14 | GC09P129483 | 5.871235371 |
| TIGAR | TP53 Induced Glycolysis Regulatory Phosphatase | Protein Coding | 33 | GC12P019786 | 5.867911816 |
| OGFR | Opioid Growth Factor Receptor | Protein Coding | 34 | GC20P062804 | 5.865734577 |
| SREBF2 | Sterol Regulatory Element Binding Transcription Factor 2 | Protein Coding | 38 | GC22P041833 | 5.861398697 |
| SPIDR | Scaffold Protein Involved In DNA Repair | Protein Coding | 30 | GC08P047260 | 5.859302998 |
| PRRC2A | Proline Rich Coiled-Coil 2A | Protein Coding | 30 | GC06P080356 | 5.856046677 |
| GLS | Glutaminase | Protein Coding | 44 | GC02P190880 | 5.849218845 |
| AVPR2 | Arginine Vasopressin Receptor 2 | Protein Coding | 42 | GC0XP153902 | 5.846935272 |
| RIT1 | Ras Like Without CAAX 1 | Protein Coding | 42 | GC01M155897 | 5.841966629 |
| RXFP2 | Relaxin Family Peptide Receptor 2 | Protein Coding | 38 | GC13P031739 | 5.841170311 |
| RPL31 | Ribosomal Protein L31 | Protein Coding | 37 | GC02P100985 | 5.839569569 |
| EIF3M | Eukaryotic Translation Initiation Factor 3 Subunit M | Protein Coding | 34 | GC11P032585 | 5.837485313 |
| KLK14 | Kallikrein Related Peptidase 14 | Protein Coding | 35 | GC19M051077 | 5.835551262 |
| RDH11 | Retinol Dehydrogenase 11 | Protein Coding | 40 | GC14M067676 | 5.830632687 |
| PFKFB4 | 6-Phosphofructo-2-Kinase/Fructose-2,6-Biphosphatase 4 | Protein Coding | 38 | GC03M048517 | 5.828817368 |
| TMSB4X | Thymosin Beta 4 X-Linked | Protein Coding | 36 | GC0XP012975 | 5.827301502 |
| POLR3H | RNA Polymerase III Subunit H | Protein Coding | 34 | GC22M041525 | 5.82535553 |
| TGM1 | Transglutaminase 1 | Protein Coding | 42 | GC14M024249 | 5.824409485 |
| CXCL9 | C-X-C Motif Chemokine Ligand 9 | Protein Coding | 36 | GC04M076001 | 5.822248936 |
| DDX53 | DEAD-Box Helicase 53 | Protein Coding | 31 | GC0XP022999 | 5.816867828 |
| TDRG1 | Testis Development Related 1 | RNA Gene | 22 | GC06P080563 | 5.816280365 |
| MACF1 | Microtubule Actin Crosslinking Factor 1 | Protein Coding | 38 | GC01P039082 | 5.815355778 |
| RACGAP1 | Rac GTPase Activating Protein 1 | Protein Coding | 39 | GC12M049978 | 5.81319046 |
| CX3CR1 | C-X3-C Motif Chemokine Receptor 1 | Protein Coding | 39 | GC03M039279 | 5.810565472 |
| REPS2 | RALBP1 Associated Eps Domain Containing 2 | Protein Coding | 34 | GC0XP016946 | 5.806036949 |

| | | | | | |
|---|---|---|---|---|---|
| PA2G4 | Proliferation-Associated 2G4 | Protein Coding | 36 | GC12P057155 | 5.80457592 |
| ABHD11-AS1 | ABHD11 Antisense RNA 1 (Tail To Tail) | RNA Gene | 15 | GC07P074792 | 5.799901962 |
| TMEM238L | Transmembrane Protein 238 Like | Protein Coding | 14 | GC17M010795 | 5.797876835 |
| LAMP2 | Lysosomal Associated Membrane Protein 2 | Protein Coding | 39 | GC0XM120426 | 5.796406746 |
| RRP7A | Ribosomal RNA Processing 7 Homolog A | Protein Coding | 36 | GC22M042508 | 5.795001507 |
| COTL1 | Coactosin Like F-Actin Binding Protein 1 | Protein Coding | 36 | GC16M084566 | 5.79369545 |
| LINC00473 | Long Intergenic Non-Protein Coding RNA 473 | RNA Gene | 19 | GC06M165328 | 5.792210102 |
| H1-3 | H1.3 Linker Histone, Cluster Member | Protein Coding | 30 | GC06M064151 | 5.790483475 |
| MLST8 | MTOR Associated Protein, LST8 Homolog | Protein Coding | 36 | GC16P002204 | 5.789827824 |
| TMPRSS6 | Transmembrane Serine Protease 6 | Protein Coding | 40 | GC22M037068 | 5.789143562 |
| CRISP3 | Cysteine Rich Secretory Protein 3 | Protein Coding | 34 | GC06M049727 | 5.788677216 |
| KDM3A | Lysine Demethylase 3A | Protein Coding | 36 | GC02P086440 | 5.788064003 |
| PI16 | Peptidase Inhibitor 16 | Protein Coding | 34 | GC06P080541 | 5.78638649 |
| TGM3 | Transglutaminase 3 | Protein Coding | 39 | GC20P002296 | 5.78629303 |
| PGA3 | Pepsinogen A3 | Protein Coding | 30 | GC11P061203 | 5.785113811 |
| SIK1 | Salt Inducible Kinase 1 | Protein Coding | 42 | GC21M043414 | 5.784783363 |
| BATF2 | Basic Leucine Zipper ATF-Like Transcription Factor 2 | Protein Coding | 32 | GC11M064987 | 5.780665398 |
| SDCBP | Syndecan Binding Protein | Protein Coding | 36 | GC08P058539 | 5.779524803 |
| HTATIP2 | HIV-1 Tat Interactive Protein 2 | Protein Coding | 36 | GC11P020363 | 5.77556324 |
| KIF11 | Kinesin Family Member 11 | Protein Coding | 43 | GC10P092574 | 5.774403095 |
| MIR9-2 | MicroRNA 9-2 | RNA Gene | 19 | GC05M088666 | 5.771593094 |
| DACT1 | Dishevelled Binding Antagonist Of Beta Catenin 1 | Protein Coding | 36 | GC14P058633 | 5.770258427 |
| IL9 | Interleukin 9 | Protein Coding | 38 | GC05M135891 | 5.769422531 |
| OPRM1 | Opioid Receptor Mu 1 | Protein Coding | 43 | GC06P154075 | 5.76852417 |
| PRKCZ | Protein Kinase C Zeta | Protein Coding | 44 | GC01P002050 | 5.767866611 |
| CCL27 | C-C Motif Chemokine Ligand 27 | Protein Coding | 34 | GC09M034662 | 5.765845299 |
| CARS1 | Cysteinyl-TRNA Synthetase 1 | Protein Coding | 32 | GC11M003000 | 5.763292789 |
| LDLR | Low Density Lipoprotein Receptor | Protein Coding | 46 | GC19P011091 | 5.762818813 |
| RPL34-DT | RPL34 Divergent Transcript | RNA Gene | 17 | GC04M108539 | 5.762095451 |
| GAS8 | Growth Arrest Specific 8 | Protein Coding | 35 | GC16P090019 | 5.758178711 |
| TTC7A | Tetratricopeptide Repeat Domain 7A | Protein Coding | 34 | GC02P046906 | 5.757279873 |
| ID3 | Inhibitor Of DNA Binding 3, HLH Protein | Protein Coding | 36 | GC01M023557 | 5.754152298 |
| NKD1 | NKD Inhibitor Of WNT Signaling Pathway 1 | Protein Coding | 31 | GC16P050548 | 5.753753185 |
| THRSP | Thyroid Hormone Responsive | Protein Coding | 36 | GC11P078063 | 5.750033855 |
| SI | Sucrase-Isomaltase | Protein Coding | 40 | GC03M164978 | 5.746524811 |
| RUNX1T1 | RUNX1 Partner Transcriptional Co-Repressor 1 | Protein Coding | 38 | GC08M091954 | 5.742425442 |
| HEY1 | Hes Related Family BHLH Transcription Factor With YRPW Motif 1 | Protein Coding | 38 | GC08M079764 | 5.738975048 |
| DEPDC1 | DEP Domain Containing 1 | Protein Coding | 31 | GC01M068474 | 5.737795353 |
| LPXN | Leupaxin | Protein Coding | 36 | GC11M086973 | 5.737317085 |
| DHDH | Dihydrodiol Dehydrogenase | Protein Coding | 34 | GC19P064029 | 5.73726368 |
| PBX1 | PBX Homeobox 1 | Protein Coding | 44 | GC01P164524 | 5.736215591 |
| NFATC1 | Nuclear Factor Of Activated T Cells 1 | Protein Coding | 42 | GC18P079395 | 5.735805511 |
| IHH | Indian Hedgehog Signaling Molecule | Protein Coding | 42 | GC02M219054 | 5.731611252 |
| MIR511 | MicroRNA 511 | RNA Gene | 15 | GC10P017845 | 5.730820656 |
| CPOX | Coproporphyrinogen Oxidase | Protein Coding | 39 | GC03M098576 | 5.727603912 |
| TRRAP | Transformation/Transcription Domain Associated Protein | Protein Coding | 40 | GC07P098877 | 5.727334976 |
| CYP2C8 | Cytochrome P450 Family 2 Subfamily C Member 8 | Protein Coding | 43 | GC10M095038 | 5.72641468 |
| STN1 | STN1 Subunit Of CST Complex | Protein Coding | 32 | GC10M103922 | 5.725865364 |
| PAPPA | Pappalysin 1 | Protein Coding | 39 | GC09P118637 | 5.725562572 |
| RPL35 | Ribosomal Protein L35 | Protein Coding | 39 | GC09M124857 | 5.725524426 |
| SCTR | Secretin Receptor | Protein Coding | 38 | GC02M119439 | 5.724347591 |
| CBX4 | Chromobox 4 | Protein Coding | 37 | GC17M079833 | 5.722150803 |
| AGT | Angiotensinogen | Protein Coding | 44 | GC01M230702 | 5.720733643 |
| USH2A | Usherin | Protein Coding | 35 | GC01M215622 | 5.720704079 |
| UGT1A4 | UDP Glucuronosyltransferase Family 1 Member A4 | Protein Coding | 38 | GC02P233718 | 5.712836266 |
| VOPP1 | VOPP1 WW Domain Binding Protein | Protein Coding | 28 | GC07M055434 | 5.712337971 |
| PLA2G1B | Phospholipase A2 Group IB | Protein Coding | 40 | GC12M120322 | 5.708392143 |
| RBM15 | RNA Binding Motif Protein 15 | Protein Coding | 34 | GC01P110338 | 5.708090305 |
| MIR744 | MicroRNA 744 | RNA Gene | 16 | GC17P012081 | 5.70774889 |
| TNFRSF9 | TNF Receptor Superfamily Member 9 | Protein Coding | 38 | GC01M007915 | 5.705107689 |
| TAF15 | TATA-Box Binding Protein Associated Factor 15 | Protein Coding | 38 | GC17P052742 | 5.702683449 |
| HSPA9 | Heat Shock Protein Family A (Hsp70) Member 9 | Protein Coding | 42 | GC05M138554 | 5.699550629 |
| APOBEC3B | Apolipoprotein B MRNA Editing Enzyme Catalytic Subunit 3B | Protein Coding | 36 | GC22P038982 | 5.69618845 |
| MIR622 | MicroRNA 622 | RNA Gene | 13 | GC13P090231 | 5.694272041 |
| TUSC1 | Tumor Suppressor Candidate 1 | Protein Coding | 26 | GC09M025668 | 5.691157341 |
| APP | Amyloid Beta Precursor Protein | Protein Coding | 46 | GC21M025880 | 5.689314842 |
| GAPLINC | Gastric Adenocarcinoma Associated, Positive CD44 Regulator, Long Intergenic Non-Coding RNA | RNA Gene | 14 | GC18P003467 | 5.689172268 |
| MBD2 | Methyl-CpG Binding Domain Protein 2 | Protein Coding | 36 | GC18M054151 | 5.687479973 |
| PRPF8 | Pre-MRNA Processing Factor 8 | Protein Coding | 37 | GC17M001650 | 5.686435699 |
| UPK2 | Uroplakin 2 | Protein Coding | 33 | GC11P118925 | 5.6839571 |
| EEF1G | Eukaryotic Translation Elongation Factor 1 Gamma | Protein Coding | 35 | GC11M087120 | 5.679320335 |
| HOXA10 | Homeobox A10 | Protein Coding | 36 | GC07M027551 | 5.678555965 |
| GJB5 | Gap Junction Protein Beta 5 | Protein Coding | 36 | GC01P034755 | 5.673016071 |
| HSPH1 | Heat Shock Protein Family H (Hsp110) Member 1 | Protein Coding | 38 | GC13M031134 | 5.663099289 |
| SH2D1A | SH2 Domain Containing 1A | Protein Coding | 41 | GC0XP124227 | 5.662055016 |
| GPA33 | Glycoprotein A33 | Protein Coding | 35 | GC01M167052 | 5.660791874 |
| KIR3DL1 | Killer Cell Immunoglobulin Like Receptor, Three Ig Domains And Long Cytoplasmic Tail 1 | Protein Coding | 39 | GC19P064345 | 5.659858704 |
| PLCB1 | Phospholipase C Beta 1 | Protein Coding | 43 | GC20P008061 | 5.659686565 |
| HSD17B13 | Hydroxysteroid 17-Beta Dehydrogenase 13 | Protein Coding | 34 | GC04M087303 | 5.659575462 |
| PTPRT | Protein Tyrosine Phosphatase Receptor Type T | Protein Coding | 40 | GC20M042072 | 5.655676842 |
| MTX2 | Metaxin 2 | Protein Coding | 38 | GC02P176269 | 5.654241085 |
| FLNB | Filamin B | Protein Coding | 43 | GC03P058008 | 5.649643898 |
| PGA5 | Pepsinogen A5 | Protein Coding | 31 | GC11P061241 | 5.641642094 |
| ITGAL | Integrin Subunit Alpha L | Protein Coding | 42 | GC16P030472 | 5.638797283 |
| IL12B | Interleukin 12B | Protein Coding | 41 | GC05M159314 | 5.628295898 |
| CYP11B2 | Cytochrome P450 Family 11 Subfamily B Member 2 | Protein Coding | 43 | GC08M142910 | 5.62759304 |
| LAMA2 | Laminin Subunit Alpha 2 | Protein Coding | 40 | GC06P128863 | 5.626717567 |
| E2F6 | E2F Transcription Factor 6 | Protein Coding | 36 | GC02M011434 | 5.625354767 |
| SUMO1P3 | SUMO1 Pseudogene 3 | Pseudogene | 10 | GC01P160317 | 5.621440411 |
| PGRMC1 | Progesterone Receptor Membrane Component 1 | Protein Coding | 40 | GC0XP119236 | 5.619216919 |
| TNFRSF4 | TNF Receptor Superfamily Member 4 | Protein Coding | 39 | GC01M001211 | 5.619017124 |
| GRK2 | G Protein-Coupled Receptor Kinase 2 | Protein Coding | 37 | GC11P067266 | 5.618943214 |
| PHLDA1 | Pleckstrin Homology Like Domain Family A Member 1 | Protein Coding | 31 | GC12M076025 | 5.613395214 |

| | | | | | |
|---|---|---|---|---|---|
| IRS4 | Insulin Receptor Substrate 4 | Protein Coding | 36 | GC0XM108720 | 5.61233139 |
| NISCH | Nischarin | Protein Coding | 39 | GC03P052455 | 5.610683441 |
| RBBP7 | RB Binding Protein 7, Chromatin Remodeling Factor | Protein Coding | 37 | GC0XM016839 | 5.610033989 |
| FAT2 | FAT Atypical Cadherin 2 | Protein Coding | 34 | GC05M151504 | 5.609570503 |
| HSD11B1 | Hydroxysteroid 11-Beta Dehydrogenase 1 | Protein Coding | 46 | GC01P209686 | 5.608263016 |
| PDCD6 | Programmed Cell Death 6 | Protein Coding | 35 | GC05P000272 | 5.606873989 |
| ZNF276 | Zinc Finger Protein 276 | Protein Coding | 31 | GC16P089720 | 5.606655121 |
| DCST1-AS1 | DCST1 Antisense RNA 1 | RNA Gene | 13 | GC01M155167 | 5.605815887 |
| CFAP300 | Cilia And Flagella Associated Protein 300 | Protein Coding | 26 | GC11P102048 | 5.604939938 |
| MBP | Myelin Basic Protein | Protein Coding | 40 | GC18M076978 | 5.60296154 |
| PGA4 | Pepsinogen A4 | Protein Coding | 28 | GC11P061222 | 5.602603912 |
| SAMD9L | Sterile Alpha Motif Domain Containing 9 Like | Protein Coding | 33 | GC07M093130 | 5.590653419 |
| MIR302A | MicroRNA 302a | RNA Gene | 19 | GC04M112762 | 5.590014935 |
| FNDC11 | Fibronectin Type III Domain Containing 11 | Protein Coding | 24 | GC20P063562 | 5.58953619 |
| GDF2 | Growth Differentiation Factor 2 | Protein Coding | 39 | GC10P047322 | 5.589007378 |
| GNAS-AS1 | GNAS Antisense RNA 1 | RNA Gene | 22 | GC20M058811 | 5.58733654 |
| SLC2A4 | Solute Carrier Family 2 Member 4 | Protein Coding | 41 | GC17P010858 | 5.584294319 |
| VANGL1 | VANGL Planar Cell Polarity Protein 1 | Protein Coding | 39 | GC01P115641 | 5.584119797 |
| TMEM170B | Transmembrane Protein 170B | Protein Coding | 26 | GC06P011537 | 5.582253933 |
| PLEK | Pleckstrin | Protein Coding | 36 | GC02P068365 | 5.581676483 |
| PLXDC1 | Plexin Domain Containing 1 | Protein Coding | 34 | GC17M039063 | 5.581373215 |
| CSAG2 | CSAG Family Member 2 | Protein Coding | 17 | GC0XM152708 | 5.580730915 |
| C20orf85 | Chromosome 20 Open Reading Frame 85 | Protein Coding | 27 | GC20P058150 | 5.575335026 |
| LMO4 | LIM Domain Only 4 | Protein Coding | 35 | GC01P087329 | 5.57502985 |
| KMT5B | Lysine Methyltransferase 5B | Protein Coding | 33 | GC11M087425 | 5.572943211 |
| ATP2A2 | ATPase Sarcoplasmic/Endoplasmic Reticulum Ca2+ Transporting 2 | Protein Coding | 46 | GC12P110280 | 5.570585251 |
| PSMD10 | Proteasome 26S Subunit, Non-ATPase 10 | Protein Coding | 34 | GC0XM108084 | 5.570376396 |
| REG3A | Regenerating Family Member 3 Alpha | Protein Coding | 34 | GC02M079157 | 5.569748402 |
| ALMS1 | ALMS1 Centrosome And Basal Body Associated Protein | Protein Coding | 38 | GC02P073385 | 5.569573402 |
| UPK3A | Uroplakin 3A | Protein Coding | 33 | GC22P045284 | 5.56724453 |
| INHBB | Inhibin Subunit Beta B | Protein Coding | 38 | GC02P121749 | 5.563691139 |
| DOT1L | DOT1 Like Histone Lysine Methyltransferase | Protein Coding | 40 | GC19P002654 | 5.56289959 |
| ACIN1 | Apoptotic Chromatin Condensation Inducer 1 | Protein Coding | 35 | GC14M023058 | 5.56126833 |
| HOXC13 | Homeobox C13 | Protein Coding | 37 | GC12P053938 | 5.561097622 |
| GAS6 | Growth Arrest Specific 6 | Protein Coding | 40 | GC13M113820 | 5.560196877 |
| KRT3 | Keratin 3 | Protein Coding | 38 | GC12M052789 | 5.559882641 |
| CPS1-IT1 | CPS1 Intronic Transcript 1 | RNA Gene | 15 | GC02P210617 | 5.559432983 |
| SMARCC2 | SWI/SNF Related, Matrix Associated, Actin Dependent Regulator Of Chromatin Subfamily C Member 2 | Protein Coding | 40 | GC12M056582 | 5.558614731 |
| NKX2-5 | NK2 Homeobox 5 | Protein Coding | 40 | GC05M173232 | 5.558196068 |
| DNAJB1 | DnaJ Heat Shock Protein Family (Hsp40) Member B1 | Protein Coding | 38 | GC19M014514 | 5.556268215 |
| MIR374A | MicroRNA 374a | RNA Gene | 17 | GC0XM074296 | 5.554965973 |
| DHCR7 | 7-Dehydrocholesterol Reductase | Protein Coding | 42 | GC11M071428 | 5.554672241 |
| SFTPA2 | Surfactant Protein A2 | Protein Coding | 36 | GC10M081648 | 5.550236702 |
| SMAD9 | SMAD Family Member 9 | Protein Coding | 41 | GC13M036844 | 5.549544334 |
| CENPW | Centromere Protein W | Protein Coding | 31 | GC06P126339 | 5.548700333 |
| EGFLAM-AS1 | EGFLAM Antisense RNA 1 | RNA Gene | 14 | GC05M038426 | 5.54692173 |
| OBSCN | Obscurin, Cytoskeletal Calmodulin And Titin-Interacting RhoGEF | Protein Coding | 35 | GC01P228208 | 5.54358387 |
| MIR637 | MicroRNA 637 | RNA Gene | 15 | GC19M003961 | 5.540541649 |
| WIPF1 | WAS/WASL Interacting Protein Family Member 1 | Protein Coding | 39 | GC02M174559 | 5.536983013 |
| GP5 | Glycoprotein V Platelet | Protein Coding | 36 | GC03M194395 | 5.536355495 |
| DHX9 | DExH-Box Helicase 9 | Protein Coding | 36 | GC01P182839 | 5.535982132 |
| TXNIP | Thioredoxin Interacting Protein | Protein Coding | 34 | GC01M145992 | 5.534115791 |
| CD1D | CD1d Molecule | Protein Coding | 39 | GC01P158178 | 5.533557892 |
| ATRIP | ATR Interacting Protein | Protein Coding | 37 | GC03P048877 | 5.529435635 |
| TNFSF13B | TNF Superfamily Member 13b | Protein Coding | 40 | GC13P108251 | 5.528532982 |
| KDM6B | Lysine Demethylase 6B | Protein Coding | 39 | GC17P007834 | 5.528322697 |
| RNF216 | Ring Finger Protein 216 | Protein Coding | 38 | GC07M005620 | 5.528126717 |
| TCOF1 | Treacle Ribosome Biogenesis Factor 1 | Protein Coding | 38 | GC05P150358 | 5.527909756 |
| GPI | Glucose-6-Phosphate Isomerase | Protein Coding | 43 | GC19P063413 | 5.524013042 |
| SEC11A | SEC11 Homolog A, Signal Peptidase Complex Subunit | Protein Coding | 34 | GC15M084669 | 5.522274971 |
| MDC1 | Mediator Of DNA Damage Checkpoint 1 | Protein Coding | 36 | GC06M063527 | 5.521120548 |
| MIR103A2 | MicroRNA 103a-2 | RNA Gene | 17 | GC20P003917 | 5.520917892 |
| VTRNA2-1 | Vault RNA 2-1 | RNA Gene | 14 | GC05M136081 | 5.52076292 |
| ASAH1 | N-Acylsphingosine Amidohydrolase 1 | Protein Coding | 42 | GC08M018055 | 5.520346642 |
| MIR409 | MicroRNA 409 | RNA Gene | 18 | GC14P109533 | 5.519752502 |
| ST3GAL4 | ST3 Beta-Galactoside Alpha-2,3-Sialyltransferase 4 | Protein Coding | 37 | GC11P126355 | 5.516637802 |
| CCKAR | Cholecystokinin A Receptor | Protein Coding | 41 | GC04M026483 | 5.516244411 |
| SLC40A1 | Solute Carrier Family 40 Member 1 | Protein Coding | 41 | GC02M189560 | 5.512548447 |
| CYP2B6 | Cytochrome P450 Family 2 Subfamily B Member 6 | Protein Coding | 42 | GC19P040991 | 5.511484146 |
| PREX2 | Phosphatidylinositol-3,4,5-Trisphosphate Dependent Rac Exchange Factor 2 | Protein Coding | 34 | GC08P067952 | 5.510730743 |
| HLA-DPB1 | Major Histocompatibility Complex, Class II, DP Beta 1 | Protein Coding | 40 | GC06P080395 | 5.509568214 |
| HSD17B2 | Hydroxysteroid 17-Beta Dehydrogenase 2 | Protein Coding | 40 | GC16P082068 | 5.504716873 |
| CCL18 | C-C Motif Chemokine Ligand 18 | Protein Coding | 31 | GC17P036064 | 5.502711296 |
| GAS5-AS1 | GAS5 Antisense RNA 1 | RNA Gene | 14 | GC01P173863 | 5.498695374 |
| LIMA1 | LIM Domain And Actin Binding 1 | Protein Coding | 35 | GC12M050175 | 5.498511314 |
| PERP | P53 Apoptosis Effector Related To PMP22 | Protein Coding | 36 | GC06M138088 | 5.493888378 |
| PRNP | Prion Protein | Protein Coding | 42 | GC20P004686 | 5.492622852 |
| RBM39 | RNA Binding Motif Protein 39 | Protein Coding | 34 | GC20M035703 | 5.491549015 |
| ATXN2 | Ataxin 2 | Protein Coding | 38 | GC12M111443 | 5.488814354 |
| SEZ6L | Seizure Related 6 Homolog Like | Protein Coding | 35 | GC22P026169 | 5.486113071 |
| LINC00941 | Long Intergenic Non-Protein Coding RNA 941 | RNA Gene | 14 | GC12P030757 | 5.485995293 |
| HYOU1 | Hypoxia Up-Regulated 1 | Protein Coding | 40 | GC11M119170 | 5.485959053 |
| LAPTM4B | Lysosomal Protein Transmembrane 4 Beta | Protein Coding | 35 | GC08P097775 | 5.484660625 |
| POU4F2 | POU Class 4 Homeobox 2 | Protein Coding | 33 | GC04P146638 | 5.483643532 |
| ASIP | Agouti Signaling Protein | Protein Coding | 35 | GC20P034442 | 5.479462624 |
| ADIPOR1 | Adiponectin Receptor 1 | Protein Coding | 40 | GC01M202940 | 5.47865057 |
| MIR454 | MicroRNA 454 | RNA Gene | 18 | GC17M059137 | 5.478158951 |
| NUDC | Nuclear Distribution C, Dynein Complex Regulator | Protein Coding | 38 | GC01P027597 | 5.475399971 |
| TNFSF13 | TNF Superfamily Member 13 | Protein Coding | 40 | GC17P007558 | 5.474305153 |
| UBR5 | Ubiquitin Protein Ligase E3 Component N-Recognin 5 | Protein Coding | 37 | GC08M102252 | 5.472173691 |
| TBP | TATA-Box Binding Protein | Protein Coding | 43 | GC06P170554 | 5.471645832 |
| PAX8-AS1 | PAX8 Antisense RNA 1 | RNA Gene | 16 | GC02P113211 | 5.471291542 |
| STARD8 | StAR Related Lipid Transfer Domain Containing 8 | Protein Coding | 32 | GC0XP068647 | 5.470898628 |
| F10 | Coagulation Factor X | Protein Coding | 45 | GC13P113122 | 5.468662262 |

| | | | | | |
|---|---|---|---|---|---|
| HSD11B2 | Hydroxysteroid 11-Beta Dehydrogenase 2 | Protein Coding | 42 | GC16P067433 | 5.468507767 |
| SHOC2 | SHOC2 Leucine Rich Repeat Scaffold Protein | Protein Coding | 37 | GC10P110919 | 5.463836193 |
| CEP55 | Centrosomal Protein 55 | Protein Coding | 36 | GC10P093496 | 5.462501526 |
| TRA | T Cell Receptor Alpha Locus | Protein Coding | 18 | GC14P021621 | 5.46079731 |
| ARSA | Arylsulfatase A | Protein Coding | 43 | GC22M050622 | 5.459334373 |
| CLSPN | Claspin | Protein Coding | 35 | GC01M035720 | 5.458480835 |
| HADHB | Hydroxyacyl-CoA Dehydrogenase Trifunctional Multienzyme Complex Subunit Beta | Protein Coding | 42 | GC02P026243 | 5.458025455 |
| MIR4732 | MicroRNA 4732 | RNA Gene | 14 | GC17M034588 | 5.457748413 |
| TET1 | Tet Methylcytosine Dioxygenase 1 | Protein Coding | 34 | GC10P068560 | 5.455079556 |
| RNASET2 | Ribonuclease T2 | Protein Coding | 40 | GC06M166924 | 5.453835487 |
| WWP1 | WW Domain Containing E3 Ubiquitin Protein Ligase 1 | Protein Coding | 36 | GC08P086342 | 5.451229572 |
| TATDN1 | TatD DNase Domain Containing 1 | Protein Coding | 32 | GC08M124488 | 5.44662571 |
| RAG1 | Recombination Activating 1 | Protein Coding | 41 | GC11P036536 | 5.443050861 |
| LINC00857 | Long Intergenic Non-Protein Coding RNA 857 | RNA Gene | 15 | GC10P091442 | 5.441390038 |
| IRF8 | Interferon Regulatory Factor 8 | Protein Coding | 40 | GC16P085898 | 5.439935684 |
| FZD2 | Frizzled Class Receptor 2 | Protein Coding | 43 | GC17P044557 | 5.43874073 |
| HRH2 | Histamine Receptor H2 | Protein Coding | 42 | GC05P175659 | 5.438251972 |
| CRYAB | Crystallin Alpha B | Protein Coding | 41 | GC11M111908 | 5.438235283 |
| PPP1R13L | Protein Phosphatase 1 Regulatory Subunit 13 Like | Protein Coding | 36 | GC19M045379 | 5.438005447 |
| KPNA2 | Karyopherin Subunit Alpha 2 | Protein Coding | 39 | GC17P068035 | 5.436148643 |
| MAOA | Monoamine Oxidase A | Protein Coding | 44 | GC0XP043654 | 5.435246468 |
| UGT2B7 | UDP Glucuronosyltransferase Family 2 Member B7 | Protein Coding | 40 | GC04P069051 | 5.431424618 |
| LHX4 | LIM Homeobox 4 | Protein Coding | 40 | GC01P180230 | 5.429026127 |
| P4HB | Prolyl 4-Hydroxylase Subunit Beta | Protein Coding | 43 | GC17M081843 | 5.427019119 |
| HOTAIRM1 | HOXA Transcript Antisense RNA, Myeloid-Specific 1 | RNA Gene | 19 | GC07P027095 | 5.426673412 |
| TLCD3A | TLC Domain Containing 3A | Protein Coding | 25 | GC17P001457 | 5.425094128 |
| CDKN2D | Cyclin Dependent Kinase Inhibitor 2D | Protein Coding | 36 | GC19M010566 | 5.425029755 |
| MCM5 | Minichromosome Maintenance Complex Component 5 | Protein Coding | 40 | GC22P035400 | 5.422254086 |
| MIR99B | MicroRNA 99b | RNA Gene | 20 | GC19P051692 | 5.421275139 |
| TRIM25 | Tripartite Motif Containing 25 | Protein Coding | 38 | GC17M056836 | 5.420145988 |
| MUC3A | Mucin 3A, Cell Surface Associated | Protein Coding | 31 | GC07P100949 | 5.419648647 |
| FYN | FYN Proto-Oncogene, Src Family Tyrosine Kinase | Protein Coding | 42 | GC06M111660 | 5.4195261 |
| GSDMA | Gasdermin A | Protein Coding | 32 | GC17P053048 | 5.417953014 |
| CNR1 | Cannabinoid Receptor 1 | Protein Coding | 42 | GC06M088139 | 5.415354252 |
| NR5A2 | Nuclear Receptor Subfamily 5 Group A Member 2 | Protein Coding | 41 | GC01P199996 | 5.411871433 |
| PRSS2 | Serine Protease 2 | Protein Coding | 36 | GC07P147987 | 5.410549641 |
| F7 | Coagulation Factor VII | Protein Coding | 43 | GC13P113105 | 5.410394669 |
| PSEN1 | Presenilin 1 | Protein Coding | 47 | GC14P073136 | 5.408677578 |
| TGM4 | Transglutaminase 4 | Protein Coding | 36 | GC03P044874 | 5.408009529 |
| CLDN8 | Claudin 8 | Protein Coding | 34 | GC21M030214 | 5.406997681 |
| BCL11B | BAF Chromatin Remodeling Complex Subunit BCL11B | Protein Coding | 38 | GC14M099169 | 5.406009197 |
| EMCN | Endomucin | Protein Coding | 33 | GC04M100395 | 5.400956154 |
| TPMT | Thiopurine S-Methyltransferase | Protein Coding | 42 | GC06M018128 | 5.400096416 |
| FAIM2 | Fas Apoptotic Inhibitory Molecule 2 | Protein Coding | 31 | GC12M049866 | 5.394169331 |
| FOXG1 | Forkhead Box G1 | Protein Coding | 39 | GC14P028766 | 5.393437862 |
| TRPC6 | Transient Receptor Potential Cation Channel Subfamily C Member 6 | Protein Coding | 44 | GC11M101451 | 5.390823364 |
| RAD17 | RAD17 Checkpoint Clamp Loader Component | Protein Coding | 38 | GC05P069369 | 5.386546135 |
| ASTN2 | Astrotactin 2 | Protein Coding | 33 | GC09M116425 | 5.383330822 |
| TSHZ2 | Teashirt Zinc Finger Homeobox 2 | Protein Coding | 34 | GC20P052972 | 5.379026413 |
| BCL2L2 | BCL2 Like 2 | Protein Coding | 40 | GC14P031856 | 5.378561974 |
| THOC5 | THO Complex 5 | Protein Coding | 32 | GC22M029505 | 5.377447128 |
| NEIL1 | Nei Like DNA Glycosylase 1 | Protein Coding | 35 | GC15P075346 | 5.376281738 |
| TFAP2B | Transcription Factor AP-2 Beta | Protein Coding | 40 | GC06P080662 | 5.373903275 |
| ARHGAP26 | Rho GTPase Activating Protein 26 | Protein Coding | 39 | GC05P142770 | 5.373822212 |
| IRF6 | Interferon Regulatory Factor 6 | Protein Coding | 39 | GC01M209785 | 5.372117996 |
| LACTB | Lactamase Beta | Protein Coding | 32 | GC15P115764 | 5.37139225 |
| TNFRSF13B | TNF Receptor Superfamily Member 13B | Protein Coding | 42 | GC17M016929 | 5.371325016 |
| LINC01488 | Long Intergenic Non-Protein Coding RNA 1488 | RNA Gene | 13 | GC11P069839 | 5.371242523 |
| KAT6B | Lysine Acetyltransferase 6B | Protein Coding | 40 | GC10P074864 | 5.368112087 |
| PHLDB1 | Pleckstrin Homology Like Domain Family B Member 1 | Protein Coding | 32 | GC11P118606 | 5.367613792 |
| IL18R1 | Interleukin 18 Receptor 1 | Protein Coding | 38 | GC02P102311 | 5.3671422 |
| DNM2 | Dynamin 2 | Protein Coding | 44 | GC19P010718 | 5.363223076 |
| HEPN1 | Hepatocellular Carcinoma, Down-Regulated 1 | RNA Gene | 23 | GC11P124919 | 5.359701633 |
| UFL1 | UFM1 Specific Ligase 1 | Protein Coding | 31 | GC06P096521 | 5.357933521 |
| NEMF | Nuclear Export Mediator Factor | Protein Coding | 33 | GC14M049782 | 5.357509613 |
| MAB21L1 | Mab-21 Like 1 | Protein Coding | 35 | GC13M035473 | 5.355730057 |
| SMYD3 | SET And MYND Domain Containing 3 | Protein Coding | 36 | GC01M245749 | 5.353146553 |
| ETV5 | ETS Variant Transcription Factor 5 | Protein Coding | 37 | GC03M186046 | 5.352415085 |
| KIAA1549 | KIAA1549 | Protein Coding | 34 | GC07M138831 | 5.348274231 |
| DHRS9 | Dehydrogenase/Reductase 9 | Protein Coding | 35 | GC02P169064 | 5.342116356 |
| PRDM1 | PR/SET Domain 1 | Protein Coding | 40 | GC06P105993 | 5.33683157 |
| PIWIL4 | Piwi Like RNA-Mediated Gene Silencing 4 | Protein Coding | 34 | GC11P094543 | 5.336787701 |
| LATS1 | Large Tumor Suppressor Kinase 1 | Protein Coding | 40 | GC06M149658 | 5.335949898 |
| PLOD1 | Procollagen-Lysine,2-Oxoglutarate 5-Dioxygenase 1 | Protein Coding | 39 | GC01P011934 | 5.335519314 |
| IRF2BP2 | Interferon Regulatory Factor 2 Binding Protein 2 | Protein Coding | 32 | GC01M234604 | 5.334563255 |
| GLUL | Glutamate-Ammonia Ligase | Protein Coding | 43 | GC01M182378 | 5.332746983 |
| GNG4 | G Protein Subunit Gamma 4 | Protein Coding | 35 | GC01M235547 | 5.331912041 |
| GGPS1 | Geranylgeranyl Diphosphate Synthase 1 | Protein Coding | 40 | GC01P235327 | 5.327868938 |
| MIR7-3 | MicroRNA 7-3 | RNA Gene | 17 | GC19P004770 | 5.327226162 |
| PTPN6 | Protein Tyrosine Phosphatase Non-Receptor Type 6 | Protein Coding | 44 | GC12P019853 | 5.326177597 |
| NDRG2 | NDRG Family Member 2 | Protein Coding | 34 | GC14M021016 | 5.324286938 |
| PCBP2-OT1 | PCBP2 Overlapping Transcript 1 | RNA Gene | 11 | GC12P053464 | 5.318205833 |
| SH3PXD2A | SH3 And PX Domains 2A | Protein Coding | 35 | GC10M103594 | 5.315610409 |
| SIN3A | SIN3 Transcription Regulator Family Member A | Protein Coding | 41 | GC15M075369 | 5.315547943 |
| KRT16 | Keratin 16 | Protein Coding | 39 | GC17M041609 | 5.315045357 |
| PRR5 | Proline Rich 5 | Protein Coding | 33 | GC22P044668 | 5.313551903 |
| LHB | Luteinizing Hormone Subunit Beta | Protein Coding | 37 | GC19M049015 | 5.313313484 |
| DNM3OS | DNM3 Opposite Strand/Antisense RNA | RNA Gene | 18 | GC01M172217 | 5.31237936 |
| PFKP | Phosphofructokinase, Platelet | Protein Coding | 40 | GC10P003066 | 5.310538769 |
| MYEF2 | Myelin Expression Factor 2 | Protein Coding | 34 | GC15M048134 | 5.309758186 |
| PIBF1 | Progesterone Immunomodulatory Binding Factor 1 | Protein Coding | 37 | GC13P072782 | 5.3084445 |
| WFDC1 | WAP Four-Disulfide Core Domain 1 | Protein Coding | 33 | GC16P084328 | 5.301987648 |
| FADS2 | Fatty Acid Desaturase 2 | Protein Coding | 40 | GC11P061792 | 5.301615238 |
| IFNGR1 | Interferon Gamma Receptor 1 | Protein Coding | 44 | GC06M137197 | 5.300624847 |

| | | | | | |
|---|---|---|---|---|---|
| RMI1 | RecQ Mediated Genome Instability 1 | Protein Coding | 34 | GC09P083980 | 5.29938221 |
| TFR2 | Transferrin Receptor 2 | Protein Coding | 40 | GC07M100620 | 5.29691124 |
| LRRK2 | Leucine Rich Repeat Kinase 2 | Protein Coding | 44 | GC12P040196 | 5.296653748 |
| AGRN | Agrin | Protein Coding | 41 | GC01P001020 | 5.296281815 |
| UGT1A10 | UDP Glucuronosyltransferase Family 1 Member A10 | Protein Coding | 38 | GC02P233636 | 5.293197632 |
| GDF9 | Growth Differentiation Factor 9 | Protein Coding | 38 | GC05M132861 | 5.292296886 |
| PITX2 | Paired Like Homeodomain 2 | Protein Coding | 42 | GC04M110617 | 5.291934967 |
| ZMYND10 | Zinc Finger MYND-Type Containing 10 | Protein Coding | 34 | GC03M051110 | 5.291619301 |
| RGMB-AS1 | RGMB Antisense RNA 1 | RNA Gene | 14 | GC05M098769 | 5.291483879 |
| TLR10 | Toll Like Receptor 10 | Protein Coding | 35 | GC04M038773 | 5.286815643 |
| SART1 | Spliceosome Associated Factor 1, Recruiter Of U4/U6.U5 Tri-SnRNP | Protein Coding | 36 | GC11P069582 | 5.286665916 |
| DLEU2 | Deleted In Lymphocytic Leukemia 2 | RNA Gene | 22 | GC13M049913 | 5.278984547 |
| TUSC8 | Tumor Suppressor Candidate 8 | RNA Gene | 11 | GC13M044400 | 5.278393745 |
| TMEM14A | Transmembrane Protein 14A | Protein Coding | 31 | GC06P052671 | 5.277900219 |
| ANAPC5 | Anaphase Promoting Complex Subunit 5 | Protein Coding | 34 | GC12M121308 | 5.275200844 |
| SUMO1 | Small Ubiquitin Like Modifier 1 | Protein Coding | 40 | GC02M202206 | 5.275000095 |
| CABLES1 | Cdk5 And Abl Enzyme Substrate 1 | Protein Coding | 35 | GC18P023134 | 5.274373055 |
| DNASE1 | Deoxyribonuclease 1 | Protein Coding | 39 | GC16P003611 | 5.272857666 |
| AMFR | Autocrine Motility Factor Receptor | Protein Coding | 38 | GC16M056361 | 5.268645287 |
| PNP | Purine Nucleoside Phosphorylase | Protein Coding | 42 | GC14P020468 | 5.268437386 |
| TUBB6 | Tubulin Beta 6 Class V | Protein Coding | 37 | GC18P012307 | 5.264788151 |
| EPGN | Epithelial Mitogen | Protein Coding | 34 | GC04P074309 | 5.264734268 |
| KRT6A | Keratin 6A | Protein Coding | 39 | GC12M052488 | 5.262854576 |
| CFH | Complement Factor H | Protein Coding | 42 | GC01P196621 | 5.262760639 |
| MCTS1 | MCTS1 Re-Initiation And Release Factor | Protein Coding | 32 | GC0XP120594 | 5.262004852 |
| GPR35 | G Protein-Coupled Receptor 35 | Protein Coding | 40 | GC02P240605 | 5.261771202 |
| RPS7 | Ribosomal Protein S7 | Protein Coding | 36 | GC02P003575 | 5.26139164 |
| MIR487B | MicroRNA 487b | RNA Gene | 17 | GC14P109539 | 5.260332108 |
| PPIEL | Peptidylprolyl Isomerase E Like Pseudogene | Pseudogene | 13 | GC01M039522 | 5.258053303 |
| BRS3 | Bombesin Receptor Subtype 3 | Protein Coding | 37 | GC0XP136493 | 5.258004189 |
| SLC16A1-AS1 | SLC16A1 Antisense RNA 1 | RNA Gene | 15 | GC01P112956 | 5.256656647 |
| ARID2 | AT-Rich Interaction Domain 2 | Protein Coding | 37 | GC12P045729 | 5.256446362 |
| GPX4 | Glutathione Peroxidase 4 | Protein Coding | 42 | GC19P001103 | 5.256167412 |
| ACVRL1 | Activin A Receptor Like Type 1 | Protein Coding | 44 | GC12P051906 | 5.254202366 |
| CIITA | Class II Major Histocompatibility Complex Transactivator | Protein Coding | 40 | GC16P011285 | 5.253146648 |
| CAD | Carbamoyl-Phosphate Synthetase 2, Aspartate Transcarbamylase, And Dihydroorotase | Protein Coding | 44 | GC02P027217 | 5.251112938 |
| ZNF148 | Zinc Finger Protein 148 | Protein Coding | 37 | GC03M125225 | 5.250279903 |
| PTPRD | Protein Tyrosine Phosphatase Receptor Type D | Protein Coding | 42 | GC09M008307 | 5.249402046 |
| VWA2 | Von Willebrand Factor A Domain Containing 2 | Protein Coding | 32 | GC10P114239 | 5.248811245 |
| GTF2E2 | General Transcription Factor IIE Subunit 2 | Protein Coding | 38 | GC08M030578 | 5.248594284 |
| FNDC3B | Fibronectin Type III Domain Containing 3B | Protein Coding | 32 | GC03P172039 | 5.246126652 |
| GAD1 | Glutamate Decarboxylase 1 | Protein Coding | 45 | GC02P170813 | 5.245885849 |
| PTGES2 | Prostaglandin E Synthase 2 | Protein Coding | 38 | GC09M128120 | 5.244220257 |
| MIR371A | MicroRNA 371a | RNA Gene | 17 | GC19P053787 | 5.241431713 |
| LINC02633 | Long Intergenic Non-Protein Coding RNA 2633 | RNA Gene | 9 | GC10P043313 | 5.238765717 |
| RPA2 | Replication Protein A2 | Protein Coding | 39 | GC01M027909 | 5.236919403 |
| SLCO1B3 | Solute Carrier Organic Anion Transporter Family Member 1B3 | Protein Coding | 40 | GC12P020810 | 5.236352444 |
| CD151 | CD151 Molecule (Raph Blood Group) | Protein Coding | 40 | GC11P001684 | 5.235839367 |
| MYO5A | Myosin VA | Protein Coding | 40 | GC15M082019 | 5.234594345 |
| GAA | Alpha Glucosidase | Protein Coding | 44 | GC17P080101 | 5.233754635 |
| CNOT9 | CCR4-NOT Transcription Complex Subunit 9 | Protein Coding | 30 | GC02P218569 | 5.231062889 |
| LAMA3 | Laminin Subunit Alpha 3 | Protein Coding | 41 | GC18P023689 | 5.230981827 |
| DUXAP8 | Double Homeobox A Pseudogene 8 | Pseudogene | 7 | GC22P015784 | 5.230701923 |
| P4HA2 | Prolyl 4-Hydroxylase Subunit Alpha 2 | Protein Coding | 42 | GC05M132191 | 5.227843761 |
| CILK1 | Ciliogenesis Associated Kinase 1 | Protein Coding | 35 | GC06M064009 | 5.22453928 |
| IRAK1 | Interleukin 1 Receptor Associated Kinase 1 | Protein Coding | 44 | GC0XM154010 | 5.223579884 |
| TOPBP1 | DNA Topoisomerase II Binding Protein 1 | Protein Coding | 36 | GC03M133600 | 5.22317791 |
| LRIG1 | Leucine Rich Repeats And Immunoglobulin Like Domains 1 | Protein Coding | 37 | GC03M066379 | 5.220682144 |
| MUC7 | Mucin 7, Secreted | Protein Coding | 33 | GC04P070430 | 5.21819067 |
| TULP1 | TUB Like Protein 1 | Protein Coding | 38 | GC06M063750 | 5.217067242 |
| ZNF300P1 | Zinc Finger Protein 300 Pseudogene 1 | Pseudogene | 14 | GC05M150930 | 5.212088585 |
| ADAMTS9-AS2 | ADAMTS9 Antisense RNA 2 | RNA Gene | 17 | GC03P064671 | 5.211145401 |
| PSMB7 | Proteasome 20S Subunit Beta 7 | Protein Coding | 40 | GC09M124353 | 5.209429741 |
| SAMD9 | Sterile Alpha Motif Domain Containing 9 | Protein Coding | 32 | GC07M093099 | 5.208535671 |
| MIR657 | MicroRNA 657 | RNA Gene | 15 | GC17M081125 | 5.20630455 |
| SAGE1 | Sarcoma Antigen 1 | Protein Coding | 26 | GC0XP135889 | 5.204949379 |
| RPS9 | Ribosomal Protein S9 | Protein Coding | 37 | GC19P066185 | 5.204831123 |
| PIK3C2A | Phosphatidylinositol-4-Phosphate 3-Kinase Catalytic Subunit Type 2 Alpha | Protein Coding | 42 | GC11M017542 | 5.204809189 |
| INSM1 | INSM Transcriptional Repressor 1 | Protein Coding | 34 | GC20P020368 | 5.204476357 |
| RAB23 | RAB23, Member RAS Oncogene Family | Protein Coding | 38 | GC06M064066 | 5.202368736 |
| CDK9 | Cyclin Dependent Kinase 9 | Protein Coding | 40 | GC09P127987 | 5.201885223 |
| POC1A | POC1 Centriolar Protein A | Protein Coding | 34 | GC03M052109 | 5.200474262 |
| ICAM5 | Intercellular Adhesion Molecule 5 | Protein Coding | 36 | GC19P010289 | 5.198250294 |
| ISL1 | ISL LIM Homeobox 1 | Protein Coding | 40 | GC05P051383 | 5.196723938 |
| HCFC1R1 | Host Cell Factor C1 Regulator 1 | Protein Coding | 29 | GC16M003022 | 5.196078777 |
| MAGI2 | Membrane Associated Guanylate Kinase, WW And PDZ Domain Containing 2 | Protein Coding | 39 | GC07M078017 | 5.193567276 |
| C4A | Complement C4A (Rodgers Blood Group) | Protein Coding | 40 | GC06P080381 | 5.192584991 |
| COL17A1 | Collagen Type XVII Alpha 1 Chain | Protein Coding | 40 | GC10M104031 | 5.19164896 |
| ZNHIT6 | Zinc Finger HIT-Type Containing 6 | Protein Coding | 30 | GC01M085649 | 5.183494568 |
| SLC25A13 | Solute Carrier Family 25 Member 13 | Protein Coding | 42 | GC07M096120 | 5.181975842 |
| PLA2G6 | Phospholipase A2 Group VI | Protein Coding | 43 | GC22M057064 | 5.181731224 |
| ATF4 | Activating Transcription Factor 4 | Protein Coding | 40 | GC22P039606 | 5.181692123 |
| PRDX5 | Peroxiredoxin 5 | Protein Coding | 40 | GC11P064321 | 5.181536674 |
| ALDH3A1 | Aldehyde Dehydrogenase 3 Family Member A1 | Protein Coding | 39 | GC17M019737 | 5.17536974 |
| TBX2 | T-Box Transcription Factor 2 | Protein Coding | 41 | GC17P061399 | 5.175336361 |
| ANXA6 | Annexin A6 | Protein Coding | 37 | GC05M151100 | 5.173226833 |
| SSTR3 | Somatostatin Receptor 3 | Protein Coding | 41 | GC22M037204 | 5.16852951 |
| NR1H4 | Nuclear Receptor Subfamily 1 Group H Member 4 | Protein Coding | 43 | GC12P100473 | 5.168469429 |
| NKX2-8 | NK2 Homeobox 8 | Protein Coding | 31 | GC14M036580 | 5.168072701 |
| CASK | Calcium/Calmodulin Dependent Serine Protein Kinase | Protein Coding | 43 | GC0XM041514 | 5.164831161 |
| KCNN4 | Potassium Calcium-Activated Channel Subfamily N Member 4 | Protein Coding | 44 | GC19M063902 | 5.164050102 |
| SCRIB | Scribble Planar Cell Polarity Protein | Protein Coding | 36 | GC08M144112 | 5.161774635 |
| POP1 | POP1 Homolog, Ribonuclease P/MRP Subunit | Protein Coding | 35 | GC08P098117 | 5.160481453 |
| SRSF1 | Serine And Arginine Rich Splicing Factor 1 | Protein Coding | 36 | GC17M058000 | 5.156620026 |

| | | | | | |
|---|---|---|---|---|---|
| CR1 | Complement C3b/C4b Receptor 1 (Knops Blood Group) | Protein Coding | 42 | GC01P207496 | 5.155119419 |
| HADHA | Hydroxyacyl-CoA Dehydrogenase Trifunctional Multienzyme Complex Subunit Alpha | Protein Coding | 42 | GC02M026190 | 5.153599262 |
| MT2A | Metallothionein 2A | Protein Coding | 38 | GC16P056993 | 5.153359413 |
| UBE2I | Ubiquitin Conjugating Enzyme E2 I | Protein Coding | 41 | GC16P010657 | 5.149501801 |
| WNK1 | WNK Lysine Deficient Protein Kinase 1 | Protein Coding | 43 | GC12P000733 | 5.148064613 |
| RNF8 | Ring Finger Protein 8 | Protein Coding | 37 | GC06P080545 | 5.146764278 |
| ACTA1 | Actin Alpha 1, Skeletal Muscle | Protein Coding | 43 | GC01M229466 | 5.14554882 |
| POLE2 | DNA Polymerase Epsilon 2, Accessory Subunit | Protein Coding | 38 | GC14M049643 | 5.141996861 |
| TACR1 | Tachykinin Receptor 1 | Protein Coding | 40 | GC02M075051 | 5.141569138 |
| PPP1CA | Protein Phosphatase 1 Catalytic Subunit Alpha | Protein Coding | 42 | GC11M087381 | 5.140898705 |
| OFD1 | OFD1 Centriole And Centriolar Satellite Protein | Protein Coding | 38 | GC0XP013734 | 5.140039444 |
| MDC1-AS1 | MDC1 Antisense RNA 1 | RNA Gene | 13 | GC06P080330 | 5.139794827 |
| TEX14 | Testis Expressed 14, Intercellular Bridge Forming Factor | Protein Coding | 35 | GC17M058556 | 5.138296604 |
| PRAL | P53 Regulation Associated LncRNA | RNA Gene | 9 | GC17M006773 | 5.137021542 |
| ERLNC1 | Estrogen Receptor Responsive LncRNA 1 | RNA Gene | 11 | GC01P204141 | 5.136617184 |
| BPHL | Biphenyl Hydrolase Like | Protein Coding | 35 | GC06P003118 | 5.13476181 |
| SLC6A17 | Solute Carrier Family 6 Member 17 | Protein Coding | 37 | GC01P110150 | 5.133689404 |
| TNFSF4 | TNF Superfamily Member 4 | Protein Coding | 38 | GC01M173183 | 5.130982399 |
| ZNF668 | Zinc Finger Protein 668 | Protein Coding | 32 | GC16M036572 | 5.129206657 |
| PLEC | Plectin | Protein Coding | 39 | GC08M144130 | 5.129047394 |
| TRIM29 | Tripartite Motif Containing 29 | Protein Coding | 35 | GC11M120111 | 5.128734589 |
| AKR1A1 | Aldo-Keto Reductase Family 1 Member A1 | Protein Coding | 39 | GC01P045550 | 5.128190041 |
| CUL4A | Cullin 4A | Protein Coding | 38 | GC13P113208 | 5.128170013 |
| EIF5A | Eukaryotic Translation Initiation Factor 5A | Protein Coding | 39 | GC17P007306 | 5.12816 |
| FREM2 | FRAS1 Related Extracellular Matrix 2 | Protein Coding | 36 | GC13P038687 | 5.127381802 |
| CCL22 | C-C Motif Chemokine Ligand 22 | Protein Coding | 35 | GC16P057359 | 5.126989841 |
| LOC110806306 | Telomerase RNA Component (TERC) Promoter | Biological Region | 3 | GC03P169764 | 5.126343727 |
| ARID5B | AT-Rich Interaction Domain 5B | Protein Coding | 37 | GC10P061901 | 5.12558794 |
| PNMA2 | PNMA Family Member 2 | Protein Coding | 32 | GC08M026504 | 5.125327587 |
| NPY | Neuropeptide Y | Protein Coding | 40 | GC07P024290 | 5.125225544 |
| ERCC6L2 | ERCC Excision Repair 6 Like 2 | Protein Coding | 34 | GC09P095871 | 5.124685764 |
| NMRAL2P | NmrA Like Redox Sensor 2, Pseudogene | Pseudogene | 10 | GC03P185961 | 5.124373913 |
| MIR363 | MicroRNA 363 | RNA Gene | 15 | GC0XM134299 | 5.120986462 |
| SNHG8 | Small Nucleolar RNA Host Gene 8 | RNA Gene | 19 | GC04P118278 | 5.119538307 |
| LINC01433 | Long Intergenic Non-Protein Coding RNA 1433 | RNA Gene | 14 | GC20P004199 | 5.119250298 |
| BCL3 | BCL3 Transcription Coactivator | Protein Coding | 36 | GC19P044742 | 5.11837244 |
| SPINK2 | Serine Peptidase Inhibitor Kazal Type 2 | Protein Coding | 35 | GC04M056809 | 5.115675926 |
| RNF213 | Ring Finger Protein 213 | Protein Coding | 35 | GC17P080260 | 5.115139961 |
| TGFBI | Transforming Growth Factor Beta Induced | Protein Coding | 42 | GC05P136027 | 5.114991665 |
| SLCO1B1 | Solute Carrier Organic Anion Transporter Family Member 1B1 | Protein Coding | 43 | GC12P021132 | 5.112770081 |
| E2F7 | E2F Transcription Factor 7 | Protein Coding | 35 | GC12M077021 | 5.112514496 |
| ADAMTS1 | ADAM Metallopeptidase With Thrombospondin Type 1 Motif 1 | Protein Coding | 41 | GC21M026835 | 5.112284184 |
| ACLY | ATP Citrate Lyase | Protein Coding | 42 | GC17M041866 | 5.107777596 |
| ANK1 | Ankyrin 1 | Protein Coding | 40 | GC08M041653 | 5.105013847 |
| ARNTL | Aryl Hydrocarbon Receptor Nuclear Translocator Like | Protein Coding | 37 | GC11P013276 | 5.104911804 |
| MAGEA10 | MAGE Family Member A10 | Protein Coding | 32 | GC0XM152133 | 5.102962494 |
| TCL1A | TCL1 Family AKT Coactivator A | Protein Coding | 39 | GC14M100467 | 5.101761818 |
| SLC4A7 | Solute Carrier Family 4 Member 7 | Protein Coding | 38 | GC03M027372 | 5.099075317 |
| MAP3K5 | Mitogen-Activated Protein Kinase Kinase Kinase 5 | Protein Coding | 42 | GC06M136557 | 5.098921776 |
| CD14 | CD14 Molecule | Protein Coding | 41 | GC05M140631 | 5.095517159 |
| IFNAR2 | Interferon Alpha And Beta Receptor Subunit 2 | Protein Coding | 44 | GC21P033229 | 5.092758656 |
| CHL1 | Cell Adhesion Molecule L1 Like | Protein Coding | 38 | GC03P000213 | 5.092078686 |
| VPS13B | Vacuolar Protein Sorting 13 Homolog B | Protein Coding | 36 | GC08P099011 | 5.091945648 |
| ATAD3B | ATPase Family AAA Domain Containing 3B | Protein Coding | 33 | GC01P003616 | 5.091795921 |
| DPAGT1 | Dolichyl-Phosphate N-Acetylglucosaminephosphotransferase 1 | Protein Coding | 40 | GC11M119096 | 5.091776848 |
| PER3 | Period Circadian Regulator 3 | Protein Coding | 38 | GC01P007785 | 5.089749813 |
| ADAM29 | ADAM Metallopeptidase Domain 29 | Protein Coding | 34 | GC04P174831 | 5.088877678 |
| DDX47 | DEAD-Box Helicase 47 | Protein Coding | 34 | GC12P020076 | 5.087636471 |
| H3-3B | H3.3 Histone B | Protein Coding | 32 | GC17M075807 | 5.087319851 |
| UGT1A3 | UDP Glucuronosyltransferase Family 1 Member A3 | Protein Coding | 35 | GC02P233729 | 5.086287498 |
| GRIN2A | Glutamate Ionotropic Receptor NMDA Type Subunit 2A | Protein Coding | 47 | GC16M009753 | 5.082766056 |
| MIR539 | MicroRNA 539 | RNA Gene | 17 | GC14P109544 | 5.082711697 |
| CENPA | Centromere Protein A | Protein Coding | 35 | GC02P026765 | 5.081109047 |
| CLCA1 | Chloride Channel Accessory 1 | Protein Coding | 38 | GC01P086468 | 5.077444553 |
| NCRUPAR | Non-Protein Coding RNA, Upstream Of F2R/PAR1 | RNA Gene | 13 | GC05P076711 | 5.075647354 |
| CCDC136 | Coiled-Coil Domain Containing 136 | Protein Coding | 31 | GC07P131672 | 5.07243681 |
| EIF4G1 | Eukaryotic Translation Initiation Factor 4 Gamma 1 | Protein Coding | 42 | GC03P184314 | 5.071403503 |
| EGLN3 | Egl-9 Family Hypoxia Inducible Factor 3 | Protein Coding | 42 | GC14M033924 | 5.070456505 |
| MEST | Mesoderm Specific Transcript | Protein Coding | 36 | GC07P130486 | 5.070156097 |
| GIMAP6 | GTPase, IMAP Family Member 6 | Protein Coding | 31 | GC07M150625 | 5.070066929 |
| SPANXA1 | Sperm Protein Associated With The Nucleus, X-Linked, Family Member A1 | Protein Coding | 24 | GC0XM141583 | 5.06467247 |
| LINC00092 | Long Intergenic Non-Protein Coding RNA 92 | RNA Gene | 17 | GC09M096019 | 5.064342499 |
| SGO1-AS1 | SGO1 Antisense RNA 1 | RNA Gene | 14 | GC03P020174 | 5.063592911 |
| PHF21A | PHD Finger Protein 21A | Protein Coding | 39 | GC11M086858 | 5.062745571 |
| GC | GC Vitamin D Binding Protein | Protein Coding | 40 | GC04M071741 | 5.062307358 |
| DDB1 | Damage Specific DNA Binding Protein 1 | Protein Coding | 39 | GC11M087077 | 5.056637764 |
| FKTN | Fukutin | Protein Coding | 35 | GC09P105558 | 5.056020737 |
| CARS2 | Cysteinyl-TRNA Synthetase 2, Mitochondrial | Protein Coding | 38 | GC13M110641 | 5.055632114 |
| CIC | Capicua Transcriptional Repressor | Protein Coding | 38 | GC19P042268 | 5.053683281 |
| TYRO3 | TYRO3 Protein Tyrosine Kinase | Protein Coding | 43 | GC15P041557 | 5.051902771 |
| DPP9 | Dipeptidyl Peptidase 9 | Protein Coding | 38 | GC19M004675 | 5.051825047 |
| ELP1 | Elongator Acetyltransferase Complex Subunit 1 | Protein Coding | 34 | GC09M108868 | 5.051721573 |
| LTB4R | Leukotriene B4 Receptor | Protein Coding | 40 | GC14P024311 | 5.051089287 |
| GP6 | Glycoprotein VI Platelet | Protein Coding | 41 | GC19M055013 | 5.05102396 |
| ARHGAP11A | Rho GTPase Activating Protein 11A | Protein Coding | 31 | GC15P032615 | 5.050933838 |
| PARK7 | Parkinsonism Associated Deglycase | Protein Coding | 40 | GC01P008090 | 5.049918175 |
| LRRFIP2 | LRR Binding FLII Interacting Protein 2 | Protein Coding | 35 | GC03M037052 | 5.049037933 |
| MIR124-2 | MicroRNA 124-2 | RNA Gene | 18 | GC08P064379 | 5.048075676 |
| CCNB3 | Cyclin B3 | Protein Coding | 32 | GC0XP050202 | 5.046703339 |
| ZNF652 | Zinc Finger Protein 652 | Protein Coding | 31 | GC17M049289 | 5.044558525 |
| LINC00210 | Long Intergenic Non-Protein Coding RNA 210 | RNA Gene | 13 | GC01P217892 | 5.043516159 |
| PKN1 | Protein Kinase N1 | Protein Coding | 41 | GC19P014433 | 5.043118477 |
| TMPO | Thymopoietin | Protein Coding | 41 | GC12P098515 | 5.039853573 |
| CUL2 | Cullin 2 | Protein Coding | 37 | GC10M035046 | 5.039754391 |

| | | | | | |
|---|---|---|---|---|---|
| RFC1 | Replication Factor C Subunit 1 | Protein Coding | 42 | GC04M039291 | 5.038022995 |
| ANKRD11 | Ankyrin Repeat Domain Containing 11 | Protein Coding | 35 | GC16M089267 | 5.033390045 |
| TNKS | Tankyrase | Protein Coding | 41 | GC08P009555 | 5.030833721 |
| CSAG3 | CSAG Family Member 3 | Protein Coding | 15 | GC0XP152753 | 5.029365063 |
| EIF2S1 | Eukaryotic Translation Initiation Factor 2 Subunit Alpha | Protein Coding | 39 | GC14P067359 | 5.029092789 |
| LINC00623 | Long Intergenic Non-Protein Coding RNA 623 | RNA Gene | 14 | GC01P121209 | 5.028814316 |
| CISH | Cytokine Inducible SH2 Containing Protein | Protein Coding | 41 | GC03M051119 | 5.028710365 |
| EDN3 | Endothelin 3 | Protein Coding | 42 | GC20P059300 | 5.028588295 |
| LINC00313 | Long Intergenic Non-Protein Coding RNA 313 | RNA Gene | 19 | GC21M043440 | 5.024767399 |
| UBE2L3 | Ubiquitin Conjugating Enzyme E2 L3 | Protein Coding | 40 | GC22P021549 | 5.023697376 |
| TRPV1 | Transient Receptor Potential Cation Channel Subfamily V Member 1 | Protein Coding | 42 | GC17M003565 | 5.023572922 |
| RFC3 | Replication Factor C Subunit 3 | Protein Coding | 35 | GC13P033818 | 5.022357941 |
| GBA | Glucosylceramidase Beta | Protein Coding | 45 | GC01M155234 | 5.022312641 |
| PDGFC | Platelet Derived Growth Factor C | Protein Coding | 39 | GC04M156760 | 5.019841194 |
| BIN1 | Bridging Integrator 1 | Protein Coding | 40 | GC02M127048 | 5.019824028 |
| NXF2 | Nuclear RNA Export Factor 2 | Protein Coding | 28 | GC0XP102247 | 5.019478321 |
| NUP133 | Nucleoporin 133 | Protein Coding | 36 | GC01M229484 | 5.019286156 |
| LINC00968 | Long Intergenic Non-Protein Coding RNA 968 | RNA Gene | 14 | GC08M056496 | 5.019138813 |
| KNG1 | Kininogen 1 | Protein Coding | 42 | GC03P186717 | 5.018423557 |
| CXCL2 | C-X-C Motif Chemokine Ligand 2 | Protein Coding | 35 | GC04M074097 | 5.013587475 |
| CD209 | CD209 Molecule | Protein Coding | 39 | GC19M007739 | 5.011932373 |
| A2M | Alpha-2-Macroglobulin | Protein Coding | 40 | GC12M009067 | 5.011802197 |
| GIPC3 | GIPC PDZ Domain Containing Family Member 3 | Protein Coding | 34 | GC19P003585 | 5.011521339 |
| FUBP1 | Far Upstream Element Binding Protein 1 | Protein Coding | 35 | GC01M077944 | 5.009647369 |
| CBLB | Cbl Proto-Oncogene B | Protein Coding | 40 | GC03M105655 | 5.007861137 |
| LINC01589 | Long Intergenic Non-Protein Coding RNA 1589 | RNA Gene | 13 | GC22M056167 | 5.007210732 |
| HTT | Huntingtin | Protein Coding | 39 | GC04P003041 | 5.006364822 |
| BCL2L12 | BCL2 Like 12 | Protein Coding | 34 | GC19P064081 | 5.003405571 |
| SBF2-AS1 | SBF2 Antisense RNA 1 | RNA Gene | 15 | GC11P009758 | 5.001957417 |
| DHX38 | DEAH-Box Helicase 38 | Protein Coding | 38 | GC16P072127 | 5.001885414 |
| DONSON | DNA Replication Fork Stabilization Factor DONSON | Protein Coding | 31 | GC21M033559 | 5.001728535 |
| CST3 | Cystatin C | Protein Coding | 39 | GC20M023657 | 4.999483109 |
| RBM28 | RNA Binding Motif Protein 28 | Protein Coding | 35 | GC07M128399 | 4.998297691 |
| GH-LCR | Growth Hormone Locus Control Region | Biological Region | 3 | GC17P063917 | 4.997074604 |
| SMAD5 | SMAD Family Member 5 | Protein Coding | 36 | GC05P136132 | 4.996246815 |
| DNM1L | Dynamin 1 Like | Protein Coding | 42 | GC12P032679 | 4.994317532 |
| SOX6 | SRY-Box Transcription Factor 6 | Protein Coding | 39 | GC11M015949 | 4.992684364 |
| CYP4Z1 | Cytochrome P450 Family 4 Subfamily Z Member 1 | Protein Coding | 32 | GC01P047067 | 4.991425037 |
| AK9 | Adenylate Kinase 9 | Protein Coding | 30 | GC06M109492 | 4.990423203 |
| ZNF461 | Zinc Finger Protein 461 | Protein Coding | 31 | GC19M063750 | 4.98679924 |
| ATP2A3 | ATPase Sarcoplasmic/Endoplasmic Reticulum Ca2+ Transporting 3 | Protein Coding | 41 | GC17M003923 | 4.983792782 |
| IL23R | Interleukin 23 Receptor | Protein Coding | 40 | GC01P067138 | 4.982830048 |
| C14orf132 | Chromosome 14 Open Reading Frame 132 | Protein Coding | 21 | GC14P096040 | 4.98196888 |
| KRT2 | Keratin 2 | Protein Coding | 38 | GC12M052701 | 4.981862068 |
| KCNH2 | Potassium Voltage-Gated Channel Subfamily H Member 2 | Protein Coding | 46 | GC07M150944 | 4.981368065 |
| ACTR3 | Actin Related Protein 3 | Protein Coding | 36 | GC02P113889 | 4.980555534 |
| SSTR4 | Somatostatin Receptor 4 | Protein Coding | 39 | GC20P023035 | 4.976800919 |
| OLIG2 | Oligodendrocyte Transcription Factor 2 | Protein Coding | 36 | GC21P033025 | 4.974297523 |
| MSH4 | MutS Homolog 4 | Protein Coding | 34 | GC01P075796 | 4.97428894 |
| MIR572 | MicroRNA 572 | RNA Gene | 14 | GC04P017692 | 4.972788334 |
| ATP4A | ATPase H+/K+ Transporting Subunit Alpha | Protein Coding | 37 | GC19M064561 | 4.969070435 |
| MAGEA2 | MAGE Family Member A2 | Protein Coding | 26 | GC0XM152749 | 4.963074684 |
| STK33 | Serine/Threonine Kinase 33 | Protein Coding | 38 | GC11M008335 | 4.961929321 |
| NFATC2 | Nuclear Factor Of Activated T Cells 2 | Protein Coding | 39 | GC20M051386 | 4.960409164 |
| EGLN1 | Egl-9 Family Hypoxia Inducible Factor 1 | Protein Coding | 44 | GC01M231363 | 4.956282616 |
| ARTN | Artemin | Protein Coding | 37 | GC01P043933 | 4.956092834 |
| SERPINE2 | Serpin Family E Member 2 | Protein Coding | 38 | GC02M223975 | 4.955920219 |
| IGSF9B | Immunoglobulin Superfamily Member 9B | Protein Coding | 28 | GC11M133978 | 4.955321312 |
| NXF2B | Nuclear RNA Export Factor 2B | Protein Coding | 24 | GC0XM102360 | 4.954352379 |
| ATAD5 | ATPase Family AAA Domain Containing 5 | Protein Coding | 30 | GC17P052589 | 4.952816963 |
| BRCC3 | BRCA1/BRCA2-Containing Complex Subunit 3 | Protein Coding | 39 | GC0XP155071 | 4.952674866 |
| TUBB2A | Tubulin Beta 2A Class IIa | Protein Coding | 40 | GC06M003153 | 4.952548027 |
| SLC7A11 | Solute Carrier Family 7 Member 11 | Protein Coding | 39 | GC04M138164 | 4.95241642 |
| SUV39H1 | SUV39H1 Histone Lysine Methyltransferase | Protein Coding | 39 | GC0XP050511 | 4.952056885 |
| H2AC21 | H2A Clustered Histone 21 | Protein Coding | 26 | GC01M151593 | 4.950304031 |
| MAPRE2 | Microtubule Associated Protein RP/EB Family Member 2 | Protein Coding | 38 | GC18P034976 | 4.950170994 |
| MIR302C | MicroRNA 302c | RNA Gene | 19 | GC04M112763 | 4.9487257 |
| RNF113A | Ring Finger Protein 113A | Protein Coding | 33 | GC0XM119870 | 4.947847366 |
| TFEB | Transcription Factor EB | Protein Coding | 36 | GC06M063818 | 4.947451591 |
| MIR3936HG | MIR3936 Host Gene | RNA Gene | 13 | GC05M132312 | 4.946856022 |
| IRF3 | Interferon Regulatory Factor 3 | Protein Coding | 40 | GC19M049659 | 4.945557117 |
| EPPIN | Epididymal Peptidase Inhibitor | Protein Coding | 31 | GC20M045613 | 4.944204807 |
| CGB7 | Chorionic Gonadotropin Subunit Beta 7 | Protein Coding | 30 | GC19M049054 | 4.943185329 |
| STARD10 | StAR Related Lipid Transfer Domain Containing 10 | Protein Coding | 34 | GC11M087548 | 4.942797184 |
| HAGLROS | HAGLR Opposite Strand LncRNA | RNA Gene | 14 | GC02P176237 | 4.942245483 |
| CA12 | Carbonic Anhydrase 12 | Protein Coding | 44 | GC15M063321 | 4.941808224 |
| LINC01852 | Long Intergenic Non-Protein Coding RNA 1852 | RNA Gene | 12 | GC15M038062 | 4.941483498 |
| ADH5 | Alcohol Dehydrogenase 5 (Class III), Chi Polypeptide | Protein Coding | 42 | GC04M099070 | 4.940793991 |
| EIF2A | Eukaryotic Translation Initiation Factor 2A | Protein Coding | 35 | GC03P150546 | 4.938274384 |
| KIF4A | Kinesin Family Member 4A | Protein Coding | 35 | GC0XP070290 | 4.938241482 |
| MAGED1 | MAGE Family Member D1 | Protein Coding | 37 | GC0XP051803 | 4.936894417 |
| CEMIP2 | Cell Migration Inducing Hyaluronidase 2 | Protein Coding | 29 | GC09M071684 | 4.936131477 |
| MIR1247 | MicroRNA 1247 | RNA Gene | 17 | GC14M101560 | 4.935015678 |
| DCD | Dermcidin | Protein Coding | 34 | GC12M054644 | 4.934580326 |
| LARP1 | La Ribonucleoprotein 1, Translational Regulator | Protein Coding | 35 | GC05P154682 | 4.929757118 |
| ALDH1L1 | Aldehyde Dehydrogenase 1 Family Member L1 | Protein Coding | 35 | GC03M126103 | 4.929436684 |
| NOP2 | NOP2 Nucleolar Protein | Protein Coding | 32 | GC12M006556 | 4.926065922 |
| SERPINH1 | Serpin Family H Member 1 | Protein Coding | 41 | GC11P075562 | 4.925755978 |
| SEMA3C | Semaphorin 3C | Protein Coding | 40 | GC07M080742 | 4.925752163 |
| VEZT | Vezatin, Adherens Junctions Transmembrane Protein | Protein Coding | 31 | GC12P095217 | 4.925257683 |
| LMTK2 | Lemur Tyrosine Kinase 2 | Protein Coding | 35 | GC07P098106 | 4.92321825 |
| TNN | Tenascin N | Protein Coding | 32 | GC01P175067 | 4.922466278 |
| FERMT1 | FERM Domain Containing Kindlin 1 | Protein Coding | 39 | GC20M006074 | 4.921780109 |
| KTN1 | Kinectin 1 | Protein Coding | 36 | GC14P055559 | 4.921311378 |
| RHD | Rh Blood Group D Antigen | Protein Coding | 38 | GC01P025272 | 4.913354874 |

| | | | | | |
|---|---|---|---|---|---|
| BCCIP | BRCA2 And CDKN1A Interacting Protein | Protein Coding | 32 | GC10P125823 | 4.913170815 |
| MBOAT7 | Membrane Bound O-Acyltransferase Domain Containing 7 | Protein Coding | 36 | GC19M054173 | 4.912611961 |
| HDC | Histidine Decarboxylase | Protein Coding | 40 | GC15M050241 | 4.911090851 |
| GJB1 | Gap Junction Protein Beta 1 | Protein Coding | 42 | GC0XP071212 | 4.91063118 |
| HTR3A | 5-Hydroxytryptamine Receptor 3A | Protein Coding | 42 | GC11P113974 | 4.910252571 |
| GNE | Glucosamine (UDP-N-Acetyl)-2-Epimerase/N-Acetylmannosamine Kinase | Protein Coding | 39 | GC09M036214 | 4.910132408 |
| SSX3 | SSX Family Member 3 | Protein Coding | 31 | GC0XM048346 | 4.907766342 |
| ANKRD30B | Ankyrin Repeat Domain 30B | Protein Coding | 24 | GC18P017200 | 4.907586098 |
| PIM2 | Pim-2 Proto-Oncogene, Serine/Threonine Kinase | Protein Coding | 39 | GC0XM048913 | 4.90666008 |
| BMP1 | Bone Morphogenetic Protein 1 | Protein Coding | 44 | GC08P022164 | 4.906407356 |
| UBL4B | Ubiquitin Like 4B | Protein Coding | 28 | GC01P110357 | 4.90576458 |
| SUSD3 | Sushi Domain Containing 3 | Protein Coding | 32 | GC09P093058 | 4.905246735 |
| UBE2D1 | Ubiquitin Conjugating Enzyme E2 D1 | Protein Coding | 40 | GC10P058334 | 4.905092239 |
| SPRED2 | Sprouty Related EVH1 Domain Containing 2 | Protein Coding | 38 | GC02M065307 | 4.904588223 |
| MIR627 | MicroRNA 627 | RNA Gene | 15 | GC15M042199 | 4.90412569 |
| SLC2A3 | Solute Carrier Family 2 Member 3 | Protein Coding | 43 | GC12M007919 | 4.903609276 |
| SLCO2B1 | Solute Carrier Organic Anion Transporter Family Member 2B1 | Protein Coding | 39 | GC11P077658 | 4.902615547 |
| CD33 | CD33 Molecule | Protein Coding | 40 | GC19P064163 | 4.902365208 |
| PPIA | Peptidylprolyl Isomerase A | Protein Coding | 42 | GC07P044807 | 4.901844025 |
| HAX1 | HCLS1 Associated Protein X-1 | Protein Coding | 38 | GC01P154273 | 4.901639462 |
| BAGE | B Melanoma Antigen | Protein Coding | 15 | GC21U900396 | 4.898623466 |
| MIR92B | MicroRNA 92b | RNA Gene | 17 | GC01P155195 | 4.896132946 |
| TSR2 | TSR2 Ribosome Maturation Factor | Protein Coding | 32 | GC0XP054509 | 4.895929813 |
| DCTN1 | Dynactin Subunit 1 | Protein Coding | 41 | GC02M074361 | 4.895898819 |
| CARM1 | Coactivator Associated Arginine Methyltransferase 1 | Protein Coding | 42 | GC19P010871 | 4.895013332 |
| UNG | Uracil DNA Glycosylase | Protein Coding | 42 | GC12P109097 | 4.893100739 |
| GFRA1 | GDNF Family Receptor Alpha 1 | Protein Coding | 39 | GC10M116056 | 4.892486572 |
| PYCR1 | Pyrroline-5-Carboxylate Reductase 1 | Protein Coding | 41 | GC17M081932 | 4.892393112 |
| EPG5 | Ectopic P-Granules 5 Autophagy Tethering Factor | Protein Coding | 34 | GC18M045800 | 4.890961647 |
| FBXL19-AS1 | FBXL19 Antisense RNA 1 | RNA Gene | 18 | GC16M036568 | 4.889866829 |
| TNFRSF19 | TNF Receptor Superfamily Member 19 | Protein Coding | 35 | GC13P023570 | 4.888342381 |
| SIPA1L1 | Signal Induced Proliferation Associated 1 Like 1 | Protein Coding | 34 | GC14P071320 | 4.888022423 |
| LPCAT1 | Lysophosphatidylcholine Acyltransferase 1 | Protein Coding | 32 | GC05M001456 | 4.886260986 |
| TIMP4 | TIMP Metallopeptidase Inhibitor 4 | Protein Coding | 35 | GC03M012153 | 4.884916306 |
| TRIM27 | Tripartite Motif Containing 27 | Protein Coding | 38 | GC06M028903 | 4.884830475 |
| PRICKLE4 | Prickle Planar Cell Polarity Protein 4 | Protein Coding | 31 | GC06P041780 | 4.883995533 |
| TXNRD1 | Thioredoxin Reductase 1 | Protein Coding | 42 | GC12P104215 | 4.881686211 |
| APRT | Adenine Phosphoribosyltransferase | Protein Coding | 42 | GC16M088810 | 4.878793716 |
| MVK | Mevalonate Kinase | Protein Coding | 43 | GC12P109573 | 4.874699593 |
| SLC2A2 | Solute Carrier Family 2 Member 2 | Protein Coding | 44 | GC03M170996 | 4.87451458 |
| PLCG2 | Phospholipase C Gamma 2 | Protein Coding | 47 | GC16P081773 | 4.874278069 |
| COPB1 | COPI Coat Complex Subunit Beta 1 | Protein Coding | 36 | GC11M014436 | 4.873810768 |
| MAPKAP1 | MAPK Associated Protein 1 | Protein Coding | 38 | GC09M125437 | 4.872661591 |
| NR2F2 | Nuclear Receptor Subfamily 2 Group F Member 2 | Protein Coding | 45 | GC15P096325 | 4.872429848 |
| LMO2 | LIM Domain Only 2 | Protein Coding | 38 | GC11M033858 | 4.871370792 |
| DDX17 | DEAD-Box Helicase 17 | Protein Coding | 36 | GC22M038483 | 4.869020462 |
| GNB2 | G Protein Subunit Beta 2 | Protein Coding | 38 | GC07P100673 | 4.868811131 |
| ELP4 | Elongator Acetyltransferase Complex Subunit 4 | Protein Coding | 35 | GC11P031509 | 4.868697643 |
| PMM2 | Phosphomannomutase 2 | Protein Coding | 42 | GC16P008788 | 4.868171692 |
| PI4KA | Phosphatidylinositol 4-Kinase Alpha | Protein Coding | 43 | GC22M020707 | 4.867447853 |
| DSCR8 | Down Syndrome Critical Region 8 | RNA Gene | 23 | GC21P038121 | 4.866728783 |
| MAMLD1 | Mastermind Like Domain Containing 1 | Protein Coding | 32 | GC0XP150361 | 4.866575718 |
| AICDA | Activation Induced Cytidine Deaminase | Protein Coding | 39 | GC12M008602 | 4.863744259 |
| MIR662 | MicroRNA 662 | RNA Gene | 15 | GC16P010652 | 4.861706257 |
| MT-ND3 | Mitochondrially Encoded NADH:Ubiquinone Oxidoreductase Core Subunit 3 | Protein Coding | 29 | GCMTP010061 | 4.859936714 |
| LDHC | Lactate Dehydrogenase C | Protein Coding | 38 | GC11P018487 | 4.858883858 |
| PLD1 | Phospholipase D1 | Protein Coding | 44 | GC03M171600 | 4.857728958 |
| KCNJ5 | Potassium Inwardly Rectifying Channel Subfamily J Member 5 | Protein Coding | 42 | GC11P128891 | 4.854741096 |
| SLC7A1 | Solute Carrier Family 7 Member 1 | Protein Coding | 39 | GC13M029509 | 4.852545738 |
| NR4A2 | Nuclear Receptor Subfamily 4 Group A Member 2 | Protein Coding | 42 | GC02M156324 | 4.851793766 |
| CCR1 | C-C Motif Chemokine Receptor 1 | Protein Coding | 41 | GC03M046218 | 4.851389885 |
| CES2 | Carboxylesterase 2 | Protein Coding | 40 | GC16P066934 | 4.850315571 |
| RPS3 | Ribosomal Protein S3 | Protein Coding | 38 | GC11P077659 | 4.849243164 |
| FBXO31 | F-Box Protein 31 | Protein Coding | 32 | GC16M087326 | 4.848374367 |
| TUBA4B | Tubulin Alpha 4b | Protein Coding | 21 | GC02P219253 | 4.8471241 |
| NELFA | Negative Elongation Factor Complex Member A | Protein Coding | 31 | GC04M002151 | 4.845867157 |
| TH | Tyrosine Hydroxylase | Protein Coding | 46 | GC11M002163 | 4.845444202 |
| FADS1 | Fatty Acid Desaturase 1 | Protein Coding | 40 | GC11M061799 | 4.844526291 |
| HBA1 | Hemoglobin Subunit Alpha 1 | Protein Coding | 38 | GC16P010620 | 4.844357967 |
| TLR6 | Toll Like Receptor 6 | Protein Coding | 39 | GC04M038828 | 4.842726707 |
| OXTR | Oxytocin Receptor | Protein Coding | 42 | GC03M008767 | 4.842313766 |
| PCNT | Pericentrin | Protein Coding | 39 | GC21P046324 | 4.836910248 |
| POLR2E | RNA Polymerase II, I And III Subunit E | Protein Coding | 38 | GC19M001086 | 4.835144997 |
| C1S | Complement C1s | Protein Coding | 44 | GC12P019859 | 4.835076332 |
| HSD17B4 | Hydroxysteroid 17-Beta Dehydrogenase 4 | Protein Coding | 41 | GC05P119452 | 4.834786415 |
| MT-ATP6 | Mitochondrially Encoded ATP Synthase Membrane Subunit 6 | Protein Coding | 29 | GCMTP008531 | 4.833367825 |
| MIR20B | MicroRNA 20b | RNA Gene | 16 | GC0XM134302 | 4.831531525 |
| ETV7 | ETS Variant Transcription Factor 7 | Protein Coding | 34 | GC06M063759 | 4.831004143 |
| OVAAL | Ovarian Adenocarcinoma Amplified Long Non-Coding RNA | RNA Gene | 14 | GC01P180509 | 4.827997208 |
| CKAP4 | Cytoskeleton Associated Protein 4 | Protein Coding | 33 | GC12M106237 | 4.82610321 |
| PPP6C | Protein Phosphatase 6 Catalytic Subunit | Protein Coding | 39 | GC09M125152 | 4.823444366 |
| PUF60 | Poly(U) Binding Splicing Factor 60 | Protein Coding | 37 | GC08M143816 | 4.822824955 |
| PLD2 | Phospholipase D2 | Protein Coding | 43 | GC17P004808 | 4.822061539 |
| CCL7 | C-C Motif Chemokine Ligand 7 | Protein Coding | 38 | GC17P034270 | 4.821123123 |
| ZNF793 | Zinc Finger Protein 793 | Protein Coding | 28 | GC19P037506 | 4.820638657 |
| SORL1 | Sortilin Related Receptor 1 | Protein Coding | 42 | GC11P121452 | 4.82036829 |
| CDR2 | Cerebellar Degeneration Related Protein 2 | Protein Coding | 35 | GC16M023011 | 4.819624901 |
| PSMB9 | Proteasome 20S Subunit Beta 9 | Protein Coding | 42 | GC06P080391 | 4.81571722 |
| ASAP3 | ArfGAP With SH3 Domain, Ankyrin Repeat And PH Domain 3 | Protein Coding | 30 | GC01M023428 | 4.815396309 |
| CRY2 | Cryptochrome Circadian Regulator 2 | Protein Coding | 36 | GC11P046453 | 4.814908504 |
| SNW1 | SNW Domain Containing 1 | Protein Coding | 38 | GC14M077717 | 4.810751915 |
| STAG2 | Stromal Antigen 2 | Protein Coding | 40 | GC0XP123960 | 4.810135841 |
| ASPM | Assembly Factor For Spindle Microtubules | Protein Coding | 35 | GC01M197084 | 4.80963707 |
| DAG1 | Dystroglycan 1 | Protein Coding | 41 | GC03P049587 | 4.8082304 |
| CAMTA1 | Calmodulin Binding Transcription Activator 1 | Protein Coding | 37 | GC01P006947 | 4.807750702 |

| | | | | | |
|---|---|---|---|---|---|
| LMOD1 | Leiomodin 1 | Protein Coding | 38 | GC01M201896 | 4.806700706 |
| KIF26B | Kinesin Family Member 26B | Protein Coding | 32 | GC01P245154 | 4.805636406 |
| LOC111099027 | Transmembrane Protease Serine 2 Breakpoint Cluster Recombination Region | Biological Region | 2 | GC21P041485 | 4.803606987 |
| LOC111099028 | ERG, ETS Transcription Factor Breakpoint Cluster Recombination Region | Biological Region | 2 | GC21P038454 | 4.803606987 |
| GART | Phosphoribosylglycinamide Formyltransferase, Phosphoribosylglycinamide Synthetase, Phosphoribosylaminoimidazole Synthetase | Protein Coding | 38 | GC21M033503 | 4.803447723 |
| LZTS2 | Leucine Zipper Tumor Suppressor 2 | Protein Coding | 31 | GC10P100996 | 4.802122593 |
| RBP4 | Retinol Binding Protein 4 | Protein Coding | 41 | GC10M093591 | 4.801847935 |
| LPA | Lipoprotein(A) | Protein Coding | 37 | GC06M160531 | 4.801377773 |
| DGKE | Diacylglycerol Kinase Epsilon | Protein Coding | 43 | GC17P056834 | 4.799853325 |
| SLC1A5 | Solute Carrier Family 1 Member 5 | Protein Coding | 39 | GC19M064014 | 4.79902935 |
| GLO1 | Glyoxalase I | Protein Coding | 41 | GC06M063769 | 4.798811913 |
| FARP1 | FERM, ARH/RhoGEF And Pleckstrin Domain Protein 1 | Protein Coding | 34 | GC13P098142 | 4.797257423 |
| CENPC | Centromere Protein C | Protein Coding | 30 | GC04M067471 | 4.795911789 |
| TRIM21 | Tripartite Motif Containing 21 | Protein Coding | 38 | GC11M004384 | 4.791037083 |
| VAV1 | Vav Guanine Nucleotide Exchange Factor 1 | Protein Coding | 41 | GC19P006772 | 4.785733223 |
| RRP1B | Ribosomal RNA Processing 1B | Protein Coding | 34 | GC21P043659 | 4.78426218 |
| IL1R2 | Interleukin 1 Receptor Type 2 | Protein Coding | 40 | GC02P101991 | 4.784140587 |
| GAS1 | Growth Arrest Specific 1 | Protein Coding | 38 | GC09M086944 | 4.782791138 |
| ITPR3 | Inositol 1,4,5-Trisphosphate Receptor Type 3 | Protein Coding | 41 | GC06P033620 | 4.781419277 |
| MSTO2P | Misato Family Member 2, Pseudogene | Pseudogene | 12 | GC01P155745 | 4.774504185 |
| KAT2A | Lysine Acetyltransferase 2A | Protein Coding | 42 | GC17M042113 | 4.774433136 |
| CDR1 | Cerebellar Degeneration Related Protein 1 | Protein Coding | 31 | GC0XM140782 | 4.774364471 |
| CD79B | CD79b Molecule | Protein Coding | 42 | GC17M063928 | 4.774235725 |
| ATG5 | Autophagy Related 5 | Protein Coding | 39 | GC06M106045 | 4.773998737 |
| DUSP13 | Dual Specificity Phosphatase 13 | Protein Coding | 35 | GC10M075094 | 4.773495674 |
| CCL21 | C-C Motif Chemokine Ligand 21 | Protein Coding | 37 | GC09M034709 | 4.772383213 |
| SIRT2 | Sirtuin 2 | Protein Coding | 43 | GC19M038878 | 4.769716263 |
| MAFA-AS1 | MAFA Antisense RNA 1 | RNA Gene | 15 | GC08P143417 | 4.768903255 |
| MAGEA9 | MAGE Family Member A9 | Protein Coding | 29 | GC0XP149781 | 4.768284321 |
| PINX1 | PIN2 (TERF1) Interacting Telomerase Inhibitor 1 | Protein Coding | 35 | GC08M010764 | 4.764407158 |
| NUF2 | NUF2 Component Of NDC80 Kinetochore Complex | Protein Coding | 34 | GC01P163266 | 4.763676167 |
| MCM6 | Minichromosome Maintenance Complex Component 6 | Protein Coding | 38 | GC02M135839 | 4.762781143 |
| REN | Renin | Protein Coding | 44 | GC01M204154 | 4.76198864 |
| NEFL | Neurofilament Light Chain | Protein Coding | 40 | GC08M024950 | 4.7613554 |
| PRSS58 | Serine Protease 58 | Protein Coding | 29 | GC07M142263 | 4.760660172 |
| TUBA1B | Tubulin Alpha 1b | Protein Coding | 38 | GC12M049127 | 4.760059834 |
| FECH | Ferrochelatase | Protein Coding | 42 | GC18M057544 | 4.759865284 |
| CD83 | CD83 Molecule | Protein Coding | 38 | GC06P014117 | 4.756804466 |
| FGF21 | Fibroblast Growth Factor 21 | Protein Coding | 36 | GC19P064016 | 4.750538826 |
| MIR583HG | MIR583 Host Gene | RNA Gene | 11 | GC05P096051 | 4.750257492 |
| KRIT1 | KRIT1 Ankyrin Repeat Containing | Protein Coding | 39 | GC07M092198 | 4.749622822 |
| ANKRD26 | Ankyrin Repeat Domain Containing 26 | Protein Coding | 36 | GC10M026938 | 4.747881413 |
| FAH | Fumarylacetoacetate Hydrolase | Protein Coding | 42 | GC15P080152 | 4.746041298 |
| SFTA1P | Surfactant Associated 1, LncRNA | Pseudogene | 17 | GC10M010784 | 4.746029854 |
| PC | Pyruvate Carboxylase | Protein Coding | 42 | GC11M066848 | 4.745041847 |
| CSTA | Cystatin A | Protein Coding | 40 | GC03P122325 | 4.741933823 |
| MTMR2 | Myotubularin Related Protein 2 | Protein Coding | 40 | GC11M095942 | 4.741735458 |
| FAR2P1 | Fatty Acyl-CoA Reductase 2 Pseudogene 1 | Pseudogene | 11 | GC02M130012 | 4.741438866 |
| EPHB3 | EPH Receptor B3 | Protein Coding | 42 | GC03P184561 | 4.740508556 |
| NTF3 | Neurotrophin 3 | Protein Coding | 38 | GC12P005432 | 4.73814106 |
| CARD11 | Caspase Recruitment Domain Family Member 11 | Protein Coding | 42 | GC07M002906 | 4.737885952 |
| SNRK | SNF Related Kinase | Protein Coding | 35 | GC03P043303 | 4.73752594 |
| THBS2 | Thrombospondin 2 | Protein Coding | 41 | GC06M169215 | 4.737342834 |
| INPP4B | Inositol Polyphosphate-4-Phosphatase Type II B | Protein Coding | 34 | GC04M142023 | 4.735616207 |
| TNIK | TRAF2 And NCK Interacting Kinase | Protein Coding | 39 | GC03M171061 | 4.735396862 |
| IL3RA | Interleukin 3 Receptor Subunit Alpha | Protein Coding | 38 | GC0XP001336 | 4.735356331 |
| SYNPO2 | Synaptopodin 2 | Protein Coding | 32 | GC04P118850 | 4.735284805 |
| HIP1 | Huntingtin Interacting Protein 1 | Protein Coding | 36 | GC07M075533 | 4.734992981 |
| TCP1 | T-Complex 1 | Protein Coding | 38 | GC06M159778 | 4.734492302 |
| RECQL5 | RecQ Like Helicase 5 | Protein Coding | 35 | GC17M075626 | 4.73259449 |
| SIRT7 | Sirtuin 7 | Protein Coding | 36 | GC17M081911 | 4.728888512 |
| MT-ND2 | Mitochondrially Encoded NADH:Ubiquinone Oxidoreductase Core Subunit 2 | Protein Coding | 31 | GCMTP004472 | 4.728731632 |
| NTN4 | Netrin 4 | Protein Coding | 35 | GC12M095657 | 4.728415012 |
| RHOD | Ras Homolog Family Member D | Protein Coding | 34 | GC11P069689 | 4.727797985 |
| PTPRN2 | Protein Tyrosine Phosphatase Receptor Type N2 | Protein Coding | 40 | GC07M157539 | 4.727274418 |
| MACROH2A1 | MacroH2A.1 Histone | Protein Coding | 32 | GC05M135334 | 4.723358154 |
| CYP4B1 | Cytochrome P450 Family 4 Subfamily B Member 1 | Protein Coding | 39 | GC01P046757 | 4.72301054 |
| DBN1 | Drebrin 1 | Protein Coding | 36 | GC05M177456 | 4.721851349 |
| NEU1 | Neuraminidase 1 | Protein Coding | 40 | GC06M031857 | 4.72180891 |
| DLG5 | Discs Large MAGUK Scaffold Protein 5 | Protein Coding | 35 | GC10M077790 | 4.721253395 |
| TFDP3 | Transcription Factor Dp Family Member 3 | Protein Coding | 28 | GC0XM133216 | 4.721083641 |
| NOTUM | Notum, Palmitoleoyl-Protein Carboxylesterase | Protein Coding | 32 | GC17M081952 | 4.719828606 |
| NEDD9 | Neural Precursor Cell Expressed, Developmentally Down-Regulated 9 | Protein Coding | 38 | GC06M011183 | 4.718232155 |
| CCR4 | C-C Motif Chemokine Receptor 4 | Protein Coding | 40 | GC03P032951 | 4.716775894 |
| NAA10 | N-Alpha-Acetyltransferase 10, NatA Catalytic Subunit | Protein Coding | 38 | GC0XM153929 | 4.716059685 |
| HARBI1 | Harbinger Transposase Derived 1 | Protein Coding | 28 | GC11M086864 | 4.715942383 |
| HOXB8 | Homeobox B8 | Protein Coding | 32 | GC17M048611 | 4.712140083 |
| BCL9L | BCL9 Like | Protein Coding | 31 | GC11M118893 | 4.711149693 |
| ABCB6 | ATP Binding Cassette Subfamily B Member 6 (Langereis Blood Group) | Protein Coding | 42 | GC02M219209 | 4.702810764 |
| MERTK | MER Proto-Oncogene, Tyrosine Kinase | Protein Coding | 46 | GC02P111898 | 4.6991539 |
| PSMA7 | Proteasome 20S Subunit Alpha 7 | Protein Coding | 40 | GC20M062136 | 4.694903374 |
| PPIL4 | Peptidylprolyl Isomerase Like 4 | Protein Coding | 32 | GC06M149504 | 4.693579674 |
| MT-ND5 | Mitochondrially Encoded NADH:Ubiquinone Oxidoreductase Core Subunit 5 | Protein Coding | 30 | GCMTP012339 | 4.692240238 |
| RHBDD2 | Rhomboid Domain Containing 2 | Protein Coding | 32 | GC07P075842 | 4.685530186 |
| POLR3A | RNA Polymerase III Subunit A | Protein Coding | 39 | GC10M078440 | 4.680984974 |
| CRKL | CRK Like Proto-Oncogene, Adaptor Protein | Protein Coding | 41 | GC22P020917 | 4.679470062 |
| UBR4 | Ubiquitin Protein Ligase E3 Component N-Recognin 4 | Protein Coding | 35 | GC01M019074 | 4.67932415 |
| SLCO2A1 | Solute Carrier Organic Anion Transporter Family Member 2A1 | Protein Coding | 39 | GC03M133932 | 4.671932697 |
| GSDMB | Gasdermin B | Protein Coding | 32 | GC17M039904 | 4.671636105 |
| TTN-AS1 | TTN Antisense RNA 1 | RNA Gene | 15 | GC02P178521 | 4.670701027 |
| PWRN1 | Prader-Willi Region Non-Protein Coding RNA 1 | RNA Gene | 19 | GC15P040419 | 4.669839859 |
| ENPEP | Glutamyl Aminopeptidase | Protein Coding | 42 | GC04P110365 | 4.668229103 |
| LINC00880 | Long Intergenic Non-Protein Coding RNA 880 | RNA Gene | 15 | GC03M157081 | 4.667735577 |

| | | | | | |
|---|---|---|---|---|---|
| APPL1 | Adaptor Protein, Phosphotyrosine Interacting With PH Domain And Leucine Zipper 1 | Protein Coding | 40 | GC03P057227 | 4.666339397 |
| BABAM1 | BRISC And BRCA1 A Complex Member 1 | Protein Coding | 32 | GC19P063104 | 4.666136742 |
| UGCG | UDP-Glucose Ceramide Glucosyltransferase | Protein Coding | 38 | GC09P111896 | 4.661430359 |
| INTS7 | Integrator Complex Subunit 7 | Protein Coding | 32 | GC01M211940 | 4.660121441 |
| PCBP1 | Poly(RC) Binding Protein 1 | Protein Coding | 37 | GC02P070087 | 4.656996727 |
| AQP2 | Aquaporin 2 | Protein Coding | 42 | GC12P049950 | 4.654447556 |
| RNF115 | Ring Finger Protein 115 | Protein Coding | 31 | GC01M145738 | 4.649508476 |
| SLC35A2 | Solute Carrier Family 35 Member A2 | Protein Coding | 37 | GC0XM048903 | 4.648900986 |
| NBEA | Neurobeachin | Protein Coding | 39 | GC13P034942 | 4.648389339 |
| UVRAG | UV Radiation Resistance Associated | Protein Coding | 37 | GC11P075815 | 4.647399426 |
| SLC5A1 | Solute Carrier Family 5 Member 1 | Protein Coding | 44 | GC22P032043 | 4.645051003 |
| HK1 | Hexokinase 1 | Protein Coding | 44 | GC10P069269 | 4.644629955 |
| POU4F1 | POU Class 4 Homeobox 1 | Protein Coding | 38 | GC13M078598 | 4.643471718 |
| SOX21-AS1 | SOX21 Antisense Divergent Transcript 1 | RNA Gene | 17 | GC13P094712 | 4.643250942 |
| UBB | Ubiquitin B | Protein Coding | 38 | GC17P016380 | 4.643172741 |
| SLC22A2 | Solute Carrier Family 22 Member 2 | Protein Coding | 40 | GC06M160187 | 4.642246246 |
| PMEL | Premelanosome Protein | Protein Coding | 36 | GC12M055954 | 4.641553402 |
| DHRS2 | Dehydrogenase/Reductase 2 | Protein Coding | 36 | GC14P031749 | 4.639630318 |
| CDK10 | Cyclin Dependent Kinase 10 | Protein Coding | 40 | GC16P089680 | 4.639058113 |
| PRTN3 | Proteinase 3 | Protein Coding | 41 | GC19P000840 | 4.638233662 |
| ATN1 | Atrophin 1 | Protein Coding | 40 | GC12P019852 | 4.636321068 |
| ECE1 | Endothelin Converting Enzyme 1 | Protein Coding | 44 | GC01M021217 | 4.634345055 |
| HPS3 | HPS3 Biogenesis Of Lysosomal Organelles Complex 2 Subunit 1 | Protein Coding | 36 | GC03P149129 | 4.633871555 |
| USP1 | Ubiquitin Specific Peptidase 1 | Protein Coding | 40 | GC01P062436 | 4.632422447 |
| MIR708 | MicroRNA 708 | RNA Gene | 18 | GC11M079402 | 4.626075745 |
| KCNMB2-AS1 | KCNMB2 Antisense RNA 1 | RNA Gene | 14 | GC03M178525 | 4.624890804 |
| DLGAP2 | DLG Associated Protein 2 | Protein Coding | 34 | GC08P000739 | 4.62153244 |
| E2F8 | E2F Transcription Factor 8 | Protein Coding | 32 | GC11M019203 | 4.620728493 |
| SYTL2 | Synaptotagmin Like 2 | Protein Coding | 34 | GC11M085694 | 4.619729996 |
| TOP3A | DNA Topoisomerase III Alpha | Protein Coding | 37 | GC17M025181 | 4.619315624 |
| CLCN1 | Chloride Voltage-Gated Channel 1 | Protein Coding | 40 | GC07P143316 | 4.617966652 |
| NCAPG | Non-SMC Condensin I Complex Subunit G | Protein Coding | 35 | GC04P017904 | 4.616565704 |
| AFF1 | AF4/FMR2 Family Member 1 | Protein Coding | 34 | GC04P086934 | 4.616409302 |
| MIR635 | MicroRNA 635 | RNA Gene | 14 | GC17M068424 | 4.615202904 |
| RPL6 | Ribosomal Protein L6 | Protein Coding | 35 | GC12M112320 | 4.614066124 |
| SELL | Selectin L | Protein Coding | 39 | GC01M169690 | 4.612757206 |
| ICOS | Inducible T Cell Costimulator | Protein Coding | 40 | GC02P203937 | 4.611907959 |
| MCM8 | Minichromosome Maintenance 8 Homologous Recombination Repair Factor | Protein Coding | 36 | GC20P006162 | 4.611613274 |
| FAT1 | FAT Atypical Cadherin 1 | Protein Coding | 36 | GC04M186587 | 4.610749722 |
| MALINC1 | Mitosis Associated Long Intergenic Non-Coding RNA 1 | RNA Gene | 14 | GC05M140073 | 4.610081196 |
| MAGEA6 | MAGE Family Member A6 | Protein Coding | 30 | GC0XM152766 | 4.610036373 |
| GTF2H4 | General Transcription Factor IIH Subunit 4 | Protein Coding | 36 | GC06P080335 | 4.609107494 |
| AQP3 | Aquaporin 3 (Gill Blood Group) | Protein Coding | 42 | GC09M033431 | 4.606965065 |
| GUCY1B2 | Guanylate Cyclase 1 Soluble Subunit Beta 2 (Pseudogene) | Pseudogene | 27 | GC13M050994 | 4.606926918 |
| KAT6A | Lysine Acetyltransferase 6A | Protein Coding | 39 | GC08M041929 | 4.606871605 |
| SMIM22 | Small Integral Membrane Protein 22 | Protein Coding | 21 | GC16P004788 | 4.605697632 |
| MUS81 | MUS81 Structure-Specific Endonuclease Subunit | Protein Coding | 38 | GC11P069569 | 4.60535717 |
| GAB2 | GRB2 Associated Binding Protein 2 | Protein Coding | 39 | GC11M078215 | 4.6011796 |
| LRRC26 | Leucine Rich Repeat Containing 26 | Protein Coding | 30 | GC09M137479 | 4.599226952 |
| TPI1 | Triosephosphate Isomerase 1 | Protein Coding | 42 | GC12P019844 | 4.59832859 |
| HID1 | HID1 Domain Containing | Protein Coding | 30 | GC17M074950 | 4.597505569 |
| ITGAX | Integrin Subunit Alpha X | Protein Coding | 40 | GC16P040315 | 4.596244812 |
| NPPA | Natriuretic Peptide A | Protein Coding | 41 | GC01M011846 | 4.596048832 |
| RNASE1 | Ribonuclease A Family Member 1, Pancreatic | Protein Coding | 38 | GC14M020801 | 4.59327507 |
| BRD7 | Bromodomain Containing 7 | Protein Coding | 38 | GC16M050313 | 4.592576504 |
| HSPA14 | Heat Shock Protein Family A (Hsp70) Member 14 | Protein Coding | 34 | GC10P014847 | 4.588801384 |
| THRA | Thyroid Hormone Receptor Alpha | Protein Coding | 43 | GC17P040058 | 4.588372231 |
| MGA | MAX Dimerization Protein MGA | Protein Coding | 34 | GC15P041621 | 4.586052895 |
| MGAT4B | Alpha-1,3-Mannosyl-Glycoprotein 4-Beta-N-Acetylglucosaminyltransferase B | Protein Coding | 36 | GC05M179797 | 4.582724571 |
| TNFRSF13C | TNF Receptor Superfamily Member 13C | Protein Coding | 39 | GC22M056119 | 4.582079411 |
| SLC17A5 | Solute Carrier Family 17 Member 5 | Protein Coding | 39 | GC06M073593 | 4.581259727 |
| DYSF | Dysferlin | Protein Coding | 39 | GC02P071453 | 4.578912735 |
| CSN1S1 | Casein Alpha S1 | Protein Coding | 32 | GC04P069932 | 4.578480721 |
| FER | FER Tyrosine Kinase | Protein Coding | 42 | GC05P108747 | 4.576915741 |
| DYNC2H1 | Dynein Cytoplasmic 2 Heavy Chain 1 | Protein Coding | 35 | GC11P103109 | 4.576655388 |
| FRS2 | Fibroblast Growth Factor Receptor Substrate 2 | Protein Coding | 36 | GC12P069471 | 4.576433182 |
| HIF1AN | Hypoxia Inducible Factor 1 Subunit Alpha Inhibitor | Protein Coding | 40 | GC10P100529 | 4.575809479 |
| RORA | RAR Related Orphan Receptor A | Protein Coding | 43 | GC15M060488 | 4.575572014 |
| NOS1 | Nitric Oxide Synthase 1 | Protein Coding | 44 | GC12M117208 | 4.574914455 |
| PTS | 6-Pyruvoyltetrahydropterin Synthase | Protein Coding | 42 | GC11P112226 | 4.573009968 |
| UTS2 | Urotensin 2 | Protein Coding | 36 | GC01M007843 | 4.57118988 |
| AOC4P | Amine Oxidase Copper Containing 4, Pseudogene | Pseudogene | 10 | GC17P042865 | 4.569450378 |
| PLAC8 | Placenta Associated 8 | Protein Coding | 32 | GC04M083090 | 4.568263054 |
| POLR2A | RNA Polymerase II Subunit A | Protein Coding | 40 | GC17P010879 | 4.568117142 |
| SIRT6 | Sirtuin 6 | Protein Coding | 40 | GC19M004174 | 4.567745209 |
| ABCB5 | ATP Binding Cassette Subfamily B Member 5 | Protein Coding | 39 | GC07P020615 | 4.567349911 |
| CITED2 | Cbp/P300 Interacting Transactivator With Glu/Asp Rich Carboxy-Terminal Domain 2 | Protein Coding | 39 | GC06M139371 | 4.56611681 |
| MIR1277 | MicroRNA 1277 | RNA Gene | 15 | GC0XP118386 | 4.563695908 |
| ZFHX4-AS1 | ZFHX4 Antisense RNA 1 | RNA Gene | 16 | GC08M076491 | 4.56302166 |
| AFF4 | AF4/FMR2 Family Member 4 | Protein Coding | 38 | GC05M132875 | 4.562683105 |
| PTPN14 | Protein Tyrosine Phosphatase Non-Receptor Type 14 | Protein Coding | 40 | GC01M214348 | 4.560811996 |
| NAP1L4 | Nucleosome Assembly Protein 1 Like 4 | Protein Coding | 34 | GC11M002944 | 4.560031891 |
| RFWD3 | Ring Finger And WD Repeat Domain 3 | Protein Coding | 35 | GC16M074621 | 4.559958935 |
| MYH1 | Myosin Heavy Chain 1 | Protein Coding | 36 | GC17M010492 | 4.558732986 |
| NEK10 | NIMA Related Kinase 10 | Protein Coding | 34 | GC03M027128 | 4.557228565 |
| NAGLU | N-Acetyl-Alpha-Glucosaminidase | Protein Coding | 38 | GC17P053164 | 4.555870056 |
| GLB1 | Galactosidase Beta 1 | Protein Coding | 44 | GC03M032963 | 4.554983139 |
| COL4A6 | Collagen Type IV Alpha 6 Chain | Protein Coding | 40 | GC0XM108155 | 4.554450035 |
| USP36 | Ubiquitin Specific Peptidase 36 | Protein Coding | 33 | GC17M078787 | 4.552510262 |
| RBM10 | RNA Binding Motif Protein 10 | Protein Coding | 36 | GC0XP047503 | 4.552311897 |
| SPTLC1 | Serine Palmitoyltransferase Long Chain Base Subunit 1 | Protein Coding | 42 | GC09M092703 | 4.55052948 |
| MIR551A | MicroRNA 551a | RNA Gene | 18 | GC01M003560 | 4.550189495 |
| CYFIP1 | Cytoplasmic FMR1 Interacting Protein 1 | Protein Coding | 36 | GC15M022867 | 4.549844742 |

| | | | | | |
|---|---|---|---|---|---|
| LAMP1 | Lysosomal Associated Membrane Protein 1 | Protein Coding | 38 | GC13P113297 | 4.549344063 |
| PEX14 | Peroxisomal Biogenesis Factor 14 | Protein Coding | 40 | GC01P010472 | 4.547152519 |
| LDHB | Lactate Dehydrogenase B | Protein Coding | 42 | GC12M021635 | 4.545855999 |
| CPS1 | Carbamoyl-Phosphate Synthase 1 | Protein Coding | 43 | GC02P210477 | 4.540133476 |
| EPS8 | Epidermal Growth Factor Receptor Pathway Substrate 8 | Protein Coding | 42 | GC12M020525 | 4.539878845 |
| SCG5 | Secretogranin V | Protein Coding | 34 | GC15P032641 | 4.539270401 |
| PRDM5 | PR/SET Domain 5 | Protein Coding | 35 | GC04M120686 | 4.537301064 |
| FBXO11 | F-Box Protein 11 | Protein Coding | 39 | GC02M047789 | 4.537164688 |
| ITGB2 | Integrin Subunit Beta 2 | Protein Coding | 46 | GC21M044885 | 4.537021637 |
| SCNN1B | Sodium Channel Epithelial 1 Subunit Beta | Protein Coding | 44 | GC16P023278 | 4.535329819 |
| FBP1 | Fructose-Bisphosphatase 1 | Protein Coding | 44 | GC09M094603 | 4.534539223 |
| PTPRN | Protein Tyrosine Phosphatase Receptor Type N | Protein Coding | 40 | GC02M219289 | 4.533844948 |
| MIR4713HG | MIR4713 Host Gene | RNA Gene | 13 | GC15P051039 | 4.533007622 |
| NPAS2 | Neuronal PAS Domain Protein 2 | Protein Coding | 36 | GC02P100820 | 4.531914711 |
| RALY | RALY Heterogeneous Nuclear Ribonucleoprotein | Protein Coding | 34 | GC20P033993 | 4.531001091 |
| PKD2 | Polycystin 2, Transient Receptor Potential Cation Channel | Protein Coding | 43 | GC04P088007 | 4.530516148 |
| SYCP1 | Synaptonemal Complex Protein 1 | Protein Coding | 32 | GC01P114854 | 4.530097961 |
| OR4K1 | Olfactory Receptor Family 4 Subfamily K Member 1 | Protein Coding | 28 | GC14P031431 | 4.529210091 |
| BRAP | BRCA1 Associated Protein | Protein Coding | 38 | GC12M111642 | 4.528957367 |
| MLXIPL | MLX Interacting Protein Like | Protein Coding | 38 | GC07M073593 | 4.528919697 |
| LDB1 | LIM Domain Binding 1 | Protein Coding | 35 | GC10M102106 | 4.528521061 |
| MIR1266 | MicroRNA 1266 | RNA Gene | 15 | GC15M082001 | 4.528286934 |
| TOP2B | DNA Topoisomerase II Beta | Protein Coding | 42 | GC03M025598 | 4.528051853 |
| GNAI2 | G Protein Subunit Alpha I2 | Protein Coding | 41 | GC03P050226 | 4.527936935 |
| KANSL1 | KAT8 Regulatory NSL Complex Subunit 1 | Protein Coding | 34 | GC17M046081 | 4.527774811 |
| STK11IP | Serine/Threonine Kinase 11 Interacting Protein | Protein Coding | 31 | GC02P219597 | 4.523080349 |
| LAMB2 | Laminin Subunit Beta 2 | Protein Coding | 42 | GC03M049121 | 4.522243023 |
| ACKR1 | Atypical Chemokine Receptor 1 (Duffy Blood Group) | Protein Coding | 32 | GC01P159203 | 4.520917416 |
| FTH1 | Ferritin Heavy Chain 1 | Protein Coding | 44 | GC11M061959 | 4.519809246 |
| ACCS | 1-Aminocyclopropane-1-Carboxylate Synthase Homolog (Inactive) | Protein Coding | 35 | GC11P044045 | 4.51915741 |
| LGI1 | Leucine Rich Glioma Inactivated 1 | Protein Coding | 39 | GC10P093757 | 4.519058228 |
| MCPH1 | Microcephalin 1 | Protein Coding | 36 | GC08P006406 | 4.519022465 |
| GABPB1-AS1 | GABPB1 Antisense RNA 1 | RNA Gene | 14 | GC15P050354 | 4.518292427 |
| CCL1 | C-C Motif Chemokine Ligand 1 | Protein Coding | 35 | GC17M034763 | 4.518260002 |
| BRF1 | BRF1 RNA Polymerase III Transcription Initiation Factor Subunit | Protein Coding | 36 | GC14M112395 | 4.518203735 |
| ANKLE1 | Ankyrin Repeat And LEM Domain Containing 1 | Protein Coding | 31 | GC19P063101 | 4.517230511 |
| CPE | Carboxypeptidase E | Protein Coding | 41 | GC04P165361 | 4.516043663 |
| AKR1B1 | Aldo-Keto Reductase Family 1 Member B | Protein Coding | 42 | GC07M134442 | 4.515057564 |
| SLC6A2 | Solute Carrier Family 6 Member 2 | Protein Coding | 43 | GC16P055656 | 4.514614105 |
| CHFR | Checkpoint With Forkhead And Ring Finger Domains | Protein Coding | 35 | GC12M132822 | 4.514076233 |
| MARK4 | Microtubule Affinity Regulating Kinase 4 | Protein Coding | 40 | GC19P045079 | 4.511196136 |
| ARHGAP27P1 | Rho GTPase Activating Protein 27 Pseudogene 1 | Pseudogene | 9 | GC17M065239 | 4.508464813 |
| CTNND2 | Catenin Delta 2 | Protein Coding | 38 | GC05M010971 | 4.508379459 |
| RRM2B | Ribonucleotide Reductase Regulatory TP53 Inducible Subunit M2B | Protein Coding | 44 | GC08M102204 | 4.507914543 |
| LINC00515 | Long Intergenic Non-Protein Coding RNA 515 | RNA Gene | 14 | GC21M025582 | 4.504232407 |
| COL5A1 | Collagen Type V Alpha 1 Chain | Protein Coding | 42 | GC09P134641 | 4.50340271 |
| TPT1 | Tumor Protein, Translationally-Controlled 1 | Protein Coding | 41 | GC13M045333 | 4.502604961 |
| SLAMF1 | Signaling Lymphocytic Activation Molecule Family Member 1 | Protein Coding | 36 | GC01M160608 | 4.50083828 |
| CAVIN1 | Caveolae Associated Protein 1 | Protein Coding | 32 | GC17M042404 | 4.499809265 |
| CCN3 | Cellular Communication Network Factor 3 | Protein Coding | 32 | GC08P119416 | 4.499509335 |
| EXOSC3 | Exosome Component 3 | Protein Coding | 38 | GC09M037772 | 4.498305321 |
| HOXA1 | Homeobox A1 | Protein Coding | 41 | GC07M027092 | 4.497234821 |
| LINC00342 | Long Intergenic Non-Protein Coding RNA 342 | RNA Gene | 17 | GC02M095807 | 4.496660233 |
| LOC113664106 | CFTR Intron 2 DNase I Hypersensitive Site | Biological Region | 3 | GC07P117508 | 4.496538162 |
| CD58 | CD58 Molecule | Protein Coding | 36 | GC01M116514 | 4.494840622 |
| LINC00226 | Long Intergenic Non-Protein Coding RNA 226 | RNA Gene | 13 | GC14P106287 | 4.49425602 |
| MGAT5 | Alpha-1,6-Mannosylglycoprotein 6-Beta-N-Acetylglucosaminyltransferase | Protein Coding | 36 | GC02P134119 | 4.494035721 |
| BCLAF1 | BCL2 Associated Transcription Factor 1 | Protein Coding | 36 | GC06M136256 | 4.493853092 |
| POU2F1 | POU Class 2 Homeobox 1 | Protein Coding | 39 | GC01P167190 | 4.492649078 |
| RFC4 | Replication Factor C Subunit 4 | Protein Coding | 36 | GC03M186789 | 4.491952896 |
| PGC | Progastricsin | Protein Coding | 39 | GC06M041736 | 4.49040699 |
| POLI | DNA Polymerase Iota | Protein Coding | 38 | GC18P054274 | 4.490274429 |
| DLST | Dihydrolipoamide S-Succinyltransferase | Protein Coding | 41 | GC14P074881 | 4.488838196 |
| MIR138-2 | MicroRNA 138-2 | RNA Gene | 20 | GC16P057024 | 4.487437248 |
| NDN | Necdin, MAGE Family Member | Protein Coding | 38 | GC15M023701 | 4.486269951 |
| CRABP1 | Cellular Retinoic Acid Binding Protein 1 | Protein Coding | 37 | GC15P078340 | 4.484910011 |
| ZNF558 | Zinc Finger Protein 558 | Protein Coding | 31 | GC19M008806 | 4.484779835 |
| C2orf69 | Chromosome 2 Open Reading Frame 69 | Protein Coding | 30 | GC02P199911 | 4.484779835 |
| ENSG00000266919 | Hsa-Mir-423 | Uncategorized | 7 | GC17M034618 | 4.481867313 |
| MIR518B | MicroRNA 518b | RNA Gene | 18 | GC19P064302 | 4.481539249 |
| HDLBP | High Density Lipoprotein Binding Protein | Protein Coding | 39 | GC02M241227 | 4.480726242 |
| SNORD29 | Small Nucleolar RNA, C/D Box 29 | RNA Gene | 14 | GC11M062853 | 4.476639271 |
| TSKS | Testis Specific Serine Kinase Substrate | Protein Coding | 29 | GC19M049739 | 4.475223064 |
| SCD | Stearoyl-CoA Desaturase | Protein Coding | 44 | GC10P100347 | 4.474855423 |
| FAAH | Fatty Acid Amide Hydrolase | Protein Coding | 43 | GC01P046394 | 4.4739151 |
| SOX7 | SRY-Box Transcription Factor 7 | Protein Coding | 33 | GC08M010723 | 4.472898483 |
| TNNI3 | Troponin I3, Cardiac Type | Protein Coding | 44 | GC19M055151 | 4.471852779 |
| HDAC7 | Histone Deacetylase 7 | Protein Coding | 40 | GC12M047782 | 4.470994949 |
| HORMAD1 | HORMA Domain Containing 1 | Protein Coding | 32 | GC01M151647 | 4.469778538 |
| PFKFB3 | 6-Phosphofructo-2-Kinase/Fructose-2,6-Biphosphatase 3 | Protein Coding | 40 | GC10P006144 | 4.468929291 |
| ASL | Argininosuccinate Lyase | Protein Coding | 40 | GC07P066075 | 4.466559887 |
| SPOCK1 | SPARC (Osteonectin), Cwcv And Kazal Like Domains Proteoglycan 1 | Protein Coding | 35 | GC05M136975 | 4.466014385 |
| CSNK2A3 | Casein Kinase 2 Alpha 3 | Protein Coding | 24 | GC11M011351 | 4.465011597 |
| YTHDF1 | YTH N6-Methyladenosine RNA Binding Protein 1 | Protein Coding | 33 | GC20M063195 | 4.463563919 |
| TNRC6B | Trinucleotide Repeat Containing Adaptor 6B | Protein Coding | 34 | GC22P040044 | 4.463438988 |
| LINC01186 | Long Intergenic Non-Protein Coding RNA 1186 | RNA Gene | 14 | GC0XM046258 | 4.461325645 |
| HELZ | Helicase With Zinc Finger | Protein Coding | 32 | GC17M067070 | 4.46054554 |
| SKI | SKI Proto-Oncogene | Protein Coding | 40 | GC01P002228 | 4.458461761 |
| AQP5 | Aquaporin 5 | Protein Coding | 42 | GC12P049961 | 4.45820713 |
| XAGE2 | X Antigen Family Member 2 | Protein Coding | 24 | GC0XP052368 | 4.45745039 |
| STAP2 | Signal Transducing Adaptor Family Member 2 | Protein Coding | 35 | GC19M004324 | 4.455488205 |
| RASAL2 | RAS Protein Activator Like 2 | Protein Coding | 34 | GC01P178093 | 4.455084801 |
| LGR6 | Leucine Rich Repeat Containing G Protein-Coupled Receptor 6 | Protein Coding | 37 | GC01P202193 | 4.45488596 |
| IL2RG | Interleukin 2 Receptor Subunit Gamma | Protein Coding | 42 | GC0XM071108 | 4.454727173 |
| FOXR2 | Forkhead Box R2 | Protein Coding | 28 | GC0XP055623 | 4.451059818 |

| | | | | | |
|---|---|---|---|---|---|
| LINC01088 | Long Intergenic Non-Protein Coding RNA 1088 | RNA Gene | 15 | GC04P078971 | 4.450767994 |
| SMN1 | Survival Of Motor Neuron 1, Telomeric | Protein Coding | 38 | GC05P070924 | 4.450183868 |
| IER3 | Immediate Early Response 3 | Protein Coding | 36 | GC06M030743 | 4.449716091 |
| BTG3-AS1 | BTG3 Antisense RNA 1 | RNA Gene | 11 | GC21P017612 | 4.448703766 |
| EPHB1 | EPH Receptor B1 | Protein Coding | 42 | GC03P134795 | 4.448033333 |
| FCRL5 | Fc Receptor Like 5 | Protein Coding | 35 | GC01M157514 | 4.446436882 |
| KARS1 | Lysyl-TRNA Synthetase 1 | Protein Coding | 36 | GC16M075734 | 4.444832802 |
| MIR506 | MicroRNA 506 | RNA Gene | 15 | GC0XM147230 | 4.444279194 |
| NEXN-AS1 | NEXN Antisense RNA 1 | RNA Gene | 19 | GC01M077962 | 4.443935394 |
| ARF1 | ADP Ribosylation Factor 1 | Protein Coding | 41 | GC01P228082 | 4.443484306 |
| IL1RL1 | Interleukin 1 Receptor Like 1 | Protein Coding | 37 | GC02P102294 | 4.442670345 |
| LINC00572 | Long Intergenic Non-Protein Coding RNA 572 | RNA Gene | 14 | GC13M029918 | 4.441756725 |
| PEX6 | Peroxisomal Biogenesis Factor 6 | Protein Coding | 39 | GC06M042963 | 4.4410882 |
| COL10A1 | Collagen Type X Alpha 1 Chain | Protein Coding | 38 | GC06M116118 | 4.44079113 |
| CDKL5 | Cyclin Dependent Kinase Like 5 | Protein Coding | 39 | GC0XP018425 | 4.439026356 |
| PSIP1 | PC4 And SFRS1 Interacting Protein 1 | Protein Coding | 35 | GC09M015464 | 4.43705368 |
| FKBP4 | FKBP Prolyl Isomerase 4 | Protein Coding | 40 | GC12P002795 | 4.435834885 |
| TNKS2 | Tankyrase 2 | Protein Coding | 40 | GC10P091798 | 4.434664726 |
| OR4N2 | Olfactory Receptor Family 4 Subfamily N Member 2 | Protein Coding | 29 | GC14P031414 | 4.434108734 |
| OR4K2 | Olfactory Receptor Family 4 Subfamily K Member 2 | Protein Coding | 28 | GC14P031429 | 4.434108734 |
| OR4M1 | Olfactory Receptor Family 4 Subfamily M Member 1 | Protein Coding | 28 | GC14P031424 | 4.434108734 |
| OR4C11 | Olfactory Receptor Family 4 Subfamily C Member 11 | Protein Coding | 27 | GC11M086934 | 4.434108734 |
| OR4K5 | Olfactory Receptor Family 4 Subfamily K Member 5 | Protein Coding | 27 | GC14P019920 | 4.434108734 |
| OR4P4 | Olfactory Receptor Family 4 Subfamily P Member 4 | Protein Coding | 27 | GC11P055638 | 4.434108734 |
| OR11H12 | Olfactory Receptor Family 11 Subfamily H Member 12 | Protein Coding | 22 | GC14P018601 | 4.434108734 |
| PEPD | Peptidase D | Protein Coding | 41 | GC19M033386 | 4.430402756 |
| TCF20 | Transcription Factor 20 | Protein Coding | 34 | GC22M042160 | 4.430334091 |
| MAP1LC3B | Microtubule Associated Protein 1 Light Chain 3 Beta | Protein Coding | 36 | GC16P087384 | 4.430068493 |
| IVL | Involucrin | Protein Coding | 34 | GC01P152881 | 4.430005074 |
| PLXNB1 | Plexin B1 | Protein Coding | 39 | GC03M048403 | 4.429927349 |
| SFPQ | Splicing Factor Proline And Glutamine Rich | Protein Coding | 39 | GC01M035176 | 4.42903614 |
| BST2 | Bone Marrow Stromal Cell Antigen 2 | Protein Coding | 35 | GC19M017403 | 4.428919792 |
| ALPL | Alkaline Phosphatase, Biomineralization Associated | Protein Coding | 46 | GC01P021508 | 4.428832054 |
| MIR611 | MicroRNA 611 | RNA Gene | 14 | GC11M061792 | 4.428229332 |
| JMJD1C | Jumonji Domain Containing 1C | Protein Coding | 36 | GC10M063167 | 4.426996231 |
| H2AC20 | H2A Clustered Histone 20 | Protein Coding | 30 | GC01P150140 | 4.425744534 |
| GAS6-AS1 | GAS6 Antisense RNA 1 | RNA Gene | 17 | GC13P113815 | 4.425549507 |
| UHRF2 | Ubiquitin Like With PHD And Ring Finger Domains 2 | Protein Coding | 35 | GC09P006413 | 4.423418045 |
| CD248 | CD248 Molecule | Protein Coding | 34 | GC11M066314 | 4.422734737 |
| RTL1 | Retrotransposon Gag Like 1 | Protein Coding | 28 | GC14M101010 | 4.42257452 |
| TP53I3 | Tumor Protein P53 Inducible Protein 3 | Protein Coding | 37 | GC02M024078 | 4.420356274 |
| SLC39A2 | Solute Carrier Family 39 Member 2 | Protein Coding | 33 | GC14P031554 | 4.419486523 |
| LOC730101 | Uncharacterized LOC730101 | RNA Gene | 13 | GC06P052664 | 4.415875912 |
| FRAT1 | FRAT Regulator Of WNT Signaling Pathway 1 | Protein Coding | 35 | GC10P097319 | 4.415691376 |
| FGF22 | Fibroblast Growth Factor 22 | Protein Coding | 34 | GC19P000639 | 4.41296196 |
| MIR367 | MicroRNA 367 | RNA Gene | 19 | GC04M112647 | 4.412693501 |
| MAP2 | Microtubule Associated Protein 2 | Protein Coding | 39 | GC02P209424 | 4.411623001 |
| MRPS30-DT | MRPS30 Divergent Transcript | RNA Gene | 11 | GC05M044748 | 4.410746098 |
| SOST | Sclerostin | Protein Coding | 41 | GC17M043753 | 4.410650253 |
| GUCA2B | Guanylate Cyclase Activator 2B | Protein Coding | 35 | GC01P042153 | 4.406968117 |
| ZBTB20 | Zinc Finger And BTB Domain Containing 20 | Protein Coding | 38 | GC03M114315 | 4.40423584 |
| TRPM2 | Transient Receptor Potential Cation Channel Subfamily M Member 2 | Protein Coding | 38 | GC21P044350 | 4.403552055 |
| MTMR3 | Myotubularin Related Protein 3 | Protein Coding | 38 | GC22P034954 | 4.403481007 |
| MIR100HG | Mir-100-Let-7a-2-Mir-125b-1 Cluster Host Gene | RNA Gene | 17 | GC11M122029 | 4.40312767 |
| ERAP1 | Endoplasmic Reticulum Aminopeptidase 1 | Protein Coding | 41 | GC05M096760 | 4.402664185 |
| PTPN2 | Protein Tyrosine Phosphatase Non-Receptor Type 2 | Protein Coding | 42 | GC18M024017 | 4.401945114 |
| TM9SF4 | Transmembrane 9 Superfamily Member 4 | Protein Coding | 32 | GC20P032109 | 4.401642799 |
| ADRA1A | Adrenoceptor Alpha 1A | Protein Coding | 42 | GC08M026747 | 4.400559425 |
| SLC26A4 | Solute Carrier Family 26 Member 4 | Protein Coding | 39 | GC07P107660 | 4.398381233 |
| GBE1 | 1,4-Alpha-Glucan Branching Enzyme 1 | Protein Coding | 39 | GC03M081489 | 4.397000313 |
| DMPK | DM1 Protein Kinase | Protein Coding | 44 | GC19M045769 | 4.395711899 |
| NCSTN | Nicastrin | Protein Coding | 42 | GC01P160343 | 4.394264698 |
| EVC2 | EvC Ciliary Complex Subunit 2 | Protein Coding | 36 | GC04M005534 | 4.38962841 |
| GSTO2 | Glutathione S-Transferase Omega 2 | Protein Coding | 38 | GC10P104268 | 4.389025688 |
| CRK | CRK Proto-Oncogene, Adaptor Protein | Protein Coding | 39 | GC17M001420 | 4.388972759 |
| SLC9A1 | Solute Carrier Family 9 Member A1 | Protein Coding | 46 | GC01M027109 | 4.387946129 |
| RHBDF1 | Rhomboid 5 Homolog 1 | Protein Coding | 35 | GC16M000058 | 4.384637356 |
| MBTPS2 | Membrane Bound Transcription Factor Peptidase, Site 2 | Protein Coding | 38 | GC0XP021839 | 4.384480476 |
| ADAMTSL3 | ADAMTS Like 3 | Protein Coding | 32 | GC15P115049 | 4.383868217 |
| PHF20 | PHD Finger Protein 20 | Protein Coding | 35 | GC20P035771 | 4.382205486 |
| FAM168A | Family With Sequence Similarity 168 Member A | Protein Coding | 29 | GC11M073400 | 4.381998062 |
| PRDM16 | PR/SET Domain 16 | Protein Coding | 41 | GC01P003682 | 4.381787777 |
| NR4A3 | Nuclear Receptor Subfamily 4 Group A Member 3 | Protein Coding | 40 | GC09P099821 | 4.380569458 |
| MT1DP | Metallothionein 1D, Pseudogene | Pseudogene | 19 | GC16P056643 | 4.379275322 |
| RUBCNL | Rubicon Like Autophagy Enhancer | Protein Coding | 25 | GC13M046343 | 4.378725529 |
| MIR648 | MicroRNA 648 | RNA Gene | 15 | GC22M017980 | 4.37760973 |
| XAGE3 | X Antigen Family Member 3 | Protein Coding | 26 | GC0XM053137 | 4.377390385 |
| MAP1B | Microtubule Associated Protein 1B | Protein Coding | 40 | GC05P072107 | 4.377236843 |
| FARSB | Phenylalanyl-TRNA Synthetase Subunit Beta | Protein Coding | 38 | GC02M222570 | 4.377090931 |
| BDP1 | B Double Prime 1, Subunit Of RNA Polymerase III Transcription Initiation Factor IIIB | Protein Coding | 32 | GC05P072679 | 4.374209404 |
| SIPA1 | Signal-Induced Proliferation-Associated 1 | Protein Coding | 36 | GC11P065638 | 4.373135567 |
| POU5F1P4 | POU Class 5 Homeobox 1 Pseudogene 4 | Pseudogene | 14 | GC01P155444 | 4.372747898 |
| CACNA1H | Calcium Voltage-Gated Channel Subunit Alpha1 H | Protein Coding | 45 | GC16P001153 | 4.371819496 |
| ENTPD3-AS1 | ENTPD3 Antisense RNA 1 | RNA Gene | 15 | GC03M040313 | 4.369262218 |
| MC4R | Melanocortin 4 Receptor | Protein Coding | 43 | GC18M060371 | 4.368077755 |
| MTA2 | Metastasis Associated 1 Family Member 2 | Protein Coding | 35 | GC11M087125 | 4.36789608 |
| ABCC5 | ATP Binding Cassette Subfamily C Member 5 | Protein Coding | 39 | GC03M183919 | 4.367612839 |
| TRMT2A | TRNA Methyltransferase 2 Homolog A | Protein Coding | 35 | GC22M020099 | 4.366019249 |
| DNASE1L3 | Deoxyribonuclease 1 Like 3 | Protein Coding | 39 | GC03M058192 | 4.365953445 |
| PTGDS | Prostaglandin D2 Synthase | Protein Coding | 40 | GC09P137078 | 4.365562439 |
| LETM1 | Leucine Zipper And EF-Hand Containing Transmembrane Protein 1 | Protein Coding | 39 | GC04M001811 | 4.363320351 |
| POU2F3 | POU Class 2 Homeobox 3 | Protein Coding | 34 | GC11P120236 | 4.362416744 |
| DACH1 | Dachshund Family Transcription Factor 1 | Protein Coding | 36 | GC13M071437 | 4.361360073 |
| CAMKK2 | Calcium/Calmodulin Dependent Protein Kinase Kinase 2 | Protein Coding | 42 | GC12M122145 | 4.360860348 |

| | | | | | |
|---|---|---|---|---|---|
| TSPAN32 | Tetraspanin 32 | Protein Coding | 32 | GC11P002302 | 4.360433578 |
| PTCPRN | Papillary Thyroid Carcinoma With Papillary Renal Neoplasia | Genetic Locus | 2 | GC01U990360 | 4.360066414 |
| DLGAP5 | DLG Associated Protein 5 | Protein Coding | 35 | GC14M055148 | 4.360001087 |
| TCONS_00068220 | Uncharacterized TCONS_00068220 | RNA Gene | 4 | GC08M047990 | 4.358281612 |
| PIRC66 | Piwi-Interacting RNA Cluster 66 | RNA Gene | 6 | GC15U901108 | 4.357717991 |
| FABP5 | Fatty Acid Binding Protein 5 | Protein Coding | 37 | GC08P081282 | 4.357359886 |
| CCDC47 | Coiled-Coil Domain Containing 47 | Protein Coding | 31 | GC17M063745 | 4.356526375 |
| RLN2 | Relaxin 2 | Protein Coding | 35 | GC09M005328 | 4.354628563 |
| CHGB | Chromogranin B | Protein Coding | 38 | GC20P005911 | 4.354605675 |
| SNRNP200 | Small Nuclear Ribonucleoprotein U5 Subunit 200 | Protein Coding | 38 | GC02M098015 | 4.354426384 |
| RBP3 | Retinol Binding Protein 3 | Protein Coding | 38 | GC10P047348 | 4.353867054 |
| G3BP2 | G3BP Stress Granule Assembly Factor 2 | Protein Coding | 32 | GC04M075642 | 4.353408813 |
| PCSK7 | Proprotein Convertase Subtilisin/Kexin Type 7 | Protein Coding | 40 | GC11M117199 | 4.35299015 |
| FOLR2 | Folate Receptor Beta | Protein Coding | 38 | GC11P072216 | 4.350926399 |
| GCG | Glucagon | Protein Coding | 37 | GC02M162142 | 4.350604534 |
| ADAM8 | ADAM Metallopeptidase Domain 8 | Protein Coding | 39 | GC10M133262 | 4.350434303 |
| CCL8 | C-C Motif Chemokine Ligand 8 | Protein Coding | 36 | GC17P034319 | 4.348034859 |
| MMP16 | Matrix Metallopeptidase 16 | Protein Coding | 40 | GC08M088032 | 4.34800005 |
| WNT8A | Wnt Family Member 8A | Protein Coding | 37 | GC05P138139 | 4.346977711 |
| CACNA1G-AS1 | CACNA1G Antisense RNA 1 | RNA Gene | 15 | GC17M050798 | 4.346609592 |
| SLC4A1AP | Solute Carrier Family 4 Member 1 Adaptor Protein | Protein Coding | 31 | GC02P027663 | 4.346199512 |
| HEATR3 | HEAT Repeat Containing 3 | Protein Coding | 28 | GC16P050065 | 4.345308304 |
| CDO1 | Cysteine Dioxygenase Type 1 | Protein Coding | 36 | GC05M115804 | 4.344943523 |
| SP3 | Sp3 Transcription Factor | Protein Coding | 38 | GC02M173882 | 4.344560623 |
| SLC3A2 | Solute Carrier Family 3 Member 2 | Protein Coding | 36 | GC11P062856 | 4.34119463 |
| GGH | Gamma-Glutamyl Hydrolase | Protein Coding | 39 | GC08M063014 | 4.339680672 |
| MIR1915HG | MIR1915 Host Gene | RNA Gene | 22 | GC10M021494 | 4.338839054 |
| PTPRD-AS1 | PTPRD Antisense RNA 1 | RNA Gene | 13 | GC09P008860 | 4.338748932 |
| AHI1 | Abelson Helper Integration Site 1 | Protein Coding | 37 | GC06M135283 | 4.337758064 |
| LRAT | Lecithin Retinol Acyltransferase | Protein Coding | 40 | GC04P154626 | 4.336770535 |
| PHGDH | Phosphoglycerate Dehydrogenase | Protein Coding | 45 | GC01P119660 | 4.335507393 |
| EEF2K | Eukaryotic Elongation Factor 2 Kinase | Protein Coding | 40 | GC16P022217 | 4.330526352 |
| MUC20-OT1 | MUC20 Overlapping Transcript | RNA Gene | 12 | GC03P196209 | 4.329164505 |
| MIR500A | MicroRNA 500a | RNA Gene | 16 | GC0XP050008 | 4.329065323 |
| RPRD1B | Regulation Of Nuclear Pre-MRNA Domain Containing 1B | Protein Coding | 34 | GC20P038033 | 4.327167988 |
| LRRC15 | Leucine Rich Repeat Containing 15 | Protein Coding | 34 | GC03M194357 | 4.325246811 |
| CASZ1 | Castor Zinc Finger 1 | Protein Coding | 35 | GC01M010642 | 4.325098038 |
| SURF1 | SURF1 Cytochrome C Oxidase Assembly Factor | Protein Coding | 38 | GC09M133351 | 4.32382822 |
| KIAA0087 | KIAA0087 LncRNA | RNA Gene | 23 | GC07M026541 | 4.322426796 |
| KHDRBS1 | KH RNA Binding Domain Containing, Signal Transduction Associated 1 | Protein Coding | 38 | GC01P032013 | 4.321162701 |
| KIF2A | Kinesin Family Member 2A | Protein Coding | 37 | GC05P062306 | 4.320973396 |
| SMARCD1 | SWI/SNF Related, Matrix Associated, Actin Dependent Regulator Of Chromatin, Subfamily D, Member 1 | Protein Coding | 36 | GC12P050085 | 4.320477486 |
| TJP2 | Tight Junction Protein 2 | Protein Coding | 40 | GC09P069121 | 4.319459915 |
| DSC3 | Desmocollin 3 | Protein Coding | 39 | GC18M030990 | 4.318715572 |
| LINC00538 | Long Intergenic Non-Protein Coding RNA 538 | RNA Gene | 13 | GC01P213924 | 4.315013885 |
| ITIH4 | Inter-Alpha-Trypsin Inhibitor Heavy Chain 4 | Protein Coding | 39 | GC03M052812 | 4.314855099 |
| RIF1 | Replication Timing Regulatory Factor 1 | Protein Coding | 35 | GC02P151409 | 4.311799049 |
| PRDX6 | Peroxiredoxin 6 | Protein Coding | 41 | GC01P173477 | 4.311691284 |
| MIR518D | MicroRNA 518d | RNA Gene | 14 | GC19P053734 | 4.311334133 |
| UNC13D | Unc-13 Homolog D | Protein Coding | 40 | GC17M075827 | 4.309484005 |
| ARFGAP3 | ADP Ribosylation Factor GTPase Activating Protein 3 | Protein Coding | 36 | GC22M042796 | 4.30747509 |
| CFP | Complement Factor Properdin | Protein Coding | 38 | GC0XM047624 | 4.305701733 |
| MIR509-1 | MicroRNA 509-1 | RNA Gene | 15 | GC0XM147260 | 4.302872658 |
| DEF6 | DEF6 Guanine Nucleotide Exchange Factor | Protein Coding | 37 | GC06P080507 | 4.301290512 |
| KRT18P55 | Keratin 18 Pseudogene 55 | Pseudogene | 14 | GC17M028275 | 4.3005023 |
| CTAGE1 | Cutaneous T Cell Lymphoma-Associated Antigen 1 | Protein Coding | 30 | GC18M022413 | 4.297649384 |
| ARHGEF4 | Rho Guanine Nucleotide Exchange Factor 4 | Protein Coding | 36 | GC02P130836 | 4.296771049 |
| AIRE | Autoimmune Regulator | Protein Coding | 40 | GC21P044285 | 4.296139717 |
| SYNE2 | Spectrin Repeat Containing Nuclear Envelope Protein 2 | Protein Coding | 35 | GC14P063761 | 4.295174122 |
| FXR1 | FMR1 Autosomal Homolog 1 | Protein Coding | 39 | GC03P180868 | 4.292912483 |
| BRD2 | Bromodomain Containing 2 | Protein Coding | 40 | GC06P080394 | 4.289632797 |
| HOXB7 | Homeobox B7 | Protein Coding | 36 | GC17M048607 | 4.288677216 |
| LMO7 | LIM Domain 7 | Protein Coding | 36 | GC13P075620 | 4.288184643 |
| HLA-E | Major Histocompatibility Complex, Class I, E | Protein Coding | 38 | GC06P080326 | 4.286489487 |
| NECTIN3 | Nectin Cell Adhesion Molecule 3 | Protein Coding | 31 | GC03P111071 | 4.285817623 |
| TXNRD2 | Thioredoxin Reductase 2 | Protein Coding | 42 | GC22M019863 | 4.282025814 |
| APTX | Aprataxin | Protein Coding | 40 | GC09M032886 | 4.280114651 |
| APOC3 | Apolipoprotein C3 | Protein Coding | 40 | GC11P116829 | 4.276741028 |
| HNMT | Histamine N-Methyltransferase | Protein Coding | 40 | GC02P137964 | 4.27622509 |
| SMARCC1 | SWI/SNF Related, Matrix Associated, Actin Dependent Regulator Of Chromatin Subfamily C Member 1 | Protein Coding | 38 | GC03M047585 | 4.275799274 |
| PARD3 | Par-3 Family Cell Polarity Regulator | Protein Coding | 37 | GC10M034110 | 4.273835182 |
| RELN | Reelin | Protein Coding | 39 | GC07M103471 | 4.273472786 |
| TLR8 | Toll Like Receptor 8 | Protein Coding | 43 | GC0XP012924 | 4.272042274 |
| HOXD13 | Homeobox D13 | Protein Coding | 38 | GC02P176092 | 4.270992279 |
| NSF | N-Ethylmaleimide Sensitive Factor, Vesicle Fusing ATPase | Protein Coding | 40 | GC17P046590 | 4.270265579 |
| SIAH2 | Siah E3 Ubiquitin Protein Ligase 2 | Protein Coding | 39 | GC03M150741 | 4.267257214 |
| SCG2 | Secretogranin II | Protein Coding | 36 | GC02M223596 | 4.265972614 |
| DNAH9 | Dynein Axonemal Heavy Chain 9 | Protein Coding | 34 | GC17P011598 | 4.265444756 |
| MSX2 | Msh Homeobox 2 | Protein Coding | 43 | GC05P174724 | 4.264174461 |
| A2ML1 | Alpha-2-Macroglobulin Like 1 | Protein Coding | 36 | GC12P008822 | 4.262491226 |
| NMT1 | N-Myristoyltransferase 1 | Protein Coding | 39 | GC17P053256 | 4.262188435 |
| IGHMBP2 | Immunoglobulin Mu DNA Binding Protein 2 | Protein Coding | 38 | GC11P068903 | 4.261266708 |
| ARHGEF12 | Rho Guanine Nucleotide Exchange Factor 12 | Protein Coding | 38 | GC11P120336 | 4.259442806 |
| PLCD4 | Phospholipase C Delta 4 | Protein Coding | 38 | GC02P218608 | 4.258229733 |
| ORAI3 | ORAI Calcium Release-Activated Calcium Modulator 3 | Protein Coding | 32 | GC16P030949 | 4.257041931 |
| CCDC88C | Coiled-Coil Domain Containing 88C | Protein Coding | 35 | GC14M091271 | 4.256968975 |
| IRX2 | Iroquois Homeobox 2 | Protein Coding | 36 | GC05M002708 | 4.256453991 |
| MAGEA12 | MAGE Family Member A12 | Protein Coding | 31 | GC0XP152733 | 4.254245758 |
| LMX1B | LIM Homeobox Transcription Factor 1 Beta | Protein Coding | 40 | GC09P126614 | 4.25373745 |
| SIX4 | SIX Homeobox 4 | Protein Coding | 34 | GC14M060709 | 4.253568649 |
| CYP26A1 | Cytochrome P450 Family 26 Subfamily A Member 1 | Protein Coding | 40 | GC10P093073 | 4.253367901 |
| CCDC88A | Coiled-Coil Domain Containing 88A | Protein Coding | 37 | GC02M055287 | 4.252408504 |
| CD7 | CD7 Molecule | Protein Coding | 35 | GC17M082314 | 4.24777317 |

| | | | | | |
|---|---|---|---|---|---|
| PRKCH | Protein Kinase C Eta | Protein Coding | 45 | GC14P061187 | 4.247361183 |
| OGN | Osteoglycin | Protein Coding | 36 | GC09M092383 | 4.246966839 |
| MIR187 | MicroRNA 187 | RNA Gene | 18 | GC18M035904 | 4.246274471 |
| SLC4A2 | Solute Carrier Family 4 Member 2 | Protein Coding | 38 | GC07P151057 | 4.246032715 |
| SMIM31 | Small Integral Membrane Protein 31 | Protein Coding | 14 | GC04P164755 | 4.245587826 |
| DUSP19 | Dual Specificity Phosphatase 19 | Protein Coding | 35 | GC02P183078 | 4.243890762 |
| EFNB2 | Ephrin B2 | Protein Coding | 39 | GC13M106489 | 4.243625641 |
| MPG | N-Methylpurine DNA Glycosylase | Protein Coding | 36 | GC16P010616 | 4.242256641 |
| PPP1R10 | Protein Phosphatase 1 Regulatory Subunit 10 | Protein Coding | 35 | GC06M030600 | 4.240792751 |
| PUM1 | Pumilio RNA Binding Family Member 1 | Protein Coding | 38 | GC01M030931 | 4.240661621 |
| CDC14A | Cell Division Cycle 14A | Protein Coding | 41 | GC01P100351 | 4.239466667 |
| PRPS1 | Phosphoribosyl Pyrophosphate Synthetase 1 | Protein Coding | 42 | GC0XP107628 | 4.238482952 |
| NOXA1 | NADPH Oxidase Activator 1 | Protein Coding | 33 | GC09P137423 | 4.237823009 |
| UBE2N | Ubiquitin Conjugating Enzyme E2 N | Protein Coding | 42 | GC12M093406 | 4.237548828 |
| USF1 | Upstream Transcription Factor 1 | Protein Coding | 39 | GC01M161039 | 4.236567497 |
| LINC01554 | Long Intergenic Non-Protein Coding RNA 1554 | RNA Gene | 18 | GC05P095838 | 4.235049248 |
| GNL3 | G Protein Nucleolar 3 | Protein Coding | 38 | GC03P052681 | 4.232950687 |
| WFS1 | Wolframin ER Transmembrane Glycoprotein | Protein Coding | 42 | GC04P006260 | 4.231777668 |
| MAP3K20-AS1 | MAP3K20 Antisense RNA 1 | RNA Gene | 15 | GC02M173168 | 4.231735706 |
| NEO1 | Neogenin 1 | Protein Coding | 38 | GC15P073051 | 4.231467247 |
| IL17F | Interleukin 17F | Protein Coding | 40 | GC06M063970 | 4.23139143 |
| CX3CL1 | C-X3-C Motif Chemokine Ligand 1 | Protein Coding | 38 | GC16P057372 | 4.229631424 |
| CDC23 | Cell Division Cycle 23 | Protein Coding | 35 | GC05M138198 | 4.227393627 |
| B3GAT1 | Beta-1,3-Glucuronyltransferase 1 | Protein Coding | 39 | GC11M134378 | 4.226070881 |
| BCL2A1 | BCL2 Related Protein A1 | Protein Coding | 38 | GC15M081651 | 4.224312305 |
| ADGRG1 | Adhesion G Protein-Coupled Receptor G1 | Protein Coding | 35 | GC16P057610 | 4.223615646 |
| OGT | O-Linked N-Acetylglucosamine (GlcNAc) Transferase | Protein Coding | 39 | GC0XP071534 | 4.220766068 |
| MRPS26 | Mitochondrial Ribosomal Protein S26 | Protein Coding | 31 | GC20P003977 | 4.218698502 |
| KRR1 | KRR1 Small Subunit Processome Component Homolog | Protein Coding | 35 | GC12M075490 | 4.218494415 |
| CANX | Calnexin | Protein Coding | 39 | GC05P179678 | 4.216996193 |
| GRHL2 | Grainyhead Like Transcription Factor 2 | Protein Coding | 36 | GC08P101492 | 4.216346264 |
| ERAS | ES Cell Expressed Ras | Protein Coding | 32 | GC0XP050520 | 4.213690758 |
| LINC02412 | Long Intergenic Non-Protein Coding RNA 2412 | RNA Gene | 12 | GC12P093175 | 4.212704659 |
| LINC01502 | Long Intergenic Non-Protein Coding RNA 1502 | RNA Gene | 11 | GC09P135574 | 4.212704659 |
| PLAC1 | Placenta Enriched 1 | Protein Coding | 31 | GC0XM134565 | 4.208942413 |
| RBM5-AS1 | RBM5 Antisense RNA 1 | RNA Gene | 11 | GC03M050099 | 4.206269741 |
| CKAP2L | Cytoskeleton Associated Protein 2 Like | Protein Coding | 34 | GC02M112736 | 4.205992699 |
| XPO5 | Exportin 5 | Protein Coding | 36 | GC06M043522 | 4.204208374 |
| BLMH | Bleomycin Hydrolase | Protein Coding | 40 | GC17M030248 | 4.204037666 |
| MIR302B | MicroRNA 302b | RNA Gene | 19 | GC04M112764 | 4.202913761 |
| DPM1 | Dolichyl-Phosphate Mannosyltransferase Subunit 1, Catalytic | Protein Coding | 39 | GC20M050934 | 4.202261925 |
| IL1RAPL2 | Interleukin 1 Receptor Accessory Protein Like 2 | Protein Coding | 35 | GC0XP104566 | 4.20218277 |
| ASAP1 | ArfGAP With SH3 Domain, Ankyrin Repeat And PH Domain 1 | Protein Coding | 35 | GC08M130052 | 4.201786518 |
| CDC37 | Cell Division Cycle 37, HSP90 Cochaperone | Protein Coding | 36 | GC19M010391 | 4.199923992 |
| SLC46A1 | Solute Carrier Family 46 Member 1 | Protein Coding | 38 | GC17M034572 | 4.196863651 |
| ERP29 | Endoplasmic Reticulum Protein 29 | Protein Coding | 32 | GC12P112013 | 4.195755959 |
| RAB11FIP3 | RAB11 Family Interacting Protein 3 | Protein Coding | 31 | GC16P000425 | 4.195459366 |
| LINC02042 | Long Intergenic Non-Protein Coding RNA 2042 | RNA Gene | 11 | GC03M112737 | 4.19508791 |
| NR2E3 | Nuclear Receptor Subfamily 2 Group E Member 3 | Protein Coding | 38 | GC15P071792 | 4.192863464 |
| SFXN4 | Sideroflexin 4 | Protein Coding | 36 | GC10M119140 | 4.191169739 |
| ABCD1 | ATP Binding Cassette Subfamily D Member 1 | Protein Coding | 42 | GC0XP153724 | 4.190807343 |
| GSTZ1 | Glutathione S-Transferase Zeta 1 | Protein Coding | 37 | GC14P077320 | 4.188868523 |
| RPN1 | Ribophorin I | Protein Coding | 38 | GC03M128619 | 4.186928272 |
| SPATS2L | Spermatogenesis Associated Serine Rich 2 Like | Protein Coding | 31 | GC02P200305 | 4.186305046 |
| RHOH | Ras Homolog Family Member H | Protein Coding | 39 | GC04P040192 | 4.186180115 |
| GNA13 | G Protein Subunit Alpha 13 | Protein Coding | 39 | GC17M065009 | 4.185972214 |
| EYA4 | EYA Transcriptional Coactivator And Phosphatase 4 | Protein Coding | 40 | GC06P133240 | 4.184826374 |
| RALGDS | Ral Guanine Nucleotide Dissociation Stimulator | Protein Coding | 37 | GC09M133097 | 4.184491158 |
| MYEOV | Myeloma Overexpressed | Protein Coding | 30 | GC11P069836 | 4.182517052 |
| DDX1 | DEAD-Box Helicase 1 | Protein Coding | 39 | GC02P015591 | 4.181800842 |
| SOAT1 | Sterol O-Acyltransferase 1 | Protein Coding | 40 | GC01P179262 | 4.181380272 |
| RGS22 | Regulator Of G Protein Signaling 22 | Protein Coding | 31 | GC08M099960 | 4.180348396 |
| ANLN | Anillin Actin Binding Protein | Protein Coding | 38 | GC07P036389 | 4.180116653 |
| PPBP | Pro-Platelet Basic Protein | Protein Coding | 38 | GC04M073986 | 4.176712036 |
| TREX1 | Three Prime Repair Exonuclease 1 | Protein Coding | 37 | GC03P048883 | 4.176371574 |
| RPS16 | Ribosomal Protein S16 | Protein Coding | 35 | GC19M039433 | 4.175898552 |
| CSN3 | Casein Kappa | Protein Coding | 32 | GC04P070242 | 4.175731659 |
| IL21 | Interleukin 21 | Protein Coding | 39 | GC04M122612 | 4.175648212 |
| STEAP1 | STEAP Family Member 1 | Protein Coding | 35 | GC07P090154 | 4.17550087 |
| PATZ1 | POZ/BTB And AT Hook Containing Zinc Finger 1 | Protein Coding | 34 | GC22M031325 | 4.174526691 |
| H2BC21 | H2B Clustered Histone 21 | Protein Coding | 31 | GC01M151590 | 4.173885345 |
| A2M-AS1 | A2M Antisense RNA 1 | RNA Gene | 14 | GC12P009065 | 4.173641205 |
| TAP2 | Transporter 2, ATP Binding Cassette Subfamily B Member | Protein Coding | 40 | GC06M032821 | 4.172838211 |
| PAK6 | P21 (RAC1) Activated Kinase 6 | Protein Coding | 40 | GC15P040217 | 4.170770168 |
| CYSLTR2 | Cysteinyl Leukotriene Receptor 2 | Protein Coding | 43 | GC13P048653 | 4.169526577 |
| BHLHE40 | Basic Helix-Loop-Helix Family Member E40 | Protein Coding | 36 | GC03P004980 | 4.169312477 |
| ATP12A | ATPase H+/K+ Transporting Non-Gastric Alpha2 Subunit | Protein Coding | 39 | GC13P024680 | 4.165371895 |
| RPS6KA1 | Ribosomal Protein S6 Kinase A1 | Protein Coding | 45 | GC01P026540 | 4.165226936 |
| MAP3K13 | Mitogen-Activated Protein Kinase Kinase Kinase 13 | Protein Coding | 38 | GC03P185282 | 4.165019512 |
| PRKAB1 | Protein Kinase AMP-Activated Non-Catalytic Subunit Beta 1 | Protein Coding | 42 | GC12P119632 | 4.163445473 |
| SND1 | Staphylococcal Nuclease And Tudor Domain Containing 1 | Protein Coding | 36 | GC07P127652 | 4.161226749 |
| SNRPN | Small Nuclear Ribonucleoprotein Polypeptide N | Protein Coding | 39 | GC15P024823 | 4.156731606 |
| TBX4 | T-Box Transcription Factor 4 | Protein Coding | 38 | GC17P061451 | 4.156300068 |
| DCAF12 | DDB1 And CUL4 Associated Factor 12 | Protein Coding | 28 | GC09M034079 | 4.155488014 |
| NFATC3 | Nuclear Factor Of Activated T Cells 3 | Protein Coding | 38 | GC16P068119 | 4.155071259 |
| PSMC5 | Proteasome 26S Subunit, ATPase 5 | Protein Coding | 37 | GC17P063827 | 4.153312683 |
| FAM13A | Family With Sequence Similarity 13 Member A | Protein Coding | 34 | GC04M088725 | 4.152030468 |
| PDS5B | PDS5 Cohesin Associated Factor B | Protein Coding | 32 | GC13P032586 | 4.151445389 |
| ADH6 | Alcohol Dehydrogenase 6 (Class V) | Protein Coding | 36 | GC04M099202 | 4.15048027 |
| ADAMTS15 | ADAM Metallopeptidase With Thrombospondin Type 1 Motif 15 | Protein Coding | 32 | GC11P130448 | 4.148327827 |
| MAPK15 | Mitogen-Activated Protein Kinase 15 | Protein Coding | 35 | GC08P143716 | 4.146993637 |
| PHKB | Phosphorylase Kinase Regulatory Subunit Beta | Protein Coding | 40 | GC16P047436 | 4.14529705 |
| FIG4 | FIG4 Phosphoinositide 5-Phosphatase | Protein Coding | 40 | GC06P109691 | 4.144273758 |
| AURKC | Aurora Kinase C | Protein Coding | 42 | GC19P057230 | 4.143784523 |
| SPRR3 | Small Proline Rich Protein 3 | Protein Coding | 34 | GC01P153001 | 4.143491268 |

| | | | | | |
|---|---|---|---|---|---|
| MUSK | Muscle Associated Receptor Tyrosine Kinase | Protein Coding | 43 | GC09P110668 | 4.142944813 |
| EMD | Emerin | Protein Coding | 39 | GC0XP154379 | 4.142045975 |
| AFG3L2 | AFG3 Like Matrix AAA Peptidase Subunit 2 | Protein Coding | 41 | GC18M012328 | 4.141182899 |
| NFYC | Nuclear Transcription Factor Y Subunit Gamma | Protein Coding | 38 | GC01P040691 | 4.140730381 |
| SAMHD1 | SAM And HD Domain Containing Deoxynucleoside Triphosphate Triphosphohydrolase 1 | Protein Coding | 38 | GC20M036890 | 4.140453815 |
| C1QBP | Complement C1q Binding Protein | Protein Coding | 40 | GC17M005432 | 4.139705181 |
| PYGO2 | Pygopus Family PHD Finger 2 | Protein Coding | 34 | GC01M154957 | 4.13898325 |
| FAM189B | Family With Sequence Similarity 189 Member B | Protein Coding | 31 | GC01M155248 | 4.138729095 |
| VTI1A | Vesicle Transport Through Interaction With T-SNAREs 1A | Protein Coding | 34 | GC10P112446 | 4.138672352 |
| CARTPT | CART Prepropeptide | Protein Coding | 39 | GC05P071719 | 4.138132095 |
| NECTIN2 | Nectin Cell Adhesion Molecule 2 | Protein Coding | 35 | GC19P063842 | 4.136051178 |
| TBXAS1 | Thromboxane A Synthase 1 | Protein Coding | 45 | GC07P139777 | 4.135910988 |
| IL22 | Interleukin 22 | Protein Coding | 37 | GC12M068248 | 4.135401249 |
| SLC25A11 | Solute Carrier Family 25 Member 11 | Protein Coding | 39 | GC17M004937 | 4.134762764 |
| ACTA2-AS1 | ACTA2 Antisense RNA 1 | RNA Gene | 15 | GC10P088932 | 4.133190632 |
| CSH1 | Chorionic Somatomammotropin Hormone 1 | Protein Coding | 33 | GC17M063894 | 4.133078575 |
| SLC7A7 | Solute Carrier Family 7 Member 7 | Protein Coding | 42 | GC14M022773 | 4.132673264 |
| LEPQTL1 | Leptin, Serum Levels Of | Genetic Locus | 3 | GC02U903086 | 4.13243103 |
| ITPR1 | Inositol 1,4,5-Trisphosphate Receptor Type 1 | Protein Coding | 44 | GC03P004486 | 4.130491734 |
| CNBP | CCHC-Type Zinc Finger Nucleic Acid Binding Protein | Protein Coding | 38 | GC03M129167 | 4.128355026 |
| LOC110386951 | CYP19A1 Promoter II/1.3 | Biological Region | 2 | GC15P051244 | 4.127236843 |
| EIF2S2 | Eukaryotic Translation Initiation Factor 2 Subunit Beta | Protein Coding | 36 | GC20M034088 | 4.126436234 |
| PNMT | Phenylethanolamine N-Methyltransferase | Protein Coding | 39 | GC17P039667 | 4.125861168 |
| SOD3 | Superoxide Dismutase 3 | Protein Coding | 38 | GC04P024798 | 4.125331402 |
| UBE2V2 | Ubiquitin Conjugating Enzyme E2 V2 | Protein Coding | 38 | GC08P047998 | 4.123114586 |
| PPFIBP2 | PPFIA Binding Protein 2 | Protein Coding | 33 | GC11P007491 | 4.122914791 |
| PRPF3 | Pre-MRNA Processing Factor 3 | Protein Coding | 38 | GC01P150321 | 4.122676849 |
| FABP7 | Fatty Acid Binding Protein 7 | Protein Coding | 38 | GC06P122801 | 4.121336937 |
| ATP2B3 | ATPase Plasma Membrane Ca2+ Transporting 3 | Protein Coding | 43 | GC0XP153517 | 4.121290207 |
| MIR7-2 | MicroRNA 7-2 | RNA Gene | 16 | GC15P088611 | 4.119423389 |
| KDM5C | Lysine Demethylase 5C | Protein Coding | 40 | GC0XM053176 | 4.118971348 |
| SEPTIN1 | Septin 1 | Protein Coding | 28 | GC16M030378 | 4.118518829 |
| MIR421 | MicroRNA 421 | RNA Gene | 14 | GC0XM074218 | 4.118499756 |
| SRMS | Src-Related Kinase Lacking C-Terminal Regulatory Tyrosine And N-Terminal Myristylation Sites | Protein Coding | 35 | GC20M063539 | 4.117922306 |
| MCRS1 | Microspherule Protein 1 | Protein Coding | 33 | GC12M049564 | 4.116975784 |
| LONP1 | Lon Peptidase 1, Mitochondrial | Protein Coding | 41 | GC19M005691 | 4.113887787 |
| PTGIS | Prostaglandin I2 Synthase | Protein Coding | 42 | GC20M049503 | 4.113022327 |
| LMNB1 | Lamin B1 | Protein Coding | 42 | GC05P126776 | 4.112622738 |
| REV1 | REV1 DNA Directed Polymerase | Protein Coding | 37 | GC02M099383 | 4.111888409 |
| USP44 | Ubiquitin Specific Peptidase 44 | Protein Coding | 34 | GC12M095516 | 4.111444473 |
| CLDN10 | Claudin 10 | Protein Coding | 38 | GC13P095433 | 4.108140945 |
| HTRA1 | HtrA Serine Peptidase 1 | Protein Coding | 41 | GC10P122461 | 4.107728958 |
| CYP27A1 | Cytochrome P450 Family 27 Subfamily A Member 1 | Protein Coding | 43 | GC02P218781 | 4.106764317 |
| MAT2A | Methionine Adenosyltransferase 2A | Protein Coding | 41 | GC02P085782 | 4.10631752 |
| MYBPC3 | Myosin Binding Protein C3 | Protein Coding | 42 | GC11M086883 | 4.106078148 |
| ACO2 | Aconitase 2 | Protein Coding | 42 | GC22P041517 | 4.106035233 |
| FAF1 | Fas Associated Factor 1 | Protein Coding | 38 | GC01M050439 | 4.105652332 |
| BDKRB1 | Bradykinin Receptor B1 | Protein Coding | 40 | GC14P096293 | 4.105494499 |
| PRKAR2A | Protein Kinase CAMP-Dependent Type II Regulatory Subunit Alpha | Protein Coding | 40 | GC03M048744 | 4.105337143 |
| SCARB1 | Scavenger Receptor Class B Member 1 | Protein Coding | 42 | GC12M124776 | 4.105135441 |
| MEIS1 | Meis Homeobox 1 | Protein Coding | 38 | GC02P066433 | 4.105051994 |
| TEX101 | Testis Expressed 101 | Protein Coding | 32 | GC19P043388 | 4.104988098 |
| TRPM7 | Transient Receptor Potential Cation Channel Subfamily M Member 7 | Protein Coding | 41 | GC15M050552 | 4.104286194 |
| MT-ATP8 | Mitochondrially Encoded ATP Synthase Membrane Subunit 8 | Protein Coding | 27 | GCMTP008368 | 4.103758812 |
| NKD2 | NKD Inhibitor Of WNT Signaling Pathway 2 | Protein Coding | 32 | GC05P001008 | 4.100056648 |
| DRD3 | Dopamine Receptor D3 | Protein Coding | 42 | GC03M114128 | 4.099563122 |
| BRD9 | Bromodomain Containing 9 | Protein Coding | 35 | GC05M001100 | 4.098862648 |
| HAS2 | Hyaluronan Synthase 2 | Protein Coding | 37 | GC08M121594 | 4.098751068 |
| GPC5 | Glypican 5 | Protein Coding | 37 | GC13P091398 | 4.098303795 |
| DSG2 | Desmoglein 2 | Protein Coding | 41 | GC18P031498 | 4.097864628 |
| YWHAQ | Tyrosine 3-Monooxygenase/Tryptophan 5-Monooxygenase Activation Protein Theta | Protein Coding | 41 | GC02M009583 | 4.097799778 |
| PCNA-AS1 | PCNA Antisense RNA 1 | RNA Gene | 11 | GC20P005119 | 4.097441196 |
| ARHGEF1 | Rho Guanine Nucleotide Exchange Factor 1 | Protein Coding | 40 | GC19P041883 | 4.097297192 |
| WNK2 | WNK Lysine Deficient Protein Kinase 2 | Protein Coding | 36 | GC09P093184 | 4.09692812 |
| TECTA | Tectorin Alpha | Protein Coding | 35 | GC11P121101 | 4.095631599 |
| SUCLG2-DT | SUCLG2 Divergent Transcript | RNA Gene | 13 | GC03P067656 | 4.095439911 |
| MLX | MAX Dimerization Protein MLX | Protein Coding | 39 | GC17P042567 | 4.093567848 |
| APOL1 | Apolipoprotein L1 | Protein Coding | 40 | GC22P036253 | 4.092833519 |
| TBL1X | Transducin Beta Like 1 X-Linked | Protein Coding | 33 | GC0XP009463 | 4.0922966 |
| MIR512-1 | MicroRNA 512-1 | RNA Gene | 15 | GC19P064296 | 4.091264248 |
| FKBP8 | FKBP Prolyl Isomerase 8 | Protein Coding | 38 | GC19M018503 | 4.090961456 |
| LGMN | Legumain | Protein Coding | 38 | GC14M092703 | 4.090830803 |
| SFTPB | Surfactant Protein B | Protein Coding | 40 | GC02M085657 | 4.090639114 |
| TSC22D1 | TSC22 Domain Family Member 1 | Protein Coding | 36 | GC13M044432 | 4.090274811 |
| PAK5 | P21 (RAC1) Activated Kinase 5 | Protein Coding | 35 | GC20M009538 | 4.090118408 |
| BCORL1 | BCL6 Corepressor Like 1 | Protein Coding | 31 | GC0XP129980 | 4.089178562 |
| EXTL1 | Exostosin Like Glycosyltransferase 1 | Protein Coding | 35 | GC01P026032 | 4.089050293 |
| ALDH1A3 | Aldehyde Dehydrogenase 1 Family Member A3 | Protein Coding | 40 | GC15P100877 | 4.089030743 |
| ARHGAP29 | Rho GTPase Activating Protein 29 | Protein Coding | 35 | GC01M094148 | 4.088847637 |
| PRKAR1B | Protein Kinase CAMP-Dependent Type I Regulatory Subunit Beta | Protein Coding | 42 | GC07M000549 | 4.086387634 |
| BIK | BCL2 Interacting Killer | Protein Coding | 37 | GC22P043110 | 4.086057663 |
| PROCR | Protein C Receptor | Protein Coding | 38 | GC20P035357 | 4.085571289 |
| LAG3 | Lymphocyte Activating 3 | Protein Coding | 38 | GC12P019840 | 4.084394932 |
| SCAPER | S-Phase Cyclin A Associated Protein In The ER | Protein Coding | 32 | GC15M076347 | 4.084298134 |
| CD69 | CD69 Molecule | Protein Coding | 37 | GC12M020434 | 4.084197044 |
| DUSP4 | Dual Specificity Phosphatase 4 | Protein Coding | 38 | GC08M029334 | 4.083530426 |
| LINC01628 | Long Intergenic Non-Protein Coding RNA 1628 | RNA Gene | 12 | GC02M066923 | 4.083377361 |
| LINC01627 | Long Intergenic Non-Protein Coding RNA 1627 | RNA Gene | 11 | GC09M037427 | 4.083377361 |
| G3BP1 | G3BP Stress Granule Assembly Factor 1 | Protein Coding | 36 | GC05P151771 | 4.08244133 |
| ATE1 | Arginyltransferase 1 | Protein Coding | 36 | GC10M121740 | 4.081994057 |
| CETP | Cholesteryl Ester Transfer Protein | Protein Coding | 43 | GC16P056961 | 4.080750942 |
| GLUD1 | Glutamate Dehydrogenase 1 | Protein Coding | 45 | GC10M087050 | 4.078803062 |

| | | | | | |
|---|---|---|---|---|---|
| MX1 | MX Dynamin Like GTPase 1 | Protein Coding | 37 | GC21P041420 | 4.077541351 |
| SMPD1 | Sphingomyelin Phosphodiesterase 1 | Protein Coding | 44 | GC11P006390 | 4.077293873 |
| PTX3 | Pentraxin 3 | Protein Coding | 38 | GC03P157436 | 4.077262878 |
| SNHG17 | Small Nucleolar RNA Host Gene 17 | RNA Gene | 18 | GC20M038641 | 4.07707119 |
| IGHM | Immunoglobulin Heavy Constant Mu | Protein Coding | 31 | GC14M112416 | 4.076916695 |
| FUT8 | Fucosyltransferase 8 | Protein Coding | 39 | GC14P065411 | 4.07691288 |
| LGR4 | Leucine Rich Repeat Containing G Protein-Coupled Receptor 4 | Protein Coding | 36 | GC11M027365 | 4.075367451 |
| DNAJA3 | DnaJ Heat Shock Protein Family (Hsp40) Member A3 | Protein Coding | 35 | GC16P004425 | 4.073366165 |
| CAV2 | Caveolin 2 | Protein Coding | 38 | GC07P116287 | 4.073225975 |
| TMEM33 | Transmembrane Protein 33 | Protein Coding | 30 | GC04P041937 | 4.072274208 |
| AQP4 | Aquaporin 4 | Protein Coding | 42 | GC18M026852 | 4.07192421 |
| MIR2861 | MicroRNA 2861 | RNA Gene | 15 | GC09P127785 | 4.071665287 |
| ADIPOR2 | Adiponectin Receptor 2 | Protein Coding | 38 | GC12P001670 | 4.070676804 |
| SGK2 | Serum/Glucocorticoid Regulated Kinase 2 | Protein Coding | 38 | GC20P043558 | 4.069895267 |
| TARDBP | TAR DNA Binding Protein | Protein Coding | 42 | GC01P011013 | 4.069756508 |
| ZGPAT | Zinc Finger CCCH-Type And G-Patch Domain Containing | Protein Coding | 32 | GC20P063707 | 4.067498207 |
| ATIC | 5-Aminoimidazole-4-Carboxamide Ribonucleotide Formyltransferase/IMP Cyclohydrolase | Protein Coding | 41 | GC02P215311 | 4.067199707 |
| SPG7 | SPG7 Matrix AAA Peptidase Subunit, Paraplegin | Protein Coding | 40 | GC16P091167 | 4.065610409 |
| NCEH1 | Neutral Cholesterol Ester Hydrolase 1 | Protein Coding | 34 | GC03M172630 | 4.065406799 |
| GATA6-AS1 | GATA6 Antisense RNA 1 (Head To Head) | RNA Gene | 15 | GC18M024070 | 4.063619137 |
| GJB6 | Gap Junction Protein Beta 6 | Protein Coding | 39 | GC13M020221 | 4.059633255 |
| GALNT3 | Polypeptide N-Acetylgalactosaminyltransferase 3 | Protein Coding | 41 | GC02M165747 | 4.059534073 |
| TRIM32 | Tripartite Motif Containing 32 | Protein Coding | 39 | GC09P116687 | 4.058861256 |
| TEAD1 | TEA Domain Transcription Factor 1 | Protein Coding | 43 | GC11P012674 | 4.058188438 |
| CALCR | Calcitonin Receptor | Protein Coding | 43 | GC07M093424 | 4.056875706 |
| ASXL2 | ASXL Transcriptional Regulator 2 | Protein Coding | 34 | GC02M025733 | 4.055488586 |
| SLC52A3 | Solute Carrier Family 52 Member 3 | Protein Coding | 35 | GC20M000741 | 4.054026604 |
| SPATS2 | Spermatogenesis Associated Serine Rich 2 | Protein Coding | 30 | GC12P049366 | 4.051918507 |
| PIWIL2 | Piwi Like RNA-Mediated Gene Silencing 2 | Protein Coding | 33 | GC08P022275 | 4.05105114 |
| SPAST | Spastin | Protein Coding | 36 | GC02P032063 | 4.049517155 |
| EIF4A1 | Eukaryotic Translation Initiation Factor 4A1 | Protein Coding | 40 | GC17P007572 | 4.048872948 |
| PRG2 | Proteoglycan 2, Pro Eosinophil Major Basic Protein | Protein Coding | 36 | GC11M057386 | 4.048458576 |
| AFDN-DT | AFDN Divergent Transcript | RNA Gene | 17 | GC06M167829 | 4.047347546 |
| SRP54 | Signal Recognition Particle 54 | Protein Coding | 38 | GC14P034981 | 4.04600811 |
| UBE2S | Ubiquitin Conjugating Enzyme E2 S | Protein Coding | 36 | GC19M055399 | 4.044826508 |
| NOX4 | NADPH Oxidase 4 | Protein Coding | 40 | GC11M089324 | 4.044404984 |
| RAP1GAP | RAP1 GTPase Activating Protein | Protein Coding | 35 | GC01M021596 | 4.044206619 |
| SIRT5 | Sirtuin 5 | Protein Coding | 40 | GC06P013574 | 4.043362141 |
| RBCK1 | RANBP2-Type And C3HC4-Type Zinc Finger Containing 1 | Protein Coding | 38 | GC20P000407 | 4.040596962 |
| ARHGDIA | Rho GDP Dissociation Inhibitor Alpha | Protein Coding | 42 | GC17M081867 | 4.040059566 |
| TENM1 | Teneurin Transmembrane Protein 1 | Protein Coding | 32 | GC0XM124375 | 4.039726257 |
| MIR874 | MicroRNA 874 | RNA Gene | 18 | GC05M137647 | 4.039021015 |
| ACAT1 | Acetyl-CoA Acetyltransferase 1 | Protein Coding | 44 | GC11P108121 | 4.03845787 |
| PLCD1 | Phospholipase C Delta 1 | Protein Coding | 43 | GC03M038008 | 4.0380373 |
| PTGES | Prostaglandin E Synthase | Protein Coding | 36 | GC09M129738 | 4.037332535 |
| MTSS1 | MTSS I-BAR Domain Containing 1 | Protein Coding | 35 | GC08M124550 | 4.036094666 |
| AKAP6 | A-Kinase Anchoring Protein 6 | Protein Coding | 36 | GC14P032203 | 4.035153389 |
| MYH7 | Myosin Heavy Chain 7 | Protein Coding | 43 | GC14M023412 | 4.034749985 |
| TCIRG1 | T Cell Immune Regulator 1, ATPase H+ Transporting V0 Subunit A3 | Protein Coding | 40 | GC11P069769 | 4.03471756 |
| ARFGEF2 | ADP Ribosylation Factor Guanine Nucleotide Exchange Factor 2 | Protein Coding | 37 | GC20P048921 | 4.033797264 |
| DDX6 | DEAD-Box Helicase 6 | Protein Coding | 41 | GC11M118748 | 4.032862663 |
| BTG2 | BTG Anti-Proliferation Factor 2 | Protein Coding | 36 | GC01P203305 | 4.032626152 |
| SEMA5B | Semaphorin 5B | Protein Coding | 35 | GC03M122909 | 4.028122902 |
| PANTR1 | POU3F3 Adjacent Non-Coding Transcript 1 | RNA Gene | 14 | GC02M104806 | 4.027390957 |
| AFDN | Afadin, Adherens Junction Formation Factor | Protein Coding | 34 | GC06P167827 | 4.027236938 |
| ATG7 | Autophagy Related 7 | Protein Coding | 38 | GC03P012269 | 4.026332378 |
| NECTIN1 | Nectin Cell Adhesion Molecule 1 | Protein Coding | 37 | GC11M119624 | 4.026024818 |
| CDH10 | Cadherin 10 | Protein Coding | 37 | GC05M024522 | 4.026003838 |
| IFNGR2 | Interferon Gamma Receptor 2 | Protein Coding | 42 | GC21P033402 | 4.023947716 |
| NUCB2 | Nucleobindin 2 | Protein Coding | 35 | GC11P017272 | 4.023636818 |
| GRIN2D | Glutamate Ionotropic Receptor NMDA Type Subunit 2D | Protein Coding | 44 | GC19P063999 | 4.023249149 |
| PF4 | Platelet Factor 4 | Protein Coding | 36 | GC04M073980 | 4.021129131 |
| EIF2B2 | Eukaryotic Translation Initiation Factor 2B Subunit Beta | Protein Coding | 40 | GC14P075002 | 4.018043995 |
| APRG1 | APRG1 Tumor Suppressor Candidate | RNA Gene | 24 | GC03P037380 | 4.017160416 |
| ZNF277 | Zinc Finger Protein 277 | Protein Coding | 32 | GC07P112206 | 4.017150879 |
| RCC2 | Regulator Of Chromosome Condensation 2 | Protein Coding | 35 | GC01M017406 | 4.015742302 |
| RAD23A | RAD23 Homolog A, Nucleotide Excision Repair Protein | Protein Coding | 38 | GC19P013695 | 4.014527321 |
| SCN9A | Sodium Voltage-Gated Channel Alpha Subunit 9 | Protein Coding | 44 | GC02M166195 | 4.014017105 |
| SLC39A7 | Solute Carrier Family 39 Member 7 | Protein Coding | 36 | GC06P033200 | 4.013510704 |
| H2BC14 | H2B Clustered Histone 14 | Protein Coding | 27 | GC06P079941 | 4.012731075 |
| HHAT | Hedgehog Acyltransferase | Protein Coding | 38 | GC01P210328 | 4.012440681 |
| FBN2 | Fibrillin 2 | Protein Coding | 38 | GC05M128257 | 4.010702133 |
| VPS9D1-AS1 | VPS9D1 Antisense RNA 1 | RNA Gene | 14 | GC16P089711 | 4.010433674 |
| RPGR | Retinitis Pigmentosa GTPase Regulator | Protein Coding | 37 | GC0XM038269 | 4.010428905 |
| ZKSCAN3 | Zinc Finger With KRAB And SCAN Domains 3 | Protein Coding | 30 | GC06P028349 | 4.010388851 |
| OVGP1 | Oviductal Glycoprotein 1 | Protein Coding | 36 | GC01M111414 | 4.008738995 |
| PTBP2 | Polypyrimidine Tract Binding Protein 2 | Protein Coding | 36 | GC01P096721 | 4.008319855 |
| THOC1 | THO Complex 1 | Protein Coding | 32 | GC18M000204 | 4.007541656 |
| PRDM16-DT | PRDM16 Divergent Transcript | RNA Gene | 18 | GC01M005607 | 4.007293224 |
| BIN2 | Bridging Integrator 2 | Protein Coding | 32 | GC12M051281 | 4.006613255 |
| RPE65 | Retinoid Isomerohydrolase RPE65 | Protein Coding | 40 | GC01M068428 | 4.004887581 |
| IATPR | ITGB1 Adjacent Tumor Promoting LncRNA | RNA Gene | 11 | GC10M033067 | 4.003216743 |
| MTHFD1L | Methylenetetrahydrofolate Dehydrogenase (NADP+ Dependent) 1 Like | Protein Coding | 37 | GC06P150865 | 4.002025604 |
| ITCH | Itchy E3 Ubiquitin Protein Ligase | Protein Coding | 40 | GC20P034363 | 4.000163078 |
| CALM1 | Calmodulin 1 | Protein Coding | 40 | GC14P090396 | 3.997432709 |
| ZNF750 | Zinc Finger Protein 750 | Protein Coding | 32 | GC17M082829 | 3.996330738 |
| KALRN | Kalirin RhoGEF Kinase | Protein Coding | 38 | GC03P124033 | 3.996290684 |
| HJV | Hemojuvelin BMP Co-Receptor | Protein Coding | 35 | GC01M146721 | 3.99560976 |
| CLMP | CXADR Like Membrane Protein | Protein Coding | 36 | GC11M123069 | 3.994155169 |
| PDHA1 | Pyruvate Dehydrogenase E1 Subunit Alpha 1 | Protein Coding | 43 | GC0XP019343 | 3.992987156 |
| SBNO1 | Strawberry Notch Homolog 1 | Protein Coding | 32 | GC12M123289 | 3.992622852 |
| ADCY8 | Adenylate Cyclase 8 | Protein Coding | 39 | GC08M130780 | 3.99240756 |
| DPF2 | Double PHD Fingers 2 | Protein Coding | 37 | GC11P069536 | 3.991561413 |
| ATF5 | Activating Transcription Factor 5 | Protein Coding | 35 | GC19P064099 | 3.990247726 |

| | | | | | |
|---|---|---|---|---|---|
| SRSF3 | Serine And Arginine Rich Splicing Factor 3 | Protein Coding | 34 | GC06P080531 | 3.989665747 |
| EIF4A3 | Eukaryotic Translation Initiation Factor 4A3 | Protein Coding | 38 | GC17M080135 | 3.98833847 |
| SMG7 | SMG7 Nonsense Mediated MRNA Decay Factor | Protein Coding | 34 | GC01P183441 | 3.988073826 |
| ALG9 | ALG9 Alpha-1,2-Mannosyltransferase | Protein Coding | 37 | GC11M112392 | 3.987345219 |
| PEX19 | Peroxisomal Biogenesis Factor 19 | Protein Coding | 38 | GC01M160276 | 3.986799955 |
| CCL17 | C-C Motif Chemokine Ligand 17 | Protein Coding | 37 | GC16P057413 | 3.986789942 |
| H3C11 | H3 Clustered Histone 11 | Protein Coding | 26 | GC06M064160 | 3.984537601 |
| ZAR1L | Zygote Arrest 1 Like | Protein Coding | 23 | GC13M036303 | 3.983208895 |
| FOXO4 | Forkhead Box O4 | Protein Coding | 36 | GC0XP071095 | 3.982793331 |
| EXOC3 | Exocyst Complex Component 3 | Protein Coding | 32 | GC05P000443 | 3.979955196 |
| LAS1L | LAS1 Like Ribosome Biogenesis Factor | Protein Coding | 32 | GC0XM065440 | 3.979007959 |
| ALKBH1 | AlkB Homolog 1, Histone H2A Dioxygenase | Protein Coding | 31 | GC14M077672 | 3.975214243 |
| ADCYAP1 | Adenylate Cyclase Activating Polypeptide 1 | Protein Coding | 38 | GC18P000895 | 3.974437237 |
| HCN1 | Hyperpolarization Activated Cyclic Nucleotide Gated Potassium Channel 1 | Protein Coding | 42 | GC05M045260 | 3.973052502 |
| NFIX | Nuclear Factor I X | Protein Coding | 39 | GC19P013690 | 3.972843647 |
| CSPG4 | Chondroitin Sulfate Proteoglycan 4 | Protein Coding | 41 | GC15M075674 | 3.972415209 |
| ADGRE5 | Adhesion G Protein-Coupled Receptor E5 | Protein Coding | 35 | GC19P014381 | 3.970708847 |
| DENR | Density Regulated Re-Initiation And Release Factor | Protein Coding | 33 | GC12P122752 | 3.969975948 |
| ELL | Elongation Factor For RNA Polymerase II | Protein Coding | 35 | GC19M018443 | 3.968274593 |
| ZNF496-DT | ZNF496 Divergent Transcript | RNA Gene | 9 | GC01P247357 | 3.967894793 |
| SRF | Serum Response Factor | Protein Coding | 37 | GC06P043171 | 3.967765331 |
| GABRP | Gamma-Aminobutyric Acid Type A Receptor Subunit Pi | Protein Coding | 38 | GC05P170763 | 3.966088295 |
| SOCS2 | Suppressor Of Cytokine Signaling 2 | Protein Coding | 39 | GC12P093569 | 3.965967417 |
| CCR9 | C-C Motif Chemokine Receptor 9 | Protein Coding | 38 | GC03P046396 | 3.965235949 |
| NOBOX | NOBOX Oogenesis Homeobox | Protein Coding | 32 | GC07M144397 | 3.964340448 |
| NENF | Neudesin Neurotrophic Factor | Protein Coding | 35 | GC01P212432 | 3.961498737 |
| TBC1D9 | TBC1 Domain Family Member 9 | Protein Coding | 34 | GC04M140621 | 3.960260868 |
| SLC26A3 | Solute Carrier Family 26 Member 3 | Protein Coding | 42 | GC07M107765 | 3.959009886 |
| HMBS | Hydroxymethylbilane Synthase | Protein Coding | 39 | GC11P119084 | 3.958282232 |
| CBX1 | Chromobox 1 | Protein Coding | 37 | GC17M048070 | 3.957974195 |
| HELQ | Helicase, POLQ Like | Protein Coding | 33 | GC04M083407 | 3.9571805 |
| AHSG | Alpha 2-HS Glycoprotein | Protein Coding | 39 | GC03P186626 | 3.957134485 |
| PABPC1 | Poly(A) Binding Protein Cytoplasmic 1 | Protein Coding | 38 | GC08M100685 | 3.956979275 |
| ACOX1 | Acyl-CoA Oxidase 1 | Protein Coding | 41 | GC17M075941 | 3.952021599 |
| RPS18 | Ribosomal Protein S18 | Protein Coding | 34 | GC06P080420 | 3.951706886 |
| ORAI1 | ORAI Calcium Release-Activated Calcium Modulator 1 | Protein Coding | 39 | GC12P125868 | 3.949262857 |
| CFI | Complement Factor I | Protein Coding | 42 | GC04M109732 | 3.948439837 |
| BCHE | Butyrylcholinesterase | Protein Coding | 46 | GC03M165772 | 3.947552204 |
| UBQLN1 | Ubiquilin 1 | Protein Coding | 37 | GC09M083659 | 3.946787119 |
| C4B | Complement C4B (Chido Blood Group) | Protein Coding | 40 | GC06P032014 | 3.946395397 |
| MLLT11 | MLLT11 Transcription Factor 7 Cofactor | Protein Coding | 30 | GC01P151193 | 3.945920229 |
| ACER3 | Alkaline Ceramidase 3 | Protein Coding | 37 | GC11P076860 | 3.943235874 |
| MIR1-1 | MicroRNA 1-1 | RNA Gene | 19 | GC20P063298 | 3.943174839 |
| TRERNA1 | Translation Regulatory Long Non-Coding RNA 1 | RNA Gene | 14 | GC20M050040 | 3.941958904 |
| UNC5C | Unc-5 Netrin Receptor C | Protein Coding | 37 | GC04M095162 | 3.941886425 |
| IGFALS | Insulin Like Growth Factor Binding Protein Acid Labile Subunit | Protein Coding | 40 | GC16M001790 | 3.941055536 |
| CCNC | Cyclin C | Protein Coding | 36 | GC06M099542 | 3.941009045 |
| NEIL2 | Nei Like DNA Glycosylase 2 | Protein Coding | 35 | GC08P011769 | 3.941006899 |
| CXCL11 | C-X-C Motif Chemokine Ligand 11 | Protein Coding | 38 | GC04M076033 | 3.940896511 |
| LRRC3B | Leucine Rich Repeat Containing 3B | Protein Coding | 30 | GC03P026640 | 3.939912796 |
| SRD5A3 | Steroid 5 Alpha-Reductase 3 | Protein Coding | 39 | GC04P055346 | 3.939888 |
| FGB | Fibrinogen Beta Chain | Protein Coding | 43 | GC04P154587 | 3.938946724 |
| ZCCHC8 | Zinc Finger CCHC-Type Containing 8 | Protein Coding | 32 | GC12M122472 | 3.93867445 |
| ALPG | Alkaline Phosphatase, Germ Cell | Protein Coding | 32 | GC02P232407 | 3.938662767 |
| CNNM4 | Cyclin And CBS Domain Divalent Metal Cation Transport Mediator 4 | Protein Coding | 36 | GC02P096790 | 3.936933041 |
| KRT6B | Keratin 6B | Protein Coding | 37 | GC12M052446 | 3.93632555 |
| CDK11A | Cyclin Dependent Kinase 11A | Protein Coding | 34 | GC01M001702 | 3.935318708 |
| PCGF2 | Polycomb Group Ring Finger 2 | Protein Coding | 35 | GC17M038733 | 3.932047129 |
| LINC02620 | Long Intergenic Non-Protein Coding RNA 2620 | RNA Gene | 9 | GC10M104475 | 3.930585384 |
| IMPDH2 | Inosine Monophosphate Dehydrogenase 2 | Protein Coding | 43 | GC03M051015 | 3.927551031 |
| DNAJC12 | DnaJ Heat Shock Protein Family (Hsp40) Member C12 | Protein Coding | 34 | GC10M067796 | 3.924101114 |
| MYH10 | Myosin Heavy Chain 10 | Protein Coding | 42 | GC17M008474 | 3.924006224 |
| TAB2 | TGF-Beta Activated Kinase 1 (MAP3K7) Binding Protein 2 | Protein Coding | 43 | GC06P149218 | 3.920686722 |
| RAB6C | RAB6C, Member RAS Oncogene Family | Protein Coding | 30 | GC02P129979 | 3.920636654 |
| ST7 | Suppression Of Tumorigenicity 7 | Protein Coding | 32 | GC07P117077 | 3.920576334 |
| BTG1 | BTG Anti-Proliferation Factor 1 | Protein Coding | 37 | GC12M092140 | 3.916484594 |
| TNNT2 | Troponin T2, Cardiac Type | Protein Coding | 43 | GC01M201359 | 3.916110516 |
| SIAH1 | Siah E3 Ubiquitin Protein Ligase 1 | Protein Coding | 42 | GC16M048357 | 3.915918589 |
| HBA2 | Hemoglobin Subunit Alpha 2 | Protein Coding | 39 | GC16P010621 | 3.913537264 |
| ANKAR | Ankyrin And Armadillo Repeat Containing | Protein Coding | 28 | GC02P189674 | 3.912238836 |
| HPS5 | HPS5 Biogenesis Of Lysosomal Organelles Complex 2 Subunit 2 | Protein Coding | 35 | GC11M018278 | 3.912033796 |
| SP100 | SP100 Nuclear Antigen | Protein Coding | 37 | GC02P230415 | 3.911456585 |
| GSTM2 | Glutathione S-Transferase Mu 2 | Protein Coding | 36 | GC01P109668 | 3.910975456 |
| KIF5B | Kinesin Family Member 5B | Protein Coding | 40 | GC10M032764 | 3.909029961 |
| GEMIN2 | Gem Nuclear Organelle Associated Protein 2 | Protein Coding | 33 | GC14P039114 | 3.907240391 |
| BABAM2 | BRISC And BRCA1 A Complex Member 2 | Protein Coding | 31 | GC02P027894 | 3.907056808 |
| CETN2 | Centrin 2 | Protein Coding | 36 | GC0XM152827 | 3.905607224 |
| SYNJ2BP | Synaptojanin 2 Binding Protein | Protein Coding | 32 | GC14M070366 | 3.905431747 |
| PACSIN3 | Protein Kinase C And Casein Kinase Substrate In Neurons 3 | Protein Coding | 34 | GC11M086874 | 3.904767513 |
| SPAM1 | Sperm Adhesion Molecule 1 | Protein Coding | 36 | GC07P123925 | 3.904727459 |
| EYA1 | EYA Transcriptional Coactivator And Phosphatase 1 | Protein Coding | 40 | GC08M071210 | 3.903928757 |
| SORT1 | Sortilin 1 | Protein Coding | 39 | GC01M109310 | 3.903768063 |
| PROK1 | Prokineticin 1 | Protein Coding | 35 | GC01P110451 | 3.903317451 |
| LTBP4 | Latent Transforming Growth Factor Beta Binding Protein 4 | Protein Coding | 38 | GC19P040592 | 3.901187897 |
| BRI3BP | BRI3 Binding Protein | Protein Coding | 30 | GC12P124993 | 3.900738716 |
| GLS2 | Glutaminase 2 | Protein Coding | 37 | GC12M056470 | 3.90042305 |
| ATP1A3 | ATPase Na+/K+ Transporting Subunit Alpha 3 | Protein Coding | 43 | GC19M041966 | 3.90004158 |
| EGLN2 | Egl-9 Family Hypoxia Inducible Factor 2 | Protein Coding | 40 | GC19P063653 | 3.899442196 |
| UGT1A5 | UDP Glucuronosyltransferase Family 1 Member A5 | Protein Coding | 33 | GC02P233712 | 3.89811182 |
| IRF7 | Interferon Regulatory Factor 7 | Protein Coding | 43 | GC11M000612 | 3.897902012 |
| RING1 | Ring Finger Protein 1 | Protein Coding | 36 | GC06P033208 | 3.897163391 |
| CNP | 2',3'-Cyclic Nucleotide 3' Phosphodiesterase | Protein Coding | 38 | GC17P041966 | 3.896021843 |
| RPL4 | Ribosomal Protein L4 | Protein Coding | 37 | GC15M066498 | 3.895854235 |
| ZNF281 | Zinc Finger Protein 281 | Protein Coding | 34 | GC01M200404 | 3.895265818 |
| NEK9 | NIMA Related Kinase 9 | Protein Coding | 41 | GC14M075079 | 3.894646645 |

| | | | | | | |
|---|---|---|---|---|---|---|
| HEMGN | Hemogen | Protein Coding | 32 | GC09M098965 | 3.894396544 |
| FOXP4 | Forkhead Box P4 | Protein Coding | 34 | GC06P080573 | 3.894315004 |
| CLEC7A | C-Type Lectin Domain Containing 7A | Protein Coding | 41 | GC12M020440 | 3.894086123 |
| KPNA5 | Karyopherin Subunit Alpha 5 | Protein Coding | 34 | GC06P116681 | 3.891844749 |
| CTHRC1 | Collagen Triple Helix Repeat Containing 1 | Protein Coding | 36 | GC08P103371 | 3.891314745 |
| ARSB | Arylsulfatase B | Protein Coding | 42 | GC05M078777 | 3.890940905 |
| RN7SK | RNA Component Of 7SK Nuclear Ribonucleoprotein | RNA Gene | 19 | GC06P052995 | 3.890638828 |
| RAB1A | RAB1A, Member RAS Oncogene Family | Protein Coding | 36 | GC02M065072 | 3.890481949 |
| COL6A1 | Collagen Type VI Alpha 1 Chain | Protein Coding | 39 | GC21P045981 | 3.888646841 |
| FRZB | Frizzled Related Protein | Protein Coding | 37 | GC02M182833 | 3.888432741 |
| ZBTB7A | Zinc Finger And BTB Domain Containing 7A | Protein Coding | 33 | GC19M005045 | 3.888309002 |
| PEX3 | Peroxisomal Biogenesis Factor 3 | Protein Coding | 38 | GC06P143450 | 3.887996674 |
| ENC1 | Ectodermal-Neural Cortex 1 | Protein Coding | 32 | GC05M074627 | 3.887900591 |
| C19orf48 | Chromosome 19 Open Reading Frame 48 | Pseudogene | 28 | GC19M050797 | 3.887112379 |
| DEAF1 | DEAF1 Transcription Factor | Protein Coding | 35 | GC11M000644 | 3.886748075 |
| ENOX2 | Ecto-NOX Disulfide-Thiol Exchanger 2 | Protein Coding | 34 | GC0XM130623 | 3.885698795 |
| PMP22 | Peripheral Myelin Protein 22 | Protein Coding | 40 | GC17M015229 | 3.882834196 |
| BLK | BLK Proto-Oncogene, Src Family Tyrosine Kinase | Protein Coding | 45 | GC08P011486 | 3.881598234 |
| PDIA3 | Protein Disulfide Isomerase Family A Member 3 | Protein Coding | 39 | GC15P043746 | 3.878232002 |
| AKAP1 | A-Kinase Anchoring Protein 1 | Protein Coding | 35 | GC17P057085 | 3.878072262 |
| FLT3LG | Fms Related Receptor Tyrosine Kinase 3 Ligand | Protein Coding | 37 | GC19P064062 | 3.872717619 |
| NSD3 | Nuclear Receptor Binding SET Domain Protein 3 | Protein Coding | 33 | GC08M038269 | 3.871054649 |
| ADH1A | Alcohol Dehydrogenase 1A (Class I), Alpha Polypeptide | Protein Coding | 38 | GC04M099276 | 3.870818615 |
| SOX1 | SRY-Box Transcription Factor 1 | Protein Coding | 33 | GC13P112067 | 3.870791912 |
| GLA | Galactosidase Alpha | Protein Coding | 44 | GC0XM101393 | 3.870181561 |
| NLRP1 | NLR Family Pyrin Domain Containing 1 | Protein Coding | 39 | GC17M005499 | 3.867465496 |
| SERPINA6 | Serpin Family A Member 6 | Protein Coding | 41 | GC14M100444 | 3.866880894 |
| MRPS23 | Mitochondrial Ribosomal Protein S23 | Protein Coding | 34 | GC17M057834 | 3.866711378 |
| FCGR1A | Fc Gamma Receptor Ia | Protein Coding | 37 | GC01P149813 | 3.866308212 |
| IFIH1 | Interferon Induced With Helicase C Domain 1 | Protein Coding | 42 | GC02M162267 | 3.866162062 |
| TUBB1 | Tubulin Beta 1 Class VI | Protein Coding | 43 | GC20P059020 | 3.864717484 |
| HK3 | Hexokinase 3 | Protein Coding | 39 | GC05M176882 | 3.863251686 |
| ERC1 | ELKS/RAB6-Interacting/CAST Family Member 1 | Protein Coding | 37 | GC12P000972 | 3.862607718 |
| ALYREF | Aly/REF Export Factor | Protein Coding | 33 | GC17M081887 | 3.862211704 |
| NAIP | NLR Family Apoptosis Inhibitory Protein | Protein Coding | 35 | GC05M070968 | 3.861064672 |
| PIGA | Phosphatidylinositol Glycan Anchor Biosynthesis Class A | Protein Coding | 40 | GC0XM015319 | 3.860646486 |
| ARHGEF5 | Rho Guanine Nucleotide Exchange Factor 5 | Protein Coding | 34 | GC07P144355 | 3.860414505 |
| HEY2 | Hes Related Family BHLH Transcription Factor With YRPW Motif 2 | Protein Coding | 37 | GC06P125730 | 3.860142946 |
| EPRS1 | Glutamyl-Prolyl-TRNA Synthetase 1 | Protein Coding | 35 | GC01M219969 | 3.860064507 |
| SMURF2 | SMAD Specific E3 Ubiquitin Protein Ligase 2 | Protein Coding | 38 | GC17M064542 | 3.859419823 |
| FGF14 | Fibroblast Growth Factor 14 | Protein Coding | 39 | GC13M101710 | 3.85844326 |
| IGHV4-38-2 | Immunoglobulin Heavy Variable 4-38-2 | Protein Coding | 7 | GC14U901616 | 3.858166933 |
| CEBPA-DT | CEBPA Divergent Transcript | RNA Gene | 15 | GC19P063389 | 3.857962132 |
| CDK3 | Cyclin Dependent Kinase 3 | Protein Coding | 36 | GC17P076008 | 3.856243134 |
| LINC00668 | Long Intergenic Non-Protein Coding RNA 668 | RNA Gene | 16 | GC18M006922 | 3.854890108 |
| EIF3D | Eukaryotic Translation Initiation Factor 3 Subunit D | Protein Coding | 34 | GC22M036510 | 3.854568958 |
| FOXD3 | Forkhead Box D3 | Protein Coding | 38 | GC01P063323 | 3.853627443 |
| ELOB | Elongin B | Protein Coding | 31 | GC16M006762 | 3.85341239 |
| FUCA1 | Alpha-L-Fucosidase 1 | Protein Coding | 43 | GC01M023845 | 3.852789402 |
| FAU | FAU Ubiquitin Like And Ribosomal Protein S30 Fusion | Protein Coding | 34 | GC11M065120 | 3.851267099 |
| CNTNAP2 | Contactin Associated Protein 2 | Protein Coding | 40 | GC07P146116 | 3.85103941 |
| LINC00115 | Long Intergenic Non-Protein Coding RNA 115 | RNA Gene | 17 | GC01M005498 | 3.850568771 |
| MYO6 | Myosin VI | Protein Coding | 41 | GC06P075749 | 3.850130796 |
| CWC27 | CWC27 Spliceosome Associated Cyclophilin | Protein Coding | 36 | GC05P064768 | 3.84834218 |
| NUTF2P3 | Nuclear Transport Factor 2 Pseudogene 3 | Pseudogene | 7 | GC09P077580 | 3.84726119 |
| CHRDL2 | Chordin Like 2 | Protein Coding | 34 | GC11M074696 | 3.846878529 |
| CHAT | Choline O-Acetyltransferase | Protein Coding | 43 | GC10P049609 | 3.845362663 |
| PPIG | Peptidylprolyl Isomerase G | Protein Coding | 37 | GC02P169584 | 3.843872547 |
| CD109 | CD109 Molecule | Protein Coding | 37 | GC06P073695 | 3.843764067 |
| SECTM1 | Secreted And Transmembrane 1 | Protein Coding | 34 | GC17M082321 | 3.841998577 |
| INHBA-AS1 | INHBA Antisense RNA 1 | RNA Gene | 15 | GC07P041693 | 3.84181428 |
| RAD18 | RAD18 E3 Ubiquitin Protein Ligase | Protein Coding | 38 | GC03M008775 | 3.841678619 |
| MAGEA5P | MAGE Family Member A5, Pseudogene | Pseudogene | 17 | GC0XM152119 | 3.841647863 |
| BTD | Biotinidase | Protein Coding | 40 | GC03P016887 | 3.840893269 |
| ANK2 | Ankyrin 2 | Protein Coding | 38 | GC04P112706 | 3.840496063 |
| FGF18 | Fibroblast Growth Factor 18 | Protein Coding | 37 | GC05P171419 | 3.839582205 |
| TMEM106A | Transmembrane Protein 106A | Protein Coding | 29 | GC17P043211 | 3.839148045 |
| SPANXA2 | SPANX Family Member A2 | Protein Coding | 22 | GC0XP141590 | 3.838869333 |
| DEPDC1B | DEP Domain Containing 1B | Protein Coding | 31 | GC05M060596 | 3.837904453 |
| PTGER3 | Prostaglandin E Receptor 3 | Protein Coding | 42 | GC01M070852 | 3.836167097 |
| MIR383 | MicroRNA 383 | RNA Gene | 17 | GC08M014853 | 3.835490704 |
| CTIF | Cap Binding Complex Dependent Translation Initiation Factor | Protein Coding | 29 | GC18P048539 | 3.834623575 |
| UTP20 | UTP20 Small Subunit Processome Component | Protein Coding | 27 | GC12P101280 | 3.834581375 |
| TUBA1C | Tubulin Alpha 1c | Protein Coding | 38 | GC12P049188 | 3.834125042 |
| TREM1 | Triggering Receptor Expressed On Myeloid Cells 1 | Protein Coding | 36 | GC06M041267 | 3.833280563 |
| GRHL1 | Grainyhead Like Transcription Factor 1 | Protein Coding | 32 | GC02P009951 | 3.83302784 |
| FXYD5 | FXYD Domain Containing Ion Transport Regulator 5 | Protein Coding | 35 | GC19P035154 | 3.832915306 |
| CD74 | CD74 Molecule | Protein Coding | 39 | GC05M150378 | 3.831729412 |
| DLEU7-AS1 | DLEU7 Antisense RNA 1 | RNA Gene | 17 | GC13P050121 | 3.831253529 |
| DDHD2 | DDHD Domain Containing 2 | Protein Coding | 36 | GC08P038225 | 3.830132246 |
| DSE | Dermatan Sulfate Epimerase | Protein Coding | 36 | GC06P116255 | 3.830075502 |
| TLX3 | T Cell Leukemia Homeobox 3 | Protein Coding | 35 | GC05P171309 | 3.829852104 |
| IL17RB | Interleukin 17 Receptor B | Protein Coding | 37 | GC03P053855 | 3.829626799 |
| EBF3 | EBF Transcription Factor 3 | Protein Coding | 35 | GC10M129835 | 3.826844692 |
| AMOT | Angiomotin | Protein Coding | 35 | GC0XM112774 | 3.826824188 |
| ZNF304 | Zinc Finger Protein 304 | Protein Coding | 27 | GC19P057351 | 3.826794147 |
| SRSF6 | Serine And Arginine Rich Splicing Factor 6 | Protein Coding | 34 | GC20P043457 | 3.826550007 |
| RAB3GAP1 | RAB3 GTPase Activating Protein Catalytic Subunit 1 | Protein Coding | 37 | GC02P135052 | 3.82597065 |
| TP53INP2 | Tumor Protein P53 Inducible Nuclear Protein 2 | Protein Coding | 31 | GC20P034704 | 3.825268745 |
| ADGRV1 | Adhesion G Protein-Coupled Receptor V1 | Protein Coding | 35 | GC05P090529 | 3.824074268 |
| PKP2 | Plakophilin 2 | Protein Coding | 40 | GC12M032790 | 3.822969437 |
| DDX10 | DEAD-Box Helicase 10 | Protein Coding | 35 | GC11P108569 | 3.821448565 |
| ITGA1 | Integrin Subunit Alpha 1 | Protein Coding | 39 | GC05P052788 | 3.821128607 |
| APOA2 | Apolipoprotein A2 | Protein Coding | 40 | GC01M161222 | 3.819522858 |
| DCLRE1C | DNA Cross-Link Repair 1C | Protein Coding | 39 | GC10M014897 | 3.819295406 |

| | | | | | |
|---|---|---|---|---|---|
| DPEP1 | Dipeptidase 1 | Protein Coding | 38 | GC16P089613 | 3.818680525 |
| AHNAK | AHNAK Nucleoprotein | Protein Coding | 34 | GC11M087115 | 3.816730261 |
| SPRED1 | Sprouty Related EVH1 Domain Containing 1 | Protein Coding | 37 | GC15P038252 | 3.816692829 |
| FCGR3B | Fc Gamma Receptor IIIb | Protein Coding | 39 | GC01M161623 | 3.816552877 |
| SET | SET Nuclear Proto-Oncogene | Protein Coding | 40 | GC09P128889 | 3.816403389 |
| NID1 | Nidogen 1 | Protein Coding | 39 | GC01M235975 | 3.815718174 |
| OIP5 | Opa Interacting Protein 5 | Protein Coding | 30 | GC15M041309 | 3.814827442 |
| PRKAR2B | Protein Kinase CAMP-Dependent Type II Regulatory Subunit Beta | Protein Coding | 40 | GC07P107044 | 3.81456089 |
| PSENEN | Presenilin Enhancer, Gamma-Secretase Subunit | Protein Coding | 39 | GC19P063461 | 3.813931942 |
| TPTE2 | Transmembrane Phosphoinositide 3-Phosphatase And Tensin Homolog 2 | Protein Coding | 32 | GC13M019422 | 3.813797235 |
| ARID4A | AT-Rich Interaction Domain 4A | Protein Coding | 34 | GC14P058298 | 3.812638044 |
| KLF9 | Kruppel Like Factor 9 | Protein Coding | 34 | GC09M070384 | 3.812498808 |
| UROD | Uroporphyrinogen Decarboxylase | Protein Coding | 40 | GC01P045307 | 3.81155014 |
| GALNS | Galactosamine (N-Acetyl)-6-Sulfatase | Protein Coding | 43 | GC16M088813 | 3.811129093 |
| KRT7-AS | KRT7 Antisense RNA 1 | RNA Gene | 11 | GC12M052246 | 3.810881138 |
| ZFX | Zinc Finger Protein X-Linked | Protein Coding | 34 | GC0XP024148 | 3.810823441 |
| PACS1 | Phosphofurin Acidic Cluster Sorting Protein 1 | Protein Coding | 38 | GC11P069594 | 3.809080362 |
| MIR299 | MicroRNA 299 | RNA Gene | 17 | GC14P109515 | 3.808560371 |
| PTK7 | Protein Tyrosine Kinase 7 (Inactive) | Protein Coding | 41 | GC06P043076 | 3.80794692 |
| ANXA7 | Annexin A7 | Protein Coding | 38 | GC10M073375 | 3.806995869 |
| WASL | WASP Like Actin Nucleation Promoting Factor | Protein Coding | 37 | GC07M123681 | 3.806620359 |
| PTCSC3 | Papillary Thyroid Carcinoma Susceptibility Candidate 3 | RNA Gene | 15 | GC14M036155 | 3.804584265 |
| MIR1-2 | MicroRNA 1-2 | RNA Gene | 18 | GC18M024067 | 3.803628922 |
| MIR19B1 | MicroRNA 19b-1 | RNA Gene | 18 | GC13P091540 | 3.802802086 |
| P3H3 | Prolyl 3-Hydroxylase 3 | Protein Coding | 29 | GC12P019834 | 3.80252862 |
| ITGB6 | Integrin Subunit Beta 6 | Protein Coding | 43 | GC02M160099 | 3.802415848 |
| ADAM19 | ADAM Metallopeptidase Domain 19 | Protein Coding | 39 | GC05M157395 | 3.802410364 |
| ANXA2P2 | Annexin A2 Pseudogene 2 | Pseudogene | 19 | GC09P039977 | 3.800897121 |
| XAF1 | XIAP Associated Factor 1 | Protein Coding | 35 | GC17P006757 | 3.800395966 |
| CAMK2G | Calcium/Calmodulin Dependent Protein Kinase II Gamma | Protein Coding | 43 | GC10M073812 | 3.80031538 |
| MIR518A1 | MicroRNA 518a-1 | RNA Gene | 17 | GC19P064305 | 3.799110651 |
| BBS2 | Bardet-Biedl Syndrome 2 | Protein Coding | 38 | GC16M056467 | 3.798980474 |
| GOT2 | Glutamic-Oxaloacetic Transaminase 2 | Protein Coding | 42 | GC16M058707 | 3.798829556 |
| CELF1 | CUGBP Elav-Like Family Member 1 | Protein Coding | 34 | GC11M086887 | 3.798514366 |
| CTSH | Cathepsin H | Protein Coding | 43 | GC15M081635 | 3.798386097 |
| CEP192 | Centrosomal Protein 192 | Protein Coding | 32 | GC18P017111 | 3.798241615 |
| FGFBP1 | Fibroblast Growth Factor Binding Protein 1 | Protein Coding | 36 | GC04M015937 | 3.797831535 |
| PTCSC2 | Papillary Thyroid Carcinoma Susceptibility Candidate 2 | RNA Gene | 13 | GC09M097699 | 3.79702878 |
| MIR711 | MicroRNA 711 | RNA Gene | 14 | GC03M048578 | 3.796719551 |
| RLN1 | Relaxin 1 | Protein Coding | 33 | GC09M005334 | 3.796373844 |
| SHROOM2 | Shroom Family Member 2 | Protein Coding | 32 | GC0XP009786 | 3.794510841 |
| SMOX | Spermine Oxidase | Protein Coding | 35 | GC20P004120 | 3.794253349 |
| LINC02041 | Long Intergenic Non-Protein Coding RNA 2041 | RNA Gene | 10 | GC03P187449 | 3.793334246 |
| NUP85 | Nucleoporin 85 | Protein Coding | 35 | GC17P075205 | 3.792745113 |
| HPS6 | HPS6 Biogenesis Of Lysosomal Organelles Complex 2 Subunit 3 | Protein Coding | 36 | GC10P102065 | 3.791398048 |
| MIR301B | MicroRNA 301b | RNA Gene | 17 | GC22P034527 | 3.791369438 |
| USP6 | Ubiquitin Specific Peptidase 6 | Protein Coding | 36 | GC17P005116 | 3.789430618 |
| POU2AF1 | POU Class 2 Homeobox Associating Factor 1 | Protein Coding | 36 | GC11M111352 | 3.788060427 |
| GPR65 | G Protein-Coupled Receptor 65 | Protein Coding | 36 | GC14P088005 | 3.785616636 |
| RASGRF2 | Ras Protein Specific Guanine Nucleotide Releasing Factor 2 | Protein Coding | 31 | GC05P080960 | 3.784127712 |
| PREX1 | Phosphatidylinositol-3,4,5-Trisphosphate Dependent Rac Exchange Factor 1 | Protein Coding | 39 | GC20M048624 | 3.783788204 |
| LUM | Lumican | Protein Coding | 37 | GC12M091102 | 3.783318281 |
| RERE | Arginine-Glutamic Acid Dipeptide Repeats | Protein Coding | 38 | GC01M008364 | 3.782630682 |
| CSMD1 | CUB And Sushi Multiple Domains 1 | Protein Coding | 35 | GC08M002953 | 3.781176805 |
| MAGEE1 | MAGE Family Member E1 | Protein Coding | 31 | GC0XP076427 | 3.780750036 |
| KLRC1 | Killer Cell Lectin Like Receptor C1 | Protein Coding | 38 | GC12M020447 | 3.779840231 |
| ZBTB20-AS4 | ZBTB20 Antisense RNA 4 | RNA Gene | 13 | GC03P115100 | 3.779816389 |
| DDX6P1 | DEAD-Box Helicase 6 Pseudogene 1 | Pseudogene | 7 | GC06M029329 | 3.779816389 |
| HNRNPH1 | Heterogeneous Nuclear Ribonucleoprotein H1 | Protein Coding | 35 | GC05M179614 | 3.778733015 |
| GRM6 | Glutamate Metabotropic Receptor 6 | Protein Coding | 43 | GC05M178978 | 3.778547287 |
| PRPH2 | Peripherin 2 | Protein Coding | 36 | GC06M063837 | 3.777938366 |
| ATAD3A | ATPase Family AAA Domain Containing 3A | Protein Coding | 36 | GC01P003618 | 3.776044607 |
| TNFRSF10A-AS1 | TNFRSF10A Antisense RNA 1 | RNA Gene | 11 | GC08P023190 | 3.774906874 |
| LIPE | Lipase E, Hormone Sensitive Type | Protein Coding | 43 | GC19M042401 | 3.774729729 |
| GOLGB1 | Golgin B1 | Protein Coding | 32 | GC03M121663 | 3.773841858 |
| NOL8 | Nucleolar Protein 8 | Protein Coding | 32 | GC09M092297 | 3.773341656 |
| DNAJC6 | DnaJ Heat Shock Protein Family (Hsp40) Member C6 | Protein Coding | 39 | GC01P065248 | 3.772572041 |
| SLC5A4-AS1 | SLC5A4 Antisense RNA 1 | RNA Gene | 12 | GC22P035070 | 3.771232605 |
| SLC7A11-AS1 | SLC7A11 Antisense RNA 1 | RNA Gene | 14 | GC04P138027 | 3.76984024 |
| TASOR2 | Transcription Activation Suppressor Family Member 2 | Protein Coding | 26 | GC10P005684 | 3.769813538 |
| NHEJ1 | Non-Homologous End Joining Factor 1 | Protein Coding | 37 | GC02M219115 | 3.769527674 |
| SEPTIN11 | Septin 11 | Protein Coding | 27 | GC04P076964 | 3.769376755 |
| EYA2 | EYA Transcriptional Coactivator And Phosphatase 2 | Protein Coding | 36 | GC20P046894 | 3.768966198 |
| EFNA4 | Ephrin A4 | Protein Coding | 38 | GC01P155063 | 3.766939163 |
| CDH20 | Cadherin 20 | Protein Coding | 34 | GC18P061333 | 3.766370773 |
| PRPF4B | Pre-MRNA Processing Factor 4B | Protein Coding | 36 | GC06P004021 | 3.764976978 |
| KMT5A | Lysine Methyltransferase 5A | Protein Coding | 35 | GC12P125905 | 3.764817953 |
| GATA5 | GATA Binding Protein 5 | Protein Coding | 38 | GC20M062464 | 3.764757633 |
| SIRPA | Signal Regulatory Protein Alpha | Protein Coding | 39 | GC20P001894 | 3.764717817 |
| ZFPM2-AS1 | ZFPM2 Antisense RNA 1 | RNA Gene | 14 | GC08M105546 | 3.764493465 |
| C10orf143 | Chromosome 10 Open Reading Frame 143 | Protein Coding | 14 | GC10M130020 | 3.76326561 |
| MAGEB2 | MAGE Family Member B2 | Protein Coding | 31 | GC0XP030215 | 3.762154341 |
| MEGF8 | Multiple EGF Like Domains 8 | Protein Coding | 34 | GC19P042325 | 3.7602911 |
| P2RY2 | Purinergic Receptor P2Y2 | Protein Coding | 43 | GC11P073202 | 3.760171652 |
| SLC22A5 | Solute Carrier Family 22 Member 5 | Protein Coding | 43 | GC05P132369 | 3.759006977 |
| GALNT5 | Polypeptide N-Acetylgalactosaminyltransferase 5 | Protein Coding | 35 | GC02P157258 | 3.757232904 |
| EIF6 | Eukaryotic Translation Initiation Factor 6 | Protein Coding | 35 | GC20M035278 | 3.75538373 |
| ZNF318 | Zinc Finger Protein 318 | Protein Coding | 32 | GC06M063851 | 3.752943993 |
| SLC25A1 | Solute Carrier Family 25 Member 1 | Protein Coding | 42 | GC22M019311 | 3.75030899 |
| FMR1NB | FMR1 Neighbor | Protein Coding | 27 | GC0XP147981 | 3.748069048 |
| LPO | Lactoperoxidase | Protein Coding | 36 | GC17P058218 | 3.747520447 |
| H4C13 | H4 Clustered Histone 13 | Protein Coding | 26 | GC06M064169 | 3.745223999 |
| AKAP8 | A-Kinase Anchoring Protein 8 | Protein Coding | 36 | GC19M015354 | 3.744736671 |
| ERVW-1 | Endogenous Retrovirus Group W Member 1, Envelope | Protein Coding | 31 | GC07M092468 | 3.744061708 |
| RDX | Radixin | Protein Coding | 43 | GC11M109864 | 3.742994785 |

| | | | | | |
|---|---|---|---|---|---|
| CREB3L1 | CAMP Responsive Element Binding Protein 3 Like 1 | Protein Coding | 38 | GC11P046466 | 3.742152214 |
| KHSRP | KH-Type Splicing Regulatory Protein | Protein Coding | 37 | GC19M006413 | 3.741239309 |
| AZU1 | Azurocidin 1 | Protein Coding | 36 | GC19P000825 | 3.740998745 |
| CCDC62 | Coiled-Coil Domain Containing 62 | Protein Coding | 30 | GC12P125891 | 3.740304232 |
| NR1H3 | Nuclear Receptor Subfamily 1 Group H Member 3 | Protein Coding | 42 | GC11P047248 | 3.739714622 |
| KCNMA1 | Potassium Calcium-Activated Channel Subfamily M Alpha 1 | Protein Coding | 45 | GC10M076869 | 3.739642859 |
| CCR10 | C-C Motif Chemokine Receptor 10 | Protein Coding | 34 | GC17M042678 | 3.739617586 |
| POLD3 | DNA Polymerase Delta 3, Accessory Subunit | Protein Coding | 35 | GC11P074526 | 3.739035368 |
| DOCK1 | Dedicator Of Cytokinesis 1 | Protein Coding | 40 | GC10P126905 | 3.738569021 |
| EIF4A2 | Eukaryotic Translation Initiation Factor 4A2 | Protein Coding | 39 | GC03P186783 | 3.737977028 |
| BNC2-AS1 | BNC2 Antisense RNA 1 | RNA Gene | 14 | GC09P016727 | 3.733263254 |
| FOXF1 | Forkhead Box F1 | Protein Coding | 38 | GC16P086510 | 3.732960939 |
| FLOT1 | Flotillin 1 | Protein Coding | 36 | GC06M063532 | 3.731366158 |
| FSCB | Fibrous Sheath CABYR Binding Protein | Protein Coding | 31 | GC14M044504 | 3.730916023 |
| SLC29A3 | Solute Carrier Family 29 Member 3 | Protein Coding | 40 | GC10P071320 | 3.730112791 |
| GP1BA | Glycoprotein Ib Platelet Subunit Alpha | Protein Coding | 42 | GC17P004932 | 3.729935646 |
| PSTPIP1 | Proline-Serine-Threonine Phosphatase Interacting Protein 1 | Protein Coding | 40 | GC15P076993 | 3.729307652 |
| CDH4 | Cadherin 4 | Protein Coding | 38 | GC20P061252 | 3.729252338 |
| TCN2 | Transcobalamin 2 | Protein Coding | 38 | GC22P034983 | 3.729147196 |
| PCSK1 | Proprotein Convertase Subtilisin/Kexin Type 1 | Protein Coding | 44 | GC05M096391 | 3.728699207 |
| RAB31 | RAB31, Member RAS Oncogene Family | Protein Coding | 35 | GC18P009701 | 3.726445675 |
| MACC1-AS1 | MACC1 Antisense RNA 1 | RNA Gene | 15 | GC07P020183 | 3.726291656 |
| CDCA7 | Cell Division Cycle Associated 7 | Protein Coding | 38 | GC02P173354 | 3.725506783 |
| LOC110485085 | BRCA1P1 Intergenic Recombination Region | Biological Region | 2 | GC17P053869 | 3.724421501 |
| ADCY10 | Adenylate Cyclase 10 | Protein Coding | 40 | GC01M167809 | 3.724364281 |
| CLIP1 | CAP-Gly Domain Containing Linker Protein 1 | Protein Coding | 39 | GC12M122271 | 3.723872662 |
| SART3 | Spliceosome Associated Factor 3, U4/U6 Recycling Protein | Protein Coding | 32 | GC12M108522 | 3.722851276 |
| CSTB | Cystatin B | Protein Coding | 42 | GC21M043772 | 3.720078945 |
| FLII | FLII Actin Remodeling Protein | Protein Coding | 39 | GC17M018244 | 3.719997644 |
| LINC01101 | Long Intergenic Non-Protein Coding RNA 1101 | RNA Gene | 15 | GC02M120464 | 3.719118118 |
| TRIB3 | Tribbles Pseudokinase 3 | Protein Coding | 38 | GC20P000361 | 3.718863487 |
| SULT1A2 | Sulfotransferase Family 1A Member 2 | Protein Coding | 38 | GC16M028591 | 3.718719482 |
| EPHA8 | EPH Receptor A8 | Protein Coding | 39 | GC01P022563 | 3.718276024 |
| ZMAT1 | Zinc Finger Matrin-Type 1 | Protein Coding | 28 | GC0XM101882 | 3.717115164 |
| RNF32-DT | RNF32 Divergent Transcript | RNA Gene | 18 | GC07M156390 | 3.717115164 |
| WDR5 | WD Repeat Domain 5 | Protein Coding | 39 | GC09P134135 | 3.716996908 |
| KRBOX4 | KRAB Box Domain Containing 4 | Protein Coding | 29 | GC0XP046558 | 3.716319084 |
| SKIL | SKI Like Proto-Oncogene | Protein Coding | 38 | GC03P170357 | 3.715857506 |
| ZC4H2 | Zinc Finger C4H2-Type Containing | Protein Coding | 33 | GC0XM064915 | 3.714548588 |
| SAV1 | Salvador Family WW Domain Containing Protein 1 | Protein Coding | 34 | GC14M050632 | 3.714003086 |
| AKAP12 | A-Kinase Anchoring Protein 12 | Protein Coding | 37 | GC06P151239 | 3.713811159 |
| TRARG1 | Trafficking Regulator Of GLUT4 (SLC2A4) 1 | Protein Coding | 27 | GC17P001479 | 3.713243246 |
| ZIC1 | Zic Family Member 1 | Protein Coding | 40 | GC03P147393 | 3.711550236 |
| CTRL | Chymotrypsin Like | Protein Coding | 36 | GC16M067927 | 3.711316347 |
| BBS1 | Bardet-Biedl Syndrome 1 | Protein Coding | 34 | GC11P069645 | 3.710705519 |
| ESCO2 | Establishment Of Sister Chromatid Cohesion N-Acetyltransferase 2 | Protein Coding | 35 | GC08P027771 | 3.709608078 |
| MGLL | Monoglyceride Lipase | Protein Coding | 40 | GC03M127689 | 3.708191395 |
| TAF1 | TATA-Box Binding Protein Associated Factor 1 | Protein Coding | 40 | GC0XP071366 | 3.708178043 |
| ZMYM2 | Zinc Finger MYM-Type Containing 2 | Protein Coding | 37 | GC13P020050 | 3.70757103 |
| CYBB | Cytochrome B-245 Beta Chain | Protein Coding | 43 | GC0XP037780 | 3.707318068 |
| GJC2 | Gap Junction Protein Gamma 2 | Protein Coding | 37 | GC01P229188 | 3.707183123 |
| MRPS11 | Mitochondrial Ribosomal Protein S11 | Protein Coding | 31 | GC15P088467 | 3.705995321 |
| CYB5R3 | Cytochrome B5 Reductase 3 | Protein Coding | 40 | GC22M056126 | 3.705334902 |
| NR2F1 | Nuclear Receptor Subfamily 2 Group F Member 1 | Protein Coding | 41 | GC05P093583 | 3.705318451 |
| ACSL3 | Acyl-CoA Synthetase Long Chain Family Member 3 | Protein Coding | 36 | GC02P222860 | 3.705271721 |
| ZNF704 | Zinc Finger Protein 704 | Protein Coding | 28 | GC08M080628 | 3.704743862 |
| TCF21 | Transcription Factor 21 | Protein Coding | 35 | GC06P133889 | 3.704104424 |
| HNRNPM | Heterogeneous Nuclear Ribonucleoprotein M | Protein Coding | 34 | GC19P008444 | 3.704069853 |
| PDE4D | Phosphodiesterase 4D | Protein Coding | 44 | GC05M058969 | 3.700333595 |
| WARS1 | Tryptophanyl-TRNA Synthetase 1 | Protein Coding | 35 | GC14M100517 | 3.700150013 |
| ACKR4 | Atypical Chemokine Receptor 4 | Protein Coding | 32 | GC03P132597 | 3.699507952 |
| UNC5B | Unc-5 Netrin Receptor B | Protein Coding | 36 | GC10P071212 | 3.698685884 |
| SMG8 | SMG8 Nonsense Mediated MRNA Decay Factor | Protein Coding | 31 | GC17P059209 | 3.697438478 |
| MAP3K4 | Mitogen-Activated Protein Kinase Kinase Kinase 4 | Protein Coding | 39 | GC06P160991 | 3.697296143 |
| MIR369 | MicroRNA 369 | RNA Gene | 19 | GC14P109522 | 3.696402788 |
| TNFSF8 | TNF Superfamily Member 8 | Protein Coding | 35 | GC09M114893 | 3.696004629 |
| RCE1 | Ras Converting CAAX Endopeptidase 1 | Protein Coding | 35 | GC11P066842 | 3.69546175 |
| H4C4 | H4 Clustered Histone 4 | Protein Coding | 26 | GC06M064170 | 3.693936825 |
| DIAPH1 | Diaphanous Related Formin 1 | Protein Coding | 42 | GC05M141516 | 3.693885088 |
| LFS3 | Li-Fraumeni Syndrome 3 | Genetic Locus | 2 | GC00U936829 | 3.692983627 |
| SIRLNT | SIRT1 Regulating LncRNA Tumor Promoter | RNA Gene | 10 | GC08M040300 | 3.692693233 |
| HIPK1 | Homeodomain Interacting Protein Kinase 1 | Protein Coding | 36 | GC01P113929 | 3.691422939 |
| PLPP4 | Phospholipid Phosphatase 4 | Protein Coding | 28 | GC10P120457 | 3.688180685 |
| DEFB1 | Defensin Beta 1 | Protein Coding | 35 | GC08M006870 | 3.684690714 |
| USP2 | Ubiquitin Specific Peptidase 2 | Protein Coding | 40 | GC11M119355 | 3.682838678 |
| CDC16 | Cell Division Cycle 16 | Protein Coding | 35 | GC13P114234 | 3.682464123 |
| FAM107A | Family With Sequence Similarity 107 Member A | Protein Coding | 33 | GC03M058729 | 3.681524754 |
| RUNX1-IT1 | RUNX1 Intronic Transcript 1 | RNA Gene | 16 | GC21M035037 | 3.680258751 |
| ZMYND11 | Zinc Finger MYND-Type Containing 11 | Protein Coding | 37 | GC10P000134 | 3.67886138 |
| PGPEP1 | Pyroglutamyl-Peptidase I | Protein Coding | 32 | GC19P063123 | 3.676679611 |
| ACSL5 | Acyl-CoA Synthetase Long Chain Family Member 5 | Protein Coding | 38 | GC10P112374 | 3.676298141 |
| CLDN11 | Claudin 11 | Protein Coding | 36 | GC03P170418 | 3.673812151 |
| MAGEA8 | MAGE Family Member A8 | Protein Coding | 31 | GC0XP149881 | 3.672959089 |
| ERRFI1 | ERBB Receptor Feedback Inhibitor 1 | Protein Coding | 34 | GC01M008004 | 3.670199394 |
| TRAP1 | TNF Receptor Associated Protein 1 | Protein Coding | 39 | GC16M006857 | 3.669699907 |
| ABL2 | ABL Proto-Oncogene 2, Non-Receptor Tyrosine Kinase | Protein Coding | 40 | GC01M179128 | 3.669240236 |
| POLR2L | RNA Polymerase II, I And III Subunit L | Protein Coding | 36 | GC11M002934 | 3.668922186 |
| HOOK1 | Hook Microtubule Tethering Protein 1 | Protein Coding | 35 | GC01P059814 | 3.668882847 |
| ATXN3 | Ataxin 3 | Protein Coding | 40 | GC14M100415 | 3.668093204 |
| CACNA1C | Calcium Voltage-Gated Channel Subunit Alpha1 C | Protein Coding | 45 | GC12P001970 | 3.667553902 |
| ZNF132 | Zinc Finger Protein 132 | Protein Coding | 30 | GC19M058432 | 3.6667943 |
| PSME3 | Proteasome Activator Subunit 3 | Protein Coding | 36 | GC17P042824 | 3.666786909 |
| CERS2 | Ceramide Synthase 2 | Protein Coding | 36 | GC01M150960 | 3.666517019 |
| CNTN1 | Contactin 1 | Protein Coding | 40 | GC12P040692 | 3.666001797 |
| LINC02224 | Long Intergenic Non-Protein Coding RNA 2224 | RNA Gene | 10 | GC05M044499 | 3.665185452 |

| | | | | | |
|---|---|---|---|---|---|
| MT-ND4 | Mitochondrially Encoded NADH:Ubiquinone Oxidoreductase Core Subunit 4 | Protein Coding | 30 | GCMTP010762 | 3.664828062 |
| PPP1R12A | Protein Phosphatase 1 Regulatory Subunit 12A | Protein Coding | 38 | GC12M079773 | 3.663789988 |
| STK39 | Serine/Threonine Kinase 39 | Protein Coding | 39 | GC02M167954 | 3.662127018 |
| GFPT1 | Glutamine--Fructose-6-Phosphate Transaminase 1 | Protein Coding | 41 | GC02M069283 | 3.661580563 |
| MCU | Mitochondrial Calcium Uniporter | Protein Coding | 32 | GC10P072692 | 3.661520481 |
| SILC1 | Sciatic Injury Induced LincRNA Upregulator Of SOX11 | RNA Gene | 14 | GC02P005933 | 3.661405802 |
| XAB2 | XPA Binding Protein 2 | Protein Coding | 34 | GC19M007619 | 3.660622597 |
| ELF5 | E74 Like ETS Transcription Factor 5 | Protein Coding | 35 | GC11M034500 | 3.660621643 |
| STON1-GTF2A1L | STON1-GTF2A1L Readthrough | Protein Coding | 26 | GC02P048529 | 3.660470486 |
| EPHA10 | EPH Receptor A10 | Protein Coding | 35 | GC01M037713 | 3.660320282 |
| MED14 | Mediator Complex Subunit 14 | Protein Coding | 34 | GC0XM040648 | 3.660191536 |
| SMC2 | Structural Maintenance Of Chromosomes 2 | Protein Coding | 34 | GC09P104094 | 3.660057783 |
| RPLP0 | Ribosomal Protein Lateral Stalk Subunit P0 | Protein Coding | 36 | GC12M120196 | 3.655363083 |
| MRGBP | MRG Domain Binding Protein | Protein Coding | 28 | GC20P063304 | 3.655190945 |
| GTF2H1 | General Transcription Factor IIH Subunit 1 | Protein Coding | 38 | GC11P018483 | 3.654026508 |
| PPA1 | Inorganic Pyrophosphatase 1 | Protein Coding | 36 | GC10M070202 | 3.653538704 |
| GALC | Galactosylceramidase | Protein Coding | 41 | GC14M087837 | 3.650231361 |
| LBP | Lipopolysaccharide Binding Protein | Protein Coding | 39 | GC20P038346 | 3.649563313 |
| PAF1 | PAF1 Homolog, Paf1/RNA Polymerase II Complex Component | Protein Coding | 31 | GC19M039385 | 3.649199486 |
| SPANXB1 | SPANX Family Member B1 | Protein Coding | 25 | GC0XP141002 | 3.649175167 |
| RCHY1 | Ring Finger And CHY Zinc Finger Domain Containing 1 | Protein Coding | 35 | GC04M075479 | 3.646914005 |
| TAX1BP1 | Tax1 Binding Protein 1 | Protein Coding | 35 | GC07P027739 | 3.646796227 |
| UPF1 | UPF1 RNA Helicase And ATPase | Protein Coding | 35 | GC19P018831 | 3.646055222 |
| KCNA5 | Potassium Voltage-Gated Channel Subfamily A Member 5 | Protein Coding | 42 | GC12P005043 | 3.646029234 |
| APOBEC3A | Apolipoprotein B MRNA Editing Enzyme Catalytic Subunit 3A | Protein Coding | 36 | GC22P038952 | 3.64525795 |
| TFAM | Transcription Factor A, Mitochondrial | Protein Coding | 39 | GC10P058385 | 3.643566608 |
| PRKAG2 | Protein Kinase AMP-Activated Non-Catalytic Subunit Gamma 2 | Protein Coding | 44 | GC07M151556 | 3.642923355 |
| SPAG1 | Sperm Associated Antigen 1 | Protein Coding | 35 | GC08P100157 | 3.642868519 |
| DDX18 | DEAD-Box Helicase 18 | Protein Coding | 36 | GC02P121691 | 3.642773867 |
| F9 | Coagulation Factor IX | Protein Coding | 43 | GC0XP139530 | 3.642415285 |
| ABCC6 | ATP Binding Cassette Subfamily C Member 6 | Protein Coding | 42 | GC16M016148 | 3.642145395 |
| DYNC1H1 | Dynein Cytoplasmic 1 Heavy Chain 1 | Protein Coding | 39 | GC14P109254 | 3.642117977 |
| F12 | Coagulation Factor XII | Protein Coding | 45 | GC05M177402 | 3.641447067 |
| EIF3B | Eukaryotic Translation Initiation Factor 3 Subunit B | Protein Coding | 34 | GC07P002354 | 3.640553951 |
| FATE1 | Fetal And Adult Testis Expressed 1 | Protein Coding | 30 | GC0XP151716 | 3.639186621 |
| NEDD4L | NEDD4 Like E3 Ubiquitin Protein Ligase | Protein Coding | 42 | GC18P058044 | 3.637785912 |
| NBPF3 | NBPF Member 3 | Protein Coding | 31 | GC01P021809 | 3.636919498 |
| HPD | 4-Hydroxyphenylpyruvate Dioxygenase | Protein Coding | 40 | GC12M121839 | 3.634395838 |
| HNRNPF | Heterogeneous Nuclear Ribonucleoprotein F | Protein Coding | 35 | GC10M043385 | 3.634162426 |
| NAA25 | N-Alpha-Acetyltransferase 25, NatB Auxiliary Subunit | Protein Coding | 31 | GC12M112026 | 3.63410902 |
| SMURF1 | SMAD Specific E3 Ubiquitin Protein Ligase 1 | Protein Coding | 40 | GC07M099027 | 3.633199215 |
| DSPP | Dentin Sialophosphoprotein | Protein Coding | 35 | GC04P087608 | 3.633101702 |
| ARHGAP1 | Rho GTPase Activating Protein 1 | Protein Coding | 39 | GC11M086865 | 3.632814407 |
| MIR362 | MicroRNA 362 | RNA Gene | 15 | GC0XP050565 | 3.629173994 |
| LRP2 | LDL Receptor Related Protein 2 | Protein Coding | 44 | GC02M169127 | 3.629166126 |
| FXN | Frataxin | Protein Coding | 41 | GC09P069035 | 3.62884903 |
| LCAT | Lecithin-Cholesterol Acyltransferase | Protein Coding | 43 | GC16M067939 | 3.628701925 |
| TNXA | Tenascin XA (Pseudogene) | Pseudogene | 19 | GC06M063630 | 3.628555298 |
| CEBPD | CCAAT Enhancer Binding Protein Delta | Protein Coding | 34 | GC08M047759 | 3.628303051 |
| SULT2A1 | Sulfotransferase Family 2A Member 1 | Protein Coding | 39 | GC19M047870 | 3.628287554 |
| MED28 | Mediator Complex Subunit 28 | Protein Coding | 33 | GC04P017777 | 3.627975464 |
| KRT9 | Keratin 9 | Protein Coding | 38 | GC17M041565 | 3.627038479 |
| H4C9 | H4 Clustered Histone 9 | Protein Coding | 27 | GC06P081047 | 3.625943422 |
| KCTD13 | Potassium Channel Tetramerization Domain Containing 13 | Protein Coding | 32 | GC16M029905 | 3.623393536 |
| FAM120A | Family With Sequence Similarity 120A | Protein Coding | 33 | GC09P093736 | 3.623213291 |
| CCNT1 | Cyclin T1 | Protein Coding | 36 | GC12M048688 | 3.622972488 |
| CCNG2 | Cyclin G2 | Protein Coding | 35 | GC04P077158 | 3.621949196 |
| CRAT | Carnitine O-Acetyltransferase | Protein Coding | 40 | GC09M129094 | 3.620429993 |
| PTPN9 | Protein Tyrosine Phosphatase Non-Receptor Type 9 | Protein Coding | 36 | GC15M075463 | 3.618000507 |
| ARMC12 | Armadillo Repeat Containing 12 | Protein Coding | 27 | GC06P080523 | 3.617790937 |
| AKR7L | Aldo-Keto Reductase Family 7 Like (Gene/Pseudogene) | Protein Coding | 26 | GC01M019265 | 3.617717028 |
| MATR3 | Matrin 3 | Protein Coding | 38 | GC05P139274 | 3.616630077 |
| ATP11A | ATPase Phospholipid Transporting 11A | Protein Coding | 38 | GC13P112690 | 3.61593771 |
| SLC34A1 | Solute Carrier Family 34 Member 1 | Protein Coding | 39 | GC05P177472 | 3.613817215 |
| BBS9 | Bardet-Biedl Syndrome 9 | Protein Coding | 35 | GC07P033223 | 3.613144875 |
| CFHR5 | Complement Factor H Related 5 | Protein Coding | 37 | GC01P196977 | 3.612778187 |
| IGKC | Immunoglobulin Kappa Constant | Protein Coding | 31 | GC02M090797 | 3.612022161 |
| LINC01612 | Long Intergenic Non-Protein Coding RNA 1612 | RNA Gene | 11 | GC04P170226 | 3.611973286 |
| GSTA2 | Glutathione S-Transferase Alpha 2 | Protein Coding | 37 | GC06M052750 | 3.611286402 |
| MARCKS | Myristoylated Alanine Rich Protein Kinase C Substrate | Protein Coding | 35 | GC06P113857 | 3.610671043 |
| MAZ | MYC Associated Zinc Finger Protein | Protein Coding | 36 | GC16P029806 | 3.610413074 |
| RAD51AP1 | RAD51 Associated Protein 1 | Protein Coding | 32 | GC12P019787 | 3.609788418 |
| CMM | Cutaneous Malignant Melanoma/Dysplastic Nevus | Genetic Locus | 3 | GC01U990027 | 3.609564304 |
| RBM3 | RNA Binding Motif Protein 3 | Protein Coding | 34 | GC0XP048574 | 3.608455181 |
| RPS8 | Ribosomal Protein S8 | Protein Coding | 34 | GC01P044775 | 3.608415604 |
| SARM1 | Sterile Alpha And TIR Motif Containing 1 | Protein Coding | 35 | GC17P028364 | 3.608089924 |
| CISD2 | CDGSH Iron Sulfur Domain 2 | Protein Coding | 37 | GC04P102868 | 3.606340647 |
| H4C6 | H4 Clustered Histone 6 | Protein Coding | 26 | GC06P081046 | 3.605430126 |
| TPTE | Transmembrane Phosphatase With Tensin Homology | Protein Coding | 33 | GC21P010521 | 3.604643822 |
| INA | Internexin Neuronal Intermediate Filament Protein Alpha | Protein Coding | 35 | GC10P103277 | 3.603865147 |
| FLOT2 | Flotillin 2 | Protein Coding | 36 | GC17M034586 | 3.602081299 |
| INPP5E | Inositol Polyphosphate-5-Phosphatase E | Protein Coding | 39 | GC09M136428 | 3.601693153 |
| HUWE1 | HECT, UBA And WWE Domain Containing E3 Ubiquitin Protein Ligase 1 | Protein Coding | 39 | GC0XM053532 | 3.601502419 |
| MIR26A2 | MicroRNA 26a-2 | RNA Gene | 19 | GC12M057824 | 3.597929478 |
| RPP38 | Ribonuclease P/MRP Subunit P38 | Protein Coding | 31 | GC10P015097 | 3.597655535 |
| CHAF1B | Chromatin Assembly Factor 1 Subunit B | Protein Coding | 36 | GC21P036385 | 3.597314119 |
| PXDNL | Peroxidasin Like | Protein Coding | 31 | GC08M051319 | 3.596999645 |
| NR2F1-AS1 | NR2F1 Antisense RNA 1 | RNA Gene | 17 | GC05M093409 | 3.596466303 |
| PEG3 | Paternally Expressed 3 | Protein Coding | 35 | GC19M056810 | 3.595702171 |
| EMP2 | Epithelial Membrane Protein 2 | Protein Coding | 39 | GC16M010541 | 3.594908237 |
| TRPM4 | Transient Receptor Potential Cation Channel Subfamily M Member 4 | Protein Coding | 40 | GC19P049157 | 3.593902826 |
| PDLIM5 | PDZ And LIM Domain 5 | Protein Coding | 35 | GC04P094451 | 3.591308594 |
| PPP2R2B | Protein Phosphatase 2 Regulatory Subunit Bbeta | Protein Coding | 40 | GC05M146582 | 3.590724468 |
| PCDH11X | Protocadherin 11 X-Linked | Protein Coding | 32 | GC0XP091779 | 3.590541363 |
| ABCC9 | ATP Binding Cassette Subfamily C Member 9 | Protein Coding | 42 | GC12M021797 | 3.590419769 |

| | | | | | |
|---|---|---|---|---|---|
| EBF1 | EBF Transcription Factor 1 | Protein Coding | 36 | GC05M158695 | 3.589778185 |
| WASF2 | WASP Family Member 2 | Protein Coding | 38 | GC01M027404 | 3.588263035 |
| CCNDBP1 | Cyclin D1 Binding Protein 1 | Protein Coding | 31 | GC15P043185 | 3.58804059 |
| VLDLR | Very Low Density Lipoprotein Receptor | Protein Coding | 43 | GC09P002611 | 3.587836981 |
| DLX4 | Distal-Less Homeobox 4 | Protein Coding | 38 | GC17P049968 | 3.587734699 |
| DLL3 | Delta Like Canonical Notch Ligand 3 | Protein Coding | 39 | GC19P039498 | 3.587605953 |
| RPL7 | Ribosomal Protein L7 | Protein Coding | 37 | GC08M073290 | 3.587023735 |
| ASF1B | Anti-Silencing Function 1B Histone Chaperone | Protein Coding | 36 | GC19M014119 | 3.586907864 |
| CACNA1A | Calcium Voltage-Gated Channel Subunit Alpha1 A | Protein Coding | 43 | GC19M013206 | 3.585238934 |
| ATF7 | Activating Transcription Factor 7 | Protein Coding | 36 | GC12M053527 | 3.585083485 |
| PRUNE1 | Prune Exopolyphosphatase 1 | Protein Coding | 32 | GC01P151008 | 3.584516287 |
| CNTN6 | Contactin 6 | Protein Coding | 36 | GC03P000978 | 3.583545923 |
| ILF3 | Interleukin Enhancer Binding Factor 3 | Protein Coding | 35 | GC19P010655 | 3.583275318 |
| CDC5L | Cell Division Cycle 5 Like | Protein Coding | 36 | GC06P044387 | 3.582583427 |
| PHIP | Pleckstrin Homology Domain Interacting Protein | Protein Coding | 37 | GC06M078934 | 3.582049608 |
| DDX39B | DExD-Box Helicase 39B | Protein Coding | 34 | GC06M031530 | 3.581954956 |
| BNIP3L | BCL2 Interacting Protein 3 Like | Protein Coding | 36 | GC08P026296 | 3.581709385 |
| CITED1 | Cbp/P300 Interacting Transactivator With Glu/Asp Rich Carboxy-Terminal Domain 1 | Protein Coding | 33 | GC0XM072301 | 3.581156731 |
| ANXA8 | Annexin A8 | Protein Coding | 31 | GC10M047460 | 3.580718994 |
| PVALB | Parvalbumin | Protein Coding | 36 | GC22M036800 | 3.577981472 |
| MTM1 | Myotubularin 1 | Protein Coding | 39 | GC0XP150562 | 3.576954126 |
| RTN4 | Reticulon 4 | Protein Coding | 40 | GC02M054934 | 3.576379776 |
| NRCAM | Neuronal Cell Adhesion Molecule | Protein Coding | 38 | GC07M108147 | 3.575484037 |
| ACADM | Acyl-CoA Dehydrogenase Medium Chain | Protein Coding | 40 | GC01P075724 | 3.574665546 |
| SRPRA | SRP Receptor Subunit Alpha | Protein Coding | 30 | GC11M126255 | 3.573185205 |
| FRAS1 | Fraser Extracellular Matrix Complex Subunit 1 | Protein Coding | 35 | GC04P078057 | 3.572789192 |
| OSMR | Oncostatin M Receptor | Protein Coding | 40 | GC05P038845 | 3.571794987 |
| SLC25A4 | Solute Carrier Family 25 Member 4 | Protein Coding | 44 | GC04P185143 | 3.567798615 |
| EFL1 | Elongation Factor Like GTPase 1 | Protein Coding | 31 | GC15M088703 | 3.567722559 |
| ATG16L1 | Autophagy Related 16 Like 1 | Protein Coding | 38 | GC02P233249 | 3.567038536 |
| H2AC4 | H2A Clustered Histone 4 | Protein Coding | 28 | GC06M026034 | 3.566936731 |
| MAP3K14 | Mitogen-Activated Protein Kinase Kinase Kinase 14 | Protein Coding | 39 | GC17M045263 | 3.566524029 |
| S100A10 | S100 Calcium Binding Protein A10 | Protein Coding | 39 | GC01M151955 | 3.564526558 |
| MIR302D | MicroRNA 302d | RNA Gene | 20 | GC04M112648 | 3.563422203 |
| CCNL1 | Cyclin L1 | Protein Coding | 34 | GC03M157146 | 3.563416004 |
| WASF1 | WASP Family Member 1 | Protein Coding | 40 | GC06M110099 | 3.561873674 |
| SCNN1A | Sodium Channel Epithelial 1 Subunit Alpha | Protein Coding | 43 | GC12M006346 | 3.56078887 |
| PTPRU | Protein Tyrosine Phosphatase Receptor Type U | Protein Coding | 39 | GC01P029236 | 3.560406923 |
| LOXL4 | Lysyl Oxidase Like 4 | Protein Coding | 39 | GC10M098247 | 3.559153557 |
| FBXO4 | F-Box Protein 4 | Protein Coding | 32 | GC05P042000 | 3.558928251 |
| ADCY5 | Adenylate Cyclase 5 | Protein Coding | 44 | GC03M123282 | 3.557750225 |
| MYLK-AS1 | MYLK Antisense RNA 1 | RNA Gene | 17 | GC03P123588 | 3.557296276 |
| SLFN12 | Schlafen Family Member 12 | Protein Coding | 29 | GC17M035556 | 3.556241035 |
| CARD14 | Caspase Recruitment Domain Family Member 14 | Protein Coding | 38 | GC17P080170 | 3.555615902 |
| TEAD4 | TEA Domain Transcription Factor 4 | Protein Coding | 36 | GC12P002959 | 3.555413008 |
| RIOK1 | RIO Kinase 1 | Protein Coding | 35 | GC06P007389 | 3.555054665 |
| HSPA1L | Heat Shock Protein Family A (Hsp70) Member 1 Like | Protein Coding | 40 | GC06M031809 | 3.555019855 |
| PFKM | Phosphofructokinase, Muscle | Protein Coding | 44 | GC12P048105 | 3.554563046 |
| CCT4 | Chaperonin Containing TCP1 Subunit 4 | Protein Coding | 36 | GC02M061868 | 3.554235935 |
| SDC4 | Syndecan 4 | Protein Coding | 39 | GC20M045325 | 3.55389452 |
| DSG1 | Desmoglein 1 | Protein Coding | 39 | GC18P031318 | 3.553836346 |
| CEP152 | Centrosomal Protein 152 | Protein Coding | 34 | GC15M048663 | 3.553765774 |
| SMYD2 | SET And MYND Domain Containing 2 | Protein Coding | 38 | GC01P214281 | 3.553446531 |
| ARRB2 | Arrestin Beta 2 | Protein Coding | 41 | GC17P004711 | 3.553401947 |
| NRG3 | Neuregulin 3 | Protein Coding | 38 | GC10P091448 | 3.551903725 |
| CSNK1A1L | Casein Kinase 1 Alpha 1 Like | Protein Coding | 32 | GC13M037103 | 3.551617146 |
| HSPA1B | Heat Shock Protein Family A (Hsp70) Member 1B | Protein Coding | 36 | GC06P080372 | 3.551552296 |
| AIF1 | Allograft Inflammatory Factor 1 | Protein Coding | 34 | GC06P080352 | 3.550629139 |
| CFL2 | Cofilin 2 | Protein Coding | 39 | GC14M034706 | 3.550364494 |
| PRPF6 | Pre-MRNA Processing Factor 6 | Protein Coding | 36 | GC20P063981 | 3.545448065 |
| GNA12 | G Protein Subunit Alpha 12 | Protein Coding | 37 | GC07M002728 | 3.545170546 |
| ETFA | Electron Transfer Flavoprotein Subunit Alpha | Protein Coding | 41 | GC15M081540 | 3.544822693 |
| FOXRED1 | FAD Dependent Oxidoreductase Domain Containing 1 | Protein Coding | 36 | GC11P126269 | 3.544518471 |
| PPY | Pancreatic Polypeptide | Protein Coding | 36 | GC17M043940 | 3.543777466 |
| UBA52 | Ubiquitin A-52 Residue Ribosomal Protein Fusion Product 1 | Protein Coding | 36 | GC19P018563 | 3.543228626 |
| COL5A2 | Collagen Type V Alpha 2 Chain | Protein Coding | 39 | GC02M189031 | 3.542736053 |
| DUOX2 | Dual Oxidase 2 | Protein Coding | 40 | GC15M045092 | 3.542663813 |
| HCK | HCK Proto-Oncogene, Src Family Tyrosine Kinase | Protein Coding | 43 | GC20P032052 | 3.542215586 |
| SLC4A1 | Solute Carrier Family 4 Member 1 (Diego Blood Group) | Protein Coding | 44 | GC17M044847 | 3.540250063 |
| BOC | BOC Cell Adhesion Associated, Oncogene Regulated | Protein Coding | 36 | GC03P113211 | 3.53965044 |
| SF3B4 | Splicing Factor 3b Subunit 4 | Protein Coding | 39 | GC01M149923 | 3.53886652 |
| PPM1F | Protein Phosphatase, Mg2+/Mn2+ Dependent 1F | Protein Coding | 37 | GC22M021919 | 3.538753986 |
| RAB18 | RAB18, Member RAS Oncogene Family | Protein Coding | 35 | GC10P027504 | 3.538700342 |
| NID2 | Nidogen 2 | Protein Coding | 35 | GC14M052004 | 3.537167072 |
| EOMES | Eomesodermin | Protein Coding | 39 | GC03M027715 | 3.536060333 |
| PTPRA | Protein Tyrosine Phosphatase Receptor Type A | Protein Coding | 41 | GC20P002864 | 3.535796642 |
| MARK3 | Microtubule Affinity Regulating Kinase 3 | Protein Coding | 44 | GC14P103385 | 3.535139084 |
| SLC45A2 | Solute Carrier Family 45 Member 2 | Protein Coding | 35 | GC05M033944 | 3.53431201 |
| ARF6 | ADP Ribosylation Factor 6 | Protein Coding | 39 | GC14P049895 | 3.533871651 |
| RPL7A | Ribosomal Protein L7a | Protein Coding | 38 | GC09P133348 | 3.533854008 |
| SESN2 | Sestrin 2 | Protein Coding | 34 | GC01P028270 | 3.533334494 |
| MED23 | Mediator Complex Subunit 23 | Protein Coding | 38 | GC06M131573 | 3.531401396 |
| HAS1 | Hyaluronan Synthase 1 | Protein Coding | 35 | GC19M064254 | 3.531349659 |
| CRY1 | Cryptochrome Circadian Regulator 1 | Protein Coding | 40 | GC12M106991 | 3.530887604 |
| LINC00665 | Long Intergenic Non-Protein Coding RNA 665 | RNA Gene | 15 | GC19M064771 | 3.530272007 |
| TRH | Thyrotropin Releasing Hormone | Protein Coding | 38 | GC03P129974 | 3.530252934 |
| HFE-AS1 | HFE Antisense RNA 1 | RNA Gene | 9 | GC06M026085 | 3.527951241 |
| CALM2 | Calmodulin 2 | Protein Coding | 40 | GC02M047124 | 3.527310133 |
| PPP1R15A | Protein Phosphatase 1 Regulatory Subunit 15A | Protein Coding | 35 | GC19P048872 | 3.527208328 |
| C9orf72 | C9orf72-SMCR8 Complex Subunit | Protein Coding | 36 | GC09M028240 | 3.527053356 |
| SOX18 | SRY-Box Transcription Factor 18 | Protein Coding | 36 | GC20M064047 | 3.526587486 |
| PPP2R5D | Protein Phosphatase 2 Regulatory Subunit B'Delta | Protein Coding | 40 | GC06P080594 | 3.526432514 |
| UBE2D3 | Ubiquitin Conjugating Enzyme E2 D3 | Protein Coding | 40 | GC04M102794 | 3.525425434 |
| DCAF17 | DDB1 And CUL4 Associated Factor 17 | Protein Coding | 34 | GC02P171434 | 3.525131702 |

| | | | | | |
|---|---|---|---|---|---|
| KLHL7 | Kelch Like Family Member 7 | Protein Coding | 36 | GC07P023105 | 3.524590731 |
| LIM2-AS1 | LIM2 And SIGLEC10 Antisense RNA 1 | RNA Gene | 9 | GC19P064173 | 3.523923159 |
| PHC3 | Polyhomeotic Homolog 3 | Protein Coding | 34 | GC03M170086 | 3.523247242 |
| UBE4B | Ubiquitination Factor E4B | Protein Coding | 36 | GC01P010032 | 3.522761583 |
| S1PR1 | Sphingosine-1-Phosphate Receptor 1 | Protein Coding | 39 | GC01P101236 | 3.522312164 |
| CCT7 | Chaperonin Containing TCP1 Subunit 7 | Protein Coding | 37 | GC02P073233 | 3.520041466 |
| MYO9B | Myosin IXB | Protein Coding | 40 | GC19P063097 | 3.519849777 |
| ARHGAP45 | Rho GTPase Activating Protein 45 | Protein Coding | 30 | GC19P001065 | 3.519520044 |
| TSNAX-DISC1 | TSNAX-DISC1 Readthrough (NMD Candidate) | RNA Gene | 16 | GC01P231528 | 3.518186808 |
| COPA | COPI Coat Complex Subunit Alpha | Protein Coding | 38 | GC01M160288 | 3.517347813 |
| ACTN1 | Actinin Alpha 1 | Protein Coding | 44 | GC14M068874 | 3.517323494 |
| LTBP1 | Latent Transforming Growth Factor Beta Binding Protein 1 | Protein Coding | 41 | GC02P033076 | 3.516556263 |
| CFHR1 | Complement Factor H Related 1 | Protein Coding | 39 | GC01P196822 | 3.515022278 |
| MIR216B | MicroRNA 216b | RNA Gene | 17 | GC02M056000 | 3.51357317 |
| PPM1E | Protein Phosphatase, Mg2+/Mn2+ Dependent 1E | Protein Coding | 33 | GC17P058756 | 3.512541771 |
| RANBP1 | RAN Binding Protein 1 | Protein Coding | 38 | GC22P020115 | 3.510804653 |
| DOK1 | Docking Protein 1 | Protein Coding | 38 | GC02P074549 | 3.510188341 |
| USP22 | Ubiquitin Specific Peptidase 22 | Protein Coding | 36 | GC17M020999 | 3.509346485 |
| EFEMP2 | EGF Containing Fibulin Extracellular Matrix Protein 2 | Protein Coding | 41 | GC11M087300 | 3.508619308 |
| RAP2A | RAP2A, Member Of RAS Oncogene Family | Protein Coding | 36 | GC13P097436 | 3.507924795 |
| CRTC3 | CREB Regulated Transcription Coactivator 3 | Protein Coding | 35 | GC15P090529 | 3.507787228 |
| SETD1A | SET Domain Containing 1A, Histone Lysine Methyltransferase | Protein Coding | 37 | GC16P040282 | 3.507038116 |
| SDF4 | Stromal Cell Derived Factor 4 | Protein Coding | 34 | GC01M001216 | 3.506546497 |
| ZIC2 | Zic Family Member 2 | Protein Coding | 38 | GC13P099981 | 3.506286144 |
| RPRD1A | Regulation Of Nuclear Pre-MRNA Domain Containing 1A | Protein Coding | 32 | GC18M035984 | 3.50618577 |
| MASP1 | MBL Associated Serine Protease 1 | Protein Coding | 44 | GC03M187216 | 3.505939007 |
| SETD5 | SET Domain Containing 5 | Protein Coding | 34 | GC03P009402 | 3.505837917 |
| RRP9 | Ribosomal RNA Processing 9, U3 Small Nucleolar RNA Binding Protein | Protein Coding | 30 | GC03M051943 | 3.505824327 |
| STXBP2 | Syntaxin Binding Protein 2 | Protein Coding | 40 | GC19P007820 | 3.505563021 |
| ENTPD1-AS1 | ENTPD1 Antisense RNA 1 | RNA Gene | 15 | GC10M095753 | 3.505456448 |
| UQCC2 | Ubiquinol-Cytochrome C Reductase Complex Assembly Factor 2 | Protein Coding | 31 | GC06M033694 | 3.505217552 |
| PTPN7 | Protein Tyrosine Phosphatase Non-Receptor Type 7 | Protein Coding | 39 | GC01M202147 | 3.504759312 |
| MIB2 | MIB E3 Ubiquitin Protein Ligase 2 | Protein Coding | 34 | GC01P003630 | 3.502350807 |
| UCP2 | Uncoupling Protein 2 | Protein Coding | 41 | GC11M073974 | 3.501392841 |
| GHSR | Growth Hormone Secretagogue Receptor | Protein Coding | 42 | GC03M172443 | 3.500982285 |
| BCAT1 | Branched Chain Amino Acid Transaminase 1 | Protein Coding | 41 | GC12M024732 | 3.500741959 |
| BOD1L1 | Biorientation Of Chromosomes In Cell Division 1 Like 1 | Protein Coding | 28 | GC04M013571 | 3.500266075 |
| HES5 | Hes Family BHLH Transcription Factor 5 | Protein Coding | 32 | GC01M002528 | 3.499127865 |
| OLR1 | Oxidized Low Density Lipoprotein Receptor 1 | Protein Coding | 38 | GC12M020441 | 3.49870491 |
| SRCIN1 | SRC Kinase Signaling Inhibitor 1 | Protein Coding | 31 | GC17M038530 | 3.497154951 |
| RNF182 | Ring Finger Protein 182 | Protein Coding | 32 | GC06P013924 | 3.495691776 |
| GANAB | Glucosidase II Alpha Subunit | Protein Coding | 42 | GC11M087128 | 3.494862556 |
| LGALS2 | Galectin 2 | Protein Coding | 36 | GC22M037570 | 3.494688988 |
| ARCN1 | Archain 1 | Protein Coding | 38 | GC11P118572 | 3.494609356 |
| TICAM1 | Toll Like Receptor Adaptor Molecule 1 | Protein Coding | 39 | GC19M004815 | 3.494026184 |
| H1-5 | H1.5 Linker Histone, Cluster Member | Protein Coding | 33 | GC06M064152 | 3.49397707 |
| USP17L2 | Ubiquitin Specific Peptidase 17 Like Family Member 2 | Protein Coding | 26 | GC08M012638 | 3.493216515 |
| RPS12 | Ribosomal Protein S12 | Protein Coding | 37 | GC06P132814 | 3.492677927 |
| USP14 | Ubiquitin Specific Peptidase 14 | Protein Coding | 41 | GC18P000158 | 3.492447853 |
| MIR520H | MicroRNA 520h | RNA Gene | 18 | GC19P064307 | 3.492191076 |
| EPPK1 | Epiplakin 1 | Protein Coding | 30 | GC08M143857 | 3.492102385 |
| PITX1 | Paired Like Homeodomain 1 | Protein Coding | 41 | GC05M135027 | 3.490223408 |
| COL6A3 | Collagen Type VI Alpha 3 Chain | Protein Coding | 40 | GC02M237324 | 3.489201307 |
| RBBP5 | RB Binding Protein 5, Histone Lysine Methyltransferase Complex Subunit | Protein Coding | 34 | GC01M205055 | 3.488726854 |
| BMP5 | Bone Morphogenetic Protein 5 | Protein Coding | 38 | GC06M055753 | 3.488489628 |
| SLC10A2 | Solute Carrier Family 10 Member 2 | Protein Coding | 40 | GC13M103043 | 3.487535954 |
| SLC25A5 | Solute Carrier Family 25 Member 5 | Protein Coding | 39 | GC0XP119468 | 3.487471819 |
| PHF8 | PHD Finger Protein 8 | Protein Coding | 38 | GC0XM053936 | 3.487307072 |
| ARAP3 | ArfGAP With RhoGAP Domain, Ankyrin Repeat And PH Domain 3 | Protein Coding | 35 | GC05M141653 | 3.485301971 |
| MLF2 | Myeloid Leukemia Factor 2 | Protein Coding | 32 | GC12M006885 | 3.48511982 |
| DIP2C | Disco Interacting Protein 2 Homolog C | Protein Coding | 30 | GC10M000274 | 3.48494482 |
| RAB11FIP4 | RAB11 Family Interacting Protein 4 | Protein Coding | 33 | GC17P031391 | 3.484731674 |
| TOLLIP | Toll Interacting Protein | Protein Coding | 39 | GC11M001274 | 3.484181404 |
| SLC25A24 | Solute Carrier Family 25 Member 24 | Protein Coding | 39 | GC01M108134 | 3.483990908 |
| DST | Dystonin | Protein Coding | 38 | GC06M056457 | 3.483851194 |
| NCK1 | NCK Adaptor Protein 1 | Protein Coding | 39 | GC03P136862 | 3.483795881 |
| STARD3 | StAR Related Lipid Transfer Domain Containing 3 | Protein Coding | 35 | GC17P039637 | 3.482721329 |
| TRIM14 | Tripartite Motif Containing 14 | Protein Coding | 34 | GC09M098966 | 3.48171258 |
| SEPTIN2 | Septin 2 | Protein Coding | 31 | GC02P241316 | 3.478033781 |
| CHD3 | Chromodomain Helicase DNA Binding Protein 3 | Protein Coding | 38 | GC17P010899 | 3.476783276 |
| DNAJC13 | DnaJ Heat Shock Protein Family (Hsp40) Member C13 | Protein Coding | 35 | GC03P132417 | 3.476600647 |
| RPL8 | Ribosomal Protein L8 | Protein Coding | 36 | GC08M145378 | 3.475677967 |
| GFI1 | Growth Factor Independent 1 Transcriptional Repressor | Protein Coding | 39 | GC01M092474 | 3.475327492 |
| PTPRO | Protein Tyrosine Phosphatase Receptor Type O | Protein Coding | 42 | GC12P020190 | 3.475177526 |
| RAD9A | RAD9 Checkpoint Clamp Component A | Protein Coding | 36 | GC11P069707 | 3.474155188 |
| FBLN5 | Fibulin 5 | Protein Coding | 42 | GC14M091869 | 3.47395134 |
| MT-CO3 | Mitochondrially Encoded Cytochrome C Oxidase III | Protein Coding | 28 | GCMTP009209 | 3.473758698 |
| PLA2G7 | Phospholipase A2 Group VII | Protein Coding | 44 | GC06M063898 | 3.47351265 |
| NLRP8 | NLR Family Pyrin Domain Containing 8 | Protein Coding | 30 | GC19P064387 | 3.473008394 |
| ADAMTS18 | ADAM Metallopeptidase With Thrombospondin Type 1 Motif 18 | Protein Coding | 40 | GC16M077316 | 3.472668648 |
| ABCF2 | ATP Binding Cassette Subfamily F Member 2 | Protein Coding | 35 | GC07M151211 | 3.472610474 |
| LY75 | Lymphocyte Antigen 75 | Protein Coding | 36 | GC02M159803 | 3.472055435 |
| WDR19 | WD Repeat Domain 19 | Protein Coding | 34 | GC04P039184 | 3.471627474 |
| RFX1 | Regulatory Factor X1 | Protein Coding | 35 | GC19M013961 | 3.470996141 |
| HSD17B8 | Hydroxysteroid 17-Beta Dehydrogenase 8 | Protein Coding | 36 | GC06P080415 | 3.470710754 |
| CCL19 | C-C Motif Chemokine Ligand 19 | Protein Coding | 36 | GC09M034694 | 3.468977451 |
| SH3RF3-AS1 | SH3RF3 Antisense RNA 1 | RNA Gene | 13 | GC02M109127 | 3.467926025 |
| LINC01097 | Long Intergenic Non-Protein Coding RNA 1097 | RNA Gene | 12 | GC04M013529 | 3.467926025 |
| DRD1 | Dopamine Receptor D1 | Protein Coding | 42 | GC05M175440 | 3.467402697 |
| CSMD3 | CUB And Sushi Multiple Domains 3 | Protein Coding | 32 | GC08M112223 | 3.466950893 |
| RPS4X | Ribosomal Protein S4 X-Linked | Protein Coding | 34 | GC0XM072255 | 3.466651201 |
| NEK6 | NIMA Related Kinase 6 | Protein Coding | 35 | GC09P124259 | 3.466502666 |
| STK36 | Serine/Threonine Kinase 36 | Protein Coding | 40 | GC02P218672 | 3.464898825 |
| CBX5 | Chromobox 5 | Protein Coding | 39 | GC12M054230 | 3.464599848 |
| MICAL2 | Microtubule Associated Monooxygenase, Calponin And LIM Domain Containing 2 | Protein Coding | 34 | GC11P012115 | 3.464148521 |

| | | | | | |
|---|---|---|---|---|---|
| LEPROTL1 | Leptin Receptor Overlapping Transcript Like 1 | Protein Coding | 31 | GC08P030095 | 3.462983131 |
| AHRR | Aryl Hydrocarbon Receptor Repressor | Protein Coding | 31 | GC05P000321 | 3.462656975 |
| EIF2AK4 | Eukaryotic Translation Initiation Factor 2 Alpha Kinase 4 | Protein Coding | 41 | GC15P039934 | 3.461089373 |
| ERAL1 | Era Like 12S Mitochondrial RRNA Chaperone 1 | Protein Coding | 35 | GC17P052520 | 3.459642172 |
| UBA1 | Ubiquitin Like Modifier Activating Enzyme 1 | Protein Coding | 41 | GC0XP047190 | 3.45962286 |
| DCAF1 | DDB1 And CUL4 Associated Factor 1 | Protein Coding | 30 | GC03M051395 | 3.45862937 |
| CXCL6 | C-X-C Motif Chemokine Ligand 6 | Protein Coding | 35 | GC04P073837 | 3.457868338 |
| KIF16B | Kinesin Family Member 16B | Protein Coding | 33 | GC20M016272 | 3.456816196 |
| LINC02461 | Long Intergenic Non-Protein Coding RNA 2461 | RNA Gene | 11 | GC12P043067 | 3.456529856 |
| MSH5 | MutS Homolog 5 | Protein Coding | 37 | GC06P080368 | 3.456377506 |
| PYROXD1 | Pyridine Nucleotide-Disulphide Oxidoreductase Domain 1 | Protein Coding | 32 | GC12P021437 | 3.456002712 |
| CSGALNACT1 | Chondroitin Sulfate N-Acetylgalactosaminyltransferase 1 | Protein Coding | 36 | GC08M019404 | 3.455851078 |
| HSPA6 | Heat Shock Protein Family A (Hsp70) Member 6 | Protein Coding | 40 | GC01P161524 | 3.455846786 |
| ZFP57 | ZFP57 Zinc Finger Protein | Protein Coding | 35 | GC06M029672 | 3.45555687 |
| PARP4 | Poly(ADP-Ribose) Polymerase Family Member 4 | Protein Coding | 38 | GC13M024420 | 3.454987049 |
| ANTXR2 | ANTXR Cell Adhesion Molecule 2 | Protein Coding | 42 | GC04M079901 | 3.454664707 |
| MIR410 | MicroRNA 410 | RNA Gene | 17 | GC14P109534 | 3.454568624 |
| RPGRIP1L | RPGRIP1 Like | Protein Coding | 35 | GC16M053597 | 3.454510212 |
| EGR2 | Early Growth Response 2 | Protein Coding | 42 | GC10M062811 | 3.454180241 |
| KRT81 | Keratin 81 | Protein Coding | 35 | GC12M052286 | 3.453403473 |
| GPHN | Gephyrin | Protein Coding | 43 | GC14P066507 | 3.451034546 |
| NMI | N-Myc And STAT Interactor | Protein Coding | 34 | GC02M151270 | 3.450870514 |
| WAC | WW Domain Containing Adaptor With Coiled-Coil | Protein Coding | 35 | GC10P028557 | 3.450513601 |
| PDCD10 | Programmed Cell Death 10 | Protein Coding | 38 | GC03M167683 | 3.448936701 |
| TMSB10 | Thymosin Beta 10 | Protein Coding | 33 | GC02P084905 | 3.44835043 |
| PPARGC1B | PPARG Coactivator 1 Beta | Protein Coding | 34 | GC05P149730 | 3.447161913 |
| JAM3 | Junctional Adhesion Molecule 3 | Protein Coding | 39 | GC11P134068 | 3.446932316 |
| ARHGAP35 | Rho GTPase Activating Protein 35 | Protein Coding | 34 | GC19P046860 | 3.446645737 |
| PDLIM7 | PDZ And LIM Domain 7 | Protein Coding | 36 | GC05M177483 | 3.444470882 |
| CPT1A | Carnitine Palmitoyltransferase 1A | Protein Coding | 43 | GC11M068754 | 3.444434166 |
| LINC01856 | Long Intergenic Non-Protein Coding RNA 1856 | RNA Gene | 10 | GC02P129924 | 3.443011999 |
| PFKL | Phosphofructokinase, Liver Type | Protein Coding | 40 | GC21P044300 | 3.442947388 |
| DSG3 | Desmoglein 3 | Protein Coding | 37 | GC18P031447 | 3.442946911 |
| QKI | QKI, KH Domain Containing RNA Binding | Protein Coding | 38 | GC06P163414 | 3.44268322 |
| ADRA1B | Adrenoceptor Alpha 1B | Protein Coding | 42 | GC05P159867 | 3.441527605 |
| QSOX1 | Quiescin Sulfhydryl Oxidase 1 | Protein Coding | 35 | GC01P180154 | 3.440714359 |
| SCO2 | Synthesis Of Cytochrome C Oxidase 2 | Protein Coding | 40 | GC22M050523 | 3.440517426 |
| CBX8 | Chromobox 8 | Protein Coding | 34 | GC17M079794 | 3.440457582 |
| PTPRR | Protein Tyrosine Phosphatase Receptor Type R | Protein Coding | 37 | GC12M070638 | 3.439804554 |
| KPNA1 | Karyopherin Subunit Alpha 1 | Protein Coding | 36 | GC03M122421 | 3.439391375 |
| GRIP1 | Glutamate Receptor Interacting Protein 1 | Protein Coding | 38 | GC12M066347 | 3.437602043 |
| TALDO1 | Transaldolase 1 | Protein Coding | 42 | GC11P001672 | 3.43717885 |
| SURF4 | Surfeit 4 | Protein Coding | 34 | GC09M133361 | 3.436796904 |
| RPL3 | Ribosomal Protein L3 | Protein Coding | 36 | GC22M056106 | 3.436434746 |
| BANF1 | BAF Nuclear Assembly Factor 1 | Protein Coding | 38 | GC11P066002 | 3.436403275 |
| CENPE | Centromere Protein E | Protein Coding | 40 | GC04M103105 | 3.435665846 |
| NME8 | NME/NM23 Family Member 8 | Protein Coding | 35 | GC07P037889 | 3.433764219 |
| KCNK9 | Potassium Two Pore Domain Channel Subfamily K Member 9 | Protein Coding | 43 | GC08M139585 | 3.432832003 |
| MYT1 | Myelin Transcription Factor 1 | Protein Coding | 36 | GC20P064986 | 3.432725906 |
| SCNN1G | Sodium Channel Epithelial 1 Subunit Gamma | Protein Coding | 43 | GC16P023182 | 3.432428598 |
| CAPN2 | Calpain 2 | Protein Coding | 42 | GC01P223701 | 3.431907654 |
| RUVBL2 | RuvB Like AAA ATPase 2 | Protein Coding | 39 | GC19P048993 | 3.429640532 |
| SLC22A3 | Solute Carrier Family 22 Member 3 | Protein Coding | 40 | GC06P160348 | 3.429124832 |
| BOK | BCL2 Family Apoptosis Regulator BOK | Protein Coding | 35 | GC02P241558 | 3.419725895 |
| SLC6A14 | Solute Carrier Family 6 Member 14 | Protein Coding | 37 | GC0XP116436 | 3.417843103 |
| ESRP1 | Epithelial Splicing Regulatory Protein 1 | Protein Coding | 33 | GC08P094831 | 3.416340828 |
| KPNB1 | Karyopherin Subunit Beta 1 | Protein Coding | 38 | GC17P047649 | 3.416302681 |
| PRKX | Protein Kinase X-Linked | Protein Coding | 38 | GC0XM003604 | 3.416110039 |
| CCT2 | Chaperonin Containing TCP1 Subunit 2 | Protein Coding | 36 | GC12P069585 | 3.415890455 |
| FGF20 | Fibroblast Growth Factor 20 | Protein Coding | 36 | GC08M016992 | 3.415766716 |
| RPS2 | Ribosomal Protein S2 | Protein Coding | 37 | GC16M006682 | 3.414138794 |
| PTOV1 | PTOV1 Extended AT-Hook Containing Adaptor Protein | Protein Coding | 34 | GC19P064095 | 3.412050724 |
| GAD2 | Glutamate Decarboxylase 2 | Protein Coding | 39 | GC10P026216 | 3.411441326 |
| KLK1 | Kallikrein 1 | Protein Coding | 42 | GC19M050819 | 3.411324739 |
| MPZ | Myelin Protein Zero | Protein Coding | 40 | GC01M161304 | 3.410996437 |
| DAZL | Deleted In Azoospermia Like | Protein Coding | 36 | GC03M016586 | 3.410811663 |
| ATF6 | Activating Transcription Factor 6 | Protein Coding | 42 | GC01P161766 | 3.410788298 |
| RBM19 | RNA Binding Motif Protein 19 | Protein Coding | 31 | GC12M113816 | 3.41069746 |
| RPL28 | Ribosomal Protein L28 | Protein Coding | 35 | GC19P064363 | 3.410673618 |
| DLX6-AS1 | DLX6 Antisense RNA 1 | RNA Gene | 20 | GC07M096969 | 3.41027832 |
| DPP3 | Dipeptidyl Peptidase 3 | Protein Coding | 36 | GC11P069643 | 3.409979582 |
| GRIK3 | Glutamate Ionotropic Receptor Kainate Type Subunit 3 | Protein Coding | 39 | GC01M036795 | 3.409580946 |
| DEPDC5 | DEP Domain Containing 5, GATOR1 Subcomplex Subunit | Protein Coding | 35 | GC22P031753 | 3.409259558 |
| DOCK6 | Dedicator Of Cytokinesis 6 | Protein Coding | 36 | GC19M011199 | 3.409142733 |
| PRKACG | Protein Kinase CAMP-Activated Catalytic Subunit Gamma | Protein Coding | 43 | GC09M069027 | 3.408956289 |
| ALDOB | Aldolase, Fructose-Bisphosphate B | Protein Coding | 40 | GC09M101420 | 3.408952713 |
| IL13RA1 | Interleukin 13 Receptor Subunit Alpha 1 | Protein Coding | 36 | GC0XP118727 | 3.408875465 |
| ABO | ABO, Alpha 1-3-N-Acetylgalactosaminyltransferase And Alpha 1-3-Galactosyltransferase | Protein Coding | 34 | GC09M133250 | 3.408842325 |
| RNF31 | Ring Finger Protein 31 | Protein Coding | 36 | GC14P024146 | 3.408062935 |
| ATP5F1B | ATP Synthase F1 Subunit Beta | Protein Coding | 33 | GC12M056639 | 3.407731533 |
| GOLGA5 | Golgin A5 | Protein Coding | 34 | GC14P092794 | 3.404994011 |
| KDM2B | Lysine Demethylase 2B | Protein Coding | 38 | GC12M121402 | 3.403453112 |
| NOL4 | Nucleolar Protein 4 | Protein Coding | 34 | GC18M033851 | 3.403431177 |
| PRDX4 | Peroxiredoxin 4 | Protein Coding | 38 | GC0XP023665 | 3.40307188 |
| DHCR24 | 24-Dehydrocholesterol Reductase | Protein Coding | 40 | GC01M054849 | 3.402556419 |
| VAMP7 | Vesicle Associated Membrane Protein 7 | Protein Coding | 36 | GC0XP155881 | 3.401736259 |
| YBX3 | Y-Box Binding Protein 3 | Protein Coding | 34 | GC12M020452 | 3.401207924 |
| IGHE | Immunoglobulin Heavy Constant Epsilon | Protein Coding | 26 | GC14M112401 | 3.40089035 |
| CAPN5 | Calpain 5 | Protein Coding | 39 | GC11P077066 | 3.399509668 |
| TMEM205 | Transmembrane Protein 205 | Protein Coding | 27 | GC19M011342 | 3.399082899 |
| ERLIN2 | ER Lipid Raft Associated 2 | Protein Coding | 37 | GC08P037736 | 3.398857117 |
| ANP32D | Acidic Nuclear Phosphoprotein 32 Family Member D | Protein Coding | 26 | GC12P048472 | 3.398380995 |
| CEP63 | Centrosomal Protein 63 | Protein Coding | 36 | GC03P134485 | 3.397951126 |
| NOP9 | NOP9 Nucleolar Protein | Protein Coding | 28 | GC14P024299 | 3.397663832 |

| | | | | | |
|---|---|---|---|---|---|
| MAPK8IP1 | Mitogen-Activated Protein Kinase 8 Interacting Protein 1 | Protein Coding | 40 | GC11P046454 | 3.397133827 |
| TCEANC2 | Transcription Elongation Factor A N-Terminal And Central Domain Containing 2 | Protein Coding | 28 | GC01P054053 | 3.396953106 |
| HCP5 | HLA Complex P5 | RNA Gene | 26 | GC06P031400 | 3.395922422 |
| ARHGEF39 | Rho Guanine Nucleotide Exchange Factor 39 | Protein Coding | 30 | GC09M035658 | 3.391544342 |
| GYPC | Glycophorin C (Gerbich Blood Group) | Protein Coding | 39 | GC02P126655 | 3.389768124 |
| ATP1A1 | ATPase Na+/K+ Transporting Subunit Alpha 1 | Protein Coding | 44 | GC01P116372 | 3.388814926 |
| NOB1 | NIN1 (RPN12) Binding Protein 1 Homolog | Protein Coding | 33 | GC16M070188 | 3.388434649 |
| PSMB4 | Proteasome 20S Subunit Beta 4 | Protein Coding | 39 | GC01P151372 | 3.387170315 |
| STYK1 | Serine/Threonine/Tyrosine Kinase 1 | Protein Coding | 37 | GC12M020451 | 3.38715601 |
| OGDHL | Oxoglutarate Dehydrogenase L | Protein Coding | 36 | GC10M049734 | 3.386868 |
| CAPN1 | Calpain 1 | Protein Coding | 44 | GC11P069519 | 3.385017872 |
| PRSS3 | Serine Protease 3 | Protein Coding | 39 | GC09P033750 | 3.383553982 |
| CHST3 | Carbohydrate Sulfotransferase 3 | Protein Coding | 39 | GC10P071964 | 3.383290529 |
| LGALS9 | Galectin 9 | Protein Coding | 35 | GC17P027629 | 3.38229394 |
| GABRA2 | Gamma-Aminobutyric Acid Type A Receptor Subunit Alpha2 | Protein Coding | 42 | GC04M046243 | 3.381641865 |
| PPP1R13B | Protein Phosphatase 1 Regulatory Subunit 13B | Protein Coding | 33 | GC14M103733 | 3.381552458 |
| JADE3 | Jade Family PHD Finger 3 | Protein Coding | 27 | GC0XP046912 | 3.380885124 |
| FMNL1 | Formin Like 1 | Protein Coding | 34 | GC17P053268 | 3.380661249 |
| HHIP | Hedgehog Interacting Protein | Protein Coding | 37 | GC04P144645 | 3.3801651 |
| SNIP1 | Smad Nuclear Interacting Protein 1 | Protein Coding | 34 | GC01M037534 | 3.379301548 |
| YWHAH | Tyrosine 3-Monooxygenase/Tryptophan 5-Monooxygenase Activation Protein Eta | Protein Coding | 42 | GC22P031944 | 3.379177809 |
| TFDP2 | Transcription Factor Dp-2 | Protein Coding | 34 | GC03M141944 | 3.378367662 |
| AP2B1 | Adaptor Related Protein Complex 2 Subunit Beta 1 | Protein Coding | 37 | GC17P035578 | 3.377966404 |
| H4C15 | H4 Clustered Histone 15 | Protein Coding | 24 | GC01M151586 | 3.377905369 |
| CELF2 | CUGBP Elav-Like Family Member 2 | Protein Coding | 39 | GC10P010462 | 3.3768363 |
| SDC3 | Syndecan 3 | Protein Coding | 38 | GC01M030869 | 3.376046419 |
| POFUT1 | Protein O-Fucosyltransferase 1 | Protein Coding | 40 | GC20P032207 | 3.375099182 |
| OIP5-AS1 | OIP5 Antisense RNA 1 | RNA Gene | 17 | GC15P041300 | 3.374912739 |
| NUDT10 | Nudix Hydrolase 10 | Protein Coding | 30 | GC0XP051332 | 3.37364769 |
| TEP1 | Telomerase Associated Protein 1 | Protein Coding | 36 | GC14M020365 | 3.371824026 |
| SMS | Spermine Synthase | Protein Coding | 39 | GC0XP021958 | 3.371798515 |
| STXBP4 | Syntaxin Binding Protein 4 | Protein Coding | 33 | GC17P054968 | 3.371604919 |
| SPECC1 | Sperm Antigen With Calponin Homology And Coiled-Coil Domains 1 | Protein Coding | 30 | GC17P052335 | 3.371280909 |
| NME2 | NME/NM23 Nucleoside Diphosphate Kinase 2 | Protein Coding | 42 | GC17P051165 | 3.369747162 |
| SPRR1A | Small Proline Rich Protein 1A | Protein Coding | 32 | GC01P152984 | 3.368835211 |
| SEM1 | SEM1 26S Proteasome Subunit | Protein Coding | 31 | GC07M096483 | 3.368650913 |
| OTUB1 | OTU Deubiquitinase, Ubiquitin Aldehyde Binding 1 | Protein Coding | 35 | GC11P063985 | 3.367837906 |
| PSMC4 | Proteasome 26S Subunit, ATPase 4 | Protein Coding | 36 | GC19P063589 | 3.367750406 |
| FERMT2 | FERM Domain Containing Kindlin 2 | Protein Coding | 35 | GC14M052857 | 3.367554903 |
| PRICKLE3 | Prickle Planar Cell Polarity Protein 3 | Protein Coding | 31 | GC0XM049222 | 3.367022276 |
| ADM5 | Adrenomedullin 5 (Putative) | Protein Coding | 19 | GC19P064075 | 3.366446733 |
| GET3 | Guided Entry Of Tail-Anchored Proteins Factor 3, ATPase | Protein Coding | 32 | GC19P012737 | 3.365837574 |
| ADORA3 | Adenosine A3 Receptor | Protein Coding | 41 | GC01M111499 | 3.363676548 |
| IL10RA | Interleukin 10 Receptor Subunit Alpha | Protein Coding | 42 | GC11P117987 | 3.36303854 |
| POLR2F | RNA Polymerase II, I And III Subunit F | Protein Coding | 35 | GC22P037952 | 3.362873793 |
| GNG12 | G Protein Subunit Gamma 12 | Protein Coding | 34 | GC01M067701 | 3.361200094 |
| ISG20 | Interferon Stimulated Exonuclease Gene 20 | Protein Coding | 36 | GC15P088635 | 3.358908653 |
| FTL | Ferritin Light Chain | Protein Coding | 42 | GC19P048965 | 3.35811305 |
| ELK3 | ETS Transcription Factor ELK3 | Protein Coding | 34 | GC12P096194 | 3.357376337 |
| SHMT2 | Serine Hydroxymethyltransferase 2 | Protein Coding | 40 | GC12P057229 | 3.356343746 |
| GSTO1 | Glutathione S-Transferase Omega 1 | Protein Coding | 40 | GC10P104235 | 3.355869293 |
| HNRNPD | Heterogeneous Nuclear Ribonucleoprotein D | Protein Coding | 38 | GC04M082352 | 3.355384827 |
| PPFIA1 | PTPRF Interacting Protein Alpha 1 | Protein Coding | 34 | GC11P070270 | 3.355052471 |
| TAGLN2 | Transgelin 2 | Protein Coding | 36 | GC01M159918 | 3.354852676 |
| TBX22 | T-Box Transcription Factor 22 | Protein Coding | 38 | GC0XP080014 | 3.35229063 |
| CLASRP | CLK4 Associating Serine/Arginine Rich Protein | Protein Coding | 28 | GC19P045039 | 3.352275372 |
| CHP2 | Calcineurin Like EF-Hand Protein 2 | Protein Coding | 28 | GC16P024200 | 3.351319075 |
| C5AR1 | Complement C5a Receptor 1 | Protein Coding | 39 | GC19P047290 | 3.35125351 |
| F11R | F11 Receptor | Protein Coding | 38 | GC01M160995 | 3.351197004 |
| COL19A1 | Collagen Type XIX Alpha 1 Chain | Protein Coding | 35 | GC06P069866 | 3.351128101 |
| CYB5R4 | Cytochrome B5 Reductase 4 | Protein Coding | 36 | GC06P083859 | 3.351050854 |
| CPSF3 | Cleavage And Polyadenylation Specific Factor 3 | Protein Coding | 33 | GC02P009423 | 3.350803852 |
| SUGP1 | SURP And G-Patch Domain Containing 1 | Protein Coding | 31 | GC19M019276 | 3.349796772 |
| RPL22 | Ribosomal Protein L22 | Protein Coding | 38 | GC01M006179 | 3.347779989 |
| RPS13 | Ribosomal Protein S13 | Protein Coding | 37 | GC11M017543 | 3.347545385 |
| PSPH | Phosphoserine Phosphatase | Protein Coding | 41 | GC07M056010 | 3.346102476 |
| FAM131A | Family With Sequence Similarity 131 Member A | Protein Coding | 27 | GC03P184335 | 3.345436573 |
| BACE2 | Beta-Secretase 2 | Protein Coding | 37 | GC21P041168 | 3.345406532 |
| STK19 | Serine/Threonine Kinase 19 | Protein Coding | 35 | GC06P031971 | 3.344346046 |
| IVNS1ABP | Influenza Virus NS1A Binding Protein | Protein Coding | 36 | GC01M185307 | 3.341965675 |
| TOPORS | TOP1 Binding Arginine/Serine Rich Protein, E3 Ubiquitin Ligase | Protein Coding | 39 | GC09M032540 | 3.340660572 |
| BMP8A | Bone Morphogenetic Protein 8a | Protein Coding | 34 | GC01P039492 | 3.340529442 |
| TAFAZZIN | Tafazzin, Phospholipid-Lysophospholipid Transacylase | Protein Coding | 32 | GC0XP154413 | 3.340354919 |
| KLF2 | Kruppel Like Factor 2 | Protein Coding | 36 | GC19P063082 | 3.339887381 |
| CDK15 | Cyclin Dependent Kinase 15 | Protein Coding | 35 | GC02P201790 | 3.338341713 |
| TSGA10 | Testis Specific 10 | Protein Coding | 31 | GC02M098997 | 3.337901354 |
| CYP3A43 | Cytochrome P450 Family 3 Subfamily A Member 43 | Protein Coding | 38 | GC07P099829 | 3.337761402 |
| ING4 | Inhibitor Of Growth Family Member 4 | Protein Coding | 33 | GC12M006650 | 3.337465763 |
| ALAD | Aminolevulinate Dehydratase | Protein Coding | 42 | GC09M113386 | 3.33626461 |
| NFIC | Nuclear Factor I C | Protein Coding | 36 | GC19P003314 | 3.335147858 |
| ST3GAL1 | ST3 Beta-Galactoside Alpha-2,3-Sialyltransferase 1 | Protein Coding | 38 | GC08M133454 | 3.335078955 |
| LINC00707 | Long Intergenic Non-Protein Coding RNA 707 | RNA Gene | 15 | GC10P006780 | 3.334836483 |
| SP7 | Sp7 Transcription Factor | Protein Coding | 39 | GC12M053326 | 3.334079742 |
| MAT1A | Methionine Adenosyltransferase 1A | Protein Coding | 42 | GC10M080271 | 3.332602262 |
| TTLL3 | Tubulin Tyrosine Ligase Like 3 | Protein Coding | 32 | GC03P009808 | 3.331962347 |
| BBS4 | Bardet-Biedl Syndrome 4 | Protein Coding | 38 | GC15P072686 | 3.331342936 |
| TMEM161A | Transmembrane Protein 161A | Protein Coding | 31 | GC19M019120 | 3.33127284 |
| MIR9-3HG | MIR9-3 Host Gene | RNA Gene | 15 | GC15P115248 | 3.331184387 |
| GNB4 | G Protein Subunit Beta 4 | Protein Coding | 38 | GC03M179397 | 3.330995083 |
| TXN2 | Thioredoxin 2 | Protein Coding | 40 | GC22M036467 | 3.330299854 |
| IL27 | Interleukin 27 | Protein Coding | 33 | GC16M036280 | 3.330014467 |
| RFC5 | Replication Factor C Subunit 5 | Protein Coding | 35 | GC12P118013 | 3.32979846 |
| RNASEH2C | Ribonuclease H2 Subunit C | Protein Coding | 35 | GC11M065714 | 3.329282761 |
| MPP1 | MAGUK P55 Scaffold Protein 1 | Protein Coding | 35 | GC0XM154779 | 3.328615665 |
| FOXH1 | Forkhead Box H1 | Protein Coding | 39 | GC08M144473 | 3.327713013 |

| | | | | | |
|---|---|---|---|---|---|
| EIF3E | Eukaryotic Translation Initiation Factor 3 Subunit E | Protein Coding | 33 | GC08M108163 | 3.325230122 |
| CPNE7 | Copine 7 | Protein Coding | 33 | GC16P089575 | 3.324728489 |
| VASP | Vasodilator Stimulated Phosphoprotein | Protein Coding | 38 | GC19P063864 | 3.324448347 |
| PQBP1 | Polyglutamine Binding Protein 1 | Protein Coding | 35 | GC0XP048890 | 3.324151278 |
| UBAC2 | UBA Domain Containing 2 | Protein Coding | 34 | GC13P099200 | 3.324123621 |
| TCTN3 | Tectonic Family Member 3 | Protein Coding | 36 | GC10M095663 | 3.32238698 |
| DOP1A | DOP1 Leucine Zipper Like Protein A | Protein Coding | 25 | GC06P083076 | 3.322314978 |
| LUZP1 | Leucine Zipper Protein 1 | Protein Coding | 32 | GC01M023085 | 3.321275711 |
| CCNK | Cyclin K | Protein Coding | 39 | GC14P099483 | 3.320667744 |
| CEP78 | Centrosomal Protein 78 | Protein Coding | 32 | GC09P078236 | 3.319370985 |
| H4C14 | H4 Clustered Histone 14 | Protein Coding | 27 | GC01P149832 | 3.319323063 |
| TBX21 | T-Box Transcription Factor 21 | Protein Coding | 41 | GC17P047733 | 3.318357468 |
| SEMA4C | Semaphorin 4C | Protein Coding | 34 | GC02M096859 | 3.318357229 |
| NOA1 | Nitric Oxide Associated 1 | Protein Coding | 30 | GC04M056963 | 3.318116665 |
| CACNB2 | Calcium Voltage-Gated Channel Auxiliary Subunit Beta 2 | Protein Coding | 42 | GC10P018165 | 3.317284346 |
| ARHGAP31 | Rho GTPase Activating Protein 31 | Protein Coding | 36 | GC03P119294 | 3.317051888 |
| PUS10 | Pseudouridine Synthase 10 | Protein Coding | 31 | GC02M060940 | 3.315265656 |
| ITK | IL2 Inducible T Cell Kinase | Protein Coding | 47 | GC05P157158 | 3.314605951 |
| ACYP2 | Acylphosphatase 2 | Protein Coding | 36 | GC02P053970 | 3.314419985 |
| RPL10 | Ribosomal Protein L10 | Protein Coding | 40 | GC0XP154389 | 3.3139956 |
| NCF2 | Neutrophil Cytosolic Factor 2 | Protein Coding | 42 | GC01M183555 | 3.313894272 |
| UBAP2L | Ubiquitin Associated Protein 2 Like | Protein Coding | 33 | GC01P154219 | 3.313071251 |
| SLCO1A2 | Solute Carrier Organic Anion Transporter Family Member 1A2 | Protein Coding | 37 | GC12M021264 | 3.312925816 |
| RIPK4 | Receptor Interacting Serine/Threonine Kinase 4 | Protein Coding | 40 | GC21M041739 | 3.312775612 |
| DSC2 | Desmocollin 2 | Protein Coding | 41 | GC18M031058 | 3.311815262 |
| NRG2 | Neuregulin 2 | Protein Coding | 38 | GC05M139810 | 3.309884787 |
| USP39 | Ubiquitin Specific Peptidase 39 | Protein Coding | 32 | GC02P085789 | 3.309746027 |
| RREB1 | Ras Responsive Element Binding Protein 1 | Protein Coding | 39 | GC06P007107 | 3.309400082 |
| NAT10 | N-Acetyltransferase 10 | Protein Coding | 34 | GC11P034105 | 3.309385538 |
| NUCKS1 | Nuclear Casein Kinase And Cyclin Dependent Kinase Substrate 1 | Protein Coding | 33 | GC01M205712 | 3.308247328 |
| SPAG6 | Sperm Associated Antigen 6 | Protein Coding | 34 | GC10P022345 | 3.307235241 |
| CERT1 | Ceramide Transporter 1 | Protein Coding | 32 | GC05M075356 | 3.306408405 |
| ZFP64 | ZFP64 Zinc Finger Protein | Protein Coding | 33 | GC20M052051 | 3.306379318 |
| TUBA4A | Tubulin Alpha 4a | Protein Coding | 41 | GC02M219249 | 3.306211472 |
| MIB1 | MIB E3 Ubiquitin Protein Ligase 1 | Protein Coding | 40 | GC18P021704 | 3.306138754 |
| H4C12 | H4 Clustered Histone 12 | Protein Coding | 27 | GC06M064168 | 3.306034565 |
| KAT8 | Lysine Acetyltransferase 8 | Protein Coding | 37 | GC16P040296 | 3.305906296 |
| BEST1 | Bestrophin 1 | Protein Coding | 39 | GC11P061949 | 3.305801153 |
| MED11 | Mediator Complex Subunit 11 | Protein Coding | 28 | GC17P004731 | 3.305608511 |
| JPT2 | Jupiter Microtubule Associated Homolog 2 | Protein Coding | 25 | GC16P010687 | 3.305152893 |
| HOXA3 | Homeobox A3 | Protein Coding | 36 | GC07M027523 | 3.303684235 |
| L3MBTL1 | L3MBTL Histone Methyl-Lysine Binding Protein 1 | Protein Coding | 35 | GC20P043559 | 3.303424835 |
| LINC01568 | Long Intergenic Non-Protein Coding RNA 1568 | RNA Gene | 13 | GC16P073386 | 3.303258181 |
| H4C11 | H4 Clustered Histone 11 | Protein Coding | 27 | GC06P079945 | 3.302922487 |
| ACAN | Aggrecan | Protein Coding | 43 | GC15P115216 | 3.302583933 |
| ACTR2 | Actin Related Protein 2 | Protein Coding | 38 | GC02P065227 | 3.302360535 |
| MIR411 | MicroRNA 411 | RNA Gene | 14 | GC14P109535 | 3.301331043 |
| DOK7 | Docking Protein 7 | Protein Coding | 35 | GC04P003465 | 3.30081749 |
| PROM2 | Prominin 2 | Protein Coding | 32 | GC02P095274 | 3.300066233 |
| CHRNG | Cholinergic Receptor Nicotinic Gamma Subunit | Protein Coding | 37 | GC02P232539 | 3.299891472 |
| CELA1 | Chymotrypsin Like Elastase 1 | Protein Coding | 36 | GC12M051328 | 3.299703121 |
| BAZ1B | Bromodomain Adjacent To Zinc Finger Domain 1B | Protein Coding | 37 | GC07M073440 | 3.299631119 |
| PANK2 | Pantothenate Kinase 2 | Protein Coding | 39 | GC20P004013 | 3.298904896 |
| NPPB | Natriuretic Peptide B | Protein Coding | 39 | GC01M011858 | 3.298646688 |
| UQCRFS1 | Ubiquinol-Cytochrome C Reductase, Rieske Iron-Sulfur Polypeptide 1 | Protein Coding | 41 | GC19M029205 | 3.29856205 |
| CDON | Cell Adhesion Associated, Oncogene Regulated | Protein Coding | 40 | GC11M125955 | 3.29776001 |
| SPTBN2 | Spectrin Beta, Non-Erythrocytic 2 | Protein Coding | 38 | GC11M087360 | 3.297726631 |
| KIF1A | Kinesin Family Member 1A | Protein Coding | 38 | GC02M240713 | 3.296929359 |
| SMG1 | SMG1 Nonsense Mediated MRNA Decay Associated PI3K Related Kinase | Protein Coding | 38 | GC16M019197 | 3.296907663 |
| RIPK3 | Receptor Interacting Serine/Threonine Kinase 3 | Protein Coding | 38 | GC14M024336 | 3.294997931 |
| HNRNPL | Heterogeneous Nuclear Ribonucleoprotein L | Protein Coding | 35 | GC19M038836 | 3.292515755 |
| RNF2 | Ring Finger Protein 2 | Protein Coding | 40 | GC01P185045 | 3.291481972 |
| TBC1D4 | TBC1 Domain Family Member 4 | Protein Coding | 39 | GC13M075284 | 3.289624929 |
| YTHDF2 | YTH N6-Methyladenosine RNA Binding Protein 2 | Protein Coding | 34 | GC01P028770 | 3.289257765 |
| KCNIP3 | Potassium Voltage-Gated Channel Interacting Protein 3 | Protein Coding | 36 | GC02P095473 | 3.288188934 |
| INTS13 | Integrator Complex Subunit 13 | Protein Coding | 27 | GC12M026906 | 3.287307024 |
| H4C8 | H4 Clustered Histone 8 | Protein Coding | 27 | GC06M064172 | 3.287182331 |
| IL23A | Interleukin 23 Subunit Alpha | Protein Coding | 35 | GC12P057193 | 3.286087275 |
| OTOF | Otoferlin | Protein Coding | 38 | GC02M026458 | 3.284396648 |
| DDX58 | DExD/H-Box Helicase 58 | Protein Coding | 42 | GC09M032455 | 3.283842564 |
| SNRPG | Small Nuclear Ribonucleoprotein Polypeptide G | Protein Coding | 33 | GC02M070281 | 3.283789158 |
| KATNB1 | Katanin Regulatory Subunit B1 | Protein Coding | 37 | GC16P057735 | 3.283710957 |
| MRI1 | Methylthioribose-1-Phosphate Isomerase 1 | Protein Coding | 34 | GC19P013764 | 3.283256531 |
| UQCRC2 | Ubiquinol-Cytochrome C Reductase Core Protein 2 | Protein Coding | 41 | GC16P022171 | 3.283158302 |
| H2AC16 | H2A Clustered Histone 16 | Protein Coding | 26 | GC06P079952 | 3.283015966 |
| PTMA | Prothymosin Alpha | Protein Coding | 36 | GC02P231707 | 3.282985687 |
| DIO1 | Iodothyronine Deiodinase 1 | Protein Coding | 38 | GC01P053891 | 3.282924652 |
| UPP1 | Uridine Phosphorylase 1 | Protein Coding | 35 | GC07P048088 | 3.282463551 |
| AIFM2 | Apoptosis Inducing Factor Mitochondria Associated 2 | Protein Coding | 34 | GC10M070098 | 3.282362461 |
| LINC01096 | Long Intergenic Non-Protein Coding RNA 1096 | RNA Gene | 12 | GC04M013548 | 3.281944752 |
| SLC39A10 | Solute Carrier Family 39 Member 10 | Protein Coding | 35 | GC02P195575 | 3.281184673 |
| RPL29 | Ribosomal Protein L29 | Protein Coding | 35 | GC03M052025 | 3.280273914 |
| MIR188 | MicroRNA 188 | RNA Gene | 17 | GC0XP050003 | 3.28022337 |
| UBE2A | Ubiquitin Conjugating Enzyme E2 A | Protein Coding | 40 | GC0XP119574 | 3.280142784 |
| HSD17B7 | Hydroxysteroid 17-Beta Dehydrogenase 7 | Protein Coding | 38 | GC01P162790 | 3.278212547 |
| CAST | Calpastatin | Protein Coding | 42 | GC05P096525 | 3.277962208 |
| H4C3 | H4 Clustered Histone 3 | Protein Coding | 28 | GC06P079880 | 3.277156115 |
| MFN1 | Mitofusin 1 | Protein Coding | 38 | GC03P179347 | 3.276950359 |
| LGALS7 | Galectin 7 | Protein Coding | 33 | GC19M038770 | 3.275892019 |
| OPA1 | OPA1 Mitochondrial Dynamin Like GTPase | Protein Coding | 40 | GC03P193594 | 3.27417469 |
| SRGAP3 | SLIT-ROBO Rho GTPase Activating Protein 3 | Protein Coding | 36 | GC03M008980 | 3.273190022 |
| SORBS1 | Sorbin And SH3 Domain Containing 1 | Protein Coding | 36 | GC10M095311 | 3.273133993 |
| HEXA | Hexosaminidase Subunit Alpha | Protein Coding | 42 | GC15M072340 | 3.272335052 |
| MAP3K11 | Mitogen-Activated Protein Kinase Kinase Kinase 11 | Protein Coding | 42 | GC11M087276 | 3.271937847 |
| PIK3C2G | Phosphatidylinositol-4-Phosphate 3-Kinase Catalytic Subunit Type 2 Gamma | Protein Coding | 39 | GC12P018242 | 3.271036625 |

| | | | | | |
|---|---|---|---|---|---|
| MBNL1 | Muscleblind Like Splicing Regulator 1 | Protein Coding | 35 | GC03P152243 | 3.27004838 |
| TARBP2 | TARBP2 Subunit Of RISC Loading Complex | Protein Coding | 36 | GC12P053499 | 3.269869328 |
| TTF2 | Transcription Termination Factor 2 | Protein Coding | 35 | GC01P117060 | 3.268805742 |
| PAICS | Phosphoribosylaminoimidazole Carboxylase And Phosphoribosylaminoimidazolesuccinocarboxamide Synthase | Protein Coding | 36 | GC04P056410 | 3.268038034 |
| RPL19 | Ribosomal Protein L19 | Protein Coding | 37 | GC17P039200 | 3.266865253 |
| HCRT | Hypocretin Neuropeptide Precursor | Protein Coding | 38 | GC17M042185 | 3.266337156 |
| ITGB5 | Integrin Subunit Beta 5 | Protein Coding | 42 | GC03M124761 | 3.264051914 |
| CPSF6 | Cleavage And Polyadenylation Specific Factor 6 | Protein Coding | 34 | GC12P069239 | 3.263938427 |
| POMGNT2 | Protein O-Linked Mannose N-Acetylglucosaminyltransferase 2 (Beta 1,4-) | Protein Coding | 32 | GC03M043121 | 3.263819933 |
| TNFRSF25 | TNF Receptor Superfamily Member 25 | Protein Coding | 37 | GC01M006460 | 3.263151407 |
| MIR30C2 | MicroRNA 30c-2 | RNA Gene | 18 | GC06M071382 | 3.262892246 |
| MCM3AP | Minichromosome Maintenance Complex Component 3 Associated Protein | Protein Coding | 37 | GC21M046235 | 3.262546778 |
| PARVA | Parvin Alpha | Protein Coding | 38 | GC11P012398 | 3.262068272 |
| NTF4 | Neurotrophin 4 | Protein Coding | 39 | GC19M064099 | 3.26198411 |
| SPI1 | Spi-1 Proto-Oncogene | Protein Coding | 39 | GC11M086884 | 3.261183023 |
| CIT | Citron Rho-Interacting Serine/Threonine Kinase | Protein Coding | 42 | GC12M119650 | 3.260592699 |
| CRHR1 | Corticotropin Releasing Hormone Receptor 1 | Protein Coding | 42 | GC17P045784 | 3.260416508 |
| GLG1 | Golgi Glycoprotein 1 | Protein Coding | 35 | GC16M074550 | 3.260230064 |
| IL15RA | Interleukin 15 Receptor Subunit Alpha | Protein Coding | 36 | GC10M005943 | 3.259682894 |
| HOXA4 | Homeobox A4 | Protein Coding | 36 | GC07M027128 | 3.259302616 |
| CAPN6 | Calpain 6 | Protein Coding | 35 | GC0XM111245 | 3.258532763 |
| RAB5IF | RAB5 Interacting Factor | Protein Coding | 25 | GC20P036606 | 3.257885695 |
| CELSR1 | Cadherin EGF LAG Seven-Pass G-Type Receptor 1 | Protein Coding | 38 | GC22M046360 | 3.25774169 |
| WASHC5 | WASH Complex Subunit 5 | Protein Coding | 31 | GC08M131761 | 3.257285595 |
| GAL | Galanin And GMAP Prepropeptide | Protein Coding | 40 | GC11P069796 | 3.256761312 |
| HMGB2 | High Mobility Group Box 2 | Protein Coding | 37 | GC04M173331 | 3.25622797 |
| MIR519D | MicroRNA 519d | RNA Gene | 18 | GC19P053713 | 3.256053925 |
| MEF2D | Myocyte Enhancer Factor 2D | Protein Coding | 40 | GC01M156463 | 3.255146027 |
| HOOK2 | Hook Microtubule Tethering Protein 2 | Protein Coding | 33 | GC19M013524 | 3.254450798 |
| BAG6 | BAG Cochaperone 6 | Protein Coding | 32 | GC06M031639 | 3.252605438 |
| BLZF1 | Basic Leucine Zipper Nuclear Factor 1 | Protein Coding | 35 | GC01P169367 | 3.25201726 |
| MIR101-2 | MicroRNA 101-2 | RNA Gene | 17 | GC09P004863 | 3.251534939 |
| MYF5 | Myogenic Factor 5 | Protein Coding | 38 | GC12P080716 | 3.246944904 |
| FHOD1 | Formin Homology 2 Domain Containing 1 | Protein Coding | 34 | GC16M067230 | 3.246070623 |
| ULK1 | Unc-51 Like Autophagy Activating Kinase 1 | Protein Coding | 39 | GC12P131894 | 3.245423317 |
| SERPING1 | Serpin Family G Member 1 | Protein Coding | 42 | GC11P057597 | 3.245283604 |
| NR1I3 | Nuclear Receptor Subfamily 1 Group I Member 3 | Protein Coding | 40 | GC01M161229 | 3.244786263 |
| SH2D3A | SH2 Domain Containing 3A | Protein Coding | 31 | GC19M006752 | 3.243756771 |
| COQ8A | Coenzyme Q8A | Protein Coding | 35 | GC01P226939 | 3.243683815 |
| PGAM1 | Phosphoglycerate Mutase 1 | Protein Coding | 39 | GC10P097426 | 3.242429256 |
| SP110 | SP110 Nuclear Body Protein | Protein Coding | 37 | GC02M230167 | 3.242041588 |
| MARS1 | Methionyl-TRNA Synthetase 1 | Protein Coding | 35 | GC12P057476 | 3.241816998 |
| ARFRP1 | ADP Ribosylation Factor Related Protein 1 | Protein Coding | 34 | GC20M063698 | 3.241642475 |
| MLLT3 | MLLT3 Super Elongation Complex Subunit | Protein Coding | 35 | GC09M020341 | 3.240386724 |
| SUPT3H | SPT3 Homolog, SAGA And STAGA Complex Component | Protein Coding | 36 | GC06M063885 | 3.239960432 |
| FBXO8 | F-Box Protein 8 | Protein Coding | 32 | GC04M174236 | 3.239406586 |
| ARHGDIB | Rho GDP Dissociation Inhibitor Beta | Protein Coding | 38 | GC12M014942 | 3.238579035 |
| H4C2 | H4 Clustered Histone 2 | Protein Coding | 28 | GC06M026026 | 3.237783432 |
| H4C5 | H4 Clustered Histone 5 | Protein Coding | 26 | GC06P081045 | 3.237783432 |
| RPS5 | Ribosomal Protein S5 | Protein Coding | 36 | GC19P058386 | 3.237751007 |
| SIL1 | SIL1 Nucleotide Exchange Factor | Protein Coding | 36 | GC05M138957 | 3.235401154 |
| ADSL | Adenylosuccinate Lyase | Protein Coding | 42 | GC22P040346 | 3.235188007 |
| CNTNAP1 | Contactin Associated Protein 1 | Protein Coding | 39 | GC17P053173 | 3.234889984 |
| METTL14 | Methyltransferase 14, N6-Adenosine-Methyltransferase Subunit | Protein Coding | 34 | GC04P118685 | 3.232387781 |
| MAGEA2B | MAGE Family Member A2B | Protein Coding | 25 | GC0XP152714 | 3.2319417 |
| OS9 | OS9 Endoplasmic Reticulum Lectin | Protein Coding | 37 | GC12P057693 | 3.231475353 |
| TRIM26 | Tripartite Motif Containing 26 | Protein Coding | 33 | GC06M030184 | 3.230251789 |
| POGZ | Pogo Transposable Element Derived With ZNF Domain | Protein Coding | 38 | GC01M151402 | 3.229856491 |
| MAP2K7 | Mitogen-Activated Protein Kinase Kinase 7 | Protein Coding | 40 | GC19P007903 | 3.225837231 |
| RASSF2 | Ras Association Domain Family Member 2 | Protein Coding | 35 | GC20M004780 | 3.225586653 |
| ABCB7 | ATP Binding Cassette Subfamily B Member 7 | Protein Coding | 39 | GC0XM075053 | 3.22555995 |
| ALOX5AP | Arachidonate 5-Lipoxygenase Activating Protein | Protein Coding | 39 | GC13P030713 | 3.225047112 |
| DLG1 | Discs Large MAGUK Scaffold Protein 1 | Protein Coding | 39 | GC03M197042 | 3.223777771 |
| TBX20 | T-Box Transcription Factor 20 | Protein Coding | 38 | GC07M035237 | 3.222892284 |
| ASCC1 | Activating Signal Cointegrator 1 Complex Subunit 1 | Protein Coding | 37 | GC10M072096 | 3.220883608 |
| RAI1 | Retinoic Acid Induced 1 | Protein Coding | 36 | GC17P017682 | 3.220788479 |
| LTBP2 | Latent Transforming Growth Factor Beta Binding Protein 2 | Protein Coding | 40 | GC14M074498 | 3.220266342 |
| PAH | Phenylalanine Hydroxylase | Protein Coding | 43 | GC12M102836 | 3.220232248 |
| DYNLRB1 | Dynein Light Chain Roadblock-Type 1 | Protein Coding | 33 | GC20P034607 | 3.220091581 |
| CIZ1 | CDKN1A Interacting Zinc Finger Protein 1 | Protein Coding | 35 | GC09M128181 | 3.219968557 |
| ECD | Ecdysoneless Cell Cycle Regulator | Protein Coding | 33 | GC10M073130 | 3.219049454 |
| FRK | Fyn Related Src Family Tyrosine Kinase | Protein Coding | 41 | GC06M115931 | 3.218360424 |
| EMG1 | EMG1 N1-Specific Pseudouridine Methyltransferase | Protein Coding | 35 | GC12P006970 | 3.217867613 |
| SMTN | Smoothelin | Protein Coding | 35 | GC22P035012 | 3.217745304 |
| UBTF | Upstream Binding Transcription Factor | Protein Coding | 38 | GC17M044205 | 3.216819763 |
| CRACD | Capping Protein Inhibiting Regulator Of Actin Dynamics | Protein Coding | 24 | GC04P056050 | 3.216614246 |
| RIOK2 | RIO Kinase 2 | Protein Coding | 36 | GC05M097160 | 3.216515064 |
| PTGER1 | Prostaglandin E Receptor 1 | Protein Coding | 39 | GC19M014474 | 3.216167927 |
| RNF20 | Ring Finger Protein 20 | Protein Coding | 34 | GC09P101533 | 3.21591711 |
| DMRT3 | Doublesex And Mab-3 Related Transcription Factor 3 | Protein Coding | 32 | GC09P000976 | 3.215536118 |
| MAPK11 | Mitogen-Activated Protein Kinase 11 | Protein Coding | 42 | GC22M050263 | 3.215453863 |
| ABCC8 | ATP Binding Cassette Subfamily C Member 8 | Protein Coding | 43 | GC11M017392 | 3.215435028 |
| MGP | Matrix Gla Protein | Protein Coding | 38 | GC12M020512 | 3.215224743 |
| INHBE | Inhibin Subunit Beta E | Protein Coding | 35 | GC12P057452 | 3.215072155 |
| LITAF | Lipopolysaccharide Induced TNF Factor | Protein Coding | 39 | GC16M011547 | 3.213017225 |
| ALOXE3 | Arachidonate Lipoxygenase 3 | Protein Coding | 39 | GC17M010268 | 3.21297574 |
| FHL1 | Four And A Half LIM Domains 1 | Protein Coding | 41 | GC0XP136146 | 3.212419748 |
| TLL1 | Tolloid Like 1 | Protein Coding | 40 | GC04P165873 | 3.211267948 |
| MVD | Mevalonate Diphosphate Decarboxylase | Protein Coding | 40 | GC16M088651 | 3.210766315 |
| EIF5 | Eukaryotic Translation Initiation Factor 5 | Protein Coding | 37 | GC14P103333 | 3.21064806 |
| SLC16A6 | Solute Carrier Family 16 Member 6 | Protein Coding | 33 | GC17M068267 | 3.210061073 |
| RPS6KA6 | Ribosomal Protein S6 Kinase A6 | Protein Coding | 38 | GC0XM084058 | 3.209490538 |
| BRAT1 | BRCA1 Associated ATM Activator 1 | Protein Coding | 33 | GC07M002739 | 3.208956003 |
| FNBP1L | Formin Binding Protein 1 Like | Protein Coding | 32 | GC01P093448 | 3.208237171 |

| | | | | | |
|---|---|---|---|---|---|
| KCTD11 | Potassium Channel Tetramerization Domain Containing 11 | Protein Coding | 32 | GC17P007351 | 3.207393646 |
| HOOK3 | Hook Microtubule Tethering Protein 3 | Protein Coding | 31 | GC08P042896 | 3.207173824 |
| RPL27A | Ribosomal Protein L27a | Protein Coding | 36 | GC11P008682 | 3.206004858 |
| HUS1 | HUS1 Checkpoint Clamp Component | Protein Coding | 36 | GC07M048029 | 3.20596838 |
| H2AC12 | H2A Clustered Histone 12 | Protein Coding | 26 | GC06P081031 | 3.205845833 |
| BAALC | BAALC Binder Of MAP3K1 And KLF4 | Protein Coding | 32 | GC08P103149 | 3.205648184 |
| ZNF569 | Zinc Finger Protein 569 | Protein Coding | 31 | GC19M063762 | 3.205384254 |
| TNFRSF17 | TNF Receptor Superfamily Member 17 | Protein Coding | 39 | GC16P011965 | 3.204716921 |
| LINC00926 | Long Intergenic Non-Protein Coding RNA 926 | RNA Gene | 15 | GC15P057300 | 3.203579903 |
| PDE5A | Phosphodiesterase 5A | Protein Coding | 41 | GC04M119494 | 3.203030825 |
| KLRD1 | Killer Cell Lectin Like Receptor D1 | Protein Coding | 38 | GC12P010226 | 3.203015089 |
| ACTL6A | Actin Like 6A | Protein Coding | 36 | GC03P179562 | 3.20222497 |
| IL4I1 | Interleukin 4 Induced 1 | Protein Coding | 35 | GC19M064139 | 3.201802492 |
| NUP93 | Nucleoporin 93 | Protein Coding | 36 | GC16P057015 | 3.201339245 |
| GRIK1 | Glutamate Ionotropic Receptor Kainate Type Subunit 1 | Protein Coding | 41 | GC21M029536 | 3.200549841 |
| EZH1 | Enhancer Of Zeste 1 Polycomb Repressive Complex 2 Subunit | Protein Coding | 40 | GC17M042700 | 3.199942112 |
| CTSV | Cathepsin V | Protein Coding | 37 | GC09M097029 | 3.19898653 |
| TMEM47 | Transmembrane Protein 47 | Protein Coding | 31 | GC0XM034555 | 3.198326111 |
| SLC26A2 | Solute Carrier Family 26 Member 2 | Protein Coding | 40 | GC05P149944 | 3.198192835 |
| POTEF | POTE Ankyrin Domain Family Member F | Protein Coding | 25 | GC02M130073 | 3.197711945 |
| PLAAT4 | Phospholipase A And Acyltransferase 4 | Protein Coding | 28 | GC11P063678 | 3.197499275 |
| STT3A | STT3 Oligosaccharyltransferase Complex Catalytic Subunit A | Protein Coding | 40 | GC11P125592 | 3.197453976 |
| PYGM | Glycogen Phosphorylase, Muscle Associated | Protein Coding | 42 | GC11M064746 | 3.194470882 |
| GALNT14 | Polypeptide N-Acetylgalactosaminyltransferase 14 | Protein Coding | 38 | GC02M030888 | 3.194373846 |
| DEFB4A | Defensin Beta 4A | Protein Coding | 31 | GC08P007895 | 3.192878485 |
| DHX30 | DExH-Box Helicase 30 | Protein Coding | 36 | GC03P047802 | 3.192699671 |
| LTA4H | Leukotriene A4 Hydrolase | Protein Coding | 40 | GC12M096000 | 3.192675352 |
| ALS2CL | ALS2 C-Terminal Like | Protein Coding | 31 | GC03M046685 | 3.19244957 |
| VPS37A | VPS37A Subunit Of ESCRT-I | Protein Coding | 38 | GC08P017246 | 3.192122936 |
| MIR382 | MicroRNA 382 | RNA Gene | 15 | GC14P109532 | 3.192039967 |
| KRT76 | Keratin 76 | Protein Coding | 31 | GC12M052768 | 3.191509247 |
| YWHAG | Tyrosine 3-Monooxygenase/Tryptophan 5-Monooxygenase Activation Protein Gamma | Protein Coding | 44 | GC07M076724 | 3.191112995 |
| GLYAT | Glycine-N-Acyltransferase | Protein Coding | 36 | GC11M086974 | 3.188037157 |
| RAB7A | RAB7A, Member RAS Oncogene Family | Protein Coding | 42 | GC03P133819 | 3.186073542 |
| GALR1 | Galanin Receptor 1 | Protein Coding | 42 | GC18P077250 | 3.185678959 |
| CHRM3 | Cholinergic Receptor Muscarinic 3 | Protein Coding | 44 | GC01P239386 | 3.185241699 |
| TRPC4 | Transient Receptor Potential Cation Channel Subfamily C Member 4 | Protein Coding | 40 | GC13M037636 | 3.185238361 |
| TMEM43 | Transmembrane Protein 43 | Protein Coding | 38 | GC03P014124 | 3.184820652 |
| FABP2 | Fatty Acid Binding Protein 2 | Protein Coding | 38 | GC04M119317 | 3.184638262 |
| HS2ST1 | Heparan Sulfate 2-O-Sulfotransferase 1 | Protein Coding | 38 | GC01P086914 | 3.184586525 |
| ADARB2 | Adenosine Deaminase RNA Specific B2 (Inactive) | Protein Coding | 36 | GC10M001200 | 3.184250832 |
| JAKMIP1 | Janus Kinase And Microtubule Interacting Protein 1 | Protein Coding | 33 | GC04M006026 | 3.184177876 |
| BACH2 | BTB Domain And CNC Homolog 2 | Protein Coding | 39 | GC06M089926 | 3.183689356 |
| DUSP22 | Dual Specificity Phosphatase 22 | Protein Coding | 35 | GC06P000295 | 3.182547808 |
| PEMT | Phosphatidylethanolamine N-Methyltransferase | Protein Coding | 36 | GC17M017551 | 3.181008101 |
| CD2AP | CD2 Associated Protein | Protein Coding | 38 | GC06P080627 | 3.181004763 |
| MT1F | Metallothionein 1F | Protein Coding | 32 | GC16P056657 | 3.181004286 |
| UBE2D2 | Ubiquitin Conjugating Enzyme E2 D2 | Protein Coding | 39 | GC05P139526 | 3.179961681 |
| PIK3R5 | Phosphoinositide-3-Kinase Regulatory Subunit 5 | Protein Coding | 40 | GC17M008878 | 3.179927349 |
| NIP7 | Nucleolar Pre-RRNA Processing Protein NIP7 | Protein Coding | 34 | GC16P069456 | 3.178059101 |
| CDH15 | Cadherin 15 | Protein Coding | 40 | GC16P089171 | 3.177364826 |
| SLC19A3 | Solute Carrier Family 19 Member 3 | Protein Coding | 42 | GC02M227685 | 3.177197456 |
| ONECUT2 | One Cut Homeobox 2 | Protein Coding | 34 | GC18P057436 | 3.17713356 |
| CAB39L | Calcium Binding Protein 39 Like | Protein Coding | 31 | GC13M049308 | 3.177084684 |
| CAP1 | Cyclase Associated Actin Cytoskeleton Regulatory Protein 1 | Protein Coding | 37 | GC01P040118 | 3.177028179 |
| TPP1 | Tripeptidyl Peptidase 1 | Protein Coding | 39 | GC11M006620 | 3.176895142 |
| RBM4 | RNA Binding Motif Protein 4 | Protein Coding | 35 | GC11P066638 | 3.176423073 |
| MBD1 | Methyl-CpG Binding Domain Protein 1 | Protein Coding | 36 | GC18M050266 | 3.176303148 |
| H2AC1 | H2A Clustered Histone 1 | Protein Coding | 28 | GC06M025961 | 3.176131725 |
| TTC3 | Tetratricopeptide Repeat Domain 3 | Protein Coding | 36 | GC21P037073 | 3.175731182 |
| TRPC5 | Transient Receptor Potential Cation Channel Subfamily C Member 5 | Protein Coding | 38 | GC0XM111774 | 3.174523592 |
| FGF16 | Fibroblast Growth Factor 16 | Protein Coding | 36 | GC0XP077447 | 3.174033403 |
| PAFAH1B1 | Platelet Activating Factor Acetylhydrolase 1b Regulatory Subunit 1 | Protein Coding | 40 | GC17P002593 | 3.172246218 |
| RAB3GAP2 | RAB3 GTPase Activating Non-Catalytic Protein Subunit 2 | Protein Coding | 35 | GC01M220149 | 3.172221184 |
| CTNNA3 | Catenin Alpha 3 | Protein Coding | 36 | GC10M065912 | 3.172116995 |
| C5 | Complement C5 | Protein Coding | 42 | GC09M120952 | 3.171663523 |
| USP53 | Ubiquitin Specific Peptidase 53 | Protein Coding | 36 | GC04P119212 | 3.170654297 |
| APLN | Apelin | Protein Coding | 35 | GC0XM129645 | 3.169831753 |
| HPGDS | Hematopoietic Prostaglandin D Synthase | Protein Coding | 36 | GC04M094298 | 3.168498516 |
| FOSB | FosB Proto-Oncogene, AP-1 Transcription Factor Subunit | Protein Coding | 37 | GC19P045467 | 3.166756392 |
| SMN2 | Survival Of Motor Neuron 2, Centromeric | Protein Coding | 38 | GC05P070049 | 3.166707277 |
| TUFM | Tu Translation Elongation Factor, Mitochondrial | Protein Coding | 41 | GC16M036335 | 3.166114807 |
| EIF5B | Eukaryotic Translation Initiation Factor 5B | Protein Coding | 33 | GC02P099320 | 3.166093349 |
| MAGOHB | Mago Homolog B, Exon Junction Complex Subunit | Protein Coding | 31 | GC12M020450 | 3.165929317 |
| SMC6 | Structural Maintenance Of Chromosomes 6 | Protein Coding | 31 | GC02M017663 | 3.165629864 |
| UTP14A | UTP14A Small Subunit Processome Component | Protein Coding | 31 | GC0XP129906 | 3.165188313 |
| VMP1 | Vacuole Membrane Protein 1 | Protein Coding | 31 | GC17P059707 | 3.164642334 |
| SRP19 | Signal Recognition Particle 19 | Protein Coding | 35 | GC05P112862 | 3.164638042 |
| IMPDH1 | Inosine Monophosphate Dehydrogenase 1 | Protein Coding | 45 | GC07M128392 | 3.163689852 |
| DCT | Dopachrome Tautomerase | Protein Coding | 40 | GC13M094436 | 3.163167477 |
| MIR520A | MicroRNA 520a | RNA Gene | 18 | GC19P053690 | 3.162411213 |
| CDC7 | Cell Division Cycle 7 | Protein Coding | 40 | GC01P091500 | 3.161286831 |
| LINC02878 | Long Intergenic Non-Protein Coding RNA 2878 | RNA Gene | 13 | GC08P144097 | 3.160753727 |
| MRPL28 | Mitochondrial Ribosomal Protein L28 | Protein Coding | 33 | GC16M000366 | 3.160036802 |
| MARK1 | Microtubule Affinity Regulating Kinase 1 | Protein Coding | 38 | GC01P220528 | 3.159844398 |
| PIGT | Phosphatidylinositol Glycan Anchor Biosynthesis Class T | Protein Coding | 36 | GC20P045416 | 3.159575939 |
| B3GALNT2 | Beta-1,3-N-Acetylgalactosaminyltransferase 2 | Protein Coding | 36 | GC01M235440 | 3.159571648 |
| PDZK1 | PDZ Domain Containing 1 | Protein Coding | 36 | GC01M145670 | 3.159485817 |
| CDK11B | Cyclin Dependent Kinase 11B | Protein Coding | 36 | GC01M005553 | 3.158949375 |
| GALK1 | Galactokinase 1 | Protein Coding | 43 | GC17M075751 | 3.158619881 |
| SS18L1 | SS18L1 Subunit Of BAF Chromatin Remodeling Complex | Protein Coding | 35 | GC20P062143 | 3.15811491 |
| AKAP4 | A-Kinase Anchoring Protein 4 | Protein Coding | 32 | GC0XM050190 | 3.157338619 |
| FLVCR1 | FLVCR Heme Transporter 1 | Protein Coding | 37 | GC01P212858 | 3.157229662 |
| SYNCRIP | Synaptotagmin Binding Cytoplasmic RNA Interacting Protein | Protein Coding | 36 | GC06M085607 | 3.155750036 |

| | | | | | |
|---|---|---|---|---|---|
| CC2D2A | Coiled-Coil And C2 Domain Containing 2A | Protein Coding | 35 | GC04P017733 | 3.15551877 |
| CHTF18 | Chromosome Transmission Fidelity Factor 18 | Protein Coding | 32 | GC16P010653 | 3.154246807 |
| MAOB | Monoamine Oxidase B | Protein Coding | 39 | GC0XM043766 | 3.154009342 |
| ESPL1 | Extra Spindle Pole Bodies Like 1, Separase | Protein Coding | 37 | GC12P053268 | 3.153986454 |
| BCL7A | BAF Chromatin Remodeling Complex Subunit BCL7A | Protein Coding | 32 | GC12P122019 | 3.152136087 |
| USP10 | Ubiquitin Specific Peptidase 10 | Protein Coding | 39 | GC16P084702 | 3.15157795 |
| GCK | Glucokinase | Protein Coding | 45 | GC07M044433 | 3.150957108 |
| H2AZ1 | H2A.Z Variant Histone 1 | Protein Coding | 31 | GC04M099949 | 3.150749683 |
| C8orf17 | Chromosome 8 Putative Open Reading Frame 17 | RNA Gene | 19 | GC08P139932 | 3.150405407 |
| TAPBP | TAP Binding Protein | Protein Coding | 40 | GC06M033299 | 3.149727345 |
| SNTB1 | Syntrophin Beta 1 | Protein Coding | 34 | GC08M120535 | 3.149655581 |
| PNLIPRP1 | Pancreatic Lipase Related Protein 1 | Protein Coding | 36 | GC10P116590 | 3.149534464 |
| TEKT4 | Tektin 4 | Protein Coding | 31 | GC02P094872 | 3.148498058 |
| SETD7 | SET Domain Containing 7, Histone Lysine Methyltransferase | Protein Coding | 39 | GC04M139495 | 3.147953033 |
| EFTUD2 | Elongation Factor Tu GTP Binding Domain Containing 2 | Protein Coding | 38 | GC17M044861 | 3.147881985 |
| YWHAB | Tyrosine 3-Monooxygenase/Tryptophan 5-Monooxygenase Activation Protein Beta | Protein Coding | 42 | GC20P044885 | 3.147838354 |
| PCBP2 | Poly(RC) Binding Protein 2 | Protein Coding | 36 | GC12P053452 | 3.146844625 |
| NAA80 | N-Alpha-Acetyltransferase 80, NatH Catalytic Subunit | Protein Coding | 26 | GC03M051097 | 3.146129608 |
| MIR449B | MicroRNA 449b | RNA Gene | 18 | GC05M055172 | 3.146054983 |
| CPQ | Carboxypeptidase Q | Protein Coding | 33 | GC08P096645 | 3.145840883 |
| NETO1 | Neuropilin And Tolloid Like 1 | Protein Coding | 33 | GC18M072742 | 3.14503026 |
| PLA2G3 | Phospholipase A2 Group III | Protein Coding | 34 | GC22M036195 | 3.14406538 |
| MAML1 | Mastermind Like Transcriptional Coactivator 1 | Protein Coding | 35 | GC05P179732 | 3.143844604 |
| CRB1 | Crumbs Cell Polarity Complex Component 1 | Protein Coding | 40 | GC01P197170 | 3.143598557 |
| CXXC4 | CXXC Finger Protein 4 | Protein Coding | 34 | GC04M104468 | 3.142954588 |
| CHPF | Chondroitin Polymerizing Factor | Protein Coding | 34 | GC02M219538 | 3.142236233 |
| POLDIP2 | DNA Polymerase Delta Interacting Protein 2 | Protein Coding | 32 | GC17M028346 | 3.141882896 |
| SMARCA1 | SWI/SNF Related, Matrix Associated, Actin Dependent Regulator Of Chromatin, Subfamily A, Member 1 | Protein Coding | 35 | GC0XM129447 | 3.141364098 |
| SLC9A2 | Solute Carrier Family 9 Member A2 | Protein Coding | 37 | GC02P102620 | 3.14124012 |
| NRG4 | Neuregulin 4 | Protein Coding | 37 | GC15M081534 | 3.140955687 |
| SLC16A3 | Solute Carrier Family 16 Member 3 | Protein Coding | 40 | GC17P082217 | 3.14063549 |
| EIF4H | Eukaryotic Translation Initiation Factor 4H | Protein Coding | 37 | GC07P074174 | 3.138876677 |
| IDE | Insulin Degrading Enzyme | Protein Coding | 43 | GC10M092451 | 3.138774872 |
| TUBB4A | Tubulin Beta 4A Class IVa | Protein Coding | 40 | GC19M006496 | 3.138566732 |
| H1-4 | H1.4 Linker Histone, Cluster Member | Protein Coding | 34 | GC06P081029 | 3.138065577 |
| DND1 | DND MicroRNA-Mediated Repression Inhibitor 1 | Protein Coding | 31 | GC05M140670 | 3.136879921 |
| CYP3A7 | Cytochrome P450 Family 3 Subfamily A Member 7 | Protein Coding | 38 | GC07M099705 | 3.136224747 |
| MICAL1 | Microtubule Associated Monooxygenase, Calponin And LIM Domain Containing 1 | Protein Coding | 38 | GC06M109444 | 3.135746956 |
| SIK2 | Salt Inducible Kinase 2 | Protein Coding | 39 | GC11P111711 | 3.13531971 |
| NUP88 | Nucleoporin 88 | Protein Coding | 35 | GC17M006088 | 3.135177135 |
| NEUROD1 | Neuronal Differentiation 1 | Protein Coding | 42 | GC02M181673 | 3.132612228 |
| RPN2 | Ribophorin II | Protein Coding | 36 | GC20P037178 | 3.131732702 |
| VPS72 | Vacuolar Protein Sorting 72 Homolog | Protein Coding | 32 | GC01M151176 | 3.131673574 |
| PPP1R9B | Protein Phosphatase 1 Regulatory Subunit 9B | Protein Coding | 36 | GC17M050133 | 3.131131649 |
| ATXN10 | Ataxin 10 | Protein Coding | 38 | GC22P045673 | 3.1294837 |
| RPS3A | Ribosomal Protein S3A | Protein Coding | 36 | GC04P151099 | 3.129024267 |
| LZTFL1 | Leucine Zipper Transcription Factor Like 1 | Protein Coding | 35 | GC03M045823 | 3.128865242 |
| ARHGAP24 | Rho GTPase Activating Protein 24 | Protein Coding | 35 | GC04P085475 | 3.128256798 |
| MAN1B1 | Mannosidase Alpha Class 1B Member 1 | Protein Coding | 42 | GC09P137086 | 3.127962589 |
| ASMT | Acetylserotonin O-Methyltransferase | Protein Coding | 33 | GC0XP001595 | 3.127310038 |
| RAB11B | RAB11B, Member RAS Oncogene Family | Protein Coding | 38 | GC19P008393 | 3.126945496 |
| MCOLN1 | Mucolipin TRP Cation Channel 1 | Protein Coding | 38 | GC19P007523 | 3.126796246 |
| PTPRS | Protein Tyrosine Phosphatase Receptor Type S | Protein Coding | 41 | GC19M005157 | 3.126587868 |
| STOML2 | Stomatin Like 2 | Protein Coding | 35 | GC09M035099 | 3.125965118 |
| MINCR | MYC-Induced Long Non-Coding RNA | RNA Gene | 13 | GC08M143281 | 3.125627518 |
| TRO | Trophinin | Protein Coding | 34 | GC0XP054920 | 3.125577688 |
| RCL1 | RNA Terminal Phosphate Cyclase Like 1 | Protein Coding | 33 | GC09P004782 | 3.125336885 |
| EPHX2 | Epoxide Hydrolase 2 | Protein Coding | 43 | GC08P027490 | 3.125108719 |
| CSPP1 | Centrosome And Spindle Pole Associated Protein 1 | Protein Coding | 35 | GC08P067062 | 3.124605417 |
| CALM3 | Calmodulin 3 | Protein Coding | 38 | GC19P046601 | 3.124099016 |
| TDG | Thymine DNA Glycosylase | Protein Coding | 38 | GC12P103965 | 3.123306513 |
| FNIP1 | Folliculin Interacting Protein 1 | Protein Coding | 33 | GC05M131641 | 3.123028755 |
| ICE2 | Interactor Of Little Elongation Complex ELL Subunit 2 | Protein Coding | 28 | GC15M060419 | 3.122904539 |
| HMGN1 | High Mobility Group Nucleosome Binding Domain 1 | Protein Coding | 36 | GC21M039342 | 3.122696638 |
| TENT4A | Terminal Nucleotidyltransferase 4A | Protein Coding | 30 | GC05P006713 | 3.12225461 |
| NLRC5 | NLR Family CARD Domain Containing 5 | Protein Coding | 35 | GC16P056994 | 3.122138739 |
| CXCL3 | C-X-C Motif Chemokine Ligand 3 | Protein Coding | 33 | GC04M074036 | 3.121557236 |
| EIF4B | Eukaryotic Translation Initiation Factor 4B | Protein Coding | 37 | GC12P053006 | 3.121412039 |
| ALX1 | ALX Homeobox 1 | Protein Coding | 35 | GC12P085279 | 3.121140718 |
| GOLPH3 | Golgi Phosphoprotein 3 | Protein Coding | 35 | GC05M032124 | 3.120897055 |
| SCP2 | Sterol Carrier Protein 2 | Protein Coding | 41 | GC01P052927 | 3.120767117 |
| HNRNPC | Heterogeneous Nuclear Ribonucleoprotein C | Protein Coding | 36 | GC14M022213 | 3.120256901 |
| ADCY9 | Adenylate Cyclase 9 | Protein Coding | 40 | GC16M003953 | 3.119839191 |
| WTAP | WT1 Associated Protein | Protein Coding | 35 | GC06P159725 | 3.119297504 |
| MAEL | Maelstrom Spermatogenic Transposon Silencer | Protein Coding | 31 | GC01P166922 | 3.119005919 |
| STRN | Striatin | Protein Coding | 36 | GC02M036815 | 3.117678404 |
| CLCN6 | Chloride Voltage-Gated Channel 6 | Protein Coding | 39 | GC01P011806 | 3.116965055 |
| CTSC | Cathepsin C | Protein Coding | 43 | GC11M089158 | 3.116544008 |
| CLEC1A | C-Type Lectin Domain Family 1 Member A | Protein Coding | 36 | GC12M020438 | 3.115505219 |
| SSB | Small RNA Binding Exonuclease Protection Factor La | Protein Coding | 38 | GC02P169791 | 3.115103722 |
| SPAG5 | Sperm Associated Antigen 5 | Protein Coding | 34 | GC17M028577 | 3.112010717 |
| MCM9 | Minichromosome Maintenance 9 Homologous Recombination Repair Factor | Protein Coding | 35 | GC06M118813 | 3.109786034 |
| ATRAID | All-Trans Retinoic Acid Induced Differentiation Factor | Protein Coding | 32 | GC02P027212 | 3.108513117 |
| CHTF8 | Chromosome Transmission Fidelity Factor 8 | Protein Coding | 29 | GC16M069122 | 3.108492851 |
| POLR1A | RNA Polymerase I Subunit A | Protein Coding | 39 | GC02M086021 | 3.108073473 |
| CAPN3 | Calpain 3 | Protein Coding | 43 | GC15P042359 | 3.107445002 |
| PEAK1 | Pseudopodium Enriched Atypical Kinase 1 | Protein Coding | 31 | GC15M077100 | 3.107306719 |
| BAIAP2L1 | BAR/IMD Domain Containing Adaptor Protein 2 Like 1 | Protein Coding | 35 | GC07M098294 | 3.106497288 |
| TSPAN8 | Tetraspanin 8 | Protein Coding | 35 | GC12M071125 | 3.106215 |
| GJA5 | Gap Junction Protein Alpha 5 | Protein Coding | 41 | GC01M147756 | 3.105591774 |
| FAM210A | Family With Sequence Similarity 210 Member A | Protein Coding | 26 | GC18M013663 | 3.105582714 |
| GUK1 | Guanylate Kinase 1 | Protein Coding | 37 | GC01P228139 | 3.104740858 |
| GTF2A1 | General Transcription Factor IIA Subunit 1 | Protein Coding | 31 | GC14M081175 | 3.104193211 |
| BRD3 | Bromodomain Containing 3 | Protein Coding | 37 | GC09M134030 | 3.103206635 |

| | | | | | |
|---|---|---|---|---|---|
| KIF2C | Kinesin Family Member 2C | Protein Coding | 38 | GC01P044739 | 3.100214958 |
| FBL | Fibrillarin | Protein Coding | 38 | GC19M039834 | 3.100078106 |
| UTS2R | Urotensin 2 Receptor | Protein Coding | 38 | GC17P082374 | 3.100005627 |
| NLGN4X | Neuroligin 4 X-Linked | Protein Coding | 36 | GC0XM005840 | 3.099076986 |
| ACE2 | Angiotensin Converting Enzyme 2 | Protein Coding | 43 | GC0XM015494 | 3.098317146 |
| YEATS2 | YEATS Domain Containing 2 | Protein Coding | 32 | GC03P183698 | 3.098049641 |
| SHOX | Short Stature Homeobox | Protein Coding | 35 | GC0XP000624 | 3.0976758 |
| MAGEL2 | MAGE Family Member L2 | Protein Coding | 33 | GC15M023643 | 3.096977234 |
| PINK1 | PTEN Induced Kinase 1 | Protein Coding | 42 | GC01P020634 | 3.095927954 |
| ALOX12B | Arachidonate 12-Lipoxygenase, 12R Type | Protein Coding | 38 | GC17M010270 | 3.09436655 |
| ATP2B2 | ATPase Plasma Membrane Ca2+ Transporting 2 | Protein Coding | 42 | GC03M010324 | 3.09427309 |
| STIL | STIL Centriolar Assembly Protein | Protein Coding | 36 | GC01M047250 | 3.093790054 |
| PRRX1 | Paired Related Homeobox 1 | Protein Coding | 38 | GC01P170662 | 3.093488693 |
| PCSK2 | Proprotein Convertase Subtilisin/Kexin Type 2 | Protein Coding | 40 | GC20P017226 | 3.09339571 |
| STIP1 | Stress Induced Phosphoprotein 1 | Protein Coding | 40 | GC11P064351 | 3.093038559 |
| GABARAP | GABA Type A Receptor-Associated Protein | Protein Coding | 38 | GC17M007240 | 3.090829134 |
| RAB5A | RAB5A, Member RAS Oncogene Family | Protein Coding | 40 | GC03P019948 | 3.090355396 |
| TRIP11 | Thyroid Hormone Receptor Interactor 11 | Protein Coding | 38 | GC14M091965 | 3.090325594 |
| KDM4A | Lysine Demethylase 4A | Protein Coding | 38 | GC01P043650 | 3.088568211 |
| PLXNC1 | Plexin C1 | Protein Coding | 35 | GC12P094150 | 3.088471413 |
| ZBTB3 | Zinc Finger And BTB Domain Containing 3 | Protein Coding | 28 | GC11M062748 | 3.087422848 |
| RRBP1 | Ribosome Binding Protein 1 | Protein Coding | 35 | GC20M017613 | 3.087351799 |
| ST6GALNAC1 | ST6 N-Acetylgalactosaminide Alpha-2,6-Sialyltransferase 1 | Protein Coding | 35 | GC17M076624 | 3.08526659 |
| POLR1D | RNA Polymerase I And III Subunit D | Protein Coding | 39 | GC13P027620 | 3.084877253 |
| ALKBH5 | AlkB Homolog 5, RNA Demethylase | Protein Coding | 30 | GC17P018183 | 3.084823132 |
| CFAP47 | Cilia And Flagella Associated Protein 47 | Protein Coding | 26 | GC0XP035919 | 3.083708286 |
| MIR3664 | MicroRNA 3664 | RNA Gene | 11 | GC11M070872 | 3.083547592 |
| HNRNPR | Heterogeneous Nuclear Ribonucleoprotein R | Protein Coding | 35 | GC01M023303 | 3.082694292 |
| HAND2 | Heart And Neural Crest Derivatives Expressed 2 | Protein Coding | 39 | GC04M173524 | 3.081888676 |
| TSHB | Thyroid Stimulating Hormone Subunit Beta | Protein Coding | 40 | GC01P115029 | 3.081633329 |
| LIPG | Lipase G, Endothelial Type | Protein Coding | 39 | GC18P049560 | 3.081082821 |
| ADD1 | Adducin 1 | Protein Coding | 40 | GC04P002855 | 3.080384254 |
| TMEFF1 | Transmembrane Protein With EGF Like And Two Follistatin Like Domains 1 | Protein Coding | 34 | GC09P100473 | 3.079955339 |
| PLIN1 | Perilipin 1 | Protein Coding | 41 | GC15M089664 | 3.079708576 |
| METAP2 | Methionyl Aminopeptidase 2 | Protein Coding | 40 | GC12P095473 | 3.079404354 |
| CLEC16A | C-Type Lectin Domain Containing 16A | Protein Coding | 33 | GC16P010944 | 3.07934618 |
| BCL7B | BAF Chromatin Remodeling Complex Subunit BCL7B | Protein Coding | 34 | GC07M073536 | 3.079061985 |
| CCL28 | C-C Motif Chemokine Ligand 28 | Protein Coding | 36 | GC05M043356 | 3.07877779 |
| ATP5F1A | ATP Synthase F1 Subunit Alpha | Protein Coding | 36 | GC18M046081 | 3.077923298 |
| KLF12 | Kruppel Like Factor 12 | Protein Coding | 34 | GC13M073686 | 3.077336311 |
| RIN2 | Ras And Rab Interactor 2 | Protein Coding | 37 | GC20P019759 | 3.077250957 |
| CTSS | Cathepsin S | Protein Coding | 41 | GC01M150730 | 3.0770154 |
| DGKA | Diacylglycerol Kinase Alpha | Protein Coding | 39 | GC12P055927 | 3.076588154 |
| MAFG | MAF BZIP Transcription Factor G | Protein Coding | 35 | GC17M081918 | 3.076502562 |
| CASP4 | Caspase 4 | Protein Coding | 42 | GC11M104942 | 3.074968815 |
| ARG2 | Arginase 2 | Protein Coding | 40 | GC14P067619 | 3.073765039 |
| STK10 | Serine/Threonine Kinase 10 | Protein Coding | 39 | GC05M172042 | 3.073650837 |
| CXCL16 | C-X-C Motif Chemokine Ligand 16 | Protein Coding | 35 | GC17M004733 | 3.073489666 |
| SLC22A9 | Solute Carrier Family 22 Member 9 | Protein Coding | 34 | GC11P063388 | 3.073478699 |
| STING1 | Stimulator Of Interferon Response CGAMP Interactor 1 | Protein Coding | 32 | GC05M139476 | 3.073300362 |
| ATXN7 | Ataxin 7 | Protein Coding | 37 | GC03P063864 | 3.07216835 |
| KLF11 | Kruppel Like Factor 11 | Protein Coding | 37 | GC02P010044 | 3.071866512 |
| CDK20 | Cyclin Dependent Kinase 20 | Protein Coding | 37 | GC09M087966 | 3.070887089 |
| ZMPSTE24 | Zinc Metallopeptidase STE24 | Protein Coding | 37 | GC01P040258 | 3.070652485 |
| NEDD8 | NEDD8 Ubiquitin Like Modifier | Protein Coding | 35 | GC14M024216 | 3.070543051 |
| PNN | Pinin, Desmosome Associated Protein | Protein Coding | 31 | GC14P039175 | 3.070307732 |
| KCTD5 | Potassium Channel Tetramerization Domain Containing 5 | Protein Coding | 31 | GC16P002682 | 3.070113659 |
| COQ2 | Coenzyme Q2, Polyprenyltransferase | Protein Coding | 36 | GC04M083261 | 3.069710255 |
| DCHS2 | Dachsous Cadherin-Related 2 | Protein Coding | 30 | GC04M154232 | 3.069667816 |
| RALGAPA1 | Ral GTPase Activating Protein Catalytic Subunit Alpha 1 | Protein Coding | 36 | GC14M035538 | 3.068037987 |
| TOP3B | DNA Topoisomerase III Beta | Protein Coding | 36 | GC22M021957 | 3.067961216 |
| PCDH8 | Protocadherin 8 | Protein Coding | 36 | GC13M052842 | 3.067013741 |
| BAGE3 | BAGE Family Member 3 | Protein Coding | 15 | GC21U900493 | 3.066934347 |
| TEX15 | Testis Expressed 15, Meiosis And Synapsis Associated | Protein Coding | 31 | GC08M030808 | 3.066779137 |
| PNKP | Polynucleotide Kinase 3'-Phosphatase | Protein Coding | 41 | GC19M064132 | 3.066669941 |
| SLC20A1 | Solute Carrier Family 20 Member 1 | Protein Coding | 40 | GC02P121635 | 3.065528393 |
| ATP1B1 | ATPase Na+/K+ Transporting Subunit Beta 1 | Protein Coding | 43 | GC01P169105 | 3.065286875 |
| MYH6 | Myosin Heavy Chain 6 | Protein Coding | 42 | GC14M023381 | 3.064663649 |
| MAMDC4 | MAM Domain Containing 4 | Protein Coding | 28 | GC09P136850 | 3.064237595 |
| LORICRIN | Loricrin Cornified Envelope Precursor Protein | Protein Coding | 27 | GC01P153262 | 3.064119339 |
| YY1AP1 | YY1 Associated Protein 1 | Protein Coding | 35 | GC01M155659 | 3.063656092 |
| UNC45B | Unc-45 Myosin Chaperone B | Protein Coding | 36 | GC17P052724 | 3.063373089 |
| SLC12A3 | Solute Carrier Family 12 Member 3 | Protein Coding | 43 | GC16P056865 | 3.063101292 |
| PLCB2 | Phospholipase C Beta 2 | Protein Coding | 42 | GC15M040278 | 3.062788963 |
| FBXW2 | F-Box And WD Repeat Domain Containing 2 | Protein Coding | 32 | GC09M120751 | 3.062629938 |
| MT-TF | Mitochondrially Encoded TRNA-Phe (UUU/C) | RNA Gene | 13 | GCMTP000580 | 3.062434196 |
| UHMK1 | U2AF Homology Motif Kinase 1 | Protein Coding | 36 | GC01P162467 | 3.061804771 |
| DYNLRB2 | Dynein Light Chain Roadblock-Type 2 | Protein Coding | 35 | GC16P080540 | 3.061504126 |
| DPYSL2 | Dihydropyrimidinase Like 2 | Protein Coding | 40 | GC08P026514 | 3.0609231 |
| LAMP3 | Lysosomal Associated Membrane Protein 3 | Protein Coding | 35 | GC03M183122 | 3.060167551 |
| ANXA11 | Annexin A11 | Protein Coding | 40 | GC10M080150 | 3.059877396 |
| GIGYF2 | GRB10 Interacting GYF Protein 2 | Protein Coding | 35 | GC02P232698 | 3.059873104 |
| GABRA4 | Gamma-Aminobutyric Acid Type A Receptor Subunit Alpha4 | Protein Coding | 41 | GC04M046836 | 3.058561325 |
| MED13L | Mediator Complex Subunit 13L | Protein Coding | 35 | GC12M115953 | 3.058383465 |
| KIAA0753 | KIAA0753 | Protein Coding | 30 | GC17M006578 | 3.057025909 |
| PPOX | Protoporphyrinogen Oxidase | Protein Coding | 39 | GC01P161322 | 3.055382252 |
| RPL14 | Ribosomal Protein L14 | Protein Coding | 34 | GC03P040458 | 3.054849863 |
| DCTN5 | Dynactin Subunit 5 | Protein Coding | 32 | GC16P024181 | 3.053391457 |
| UNC119 | Unc-119 Lipid Binding Chaperone | Protein Coding | 38 | GC17M028546 | 3.053156376 |
| SLC12A2 | Solute Carrier Family 12 Member 2 | Protein Coding | 45 | GC05P128083 | 3.053017139 |
| TMEM176A | Transmembrane Protein 176A | Protein Coding | 30 | GC07P150800 | 3.051147938 |
| SNTB2 | Syntrophin Beta 2 | Protein Coding | 34 | GC16P069187 | 3.049997568 |
| ATP2B4 | ATPase Plasma Membrane Ca2+ Transporting 4 | Protein Coding | 40 | GC01P203626 | 3.049726963 |
| PPIB | Peptidylprolyl Isomerase B | Protein Coding | 45 | GC15M064155 | 3.049368143 |
| MYOF | Myoferlin | Protein Coding | 39 | GC10M093306 | 3.049338341 |

| | | | | | |
|---|---|---|---|---|---|
| MAST2 | Microtubule Associated Serine/Threonine Kinase 2 | Protein Coding | 36 | GC01P045786 | 3.046970606 |
| ARHGEF7 | Rho Guanine Nucleotide Exchange Factor 7 | Protein Coding | 37 | GC13P111114 | 3.046376705 |
| CALB1 | Calbindin 1 | Protein Coding | 38 | GC08M090058 | 3.045982122 |
| GABARAPL1 | GABA Type A Receptor Associated Protein Like 1 | Protein Coding | 36 | GC12P010212 | 3.044535398 |
| POTEE | POTE Ankyrin Domain Family Member E | Protein Coding | 28 | GC02P136493 | 3.044389248 |
| IQGAP3 | IQ Motif Containing GTPase Activating Protein 3 | Protein Coding | 34 | GC01M156525 | 3.044335365 |
| H2AC6 | H2A Clustered Histone 6 | Protein Coding | 26 | GC06P081032 | 3.043317556 |
| FOXF2 | Forkhead Box F2 | Protein Coding | 32 | GC06P001390 | 3.042684555 |
| UQCC1 | Ubiquinol-Cytochrome C Reductase Complex Assembly Factor 1 | Protein Coding | 29 | GC20M035302 | 3.042183876 |
| EME1 | Essential Meiotic Structure-Specific Endonuclease 1 | Protein Coding | 35 | GC17P050373 | 3.041907787 |
| DIS3 | DIS3 Homolog, Exosome Endoribonuclease And 3'-5' Exoribonuclease | Protein Coding | 37 | GC13M072752 | 3.041725159 |
| NPHS1 | NPHS1 Adhesion Molecule, Nephrin | Protein Coding | 41 | GC19M035825 | 3.041509151 |
| NEK1 | NIMA Related Kinase 1 | Protein Coding | 40 | GC04M169393 | 3.040530682 |
| BCAM | Basal Cell Adhesion Molecule (Lutheran Blood Group) | Protein Coding | 36 | GC19P063841 | 3.039484978 |
| THSD4 | Thrombospondin Type 1 Domain Containing 4 | Protein Coding | 33 | GC15P071096 | 3.03916955 |
| ZFYVE9 | Zinc Finger FYVE-Type Containing 9 | Protein Coding | 37 | GC01P052142 | 3.038538694 |
| EVC | EvC Ciliary Complex Subunit 1 | Protein Coding | 34 | GC04P005712 | 3.038509369 |
| RPL24 | Ribosomal Protein L24 | Protein Coding | 35 | GC03M101681 | 3.037809372 |
| DTL | Denticleless E3 Ubiquitin Protein Ligase Homolog | Protein Coding | 34 | GC01P212035 | 3.037765741 |
| GUCY2F | Guanylate Cyclase 2F, Retinal | Protein Coding | 34 | GC0XM109372 | 3.037659645 |
| PSMA5 | Proteasome 20S Subunit Alpha 5 | Protein Coding | 38 | GC01M109399 | 3.036711454 |
| FRMPD1 | FERM And PDZ Domain Containing 1 | Protein Coding | 30 | GC09P046292 | 3.03655529 |
| URB2 | URB2 Ribosome Biogenesis Homolog | Protein Coding | 30 | GC01P229626 | 3.036488533 |
| SRRT | Serrate, RNA Effector Molecule | Protein Coding | 35 | GC07P100997 | 3.034103394 |
| HAT1 | Histone Acetyltransferase 1 | Protein Coding | 39 | GC02P171922 | 3.032871962 |
| RPL10A | Ribosomal Protein L10a | Protein Coding | 37 | GC06P080514 | 3.031725407 |
| GTF2IRD1 | GTF2I Repeat Domain Containing 1 | Protein Coding | 38 | GC07P074824 | 3.0259583 |
| UFC1 | Ubiquitin-Fold Modifier Conjugating Enzyme 1 | Protein Coding | 33 | GC01P161312 | 3.025658846 |
| SIRT4 | Sirtuin 4 | Protein Coding | 38 | GC12P120291 | 3.024677038 |
| RTKN | Rhotekin | Protein Coding | 35 | GC02M075035 | 3.024414778 |
| PIGO | Phosphatidylinositol Glycan Anchor Biosynthesis Class O | Protein Coding | 37 | GC09M035088 | 3.022963285 |
| CNGB3 | Cyclic Nucleotide Gated Channel Subunit Beta 3 | Protein Coding | 37 | GC08M086553 | 3.020954609 |
| GOLIM4 | Golgi Integral Membrane Protein 4 | Protein Coding | 31 | GC03M168008 | 3.020506382 |
| GNAI1 | G Protein Subunit Alpha I1 | Protein Coding | 41 | GC07P079769 | 3.019683599 |
| PZP | PZP Alpha-2-Macroglobulin Like | Protein Coding | 38 | GC12M009148 | 3.01930809 |
| H3C4 | H3 Clustered Histone 4 | Protein Coding | 28 | GC06M064164 | 3.019304276 |
| PPP2CB | Protein Phosphatase 2 Catalytic Subunit Beta | Protein Coding | 41 | GC08M030762 | 3.018330574 |
| DNER | Delta/Notch Like EGF Repeat Containing | Protein Coding | 36 | GC02M229357 | 3.018253803 |
| ADCY1 | Adenylate Cyclase 1 | Protein Coding | 44 | GC07P045580 | 3.017948151 |
| KDM8 | Lysine Demethylase 8 | Protein Coding | 31 | GC16P027817 | 3.017881632 |
| ANAPC4 | Anaphase Promoting Complex Subunit 4 | Protein Coding | 30 | GC04P025379 | 3.017139435 |
| LPIN1 | Lipin 1 | Protein Coding | 42 | GC02P011677 | 3.015858889 |
| MAEA | Macrophage Erythroblast Attacher, E3 Ubiquitin Ligase | Protein Coding | 34 | GC04P001289 | 3.015773058 |
| CTH | Cystathionine Gamma-Lyase | Protein Coding | 44 | GC01P070411 | 3.015400171 |
| DTX3L | Deltex E3 Ubiquitin Ligase 3L | Protein Coding | 32 | GC03P122564 | 3.015298128 |
| PMVK | Phosphomevalonate Kinase | Protein Coding | 38 | GC01M154924 | 3.014976263 |
| RFX2 | Regulatory Factor X2 | Protein Coding | 37 | GC19M005993 | 3.014493942 |
| MMS19 | MMS19 Homolog, Cytosolic Iron-Sulfur Assembly Component | Protein Coding | 32 | GC10M097458 | 3.013739586 |
| MRPS30 | Mitochondrial Ribosomal Protein S30 | Protein Coding | 33 | GC05P044808 | 3.013081074 |
| GLRX3 | Glutaredoxin 3 | Protein Coding | 36 | GC10P130136 | 3.012703896 |
| TCL1B | TCL1 Family AKT Coactivator B | Protein Coding | 35 | GC14P095686 | 3.0119524 |
| AP1M1 | Adaptor Related Protein Complex 1 Subunit Mu 1 | Protein Coding | 36 | GC19P063081 | 3.011549234 |
| SPRY1 | Sprouty RTK Signaling Antagonist 1 | Protein Coding | 38 | GC04P123396 | 3.010934591 |
| TNPO3 | Transportin 3 | Protein Coding | 36 | GC07M128954 | 3.010242224 |
| S1PR3 | Sphingosine-1-Phosphate Receptor 3 | Protein Coding | 40 | GC09P089224 | 3.010159492 |
| DHRS11 | Dehydrogenase/Reductase 11 | Protein Coding | 32 | GC17P052841 | 3.010056496 |
| GNAI3 | G Protein Subunit Alpha I3 | Protein Coding | 41 | GC01P109548 | 3.008237362 |
| CAPG | Capping Actin Protein, Gelsolin Like | Protein Coding | 36 | GC02M085394 | 3.008191586 |
| TOR1AIP1 | Torsin 1A Interacting Protein 1 | Protein Coding | 36 | GC01P179882 | 3.006786823 |
| AUTS2 | Activator Of Transcription And Developmental Regulator AUTS2 | Protein Coding | 37 | GC07P069598 | 3.005352497 |
| SLC25A16 | Solute Carrier Family 25 Member 16 | Protein Coding | 34 | GC10M068477 | 3.005101919 |
| ADRM1 | ADRM1 26S Proteasome Ubiquitin Receptor | Protein Coding | 34 | GC20P062302 | 3.004952431 |
| CSDE1 | Cold Shock Domain Containing E1 | Protein Coding | 34 | GC01M114716 | 3.004698038 |
| MTMR10 | Myotubularin Related Protein 10 | Protein Coding | 31 | GC15M030919 | 3.004667759 |
| NCR3 | Natural Cytotoxicity Triggering Receptor 3 | Protein Coding | 35 | GC06M031588 | 3.004188776 |
| COG3 | Component Of Oligomeric Golgi Complex 3 | Protein Coding | 32 | GC13P045464 | 3.003372192 |
| AGTR2 | Angiotensin II Receptor Type 2 | Protein Coding | 41 | GC0XP116170 | 3.003280163 |
| CTPS1 | CTP Synthase 1 | Protein Coding | 41 | GC01P040979 | 3.002977371 |
| MARCKSL1 | MARCKS Like 1 | Protein Coding | 34 | GC01M032334 | 3.002766371 |
| ADORA1 | Adenosine A1 Receptor | Protein Coding | 42 | GC01P203090 | 3.002680779 |
| MMP19 | Matrix Metallopeptidase 19 | Protein Coding | 44 | GC12M055835 | 3.00224328 |
| POGLUT1 | Protein O-Glucosyltransferase 1 | Protein Coding | 36 | GC03P119468 | 3.000596046 |
| GSX1 | GS Homeobox 1 | Protein Coding | 31 | GC13P027792 | 2.999310732 |
| PLPP5 | Phospholipid Phosphatase 5 | Protein Coding | 28 | GC08M038261 | 2.998737097 |
| FABP1 | Fatty Acid Binding Protein 1 | Protein Coding | 39 | GC02M088122 | 2.998008251 |
| POLD2 | DNA Polymerase Delta 2, Accessory Subunit | Protein Coding | 35 | GC07M044114 | 2.997585773 |
| GGA1 | Golgi Associated, Gamma Adaptin Ear Containing, ARF Binding Protein 1 | Protein Coding | 33 | GC22P037608 | 2.99733305 |
| PRKD2 | Protein Kinase D2 | Protein Coding | 41 | GC19M046674 | 2.996912718 |
| NOMO3 | NODAL Modulator 3 | Protein Coding | 28 | GC16P016232 | 2.996452808 |
| MAS1 | MAS1 Proto-Oncogene, G Protein-Coupled Receptor | Protein Coding | 34 | GC06P160439 | 2.996389151 |
| CEACAM19 | CEA Cell Adhesion Molecule 19 | Protein Coding | 29 | GC19P063831 | 2.996203423 |
| LNCNEF | LncRNA Neighboring Enhancer Of FOXA2 | RNA Gene | 12 | GC20M022588 | 2.995888233 |
| MYO7A | Myosin VIIA | Protein Coding | 39 | GC11P077128 | 2.99578619 |
| CCNO | Cyclin O | Protein Coding | 38 | GC05M055231 | 2.99479866 |
| P2RY13 | Purinergic Receptor P2Y13 | Protein Coding | 37 | GC03M151326 | 2.994130611 |
| PCDH15 | Protocadherin Related 15 | Protein Coding | 37 | GC10M053802 | 2.993369579 |
| TNFRSF18 | TNF Receptor Superfamily Member 18 | Protein Coding | 37 | GC01M001203 | 2.992921829 |
| TRIO | Trio Rho Guanine Nucleotide Exchange Factor | Protein Coding | 40 | GC05P014143 | 2.992530346 |
| PHOX2B-AS1 | PHOX2B Antisense RNA 1 | RNA Gene | 10 | GC04P041755 | 2.991782427 |
| CCT5 | Chaperonin Containing TCP1 Subunit 5 | Protein Coding | 40 | GC05P010236 | 2.990447998 |
| MBD3 | Methyl-CpG Binding Domain Protein 3 | Protein Coding | 37 | GC19M004916 | 2.990208626 |
| H3C12 | H3 Clustered Histone 12 | Protein Coding | 27 | GC06M064161 | 2.988516808 |
| BDNF-AS | BDNF Antisense RNA | RNA Gene | 19 | GC11P027549 | 2.9884727 |
| OSBPL11 | Oxysterol Binding Protein Like 11 | Protein Coding | 32 | GC03M125529 | 2.986988544 |
| ABCF1 | ATP Binding Cassette Subfamily F Member 1 | Protein Coding | 35 | GC06P030571 | 2.985971928 |

| | | | | | |
|---|---|---|---|---|---|
| MNT | MAX Network Transcriptional Repressor | Protein Coding | 32 | GC17M002384 | 2.98453474 |
| MBD6 | Methyl-CpG Binding Domain Protein 6 | Protein Coding | 32 | GC12P057520 | 2.983153582 |
| RAB27B | RAB27B, Member RAS Oncogene Family | Protein Coding | 34 | GC18P054717 | 2.983087063 |
| ATG4B | Autophagy Related 4B Cysteine Peptidase | Protein Coding | 37 | GC02P241637 | 2.982928514 |
| CDH16 | Cadherin 16 | Protein Coding | 36 | GC16M066908 | 2.982109785 |
| MMACHC | Metabolism Of Cobalamin Associated C | Protein Coding | 38 | GC01P045500 | 2.981279135 |
| SNRNP70 | Small Nuclear Ribonucleoprotein U1 Subunit 70 | Protein Coding | 34 | GC19P049085 | 2.981001377 |
| DRD4 | Dopamine Receptor D4 | Protein Coding | 42 | GC11P001664 | 2.980924129 |
| XPNPEP3 | X-Prolyl Aminopeptidase 3 | Protein Coding | 38 | GC22P040857 | 2.979798317 |
| NBPF10 | NBPF Member 10 | Protein Coding | 23 | GC01M146064 | 2.979158878 |
| IDH3B | Isocitrate Dehydrogenase (NAD(+)) 3 Non-Catalytic Subunit Beta | Protein Coding | 40 | GC20M002658 | 2.979043007 |
| ADHFE1 | Alcohol Dehydrogenase Iron Containing 1 | Protein Coding | 34 | GC08P066432 | 2.97765398 |
| SCGB3A2 | Secretoglobin Family 3A Member 2 | Protein Coding | 35 | GC05P147870 | 2.977420807 |
| PIGG | Phosphatidylinositol Glycan Anchor Biosynthesis Class G | Protein Coding | 35 | GC04P000758 | 2.977368116 |
| NELFB | Negative Elongation Factor Complex Member B | Protein Coding | 30 | GC09P137255 | 2.976359367 |
| N4BP2L1 | NEDD4 Binding Protein 2 Like 1 | Protein Coding | 27 | GC13M036305 | 2.976179123 |
| UACA | Uveal Autoantigen With Coiled-Coil Domains And Ankyrin Repeats | Protein Coding | 34 | GC15M070654 | 2.97481966 |
| SERHL | Serine Hydrolase Like (Pseudogene) | Pseudogene | 22 | GC22P042500 | 2.974173069 |
| PLEKHA8P1 | Pleckstrin Homology Domain Containing A8 Pseudogene 1 | Pseudogene | 19 | GC12M045174 | 2.973954201 |
| NCKAP1L | NCK Associated Protein 1 Like | Protein Coding | 34 | GC12P054497 | 2.973278046 |
| SCN1A | Sodium Voltage-Gated Channel Alpha Subunit 1 | Protein Coding | 43 | GC02M165989 | 2.973237038 |
| MT3 | Metallothionein 3 | Protein Coding | 35 | GC16P056589 | 2.97270155 |
| LINC00173 | Long Intergenic Non-Protein Coding RNA 173 | RNA Gene | 19 | GC12P116533 | 2.972001076 |
| INPP5K | Inositol Polyphosphate-5-Phosphatase K | Protein Coding | 39 | GC17M001494 | 2.971997738 |
| KCNJ3 | Potassium Inwardly Rectifying Channel Subfamily J Member 3 | Protein Coding | 42 | GC02P154698 | 2.971803188 |
| SHARPIN | SHANK Associated RH Domain Interactor | Protein Coding | 36 | GC08M144098 | 2.971066713 |
| CAMK2B | Calcium/Calmodulin Dependent Protein Kinase II Beta | Protein Coding | 44 | GC07M044217 | 2.970318317 |
| H2AC13 | H2A Clustered Histone 13 | Protein Coding | 28 | GC06P079940 | 2.969906807 |
| ZNF764 | Zinc Finger Protein 764 | Protein Coding | 30 | GC16M030553 | 2.969858646 |
| C19orf12 | Chromosome 19 Open Reading Frame 12 | Protein Coding | 32 | GC19M032761 | 2.969748974 |
| MYO18A | Myosin XVIIIA | Protein Coding | 35 | GC17M034591 | 2.96950388 |
| PARP2 | Poly(ADP-Ribose) Polymerase 2 | Protein Coding | 40 | GC14P020343 | 2.968345642 |
| NDUFS4 | NADH:Ubiquinone Oxidoreductase Subunit S4 | Protein Coding | 39 | GC05P053560 | 2.967610836 |
| GGCT | Gamma-Glutamylcyclotransferase | Protein Coding | 34 | GC07M030496 | 2.967391491 |
| TTC9 | Tetratricopeptide Repeat Domain 9 | Protein Coding | 27 | GC14P070641 | 2.967323303 |
| IRX1 | Iroquois Homeobox 1 | Protein Coding | 35 | GC05P003596 | 2.967273712 |
| ENOX1 | Ecto-NOX Disulfide-Thiol Exchanger 1 | Protein Coding | 33 | GC13M043213 | 2.967122555 |
| ATOX1 | Antioxidant 1 Copper Chaperone | Protein Coding | 35 | GC05M151743 | 2.967026234 |
| HNF4G | Hepatocyte Nuclear Factor 4 Gamma | Protein Coding | 39 | GC08P075407 | 2.965249538 |
| PCDH7 | Protocadherin 7 | Protein Coding | 36 | GC04P030722 | 2.964711189 |
| MIR654 | MicroRNA 654 | RNA Gene | 17 | GC14P109547 | 2.964452028 |
| DMP1 | Dentin Matrix Acidic Phosphoprotein 1 | Protein Coding | 39 | GC04P087650 | 2.964079142 |
| XIRP1 | Xin Actin Binding Repeat Containing 1 | Protein Coding | 32 | GC03M039200 | 2.963822365 |
| AP4B1 | Adaptor Related Protein Complex 4 Subunit Beta 1 | Protein Coding | 37 | GC01M113894 | 2.963748932 |
| SCUBE2 | Signal Peptide, CUB Domain And EGF Like Domain Containing 2 | Protein Coding | 36 | GC11M009019 | 2.9636271 |
| ECI2 | Enoyl-CoA Delta Isomerase 2 | Protein Coding | 35 | GC06M004115 | 2.962329388 |
| RBMS1 | RNA Binding Motif Single Stranded Interacting Protein 1 | Protein Coding | 35 | GC02M160272 | 2.962164402 |
| HYAL2 | Hyaluronidase 2 | Protein Coding | 40 | GC03M050317 | 2.960332394 |
| ILF2 | Interleukin Enhancer Binding Factor 2 | Protein Coding | 34 | GC01M153661 | 2.95934248 |
| RO60 | Ro60, Y RNA Binding Protein | Protein Coding | 30 | GC01P193059 | 2.959075451 |
| DNAJC7 | DnaJ Heat Shock Protein Family (Hsp40) Member C7 | Protein Coding | 34 | GC17M041977 | 2.958703279 |
| PIP4K2B | Phosphatidylinositol-5-Phosphate 4-Kinase Type 2 Beta | Protein Coding | 36 | GC17M038765 | 2.957455635 |
| CKS2 | CDC28 Protein Kinase Regulatory Subunit 2 | Protein Coding | 34 | GC09P089311 | 2.956930161 |
| PPP4R3A | Protein Phosphatase 4 Regulatory Subunit 3A | Protein Coding | 28 | GC14M100411 | 2.956920624 |
| KLK12 | Kallikrein Related Peptidase 12 | Protein Coding | 34 | GC19M064225 | 2.956683636 |
| NR2C2 | Nuclear Receptor Subfamily 2 Group C Member 2 | Protein Coding | 39 | GC03P014947 | 2.956140041 |
| GPD1 | Glycerol-3-Phosphate Dehydrogenase 1 | Protein Coding | 39 | GC12P050116 | 2.955947638 |
| KCTD12 | Potassium Channel Tetramerization Domain Containing 12 | Protein Coding | 32 | GC13M076880 | 2.955734253 |
| ANAPC7 | Anaphase Promoting Complex Subunit 7 | Protein Coding | 35 | GC12M110372 | 2.955639362 |
| NDUFAF4 | NADH:Ubiquinone Oxidoreductase Complex Assembly Factor 4 | Protein Coding | 38 | GC06M096889 | 2.954573154 |
| CRLF3 | Cytokine Receptor Like Factor 3 | Protein Coding | 33 | GC17M030769 | 2.954339027 |
| RHOXF2 | Rhox Homeobox Family Member 2 | Protein Coding | 28 | GC0XP120158 | 2.954033852 |
| RTRAF | RNA Transcription, Translation And Transport Factor | Protein Coding | 29 | GC14P051992 | 2.954012632 |
| HARS1 | Histidyl-TRNA Synthetase 1 | Protein Coding | 34 | GC05M140673 | 2.953124523 |
| MYCBP2 | MYC Binding Protein 2 | Protein Coding | 33 | GC13M077044 | 2.951803923 |
| PPA2 | Inorganic Pyrophosphatase 2 | Protein Coding | 37 | GC04M105369 | 2.951720238 |
| PMPCA | Peptidase, Mitochondrial Processing Subunit Alpha | Protein Coding | 39 | GC09P136410 | 2.951680183 |
| IRF2 | Interferon Regulatory Factor 2 | Protein Coding | 39 | GC04M184387 | 2.951482296 |
| DNAJB6 | DnaJ Heat Shock Protein Family (Hsp40) Member B6 | Protein Coding | 38 | GC07P157335 | 2.951392651 |
| PLEKHM1 | Pleckstrin Homology And RUN Domain Containing M1 | Protein Coding | 37 | GC17M045435 | 2.951385498 |
| PLEKHS1 | Pleckstrin Homology Domain Containing S1 | Protein Coding | 26 | GC10P113751 | 2.950688124 |
| ITPKB | Inositol-Trisphosphate 3-Kinase B | Protein Coding | 39 | GC01M226631 | 2.94975996 |
| GDPD2 | Glycerophosphodiester Phosphodiesterase Domain Containing 2 | Protein Coding | 32 | GC0XP070423 | 2.948869705 |
| DCAF7 | DDB1 And CUL4 Associated Factor 7 | Protein Coding | 31 | GC17P063550 | 2.947792292 |
| RUFY1 | RUN And FYVE Domain Containing 1 | Protein Coding | 34 | GC05P179550 | 2.947740555 |
| HRNR | Hornerin | Protein Coding | 32 | GC01M152184 | 2.947572231 |
| PYGL | Glycogen Phosphorylase L | Protein Coding | 42 | GC14M050857 | 2.946229219 |
| OSGEP | O-Sialoglycoprotein Endopeptidase | Protein Coding | 38 | GC14M020446 | 2.946111202 |
| SPATA18 | Spermatogenesis Associated 18 | Protein Coding | 30 | GC04P052051 | 2.945621252 |
| PSMD14 | Proteasome 26S Subunit, Non-ATPase 14 | Protein Coding | 38 | GC02P161308 | 2.945349693 |
| ERV3-1 | Endogenous Retrovirus Group 3 Member 1, Envelope | Protein Coding | 28 | GC07M065602 | 2.94425106 |
| RPL38 | Ribosomal Protein L38 | Protein Coding | 33 | GC17P074204 | 2.94415617 |
| SPIB | Spi-B Transcription Factor | Protein Coding | 35 | GC19P050418 | 2.943977833 |
| ORC2 | Origin Recognition Complex Subunit 2 | Protein Coding | 34 | GC02M200908 | 2.943408012 |
| KCNN3 | Potassium Calcium-Activated Channel Subfamily N Member 3 | Protein Coding | 40 | GC01M154697 | 2.943247795 |
| SACS | Sacsin Molecular Chaperone | Protein Coding | 34 | GC13M023288 | 2.942775249 |
| MIR548C | MicroRNA 548c | RNA Gene | 14 | GC12P064622 | 2.942336559 |
| LY6G6C | Lymphocyte Antigen 6 Family Member G6C | Protein Coding | 31 | GC06M063618 | 2.941741943 |
| FZD9 | Frizzled Class Receptor 9 | Protein Coding | 40 | GC07P073433 | 2.941631556 |
| NPAP1 | Nuclear Pore Associated Protein 1 | Protein Coding | 26 | GC15P024675 | 2.940909147 |
| KBTBD8 | Kelch Repeat And BTB Domain Containing 8 | Protein Coding | 32 | GC03P066998 | 2.940659046 |
| CAPN10 | Calpain 10 | Protein Coding | 37 | GC02P240586 | 2.940348864 |
| EPYC | Epiphycan | Protein Coding | 32 | GC12M090963 | 2.939630508 |
| PURA | Purine Rich Element Binding Protein A | Protein Coding | 40 | GC05P140076 | 2.939513206 |
| APCS | Amyloid P Component, Serum | Protein Coding | 39 | GC01P159587 | 2.939489841 |

| | | | | | |
|---|---|---|---|---|---|
| FOXD1 | Forkhead Box D1 | Protein Coding | 32 | GC05M073444 | 2.939053297 |
| SSPN | Sarcospan | Protein Coding | 32 | GC12P026118 | 2.938754082 |
| MIR379 | MicroRNA 379 | RNA Gene | 16 | GC14P109528 | 2.938251972 |
| PDE4DIP | Phosphodiesterase 4D Interacting Protein | Protein Coding | 37 | GC01P148808 | 2.93802309 |
| MED7 | Mediator Complex Subunit 7 | Protein Coding | 32 | GC05M157137 | 2.937740803 |
| SPINK5 | Serine Peptidase Inhibitor Kazal Type 5 | Protein Coding | 38 | GC05P148025 | 2.937246323 |
| AP2A1 | Adaptor Related Protein Complex 2 Subunit Alpha 1 | Protein Coding | 35 | GC19P064091 | 2.936925411 |
| ANAPC16 | Anaphase Promoting Complex Subunit 16 | Protein Coding | 30 | GC10P072278 | 2.936773777 |
| NSMCE2 | NSE2 (MMS21) Homolog, SMC5-SMC6 Complex SUMO Ligase | Protein Coding | 35 | GC08P125091 | 2.935619831 |
| FCRL3 | Fc Receptor Like 3 | Protein Coding | 34 | GC01M157674 | 2.935454845 |
| NRF1 | Nuclear Respiratory Factor 1 | Protein Coding | 38 | GC07P129611 | 2.935004711 |
| CNR2 | Cannabinoid Receptor 2 | Protein Coding | 40 | GC01M023870 | 2.933666468 |
| IFT140 | Intraflagellar Transport 140 | Protein Coding | 36 | GC16M006658 | 2.932385445 |
| CYBRD1 | Cytochrome B Reductase 1 | Protein Coding | 35 | GC02P171522 | 2.930805445 |
| DDX21 | DExD-Box Helicase 21 | Protein Coding | 34 | GC10P068956 | 2.930721283 |
| ACSM1 | Acyl-CoA Synthetase Medium Chain Family Member 1 | Protein Coding | 35 | GC16M020634 | 2.930140495 |
| NLRP9 | NLR Family Pyrin Domain Containing 9 | Protein Coding | 31 | GC19M064439 | 2.929783106 |
| ERBIN | Erbb2 Interacting Protein | Protein Coding | 32 | GC05P065931 | 2.929712296 |
| SPAG17 | Sperm Associated Antigen 17 | Protein Coding | 31 | GC01M117953 | 2.929278851 |
| EFNA5 | Ephrin A5 | Protein Coding | 39 | GC05M107376 | 2.928958178 |
| PREPL | Prolyl Endopeptidase Like | Protein Coding | 38 | GC02M044281 | 2.927121162 |
| FOXE3 | Forkhead Box E3 | Protein Coding | 35 | GC01P047416 | 2.925116301 |
| AGL | Amylo-Alpha-1, 6-Glucosidase, 4-Alpha-Glucanotransferase | Protein Coding | 40 | GC01P099850 | 2.924598932 |
| EIPR1 | EARP Complex And GARP Complex Interacting Protein 1 | Protein Coding | 27 | GC02M003188 | 2.923760414 |
| CHPT1 | Choline Phosphotransferase 1 | Protein Coding | 36 | GC12P101696 | 2.922701836 |
| EMP1 | Epithelial Membrane Protein 1 | Protein Coding | 35 | GC12P013196 | 2.92214179 |
| ST3GAL3 | ST3 Beta-Galactoside Alpha-2,3-Sialyltransferase 3 | Protein Coding | 40 | GC01P043705 | 2.921861172 |
| EPB41 | Erythrocyte Membrane Protein Band 4.1 | Protein Coding | 42 | GC01P028887 | 2.921667576 |
| TRIP12 | Thyroid Hormone Receptor Interactor 12 | Protein Coding | 39 | GC02M229763 | 2.920048237 |
| TNFSF9 | TNF Superfamily Member 9 | Protein Coding | 32 | GC19P006531 | 2.91998291 |
| VPS13A | Vacuolar Protein Sorting 13 Homolog A | Protein Coding | 36 | GC09P077177 | 2.919698715 |
| TUBB4B | Tubulin Beta 4B Class IVb | Protein Coding | 39 | GC09P137241 | 2.919554472 |
| CECR7 | Cat Eye Syndrome Chromosome Region, Candidate 7 | RNA Gene | 17 | GC22P018389 | 2.918888569 |
| MT-ND6 | Mitochondrially Encoded NADH:Ubiquinone Oxidoreductase Core Subunit 6 | Protein Coding | 29 | GCMTM014151 | 2.918328762 |
| DHX32 | DEAH-Box Helicase 32 (Putative) | Protein Coding | 32 | GC10M125836 | 2.916983366 |
| CORO1C | Coronin 1C | Protein Coding | 35 | GC12M108645 | 2.916454792 |
| NDE1 | NudE Neurodevelopment Protein 1 | Protein Coding | 38 | GC16P017317 | 2.915747166 |
| SERPINA5 | Serpin Family A Member 5 | Protein Coding | 39 | GC14P094563 | 2.914983511 |
| PHF3 | PHD Finger Protein 3 | Protein Coding | 33 | GC06P063635 | 2.914407253 |
| CST4 | Cystatin S | Protein Coding | 32 | GC20M023685 | 2.913997889 |
| TCN1 | Transcobalamin 1 | Protein Coding | 38 | GC11M087019 | 2.913950443 |
| ROPN1 | Rhophilin Associated Tail Protein 1 | Protein Coding | 30 | GC03M123968 | 2.913638592 |
| MCM3AP-AS1 | MCM3AP Antisense RNA 1 | RNA Gene | 18 | GC21P046229 | 2.912668705 |
| MIR374B | MicroRNA 374b | RNA Gene | 15 | GC0XM074227 | 2.912658691 |
| RUNDC3B | RUN Domain Containing 3B | Protein Coding | 28 | GC07P087627 | 2.912327766 |
| FMNL3 | Formin Like 3 | Protein Coding | 32 | GC12M049636 | 2.91224575 |
| SLC22A4 | Solute Carrier Family 22 Member 4 | Protein Coding | 40 | GC05P132294 | 2.911839485 |
| DAP3 | Death Associated Protein 3 | Protein Coding | 35 | GC01P155775 | 2.910987139 |
| PKDREJ | Polycystin Family Receptor For Egg Jelly | Protein Coding | 30 | GC22M046255 | 2.90982461 |
| TTC36 | Tetratricopeptide Repeat Domain 36 | Protein Coding | 28 | GC11P118527 | 2.909376144 |
| AGGF1 | Angiogenic Factor With G-Patch And FHA Domains 1 | Protein Coding | 37 | GC05P077029 | 2.909367561 |
| HSD17B10 | Hydroxysteroid 17-Beta Dehydrogenase 10 | Protein Coding | 40 | GC0XM053431 | 2.909343243 |
| SIM2 | SIM BHLH Transcription Factor 2 | Protein Coding | 35 | GC21P036699 | 2.909156799 |
| PSMA2 | Proteasome 20S Subunit Alpha 2 | Protein Coding | 38 | GC07M042916 | 2.908760071 |
| PRR12 | Proline Rich 12 | Protein Coding | 28 | GC19P049591 | 2.908490419 |
| RGS2 | Regulator Of G Protein Signaling 2 | Protein Coding | 38 | GC01P192809 | 2.908108711 |
| RBFOX2 | RNA Binding Fox-1 Homolog 2 | Protein Coding | 35 | GC22M035738 | 2.907152653 |
| UBR1 | Ubiquitin Protein Ligase E3 Component N-Recognin 1 | Protein Coding | 39 | GC15M042942 | 2.906545162 |
| ACAP1 | ArfGAP With Coiled-Coil, Ankyrin Repeat And PH Domains 1 | Protein Coding | 35 | GC17P010866 | 2.906252623 |
| MRPS7 | Mitochondrial Ribosomal Protein S7 | Protein Coding | 34 | GC17P075531 | 2.905625105 |
| DUSP28 | Dual Specificity Phosphatase 28 | Protein Coding | 27 | GC02P240560 | 2.90534687 |
| LRRC7 | Leucine Rich Repeat Containing 7 | Protein Coding | 35 | GC01P069567 | 2.903219223 |
| ABCG8 | ATP Binding Cassette Subfamily G Member 8 | Protein Coding | 41 | GC02P043828 | 2.903209209 |
| SLC22A1 | Solute Carrier Family 22 Member 1 | Protein Coding | 39 | GC06P160121 | 2.901875973 |
| SLC30A8 | Solute Carrier Family 30 Member 8 | Protein Coding | 38 | GC08P116950 | 2.901740074 |
| FCHO1 | FCH And Mu Domain Containing Endocytic Adaptor 1 | Protein Coding | 33 | GC19P063111 | 2.901652098 |
| MAP4 | Microtubule Associated Protein 4 | Protein Coding | 37 | GC03M047850 | 2.901543379 |
| GGA3 | Golgi Associated, Gamma Adaptin Ear Containing, ARF Binding Protein 3 | Protein Coding | 35 | GC17M075225 | 2.901379824 |
| C12orf57 | Chromosome 12 Open Reading Frame 57 | Protein Coding | 32 | GC12P019854 | 2.89996624 |
| COLEC10 | Collectin Subfamily Member 10 | Protein Coding | 36 | GC08P118952 | 2.899610043 |
| DOCK4 | Dedicator Of Cytokinesis 4 | Protein Coding | 36 | GC07M111726 | 2.898852348 |
| GLP1R | Glucagon Like Peptide 1 Receptor | Protein Coding | 42 | GC06P039048 | 2.898144484 |
| ARPC2 | Actin Related Protein 2/3 Complex Subunit 2 | Protein Coding | 36 | GC02P218217 | 2.898018837 |
| VAPB | VAMP Associated Protein B And C | Protein Coding | 40 | GC20P058389 | 2.897716045 |
| STAM2 | Signal Transducing Adaptor Molecule 2 | Protein Coding | 34 | GC02M152116 | 2.897362709 |
| CDCA2 | Cell Division Cycle Associated 2 | Protein Coding | 35 | GC08P025458 | 2.897178888 |
| GFI1B | Growth Factor Independent 1B Transcriptional Repressor | Protein Coding | 38 | GC09P132945 | 2.896504879 |
| KAT7 | Lysine Acetyltransferase 7 | Protein Coding | 38 | GC17P049788 | 2.895930767 |
| SEC14L1 | SEC14 Like Lipid Binding 1 | Protein Coding | 35 | GC17P077086 | 2.895013332 |
| ELP6 | Elongator Acetyltransferase Complex Subunit 6 | Protein Coding | 29 | GC03M047495 | 2.894946814 |
| FASTKD2 | FAST Kinase Domains 2 | Protein Coding | 35 | GC02P206766 | 2.894922018 |
| PCSK9 | Proprotein Convertase Subtilisin/Kexin Type 9 | Protein Coding | 46 | GC01P055039 | 2.893357992 |
| LOC117134608 | TMPRSS2 Androgen Receptor-Responsive -13 Kb Enhancer | Biological Region | 2 | GC21P041519 | 2.89279151 |
| TCEA1 | Transcription Elongation Factor A1 | Protein Coding | 34 | GC08M053966 | 2.892755032 |
| HGS | Hepatocyte Growth Factor-Regulated Tyrosine Kinase Substrate | Protein Coding | 39 | GC17P081683 | 2.891962051 |
| TREM2 | Triggering Receptor Expressed On Myeloid Cells 2 | Protein Coding | 39 | GC06M063805 | 2.891130209 |
| FCN1 | Ficolin 1 | Protein Coding | 36 | GC09M135118 | 2.888628006 |
| TAF6 | TATA-Box Binding Protein Associated Factor 6 | Protein Coding | 35 | GC07M101718 | 2.888411999 |
| RASAL1 | RAS Protein Activator Like 1 | Protein Coding | 34 | GC12M113098 | 2.888314724 |
| AKR1C4 | Aldo-Keto Reductase Family 1 Member C4 | Protein Coding | 43 | GC10P005195 | 2.887336254 |
| ANO6 | Anoctamin 6 | Protein Coding | 37 | GC12P045215 | 2.887061357 |
| NOLC1 | Nucleolar And Coiled-Body Phosphoprotein 1 | Protein Coding | 36 | GC10P102152 | 2.886879921 |
| FLVCR2 | FLVCR Heme Transporter 2 | Protein Coding | 37 | GC14P075578 | 2.886135578 |
| RFC2 | Replication Factor C Subunit 2 | Protein Coding | 39 | GC07M074231 | 2.885787487 |
| H2AC17 | H2A Clustered Histone 17 | Protein Coding | 26 | GC06M064155 | 2.884852409 |

| | | | | | |
|---|---|---|---|---|---|
| PPFIBP1 | PPFIA Binding Protein 1 | Protein Coding | 36 | GC12P027523 | 2.884066105 |
| ANXA4 | Annexin A4 | Protein Coding | 39 | GC02P069644 | 2.883305788 |
| RNF168 | Ring Finger Protein 168 | Protein Coding | 38 | GC03M196468 | 2.882648468 |
| GRK6 | G Protein-Coupled Receptor Kinase 6 | Protein Coding | 40 | GC05P177403 | 2.882395267 |
| RNASE3 | Ribonuclease A Family Member 3 | Protein Coding | 36 | GC14P020891 | 2.881335497 |
| SRP72 | Signal Recognition Particle 72 | Protein Coding | 36 | GC04P056466 | 2.881334066 |
| PDZK1IP1 | PDZK1 Interacting Protein 1 | Protein Coding | 30 | GC01M047183 | 2.881333828 |
| MTHFD2 | Methylenetetrahydrofolate Dehydrogenase (NADP+ Dependent) 2, Methenyltetrahydrofolate Cyclohydrolase | Protein Coding | 36 | GC02P074186 | 2.880500317 |
| FABP12 | Fatty Acid Binding Protein 12 | Protein Coding | 31 | GC08M081524 | 2.880433559 |
| ZNF22 | Zinc Finger Protein 22 | Protein Coding | 30 | GC10P045000 | 2.878929138 |
| PPM1G | Protein Phosphatase, Mg2+/Mn2+ Dependent 1G | Protein Coding | 38 | GC02M028058 | 2.878151417 |
| CGAS | Cyclic GMP-AMP Synthase | Protein Coding | 29 | GC06M073414 | 2.878067017 |
| DERL1 | Derlin 1 | Protein Coding | 32 | GC08M123013 | 2.872694731 |
| CEP250 | Centrosomal Protein 250 | Protein Coding | 35 | GC20P035455 | 2.871928215 |
| LGALS8 | Galectin 8 | Protein Coding | 37 | GC01P236518 | 2.870658875 |
| ETFDH | Electron Transfer Flavoprotein Dehydrogenase | Protein Coding | 42 | GC04P158672 | 2.870395899 |
| LRRFIP1 | LRR Binding FLII Interacting Protein 1 | Protein Coding | 35 | GC02P237627 | 2.869846344 |
| ZNF23 | Zinc Finger Protein 23 | Protein Coding | 34 | GC16M071463 | 2.869015932 |
| ANKK1 | Ankyrin Repeat And Kinase Domain Containing 1 | Protein Coding | 35 | GC11P113387 | 2.868778229 |
| MAP3K7CL | MAP3K7 C-Terminal Like | Protein Coding | 28 | GC21P029077 | 2.868247032 |
| RAMP2 | Receptor Activity Modifying Protein 2 | Protein Coding | 38 | GC17P042758 | 2.867456913 |
| PIK3C2B | Phosphatidylinositol-4-Phosphate 3-Kinase Catalytic Subunit Type 2 Beta | Protein Coding | 42 | GC01M204422 | 2.866876125 |
| AJUBA | Ajuba LIM Protein | Protein Coding | 34 | GC14M022971 | 2.865885258 |
| PIR | Pirin | Protein Coding | 35 | GC0XM015402 | 2.865488052 |
| TPCN1 | Two Pore Segment Channel 1 | Protein Coding | 33 | GC12P113221 | 2.864422321 |
| GRIK2 | Glutamate Ionotropic Receptor Kainate Type Subunit 2 | Protein Coding | 43 | GC06P100962 | 2.864390373 |
| DCUN1D3 | Defective In Cullin Neddylation 1 Domain Containing 3 | Protein Coding | 30 | GC16M020869 | 2.864202976 |
| ELF1 | E74 Like ETS Transcription Factor 1 | Protein Coding | 36 | GC13M040963 | 2.864175081 |
| WBP2 | WW Domain Binding Protein 2 | Protein Coding | 35 | GC17M075845 | 2.864132404 |
| ACKR2 | Atypical Chemokine Receptor 2 | Protein Coding | 34 | GC03P042804 | 2.86387372 |
| ALG3 | ALG3 Alpha-1,3- Mannosyltransferase | Protein Coding | 36 | GC03M184244 | 2.863664627 |
| FLAD1 | Flavin Adenine Dinucleotide Synthetase 1 | Protein Coding | 36 | GC01P154983 | 2.862603188 |
| EMC1 | ER Membrane Protein Complex Subunit 1 | Protein Coding | 32 | GC01M019215 | 2.862461567 |
| EDA2R | Ectodysplasin A2 Receptor | Protein Coding | 38 | GC0XM066595 | 2.861947775 |
| DDX59 | DEAD-Box Helicase 59 | Protein Coding | 34 | GC01M200594 | 2.861778736 |
| TCAF1 | TRPM8 Channel Associated Factor 1 | Protein Coding | 27 | GC07M143852 | 2.860503435 |
| LILRB1 | Leukocyte Immunoglobulin Like Receptor B1 | Protein Coding | 39 | GC19P064337 | 2.860285282 |
| DPP6 | Dipeptidyl Peptidase Like 6 | Protein Coding | 40 | GC07P153748 | 2.859742165 |
| GRB10 | Growth Factor Receptor Bound Protein 10 | Protein Coding | 37 | GC07M050590 | 2.859461308 |
| CD200 | CD200 Molecule | Protein Coding | 36 | GC03P112332 | 2.858397961 |
| LINC00672 | Long Intergenic Non-Protein Coding RNA 672 | RNA Gene | 16 | GC17P038925 | 2.858323336 |
| C1QB | Complement C1q B Chain | Protein Coding | 40 | GC01P022652 | 2.858196259 |
| OTUD4 | OTU Deubiquitinase 4 | Protein Coding | 33 | GC04M145110 | 2.857480764 |
| CDK13 | Cyclin Dependent Kinase 13 | Protein Coding | 38 | GC07P040475 | 2.85699892 |
| MAPKAPK2 | MAPK Activated Protein Kinase 2 | Protein Coding | 43 | GC01P206684 | 2.856571198 |
| SMARCAD1 | SWI/SNF-Related, Matrix-Associated Actin-Dependent Regulator Of Chromatin, Subfamily A, Containing DEAD/H Box 1 | Protein Coding | 41 | GC04P094207 | 2.85611248 |
| POMGNT1 | Protein O-Linked Mannose N-Acetylglucosaminyltransferase 1 (Beta 1,2-) | Protein Coding | 40 | GC01M046188 | 2.855996609 |
| CTSZ | Cathepsin Z | Protein Coding | 42 | GC20M058995 | 2.85564518 |
| LYAR | Ly1 Antibody Reactive | Protein Coding | 35 | GC04M004274 | 2.855552673 |
| SSH3 | Slingshot Protein Phosphatase 3 | Protein Coding | 34 | GC11P067303 | 2.855260849 |
| ABCB8 | ATP Binding Cassette Subfamily B Member 8 | Protein Coding | 38 | GC07P151028 | 2.85520792 |
| SEMG1 | Semenogelin 1 | Protein Coding | 31 | GC20P045206 | 2.854692459 |
| PNKD | PNKD Metallo-Beta-Lactamase Domain Containing | Protein Coding | 39 | GC02P218270 | 2.854504347 |
| CBR3 | Carbonyl Reductase 3 | Protein Coding | 38 | GC21P036134 | 2.853886604 |
| MROH7 | Maestro Heat Like Repeat Family Member 7 | Protein Coding | 27 | GC01P054643 | 2.853749752 |
| ERLEC1 | Endoplasmic Reticulum Lectin 1 | Protein Coding | 32 | GC02P053786 | 2.853651047 |
| BBIP1 | BBSome Interacting Protein 1 | Protein Coding | 30 | GC10M110898 | 2.853455544 |
| GDF11 | Growth Differentiation Factor 11 | Protein Coding | 38 | GC12P055743 | 2.853286982 |
| MIER1 | MIER1 Transcriptional Regulator | Protein Coding | 30 | GC01P066924 | 2.852941036 |
| LOC113939944 | Sharpr-MPRA Regulatory Region 9539 | Biological Region | 3 | GC15P048520 | 2.852901936 |
| SLC25A6 | Solute Carrier Family 25 Member 6 | Protein Coding | 37 | GC0XM001386 | 2.852746725 |
| ANAPC2 | Anaphase Promoting Complex Subunit 2 | Protein Coding | 35 | GC09M137174 | 2.852230072 |
| KRT78 | Keratin 78 | Protein Coding | 31 | GC12M052837 | 2.850912094 |
| RPS23 | Ribosomal Protein S23 | Protein Coding | 36 | GC05M082273 | 2.850340366 |
| MTMR8 | Myotubularin Related Protein 8 | Protein Coding | 31 | GC0XM064268 | 2.850076437 |
| FSTL5 | Follistatin Like 5 | Protein Coding | 32 | GC04M161383 | 2.849283218 |
| ECHDC1 | Ethylmalonyl-CoA Decarboxylase 1 | Protein Coding | 31 | GC06M127288 | 2.849012136 |
| GOLM1 | Golgi Membrane Protein 1 | Protein Coding | 35 | GC09M086026 | 2.848731518 |
| NAP1L1 | Nucleosome Assembly Protein 1 Like 1 | Protein Coding | 34 | GC12M076036 | 2.848709106 |
| CUX2 | Cut Like Homeobox 2 | Protein Coding | 38 | GC12P111034 | 2.847220421 |
| KCNU1 | Potassium Calcium-Activated Channel Subfamily U Member 1 | Protein Coding | 35 | GC08P036784 | 2.846699953 |
| TTC8 | Tetratricopeptide Repeat Domain 8 | Protein Coding | 36 | GC14P090066 | 2.846544743 |
| NHS | NHS Actin Remodeling Regulator | Protein Coding | 34 | GC0XP017393 | 2.846308708 |
| GARS1 | Glycyl-TRNA Synthetase 1 | Protein Coding | 35 | GC07P030580 | 2.846119881 |
| DCDC2 | Doublecortin Domain Containing 2 | Protein Coding | 35 | GC06M024171 | 2.84458971 |
| LYST | Lysosomal Trafficking Regulator | Protein Coding | 35 | GC01M235661 | 2.844444752 |
| GDF3 | Growth Differentiation Factor 3 | Protein Coding | 38 | GC12M007689 | 2.843809128 |
| GFRAL | GDNF Family Receptor Alpha Like | Protein Coding | 28 | GC06P080747 | 2.843391418 |
| C2CD6 | C2 Calcium Dependent Domain Containing 6 | Protein Coding | 25 | GC02M201488 | 2.842632294 |
| FTHL17 | Ferritin Heavy Chain Like 17 | Protein Coding | 29 | GC0XM030999 | 2.842063904 |
| GUCY1A1 | Guanylate Cyclase 1 Soluble Subunit Alpha 1 | Protein Coding | 35 | GC04P155667 | 2.841629267 |
| NUP62 | Nucleoporin 62 | Protein Coding | 40 | GC19M049906 | 2.840921879 |
| RPL23A | Ribosomal Protein L23a | Protein Coding | 36 | GC17P028719 | 2.839840889 |
| CAV3 | Caveolin 3 | Protein Coding | 38 | GC03P008733 | 2.838946819 |
| COPS6 | COP9 Signalosome Subunit 6 | Protein Coding | 32 | GC07P100088 | 2.838750601 |
| MYO1C | Myosin IC | Protein Coding | 38 | GC17M001464 | 2.838588238 |
| CEL | Carboxyl Ester Lipase | Protein Coding | 43 | GC09P133061 | 2.83698988 |
| PIEZO1 | Piezo Type Mechanosensitive Ion Channel Component 1 | Protein Coding | 35 | GC16M088715 | 2.83641243 |
| PNPLA2 | Patatin Like Phospholipase Domain Containing 2 | Protein Coding | 40 | GC11P001679 | 2.836400032 |
| CCT6A | Chaperonin Containing TCP1 Subunit 6A | Protein Coding | 35 | GC07P056051 | 2.836225033 |
| ZMIZ2 | Zinc Finger MIZ-Type Containing 2 | Protein Coding | 31 | GC07P044756 | 2.836005688 |
| CCL26 | C-C Motif Chemokine Ligand 26 | Protein Coding | 35 | GC07M075769 | 2.83569169 |
| SENP1 | SUMO Specific Peptidase 1 | Protein Coding | 39 | GC12M048042 | 2.835544586 |

| | | | | | |
|---|---|---|---|---|---|
| NDUFA8 | NADH:Ubiquinone Oxidoreductase Subunit A8 | Protein Coding | 39 | GC09M122132 | 2.834970951 |
| VSIR | V-Set Immunoregulatory Receptor | Protein Coding | 32 | GC10M071748 | 2.834057331 |
| CTSG | Cathepsin G | Protein Coding | 41 | GC14M024573 | 2.833837032 |
| MAPKAPK5 | MAPK Activated Protein Kinase 5 | Protein Coding | 39 | GC12P111842 | 2.833636045 |
| DIAPH3 | Diaphanous Related Formin 3 | Protein Coding | 38 | GC13M059665 | 2.833020449 |
| AFF3 | AF4/FMR2 Family Member 3 | Protein Coding | 34 | GC02M099545 | 2.832855701 |
| DGCR8 | DGCR8 Microprocessor Complex Subunit | Protein Coding | 36 | GC22P020080 | 2.831817627 |
| GDF5 | Growth Differentiation Factor 5 | Protein Coding | 42 | GC20M035433 | 2.831810236 |
| BPI | Bactericidal Permeability Increasing Protein | Protein Coding | 38 | GC20P038304 | 2.83070612 |
| TP53INP1 | Tumor Protein P53 Inducible Nuclear Protein 1 | Protein Coding | 32 | GC08M094925 | 2.830514431 |
| INPP5D | Inositol Polyphosphate-5-Phosphatase D | Protein Coding | 40 | GC02P233059 | 2.830173492 |
| MLLT1 | MLLT1 Super Elongation Complex Subunit | Protein Coding | 36 | GC19M006210 | 2.829977036 |
| FIBP | FGF1 Intracellular Binding Protein | Protein Coding | 37 | GC11M087299 | 2.829574585 |
| SF3A1 | Splicing Factor 3a Subunit 1 | Protein Coding | 34 | GC22M030331 | 2.829557896 |
| DHX8 | DEAH-Box Helicase 8 | Protein Coding | 33 | GC17P043483 | 2.829316616 |
| TKTL1 | Transketolase Like 1 | Protein Coding | 36 | GC0XP154295 | 2.828788519 |
| CNGA2 | Cyclic Nucleotide Gated Channel Subunit Alpha 2 | Protein Coding | 36 | GC0XP151734 | 2.828639984 |
| HECTD4 | HECT Domain E3 Ubiquitin Protein Ligase 4 | Protein Coding | 28 | GC12M112160 | 2.828533411 |
| SETD4 | SET Domain Containing 4 | Protein Coding | 30 | GC21M036034 | 2.828467131 |
| ASPN | Asporin | Protein Coding | 38 | GC09M092753 | 2.828446865 |
| HINT1 | Histidine Triad Nucleotide Binding Protein 1 | Protein Coding | 41 | GC05M131159 | 2.828023434 |
| CCDC25 | Coiled-Coil Domain Containing 25 | Protein Coding | 32 | GC08M027733 | 2.827652454 |
| GALE | UDP-Galactose-4-Epimerase | Protein Coding | 38 | GC01M023795 | 2.82757163 |
| LPAR6 | Lysophosphatidic Acid Receptor 6 | Protein Coding | 39 | GC13M048389 | 2.827395201 |
| PSMA4 | Proteasome 20S Subunit Alpha 4 | Protein Coding | 39 | GC15P078540 | 2.826435566 |
| ARL2BP | ADP Ribosylation Factor Like GTPase 2 Binding Protein | Protein Coding | 34 | GC16P057245 | 2.82631731 |
| NDUFV1 | NADH:Ubiquinone Oxidoreductase Core Subunit V1 | Protein Coding | 41 | GC11P069737 | 2.825840473 |
| CHMP2B | Charged Multivesicular Body Protein 2B | Protein Coding | 39 | GC03P087227 | 2.825111866 |
| TMEM8B | Transmembrane Protein 8B | Protein Coding | 31 | GC09P035814 | 2.824474812 |
| GATA3-AS1 | GATA3 Antisense RNA 1 | RNA Gene | 16 | GC10M008016 | 2.824377298 |
| UCP1 | Uncoupling Protein 1 | Protein Coding | 39 | GC04M140559 | 2.824101448 |
| LIMS1 | LIM Zinc Finger Domain Containing 1 | Protein Coding | 36 | GC02P108534 | 2.824022055 |
| NNAT | Neuronatin | Protein Coding | 30 | GC20P037521 | 2.823155642 |
| SUGP2 | SURP And G-Patch Domain Containing 2 | Protein Coding | 31 | GC19M018990 | 2.822224617 |
| FERD3L | Fer3 Like BHLH Transcription Factor | Protein Coding | 31 | GC07M019144 | 2.821681499 |
| SHANK1 | SH3 And Multiple Ankyrin Repeat Domains 1 | Protein Coding | 34 | GC19M050659 | 2.820971966 |
| ALG1 | ALG1 Chitobiosyldiphosphodolichol Beta-Mannosyltransferase | Protein Coding | 38 | GC16P005033 | 2.820762873 |
| LOC106736614 | RET 5' Regulatory Region | Biological Region | 2 | GC10P043072 | 2.820149422 |
| ELMO1 | Engulfment And Cell Motility 1 | Protein Coding | 36 | GC07M036860 | 2.820070982 |
| KCND3 | Potassium Voltage-Gated Channel Subfamily D Member 3 | Protein Coding | 43 | GC01M111770 | 2.819601297 |
| ZNF695 | Zinc Finger Protein 695 | Protein Coding | 31 | GC01M246945 | 2.818984985 |
| PDS5A | PDS5 Cohesin Associated Factor A | Protein Coding | 34 | GC04M039824 | 2.817217827 |
| OSTC | Oligosaccharyltransferase Complex Non-Catalytic Subunit | Protein Coding | 31 | GC04P108650 | 2.816804886 |
| RNASEH2B | Ribonuclease H2 Subunit B | Protein Coding | 32 | GC13P050909 | 2.81677103 |
| PRKAG1 | Protein Kinase AMP-Activated Non-Catalytic Subunit Gamma 1 | Protein Coding | 43 | GC12M049002 | 2.816282511 |
| BICC1 | BicC Family RNA Binding Protein 1 | Protein Coding | 34 | GC10P058513 | 2.815928221 |
| CSTF1 | Cleavage Stimulation Factor Subunit 1 | Protein Coding | 32 | GC20P056392 | 2.814513683 |
| MID1 | Midline 1 | Protein Coding | 39 | GC0XM010445 | 2.813466072 |
| CLSTN2 | Calsyntenin 2 | Protein Coding | 36 | GC03P139935 | 2.811803579 |
| XK | X-Linked Kx Blood Group | Protein Coding | 36 | GC0XP037685 | 2.811684847 |
| TRMT10A | TRNA Methyltransferase 10A | Protein Coding | 32 | GC04M099546 | 2.810747623 |
| AP2M1 | Adaptor Related Protein Complex 2 Subunit Mu 1 | Protein Coding | 39 | GC03P184174 | 2.810632944 |
| LNPEP | Leucyl And Cystinyl Aminopeptidase | Protein Coding | 42 | GC05P096935 | 2.810280323 |
| TSPAN4 | Tetraspanin 4 | Protein Coding | 33 | GC11P001683 | 2.809564114 |
| LRG1 | Leucine Rich Alpha-2-Glycoprotein 1 | Protein Coding | 36 | GC19M005068 | 2.808745861 |
| RPLP2 | Ribosomal Protein Lateral Stalk Subunit P2 | Protein Coding | 36 | GC11P001680 | 2.808270216 |
| GNB1L | G Protein Subunit Beta 1 Like | Protein Coding | 32 | GC22M019783 | 2.806537151 |
| ZFR | Zinc Finger RNA Binding Protein | Protein Coding | 34 | GC05M032390 | 2.806479454 |
| NT5C2 | 5'-Nucleotidase, Cytosolic II | Protein Coding | 42 | GC10M103088 | 2.8062644 |
| UCHL3 | Ubiquitin C-Terminal Hydrolase L3 | Protein Coding | 38 | GC13P075550 | 2.806216955 |
| PPAT | Phosphoribosyl Pyrophosphate Amidotransferase | Protein Coding | 39 | GC04M056393 | 2.805913925 |
| UGT2B28 | UDP Glucuronosyltransferase Family 2 Member B28 | Protein Coding | 35 | GC04P069280 | 2.805199862 |
| VAV2 | Vav Guanine Nucleotide Exchange Factor 2 | Protein Coding | 38 | GC09M133761 | 2.805015564 |
| AZIN1 | Antizyme Inhibitor 1 | Protein Coding | 35 | GC08M102826 | 2.804273129 |
| CCT3 | Chaperonin Containing TCP1 Subunit 3 | Protein Coding | 36 | GC01M156308 | 2.802947044 |
| NMU | Neuromedin U | Protein Coding | 35 | GC04M055595 | 2.802802086 |
| GRM3 | Glutamate Metabotropic Receptor 3 | Protein Coding | 42 | GC07P086643 | 2.802549124 |
| NLE1 | Notchless Homolog 1 | Protein Coding | 30 | GC17M035128 | 2.802445412 |
| FSBP | Fibrinogen Silencer Binding Protein | Protein Coding | 26 | GC08M094378 | 2.802084446 |
| FSCN2 | Fascin Actin-Bundling Protein 2, Retinal | Protein Coding | 36 | GC17P081544 | 2.801875353 |
| RPL9 | Ribosomal Protein L9 | Protein Coding | 36 | GC04M039452 | 2.801808357 |
| CRBN | Cereblon | Protein Coding | 39 | GC03M003144 | 2.801519871 |
| SAP30 | Sin3A Associated Protein 30 | Protein Coding | 35 | GC04P173369 | 2.800531387 |
| GCH1 | GTP Cyclohydrolase 1 | Protein Coding | 43 | GC14M054842 | 2.800441504 |
| PHF20L1 | PHD Finger Protein 20 Like 1 | Protein Coding | 30 | GC08P132775 | 2.800011873 |
| CIRBP | Cold Inducible RNA Binding Protein | Protein Coding | 34 | GC19P001259 | 2.799917459 |
| CLCNKA | Chloride Voltage-Gated Channel Ka | Protein Coding | 38 | GC01P016018 | 2.79903841 |
| CPNE3 | Copine 3 | Protein Coding | 34 | GC08P086514 | 2.797447681 |
| MAGED4B | MAGE Family Member D4B | Protein Coding | 27 | GC0XM052061 | 2.797291517 |
| POLR3B | RNA Polymerase III Subunit B | Protein Coding | 39 | GC12P106357 | 2.796413422 |
| TUT1 | Terminal Uridylyl Transferase 1, U6 SnRNA-Specific | Protein Coding | 32 | GC11M087122 | 2.795682907 |
| WDR48 | WD Repeat Domain 48 | Protein Coding | 35 | GC03P039052 | 2.795386314 |
| KDM2A | Lysine Demethylase 2A | Protein Coding | 37 | GC11P067119 | 2.794898272 |
| APOH | Apolipoprotein H | Protein Coding | 40 | GC17M066212 | 2.794677258 |
| CLIC4 | Chloride Intracellular Channel 4 | Protein Coding | 35 | GC01P024745 | 2.794642448 |
| B3GLCT | Beta 3-Glucosyltransferase | Protein Coding | 33 | GC13P031200 | 2.79413414 |
| LOC110283621 | SPP1 5' Regulatory Region | Biological Region | 2 | GC04P087973 | 2.793792725 |
| SKA3 | Spindle And Kinetochore Associated Complex Subunit 3 | Protein Coding | 30 | GC13M021153 | 2.793747663 |
| GALNT6 | Polypeptide N-Acetylgalactosaminyltransferase 6 | Protein Coding | 35 | GC12M051351 | 2.79283762 |
| ABCB10 | ATP Binding Cassette Subfamily B Member 10 | Protein Coding | 36 | GC01M229516 | 2.792212486 |
| DAPK3 | Death Associated Protein Kinase 3 | Protein Coding | 39 | GC19M003958 | 2.790745258 |
| CPD | Carboxypeptidase D | Protein Coding | 39 | GC17P030378 | 2.790634632 |
| DAZAP1 | DAZ Associated Protein 1 | Protein Coding | 34 | GC19P001407 | 2.790054321 |
| ST13 | ST13 Hsp70 Interacting Protein | Protein Coding | 34 | GC22M056114 | 2.789479971 |
| SUV39H2 | SUV39H2 Histone Lysine Methyltransferase | Protein Coding | 38 | GC10P014878 | 2.789200306 |

| | | | | | |
|---|---|---|---|---|---|
| PSMA3 | Proteasome 20S Subunit Alpha 3 | Protein Coding | 39 | GC14P058244 | 2.788906097 |
| CLDN5 | Claudin 5 | Protein Coding | 36 | GC22M019523 | 2.788903236 |
| SEMA3D | Semaphorin 3D | Protein Coding | 36 | GC07M084995 | 2.786397219 |
| OMA1 | OMA1 Zinc Metallopeptidase | Protein Coding | 32 | GC01M058415 | 2.786268234 |
| TPSAB1 | Tryptase Alpha/Beta 1 | Protein Coding | 40 | GC16P001240 | 2.786144733 |
| RDH12 | Retinol Dehydrogenase 12 | Protein Coding | 39 | GC14P067701 | 2.786027431 |
| SULT2B1 | Sulfotransferase Family 2B Member 1 | Protein Coding | 40 | GC19P048552 | 2.785985947 |
| CACNA2D1 | Calcium Voltage-Gated Channel Auxiliary Subunit Alpha2delta 1 | Protein Coding | 43 | GC07M081946 | 2.785320282 |
| MIR4484 | MicroRNA 4484 | RNA Gene | 15 | GC10P125819 | 2.785036087 |
| RMI2 | RecQ Mediated Genome Instability 2 | Protein Coding | 30 | GC16P011260 | 2.784481049 |
| EIF3F | Eukaryotic Translation Initiation Factor 3 Subunit F | Protein Coding | 37 | GC11P007966 | 2.78279686 |
| AMD1 | Adenosylmethionine Decarboxylase 1 | Protein Coding | 40 | GC06P110814 | 2.782077312 |
| MT1E | Metallothionein 1E | Protein Coding | 34 | GC16P056625 | 2.781559467 |
| ARMCX3 | Armadillo Repeat Containing X-Linked 3 | Protein Coding | 30 | GC0XP101622 | 2.781058788 |
| HTRA2 | HtrA Serine Peptidase 2 | Protein Coding | 40 | GC02P074529 | 2.778922081 |
| NMNAT1 | Nicotinamide Nucleotide Adenylyltransferase 1 | Protein Coding | 42 | GC01P009944 | 2.777966976 |
| XRRA1 | X-Ray Radiation Resistance Associated 1 | Protein Coding | 30 | GC11M074807 | 2.777534008 |
| FARP2 | FERM, ARH/RhoGEF And Pleckstrin Domain Protein 2 | Protein Coding | 35 | GC02P241357 | 2.777170181 |
| PHLDA3 | Pleckstrin Homology Like Domain Family A Member 3 | Protein Coding | 32 | GC01M201467 | 2.776706696 |
| GPN1 | GPN-Loop GTPase 1 | Protein Coding | 34 | GC02P027628 | 2.776314735 |
| PNO1 | Partner Of NOB1 Homolog | Protein Coding | 32 | GC02P068157 | 2.776167154 |
| FILIP1 | Filamin A Interacting Protein 1 | Protein Coding | 31 | GC06M075291 | 2.775398731 |
| TNFRSF14 | TNF Receptor Superfamily Member 14 | Protein Coding | 38 | GC01P002555 | 2.775378227 |
| MPV17 | Mitochondrial Inner Membrane Protein MPV17 | Protein Coding | 38 | GC02M027309 | 2.774421215 |
| BARX2 | BARX Homeobox 2 | Protein Coding | 32 | GC11P129375 | 2.773905754 |
| ARHGEF38 | Rho Guanine Nucleotide Exchange Factor 38 | Protein Coding | 27 | GC04P105552 | 2.773483038 |
| RSL1D1 | Ribosomal L1 Domain Containing 1 | Protein Coding | 34 | GC16M011833 | 2.773421288 |
| MIR433 | MicroRNA 433 | RNA Gene | 21 | GC14P109202 | 2.772954941 |
| LIMD2 | LIM Domain Containing 2 | Protein Coding | 30 | GC17M063695 | 2.771332264 |
| GNAT1 | G Protein Subunit Alpha Transducin 1 | Protein Coding | 41 | GC03P050321 | 2.769747496 |
| FHL3 | Four And A Half LIM Domains 3 | Protein Coding | 37 | GC01M038093 | 2.769612312 |
| TLN1 | Talin 1 | Protein Coding | 36 | GC09M035696 | 2.768932343 |
| RPL17 | Ribosomal Protein L17 | Protein Coding | 33 | GC18M049488 | 2.768094778 |
| EPS15 | Epidermal Growth Factor Receptor Pathway Substrate 15 | Protein Coding | 40 | GC01M051354 | 2.767458677 |
| MIR1246 | MicroRNA 1246 | RNA Gene | 14 | GC02M176600 | 2.767392397 |
| CHSY1 | Chondroitin Sulfate Synthase 1 | Protein Coding | 39 | GC15M101175 | 2.767137051 |
| CDH24 | Cadherin 24 | Protein Coding | 32 | GC14M023047 | 2.767127752 |
| SUPT6H | SPT6 Homolog, Histone Chaperone And Transcription Elongation Factor | Protein Coding | 35 | GC17P028662 | 2.766058922 |
| RAB5C | RAB5C, Member RAS Oncogene Family | Protein Coding | 36 | GC17M042124 | 2.765439034 |
| HNRNPA3 | Heterogeneous Nuclear Ribonucleoprotein A3 | Protein Coding | 35 | GC02P177212 | 2.765320778 |
| C1orf87 | Chromosome 1 Open Reading Frame 87 | Protein Coding | 30 | GC01M059987 | 2.765001297 |
| TSPAN9 | Tetraspanin 9 | Protein Coding | 35 | GC12P003078 | 2.764775515 |
| SCLL | Chromosome 8p11 Myeloproliferative Syndrome | Genetic Locus | 1 | GC08U901516 | 2.764573097 |
| SV2A | Synaptic Vesicle Glycoprotein 2A | Protein Coding | 40 | GC01M149903 | 2.764305353 |
| PSMG1 | Proteasome Assembly Chaperone 1 | Protein Coding | 34 | GC21M039174 | 2.764009953 |
| TOX | Thymocyte Selection Associated High Mobility Group Box | Protein Coding | 35 | GC08M058791 | 2.763744354 |
| CHRND | Cholinergic Receptor Nicotinic Delta Subunit | Protein Coding | 39 | GC02P232525 | 2.763210297 |
| ERVK-6 | Endogenous Retrovirus Group K Member 6, Envelope | Protein Coding | 15 | GC07U903184 | 2.762627125 |
| VAC14 | VAC14 Component Of PIKFYVE Complex | Protein Coding | 39 | GC16M070688 | 2.762260437 |
| UBE3C | Ubiquitin Protein Ligase E3C | Protein Coding | 36 | GC07P157138 | 2.761972189 |
| MIR671 | MicroRNA 671 | RNA Gene | 17 | GC07P151238 | 2.761430502 |
| MIR615 | MicroRNA 615 | RNA Gene | 19 | GC12P054033 | 2.760487556 |
| GRM7 | Glutamate Metabotropic Receptor 7 | Protein Coding | 42 | GC03P006770 | 2.760140181 |
| ANKRD55 | Ankyrin Repeat Domain 55 | Protein Coding | 31 | GC05M056099 | 2.759907722 |
| SPO11 | SPO11 Initiator Of Meiotic Double Stranded Breaks | Protein Coding | 34 | GC20P057329 | 2.759549379 |
| FCHSD2 | FCH And Double SH3 Domains 2 | Protein Coding | 32 | GC11M072836 | 2.75931859 |
| DDX52 | DExD-Box Helicase 52 | Protein Coding | 31 | GC17M037609 | 2.75897646 |
| ARMCX1 | Armadillo Repeat Containing X-Linked 1 | Protein Coding | 28 | GC0XP101550 | 2.758833408 |
| HAPLN1 | Hyaluronan And Proteoglycan Link Protein 1 | Protein Coding | 38 | GC05M083637 | 2.758117437 |
| ARHGEF6 | Rac/Cdc42 Guanine Nucleotide Exchange Factor 6 | Protein Coding | 38 | GC0XM136665 | 2.757780552 |
| STRBP | Spermatid Perinuclear RNA Binding Protein | Protein Coding | 32 | GC09M123640 | 2.757394314 |
| C1QA | Complement C1q A Chain | Protein Coding | 42 | GC01P022636 | 2.75719285 |
| NOL11 | Nucleolar Protein 11 | Protein Coding | 30 | GC17P067717 | 2.756735802 |
| FRG1 | FSHD Region Gene 1 | Protein Coding | 38 | GC04P189940 | 2.755273342 |
| PTP4A2 | Protein Tyrosine Phosphatase 4A2 | Protein Coding | 36 | GC01M031909 | 2.755271912 |
| CBX3 | Chromobox 3 | Protein Coding | 37 | GC07P026201 | 2.754601955 |
| CNOT3 | CCR4-NOT Transcription Complex Subunit 3 | Protein Coding | 38 | GC19P064318 | 2.753796577 |
| CDV3 | CDV3 Homolog | Protein Coding | 28 | GC03P133573 | 2.753612041 |
| SGPL1 | Sphingosine-1-Phosphate Lyase 1 | Protein Coding | 42 | GC10P070815 | 2.753199577 |
| DSTN | Destrin, Actin Depolymerizing Factor | Protein Coding | 34 | GC20P017550 | 2.75269556 |
| INCENP | Inner Centromere Protein | Protein Coding | 36 | GC11P062142 | 2.752147675 |
| TET3 | Tet Methylcytosine Dioxygenase 3 | Protein Coding | 36 | GC02P073986 | 2.752096653 |
| POLA1 | DNA Polymerase Alpha 1, Catalytic Subunit | Protein Coding | 40 | GC0XP024693 | 2.75198245 |
| RRP1 | Ribosomal RNA Processing 1 | Protein Coding | 31 | GC21P043789 | 2.751766205 |
| EFNA2 | Ephrin A2 | Protein Coding | 36 | GC19P002581 | 2.751747847 |
| DBH | Dopamine Beta-Hydroxylase | Protein Coding | 46 | GC09P133636 | 2.7515769 |
| SETD6 | SET Domain Containing 6, Protein Lysine Methyltransferase | Protein Coding | 32 | GC16P058514 | 2.751375437 |
| TRG | T Cell Receptor Gamma Locus | Protein Coding | 12 | GC07M038240 | 2.74990654 |
| MIR492 | MicroRNA 492 | RNA Gene | 18 | GC12P094834 | 2.74922657 |
| CREB3 | CAMP Responsive Element Binding Protein 3 | Protein Coding | 36 | GC09P040164 | 2.749179125 |
| PYCR2 | Pyrroline-5-Carboxylate Reductase 2 | Protein Coding | 40 | GC01M225919 | 2.748980522 |
| MIR3646 | MicroRNA 3646 | RNA Gene | 11 | GC20P044409 | 2.748971224 |
| GCKR | Glucokinase Regulator | Protein Coding | 38 | GC02P027496 | 2.748594761 |
| PI3 | Peptidase Inhibitor 3 | Protein Coding | 35 | GC20P045174 | 2.747868776 |
| CYBA | Cytochrome B-245 Alpha Chain | Protein Coding | 43 | GC16M088643 | 2.74783349 |
| TRIM31 | Tripartite Motif Containing 31 | Protein Coding | 34 | GC06M063508 | 2.747197151 |
| FBXO32 | F-Box Protein 32 | Protein Coding | 35 | GC08M123507 | 2.746527433 |
| ADAM28 | ADAM Metallopeptidase Domain 28 | Protein Coding | 36 | GC08P024294 | 2.746307373 |
| COL11A2 | Collagen Type XI Alpha 2 Chain | Protein Coding | 39 | GC06M033162 | 2.746103525 |
| SPRY2 | Sprouty RTK Signaling Antagonist 2 | Protein Coding | 41 | GC13M080335 | 2.74523592 |
| NFKBIE | NFKB Inhibitor Epsilon | Protein Coding | 35 | GC06M044258 | 2.744978905 |
| HRH1 | Histamine Receptor H1 | Protein Coding | 40 | GC03P012268 | 2.744121552 |
| TIMM17A | Translocase Of Inner Mitochondrial Membrane 17A | Protein Coding | 36 | GC01P201955 | 2.743983984 |
| TUBD1 | Tubulin Delta 1 | Protein Coding | 34 | GC17M059859 | 2.743062019 |
| ST3GAL6 | ST3 Beta-Galactoside Alpha-2,3-Sialyltransferase 6 | Protein Coding | 36 | GC03P098732 | 2.742920876 |

| | | | | | |
|---|---|---|---|---|---|
| RPS25 | Ribosomal Protein S25 | Protein Coding | 33 | GC11M119015 | 2.742609024 |
| CAPZB | Capping Actin Protein Of Muscle Z-Line Subunit Beta | Protein Coding | 36 | GC01M019339 | 2.742387772 |
| PPP1R1A | Protein Phosphatase 1 Regulatory Inhibitor Subunit 1A | Protein Coding | 37 | GC12M054851 | 2.742258072 |
| ANAPC10 | Anaphase Promoting Complex Subunit 10 | Protein Coding | 36 | GC04M144831 | 2.741794586 |
| GRHL3 | Grainyhead Like Transcription Factor 3 | Protein Coding | 35 | GC01P024511 | 2.741232395 |
| UBAP1 | Ubiquitin Associated Protein 1 | Protein Coding | 36 | GC09P034179 | 2.740935564 |
| TRAPPC8 | Trafficking Protein Particle Complex Subunit 8 | Protein Coding | 30 | GC18M031829 | 2.74085474 |
| CD244 | CD244 Molecule | Protein Coding | 39 | GC01M160830 | 2.739411354 |
| SAR1A | Secretion Associated Ras Related GTPase 1A | Protein Coding | 36 | GC10M070147 | 2.739322186 |
| KIDINS220 | Kinase D Interacting Substrate 220 | Protein Coding | 37 | GC02M008724 | 2.737658024 |
| GDAP1 | Ganglioside Induced Differentiation Associated Protein 1 | Protein Coding | 39 | GC08P074315 | 2.736508846 |
| MCF2L | MCF.2 Cell Line Derived Transforming Sequence Like | Protein Coding | 38 | GC13P112894 | 2.735785246 |
| NDUFS2 | NADH:Ubiquinone Oxidoreductase Core Subunit S2 | Protein Coding | 40 | GC01P161197 | 2.735523701 |
| SULT1A3 | Sulfotransferase Family 1A Member 3 | Protein Coding | 34 | GC16P030199 | 2.734817028 |
| EDIL3 | EGF Like Repeats And Discoidin Domains 3 | Protein Coding | 36 | GC05M083940 | 2.734643459 |
| GCN1 | GCN1 Activator Of EIF2AK4 | Protein Coding | 29 | GC12M120128 | 2.732814312 |
| PSMA6 | Proteasome 20S Subunit Alpha 6 | Protein Coding | 40 | GC14P035278 | 2.732334614 |
| IL19 | Interleukin 19 | Protein Coding | 35 | GC01P206770 | 2.731936455 |
| ITPKC | Inositol-Trisphosphate 3-Kinase C | Protein Coding | 38 | GC19P063649 | 2.731747866 |
| MLPH | Melanophilin | Protein Coding | 38 | GC02P237485 | 2.731548786 |
| SSRP1 | Structure Specific Recognition Protein 1 | Protein Coding | 36 | GC11M086954 | 2.730618238 |
| DYRK2 | Dual Specificity Tyrosine Phosphorylation Regulated Kinase 2 | Protein Coding | 40 | GC12P067558 | 2.730023146 |
| EIF2B4 | Eukaryotic Translation Initiation Factor 2B Subunit Delta | Protein Coding | 40 | GC02M027364 | 2.729259491 |
| COX15 | Cytochrome C Oxidase Assembly Homolog COX15 | Protein Coding | 38 | GC10M099696 | 2.729132891 |
| PDLIM4 | PDZ And LIM Domain 4 | Protein Coding | 36 | GC05P132257 | 2.729041576 |
| FEM1C | Fem-1 Homolog C | Protein Coding | 32 | GC05M115520 | 2.727745056 |
| RNMT | RNA Guanine-7 Methyltransferase | Protein Coding | 37 | GC18P017135 | 2.727673292 |
| MCF2L2 | MCF.2 Cell Line Derived Transforming Sequence-Like 2 | Protein Coding | 31 | GC03M183183 | 2.727553368 |
| SLC39A11 | Solute Carrier Family 39 Member 11 | Protein Coding | 32 | GC17M072645 | 2.727436066 |
| HEXIM1 | HEXIM P-TEFb Complex Subunit 1 | Protein Coding | 34 | GC17P053266 | 2.72738266 |
| GAS7 | Growth Arrest Specific 7 | Protein Coding | 35 | GC17M009910 | 2.727304935 |
| LTB4R2 | Leukotriene B4 Receptor 2 | Protein Coding | 38 | GC14P031762 | 2.727250576 |
| PHF1 | PHD Finger Protein 1 | Protein Coding | 36 | GC06P080435 | 2.726927996 |
| AQP8 | Aquaporin 8 | Protein Coding | 35 | GC16P027802 | 2.726499557 |
| GPBAR1 | G Protein-Coupled Bile Acid Receptor 1 | Protein Coding | 38 | GC02P218259 | 2.726309299 |
| PGD | Phosphogluconate Dehydrogenase | Protein Coding | 42 | GC01P010398 | 2.726202726 |
| DNAJC2 | DnaJ Heat Shock Protein Family (Hsp40) Member C2 | Protein Coding | 32 | GC07M103312 | 2.726110458 |
| ZNF532 | Zinc Finger Protein 532 | Protein Coding | 31 | GC18P058862 | 2.725529194 |
| TMEM67 | Transmembrane Protein 67 | Protein Coding | 34 | GC08P093754 | 2.725157499 |
| HSPE1 | Heat Shock Protein Family E (Hsp10) Member 1 | Protein Coding | 37 | GC02P197501 | 2.72495079 |
| PPM1B | Protein Phosphatase, Mg2+/Mn2+ Dependent 1B | Protein Coding | 39 | GC02P044167 | 2.72470355 |
| KLF8 | Kruppel Like Factor 8 | Protein Coding | 34 | GC0XP055909 | 2.723731995 |
| SCN3B | Sodium Voltage-Gated Channel Beta Subunit 3 | Protein Coding | 39 | GC11M123629 | 2.72367239 |
| DSG4 | Desmoglein 4 | Protein Coding | 38 | GC18P031377 | 2.723598003 |
| FBXO22 | F-Box Protein 22 | Protein Coding | 30 | GC15P075903 | 2.72245121 |
| NEUROD2 | Neuronal Differentiation 2 | Protein Coding | 38 | GC17M039603 | 2.722103834 |
| MAGEA9B | MAGE Family Member A9B | Protein Coding | 22 | GC0XM149582 | 2.721705675 |
| USP21 | Ubiquitin Specific Peptidase 21 | Protein Coding | 34 | GC01P161159 | 2.721641541 |
| SETD3 | SET Domain Containing 3, Actin Histidine Methyltransferase | Protein Coding | 32 | GC14M099397 | 2.721250057 |
| KCNAB2 | Potassium Voltage-Gated Channel Subfamily A Regulatory Beta Subunit 2 | Protein Coding | 38 | GC01P006020 | 2.720572472 |
| TM4SF1 | Transmembrane 4 L Six Family Member 1 | Protein Coding | 33 | GC03M149370 | 2.720453262 |
| IL37 | Interleukin 37 | Protein Coding | 33 | GC02P121638 | 2.72021246 |
| CDCA7L | Cell Division Cycle Associated 7 Like | Protein Coding | 37 | GC07M021900 | 2.71995306 |
| CA8 | Carbonic Anhydrase 8 | Protein Coding | 41 | GC08M060187 | 2.719853878 |
| B4GALNT2 | Beta-1,4-N-Acetyl-Galactosaminyltransferase 2 | Protein Coding | 35 | GC17P053507 | 2.719709873 |
| TBL2 | Transducin Beta Like 2 | Protein Coding | 36 | GC07M073568 | 2.719597578 |
| PPIL3 | Peptidylprolyl Isomerase Like 3 | Protein Coding | 35 | GC02M200870 | 2.719594955 |
| DNAJB11 | DnaJ Heat Shock Protein Family (Hsp40) Member B11 | Protein Coding | 36 | GC03P186567 | 2.719304562 |
| ZCCHC14 | Zinc Finger CCHC-Type Containing 14 | Protein Coding | 30 | GC16M087406 | 2.719078541 |
| LEFTY2 | Left-Right Determination Factor 2 | Protein Coding | 39 | GC01M225937 | 2.718133926 |
| PRSS23 | Serine Protease 23 | Protein Coding | 36 | GC11P086791 | 2.717993736 |
| RGL1 | Ral Guanine Nucleotide Dissociation Stimulator Like 1 | Protein Coding | 35 | GC01P183636 | 2.717705011 |
| PCDHB15 | Protocadherin Beta 15 | Protein Coding | 31 | GC05P145821 | 2.717705011 |
| BCAR1P1 | BCAR1 Pseudogene 1 | Pseudogene | 4 | GC15M020072 | 2.71703434 |
| BCAR1P2 | BCAR1 Pseudogene 2 | Pseudogene | 4 | GC15M021080 | 2.71703434 |
| JMJD6 | Jumonji Domain Containing 6, Arginine Demethylase And Lysine Hydroxylase | Protein Coding | 38 | GC17M076718 | 2.716748953 |
| PPP2R5C | Protein Phosphatase 2 Regulatory Subunit B'Gamma | Protein Coding | 39 | GC14P109615 | 2.71582365 |
| KHDRBS3 | KH RNA Binding Domain Containing, Signal Transduction Associated 3 | Protein Coding | 34 | GC08P135457 | 2.715753794 |
| COPB2 | COPI Coat Complex Subunit Beta 2 | Protein Coding | 41 | GC03M139355 | 2.715616703 |
| USP11 | Ubiquitin Specific Peptidase 11 | Protein Coding | 38 | GC0XP047232 | 2.71559 |
| CD2BP2 | CD2 Cytoplasmic Tail Binding Protein 2 | Protein Coding | 33 | GC16M030350 | 2.715163708 |
| NOP56 | NOP56 Ribonucleoprotein | Protein Coding | 39 | GC20P003959 | 2.714375019 |
| SUB1 | SUB1 Regulator Of Transcription | Protein Coding | 34 | GC05P032614 | 2.714336872 |
| PADI4 | Peptidyl Arginine Deiminase 4 | Protein Coding | 39 | GC01P017308 | 2.713922501 |
| B4GALT1 | Beta-1,4-Galactosyltransferase 1 | Protein Coding | 41 | GC09M033100 | 2.713582039 |
| RIN1 | Ras And Rab Interactor 1 | Protein Coding | 33 | GC11M087322 | 2.712018967 |
| MTTP | Microsomal Triglyceride Transfer Protein | Protein Coding | 39 | GC04P099563 | 2.711951971 |
| STAU1 | Staufen Double-Stranded RNA Binding Protein 1 | Protein Coding | 34 | GC20M049113 | 2.711939096 |
| CLDN16 | Claudin 16 | Protein Coding | 40 | GC03P190290 | 2.711872339 |
| NFYA | Nuclear Transcription Factor Y Subunit Alpha | Protein Coding | 34 | GC06P080568 | 2.711410761 |
| MANF | Mesencephalic Astrocyte Derived Neurotrophic Factor | Protein Coding | 34 | GC03P051385 | 2.709932327 |
| MIR513A1 | MicroRNA 513a-1 | RNA Gene | 15 | GC0XM147213 | 2.709716797 |
| NCF4 | Neutrophil Cytosolic Factor 4 | Protein Coding | 43 | GC22P036860 | 2.709471703 |
| COMMD1 | Copper Metabolism Domain Containing 1 | Protein Coding | 38 | GC02P061888 | 2.709404469 |
| RAB35 | RAB35, Member RAS Oncogene Family | Protein Coding | 36 | GC12M120096 | 2.709301949 |
| PIGS | Phosphatidylinositol Glycan Anchor Biosynthesis Class S | Protein Coding | 35 | GC17M028553 | 2.708852291 |
| DLG3 | Discs Large MAGUK Scaffold Protein 3 | Protein Coding | 38 | GC0XP070444 | 2.70871973 |
| PRPH | Peripherin | Protein Coding | 39 | GC12P049293 | 2.708542824 |
| DAAM1 | Dishevelled Associated Activator Of Morphogenesis 1 | Protein Coding | 37 | GC14P059188 | 2.708199024 |
| RFT1 | RFT1 Homolog | Protein Coding | 35 | GC03M053071 | 2.708072186 |
| VPS52 | VPS52 Subunit Of GARP Complex | Protein Coding | 31 | GC06M063702 | 2.707244873 |
| MAFF | MAF BZIP Transcription Factor F | Protein Coding | 36 | GC22P038200 | 2.707139492 |
| FGG | Fibrinogen Gamma Chain | Protein Coding | 44 | GC04M154604 | 2.707052708 |
| ZNF25 | Zinc Finger Protein 25 | Protein Coding | 32 | GC10M037982 | 2.706308842 |
| MFSD9 | Major Facilitator Superfamily Domain Containing 9 | Protein Coding | 30 | GC02M102700 | 2.706308842 |

| | | | | | |
|---|---|---|---|---|---|
| EXOC3L1 | Exocyst Complex Component 3 Like 1 | Protein Coding | 29 | GC16M067184 | 2.706308842 |
| ZNF432 | Zinc Finger Protein 432 | Protein Coding | 29 | GC19M052031 | 2.706308842 |
| SNRPE | Small Nuclear Ribonucleoprotein Polypeptide E | Protein Coding | 36 | GC01P203908 | 2.705467224 |
| CTDSP1 | CTD Small Phosphatase 1 | Protein Coding | 35 | GC02P218398 | 2.705276251 |
| ZNF57 | Zinc Finger Protein 57 | Protein Coding | 28 | GC19P002900 | 2.705030918 |
| MYOC | Myocilin | Protein Coding | 38 | GC01M171604 | 2.704907894 |
| FOXK1 | Forkhead Box K1 | Protein Coding | 30 | GC07P004682 | 2.704899073 |
| MIR450B | MicroRNA 450b | RNA Gene | 16 | GC0XM134540 | 2.704763651 |
| SEL1L | SEL1L Adaptor Subunit Of ERAD E3 Ubiquitin Ligase | Protein Coding | 39 | GC14M081471 | 2.704262257 |
| CSF2RA | Colony Stimulating Factor 2 Receptor Subunit Alpha | Protein Coding | 40 | GC0XP001614 | 2.704235792 |
| M6PR | Mannose-6-Phosphate Receptor, Cation Dependent | Protein Coding | 39 | GC12M009012 | 2.703985929 |
| SMPD4 | Sphingomyelin Phosphodiesterase 4 | Protein Coding | 35 | GC02M130151 | 2.703635693 |
| COL12A1 | Collagen Type XII Alpha 1 Chain | Protein Coding | 38 | GC06M075084 | 2.703591585 |
| ESS2 | Ess-2 Splicing Factor Homolog | Protein Coding | 28 | GC22M019130 | 2.702548265 |
| AMOTL1 | Angiomotin Like 1 | Protein Coding | 34 | GC11P094686 | 2.701879025 |
| KIF23 | Kinesin Family Member 23 | Protein Coding | 40 | GC15P069414 | 2.701803207 |
| LY6D | Lymphocyte Antigen 6 Family Member D | Protein Coding | 32 | GC08M142784 | 2.701796532 |
| SKA2 | Spindle And Kinetochore Associated Complex Subunit 2 | Protein Coding | 32 | GC17M059109 | 2.700286865 |
| ADCY3 | Adenylate Cyclase 3 | Protein Coding | 42 | GC02M024819 | 2.699868679 |
| DHODH | Dihydroorotate Dehydrogenase (Quinone) | Protein Coding | 42 | GC16P072008 | 2.699785709 |
| PSMC6 | Proteasome 26S Subunit, ATPase 6 | Protein Coding | 35 | GC14P052707 | 2.698986053 |
| THY1-AS1 | THY1 Antisense RNA 1 | RNA Gene | 9 | GC11P120140 | 2.698937893 |
| ZNF436 | Zinc Finger Protein 436 | Protein Coding | 34 | GC01M023359 | 2.698479176 |
| HNRNPH2 | Heterogeneous Nuclear Ribonucleoprotein H2 | Protein Coding | 34 | GC0XP101408 | 2.698036671 |
| USP48 | Ubiquitin Specific Peptidase 48 | Protein Coding | 35 | GC01M021678 | 2.697971344 |
| MIR501 | MicroRNA 501 | RNA Gene | 16 | GC0XP050566 | 2.697772503 |
| MIR493 | MicroRNA 493 | RNA Gene | 18 | GC14P109540 | 2.697614193 |
| SLC38A2 | Solute Carrier Family 38 Member 2 | Protein Coding | 36 | GC12M046358 | 2.697418928 |
| CYP2J2 | Cytochrome P450 Family 2 Subfamily J Member 2 | Protein Coding | 40 | GC01M059893 | 2.696718216 |
| CLDN15 | Claudin 15 | Protein Coding | 34 | GC07M101232 | 2.696525097 |
| CLCA4 | Chloride Channel Accessory 4 | Protein Coding | 34 | GC01P086547 | 2.696518898 |
| ABHD12B | Abhydrolase Domain Containing 12B | Protein Coding | 31 | GC14P050872 | 2.696370125 |
| GPT2 | Glutamic--Pyruvic Transaminase 2 | Protein Coding | 40 | GC16P046885 | 2.696264744 |
| RBMS3 | RNA Binding Motif Single Stranded Interacting Protein 3 | Protein Coding | 32 | GC03P028575 | 2.696161747 |
| FAM91A1 | Family With Sequence Similarity 91 Member A1 | Protein Coding | 30 | GC08P123768 | 2.696012259 |
| MPHOSPH8 | M-Phase Phosphoprotein 8 | Protein Coding | 33 | GC13P019633 | 2.695094109 |
| ABCE1 | ATP Binding Cassette Subfamily E Member 1 | Protein Coding | 35 | GC04P145097 | 2.694701195 |
| NBEAL2 | Neurobeachin Like 2 | Protein Coding | 38 | GC03P046979 | 2.694152832 |
| ITPR2 | Inositol 1,4,5-Trisphosphate Receptor Type 2 | Protein Coding | 42 | GC12M026336 | 2.693264008 |
| GPAT3 | Glycerol-3-Phosphate Acyltransferase 3 | Protein Coding | 32 | GC04P083536 | 2.692986488 |
| MYL6 | Myosin Light Chain 6 | Protein Coding | 36 | GC12P056158 | 2.692854643 |
| CAMK2D | Calcium/Calmodulin Dependent Protein Kinase II Delta | Protein Coding | 44 | GC04M113418 | 2.692809105 |
| RBM8A | RNA Binding Motif Protein 8A | Protein Coding | 36 | GC01M145921 | 2.692298174 |
| EIF3L | Eukaryotic Translation Initiation Factor 3 Subunit L | Protein Coding | 35 | GC22P037848 | 2.691464424 |
| TUBB2B | Tubulin Beta 2B Class IIb | Protein Coding | 39 | GC06M003224 | 2.691334248 |
| ORC3 | Origin Recognition Complex Subunit 3 | Protein Coding | 32 | GC06P087590 | 2.689496994 |
| SPTA1 | Spectrin Alpha, Erythrocytic 1 | Protein Coding | 41 | GC01M158610 | 2.688894033 |
| POLR2B | RNA Polymerase II Subunit B | Protein Coding | 38 | GC04P056977 | 2.688786983 |
| SLITRK2 | SLIT And NTRK Like Family Member 2 | Protein Coding | 33 | GC0XP145817 | 2.687880039 |
| LEMD3 | LEM Domain Containing 3 | Protein Coding | 38 | GC12P065169 | 2.687650681 |
| ALDH3A2 | Aldehyde Dehydrogenase 3 Family Member A2 | Protein Coding | 40 | GC17P019648 | 2.687257767 |
| AHSA2P | Activator Of HSP90 ATPase Homolog 2, Pseudogene | Pseudogene | 23 | GC02P061179 | 2.687105179 |
| SHOX2 | Short Stature Homeobox 2 | Protein Coding | 36 | GC03M158095 | 2.686591148 |
| SNRPB2 | Small Nuclear Ribonucleoprotein Polypeptide B2 | Protein Coding | 32 | GC20P016730 | 2.686229229 |
| SELENOF | Selenoprotein F | Protein Coding | 26 | GC01M086863 | 2.686225176 |
| WDR37 | WD Repeat Domain 37 | Protein Coding | 34 | GC10P001164 | 2.685313702 |
| ERO1A | Endoplasmic Reticulum Oxidoreductase 1 Alpha | Protein Coding | 34 | GC14M052640 | 2.684505939 |
| AFAP1L2 | Actin Filament Associated Protein 1 Like 2 | Protein Coding | 34 | GC10M114281 | 2.684323788 |
| KCNT2 | Potassium Sodium-Activated Channel Subfamily T Member 2 | Protein Coding | 36 | GC01M196225 | 2.684222221 |
| NEFH | Neurofilament Heavy Chain | Protein Coding | 40 | GC22P029480 | 2.683733225 |
| ZNF644 | Zinc Finger Protein 644 | Protein Coding | 35 | GC01M090915 | 2.682962894 |
| SOAT2 | Sterol O-Acyltransferase 2 | Protein Coding | 39 | GC12P053103 | 2.682199717 |
| PSMB6 | Proteasome 20S Subunit Beta 6 | Protein Coding | 37 | GC17P004796 | 2.681576729 |
| OAT | Ornithine Aminotransferase | Protein Coding | 43 | GC10M124397 | 2.681540012 |
| TBC1D24 | TBC1 Domain Family Member 24 | Protein Coding | 34 | GC16P002475 | 2.681381702 |
| FABP6 | Fatty Acid Binding Protein 6 | Protein Coding | 38 | GC05P160187 | 2.68134141 |
| ACAD9 | Acyl-CoA Dehydrogenase Family Member 9 | Protein Coding | 40 | GC03P133827 | 2.681156397 |
| GDI2 | GDP Dissociation Inhibitor 2 | Protein Coding | 35 | GC10M005765 | 2.680768967 |
| ANKRD29 | Ankyrin Repeat Domain 29 | Protein Coding | 28 | GC18M023598 | 2.680460215 |
| AARS1 | Alanyl-TRNA Synthetase 1 | Protein Coding | 35 | GC16M070638 | 2.680309534 |
| DFFA | DNA Fragmentation Factor Subunit Alpha | Protein Coding | 39 | GC01M010456 | 2.680093765 |
| PSMD9 | Proteasome 26S Subunit, Non-ATPase 9 | Protein Coding | 38 | GC12P125878 | 2.679803371 |
| NPR1 | Natriuretic Peptide Receptor 1 | Protein Coding | 43 | GC01P153858 | 2.679171085 |
| TDO2 | Tryptophan 2,3-Dioxygenase | Protein Coding | 40 | GC04P155854 | 2.679113388 |
| UTP4 | UTP4 Small Subunit Processome Component | Protein Coding | 31 | GC16P069132 | 2.678785801 |
| DAAM2 | Dishevelled Associated Activator Of Morphogenesis 2 | Protein Coding | 35 | GC06P039792 | 2.678766966 |
| ADAT3 | Adenosine Deaminase TRNA Specific 3 | Protein Coding | 31 | GC19P002640 | 2.678614616 |
| KCNJ1 | Potassium Inwardly Rectifying Channel Subfamily J Member 1 | Protein Coding | 44 | GC11M128741 | 2.678152323 |
| DCTN2 | Dynactin Subunit 2 | Protein Coding | 36 | GC12M057687 | 2.677975178 |
| MIR4712 | MicroRNA 4712 | RNA Gene | 10 | GC15P050360 | 2.67730546 |
| HSCB | HscB Mitochondrial Iron-Sulfur Cluster Cochaperone | Protein Coding | 33 | GC22P034895 | 2.676925659 |
| CTNNBL1 | Catenin Beta Like 1 | Protein Coding | 35 | GC20P037693 | 2.676792145 |
| POLDIP3 | DNA Polymerase Delta Interacting Protein 3 | Protein Coding | 34 | GC22M042583 | 2.676764011 |
| ING5 | Inhibitor Of Growth Family Member 5 | Protein Coding | 34 | GC02P241702 | 2.676674604 |
| LRP8 | LDL Receptor Related Protein 8 | Protein Coding | 38 | GC01M053243 | 2.675998688 |
| MFF | Mitochondrial Fission Factor | Protein Coding | 34 | GC02P227325 | 2.675693274 |
| SEC61G | SEC61 Translocon Subunit Gamma | Protein Coding | 31 | GC07M054752 | 2.675190449 |
| BCS1L | BCS1 Homolog, Ubiquinol-Cytochrome C Reductase Complex Chaperone | Protein Coding | 40 | GC02P218658 | 2.674691677 |
| FOXL1 | Forkhead Box L1 | Protein Coding | 33 | GC16P086576 | 2.673410654 |
| MIR582 | MicroRNA 582 | RNA Gene | 19 | GC05M059703 | 2.673281193 |
| PPP4R3B | Protein Phosphatase 4 Regulatory Subunit 3B | Protein Coding | 27 | GC02M055547 | 2.673016548 |
| TDGF1P3 | Teratocarcinoma-Derived Growth Factor 1 Pseudogene 3 | Protein Coding | 18 | GC0XP110520 | 2.672880888 |
| CNGB1 | Cyclic Nucleotide Gated Channel Subunit Beta 1 | Protein Coding | 39 | GC16M057884 | 2.67275548 |
| TFAP2D | Transcription Factor AP-2 Delta | Protein Coding | 32 | GC06P050713 | 2.672154427 |
| MYRF | Myelin Regulatory Factor | Protein Coding | 32 | GC11P061753 | 2.671792269 |

| | | | | | |
|---|---|---|---|---|---|
| CKAP2 | Cytoskeleton Associated Protein 2 | Protein Coding | 32 | GC13P052455 | 2.671521902 |
| ADAMTS19 | ADAM Metallopeptidase With Thrombospondin Type 1 Motif 19 | Protein Coding | 34 | GC05P129460 | 2.671317339 |
| SNRPB | Small Nuclear Ribonucleoprotein Polypeptides B And B1 | Protein Coding | 39 | GC20M002461 | 2.67125845 |
| POLR1B | RNA Polymerase I Subunit B | Protein Coding | 37 | GC02P121630 | 2.669961691 |
| SYT3 | Synaptotagmin 3 | Protein Coding | 33 | GC19M050621 | 2.669204712 |
| KIFC1 | Kinesin Family Member C1 | Protein Coding | 36 | GC06P033391 | 2.668997765 |
| EDN2 | Endothelin 2 | Protein Coding | 38 | GC01M041478 | 2.66850996 |
| FGF12 | Fibroblast Growth Factor 12 | Protein Coding | 38 | GC03M192139 | 2.666802406 |
| H3C6 | H3 Clustered Histone 6 | Protein Coding | 26 | GC06P081043 | 2.666757345 |
| VWDE | Von Willebrand Factor D And EGF Domains | Protein Coding | 28 | GC07M012338 | 2.666298866 |
| LRP12 | LDL Receptor Related Protein 12 | Protein Coding | 36 | GC08M104489 | 2.666293383 |
| RPL39 | Ribosomal Protein L39 | Protein Coding | 31 | GC0XM119786 | 2.66467762 |
| NELFCD | Negative Elongation Factor Complex Member C/D | Protein Coding | 31 | GC20P058981 | 2.664560795 |
| SCN8A | Sodium Voltage-Gated Channel Alpha Subunit 8 | Protein Coding | 44 | GC12P051590 | 2.664374352 |
| PYM1 | PYM Homolog 1, Exon Junction Complex Associated Factor | Protein Coding | 27 | GC12M055902 | 2.662875175 |
| ATXN1 | Ataxin 1 | Protein Coding | 39 | GC06M016299 | 2.662585258 |
| MBS1 | Moebius Syndrome 1 | Genetic Locus | 3 | GC13U990023 | 2.662568092 |
| IPO7 | Importin 7 | Protein Coding | 35 | GC11P009384 | 2.662302017 |
| CUTC | CutC Copper Transporter | Protein Coding | 32 | GC10P099702 | 2.661350727 |
| RPLP1 | Ribosomal Protein Lateral Stalk Subunit P1 | Protein Coding | 35 | GC15P114666 | 2.661254644 |
| CD177 | CD177 Molecule | Protein Coding | 35 | GC19P043353 | 2.661178589 |
| CLCN7 | Chloride Voltage-Gated Channel 7 | Protein Coding | 41 | GC16M001444 | 2.660632372 |
| BAIAP2 | BAR/IMD Domain Containing Adaptor Protein 2 | Protein Coding | 37 | GC17P081035 | 2.660357475 |
| GATAD1 | GATA Zinc Finger Domain Containing 1 | Protein Coding | 34 | GC07P092447 | 2.659811974 |
| ZNF367 | Zinc Finger Protein 367 | Protein Coding | 28 | GC09M096385 | 2.65934515 |
| PIGL | Phosphatidylinositol Glycan Anchor Biosynthesis Class L | Protein Coding | 35 | GC17P016217 | 2.659289598 |
| ITGA7 | Integrin Subunit Alpha 7 | Protein Coding | 43 | GC12M055684 | 2.65857172 |
| FAM83A-AS1 | FAM83A Antisense RNA 1 | RNA Gene | 13 | GC08M123201 | 2.65806818 |
| MFNG | MFNG O-Fucosylpeptide 3-Beta-N-Acetylglucosaminyltransferase | Protein Coding | 38 | GC22M037469 | 2.658041716 |
| RUSF1 | RUS Family Member 1 | Protein Coding | 25 | GC16M036597 | 2.656560421 |
| H1-2 | H1.2 Linker Histone, Cluster Member | Protein Coding | 32 | GC06M026056 | 2.656410933 |
| NASP | Nuclear Autoantigenic Sperm Protein | Protein Coding | 33 | GC01P045583 | 2.656005144 |
| CDC42BPB | CDC42 Binding Protein Kinase Beta | Protein Coding | 38 | GC14M102932 | 2.655508995 |
| LIMK2 | LIM Domain Kinase 2 | Protein Coding | 42 | GC22P031212 | 2.655043125 |
| PADI3 | Peptidyl Arginine Deiminase 3 | Protein Coding | 37 | GC01P017249 | 2.654514313 |
| PLOD2 | Procollagen-Lysine,2-Oxoglutarate 5-Dioxygenase 2 | Protein Coding | 41 | GC03M146069 | 2.653082609 |
| ERCC6L | ERCC Excision Repair 6 Like, Spindle Assembly Checkpoint Helicase | Protein Coding | 32 | GC0XM072204 | 2.652594566 |
| CACUL1 | CDK2 Associated Cullin Domain 1 | Protein Coding | 28 | GC10M118674 | 2.651970387 |
| DHX15 | DEAH-Box Helicase 15 | Protein Coding | 33 | GC04M024519 | 2.651786089 |
| NPBWR1 | Neuropeptides B And W Receptor 1 | Protein Coding | 32 | GC08P052938 | 2.65178442 |
| LINC01139 | Long Intergenic Non-Protein Coding RNA 1139 | RNA Gene | 14 | GC01M238480 | 2.651663542 |
| RAB38 | RAB38, Member RAS Oncogene Family | Protein Coding | 35 | GC11M087809 | 2.651175499 |
| TENT5A | Terminal Nucleotidyltransferase 5A | Protein Coding | 30 | GC06M081495 | 2.65100193 |
| ATP11C | ATPase Phospholipid Transporting 11C | Protein Coding | 34 | GC0XM139726 | 2.650647163 |
| WRNIP1 | WRN Helicase Interacting Protein 1 | Protein Coding | 33 | GC06P002766 | 2.650387049 |
| USHBP1 | USH1 Protein Network Component Harmonin Binding Protein 1 | Protein Coding | 30 | GC19M017249 | 2.649822712 |
| RBM14 | RNA Binding Motif Protein 14 | Protein Coding | 33 | GC11P069659 | 2.649534702 |
| ATG12 | Autophagy Related 12 | Protein Coding | 36 | GC05M115828 | 2.649497032 |
| LOXL1 | Lysyl Oxidase Like 1 | Protein Coding | 39 | GC15P073925 | 2.649378777 |
| PPP1R9A | Protein Phosphatase 1 Regulatory Subunit 9A | Protein Coding | 35 | GC07P094907 | 2.648751736 |
| PALM2AKAP2 | PALM2 And AKAP2 Fusion | Protein Coding | 19 | GC09P109499 | 2.648675919 |
| TRAT1 | T Cell Receptor Associated Transmembrane Adaptor 1 | Protein Coding | 32 | GC03P108823 | 2.648248672 |
| PRMT6 | Protein Arginine Methyltransferase 6 | Protein Coding | 35 | GC01P107056 | 2.647713184 |
| NUSAP1 | Nucleolar And Spindle Associated Protein 1 | Protein Coding | 30 | GC15P041783 | 2.647515297 |
| ATP2C2 | ATPase Secretory Pathway Ca2+ Transporting 2 | Protein Coding | 37 | GC16P084368 | 2.647116661 |
| CCL25 | C-C Motif Chemokine Ligand 25 | Protein Coding | 35 | GC19P008052 | 2.646729231 |
| PRMT3 | Protein Arginine Methyltransferase 3 | Protein Coding | 37 | GC11P020409 | 2.646623135 |
| ALPI | Alkaline Phosphatase, Intestinal | Protein Coding | 41 | GC02P232456 | 2.646432877 |
| SHPRH | SNF2 Histone Linker PHD RING Helicase | Protein Coding | 35 | GC06M145863 | 2.646267891 |
| DDX11-AS1 | DDX11 Antisense RNA 1 | RNA Gene | 14 | GC12M031020 | 2.645091295 |
| DDX39A | DExD-Box Helicase 39A | Protein Coding | 33 | GC19M014408 | 2.644880295 |
| DAP | Death Associated Protein | Protein Coding | 35 | GC05M010679 | 2.644687176 |
| STX1A | Syntaxin 1A | Protein Coding | 42 | GC07M073700 | 2.644637823 |
| CNTN4 | Contactin 4 | Protein Coding | 36 | GC03P002117 | 2.643498421 |
| RDH5 | Retinol Dehydrogenase 5 | Protein Coding | 42 | GC12P055720 | 2.642442703 |
| MYH14 | Myosin Heavy Chain 14 | Protein Coding | 42 | GC19P064121 | 2.641949654 |
| ABCA12 | ATP Binding Cassette Subfamily A Member 12 | Protein Coding | 39 | GC02M214931 | 2.641788721 |
| CWC22 | CWC22 Spliceosome Associated Protein Homolog | Protein Coding | 30 | GC02M179944 | 2.641770124 |
| CHRNA1 | Cholinergic Receptor Nicotinic Alpha 1 Subunit | Protein Coding | 42 | GC02M174747 | 2.641451836 |
| YEATS4 | YEATS Domain Containing 4 | Protein Coding | 33 | GC12P069359 | 2.641158581 |
| DRG1 | Developmentally Regulated GTP Binding Protein 1 | Protein Coding | 38 | GC22P031399 | 2.640887499 |
| PEX1 | Peroxisomal Biogenesis Factor 1 | Protein Coding | 41 | GC07M092487 | 2.640425205 |
| CKAP5 | Cytoskeleton Associated Protein 5 | Protein Coding | 35 | GC11M086868 | 2.639673233 |
| H3C3 | H3 Clustered Histone 3 | Protein Coding | 28 | GC06P079876 | 2.639203072 |
| MNAT1 | MNAT1 Component Of CDK Activating Kinase | Protein Coding | 38 | GC14P060734 | 2.638779402 |
| SH3BP4 | SH3 Domain Binding Protein 4 | Protein Coding | 37 | GC02P234951 | 2.638644934 |
| KLF14 | Kruppel Like Factor 14 | Protein Coding | 32 | GC07M130731 | 2.638524532 |
| NOP58 | NOP58 Ribonucleoprotein | Protein Coding | 34 | GC02P202265 | 2.638111591 |
| BUD23 | BUD23 RRNA Methyltransferase And Ribosome Maturation Factor | Protein Coding | 31 | GC07P074789 | 2.637642384 |
| ATP6V1A | ATPase H+ Transporting V1 Subunit A | Protein Coding | 40 | GC03P113747 | 2.636511803 |
| SCYL1 | SCY1 Like Pseudokinase 1 | Protein Coding | 38 | GC11P065525 | 2.636104107 |
| CBX7 | Chromobox 7 | Protein Coding | 34 | GC22M056105 | 2.635710716 |
| HSD3BP4 | Hydroxy-Delta-5-Steroid Dehydrogenase, 3 Beta, Pseudogene 4 | Pseudogene | 11 | GC01P119563 | 2.635634661 |
| ST8SIA4 | ST8 Alpha-N-Acetyl-Neuraminide Alpha-2,8-Sialyltransferase 4 | Protein Coding | 36 | GC05M100806 | 2.634547949 |
| KIAA0586 | KIAA0586 | Protein Coding | 32 | GC14P058427 | 2.633767605 |
| STK3 | Serine/Threonine Kinase 3 | Protein Coding | 40 | GC08M098372 | 2.633695602 |
| TAF12 | TATA-Box Binding Protein Associated Factor 12 | Protein Coding | 33 | GC01M028816 | 2.633656263 |
| MPRIP | Myosin Phosphatase Rho Interacting Protein | Protein Coding | 34 | GC17P017042 | 2.633270741 |
| COLQ | Collagen Like Tail Subunit Of Asymmetric Acetylcholinesterase | Protein Coding | 36 | GC03M020243 | 2.632515907 |
| SLC7A6 | Solute Carrier Family 7 Member 6 | Protein Coding | 39 | GC16P068263 | 2.632370949 |
| GRK3 | G Protein-Coupled Receptor Kinase 3 | Protein Coding | 38 | GC22P034811 | 2.632229805 |
| ANO5 | Anoctamin 5 | Protein Coding | 35 | GC11P021799 | 2.631289244 |
| MYL9 | Myosin Light Chain 9 | Protein Coding | 39 | GC20P036541 | 2.630989552 |
| TUBG2 | Tubulin Gamma 2 | Protein Coding | 32 | GC17P042659 | 2.630884171 |
| MAL2 | Mal, T Cell Differentiation Protein 2 | Protein Coding | 31 | GC08P119165 | 2.630327702 |

| | | | | | |
|---|---|---|---|---|---|
| DDC | Dopa Decarboxylase | Protein Coding | 46 | GC07M050458 | 2.630173445 |
| LONRF2 | LON Peptidase N-Terminal Domain And Ring Finger 2 | Protein Coding | 31 | GC02M100274 | 2.629994392 |
| HSPA2 | Heat Shock Protein Family A (Hsp70) Member 2 | Protein Coding | 40 | GC14P064535 | 2.628348827 |
| PKLR | Pyruvate Kinase L/R | Protein Coding | 42 | GC01M155289 | 2.628235579 |
| GATD3 | Glutamine Amidotransferase Class 1 Domain Containing 3 | Protein Coding | 26 | GC21P044135 | 2.627562523 |
| CAMSAP2 | Calmodulin Regulated Spectrin Associated Protein Family Member 2 | Protein Coding | 29 | GC01P200739 | 2.627324104 |
| MKKS | MKKS Centrosomal Shuttling Protein | Protein Coding | 35 | GC20M010424 | 2.627138615 |
| DDX27 | DEAD-Box Helicase 27 | Protein Coding | 32 | GC20P049219 | 2.626868486 |
| CFHR3 | Complement Factor H Related 3 | Protein Coding | 37 | GC01P196774 | 2.626465797 |
| USP16 | Ubiquitin Specific Peptidase 16 | Protein Coding | 36 | GC21P029024 | 2.626446247 |
| HGFAC | HGF Activator | Protein Coding | 38 | GC04P003443 | 2.626204252 |
| AURKAIP1 | Aurora Kinase A Interacting Protein 1 | Protein Coding | 32 | GC01M001373 | 2.626085281 |
| CHD9 | Chromodomain Helicase DNA Binding Protein 9 | Protein Coding | 34 | GC16P053041 | 2.625555992 |
| GTPBP4 | GTP Binding Protein 4 | Protein Coding | 34 | GC10P000988 | 2.624523401 |
| GABRA3 | Gamma-Aminobutyric Acid Type A Receptor Subunit Alpha3 | Protein Coding | 41 | GC0XM152166 | 2.624408484 |
| APEH | Acylaminoacyl-Peptide Hydrolase | Protein Coding | 37 | GC03P049673 | 2.623438358 |
| IDH3A | Isocitrate Dehydrogenase (NAD(+)) 3 Catalytic Subunit Alpha | Protein Coding | 41 | GC15P078131 | 2.622554302 |
| AIMP1 | Aminoacyl TRNA Synthetase Complex Interacting Multifunctional Protein 1 | Protein Coding | 39 | GC04P106315 | 2.622476101 |
| SLC5A2 | Solute Carrier Family 5 Member 2 | Protein Coding | 42 | GC16P040324 | 2.621765137 |
| ZNF577 | Zinc Finger Protein 577 | Protein Coding | 29 | GC19M051804 | 2.621471643 |
| MMP21 | Matrix Metallopeptidase 21 | Protein Coding | 36 | GC10M125756 | 2.619926453 |
| FAAP24 | FA Core Complex Associated Protein 24 | Protein Coding | 28 | GC19P063380 | 2.619452953 |
| PDZD4 | PDZ Domain Containing 4 | Protein Coding | 30 | GC0XM153802 | 2.6185956 |
| SLC10A1 | Solute Carrier Family 10 Member 1 | Protein Coding | 40 | GC14M069775 | 2.618112326 |
| NEK7 | NIMA Related Kinase 7 | Protein Coding | 36 | GC01P198156 | 2.617483139 |
| FSIP1 | Fibrous Sheath Interacting Protein 1 | Protein Coding | 31 | GC15M039594 | 2.617475033 |
| DNAJC24 | DnaJ Heat Shock Protein Family (Hsp40) Member C24 | Protein Coding | 31 | GC11P031369 | 2.617177486 |
| FRMD3 | FERM Domain Containing 3 | Protein Coding | 30 | GC09M083242 | 2.617063999 |
| EEF1D | Eukaryotic Translation Elongation Factor 1 Delta | Protein Coding | 37 | GC08M143579 | 2.616484642 |
| SNRPD3 | Small Nuclear Ribonucleoprotein D3 Polypeptide | Protein Coding | 31 | GC22P024555 | 2.616167545 |
| MEOX1 | Mesenchyme Homeobox 1 | Protein Coding | 38 | GC17M043640 | 2.615426064 |
| TMOD3 | Tropomodulin 3 | Protein Coding | 35 | GC15P051829 | 2.615288973 |
| WBP4 | WW Domain Binding Protein 4 | Protein Coding | 31 | GC13P041061 | 2.614384174 |
| ODAM | Odontogenic, Ameloblast Associated | Protein Coding | 31 | GC04P070195 | 2.612930298 |
| GUCY2D | Guanylate Cyclase 2D, Retinal | Protein Coding | 42 | GC17P008002 | 2.612608433 |
| GLCE | Glucuronic Acid Epimerase | Protein Coding | 35 | GC15P114663 | 2.6121068 |
| HRH4 | Histamine Receptor H4 | Protein Coding | 40 | GC18P024460 | 2.612002373 |
| MAFB | MAF BZIP Transcription Factor B | Protein Coding | 39 | GC20M040685 | 2.611542702 |
| SMNDC1 | Survival Motor Neuron Domain Containing 1 | Protein Coding | 34 | GC10M110290 | 2.611290932 |
| DZIP1L | DAZ Interacting Zinc Finger Protein 1 Like | Protein Coding | 32 | GC03M138061 | 2.610260963 |
| CDKAL1 | CDK5 Regulatory Subunit Associated Protein 1 Like 1 | Protein Coding | 35 | GC06P020534 | 2.610032082 |
| POLL | DNA Polymerase Lambda | Protein Coding | 39 | GC10M101578 | 2.609117508 |
| TBX19 | T-Box Transcription Factor 19 | Protein Coding | 39 | GC01P168280 | 2.607843399 |
| EGFL6 | EGF Like Domain Multiple 6 | Protein Coding | 33 | GC0XP013569 | 2.607718945 |
| LOC110599580 | CYP1B1 Promoter | Biological Region | 2 | GC02P038077 | 2.606786728 |
| DNALI1 | Dynein Axonemal Light Intermediate Chain 1 | Protein Coding | 34 | GC01P037577 | 2.605950356 |
| LINC00589 | Long Intergenic Non-Protein Coding RNA 589 | RNA Gene | 17 | GC08M029673 | 2.605912209 |
| GAGE2A | G Antigen 2A | Protein Coding | 21 | GC0XP049589 | 2.605504513 |
| FFAR4 | Free Fatty Acid Receptor 4 | Protein Coding | 38 | GC10P093566 | 2.605014086 |
| CPSF1 | Cleavage And Polyadenylation Specific Factor 1 | Protein Coding | 35 | GC08M144393 | 2.60459137 |
| HTR1B | 5-Hydroxytryptamine Receptor 1B | Protein Coding | 40 | GC06M077478 | 2.604258537 |
| LOC110806262 | Solute Carrier Family 6 Member 4 Gene Promoter | Biological Region | 2 | GC17P030235 | 2.603791237 |
| COPG2 | COPI Coat Complex Subunit Gamma 2 | Protein Coding | 33 | GC07M130506 | 2.60351038 |
| PRR33 | Proline Rich 33 | Protein Coding | 13 | GC11M003014 | 2.60277319 |
| RPS11 | Ribosomal Protein S11 | Protein Coding | 35 | GC19P049496 | 2.601858616 |
| HPSE2 | Heparanase 2 (Inactive) | Protein Coding | 38 | GC10M098457 | 2.601500511 |
| NFAT5 | Nuclear Factor Of Activated T Cells 5 | Protein Coding | 38 | GC16P069565 | 2.601387262 |
| SF3A3 | Splicing Factor 3a Subunit 3 | Protein Coding | 32 | GC01M037956 | 2.601308346 |
| FKBP1A | FKBP Prolyl Isomerase 1A | Protein Coding | 41 | GC20M001369 | 2.600098848 |
| HOXA7 | Homeobox A7 | Protein Coding | 36 | GC07M027153 | 2.598751545 |
| ORM1 | Orosomucoid 1 | Protein Coding | 37 | GC09P114323 | 2.598592281 |
| CBFA2T2 | CBFA2/RUNX1 Partner Transcriptional Co-Repressor 2 | Protein Coding | 34 | GC20P033490 | 2.597445965 |
| DNAH11 | Dynein Axonemal Heavy Chain 11 | Protein Coding | 39 | GC07P021543 | 2.596889496 |
| NCBP1 | Nuclear Cap Binding Protein Subunit 1 | Protein Coding | 34 | GC09P097633 | 2.595932007 |
| TRIM22 | Tripartite Motif Containing 22 | Protein Coding | 35 | GC11P005689 | 2.595637321 |
| NPY1R | Neuropeptide Y Receptor Y1 | Protein Coding | 42 | GC04M163323 | 2.59510231 |
| NPHS2 | NPHS2 Stomatin Family Member, Podocin | Protein Coding | 38 | GC01M179519 | 2.594355106 |
| SLC30A1 | Solute Carrier Family 30 Member 1 | Protein Coding | 36 | GC01M211571 | 2.593285561 |
| ADORA2B | Adenosine A2b Receptor | Protein Coding | 42 | GC17P015927 | 2.592259884 |
| KCNB2 | Potassium Voltage-Gated Channel Subfamily B Member 2 | Protein Coding | 39 | GC08P072532 | 2.592222452 |
| SMUG1 | Single-Strand-Selective Monofunctional Uracil-DNA Glycosylase 1 | Protein Coding | 38 | GC12M054121 | 2.592015028 |
| TOMM6 | Translocase Of Outer Mitochondrial Membrane 6 | Protein Coding | 26 | GC06P041787 | 2.591402054 |
| MDH1 | Malate Dehydrogenase 1 | Protein Coding | 41 | GC02P063557 | 2.59091568 |
| PDK2 | Pyruvate Dehydrogenase Kinase 2 | Protein Coding | 39 | GC17P053534 | 2.590689182 |
| PCCB | Propionyl-CoA Carboxylase Subunit Beta | Protein Coding | 42 | GC03P136250 | 2.589411497 |
| GNG7 | G Protein Subunit Gamma 7 | Protein Coding | 35 | GC19M002511 | 2.589020729 |
| MELTF | Melanotransferrin | Protein Coding | 34 | GC03M196980 | 2.588544369 |
| FBH1 | F-Box DNA Helicase 1 | Protein Coding | 26 | GC10P005889 | 2.588430882 |
| HOXC6 | Homeobox C6 | Protein Coding | 34 | GC12P053990 | 2.588206768 |
| PABPC4 | Poly(A) Binding Protein Cytoplasmic 4 | Protein Coding | 36 | GC01M039560 | 2.588050365 |
| INF2 | Inverted Formin 2 | Protein Coding | 36 | GC14P109279 | 2.587997437 |
| SSNA1 | SS Nuclear Autoantigen 1 | Protein Coding | 32 | GC09P137188 | 2.587493658 |
| MIR194-1 | MicroRNA 194-1 | RNA Gene | 16 | GC01M220118 | 2.585705996 |
| DMC1 | DNA Meiotic Recombinase 1 | Protein Coding | 38 | GC22M056099 | 2.585481644 |
| TBX6 | T-Box Transcription Factor 6 | Protein Coding | 38 | GC16M030085 | 2.585333824 |
| TRPC3 | Transient Receptor Potential Cation Channel Subfamily C Member 3 | Protein Coding | 43 | GC04M121879 | 2.585152149 |
| KIF22 | Kinesin Family Member 22 | Protein Coding | 40 | GC16P040189 | 2.584855556 |
| CREG1 | Cellular Repressor Of E1A Stimulated Genes 1 | Protein Coding | 35 | GC01M167498 | 2.584205389 |
| LOC109621227 | Nuclear Receptor Coactivator 3 Repeat Instability Region | Biological Region | 2 | GC20P047653 | 2.583591938 |
| DHTKD1 | Dehydrogenase E1 And Transketolase Domain Containing 1 | Protein Coding | 38 | GC10P012068 | 2.583442211 |
| NEUROG1 | Neurogenin 1 | Protein Coding | 35 | GC05M135534 | 2.583377838 |
| JRK | Jrk Helix-Turn-Helix Protein | Protein Coding | 32 | GC08M142657 | 2.582642794 |
| ARC | Activity Regulated Cytoskeleton Associated Protein | Protein Coding | 33 | GC08M142611 | 2.582564354 |
| BPTF | Bromodomain PHD Finger Transcription Factor | Protein Coding | 37 | GC17P067825 | 2.58221221 |
| INTS11 | Integrator Complex Subunit 11 | Protein Coding | 29 | GC01M005536 | 2.581918716 |

| | | | | | |
|---|---|---|---|---|---|
| RBMX | RNA Binding Motif Protein X-Linked | Protein Coding | 38 | GC0XM136848 | 2.581204414 |
| DNAJA1 | DnaJ Heat Shock Protein Family (Hsp40) Member A1 | Protein Coding | 36 | GC09P033025 | 2.58114934 |
| PRKCG | Protein Kinase C Gamma | Protein Coding | 46 | GC19P053879 | 2.580849171 |
| H3C10 | H3 Clustered Histone 10 | Protein Coding | 27 | GC06P079942 | 2.580686331 |
| RPL12 | Ribosomal Protein L12 | Protein Coding | 36 | GC09M127447 | 2.580557346 |
| NPAT | Nuclear Protein, Coactivator Of Histone Transcription | Protein Coding | 31 | GC11M108157 | 2.580450058 |
| ZDBF2 | Zinc Finger DBF-Type Containing 2 | Protein Coding | 28 | GC02P206277 | 2.580113411 |
| KCNJ2 | Potassium Inwardly Rectifying Channel Subfamily J Member 2 | Protein Coding | 43 | GC17P070168 | 2.580093384 |
| ATP6V1B2 | ATPase H+ Transporting V1 Subunit B2 | Protein Coding | 43 | GC08P020197 | 2.579296589 |
| ROBO2 | Roundabout Guidance Receptor 2 | Protein Coding | 39 | GC03P075955 | 2.578211308 |
| HPX | Hemopexin | Protein Coding | 37 | GC11M006438 | 2.576322317 |
| RNF4 | Ring Finger Protein 4 | Protein Coding | 36 | GC04P002462 | 2.576100349 |
| MICU1 | Mitochondrial Calcium Uptake 1 | Protein Coding | 36 | GC10M072367 | 2.575874329 |
| SERPINB6 | Serpin Family B Member 6 | Protein Coding | 39 | GC06M002948 | 2.575766802 |
| CENPH | Centromere Protein H | Protein Coding | 30 | GC05P069189 | 2.575582981 |
| NACA | Nascent Polypeptide Associated Complex Subunit Alpha | Protein Coding | 34 | GC12M056712 | 2.57503438 |
| IFITM3 | Interferon Induced Transmembrane Protein 3 | Protein Coding | 38 | GC11M000319 | 2.574710846 |
| TMEM106B | Transmembrane Protein 106B | Protein Coding | 35 | GC07P012227 | 2.57439518 |
| TRIP10 | Thyroid Hormone Receptor Interactor 10 | Protein Coding | 35 | GC19P006737 | 2.573705673 |
| CKMT1A | Creatine Kinase, Mitochondrial 1A | Protein Coding | 33 | GC15P043693 | 2.573049068 |
| ZNFX1 | Zinc Finger NFX1-Type Containing 1 | Protein Coding | 31 | GC20M049238 | 2.572900295 |
| TTLL5 | Tubulin Tyrosine Ligase Like 5 | Protein Coding | 36 | GC14P075633 | 2.572773218 |
| TCAP | Titin-Cap | Protein Coding | 38 | GC17P053039 | 2.571609259 |
| SLC2A5 | Solute Carrier Family 2 Member 5 | Protein Coding | 39 | GC01M009036 | 2.571222305 |
| NR6A1 | Nuclear Receptor Subfamily 6 Group A Member 1 | Protein Coding | 37 | GC09M124517 | 2.57111907 |
| MXD1 | MAX Dimerization Protein 1 | Protein Coding | 35 | GC02P069897 | 2.570840836 |
| USP37 | Ubiquitin Specific Peptidase 37 | Protein Coding | 34 | GC02M218450 | 2.570505619 |
| HTR1A | 5-Hydroxytryptamine Receptor 1A | Protein Coding | 42 | GC05M063960 | 2.570267916 |
| PTPN23 | Protein Tyrosine Phosphatase Non-Receptor Type 23 | Protein Coding | 38 | GC03P047433 | 2.569894791 |
| PHF7 | PHD Finger Protein 7 | Protein Coding | 32 | GC03P052411 | 2.56982708 |
| AHCTF1 | AT-Hook Containing Transcription Factor 1 | Protein Coding | 32 | GC01M246840 | 2.568848133 |
| LSM2 | LSM2 Homolog, U6 Small Nuclear RNA And MRNA Degradation Associated | Protein Coding | 35 | GC06M063624 | 2.568380833 |
| GPC2 | Glypican 2 | Protein Coding | 33 | GC07M101722 | 2.568309784 |
| CHTOP | Chromatin Target Of PRMT1 | Protein Coding | 29 | GC01P153633 | 2.567469835 |
| THRAP3 | Thyroid Hormone Receptor Associated Protein 3 | Protein Coding | 34 | GC01P036224 | 2.567414761 |
| RAB10 | RAB10, Member RAS Oncogene Family | Protein Coding | 38 | GC02P026033 | 2.566298008 |
| TSR1 | TSR1 Ribosome Maturation Factor | Protein Coding | 35 | GC17M002322 | 2.565351963 |
| KCTD2 | Potassium Channel Tetramerization Domain Containing 2 | Protein Coding | 31 | GC17P075032 | 2.5650599 |
| ADAM23 | ADAM Metallopeptidase Domain 23 | Protein Coding | 38 | GC02P206443 | 2.564001322 |
| D2HGDH | D-2-Hydroxyglutarate Dehydrogenase | Protein Coding | 37 | GC02P241734 | 2.563413143 |
| CREBL2 | CAMP Responsive Element Binding Protein Like 2 | Protein Coding | 30 | GC12P012611 | 2.561595201 |
| PPP1CC | Protein Phosphatase 1 Catalytic Subunit Gamma | Protein Coding | 39 | GC12M110709 | 2.561155319 |
| ADCY2 | Adenylate Cyclase 2 | Protein Coding | 40 | GC05P007396 | 2.561002731 |
| RASGEF1A | RasGEF Domain Family Member 1A | Protein Coding | 34 | GC10M043194 | 2.56099081 |
| XCL1 | X-C Motif Chemokine Ligand 1 | Protein Coding | 34 | GC01P168576 | 2.560822964 |
| C8orf34 | Chromosome 8 Open Reading Frame 34 | Protein Coding | 27 | GC08P068330 | 2.560687065 |
| UBE2E1 | Ubiquitin Conjugating Enzyme E2 E1 | Protein Coding | 35 | GC03P023805 | 2.55991745 |
| AEBP1 | AE Binding Protein 1 | Protein Coding | 38 | GC07P044106 | 2.559854269 |
| CDC26 | Cell Division Cycle 26 | Protein Coding | 28 | GC09M113255 | 2.559766769 |
| CAMK1 | Calcium/Calmodulin Dependent Protein Kinase I | Protein Coding | 39 | GC03M009774 | 2.559365273 |
| PRRG1 | Proline Rich And Gla Domain 1 | Protein Coding | 29 | GC0XP037350 | 2.559178829 |
| ZFP42 | ZFP42 Zinc Finger Protein | Protein Coding | 35 | GC04P187995 | 2.558676481 |
| EFCAB13 | EF-Hand Calcium Binding Domain 13 | Protein Coding | 26 | GC17P047323 | 2.558128834 |
| TBC1D10A | TBC1 Domain Family Member 10A | Protein Coding | 32 | GC22M030291 | 2.55731678 |
| DNA2 | DNA Replication Helicase/Nuclease 2 | Protein Coding | 36 | GC10M068414 | 2.556247234 |
| CPB2 | Carboxypeptidase B2 | Protein Coding | 41 | GC13M046053 | 2.554865837 |
| TDP1 | Tyrosyl-DNA Phosphodiesterase 1 | Protein Coding | 41 | GC14P089954 | 2.554853439 |
| GFER | Growth Factor, Augmenter Of Liver Regeneration | Protein Coding | 41 | GC16P001984 | 2.552116632 |
| AGPAT2 | 1-Acylglycerol-3-Phosphate O-Acyltransferase 2 | Protein Coding | 41 | GC09M136673 | 2.552103758 |
| MIR155HG | MIR155 Host Gene | RNA Gene | 21 | GC21P025585 | 2.551664829 |
| MT-TK | Mitochondrially Encoded TRNA-Lys (AAA/G) | RNA Gene | 13 | GCMTP008297 | 2.551119089 |
| MTNR1A | Melatonin Receptor 1A | Protein Coding | 40 | GC04M186533 | 2.550995588 |
| BAIAP2-DT | BAIAP2 Divergent Transcript | RNA Gene | 14 | GC17M081103 | 2.550912857 |
| IDUA | Alpha-L-Iduronidase | Protein Coding | 40 | GC04P000986 | 2.55066824 |
| RPL34 | Ribosomal Protein L34 | Protein Coding | 34 | GC04P108620 | 2.55025053 |
| SLC25A3 | Solute Carrier Family 25 Member 3 | Protein Coding | 40 | GC12P098593 | 2.550218821 |
| RSU1 | Ras Suppressor Protein 1 | Protein Coding | 35 | GC10M016672 | 2.549816847 |
| DCAF8 | DDB1 And CUL4 Associated Factor 8 | Protein Coding | 34 | GC01M160215 | 2.549763918 |
| TRAPPC12 | Trafficking Protein Particle Complex Subunit 12 | Protein Coding | 34 | GC02P003383 | 2.549725056 |
| EEC1 | Ectrodactyly, Ectodermal Dysplasia And Cleft Lip/Palate Syndrome 1 | Genetic Locus | 2 | GC07U990021 | 2.549584389 |
| MASTL | Microtubule Associated Serine/Threonine Kinase Like | Protein Coding | 38 | GC10P027154 | 2.548643351 |
| NAB1 | NGFI-A Binding Protein 1 | Protein Coding | 35 | GC02P190646 | 2.548116684 |
| CHRNA7 | Cholinergic Receptor Nicotinic Alpha 7 Subunit | Protein Coding | 43 | GC15P031923 | 2.547417641 |
| TNNT3 | Troponin T3, Fast Skeletal Type | Protein Coding | 39 | GC11P001920 | 2.546760082 |
| MRPS34 | Mitochondrial Ribosomal Protein S34 | Protein Coding | 33 | GC16M001771 | 2.54652071 |
| MIA | MIA SH3 Domain Containing | Protein Coding | 34 | GC19P040771 | 2.546228886 |
| GPR143 | G Protein-Coupled Receptor 143 | Protein Coding | 38 | GC0XM009725 | 2.545435429 |
| CARD10 | Caspase Recruitment Domain Family Member 10 | Protein Coding | 36 | GC22M056097 | 2.54496479 |
| TSEN54 | TRNA Splicing Endonuclease Subunit 54 | Protein Coding | 34 | GC17P075515 | 2.54339695 |
| DNM3 | Dynamin 3 | Protein Coding | 39 | GC01P171848 | 2.542904854 |
| COL9A1 | Collagen Type IX Alpha 1 Chain | Protein Coding | 38 | GC06M070215 | 2.542312622 |
| EMX2 | Empty Spiracles Homeobox 2 | Protein Coding | 40 | GC10P117542 | 2.541829109 |
| FOXJ1 | Forkhead Box J1 | Protein Coding | 36 | GC17M076136 | 2.541387558 |
| STOM | Stomatin | Protein Coding | 34 | GC09M121338 | 2.540901661 |
| SLC8A1 | Solute Carrier Family 8 Member A1 | Protein Coding | 40 | GC02M040078 | 2.540119648 |
| BOP1 | BOP1 Ribosomal Biogenesis Factor | Protein Coding | 32 | GC08M144262 | 2.539777756 |
| ANKRD36B | Ankyrin Repeat Domain 36B | Protein Coding | 25 | GC02M098052 | 2.539399862 |
| USP54 | Ubiquitin Specific Peptidase 54 | Protein Coding | 31 | GC10M073497 | 2.539376736 |
| DBF4 | DBF4 Zinc Finger | Protein Coding | 35 | GC07P087877 | 2.539066792 |
| EFS | Embryonal Fyn-Associated Substrate | Protein Coding | 32 | GC14M023356 | 2.539065599 |
| DYNC2LI1 | Dynein Cytoplasmic 2 Light Intermediate Chain 1 | Protein Coding | 35 | GC02P043786 | 2.538765669 |
| TRIM8 | Tripartite Motif Containing 8 | Protein Coding | 35 | GC10P102643 | 2.538728952 |
| KRTAP10-8 | Keratin Associated Protein 10-8 | Protein Coding | 27 | GC21P044612 | 2.537491322 |
| CTU1 | Cytosolic Thiouridylase Subunit 1 | Protein Coding | 30 | GC19M051097 | 2.537320137 |
| APOC4 | Apolipoprotein C4 | Protein Coding | 34 | GC19P063847 | 2.537313938 |

| | | | | | |
|---|---|---|---|---|---|
| SIX6 | SIX Homeobox 6 | Protein Coding | 39 | GC14P060508 | 2.535941601 |
| RFX5 | Regulatory Factor X5 | Protein Coding | 38 | GC01M151340 | 2.535386324 |
| PSMB2 | Proteasome 20S Subunit Beta 2 | Protein Coding | 36 | GC01M035599 | 2.534930468 |
| TSSC4 | Tumor Suppressing Subtransferable Candidate 4 | Protein Coding | 29 | GC11P002518 | 2.532859087 |
| CDCA5 | Cell Division Cycle Associated 5 | Protein Coding | 34 | GC11M087239 | 2.532256365 |
| GGCX | Gamma-Glutamyl Carboxylase | Protein Coding | 43 | GC02M085544 | 2.531984329 |
| FCER2 | Fc Epsilon Receptor II | Protein Coding | 39 | GC19M007689 | 2.531599045 |
| RPRM | Reprimo, TP53 Dependent G2 Arrest Mediator Homolog | Protein Coding | 32 | GC02M153477 | 2.531587601 |
| ZYX | Zyxin | Protein Coding | 40 | GC07P143381 | 2.531308413 |
| RGS4 | Regulator Of G Protein Signaling 4 | Protein Coding | 39 | GC01P163038 | 2.530745745 |
| AGA | Aspartylglucosaminidase | Protein Coding | 40 | GC04M177430 | 2.530196667 |
| UNC5A | Unc-5 Netrin Receptor A | Protein Coding | 35 | GC05P177079 | 2.529761791 |
| SPOCD1 | SPOC Domain Containing 1 | Protein Coding | 27 | GC01M031790 | 2.529663086 |
| CPLANE1 | Ciliogenesis And Planar Polarity Effector Complex Subunit 1 | Protein Coding | 28 | GC05M037221 | 2.529584408 |
| NEBL | Nebulette | Protein Coding | 35 | GC10M020779 | 2.529383183 |
| BBS10 | Bardet-Biedl Syndrome 10 | Protein Coding | 36 | GC12M076344 | 2.528635025 |
| GCLM | Glutamate-Cysteine Ligase Modifier Subunit | Protein Coding | 36 | GC01M093885 | 2.527311087 |
| STAT2 | Signal Transducer And Activator Of Transcription 2 | Protein Coding | 42 | GC12M056341 | 2.527175903 |
| C1RL | Complement C1r Subcomponent Like | Protein Coding | 36 | GC12M007683 | 2.527092457 |
| TYROBP | Transmembrane Immune Signaling Adaptor TYROBP | Protein Coding | 39 | GC19M035904 | 2.525931597 |
| WDR88 | WD Repeat Domain 88 | Protein Coding | 29 | GC19P033132 | 2.525918007 |
| EDC3 | Enhancer Of MRNA Decapping 3 | Protein Coding | 37 | GC15M081446 | 2.525587082 |
| ZNF655 | Zinc Finger Protein 655 | Protein Coding | 32 | GC07P099678 | 2.525318146 |
| GRWD1 | Glutamate Rich WD Repeat Containing 1 | Protein Coding | 28 | GC19P048445 | 2.525007248 |
| TIPARP | TCDD Inducible Poly(ADP-Ribose) Polymerase | Protein Coding | 32 | GC03P156673 | 2.524313927 |
| ERMARD | ER Membrane Associated RNA Degradation | Protein Coding | 29 | GC06P169751 | 2.523080349 |
| AMPD2 | Adenosine Monophosphate Deaminase 2 | Protein Coding | 42 | GC01P109616 | 2.52214241 |
| TRPC1 | Transient Receptor Potential Cation Channel Subfamily C Member 1 | Protein Coding | 37 | GC03P142724 | 2.521846533 |
| SERPINB9 | Serpin Family B Member 9 | Protein Coding | 35 | GC06M002887 | 2.521509171 |
| MIR484 | MicroRNA 484 | RNA Gene | 17 | GC16P017316 | 2.521086693 |
| PARVB | Parvin Beta | Protein Coding | 35 | GC22P043999 | 2.520448446 |
| HMGB3 | High Mobility Group Box 3 | Protein Coding | 37 | GC0XP150980 | 2.520318508 |
| PDCD6IP | Programmed Cell Death 6 Interacting Protein | Protein Coding | 38 | GC03P033798 | 2.519750118 |
| ARPC1B | Actin Related Protein 2/3 Complex Subunit 1B | Protein Coding | 38 | GC07P099374 | 2.51783514 |
| SLC17A6 | Solute Carrier Family 17 Member 6 | Protein Coding | 36 | GC11P022359 | 2.516970634 |
| TAAR6 | Trace Amine Associated Receptor 6 | Protein Coding | 34 | GC06P132570 | 2.516260862 |
| RPL21 | Ribosomal Protein L21 | Protein Coding | 39 | GC13P027251 | 2.516070366 |
| VSIG1 | V-Set And Immunoglobulin Domain Containing 1 | Protein Coding | 32 | GC0XP108044 | 2.514871597 |
| MIR208A | MicroRNA 208a | RNA Gene | 17 | GC14M023388 | 2.514838934 |
| GPAM | Glycerol-3-Phosphate Acyltransferase, Mitochondrial | Protein Coding | 38 | GC10M112148 | 2.514178276 |
| OXA1L | OXA1L Mitochondrial Inner Membrane Protein | Protein Coding | 35 | GC14P022766 | 2.514031887 |
| DNAJC1 | DnaJ Heat Shock Protein Family (Hsp40) Member C1 | Protein Coding | 32 | GC10M021762 | 2.512548923 |
| CLEC4M | C-Type Lectin Domain Family 4 Member M | Protein Coding | 36 | GC19P007763 | 2.511719942 |
| KCTD10 | Potassium Channel Tetramerization Domain Containing 10 | Protein Coding | 32 | GC12M109448 | 2.511675835 |
| AMIGO2 | Adhesion Molecule With Ig Like Domain 2 | Protein Coding | 35 | GC12M047077 | 2.510870218 |
| PEX5 | Peroxisomal Biogenesis Factor 5 | Protein Coding | 39 | GC12P019864 | 2.510111809 |
| MIR760 | MicroRNA 760 | RNA Gene | 18 | GC01P093846 | 2.509376049 |
| ABAT | 4-Aminobutyrate Aminotransferase | Protein Coding | 41 | GC16P008674 | 2.509218454 |
| UTRN | Utrophin | Protein Coding | 36 | GC06P144285 | 2.509115696 |
| ZWILCH | Zwilch Kinetochore Protein | Protein Coding | 31 | GC15P066504 | 2.508953571 |
| RNF5 | Ring Finger Protein 5 | Protein Coding | 38 | GC06P080385 | 2.50823164 |
| RPL30 | Ribosomal Protein L30 | Protein Coding | 35 | GC08M098024 | 2.508000612 |
| TNFAIP8L2 | TNF Alpha Induced Protein 8 Like 2 | Protein Coding | 30 | GC01P151156 | 2.507953644 |
| STX3 | Syntaxin 3 | Protein Coding | 37 | GC11P059713 | 2.507464409 |
| ACTBL2 | Actin Beta Like 2 | Protein Coding | 31 | GC05M057480 | 2.507463217 |
| TRAF5 | TNF Receptor Associated Factor 5 | Protein Coding | 37 | GC01P211326 | 2.507405519 |
| CYC1 | Cytochrome C1 | Protein Coding | 41 | GC08P144095 | 2.506602764 |
| H2AC14 | H2A Clustered Histone 14 | Protein Coding | 26 | GC06M063337 | 2.506580353 |
| TRA2B | Transformer 2 Beta Homolog | Protein Coding | 35 | GC03M185914 | 2.50597024 |
| PCDH12 | Protocadherin 12 | Protein Coding | 37 | GC05M141943 | 2.505965471 |
| PCDH10 | Protocadherin 10 | Protein Coding | 35 | GC04P133149 | 2.504435301 |
| CACNA1B | Calcium Voltage-Gated Channel Subunit Alpha1 B | Protein Coding | 44 | GC09P137877 | 2.504081726 |
| CDS1 | CDP-Diacylglycerol Synthase 1 | Protein Coding | 37 | GC04P084582 | 2.504021168 |
| PIP5K1A | Phosphatidylinositol-4-Phosphate 5-Kinase Type 1 Alpha | Protein Coding | 38 | GC01P151198 | 2.503987789 |
| FAM20A | FAM20A Golgi Associated Secretory Pathway Pseudokinase | Protein Coding | 37 | GC17M068535 | 2.503750324 |
| ARHGEF11 | Rho Guanine Nucleotide Exchange Factor 11 | Protein Coding | 37 | GC01M156904 | 2.503446102 |
| NAV3 | Neuron Navigator 3 | Protein Coding | 31 | GC12P077341 | 2.503327131 |
| RPL36 | Ribosomal Protein L36 | Protein Coding | 35 | GC19P005674 | 2.503295422 |
| MIIP | Migration And Invasion Inhibitory Protein | Protein Coding | 31 | GC01P012019 | 2.50320363 |
| PABPN1 | Poly(A) Binding Protein Nuclear 1 | Protein Coding | 40 | GC14P032055 | 2.502999306 |
| COPG1 | COPI Coat Complex Subunit Gamma 1 | Protein Coding | 34 | GC03P129249 | 2.50237751 |
| ZNF165 | Zinc Finger Protein 165 | Protein Coding | 32 | GC06P028080 | 2.502162218 |
| ERF | ETS2 Repressor Factor | Protein Coding | 38 | GC19M042247 | 2.501796722 |
| THADA | THADA Armadillo Repeat Containing | Protein Coding | 34 | GC02M043193 | 2.501309633 |
| FPR1 | Formyl Peptide Receptor 1 | Protein Coding | 43 | GC19M051745 | 2.50109148 |
| MIR543 | MicroRNA 543 | RNA Gene | 16 | GC14P109248 | 2.500061035 |
| RPL18A | Ribosomal Protein L18a | Protein Coding | 34 | GC19P063112 | 2.499806881 |
| HNRNPUL1 | Heterogeneous Nuclear Ribonucleoprotein U Like 1 | Protein Coding | 34 | GC19P041262 | 2.499798298 |
| EVL | Enah/Vasp-Like | Protein Coding | 34 | GC14P099971 | 2.4992733 |
| APOC1 | Apolipoprotein C1 | Protein Coding | 35 | GC19P044914 | 2.499152184 |
| FSTL3 | Follistatin Like 3 | Protein Coding | 36 | GC19P000676 | 2.498929262 |
| FGD1 | FYVE, RhoGEF And PH Domain Containing 1 | Protein Coding | 36 | GC0XM054488 | 2.498354197 |
| DDOST | Dolichyl-Diphosphooligosaccharide--Protein Glycosyltransferase Non-Catalytic Subunit | Protein Coding | 40 | GC01M020651 | 2.497781038 |
| GMPS | Guanine Monophosphate Synthase | Protein Coding | 39 | GC03P155870 | 2.49584651 |
| TOR1A | Torsin Family 1 Member A | Protein Coding | 41 | GC09M129812 | 2.495778084 |
| IPMK | Inositol Polyphosphate Multikinase | Protein Coding | 34 | GC10M058191 | 2.495694399 |
| COP1 | COP1 E3 Ubiquitin Ligase | Protein Coding | 32 | GC01M175944 | 2.495130301 |
| HLF | HLF Transcription Factor, PAR BZIP Family Member | Protein Coding | 35 | GC17P055264 | 2.494199038 |
| HEPACAM2 | HEPACAM Family Member 2 | Protein Coding | 31 | GC07M093188 | 2.494125843 |
| DNAAF6 | Dynein Axonemal Assembly Factor 6 | Protein Coding | 26 | GC0XP107207 | 2.494093895 |
| TCL6 | T Cell Leukemia/Lymphoma 6 | RNA Gene | 24 | GC14P095650 | 2.493520737 |
| SCARF2 | Scavenger Receptor Class F Member 2 | Protein Coding | 37 | GC22M020424 | 2.492958069 |
| METAP1D | Methionyl Aminopeptidase Type 1D, Mitochondrial | Protein Coding | 32 | GC02P172000 | 2.49209857 |
| RPL13A | Ribosomal Protein L13a | Protein Coding | 38 | GC19P049487 | 2.491897583 |

| | | | | | |
|---|---|---|---|---|---|
| DDX43 | DEAD-Box Helicase 43 | Protein Coding | 31 | GC06P073394 | 2.491553307 |
| SPICE1 | Spindle And Centriole Associated Protein 1 | Protein Coding | 30 | GC03M113442 | 2.491317272 |
| ZNF180 | Zinc Finger Protein 180 | Protein Coding | 33 | GC19M063920 | 2.490529537 |
| GPR87 | G Protein-Coupled Receptor 87 | Protein Coding | 34 | GC03M151294 | 2.490199804 |
| CCNI | Cyclin I | Protein Coding | 32 | GC04M077047 | 2.489927769 |
| SRI | Sorcin | Protein Coding | 39 | GC07M088205 | 2.489658594 |
| SDCBP2 | Syndecan Binding Protein 2 | Protein Coding | 33 | GC20M001309 | 2.488689661 |
| LRRC59 | Leucine Rich Repeat Containing 59 | Protein Coding | 31 | GC17M050375 | 2.488158226 |
| BHMT | Betaine--Homocysteine S-Methyltransferase | Protein Coding | 36 | GC05P079111 | 2.488028526 |
| SEPTIN14 | Septin 14 | Protein Coding | 23 | GC07M060186 | 2.487905502 |
| OASL | 2'-5'-Oligoadenylate Synthetase Like | Protein Coding | 36 | GC12M122126 | 2.487155914 |
| VKORC1 | Vitamin K Epoxide Reductase Complex Subunit 1 | Protein Coding | 43 | GC16M036578 | 2.486868858 |
| ADGRG6 | Adhesion G Protein-Coupled Receptor G6 | Protein Coding | 36 | GC06P142301 | 2.486080408 |
| CDK2AP2 | Cyclin Dependent Kinase 2 Associated Protein 2 | Protein Coding | 31 | GC11M067506 | 2.485495806 |
| PIF1 | PIF1 5'-To-3' DNA Helicase | Protein Coding | 34 | GC15M064815 | 2.48544383 |
| TSLP | Thymic Stromal Lymphopoietin | Protein Coding | 36 | GC05P111070 | 2.484405041 |
| CFB | Complement Factor B | Protein Coding | 42 | GC06P031945 | 2.483677626 |
| ANKRD28 | Ankyrin Repeat Domain 28 | Protein Coding | 32 | GC03M015667 | 2.483577967 |
| SLC39A12 | Solute Carrier Family 39 Member 12 | Protein Coding | 32 | GC10P017951 | 2.483439207 |
| CHD2 | Chromodomain Helicase DNA Binding Protein 2 | Protein Coding | 40 | GC15P092900 | 2.482123137 |
| H2AC11 | H2A Clustered Histone 11 | Protein Coding | 28 | GC06P081030 | 2.481210232 |
| GMPR2 | Guanosine Monophosphate Reductase 2 | Protein Coding | 34 | GC14P024232 | 2.48072052 |
| HCAR1 | Hydroxycarboxylic Acid Receptor 1 | Protein Coding | 35 | GC12M122726 | 2.480381966 |
| ING2 | Inhibitor Of Growth Family Member 2 | Protein Coding | 32 | GC04P183504 | 2.480158091 |
| HBG2 | Hemoglobin Subunit Gamma 2 | Protein Coding | 38 | GC11M006328 | 2.480004787 |
| PXDC1 | PX Domain Containing 1 | Protein Coding | 26 | GC06M003723 | 2.479635239 |
| ARID3B | AT-Rich Interaction Domain 3B | Protein Coding | 34 | GC15P074541 | 2.47962904 |
| ZC3H14 | Zinc Finger CCCH-Type Containing 14 | Protein Coding | 36 | GC14P088562 | 2.479299068 |
| SPECC1L | Sperm Antigen With Calponin Homology And Coiled-Coil Domains 1 Like | Protein Coding | 35 | GC22P034757 | 2.479208469 |
| MZF1 | Myeloid Zinc Finger 1 | Protein Coding | 33 | GC19M064558 | 2.478936434 |
| AATF | Apoptosis Antagonizing Transcription Factor | Protein Coding | 35 | GC17P036948 | 2.478820324 |
| PPL | Periplakin | Protein Coding | 36 | GC16M006910 | 2.47863555 |
| NAF1 | Nuclear Assembly Factor 1 Ribonucleoprotein | Protein Coding | 29 | GC04M163109 | 2.478553534 |
| CPLX1 | Complexin 1 | Protein Coding | 38 | GC04M000784 | 2.478449821 |
| FBXO30 | F-Box Protein 30 | Protein Coding | 28 | GC06M145795 | 2.477814198 |
| ROBO4 | Roundabout Guidance Receptor 4 | Protein Coding | 39 | GC11M124883 | 2.477426529 |
| SYT9 | Synaptotagmin 9 | Protein Coding | 33 | GC11P007238 | 2.477128983 |
| LIPI | Lipase I | Protein Coding | 33 | GC21M014108 | 2.475522995 |
| DBT | Dihydrolipoamide Branched Chain Transacylase E2 | Protein Coding | 38 | GC01M100186 | 2.475324392 |
| GNAO1 | G Protein Subunit Alpha O1 | Protein Coding | 42 | GC16P056231 | 2.474069118 |
| MRAP | Melanocortin 2 Receptor Accessory Protein | Protein Coding | 34 | GC21P032291 | 2.473018169 |
| AMBRA1 | Autophagy And Beclin 1 Regulator 1 | Protein Coding | 34 | GC11M086860 | 2.4726336 |
| MIR1271 | MicroRNA 1271 | RNA Gene | 15 | GC05P176367 | 2.472404242 |
| ZDHHC4 | Zinc Finger DHHC-Type Palmitoyltransferase 4 | Protein Coding | 31 | GC07P006577 | 2.4723382 |
| GPS2 | G Protein Pathway Suppressor 2 | Protein Coding | 31 | GC17M007311 | 2.471506834 |
| VGF | VGF Nerve Growth Factor Inducible | Protein Coding | 35 | GC07M101162 | 2.471380711 |
| SERPINA7 | Serpin Family A Member 7 | Protein Coding | 36 | GC0XM106032 | 2.471004486 |
| PKP3 | Plakophilin 3 | Protein Coding | 34 | GC11P000441 | 2.470728397 |
| TDRD3 | Tudor Domain Containing 3 | Protein Coding | 32 | GC13P060396 | 2.470594883 |
| SARNP | SAP Domain Containing Ribonucleoprotein | Protein Coding | 32 | GC12M055752 | 2.468758821 |
| CNTROB | Centrobin, Centriole Duplication And Spindle Assembly Protein | Protein Coding | 33 | GC17P007932 | 2.468580246 |
| GAR1 | GAR1 Ribonucleoprotein | Protein Coding | 34 | GC04P109815 | 2.468361378 |
| TMEM132C | Transmembrane Protein 132C | Protein Coding | 30 | GC12P128267 | 2.46822238 |
| EFHD1 | EF-Hand Domain Family Member D1 | Protein Coding | 32 | GC02P232606 | 2.468063354 |
| ISLR | Immunoglobulin Superfamily Containing Leucine Rich Repeat | Protein Coding | 33 | GC15P074173 | 2.467998505 |
| OTC | Ornithine Transcarbamylase | Protein Coding | 42 | GC0XP038353 | 2.46707654 |
| TSC22D4 | TSC22 Domain Family Member 4 | Protein Coding | 31 | GC07M100463 | 2.466551781 |
| MASP2 | MBL Associated Serine Protease 2 | Protein Coding | 42 | GC01M011026 | 2.466337919 |
| MIR448 | MicroRNA 448 | RNA Gene | 13 | GC0XP114823 | 2.46626997 |
| H3C8 | H3 Clustered Histone 8 | Protein Coding | 26 | GC06M064166 | 2.46569252 |
| ANGPTL2 | Angiopoietin Like 2 | Protein Coding | 34 | GC09M127087 | 2.465389967 |
| AIPL1 | Aryl Hydrocarbon Receptor Interacting Protein Like 1 | Protein Coding | 38 | GC17M006393 | 2.465074062 |
| MAP4K4 | Mitogen-Activated Protein Kinase Kinase Kinase Kinase 4 | Protein Coding | 43 | GC02P101696 | 2.464645863 |
| ZBTB33 | Zinc Finger And BTB Domain Containing 33 | Protein Coding | 34 | GC0XP120250 | 2.464593649 |
| RCOR1 | REST Corepressor 1 | Protein Coding | 36 | GC14P102592 | 2.464579582 |
| GNG5 | G Protein Subunit Gamma 5 | Protein Coding | 36 | GC01M084498 | 2.464531183 |
| TBC1D5 | TBC1 Domain Family Member 5 | Protein Coding | 32 | GC03M017157 | 2.463691711 |
| CA4 | Carbonic Anhydrase 4 | Protein Coding | 42 | GC17P060149 | 2.463361263 |
| ADAMTS17 | ADAM Metallopeptidase With Thrombospondin Type 1 Motif 17 | Protein Coding | 38 | GC15M099971 | 2.463191509 |
| SH3BP2 | SH3 Domain Binding Protein 2 | Protein Coding | 36 | GC04P002794 | 2.463023663 |
| LARGE1 | LARGE Xylosyl- And Glucuronyltransferase 1 | Protein Coding | 32 | GC22M036278 | 2.462776184 |
| MTF1 | Metal Regulatory Transcription Factor 1 | Protein Coding | 36 | GC01M037810 | 2.462675095 |
| P4HA1 | Prolyl 4-Hydroxylase Subunit Alpha 1 | Protein Coding | 39 | GC10M073007 | 2.462171555 |
| HLCS | Holocarboxylase Synthetase | Protein Coding | 38 | GC21M036750 | 2.461101532 |
| SEMA5A | Semaphorin 5A | Protein Coding | 37 | GC05M009036 | 2.461045027 |
| SLC9A3 | Solute Carrier Family 9 Member A3 | Protein Coding | 43 | GC05M000472 | 2.460988522 |
| RASSF6 | Ras Association Domain Family Member 6 | Protein Coding | 33 | GC04M073571 | 2.460522652 |
| NNT | Nicotinamide Nucleotide Transhydrogenase | Protein Coding | 41 | GC05P044262 | 2.459847212 |
| SYNM | Synemin | Protein Coding | 33 | GC15P099098 | 2.45964694 |
| CALR3 | Calreticulin 3 | Protein Coding | 34 | GC19M016450 | 2.4584198 |
| GDI1 | GDP Dissociation Inhibitor 1 | Protein Coding | 39 | GC0XP154436 | 2.457036972 |
| MT1G | Metallothionein 1G | Protein Coding | 29 | GC16M056666 | 2.456675768 |
| CORO1B | Coronin 1B | Protein Coding | 32 | GC11M067435 | 2.455480814 |
| TPCN2 | Two Pore Segment Channel 2 | Protein Coding | 35 | GC11P069813 | 2.454834938 |
| PTCD3 | Pentatricopeptide Repeat Domain 3 | Protein Coding | 34 | GC02P086106 | 2.454799414 |
| PDXK | Pyridoxal Kinase | Protein Coding | 42 | GC21P043719 | 2.453614712 |
| ASXL3 | ASXL Transcriptional Regulator 3 | Protein Coding | 31 | GC18P033578 | 2.453478575 |
| MOB1A | MOB Kinase Activator 1A | Protein Coding | 34 | GC02M074152 | 2.453181744 |
| ELK4 | ETS Transcription Factor ELK4 | Protein Coding | 31 | GC01M205577 | 2.452777863 |
| LOC110386949 | CYP19A1 Promoter I.7 | Biological Region | 2 | GC15P051282 | 2.452611208 |
| SETMAR | SET Domain And Mariner Transposase Fusion Gene | Protein Coding | 36 | GC03P004303 | 2.452309132 |
| LOC111589216 | BRCA1 Intron 2 Regulatory Region | Biological Region | 2 | GC17P053874 | 2.45209074 |
| ZFP91 | ZFP91 Zinc Finger Protein, Atypical E3 Ubiquitin Ligase | Protein Coding | 31 | GC11P058580 | 2.449672699 |
| NBAS | NBAS Subunit Of NRZ Tethering Complex | Protein Coding | 36 | GC02M014998 | 2.449661255 |
| B3GNT8 | UDP-GlcNAc:BetaGal Beta-1,3-N-Acetylglucosaminyltransferase 8 | Protein Coding | 31 | GC19M041425 | 2.44931078 |

| | | | | | |
|---|---|---|---|---|---|
| EPB41L5 | Erythrocyte Membrane Protein Band 4.1 Like 5 | Protein Coding | 32 | GC02P120013 | 2.448459148 |
| CNTF | Ciliary Neurotrophic Factor | Protein Coding | 36 | GC11P058622 | 2.448220015 |
| INTS14 | Integrator Complex Subunit 14 | Protein Coding | 26 | GC15M081186 | 2.448014975 |
| BTF3 | Basic Transcription Factor 3 | Protein Coding | 35 | GC05P073498 | 2.447591782 |
| TRIM63 | Tripartite Motif Containing 63 | Protein Coding | 37 | GC01M026062 | 2.44709301 |
| NFXL1 | Nuclear Transcription Factor, X-Box Binding Like 1 | Protein Coding | 31 | GC04M047849 | 2.446957827 |
| BRWD1 | Bromodomain And WD Repeat Domain Containing 1 | Protein Coding | 35 | GC21M039184 | 2.446904182 |
| PRX | Periaxin | Protein Coding | 36 | GC19M040393 | 2.446714401 |
| ENTPD1 | Ectonucleoside Triphosphate Diphosphohydrolase 1 | Protein Coding | 43 | GC10P095711 | 2.446675539 |
| SLC1A2 | Solute Carrier Family 1 Member 2 | Protein Coding | 45 | GC11M035260 | 2.446548939 |
| BEND7 | BEN Domain Containing 7 | Protein Coding | 30 | GC10M013392 | 2.445873737 |
| CXCR6 | C-X-C Motif Chemokine Receptor 6 | Protein Coding | 37 | GC03P046399 | 2.445868254 |
| TCEA3 | Transcription Elongation Factor A3 | Protein Coding | 33 | GC01M023382 | 2.445770741 |
| MKS1 | MKS Transition Zone Complex Subunit 1 | Protein Coding | 36 | GC17M058205 | 2.445618391 |
| WDR75 | WD Repeat Domain 75 | Protein Coding | 29 | GC02P189441 | 2.444826841 |
| RORC | RAR Related Orphan Receptor C | Protein Coding | 42 | GC01M151806 | 2.444783211 |
| H3C7 | H3 Clustered Histone 7 | Protein Coding | 27 | GC06M064165 | 2.444703579 |
| UQCRC1 | Ubiquinol-Cytochrome C Reductase Core Protein 1 | Protein Coding | 38 | GC03M048598 | 2.442860126 |
| PTPRE | Protein Tyrosine Phosphatase Receptor Type E | Protein Coding | 37 | GC10P127907 | 2.442804575 |
| LINC00319 | Long Intergenic Non-Protein Coding RNA 319 | RNA Gene | 19 | GC21P043446 | 2.4425385 |
| BRWD3 | Bromodomain And WD Repeat Domain Containing 3 | Protein Coding | 31 | GC0XM080669 | 2.442499638 |
| CASP14 | Caspase 14 | Protein Coding | 42 | GC19P015049 | 2.441202164 |
| COQ6 | Coenzyme Q6, Monooxygenase | Protein Coding | 37 | GC14P073949 | 2.441013098 |
| THAP10 | THAP Domain Containing 10 | Protein Coding | 27 | GC15M070881 | 2.440668344 |
| TRAIP | TRAF Interacting Protein | Protein Coding | 36 | GC03M051065 | 2.440051556 |
| MAL | Mal, T Cell Differentiation Protein | Protein Coding | 35 | GC02P095025 | 2.439842463 |
| RGN | Regucalcin | Protein Coding | 35 | GC0XP047486 | 2.439800739 |
| RAPSN | Receptor Associated Protein Of The Synapse | Protein Coding | 38 | GC11M086886 | 2.43898201 |
| RBM23 | RNA Binding Motif Protein 23 | Protein Coding | 32 | GC14M022902 | 2.438965082 |
| SORCS1 | Sortilin Related VPS10 Domain Containing Receptor 1 | Protein Coding | 35 | GC10M106573 | 2.438312531 |
| OPRD1 | Opioid Receptor Delta 1 | Protein Coding | 42 | GC01P028812 | 2.437678814 |
| FAM83F | Family With Sequence Similarity 83 Member F | Protein Coding | 30 | GC22P039994 | 2.437003613 |
| CHST11 | Carbohydrate Sulfotransferase 11 | Protein Coding | 38 | GC12P104455 | 2.436445236 |
| BCL6B | BCL6B Transcription Repressor | Protein Coding | 32 | GC17P007023 | 2.436443329 |
| PDHB | Pyruvate Dehydrogenase E1 Subunit Beta | Protein Coding | 41 | GC03M058428 | 2.436059713 |
| EIF3I | Eukaryotic Translation Initiation Factor 3 Subunit I | Protein Coding | 34 | GC01P032221 | 2.435880661 |
| TET2-AS1 | TET2 Antisense RNA 1 | RNA Gene | 14 | GC04M105171 | 2.434741497 |
| GRIN1 | Glutamate Ionotropic Receptor NMDA Type Subunit 1 | Protein Coding | 45 | GC09P137138 | 2.434660435 |
| OCRL | OCRL Inositol Polyphosphate-5-Phosphatase | Protein Coding | 40 | GC0XP129539 | 2.434470654 |
| ADRB1 | Adrenoceptor Beta 1 | Protein Coding | 43 | GC10P114044 | 2.433076382 |
| HMCN1 | Hemicentin 1 | Protein Coding | 35 | GC01P185734 | 2.433001995 |
| RASEF | RAS And EF-Hand Domain Containing | Protein Coding | 33 | GC09M093839 | 2.432940483 |
| PRR11 | Proline Rich 11 | Protein Coding | 30 | GC17P059155 | 2.432227373 |
| COMP | Cartilage Oligomeric Matrix Protein | Protein Coding | 42 | GC19M018783 | 2.432134151 |
| FSTL1 | Follistatin Like 1 | Protein Coding | 38 | GC03M120392 | 2.431631088 |
| DIO2 | Iodothyronine Deiodinase 2 | Protein Coding | 39 | GC14M080197 | 2.431392431 |
| RASL10B | RAS Like Family 10 Member B | Protein Coding | 28 | GC17P035731 | 2.431085587 |
| ADGRB1 | Adhesion G Protein-Coupled Receptor B1 | Protein Coding | 32 | GC08P142449 | 2.431056023 |
| IBA57 | Iron-Sulfur Cluster Assembly Factor IBA57 | Protein Coding | 31 | GC01P228165 | 2.430962086 |
| WDR43 | WD Repeat Domain 43 | Protein Coding | 30 | GC02P028894 | 2.430821896 |
| FLG2 | Filaggrin 2 | Protein Coding | 34 | GC01M152321 | 2.430685282 |
| NLGN3 | Neuroligin 3 | Protein Coding | 39 | GC0XP071144 | 2.43063736 |
| MEMO1 | Mediator Of Cell Motility 1 | Protein Coding | 34 | GC02M031865 | 2.430523396 |
| RGS6 | Regulator Of G Protein Signaling 6 | Protein Coding | 37 | GC14P071867 | 2.430352688 |
| PAN2 | Poly(A) Specific Ribonuclease Subunit PAN2 | Protein Coding | 32 | GC12M056316 | 2.430217743 |
| APOA4 | Apolipoprotein A4 | Protein Coding | 37 | GC11M116820 | 2.428732872 |
| MAP7D2 | MAP7 Domain Containing 2 | Protein Coding | 28 | GC0XM020006 | 2.428642988 |
| COG1 | Component Of Oligomeric Golgi Complex 1 | Protein Coding | 34 | GC17P073193 | 2.428263426 |
| ZNF331 | Zinc Finger Protein 331 | Protein Coding | 35 | GC19P064281 | 2.42811203 |
| ABHD8 | Abhydrolase Domain Containing 8 | Protein Coding | 28 | GC19M017292 | 2.427838564 |
| PACC1 | Proton Activated Chloride Channel 1 | Protein Coding | 26 | GC01M212365 | 2.427719831 |
| GATAD2A | GATA Zinc Finger Domain Containing 2A | Protein Coding | 34 | GC19P063133 | 2.426895618 |
| UGT2B4 | UDP Glucuronosyltransferase Family 2 Member B4 | Protein Coding | 37 | GC04M069484 | 2.426425219 |
| OR2B6 | Olfactory Receptor Family 2 Subfamily B Member 6 | Protein Coding | 31 | GC06P079957 | 2.425495625 |
| EHF | ETS Homologous Factor | Protein Coding | 35 | GC11P034621 | 2.424798965 |
| GDF10 | Growth Differentiation Factor 10 | Protein Coding | 32 | GC10P047300 | 2.424425125 |
| EIF2B5 | Eukaryotic Translation Initiation Factor 2B Subunit Epsilon | Protein Coding | 39 | GC03P184135 | 2.424247265 |
| DNPH1 | 2'-Deoxynucleoside 5'-Phosphate N-Hydrolase 1 | Protein Coding | 31 | GC06M063848 | 2.424214602 |
| UBE2B | Ubiquitin Conjugating Enzyme E2 B | Protein Coding | 39 | GC05P134371 | 2.42335701 |
| INO80 | INO80 Complex ATPase Subunit | Protein Coding | 33 | GC15M040979 | 2.423250437 |
| MAN2B1 | Mannosidase Alpha Class 2B Member 1 | Protein Coding | 40 | GC19M012663 | 2.422383785 |
| CHN1 | Chimerin 1 | Protein Coding | 42 | GC02M174799 | 2.421561003 |
| ODF2 | Outer Dense Fiber Of Sperm Tails 2 | Protein Coding | 34 | GC09P128455 | 2.421479225 |
| DCLRE1B | DNA Cross-Link Repair 1B | Protein Coding | 31 | GC01P113905 | 2.421258688 |
| TTC37 | Tetratricopeptide Repeat Domain 37 | Protein Coding | 35 | GC05M095463 | 2.42114687 |
| ZIC5 | Zic Family Member 5 | Protein Coding | 31 | GC13M099962 | 2.420419216 |
| COX11 | Cytochrome C Oxidase Copper Chaperone COX11 | Protein Coding | 34 | GC17M054951 | 2.420262575 |
| MMP20 | Matrix Metallopeptidase 20 | Protein Coding | 40 | GC11M102576 | 2.4194417 |
| LOC106799833 | CYP11B1 Recombination Region | Biological Region | 3 | GC08P142874 | 2.419112682 |
| S100A12 | S100 Calcium Binding Protein A12 | Protein Coding | 35 | GC01M153373 | 2.419074297 |
| PADI2 | Peptidyl Arginine Deiminase 2 | Protein Coding | 38 | GC01M017066 | 2.419002771 |
| CYP4Z2P | Cytochrome P450 Family 4 Subfamily Z Member 2, Pseudogene | Pseudogene | 15 | GC01M046843 | 2.418647051 |
| NCF1 | Neutrophil Cytosolic Factor 1 | Protein Coding | 42 | GC07P074787 | 2.418427229 |
| ADCY6 | Adenylate Cyclase 6 | Protein Coding | 43 | GC12M048766 | 2.418304205 |
| MTNR1B | Melatonin Receptor 1B | Protein Coding | 43 | GC11P092969 | 2.4182024 |
| H6PD | Hexose-6-Phosphate Dehydrogenase/Glucose 1-Dehydrogenase | Protein Coding | 39 | GC01P009234 | 2.418018818 |
| PCSK6 | Proprotein Convertase Subtilisin/Kexin Type 6 | Protein Coding | 38 | GC15M116031 | 2.417847633 |
| RAB11A | RAB11A, Member RAS Oncogene Family | Protein Coding | 42 | GC15P114654 | 2.417024136 |
| OR7C1 | Olfactory Receptor Family 7 Subfamily C Member 1 | Protein Coding | 31 | GC19M014789 | 2.416019201 |
| SEPTIN7 | Septin 7 | Protein Coding | 31 | GC07P036016 | 2.414969921 |
| CPEB1 | Cytoplasmic Polyadenylation Element Binding Protein 1 | Protein Coding | 35 | GC15M082543 | 2.41494894 |
| ACTN2 | Actinin Alpha 2 | Protein Coding | 42 | GC01P236686 | 2.414118767 |
| MSTO1 | Misato Mitochondrial Distribution And Morphology Regulator 1 | Protein Coding | 35 | GC01P155610 | 2.414062977 |
| TBC1D25 | TBC1 Domain Family Member 25 | Protein Coding | 32 | GC0XP048539 | 2.413538694 |
| CLRN1-AS1 | CLRN1 Antisense RNA 1 | RNA Gene | 17 | GC03P150852 | 2.413357019 |

| | | | | | |
|---|---|---|---|---|---|
| HLA-F | Major Histocompatibility Complex, Class I, F | Protein Coding | 36 | GC06P080295 | 2.4130795 |
| CTR9 | CTR9 Homolog, Paf1/RNA Polymerase II Complex Component | Protein Coding | 34 | GC11P010772 | 2.411877632 |
| FGR | FGR Proto-Oncogene, Src Family Tyrosine Kinase | Protein Coding | 43 | GC01M027809 | 2.411375523 |
| SLC8A3 | Solute Carrier Family 8 Member A3 | Protein Coding | 39 | GC14M070044 | 2.411301136 |
| IRAK4 | Interleukin 1 Receptor Associated Kinase 4 | Protein Coding | 42 | GC12P043758 | 2.41106081 |
| MLN | Motilin | Protein Coding | 33 | GC06M033794 | 2.411039829 |
| CHN2 | Chimerin 2 | Protein Coding | 38 | GC07P029186 | 2.410291433 |
| IFRD2 | Interferon Related Developmental Regulator 2 | Protein Coding | 32 | GC03M050287 | 2.41027379 |
| PLP1 | Proteolipid Protein 1 | Protein Coding | 38 | GC0XP103773 | 2.410091639 |
| NAA40 | N-Alpha-Acetyltransferase 40, NatD Catalytic Subunit | Protein Coding | 30 | GC11P063938 | 2.409959078 |
| TIRAP | TIR Domain Containing Adaptor Protein | Protein Coding | 38 | GC11P126284 | 2.409085035 |
| YBX2 | Y-Box Binding Protein 2 | Protein Coding | 35 | GC17M007288 | 2.408804417 |
| SMARCD2 | SWI/SNF Related, Matrix Associated, Actin Dependent Regulator Of Chromatin, Subfamily D, Member 2 | Protein Coding | 38 | GC17M063832 | 2.408246994 |
| HNRNPDL | Heterogeneous Nuclear Ribonucleoprotein D Like | Protein Coding | 36 | GC04M082422 | 2.40822053 |
| ESD | Esterase D | Protein Coding | 38 | GC13M046771 | 2.407656193 |
| DAO | D-Amino Acid Oxidase | Protein Coding | 40 | GC12P108859 | 2.40679121 |
| HCFC1 | Host Cell Factor C1 | Protein Coding | 42 | GC0XM153947 | 2.406712532 |
| EEF1B2 | Eukaryotic Translation Elongation Factor 1 Beta 2 | Protein Coding | 37 | GC02P206159 | 2.405642986 |
| DDX11 | DEAD/H-Box Helicase 11 | Protein Coding | 39 | GC12P031073 | 2.405620098 |
| NSA2 | NSA2 Ribosome Biogenesis Factor | Protein Coding | 31 | GC05P074766 | 2.405567169 |
| PPP1R12C | Protein Phosphatase 1 Regulatory Subunit 12C | Protein Coding | 30 | GC19M064393 | 2.405386925 |
| MIR135A2 | MicroRNA 135a-2 | RNA Gene | 17 | GC12P097563 | 2.404121161 |
| MIR888 | MicroRNA 888 | RNA Gene | 11 | GC0XM145996 | 2.404071093 |
| PHF5A | PHD Finger Protein 5A | Protein Coding | 30 | GC22M041459 | 2.403883457 |
| GPS1 | G Protein Pathway Suppressor 1 | Protein Coding | 34 | GC17P082050 | 2.403753757 |
| MIR153-1 | MicroRNA 153-1 | RNA Gene | 15 | GC02M219294 | 2.4032166 |
| GM2A | GM2 Ganglioside Activator | Protein Coding | 38 | GC05P151229 | 2.401146412 |
| NUDT5 | Nudix Hydrolase 5 | Protein Coding | 35 | GC10M012165 | 2.400624752 |
| PSMD7 | Proteasome 26S Subunit, Non-ATPase 7 | Protein Coding | 39 | GC16P074296 | 2.400429249 |
| SLC44A4 | Solute Carrier Family 44 Member 4 | Protein Coding | 37 | GC06M031863 | 2.400070667 |
| MIR873 | MicroRNA 873 | RNA Gene | 17 | GC09M028880 | 2.399808884 |
| GOLPH3L | Golgi Phosphoprotein 3 Like | Protein Coding | 31 | GC01M151644 | 2.399690151 |
| RAP1GDS1 | Rap1 GTPase-GDP Dissociation Stimulator 1 | Protein Coding | 38 | GC04P098261 | 2.399528027 |
| KIF6 | Kinesin Family Member 6 | Protein Coding | 34 | GC06M063777 | 2.39943099 |
| MPI | Mannose Phosphate Isomerase | Protein Coding | 39 | GC15P074890 | 2.399148941 |
| RAP1B | RAP1B, Member Of RAS Oncogene Family | Protein Coding | 39 | GC12P068610 | 2.39910078 |
| TSPAN1 | Tetraspanin 1 | Protein Coding | 36 | GC01P046175 | 2.399013281 |
| PLCD3 | Phospholipase C Delta 3 | Protein Coding | 37 | GC17M045108 | 2.398424149 |
| HSP90AA2P | Heat Shock Protein 90 Alpha Family Class A Member 2, Pseudogene | Pseudogene | 17 | GC11M027888 | 2.398225784 |
| CNKSR2 | Connector Enhancer Of Kinase Suppressor Of Ras 2 | Protein Coding | 36 | GC0XP021392 | 2.398051262 |
| IMPA1 | Inositol Monophosphatase 1 | Protein Coding | 42 | GC08M081656 | 2.39703846 |
| DOCK5 | Dedicator Of Cytokinesis 5 | Protein Coding | 34 | GC08P025184 | 2.396995068 |
| MUC17 | Mucin 17, Cell Surface Associated | Protein Coding | 32 | GC07P101020 | 2.396907806 |
| BBS5 | Bardet-Biedl Syndrome 5 | Protein Coding | 35 | GC02P169480 | 2.396551609 |
| FGL2 | Fibrinogen Like 2 | Protein Coding | 37 | GC07M077193 | 2.396027803 |
| IMMT | Inner Membrane Mitochondrial Protein | Protein Coding | 37 | GC02M086144 | 2.395829201 |
| VAPA | VAMP Associated Protein A | Protein Coding | 38 | GC18P009904 | 2.395811081 |
| DCTN4 | Dynactin Subunit 4 | Protein Coding | 34 | GC05M150708 | 2.395807743 |
| TNFAIP2 | TNF Alpha Induced Protein 2 | Protein Coding | 35 | GC14P109261 | 2.394309759 |
| GRM5 | Glutamate Metabotropic Receptor 5 | Protein Coding | 43 | GC11M088504 | 2.394130468 |
| TAF8 | TATA-Box Binding Protein Associated Factor 8 | Protein Coding | 32 | GC06P042050 | 2.393595934 |
| MED27 | Mediator Complex Subunit 27 | Protein Coding | 32 | GC09M131860 | 2.393185377 |
| MGAT2 | Alpha-1,6-Mannosyl-Glycoprotein 2-Beta-N-Acetylglucosaminyltransferase | Protein Coding | 39 | GC14P049620 | 2.392751455 |
| VDAC2 | Voltage Dependent Anion Channel 2 | Protein Coding | 39 | GC10P075210 | 2.391092777 |
| RPS6KA4 | Ribosomal Protein S6 Kinase A4 | Protein Coding | 42 | GC11P064363 | 2.390418768 |
| VANGL2 | VANGL Planar Cell Polarity Protein 2 | Protein Coding | 38 | GC01P160400 | 2.389793634 |
| SYVN1 | Synoviolin 1 | Protein Coding | 36 | GC11M087246 | 2.38953042 |
| CALML3 | Calmodulin Like 3 | Protein Coding | 36 | GC10P005556 | 2.389450073 |
| CUL7 | Cullin 7 | Protein Coding | 37 | GC06M043037 | 2.38941288 |
| ZNF326 | Zinc Finger Protein 326 | Protein Coding | 32 | GC01P089995 | 2.389223099 |
| GINS2 | GINS Complex Subunit 2 | Protein Coding | 33 | GC16M085676 | 2.388649464 |
| ARF4 | ADP Ribosylation Factor 4 | Protein Coding | 37 | GC03M057572 | 2.388468742 |
| CD3D | CD3 Delta Subunit Of T-Cell Receptor Complex | Protein Coding | 42 | GC11M118338 | 2.386170626 |
| SLC35F2 | Solute Carrier Family 35 Member F2 | Protein Coding | 32 | GC11M107790 | 2.38513422 |
| UFSP2 | UFM1 Specific Peptidase 2 | Protein Coding | 35 | GC04M185399 | 2.383722782 |
| KLHL9 | Kelch Like Family Member 9 | Protein Coding | 35 | GC09M021329 | 2.383157015 |
| LCN1 | Lipocalin 1 | Protein Coding | 36 | GC09P135521 | 2.382945538 |
| ZNF146 | Zinc Finger Protein 146 | Protein Coding | 33 | GC19P063477 | 2.382808924 |
| COPS3 | COP9 Signalosome Subunit 3 | Protein Coding | 35 | GC17M017246 | 2.381658554 |
| SIGLEC5 | Sialic Acid Binding Ig Like Lectin 5 | Protein Coding | 35 | GC19M064252 | 2.381531239 |
| KCTD3 | Potassium Channel Tetramerization Domain Containing 3 | Protein Coding | 34 | GC01P215567 | 2.380744219 |
| TBRG1 | Transforming Growth Factor Beta Regulator 1 | Protein Coding | 29 | GC11P124622 | 2.380142212 |
| SGCB | Sarcoglycan Beta | Protein Coding | 35 | GC04M052019 | 2.379640579 |
| MOS | MOS Proto-Oncogene, Serine/Threonine Kinase | Protein Coding | 33 | GC08M056112 | 2.379616261 |
| DMAP1 | DNA Methyltransferase 1 Associated Protein 1 | Protein Coding | 35 | GC01P044214 | 2.379595757 |
| MCCC2 | Methylcrotonyl-CoA Carboxylase Subunit 2 | Protein Coding | 39 | GC05P072682 | 2.378815413 |
| RHBDL1 | Rhomboid Like 1 | Protein Coding | 31 | GC16P010644 | 2.378428936 |
| HLA-DMA | Major Histocompatibility Complex, Class II, DM Alpha | Protein Coding | 36 | GC06M063676 | 2.37799263 |
| PEX2 | Peroxisomal Biogenesis Factor 2 | Protein Coding | 40 | GC08M076980 | 2.377562046 |
| SCAF1 | SR-Related CTD Associated Factor 1 | Protein Coding | 28 | GC19P064078 | 2.376723051 |
| CMM4 | Melanoma, Cutaneous Malignant, 4 | Genetic Locus | 2 | GC01U901101 | 2.376581907 |
| CMM7 | Melanoma, Cutaneous Malignant, Susceptibility To, 7 | Genetic Locus | 1 | GC20U900424 | 2.376581907 |
| SLIT1 | Slit Guidance Ligand 1 | Protein Coding | 39 | GC10M096998 | 2.376111984 |
| SCARB2 | Scavenger Receptor Class B Member 2 | Protein Coding | 40 | GC04M076158 | 2.376083136 |
| IL20 | Interleukin 20 | Protein Coding | 34 | GC01P206866 | 2.375543118 |
| TMOD1 | Tropomodulin 1 | Protein Coding | 34 | GC09P097501 | 2.37471962 |
| TANC1 | Tetratricopeptide Repeat, Ankyrin Repeat And Coiled-Coil Containing 1 | Protein Coding | 30 | GC02P158968 | 2.374649048 |
| CENPB | Centromere Protein B | Protein Coding | 32 | GC20M003783 | 2.373955727 |
| DACT2 | Dishevelled Binding Antagonist Of Beta Catenin 2 | Protein Coding | 31 | GC06M168292 | 2.373940945 |
| COPS2 | COP9 Signalosome Subunit 2 | Protein Coding | 34 | GC15M049106 | 2.373874903 |
| CEACAM8 | CEA Cell Adhesion Molecule 8 | Protein Coding | 35 | GC19M042580 | 2.373868704 |
| EPB41L1 | Erythrocyte Membrane Protein Band 4.1 Like 1 | Protein Coding | 37 | GC20P036064 | 2.373723507 |
| COX4I1 | Cytochrome C Oxidase Subunit 4I1 | Protein Coding | 40 | GC16P085798 | 2.373566628 |
| KNTC1 | Kinetochore Associated 1 | Protein Coding | 31 | GC12P122527 | 2.373345137 |

| | | | | | |
|---|---|---|---|---|---|
| PTGR1 | Prostaglandin Reductase 1 | Protein Coding | 36 | GC09M111549 | 2.373278618 |
| ADAM33 | ADAM Metallopeptidase Domain 33 | Protein Coding | 36 | GC20M003669 | 2.373120546 |
| TWNK | Twinkle MtDNA Helicase | Protein Coding | 32 | GC10P100993 | 2.372903824 |
| TIAL1 | TIA1 Cytotoxic Granule Associated RNA Binding Protein Like 1 | Protein Coding | 36 | GC10M119571 | 2.372788429 |
| CPSF7 | Cleavage And Polyadenylation Specific Factor 7 | Protein Coding | 31 | GC11M061402 | 2.372779131 |
| MAPKAPK3 | MAPK Activated Protein Kinase 3 | Protein Coding | 44 | GC03P050611 | 2.372313023 |
| GABRD | Gamma-Aminobutyric Acid Type A Receptor Subunit Delta | Protein Coding | 42 | GC01P002019 | 2.372169971 |
| KCNH5 | Potassium Voltage-Gated Channel Subfamily H Member 5 | Protein Coding | 40 | GC14M062699 | 2.37165761 |
| LRPPRC | Leucine Rich Pentatricopeptide Repeat Containing | Protein Coding | 38 | GC02M043850 | 2.371093512 |
| KRT86 | Keratin 86 | Protein Coding | 35 | GC12P052249 | 2.371006727 |
| HLA-DRA | Major Histocompatibility Complex, Class II, DR Alpha | Protein Coding | 42 | GC06P032439 | 2.370658398 |
| PKN3 | Protein Kinase N3 | Protein Coding | 36 | GC09P128702 | 2.370643854 |
| SLC6A5 | Solute Carrier Family 6 Member 5 | Protein Coding | 42 | GC11P020599 | 2.37046814 |
| RPA3 | Replication Protein A3 | Protein Coding | 37 | GC07M008081 | 2.370378256 |
| H3-4 | H3.4 Histone | Protein Coding | 31 | GC01M228427 | 2.369826555 |
| IL31 | Interleukin 31 | Protein Coding | 34 | GC12M122174 | 2.368839979 |
| TCERG1L | Transcription Elongation Regulator 1 Like | Protein Coding | 29 | GC10M131092 | 2.367980719 |
| TDP2 | Tyrosyl-DNA Phosphodiesterase 2 | Protein Coding | 38 | GC06M024651 | 2.367527962 |
| GNGT2 | G Protein Subunit Gamma Transducin 2 | Protein Coding | 34 | GC17M049202 | 2.367518902 |
| CAND1 | Cullin Associated And Neddylation Dissociated 1 | Protein Coding | 34 | GC12P067270 | 2.367422581 |
| PDK4 | Pyruvate Dehydrogenase Kinase 4 | Protein Coding | 40 | GC07M095583 | 2.36741972 |
| ADORA2A | Adenosine A2a Receptor | Protein Coding | 43 | GC22P024417 | 2.367136002 |
| HAGH | Hydroxyacylglutathione Hydrolase | Protein Coding | 38 | GC16M001795 | 2.366912603 |
| MED17 | Mediator Complex Subunit 17 | Protein Coding | 35 | GC11P093784 | 2.366786718 |
| PDSS2 | Decaprenyl Diphosphate Synthase Subunit 2 | Protein Coding | 36 | GC06M107152 | 2.366699219 |
| SQLE | Squalene Epoxidase | Protein Coding | 40 | GC08P124998 | 2.366531849 |
| ABHD12 | Abhydrolase Domain Containing 12, Lysophospholipase | Protein Coding | 39 | GC20M025294 | 2.366076231 |
| PTPRZ1 | Protein Tyrosine Phosphatase Receptor Type Z1 | Protein Coding | 39 | GC07P121873 | 2.365779877 |
| CCP110 | Centriolar Coiled-Coil Protein 110 | Protein Coding | 33 | GC16P019536 | 2.365516663 |
| PSMD1 | Proteasome 26S Subunit, Non-ATPase 1 | Protein Coding | 34 | GC02P231056 | 2.365370035 |
| COLGALT2 | Collagen Beta(1-O)Galactosyltransferase 2 | Protein Coding | 32 | GC01M183899 | 2.36510849 |
| ST6GALNAC2 | ST6 N-Acetylgalactosaminide Alpha-2,6-Sialyltransferase 2 | Protein Coding | 34 | GC17M076565 | 2.364986181 |
| MYBBP1A | MYB Binding Protein 1a | Protein Coding | 35 | GC17M004538 | 2.364224195 |
| SLC11A2 | Solute Carrier Family 11 Member 2 | Protein Coding | 43 | GC12M050952 | 2.363960743 |
| TAF7L | TATA-Box Binding Protein Associated Factor 7 Like | Protein Coding | 31 | GC0XM101268 | 2.36368227 |
| ANXA13 | Annexin A13 | Protein Coding | 34 | GC08M123680 | 2.362376928 |
| MIR520B | MicroRNA 520b | RNA Gene | 15 | GC19P064301 | 2.362218618 |
| KNSTRN | Kinetochore Localized Astrin (SPAG5) Binding Protein | Protein Coding | 33 | GC15P040382 | 2.362211466 |
| KCTD17 | Potassium Channel Tetramerization Domain Containing 17 | Protein Coding | 35 | GC22P037051 | 2.362012386 |
| SLC30A7 | Solute Carrier Family 30 Member 7 | Protein Coding | 34 | GC01P100896 | 2.361811161 |
| SHC3 | SHC Adaptor Protein 3 | Protein Coding | 34 | GC09M089005 | 2.361766577 |
| DSCAML1 | DS Cell Adhesion Molecule Like 1 | Protein Coding | 35 | GC11M117427 | 2.361414433 |
| CKMT1B | Creatine Kinase, Mitochondrial 1B | Protein Coding | 32 | GC15P043593 | 2.360429049 |
| PTCSC1 | Papillary Thyroid Carcinoma Susceptibility Candidate 1 | RNA Gene | 15 | GC08P133054 | 2.359681606 |
| LHX3 | LIM Homeobox 3 | Protein Coding | 38 | GC09M136196 | 2.359610081 |
| DPYS | Dihydropyrimidinase | Protein Coding | 40 | GC08M104331 | 2.359524965 |
| LYPD1 | LY6/PLAUR Domain Containing 1 | Protein Coding | 35 | GC02M133869 | 2.359095573 |
| TSPEAR | Thrombospondin Type Laminin G Domain And EAR Repeats | Protein Coding | 31 | GC21M044497 | 2.35907197 |
| POLR1F | RNA Polymerase I Subunit F | Protein Coding | 27 | GC07M019695 | 2.358939171 |
| RCC1 | Regulator Of Chromosome Condensation 1 | Protein Coding | 37 | GC01P028505 | 2.358439445 |
| UBN1 | Ubinuclein 1 | Protein Coding | 33 | GC16P010888 | 2.358156443 |
| AGO1 | Argonaute RISC Component 1 | Protein Coding | 33 | GC01P035869 | 2.356744766 |
| PSMD2 | Proteasome 26S Subunit Ubiquitin Receptor, Non-ATPase 2 | Protein Coding | 38 | GC03P184298 | 2.35564971 |
| GJA4 | Gap Junction Protein Alpha 4 | Protein Coding | 40 | GC01P034792 | 2.355561733 |
| MICALL1 | MICAL Like 1 | Protein Coding | 31 | GC22P037905 | 2.354914665 |
| RYR2 | Ryanodine Receptor 2 | Protein Coding | 43 | GC01P237042 | 2.354682922 |
| SIGMAR1 | Sigma Non-Opioid Intracellular Receptor 1 | Protein Coding | 44 | GC09M034634 | 2.3546772 |
| LUC7L3 | LUC7 Like 3 Pre-MRNA Splicing Factor | Protein Coding | 33 | GC17P050719 | 2.35462141 |
| SEMA4G | Semaphorin 4G | Protein Coding | 36 | GC10P100969 | 2.354487419 |
| TNFAIP8 | TNF Alpha Induced Protein 8 | Protein Coding | 33 | GC05P119268 | 2.354415655 |
| ZNF155 | Zinc Finger Protein 155 | Protein Coding | 28 | GC19P043967 | 2.353048563 |
| CCT8 | Chaperonin Containing TCP1 Subunit 8 | Protein Coding | 35 | GC21M029055 | 2.35214448 |
| CACNA1D | Calcium Voltage-Gated Channel Subunit Alpha1 D | Protein Coding | 43 | GC03P053328 | 2.351146698 |
| BZW1 | Basic Leucine Zipper And W2 Domains 1 | Protein Coding | 31 | GC02P200810 | 2.350798845 |
| PURG | Purine Rich Element Binding Protein G | Protein Coding | 29 | GC08M030995 | 2.350653172 |
| BAG5 | BAG Cochaperone 5 | Protein Coding | 36 | GC14M103556 | 2.349836349 |
| KCNJ6 | Potassium Inwardly Rectifying Channel Subfamily J Member 6 | Protein Coding | 43 | GC21M037607 | 2.349833012 |
| ATP2B1 | ATPase Plasma Membrane Ca2+ Transporting 1 | Protein Coding | 39 | GC12M089588 | 2.349188089 |
| N4BP2 | NEDD4 Binding Protein 2 | Protein Coding | 30 | GC04P040058 | 2.349013329 |
| NDUFA2 | NADH:Ubiquinone Oxidoreductase Subunit A2 | Protein Coding | 38 | GC05M140672 | 2.348759651 |
| ESF1 | ESF1 Nucleolar Pre-RRNA Processing Protein Homolog | Protein Coding | 28 | GC20M013714 | 2.347912788 |
| CDC42BPA | CDC42 Binding Protein Kinase Alpha | Protein Coding | 38 | GC01M226989 | 2.347793579 |
| STX12 | Syntaxin 12 | Protein Coding | 33 | GC01P027783 | 2.346586227 |
| YTHDC2 | YTH Domain Containing 2 | Protein Coding | 32 | GC05P113513 | 2.346368074 |
| BCL7C | BAF Chromatin Remodeling Complex Subunit BCL7C | Protein Coding | 31 | GC16M036563 | 2.346065998 |
| SLC5A6 | Solute Carrier Family 5 Member 6 | Protein Coding | 41 | GC02M027201 | 2.345190048 |
| DHX16 | DEAH-Box Helicase 16 | Protein Coding | 35 | GC06M030653 | 2.345109463 |
| RP2 | RP2 Activator Of ARL3 GTPase | Protein Coding | 37 | GC0XP046836 | 2.345070839 |
| SLC27A4 | Solute Carrier Family 27 Member 4 | Protein Coding | 40 | GC09P128340 | 2.345035315 |
| GNG2 | G Protein Subunit Gamma 2 | Protein Coding | 38 | GC14P051826 | 2.344420195 |
| PHACTR1 | Phosphatase And Actin Regulator 1 | Protein Coding | 34 | GC06P012717 | 2.344323874 |
| RPS21 | Ribosomal Protein S21 | Protein Coding | 34 | GC20P062387 | 2.344208479 |
| TMED1 | Transmembrane P24 Trafficking Protein 1 | Protein Coding | 34 | GC19M010832 | 2.343926907 |
| ASH2L | ASH2 Like, Histone Lysine Methyltransferase Complex Subunit | Protein Coding | 37 | GC08P038104 | 2.342371464 |
| LACC1 | Laccase Domain Containing 1 | Protein Coding | 31 | GC13P043879 | 2.342247009 |
| PDF | Peptide Deformylase, Mitochondrial | Protein Coding | 31 | GC16M069968 | 2.341721773 |
| ZC3H11A | Zinc Finger CCCH-Type Containing 11A | Protein Coding | 32 | GC01P203795 | 2.341704845 |
| SRSF7 | Serine And Arginine Rich Splicing Factor 7 | Protein Coding | 35 | GC02M038743 | 2.340895653 |
| YY2 | YY2 Transcription Factor | Protein Coding | 28 | GC0XP021855 | 2.338703632 |
| RNF149 | Ring Finger Protein 149 | Protein Coding | 31 | GC02M101271 | 2.338138342 |
| NFX1 | Nuclear Transcription Factor, X-Box Binding 1 | Protein Coding | 31 | GC09P033292 | 2.338070393 |
| EIF4EBP2 | Eukaryotic Translation Initiation Factor 4E Binding Protein 2 | Protein Coding | 35 | GC10P070404 | 2.337122679 |
| FAM83B | Family With Sequence Similarity 83 Member B | Protein Coding | 31 | GC06P054846 | 2.33710146 |
| DAPK2 | Death Associated Protein Kinase 2 | Protein Coding | 36 | GC15M063907 | 2.336724281 |
| MAU2 | MAU2 Sister Chromatid Cohesion Factor | Protein Coding | 29 | GC19P019320 | 2.336713314 |

| | | | | | |
|---|---|---|---|---|---|
| H2AW | H2A.W Histone | Protein Coding | 27 | GC01M228544 | 2.336458206 |
| SEMA4B | Semaphorin 4B | Protein Coding | 36 | GC15P090160 | 2.336441517 |
| CLEC3A | C-Type Lectin Domain Family 3 Member A | Protein Coding | 29 | GC16P078022 | 2.33610034 |
| LRRC41 | Leucine Rich Repeat Containing 41 | Protein Coding | 31 | GC01M046261 | 2.336095333 |
| RNF126 | Ring Finger Protein 126 | Protein Coding | 32 | GC19M000647 | 2.335856676 |
| PLOD3 | Procollagen-Lysine,2-Oxoglutarate 5-Dioxygenase 3 | Protein Coding | 42 | GC07M101205 | 2.335695267 |
| UBA2 | Ubiquitin Like Modifier Activating Enzyme 2 | Protein Coding | 39 | GC19P034428 | 2.335675716 |
| LINC01614 | Long Intergenic Non-Protein Coding RNA 1614 | RNA Gene | 11 | GC02P215717 | 2.335623741 |
| RLBP1 | Retinaldehyde Binding Protein 1 | Protein Coding | 40 | GC15M089209 | 2.335535049 |
| NFATC4 | Nuclear Factor Of Activated T Cells 4 | Protein Coding | 39 | GC14P024365 | 2.335429668 |
| SGSM3 | Small G Protein Signaling Modulator 3 | Protein Coding | 34 | GC22P040370 | 2.33490324 |
| KRT83 | Keratin 83 | Protein Coding | 35 | GC12M052314 | 2.334646463 |
| EMP3 | Epithelial Membrane Protein 3 | Protein Coding | 35 | GC19P048321 | 2.334279537 |
| LTBP3 | Latent Transforming Growth Factor Beta Binding Protein 3 | Protein Coding | 39 | GC11M065538 | 2.333823204 |
| RNF7 | Ring Finger Protein 7 | Protein Coding | 35 | GC03P141738 | 2.332647085 |
| DHDDS | Dehydrodolichyl Diphosphate Synthase Subunit | Protein Coding | 38 | GC01P026432 | 2.332357407 |
| NPHP3 | Nephrocystin 3 | Protein Coding | 35 | GC03M132683 | 2.332317829 |
| ZNHIT2 | Zinc Finger HIT-Type Containing 2 | Protein Coding | 28 | GC11M087244 | 2.332122326 |
| TBC1D9B | TBC1 Domain Family Member 9B | Protein Coding | 27 | GC05M179862 | 2.332122326 |
| PSPC1 | Paraspeckle Component 1 | Protein Coding | 31 | GC13M019674 | 2.331566095 |
| C17orf80 | Chromosome 17 Open Reading Frame 80 | Protein Coding | 29 | GC17P073232 | 2.331192017 |
| PTGDR | Prostaglandin D2 Receptor | Protein Coding | 42 | GC14P052267 | 2.33022356 |
| MPDU1 | Mannose-P-Dolichol Utilization Defect 1 | Protein Coding | 38 | GC17P007583 | 2.32980299 |
| ZC3HAV1 | Zinc Finger CCCH-Type Containing, Antiviral 1 | Protein Coding | 35 | GC07M139051 | 2.328874111 |
| TMEM41B | Transmembrane Protein 41B | Protein Coding | 29 | GC11M009280 | 2.3287642 |
| PREP | Prolyl Endopeptidase | Protein Coding | 38 | GC06M105277 | 2.328625441 |
| ZNF471 | Zinc Finger Protein 471 | Protein Coding | 29 | GC19P064407 | 2.328510761 |
| H2AC7 | H2A Clustered Histone 7 | Protein Coding | 25 | GC06M064156 | 2.328419209 |
| FAM83A | Family With Sequence Similarity 83 Member A | Protein Coding | 30 | GC08P123178 | 2.327970743 |
| RNU4ATAC | RNA, U4atac Small Nuclear (U12-Dependent Splicing) | RNA Gene | 19 | GC02P121766 | 2.327049255 |
| SURF2 | Surfeit 2 | Protein Coding | 32 | GC09P134147 | 2.326236248 |
| LIN9 | Lin-9 DREAM MuvB Core Complex Component | Protein Coding | 31 | GC01M226231 | 2.325077772 |
| TNFSF14 | TNF Superfamily Member 14 | Protein Coding | 37 | GC19M006663 | 2.325022697 |
| PDE4A | Phosphodiesterase 4A | Protein Coding | 40 | GC19P010416 | 2.324600697 |
| DHX58 | DExH-Box Helicase 58 | Protein Coding | 34 | GC17M042101 | 2.322952271 |
| HSD17B11 | Hydroxysteroid 17-Beta Dehydrogenase 11 | Protein Coding | 35 | GC04M087336 | 2.322733879 |
| GINS1 | GINS Complex Subunit 1 | Protein Coding | 36 | GC20P025440 | 2.321852922 |
| CAMK4 | Calcium/Calmodulin Dependent Protein Kinase IV | Protein Coding | 40 | GC05P111223 | 2.321760654 |
| ALX3 | ALX Homeobox 3 | Protein Coding | 33 | GC01M110059 | 2.321335077 |
| EXOC4 | Exocyst Complex Component 4 | Protein Coding | 35 | GC07P133253 | 2.321321964 |
| SAP18 | Sin3A Associated Protein 18 | Protein Coding | 34 | GC13P021140 | 2.320897102 |
| NEXN | Nexilin F-Actin Binding Protein | Protein Coding | 35 | GC01P077898 | 2.320853233 |
| GTF2H2 | General Transcription Factor IIH Subunit 2 | Protein Coding | 34 | GC05M071032 | 2.320351601 |
| IL11RA | Interleukin 11 Receptor Subunit Alpha | Protein Coding | 40 | GC09P040088 | 2.31947732 |
| SSBP4 | Single Stranded DNA Binding Protein 4 | Protein Coding | 32 | GC19P018418 | 2.319280148 |
| CLIP2 | CAP-Gly Domain Containing Linker Protein 2 | Protein Coding | 35 | GC07P074289 | 2.319194317 |
| SRSF5 | Serine And Arginine Rich Splicing Factor 5 | Protein Coding | 35 | GC14P069727 | 2.319175959 |
| TMEM59 | Transmembrane Protein 59 | Protein Coding | 35 | GC01M054031 | 2.318764687 |
| SLC29A2 | Solute Carrier Family 29 Member 2 | Protein Coding | 41 | GC11M087331 | 2.31801796 |
| SLC25A37 | Solute Carrier Family 25 Member 37 | Protein Coding | 34 | GC08P023528 | 2.316981792 |
| GPR180 | G Protein-Coupled Receptor 180 | Protein Coding | 32 | GC13P094601 | 2.316538811 |
| DIAPH2 | Diaphanous Related Formin 2 | Protein Coding | 39 | GC0XP096684 | 2.316146851 |
| ZNF283 | Zinc Finger Protein 283 | Protein Coding | 27 | GC19P043827 | 2.315270424 |
| SELENOK | Selenoprotein K | Protein Coding | 26 | GC03M053884 | 2.315067291 |
| SLC25A51 | Solute Carrier Family 25 Member 51 | Protein Coding | 30 | GC09M037878 | 2.314943314 |
| TBXA2R | Thromboxane A2 Receptor | Protein Coding | 44 | GC19M003594 | 2.314676762 |
| TADA3 | Transcriptional Adaptor 3 | Protein Coding | 32 | GC03M009779 | 2.314609528 |
| MTX1 | Metaxin 1 | Protein Coding | 35 | GC01P155208 | 2.314490557 |
| PSMC2 | Proteasome 26S Subunit, ATPase 2 | Protein Coding | 36 | GC07P103328 | 2.314485312 |
| SSX2IP | SSX Family Member 2 Interacting Protein | Protein Coding | 34 | GC01M084643 | 2.314127684 |
| CRYBG2 | Crystallin Beta-Gamma Domain Containing 2 | Protein Coding | 25 | GC01M026321 | 2.314021587 |
| ANGPTL8 | Angiopoietin Like 8 | Protein Coding | 26 | GC19P011238 | 2.313818932 |
| VAMP1 | Vesicle Associated Membrane Protein 1 | Protein Coding | 39 | GC12M006462 | 2.312906981 |
| TEC | Tec Protein Tyrosine Kinase | Protein Coding | 41 | GC04M048140 | 2.312658787 |
| ITPRID2 | ITPR Interacting Domain Containing 2 | Protein Coding | 27 | GC02P181892 | 2.31222105 |
| RCN2 | Reticulocalbin 2 | Protein Coding | 36 | GC15P076931 | 2.311491966 |
| GID8 | GID Complex Subunit 8 Homolog | Protein Coding | 30 | GC20P062938 | 2.3105793 |
| PDE3A | Phosphodiesterase 3A | Protein Coding | 44 | GC12P020789 | 2.310318947 |
| CYP2R1 | Cytochrome P450 Family 2 Subfamily R Member 1 | Protein Coding | 41 | GC11M014877 | 2.310004711 |
| FRMD7 | FERM Domain Containing 7 | Protein Coding | 35 | GC0XM132077 | 2.309726 |
| ADAMTSL4 | ADAMTS Like 4 | Protein Coding | 35 | GC01P150549 | 2.309546471 |
| ARFGAP1 | ADP Ribosylation Factor GTPase Activating Protein 1 | Protein Coding | 36 | GC20P063272 | 2.309427738 |
| ETAA1 | ETAA1 Activator Of ATR Kinase | Protein Coding | 28 | GC02P067397 | 2.30905056 |
| MANEA | Mannosidase Endo-Alpha | Protein Coding | 34 | GC06P095577 | 2.308848381 |
| PIGN | Phosphatidylinositol Glycan Anchor Biosynthesis Class N | Protein Coding | 37 | GC18M061905 | 2.308829308 |
| PUM2 | Pumilio RNA Binding Family Member 2 | Protein Coding | 37 | GC02M020321 | 2.308691978 |
| DLAT | Dihydrolipoamide S-Acetyltransferase | Protein Coding | 41 | GC11P112026 | 2.308274746 |
| TLK2 | Tousled Like Kinase 2 | Protein Coding | 40 | GC17P062458 | 2.307296753 |
| CCR8 | C-C Motif Chemokine Receptor 8 | Protein Coding | 38 | GC03P039840 | 2.306981802 |
| MGST1 | Microsomal Glutathione S-Transferase 1 | Protein Coding | 37 | GC12P016347 | 2.306034803 |
| DYNC1I2 | Dynein Cytoplasmic 1 Intermediate Chain 2 | Protein Coding | 36 | GC02P171687 | 2.305598974 |
| AGFG1 | ArfGAP With FG Repeats 1 | Protein Coding | 36 | GC02P227473 | 2.305378914 |
| ZNF563 | Zinc Finger Protein 563 | Protein Coding | 31 | GC19M012317 | 2.305306196 |
| MIR4695 | MicroRNA 4695 | RNA Gene | 11 | GC01M018883 | 2.305194378 |
| GLDN | Gliomedin | Protein Coding | 36 | GC15P051341 | 2.304776192 |
| RHBDL3 | Rhomboid Like 3 | Protein Coding | 31 | GC17P052654 | 2.30398798 |
| RGS16 | Regulator Of G Protein Signaling 16 | Protein Coding | 36 | GC01M182598 | 2.30394125 |
| DNAAF1 | Dynein Axonemal Assembly Factor 1 | Protein Coding | 33 | GC16P084145 | 2.303272724 |
| SUDS3 | SDS3 Homolog, SIN3A Corepressor Complex Component | Protein Coding | 31 | GC12P118376 | 2.302935123 |
| LYL1 | LYL1 Basic Helix-Loop-Helix Family Member | Protein Coding | 35 | GC19M013549 | 2.300873041 |
| POLM | DNA Polymerase Mu | Protein Coding | 35 | GC07M044417 | 2.300516605 |
| CMA1 | Chymase 1 | Protein Coding | 39 | GC14M024506 | 2.299747944 |
| CAP2 | Cyclase Associated Actin Cytoskeleton Regulatory Protein 2 | Protein Coding | 35 | GC06P017393 | 2.299152613 |
| CRYBG1 | Crystallin Beta-Gamma Domain Containing 1 | Protein Coding | 26 | GC06P106361 | 2.298809767 |
| MOSPD1 | Motile Sperm Domain Containing 1 | Protein Coding | 30 | GC0XM134887 | 2.298808098 |

| | | | | | |
|---|---|---|---|---|---|
| F11 | Coagulation Factor XI | Protein Coding | 42 | GC04P186265 | 2.298505306 |
| MIR431 | MicroRNA 431 | RNA Gene | 20 | GC14P109203 | 2.298148394 |
| GOSR1 | Golgi SNAP Receptor Complex Member 1 | Protein Coding | 35 | GC17P030477 | 2.298024178 |
| LONRF3 | LON Peptidase N-Terminal Domain And Ring Finger 3 | Protein Coding | 30 | GC0XP118974 | 2.29766798 |
| SRCAP | Snf2 Related CREBBP Activator Protein | Protein Coding | 36 | GC16P040270 | 2.296504498 |
| TMEM219 | Transmembrane Protein 219 | Protein Coding | 30 | GC16P040198 | 2.29612565 |
| BYSL | Bystin Like | Protein Coding | 34 | GC06P080580 | 2.296087742 |
| DLX2 | Distal-Less Homeobox 2 | Protein Coding | 35 | GC02M172099 | 2.295086622 |
| MT-TL2 | Mitochondrially Encoded TRNA-Leu (CUN) 2 | RNA Gene | 12 | GCMTP012268 | 2.294161797 |
| CAPRIN1 | Cell Cycle Associated Protein 1 | Protein Coding | 34 | GC11P034051 | 2.293914795 |
| SLK | STE20 Like Kinase | Protein Coding | 40 | GC10P103967 | 2.293715239 |
| PARG | Poly(ADP-Ribose) Glycohydrolase | Protein Coding | 36 | GC10M049818 | 2.292858362 |
| ABCG1 | ATP Binding Cassette Subfamily G Member 1 | Protein Coding | 39 | GC21P042199 | 2.292404413 |
| RPL23 | Ribosomal Protein L23 | Protein Coding | 35 | GC17M038847 | 2.291607857 |
| DUT | Deoxyuridine Triphosphatase | Protein Coding | 38 | GC15P048331 | 2.290938377 |
| DNAAF4 | Dynein Axonemal Assembly Factor 4 | Protein Coding | 30 | GC15M081679 | 2.290881157 |
| ANK3 | Ankyrin 3 | Protein Coding | 39 | GC10M060026 | 2.290846825 |
| TMTC4 | Transmembrane O-Mannosyltransferase Targeting Cadherins 4 | Protein Coding | 31 | GC13M100603 | 2.290779591 |
| NOP53 | NOP53 Ribosome Biogenesis Factor | Protein Coding | 28 | GC19P063955 | 2.28986311 |
| PSMC3 | Proteasome 26S Subunit, ATPase 3 | Protein Coding | 39 | GC11M086885 | 2.289274216 |
| CNTRL | Centriolin | Protein Coding | 33 | GC09P121074 | 2.289160728 |
| SOX8 | SRY-Box Transcription Factor 8 | Protein Coding | 34 | GC16P000981 | 2.288846254 |
| ELP3 | Elongator Acetyltransferase Complex Subunit 3 | Protein Coding | 36 | GC08P028089 | 2.287904739 |
| CILP | Cartilage Intermediate Layer Protein | Protein Coding | 38 | GC15M065194 | 2.287782669 |
| TNS3 | Tensin 3 | Protein Coding | 34 | GC07M047281 | 2.287502289 |
| NPHP4 | Nephrocystin 4 | Protein Coding | 36 | GC01M005863 | 2.287374258 |
| ANKRD30BP2 | Ankyrin Repeat Domain 30B Pseudogene 2 | Pseudogene | 11 | GC21P013038 | 2.287098646 |
| FXYD3 | FXYD Domain Containing Ion Transport Regulator 3 | Protein Coding | 32 | GC19P035115 | 2.286970854 |
| ZNF462 | Zinc Finger Protein 462 | Protein Coding | 36 | GC09P106860 | 2.286902666 |
| FMN1 | Formin 1 | Protein Coding | 34 | GC15M032765 | 2.286585569 |
| ENDOG | Endonuclease G | Protein Coding | 38 | GC09P128818 | 2.286318779 |
| PTGES3 | Prostaglandin E Synthase 3 | Protein Coding | 40 | GC12M056667 | 2.286133289 |
| PSMD4 | Proteasome 26S Subunit Ubiquitin Receptor, Non-ATPase 4 | Protein Coding | 39 | GC01P151256 | 2.28593111 |
| SALL3 | Spalt Like Transcription Factor 3 | Protein Coding | 32 | GC18P078980 | 2.285484791 |
| SNRPA | Small Nuclear Ribonucleoprotein Polypeptide A | Protein Coding | 35 | GC19P040750 | 2.284524679 |
| SLC25A27 | Solute Carrier Family 25 Member 27 | Protein Coding | 34 | GC06P046652 | 2.284331322 |
| RTN4R | Reticulon 4 Receptor | Protein Coding | 39 | GC22M020241 | 2.283747673 |
| MRPL44 | Mitochondrial Ribosomal Protein L44 | Protein Coding | 36 | GC02P223957 | 2.283691168 |
| ATG10 | Autophagy Related 10 | Protein Coding | 34 | GC05P081972 | 2.283238173 |
| HYDIN | HYDIN Axonemal Central Pair Apparatus Protein | Protein Coding | 34 | GC16M070913 | 2.282964945 |
| ADAMTS4 | ADAM Metallopeptidase With Thrombospondin Type 1 Motif 4 | Protein Coding | 39 | GC01M161184 | 2.282347679 |
| ADAM2 | ADAM Metallopeptidase Domain 2 | Protein Coding | 34 | GC08M039743 | 2.281502008 |
| KIAA1217 | KIAA1217 | Protein Coding | 32 | GC10P023695 | 2.281158209 |
| AOC1 | Amine Oxidase Copper Containing 1 | Protein Coding | 35 | GC07P150824 | 2.281044722 |
| PUS7 | Pseudouridine Synthase 7 | Protein Coding | 36 | GC07M105439 | 2.280912399 |
| PCDH20 | Protocadherin 20 | Protein Coding | 30 | GC13M061409 | 2.279907465 |
| SLC25A21 | Solute Carrier Family 25 Member 21 | Protein Coding | 39 | GC14M036677 | 2.279223442 |
| ISYNA1 | Inositol-3-Phosphate Synthase 1 | Protein Coding | 36 | GC19M018406 | 2.279060125 |
| BHLHE41 | Basic Helix-Loop-Helix Family Member E41 | Protein Coding | 36 | GC12M026120 | 2.278736115 |
| TSPAN13 | Tetraspanin 13 | Protein Coding | 32 | GC07P016753 | 2.278032303 |
| DSC1 | Desmocollin 1 | Protein Coding | 35 | GC18M031129 | 2.277840853 |
| SAP130 | Sin3A Associated Protein 130 | Protein Coding | 32 | GC02M128229 | 2.277663469 |
| FUCA2 | Alpha-L-Fucosidase 2 | Protein Coding | 36 | GC06M143494 | 2.277554035 |
| KIF20A | Kinesin Family Member 20A | Protein Coding | 36 | GC05P138189 | 2.277444363 |
| FAM120C | Family With Sequence Similarity 120C | Protein Coding | 31 | GC0XM054069 | 2.277399302 |
| NEPRO | Nucleolus And Neural Progenitor Protein | Protein Coding | 30 | GC03M113009 | 2.27588129 |
| STX5 | Syntaxin 5 | Protein Coding | 35 | GC11M062806 | 2.275601387 |
| TESK1 | Testis Associated Actin Remodelling Kinase 1 | Protein Coding | 38 | GC09P035605 | 2.275596857 |
| CST9 | Cystatin 9 | Protein Coding | 31 | GC20M023651 | 2.275461674 |
| TTF1 | Transcription Termination Factor 1 | Protein Coding | 32 | GC09M132375 | 2.275214911 |
| COL13A1 | Collagen Type XIII Alpha 1 Chain | Protein Coding | 36 | GC10P069801 | 2.275188923 |
| PITPNM3 | PITPNM Family Member 3 | Protein Coding | 35 | GC17M006451 | 2.274686813 |
| HOXD10 | Homeobox D10 | Protein Coding | 38 | GC02P176108 | 2.274684668 |
| RPGRIP1 | RPGR Interacting Protein 1 | Protein Coding | 35 | GC14P031578 | 2.274647713 |
| GABBR1 | Gamma-Aminobutyric Acid Type B Receptor Subunit 1 | Protein Coding | 42 | GC06M029555 | 2.274459362 |
| COASY | Coenzyme A Synthase | Protein Coding | 40 | GC17P042561 | 2.274441719 |
| OGDH | Oxoglutarate Dehydrogenase | Protein Coding | 41 | GC07P044606 | 2.274349213 |
| SIN3B | SIN3 Transcription Regulator Family Member B | Protein Coding | 36 | GC19P016829 | 2.274296284 |
| SGCE | Sarcoglycan Epsilon | Protein Coding | 39 | GC07M094524 | 2.274142742 |
| SCN1B | Sodium Voltage-Gated Channel Beta Subunit 1 | Protein Coding | 41 | GC19P035030 | 2.273622036 |
| CANT1 | Calcium Activated Nucleotidase 1 | Protein Coding | 40 | GC17M078992 | 2.273117065 |
| SOSTDC1 | Sclerostin Domain Containing 1 | Protein Coding | 36 | GC07M016467 | 2.272614479 |
| RBM25 | RNA Binding Motif Protein 25 | Protein Coding | 31 | GC14P073058 | 2.272481203 |
| NCAPD2 | Non-SMC Condensin I Complex Subunit D2 | Protein Coding | 36 | GC12P006493 | 2.27219677 |
| EPB41L2 | Erythrocyte Membrane Protein Band 4.1 Like 2 | Protein Coding | 35 | GC06M130820 | 2.272032499 |
| ACVR2B | Activin A Receptor Type 2B | Protein Coding | 45 | GC03P038453 | 2.271967888 |
| ARL6IP5 | ADP Ribosylation Factor Like GTPase 6 Interacting Protein 5 | Protein Coding | 33 | GC03P069084 | 2.271870136 |
| IL17B | Interleukin 17B | Protein Coding | 35 | GC05M149371 | 2.271808624 |
| TMEM116 | Transmembrane Protein 116 | Protein Coding | 25 | GC12M111894 | 2.270712376 |
| KRT75 | Keratin 75 | Protein Coding | 32 | GC12M052425 | 2.270683527 |
| SLC28A1 | Solute Carrier Family 28 Member 1 | Protein Coding | 39 | GC15P084884 | 2.270233393 |
| GAGE2C | G Antigen 2C | Protein Coding | 16 | GC0XU900879 | 2.270219803 |
| SUCLA2 | Succinate-CoA Ligase ADP-Forming Subunit Beta | Protein Coding | 42 | GC13M047745 | 2.269868374 |
| STAG3L2 | Stromal Antigen 3-Like 2 (Pseudogene) | Pseudogene | 19 | GC07M074843 | 2.268959045 |
| HHIPL2 | HHIP Like 2 | Protein Coding | 32 | GC01M223724 | 2.268614769 |
| LARP4 | La Ribonucleoprotein 4 | Protein Coding | 30 | GC12P050392 | 2.267537117 |
| NDUFA3 | NADH:Ubiquinone Oxidoreductase Subunit A3 | Protein Coding | 34 | GC19P054102 | 2.267254114 |
| WDR45 | WD Repeat Domain 45 | Protein Coding | 36 | GC0XM049074 | 2.266981602 |
| TAAR1 | Trace Amine Associated Receptor 1 | Protein Coding | 35 | GC06M132644 | 2.266653538 |
| RAMP1 | Receptor Activity Modifying Protein 1 | Protein Coding | 39 | GC02P237858 | 2.266559839 |
| AFF2 | AF4/FMR2 Family Member 2 | Protein Coding | 38 | GC0XP148500 | 2.26622963 |
| LINC00310 | Long Intergenic Non-Protein Coding RNA 310 | RNA Gene | 15 | GC21P034157 | 2.26590848 |
| CHRNA4 | Cholinergic Receptor Nicotinic Alpha 4 Subunit | Protein Coding | 43 | GC20M063343 | 2.265805244 |
| NELL1 | Neural EGFL Like 1 | Protein Coding | 35 | GC11P020669 | 2.26576829 |
| SH3PXD2B | SH3 And PX Domains 2B | Protein Coding | 36 | GC05M172325 | 2.265671015 |

| | | | | | |
|---|---|---|---|---|---|
| TRIP4 | Thyroid Hormone Receptor Interactor 4 | Protein Coding | 36 | GC15P114639 | 2.265663385 |
| ATXN8OS | ATXN8 Opposite Strand LncRNA | RNA Gene | 25 | GC13P070107 | 2.265643835 |
| PAFAH1B2 | Platelet Activating Factor Acetylhydrolase 1b Catalytic Subunit 2 | Protein Coding | 38 | GC11P117144 | 2.264965534 |
| MIR4766 | MicroRNA 4766 | RNA Gene | 14 | GC22M040813 | 2.264568806 |
| FAM76B | Family With Sequence Similarity 76 Member B | Protein Coding | 31 | GC11M095768 | 2.264359236 |
| TTPA | Alpha Tocopherol Transfer Protein | Protein Coding | 38 | GC08M063048 | 2.264146328 |
| MPDZ | Multiple PDZ Domain Crumbs Cell Polarity Complex Component | Protein Coding | 40 | GC09M013095 | 2.264001131 |
| PPIF | Peptidylprolyl Isomerase F | Protein Coding | 40 | GC10P091436 | 2.263849497 |
| COL25A1 | Collagen Type XXV Alpha 1 Chain | Protein Coding | 35 | GC04M108810 | 2.263698578 |
| STAM | Signal Transducing Adaptor Molecule | Protein Coding | 36 | GC10P017682 | 2.263255119 |
| SH3GLB1 | SH3 Domain Containing GRB2 Like, Endophilin B1 | Protein Coding | 36 | GC01P086704 | 2.263188839 |
| PIP5K1C | Phosphatidylinositol-4-Phosphate 5-Kinase Type 1 Gamma | Protein Coding | 44 | GC19M005027 | 2.262738705 |
| MIR1225 | MicroRNA 1225 | RNA Gene | 16 | GC16M002090 | 2.262244225 |
| TELO2 | Telomere Maintenance 2 | Protein Coding | 35 | GC16P001493 | 2.261286497 |
| PANK4 | Pantothenate Kinase 4 (Inactive) | Protein Coding | 36 | GC01M002508 | 2.261190891 |
| FUZ | Fuzzy Planar Cell Polarity Protein | Protein Coding | 35 | GC19M049806 | 2.261144876 |
| SLC33A1 | Solute Carrier Family 33 Member 1 | Protein Coding | 38 | GC03M155821 | 2.260728359 |
| PRAF2 | PRA1 Domain Family Member 2 | Protein Coding | 31 | GC0XM049199 | 2.259999514 |
| BCL2L14 | BCL2 Like 14 | Protein Coding | 34 | GC12P012049 | 2.259914398 |
| LARP7 | La Ribonucleoprotein 7, Transcriptional Regulator | Protein Coding | 38 | GC04P112636 | 2.258869886 |
| TREML1 | Triggering Receptor Expressed On Myeloid Cells Like 1 | Protein Coding | 33 | GC06M063804 | 2.258177042 |
| NLRP14 | NLR Family Pyrin Domain Containing 14 | Protein Coding | 32 | GC11P007020 | 2.258177042 |
| DYNLL1 | Dynein Light Chain LC8-Type 1 | Protein Coding | 36 | GC12P120469 | 2.258051395 |
| CD93 | CD93 Molecule | Protein Coding | 36 | GC20M023079 | 2.257761478 |
| CCRL2 | C-C Motif Chemokine Receptor Like 2 | Protein Coding | 37 | GC03P046407 | 2.257222652 |
| ZNF646 | Zinc Finger Protein 646 | Protein Coding | 31 | GC16P040293 | 2.256743908 |
| RPL36AL | Ribosomal Protein L36a Like | Protein Coding | 34 | GC14M049619 | 2.256733179 |
| RAB22A | RAB22A, Member RAS Oncogene Family | Protein Coding | 32 | GC20P058309 | 2.25647974 |
| NSDHL | NAD(P) Dependent Steroid Dehydrogenase-Like | Protein Coding | 39 | GC0XP152830 | 2.255919933 |
| CHORDC1 | Cysteine And Histidine Rich Domain Containing 1 | Protein Coding | 32 | GC11M090200 | 2.254648209 |
| PRRT2 | Proline Rich Transmembrane Protein 2 | Protein Coding | 35 | GC16P029811 | 2.254524708 |
| HSD17B12 | Hydroxysteroid 17-Beta Dehydrogenase 12 | Protein Coding | 36 | GC11P043756 | 2.254334688 |
| ERH | ERH MRNA Splicing And Mitosis Factor | Protein Coding | 32 | GC14M069380 | 2.254228115 |
| FAR1 | Fatty Acyl-CoA Reductase 1 | Protein Coding | 38 | GC11P013668 | 2.254131794 |
| TAS2R64P | Taste 2 Receptor Member 64 Pseudogene | Pseudogene | 8 | GC12M020463 | 2.253406525 |
| MINAR1 | Membrane Integral NOTCH2 Associated Receptor 1 | Protein Coding | 24 | GC15P114935 | 2.252849579 |
| KRT73 | Keratin 73 | Protein Coding | 30 | GC12M052607 | 2.252575159 |
| KCNQ5 | Potassium Voltage-Gated Channel Subfamily Q Member 5 | Protein Coding | 39 | GC06P072621 | 2.252432346 |
| HELZ2 | Helicase With Zinc Finger 2 | Protein Coding | 31 | GC20M063558 | 2.251439095 |
| TMEM45B | Transmembrane Protein 45B | Protein Coding | 28 | GC11P129815 | 2.251439095 |
| PDCD5 | Programmed Cell Death 5 | Protein Coding | 35 | GC19P032581 | 2.251101971 |
| FPR2 | Formyl Peptide Receptor 2 | Protein Coding | 41 | GC19P051752 | 2.250995398 |
| VLDLR-AS1 | VLDLR Antisense RNA 1 | RNA Gene | 14 | GC09M002411 | 2.250488758 |
| MT1X | Metallothionein 1X | Protein Coding | 33 | GC16P057010 | 2.250271797 |
| USP18 | Ubiquitin Specific Peptidase 18 | Protein Coding | 36 | GC22P018149 | 2.249971628 |
| DGKG | Diacylglycerol Kinase Gamma | Protein Coding | 38 | GC03M186105 | 2.249842167 |
| UGT8 | UDP Glycosyltransferase 8 | Protein Coding | 40 | GC04P114598 | 2.249784946 |
| RMND1 | Required For Meiotic Nuclear Division 1 Homolog | Protein Coding | 33 | GC06M151404 | 2.249646664 |
| CDK5RAP3 | CDK5 Regulatory Subunit Associated Protein 3 | Protein Coding | 34 | GC17P047967 | 2.24947381 |
| PROC | Protein C, Inactivator Of Coagulation Factors Va And VIIIa | Protein Coding | 45 | GC02P127418 | 2.249268293 |
| SEC16A | SEC16 Homolog A, Endoplasmic Reticulum Export Factor | Protein Coding | 32 | GC09M136440 | 2.249260426 |
| DNAH17 | Dynein Axonemal Heavy Chain 17 | Protein Coding | 32 | GC17M078423 | 2.248260498 |
| ANKEF1 | Ankyrin Repeat And EF-Hand Domain Containing 1 | Protein Coding | 30 | GC20P009987 | 2.248136997 |
| DAW1 | Dynein Assembly Factor With WD Repeats 1 | Protein Coding | 26 | GC02P227871 | 2.248136997 |
| STPG1 | Sperm Tail PG-Rich Repeat Containing 1 | Protein Coding | 26 | GC01M024356 | 2.248136997 |
| LOC110408762 | CYP17A1 Promoter | Biological Region | 2 | GC10P102837 | 2.247802019 |
| C1orf109 | Chromosome 1 Open Reading Frame 109 | Protein Coding | 28 | GC01M037681 | 2.247414351 |
| TAMM41 | TAM41 Mitochondrial Translocator Assembly And Maintenance Homolog | Protein Coding | 30 | GC03M011721 | 2.247413158 |
| FLACC1 | Flagellum Associated Containing Coiled-Coil Domains 1 | Protein Coding | 27 | GC02M201401 | 2.246304989 |
| HIP1R | Huntingtin Interacting Protein 1 Related | Protein Coding | 38 | GC12P122834 | 2.246186256 |
| ZNF469 | Zinc Finger Protein 469 | Protein Coding | 30 | GC16P088382 | 2.245857239 |
| XRN1 | 5'-3' Exoribonuclease 1 | Protein Coding | 31 | GC03M142306 | 2.245372295 |
| AHDC1 | AT-Hook DNA Binding Motif Containing 1 | Protein Coding | 31 | GC01M027534 | 2.245190382 |
| SYT6 | Synaptotagmin 6 | Protein Coding | 33 | GC01M114089 | 2.244875669 |
| CYP4V2 | Cytochrome P450 Family 4 Subfamily V Member 2 | Protein Coding | 38 | GC04P186191 | 2.244796276 |
| ZNF687 | Zinc Finger Protein 687 | Protein Coding | 33 | GC01P151281 | 2.244644165 |
| TNFRSF21 | TNF Receptor Superfamily Member 21 | Protein Coding | 40 | GC06M047231 | 2.244322538 |
| POMT1 | Protein O-Mannosyltransferase 1 | Protein Coding | 41 | GC09P131502 | 2.244296074 |
| VRTN | Vertebrae Development Associated | Protein Coding | 27 | GC14P074303 | 2.244044542 |
| MIR1228 | MicroRNA 1228 | RNA Gene | 15 | GC12P057194 | 2.243992805 |
| BNC2 | Basonuclin 2 | Protein Coding | 35 | GC09M016410 | 2.243735313 |
| OTUD7A | OTU Deubiquitinase 7A | Protein Coding | 34 | GC15M031475 | 2.243717909 |
| CRB3 | Crumbs Cell Polarity Complex Component 3 | Protein Coding | 31 | GC19P006463 | 2.243535519 |
| MIR33B | MicroRNA 33b | RNA Gene | 18 | GC17M017813 | 2.243492842 |
| PSMD3 | Proteasome 26S Subunit, Non-ATPase 3 | Protein Coding | 38 | GC17P039980 | 2.243321419 |
| PLPP3 | Phospholipid Phosphatase 3 | Protein Coding | 35 | GC01M056495 | 2.242842674 |
| PSMC1 | Proteasome 26S Subunit, ATPase 1 | Protein Coding | 36 | GC14P090256 | 2.242841244 |
| PDZD7 | PDZ Domain Containing 7 | Protein Coding | 34 | GC10M101007 | 2.24283886 |
| OXSM | 3-Oxoacyl-ACP Synthase, Mitochondrial | Protein Coding | 31 | GC03P025782 | 2.242545128 |
| H2AC8 | H2A Clustered Histone 8 | Protein Coding | 25 | GC06P081034 | 2.242249966 |
| DCLRE1A | DNA Cross-Link Repair 1A | Protein Coding | 32 | GC10M113834 | 2.241353035 |
| DIO3 | Iodothyronine Deiodinase 3 | Protein Coding | 36 | GC14P109322 | 2.241053343 |
| CREB3L2 | CAMP Responsive Element Binding Protein 3 Like 2 | Protein Coding | 35 | GC07M137874 | 2.24077034 |
| CYSLTR1 | Cysteinyl Leukotriene Receptor 1 | Protein Coding | 40 | GC0XM078271 | 2.24057579 |
| SNU13 | Small Nuclear Ribonucleoprotein 13 | Protein Coding | 31 | GC22M056117 | 2.240389585 |
| GPR45 | G Protein-Coupled Receptor 45 | Protein Coding | 35 | GC02P105241 | 2.240058899 |
| KIF24 | Kinesin Family Member 24 | Protein Coding | 32 | GC09M034252 | 2.239978075 |
| NUP210 | Nucleoporin 210 | Protein Coding | 36 | GC03M020199 | 2.239804745 |
| MIR1290 | MicroRNA 1290 | RNA Gene | 13 | GC01M018897 | 2.239789963 |
| CLCNKB | Chloride Voltage-Gated Channel Kb | Protein Coding | 39 | GC01P016043 | 2.239690781 |
| SERPINF2 | Serpin Family F Member 2 | Protein Coding | 40 | GC17P001742 | 2.239574194 |
| LMF2 | Lipase Maturation Factor 2 | Protein Coding | 29 | GC22M050502 | 2.239325762 |
| GRK5 | G Protein-Coupled Receptor Kinase 5 | Protein Coding | 38 | GC10P119207 | 2.238267183 |
| NELL2 | Neural EGFL Like 2 | Protein Coding | 36 | GC12M044509 | 2.237812996 |
| STXBP1 | Syntaxin Binding Protein 1 | Protein Coding | 44 | GC09P127582 | 2.237658262 |

| | | | | | |
|---|---|---|---|---|---|
| VPS4A | Vacuolar Protein Sorting 4 Homolog A | Protein Coding | 38 | GC16P069311 | 2.237514973 |
| DHPS | Deoxyhypusine Synthase | Protein Coding | 38 | GC19M013514 | 2.237484932 |
| TRIM39 | Tripartite Motif Containing 39 | Protein Coding | 34 | GC06P080321 | 2.237425804 |
| PPP1R7 | Protein Phosphatase 1 Regulatory Subunit 7 | Protein Coding | 33 | GC02P241150 | 2.237074852 |
| SNCB | Synuclein Beta | Protein Coding | 39 | GC05M176620 | 2.236983299 |
| RTP1 | Receptor Transporter Protein 1 | Protein Coding | 30 | GC03P187197 | 2.236740828 |
| ZNF546 | Zinc Finger Protein 546 | Protein Coding | 29 | GC19P039984 | 2.236740828 |
| OTOGL | Otogelin Like | Protein Coding | 28 | GC12P080099 | 2.236740828 |
| NEU2 | Neuraminidase 2 | Protein Coding | 36 | GC02P233032 | 2.236448288 |
| ZBTB12 | Zinc Finger And BTB Domain Containing 12 | Protein Coding | 30 | GC06M031899 | 2.236302137 |
| FUBP3 | Far Upstream Element Binding Protein 3 | Protein Coding | 33 | GC09P130580 | 2.23625493 |
| NPY5R | Neuropeptide Y Receptor Y5 | Protein Coding | 38 | GC04P163343 | 2.236199379 |
| TRAPPC9 | Trafficking Protein Particle Complex Subunit 9 | Protein Coding | 35 | GC08M139728 | 2.235473156 |
| ZBTB7B | Zinc Finger And BTB Domain Containing 7B | Protein Coding | 35 | GC01P155002 | 2.234357834 |
| MYLK2 | Myosin Light Chain Kinase 2 | Protein Coding | 41 | GC20P031819 | 2.233519554 |
| SEC31A | SEC31 Homolog A, COPII Coat Complex Component | Protein Coding | 38 | GC04M082818 | 2.233327866 |
| SLC19A2 | Solute Carrier Family 19 Member 2 | Protein Coding | 40 | GC01M169463 | 2.233229637 |
| TRIM16 | Tripartite Motif Containing 16 | Protein Coding | 30 | GC17M015627 | 2.233015299 |
| SNRPD1 | Small Nuclear Ribonucleoprotein D1 Polypeptide | Protein Coding | 34 | GC18P021612 | 2.233003616 |
| RPH3AL | Rabphilin 3A Like (Without C2 Domains) | Protein Coding | 34 | GC17M000212 | 2.232770681 |
| RUSC1-AS1 | RUSC1 Antisense RNA 1 | RNA Gene | 21 | GC01M155317 | 2.232741356 |
| PTBP3 | Polypyrimidine Tract Binding Protein 3 | Protein Coding | 31 | GC09M112217 | 2.231850624 |
| MED4 | Mediator Complex Subunit 4 | Protein Coding | 32 | GC13M048053 | 2.231058121 |
| OGA | O-GlcNAcase | Protein Coding | 32 | GC10M101785 | 2.230757236 |
| LSINCT5 | Long Stress-Induced Non-Coding Transcript 5 | RNA Gene | 10 | GC05P002712 | 2.230031252 |
| RRS1 | Ribosome Biogenesis Regulator 1 Homolog | Protein Coding | 31 | GC08P066429 | 2.229936838 |
| CNN1 | Calponin 1 | Protein Coding | 36 | GC19P011539 | 2.229578257 |
| AP3B1 | Adaptor Related Protein Complex 3 Subunit Beta 1 | Protein Coding | 40 | GC05M078000 | 2.22944212 |
| GTF3C2 | General Transcription Factor IIIC Subunit 2 | Protein Coding | 34 | GC02M027325 | 2.228590012 |
| H2AC19 | H2A Clustered Histone 19 | Protein Coding | 19 | GC01P150138 | 2.228150368 |
| PRRT1 | Proline Rich Transmembrane Protein 1 | Protein Coding | 29 | GC06M063635 | 2.226428509 |
| KDM7A | Lysine Demethylase 7A | Protein Coding | 32 | GC07M140084 | 2.226349354 |
| MTCP1 | Mature T Cell Proliferation 1 | Protein Coding | 29 | GC0XM155065 | 2.226024151 |
| LRWD1 | Leucine Rich Repeats And WD Repeat Domain Containing 1 | Protein Coding | 32 | GC07P102464 | 2.225741148 |
| L3MBTL3 | L3MBTL Histone Methyl-Lysine Binding Protein 3 | Protein Coding | 32 | GC06P130013 | 2.225510836 |
| ROMO1 | Reactive Oxygen Species Modulator 1 | Protein Coding | 30 | GC20P035699 | 2.225418329 |
| TCEAL1 | Transcription Elongation Factor A Like 1 | Protein Coding | 33 | GC0XP103628 | 2.225152254 |
| NTSR2 | Neurotensin Receptor 2 | Protein Coding | 39 | GC02M011649 | 2.224186659 |
| SALL1 | Spalt Like Transcription Factor 1 | Protein Coding | 41 | GC16M051135 | 2.224098921 |
| ATG14 | Autophagy Related 14 | Protein Coding | 31 | GC14M055366 | 2.223527431 |
| ABCG5 | ATP Binding Cassette Subfamily G Member 5 | Protein Coding | 41 | GC02M043806 | 2.223486423 |
| SVIL | Supervillin | Protein Coding | 37 | GC10M032713 | 2.222991467 |
| CYP7B1 | Cytochrome P450 Family 7 Subfamily B Member 1 | Protein Coding | 41 | GC08M064587 | 2.222899199 |
| EN2 | Engrailed Homeobox 2 | Protein Coding | 36 | GC07P155459 | 2.222606659 |
| SUPT5H | SPT5 Homolog, DSIF Elongation Factor Subunit | Protein Coding | 35 | GC19P039436 | 2.221259594 |
| PLAAT1 | Phospholipase A And Acyltransferase 1 | Protein Coding | 26 | GC03P193244 | 2.221211195 |
| TMSB15A | Thymosin Beta 15A | Protein Coding | 28 | GC0XM102515 | 2.221162796 |
| MYO5B | Myosin VB | Protein Coding | 40 | GC18M049822 | 2.221072674 |
| LYZ | Lysozyme | Protein Coding | 42 | GC12P069348 | 2.220145941 |
| MT1A | Metallothionein 1A | Protein Coding | 33 | GC16P056638 | 2.219722748 |
| RPL32 | Ribosomal Protein L32 | Protein Coding | 34 | GC03M012834 | 2.219545841 |
| MIR630 | MicroRNA 630 | RNA Gene | 12 | GC15P072587 | 2.219530106 |
| PRPF19 | Pre-MRNA Processing Factor 19 | Protein Coding | 32 | GC11M060890 | 2.219449043 |
| DLX1 | Distal-Less Homeobox 1 | Protein Coding | 34 | GC02P172084 | 2.218428135 |
| RCCD1 | RCC1 Domain Containing 1 | Protein Coding | 29 | GC15P090954 | 2.218386173 |
| CTDP1 | CTD Phosphatase Subunit 1 | Protein Coding | 38 | GC18P079679 | 2.218287468 |
| LUADT1 | Lung Adenocarcinoma Associated Transcript 1 | RNA Gene | 13 | GC06P147159 | 2.218092442 |
| PSMG2 | Proteasome Assembly Chaperone 2 | Protein Coding | 33 | GC18P017101 | 2.217806101 |
| TAS2R13 | Taste 2 Receptor Member 13 | Protein Coding | 28 | GC12M010907 | 2.217351437 |
| IRF2BPL | Interferon Regulatory Factor 2 Binding Protein Like | Protein Coding | 32 | GC14M077024 | 2.217155695 |
| PPIL2 | Peptidylprolyl Isomerase Like 2 | Protein Coding | 35 | GC22P035682 | 2.217152119 |
| ALDH7A1P4 | Aldehyde Dehydrogenase 7 Family Member A1 Pseudogene 4 | Pseudogene | 7 | GC10M062740 | 2.216010571 |
| DIDO1 | Death Inducer-Obliterator 1 | Protein Coding | 34 | GC20M062877 | 2.215307713 |
| POLR1G | RNA Polymerase I Subunit G | Protein Coding | 28 | GC19P063860 | 2.215059757 |
| MYL3 | Myosin Light Chain 3 | Protein Coding | 40 | GC03M046836 | 2.214707851 |
| PSMD11 | Proteasome 26S Subunit, Non-ATPase 11 | Protein Coding | 36 | GC17P032444 | 2.214661598 |
| DRD5 | Dopamine Receptor D5 | Protein Coding | 44 | GC04P009783 | 2.214530706 |
| GUF1 | GTP Binding Elongation Factor GUF1 | Protein Coding | 34 | GC04P044680 | 2.21446991 |
| FOXN3 | Forkhead Box N3 | Protein Coding | 34 | GC14M100406 | 2.214359283 |
| SH3TC1 | SH3 Domain And Tetratricopeptide Repeats 1 | Protein Coding | 29 | GC04P008183 | 2.214289904 |
| KRT10-AS1 | KRT10 Antisense RNA 1 | RNA Gene | 20 | GC17P053084 | 2.213508368 |
| RAB2A | RAB2A, Member RAS Oncogene Family | Protein Coding | 38 | GC08P060516 | 2.213085651 |
| GCC2 | GRIP And Coiled-Coil Domain Containing 2 | Protein Coding | 33 | GC02P108432 | 2.212426186 |
| CDK14 | Cyclin Dependent Kinase 14 | Protein Coding | 36 | GC07P090471 | 2.211457968 |
| RN7SL1 | RNA Component Of Signal Recognition Particle 7SL1 | RNA Gene | 17 | GC14P049747 | 2.21126771 |
| HOXB5 | Homeobox B5 | Protein Coding | 35 | GC17M048591 | 2.210464478 |
| PAPSS1 | 3'-Phosphoadenosine 5'-Phosphosulfate Synthase 1 | Protein Coding | 37 | GC04M107590 | 2.210326195 |
| RGS1 | Regulator Of G Protein Signaling 1 | Protein Coding | 36 | GC01P192575 | 2.209729195 |
| VGLL4 | Vestigial Like Family Member 4 | Protein Coding | 32 | GC03M011744 | 2.209513664 |
| GSTM5 | Glutathione S-Transferase Mu 5 | Protein Coding | 36 | GC01P109711 | 2.209309101 |
| MIR1208 | MicroRNA 1208 | RNA Gene | 14 | GC08P128150 | 2.209067583 |
| DOCK2 | Dedicator Of Cytokinesis 2 | Protein Coding | 40 | GC05P169637 | 2.208770514 |
| L2HGDH | L-2-Hydroxyglutarate Dehydrogenase | Protein Coding | 36 | GC14M050237 | 2.20866251 |
| TAT | Tyrosine Aminotransferase | Protein Coding | 40 | GC16M071565 | 2.208447218 |
| OTUD7B | OTU Deubiquitinase 7B | Protein Coding | 32 | GC01M149937 | 2.208125114 |
| NUP188 | Nucleoporin 188 | Protein Coding | 32 | GC09P128947 | 2.208037376 |
| GNG11 | G Protein Subunit Gamma 11 | Protein Coding | 32 | GC07P093921 | 2.207686424 |
| PIP4K2A | Phosphatidylinositol-5-Phosphate 4-Kinase Type 2 Alpha | Protein Coding | 40 | GC10M022484 | 2.207558632 |
| PGAM5 | PGAM Family Member 5, Mitochondrial Serine/Threonine Protein Phosphatase | Protein Coding | 31 | GC12P132710 | 2.207075596 |
| EPX | Eosinophil Peroxidase | Protein Coding | 40 | GC17P058192 | 2.206205368 |
| CACNA1S | Calcium Voltage-Gated Channel Subunit Alpha1 S | Protein Coding | 44 | GC01M201008 | 2.205893517 |
| ADAMTS9 | ADAM Metallopeptidase With Thrombospondin Type 1 Motif 9 | Protein Coding | 35 | GC03M064501 | 2.205377102 |
| LINC01969 | Long Intergenic Non-Protein Coding RNA 1969 | RNA Gene | 11 | GC17P053540 | 2.205163479 |
| DMRTA1 | DMRT Like Family A1 | Protein Coding | 32 | GC09P022436 | 2.204215288 |
| FYTTD1 | Forty-Two-Three Domain Containing 1 | Protein Coding | 31 | GC03P197737 | 2.203595161 |

| | | | | | |
|---|---|---|---|---|---|
| SLC2A10 | Solute Carrier Family 2 Member 10 | Protein Coding | 39 | GC20P046709 | 2.202530384 |
| GGNBP2 | Gametogenetin Binding Protein 2 | Protein Coding | 27 | GC17P036544 | 2.201383114 |
| HNRNPAB | Heterogeneous Nuclear Ribonucleoprotein A/B | Protein Coding | 34 | GC05P178204 | 2.201176167 |
| GTF2B | General Transcription Factor IIB | Protein Coding | 36 | GC01M088853 | 2.19937396 |
| RGMA | Repulsive Guidance Molecule BMP Co-Receptor A | Protein Coding | 38 | GC15M093035 | 2.199163914 |
| MIR638 | MicroRNA 638 | RNA Gene | 17 | GC19P010719 | 2.198773861 |
| DHX35 | DEAH-Box Helicase 35 | Protein Coding | 33 | GC20P038963 | 2.198395252 |
| IARS1 | Isoleucyl-TRNA Synthetase 1 | Protein Coding | 34 | GC09M092718 | 2.198187113 |
| TMEM50A | Transmembrane Protein 50A | Protein Coding | 29 | GC01P025337 | 2.197619438 |
| AGPS | Alkylglycerone Phosphate Synthase | Protein Coding | 38 | GC02P177392 | 2.197285652 |
| MRPS9 | Mitochondrial Ribosomal Protein S9 | Protein Coding | 34 | GC02P105020 | 2.197226048 |
| CEP131 | Centrosomal Protein 131 | Protein Coding | 30 | GC17M081189 | 2.196256638 |
| DDX60 | DExD/H-Box Helicase 60 | Protein Coding | 31 | GC04M168216 | 2.195937634 |
| MEAF6 | MYST/Esa1 Associated Factor 6 | Protein Coding | 31 | GC01M037489 | 2.195723295 |
| GDF6 | Growth Differentiation Factor 6 | Protein Coding | 39 | GC08M096142 | 2.195643425 |
| ZNF624 | Zinc Finger Protein 624 | Protein Coding | 29 | GC17M016620 | 2.194782019 |
| DDX50 | DExD-Box Helicase 50 | Protein Coding | 34 | GC10P068901 | 2.194544792 |
| CREB5 | CAMP Responsive Element Binding Protein 5 | Protein Coding | 35 | GC07P028305 | 2.19451189 |
| LAP3 | Leucine Aminopeptidase 3 | Protein Coding | 38 | GC04P017781 | 2.194080591 |
| SNX8 | Sorting Nexin 8 | Protein Coding | 31 | GC07M002251 | 2.193636417 |
| BBS7 | Bardet-Biedl Syndrome 7 | Protein Coding | 34 | GC04M121824 | 2.192579031 |
| NARS1 | Asparaginyl-TRNA Synthetase 1 | Protein Coding | 34 | GC18M057601 | 2.192471981 |
| KIFBP | Kinesin Family Binding Protein | Protein Coding | 30 | GC10P068990 | 2.192234516 |
| TSTD1 | Thiosulfate Sulfurtransferase Like Domain Containing 1 | Protein Coding | 28 | GC01M161037 | 2.19200182 |
| CAPNS1 | Calpain Small Subunit 1 | Protein Coding | 36 | GC19P063476 | 2.191766739 |
| SUPT4H1 | SPT4 Homolog, DSIF Elongation Factor Subunit | Protein Coding | 35 | GC17M058345 | 2.191736698 |
| SLC1A7 | Solute Carrier Family 1 Member 7 | Protein Coding | 38 | GC01M053087 | 2.190882683 |
| ACTL8 | Actin Like 8 | Protein Coding | 29 | GC01P017772 | 2.190479279 |
| LINC01513 | Long Intergenic Non-Protein Coding RNA 1513 | RNA Gene | 10 | GC05P010480 | 2.190317154 |
| CNGA3 | Cyclic Nucleotide Gated Channel Subunit Alpha 3 | Protein Coding | 38 | GC02P098329 | 2.189586163 |
| SLC6A8 | Solute Carrier Family 6 Member 8 | Protein Coding | 42 | GC0XP153688 | 2.189357281 |
| LXN | Latexin | Protein Coding | 32 | GC03M158645 | 2.189221859 |
| MFSD1 | Major Facilitator Superfamily Domain Containing 1 | Protein Coding | 32 | GC03P158733 | 2.188990355 |
| BAMBI | BMP And Activin Membrane Bound Inhibitor | Protein Coding | 38 | GC10P028685 | 2.188974619 |
| MIR548B | MicroRNA 548b | RNA Gene | 15 | GC06M119069 | 2.188524485 |
| CCNB1IP1 | Cyclin B1 Interacting Protein 1 | Protein Coding | 32 | GC14M020311 | 2.188163757 |
| FTSJ3 | FtsJ RNA 2'-O-Methyltransferase 3 | Protein Coding | 32 | GC17M063819 | 2.18778038 |
| SEMA7A | Semaphorin 7A (John Milton Hagen Blood Group) | Protein Coding | 38 | GC15M074409 | 2.187132835 |
| SLC18A2 | Solute Carrier Family 18 Member A2 | Protein Coding | 42 | GC10P117241 | 2.187131405 |
| MEF2C | Myocyte Enhancer Factor 2C | Protein Coding | 43 | GC05M088718 | 2.186949015 |
| OSBP | Oxysterol Binding Protein | Protein Coding | 36 | GC11M086987 | 2.185647249 |
| KCNA4 | Potassium Voltage-Gated Channel Subfamily A Member 4 | Protein Coding | 40 | GC11M030009 | 2.185504436 |
| POP4 | POP4 Homolog, Ribonuclease P/MRP Subunit | Protein Coding | 34 | GC19P063349 | 2.184854746 |
| NCKAP1 | NCK Associated Protein 1 | Protein Coding | 35 | GC02M182909 | 2.184822559 |
| PAQR3 | Progestin And AdipoQ Receptor Family Member 3 | Protein Coding | 31 | GC04M078887 | 2.184401035 |
| COLEC11 | Collectin Subfamily Member 11 | Protein Coding | 38 | GC02P003594 | 2.184062958 |
| TNPO1 | Transportin 1 | Protein Coding | 36 | GC05P072816 | 2.182145596 |
| TRMU | TRNA Mitochondrial 2-Thiouridylase | Protein Coding | 35 | GC22P046330 | 2.181565285 |
| GATAD2B | GATA Zinc Finger Domain Containing 2B | Protein Coding | 39 | GC01M153805 | 2.181023359 |
| IFI6 | Interferon Alpha Inducible Protein 6 | Protein Coding | 33 | GC01M027666 | 2.181001663 |
| CEP70 | Centrosomal Protein 70 | Protein Coding | 33 | GC03M138494 | 2.180937767 |
| GSTM4 | Glutathione S-Transferase Mu 4 | Protein Coding | 35 | GC01P109657 | 2.18082118 |
| COQ8B | Coenzyme Q8B | Protein Coding | 32 | GC19M063800 | 2.179980278 |
| GABPA | GA Binding Protein Transcription Factor Subunit Alpha | Protein Coding | 36 | GC21P025734 | 2.179505825 |
| DDIT4 | DNA Damage Inducible Transcript 4 | Protein Coding | 38 | GC10P072273 | 2.179496288 |
| OR12D3 | Olfactory Receptor Family 12 Subfamily D Member 3 | Protein Coding | 28 | GC06M029373 | 2.179109812 |
| ZBTB17 | Zinc Finger And BTB Domain Containing 17 | Protein Coding | 36 | GC01M015943 | 2.178761959 |
| OTX1 | Orthodenticle Homeobox 1 | Protein Coding | 37 | GC02P063050 | 2.178283691 |
| MCM10 | Minichromosome Maintenance 10 Replication Initiation Factor | Protein Coding | 34 | GC10P013161 | 2.17814064 |
| ARPP19 | CAMP Regulated Phosphoprotein 19 | Protein Coding | 34 | GC15M052547 | 2.177842617 |
| GON4L | Gon-4 Like | Protein Coding | 31 | GC01M155754 | 2.177363873 |
| BMP3 | Bone Morphogenetic Protein 3 | Protein Coding | 38 | GC04P081030 | 2.177228451 |
| OSGIN1 | Oxidative Stress Induced Growth Inhibitor 1 | Protein Coding | 32 | GC16P083934 | 2.176848888 |
| QDPR | Quinoid Dihydropteridine Reductase | Protein Coding | 42 | GC04M017460 | 2.176223516 |
| ULBP2 | UL16 Binding Protein 2 | Protein Coding | 35 | GC06P149941 | 2.175843239 |
| LIME1 | Lck Interacting Transmembrane Adaptor 1 | Protein Coding | 30 | GC20P063736 | 2.175816298 |
| CBY1 | Chibby Family Member 1, Beta Catenin Antagonist | Protein Coding | 36 | GC22P038656 | 2.175737381 |
| DOCK7 | Dedicator Of Cytokinesis 7 | Protein Coding | 38 | GC01M062454 | 2.175581455 |
| PANX2 | Pannexin 2 | Protein Coding | 35 | GC22P050170 | 2.175238371 |
| COIL | Coilin | Protein Coding | 36 | GC17M056938 | 2.174838066 |
| EXOSC10 | Exosome Component 10 | Protein Coding | 33 | GC01M011067 | 2.174779177 |
| EXOSC9 | Exosome Component 9 | Protein Coding | 35 | GC04P121801 | 2.174626589 |
| CHD6 | Chromodomain Helicase DNA Binding Protein 6 | Protein Coding | 34 | GC20M041402 | 2.174545765 |
| MORF4L1 | Mortality Factor 4 Like 1 | Protein Coding | 36 | GC15P078810 | 2.174320221 |
| GAS2 | Growth Arrest Specific 2 | Protein Coding | 35 | GC11P022626 | 2.173381805 |
| CXCL17 | C-X-C Motif Chemokine Ligand 17 | Protein Coding | 28 | GC19M042428 | 2.172835827 |
| EFR3A | EFR3 Homolog A | Protein Coding | 31 | GC08P131904 | 2.172468424 |
| TRIB1 | Tribbles Pseudokinase 1 | Protein Coding | 35 | GC08P125430 | 2.172402859 |
| TRIP6 | Thyroid Hormone Receptor Interactor 6 | Protein Coding | 34 | GC07P100867 | 2.171638489 |
| ZNF295-AS1 | ZNF295 Antisense RNA 1 | RNA Gene | 19 | GC21P042009 | 2.171272755 |
| PCNX3 | Pecanex 3 | Protein Coding | 25 | GC11P069551 | 2.170826435 |
| DNAJB7 | DnaJ Heat Shock Protein Family (Hsp40) Member B7 | Protein Coding | 28 | GC22M040859 | 2.169329166 |
| RCAN1 | Regulator Of Calcineurin 1 | Protein Coding | 38 | GC21M034513 | 2.168015003 |
| KLF16 | Kruppel Like Factor 16 | Protein Coding | 31 | GC19M001852 | 2.167861462 |
| PCYT1A | Phosphate Cytidylyltransferase 1A, Choline | Protein Coding | 43 | GC03M196214 | 2.167533636 |
| HIVEP3 | HIVEP Zinc Finger 3 | Protein Coding | 32 | GC01M041506 | 2.167238474 |
| TRPM3 | Transient Receptor Potential Cation Channel Subfamily M Member 3 | Protein Coding | 39 | GC09M070529 | 2.166378021 |
| AUNIP | Aurora Kinase A And Ninein Interacting Protein | Protein Coding | 26 | GC01M025862 | 2.164550304 |
| AHCYL2 | Adenosylhomocysteinase Like 2 | Protein Coding | 35 | GC07P129225 | 2.164522886 |
| PDIA6 | Protein Disulfide Isomerase Family A Member 6 | Protein Coding | 35 | GC02M010784 | 2.164288282 |
| HACD3 | 3-Hydroxyacyl-CoA Dehydratase 3 | Protein Coding | 31 | GC15P065530 | 2.164179802 |
| MAP3K9 | Mitogen-Activated Protein Kinase Kinase Kinase 9 | Protein Coding | 39 | GC14M070722 | 2.164165497 |
| SF3B3 | Splicing Factor 3b Subunit 3 | Protein Coding | 34 | GC16P070523 | 2.164132595 |
| CRHR2 | Corticotropin Releasing Hormone Receptor 2 | Protein Coding | 40 | GC07M030651 | 2.163242579 |
| NDUFV2 | NADH:Ubiquinone Oxidoreductase Core Subunit V2 | Protein Coding | 39 | GC18P009092 | 2.163044691 |

| | | | | | |
|---|---|---|---|---|---|
| IRX3 | Iroquois Homeobox 3 | Protein Coding | 35 | GC16M054283 | 2.162928343 |
| ANKLE2 | Ankyrin Repeat And LEM Domain Containing 2 | Protein Coding | 35 | GC12M132725 | 2.162725925 |
| ELOVL5 | ELOVL Fatty Acid Elongase 5 | Protein Coding | 38 | GC06M053267 | 2.161649942 |
| MAVS | Mitochondrial Antiviral Signaling Protein | Protein Coding | 34 | GC20P004009 | 2.161114216 |
| DDO | D-Aspartate Oxidase | Protein Coding | 35 | GC06M110391 | 2.161039352 |
| GIMAP1 | GTPase, IMAP Family Member 1 | Protein Coding | 30 | GC07P150717 | 2.161039352 |
| GCSH | Glycine Cleavage System Protein H | Protein Coding | 39 | GC16M081081 | 2.160916328 |
| MAP3K10 | Mitogen-Activated Protein Kinase Kinase Kinase 10 | Protein Coding | 38 | GC19P040191 | 2.160425663 |
| PPP2R5A | Protein Phosphatase 2 Regulatory Subunit B'Alpha | Protein Coding | 35 | GC01P212285 | 2.160214901 |
| NSUN5 | NOP2/Sun RNA Methyltransferase 5 | Protein Coding | 34 | GC07M073302 | 2.159620285 |
| CTDSPL | CTD Small Phosphatase Like | Protein Coding | 34 | GC03P037861 | 2.159546614 |
| ATP8B2 | ATPase Phospholipid Transporting 8B2 | Protein Coding | 35 | GC01P154325 | 2.159260511 |
| USP5 | Ubiquitin Specific Peptidase 5 | Protein Coding | 40 | GC12P019839 | 2.158922195 |
| INTU | Inturned Planar Cell Polarity Protein | Protein Coding | 32 | GC04P127623 | 2.158593178 |
| MIR660 | MicroRNA 660 | RNA Gene | 14 | GC0XP050013 | 2.158395529 |
| DARS1 | Aspartyl-TRNA Synthetase 1 | Protein Coding | 35 | GC02M135905 | 2.157640934 |
| LTBR | Lymphotoxin Beta Receptor | Protein Coding | 37 | GC12P006375 | 2.157607555 |
| ADAL | Adenosine Deaminase Like | Protein Coding | 30 | GC15P043330 | 2.157375813 |
| SLC25A46 | Solute Carrier Family 25 Member 46 | Protein Coding | 34 | GC05P110738 | 2.157015085 |
| GNL2 | G Protein Nucleolar 2 | Protein Coding | 31 | GC01M037566 | 2.156917334 |
| NDUFA1 | NADH:Ubiquinone Oxidoreductase Subunit A1 | Protein Coding | 40 | GC0XP119871 | 2.156742334 |
| SCAMP3 | Secretory Carrier Membrane Protein 3 | Protein Coding | 34 | GC01M155255 | 2.156725645 |
| ALDH7A1 | Aldehyde Dehydrogenase 7 Family Member A1 | Protein Coding | 43 | GC05M126541 | 2.155997276 |
| CAVIN2 | Caveolae Associated Protein 2 | Protein Coding | 29 | GC02M191835 | 2.155516148 |
| NCAPD3 | Non-SMC Condensin II Complex Subunit D3 | Protein Coding | 37 | GC11M134150 | 2.155363798 |
| ARFGEF1 | ADP Ribosylation Factor Guanine Nucleotide Exchange Factor 1 | Protein Coding | 34 | GC08M067173 | 2.154915571 |
| IL17RC | Interleukin 17 Receptor C | Protein Coding | 38 | GC03P009917 | 2.154779196 |
| BTLA | B And T Lymphocyte Associated | Protein Coding | 35 | GC03M112463 | 2.154626846 |
| ABCD4 | ATP Binding Cassette Subfamily D Member 4 | Protein Coding | 39 | GC14M074285 | 2.154593229 |
| GNG13 | G Protein Subunit Gamma 13 | Protein Coding | 33 | GC16M006620 | 2.154409647 |
| UBE2F | Ubiquitin Conjugating Enzyme E2 F (Putative) | Protein Coding | 35 | GC02P238003 | 2.154019117 |
| ADARB1 | Adenosine Deaminase RNA Specific B1 | Protein Coding | 40 | GC21P045073 | 2.153588533 |
| ZNF385D | Zinc Finger Protein 385D | Protein Coding | 29 | GC03M021412 | 2.153526783 |
| CAMK1D | Calcium/Calmodulin Dependent Protein Kinase ID | Protein Coding | 37 | GC10P012349 | 2.153455734 |
| GSDMC | Gasdermin C | Protein Coding | 30 | GC08M129705 | 2.153295994 |
| ATP6V0B | ATPase H+ Transporting V0 Subunit B | Protein Coding | 33 | GC01P043974 | 2.153270006 |
| KIF5A | Kinesin Family Member 5A | Protein Coding | 40 | GC12P057549 | 2.152782679 |
| MYO1E | Myosin IE | Protein Coding | 39 | GC15M059132 | 2.152329683 |
| AP3D1 | Adaptor Related Protein Complex 3 Subunit Delta 1 | Protein Coding | 36 | GC19M004958 | 2.151820183 |
| BLVRB | Biliverdin Reductase B | Protein Coding | 35 | GC19M040447 | 2.151582718 |
| IFRD1 | Interferon Related Developmental Regulator 1 | Protein Coding | 36 | GC07P112422 | 2.15151 |
| ATP5MK | ATP Synthase Membrane Subunit K | Protein Coding | 28 | GC10M103391 | 2.151340008 |
| STMN3 | Stathmin 3 | Protein Coding | 33 | GC20M063639 | 2.151088238 |
| SLC29A4 | Solute Carrier Family 29 Member 4 | Protein Coding | 38 | GC07P005289 | 2.151032448 |
| MT-TH | Mitochondrially Encoded TRNA-His (CAU/C) | RNA Gene | 13 | GCMTP012140 | 2.150928736 |
| LINC00328 | Long Intergenic Non-Protein Coding RNA 328 | RNA Gene | 7 | GC21U900445 | 2.150922298 |
| GBP1 | Guanylate Binding Protein 1 | Protein Coding | 36 | GC01M089052 | 2.150623322 |
| SPTLC2 | Serine Palmitoyltransferase Long Chain Base Subunit 2 | Protein Coding | 43 | GC14M077505 | 2.150569201 |
| GNB5 | G Protein Subunit Beta 5 | Protein Coding | 40 | GC15M081700 | 2.150515079 |
| CUL9 | Cullin 9 | Protein Coding | 34 | GC06P043182 | 2.15040803 |
| ANO9 | Anoctamin 9 | Protein Coding | 32 | GC11M002867 | 2.150337696 |
| IGSF11 | Immunoglobulin Superfamily Member 11 | Protein Coding | 32 | GC03M118900 | 2.150330305 |
| RASIP1 | Ras Interacting Protein 1 | Protein Coding | 34 | GC19M048720 | 2.150314569 |
| VIRMA | Vir Like M6A Methyltransferase Associated | Protein Coding | 27 | GC08M094490 | 2.149802208 |
| ITGB7 | Integrin Subunit Beta 7 | Protein Coding | 40 | GC12M053191 | 2.149305344 |
| ATG9B | Autophagy Related 9B | Protein Coding | 31 | GC07M151012 | 2.149187565 |
| CAPZA1 | Capping Actin Protein Of Muscle Z-Line Subunit Alpha 1 | Protein Coding | 38 | GC01P112619 | 2.147975922 |
| SERPINB1 | Serpin Family B Member 1 | Protein Coding | 36 | GC06M002833 | 2.147405624 |
| CLCN3 | Chloride Voltage-Gated Channel 3 | Protein Coding | 40 | GC04P169612 | 2.147103786 |
| ZNF292 | Zinc Finger Protein 292 | Protein Coding | 32 | GC06P087153 | 2.146358252 |
| U2SURP | U2 SnRNP Associated SURP Domain Containing | Protein Coding | 31 | GC03P142964 | 2.146341801 |
| CST1 | Cystatin SN | Protein Coding | 34 | GC20M023728 | 2.14583993 |
| STIM2 | Stromal Interaction Molecule 2 | Protein Coding | 35 | GC04P026859 | 2.145813942 |
| LOC105447645 | Uncharacterized LOC105447645 | RNA Gene | 7 | GC19M064077 | 2.145799637 |
| PARP9 | Poly(ADP-Ribose) Polymerase Family Member 9 | Protein Coding | 35 | GC03M122527 | 2.145798683 |
| CADPS | Calcium Dependent Secretion Activator | Protein Coding | 38 | GC03M062398 | 2.145774603 |
| PAGR1 | PAXIP1 Associated Glutamate Rich Protein 1 | Protein Coding | 28 | GC16P040194 | 2.145411968 |
| STK38 | Serine/Threonine Kinase 38 | Protein Coding | 39 | GC06M036493 | 2.145011902 |
| LSM4 | LSM4 Homolog, U6 Small Nuclear RNA And MRNA Degradation Associated | Protein Coding | 34 | GC19M018306 | 2.144993305 |
| TMEM39A | Transmembrane Protein 39A | Protein Coding | 30 | GC03M119428 | 2.144922733 |
| CATSPERE | Catsper Channel Auxiliary Subunit Epsilon | Protein Coding | 22 | GC01P244452 | 2.144922733 |
| IL25 | Interleukin 25 | Protein Coding | 35 | GC14P031744 | 2.144799471 |
| BASP1 | Brain Abundant Membrane Attached Signal Protein 1 | Protein Coding | 34 | GC05P017065 | 2.144109488 |
| MIER3 | MIER Family Member 3 | Protein Coding | 28 | GC05M056920 | 2.143981457 |
| SLC35E1 | Solute Carrier Family 35 Member E1 | Protein Coding | 30 | GC19M016549 | 2.143849373 |
| MAP3K3 | Mitogen-Activated Protein Kinase Kinase Kinase 3 | Protein Coding | 42 | GC17P063622 | 2.143827438 |
| MRPL58 | Mitochondrial Ribosomal Protein L58 | Protein Coding | 31 | GC17P075015 | 2.143575907 |
| IGSF10 | Immunoglobulin Superfamily Member 10 | Protein Coding | 32 | GC03M151425 | 2.143499851 |
| ZNF510 | Zinc Finger Protein 510 | Protein Coding | 30 | GC09M096755 | 2.143143892 |
| SSBP1 | Single Stranded DNA Binding Protein 1 | Protein Coding | 35 | GC07P148227 | 2.142759323 |
| IFT172 | Intraflagellar Transport 172 | Protein Coding | 35 | GC02M028071 | 2.142735004 |
| FBXL7 | F-Box And Leucine Rich Repeat Protein 7 | Protein Coding | 32 | GC05P015553 | 2.142636776 |
| CIAO2B | Cytosolic Iron-Sulfur Assembly Component 2B | Protein Coding | 29 | GC16M066988 | 2.142175436 |
| CACNG5 | Calcium Voltage-Gated Channel Auxiliary Subunit Gamma 5 | Protein Coding | 36 | GC17P066835 | 2.141566753 |
| KCNA1 | Potassium Voltage-Gated Channel Subfamily A Member 1 | Protein Coding | 41 | GC12P019790 | 2.141396284 |
| COG2 | Component Of Oligomeric Golgi Complex 2 | Protein Coding | 37 | GC01P230642 | 2.141159534 |
| PCBD1 | Pterin-4 Alpha-Carbinolamine Dehydratase 1 | Protein Coding | 40 | GC10M070882 | 2.140846252 |
| DDX20 | DEAD-Box Helicase 20 | Protein Coding | 38 | GC01P111755 | 2.140685081 |
| ADAMTS8 | ADAM Metallopeptidase With Thrombospondin Type 1 Motif 8 | Protein Coding | 37 | GC11M130404 | 2.140557528 |
| CHMP1A | Charged Multivesicular Body Protein 1A | Protein Coding | 36 | GC16M089644 | 2.140513182 |
| NRARP | NOTCH Regulated Ankyrin Repeat Protein | Protein Coding | 31 | GC09M137299 | 2.140301228 |
| TIGIT | T Cell Immunoreceptor With Ig And ITIM Domains | Protein Coding | 35 | GC03P114276 | 2.140217304 |
| TRA2A | Transformer 2 Alpha Homolog | Protein Coding | 35 | GC07M023504 | 2.140207767 |
| KLHL22 | Kelch Like Family Member 22 | Protein Coding | 33 | GC22M020442 | 2.139373302 |
| NFKBIB | NFKB Inhibitor Beta | Protein Coding | 36 | GC19P038899 | 2.139266491 |

| | | | | | |
|---|---|---|---|---|---|
| ZC3H7B | Zinc Finger CCCH-Type Containing 7B | Protein Coding | 33 | GC22P041301 | 2.13863039 |
| S100A16 | S100 Calcium Binding Protein A16 | Protein Coding | 33 | GC01M153606 | 2.137934923 |
| NDUFB9 | NADH:Ubiquinone Oxidoreductase Subunit B9 | Protein Coding | 40 | GC08P124539 | 2.137649298 |
| YPEL2 | Yippee Like 2 | Protein Coding | 32 | GC17P059331 | 2.1368258 |
| KIAA1109 | KIAA1109 | Protein Coding | 32 | GC04P122152 | 2.136649609 |
| RAET1G | Retinoic Acid Early Transcript 1G | Protein Coding | 30 | GC06M149916 | 2.136508942 |
| ZC3H12A | Zinc Finger CCCH-Type Containing 12A | Protein Coding | 33 | GC01P037474 | 2.136205196 |
| ADGRF4 | Adhesion G Protein-Coupled Receptor F4 | Protein Coding | 30 | GC06P047685 | 2.1358459 |
| FASTKD3 | FAST Kinase Domains 3 | Protein Coding | 29 | GC05M007860 | 2.1358459 |
| AGTPBP1 | ATP/GTP Binding Carboxypeptidase 1 | Protein Coding | 39 | GC09M085546 | 2.134513855 |
| PPID | Peptidylprolyl Isomerase D | Protein Coding | 39 | GC04M158709 | 2.134435177 |
| PRPF38B | Pre-MRNA Processing Factor 38B | Protein Coding | 28 | GC01P108692 | 2.133476257 |
| CAMSAP1 | Calmodulin Regulated Spectrin Associated Protein 1 | Protein Coding | 32 | GC09M135808 | 2.13334775 |
| TRIM46 | Tripartite Motif Containing 46 | Protein Coding | 30 | GC01P155173 | 2.132838249 |
| SCARA5 | Scavenger Receptor Class A Member 5 | Protein Coding | 36 | GC08M027869 | 2.132653713 |
| RARRES2 | Retinoic Acid Receptor Responder 2 | Protein Coding | 35 | GC07M150333 | 2.131342411 |
| SNRNP25 | Small Nuclear Ribonucleoprotein U11/U12 Subunit 25 | Protein Coding | 30 | GC16P000053 | 2.131164074 |
| SSR2 | Signal Sequence Receptor Subunit 2 | Protein Coding | 36 | GC01M156009 | 2.130225182 |
| GRB14 | Growth Factor Receptor Bound Protein 14 | Protein Coding | 39 | GC02M164492 | 2.129986763 |
| UGT2B11 | UDP Glucuronosyltransferase Family 2 Member B11 | Protein Coding | 34 | GC04M069199 | 2.12961936 |
| PURB | Purine Rich Element Binding Protein B | Protein Coding | 32 | GC07M044879 | 2.129512787 |
| MIR613 | MicroRNA 613 | RNA Gene | 13 | GC12P020073 | 2.129491329 |
| PRIM1 | DNA Primase Subunit 1 | Protein Coding | 38 | GC12M056731 | 2.129258394 |
| PART1 | Prostate Androgen-Regulated Transcript 1 | RNA Gene | 22 | GC05P060524 | 2.127700806 |
| GUCY1A2 | Guanylate Cyclase 1 Soluble Subunit Alpha 2 | Protein Coding | 36 | GC11M106591 | 2.127675056 |
| FAM72A | Family With Sequence Similarity 72 Member A | Protein Coding | 27 | GC01M206186 | 2.12733984 |
| FA2H | Fatty Acid 2-Hydroxylase | Protein Coding | 40 | GC16M074712 | 2.126280785 |
| TUT4 | Terminal Uridylyl Transferase 4 | Protein Coding | 28 | GC01M052410 | 2.126220703 |
| RPP14 | Ribonuclease P/MRP Subunit P14 | Protein Coding | 31 | GC03P058295 | 2.125951767 |
| OR1N1 | Olfactory Receptor Family 1 Subfamily N Member 1 | Protein Coding | 28 | GC09M122526 | 2.125805616 |
| TRIML1 | Tripartite Motif Family Like 1 | Protein Coding | 28 | GC04P188137 | 2.125805616 |
| SPATA21 | Spermatogenesis Associated 21 | Protein Coding | 27 | GC01M016387 | 2.125805616 |
| ZNF438 | Zinc Finger Protein 438 | Protein Coding | 27 | GC10M030820 | 2.125805616 |
| DIMT1 | DIM1 RRNA Methyltransferase And Ribosome Maturation Factor | Protein Coding | 32 | GC05M062387 | 2.12437892 |
| RNF130 | Ring Finger Protein 130 | Protein Coding | 35 | GC05M179911 | 2.124377966 |
| UBE2Q1 | Ubiquitin Conjugating Enzyme E2 Q1 | Protein Coding | 33 | GC01M154548 | 2.124276161 |
| FUT1 | Fucosyltransferase 1 (H Blood Group) | Protein Coding | 38 | GC19M048748 | 2.124181032 |
| POU2F2 | POU Class 2 Homeobox 2 | Protein Coding | 38 | GC19M042086 | 2.123861551 |
| NME4 | NME/NM23 Nucleoside Diphosphate Kinase 4 | Protein Coding | 37 | GC16P000396 | 2.123524666 |
| MIR556 | MicroRNA 556 | RNA Gene | 16 | GC01P162342 | 2.12350893 |
| NCR2 | Natural Cytotoxicity Triggering Receptor 2 | Protein Coding | 38 | GC06P080570 | 2.123190165 |
| EIF4G2 | Eukaryotic Translation Initiation Factor 4 Gamma 2 | Protein Coding | 38 | GC11M011036 | 2.122994423 |
| BICRA | BRD4 Interacting Chromatin Remodeling Complex Associated Protein | Protein Coding | 27 | GC19P063949 | 2.122197866 |
| ABCC10 | ATP Binding Cassette Subfamily C Member 10 | Protein Coding | 36 | GC06P043427 | 2.122166157 |
| FDFT1 | Farnesyl-Diphosphate Farnesyltransferase 1 | Protein Coding | 39 | GC08P011795 | 2.122120857 |
| CSRP3 | Cysteine And Glycine Rich Protein 3 | Protein Coding | 38 | GC11M019160 | 2.121662617 |
| TEN1 | TEN1 Subunit Of CST Complex | Protein Coding | 26 | GC17P075987 | 2.121598244 |
| YME1L1 | YME1 Like 1 ATPase | Protein Coding | 39 | GC10M027110 | 2.121263266 |
| MYOCD | Myocardin | Protein Coding | 38 | GC17P012665 | 2.121101856 |
| KIR2DL3 | Killer Cell Immunoglobulin Like Receptor, Two Ig Domains And Long Cytoplasmic Tail 3 | Protein Coding | 35 | GC19P064973 | 2.120776653 |
| EXOSC1 | Exosome Component 1 | Protein Coding | 34 | GC10M097435 | 2.120461226 |
| PPIE | Peptidylprolyl Isomerase E | Protein Coding | 36 | GC01P039692 | 2.120227575 |
| NPBWR2 | Neuropeptides B And W Receptor 2 | Protein Coding | 31 | GC20M064105 | 2.120103598 |
| FNDC5 | Fibronectin Type III Domain Containing 5 | Protein Coding | 32 | GC01M033137 | 2.120004416 |
| CHCHD3 | Coiled-Coil-Helix-Coiled-Coil-Helix Domain Containing 3 | Protein Coding | 33 | GC07M132784 | 2.11993432 |
| TENT4B | Terminal Nucleotidyltransferase 4B | Protein Coding | 28 | GC16P050528 | 2.119788408 |
| PPIAL4A | Peptidylprolyl Isomerase A Like 4A | Protein Coding | 24 | GC01P120889 | 2.119608164 |
| MIR524 | MicroRNA 524 | RNA Gene | 14 | GC19P053711 | 2.118647575 |
| THOC3 | THO Complex 3 | Protein Coding | 32 | GC05M175917 | 2.117890835 |
| RNASEH2A | Ribonuclease H2 Subunit A | Protein Coding | 39 | GC19P013682 | 2.117812157 |
| CHRNA9 | Cholinergic Receptor Nicotinic Alpha 9 Subunit | Protein Coding | 39 | GC04P040337 | 2.117212057 |
| NR1D1 | Nuclear Receptor Subfamily 1 Group D Member 1 | Protein Coding | 43 | GC17M040092 | 2.116595268 |
| SLC30A4 | Solute Carrier Family 30 Member 4 | Protein Coding | 35 | GC15M045479 | 2.116338253 |
| LPIN2 | Lipin 2 | Protein Coding | 38 | GC18M002916 | 2.11630249 |
| UST | Uronyl 2-Sulfotransferase | Protein Coding | 34 | GC06P148721 | 2.115691662 |
| ZMYM4 | Zinc Finger MYM-Type Containing 4 | Protein Coding | 31 | GC01P035268 | 2.11515379 |
| POLR2K | RNA Polymerase II, I And III Subunit K | Protein Coding | 32 | GC08P100150 | 2.114741325 |
| BAZ1A | Bromodomain Adjacent To Zinc Finger Domain 1A | Protein Coding | 36 | GC14M034752 | 2.114479065 |
| KCNJ15 | Potassium Inwardly Rectifying Channel Subfamily J Member 15 | Protein Coding | 38 | GC21P038157 | 2.114409447 |
| ZNF75A | Zinc Finger Protein 75a | Protein Coding | 30 | GC16P010786 | 2.114409447 |
| JAKMIP3 | Janus Kinase And Microtubule Interacting Protein 3 | Protein Coding | 28 | GC10P132065 | 2.114409447 |
| WDR53 | WD Repeat Domain 53 | Protein Coding | 28 | GC03M196554 | 2.114409447 |
| ZNF541 | Zinc Finger Protein 541 | Protein Coding | 27 | GC19M047520 | 2.114409447 |
| FAM217B | Family With Sequence Similarity 217 Member B | Protein Coding | 26 | GC20P059933 | 2.114409447 |
| C5orf34 | Chromosome 5 Open Reading Frame 34 | Protein Coding | 25 | GC05M043980 | 2.114409447 |
| HEATR9 | HEAT Repeat Containing 9 | Protein Coding | 25 | GC17M035854 | 2.114409447 |
| TMEM125 | Transmembrane Protein 125 | Protein Coding | 25 | GC01P043269 | 2.114409447 |
| C4orf50 | Chromosome 4 Open Reading Frame 50 | Protein Coding | 23 | GC04M005897 | 2.114409447 |
| KRTAP20-1 | Keratin Associated Protein 20-1 | Protein Coding | 21 | GC21P030616 | 2.114409447 |
| KRTAP21-1 | Keratin Associated Protein 21-1 | Protein Coding | 18 | GC21M030754 | 2.114409447 |
| DHX29 | DExH-Box Helicase 29 | Protein Coding | 34 | GC05M055256 | 2.114369869 |
| MFHAS1 | Multifunctional ROCO Family Signaling Regulator 1 | Protein Coding | 31 | GC08M008783 | 2.114082813 |
| TFCP2 | Transcription Factor CP2 | Protein Coding | 36 | GC12M051093 | 2.113852978 |
| LYPD6B | LY6/PLAUR Domain Containing 6B | Protein Coding | 31 | GC02P149038 | 2.113758326 |
| CDC34 | Cell Division Cycle 34, Ubiqiutin Conjugating Enzyme | Protein Coding | 41 | GC19P002455 | 2.113354206 |
| ANKRD65 | Ankyrin Repeat Domain 65 | Protein Coding | 25 | GC01M001418 | 2.113345861 |
| PDE7B | Phosphodiesterase 7B | Protein Coding | 36 | GC06P135795 | 2.113264561 |
| DOK2 | Docking Protein 2 | Protein Coding | 38 | GC08M021908 | 2.113159418 |
| MMRN2 | Multimerin 2 | Protein Coding | 31 | GC10M086935 | 2.112196684 |
| MIR133A2 | MicroRNA 133a-2 | RNA Gene | 18 | GC20P063301 | 2.111519814 |
| GLIS1 | GLIS Family Zinc Finger 1 | Protein Coding | 30 | GC01M053507 | 2.111144543 |
| SLC25A20 | Solute Carrier Family 25 Member 20 | Protein Coding | 40 | GC03M048869 | 2.111063242 |
| TOM1L2 | Target Of Myb1 Like 2 Membrane Trafficking Protein | Protein Coding | 34 | GC17M017843 | 2.111052513 |
| LIPA | Lipase A, Lysosomal Acid Type | Protein Coding | 44 | GC10M089213 | 2.110537767 |

| | | | | | |
|---|---|---|---|---|---|
| ZNF442 | Zinc Finger Protein 442 | Protein Coding | 28 | GC19M012350 | 2.110400915 |
| C11orf80 | Chromosome 11 Open Reading Frame 80 | Protein Coding | 31 | GC11P066744 | 2.108680487 |
| TRIM5 | Tripartite Motif Containing 5 | Protein Coding | 36 | GC11M006361 | 2.108240604 |
| ACSL1 | Acyl-CoA Synthetase Long Chain Family Member 1 | Protein Coding | 39 | GC04M184755 | 2.107970715 |
| COL8A1 | Collagen Type VIII Alpha 1 Chain | Protein Coding | 35 | GC03P099638 | 2.107954502 |
| FAM20C | FAM20C Golgi Associated Secretory Pathway Kinase | Protein Coding | 40 | GC07P000192 | 2.10793972 |
| SOX21 | SRY-Box Transcription Factor 21 | Protein Coding | 32 | GC13M094709 | 2.107908249 |
| POLN | DNA Polymerase Nu | Protein Coding | 30 | GC04M002155 | 2.107872725 |
| CDR1-AS | CDR1 Antisense RNA | RNA Gene | 7 | GC0XU902169 | 2.107583284 |
| ANP32A | Acidic Nuclear Phosphoprotein 32 Family Member A | Protein Coding | 38 | GC15M068778 | 2.107531071 |
| ADH4 | Alcohol Dehydrogenase 4 (Class II), Pi Polypeptide | Protein Coding | 39 | GC04M099123 | 2.107347727 |
| CENPU | Centromere Protein U | Protein Coding | 33 | GC04M184694 | 2.107130289 |
| HNRNPLL | Heterogeneous Nuclear Ribonucleoprotein L Like | Protein Coding | 32 | GC02M038561 | 2.107078075 |
| CDH7 | Cadherin 7 | Protein Coding | 36 | GC18P065750 | 2.106657505 |
| CLDN12 | Claudin 12 | Protein Coding | 35 | GC07P090383 | 2.106602669 |
| EIF3G | Eukaryotic Translation Initiation Factor 3 Subunit G | Protein Coding | 33 | GC19M010115 | 2.106048584 |
| GABRG2 | Gamma-Aminobutyric Acid Type A Receptor Subunit Gamma2 | Protein Coding | 44 | GC05P162000 | 2.105613708 |
| MIR488 | MicroRNA 488 | RNA Gene | 17 | GC01M177029 | 2.10304904 |
| CRYGC | Crystallin Gamma C | Protein Coding | 37 | GC02M208128 | 2.102282047 |
| IPO5 | Importin 5 | Protein Coding | 36 | GC13P097953 | 2.101926327 |
| PRKCSH | Protein Kinase C Substrate 80K-H | Protein Coding | 40 | GC19P011435 | 2.101706028 |
| MOGS | Mannosyl-Oligosaccharide Glucosidase | Protein Coding | 38 | GC02M074461 | 2.101522446 |
| SDS | Serine Dehydratase | Protein Coding | 38 | GC12M113392 | 2.101346254 |
| CDC42EP3 | CDC42 Effector Protein 3 | Protein Coding | 35 | GC02M037641 | 2.101185322 |
| NUP205 | Nucleoporin 205 | Protein Coding | 34 | GC07P135557 | 2.100954771 |
| DNAJB4 | DnaJ Heat Shock Protein Family (Hsp40) Member B4 | Protein Coding | 32 | GC01P077979 | 2.100828648 |
| PFN2 | Profilin 2 | Protein Coding | 35 | GC03M149964 | 2.100533962 |
| ACVR1C | Activin A Receptor Type 1C | Protein Coding | 39 | GC02M157526 | 2.100372791 |
| CARD16 | Caspase Recruitment Domain Family Member 16 | Protein Coding | 32 | GC11M105041 | 2.100193739 |
| MHRT | Myosin Heavy Chain Associated RNA Transcript | RNA Gene | 12 | GC14P032020 | 2.099012375 |
| CINP | Cyclin Dependent Kinase 2 Interacting Protein | Protein Coding | 31 | GC14M102342 | 2.098374128 |
| PGM1 | Phosphoglucomutase 1 | Protein Coding | 43 | GC01P063593 | 2.098167658 |
| SLC38A1 | Solute Carrier Family 38 Member 1 | Protein Coding | 36 | GC12M046183 | 2.09768343 |
| STAC3 | SH3 And Cysteine Rich Domain 3 | Protein Coding | 35 | GC12M057243 | 2.097672462 |
| UFD1 | Ubiquitin Recognition Factor In ER Associated Degradation 1 | Protein Coding | 34 | GC22M019450 | 2.096826077 |
| UNC45A | Unc-45 Myosin Chaperone A | Protein Coding | 33 | GC15P115315 | 2.095783472 |
| PPP1R3C | Protein Phosphatase 1 Regulatory Subunit 3C | Protein Coding | 36 | GC10M091628 | 2.095563173 |
| LOC110467515 | CYP1A1 5' Regulatory Region | Biological Region | 2 | GC15P074723 | 2.095467567 |
| ARLNC1 | Androgen Receptor Regulated Long Noncoding RNA 1 | RNA Gene | 11 | GC16M080830 | 2.09541297 |
| ASIC3 | Acid Sensing Ion Channel Subunit 3 | Protein Coding | 38 | GC07P151048 | 2.095321655 |
| ATP6V0A2 | ATPase H+ Transporting V0 Subunit A2 | Protein Coding | 40 | GC12P123712 | 2.094014645 |
| SOHLH2 | Spermatogenesis And Oogenesis Specific Basic Helix-Loop-Helix 2 | Protein Coding | 30 | GC13M036347 | 2.093521357 |
| ADO | 2-Aminoethanethiol Dioxygenase | Protein Coding | 34 | GC10P062804 | 2.092756271 |
| MSRA | Methionine Sulfoxide Reductase A | Protein Coding | 38 | GC08P010054 | 2.092648506 |
| PIMREG | PICALM Interacting Mitotic Regulator | Protein Coding | 27 | GC17P006444 | 2.091610432 |
| CAMK1G | Calcium/Calmodulin Dependent Protein Kinase IG | Protein Coding | 37 | GC01P209583 | 2.091127396 |
| LACRT | Lacritin | Protein Coding | 29 | GC12M054842 | 2.09112072 |
| PNPT1 | Polyribonucleotide Nucleotidyltransferase 1 | Protein Coding | 38 | GC02M055634 | 2.090630054 |
| CEBPZ | CCAAT Enhancer Binding Protein Zeta | Protein Coding | 32 | GC02M037201 | 2.090226173 |
| ARL2 | ADP Ribosylation Factor Like GTPase 2 | Protein Coding | 37 | GC11P065015 | 2.090009451 |
| KLKB1 | Kallikrein B1 | Protein Coding | 43 | GC04P186208 | 2.089592695 |
| LOC107372315 | OSGEP/APEX1 Bi-Directional Promoter Region | Biological Region | 3 | GC14P031495 | 2.089545965 |
| HUNK | Hormonally Up-Regulated Neu-Associated Kinase | Protein Coding | 32 | GC21P031873 | 2.089232206 |
| BCL2L15 | BCL2 Like 15 | Protein Coding | 28 | GC01M113876 | 2.088715553 |
| OXR1 | Oxidation Resistance 1 | Protein Coding | 36 | GC08P106271 | 2.088709354 |
| MED25 | Mediator Complex Subunit 25 | Protein Coding | 36 | GC19P064090 | 2.088507175 |
| SNX9 | Sorting Nexin 9 | Protein Coding | 36 | GC06P157685 | 2.08822298 |
| NFKBIL1 | NFKB Inhibitor Like 1 | Protein Coding | 34 | GC06P080350 | 2.087992668 |
| MIR18B | MicroRNA 18b | RNA Gene | 15 | GC0XM134303 | 2.08727932 |
| HAUS6 | HAUS Augmin Like Complex Subunit 6 | Protein Coding | 33 | GC09M019056 | 2.087059498 |
| CCNF | Cyclin F | Protein Coding | 37 | GC16P002429 | 2.086956739 |
| NBPF15 | NBPF Member 15 | Protein Coding | 25 | GC01M144421 | 2.086394072 |
| MED15 | Mediator Complex Subunit 15 | Protein Coding | 35 | GC22P034463 | 2.086332083 |
| MIR1236 | MicroRNA 1236 | RNA Gene | 13 | GC06M063629 | 2.086199284 |
| ARNT2 | Aryl Hydrocarbon Receptor Nuclear Translocator 2 | Protein Coding | 39 | GC15P080404 | 2.085914612 |
| MED24 | Mediator Complex Subunit 24 | Protein Coding | 32 | GC17M040019 | 2.085913897 |
| NAGA | Alpha-N-Acetylgalactosaminidase | Protein Coding | 40 | GC22M042058 | 2.085577488 |
| CAMKMT | Calmodulin-Lysine N-Methyltransferase | Protein Coding | 31 | GC02P044361 | 2.084050417 |
| GEMIN4 | Gem Nuclear Organelle Associated Protein 4 | Protein Coding | 35 | GC17M000744 | 2.083984137 |
| LBX1 | Ladybird Homeobox 1 | Protein Coding | 36 | GC10M101226 | 2.083047867 |
| RBM17 | RNA Binding Motif Protein 17 | Protein Coding | 34 | GC10P006089 | 2.083020449 |
| NIBAN2 | Niban Apoptosis Regulator 2 | Protein Coding | 30 | GC09M127564 | 2.082800627 |
| RSRC2 | Arginine And Serine Rich Coiled-Coil 2 | Protein Coding | 29 | GC12M122503 | 2.082462072 |
| SEC63 | SEC63 Homolog, Protein Translocation Regulator | Protein Coding | 39 | GC06M107867 | 2.082424641 |
| ACTR5 | Actin Related Protein 5 | Protein Coding | 32 | GC20P038748 | 2.082071781 |
| RRAGC | Ras Related GTP Binding C | Protein Coding | 36 | GC01M038857 | 2.08115983 |
| PSMB1 | Proteasome 20S Subunit Beta 1 | Protein Coding | 40 | GC06M170535 | 2.080813646 |
| ERGIC2 | ERGIC And Golgi 2 | Protein Coding | 32 | GC12M029337 | 2.080772877 |
| RIOK3 | RIO Kinase 3 | Protein Coding | 34 | GC18P023452 | 2.07969141 |
| ARHGAP32 | Rho GTPase Activating Protein 32 | Protein Coding | 34 | GC11M128965 | 2.07849741 |
| APBB1 | Amyloid Beta Precursor Protein Binding Family B Member 1 | Protein Coding | 39 | GC11M006404 | 2.078448772 |
| COBL | Cordon-Bleu WH2 Repeat Protein | Protein Coding | 31 | GC07M051016 | 2.07815671 |
| COX5B | Cytochrome C Oxidase Subunit 5B | Protein Coding | 36 | GC02P097628 | 2.077151775 |
| FBXL20 | F-Box And Leucine Rich Repeat Protein 20 | Protein Coding | 34 | GC17M039252 | 2.077132702 |
| FAM126B | Family With Sequence Similarity 126 Member B | Protein Coding | 28 | GC02M200973 | 2.076969862 |
| PTPN4 | Protein Tyrosine Phosphatase Non-Receptor Type 4 | Protein Coding | 36 | GC02P119759 | 2.076080799 |
| MYCBP | MYC Binding Protein | Protein Coding | 34 | GC01M038862 | 2.075504065 |
| EN1 | Engrailed Homeobox 1 | Protein Coding | 36 | GC02M118842 | 2.075460672 |
| KCTD7 | Potassium Channel Tetramerization Domain Containing 7 | Protein Coding | 35 | GC07P066628 | 2.07481432 |
| NEK5 | NIMA Related Kinase 5 | Protein Coding | 31 | GC13M052033 | 2.074807167 |
| ABCD3 | ATP Binding Cassette Subfamily D Member 3 | Protein Coding | 40 | GC01P094418 | 2.074665785 |
| NOD1 | Nucleotide Binding Oligomerization Domain Containing 1 | Protein Coding | 39 | GC07M030424 | 2.074107409 |
| ADPRH | ADP-Ribosylarginine Hydrolase | Protein Coding | 31 | GC03P119579 | 2.073933125 |
| NSUN4 | NOP2/Sun RNA Methyltransferase 4 | Protein Coding | 35 | GC01P046341 | 2.073468447 |
| NDUFS1 | NADH:Ubiquinone Oxidoreductase Core Subunit S1 | Protein Coding | 41 | GC02M206114 | 2.073207378 |

| | | | | | |
|---|---|---|---|---|---|
| LHPP | Phospholysine Phosphohistidine Inorganic Pyrophosphate Phosphatase | Protein Coding | 33 | GC10P124461 | 2.072090864 |
| SIGLEC1 | Sialic Acid Binding Ig Like Lectin 1 | Protein Coding | 36 | GC20M003686 | 2.07197237 |
| ENDOV | Endonuclease V | Protein Coding | 27 | GC17P080415 | 2.071866274 |
| TMEM108 | Transmembrane Protein 108 | Protein Coding | 30 | GC03P133038 | 2.071694136 |
| EIF3C | Eukaryotic Translation Initiation Factor 3 Subunit C | Protein Coding | 34 | GC16P040060 | 2.071558237 |
| VASH2 | Vasohibin 2 | Protein Coding | 31 | GC01P212935 | 2.07118845 |
| IFI30 | IFI30 Lysosomal Thiol Reductase | Protein Coding | 36 | GC19P018173 | 2.071115255 |
| FGD5 | FYVE, RhoGEF And PH Domain Containing 5 | Protein Coding | 34 | GC03P014810 | 2.070870399 |
| DNASE1L1 | Deoxyribonuclease 1 Like 1 | Protein Coding | 35 | GC0XM154401 | 2.070474148 |
| RHOG | Ras Homolog Family Member G | Protein Coding | 36 | GC11M003848 | 2.070356131 |
| ARHGEF16 | Rho Guanine Nucleotide Exchange Factor 16 | Protein Coding | 35 | GC01P003454 | 2.069613695 |
| MFAP2 | Microfibril Associated Protein 2 | Protein Coding | 37 | GC01M016974 | 2.069067717 |
| MIR3150B | MicroRNA 3150b | RNA Gene | 15 | GC08M095072 | 2.068820238 |
| BRD8 | Bromodomain Containing 8 | Protein Coding | 34 | GC05M138150 | 2.068357706 |
| GNA14 | G Protein Subunit Alpha 14 | Protein Coding | 39 | GC09M077423 | 2.06801486 |
| CLEC11A | C-Type Lectin Domain Containing 11A | Protein Coding | 35 | GC19P050723 | 2.067924023 |
| MIR1301 | MicroRNA 1301 | RNA Gene | 15 | GC02M025328 | 2.067604542 |
| EGFL7 | EGF Like Domain Multiple 7 | Protein Coding | 37 | GC09P136658 | 2.067123652 |
| WDR3 | WD Repeat Domain 3 | Protein Coding | 31 | GC01P117929 | 2.06695652 |
| DUOXA2 | Dual Oxidase Maturation Factor 2 | Protein Coding | 31 | GC15P045114 | 2.066808939 |
| PEBP4 | Phosphatidylethanolamine Binding Protein 4 | Protein Coding | 33 | GC08M022713 | 2.066289902 |
| UBL3 | Ubiquitin Like 3 | Protein Coding | 32 | GC13M029764 | 2.065279484 |
| RPP40 | Ribonuclease P/MRP Subunit P40 | Protein Coding | 30 | GC06M004994 | 2.065194607 |
| NUAK2 | NUAK Family Kinase 2 | Protein Coding | 37 | GC01M205302 | 2.064633369 |
| LOC110386948 | CYP19A1 Promoter I.4 | Biological Region | 2 | GC15P051836 | 2.064409733 |
| GNAL | G Protein Subunit Alpha L | Protein Coding | 40 | GC18P011689 | 2.064082146 |
| RGS12 | Regulator Of G Protein Signaling 12 | Protein Coding | 36 | GC04P003292 | 2.064017296 |
| RPL37 | Ribosomal Protein L37 | Protein Coding | 34 | GC05M040825 | 2.063634634 |
| DBNL | Drebrin Like | Protein Coding | 35 | GC07P044044 | 2.062219143 |
| EP400 | E1A Binding Protein P400 | Protein Coding | 32 | GC12P131949 | 2.062093973 |
| SUPT20H | SPT20 Homolog, SAGA Complex Component | Protein Coding | 32 | GC13M037009 | 2.062065125 |
| MKNK1 | MAPK Interacting Serine/Threonine Kinase 1 | Protein Coding | 42 | GC01M046557 | 2.061666489 |
| FRS3 | Fibroblast Growth Factor Receptor Substrate 3 | Protein Coding | 34 | GC06M063821 | 2.059887171 |
| MEP1B | Meprin A Subunit Beta | Protein Coding | 38 | GC18P032185 | 2.059685707 |
| FCN2 | Ficolin 2 | Protein Coding | 39 | GC09P134864 | 2.059330463 |
| PKP4 | Plakophilin 4 | Protein Coding | 36 | GC02P158456 | 2.0590837 |
| H2AC15 | H2A Clustered Histone 15 | Protein Coding | 26 | GC06M063340 | 2.057851315 |
| SBF2 | SET Binding Factor 2 | Protein Coding | 35 | GC11M009796 | 2.057531357 |
| CNOT7 | CCR4-NOT Transcription Complex Subunit 7 | Protein Coding | 35 | GC08M017224 | 2.057338953 |
| ACBD3 | Acyl-CoA Binding Domain Containing 3 | Protein Coding | 34 | GC01M226144 | 2.057248592 |
| DDX46 | DEAD-Box Helicase 46 | Protein Coding | 35 | GC05P134758 | 2.056719542 |
| SIPA1L3 | Signal Induced Proliferation Associated 1 Like 3 | Protein Coding | 35 | GC19P037906 | 2.056545258 |
| PKIA | CAMP-Dependent Protein Kinase Inhibitor Alpha | Protein Coding | 36 | GC08P078517 | 2.055725336 |
| GIT1 | GIT ArfGAP 1 | Protein Coding | 38 | GC17M029573 | 2.055714607 |
| CCDC33 | Coiled-Coil Domain Containing 33 | Protein Coding | 32 | GC15P114746 | 2.055663586 |
| SENP2 | SUMO Specific Peptidase 2 | Protein Coding | 36 | GC03P185582 | 2.055321217 |
| MIR940 | MicroRNA 940 | RNA Gene | 15 | GC16P002271 | 2.055288315 |
| GH2 | Growth Hormone 2 | Protein Coding | 35 | GC17M063880 | 2.054583788 |
| SF1 | Splicing Factor 1 | Protein Coding | 35 | GC11M064764 | 2.054559231 |
| SIM1 | SIM BHLH Transcription Factor 1 | Protein Coding | 38 | GC06M100386 | 2.054543018 |
| PPP1R8 | Protein Phosphatase 1 Regulatory Subunit 8 | Protein Coding | 35 | GC01P027830 | 2.05450058 |
| ATG3 | Autophagy Related 3 | Protein Coding | 35 | GC03M112532 | 2.054447412 |
| UBAP2 | Ubiquitin Associated Protein 2 | Protein Coding | 30 | GC09M033921 | 2.053543568 |
| RNPEP | Arginyl Aminopeptidase | Protein Coding | 37 | GC01P201982 | 2.053516388 |
| ACAD10 | Acyl-CoA Dehydrogenase Family Member 10 | Protein Coding | 35 | GC12P111686 | 2.053504467 |
| CDC42SE2 | CDC42 Small Effector 2 | Protein Coding | 31 | GC05P131245 | 2.052542686 |
| ELOA | Elongin A | Protein Coding | 30 | GC01P024450 | 2.051784039 |
| NCKIPSD | NCK Interacting Protein With SH3 Domain | Protein Coding | 33 | GC03M048667 | 2.051636934 |
| TRIM11 | Tripartite Motif Containing 11 | Protein Coding | 35 | GC01M228393 | 2.05145669 |
| ZNF230 | Zinc Finger Protein 230 | Protein Coding | 30 | GC19P044002 | 2.050983429 |
| MEG8 | Maternally Expressed 8, Small Nucleolar RNA Host Gene | RNA Gene | 19 | GC14P109507 | 2.050450087 |
| VPS13C | Vacuolar Protein Sorting 13 Homolog C | Protein Coding | 31 | GC15M061852 | 2.050443649 |
| SRPK2 | SRSF Protein Kinase 2 | Protein Coding | 40 | GC07M105110 | 2.049970388 |
| MIR4698 | MicroRNA 4698 | RNA Gene | 14 | GC12P047187 | 2.049895525 |
| BCAP31 | B Cell Receptor Associated Protein 31 | Protein Coding | 35 | GC0XM153701 | 2.049724102 |
| NPHP1 | Nephrocystin 1 | Protein Coding | 39 | GC02M110122 | 2.04897213 |
| ATG4A | Autophagy Related 4A Cysteine Peptidase | Protein Coding | 36 | GC0XP108091 | 2.048614502 |
| RNF41 | Ring Finger Protein 41 | Protein Coding | 35 | GC12M056202 | 2.04852438 |
| CDCA8 | Cell Division Cycle Associated 8 | Protein Coding | 34 | GC01P037692 | 2.048382282 |
| RAMP3 | Receptor Activity Modifying Protein 3 | Protein Coding | 38 | GC07P045163 | 2.04812479 |
| GNG10 | G Protein Subunit Gamma 10 | Protein Coding | 34 | GC09P111661 | 2.048082829 |
| NPTX1 | Neuronal Pentraxin 1 | Protein Coding | 35 | GC17M080466 | 2.04789114 |
| LSR | Lipolysis Stimulated Lipoprotein Receptor | Protein Coding | 37 | GC19P063446 | 2.047837496 |
| LAMC3 | Laminin Subunit Gamma 3 | Protein Coding | 38 | GC09P131009 | 2.047690153 |
| DEFA1 | Defensin Alpha 1 | Protein Coding | 36 | GC08M006977 | 2.047179222 |
| SLC23A2 | Solute Carrier Family 23 Member 2 | Protein Coding | 38 | GC20M004852 | 2.047168732 |
| CDKN2A-DT | CDKN2A Divergent Transcript | RNA Gene | 17 | GC09P021967 | 2.046564102 |
| SLC5A4 | Solute Carrier Family 5 Member 4 | Protein Coding | 38 | GC22M032218 | 2.046562433 |
| CTNNAL1 | Catenin Alpha Like 1 | Protein Coding | 34 | GC09M108942 | 2.04645586 |
| HRH3 | Histamine Receptor H3 | Protein Coding | 40 | GC20M062214 | 2.045076132 |
| DIP2B | Disco Interacting Protein 2 Homolog B | Protein Coding | 34 | GC12P050504 | 2.045062542 |
| FRA3B | Fragile Site, Aphidicolin Type, Common, Fra(3)(P14.2) | Uncategorized | 3 | GC00U921512 | 2.045037985 |
| SNX17 | Sorting Nexin 17 | Protein Coding | 32 | GC02P027370 | 2.044664383 |
| FGF11 | Fibroblast Growth Factor 11 | Protein Coding | 35 | GC17P010877 | 2.044569492 |
| MED16 | Mediator Complex Subunit 16 | Protein Coding | 30 | GC19M004821 | 2.044427395 |
| IK | IK Cytokine | Protein Coding | 35 | GC05P145798 | 2.044394732 |
| HSF2 | Heat Shock Transcription Factor 2 | Protein Coding | 39 | GC06P122399 | 2.044339657 |
| ATP5PO | ATP Synthase Peripheral Stalk Subunit OSCP | Protein Coding | 33 | GC21M033904 | 2.043936491 |
| HCG18 | HLA Complex Group 18 | RNA Gene | 17 | GC06M063511 | 2.043884039 |
| SLC30A5 | Solute Carrier Family 30 Member 5 | Protein Coding | 35 | GC05P069093 | 2.043200731 |
| LOC110006317 | Serine/Threonine Kinase 11 Intron 1 Alu-Mediated Recombination Region | Biological Region | 2 | GC19P002533 | 2.043170214 |
| LOC110006318 | Serine/Threonine Kinase 11 Intron 3 Alu-Mediated Recombination Region | Biological Region | 2 | GC19P002536 | 2.043170214 |
| H1-1 | H1.1 Linker Histone, Cluster Member | Protein Coding | 30 | GC06M026018 | 2.042214155 |
| LY6G5B | Lymphocyte Antigen 6 Family Member G5B | Protein Coding | 29 | GC06P080361 | 2.042113066 |
| TUBGCP4 | Tubulin Gamma Complex Associated Protein 4 | Protein Coding | 35 | GC15P043369 | 2.042103291 |

| | | | | | |
|---|---|---|---|---|---|
| KBTBD4 | Kelch Repeat And BTB Domain Containing 4 | Protein Coding | 30 | GC11M086889 | 2.042008162 |
| DCAKD | Dephospho-CoA Kinase Domain Containing | Protein Coding | 31 | GC17M045023 | 2.041502476 |
| HOXC8 | Homeobox C8 | Protein Coding | 33 | GC12P054972 | 2.041307688 |
| MIR3130-1 | MicroRNA 3130-1 | RNA Gene | 13 | GC02M206783 | 2.040416718 |
| SLC7A4 | Solute Carrier Family 7 Member 4 | Protein Coding | 34 | GC22M021028 | 2.040189028 |
| GOPC | Golgi Associated PDZ And Coiled-Coil Motif Containing | Protein Coding | 36 | GC06M117560 | 2.039500237 |
| DSCC1 | DNA Replication And Sister Chromatid Cohesion 1 | Protein Coding | 30 | GC08M119834 | 2.038181782 |
| MYL6B | Myosin Light Chain 6B | Protein Coding | 34 | GC12P057169 | 2.038061619 |
| ENPP3 | Ectonucleotide Pyrophosphatase/Phosphodiesterase 3 | Protein Coding | 39 | GC06P131617 | 2.037387609 |
| RFTN1 | Raftlin, Lipid Raft Linker 1 | Protein Coding | 34 | GC03M020274 | 2.037248611 |
| RPLP0P2 | Ribosomal Protein Lateral Stalk Subunit P0 Pseudogene 2 | Pseudogene | 14 | GC11P061633 | 2.036916733 |
| BRSK1 | BR Serine/Threonine Kinase 1 | Protein Coding | 38 | GC19P064361 | 2.03670907 |
| SLAMF6 | SLAM Family Member 6 | Protein Coding | 36 | GC01M160454 | 2.03662467 |
| ACER2 | Alkaline Ceramidase 2 | Protein Coding | 34 | GC09P019408 | 2.035946369 |
| ADCY4 | Adenylate Cyclase 4 | Protein Coding | 38 | GC14M024318 | 2.035541296 |
| RNGTT | RNA Guanylyltransferase And 5'-Phosphatase | Protein Coding | 37 | GC06M088609 | 2.03549695 |
| USP25 | Ubiquitin Specific Peptidase 25 | Protein Coding | 36 | GC21P015730 | 2.034768343 |
| PLA2R1 | Phospholipase A2 Receptor 1 | Protein Coding | 36 | GC02M159932 | 2.034479856 |
| EXOSC8 | Exosome Component 8 | Protein Coding | 36 | GC13P036998 | 2.034301758 |
| SRSF9 | Serine And Arginine Rich Splicing Factor 9 | Protein Coding | 35 | GC12M120461 | 2.033772469 |
| ELL2 | Elongation Factor For RNA Polymerase II 2 | Protein Coding | 35 | GC05M095885 | 2.033724546 |
| PYGB | Glycogen Phosphorylase B | Protein Coding | 38 | GC20P025248 | 2.033193111 |
| MSTN | Myostatin | Protein Coding | 42 | GC02M190055 | 2.032943964 |
| RYK | Receptor Like Tyrosine Kinase | Protein Coding | 38 | GC03M134065 | 2.031951427 |
| SENP3 | SUMO Specific Peptidase 3 | Protein Coding | 36 | GC17P010883 | 2.031915426 |
| HMG20A | High Mobility Group 20A | Protein Coding | 34 | GC15P077420 | 2.031831264 |
| NEDD1 | NEDD1 Gamma-Tubulin Ring Complex Targeting Factor | Protein Coding | 33 | GC12P096907 | 2.031644821 |
| PEX13 | Peroxisomal Biogenesis Factor 13 | Protein Coding | 37 | GC02P061017 | 2.031582117 |
| ZBED3 | Zinc Finger BED-Type Containing 3 | Protein Coding | 27 | GC05M077072 | 2.031387329 |
| NANOS3 | Nanos C2HC-Type Zinc Finger 3 | Protein Coding | 31 | GC19P013862 | 2.031214476 |
| WDR73 | WD Repeat Domain 73 | Protein Coding | 32 | GC15M084639 | 2.031204462 |
| MIR1297 | MicroRNA 1297 | RNA Gene | 13 | GC13M054311 | 2.03097868 |
| GRHPR | Glyoxylate And Hydroxypyruvate Reductase | Protein Coding | 42 | GC09P040221 | 2.030367136 |
| RP1 | RP1 Axonemal Microtubule Associated | Protein Coding | 34 | GC08P054555 | 2.03019309 |
| ST7L | Suppression Of Tumorigenicity 7 Like | Protein Coding | 29 | GC01M112523 | 2.030003548 |
| ITLN1 | Intelectin 1 | Protein Coding | 35 | GC01M160876 | 2.029878855 |
| ACTR3B | Actin Related Protein 3B | Protein Coding | 35 | GC07P152759 | 2.029619455 |
| EXOSC4 | Exosome Component 4 | Protein Coding | 31 | GC08P144279 | 2.029541492 |
| MRPL11 | Mitochondrial Ribosomal Protein L11 | Protein Coding | 34 | GC11M087334 | 2.029145002 |
| EPHA6 | EPH Receptor A6 | Protein Coding | 40 | GC03P096814 | 2.028978348 |
| CDHR1 | Cadherin Related Family Member 1 | Protein Coding | 36 | GC10P084194 | 2.028521776 |
| SP2 | Sp2 Transcription Factor | Protein Coding | 35 | GC17P047896 | 2.028426886 |
| CDK5RAP2 | CDK5 Regulatory Subunit Associated Protein 2 | Protein Coding | 38 | GC09M120388 | 2.027945757 |
| TAF4 | TATA-Box Binding Protein Associated Factor 4 | Protein Coding | 35 | GC20M062012 | 2.027805328 |
| HRK | Harakiri, BCL2 Interacting Protein | Protein Coding | 34 | GC12M116820 | 2.027314186 |
| MYO10 | Myosin X | Protein Coding | 36 | GC05M016661 | 2.027247667 |
| SPRR1B | Small Proline Rich Protein 1B | Protein Coding | 33 | GC01P153031 | 2.026929617 |
| ZZZ3 | Zinc Finger ZZ-Type Containing 3 | Protein Coding | 30 | GC01M077562 | 2.026379108 |
| DNAJA2 | DnaJ Heat Shock Protein Family (Hsp40) Member A2 | Protein Coding | 36 | GC16M046955 | 2.026277542 |
| PCDH17 | Protocadherin 17 | Protein Coding | 34 | GC13P057630 | 2.026250362 |
| TMEM18 | Transmembrane Protein 18 | Protein Coding | 32 | GC02M000660 | 2.026054382 |
| MNS16A | MNS16A Minisatellite Promoter | Biological Region | 2 | GC05P001260 | 2.025818825 |
| RBAK | RB Associated KRAB Zinc Finger | Protein Coding | 32 | GC07P005045 | 2.025808573 |
| SEMA6A | Semaphorin 6A | Protein Coding | 36 | GC05M116443 | 2.02568531 |
| RAB11FIP1 | RAB11 Family Interacting Protein 1 | Protein Coding | 35 | GC08M037858 | 2.024994612 |
| CROCC | Ciliary Rootlet Coiled-Coil, Rootletin | Protein Coding | 31 | GC01P017724 | 2.024619102 |
| PBX4 | PBX Homeobox 4 | Protein Coding | 32 | GC19M019561 | 2.024271011 |
| RGS19 | Regulator Of G Protein Signaling 19 | Protein Coding | 37 | GC20M064073 | 2.024059534 |
| LSM7 | LSM7 Homolog, U6 Small Nuclear RNA And MRNA Degradation Associated | Protein Coding | 33 | GC19M002321 | 2.023496389 |
| NDUFB3 | NADH:Ubiquinone Oxidoreductase Subunit B3 | Protein Coding | 38 | GC02P201071 | 2.023369312 |
| COL8A2 | Collagen Type VIII Alpha 2 Chain | Protein Coding | 38 | GC01M036095 | 2.023355961 |
| MORF4L2 | Mortality Factor 4 Like 2 | Protein Coding | 33 | GC0XM103675 | 2.023278713 |
| SFMBT1 | Scm Like With Four Mbt Domains 1 | Protein Coding | 32 | GC03M052939 | 2.023177624 |
| LYPD5 | LY6/PLAUR Domain Containing 5 | Protein Coding | 32 | GC19M063904 | 2.022486925 |
| CFAP418 | Cilia And Flagella Associated Protein 418 | Protein Coding | 26 | GC08M095245 | 2.022480488 |
| SEMA6B | Semaphorin 6B | Protein Coding | 34 | GC19M004542 | 2.021814823 |
| PRB4 | Proline Rich Protein BstNI Subfamily 4 | Protein Coding | 27 | GC12M011307 | 2.021635532 |
| MEI1 | Meiotic Double-Stranded Break Formation Protein 1 | Protein Coding | 32 | GC22P041699 | 2.02148962 |
| SUN2 | Sad1 And UNC84 Domain Containing 2 | Protein Coding | 35 | GC22M056102 | 2.021436453 |
| YTHDF3 | YTH N6-Methyladenosine RNA Binding Protein 3 | Protein Coding | 33 | GC08P063168 | 2.021303177 |
| SETD9 | SET Domain Containing 9 | Protein Coding | 27 | GC05P056909 | 2.021184444 |
| CDK17 | Cyclin Dependent Kinase 17 | Protein Coding | 36 | GC12M096278 | 2.021039963 |
| FNDC8 | Fibronectin Type III Domain Containing 8 | Protein Coding | 27 | GC17P052723 | 2.020870447 |
| MAGI3 | Membrane Associated Guanylate Kinase, WW And PDZ Domain Containing 3 | Protein Coding | 31 | GC01P113390 | 2.020727158 |
| PSMD12 | Proteasome 26S Subunit, Non-ATPase 12 | Protein Coding | 38 | GC17M067337 | 2.020560741 |
| CPSF4 | Cleavage And Polyadenylation Specific Factor 4 | Protein Coding | 34 | GC07P099438 | 2.020008802 |
| TNRC6A | Trinucleotide Repeat Containing Adaptor 6A | Protein Coding | 35 | GC16P024611 | 2.019603491 |
| ZNF225 | Zinc Finger Protein 225 | Protein Coding | 28 | GC19P063804 | 2.019275188 |
| IQUB | IQ Motif And Ubiquitin Domain Containing | Protein Coding | 27 | GC07M123451 | 2.01915741 |
| ADAMTS3 | ADAM Metallopeptidase With Thrombospondin Type 1 Motif 3 | Protein Coding | 38 | GC04M072280 | 2.019035339 |
| VPS45 | Vacuolar Protein Sorting 45 Homolog | Protein Coding | 35 | GC01P150114 | 2.019003391 |
| RBPMS | RNA Binding Protein, MRNA Processing Factor | Protein Coding | 35 | GC08P030361 | 2.018925667 |
| NR2C1 | Nuclear Receptor Subfamily 2 Group C Member 1 | Protein Coding | 35 | GC12M095022 | 2.018509388 |
| COX6B1 | Cytochrome C Oxidase Subunit 6B1 | Protein Coding | 38 | GC19P063455 | 2.017025471 |
| CCDC7 | Coiled-Coil Domain Containing 7 | Protein Coding | 28 | GC10P032454 | 2.016853094 |
| JPT1 | Jupiter Microtubule Associated Homolog 1 | Protein Coding | 26 | GC17M075136 | 2.016490936 |
| DEPTOR | DEP Domain Containing MTOR Interacting Protein | Protein Coding | 34 | GC08P119873 | 2.016332388 |
| ATP6AP1 | ATPase H+ Transporting Accessory Protein 1 | Protein Coding | 38 | GC0XP154428 | 2.016180992 |
| UBQLN2 | Ubiquilin 2 | Protein Coding | 38 | GC0XP056563 | 2.016072035 |
| ADAD1 | Adenosine Deaminase Domain Containing 1 | Protein Coding | 32 | GC04P122378 | 2.015862226 |
| USP38 | Ubiquitin Specific Peptidase 38 | Protein Coding | 30 | GC04P143184 | 2.015674353 |
| SHC2 | SHC Adaptor Protein 2 | Protein Coding | 34 | GC19M004777 | 2.015331507 |
| HOXC9 | Homeobox C9 | Protein Coding | 32 | GC12P054974 | 2.015240669 |
| C2orf80 | Chromosome 2 Open Reading Frame 80 | Protein Coding | 27 | GC02M208165 | 2.015042067 |
| TRDMT1 | TRNA Aspartic Acid Methyltransferase 1 | Protein Coding | 36 | GC10M017138 | 2.014940739 |

| | | | | | |
|---|---|---|---|---|---|
| A1CF | APOBEC1 Complementation Factor | Protein Coding | 33 | GC10M050799 | 2.014666319 |
| HTR3B | 5-Hydroxytryptamine Receptor 3B | Protein Coding | 40 | GC11P113940 | 2.014606953 |
| SLC12A5 | Solute Carrier Family 12 Member 5 | Protein Coding | 43 | GC20P046021 | 2.014583826 |
| RS1 | Retinoschisin 1 | Protein Coding | 35 | GC0XM018639 | 2.014563799 |
| CELSR2 | Cadherin EGF LAG Seven-Pass G-Type Receptor 2 | Protein Coding | 38 | GC01P109250 | 2.014561653 |
| TSN | Translin | Protein Coding | 36 | GC02P121737 | 2.014461994 |
| CCHCR1 | Coiled-Coil Alpha-Helical Rod Protein 1 | Protein Coding | 35 | GC06M063553 | 2.014242649 |
| HIF3A | Hypoxia Inducible Factor 3 Subunit Alpha | Protein Coding | 35 | GC19P046297 | 2.014116764 |
| GABBR2 | Gamma-Aminobutyric Acid Type B Receptor Subunit 2 | Protein Coding | 43 | GC09M098288 | 2.014046907 |
| SLC30A10 | Solute Carrier Family 30 Member 10 | Protein Coding | 38 | GC01M219685 | 2.013790369 |
| TONSL | Tonsoku Like, DNA Repair Protein | Protein Coding | 33 | GC08M144428 | 2.013365984 |
| CHST7 | Carbohydrate Sulfotransferase 7 | Protein Coding | 32 | GC0XP046574 | 2.012776375 |
| TBX15 | T-Box Transcription Factor 15 | Protein Coding | 37 | GC01M118883 | 2.012057304 |
| LUC7L | LUC7 Like | Protein Coding | 34 | GC16M000188 | 2.011769295 |
| MTFP1 | Mitochondrial Fission Process 1 | Protein Coding | 28 | GC22P034975 | 2.011715651 |
| ME1 | Malic Enzyme 1 | Protein Coding | 36 | GC06M083210 | 2.01140213 |
| RBM27 | RNA Binding Motif Protein 27 | Protein Coding | 31 | GC05P146204 | 2.011373758 |
| TAF5L | TATA-Box Binding Protein Associated Factor 5 Like | Protein Coding | 36 | GC01M229593 | 2.011248589 |
| ORC5 | Origin Recognition Complex Subunit 5 | Protein Coding | 32 | GC07M104126 | 2.011095524 |
| U2AF2 | U2 Small Nuclear RNA Auxiliary Factor 2 | Protein Coding | 32 | GC19P055654 | 2.011057615 |
| PI4KB | Phosphatidylinositol 4-Kinase Beta | Protein Coding | 39 | GC01M151291 | 2.010230064 |
| CA1 | Carbonic Anhydrase 1 | Protein Coding | 43 | GC08M085327 | 2.010213614 |
| GABARAPL2 | GABA Type A Receptor Associated Protein Like 2 | Protein Coding | 38 | GC16P075566 | 2.010191202 |
| PRAP1 | Proline Rich Acidic Protein 1 | Protein Coding | 31 | GC10P133347 | 2.009583235 |
| EHD1 | EH Domain Containing 1 | Protein Coding | 36 | GC11M087220 | 2.009371281 |
| USP4 | Ubiquitin Specific Peptidase 4 | Protein Coding | 39 | GC03M049277 | 2.008853436 |
| TSHZ3 | Teashirt Zinc Finger Homeobox 3 | Protein Coding | 34 | GC19M032784 | 2.00885272 |
| CNOT11 | CCR4-NOT Transcription Complex Subunit 11 | Protein Coding | 26 | GC02P101252 | 2.008145094 |
| UGP2 | UDP-Glucose Pyrophosphorylase 2 | Protein Coding | 38 | GC02P063840 | 2.007869244 |
| IRAK3 | Interleukin 1 Receptor Associated Kinase 3 | Protein Coding | 42 | GC12P066266 | 2.007219315 |
| WDR26 | WD Repeat Domain 26 | Protein Coding | 37 | GC01M224385 | 2.007174969 |
| GTF2IRD2 | GTF2I Repeat Domain Containing 2 | Protein Coding | 30 | GC07M074796 | 2.005931854 |
| LARGE2 | LARGE Xylosyl- And Glucuronyltransferase 2 | Protein Coding | 29 | GC11P046456 | 2.005801678 |
| PI4K2A | Phosphatidylinositol 4-Kinase Type 2 Alpha | Protein Coding | 36 | GC10P097640 | 2.005712986 |
| HBG1 | Hemoglobin Subunit Gamma 1 | Protein Coding | 38 | GC11M006311 | 2.005248785 |
| ADH7 | Alcohol Dehydrogenase 7 (Class IV), Mu Or Sigma Polypeptide | Protein Coding | 39 | GC04M099412 | 2.005004406 |
| SLC3A1 | Solute Carrier Family 3 Member 1 | Protein Coding | 43 | GC02P044275 | 2.004311085 |
| RTCB | RNA 2',3'-Cyclic Phosphate And 5'-OH Ligase | Protein Coding | 31 | GC22M032387 | 2.004170418 |
| PTPMT1 | Protein Tyrosine Phosphatase Mitochondrial 1 | Protein Coding | 32 | GC11P047568 | 2.003760099 |
| ARHGEF10 | Rho Guanine Nucleotide Exchange Factor 10 | Protein Coding | 39 | GC08P001823 | 2.003516197 |
| TMBIM6 | Transmembrane BAX Inhibitor Motif Containing 6 | Protein Coding | 34 | GC12P049707 | 2.003390551 |
| NBPF20 | NBPF Member 20 | Protein Coding | 21 | GC01M145289 | 2.003290176 |
| ALG12 | ALG12 Alpha-1,6-Mannosyltransferase | Protein Coding | 34 | GC22M056230 | 2.003204107 |
| SEC23A | SEC23 Homolog A, COPII Coat Complex Component | Protein Coding | 40 | GC14M039031 | 2.003112316 |
| SUMF1 | Sulfatase Modifying Factor 1 | Protein Coding | 38 | GC03M003700 | 2.002588272 |
| FKBPL | FKBP Prolyl Isomerase Like | Protein Coding | 33 | GC06M063634 | 2.002527237 |
| YTHDC1 | YTH Domain Containing 1 | Protein Coding | 32 | GC04M068310 | 2.002214432 |
| GTF2H2C | GTF2H2 Family Member C | Protein Coding | 30 | GC05P069560 | 2.002151012 |
| ZCCHC10 | Zinc Finger CCHC-Type Containing 10 | Protein Coding | 29 | GC05M132996 | 2.002109766 |
| SLC25A22 | Solute Carrier Family 25 Member 22 | Protein Coding | 36 | GC11M002927 | 2.001843691 |
| HMGN5 | High Mobility Group Nucleosome Binding Domain 5 | Protein Coding | 26 | GC0XM081113 | 2.001764774 |
| CPM | Carboxypeptidase M | Protein Coding | 39 | GC12M068842 | 2.001692057 |
| SLCO6A1 | Solute Carrier Organic Anion Transporter Family Member 6A1 | Protein Coding | 32 | GC05M102371 | 2.00159812 |
| SNTA1 | Syntrophin Alpha 1 | Protein Coding | 40 | GC20M033407 | 2.001071453 |
| DRAM2 | DNA Damage Regulated Autophagy Modulator 2 | Protein Coding | 34 | GC01M111117 | 2.000702858 |
| PARP3 | Poly(ADP-Ribose) Polymerase Family Member 3 | Protein Coding | 36 | GC03P052260 | 2.000654221 |
| BDKRB2 | Bradykinin Receptor B2 | Protein Coding | 40 | GC14P096205 | 2.000581503 |
| TWF1 | Twinfilin Actin Binding Protein 1 | Protein Coding | 34 | GC12M043793 | 2.000252724 |
| CLASP1 | Cytoplasmic Linker Associated Protein 1 | Protein Coding | 37 | GC02M121337 | 1.999840021 |
| SLC9A7 | Solute Carrier Family 9 Member A7 | Protein Coding | 35 | GC0XM046599 | 1.999568939 |
| TSPYL2 | TSPY Like 2 | Protein Coding | 34 | GC0XP053082 | 1.999329329 |
| HKDC1 | Hexokinase Domain Containing 1 | Protein Coding | 36 | GC10P069220 | 1.999090672 |
| MANSC4 | MANSC Domain Containing 4 | Protein Coding | 21 | GC12M027762 | 1.998913765 |
| UXT | Ubiquitously Expressed Prefoldin Like Chaperone | Protein Coding | 32 | GC0XM047651 | 1.998253345 |
| WDR36 | WD Repeat Domain 36 | Protein Coding | 34 | GC05P111091 | 1.997493148 |
| RXFP1 | Relaxin Family Peptide Receptor 1 | Protein Coding | 39 | GC04P158315 | 1.997369051 |
| PAK1IP1 | PAK1 Interacting Protein 1 | Protein Coding | 31 | GC06P010714 | 1.997204781 |
| SEMA6D | Semaphorin 6D | Protein Coding | 36 | GC15P047184 | 1.996956348 |
| CSRNP3 | Cysteine And Serine Rich Nuclear Protein 3 | Protein Coding | 31 | GC02P165469 | 1.996543884 |
| NUMBL | NUMB Like Endocytic Adaptor Protein | Protein Coding | 34 | GC19M040665 | 1.996372223 |
| DYNLT1 | Dynein Light Chain Tctex-Type 1 | Protein Coding | 33 | GC06M158636 | 1.995616198 |
| CHAF1A | Chromatin Assembly Factor 1 Subunit A | Protein Coding | 34 | GC19P004402 | 1.995565653 |
| ATAD3C | ATPase Family AAA Domain Containing 3C | Protein Coding | 30 | GC01P001449 | 1.994748354 |
| EFHC1 | EF-Hand Domain Containing 1 | Protein Coding | 37 | GC06P052362 | 1.99464798 |
| TBX18 | T-Box Transcription Factor 18 | Protein Coding | 38 | GC06M084687 | 1.994603515 |
| TRMT10C | TRNA Methyltransferase 10C, Mitochondrial RNase P Subunit | Protein Coding | 31 | GC03P101561 | 1.994570732 |
| FAM83H | Family With Sequence Similarity 83 Member H | Protein Coding | 34 | GC08M143723 | 1.99399209 |
| USO1 | USO1 Vesicle Transport Factor | Protein Coding | 34 | GC04P075724 | 1.993858337 |
| SETX | Senataxin | Protein Coding | 36 | GC09M132261 | 1.993631601 |
| AARS2 | Alanyl-TRNA Synthetase 2, Mitochondrial | Protein Coding | 38 | GC06M044297 | 1.993517399 |
| DUSP5 | Dual Specificity Phosphatase 5 | Protein Coding | 37 | GC10P110497 | 1.992933631 |
| MXD3 | MAX Dimerization Protein 3 | Protein Coding | 31 | GC05M177910 | 1.992674828 |
| TAOK1 | TAO Kinase 1 | Protein Coding | 39 | GC17P052527 | 1.992456436 |
| FBXL2 | F-Box And Leucine Rich Repeat Protein 2 | Protein Coding | 32 | GC03P033277 | 1.992064595 |
| MIR1179 | MicroRNA 1179 | RNA Gene | 15 | GC15P088608 | 1.991685987 |
| RANGAP1 | Ran GTPase Activating Protein 1 | Protein Coding | 36 | GC22M041244 | 1.9913975 |
| APOM | Apolipoprotein M | Protein Coding | 36 | GC06P080362 | 1.991143227 |
| VTI1B | Vesicle Transport Through Interaction With T-SNAREs 1B | Protein Coding | 36 | GC14M067647 | 1.990982771 |
| AATK | Apoptosis Associated Tyrosine Kinase | Protein Coding | 35 | GC17M081117 | 1.990961552 |
| MIR505 | MicroRNA 505 | RNA Gene | 17 | GC0XM139924 | 1.990428448 |
| MAFK | MAF BZIP Transcription Factor K | Protein Coding | 35 | GC07P001977 | 1.990260363 |
| CNMD | Chondromodulin | Protein Coding | 30 | GC13M052704 | 1.990208507 |
| GNAT2 | G Protein Subunit Alpha Transducin 2 | Protein Coding | 41 | GC01M109603 | 1.99011898 |
| MIR371B | MicroRNA 371b | RNA Gene | 10 | GC19M053787 | 1.989897251 |
| TCEAL7 | Transcription Elongation Factor A Like 7 | Protein Coding | 28 | GC0XP103330 | 1.98985076 |

| | | | | | | |
|---|---|---|---|---|---|---|
| ETFB | Electron Transfer Flavoprotein Subunit Beta | Protein Coding | 40 | GC19M051345 | 1.98984158 |
| MIR526B | MicroRNA 526b | RNA Gene | 17 | GC19P053694 | 1.989136815 |
| TPH1 | Tryptophan Hydroxylase 1 | Protein Coding | 39 | GC11M018040 | 1.98895669 |
| TANK | TRAF Family Member Associated NFKB Activator | Protein Coding | 38 | GC02P161136 | 1.988613963 |
| RASA4 | RAS P21 Protein Activator 4 | Protein Coding | 33 | GC07M103115 | 1.988302469 |
| DCST2 | DC-STAMP Domain Containing 2 | Protein Coding | 28 | GC01M155221 | 1.988122344 |
| OPHN1 | Oligophrenin 1 | Protein Coding | 38 | GC0XM067949 | 1.98725462 |
| MYOT | Myotilin | Protein Coding | 37 | GC05P137867 | 1.987112522 |
| KRTCAP2 | Keratinocyte Associated Protein 2 | Protein Coding | 30 | GC01M155229 | 1.986857772 |
| ADD3 | Adducin 3 | Protein Coding | 39 | GC10P109996 | 1.986570001 |
| SH3TC2 | SH3 Domain And Tetratricopeptide Repeats 2 | Protein Coding | 34 | GC05M148923 | 1.986306071 |
| TUBA8 | Tubulin Alpha 8 | Protein Coding | 40 | GC22P018110 | 1.986137867 |
| FBP2 | Fructose-Bisphosphatase 2 | Protein Coding | 38 | GC09M094558 | 1.985984087 |
| NCR3LG1 | Natural Killer Cell Cytotoxicity Receptor 3 Ligand 1 | Protein Coding | 28 | GC11P017351 | 1.985711336 |
| PAK3 | P21 (RAC1) Activated Kinase 3 | Protein Coding | 45 | GC0XP110944 | 1.985543489 |
| LMAN1L | Lectin, Mannose Binding 1 Like | Protein Coding | 32 | GC15P074812 | 1.985191584 |
| FRAT2 | FRAT Regulator Of WNT Signaling Pathway 2 | Protein Coding | 33 | GC10M097332 | 1.985032201 |
| MTERF4 | Mitochondrial Transcription Termination Factor 4 | Protein Coding | 28 | GC02M241072 | 1.984545112 |
| KDF1 | Keratinocyte Differentiation Factor 1 | Protein Coding | 29 | GC01M026949 | 1.984293938 |
| PDLIM1 | PDZ And LIM Domain 1 | Protein Coding | 36 | GC10M095237 | 1.984143376 |
| HRG | Histidine Rich Glycoprotein | Protein Coding | 41 | GC03P186660 | 1.984046578 |
| NOL7 | Nucleolar Protein 7 | Protein Coding | 31 | GC06P013615 | 1.9840312 |
| HMGN2 | High Mobility Group Nucleosomal Binding Domain 2 | Protein Coding | 36 | GC01P026473 | 1.983951926 |
| FBLN2 | Fibulin 2 | Protein Coding | 39 | GC03P013565 | 1.982781768 |
| ADRA2A | Adrenoceptor Alpha 2A | Protein Coding | 42 | GC10P111077 | 1.982727289 |
| HIRA | Histone Cell Cycle Regulator | Protein Coding | 39 | GC22M019318 | 1.982633233 |
| SP4 | Sp4 Transcription Factor | Protein Coding | 36 | GC07P021434 | 1.98250103 |
| LTK | Leukocyte Receptor Tyrosine Kinase | Protein Coding | 39 | GC15M041503 | 1.982491136 |
| STRA6 | Signaling Receptor And Transporter Of Retinol STRA6 | Protein Coding | 38 | GC15M074179 | 1.982408166 |
| MED21 | Mediator Complex Subunit 21 | Protein Coding | 31 | GC12P027022 | 1.98229301 |
| TFPT | TCF3 Fusion Partner | Protein Coding | 31 | GC19M054107 | 1.981832266 |
| GUCA1A | Guanylate Cyclase Activator 1A | Protein Coding | 36 | GC06P080584 | 1.981811047 |
| IGHG2 | Immunoglobulin Heavy Constant Gamma 2 (G2m Marker) | Protein Coding | 27 | GC14M112403 | 1.981508613 |
| ADGRF1 | Adhesion G Protein-Coupled Receptor F1 | Protein Coding | 30 | GC06M046997 | 1.98127985 |
| PPP1R14A | Protein Phosphatase 1 Regulatory Inhibitor Subunit 14A | Protein Coding | 35 | GC19M038251 | 1.981219172 |
| TRIM44 | Tripartite Motif Containing 44 | Protein Coding | 35 | GC11P035684 | 1.981138706 |
| AMOTL2 | Angiomotin Like 2 | Protein Coding | 32 | GC03M134355 | 1.979773998 |
| GLE1 | GLE1 RNA Export Mediator | Protein Coding | 36 | GC09P128504 | 1.978283167 |
| PPP3CA | Protein Phosphatase 3 Catalytic Subunit Alpha | Protein Coding | 47 | GC04M101024 | 1.978224277 |
| VAT1 | Vesicle Amine Transport 1 | Protein Coding | 32 | GC17M043014 | 1.97817564 |
| PWP1 | PWP1 Homolog, Endonuclein | Protein Coding | 30 | GC12P107685 | 1.977480412 |
| F2RL2 | Coagulation Factor II Thrombin Receptor Like 2 | Protein Coding | 39 | GC05M076615 | 1.977134943 |
| GLYR1 | Glyoxylate Reductase 1 Homolog | Protein Coding | 31 | GC16M006905 | 1.976432323 |
| GPAT2 | Glycerol-3-Phosphate Acyltransferase 2, Mitochondrial | Protein Coding | 28 | GC02M097997 | 1.976302147 |
| KLF15 | Kruppel Like Factor 15 | Protein Coding | 36 | GC03M126293 | 1.976175785 |
| C1D | C1D Nuclear Receptor Corepressor | Protein Coding | 34 | GC02M068041 | 1.976156354 |
| MEIOB | Meiosis Specific With OB-Fold | Protein Coding | 30 | GC16M001833 | 1.976149797 |
| KCNK2 | Potassium Two Pore Domain Channel Subfamily K Member 2 | Protein Coding | 38 | GC01P215005 | 1.976060867 |
| RF00017-749 | | RNA Gene | 5 | GC10P121558 | 1.975004435 |
| lnc-ATE1-6 | | RNA Gene | 5 | GC10M121564 | 1.975004435 |
| RNF144A | Ring Finger Protein 144A | Protein Coding | 35 | GC02P006917 | 1.974792004 |
| LY6E | Lymphocyte Antigen 6 Family Member E | Protein Coding | 35 | GC08P143017 | 1.974465489 |
| FAM53B | Family With Sequence Similarity 53 Member B | Protein Coding | 30 | GC10M124619 | 1.974462509 |
| IFT74 | Intraflagellar Transport 74 | Protein Coding | 34 | GC09P026947 | 1.97436893 |
| CEP170B | Centrosomal Protein 170B | Protein Coding | 27 | GC14P104865 | 1.973778248 |
| PKNOX1 | PBX/Knotted 1 Homeobox 1 | Protein Coding | 35 | GC21P042974 | 1.973534822 |
| TRIM13 | Tripartite Motif Containing 13 | Protein Coding | 32 | GC13P049995 | 1.973391294 |
| NAV1 | Neuron Navigator 1 | Protein Coding | 33 | GC01P201561 | 1.972284555 |
| CPNE1 | Copine 1 | Protein Coding | 35 | GC20M035626 | 1.972221851 |
| NSMCE3 | NSE3 Homolog, SMC5-SMC6 Complex Component | Protein Coding | 31 | GC15M029272 | 1.971663952 |
| WLS | Wnt Ligand Secretion Mediator | Protein Coding | 35 | GC01M068098 | 1.971453547 |
| MRGPRD | MAS Related GPR Family Member D | Protein Coding | 32 | GC11M068980 | 1.971389294 |
| HMGCS1 | 3-Hydroxy-3-Methylglutaryl-CoA Synthase 1 | Protein Coding | 36 | GC05M043454 | 1.970792532 |
| GABRR3 | Gamma-Aminobutyric Acid Type A Receptor Subunit Rho3 | Protein Coding | 35 | GC03M097986 | 1.970015883 |
| NXF1 | Nuclear RNA Export Factor 1 | Protein Coding | 36 | GC11M087137 | 1.969836116 |
| MRPS6 | Mitochondrial Ribosomal Protein S6 | Protein Coding | 33 | GC21P034132 | 1.969214201 |
| VPS18 | VPS18 Core Subunit Of CORVET And HOPS Complexes | Protein Coding | 32 | GC15P040894 | 1.968829155 |
| LNX1 | Ligand Of Numb-Protein X 1 | Protein Coding | 38 | GC04M053551 | 1.968327761 |
| HBB-LCR | Beta-Globin Locus Control Region | Biological Region | 6 | GC11P005350 | 1.968315125 |
| MED13 | Mediator Complex Subunit 13 | Protein Coding | 35 | GC17M061942 | 1.968264699 |
| RGL2 | Ral Guanine Nucleotide Dissociation Stimulator Like 2 | Protein Coding | 35 | GC06M033291 | 1.968208194 |
| MRPL45 | Mitochondrial Ribosomal Protein L45 | Protein Coding | 30 | GC17P038297 | 1.967960119 |
| STT3B | STT3 Oligosaccharyltransferase Complex Catalytic Subunit B | Protein Coding | 39 | GC03P031550 | 1.967872143 |
| STK24 | Serine/Threonine Kinase 24 | Protein Coding | 40 | GC13M098445 | 1.966903925 |
| GOT1 | Glutamic-Oxaloacetic Transaminase 1 | Protein Coding | 40 | GC10M099396 | 1.965945482 |
| XPR1 | Xenotropic And Polytropic Retrovirus Receptor 1 | Protein Coding | 39 | GC01P180632 | 1.965831518 |
| KCNH4 | Potassium Voltage-Gated Channel Subfamily H Member 4 | Protein Coding | 35 | GC17M042160 | 1.965643883 |
| ZG16B | Zymogen Granule Protein 16B | Protein Coding | 31 | GC16P010750 | 1.965421677 |
| DNTTIP1 | Deoxynucleotidyltransferase Terminal Interacting Protein 1 | Protein Coding | 32 | GC20P045791 | 1.965349674 |
| RPS4Y1 | Ribosomal Protein S4 Y-Linked 1 | Protein Coding | 28 | GC0YP002841 | 1.965315104 |
| HNRNPA0 | Heterogeneous Nuclear Ribonucleoprotein A0 | Protein Coding | 34 | GC05M137750 | 1.964539409 |
| H1-10 | H1.10 Linker Histone | Protein Coding | 27 | GC03M130196 | 1.964480758 |
| ERVFRD-1 | Endogenous Retrovirus Group FRD Member 1, Envelope | Protein Coding | 29 | GC06M011103 | 1.964454412 |
| HBS1L | HBS1 Like Translational GTPase | Protein Coding | 35 | GC06M134960 | 1.963167787 |
| PPP1R12B | Protein Phosphatase 1 Regulatory Subunit 12B | Protein Coding | 34 | GC01P202348 | 1.96288681 |
| HCRTR2 | Hypocretin Receptor 2 | Protein Coding | 40 | GC06P055106 | 1.962705374 |
| LILRB2 | Leukocyte Immunoglobulin Like Receptor B2 | Protein Coding | 36 | GC19M064363 | 1.962702513 |
| CCNL2 | Cyclin L2 | Protein Coding | 32 | GC01M001385 | 1.962176442 |
| RALGAPB | Ral GTPase Activating Protein Non-Catalytic Subunit Beta | Protein Coding | 31 | GC20P038472 | 1.962128401 |
| OVCH1-AS1 | OVCH1 Antisense RNA 1 | RNA Gene | 14 | GC12P029389 | 1.961753011 |
| AMPD1 | Adenosine Monophosphate Deaminase 1 | Protein Coding | 42 | GC01M114673 | 1.961684465 |
| PHRF1 | PHD And Ring Finger Domains 1 | Protein Coding | 31 | GC11P001656 | 1.961419582 |
| WDR4 | WD Repeat Domain 4 | Protein Coding | 35 | GC21M042843 | 1.961207032 |
| TMEM184B | Transmembrane Protein 184B | Protein Coding | 31 | GC22M057113 | 1.960924506 |
| ZC3H13 | Zinc Finger CCCH-Type Containing 13 | Protein Coding | 29 | GC13M045954 | 1.960583329 |

| | | | | | |
|---|---|---|---|---|---|
| ANGPT4 | Angiopoietin 4 | Protein Coding | 36 | GC20M000869 | 1.960552454 |
| SLC26A1 | Solute Carrier Family 26 Member 1 | Protein Coding | 34 | GC04M000979 | 1.960518599 |
| IDH3G | Isocitrate Dehydrogenase (NAD(+)) 3 Non-Catalytic Subunit Gamma | Protein Coding | 36 | GC0XM153785 | 1.960242748 |
| PFDN1 | Prefoldin Subunit 1 | Protein Coding | 33 | GC05M140297 | 1.960224152 |
| DCTD | DCMP Deaminase | Protein Coding | 37 | GC04M182890 | 1.960129976 |
| SLC9A3R2 | SLC9A3 Regulator 2 | Protein Coding | 34 | GC16P010712 | 1.959791064 |
| PARD6B | Par-6 Family Cell Polarity Regulator Beta | Protein Coding | 35 | GC20P050731 | 1.959020615 |
| MIR4693 | MicroRNA 4693 | RNA Gene | 12 | GC11P103849 | 1.958973169 |
| IL10RB | Interleukin 10 Receptor Subunit Beta | Protein Coding | 39 | GC21P033266 | 1.958686829 |
| SLC2A12 | Solute Carrier Family 2 Member 12 | Protein Coding | 35 | GC06M133987 | 1.958033919 |
| SHC4 | SHC Adaptor Protein 4 | Protein Coding | 32 | GC15M048823 | 1.957446098 |
| RBM33 | RNA Binding Motif Protein 33 | Protein Coding | 28 | GC07P155644 | 1.957286 |
| CTNS | Cystinosin, Lysosomal Cystine Transporter | Protein Coding | 41 | GC17P003636 | 1.956979752 |
| SLC39A8 | Solute Carrier Family 39 Member 8 | Protein Coding | 39 | GC04M102252 | 1.956287622 |
| RAB1B | RAB1B, Member RAS Oncogene Family | Protein Coding | 37 | GC11P069606 | 1.955946684 |
| HOXC10 | Homeobox C10 | Protein Coding | 34 | GC12P054971 | 1.955519676 |
| PLAGL2 | PLAG1 Like Zinc Finger 2 | Protein Coding | 33 | GC20M032192 | 1.955268145 |
| INTS10 | Integrator Complex Subunit 10 | Protein Coding | 31 | GC08P019817 | 1.955192685 |
| MIA3 | MIA SH3 Domain ER Export Factor 3 | Protein Coding | 34 | GC01P222618 | 1.954892159 |
| NEMP1 | Nuclear Envelope Integral Membrane Protein 1 | Protein Coding | 26 | GC12M057056 | 1.952837348 |
| NUP160 | Nucleoporin 160 | Protein Coding | 35 | GC11M086897 | 1.952799797 |
| PARD6A | Par-6 Family Cell Polarity Regulator Alpha | Protein Coding | 37 | GC16P067661 | 1.952319384 |
| MYNN | Myoneurin | Protein Coding | 34 | GC03P169773 | 1.952101231 |
| PARP8 | Poly(ADP-Ribose) Polymerase Family Member 8 | Protein Coding | 36 | GC05P050665 | 1.952070951 |
| TFCP2L1 | Transcription Factor CP2 Like 1 | Protein Coding | 33 | GC02M121216 | 1.951805592 |
| PRRC2C | Proline Rich Coiled-Coil 2C | Protein Coding | 30 | GC01P171486 | 1.951286912 |
| TASP1 | Taspase 1 | Protein Coding | 34 | GC20M013105 | 1.948962927 |
| C14orf93 | Chromosome 14 Open Reading Frame 93 | Protein Coding | 27 | GC14M022985 | 1.948577642 |
| LMAN1 | Lectin, Mannose Binding 1 | Protein Coding | 40 | GC18M059327 | 1.948519468 |
| RIMS1 | Regulating Synaptic Membrane Exocytosis 1 | Protein Coding | 34 | GC06P071886 | 1.948072195 |
| SHKBP1 | SH3KBP1 Binding Protein 1 | Protein Coding | 32 | GC19P040576 | 1.948035717 |
| SUPT16H | SPT16 Homolog, Facilitates Chromatin Remodeling Subunit | Protein Coding | 35 | GC14M021351 | 1.947436094 |
| WAPL | WAPL Cohesin Release Factor | Protein Coding | 31 | GC10M086436 | 1.947237849 |
| GOLGA2 | Golgin A2 | Protein Coding | 36 | GC09M128255 | 1.947215438 |
| SFI1 | SFI1 Centrin Binding Protein | Protein Coding | 31 | GC22P031488 | 1.946723819 |
| ARL8A | ADP Ribosylation Factor Like GTPase 8A | Protein Coding | 30 | GC01M202133 | 1.946508408 |
| CMSS1 | Cms1 Ribosomal Small Subunit Homolog | Protein Coding | 27 | GC03P099817 | 1.946347713 |
| SLC2A4RG | SLC2A4 Regulator | Protein Coding | 33 | GC20P063739 | 1.944946766 |
| KREMEN1 | Kringle Containing Transmembrane Protein 1 | Protein Coding | 40 | GC22P029073 | 1.944765329 |
| PAIP1 | Poly(A) Binding Protein Interacting Protein 1 | Protein Coding | 32 | GC05M043526 | 1.944019318 |
| KATNA1 | Katanin Catalytic Subunit A1 | Protein Coding | 36 | GC06M149594 | 1.94191587 |
| NRK | Nik Related Kinase | Protein Coding | 31 | GC0XP105822 | 1.941912651 |
| HES6 | Hes Family BHLH Transcription Factor 6 | Protein Coding | 35 | GC02M238238 | 1.941553831 |
| BAG2 | BAG Cochaperone 2 | Protein Coding | 35 | GC06P057172 | 1.94147253 |
| EDF1 | Endothelial Differentiation Related Factor 1 | Protein Coding | 34 | GC09M136862 | 1.941102386 |
| PLCB3 | Phospholipase C Beta 3 | Protein Coding | 44 | GC11P064251 | 1.940294266 |
| CLPX | Caseinolytic Mitochondrial Matrix Peptidase Chaperone Subunit X | Protein Coding | 37 | GC15M065148 | 1.940051556 |
| FRY | FRY Microtubule Binding Protein | Protein Coding | 31 | GC13P031880 | 1.939715385 |
| SRP14 | Signal Recognition Particle 14 | Protein Coding | 32 | GC15M040035 | 1.939342499 |
| RNF114 | Ring Finger Protein 114 | Protein Coding | 35 | GC20P049936 | 1.939195275 |
| B3GALT4 | Beta-1,3-Galactosyltransferase 4 | Protein Coding | 36 | GC06P033277 | 1.939029336 |
| SLIRP | SRA Stem-Loop Interacting RNA Binding Protein | Protein Coding | 30 | GC14P077708 | 1.93882966 |
| PCCA | Propionyl-CoA Carboxylase Subunit Alpha | Protein Coding | 42 | GC13P100089 | 1.938267946 |
| TSPAN12 | Tetraspanin 12 | Protein Coding | 39 | GC07M120787 | 1.937577486 |
| LRRC47 | Leucine Rich Repeat Containing 47 | Protein Coding | 26 | GC01M003778 | 1.937505245 |
| NAV2 | Neuron Navigator 2 | Protein Coding | 35 | GC11P019345 | 1.937197566 |
| THOC6 | THO Complex 6 | Protein Coding | 34 | GC16P003024 | 1.936769605 |
| LAMTOR3 | Late Endosomal/Lysosomal Adaptor, MAPK And MTOR Activator 3 | Protein Coding | 35 | GC04M099878 | 1.936203003 |
| ETF1 | Eukaryotic Translation Termination Factor 1 | Protein Coding | 38 | GC05M138506 | 1.935553432 |
| VARS2 | Valyl-TRNA Synthetase 2, Mitochondrial | Protein Coding | 38 | GC06P080337 | 1.935475588 |
| H2AZ2 | H2A.Z Variant Histone 2 | Protein Coding | 27 | GC07M044829 | 1.934976101 |
| SKA1 | Spindle And Kinetochore Associated Complex Subunit 1 | Protein Coding | 32 | GC18P050374 | 1.934499502 |
| SRSF10 | Serine And Arginine Rich Splicing Factor 10 | Protein Coding | 31 | GC01M023964 | 1.934373379 |
| SIX2 | SIX Homeobox 2 | Protein Coding | 36 | GC02M045005 | 1.933142662 |
| PLAAT3 | Phospholipase A And Acyltransferase 3 | Protein Coding | 31 | GC11M087164 | 1.932719231 |
| KPNA4 | Karyopherin Subunit Alpha 4 | Protein Coding | 37 | GC03M160494 | 1.93241179 |
| DUS3L | Dihydrouridine Synthase 3 Like | Protein Coding | 30 | GC19M005786 | 1.932357788 |
| BVES | Blood Vessel Epicardial Substance | Protein Coding | 37 | GC06M105096 | 1.93214643 |
| LRRC32 | Leucine Rich Repeat Containing 32 | Protein Coding | 37 | GC11M076657 | 1.931958199 |
| AEBP2 | AE Binding Protein 2 | Protein Coding | 33 | GC12P019404 | 1.931832552 |
| ADGRL2 | Adhesion G Protein-Coupled Receptor L2 | Protein Coding | 35 | GC01P081306 | 1.931566238 |
| EFHD2 | EF-Hand Domain Family Member D2 | Protein Coding | 33 | GC01P015409 | 1.931542635 |
| DCAF15 | DDB1 And CUL4 Associated Factor 15 | Protein Coding | 26 | GC19P013952 | 1.931405544 |
| BCL2L10 | BCL2 Like 10 | Protein Coding | 34 | GC15M081678 | 1.930878639 |
| AP4E1 | Adaptor Related Protein Complex 4 Subunit Epsilon 1 | Protein Coding | 33 | GC15P050908 | 1.930703163 |
| LARP4B | La Ribonucleoprotein 4B | Protein Coding | 29 | GC10M000806 | 1.930205941 |
| MAP7 | Microtubule Associated Protein 7 | Protein Coding | 32 | GC06M136342 | 1.930166245 |
| YPEL5 | Yippee Like 5 | Protein Coding | 32 | GC02P030108 | 1.929268241 |
| VPS4B | Vacuolar Protein Sorting 4 Homolog B | Protein Coding | 37 | GC18M063389 | 1.929249525 |
| CTSE | Cathepsin E | Protein Coding | 38 | GC01M206009 | 1.928515911 |
| RPL37A | Ribosomal Protein L37a | Protein Coding | 33 | GC02P216498 | 1.928474188 |
| G0S2 | G0/G1 Switch 2 | Protein Coding | 29 | GC01P209675 | 1.928141117 |
| SMARCD3 | SWI/SNF Related, Matrix Associated, Actin Dependent Regulator Of Chromatin, Subfamily D, Member 3 | Protein Coding | 36 | GC07M151238 | 1.927807212 |
| CD3G | CD3 Gamma Subunit Of T-Cell Receptor Complex | Protein Coding | 42 | GC11P118344 | 1.927749157 |
| TAF10 | TATA-Box Binding Protein Associated Factor 10 | Protein Coding | 34 | GC11M006608 | 1.927293062 |
| ST8SIA6 | ST8 Alpha-N-Acetyl-Neuraminide Alpha-2,8-Sialyltransferase 6 | Protein Coding | 31 | GC10M017315 | 1.926674962 |
| GTSE1 | G2 And S-Phase Expressed 1 | Protein Coding | 34 | GC22P046296 | 1.925705671 |
| TENT5C | Terminal Nucleotidyltransferase 5C | Protein Coding | 27 | GC01P117606 | 1.925359011 |
| FBXW11 | F-Box And WD Repeat Domain Containing 11 | Protein Coding | 39 | GC05M171861 | 1.923592687 |
| MKRN1 | Makorin Ring Finger Protein 1 | Protein Coding | 36 | GC07M140453 | 1.923130155 |
| RSPO3 | R-Spondin 3 | Protein Coding | 37 | GC06P127118 | 1.92305994 |
| EDRF1 | Erythroid Differentiation Regulatory Factor 1 | Protein Coding | 25 | GC10P125719 | 1.922994375 |
| VASH1 | Vasohibin 1 | Protein Coding | 33 | GC14P076761 | 1.922658205 |
| CLN6 | CLN6 Transmembrane ER Protein | Protein Coding | 34 | GC15M068206 | 1.921269655 |

| | | | | | |
|---|---|---|---|---|---|
| ARHGAP8 | Rho GTPase Activating Protein 8 | Protein Coding | 31 | GC22P044752 | 1.921197057 |
| PPT1 | Palmitoyl-Protein Thioesterase 1 | Protein Coding | 42 | GC01M040072 | 1.920995116 |
| SERPINB7 | Serpin Family B Member 7 | Protein Coding | 37 | GC18P063752 | 1.920726299 |
| PRIMA1 | Proline Rich Membrane Anchor 1 | Protein Coding | 30 | GC14M093718 | 1.920659542 |
| LRIG3 | Leucine Rich Repeats And Immunoglobulin Like Domains 3 | Protein Coding | 35 | GC12M058872 | 1.920348167 |
| IL1RL2 | Interleukin 1 Receptor Like 2 | Protein Coding | 38 | GC02P102186 | 1.919724464 |
| RPS27L | Ribosomal Protein S27 Like | Protein Coding | 33 | GC15M081133 | 1.91894722 |
| MEF2A | Myocyte Enhancer Factor 2A | Protein Coding | 42 | GC15P099565 | 1.918694496 |
| NKRF | NFKB Repressing Factor | Protein Coding | 32 | GC0XM119588 | 1.918522239 |
| NOX5 | NADPH Oxidase 5 | Protein Coding | 34 | GC15P114661 | 1.918294907 |
| HYLS1 | HYLS1 Centriolar And Ciliogenesis Associated | Protein Coding | 34 | GC11P125883 | 1.917793632 |
| ERI1 | Exoribonuclease 1 | Protein Coding | 33 | GC08P008892 | 1.917363405 |
| MRPL47 | Mitochondrial Ribosomal Protein L47 | Protein Coding | 30 | GC03M179588 | 1.917285681 |
| TAX1BP3 | Tax1 Binding Protein 3 | Protein Coding | 33 | GC17M004110 | 1.917020321 |
| NANOGP8 | Nanog Homeobox Retrogene P8 | Protein Coding | 22 | GC15M035083 | 1.916433454 |
| LFNG | LFNG O-Fucosylpeptide 3-Beta-N-Acetylglucosaminyltransferase | Protein Coding | 42 | GC07P002512 | 1.916432142 |
| LONRF1 | LON Peptidase N-Terminal Domain And Ring Finger 1 | Protein Coding | 30 | GC08M012721 | 1.916325569 |
| DHX40 | DEAH-Box Helicase 40 | Protein Coding | 31 | GC17P059565 | 1.916246653 |
| PHF14 | PHD Finger Protein 14 | Protein Coding | 28 | GC07P010973 | 1.916038632 |
| PCMT1 | Protein-L-Isoaspartate (D-Aspartate) O-Methyltransferase | Protein Coding | 36 | GC06P149749 | 1.91574955 |
| P2RY12 | Purinergic Receptor P2Y12 | Protein Coding | 43 | GC03M151336 | 1.915572405 |
| RABGAP1L | RAB GTPase Activating Protein 1 Like | Protein Coding | 31 | GC01P174159 | 1.914825439 |
| CD300E | CD300e Molecule | Protein Coding | 31 | GC17M074609 | 1.914557457 |
| VAMP8 | Vesicle Associated Membrane Protein 8 | Protein Coding | 36 | GC02P085561 | 1.913904905 |
| ST2 | Suppression Of Tumorigenicity 2 | Genetic Locus | 7 | GC11U990127 | 1.913520575 |
| PLEKHA7 | Pleckstrin Homology Domain Containing A7 | Protein Coding | 34 | GC11M016778 | 1.91326189 |
| GET4 | Guided Entry Of Tail-Anchored Proteins Factor 4 | Protein Coding | 30 | GC07P000876 | 1.913200855 |
| ARHGEF18 | Rho/Rac Guanine Nucleotide Exchange Factor 18 | Protein Coding | 38 | GC19P007351 | 1.912721872 |
| IGSF21 | Immunoglobin Superfamily Member 21 | Protein Coding | 32 | GC01P018107 | 1.912592411 |
| EHD2 | EH Domain Containing 2 | Protein Coding | 33 | GC19P047713 | 1.912384391 |
| PIAS4 | Protein Inhibitor Of Activated STAT 4 | Protein Coding | 37 | GC19P004007 | 1.911779642 |
| CLINT1 | Clathrin Interactor 1 | Protein Coding | 35 | GC05M157785 | 1.911497355 |
| LGALS13 | Galectin 13 | Protein Coding | 32 | GC19P039602 | 1.911329508 |
| GTF2H3 | General Transcription Factor IIH Subunit 3 | Protein Coding | 34 | GC12P123633 | 1.911243796 |
| BIRC8 | Baculoviral IAP Repeat Containing 8 | Pseudogene | 28 | GC19M053289 | 1.911240816 |
| NRBF2 | Nuclear Receptor Binding Factor 2 | Protein Coding | 32 | GC10P063133 | 1.911235332 |
| ST3GAL2 | ST3 Beta-Galactoside Alpha-2,3-Sialyltransferase 2 | Protein Coding | 35 | GC16M070375 | 1.911051154 |
| NCOA7 | Nuclear Receptor Coactivator 7 | Protein Coding | 32 | GC06P125781 | 1.910694242 |
| RNPC3 | RNA Binding Region (RNP1, RRM) Containing 3 | Protein Coding | 31 | GC01P103525 | 1.910583019 |
| SON | SON DNA And RNA Binding Protein | Protein Coding | 37 | GC21P033542 | 1.910432577 |
| TRD | T Cell Receptor Delta Locus | Protein Coding | 13 | GC14P031658 | 1.910321712 |
| GRID2 | Glutamate Ionotropic Receptor Delta Type Subunit 2 | Protein Coding | 42 | GC04P092304 | 1.910314798 |
| UCN | Urocortin | Protein Coding | 32 | GC02M027308 | 1.909714699 |
| SPATC1L | Spermatogenesis And Centriole Associated 1 Like | Protein Coding | 29 | GC21M050458 | 1.909151196 |
| TRIT1 | TRNA Isopentenyltransferase 1 | Protein Coding | 38 | GC01M039842 | 1.908292294 |
| ASPG | Asparaginase | Protein Coding | 32 | GC14P104085 | 1.908262491 |
| BUD13 | BUD13 Homolog | Protein Coding | 32 | GC11M116749 | 1.90800786 |
| FAF2 | Fas Associated Factor Family Member 2 | Protein Coding | 34 | GC05P176447 | 1.90793848 |
| RPL36A | Ribosomal Protein L36a | Protein Coding | 31 | GC0XP101392 | 1.907913446 |
| TMEM165 | Transmembrane Protein 165 | Protein Coding | 35 | GC04P055395 | 1.907809019 |
| IBTK | Inhibitor Of Bruton Tyrosine Kinase | Protein Coding | 35 | GC06M082169 | 1.907454133 |
| GABRA6 | Gamma-Aminobutyric Acid Type A Receptor Subunit Alpha6 | Protein Coding | 40 | GC05P161547 | 1.907398939 |
| CDCA3 | Cell Division Cycle Associated 3 | Protein Coding | 34 | GC12M006844 | 1.906392574 |
| SLCO4A1 | Solute Carrier Organic Anion Transporter Family Member 4A1 | Protein Coding | 36 | GC20P063303 | 1.905732751 |
| MYL12A | Myosin Light Chain 12A | Protein Coding | 35 | GC18P003238 | 1.905263662 |
| MIR1915 | MicroRNA 1915 | RNA Gene | 16 | GC10M021496 | 1.905259967 |
| PHETA1 | PH Domain Containing Endocytic Trafficking Adaptor 1 | Protein Coding | 27 | GC12M111361 | 1.905114651 |
| TFB1M | Transcription Factor B1, Mitochondrial | Protein Coding | 38 | GC06M155247 | 1.90505743 |
| SPOCK2 | SPARC (Osteonectin), Cwcv And Kazal Like Domains Proteoglycan 2 | Protein Coding | 34 | GC10M072059 | 1.904844642 |
| TIMM50 | Translocase Of Inner Mitochondrial Membrane 50 | Protein Coding | 35 | GC19P039480 | 1.904795527 |
| PBX3 | PBX Homeobox 3 | Protein Coding | 36 | GC09P125747 | 1.904613137 |
| FBLIM1 | Filamin Binding LIM Protein 1 | Protein Coding | 34 | GC01P015756 | 1.904469252 |
| RSL24D1 | Ribosomal L24 Domain Containing 1 | Protein Coding | 33 | GC15M055180 | 1.904456496 |
| IFNG-AS1 | IFNG Antisense RNA 1 | RNA Gene | 17 | GC12P067989 | 1.904037952 |
| P3R3URF-PIK3R3 | P3R3URF-PIK3R3 Readthrough | Protein Coding | 11 | GC01M046237 | 1.90396595 |
| CGGBP1 | CGG Triplet Repeat Binding Protein 1 | Protein Coding | 29 | GC03M088051 | 1.9033916 |
| GPRC6A | G Protein-Coupled Receptor Class C Group 6 Member A | Protein Coding | 35 | GC06M116793 | 1.903281927 |
| PSMD13 | Proteasome 26S Subunit, Non-ATPase 13 | Protein Coding | 35 | GC11P000236 | 1.902832627 |
| GABPB1 | GA Binding Protein Transcription Factor Subunit Beta 1 | Protein Coding | 34 | GC15M050275 | 1.902511001 |
| ATP1B2 | ATPase Na+/K+ Transporting Subunit Beta 2 | Protein Coding | 38 | GC17P010889 | 1.901747942 |
| HHEX | Hematopoietically Expressed Homeobox | Protein Coding | 36 | GC10P092689 | 1.901513457 |
| NXPH1 | Neurexophilin 1 | Protein Coding | 35 | GC07P008440 | 1.901309729 |
| RAD1 | RAD1 Checkpoint DNA Exonuclease | Protein Coding | 32 | GC05M034905 | 1.901279211 |
| SIGLEC7 | Sialic Acid Binding Ig Like Lectin 7 | Protein Coding | 37 | GC19P051142 | 1.901241302 |
| TRAK2 | Trafficking Kinesin Protein 2 | Protein Coding | 36 | GC02M201377 | 1.901102901 |
| UBE2Q2 | Ubiquitin Conjugating Enzyme E2 Q2 | Protein Coding | 36 | GC15P075843 | 1.900870681 |
| GRM4 | Glutamate Metabotropic Receptor 4 | Protein Coding | 40 | GC06M063740 | 1.900756598 |
| DUSP2 | Dual Specificity Phosphatase 2 | Protein Coding | 36 | GC02M098007 | 1.900489569 |
| MIR1258 | MicroRNA 1258 | RNA Gene | 13 | GC02M179860 | 1.900402069 |
| MEPE | Matrix Extracellular Phosphoglycoprotein | Protein Coding | 32 | GC04P087821 | 1.900357962 |
| HEPH | Hephaestin | Protein Coding | 35 | GC0XP066162 | 1.900093317 |
| FXR2 | FMR1 Autosomal Homolog 2 | Protein Coding | 35 | GC17M007716 | 1.900041699 |
| WDR1 | WD Repeat Domain 1 | Protein Coding | 36 | GC04M010075 | 1.900016785 |
| EXTL2 | Exostosin Like Glycosyltransferase 2 | Protein Coding | 35 | GC01M100872 | 1.899783492 |
| ITSN1 | Intersectin 1 | Protein Coding | 36 | GC21P033642 | 1.89949441 |
| ARHGEF10L | Rho Guanine Nucleotide Exchange Factor 10 Like | Protein Coding | 31 | GC01P017761 | 1.899406195 |
| FEZ1 | Fasciculation And Elongation Protein Zeta 1 | Protein Coding | 34 | GC11M125445 | 1.89935565 |
| PTCRA | Pre T Cell Antigen Receptor Alpha | Protein Coding | 34 | GC06P042915 | 1.899102807 |
| GPR55 | G Protein-Coupled Receptor 55 | Protein Coding | 39 | GC02M230907 | 1.899094343 |
| KRT74 | Keratin 74 | Protein Coding | 34 | GC12M052565 | 1.898637891 |
| SRP9 | Signal Recognition Particle 9 | Protein Coding | 31 | GC01P225777 | 1.89819479 |
| CXXC5 | CXXC Finger Protein 5 | Protein Coding | 35 | GC05P139647 | 1.897956848 |
| CORO1A | Coronin 1A | Protein Coding | 39 | GC16P040217 | 1.897621632 |
| WDR18 | WD Repeat Domain 18 | Protein Coding | 30 | GC19P002515 | 1.897020578 |
| TPP2 | Tripeptidyl Peptidase 2 | Protein Coding | 40 | GC13P102596 | 1.896863699 |

| | | | | | |
|---|---|---|---|---|---|
| NGB | Neuroglobin | Protein Coding | 34 | GC14M077265 | 1.89673245 |
| PTTG1IP | PTTG1 Interacting Protein | Protein Coding | 31 | GC21M044849 | 1.895479679 |
| SIVA1 | SIVA1 Apoptosis Inducing Factor | Protein Coding | 32 | GC14P109280 | 1.894568443 |
| MMP25 | Matrix Metallopeptidase 25 | Protein Coding | 38 | GC16P010757 | 1.894430876 |
| MIR944 | MicroRNA 944 | RNA Gene | 17 | GC03P189829 | 1.894263864 |
| CPO | Carboxypeptidase O | Protein Coding | 34 | GC02P206792 | 1.893601894 |
| KIF3A | Kinesin Family Member 3A | Protein Coding | 36 | GC05M132689 | 1.893416405 |
| TBCE | Tubulin Folding Cofactor E | Protein Coding | 35 | GC01P235405 | 1.893310308 |
| CES1 | Carboxylesterase 1 | Protein Coding | 43 | GC16M055836 | 1.892974257 |
| IRF9 | Interferon Regulatory Factor 9 | Protein Coding | 39 | GC14P024161 | 1.892825723 |
| TNFAIP6 | TNF Alpha Induced Protein 6 | Protein Coding | 38 | GC02P151357 | 1.892675996 |
| KMT2E | Lysine Methyltransferase 2E (Inactive) | Protein Coding | 36 | GC07P104982 | 1.891780615 |
| HNRNPUL2 | Heterogeneous Nuclear Ribonucleoprotein U Like 2 | Protein Coding | 29 | GC11M062712 | 1.891682386 |
| TECR | Trans-2,3-Enoyl-CoA Reductase | Protein Coding | 39 | GC19P014504 | 1.891480207 |
| ASF1A | Anti-Silencing Function 1A Histone Chaperone | Protein Coding | 34 | GC06P118894 | 1.891354203 |
| SAMD4B | Sterile Alpha Motif Domain Containing 4B | Protein Coding | 31 | GC19P039342 | 1.891166568 |
| EPC1 | Enhancer Of Polycomb Homolog 1 | Protein Coding | 35 | GC10M032770 | 1.890594959 |
| MEPCE | Methylphosphate Capping Enzyme | Protein Coding | 29 | GC07P100428 | 1.890536785 |
| CCDC68 | Coiled-Coil Domain Containing 68 | Protein Coding | 31 | GC18M054901 | 1.890529513 |
| PDE3B | Phosphodiesterase 3B | Protein Coding | 39 | GC11P014643 | 1.889432192 |
| TPK1 | Thiamin Pyrophosphokinase 1 | Protein Coding | 40 | GC07M144451 | 1.889105916 |
| ENTPD5 | Ectonucleoside Triphosphate Diphosphohydrolase 5 (Inactive) | Protein Coding | 40 | GC14M073958 | 1.888839245 |
| UNC93B1 | Unc-93 Homolog B1, TLR Signaling Regulator | Protein Coding | 35 | GC11M067991 | 1.887477636 |
| SH3GL2 | SH3 Domain Containing GRB2 Like 2, Endophilin A1 | Protein Coding | 38 | GC09P017569 | 1.887251377 |
| LINC02747 | Long Intergenic Non-Protein Coding RNA 2747 | RNA Gene | 9 | GC11M087461 | 1.887052298 |
| TFAP4 | Transcription Factor AP-4 | Protein Coding | 32 | GC16M006869 | 1.88683629 |
| CAMK2A | Calcium/Calmodulin Dependent Protein Kinase II Alpha | Protein Coding | 44 | GC05M150219 | 1.886621356 |
| TBC1D2 | TBC1 Domain Family Member 2 | Protein Coding | 33 | GC09M098198 | 1.886219501 |
| NDUFAB1 | NADH:Ubiquinone Oxidoreductase Subunit AB1 | Protein Coding | 37 | GC16M023582 | 1.886174083 |
| IMPG1 | Interphotoreceptor Matrix Proteoglycan 1 | Protein Coding | 36 | GC06M075921 | 1.88561058 |
| GEMIN5 | Gem Nuclear Organelle Associated Protein 5 | Protein Coding | 32 | GC05M154887 | 1.885607362 |
| RAP2C | RAP2C, Member Of RAS Oncogene Family | Protein Coding | 32 | GC0XM132203 | 1.885551691 |
| LINC00504 | Long Intergenic Non-Protein Coding RNA 504 | RNA Gene | 15 | GC04M014476 | 1.885485649 |
| ZW10 | Zw10 Kinetochore Protein | Protein Coding | 34 | GC11M113733 | 1.88547194 |
| SNX27 | Sorting Nexin 27 | Protein Coding | 34 | GC01P151611 | 1.885175705 |
| GIGYF1 | GRB10 Interacting GYF Protein 1 | Protein Coding | 31 | GC07M100679 | 1.885072827 |
| CEP164 | Centrosomal Protein 164 | Protein Coding | 36 | GC11P117314 | 1.88441062 |
| DNAJB12 | DnaJ Heat Shock Protein Family (Hsp40) Member B12 | Protein Coding | 32 | GC10M072332 | 1.883700728 |
| MRPL20 | Mitochondrial Ribosomal Protein L20 | Protein Coding | 31 | GC01M001401 | 1.883559704 |
| PWP2 | PWP2 Small Subunit Processome Component | Protein Coding | 32 | GC21P044107 | 1.883467197 |
| SSR4 | Signal Sequence Receptor Subunit 4 | Protein Coding | 37 | GC0XP153793 | 1.883335829 |
| RAE1 | Ribonucleic Acid Export 1 | Protein Coding | 36 | GC20P057351 | 1.88261199 |
| HLA-DOB | Major Histocompatibility Complex, Class II, DO Beta | Protein Coding | 36 | GC06M063669 | 1.882160187 |
| MXRA8 | Matrix Remodeling Associated 8 | Protein Coding | 31 | GC01M001352 | 1.882045984 |
| LOC110386947 | CYP19A1 Promoter I.1 | Biological Region | 2 | GC15P051839 | 1.88186729 |
| GZMA | Granzyme A | Protein Coding | 39 | GC05P055102 | 1.881483555 |
| SEC61B | SEC61 Translocon Subunit Beta | Protein Coding | 33 | GC09P099222 | 1.881282806 |
| MIR147A | MicroRNA 147a | RNA Gene | 15 | GC09M120244 | 1.881182432 |
| UBE2M | Ubiquitin Conjugating Enzyme E2 M | Protein Coding | 36 | GC19M058555 | 1.880826712 |
| RBM20 | RNA Binding Motif Protein 20 | Protein Coding | 32 | GC10P110644 | 1.880586147 |
| MRPS21 | Mitochondrial Ribosomal Protein S21 | Protein Coding | 32 | GC01P150294 | 1.880380511 |
| FAM161A | FAM161 Centrosomal Protein A | Protein Coding | 31 | GC02M061792 | 1.880329967 |
| ARNTL2 | Aryl Hydrocarbon Receptor Nuclear Translocator Like 2 | Protein Coding | 34 | GC12P027332 | 1.879806399 |
| STMN2 | Stathmin 2 | Protein Coding | 35 | GC08P079610 | 1.879698157 |
| NCDN | Neurochondrin | Protein Coding | 32 | GC01P035557 | 1.879581451 |
| HLA-DRB3 | Major Histocompatibility Complex, Class II, DR Beta 3 | Protein Coding | 26 | GC06Mn03715 | 1.879537821 |
| GMFG | Glia Maturation Factor Gamma | Protein Coding | 32 | GC19M039328 | 1.87923336 |
| MIR652 | MicroRNA 652 | RNA Gene | 16 | GC0XP110055 | 1.879182339 |
| SELPLG | Selectin P Ligand | Protein Coding | 38 | GC12M108621 | 1.878991485 |
| P2RY1 | Purinergic Receptor P2Y1 | Protein Coding | 42 | GC03P152835 | 1.878919244 |
| S100A5 | S100 Calcium Binding Protein A5 | Protein Coding | 32 | GC01M153537 | 1.878674507 |
| LTV1 | LTV1 Ribosome Biogenesis Factor | Protein Coding | 28 | GC06P143843 | 1.878433943 |
| LOC109286556 | TBX3 Promoter Region | Biological Region | 2 | GC12P114759 | 1.878343821 |
| PLXNA2 | Plexin A2 | Protein Coding | 38 | GC01M208023 | 1.878334284 |
| SUCLG2 | Succinate-CoA Ligase GDP-Forming Subunit Beta | Protein Coding | 37 | GC03M067358 | 1.878163815 |
| MUC5B-AS1 | MUC5B Antisense RNA 1 | RNA Gene | 9 | GC11M001242 | 1.877273202 |
| ELAVL3 | ELAV Like RNA Binding Protein 3 | Protein Coding | 34 | GC19M011451 | 1.877177715 |
| PFDN4 | Prefoldin Subunit 4 | Protein Coding | 34 | GC20P054207 | 1.876716375 |
| CNOT1 | CCR4-NOT Transcription Complex Subunit 1 | Protein Coding | 36 | GC16M058519 | 1.876134872 |
| NRXN3 | Neurexin 3 | Protein Coding | 38 | GC14P077980 | 1.875659943 |
| SIX3 | SIX Homeobox 3 | Protein Coding | 39 | GC02P044941 | 1.874128342 |
| PLD3 | Phospholipase D Family Member 3 | Protein Coding | 38 | GC19P040348 | 1.87391603 |
| SRP68 | Signal Recognition Particle 68 | Protein Coding | 34 | GC17M076038 | 1.871879101 |
| MIR1972-2 | MicroRNA 1972-2 | RNA Gene | 12 | GC16P070030 | 1.871758699 |
| HORMAD2 | HORMA Domain Containing 2 | Protein Coding | 31 | GC22P030080 | 1.871545076 |
| SLC25A43 | Solute Carrier Family 25 Member 43 | Protein Coding | 31 | GC0XP119399 | 1.871474743 |
| ADGRL3 | Adhesion G Protein-Coupled Receptor L3 | Protein Coding | 32 | GC04P061201 | 1.871444464 |
| LARS1 | Leucyl-TRNA Synthetase 1 | Protein Coding | 36 | GC05M146114 | 1.870590448 |
| MEF2B | Myocyte Enhancer Factor 2B | Protein Coding | 32 | GC19M019150 | 1.870413542 |
| TMED10 | Transmembrane P24 Trafficking Protein 10 | Protein Coding | 36 | GC14M075132 | 1.870161772 |
| RGMB | Repulsive Guidance Molecule BMP Co-Receptor B | Protein Coding | 35 | GC05P098768 | 1.870060921 |
| MYO9A | Myosin IXA | Protein Coding | 35 | GC15M071822 | 1.869972944 |
| COPE | COPI Coat Complex Subunit Epsilon | Protein Coding | 35 | GC19M018899 | 1.869526148 |
| SCAMP2 | Secretory Carrier Membrane Protein 2 | Protein Coding | 34 | GC15M074843 | 1.869063616 |
| MIR298 | MicroRNA 298 | RNA Gene | 16 | GC20M058818 | 1.868693113 |
| UCHL5 | Ubiquitin C-Terminal Hydrolase L5 | Protein Coding | 37 | GC01M193012 | 1.868633509 |
| ICAM3 | Intercellular Adhesion Molecule 3 | Protein Coding | 38 | GC19M010396 | 1.868221521 |
| CALML5 | Calmodulin Like 5 | Protein Coding | 31 | GC10M005498 | 1.867572427 |
| PRAM1 | PML-RARA Regulated Adaptor Molecule 1 | Protein Coding | 30 | GC19M008490 | 1.867427111 |
| DMKN | Dermokine | Protein Coding | 31 | GC19M064575 | 1.867422104 |
| P2RX3 | Purinergic Receptor P2X 3 | Protein Coding | 38 | GC11P057356 | 1.867165208 |
| ASIC1 | Acid Sensing Ion Channel Subunit 1 | Protein Coding | 39 | GC12P050057 | 1.86659646 |
| CNKSR1 | Connector Enhancer Of Kinase Suppressor Of Ras 1 | Protein Coding | 33 | GC01P026178 | 1.865861893 |
| GJA3 | Gap Junction Protein Alpha 3 | Protein Coding | 38 | GC13M020334 | 1.865801573 |
| THOC2 | THO Complex 2 | Protein Coding | 35 | GC0XM123600 | 1.86484623 |

| | | | | | |
|---|---|---|---|---|---|
| PATJ | PATJ Crumbs Cell Polarity Complex Component | Protein Coding | 30 | GC01P061743 | 1.864745378 |
| CCL14 | C-C Motif Chemokine Ligand 14 | Protein Coding | 31 | GC17M041176 | 1.864585638 |
| UBE4A | Ubiquitination Factor E4A | Protein Coding | 36 | GC11P118359 | 1.864547729 |
| IPO4 | Importin 4 | Protein Coding | 32 | GC14M024181 | 1.86454463 |
| MAG | Myelin Associated Glycoprotein | Protein Coding | 42 | GC19P035292 | 1.864522219 |
| SRGN | Serglycin | Protein Coding | 35 | GC10P069088 | 1.864065289 |
| LSM14A | LSM14A MRNA Processing Body Assembly Factor | Protein Coding | 31 | GC19P034172 | 1.864017844 |
| TNNI3K | TNNI3 Interacting Kinase | Protein Coding | 36 | GC01P074235 | 1.863862872 |
| LOC105371267 | Uncharacterized LOC105371267 | Protein Coding | 10 | GC16M053036 | 1.863701344 |
| ARSG | Arylsulfatase G | Protein Coding | 39 | GC17P068259 | 1.863270402 |
| IPO8 | Importin 8 | Protein Coding | 35 | GC12M030628 | 1.863153458 |
| RAP2B | RAP2B, Member Of RAS Oncogene Family | Protein Coding | 33 | GC03P153162 | 1.863057017 |
| CYB5D2 | Cytochrome B5 Domain Containing 2 | Protein Coding | 29 | GC17P004143 | 1.862805128 |
| TOMM40 | Translocase Of Outer Mitochondrial Membrane 40 | Protein Coding | 36 | GC19P044890 | 1.862123609 |
| TOM1 | Target Of Myb1 Membrane Trafficking Protein | Protein Coding | 39 | GC22P035299 | 1.859910011 |
| H2BC3 | H2B Clustered Histone 3 | Protein Coding | 27 | GC06M026044 | 1.859699488 |
| ICMT | Isoprenylcysteine Carboxyl Methyltransferase | Protein Coding | 38 | GC01M006222 | 1.85932827 |
| ZBTB48 | Zinc Finger And BTB Domain Containing 48 | Protein Coding | 31 | GC01P006579 | 1.85844028 |
| INSIG2 | Insulin Induced Gene 2 | Protein Coding | 35 | GC02P118088 | 1.858305097 |
| STK31 | Serine/Threonine Kinase 31 | Protein Coding | 31 | GC07P023710 | 1.857967257 |
| NDRG4 | NDRG Family Member 4 | Protein Coding | 35 | GC16P058462 | 1.857402563 |
| UTF1 | Undifferentiated Embryonic Cell Transcription Factor 1 | Protein Coding | 29 | GC10P133230 | 1.857095122 |
| NOL3 | Nucleolar Protein 3 | Protein Coding | 39 | GC16P067205 | 1.856642246 |
| PPP1R2 | Protein Phosphatase 1 Regulatory Inhibitor Subunit 2 | Protein Coding | 35 | GC03M195515 | 1.855751038 |
| HOXD9 | Homeobox D9 | Protein Coding | 36 | GC02P176122 | 1.85532546 |
| STK32C | Serine/Threonine Kinase 32C | Protein Coding | 33 | GC10M132207 | 1.854959249 |
| IL22RA2 | Interleukin 22 Receptor Subunit Alpha 2 | Protein Coding | 36 | GC06M137143 | 1.85474658 |
| RNF40 | Ring Finger Protein 40 | Protein Coding | 35 | GC16P040277 | 1.854610205 |
| CALCOCO1 | Calcium Binding And Coiled-Coil Domain 1 | Protein Coding | 32 | GC12M053709 | 1.854554534 |
| DUSP10 | Dual Specificity Phosphatase 10 | Protein Coding | 38 | GC01M221701 | 1.854009867 |
| IGBP1 | Immunoglobulin Binding Protein 1 | Protein Coding | 38 | GC0XP070133 | 1.853864431 |
| MARK2 | Microtubule Affinity Regulating Kinase 2 | Protein Coding | 39 | GC11P063838 | 1.853625298 |
| GRSF1 | G-Rich RNA Sequence Binding Factor 1 | Protein Coding | 35 | GC04M070815 | 1.852550745 |
| FCER1A | Fc Epsilon Receptor Ia | Protein Coding | 38 | GC01P159259 | 1.852328777 |
| APLP2 | Amyloid Beta Precursor Like Protein 2 | Protein Coding | 38 | GC11P130069 | 1.852302313 |
| MAN2A1 | Mannosidase Alpha Class 2A Member 1 | Protein Coding | 39 | GC05P109689 | 1.85040307 |
| SRRM2 | Serine/Arginine Repetitive Matrix 2 | Protein Coding | 31 | GC16P010748 | 1.849938869 |
| WDR62 | WD Repeat Domain 62 | Protein Coding | 33 | GC19P036054 | 1.849254847 |
| ITPA | Inosine Triphosphatase | Protein Coding | 41 | GC20P003985 | 1.84910512 |
| PGAP2 | Post-GPI Attachment To Proteins 2 | Protein Coding | 31 | GC11P003797 | 1.848382235 |
| ADCY7 | Adenylate Cyclase 7 | Protein Coding | 40 | GC16P050514 | 1.848152995 |
| PFKFB2 | 6-Phosphofructo-2-Kinase/Fructose-2,6-Biphosphatase 2 | Protein Coding | 36 | GC01P207034 | 1.848016381 |
| MANBAL | Mannosidase Beta Like | Protein Coding | 28 | GC20P037289 | 1.847684741 |
| ARMCX2 | Armadillo Repeat Containing X-Linked 2 | Protein Coding | 30 | GC0XM101658 | 1.847376823 |
| ARHGAP10 | Rho GTPase Activating Protein 10 | Protein Coding | 34 | GC04P147732 | 1.846395731 |
| MRPS27 | Mitochondrial Ribosomal Protein S27 | Protein Coding | 32 | GC05M072219 | 1.846237063 |
| SUMO3 | Small Ubiquitin Like Modifier 3 | Protein Coding | 36 | GC21M044805 | 1.846099257 |
| NR2F6 | Nuclear Receptor Subfamily 2 Group F Member 6 | Protein Coding | 35 | GC19M017231 | 1.84566462 |
| H2BC12L | H2B Clustered Histone 12 Like | Protein Coding | 23 | GC21P043569 | 1.84508729 |
| TICRR | TOPBP1 Interacting Checkpoint And Replication Regulator | Protein Coding | 27 | GC15P089575 | 1.844330549 |
| COMTD1 | Catechol-O-Methyltransferase Domain Containing 1 | Protein Coding | 34 | GC10M075233 | 1.843862534 |
| GNA15 | G Protein Subunit Alpha 15 | Protein Coding | 36 | GC19P003209 | 1.843681335 |
| EPN1 | Epsin 1 | Protein Coding | 36 | GC19P064378 | 1.843242407 |
| PAG1 | Phosphoprotein Membrane Anchor With Glycosphingolipid Microdomains 1 | Protein Coding | 35 | GC08M080967 | 1.843209982 |
| IFT46 | Intraflagellar Transport 46 | Protein Coding | 28 | GC11M118544 | 1.843106747 |
| MED6 | Mediator Complex Subunit 6 | Protein Coding | 34 | GC14M070581 | 1.842981815 |
| PPM1H | Protein Phosphatase, Mg2+/Mn2+ Dependent 1H | Protein Coding | 34 | GC12M062643 | 1.84256649 |
| EXD2 | Exonuclease 3'-5' Domain Containing 2 | Protein Coding | 28 | GC14P069191 | 1.842328429 |
| TXLNA | Taxilin Alpha | Protein Coding | 32 | GC01P032179 | 1.84232688 |
| INSRR | Insulin Receptor Related Receptor | Protein Coding | 38 | GC01M156840 | 1.841492653 |
| EIF1AD | Eukaryotic Translation Initiation Factor 1A Domain Containing | Protein Coding | 28 | GC11M065996 | 1.84105444 |
| RHBDD3 | Rhomboid Domain Containing 3 | Protein Coding | 30 | GC22M029259 | 1.841037273 |
| PIAS2 | Protein Inhibitor Of Activated STAT 2 | Protein Coding | 38 | GC18M046808 | 1.840727568 |
| OPA3 | Outer Mitochondrial Membrane Lipid Metabolism Regulator OPA3 | Protein Coding | 35 | GC19M045527 | 1.840707302 |
| MTHFS | Methenyltetrahydrofolate Synthetase | Protein Coding | 39 | GC15M079833 | 1.840685606 |
| APLNR | Apelin Receptor | Protein Coding | 38 | GC11M057233 | 1.840376258 |
| GALNT1 | Polypeptide N-Acetylgalactosaminyltransferase 1 | Protein Coding | 35 | GC18P035581 | 1.840107918 |
| FRA7G | Fragile Site, Aphidicolin Type, Common, Fra(7)(Q31.2) | Uncategorized | 4 | GC07U990033 | 1.840052128 |
| SLC45A1 | Solute Carrier Family 45 Member 1 | Protein Coding | 30 | GC01P008317 | 1.840037584 |
| NFS1 | NFS1 Cysteine Desulfurase | Protein Coding | 41 | GC20M035668 | 1.839343309 |
| ARIH2 | Ariadne RBR E3 Ubiquitin Protein Ligase 2 | Protein Coding | 35 | GC03P048918 | 1.838775396 |
| WDR20 | WD Repeat Domain 20 | Protein Coding | 30 | GC14P109255 | 1.838564277 |
| SLC6A19 | Solute Carrier Family 6 Member 19 | Protein Coding | 40 | GC05P001201 | 1.83850646 |
| CDH19 | Cadherin 19 | Protein Coding | 35 | GC18M066501 | 1.838070631 |
| TMEM240 | Transmembrane Protein 240 | Protein Coding | 28 | GC01M005547 | 1.837829232 |
| GTF2E1 | General Transcription Factor IIE Subunit 1 | Protein Coding | 35 | GC03P120775 | 1.837755919 |
| ASCC2 | Activating Signal Cointegrator 1 Complex Subunit 2 | Protein Coding | 32 | GC22M029788 | 1.837713003 |
| PLRG1 | Pleiotropic Regulator 1 | Protein Coding | 34 | GC04M154534 | 1.837678671 |
| SORD | Sorbitol Dehydrogenase | Protein Coding | 40 | GC15P045023 | 1.837144136 |
| WDHD1 | WD Repeat And HMG-Box DNA Binding Protein 1 | Protein Coding | 32 | GC14M054938 | 1.836814046 |
| NUDCD1 | NudC Domain Containing 1 | Protein Coding | 30 | GC08M109240 | 1.836708546 |
| DZANK1 | Double Zinc Ribbon And Ankyrin Repeat Domains 1 | Protein Coding | 26 | GC20M018383 | 1.836588144 |
| TTC28 | Tetratricopeptide Repeat Domain 28 | Protein Coding | 29 | GC22M027978 | 1.836550951 |
| UAP1 | UDP-N-Acetylglucosamine Pyrophosphorylase 1 | Protein Coding | 38 | GC01P162561 | 1.835132957 |
| APOBEC3C | Apolipoprotein B MRNA Editing Enzyme Catalytic Subunit 3C | Protein Coding | 32 | GC22P039014 | 1.834916115 |
| HAS3 | Hyaluronan Synthase 3 | Protein Coding | 38 | GC16P069105 | 1.834444523 |
| RBM15B | RNA Binding Motif Protein 15B | Protein Coding | 28 | GC03P051391 | 1.834259748 |
| MIR105-1 | MicroRNA 105-1 | RNA Gene | 14 | GC0XM152392 | 1.834028244 |
| AQP7 | Aquaporin 7 | Protein Coding | 39 | GC09M033384 | 1.833453417 |
| MSBP1 | Minisatellite Binding Protein 1 | Protein Coding | 5 | GC00U990213 | 1.833133221 |
| GLRX | Glutaredoxin | Protein Coding | 39 | GC05M095752 | 1.832973242 |
| ZNHIT1 | Zinc Finger HIT-Type Containing 1 | Protein Coding | 31 | GC07P101218 | 1.832953572 |
| UCMA | Upper Zone Of Growth Plate And Cartilage Matrix Associated | Protein Coding | 31 | GC10M013222 | 1.832290053 |
| IFT88 | Intraflagellar Transport 88 | Protein Coding | 36 | GC13P020566 | 1.83208859 |
| B4GALNT1 | Beta-1,4-N-Acetyl-Galactosaminyltransferase 1 | Protein Coding | 41 | GC12M057623 | 1.83186841 |

| | | | | | |
|---|---|---|---|---|---|
| CDYL | Chromodomain Y Like | Protein Coding | 36 | GC06P004706 | 1.83182025 |
| SEC62 | SEC62 Homolog, Preprotein Translocation Factor | Protein Coding | 31 | GC03P169966 | 1.831778288 |
| PDHX | Pyruvate Dehydrogenase Complex Component X | Protein Coding | 42 | GC11P034894 | 1.831090689 |
| PKNOX2 | PBX/Knotted 1 Homeobox 2 | Protein Coding | 32 | GC11P125164 | 1.83083725 |
| INO80D | INO80 Complex Subunit D | Protein Coding | 30 | GC02M205993 | 1.830369115 |
| SLC12A6 | Solute Carrier Family 12 Member 6 | Protein Coding | 44 | GC15M034229 | 1.828916788 |
| ARHGEF9 | Cdc42 Guanine Nucleotide Exchange Factor 9 | Protein Coding | 36 | GC0XM063634 | 1.828723788 |
| DMXL2 | Dmx Like 2 | Protein Coding | 33 | GC15M051447 | 1.82843554 |
| ATP5IF1 | ATP Synthase Inhibitory Factor Subunit 1 | Protein Coding | 29 | GC01P028237 | 1.827890873 |
| DNMBP | Dynamin Binding Protein | Protein Coding | 35 | GC10M099875 | 1.827652574 |
| EIF4ENIF1 | Eukaryotic Translation Initiation Factor 4E Nuclear Import Factor 1 | Protein Coding | 33 | GC22M036215 | 1.827588081 |
| LRRC8A | Leucine Rich Repeat Containing 8 VRAC Subunit A | Protein Coding | 37 | GC09P128882 | 1.82750237 |
| AAMP | Angio Associated Migratory Cell Protein | Protein Coding | 32 | GC02M218264 | 1.827375889 |
| DGAT1 | Diacylglycerol O-Acyltransferase 1 | Protein Coding | 42 | GC08M144316 | 1.826257229 |
| MKNK2 | MAPK Interacting Serine/Threonine Kinase 2 | Protein Coding | 38 | GC19M002037 | 1.826159239 |
| PNISR | PNN Interacting Serine And Arginine Rich Protein | Protein Coding | 28 | GC06M099398 | 1.825726986 |
| NPEPPS | Aminopeptidase Puromycin Sensitive | Protein Coding | 39 | GC17P047522 | 1.82554841 |
| NFIA | Nuclear Factor I A | Protein Coding | 40 | GC01P060865 | 1.825523376 |
| ALDOC | Aldolase, Fructose-Bisphosphate C | Protein Coding | 34 | GC17M034576 | 1.825497866 |
| LINC01512 | Long Intergenic Non-Protein Coding RNA 1512 | RNA Gene | 13 | GC06P080604 | 1.825078011 |
| SSR3 | Signal Sequence Receptor Subunit 3 | Protein Coding | 33 | GC03M156540 | 1.824516773 |
| SLC31A1 | Solute Carrier Family 31 Member 1 | Protein Coding | 38 | GC09P113221 | 1.824418545 |
| WBP11 | WW Domain Binding Protein 11 | Protein Coding | 32 | GC12M014784 | 1.824232578 |
| TSSK6 | Testis Specific Serine Kinase 6 | Protein Coding | 32 | GC19M019514 | 1.822795868 |
| TBC1D13 | TBC1 Domain Family Member 13 | Protein Coding | 30 | GC09P128787 | 1.822152376 |
| SELENOW | Selenoprotein W | Protein Coding | 28 | GC19P063963 | 1.822138309 |
| PARP14 | Poly(ADP-Ribose) Polymerase Family Member 14 | Protein Coding | 32 | GC03P122680 | 1.822113514 |
| HSDL2 | Hydroxysteroid Dehydrogenase Like 2 | Protein Coding | 31 | GC09P112379 | 1.821128607 |
| IFITM4P | Interferon Induced Transmembrane Protein 4 Pseudogene | Pseudogene | 11 | GC06M063490 | 1.821071148 |
| UBQLN4 | Ubiquilin 4 | Protein Coding | 35 | GC01M156033 | 1.820980072 |
| LIN7C | Lin-7 Homolog C, Crumbs Cell Polarity Complex Component | Protein Coding | 34 | GC11M027494 | 1.820554733 |
| TEFM | Transcription Elongation Factor, Mitochondrial | Protein Coding | 30 | GC17M030897 | 1.820311785 |
| CST7 | Cystatin F | Protein Coding | 34 | GC20P024949 | 1.819731236 |
| EIF2S3 | Eukaryotic Translation Initiation Factor 2 Subunit Gamma | Protein Coding | 40 | GC0XP024054 | 1.819355011 |
| GTPBP1 | GTP Binding Protein 1 | Protein Coding | 35 | GC22P038705 | 1.818955302 |
| JARID2 | Jumonji And AT-Rich Interaction Domain Containing 2 | Protein Coding | 39 | GC06P015405 | 1.818244934 |
| MIR4423 | MicroRNA 4423 | RNA Gene | 14 | GC01P085133 | 1.81819582 |
| ADAMTS5 | ADAM Metallopeptidase With Thrombospondin Type 1 Motif 5 | Protein Coding | 40 | GC21M026918 | 1.817955852 |
| NUDT21 | Nudix Hydrolase 21 | Protein Coding | 32 | GC16M056429 | 1.817880034 |
| OXSR1 | Oxidative Stress Responsive Kinase 1 | Protein Coding | 36 | GC03P038183 | 1.817466497 |
| CCL16 | C-C Motif Chemokine Ligand 16 | Protein Coding | 32 | GC17M035976 | 1.817442417 |
| NDUFS3 | NADH:Ubiquinone Oxidoreductase Core Subunit S3 | Protein Coding | 42 | GC11P047567 | 1.817230821 |
| NPTN | Neuroplastin | Protein Coding | 36 | GC15M073560 | 1.816313505 |
| HAUS8 | HAUS Augmin Like Complex Subunit 8 | Protein Coding | 30 | GC19M017049 | 1.815859556 |
| AUP1 | AUP1 Lipid Droplet Regulating VLDL Assembly Factor | Protein Coding | 33 | GC02M074526 | 1.815650225 |
| DNAH2 | Dynein Axonemal Heavy Chain 2 | Protein Coding | 32 | GC17P007717 | 1.815215588 |
| HOGA1 | 4-Hydroxy-2-Oxoglutarate Aldolase 1 | Protein Coding | 34 | GC10P097585 | 1.815148592 |
| ZC3HC1 | Zinc Finger C3HC-Type Containing 1 | Protein Coding | 34 | GC07M130026 | 1.814753771 |
| RPL39L | Ribosomal Protein L39 Like | Protein Coding | 30 | GC03M187121 | 1.814529419 |
| ANKHD1 | Ankyrin Repeat And KH Domain Containing 1 | Protein Coding | 31 | GC05P145794 | 1.814492226 |
| LINC00662 | Long Intergenic Non-Protein Coding RNA 662 | RNA Gene | 13 | GC19M027663 | 1.814419985 |
| PLEKHA6 | Pleckstrin Homology Domain Containing A6 | Protein Coding | 34 | GC01M204218 | 1.814252615 |
| CEP170 | Centrosomal Protein 170 | Protein Coding | 35 | GC01M243124 | 1.813707113 |
| PRICKLE1 | Prickle Planar Cell Polarity Protein 1 | Protein Coding | 39 | GC12M042456 | 1.813653469 |
| GLIS2 | GLIS Family Zinc Finger 2 | Protein Coding | 36 | GC16P010806 | 1.813621283 |
| MIR203B | MicroRNA 203b | RNA Gene | 12 | GC14M104118 | 1.812949777 |
| CLMN | Calmin | Protein Coding | 32 | GC14M095181 | 1.812924743 |
| CORO6 | Coronin 6 | Protein Coding | 30 | GC17M029614 | 1.812852383 |
| RAPGEF3 | Rap Guanine Nucleotide Exchange Factor 3 | Protein Coding | 40 | GC12M047736 | 1.812521458 |
| GRIA1 | Glutamate Ionotropic Receptor AMPA Type Subunit 1 | Protein Coding | 44 | GC05P153467 | 1.812262297 |
| CCDC50 | Coiled-Coil Domain Containing 50 | Protein Coding | 35 | GC03P191329 | 1.811913729 |
| CENPJ | Centromere Protein J | Protein Coding | 38 | GC13M024882 | 1.811816454 |
| PUS1 | Pseudouridine Synthase 1 | Protein Coding | 39 | GC12P131929 | 1.811782122 |
| CDC123 | Cell Division Cycle 123 | Protein Coding | 32 | GC10P012195 | 1.811769366 |
| TRIM47 | Tripartite Motif Containing 47 | Protein Coding | 30 | GC17M075874 | 1.811620235 |
| EDC4 | Enhancer Of MRNA Decapping 4 | Protein Coding | 34 | GC16P067873 | 1.810780168 |
| CDKL2 | Cyclin Dependent Kinase Like 2 | Protein Coding | 36 | GC04M075576 | 1.810404539 |
| CCNY | Cyclin Y | Protein Coding | 34 | GC10P035254 | 1.809536457 |
| FOXD3-AS1 | FOXD3 Antisense RNA 1 | RNA Gene | 14 | GC01M063320 | 1.809345245 |
| EXTL3 | Exostosin Like Glycosyltransferase 3 | Protein Coding | 40 | GC08P028617 | 1.809083939 |
| DUSP9 | Dual Specificity Phosphatase 9 | Protein Coding | 36 | GC0XP153642 | 1.809010506 |
| CLPB | Caseinolytic Mitochondrial Matrix Peptidase Chaperone Subunit B | Protein Coding | 40 | GC11M087530 | 1.808981061 |
| UNC5B-AS1 | UNC5B Antisense RNA 1 | RNA Gene | 14 | GC10M071217 | 1.808849216 |
| EPM2AIP1 | EPM2A Interacting Protein 1 | Protein Coding | 31 | GC03M037073 | 1.808791041 |
| DEFA5 | Defensin Alpha 5 | Protein Coding | 34 | GC08M007057 | 1.808612227 |
| TBRG4 | Transforming Growth Factor Beta Regulator 4 | Protein Coding | 29 | GC07M045100 | 1.807942629 |
| CHIAP2 | Chitinase, Acidic Pseudogene 2 | Pseudogene | 12 | GC01P111280 | 1.807653785 |
| ATAD1 | ATPase Family AAA Domain Containing 1 | Protein Coding | 37 | GC10M087751 | 1.807546258 |
| CEP41 | Centrosomal Protein 41 | Protein Coding | 35 | GC07M130393 | 1.807334185 |
| POLRMT | RNA Polymerase Mitochondrial | Protein Coding | 39 | GC19M000617 | 1.807107925 |
| PTAFR | Platelet Activating Factor Receptor | Protein Coding | 40 | GC01M028147 | 1.806872487 |
| PIKFYVE | Phosphoinositide Kinase, FYVE-Type Zinc Finger Containing | Protein Coding | 43 | GC02P208266 | 1.806413174 |
| DGUOK | Deoxyguanosine Kinase | Protein Coding | 39 | GC02P073926 | 1.805737257 |
| L1TD1 | LINE1 Type Transposase Domain Containing 1 | Protein Coding | 31 | GC01P062194 | 1.805706501 |
| ZHX3 | Zinc Fingers And Homeoboxes 3 | Protein Coding | 31 | GC20M041178 | 1.805375576 |
| DPYSL3 | Dihydropyrimidinase Like 3 | Protein Coding | 36 | GC05M147390 | 1.80535078 |
| MIR802 | MicroRNA 802 | RNA Gene | 17 | GC21P035720 | 1.805238962 |
| FUT5 | Fucosyltransferase 5 | Protein Coding | 33 | GC19M005865 | 1.8049227 |
| UBA5 | Ubiquitin Like Modifier Activating Enzyme 5 | Protein Coding | 36 | GC03P132654 | 1.804587722 |
| RPP25 | Ribonuclease P And MRP Subunit P25 | Protein Coding | 30 | GC15M074954 | 1.804546118 |
| AKAP10 | A-Kinase Anchoring Protein 10 | Protein Coding | 35 | GC17M019904 | 1.804187059 |
| TIPIN | TIMELESS Interacting Protein | Protein Coding | 34 | GC15M081212 | 1.804101944 |
| CRTC2 | CREB Regulated Transcription Coactivator 2 | Protein Coding | 36 | GC01M153947 | 1.803864598 |
| DUSP14 | Dual Specificity Phosphatase 14 | Protein Coding | 33 | GC17P052865 | 1.803694248 |
| ELMO2 | Engulfment And Cell Motility 2 | Protein Coding | 37 | GC20M046366 | 1.803612232 |

| | | | | | |
|---|---|---|---|---|---|
| OSBPL5 | Oxysterol Binding Protein Like 5 | Protein Coding | 35 | GC11M003093 | 1.803565979 |
| MYLK3 | Myosin Light Chain Kinase 3 | Protein Coding | 37 | GC16M046978 | 1.80239892 |
| KIR3DL2 | Killer Cell Immunoglobulin Like Receptor, Three Ig Domains And Long Cytoplasmic Tail 2 | Protein Coding | 36 | GC19P064347 | 1.80173099 |
| NEURL4 | Neuralized E3 Ubiquitin Protein Ligase 4 | Protein Coding | 28 | GC17M007315 | 1.80140388 |
| SCYL2 | SCY1 Like Pseudokinase 2 | Protein Coding | 34 | GC12P100267 | 1.800956964 |
| EXOSC5 | Exosome Component 5 | Protein Coding | 35 | GC19M063823 | 1.80078125 |
| KDM1B | Lysine Demethylase 1B | Protein Coding | 33 | GC06P018156 | 1.800739527 |
| ATP6V1E1 | ATPase H+ Transporting V1 Subunit E1 | Protein Coding | 39 | GC22M017592 | 1.800100803 |
| ADRA2C | Adrenoceptor Alpha 2C | Protein Coding | 40 | GC04P003766 | 1.799384713 |
| SF3A2 | Splicing Factor 3a Subunit 2 | Protein Coding | 32 | GC19P002236 | 1.79793644 |
| ACADVL | Acyl-CoA Dehydrogenase Very Long Chain | Protein Coding | 40 | GC17P007219 | 1.79765296 |
| IGF2BP2-AS1 | IGF2BP2 Antisense RNA 1 | RNA Gene | 18 | GC03P185712 | 1.797613621 |
| BCRP3 | BCR Pseudogene 3 | Pseudogene | 14 | GC22P024632 | 1.797613621 |
| BMS1P20 | BMS1 Pseudogene 20 | Pseudogene | 10 | GC22P022298 | 1.797613621 |
| TDRKH | Tudor And KH Domain Containing | Protein Coding | 35 | GC01M151865 | 1.7975564 |
| AS3MT | Arsenite Methyltransferase | Protein Coding | 37 | GC10P102869 | 1.79745388 |
| PNCK | Pregnancy Up-Regulated Nonubiquitous CaM Kinase | Protein Coding | 34 | GC0XM153669 | 1.797372341 |
| PRORP | Protein Only RNase P Catalytic Subunit | Protein Coding | 28 | GC14P035337 | 1.797211051 |
| RHPN1-AS1 | RHPN1 Antisense RNA 1 (Head To Head) | RNA Gene | 17 | GC08M143366 | 1.796870708 |
| TNP1 | Transition Protein 1 | Protein Coding | 33 | GC02M216859 | 1.79651618 |
| THOC7 | THO Complex 7 | Protein Coding | 31 | GC03M063833 | 1.796344995 |
| RASSF10 | Ras Association Domain Family Member 10 | Protein Coding | 27 | GC11P012990 | 1.796124697 |
| PCNX1 | Pecanex 1 | Protein Coding | 27 | GC14P070908 | 1.796063662 |
| SRRM1 | Serine And Arginine Repetitive Matrix 1 | Protein Coding | 33 | GC01P024631 | 1.796059847 |
| CSTF3 | Cleavage Stimulation Factor Subunit 3 | Protein Coding | 32 | GC11M033077 | 1.795927525 |
| OLFM2 | Olfactomedin 2 | Protein Coding | 35 | GC19M009853 | 1.795916915 |
| LINC01087 | Long Intergenic Non-Protein Coding RNA 1087 | RNA Gene | 10 | GC02P136504 | 1.795820594 |
| AP2S1 | Adaptor Related Protein Complex 2 Subunit Sigma 1 | Protein Coding | 37 | GC19M046838 | 1.795143843 |
| AMMECR1 | AMMECR Nuclear Protein 1 | Protein Coding | 35 | GC0XM110194 | 1.794917703 |
| TUBGCP6 | Tubulin Gamma Complex Associated Protein 6 | Protein Coding | 36 | GC22M050217 | 1.794729948 |
| NCAPH | Non-SMC Condensin I Complex Subunit H | Protein Coding | 35 | GC02P096365 | 1.794103265 |
| PPIH | Peptidylprolyl Isomerase H | Protein Coding | 35 | GC01P042657 | 1.794096947 |
| ACTR1A | Actin Related Protein 1A | Protein Coding | 35 | GC10M102479 | 1.793533802 |
| CTAGE4 | CTAGE Family Member 4 | Protein Coding | 22 | GC07P144183 | 1.792904854 |
| POLR2H | RNA Polymerase II, I And III Subunit H | Protein Coding | 34 | GC03P184361 | 1.792892456 |
| SLC26A10 | Solute Carrier Family 26 Member 10 | Pseudogene | 29 | GC12P057619 | 1.792641401 |
| STRIP1 | Striatin Interacting Protein 1 | Protein Coding | 30 | GC01P110032 | 1.792235732 |
| WDR49 | WD Repeat Domain 49 | Protein Coding | 28 | GC03M167478 | 1.792226315 |
| PEX12 | Peroxisomal Biogenesis Factor 12 | Protein Coding | 36 | GC17M035574 | 1.791916728 |
| SRFBP1 | Serum Response Factor Binding Protein 1 | Protein Coding | 29 | GC05P121961 | 1.791915655 |
| LINC01638 | Long Intergenic Non-Protein Coding RNA 1638 | RNA Gene | 11 | GC22M036032 | 1.791657209 |
| WDR46 | WD Repeat Domain 46 | Protein Coding | 31 | GC06M063705 | 1.791399956 |
| FIS1 | Fission, Mitochondrial 1 | Protein Coding | 34 | GC07M101239 | 1.791152239 |
| SLC1A3 | Solute Carrier Family 1 Member 3 | Protein Coding | 44 | GC05P036643 | 1.791093826 |
| CARMIL3 | Capping Protein Regulator And Myosin 1 Linker 3 | Protein Coding | 25 | GC14P024052 | 1.790966511 |
| LIMCH1 | LIM And Calponin Homology Domains 1 | Protein Coding | 32 | GC04P041362 | 1.790252686 |
| ENSG00000285800 | Novel Transcript | RNA Gene | 7 | GC16P052548 | 1.790035725 |
| lnc-CHD9-4 | | RNA Gene | 5 | GC16P052547 | 1.790035725 |
| LOC105371265 | Uncharacterized LOC105371265 | RNA Gene | 3 | GC16P052581 | 1.790035725 |
| IDS | Iduronate 2-Sulfatase | Protein Coding | 44 | GC0XM149476 | 1.789705992 |
| TLN2 | Talin 2 | Protein Coding | 36 | GC15P062390 | 1.789695382 |
| WFDC21P | WAP Four-Disulfide Core Domain 21, Pseudogene | Pseudogene | 13 | GC17M060085 | 1.789473772 |
| TXK | TXK Tyrosine Kinase | Protein Coding | 40 | GC04M048135 | 1.789444804 |
| PPP1R3D | Protein Phosphatase 1 Regulatory Subunit 3D | Protein Coding | 30 | GC20M059936 | 1.789346933 |
| TNFAIP8L3 | TNF Alpha Induced Protein 8 Like 3 | Protein Coding | 31 | GC15M051056 | 1.788519144 |
| SLC1A4 | Solute Carrier Family 1 Member 4 | Protein Coding | 41 | GC02P064988 | 1.78840673 |
| CACNB4 | Calcium Voltage-Gated Channel Auxiliary Subunit Beta 4 | Protein Coding | 42 | GC02M151832 | 1.78831923 |
| DNM1 | Dynamin 1 | Protein Coding | 45 | GC09P128191 | 1.787561655 |
| RAD9B | RAD9 Checkpoint Clamp Component B | Protein Coding | 31 | GC12P110502 | 1.787561655 |
| MED10 | Mediator Complex Subunit 10 | Protein Coding | 31 | GC05M006371 | 1.787177563 |
| YPEL3 | Yippee Like 3 | Protein Coding | 31 | GC16M036469 | 1.786708117 |
| ALDH18A1 | Aldehyde Dehydrogenase 18 Family Member A1 | Protein Coding | 40 | GC10M095605 | 1.7865448 |
| LINC01511 | Long Intergenic Non-Protein Coding RNA 1511 | RNA Gene | 12 | GC05M001365 | 1.786217451 |
| MIR4443 | MicroRNA 4443 | RNA Gene | 11 | GC03P048848 | 1.78586781 |
| ZIC3 | Zic Family Member 3 | Protein Coding | 42 | GC0XP137566 | 1.785680294 |
| UPRT | Uracil Phosphoribosyltransferase Homolog | Protein Coding | 30 | GC0XP075156 | 1.784874439 |
| SEC13 | SEC13 Homolog, Nuclear Pore And COPII Coat Complex Component | Protein Coding | 36 | GC03M010293 | 1.784625769 |
| POMT2 | Protein O-Mannosyltransferase 2 | Protein Coding | 39 | GC14M077274 | 1.784550428 |
| DCUN1D5 | Defective In Cullin Neddylation 1 Domain Containing 5 | Protein Coding | 28 | GC11M103051 | 1.784369946 |
| ASCC3 | Activating Signal Cointegrator 1 Complex Subunit 3 | Protein Coding | 33 | GC06M100508 | 1.78428936 |
| PNPLA4 | Patatin Like Phospholipase Domain Containing 4 | Protein Coding | 32 | GC0XM007898 | 1.784245014 |
| OTUD3 | OTU Deubiquitinase 3 | Protein Coding | 31 | GC01P019881 | 1.784045696 |
| USP12 | Ubiquitin Specific Peptidase 12 | Protein Coding | 35 | GC13M027066 | 1.783261657 |
| KCTD8 | Potassium Channel Tetramerization Domain Containing 8 | Protein Coding | 31 | GC04M044173 | 1.783258438 |
| ABLIM1 | Actin Binding LIM Protein 1 | Protein Coding | 35 | GC10M114469 | 1.783052683 |
| ARGLU1 | Arginine And Glutamate Rich 1 | Protein Coding | 31 | GC13M106541 | 1.782735586 |
| GYG2 | Glycogenin 2 | Protein Coding | 35 | GC0XP002828 | 1.782697201 |
| KCNA10 | Potassium Voltage-Gated Channel Subfamily A Member 10 | Protein Coding | 38 | GC01M110517 | 1.782666683 |
| ZNF540 | Zinc Finger Protein 540 | Protein Coding | 28 | GC19P037551 | 1.782666683 |
| SCAF8 | SR-Related CTD Associated Factor 8 | Protein Coding | 30 | GC06P154733 | 1.782640338 |
| AASS | Aminoadipate-Semialdehyde Synthase | Protein Coding | 39 | GC07M122073 | 1.782585621 |
| SLC25A19 | Solute Carrier Family 25 Member 19 | Protein Coding | 39 | GC17M075273 | 1.782388449 |
| CPSF2 | Cleavage And Polyadenylation Specific Factor 2 | Protein Coding | 32 | GC14P092121 | 1.781850815 |
| CLTCL1 | Clathrin Heavy Chain Like 1 | Protein Coding | 36 | GC22M019313 | 1.780957937 |
| PKN2 | Protein Kinase N2 | Protein Coding | 40 | GC01P088684 | 1.780840278 |
| OAS3 | 2'-5'-Oligoadenylate Synthetase 3 | Protein Coding | 36 | GC12P112938 | 1.780810475 |
| LRPAP1 | LDL Receptor Related Protein Associated Protein 1 | Protein Coding | 39 | GC04M003508 | 1.778672814 |
| WASHC1 | WASH Complex Subunit 1 | Protein Coding | 23 | GC09M000016 | 1.778650999 |
| SLC38A9 | Solute Carrier Family 38 Member 9 | Protein Coding | 32 | GC05M055625 | 1.778410554 |
| CCL23 | C-C Motif Chemokine Ligand 23 | Protein Coding | 32 | GC17M036013 | 1.778226137 |
| DDX56 | DEAD-Box Helicase 56 | Protein Coding | 34 | GC07M044565 | 1.778066635 |
| ARFGAP2 | ADP Ribosylation Factor GTPase Activating Protein 2 | Protein Coding | 32 | GC11M086873 | 1.777977109 |
| GAMT | Guanidinoacetate N-Methyltransferase | Protein Coding | 40 | GC19M001397 | 1.777044177 |
| COPS4 | COP9 Signalosome Subunit 4 | Protein Coding | 30 | GC04P083034 | 1.776214242 |

| | | | | | |
|---|---|---|---|---|---|
| CLASP2 | Cytoplasmic Linker Associated Protein 2 | Protein Coding | 35 | GC03M033537 | 1.776133537 |
| LINC01355 | Long Intergenic Non-Protein Coding RNA 1355 | RNA Gene | 13 | GC01M023281 | 1.775610209 |
| SLC38A3 | Solute Carrier Family 38 Member 3 | Protein Coding | 33 | GC03P050205 | 1.77500999 |
| VGLL1 | Vestigial Like Family Member 1 | Protein Coding | 30 | GC0XP136532 | 1.774495959 |
| SYNJ2 | Synaptojanin 2 | Protein Coding | 35 | GC06P157981 | 1.774342299 |
| ZNF394 | Zinc Finger Protein 394 | Protein Coding | 32 | GC07M099480 | 1.774249077 |
| LEO1 | LEO1 Homolog, Paf1/RNA Polymerase II Complex Component | Protein Coding | 32 | GC15M081874 | 1.773636818 |
| RSAD2 | Radical S-Adenosyl Methionine Domain Containing 2 | Protein Coding | 36 | GC02P006865 | 1.773529053 |
| RRAD | RRAD, Ras Related Glycolysis Inhibitor And Calcium Channel Regulator | Protein Coding | 35 | GC16M066973 | 1.773324728 |
| MKLN1 | Muskelin 1 | Protein Coding | 34 | GC07P131110 | 1.77308929 |
| PSD4 | Pleckstrin And Sec7 Domain Containing 4 | Protein Coding | 31 | GC02P113157 | 1.773029566 |
| SRBD1 | S1 RNA Binding Domain 1 | Protein Coding | 31 | GC02M045388 | 1.772932529 |
| PRKAB2 | Protein Kinase AMP-Activated Non-Catalytic Subunit Beta 2 | Protein Coding | 41 | GC01M147155 | 1.772878647 |
| DNAH3 | Dynein Axonemal Heavy Chain 3 | Protein Coding | 30 | GC16M020944 | 1.772803783 |
| LINC00635 | Long Intergenic Non-Protein Coding RNA 635 | RNA Gene | 15 | GC03M107840 | 1.772699594 |
| PLAC9P1 | Placenta Associated 9 Pseudogene 1 | Pseudogene | 9 | GC02M129923 | 1.772699594 |
| LOC105371114 | Uncharacterized LOC105371114 | RNA Gene | 5 | GC16M019243 | 1.772699594 |
| SERTAD1 | SERTA Domain Containing 1 | Protein Coding | 31 | GC19M040421 | 1.772490263 |
| VPS41 | VPS41 Subunit Of HOPS Complex | Protein Coding | 36 | GC07M039044 | 1.772162437 |
| HERC5 | HECT And RLD Domain Containing E3 Ubiquitin Protein Ligase 5 | Protein Coding | 35 | GC04P088457 | 1.771839857 |
| CCDC154 | Coiled-Coil Domain Containing 154 | Protein Coding | 26 | GC16M001434 | 1.77181077 |
| SUMO4 | Small Ubiquitin Like Modifier 4 | Protein Coding | 35 | GC06P149401 | 1.771808028 |
| PKMYT1 | Protein Kinase, Membrane Associated Tyrosine/Threonine 1 | Protein Coding | 38 | GC16M006794 | 1.771387458 |
| CLK1 | CDC Like Kinase 1 | Protein Coding | 38 | GC02M200853 | 1.771178246 |
| RAB3C | RAB3C, Member RAS Oncogene Family | Protein Coding | 33 | GC05P058582 | 1.771086097 |
| TREX2 | Three Prime Repair Exonuclease 2 | Protein Coding | 32 | GC0XM153444 | 1.771008134 |
| BRF2 | BRF2 RNA Polymerase III Transcription Initiation Factor Subunit | Protein Coding | 32 | GC08M037820 | 1.770899773 |
| ZNF592 | Zinc Finger Protein 592 | Protein Coding | 32 | GC15P115139 | 1.77075386 |
| THEM6 | Thioesterase Superfamily Member 6 | Protein Coding | 26 | GC08P142727 | 1.770678401 |
| ARPC3 | Actin Related Protein 2/3 Complex Subunit 3 | Protein Coding | 38 | GC12M110434 | 1.770637035 |
| SYMPK | Symplekin Scaffold Protein | Protein Coding | 35 | GC19M045815 | 1.770473003 |
| CYP51A1 | Cytochrome P450 Family 51 Subfamily A Member 1 | Protein Coding | 39 | GC07M092112 | 1.770463228 |
| IGK | Immunoglobulin Kappa Locus | Protein Coding | 14 | GC02P088857 | 1.770191789 |
| RNF214 | Ring Finger Protein 214 | Protein Coding | 29 | GC11P117232 | 1.769915819 |
| TRMT112 | TRNA Methyltransferase Activator Subunit 11-2 | Protein Coding | 32 | GC11M064316 | 1.769855976 |
| POU2AF2 | POU Class 2 Homeobox Associating Factor 2 | Protein Coding | 23 | GC11P111245 | 1.769841909 |
| SNRPD2 | Small Nuclear Ribonucleoprotein D2 Polypeptide | Protein Coding | 35 | GC19M063963 | 1.769269586 |
| IL21R | Interleukin 21 Receptor | Protein Coding | 40 | GC16P027826 | 1.769076467 |
| SOCS7 | Suppressor Of Cytokine Signaling 7 | Protein Coding | 31 | GC17P052964 | 1.768761396 |
| DLX5 | Distal-Less Homeobox 5 | Protein Coding | 39 | GC07M097020 | 1.768668652 |
| SPRR2A | Small Proline Rich Protein 2A | Protein Coding | 31 | GC01M153057 | 1.768299818 |
| MIR614 | MicroRNA 614 | RNA Gene | 16 | GC12P020085 | 1.768213272 |
| AP1S2 | Adaptor Related Protein Complex 1 Subunit Sigma 2 | Protein Coding | 38 | GC0XM015825 | 1.768010378 |
| DSN1 | DSN1 Component Of MIS12 Kinetochore Complex | Protein Coding | 30 | GC20M036751 | 1.767727971 |
| AFAP1L1 | Actin Filament Associated Protein 1 Like 1 | Protein Coding | 31 | GC05P149271 | 1.767665386 |
| RALGAPA2 | Ral GTPase Activating Protein Catalytic Subunit Alpha 2 | Protein Coding | 34 | GC20M020374 | 1.767462015 |
| CHP1 | Calcineurin Like EF-Hand Protein 1 | Protein Coding | 33 | GC15P041230 | 1.767388821 |
| HTR2C | 5-Hydroxytryptamine Receptor 2C | Protein Coding | 42 | GC0XP114584 | 1.767092466 |
| CACNA1F | Calcium Voltage-Gated Channel Subunit Alpha1 F | Protein Coding | 40 | GC0XM049205 | 1.767012358 |
| EBF2 | EBF Transcription Factor 2 | Protein Coding | 34 | GC08M025841 | 1.766712904 |
| MED8 | Mediator Complex Subunit 8 | Protein Coding | 30 | GC01M043383 | 1.766698837 |
| MILIP | MYC Inducible LncRNA Inactivating P53 | RNA Gene | 11 | GC17P081929 | 1.766572952 |
| KIFC3 | Kinesin Family Member C3 | Protein Coding | 34 | GC16M057758 | 1.766472697 |
| HOXD3 | Homeobox D3 | Protein Coding | 35 | GC02P176136 | 1.766334057 |
| DECR1 | 2,4-Dienoyl-CoA Reductase 1 | Protein Coding | 36 | GC08P090001 | 1.766094208 |
| ADK | Adenosine Kinase | Protein Coding | 45 | GC10P074152 | 1.7660079 |
| NBPF6 | NBPF Member 6 | Protein Coding | 26 | GC01P108422 | 1.765818477 |
| TP53RK | TP53 Regulating Kinase | Protein Coding | 37 | GC20M046684 | 1.765388131 |
| NELFE | Negative Elongation Factor Complex Member E | Protein Coding | 29 | GC06M031952 | 1.765129089 |
| MADCAM1 | Mucosal Vascular Addressin Cell Adhesion Molecule 1 | Protein Coding | 36 | GC19P002400 | 1.764714479 |
| GPX8 | Glutathione Peroxidase 8 (Putative) | Protein Coding | 35 | GC05P055160 | 1.764442921 |
| MRTFB | Myocardin Related Transcription Factor B | Protein Coding | 29 | GC16P014071 | 1.764074564 |
| LONP2 | Lon Peptidase 2, Peroxisomal | Protein Coding | 35 | GC16P048244 | 1.763765335 |
| OSR1 | Odd-Skipped Related Transcription Factor 1 | Protein Coding | 34 | GC02M019351 | 1.763257742 |
| PIGU | Phosphatidylinositol Glycan Anchor Biosynthesis Class U | Protein Coding | 33 | GC20M034560 | 1.763131618 |
| ZNF701 | Zinc Finger Protein 701 | Protein Coding | 27 | GC19P052555 | 1.763101697 |
| PPP4C | Protein Phosphatase 4 Catalytic Subunit | Protein Coding | 40 | GC16P040208 | 1.762572765 |
| EIF4G3 | Eukaryotic Translation Initiation Factor 4 Gamma 3 | Protein Coding | 37 | GC01M020806 | 1.762151003 |
| XPO7 | Exportin 7 | Protein Coding | 35 | GC08P021919 | 1.76203537 |
| SAE1 | SUMO1 Activating Enzyme Subunit 1 | Protein Coding | 40 | GC19P063933 | 1.761711121 |
| TROAP | Trophinin Associated Protein | Protein Coding | 30 | GC12P049323 | 1.761564851 |
| ACTL9 | Actin Like 9 | Protein Coding | 31 | GC19M008697 | 1.761407614 |
| TLE5 | TLE Family Member 5, Transcriptional Modulator | Protein Coding | 30 | GC19M005005 | 1.761236668 |
| ADAMTS20 | ADAM Metallopeptidase With Thrombospondin Type 1 Motif 20 | Protein Coding | 33 | GC12M043353 | 1.760791063 |
| APOBEC1 | Apolipoprotein B MRNA Editing Enzyme Catalytic Subunit 1 | Protein Coding | 35 | GC12M007649 | 1.760511637 |
| HTR3C | 5-Hydroxytryptamine Receptor 3C | Protein Coding | 36 | GC03P184056 | 1.760190487 |
| EGR3 | Early Growth Response 3 | Protein Coding | 39 | GC08M022687 | 1.760124683 |
| ALG8 | ALG8 Alpha-1,3-Glucosyltransferase | Protein Coding | 38 | GC11M087730 | 1.759789109 |
| RND3 | Rho Family GTPase 3 | Protein Coding | 36 | GC02M150468 | 1.759632826 |
| PRCP | Prolylcarboxypeptidase | Protein Coding | 42 | GC11M087769 | 1.759608388 |
| MARVELD1 | MARVEL Domain Containing 1 | Protein Coding | 30 | GC10P097713 | 1.759531021 |
| ITGB1BP1 | Integrin Subunit Beta 1 Binding Protein 1 | Protein Coding | 35 | GC02M009391 | 1.759290695 |
| BAZ2B | Bromodomain Adjacent To Zinc Finger Domain 2B | Protein Coding | 36 | GC02M159318 | 1.759174109 |
| PIGW | Phosphatidylinositol Glycan Anchor Biosynthesis Class W | Protein Coding | 33 | GC17P036534 | 1.758933544 |
| RPRD2 | Regulation Of Nuclear Pre-MRNA Domain Containing 2 | Protein Coding | 32 | GC01P150363 | 1.758792877 |
| ARHGAP17 | Rho GTPase Activating Protein 17 | Protein Coding | 35 | GC16M024931 | 1.758659601 |
| MOK | MOK Protein Kinase | Protein Coding | 34 | GC14M102224 | 1.758657694 |
| SCFD2 | Sec1 Family Domain Containing 2 | Protein Coding | 30 | GC04M052872 | 1.758585691 |
| ZNF804A | Zinc Finger Protein 804A | Protein Coding | 31 | GC02P184598 | 1.758003712 |
| ADAM22 | ADAM Metallopeptidase Domain 22 | Protein Coding | 39 | GC07P087934 | 1.757962704 |
| MIR384 | MicroRNA 384 | RNA Gene | 10 | GC0XM076919 | 1.757900238 |
| ANKRD27 | Ankyrin Repeat Domain 27 | Protein Coding | 32 | GC19M032597 | 1.757605314 |
| MRPS18B | Mitochondrial Ribosomal Protein S18B | Protein Coding | 32 | GC06P030617 | 1.757604003 |
| GRK4 | G Protein-Coupled Receptor Kinase 4 | Protein Coding | 38 | GC04P002963 | 1.757235289 |
| SHCBP1 | SHC Binding And Spindle Associated 1 | Protein Coding | 30 | GC16M046578 | 1.75723052 |

| | | | | | |
|---|---|---|---|---|---|
| HBD | Hemoglobin Subunit Delta | Protein Coding | 35 | GC11M005232 | 1.75626862 |
| CSF2RB | Colony Stimulating Factor 2 Receptor Subunit Beta | Protein Coding | 43 | GC22P036913 | 1.756181717 |
| KRT84 | Keratin 84 | Protein Coding | 30 | GC12M052377 | 1.7559793 |
| NDUFA9 | NADH:Ubiquinone Oxidoreductase Subunit A9 | Protein Coding | 39 | GC12P004649 | 1.75544095 |
| GSPT1 | G1 To S Phase Transition 1 | Protein Coding | 35 | GC16M011868 | 1.755311966 |
| RAI14 | Retinoic Acid Induced 14 | Protein Coding | 33 | GC05P034656 | 1.755267501 |
| PRKCZ-AS1 | PRKCZ Antisense RNA 1 | RNA Gene | 13 | GC01M005578 | 1.755082726 |
| RBP7 | Retinol Binding Protein 7 | Protein Coding | 31 | GC01P009997 | 1.754960179 |
| CCDC91 | Coiled-Coil Domain Containing 91 | Protein Coding | 31 | GC12P028133 | 1.754292488 |
| HMGCLL1 | 3-Hydroxymethyl-3-Methylglutaryl-CoA Lyase Like 1 | Protein Coding | 32 | GC06M055434 | 1.753864169 |
| CHCHD2 | Coiled-Coil-Helix-Coiled-Coil-Helix Domain Containing 2 | Protein Coding | 35 | GC07M056101 | 1.753681421 |
| PTGFR | Prostaglandin F Receptor | Protein Coding | 40 | GC01P078303 | 1.753596544 |
| ADSS1 | Adenylosuccinate Synthase 1 | Protein Coding | 34 | GC14P109287 | 1.753581762 |
| ITGBL1 | Integrin Subunit Beta Like 1 | Protein Coding | 35 | GC13P101454 | 1.753255367 |
| ODAD1 | Outer Dynein Arm Docking Complex Subunit 1 | Protein Coding | 26 | GC19M064058 | 1.752299905 |
| C2CD3 | C2 Domain Containing 3 Centriole Elongation Regulator | Protein Coding | 36 | GC11M074012 | 1.751133442 |
| MRPS25 | Mitochondrial Ribosomal Protein S25 | Protein Coding | 34 | GC03M020224 | 1.750749588 |
| ATG9A | Autophagy Related 9A | Protein Coding | 35 | GC02M219219 | 1.750487804 |
| REPS1 | RALBP1 Associated Eps Domain Containing 1 | Protein Coding | 35 | GC06M138903 | 1.750439882 |
| NBPF25P | NBPF Member 25, Pseudogene | Pseudogene | 8 | GC01M145572 | 1.750315785 |
| AANAT | Aralkylamine N-Acetyltransferase | Protein Coding | 37 | GC17P076453 | 1.750212431 |
| HNRNPH3 | Heterogeneous Nuclear Ribonucleoprotein H3 | Protein Coding | 35 | GC10P068331 | 1.750147343 |
| TOX2 | TOX High Mobility Group Box Family Member 2 | Protein Coding | 33 | GC20P043914 | 1.749432206 |
| CPT1C | Carnitine Palmitoyltransferase 1C | Protein Coding | 38 | GC19P049690 | 1.749091387 |
| TNNI2 | Troponin I2, Fast Skeletal Type | Protein Coding | 40 | GC11P001839 | 1.748448491 |
| ARHGAP15 | Rho GTPase Activating Protein 15 | Protein Coding | 35 | GC02P143070 | 1.74840498 |
| ATP6V0C | ATPase H+ Transporting V0 Subunit C | Protein Coding | 37 | GC16P002513 | 1.747451305 |
| SNRPF | Small Nuclear Ribonucleoprotein Polypeptide F | Protein Coding | 34 | GC12P095858 | 1.747080564 |
| RPS6KC1 | Ribosomal Protein S6 Kinase C1 | Protein Coding | 36 | GC01P213051 | 1.746856213 |
| XRN2 | 5'-3' Exoribonuclease 2 | Protein Coding | 38 | GC20P021303 | 1.745986462 |
| MPHOSPH9 | M-Phase Phosphoprotein 9 | Protein Coding | 32 | GC12M123153 | 1.745931506 |
| SPATA6 | Spermatogenesis Associated 6 | Protein Coding | 32 | GC01M048260 | 1.745890141 |
| POMP | Proteasome Maturation Protein | Protein Coding | 37 | GC13P028659 | 1.745545864 |
| TRMT9B | TRNA Methyltransferase 9B (Putative) | Protein Coding | 27 | GC08P012945 | 1.745414734 |
| PSMB10 | Proteasome 20S Subunit Beta 10 | Protein Coding | 40 | GC16M067937 | 1.745378494 |
| ATP6V0A1 | ATPase H+ Transporting V0 Subunit A1 | Protein Coding | 38 | GC17P042458 | 1.745183229 |
| FAM83C | Family With Sequence Similarity 83 Member C | Protein Coding | 27 | GC20M035285 | 1.744749188 |
| UBR7 | Ubiquitin Protein Ligase E3 Component N-Recognin 7 | Protein Coding | 34 | GC14P093207 | 1.7446872 |
| PREB | Prolactin Regulatory Element Binding | Protein Coding | 35 | GC02M027130 | 1.744143009 |
| NKX6-1 | NK6 Homeobox 1 | Protein Coding | 36 | GC04M084491 | 1.743924379 |
| SPTY2D1 | SPT2 Chromatin Protein Domain Containing 1 | Protein Coding | 27 | GC11M018627 | 1.742890716 |
| PROKR1 | Prokineticin Receptor 1 | Protein Coding | 36 | GC02P068643 | 1.741908073 |
| TRIOBP | TRIO And F-Actin Binding Protein | Protein Coding | 38 | GC22P037697 | 1.741647124 |
| CCDC80 | Coiled-Coil Domain Containing 80 | Protein Coding | 33 | GC03M112604 | 1.741550446 |
| MOV10 | Mov10 RISC Complex RNA Helicase | Protein Coding | 35 | GC01P112673 | 1.741473675 |
| ALDH1B1 | Aldehyde Dehydrogenase 1 Family Member B1 | Protein Coding | 40 | GC09P038392 | 1.741084337 |
| ANP32E | Acidic Nuclear Phosphoprotein 32 Family Member E | Protein Coding | 31 | GC01M150218 | 1.740962029 |
| AP2A2 | Adaptor Related Protein Complex 2 Subunit Alpha 2 | Protein Coding | 33 | GC11P000924 | 1.740646362 |
| PSME2 | Proteasome Activator Subunit 2 | Protein Coding | 36 | GC14M024143 | 1.740558863 |
| TMEM164 | Transmembrane Protein 164 | Protein Coding | 26 | GC0XP110002 | 1.740466714 |
| KIF21A | Kinesin Family Member 21A | Protein Coding | 36 | GC12M039293 | 1.740312338 |
| RNF220 | Ring Finger Protein 220 | Protein Coding | 34 | GC01P044405 | 1.740264535 |
| TFEC | Transcription Factor EC | Protein Coding | 35 | GC07M115935 | 1.739857435 |
| USP24 | Ubiquitin Specific Peptidase 24 | Protein Coding | 39 | GC01M055066 | 1.738942146 |
| NCOA5 | Nuclear Receptor Coactivator 5 | Protein Coding | 32 | GC20M046060 | 1.738085985 |
| MRPL13 | Mitochondrial Ribosomal Protein L13 | Protein Coding | 34 | GC08M120377 | 1.738055229 |
| MIR653 | MicroRNA 653 | RNA Gene | 17 | GC07M093482 | 1.737666249 |
| MAP1LC3C | Microtubule Associated Protein 1 Light Chain 3 Gamma | Protein Coding | 30 | GC01M241995 | 1.736602306 |
| STAB1 | Stabilin 1 | Protein Coding | 38 | GC03P052495 | 1.736477375 |
| PDIA4 | Protein Disulfide Isomerase Family A Member 4 | Protein Coding | 36 | GC07M149003 | 1.736368537 |
| ASAH2 | N-Acylsphingosine Amidohydrolase 2 | Protein Coding | 37 | GC10M050182 | 1.73619628 |
| FBXO10 | F-Box Protein 10 | Protein Coding | 31 | GC09M037510 | 1.736163616 |
| DNAJC5 | DnaJ Heat Shock Protein Family (Hsp40) Member C5 | Protein Coding | 39 | GC20P063895 | 1.735543251 |
| HP1BP3 | Heterochromatin Protein 1 Binding Protein 3 | Protein Coding | 32 | GC01M020742 | 1.735220075 |
| IMP3 | IMP U3 Small Nucleolar Ribonucleoprotein 3 | Protein Coding | 33 | GC15M075639 | 1.734305978 |
| OTUD5 | OTU Deubiquitinase 5 | Protein Coding | 32 | GC0XM048922 | 1.733830214 |
| ZNF366 | Zinc Finger Protein 366 | Protein Coding | 31 | GC05M073270 | 1.73371911 |
| PRKG2 | Protein Kinase CGMP-Dependent 2 | Protein Coding | 42 | GC04M081087 | 1.733532429 |
| POF1B | POF1B Actin Binding Protein | Protein Coding | 32 | GC0XM085277 | 1.733177662 |
| FKRP | Fukutin Related Protein | Protein Coding | 36 | GC19P046746 | 1.7322613 |
| ZNF638 | Zinc Finger Protein 638 | Protein Coding | 35 | GC02P071276 | 1.732158422 |
| KRT72 | Keratin 72 | Protein Coding | 30 | GC12M052585 | 1.731943846 |
| PCNP | PEST Proteolytic Signal Containing Nuclear Protein | Protein Coding | 30 | GC03P101574 | 1.731250048 |
| CNOT8 | CCR4-NOT Transcription Complex Subunit 8 | Protein Coding | 35 | GC05P154867 | 1.731202841 |
| PWWP3B | PWWP Domain Containing 3B | Protein Coding | 24 | GC0XP106169 | 1.730830431 |
| TSC22D3 | TSC22 Domain Family Member 3 | Protein Coding | 36 | GC0XM107713 | 1.730226517 |
| SNRPA1 | Small Nuclear Ribonucleoprotein Polypeptide A' | Protein Coding | 32 | GC15M101281 | 1.729993582 |
| NDST3 | N-Deacetylase And N-Sulfotransferase 3 | Protein Coding | 33 | GC04P118033 | 1.729252696 |
| QPCTL | Glutaminyl-Peptide Cyclotransferase Like | Protein Coding | 30 | GC19P045692 | 1.728274345 |
| CHRDL1 | Chordin Like 1 | Protein Coding | 36 | GC0XM110674 | 1.726563215 |
| ATP6V1C1 | ATPase H+ Transporting V1 Subunit C1 | Protein Coding | 35 | GC08P103032 | 1.725789785 |
| ZNF845 | Zinc Finger Protein 845 | Protein Coding | 28 | GC19P053333 | 1.725576758 |
| POLG2 | DNA Polymerase Gamma 2, Accessory Subunit | Protein Coding | 37 | GC17M064477 | 1.725528955 |
| NCK2 | NCK Adaptor Protein 2 | Protein Coding | 38 | GC02P105744 | 1.725521564 |
| KIF18A | Kinesin Family Member 18A | Protein Coding | 32 | GC11M028020 | 1.724253654 |
| RAB6A | RAB6A, Member RAS Oncogene Family | Protein Coding | 36 | GC11M087560 | 1.724092364 |
| C1GALT1 | Core 1 Synthase, Glycoprotein-N-Acetylgalactosamine 3-Beta-Galactosyltransferase 1 | Protein Coding | 35 | GC07P007156 | 1.724007964 |
| EVI5 | Ecotropic Viral Integration Site 5 | Protein Coding | 34 | GC01M092508 | 1.723762989 |
| ZFHX4 | Zinc Finger Homeobox 4 | Protein Coding | 34 | GC08P076681 | 1.723477364 |
| TRPA1 | Transient Receptor Potential Cation Channel Subfamily A Member 1 | Protein Coding | 42 | GC08M072019 | 1.723372698 |
| DDX23 | DEAD-Box Helicase 23 | Protein Coding | 35 | GC12M048829 | 1.723240376 |
| HR | HR Lysine Demethylase And Nuclear Receptor Corepressor | Protein Coding | 39 | GC08M022114 | 1.722944975 |
| VPS11 | VPS11 Core Subunit Of CORVET And HOPS Complexes | Protein Coding | 38 | GC11P119067 | 1.722685099 |
| CEP350 | Centrosomal Protein 350 | Protein Coding | 31 | GC01P179955 | 1.722681761 |

| | | | | | |
|---|---|---|---|---|---|
| SOX14 | SRY-Box Transcription Factor 14 | Protein Coding | 34 | GC03P137764 | 1.721805334 |
| SRGAP2 | SLIT-ROBO Rho GTPase Activating Protein 2 | Protein Coding | 33 | GC01P206203 | 1.721660614 |
| RNF19A | Ring Finger Protein 19A, RBR E3 Ubiquitin Protein Ligase | Protein Coding | 35 | GC08M100257 | 1.721405745 |
| MTPAP | Mitochondrial Poly(A) Polymerase | Protein Coding | 35 | GC10M032721 | 1.720617294 |
| FERMT3 | FERM Domain Containing Kindlin 3 | Protein Coding | 39 | GC11P064352 | 1.72050333 |
| TAB3 | TGF-Beta Activated Kinase 1 (MAP3K7) Binding Protein 3 | Protein Coding | 36 | GC0XM030888 | 1.720201135 |
| HM13 | Histocompatibility Minor 13 | Protein Coding | 35 | GC20P031514 | 1.720068574 |
| SUMO2 | Small Ubiquitin Like Modifier 2 | Protein Coding | 34 | GC17M075165 | 1.71980679 |
| SHROOM3 | Shroom Family Member 3 | Protein Coding | 34 | GC04P076435 | 1.719453692 |
| PLCZ1 | Phospholipase C Zeta 1 | Protein Coding | 37 | GC12M020552 | 1.719228029 |
| SSBP3 | Single Stranded DNA Binding Protein 3 | Protein Coding | 34 | GC01M054225 | 1.718945384 |
| PHLDB2 | Pleckstrin Homology Like Domain Family B Member 2 | Protein Coding | 32 | GC03P111732 | 1.718881607 |
| UBR2 | Ubiquitin Protein Ligase E3 Component N-Recognin 2 | Protein Coding | 36 | GC06P080587 | 1.718855977 |
| MIR487A | MicroRNA 487a | RNA Gene | 14 | GC14P109538 | 1.71861124 |
| LEMD2 | LEM Domain Nuclear Envelope Protein 2 | Protein Coding | 35 | GC06M063735 | 1.718543768 |
| EMBP1 | Embigin Pseudogene 1 | Pseudogene | 9 | GC01P121519 | 1.718083143 |
| RABEP1 | Rabaptin, RAB GTPase Binding Effector Protein 1 | Protein Coding | 35 | GC17P005282 | 1.717865705 |
| ITPRID1 | ITPR Interacting Domain Containing 1 | Protein Coding | 23 | GC07P031514 | 1.717690825 |
| PPP2R5B | Protein Phosphatase 2 Regulatory Subunit B'Beta | Protein Coding | 32 | GC11P064918 | 1.71754694 |
| FREM1 | FRAS1 Related Extracellular Matrix 1 | Protein Coding | 36 | GC09M014734 | 1.717227221 |
| VARS1 | Valyl-TRNA Synthetase 1 | Protein Coding | 35 | GC06M063623 | 1.716443539 |
| ARMC8 | Armadillo Repeat Containing 8 | Protein Coding | 30 | GC03P138187 | 1.716265917 |
| AP4B1-AS1 | AP4B1 Antisense RNA 1 | RNA Gene | 13 | GC01P113813 | 1.715917587 |
| RIC3 | RIC3 Acetylcholine Receptor Chaperone | Protein Coding | 33 | GC11M008092 | 1.715772867 |
| GDE1 | Glycerophosphodiester Phosphodiesterase 1 | Protein Coding | 32 | GC16M019513 | 1.714630127 |
| BTAF1 | B-TFIID TATA-Box Binding Protein Associated Factor 1 | Protein Coding | 38 | GC10P091923 | 1.714039564 |
| CRLF1 | Cytokine Receptor Like Factor 1 | Protein Coding | 39 | GC19M018572 | 1.713891387 |
| SEPSECS | Sep (O-Phosphoserine) TRNA:Sec (Selenocysteine) TRNA Synthase | Protein Coding | 39 | GC04M025121 | 1.713850379 |
| RNFT1 | Ring Finger Protein, Transmembrane 1 | Protein Coding | 30 | GC17M059952 | 1.713318825 |
| HS3ST2 | Heparan Sulfate-Glucosamine 3-Sulfotransferase 2 | Protein Coding | 35 | GC16P022814 | 1.712948322 |
| ZNF185 | Zinc Finger Protein 185 With LIM Domain | Protein Coding | 32 | GC0XP152898 | 1.71255815 |
| DPY30 | Dpy-30 Histone Methyltransferase Complex Regulatory Subunit | Protein Coding | 32 | GC02M031867 | 1.712459087 |
| SUGT1 | SGT1 Homolog, MIS12 Kinetochore Complex Assembly Cochaperone | Protein Coding | 32 | GC13P052652 | 1.712386608 |
| NSRP1 | Nuclear Speckle Splicing Regulatory Protein 1 | Protein Coding | 30 | GC17P030115 | 1.712287784 |
| TEX10 | Testis Expressed 10 | Protein Coding | 30 | GC09M100302 | 1.711826801 |
| CHST9 | Carbohydrate Sulfotransferase 9 | Protein Coding | 32 | GC18M026906 | 1.711810827 |
| NALF2 | NALCN Channel Auxiliary Factor 2 | Protein Coding | 22 | GC0XP069506 | 1.711333394 |
| LSM11 | LSM11, U7 Small Nuclear RNA Associated | Protein Coding | 31 | GC05P157743 | 1.71124506 |
| AGBL1 | AGBL Carboxypeptidase 1 | Protein Coding | 33 | GC15P115183 | 1.71069181 |
| GRIA4 | Glutamate Ionotropic Receptor AMPA Type Subunit 4 | Protein Coding | 44 | GC11P105609 | 1.710428357 |
| CERS6 | Ceramide Synthase 6 | Protein Coding | 34 | GC02P168455 | 1.710223198 |
| MAGI1 | Membrane Associated Guanylate Kinase, WW And PDZ Domain Containing 1 | Protein Coding | 35 | GC03M065330 | 1.710208774 |
| MCAT | Malonyl-CoA-Acyl Carrier Protein Transacylase | Protein Coding | 37 | GC22M043132 | 1.71001935 |
| ERI2 | ERI1 Exoribonuclease Family Member 2 | Protein Coding | 28 | GC16M020780 | 1.709921479 |
| FARSA | Phenylalanyl-TRNA Synthetase Subunit Alpha | Protein Coding | 37 | GC19M012922 | 1.709848881 |
| MANBA | Mannosidase Beta | Protein Coding | 39 | GC04M102631 | 1.709798455 |
| KPNA6 | Karyopherin Subunit Alpha 6 | Protein Coding | 34 | GC01P032108 | 1.709634542 |
| GOLGA2P10 | GOLGA2 Pseudogene 10 | Pseudogene | 10 | GC15M082471 | 1.709596753 |
| FAM193A | Family With Sequence Similarity 193 Member A | Protein Coding | 27 | GC04P002536 | 1.709144831 |
| TLK1 | Tousled Like Kinase 1 | Protein Coding | 38 | GC02M170990 | 1.708943129 |
| AP1B1 | Adaptor Related Protein Complex 1 Subunit Beta 1 | Protein Coding | 39 | GC22M029327 | 1.70879066 |
| ATG4C | Autophagy Related 4C Cysteine Peptidase | Protein Coding | 35 | GC01P062784 | 1.70871377 |
| ASH1L | ASH1 Like Histone Lysine Methyltransferase | Protein Coding | 36 | GC01M155335 | 1.708506346 |
| RIN3 | Ras And Rab Interactor 3 | Protein Coding | 34 | GC14P092513 | 1.708441615 |
| SLC7A8 | Solute Carrier Family 7 Member 8 | Protein Coding | 36 | GC14M023125 | 1.707399368 |
| FBXO39 | F-Box Protein 39 | Protein Coding | 29 | GC17P006776 | 1.707321644 |
| STX16 | Syntaxin 16 | Protein Coding | 37 | GC20P058652 | 1.706096888 |
| TRAPPC2L | Trafficking Protein Particle Complex Subunit 2L | Protein Coding | 32 | GC16P088856 | 1.705435276 |
| KRT71 | Keratin 71 | Protein Coding | 34 | GC12M052543 | 1.705240607 |
| PEX11B | Peroxisomal Biogenesis Factor 11 Beta | Protein Coding | 36 | GC01M145911 | 1.704699755 |
| TNKS1BP1 | Tankyrase 1 Binding Protein 1 | Protein Coding | 32 | GC11M086953 | 1.704392076 |
| TAS2R4 | Taste 2 Receptor Member 4 | Protein Coding | 30 | GC07P148229 | 1.704105973 |
| SLC10A6 | Solute Carrier Family 10 Member 6 | Protein Coding | 31 | GC04M086823 | 1.703380823 |
| INTS2 | Integrator Complex Subunit 2 | Protein Coding | 31 | GC17M061865 | 1.703132629 |
| RYR3 | Ryanodine Receptor 3 | Protein Coding | 37 | GC15P033310 | 1.702536821 |
| IPW | Imprinted In Prader-Willi Syndrome | RNA Gene | 16 | GC15P025116 | 1.702457786 |
| CALU | Calumenin | Protein Coding | 38 | GC07P128739 | 1.702336431 |
| KPNA3 | Karyopherin Subunit Alpha 3 | Protein Coding | 39 | GC13M049699 | 1.701248407 |
| STK17A | Serine/Threonine Kinase 17a | Protein Coding | 37 | GC07P043582 | 1.701244712 |
| DPM3 | Dolichyl-Phosphate Mannosyltransferase Subunit 3, Regulatory | Protein Coding | 35 | GC01M155139 | 1.700732112 |
| ARMT1 | Acidic Residue Methyltransferase 1 | Protein Coding | 27 | GC06P151452 | 1.700577497 |
| LEFTY1 | Left-Right Determination Factor 1 | Protein Coding | 37 | GC01M225887 | 1.700365782 |
| ZNF496 | Zinc Finger Protein 496 | Protein Coding | 31 | GC01M247297 | 1.700222254 |
| AGFG2 | ArfGAP With FG Repeats 2 | Protein Coding | 31 | GC07P100539 | 1.700137258 |
| ZNF223 | Zinc Finger Protein 223 | Protein Coding | 27 | GC19P044051 | 1.700101852 |
| KIRREL3 | Kirre Like Nephrin Family Adhesion Molecule 3 | Protein Coding | 39 | GC11M126423 | 1.699800372 |
| KIR2DL4 | Killer Cell Immunoglobulin Like Receptor, Two Ig Domains And Long Cytoplasmic Tail 4 | Protein Coding | 35 | GC19P064344 | 1.69920969 |
| SERPINA4 | Serpin Family A Member 4 | Protein Coding | 38 | GC14P094561 | 1.698643327 |
| SLIT3 | Slit Guidance Ligand 3 | Protein Coding | 38 | GC05M168661 | 1.698503256 |
| CDH6 | Cadherin 6 | Protein Coding | 38 | GC05P031193 | 1.698176265 |
| NIPSNAP1 | Nipsnap Homolog 1 | Protein Coding | 35 | GC22M029554 | 1.697654963 |
| IL34 | Interleukin 34 | Protein Coding | 34 | GC16P071459 | 1.697113872 |
| MIR503HG | MIR503 Host Gene | RNA Gene | 17 | GC0XM134687 | 1.697086334 |
| SLC12A4 | Solute Carrier Family 12 Member 4 | Protein Coding | 40 | GC16M068078 | 1.697028279 |
| ACBD5 | Acyl-CoA Binding Domain Containing 5 | Protein Coding | 35 | GC10M027182 | 1.696948886 |
| S1PR2 | Sphingosine-1-Phosphate Receptor 2 | Protein Coding | 40 | GC19M010223 | 1.696691513 |
| S100A3 | S100 Calcium Binding Protein A3 | Protein Coding | 34 | GC01M153547 | 1.69636631 |
| GPATCH8 | G-Patch Domain Containing 8 | Protein Coding | 32 | GC17M044395 | 1.696323037 |
| ATL3 | Atlastin GTPase 3 | Protein Coding | 34 | GC11M063624 | 1.695939898 |
| TRIM59 | Tripartite Motif Containing 59 | Protein Coding | 31 | GC03M160432 | 1.69549799 |
| LYRM4 | LYR Motif Containing 4 | Protein Coding | 34 | GC06M005032 | 1.695312738 |
| LRRC4C | Leucine Rich Repeat Containing 4C | Protein Coding | 35 | GC11M040119 | 1.695166469 |
| CHRM1 | Cholinergic Receptor Muscarinic 1 | Protein Coding | 41 | GC11M087149 | 1.694617152 |
| MACROH2A2 | MacroH2A.2 Histone | Protein Coding | 27 | GC10P070053 | 1.694600344 |

| | | | | | |
|---|---|---|---|---|---|
| EFR3B | EFR3 Homolog B | Protein Coding | 30 | GC02P025002 | 1.693866849 |
| CCDC18 | Coiled-Coil Domain Containing 18 | Protein Coding | 26 | GC01P093179 | 1.6936239 |
| CLUH | Clustered Mitochondria Homolog | Protein Coding | 29 | GC17M002689 | 1.693231702 |
| COPS8 | COP9 Signalosome Subunit 8 | Protein Coding | 32 | GC02P237085 | 1.692984462 |
| ANKS1B | Ankyrin Repeat And Sterile Alpha Motif Domain Containing 1B | Protein Coding | 35 | GC12M098726 | 1.692656517 |
| LAMTOR2 | Late Endosomal/Lysosomal Adaptor, MAPK And MTOR Activator 2 | Protein Coding | 35 | GC01P156054 | 1.692280054 |
| MDN1 | Midasin AAA ATPase 1 | Protein Coding | 34 | GC06M089642 | 1.69219327 |
| FBXO6 | F-Box Protein 6 | Protein Coding | 32 | GC01P011664 | 1.69219172 |
| BMS1 | BMS1 Ribosome Biogenesis Factor | Protein Coding | 35 | GC10P042782 | 1.692130089 |
| TBR1 | T-Box Brain Transcription Factor 1 | Protein Coding | 38 | GC02P161416 | 1.691979647 |
| EYA3 | EYA Transcriptional Coactivator And Phosphatase 3 | Protein Coding | 34 | GC01M027970 | 1.691930056 |
| SIGIRR | Single Ig And TIR Domain Containing | Protein Coding | 36 | GC11M002860 | 1.691510201 |
| C10orf90 | Chromosome 10 Open Reading Frame 90 | Protein Coding | 28 | GC10M126424 | 1.690886736 |
| UQCRB | Ubiquinol-Cytochrome C Reductase Binding Protein | Protein Coding | 39 | GC08M096225 | 1.69068563 |
| NALCN | Sodium Leak Channel, Non-Selective | Protein Coding | 36 | GC13M101053 | 1.690425992 |
| RHEBL1 | RHEB Like 1 | Protein Coding | 32 | GC12M049064 | 1.69041419 |
| RAPGEF2 | Rap Guanine Nucleotide Exchange Factor 2 | Protein Coding | 36 | GC04P159106 | 1.69015193 |
| ZNF677 | Zinc Finger Protein 677 | Protein Coding | 28 | GC19M053235 | 1.690017939 |
| DHX36 | DEAH-Box Helicase 36 | Protein Coding | 35 | GC03M154272 | 1.689956188 |
| VGLL3 | Vestigial Like Family Member 3 | Protein Coding | 31 | GC03M086876 | 1.689301968 |
| MIR4654 | MicroRNA 4654 | RNA Gene | 13 | GC01P162157 | 1.689268827 |
| PIEZO2 | Piezo Type Mechanosensitive Ion Channel Component 2 | Protein Coding | 32 | GC18M010670 | 1.688993454 |
| CSNK1G2 | Casein Kinase 1 Gamma 2 | Protein Coding | 41 | GC19P001941 | 1.688569665 |
| PLTP | Phospholipid Transfer Protein | Protein Coding | 40 | GC20M045898 | 1.688009501 |
| PGAM1P5 | Phosphoglycerate Mutase 1 Pseudogene 5 | Pseudogene | 10 | GC12P095551 | 1.68794477 |
| HDAC11 | Histone Deacetylase 11 | Protein Coding | 39 | GC03P013495 | 1.687695742 |
| NOS1AP | Nitric Oxide Synthase 1 Adaptor Protein | Protein Coding | 35 | GC01P162069 | 1.687267423 |
| CRNKL1 | Crooked Neck Pre-MRNA Splicing Factor 1 | Protein Coding | 33 | GC20M020034 | 1.687170267 |
| SNX6 | Sorting Nexin 6 | Protein Coding | 32 | GC14M034561 | 1.687073231 |
| AOX1 | Aldehyde Oxidase 1 | Protein Coding | 40 | GC02P200585 | 1.686978698 |
| SEC24B | SEC24 Homolog B, COPII Coat Complex Component | Protein Coding | 36 | GC04P109433 | 1.686727524 |
| TRPC4AP | Transient Receptor Potential Cation Channel Subfamily C Member 4 Associated Protein | Protein Coding | 33 | GC20M035002 | 1.686153412 |
| LOC106728418 | LEP 5' Regulatory Region | Biological Region | 2 | GC07P128238 | 1.68608427 |
| MIR1284 | MicroRNA 1284 | RNA Gene | 15 | GC03M071541 | 1.68594861 |
| MRPL34 | Mitochondrial Ribosomal Protein L34 | Protein Coding | 30 | GC19P063103 | 1.685643077 |
| DNAJC9 | DnaJ Heat Shock Protein Family (Hsp40) Member C9 | Protein Coding | 33 | GC10M073183 | 1.685618639 |
| PLEKHG6 | Pleckstrin Homology And RhoGEF Domain Containing G6 | Protein Coding | 30 | GC12P006310 | 1.685569048 |
| G6PC3 | Glucose-6-Phosphatase Catalytic Subunit 3 | Protein Coding | 36 | GC17P044070 | 1.685360789 |
| BBS12 | Bardet-Biedl Syndrome 12 | Protein Coding | 34 | GC04P122702 | 1.68517518 |
| RAPGEF1 | Rap Guanine Nucleotide Exchange Factor 1 | Protein Coding | 37 | GC09M131576 | 1.684460878 |
| PAPOLA | Poly(A) Polymerase Alpha | Protein Coding | 37 | GC14P096501 | 1.684369922 |
| CLIC6 | Chloride Intracellular Channel 6 | Protein Coding | 35 | GC21P034671 | 1.684134007 |
| AK4 | Adenylate Kinase 4 | Protein Coding | 35 | GC01P065147 | 1.683908701 |
| TUBA3C | Tubulin Alpha 3c | Protein Coding | 32 | GC13M019173 | 1.683565378 |
| VPS35 | VPS35 Retromer Complex Component | Protein Coding | 39 | GC16M046962 | 1.683489561 |
| CCZ1 | CCZ1 Homolog, Vacuolar Protein Trafficking And Biogenesis Associated | Protein Coding | 29 | GC07P005898 | 1.682794213 |
| DCAF8L1 | DDB1 And CUL4 Associated Factor 8 Like 1 | Protein Coding | 25 | GC0XM027996 | 1.68215251 |
| ADGRL4 | Adhesion G Protein-Coupled Receptor L4 | Protein Coding | 32 | GC01M078889 | 1.681960344 |
| FNDC1 | Fibronectin Type III Domain Containing 1 | Protein Coding | 31 | GC06P160417 | 1.68190527 |
| TMEM126B | Transmembrane Protein 126B | Protein Coding | 35 | GC11P085628 | 1.681576848 |
| TLE3 | TLE Family Member 3, Transcriptional Corepressor | Protein Coding | 39 | GC15M070047 | 1.681365609 |
| SEC22B | SEC22 Homolog B, Vesicle Trafficking Protein | Protein Coding | 31 | GC01M120150 | 1.681190968 |
| DENND1A | DENN Domain Containing 1A | Protein Coding | 34 | GC09M123379 | 1.680451155 |
| SLC4A4 | Solute Carrier Family 4 Member 4 | Protein Coding | 42 | GC04P071063 | 1.680322647 |
| PRPF40A | Pre-MRNA Processing Factor 40 Homolog A | Protein Coding | 32 | GC02M152651 | 1.680062294 |
| HSALNG0054372 | | RNA Gene | 4 | GC06P151624 | 1.679772377 |
| lnc-RMND1-2 | | RNA Gene | 4 | GC06M151644 | 1.679772377 |
| SLC26A4-AS1 | SLC26A4 Antisense RNA 1 | RNA Gene | 14 | GC07M107653 | 1.67859292 |
| IPO9 | Importin 9 | Protein Coding | 32 | GC01P201829 | 1.678521752 |
| ATPAF2 | ATP Synthase Mitochondrial F1 Complex Assembly Factor 2 | Protein Coding | 34 | GC17M017977 | 1.67802012 |
| WDR82 | WD Repeat Domain 82 | Protein Coding | 32 | GC03M052254 | 1.677205086 |
| UBXN4 | UBX Domain Protein 4 | Protein Coding | 32 | GC02P135741 | 1.676775932 |
| MIR629 | MicroRNA 629 | RNA Gene | 17 | GC15M070079 | 1.676229715 |
| MIR592 | MicroRNA 592 | RNA Gene | 15 | GC07M127058 | 1.675347209 |
| MIR544A | MicroRNA 544a | RNA Gene | 14 | GC14P109546 | 1.674920678 |
| R3HDM1 | R3H Domain Containing 1 | Protein Coding | 28 | GC02P135531 | 1.674909592 |
| DTYMK | Deoxythymidylate Kinase | Protein Coding | 36 | GC02M241675 | 1.673696995 |
| LPCAT2 | Lysophosphatidylcholine Acyltransferase 2 | Protein Coding | 38 | GC16P055510 | 1.673494101 |
| GFPT2 | Glutamine-Fructose-6-Phosphate Transaminase 2 | Protein Coding | 39 | GC05M180300 | 1.67339468 |
| CLNS1A | Chloride Nucleotide-Sensitive Channel 1A | Protein Coding | 36 | GC11M087711 | 1.673361897 |
| KYNU | Kynureninase | Protein Coding | 42 | GC02P142877 | 1.673295021 |
| GRIA3 | Glutamate Ionotropic Receptor AMPA Type Subunit 3 | Protein Coding | 46 | GC0XP123184 | 1.672181249 |
| WDPCP | WD Repeat Containing Planar Cell Polarity Effector | Protein Coding | 33 | GC02M063121 | 1.671572804 |
| MIR1307 | MicroRNA 1307 | RNA Gene | 17 | GC10M103394 | 1.671490908 |
| ATAT1 | Alpha Tubulin Acetyltransferase 1 | Protein Coding | 29 | GC06P030626 | 1.670862675 |
| MFAP4 | Microfibril Associated Protein 4 | Protein Coding | 36 | GC17M019383 | 1.670705795 |
| SPRR2D | Small Proline Rich Protein 2D | Protein Coding | 25 | GC01M153039 | 1.670091629 |
| LCORL | Ligand Dependent Nuclear Receptor Corepressor Like | Protein Coding | 32 | GC04M017844 | 1.669622183 |
| CBLIF | Cobalamin Binding Intrinsic Factor | Protein Coding | 34 | GC11M059829 | 1.668738008 |
| PLIN4 | Perilipin 4 | Protein Coding | 32 | GC19M005067 | 1.6685853 |
| ITGAD | Integrin Subunit Alpha D | Protein Coding | 32 | GC16P040318 | 1.668528914 |
| NARS2 | Asparaginyl-TRNA Synthetase 2, Mitochondrial | Protein Coding | 38 | GC11M078435 | 1.668183446 |
| RHBDD1 | Rhomboid Domain Containing 1 | Protein Coding | 31 | GC02P226805 | 1.668110728 |
| KRT6C | Keratin 6C | Protein Coding | 34 | GC12M052468 | 1.667803049 |
| DENND5B | DENN Domain Containing 5B | Protein Coding | 31 | GC12M031391 | 1.667140245 |
| PTDSS1 | Phosphatidylserine Synthase 1 | Protein Coding | 39 | GC08P096261 | 1.666957378 |
| ALG13 | ALG13 UDP-N-Acetylglucosaminyltransferase Subunit | Protein Coding | 33 | GC0XP111665 | 1.666915178 |
| FBXO42 | F-Box Protein 42 | Protein Coding | 30 | GC01M016343 | 1.666068435 |
| TMPRSS15 | Transmembrane Serine Protease 15 | Protein Coding | 39 | GC21M018269 | 1.665795445 |
| RGS3 | Regulator Of G Protein Signaling 3 | Protein Coding | 34 | GC09P118613 | 1.66549778 |
| SPATA22 | Spermatogenesis Associated 22 | Protein Coding | 31 | GC17M003440 | 1.665294886 |
| TENM3 | Teneurin Transmembrane Protein 3 | Protein Coding | 34 | GC04P181448 | 1.66526401 |
| ARID3A | AT-Rich Interaction Domain 3A | Protein Coding | 35 | GC19P002497 | 1.664400578 |
| PADI6 | Peptidyl Arginine Deiminase 6 | Protein Coding | 34 | GC01P017754 | 1.664362669 |

| | | | | | |
|---|---|---|---|---|---|
| GPX7 | Glutathione Peroxidase 7 | Protein Coding | 37 | GC01P052602 | 1.664083719 |
| ATP5F1C | ATP Synthase F1 Subunit Gamma | Protein Coding | 30 | GC10P007789 | 1.663707137 |
| PRB2 | Proline Rich Protein BstNI Subfamily 2 | Protein Coding | 28 | GC12M011391 | 1.66314888 |
| DGKH | Diacylglycerol Kinase Eta | Protein Coding | 37 | GC13P042040 | 1.661903858 |
| SERPINI2 | Serpin Family I Member 2 | Protein Coding | 35 | GC03M167441 | 1.660749912 |
| NLRC4 | NLR Family CARD Domain Containing 4 | Protein Coding | 39 | GC02M032224 | 1.660724878 |
| IL20RA | Interleukin 20 Receptor Subunit Alpha | Protein Coding | 35 | GC06M136999 | 1.660472631 |
| TTBK2 | Tau Tubulin Kinase 2 | Protein Coding | 39 | GC15M042738 | 1.66036272 |
| EBNA1BP2 | EBNA1 Binding Protein 2 | Protein Coding | 32 | GC01M043165 | 1.660308838 |
| LINC00544 | Long Intergenic Non-Protein Coding RNA 544 | RNA Gene | 13 | GC13P029935 | 1.660304189 |
| LLGL2 | LLGL Scribble Cell Polarity Complex Component 2 | Protein Coding | 38 | GC17P075525 | 1.660223246 |
| GBF1 | Golgi Brefeldin A Resistant Guanine Nucleotide Exchange Factor 1 | Protein Coding | 39 | GC10P102245 | 1.660079956 |
| FREM3 | FRAS1 Related Extracellular Matrix 3 | Protein Coding | 27 | GC04M143577 | 1.659706354 |
| CLP1 | Cleavage Factor Polyribonucleotide Kinase Subunit 1 | Protein Coding | 36 | GC11P057648 | 1.658490896 |
| SMG6 | SMG6 Nonsense Mediated MRNA Decay Factor | Protein Coding | 35 | GC17M002059 | 1.658480406 |
| DDAH2 | Dimethylarginine Dimethylaminohydrolase 2 | Protein Coding | 39 | GC06M031727 | 1.658450365 |
| MRM3 | Mitochondrial RRNA Methyltransferase 3 | Protein Coding | 30 | GC17P001460 | 1.657776713 |
| QRICH1 | Glutamine Rich 1 | Protein Coding | 31 | GC03M051016 | 1.657412767 |
| IRF2BP1 | Interferon Regulatory Factor 2 Binding Protein 1 | Protein Coding | 32 | GC19M063970 | 1.657323599 |
| GSTT2 | Glutathione S-Transferase Theta 2 (Gene/Pseudogene) | Protein Coding | 32 | GC22P023980 | 1.656877637 |
| GPR160 | G Protein-Coupled Receptor 160 | Protein Coding | 33 | GC03P170037 | 1.65682745 |
| SRPRB | SRP Receptor Subunit Beta | Protein Coding | 34 | GC03P133784 | 1.65680325 |
| PRKAG3 | Protein Kinase AMP-Activated Non-Catalytic Subunit Gamma 3 | Protein Coding | 40 | GC02M218823 | 1.655812263 |
| SLC39A14 | Solute Carrier Family 39 Member 14 | Protein Coding | 39 | GC08P022367 | 1.655316472 |
| MIR105-2 | MicroRNA 105-2 | RNA Gene | 15 | GC0XM152394 | 1.655093908 |
| ZSCAN5A | Zinc Finger And SCAN Domain Containing 5A | Protein Coding | 30 | GC19M064452 | 1.654692411 |
| RP1L1 | RP1 Like 1 | Protein Coding | 32 | GC08M010606 | 1.6540308 |
| SBF1 | SET Binding Factor 1 | Protein Coding | 38 | GC22M056251 | 1.653813839 |
| TMEM119 | Transmembrane Protein 119 | Protein Coding | 30 | GC12M108589 | 1.653178334 |
| CACNB1 | Calcium Voltage-Gated Channel Auxiliary Subunit Beta 1 | Protein Coding | 38 | GC17M039173 | 1.65313077 |
| UNC13B | Unc-13 Homolog B | Protein Coding | 37 | GC09P035161 | 1.65313077 |
| GNRHR2 | Gonadotropin Releasing Hormone Receptor 2 (Pseudogene) | Pseudogene | 28 | GC01P145919 | 1.653011084 |
| CREBZF | CREB/ATF BZIP Transcription Factor | Protein Coding | 31 | GC11M085657 | 1.652567983 |
| CTPS2 | CTP Synthase 2 | Protein Coding | 35 | GC0XM016587 | 1.652500391 |
| NOSIP | Nitric Oxide Synthase Interacting Protein | Protein Coding | 33 | GC19M049555 | 1.652103424 |
| DEGS1 | Delta 4-Desaturase, Sphingolipid 1 | Protein Coding | 36 | GC01P224175 | 1.651150942 |
| ALG11 | ALG11 Alpha-1,2-Mannosyltransferase | Protein Coding | 36 | GC13P052012 | 1.650861502 |
| GJD4 | Gap Junction Protein Delta 4 | Protein Coding | 32 | GC10P035611 | 1.650472403 |
| HIPK3 | Homeodomain Interacting Protein Kinase 3 | Protein Coding | 39 | GC11P033305 | 1.650300741 |
| NT5DC2 | 5'-Nucleotidase Domain Containing 2 | Protein Coding | 31 | GC03M052524 | 1.649947882 |
| PAOX | Polyamine Oxidase | Protein Coding | 35 | GC10P133379 | 1.649778366 |
| POU3F3 | POU Class 3 Homeobox 3 | Protein Coding | 37 | GC02P104855 | 1.64956975 |
| ALPK2 | Alpha Kinase 2 | Protein Coding | 31 | GC18M058481 | 1.649408102 |
| PHKA2 | Phosphorylase Kinase Regulatory Subunit Alpha 2 | Protein Coding | 40 | GC0XM018892 | 1.649277687 |
| PDCD2L | Programmed Cell Death 2 Like | Protein Coding | 31 | GC19P034404 | 1.649095058 |
| ZBTB38 | Zinc Finger And BTB Domain Containing 38 | Protein Coding | 33 | GC03P141324 | 1.648767829 |
| DPM2 | Dolichyl-Phosphate Mannosyltransferase Subunit 2, Regulatory | Protein Coding | 34 | GC09M127935 | 1.647870779 |
| WDR45B | WD Repeat Domain 45B | Protein Coding | 32 | GC17M082614 | 1.647658587 |
| ARIH1 | Ariadne RBR E3 Ubiquitin Protein Ligase 1 | Protein Coding | 35 | GC15P072474 | 1.647470713 |
| DNAI1 | Dynein Axonemal Intermediate Chain 1 | Protein Coding | 38 | GC09P034457 | 1.647381067 |
| LINC01128 | Long Intergenic Non-Protein Coding RNA 1128 | RNA Gene | 14 | GC01P003584 | 1.646902561 |
| CNTN2 | Contactin 2 | Protein Coding | 41 | GC01P205043 | 1.646254778 |
| FBXO15 | F-Box Protein 15 | Protein Coding | 32 | GC18M074073 | 1.646249294 |
| WEE2-AS1 | WEE2 Antisense RNA 1 | RNA Gene | 17 | GC07M141704 | 1.646012068 |
| IL12RB2 | Interleukin 12 Receptor Subunit Beta 2 | Protein Coding | 39 | GC01P067307 | 1.646004438 |
| ZIC4 | Zic Family Member 4 | Protein Coding | 34 | GC03M147386 | 1.645633936 |
| FAM219B | Family With Sequence Similarity 219 Member B | Protein Coding | 27 | GC15M074899 | 1.645109653 |
| KHK | Ketohexokinase | Protein Coding | 40 | GC02P027086 | 1.643878222 |
| ZNF787 | Zinc Finger Protein 787 | Protein Coding | 27 | GC19M064447 | 1.643473387 |
| OCEL1 | Occludin/ELL Domain Containing 1 | Protein Coding | 28 | GC19P063100 | 1.643354297 |
| LAD1 | Ladinin 1 | Protein Coding | 34 | GC01M201373 | 1.643224955 |
| TIGD6 | Tigger Transposable Element Derived 6 | Protein Coding | 30 | GC05M149993 | 1.642555714 |
| GTF2F1 | General Transcription Factor IIF Subunit 1 | Protein Coding | 35 | GC19M006408 | 1.642352104 |
| CYRIA | CYFIP Related Rac1 Interactor A | Protein Coding | 26 | GC02M016550 | 1.642140865 |
| STAU2 | Staufen Double-Stranded RNA Binding Protein 2 | Protein Coding | 34 | GC08M073421 | 1.641912937 |
| MRPL18 | Mitochondrial Ribosomal Protein L18 | Protein Coding | 32 | GC06P159789 | 1.641880989 |
| NPPC | Natriuretic Peptide C | Protein Coding | 38 | GC02M231921 | 1.64156127 |
| MIR551B | MicroRNA 551b | RNA Gene | 16 | GC03P168551 | 1.641244054 |
| DAD1 | Defender Against Cell Death 1 | Protein Coding | 36 | GC14M022565 | 1.640968323 |
| CCDC22 | Coiled-Coil Domain Containing 22 | Protein Coding | 35 | GC0XP050544 | 1.640967131 |
| REEP3 | Receptor Accessory Protein 3 | Protein Coding | 32 | GC10P063521 | 1.640925527 |
| ADAMTS6 | ADAM Metallopeptidase With Thrombospondin Type 1 Motif 6 | Protein Coding | 35 | GC05M065148 | 1.640905142 |
| VSTM4 | V-Set And Transmembrane Domain Containing 4 | Protein Coding | 29 | GC10M049014 | 1.640872955 |
| SLC5A7 | Solute Carrier Family 5 Member 7 | Protein Coding | 38 | GC02P107969 | 1.64008832 |
| PRDM10 | PR/SET Domain 10 | Protein Coding | 29 | GC11M129899 | 1.639730334 |
| SCN3A | Sodium Voltage-Gated Channel Alpha Subunit 3 | Protein Coding | 44 | GC02M165087 | 1.639565825 |
| TRNT1 | TRNA Nucleotidyl Transferase 1 | Protein Coding | 37 | GC03P003126 | 1.639342308 |
| MAT2B | Methionine Adenosyltransferase 2B | Protein Coding | 35 | GC05P163504 | 1.639271498 |
| PRKRA | Protein Activator Of Interferon Induced Protein Kinase EIF2AK2 | Protein Coding | 37 | GC02M178431 | 1.638573289 |
| AVPR1A | Arginine Vasopressin Receptor 1A | Protein Coding | 42 | GC12M063142 | 1.638402939 |
| ATP13A2 | ATPase Cation Transporting 13A2 | Protein Coding | 39 | GC01M016985 | 1.638136625 |
| MFAP1 | Microfibril Associated Protein 1 | Protein Coding | 31 | GC15M043804 | 1.63769722 |
| SPART | Spartin | Protein Coding | 32 | GC13M036356 | 1.63728714 |
| ADAM1A | ADAM Metallopeptidase Domain 1A (Pseudogene) | Pseudogene | 14 | GC12P111899 | 1.637276649 |
| HTR5A | 5-Hydroxytryptamine Receptor 5A | Protein Coding | 40 | GC07P155070 | 1.637264729 |
| GRK7 | G Protein-Coupled Receptor Kinase 7 | Protein Coding | 35 | GC03P141778 | 1.637220025 |
| SAA4 | Serum Amyloid A4, Constitutive | Protein Coding | 35 | GC11M018234 | 1.637144804 |
| MIR601 | MicroRNA 601 | RNA Gene | 16 | GC09M123402 | 1.637070298 |
| NAA11 | N-Alpha-Acetyltransferase 11, NatA Catalytic Subunit | Protein Coding | 29 | GC04M079155 | 1.636417866 |
| PELI1 | Pellino E3 Ubiquitin Protein Ligase 1 | Protein Coding | 37 | GC02M064092 | 1.636053085 |
| FNBP1 | Formin Binding Protein 1 | Protein Coding | 35 | GC09M129887 | 1.635988712 |
| GAREM1 | GRB2 Associated Regulator Of MAPK1 Subtype 1 | Protein Coding | 28 | GC18M032125 | 1.635871649 |
| P4HA3 | Prolyl 4-Hydroxylase Subunit Alpha 3 | Protein Coding | 36 | GC11M074235 | 1.635810852 |
| BTG3 | BTG Anti-Proliferation Factor 3 | Protein Coding | 34 | GC21M017595 | 1.635682583 |
| N4BP2L2 | NEDD4 Binding Protein 2 Like 2 | Protein Coding | 30 | GC13M032432 | 1.635586739 |

| | | | | | |
|---|---|---|---|---|---|
| RGSL1 | Regulator Of G Protein Signaling Like 1 | Protein Coding | 27 | GC01P182409 | 1.634986997 |
| PDK3 | Pyruvate Dehydrogenase Kinase 3 | Protein Coding | 42 | GC0XP024465 | 1.634435654 |
| LINC00518 | Long Intergenic Non-Protein Coding RNA 518 | RNA Gene | 19 | GC06M010428 | 1.634226441 |
| NIFK | Nucleolar Protein Interacting With The FHA Domain Of MKI67 | Protein Coding | 31 | GC02M121726 | 1.634068251 |
| RELCH | RAB11 Binding And LisH Domain, Coiled-Coil And HEAT Repeat Containing | Protein Coding | 26 | GC18P062188 | 1.633392334 |
| SEC61A1 | SEC61 Translocon Subunit Alpha 1 | Protein Coding | 38 | GC03P128051 | 1.633364677 |
| TXNDC5 | Thioredoxin Domain Containing 5 | Protein Coding | 34 | GC06M007893 | 1.633199453 |
| SNX16 | Sorting Nexin 16 | Protein Coding | 32 | GC08M081799 | 1.633055925 |
| SLC25A10 | Solute Carrier Family 25 Member 10 | Protein Coding | 38 | GC17P081712 | 1.632958412 |
| GCSAM | Germinal Center Associated Signaling And Motility | Protein Coding | 30 | GC03M112120 | 1.632348299 |
| CACNA2D3 | Calcium Voltage-Gated Channel Auxiliary Subunit Alpha2delta 3 | Protein Coding | 38 | GC03P054156 | 1.63193047 |
| ATXN2L | Ataxin 2 Like | Protein Coding | 34 | GC16P040092 | 1.631929636 |
| LYPLA2 | Lysophospholipase 2 | Protein Coding | 34 | GC01P023790 | 1.631861448 |
| ECPAS | Ecm29 Proteasome Adaptor And Scaffold | Protein Coding | 27 | GC09M111361 | 1.631605744 |
| CPEB2 | Cytoplasmic Polyadenylation Element Binding Protein 2 | Protein Coding | 31 | GC04P017724 | 1.631369233 |
| MRPS2 | Mitochondrial Ribosomal Protein S2 | Protein Coding | 32 | GC09P135499 | 1.631066799 |
| DDRGK1 | DDRGK Domain Containing 1 | Protein Coding | 34 | GC20M003315 | 1.630932808 |
| FOXJ2 | Forkhead Box J2 | Protein Coding | 30 | GC12P008032 | 1.630921841 |
| TMEM126A | Transmembrane Protein 126A | Protein Coding | 35 | GC11P085647 | 1.630384922 |
| PTRH1 | Peptidyl-TRNA Hydrolase 1 Homolog | Protein Coding | 30 | GC09M127797 | 1.630316019 |
| E4F1 | E4F Transcription Factor 1 | Protein Coding | 31 | GC16P002223 | 1.63025856 |
| BLOC1S1 | Biogenesis Of Lysosomal Organelles Complex 1 Subunit 1 | Protein Coding | 32 | GC12P055861 | 1.629226685 |
| ZNF175 | Zinc Finger Protein 175 | Protein Coding | 34 | GC19P051571 | 1.629046917 |
| RSBN1 | Round Spermatid Basic Protein 1 | Protein Coding | 29 | GC01M113761 | 1.62899065 |
| EAPP | E2F Associated Phosphoprotein | Protein Coding | 32 | GC14M034516 | 1.628881693 |
| UBASH3B | Ubiquitin Associated And SH3 Domain Containing B | Protein Coding | 36 | GC11P122655 | 1.628757 |
| GPATCH4 | G-Patch Domain Containing 4 (Gene/Pseudogene) | Protein Coding | 30 | GC01M156596 | 1.628741741 |
| RHOT1 | Ras Homolog Family Member T1 | Protein Coding | 38 | GC17P032142 | 1.628600717 |
| LENG8 | Leukocyte Receptor Cluster Member 8 | Protein Coding | 29 | GC19P064327 | 1.628137589 |
| COL5A3 | Collagen Type V Alpha 3 Chain | Protein Coding | 33 | GC19M009931 | 1.627815247 |
| NEIL3 | Nei Like DNA Glycosylase 3 | Protein Coding | 34 | GC04P177309 | 1.627563238 |
| STK25 | Serine/Threonine Kinase 25 | Protein Coding | 36 | GC02M241492 | 1.627455235 |
| C4BPA | Complement Component 4 Binding Protein Alpha | Protein Coding | 38 | GC01P207105 | 1.62650466 |
| H2BC12 | H2B Clustered Histone 12 | Protein Coding | 26 | GC06M063260 | 1.626299143 |
| UBE2J2 | Ubiquitin Conjugating Enzyme E2 J2 | Protein Coding | 36 | GC01M005528 | 1.625182152 |
| FAM110B | Family With Sequence Similarity 110 Member B | Protein Coding | 30 | GC08P057995 | 1.624428391 |
| DIS3L | DIS3 Like Exosome 3'-5' Exoribonuclease | Protein Coding | 34 | GC15P066293 | 1.624143481 |
| BAIAP3 | BAI1 Associated Protein 3 | Protein Coding | 34 | GC16P001333 | 1.624108553 |
| REPIN1 | Replication Initiator 1 | Protein Coding | 30 | GC07P150368 | 1.624027729 |
| MINK1 | Misshapen Like Kinase 1 | Protein Coding | 37 | GC17P004833 | 1.623952508 |
| RASSF3 | Ras Association Domain Family Member 3 | Protein Coding | 32 | GC12P064507 | 1.62391901 |
| NUP153 | Nucleoporin 153 | Protein Coding | 36 | GC06M017615 | 1.623563766 |
| POTEJ | POTE Ankyrin Domain Family Member J | Protein Coding | 22 | GC02P130611 | 1.623439789 |
| VCPIP1 | Valosin Containing Protein Interacting Protein 1 | Protein Coding | 32 | GC08M066628 | 1.622360706 |
| COX7B | Cytochrome C Oxidase Subunit 7B | Protein Coding | 35 | GC0XP077899 | 1.622309089 |
| ZNF428 | Zinc Finger Protein 428 | Protein Coding | 27 | GC19M043607 | 1.622236609 |
| CPEB4 | Cytoplasmic Polyadenylation Element Binding Protein 4 | Protein Coding | 33 | GC05P173888 | 1.622128963 |
| UGT2B10 | UDP Glucuronosyltransferase Family 2 Member B10 | Protein Coding | 38 | GC04P068816 | 1.622082591 |
| USP32 | Ubiquitin Specific Peptidase 32 | Protein Coding | 35 | GC17M060255 | 1.621576905 |
| DLG2 | Discs Large MAGUK Scaffold Protein 2 | Protein Coding | 38 | GC11M083455 | 1.621355534 |
| ZNF24 | Zinc Finger Protein 24 | Protein Coding | 35 | GC18M035332 | 1.620943427 |
| GRK1 | G Protein-Coupled Receptor Kinase 1 | Protein Coding | 38 | GC13P113645 | 1.620736003 |
| PTCD1 | Pentatricopeptide Repeat Domain 1 | Protein Coding | 31 | GC07M099429 | 1.620583534 |
| GCNT1 | Glucosaminyl (N-Acetyl) Transferase 1 | Protein Coding | 37 | GC09P076420 | 1.620052099 |
| RNF169 | Ring Finger Protein 169 | Protein Coding | 30 | GC11P074826 | 1.620038748 |
| TRIM62 | Tripartite Motif Containing 62 | Protein Coding | 33 | GC01M033152 | 1.619299531 |
| MTREX | Mtr4 Exosome RNA Helicase | Protein Coding | 30 | GC05P055308 | 1.619222641 |
| ATP2A1 | ATPase Sarcoplasmic/Endoplasmic Reticulum Ca2+ Transporting 1 | Protein Coding | 44 | GC16P040095 | 1.618852139 |
| LINC00324 | Long Intergenic Non-Protein Coding RNA 324 | RNA Gene | 18 | GC17M010280 | 1.618607283 |
| MIR939 | MicroRNA 939 | RNA Gene | 14 | GC08M144394 | 1.61854589 |
| ELMOD3 | ELMO Domain Containing 3 | Protein Coding | 34 | GC02P085354 | 1.618469 |
| ENO3 | Enolase 3 | Protein Coding | 43 | GC17P004948 | 1.618439794 |
| DHX57 | DExH-Box Helicase 57 | Protein Coding | 32 | GC02M038797 | 1.617962956 |
| TUBGCP2 | Tubulin Gamma Complex Associated Protein 2 | Protein Coding | 35 | GC10M133278 | 1.617885351 |
| PNPO | Pyridoxamine 5'-Phosphate Oxidase | Protein Coding | 40 | GC17P047941 | 1.617591023 |
| ELOA2 | Elongin A2 | Protein Coding | 26 | GC18M047105 | 1.617526054 |
| SGTA | Small Glutamine Rich Tetratricopeptide Repeat Co-Chaperone Alpha | Protein Coding | 33 | GC19M002754 | 1.617263675 |
| MYO15A | Myosin XVA | Protein Coding | 32 | GC17P018108 | 1.616531014 |
| CRYBB2 | Crystallin Beta B2 | Protein Coding | 36 | GC22P034788 | 1.616496086 |
| UBAC1 | UBA Domain Containing 1 | Protein Coding | 32 | GC09M135932 | 1.616124153 |
| PEX5L | Peroxisomal Biogenesis Factor 5 Like | Protein Coding | 31 | GC03M179794 | 1.615820885 |
| B4GAT1 | Beta-1,4-Glucuronyltransferase 1 | Protein Coding | 30 | GC11M066345 | 1.615651965 |
| SKOR1 | SKI Family Transcriptional Corepressor 1 | Protein Coding | 30 | GC15P067819 | 1.615579009 |
| CYP4X1 | Cytochrome P450 Family 4 Subfamily X Member 1 | Protein Coding | 32 | GC01P046961 | 1.61514008 |
| C1orf68 | Chromosome 1 Open Reading Frame 68 | Protein Coding | 26 | GC01P152772 | 1.614786386 |
| PPP1R18 | Protein Phosphatase 1 Regulatory Subunit 18 | Protein Coding | 31 | GC06M063524 | 1.614695311 |
| TRIM56 | Tripartite Motif Containing 56 | Protein Coding | 31 | GC07P101085 | 1.614123106 |
| ANKRD12 | Ankyrin Repeat Domain 12 | Protein Coding | 31 | GC18P009136 | 1.614107132 |
| OSBPL6 | Oxysterol Binding Protein Like 6 | Protein Coding | 34 | GC02P178194 | 1.613847971 |
| VPS33A | VPS33A Core Subunit Of CORVET And HOPS Complexes | Protein Coding | 36 | GC12M122229 | 1.613774419 |
| USP6NL | USP6 N-Terminal Like | Protein Coding | 36 | GC10M011453 | 1.61335969 |
| ST8 | Suppression Of Tumorigenicity 8 (Ovarian) | Genetic Locus | 2 | GC06U990118 | 1.613176703 |
| A4GALT | Alpha 1,4-Galactosyltransferase (P Blood Group) | Protein Coding | 38 | GC22M042692 | 1.612783909 |
| VDAC3 | Voltage Dependent Anion Channel 3 | Protein Coding | 39 | GC08P042392 | 1.612160683 |
| DENND2D | DENN Domain Containing 2D | Protein Coding | 30 | GC01M111185 | 1.61077261 |
| DRG2 | Developmentally Regulated GTP Binding Protein 2 | Protein Coding | 34 | GC17P018088 | 1.610634565 |
| NFRKB | Nuclear Factor Related To KappaB Binding Protein | Protein Coding | 35 | GC11M129863 | 1.610244632 |
| CDK5RAP1 | CDK5 Regulatory Subunit Associated Protein 1 | Protein Coding | 34 | GC20M033358 | 1.610216618 |
| NUP35 | Nucleoporin 35 | Protein Coding | 32 | GC02P183117 | 1.609717011 |
| HAL | Histidine Ammonia-Lyase | Protein Coding | 38 | GC12M095972 | 1.609534264 |
| VCAN-AS1 | VCAN Antisense RNA 1 | RNA Gene | 14 | GC05M083509 | 1.608911633 |
| HECW2 | HECT, C2 And WW Domain Containing E3 Ubiquitin Protein Ligase 2 | Protein Coding | 38 | GC02M196194 | 1.608200788 |
| GALK2 | Galactokinase 2 | Protein Coding | 36 | GC15P049155 | 1.607961297 |
| MAGOH | Mago Homolog, Exon Junction Complex Subunit | Protein Coding | 33 | GC01M053226 | 1.607960939 |
| TSPAN7 | Tetraspanin 7 | Protein Coding | 39 | GC0XP038561 | 1.607936621 |

| | | | | | |
|---|---|---|---|---|---|
| TAS1R3 | Taste 1 Receptor Member 3 | Protein Coding | 34 | GC01P001331 | 1.607762456 |
| INTS9 | Integrator Complex Subunit 9 | Protein Coding | 31 | GC08M028767 | 1.607664466 |
| ZNF143 | Zinc Finger Protein 143 | Protein Coding | 36 | GC11P009483 | 1.607370138 |
| RRP7BP | Ribosomal RNA Processing 7 Homolog B, Pseudogene | Pseudogene | 14 | GC22M042555 | 1.607262492 |
| TRAF3IP2 | TRAF3 Interacting Protein 2 | Protein Coding | 40 | GC06M111555 | 1.607234716 |
| GNPAT | Glyceronephosphate O-Acyltransferase | Protein Coding | 40 | GC01P231241 | 1.607092381 |
| EXOSC2 | Exosome Component 2 | Protein Coding | 37 | GC09P130693 | 1.606829524 |
| HCG4 | HLA Complex Group 4 | RNA Gene | 17 | GC06M063496 | 1.606183767 |
| TSPYL1 | TSPY Like 1 | Protein Coding | 38 | GC06M116268 | 1.605445862 |
| SMPD3 | Sphingomyelin Phosphodiesterase 3 | Protein Coding | 36 | GC16M068358 | 1.605445862 |
| MPZL1 | Myelin Protein Zero Like 1 | Protein Coding | 36 | GC01P167721 | 1.604990244 |
| MIR1275 | MicroRNA 1275 | RNA Gene | 18 | GC06M063738 | 1.604884148 |
| OVOL1 | Ovo Like Transcriptional Repressor 1 | Protein Coding | 34 | GC11P065787 | 1.604848504 |
| CCNQ | Cyclin Q | Protein Coding | 27 | GC0XM153588 | 1.604722857 |
| SARS1 | Seryl-TRNA Synthetase 1 | Protein Coding | 35 | GC01P109214 | 1.604181051 |
| RAB12 | RAB12, Member RAS Oncogene Family | Protein Coding | 31 | GC18P008600 | 1.603927851 |
| H2BC5 | H2B Clustered Histone 5 | Protein Coding | 28 | GC06P081038 | 1.60334444 |
| CCDC69 | Coiled-Coil Domain Containing 69 | Protein Coding | 30 | GC05M151181 | 1.602759004 |
| TTLL10 | Tubulin Tyrosine Ligase Like 10 | Protein Coding | 28 | GC01P001173 | 1.6021595 |
| ERFE | Erythroferrone | Protein Coding | 25 | GC02P238159 | 1.601958036 |
| BACE1 | Beta-Secretase 1 | Protein Coding | 43 | GC11M117285 | 1.601884604 |
| DFFB | DNA Fragmentation Factor Subunit Beta | Protein Coding | 39 | GC01P003857 | 1.601832151 |
| FAAP100 | FA Core Complex Associated Protein 100 | Protein Coding | 28 | GC17M081553 | 1.601323605 |
| FBXO45 | F-Box Protein 45 | Protein Coding | 29 | GC03P196568 | 1.601027012 |
| MIR194-2 | MicroRNA 194-2 | RNA Gene | 18 | GC11M087224 | 1.600523114 |
| PPP1R3F | Protein Phosphatase 1 Regulatory Subunit 3F | Protein Coding | 31 | GC0XP049269 | 1.600117803 |
| PLBD1 | Phospholipase B Domain Containing 1 | Protein Coding | 31 | GC12M014503 | 1.600011468 |
| TBL3 | Transducin Beta Like 3 | Protein Coding | 35 | GC16P010706 | 1.599964499 |
| SMPD2 | Sphingomyelin Phosphodiesterase 2 | Protein Coding | 36 | GC06P109440 | 1.599736214 |
| ARHGEF3 | Rho Guanine Nucleotide Exchange Factor 3 | Protein Coding | 37 | GC03M056736 | 1.599609256 |
| SWT1 | SWT1 RNA Endoribonuclease Homolog | Protein Coding | 30 | GC01P185127 | 1.599275827 |
| EXOC7 | Exocyst Complex Component 7 | Protein Coding | 36 | GC17M076080 | 1.599191546 |
| DUOX1 | Dual Oxidase 1 | Protein Coding | 38 | GC15P045129 | 1.599092007 |
| LOXL1-AS1 | LOXL1 Antisense RNA 1 | RNA Gene | 17 | GC15M081419 | 1.598889947 |
| CYP21A1P | Cytochrome P450 Family 21 Subfamily A Member 1, Pseudogene | Pseudogene | 15 | GC06P032005 | 1.598763704 |
| KANK1 | KN Motif And Ankyrin Repeat Domains 1 | Protein Coding | 38 | GC09P000474 | 1.598617554 |
| ZNF404 | Zinc Finger Protein 404 | Protein Coding | 27 | GC19M043872 | 1.598397493 |
| PRICKLE2 | Prickle Planar Cell Polarity Protein 2 | Protein Coding | 35 | GC03M064079 | 1.597995162 |
| TNRC18 | Trinucleotide Repeat Containing 18 | Protein Coding | 28 | GC07M005350 | 1.597705245 |
| FADS3 | Fatty Acid Desaturase 3 | Protein Coding | 33 | GC11M061873 | 1.597674131 |
| DNAJC10 | DnaJ Heat Shock Protein Family (Hsp40) Member C10 | Protein Coding | 33 | GC02P182716 | 1.597641468 |
| MIR3074 | MicroRNA 3074 | RNA Gene | 15 | GC09M095086 | 1.597343445 |
| HECTD3 | HECT Domain E3 Ubiquitin Protein Ligase 3 | Protein Coding | 31 | GC01M045002 | 1.596940637 |
| PNMA8A | PNMA Family Member 8A | Protein Coding | 25 | GC19M063990 | 1.596894979 |
| KLHL2 | Kelch Like Family Member 2 | Protein Coding | 34 | GC04P165207 | 1.596222997 |
| RPAP1 | RNA Polymerase II Associated Protein 1 | Protein Coding | 31 | GC15M041517 | 1.596005917 |
| INSL4 | Insulin Like 4 | Protein Coding | 31 | GC09P005231 | 1.595436692 |
| PIGV | Phosphatidylinositol Glycan Anchor Biosynthesis Class V | Protein Coding | 36 | GC01P026787 | 1.594939947 |
| POTEKP | POTE Ankyrin Domain Family Member K, Pseudogene | Pseudogene | 17 | GC02P131591 | 1.594668031 |
| CLPP | Caseinolytic Mitochondrial Matrix Peptidase Proteolytic Subunit | Protein Coding | 41 | GC19P006609 | 1.59464407 |
| WDR12 | WD Repeat Domain 12 | Protein Coding | 34 | GC02M202874 | 1.594360352 |
| TRIM3 | Tripartite Motif Containing 3 | Protein Coding | 32 | GC11M006450 | 1.594347596 |
| ZKSCAN8 | Zinc Finger With KRAB And SCAN Domains 8 | Protein Coding | 28 | GC06P028141 | 1.594142914 |
| REEP1 | Receptor Accessory Protein 1 | Protein Coding | 36 | GC02M086213 | 1.594016433 |
| BRPF3 | Bromodomain And PHD Finger Containing 3 | Protein Coding | 32 | GC06P080528 | 1.59391892 |
| SMU1 | SMU1 DNA Replication Regulator And Spliceosomal Factor | Protein Coding | 33 | GC09M033041 | 1.593294144 |
| PJA1 | Praja Ring Finger Ubiquitin Ligase 1 | Protein Coding | 35 | GC0XM069160 | 1.591992855 |
| CABP1 | Calcium Binding Protein 1 | Protein Coding | 32 | GC12P120642 | 1.591925383 |
| TRIB2 | Tribbles Pseudokinase 2 | Protein Coding | 36 | GC02P012717 | 1.591673017 |
| DHX33 | DEAH-Box Helicase 33 | Protein Coding | 30 | GC17M005440 | 1.591632962 |
| POLR2C | RNA Polymerase II Subunit C | Protein Coding | 36 | GC16P057462 | 1.590737104 |
| PCSK1N | Proprotein Convertase Subtilisin/Kexin Type 1 Inhibitor | Protein Coding | 30 | GC0XM048831 | 1.58991313 |
| ALG14 | ALG14 UDP-N-Acetylglucosaminyltransferase Subunit | Protein Coding | 36 | GC01M094974 | 1.5897789 |
| PF4V1 | Platelet Factor 4 Variant 1 | Protein Coding | 31 | GC04P073853 | 1.589376211 |
| NBPF12 | NBPF Member 12 | Protein Coding | 25 | GC01P146938 | 1.58932507 |
| DCST1 | DC-STAMP Domain Containing 1 | Protein Coding | 29 | GC01P155033 | 1.589315414 |
| SNHG32 | Small Nucleolar RNA Host Gene 32 | RNA Gene | 22 | GC06P083433 | 1.588949442 |
| LRCH3 | Leucine Rich Repeats And Calponin Homology Domain Containing 3 | Protein Coding | 30 | GC03P197791 | 1.588631153 |
| MNDA | Myeloid Cell Nuclear Differentiation Antigen | Protein Coding | 38 | GC01P158801 | 1.588513136 |
| STX6 | Syntaxin 6 | Protein Coding | 35 | GC01M180972 | 1.586874604 |
| ZNF274 | Zinc Finger Protein 274 | Protein Coding | 31 | GC19P064509 | 1.586825013 |
| YARS1 | Tyrosyl-TRNA Synthetase 1 | Protein Coding | 35 | GC01M032776 | 1.586777687 |
| RSBN1L | Round Spermatid Basic Protein 1 Like | Protein Coding | 28 | GC07P077696 | 1.586557627 |
| GLIS3 | GLIS Family Zinc Finger 3 | Protein Coding | 35 | GC09M003816 | 1.58652544 |
| ATP6AP2 | ATPase H+ Transporting Accessory Protein 2 | Protein Coding | 39 | GC0XP040582 | 1.586339593 |
| OPRL1 | Opioid Related Nociceptin Receptor 1 | Protein Coding | 42 | GC20P064080 | 1.586298108 |
| CFAP44 | Cilia And Flagella Associated Protein 44 | Protein Coding | 27 | GC03M113286 | 1.586140394 |
| MIR1296 | MicroRNA 1296 | RNA Gene | 17 | GC10M063372 | 1.586002111 |
| CDIPT | CDP-Diacylglycerol--Inositol 3-Phosphatidyltransferase | Protein Coding | 36 | GC16M036458 | 1.585870981 |
| ME2 | Malic Enzyme 2 | Protein Coding | 39 | GC18P050879 | 1.585258007 |
| SNAP23 | Synaptosome Associated Protein 23 | Protein Coding | 38 | GC15P042491 | 1.585162997 |
| TNNT1 | Troponin T1, Slow Skeletal Type | Protein Coding | 38 | GC19M055132 | 1.584902763 |
| COL21A1 | Collagen Type XXI Alpha 1 Chain | Protein Coding | 32 | GC06M064044 | 1.584323525 |
| GIPR | Gastric Inhibitory Polypeptide Receptor | Protein Coding | 39 | GC19P045668 | 1.584014773 |
| UGT3A2 | UDP Glycosyltransferase Family 3 Member A2 | Protein Coding | 35 | GC05M036036 | 1.583491087 |
| HYAL3 | Hyaluronidase 3 | Protein Coding | 36 | GC03M051096 | 1.583463907 |
| KCNK5 | Potassium Two Pore Domain Channel Subfamily K Member 5 | Protein Coding | 38 | GC06M063776 | 1.583413124 |
| NCBP2AS2 | NCBP2 Antisense 2 (Head To Head) | Protein Coding | 14 | GC03P196944 | 1.583373785 |
| ADD2 | Adducin 2 | Protein Coding | 37 | GC02M070626 | 1.58315587 |
| ERICH6B | Glutamate Rich 6B | Protein Coding | 23 | GC13M045534 | 1.583087564 |
| DCHS1 | Dachsous Cadherin-Related 1 | Protein Coding | 34 | GC11M006621 | 1.583042145 |
| ZFC3H1 | Zinc Finger C3H1-Type Containing | Protein Coding | 30 | GC12M071609 | 1.583031178 |
| AP1G1 | Adaptor Related Protein Complex 1 Subunit Gamma 1 | Protein Coding | 38 | GC16M071729 | 1.582723856 |
| ERLIN1 | ER Lipid Raft Associated 1 | Protein Coding | 39 | GC10M100150 | 1.582342386 |
| SSR1 | Signal Sequence Receptor Subunit 1 | Protein Coding | 35 | GC06M007268 | 1.582278848 |

| | | | | | |
|---|---|---|---|---|---|
| ZSCAN32 | Zinc Finger And SCAN Domain Containing 32 | Protein Coding | 26 | GC16M006847 | 1.582134008 |
| MIR665 | MicroRNA 665 | RNA Gene | 14 | GC14P109550 | 1.581655145 |
| DYNC2I1 | Dynein 2 Intermediate Chain 1 | Protein Coding | 29 | GC07P158839 | 1.581543446 |
| NEFM | Neurofilament Medium Chain | Protein Coding | 38 | GC08P024913 | 1.581368446 |
| MIR4457 | MicroRNA 4457 | RNA Gene | 10 | GC05M001311 | 1.581269741 |
| C2CD2L | C2CD2 Like | Protein Coding | 30 | GC11P119102 | 1.58091855 |
| LHX6 | LIM Homeobox 6 | Protein Coding | 34 | GC09M122202 | 1.580893874 |
| IFT43 | Intraflagellar Transport 43 | Protein Coding | 33 | GC14P075902 | 1.580690384 |
| BTN3A2 | Butyrophilin Subfamily 3 Member A2 | Protein Coding | 34 | GC06P026365 | 1.580355048 |
| UGGT1 | UDP-Glucose Glycoprotein Glucosyltransferase 1 | Protein Coding | 35 | GC02P128091 | 1.580345869 |
| DSTYK | Dual Serine/Threonine And Tyrosine Protein Kinase | Protein Coding | 37 | GC01M205111 | 1.580059528 |
| EXOSC6 | Exosome Component 6 | Protein Coding | 31 | GC16M070246 | 1.579468608 |
| DSEL | Dermatan Sulfate Epimerase Like | Protein Coding | 31 | GC18M067506 | 1.579394579 |
| MIR4753 | MicroRNA 4753 | RNA Gene | 11 | GC01M235190 | 1.578939438 |
| H3C14 | H3 Clustered Histone 14 | Protein Coding | 27 | GC01M151585 | 1.57856977 |
| CENPS | Centromere Protein S | Protein Coding | 29 | GC01P010467 | 1.578058004 |
| DCBLD1 | Discoidin, CUB And LCCL Domain Containing 1 | Protein Coding | 32 | GC06P117453 | 1.577750206 |
| PEX26 | Peroxisomal Biogenesis Factor 26 | Protein Coding | 36 | GC22P018459 | 1.577400446 |
| BLVRA | Biliverdin Reductase A | Protein Coding | 39 | GC07P043758 | 1.57723248 |
| BCKDK | Branched Chain Keto Acid Dehydrogenase Kinase | Protein Coding | 42 | GC16P040299 | 1.577224374 |
| LOC109363676 | BMP2 5' Regulatory Region | Biological Region | 2 | GC20P006763 | 1.576938868 |
| LIFR-AS1 | LIFR Antisense RNA 1 | RNA Gene | 14 | GC05P038559 | 1.576908827 |
| ILRUN | Inflammation And Lipid Regulator With UBA-Like And NBR1-Like Domains | Protein Coding | 26 | GC06M064503 | 1.57689774 |
| EEF1A1P5 | Eukaryotic Translation Elongation Factor 1 Alpha 1 Pseudogene 5 | Pseudogene | 13 | GC09P133019 | 1.576862097 |
| FKBP3 | FKBP Prolyl Isomerase 3 | Protein Coding | 34 | GC14M045115 | 1.575567007 |
| CTSO | Cathepsin O | Protein Coding | 35 | GC04M155924 | 1.575368524 |
| HES2 | Hes Family BHLH Transcription Factor 2 | Protein Coding | 29 | GC01M006412 | 1.575212598 |
| MIR153-2 | MicroRNA 153-2 | RNA Gene | 15 | GC07M157574 | 1.575178504 |
| ZHX2 | Zinc Fingers And Homeoboxes 2 | Protein Coding | 34 | GC08P122781 | 1.575162888 |
| FBXO3 | F-Box Protein 3 | Protein Coding | 34 | GC11M033740 | 1.57505393 |
| MYDGF | Myeloid Derived Growth Factor | Protein Coding | 29 | GC19M004641 | 1.574928522 |
| MIR942 | MicroRNA 942 | RNA Gene | 14 | GC01P117094 | 1.574770451 |
| RAB6B | RAB6B, Member RAS Oncogene Family | Protein Coding | 35 | GC03M133824 | 1.574761152 |
| ASPRV1 | Aspartic Peptidase Retroviral Like 1 | Protein Coding | 32 | GC02M069932 | 1.574653625 |
| MAPK8IP3 | Mitogen-Activated Protein Kinase 8 Interacting Protein 3 | Protein Coding | 36 | GC16P001706 | 1.5744524 |
| ARHGAP27 | Rho GTPase Activating Protein 27 | Protein Coding | 32 | GC17M045393 | 1.574346423 |
| RNPS1 | RNA Binding Protein With Serine Rich Domain 1 | Protein Coding | 33 | GC16M002253 | 1.573961496 |
| RBM26 | RNA Binding Motif Protein 26 | Protein Coding | 33 | GC13M079311 | 1.573778152 |
| PRRX2 | Paired Related Homeobox 2 | Protein Coding | 32 | GC09P129665 | 1.572896719 |
| ZNF598 | Zinc Finger Protein 598, E3 Ubiquitin Ligase | Protein Coding | 30 | GC16M006687 | 1.57286799 |
| DAB1 | DAB Adaptor Protein 1 | Protein Coding | 38 | GC01M056994 | 1.572659016 |
| TMEM45A | Transmembrane Protein 45A | Protein Coding | 29 | GC03P100492 | 1.572498918 |
| LYNX1 | Ly6/Neurotoxin 1 | Protein Coding | 30 | GC08M142987 | 1.571629643 |
| FMOD | Fibromodulin | Protein Coding | 38 | GC01M203340 | 1.571332455 |
| GATM | Glycine Amidinotransferase | Protein Coding | 40 | GC15M045361 | 1.570506334 |
| PSMB3 | Proteasome 20S Subunit Beta 3 | Protein Coding | 36 | GC17P038752 | 1.570450902 |
| TNFAIP1 | TNF Alpha Induced Protein 1 | Protein Coding | 38 | GC17P028335 | 1.569712996 |
| TMEM26 | Transmembrane Protein 26 | Protein Coding | 28 | GC10M061406 | 1.569449544 |
| TRABD | TraB Domain Containing | Protein Coding | 30 | GC22P050185 | 1.569265604 |
| SNX32 | Sorting Nexin 32 | Protein Coding | 33 | GC11P065833 | 1.569243789 |
| ANKRD6 | Ankyrin Repeat Domain 6 | Protein Coding | 34 | GC06P089433 | 1.568539977 |
| MIR655 | MicroRNA 655 | RNA Gene | 14 | GC14P109548 | 1.568338633 |
| PICK1 | Protein Interacting With PRKCA 1 | Protein Coding | 38 | GC22P038056 | 1.568104982 |
| GLRA1 | Glycine Receptor Alpha 1 | Protein Coding | 43 | GC05M151822 | 1.568042755 |
| ZWINT | ZW10 Interacting Kinetochore Protein | Protein Coding | 33 | GC10M056357 | 1.567683458 |
| RALGPS1 | Ral GEF With PH Domain And SH3 Binding Motif 1 | Protein Coding | 33 | GC09P126914 | 1.566880941 |
| MIR496 | MicroRNA 496 | RNA Gene | 15 | GC14P109543 | 1.566570401 |
| CATSPER2 | Cation Channel Sperm Associated 2 | Protein Coding | 38 | GC15M043628 | 1.566473961 |
| CEP85 | Centrosomal Protein 85 | Protein Coding | 30 | GC01P026234 | 1.565909863 |
| ARPC4 | Actin Related Protein 2/3 Complex Subunit 4 | Protein Coding | 34 | GC03P009792 | 1.565719485 |
| MLKL | Mixed Lineage Kinase Domain Like Pseudokinase | Protein Coding | 37 | GC16M074672 | 1.565574408 |
| PHF2 | PHD Finger Protein 2 | Protein Coding | 36 | GC09P093576 | 1.565372825 |
| MIR641 | MicroRNA 641 | RNA Gene | 15 | GC19M040282 | 1.565364122 |
| COX7A2 | Cytochrome C Oxidase Subunit 7A2 | Protein Coding | 34 | GC06M075237 | 1.565141559 |
| VPS33B | VPS33B Late Endosome And Lysosome Associated | Protein Coding | 38 | GC15M090998 | 1.564926386 |
| RLF | RLF Zinc Finger | Protein Coding | 32 | GC01P040161 | 1.564915061 |
| ESYT1 | Extended Synaptotagmin 1 | Protein Coding | 34 | GC12P057160 | 1.564901233 |
| SFXN1 | Sideroflexin 1 | Protein Coding | 35 | GC05P175477 | 1.564826012 |
| MIR3613 | MicroRNA 3613 | RNA Gene | 14 | GC13M049996 | 1.564476013 |
| AGPAT1 | 1-Acylglycerol-3-Phosphate O-Acyltransferase 1 | Protein Coding | 37 | GC06M032168 | 1.564143181 |
| GBAP1 | Glucosylceramidase Beta Pseudogene 1 | Pseudogene | 13 | GC01M155213 | 1.563652992 |
| RRP12 | Ribosomal RNA Processing 12 Homolog | Protein Coding | 32 | GC10M097356 | 1.563375235 |
| POLR2G | RNA Polymerase II Subunit G | Protein Coding | 33 | GC11P062773 | 1.563073397 |
| DHRS3 | Dehydrogenase/Reductase 3 | Protein Coding | 37 | GC01M012567 | 1.562765837 |
| CPVL | Carboxypeptidase Vitellogenic Like | Protein Coding | 36 | GC07M028995 | 1.56257987 |
| FOLR3 | Folate Receptor Gamma | Protein Coding | 36 | GC11P072114 | 1.56252408 |
| SPTBN4 | Spectrin Beta, Non-Erythrocytic 4 | Protein Coding | 35 | GC19P040466 | 1.562147021 |
| LARS2 | Leucyl-TRNA Synthetase 2, Mitochondrial | Protein Coding | 39 | GC03P046366 | 1.561892748 |
| H2AJ | H2A.J Histone | Protein Coding | 26 | GC12P020180 | 1.56099844 |
| TEX264 | Testis Expressed 264, ER-Phagy Receptor | Protein Coding | 30 | GC03P051663 | 1.560791969 |
| UFM1 | Ubiquitin Fold Modifier 1 | Protein Coding | 35 | GC13P038349 | 1.560392857 |
| LRCH4 | Leucine Rich Repeats And Calponin Homology Domain Containing 4 | Protein Coding | 31 | GC07M100574 | 1.560239077 |
| CMTR1 | Cap Methyltransferase 1 | Protein Coding | 30 | GC06P080547 | 1.559515357 |
| FOXO6 | Forkhead Box O6 | Protein Coding | 28 | GC01P041361 | 1.559058189 |
| TAF9 | TATA-Box Binding Protein Associated Factor 9 | Protein Coding | 33 | GC05M069364 | 1.558743477 |
| SPATA2 | Spermatogenesis Associated 2 | Protein Coding | 30 | GC20M049903 | 1.558722019 |
| LSM12 | LSM12 Homolog | Protein Coding | 30 | GC17M044034 | 1.557665706 |
| ZDHHC14 | Zinc Finger DHHC-Type Palmitoyltransferase 14 | Protein Coding | 33 | GC06P157381 | 1.557570219 |
| ISCU | Iron-Sulfur Cluster Assembly Enzyme | Protein Coding | 40 | GC12P108561 | 1.557555437 |
| DAOA | D-Amino Acid Oxidase Activator | Protein Coding | 31 | GC13P105465 | 1.557497859 |
| UBE2E3 | Ubiquitin Conjugating Enzyme E2 E3 | Protein Coding | 36 | GC02P180967 | 1.557384253 |
| PHAX | Phosphorylated Adaptor For RNA Export | Protein Coding | 31 | GC05P126600 | 1.557327747 |
| TMX1 | Thioredoxin Related Transmembrane Protein 1 | Protein Coding | 34 | GC14P051240 | 1.557294488 |
| USP13 | Ubiquitin Specific Peptidase 13 | Protein Coding | 40 | GC03P179652 | 1.556813717 |
| CRACDL | CRACD Like | Protein Coding | 23 | GC02M098795 | 1.556660414 |

| | | | | | |
|---|---|---|---|---|---|
| CEP128 | Centrosomal Protein 128 | Protein Coding | 30 | GC14M080476 | 1.556646943 |
| FAM50B | Family With Sequence Similarity 50 Member B | Protein Coding | 27 | GC06P003906 | 1.556188583 |
| HLA-DPB2 | Major Histocompatibility Complex, Class II, DP Beta 2 (Pseudogene) | Pseudogene | 18 | GC06P080404 | 1.55609262 |
| PWWP2B | PWWP Domain Containing 2B | Protein Coding | 27 | GC10P132397 | 1.555864573 |
| TSFM | Ts Translation Elongation Factor, Mitochondrial | Protein Coding | 39 | GC12P057778 | 1.555456638 |
| TSPAN5 | Tetraspanin 5 | Protein Coding | 36 | GC04M098470 | 1.555442333 |
| AP4M1 | Adaptor Related Protein Complex 4 Subunit Mu 1 | Protein Coding | 35 | GC07P100101 | 1.55532074 |
| BAG4 | BAG Cochaperone 4 | Protein Coding | 34 | GC08P038176 | 1.554773808 |
| UCK2 | Uridine-Cytidine Kinase 2 | Protein Coding | 36 | GC01P165796 | 1.554719925 |
| DPF3 | Double PHD Fingers 3 | Protein Coding | 30 | GC14M072610 | 1.554532766 |
| LRRN2 | Leucine Rich Repeat Neuronal 2 | Protein Coding | 32 | GC01M204586 | 1.554272175 |
| P2RY14 | Purinergic Receptor P2Y14 | Protein Coding | 38 | GC03M151212 | 1.554200888 |
| PAM | Peptidylglycine Alpha-Amidating Monooxygenase | Protein Coding | 39 | GC05P102753 | 1.554136753 |
| SLC35A5 | Solute Carrier Family 35 Member A5 | Protein Coding | 30 | GC03P112561 | 1.553987145 |
| LAMTOR1 | Late Endosomal/Lysosomal Adaptor, MAPK And MTOR Activator 1 | Protein Coding | 29 | GC11M072085 | 1.553843975 |
| RIPPLY2 | Ripply Transcriptional Repressor 2 | Protein Coding | 32 | GC06P083854 | 1.553682685 |
| PITPNM1 | Phosphatidylinositol Transfer Protein Membrane Associated 1 | Protein Coding | 35 | GC11M087392 | 1.553577662 |
| MARCHF8 | Membrane Associated Ring-CH-Type Finger 8 | Protein Coding | 27 | GC10M046306 | 1.5529387 |
| C19orf53 | Chromosome 19 Open Reading Frame 53 | Protein Coding | 26 | GC19P013774 | 1.552571058 |
| DLX6 | Distal-Less Homeobox 6 | Protein Coding | 35 | GC07P097005 | 1.552387238 |
| C3AR1 | Complement C3a Receptor 1 | Protein Coding | 38 | GC12M008058 | 1.552217007 |
| GDPD5 | Glycerophosphodiester Phosphodiesterase Domain Containing 5 | Protein Coding | 33 | GC11M087643 | 1.552144051 |
| KMT5C | Lysine Methyltransferase 5C | Protein Coding | 29 | GC19P064362 | 1.551339388 |
| SCIN | Scinderin | Protein Coding | 33 | GC07P012570 | 1.551323295 |
| LRRC31 | Leucine Rich Repeat Containing 31 | Protein Coding | 27 | GC03M169839 | 1.551317096 |
| MAP3K12 | Mitogen-Activated Protein Kinase Kinase Kinase 12 | Protein Coding | 40 | GC12M053479 | 1.55126524 |
| NDUFA10 | NADH:Ubiquinone Oxidoreductase Subunit A10 | Protein Coding | 40 | GC02M239893 | 1.551163197 |
| CMAS | Cytidine Monophosphate N-Acetylneuraminic Acid Synthetase | Protein Coding | 32 | GC12P022046 | 1.550952911 |
| AP1M2 | Adaptor Related Protein Complex 1 Subunit Mu 2 | Protein Coding | 36 | GC19M010572 | 1.550857306 |
| CBLC | Cbl Proto-Oncogene C | Protein Coding | 35 | GC19P044777 | 1.550626278 |
| CYFIP2 | Cytoplasmic FMR1 Interacting Protein 2 | Protein Coding | 38 | GC05P157267 | 1.55024004 |
| OXLD1 | Oxidoreductase Like Domain Containing 1 | Protein Coding | 23 | GC17M081665 | 1.550234914 |
| RTN1 | Reticulon 1 | Protein Coding | 34 | GC14M059595 | 1.550204635 |
| C12orf29 | Chromosome 12 Open Reading Frame 29 | Protein Coding | 30 | GC12P088033 | 1.550163269 |
| PCGF6 | Polycomb Group Ring Finger 6 | Protein Coding | 31 | GC10M103302 | 1.550104856 |
| NCAM2 | Neural Cell Adhesion Molecule 2 | Protein Coding | 36 | GC21P020998 | 1.549907923 |
| CDKN2AIP | CDKN2A Interacting Protein | Protein Coding | 31 | GC04P183444 | 1.549603701 |
| ZPR1 | ZPR1 Zinc Finger | Protein Coding | 33 | GC11M116777 | 1.549522638 |
| IQSEC1 | IQ Motif And Sec7 Domain ArfGEF 1 | Protein Coding | 36 | GC03M020196 | 1.549268365 |
| ZBED6 | Zinc Finger BED-Type Containing 6 | Protein Coding | 22 | GC01P203796 | 1.549149275 |
| ULK3 | Unc-51 Like Kinase 3 | Protein Coding | 36 | GC15M074836 | 1.54914856 |
| TRMT1L | TRNA Methyltransferase 1 Like | Protein Coding | 27 | GC01M185118 | 1.548568368 |
| TCF25 | Transcription Factor 25 | Protein Coding | 34 | GC16P089873 | 1.548236012 |
| COG7 | Component Of Oligomeric Golgi Complex 7 | Protein Coding | 35 | GC16M023388 | 1.548193932 |
| EMID1 | EMI Domain Containing 1 | Protein Coding | 31 | GC22P029205 | 1.548156857 |
| CCDC120 | Coiled-Coil Domain Containing 120 | Protein Coding | 30 | GC0XP050534 | 1.547990799 |
| CLIC3 | Chloride Intracellular Channel 3 | Protein Coding | 34 | GC09M136994 | 1.547901154 |
| MAP1A | Microtubule Associated Protein 1A | Protein Coding | 36 | GC15P043516 | 1.547855139 |
| CENPT | Centromere Protein T | Protein Coding | 35 | GC16M067828 | 1.547374129 |
| LSG1 | Large 60S Subunit Nuclear Export GTPase 1 | Protein Coding | 30 | GC03M194640 | 1.54733181 |
| PPP1R11 | Protein Phosphatase 1 Regulatory Inhibitor Subunit 11 | Protein Coding | 31 | GC06P080316 | 1.546942472 |
| SBSN | Suprabasin | Protein Coding | 29 | GC19M065496 | 1.546934605 |
| GNGT1 | G Protein Subunit Gamma Transducin 1 | Protein Coding | 37 | GC07P093591 | 1.546756506 |
| DECR2 | 2,4-Dienoyl-CoA Reductase 2 | Protein Coding | 34 | GC16P010634 | 1.546620369 |
| SETD1B | SET Domain Containing 1B, Histone Lysine Methyltransferase | Protein Coding | 34 | GC12P125875 | 1.546483994 |
| FYCO1 | FYVE And Coiled-Coil Domain Autophagy Adaptor 1 | Protein Coding | 36 | GC03M045917 | 1.546139717 |
| CASTOR3 | CASTOR Family Member 3 | Pseudogene | 23 | GC07M101732 | 1.545912623 |
| SLC12A7 | Solute Carrier Family 12 Member 7 | Protein Coding | 40 | GC05M001050 | 1.54572773 |
| GADD45GIP1 | GADD45G Interacting Protein 1 | Protein Coding | 31 | GC19M012953 | 1.545420885 |
| COL6A2 | Collagen Type VI Alpha 2 Chain | Protein Coding | 40 | GC21P046098 | 1.545195341 |
| RPIA | Ribose 5-Phosphate Isomerase A | Protein Coding | 38 | GC02P088691 | 1.545093775 |
| PLIN3 | Perilipin 3 | Protein Coding | 37 | GC19M004852 | 1.544977665 |
| SRSF4 | Serine And Arginine Rich Splicing Factor 4 | Protein Coding | 34 | GC01M029147 | 1.544759274 |
| COA3 | Cytochrome C Oxidase Assembly Factor 3 | Protein Coding | 30 | GC17M042795 | 1.544616342 |
| MIR650 | MicroRNA 650 | RNA Gene | 13 | GC22P022822 | 1.544388056 |
| ARL6 | ADP Ribosylation Factor Like GTPase 6 | Protein Coding | 38 | GC03P097764 | 1.543996096 |
| TRAPPC1 | Trafficking Protein Particle Complex Subunit 1 | Protein Coding | 33 | GC17M007930 | 1.543973565 |
| CRAMP1 | Cramped Chromatin Regulator Homolog 1 | Protein Coding | 25 | GC16P010678 | 1.543852806 |
| UBXN7 | UBX Domain Protein 7 | Protein Coding | 29 | GC03M196347 | 1.543605804 |
| TRMT1 | TRNA Methyltransferase 1 | Protein Coding | 38 | GC19M013104 | 1.543282747 |
| DNAJB2 | DnaJ Heat Shock Protein Family (Hsp40) Member B2 | Protein Coding | 38 | GC02P219279 | 1.543092966 |
| SH3GLB2 | SH3 Domain Containing GRB2 Like, Endophilin B2 | Protein Coding | 34 | GC09M129007 | 1.542998195 |
| ZSCAN16 | Zinc Finger And SCAN Domain Containing 16 | Protein Coding | 28 | GC06P079969 | 1.542961597 |
| CORO2B | Coronin 2B | Protein Coding | 34 | GC15P114660 | 1.542922258 |
| NKX2-3 | NK2 Homeobox 3 | Protein Coding | 35 | GC10P099532 | 1.542771816 |
| TNNI1 | Troponin I1, Slow Skeletal Type | Protein Coding | 35 | GC01M201404 | 1.542742252 |
| UMOD | Uromodulin | Protein Coding | 39 | GC16M020344 | 1.542663097 |
| CHI3L2 | Chitinase 3 Like 2 | Protein Coding | 34 | GC01P111201 | 1.541810751 |
| CYP2S1 | Cytochrome P450 Family 2 Subfamily S Member 1 | Protein Coding | 38 | GC19P041193 | 1.541810155 |
| MID2 | Midline 2 | Protein Coding | 35 | GC0XP107825 | 1.541474462 |
| NSMCE1 | NSE1 Homolog, SMC5-SMC6 Complex Component | Protein Coding | 31 | GC16M027227 | 1.541367412 |
| AGAP1 | ArfGAP With GTPase Domain, Ankyrin Repeat And PH Domain 1 | Protein Coding | 35 | GC02P235494 | 1.539585471 |
| NCBP3 | Nuclear Cap Binding Subunit 3 | Protein Coding | 26 | GC17M004117 | 1.539321661 |
| CEBPE | CCAAT Enhancer Binding Protein Epsilon | Protein Coding | 38 | GC14M023117 | 1.539295197 |
| MAST1 | Microtubule Associated Serine/Threonine Kinase 1 | Protein Coding | 38 | GC19P013684 | 1.539167404 |
| MTMR4 | Myotubularin Related Protein 4 | Protein Coding | 36 | GC17M058489 | 1.538867474 |
| RAB40B | RAB40B, Member RAS Oncogene Family | Protein Coding | 31 | GC17M082654 | 1.538421154 |
| TPT1-AS1 | TPT1 Antisense RNA 1 | RNA Gene | 16 | GC13P045341 | 1.538128376 |
| NDUFS7 | NADH:Ubiquinone Oxidoreductase Core Subunit S7 | Protein Coding | 42 | GC19P002589 | 1.537332773 |
| RAD51-AS1 | RAD51 Antisense RNA 1 | RNA Gene | 15 | GC15M040686 | 1.536691427 |
| ATP5MG | ATP Synthase Membrane Subunit G | Protein Coding | 30 | GC11P118407 | 1.536477923 |
| CNOT2 | CCR4-NOT Transcription Complex Subunit 2 | Protein Coding | 37 | GC12P070242 | 1.536474943 |
| NLRX1 | NLR Family Member X1 | Protein Coding | 35 | GC11P119166 | 1.536093235 |
| OTULIN | OTU Deubiquitinase With Linear Linkage Specificity | Protein Coding | 35 | GC05P014667 | 1.535996795 |
| NLRP2 | NLR Family Pyrin Domain Containing 2 | Protein Coding | 37 | GC19P054953 | 1.535845399 |

| | | | | | |
|---|---|---|---|---|---|
| LUZP2 | Leucine Zipper Protein 2 | Protein Coding | 31 | GC11P024518 | 1.535500288 |
| IGLL1 | Immunoglobulin Lambda Like Polypeptide 1 | Protein Coding | 39 | GC22M023573 | 1.535268068 |
| NBPF26 | NBPF Member 26 | Protein Coding | 15 | GC01P120729 | 1.535198808 |
| RAB4A | RAB4A, Member RAS Oncogene Family | Protein Coding | 36 | GC01P229271 | 1.534907103 |
| CHDH | Choline Dehydrogenase | Protein Coding | 36 | GC03M053812 | 1.534669995 |
| HIGD1A | HIG1 Hypoxia Inducible Domain Family Member 1A | Protein Coding | 34 | GC03M042782 | 1.534633756 |
| ATG4D | Autophagy Related 4D Cysteine Peptidase | Protein Coding | 33 | GC19P010543 | 1.534577847 |
| BCAT2 | Branched Chain Amino Acid Transaminase 2 | Protein Coding | 41 | GC19M048795 | 1.534565687 |
| CIAPIN1 | Cytokine Induced Apoptosis Inhibitor 1 | Protein Coding | 34 | GC16M057428 | 1.534495711 |
| CCDC9B | Coiled-Coil Domain Containing 9B | Protein Coding | 21 | GC15M040357 | 1.534322739 |
| MRPS5 | Mitochondrial Ribosomal Protein S5 | Protein Coding | 31 | GC02M097953 | 1.534231424 |
| CSRP2 | Cysteine And Glycine Rich Protein 2 | Protein Coding | 35 | GC12M076859 | 1.534157991 |
| AQR | Aquarius Intron-Binding Spliceosomal Factor | Protein Coding | 32 | GC15M034851 | 1.533968568 |
| SYTL4 | Synaptotagmin Like 4 | Protein Coding | 34 | GC0XM100674 | 1.533871293 |
| NDUFA5 | NADH:Ubiquinone Oxidoreductase Subunit A5 | Protein Coding | 37 | GC07M123536 | 1.533653021 |
| APOC1P1 | Apolipoprotein C1 Pseudogene 1 | Pseudogene | 12 | GC19P044926 | 1.533576012 |
| LCLAT1 | Lysocardiolipin Acyltransferase 1 | Protein Coding | 33 | GC02P030447 | 1.533187866 |
| SPSB1 | SplA/Ryanodine Receptor Domain And SOCS Box Containing 1 | Protein Coding | 34 | GC01P009292 | 1.532994986 |
| ITFG1 | Integrin Alpha FG-GAP Repeat Containing 1 | Protein Coding | 34 | GC16M047156 | 1.532941103 |
| OR1E2 | Olfactory Receptor Family 1 Subfamily E Member 2 | Protein Coding | 29 | GC17M003432 | 1.532764673 |
| TCF19 | Transcription Factor 19 | Protein Coding | 36 | GC06P080341 | 1.532405376 |
| DDX54 | DEAD-Box Helicase 54 | Protein Coding | 33 | GC12M113157 | 1.532347083 |
| HFM1 | Helicase For Meiosis 1 | Protein Coding | 36 | GC01M091260 | 1.532102227 |
| UTP3 | UTP3 Small Subunit Processome Component | Protein Coding | 30 | GC04P070688 | 1.532078743 |
| RGS10 | Regulator Of G Protein Signaling 10 | Protein Coding | 39 | GC10M119499 | 1.53184545 |
| DQ485454 | | RNA Gene | 6 | GC09P022031 | 1.531757236 |
| HSALNG0070392 | | RNA Gene | 5 | GC09M021932 | 1.531757236 |
| KLRC2 | Killer Cell Lectin Like Receptor C2 | Protein Coding | 35 | GC12M020446 | 1.531633139 |
| MIR3619 | MicroRNA 3619 | RNA Gene | 14 | GC22P046091 | 1.531576395 |
| POC5 | POC5 Centriolar Protein | Protein Coding | 30 | GC05M075674 | 1.531098127 |
| MRTO4 | MRT4 Homolog, Ribosome Maturation Factor | Protein Coding | 30 | GC01P019251 | 1.531059504 |
| TTLL1 | TTL Family Tubulin Polyglutamylase Complex Subunit L1 | Protein Coding | 35 | GC22M043039 | 1.530814528 |
| LRIG2 | Leucine Rich Repeats And Immunoglobulin Like Domains 2 | Protein Coding | 37 | GC01P113073 | 1.530801773 |
| SLC25A12 | Solute Carrier Family 25 Member 12 | Protein Coding | 42 | GC02M171783 | 1.530777574 |
| NFE2L1 | NFE2 Like BZIP Transcription Factor 1 | Protein Coding | 39 | GC17P053418 | 1.530548811 |
| ORM2 | Orosomucoid 2 | Protein Coding | 34 | GC09P114329 | 1.530296564 |
| UTP18 | UTP18 Small Subunit Processome Component | Protein Coding | 31 | GC17P051260 | 1.529759169 |
| APTR | Alu-Mediated CDKN1A/P21 Transcriptional Regulator | RNA Gene | 15 | GC07M077477 | 1.529700994 |
| TOGARAM1 | TOG Array Regulator Of Axonemal Microtubules 1 | Protein Coding | 25 | GC14P044963 | 1.529432774 |
| POLD4 | DNA Polymerase Delta 4, Accessory Subunit | Protein Coding | 32 | GC11M067350 | 1.528992057 |
| DIXDC1 | DIX Domain Containing 1 | Protein Coding | 32 | GC11P111927 | 1.52811265 |
| DCAF6 | DDB1 And CUL4 Associated Factor 6 | Protein Coding | 31 | GC01P167935 | 1.527916193 |
| KXD1 | KxDL Motif Containing 1 | Protein Coding | 31 | GC19P018557 | 1.527625203 |
| THAP4 | THAP Domain Containing 4 | Protein Coding | 30 | GC02M241584 | 1.52756238 |
| RPH3A | Rabphilin 3A | Protein Coding | 35 | GC12P112570 | 1.527212381 |
| ZNF83 | Zinc Finger Protein 83 | Protein Coding | 30 | GC19M052594 | 1.526994705 |
| USP27X | Ubiquitin Specific Peptidase 27 X-Linked | Protein Coding | 28 | GC0XP049879 | 1.526992083 |
| TUBE1 | Tubulin Epsilon 1 | Protein Coding | 35 | GC06M112070 | 1.526343822 |
| USP3 | Ubiquitin Specific Peptidase 3 | Protein Coding | 35 | GC15P063504 | 1.525950074 |
| ACOXL | Acyl-CoA Oxidase Like | Protein Coding | 29 | GC02P110732 | 1.525831461 |
| BET1 | Bet1 Golgi Vesicular Membrane Trafficking Protein | Protein Coding | 33 | GC07M093962 | 1.525627851 |
| TRAPPC10 | Trafficking Protein Particle Complex Subunit 10 | Protein Coding | 33 | GC21P044012 | 1.524502754 |
| RBP2 | Retinol Binding Protein 2 | Protein Coding | 35 | GC03M139452 | 1.524240255 |
| CEP162 | Centrosomal Protein 162 | Protein Coding | 27 | GC06M084124 | 1.524063945 |
| GTPBP10 | GTP Binding Protein 10 | Protein Coding | 28 | GC07P090336 | 1.524027109 |
| MVB12A | Multivesicular Body Subunit 12A | Protein Coding | 28 | GC19P065251 | 1.523409128 |
| AHSA1 | Activator Of HSP90 ATPase Activity 1 | Protein Coding | 34 | GC14P077457 | 1.522802353 |
| DNTTIP2 | Deoxynucleotidyltransferase Terminal Interacting Protein 2 | Protein Coding | 30 | GC01M093866 | 1.522788763 |
| CLN5 | CLN5 Intracellular Trafficking Protein | Protein Coding | 37 | GC13P076990 | 1.522576571 |
| JADE1 | Jade Family PHD Finger 1 | Protein Coding | 32 | GC04P128809 | 1.522206545 |
| TRAM1 | Translocation Associated Membrane Protein 1 | Protein Coding | 35 | GC08M070573 | 1.52196908 |
| IRX4 | Iroquois Homeobox 4 | Protein Coding | 34 | GC05M001877 | 1.52158463 |
| MIR300 | MicroRNA 300 | RNA Gene | 13 | GC14P109516 | 1.521583796 |
| MIR1180 | MicroRNA 1180 | RNA Gene | 17 | GC17M019344 | 1.521361589 |
| ABT1 | Activator Of Basal Transcription 1 | Protein Coding | 31 | GC06P079903 | 1.521001339 |
| JAKMIP2 | Janus Kinase And Microtubule Interacting Protein 2 | Protein Coding | 31 | GC05M147585 | 1.520953894 |
| BPIFB2 | BPI Fold Containing Family B Member 2 | Protein Coding | 30 | GC20P033007 | 1.520953894 |
| CLSTN3 | Calsyntenin 3 | Protein Coding | 32 | GC12P019863 | 1.519606948 |
| GYS1 | Glycogen Synthase 1 | Protein Coding | 44 | GC19M064089 | 1.519451857 |
| KRTAP10-1 | Keratin Associated Protein 10-1 | Protein Coding | 25 | GC21M044538 | 1.519064188 |
| GOLGA3 | Golgin A3 | Protein Coding | 33 | GC12M132768 | 1.518587232 |
| CEP83 | Centrosomal Protein 83 | Protein Coding | 32 | GC12M094265 | 1.518389344 |
| ACSBG2 | Acyl-CoA Synthetase Bubblegum Family Member 2 | Protein Coding | 32 | GC19P006135 | 1.518351674 |
| GNG8 | G Protein Subunit Gamma 8 | Protein Coding | 31 | GC19M064003 | 1.517955184 |
| TAF2 | TATA-Box Binding Protein Associated Factor 2 | Protein Coding | 36 | GC08M119730 | 1.517612576 |
| NCS1 | Neuronal Calcium Sensor 1 | Protein Coding | 38 | GC09P130172 | 1.517225146 |
| BRINP3 | BMP/Retinoic Acid Inducible Neural Specific 3 | Protein Coding | 28 | GC01M190067 | 1.51722014 |
| TMC1 | Transmembrane Channel Like 1 | Protein Coding | 37 | GC09P072521 | 1.517105222 |
| PANX1 | Pannexin 1 | Protein Coding | 40 | GC11P094128 | 1.516655326 |
| COL15A1 | Collagen Type XV Alpha 1 Chain | Protein Coding | 35 | GC09P098943 | 1.516544342 |
| MIR520G | MicroRNA 520g | RNA Gene | 17 | GC19P053722 | 1.516135454 |
| ZNF28 | Zinc Finger Protein 28 | Protein Coding | 28 | GC19M052797 | 1.515892982 |
| CMC2 | C-X9-C Motif Containing 2 | Protein Coding | 27 | GC16M080966 | 1.515721083 |
| XYLT2 | Xylosyltransferase 2 | Protein Coding | 40 | GC17P053545 | 1.515694737 |
| GAS2L2 | Growth Arrest Specific 2 Like 2 | Protein Coding | 31 | GC17M035744 | 1.515499353 |
| DUSP21 | Dual Specificity Phosphatase 21 | Protein Coding | 27 | GC0XP044844 | 1.514933586 |
| PCNX2 | Pecanex 2 | Protein Coding | 26 | GC01M232985 | 1.514339685 |
| ASGR2 | Asialoglycoprotein Receptor 2 | Protein Coding | 36 | GC17M007101 | 1.514193416 |
| MIR573 | MicroRNA 573 | RNA Gene | 15 | GC04M024521 | 1.514180183 |
| MTCH2 | Mitochondrial Carrier 2 | Protein Coding | 37 | GC11M047604 | 1.513781786 |
| RETREG3 | Reticulophagy Regulator Family Member 3 | Protein Coding | 27 | GC17M042581 | 1.513390064 |
| KIAA1522 | KIAA1522 | Protein Coding | 26 | GC01P032741 | 1.513260365 |
| CYP2C18 | Cytochrome P450 Family 2 Subfamily C Member 18 | Protein Coding | 39 | GC10P094684 | 1.513107061 |
| ZC3H7A | Zinc Finger CCCH-Type Containing 7A | Protein Coding | 32 | GC16M011750 | 1.512915373 |
| GALNT2 | Polypeptide N-Acetylgalactosaminyltransferase 2 | Protein Coding | 42 | GC01P230057 | 1.512798309 |

| | | | | | |
|---|---|---|---|---|---|
| DYNLT2 | Dynein Light Chain Tctex-Type 2 | Protein Coding | 26 | GC06M169726 | 1.512448311 |
| KHDC4 | KH Domain Containing 4, Pre-MRNA Splicing Factor | Protein Coding | 25 | GC01M155913 | 1.512046099 |
| ARVCF | ARVCF Delta Catenin Family Member | Protein Coding | 35 | GC22M019966 | 1.51169014 |
| PIK3AP1 | Phosphoinositide-3-Kinase Adaptor Protein 1 | Protein Coding | 35 | GC10M096593 | 1.511492491 |
| TAOK2 | TAO Kinase 2 | Protein Coding | 36 | GC16P040199 | 1.51145339 |
| PLXNB2 | Plexin B2 | Protein Coding | 34 | GC22M050274 | 1.510511994 |
| GNAZ | G Protein Subunit Alpha Z | Protein Coding | 38 | GC22P023070 | 1.510399461 |
| SLC28A3 | Solute Carrier Family 28 Member 3 | Protein Coding | 35 | GC09M092479 | 1.509606361 |
| CNN2 | Calponin 2 | Protein Coding | 34 | GC19P001026 | 1.509533286 |
| STK26 | Serine/Threonine Kinase 26 | Protein Coding | 35 | GC0XP132023 | 1.509497404 |
| CDK19 | Cyclin Dependent Kinase 19 | Protein Coding | 40 | GC06M110609 | 1.508726001 |
| IFITM9P | Interferon Induced Transmembrane Protein 9 Pseudogene | Pseudogene | 8 | GC11M069303 | 1.508679986 |
| DCAF4 | DDB1 And CUL4 Associated Factor 4 | Protein Coding | 30 | GC14P072926 | 1.508611083 |
| GCNT2 | Glucosaminyl (N-Acetyl) Transferase 2 (I Blood Group) | Protein Coding | 39 | GC06P010492 | 1.508047223 |
| OPRK1 | Opioid Receptor Kappa 1 | Protein Coding | 40 | GC08M053227 | 1.507685661 |
| TNIP3 | TNFAIP3 Interacting Protein 3 | Protein Coding | 32 | GC04M121131 | 1.50756979 |
| TMF1 | TATA Element Modulatory Factor 1 | Protein Coding | 32 | GC03M069019 | 1.507144809 |
| NCLN | Nicalin | Protein Coding | 32 | GC19P003185 | 1.506730199 |
| FAM149B1 | Family With Sequence Similarity 149 Member B1 | Protein Coding | 30 | GC10P073168 | 1.506109953 |
| MRPL4 | Mitochondrial Ribosomal Protein L4 | Protein Coding | 31 | GC19P010251 | 1.505965233 |
| AKIRIN1 | Akirin 1 | Protein Coding | 30 | GC01P038991 | 1.505946875 |
| FGD4 | FYVE, RhoGEF And PH Domain Containing 4 | Protein Coding | 40 | GC12P032407 | 1.505905032 |
| ZDHHC11 | Zinc Finger DHHC-Type Containing 11 | Protein Coding | 31 | GC05M000795 | 1.505732298 |
| SUPT7L | SPT7 Like, STAGA Complex Subunit Gamma | Protein Coding | 28 | GC02M028097 | 1.505326867 |
| ZHX1 | Zinc Fingers And Homeoboxes 1 | Protein Coding | 32 | GC08M123248 | 1.504867554 |
| EMILIN1 | Elastin Microfibril Interfacer 1 | Protein Coding | 36 | GC02P027078 | 1.50447607 |
| EMB | Embigin | Protein Coding | 36 | GC05M050396 | 1.504441619 |
| TRIR | Telomerase RNA Component Interacting RNase | Protein Coding | 25 | GC19M013523 | 1.504346848 |
| ADGRF5 | Adhesion G Protein-Coupled Receptor F5 | Protein Coding | 33 | GC06M046852 | 1.504084349 |
| SEMA3G | Semaphorin 3G | Protein Coding | 35 | GC03M052433 | 1.50393033 |
| TMEM209 | Transmembrane Protein 209 | Protein Coding | 29 | GC07M130164 | 1.503922701 |
| SERPINA2 | Serpin Family A Member 2 (Gene/Pseudogene) | Protein Coding | 24 | GC14M100445 | 1.503836274 |
| BCKDHB | Branched Chain Keto Acid Dehydrogenase E1 Subunit Beta | Protein Coding | 38 | GC06P080106 | 1.503764153 |
| AMDHD2 | Amidohydrolase Domain Containing 2 | Protein Coding | 32 | GC16P010740 | 1.503529787 |
| ZBTB8B | Zinc Finger And BTB Domain Containing 8B | Protein Coding | 27 | GC01P032488 | 1.503529787 |
| ABCB9 | ATP Binding Cassette Subfamily B Member 9 | Protein Coding | 35 | GC12M122920 | 1.502813578 |
| ASAP2 | ArfGAP With SH3 Domain, Ankyrin Repeat And PH Domain 2 | Protein Coding | 34 | GC02P009206 | 1.502697468 |
| KANK2 | KN Motif And Ankyrin Repeat Domains 2 | Protein Coding | 35 | GC19M011165 | 1.502173424 |
| ANGPTL6 | Angiopoietin Like 6 | Protein Coding | 36 | GC19M010092 | 1.502019048 |
| SEC23IP | SEC23 Interacting Protein | Protein Coding | 36 | GC10P119892 | 1.501488328 |
| MIR4728 | MicroRNA 4728 | RNA Gene | 10 | GC17P039726 | 1.500741124 |
| YLPM1 | YLP Motif Containing 1 | Protein Coding | 29 | GC14P074763 | 1.500734925 |
| DCTPP1 | DCTP Pyrophosphatase 1 | Protein Coding | 32 | GC16M036522 | 1.500682473 |
| SLAIN2 | SLAIN Motif Family Member 2 | Protein Coding | 32 | GC04P048382 | 1.500667214 |
| UPF3A | UPF3A Regulator Of Nonsense Mediated MRNA Decay | Protein Coding | 32 | GC13P114281 | 1.500505805 |
| TP53I11 | Tumor Protein P53 Inducible Protein 11 | Protein Coding | 32 | GC11M044881 | 1.50041008 |
| EIF3J | Eukaryotic Translation Initiation Factor 3 Subunit J | Protein Coding | 34 | GC15P044537 | 1.499796391 |
| RAB8A | RAB8A, Member RAS Oncogene Family | Protein Coding | 35 | GC19P016111 | 1.499452353 |
| MYO1D | Myosin ID | Protein Coding | 35 | GC17M032492 | 1.499318838 |
| CER1 | Cerberus 1, DAN Family BMP Antagonist | Protein Coding | 35 | GC09M014710 | 1.498890162 |
| GORASP1 | Golgi Reassembly Stacking Protein 1 | Protein Coding | 37 | GC03M039096 | 1.498475671 |
| TEKT5 | Tektin 5 | Protein Coding | 31 | GC16M010627 | 1.497997522 |
| WBP2NL | WBP2 N-Terminal Like | Protein Coding | 29 | GC22P043144 | 1.497974873 |
| ACAP2 | ArfGAP With Coiled-Coil, Ankyrin Repeat And PH Domains 2 | Protein Coding | 33 | GC03M195274 | 1.497569203 |
| SVBP | Small Vasohibin Binding Protein | Protein Coding | 27 | GC01M042808 | 1.497351408 |
| MBIP | MAP3K12 Binding Inhibitory Protein 1 | Protein Coding | 32 | GC14M036298 | 1.497095108 |
| GSTA4 | Glutathione S-Transferase Alpha 4 | Protein Coding | 38 | GC06M052977 | 1.497000694 |
| ILKAP | ILK Associated Serine/Threonine Phosphatase | Protein Coding | 35 | GC02M238170 | 1.496967554 |
| SRXN1 | Sulfiredoxin 1 | Protein Coding | 30 | GC20M000647 | 1.496554136 |
| DNPEP | Aspartyl Aminopeptidase | Protein Coding | 36 | GC02M219373 | 1.496402502 |
| PTPRB | Protein Tyrosine Phosphatase Receptor Type B | Protein Coding | 41 | GC12M070516 | 1.496044278 |
| SLC25A40 | Solute Carrier Family 25 Member 40 | Protein Coding | 30 | GC07M087833 | 1.495642662 |
| RAVER1 | Ribonucleoprotein, PTB Binding 1 | Protein Coding | 30 | GC19M010316 | 1.495623112 |
| SAP30BP | SAP30 Binding Protein | Protein Coding | 34 | GC17P075667 | 1.495610237 |
| B3GNT3 | UDP-GlcNAc:BetaGal Beta-1,3-N-Acetylglucosaminyltransferase 3 | Protein Coding | 35 | GC19P017794 | 1.495300293 |
| NUFIP2 | Nuclear FMR1 Interacting Protein 2 | Protein Coding | 30 | GC17M034592 | 1.495279789 |
| SCN4B | Sodium Voltage-Gated Channel Beta Subunit 4 | Protein Coding | 39 | GC11M118134 | 1.49461627 |
| HLA-V | Major Histocompatibility Complex, Class I, V (Pseudogene) | Pseudogene | 10 | GC06P080298 | 1.494508266 |
| LRRC4 | Leucine Rich Repeat Containing 4 | Protein Coding | 36 | GC07M128027 | 1.493489504 |
| APMAP | Adipocyte Plasma Membrane Associated Protein | Protein Coding | 32 | GC20M024962 | 1.493361354 |
| SPR | Sepiapterin Reductase | Protein Coding | 46 | GC02P072850 | 1.4931252 |
| SALL2 | Spalt Like Transcription Factor 2 | Protein Coding | 38 | GC14M021521 | 1.492875457 |
| PLAA | Phospholipase A2 Activating Protein | Protein Coding | 40 | GC09M026903 | 1.492701292 |
| BMP2K | BMP2 Inducible Kinase | Protein Coding | 35 | GC04P078776 | 1.492670774 |
| UTY | Ubiquitously Transcribed Tetratricopeptide Repeat Containing, Y-Linked | Protein Coding | 29 | GC0YM013231 | 1.49260509 |
| LOC108281177 | SOX2 5' Regulatory Region | Biological Region | 1 | GC03P181707 | 1.49252975 |
| DNAJB9 | DnaJ Heat Shock Protein Family (Hsp40) Member B9 | Protein Coding | 34 | GC07P108569 | 1.49180162 |
| RASD1 | Ras Related Dexamethasone Induced 1 | Protein Coding | 35 | GC17M017494 | 1.491797805 |
| C1GALT1C1 | C1GALT1 Specific Chaperone 1 | Protein Coding | 32 | GC0XM120625 | 1.491780996 |
| SPACA7 | Sperm Acrosome Associated 7 | Protein Coding | 27 | GC13P112375 | 1.491755247 |
| ALPK1 | Alpha Kinase 1 | Protein Coding | 36 | GC04P112285 | 1.491722345 |
| RAI2 | Retinoic Acid Induced 2 | Protein Coding | 31 | GC0XM017818 | 1.490575552 |
| MIR575 | MicroRNA 575 | RNA Gene | 15 | GC04M082753 | 1.490505576 |
| HVCN1 | Hydrogen Voltage Gated Channel 1 | Protein Coding | 34 | GC12M110627 | 1.490378261 |
| NMT2 | N-Myristoyltransferase 2 | Protein Coding | 36 | GC10M015160 | 1.489553094 |
| NPR2 | Natriuretic Peptide Receptor 2 | Protein Coding | 44 | GC09P035782 | 1.4891994 |
| EFCAB10 | EF-Hand Calcium Binding Domain 10 | Protein Coding | 21 | GC07M105565 | 1.489118814 |
| TNFAIP8L1 | TNF Alpha Induced Protein 8 Like 1 | Protein Coding | 28 | GC19P004639 | 1.489095449 |
| BTBD7 | BTB Domain Containing 7 | Protein Coding | 30 | GC14M093237 | 1.488855839 |
| NINL | Ninein Like | Protein Coding | 33 | GC20M025452 | 1.48829937 |
| TRIM6 | Tripartite Motif Containing 6 | Protein Coding | 32 | GC11P005596 | 1.488243937 |
| STX17 | Syntaxin 17 | Protein Coding | 30 | GC09P099906 | 1.488077998 |
| A2MP1 | Alpha-2-Macroglobulin Pseudogene 1 | Pseudogene | 13 | GC12M009228 | 1.48791647 |
| SDC4P | Syndecan 4 Pseudogene | Pseudogene | 11 | GC22M030481 | 1.48791647 |
| LOC728715 | Ovostatin Homolog 2 | RNA Gene | 10 | GC12M020431 | 1.48791647 |

| | | | | | |
|---|---|---|---|---|---|
| LOC642846 | DEAD/H (Asp-Glu-Ala-Asp/His) Box Polypeptide 11-Like | RNA Gene | 9 | GC12P019936 | 1.48791647 |
| MESTP4 | Mesoderm Specific Transcript Pseudogene 4 | Pseudogene | 7 | GC03P029088 | 1.48791647 |
| RPL23AP37 | Ribosomal Protein L23a Pseudogene 37 | Pseudogene | 7 | GC02M064347 | 1.48791647 |
| SPA17P1 | Sperm Autoantigenic Protein 17 Pseudogene 1 | Pseudogene | 7 | GC10M075382 | 1.48791647 |
| MRPS7P1 | Mitochondrial Ribosomal Protein S7 Pseudogene 1 | Pseudogene | 6 | GC08M036796 | 1.48791647 |
| RPL19P8 | Ribosomal Protein L19 Pseudogene 8 | Pseudogene | 6 | GC04P178286 | 1.48791647 |
| LOC100129646 | Intraflagellar Transport 57 Homolog (Chlamydomonas) Pseudogene | Pseudogene | 2 | GC12P028166 | 1.48791647 |
| FRMPD2 | FERM And PDZ Domain Containing 2 | Protein Coding | 30 | GC10M048153 | 1.487897635 |
| RAPGEF6 | Rap Guanine Nucleotide Exchange Factor 6 | Protein Coding | 34 | GC05M131423 | 1.487713575 |
| DNAH7 | Dynein Axonemal Heavy Chain 7 | Protein Coding | 31 | GC02M195737 | 1.487709165 |
| ATMIN | ATM Interactor | Protein Coding | 33 | GC16P081035 | 1.487534285 |
| CD160 | CD160 Molecule | Protein Coding | 35 | GC01P145719 | 1.487013578 |
| ADCYAP1R1 | ADCYAP Receptor Type I | Protein Coding | 40 | GC07P031058 | 1.486969471 |
| C1orf35 | Chromosome 1 Open Reading Frame 35 | Protein Coding | 27 | GC01M228100 | 1.486916542 |
| GTF3C4 | General Transcription Factor IIIC Subunit 4 | Protein Coding | 34 | GC09P132671 | 1.486385465 |
| NOMO1 | NODAL Modulator 1 | Protein Coding | 31 | GC16P017264 | 1.485970974 |
| MRPL15 | Mitochondrial Ribosomal Protein L15 | Protein Coding | 34 | GC08P054135 | 1.485761762 |
| NTAQ1 | N-Terminal Glutamine Amidase 1 | Protein Coding | 25 | GC08P123450 | 1.485675693 |
| MRPL52 | Mitochondrial Ribosomal Protein L52 | Protein Coding | 28 | GC14P022829 | 1.485309839 |
| CCDC83 | Coiled-Coil Domain Containing 83 | Protein Coding | 27 | GC11P085855 | 1.485266924 |
| TMED9 | Transmembrane P24 Trafficking Protein 9 | Protein Coding | 31 | GC05P177594 | 1.484673977 |
| MTMR14 | Myotubularin Related Protein 14 | Protein Coding | 39 | GC03P009649 | 1.484174132 |
| TLE2 | TLE Family Member 2, Transcriptional Corepressor | Protein Coding | 37 | GC19M002997 | 1.484101176 |
| MEX3A | Mex-3 RNA Binding Family Member A | Protein Coding | 30 | GC01M156072 | 1.483832717 |
| SLC18A3 | Solute Carrier Family 18 Member A3 | Protein Coding | 39 | GC10P049610 | 1.483623266 |
| MIR219A2 | MicroRNA 219a-2 | RNA Gene | 15 | GC09M128466 | 1.483544469 |
| SF3B6 | Splicing Factor 3b Subunit 6 | Protein Coding | 28 | GC02M024067 | 1.482679725 |
| ZP3 | Zona Pellucida Glycoprotein 3 | Protein Coding | 39 | GC07P076409 | 1.482666373 |
| USP30 | Ubiquitin Specific Peptidase 30 | Protein Coding | 35 | GC12P109027 | 1.482551336 |
| FKBP10 | FKBP Prolyl Isomerase 10 | Protein Coding | 38 | GC17P041812 | 1.482434511 |
| RNPEPL1 | Arginyl Aminopeptidase Like 1 | Protein Coding | 32 | GC02P240565 | 1.482093573 |
| AMTN | Amelotin | Protein Coding | 30 | GC04P070518 | 1.482093573 |
| GDAP1L1 | Ganglioside Induced Differentiation Associated Protein 1 Like 1 | Protein Coding | 30 | GC20P044247 | 1.482093573 |
| NKX2-6 | NK2 Homeobox 6 | Protein Coding | 32 | GC08M023702 | 1.481655121 |
| AP5B1 | Adaptor Related Protein Complex 5 Subunit Beta 1 | Protein Coding | 28 | GC11M065773 | 1.481581926 |
| ADAT2 | Adenosine Deaminase TRNA Specific 2 | Protein Coding | 29 | GC06M143422 | 1.481500864 |
| AKAP8L | A-Kinase Anchoring Protein 8 Like | Protein Coding | 31 | GC19M015380 | 1.481456995 |
| ADAMTS10 | ADAM Metallopeptidase With Thrombospondin Type 1 Motif 10 | Protein Coding | 39 | GC19M008580 | 1.481446266 |
| YIF1B | Yip1 Interacting Factor Homolog B, Membrane Trafficking Protein | Protein Coding | 32 | GC19M038303 | 1.481408238 |
| STON2 | Stonin 2 | Protein Coding | 30 | GC14M081260 | 1.481369615 |
| USP20 | Ubiquitin Specific Peptidase 20 | Protein Coding | 36 | GC09P129834 | 1.481252432 |
| OPA6 | Optic Atrophy 6 (Autosomal Recessive) | Genetic Locus | 3 | GC08U900809 | 1.480822563 |
| ZNF574 | Zinc Finger Protein 574 | Protein Coding | 30 | GC19P042068 | 1.480286241 |
| UTP23 | UTP23 Small Subunit Processome Component | Protein Coding | 27 | GC08P116796 | 1.480286241 |
| CCDC93 | Coiled-Coil Domain Containing 93 | Protein Coding | 30 | GC02M117915 | 1.480128288 |
| PHF12 | PHD Finger Protein 12 | Protein Coding | 33 | GC17M028905 | 1.480078936 |
| SLC16A2 | Solute Carrier Family 16 Member 2 | Protein Coding | 40 | GC0XP074475 | 1.479830503 |
| MSL2 | MSL Complex Subunit 2 | Protein Coding | 31 | GC03M136149 | 1.47954452 |
| CPLX3 | Complexin 3 | Protein Coding | 31 | GC15P074826 | 1.47947228 |
| ARF5 | ADP Ribosylation Factor 5 | Protein Coding | 35 | GC07P127588 | 1.479361773 |
| MOSPD2 | Motile Sperm Domain Containing 2 | Protein Coding | 31 | GC0XP014891 | 1.479349613 |
| TEKT3 | Tektin 3 | Protein Coding | 32 | GC17M015303 | 1.478781343 |
| NUBP2 | NUBP Iron-Sulfur Cluster Assembly Factor 2, Cytosolic | Protein Coding | 35 | GC16P001782 | 1.478653669 |
| OTUD6B | OTU Deubiquitinase 6B | Protein Coding | 35 | GC08P091070 | 1.478622675 |
| CMTM7 | CKLF Like MARVEL Transmembrane Domain Containing 7 | Protein Coding | 34 | GC03P032409 | 1.478485107 |
| TIMM9 | Translocase Of Inner Mitochondrial Membrane 9 | Protein Coding | 34 | GC14M058408 | 1.477772236 |
| ASPA | Aspartoacylase | Protein Coding | 40 | GC17P003472 | 1.477321625 |
| ATP1A4 | ATPase Na+/K+ Transporting Subunit Alpha 4 | Protein Coding | 36 | GC01P160151 | 1.476952076 |
| TSEN34 | TRNA Splicing Endonuclease Subunit 34 | Protein Coding | 35 | GC19P066189 | 1.475796461 |
| CEP295 | Centrosomal Protein 295 | Protein Coding | 25 | GC11P093661 | 1.47578311 |
| YPEL1 | Yippee Like 1 | Protein Coding | 32 | GC22M021697 | 1.475695848 |
| KCNA3 | Potassium Voltage-Gated Channel Subfamily A Member 3 | Protein Coding | 40 | GC01M110654 | 1.475669861 |
| HARS2 | Histidyl-TRNA Synthetase 2, Mitochondrial | Protein Coding | 39 | GC05P145901 | 1.475280762 |
| MIR603 | MicroRNA 603 | RNA Gene | 13 | GC10P024275 | 1.475252867 |
| C1orf21 | Chromosome 1 Open Reading Frame 21 | Protein Coding | 28 | GC01P184356 | 1.47514677 |
| CFAP251 | Cilia And Flagella Associated Protein 251 | Protein Coding | 27 | GC12P125971 | 1.475024223 |
| NRM | Nurim | Protein Coding | 30 | GC06M063526 | 1.474586964 |
| IQGAP2 | IQ Motif Containing GTPase Activating Protein 2 | Protein Coding | 37 | GC05P076403 | 1.474364996 |
| SPON1 | Spondin 1 | Protein Coding | 35 | GC11P013940 | 1.474333286 |
| MINDY1 | MINDY Lysine 48 Deubiquitinase 1 | Protein Coding | 27 | GC01M151683 | 1.473634005 |
| NDUFAF3 | NADH:Ubiquinone Oxidoreductase Complex Assembly Factor 3 | Protein Coding | 36 | GC03P049020 | 1.473206282 |
| TRPV5 | Transient Receptor Potential Cation Channel Subfamily V Member 5 | Protein Coding | 41 | GC07M142908 | 1.473068595 |
| CEP19 | Centrosomal Protein 19 | Protein Coding | 31 | GC03M196706 | 1.472828865 |
| PWAR1 | Prader Willi/Angelman Region RNA 1 | RNA Gene | 15 | GC15P025135 | 1.472677708 |
| UBE2O | Ubiquitin Conjugating Enzyme E2 O | Protein Coding | 35 | GC17M076389 | 1.472332001 |
| LOC108961161 | POU5F1 5' Regulatory Region | Biological Region | 2 | GC06P081803 | 1.472332001 |
| OTOP2 | Otopetrin 2 | Protein Coding | 27 | GC17P074924 | 1.471781254 |
| ERICH1 | Glutamate Rich 1 | Protein Coding | 26 | GC08M000614 | 1.471781254 |
| SOGA1 | Suppressor Of Glucose, Autophagy Associated 1 | Protein Coding | 28 | GC20M036777 | 1.471485615 |
| MRPS18A | Mitochondrial Ribosomal Protein S18A | Protein Coding | 32 | GC06M043671 | 1.47127831 |
| C17orf49 | Chromosome 17 Open Reading Frame 49 | Protein Coding | 28 | GC17P007014 | 1.471186638 |
| NOSTRIN | Nitric Oxide Synthase Trafficking | Protein Coding | 33 | GC02P168786 | 1.471004963 |
| TOM1L1 | Target Of Myb1 Like 1 Membrane Trafficking Protein | Protein Coding | 32 | GC17P054901 | 1.47001946 |
| IFT27 | Intraflagellar Transport 27 | Protein Coding | 32 | GC22M036766 | 1.469806671 |
| CA11 | Carbonic Anhydrase 11 | Protein Coding | 34 | GC19M064071 | 1.469619274 |
| AMER2 | APC Membrane Recruitment Protein 2 | Protein Coding | 27 | GC13M025161 | 1.468092918 |
| TDRD7 | Tudor Domain Containing 7 | Protein Coding | 34 | GC09P097411 | 1.467914343 |
| THAP12 | THAP Domain Containing 12 | Protein Coding | 29 | GC11M076349 | 1.467656851 |
| PCOLCE | Procollagen C-Endopeptidase Enhancer | Protein Coding | 35 | GC07P100602 | 1.467514634 |
| MSL3 | MSL Complex Subunit 3 | Protein Coding | 34 | GC0XP011758 | 1.46743989 |
| CCDC124 | Coiled-Coil Domain Containing 124 | Protein Coding | 27 | GC19P063115 | 1.467334032 |
| TBX10 | T-Box Transcription Factor 10 | Protein Coding | 32 | GC11M067631 | 1.467121363 |
| MUC19 | Mucin 19, Oligomeric | Protein Coding | 27 | GC12P040393 | 1.467074156 |
| MRPS31 | Mitochondrial Ribosomal Protein S31 | Protein Coding | 32 | GC13M040729 | 1.466505527 |
| POTEG | POTE Ankyrin Domain Family Member G | Protein Coding | 25 | GC14M019402 | 1.466476917 |

| | | | | | |
|---|---|---|---|---|---|
| IKBIP | IKBKB Interacting Protein | Protein Coding | 30 | GC12M098613 | 1.466420889 |
| RER1 | Retention In Endoplasmic Reticulum Sorting Receptor 1 | Protein Coding | 31 | GC01P002391 | 1.466149926 |
| EIF2B1 | Eukaryotic Translation Initiation Factor 2B Subunit Alpha | Protein Coding | 39 | GC12M123620 | 1.46592319 |
| POLR1E | RNA Polymerase I Subunit E | Protein Coding | 31 | GC09P046278 | 1.465883613 |
| SNX1 | Sorting Nexin 1 | Protein Coding | 36 | GC15P064094 | 1.465687513 |
| OGFOD1 | 2-Oxoglutarate And Iron Dependent Oxygenase Domain Containing 1 | Protein Coding | 33 | GC16P056454 | 1.465671539 |
| OXER1 | Oxoeicosanoid Receptor 1 | Protein Coding | 34 | GC02M042762 | 1.465045929 |
| LEPROT | Leptin Receptor Overlapping Transcript | Protein Coding | 28 | GC01P065420 | 1.464316487 |
| TNR | Tenascin R | Protein Coding | 39 | GC01M175291 | 1.463855028 |
| MIR509-3 | MicroRNA 509-3 | RNA Gene | 14 | GC0XM147259 | 1.463813066 |
| NME7 | NME/NM23 Family Member 7 | Protein Coding | 37 | GC01M169134 | 1.463439465 |
| SCYL3 | SCY1 Like Pseudokinase 3 | Protein Coding | 34 | GC01M169821 | 1.463439465 |
| LINC01426 | Long Intergenic Non-Protein Coding RNA 1426 | RNA Gene | 13 | GC21P034745 | 1.462992072 |
| SMCR8 | SMCR8-C9orf72 Complex Subunit | Protein Coding | 27 | GC17P052243 | 1.46267128 |
| AP1S1 | Adaptor Related Protein Complex 1 Subunit Sigma 1 | Protein Coding | 35 | GC07P101154 | 1.462608814 |
| TRAF3IP3 | TRAF3 Interacting Protein 3 | Protein Coding | 32 | GC01P209756 | 1.462607384 |
| CHKB | Choline Kinase Beta | Protein Coding | 40 | GC22M050578 | 1.462484121 |
| CCDC137 | Coiled-Coil Domain Containing 137 | Protein Coding | 28 | GC17P081666 | 1.462434769 |
| RBM4B | RNA Binding Motif Protein 4B | Protein Coding | 31 | GC11M066664 | 1.462362766 |
| RNF111 | Ring Finger Protein 111 | Protein Coding | 33 | GC15P058866 | 1.462319374 |
| FTH1P3 | Ferritin Heavy Chain 1 Pseudogene 3 | Pseudogene | 13 | GC02M027392 | 1.461795568 |
| PRDM6 | PR/SET Domain 6 | Protein Coding | 35 | GC05P123089 | 1.461780429 |
| GOLT1A | Golgi Transport 1A | Protein Coding | 29 | GC01M204198 | 1.459988594 |
| CA10 | Carbonic Anhydrase 10 | Protein Coding | 36 | GC17M051630 | 1.459964275 |
| MT1H | Metallothionein 1H | Protein Coding | 30 | GC16P057003 | 1.458582401 |
| MRPS35 | Mitochondrial Ribosomal Protein S35 | Protein Coding | 34 | GC12P027710 | 1.458178282 |
| HLA-U | Major Histocompatibility Complex, Class I, U (Pseudogene) | Pseudogene | 9 | GC06P080310 | 1.458144188 |
| ENSG00000272221 | Novel Transcript | RNA Gene | 7 | GC06M063577 | 1.458144188 |
| WDR35 | WD Repeat Domain 35 | Protein Coding | 35 | GC02M019910 | 1.458061218 |
| ZNF451 | Zinc Finger Protein 451 | Protein Coding | 32 | GC06P057086 | 1.458040953 |
| CSNK1G3 | Casein Kinase 1 Gamma 3 | Protein Coding | 39 | GC05P123512 | 1.457998991 |
| DGAT2 | Diacylglycerol O-Acyltransferase 2 | Protein Coding | 39 | GC11P075759 | 1.457827449 |
| REXO4 | REX4 Homolog, 3'-5' Exonuclease | Protein Coding | 32 | GC09M133406 | 1.457636595 |
| LOC101929710 | Uncharacterized LOC101929710 | RNA Gene | 11 | GC05P095962 | 1.457598209 |
| IPP | Intracisternal A Particle-Promoted Polypeptide | Protein Coding | 31 | GC01M045695 | 1.457342386 |
| FAM126A | Family With Sequence Similarity 126 Member A | Protein Coding | 33 | GC07M022889 | 1.456834435 |
| AVPR1B | Arginine Vasopressin Receptor 1B | Protein Coding | 41 | GC01M206109 | 1.456833363 |
| LOC110351180 | IBSP 5' Regulatory Region | Biological Region | 2 | GC04P087794 | 1.456713438 |
| LOC108449888 | PATRR22 Recombination Region | Biological Region | 2 | GC22U901439 | 1.456267953 |
| SLC16A5 | Solute Carrier Family 16 Member 5 | Protein Coding | 34 | GC17P075541 | 1.456108332 |
| ABHD5 | Abhydrolase Domain Containing 5, Lysophosphatidic Acid Acyltransferase | Protein Coding | 40 | GC03P043707 | 1.456088662 |
| BTBD9 | BTB Domain Containing 9 | Protein Coding | 32 | GC06M038168 | 1.456056356 |
| CLK3 | CDC Like Kinase 3 | Protein Coding | 38 | GC15P074598 | 1.455784559 |
| TAF7 | TATA-Box Binding Protein Associated Factor 7 | Protein Coding | 33 | GC05M141260 | 1.455714703 |
| FOXD2 | Forkhead Box D2 | Protein Coding | 31 | GC01P047436 | 1.455400229 |
| PAQR8 | Progestin And AdipoQ Receptor Family Member 8 | Protein Coding | 32 | GC06P080686 | 1.455261469 |
| PIGY | Phosphatidylinositol Glycan Anchor Biosynthesis Class Y | Protein Coding | 32 | GC04M088520 | 1.454991817 |
| MIR3196 | MicroRNA 3196 | RNA Gene | 11 | GC20P063322 | 1.454846978 |
| SMG9 | SMG9 Nonsense Mediated MRNA Decay Factor | Protein Coding | 30 | GC19M043727 | 1.454734325 |
| KCND2 | Potassium Voltage-Gated Channel Subfamily D Member 2 | Protein Coding | 39 | GC07P120273 | 1.454483986 |
| C1QC | Complement C1q C Chain | Protein Coding | 40 | GC01P022643 | 1.454340935 |
| CYP20A1 | Cytochrome P450 Family 20 Subfamily A Member 1 | Protein Coding | 32 | GC02P203238 | 1.453969121 |
| SQOR | Sulfide Quinone Oxidoreductase | Protein Coding | 29 | GC15P045632 | 1.453652859 |
| TAF6L | TATA-Box Binding Protein Associated Factor 6 Like | Protein Coding | 32 | GC11P062771 | 1.453535914 |
| LINC00922 | Long Intergenic Non-Protein Coding RNA 922 | RNA Gene | 14 | GC16M065284 | 1.453429937 |
| PAFAH1B3 | Platelet Activating Factor Acetylhydrolase 1b Catalytic Subunit 3 | Protein Coding | 36 | GC19M042297 | 1.453019619 |
| MAN2C1 | Mannosidase Alpha Class 2C Member 1 | Protein Coding | 34 | GC15M075355 | 1.452746987 |
| PDZRN4 | PDZ Domain Containing Ring Finger 4 | Protein Coding | 32 | GC12P041188 | 1.452664137 |
| FUT7 | Fucosyltransferase 7 | Protein Coding | 37 | GC09M137030 | 1.45247221 |
| APOBEC3H | Apolipoprotein B MRNA Editing Enzyme Catalytic Subunit 3H | Protein Coding | 31 | GC22P039097 | 1.452447653 |
| ARSD | Arylsulfatase D | Protein Coding | 34 | GC0XM002903 | 1.452278256 |
| CMPK1 | Cytidine/Uridine Monophosphate Kinase 1 | Protein Coding | 38 | GC01P047333 | 1.451844692 |
| MAGEF1 | MAGE Family Member F1 | Protein Coding | 31 | GC03M184710 | 1.451472878 |
| IWS1 | Interacts With SUPT6H, CTD Assembly Factor 1 | Protein Coding | 31 | GC02M127436 | 1.451192617 |
| MRC1 | Mannose Receptor C-Type 1 | Protein Coding | 36 | GC10P017809 | 1.450993538 |
| ZDHHC7 | Zinc Finger DHHC-Type Palmitoyltransferase 7 | Protein Coding | 34 | GC16M084975 | 1.450817823 |
| HLA-F-AS1 | HLA-F Antisense RNA 1 | RNA Gene | 15 | GC06M063486 | 1.450345874 |
| FBXO46 | F-Box Protein 46 | Protein Coding | 26 | GC19M045710 | 1.450306892 |
| FCGRT | Fc Gamma Receptor And Transporter | Protein Coding | 39 | GC19P049506 | 1.449971199 |
| GBX2 | Gastrulation Brain Homeobox 2 | Protein Coding | 36 | GC02M236165 | 1.449840069 |
| IGHA1 | Immunoglobulin Heavy Constant Alpha 1 | Protein Coding | 26 | GC14M112404 | 1.449811697 |
| TRPV2 | Transient Receptor Potential Cation Channel Subfamily V Member 2 | Protein Coding | 38 | GC17P016415 | 1.449293733 |
| ACTL6B | Actin Like 6B | Protein Coding | 38 | GC07M100643 | 1.448867321 |
| OPRPN | Opiorphin Prepropeptide | Protein Coding | 24 | GC04P070398 | 1.448747396 |
| TMEM14B | Transmembrane Protein 14B | Protein Coding | 28 | GC06P010747 | 1.448540926 |
| VIPR2 | Vasoactive Intestinal Peptide Receptor 2 | Protein Coding | 39 | GC07M159028 | 1.448500872 |
| DOCK9 | Dedicator Of Cytokinesis 9 | Protein Coding | 34 | GC13M098793 | 1.448493004 |
| ICAM2 | Intercellular Adhesion Molecule 2 | Protein Coding | 39 | GC17M064002 | 1.447492242 |
| KIN | Kin17 DNA And RNA Binding Protein | Protein Coding | 32 | GC10M007750 | 1.44721508 |
| CSRP1 | Cysteine And Glycine Rich Protein 1 | Protein Coding | 38 | GC01M201484 | 1.446917176 |
| RPF2 | Ribosome Production Factor 2 Homolog | Protein Coding | 28 | GC06P110982 | 1.446913719 |
| ZC3H18 | Zinc Finger CCCH-Type Containing 18 | Protein Coding | 28 | GC16P088570 | 1.44638896 |
| KASH5 | KASH Domain Containing 5 | Protein Coding | 25 | GC19P064059 | 1.446376562 |
| ARR3 | Arrestin 3 | Protein Coding | 36 | GC0XP070269 | 1.445938587 |
| GZMH | Granzyme H | Protein Coding | 36 | GC14M024606 | 1.445049644 |
| ATP6V0A4 | ATPase H+ Transporting V0 Subunit A4 | Protein Coding | 39 | GC07M138708 | 1.444995642 |
| BICD1 | BICD Cargo Adaptor 1 | Protein Coding | 35 | GC12P032107 | 1.444477081 |
| GJC1 | Gap Junction Protein Gamma 1 | Protein Coding | 38 | GC17M044882 | 1.444436789 |
| LIPH | Lipase H | Protein Coding | 39 | GC03M185506 | 1.444389939 |
| INTS12 | Integrator Complex Subunit 12 | Protein Coding | 30 | GC04M105682 | 1.444165826 |
| TM9SF2 | Transmembrane 9 Superfamily Member 2 | Protein Coding | 34 | GC13P099446 | 1.444110513 |
| ESRP2 | Epithelial Splicing Regulatory Protein 2 | Protein Coding | 33 | GC16M068781 | 1.443933964 |
| SDAD1 | SDA1 Domain Containing 1 | Protein Coding | 30 | GC04M075940 | 1.443899274 |
| SLC15A1 | Solute Carrier Family 15 Member 1 | Protein Coding | 40 | GC13M098683 | 1.443679094 |
| DDX24 | DEAD-Box Helicase 24 | Protein Coding | 35 | GC14M094048 | 1.443677902 |

| | | | | | |
|---|---|---|---|---|---|
| ZNRD2 | Zinc Ribbon Domain Containing 2 | Protein Coding | 28 | GC11P069549 | 1.443664074 |
| MIR1205 | MicroRNA 1205 | RNA Gene | 13 | GC08P127960 | 1.443653107 |
| LINC01793 | Long Intergenic Non-Protein Coding RNA 1793 | RNA Gene | 11 | GC02P059218 | 1.443601966 |
| MARCHF6 | Membrane Associated Ring-CH-Type Finger 6 | Protein Coding | 29 | GC05P010424 | 1.442908883 |
| PLEKHG4 | Pleckstrin Homology And RhoGEF Domain Containing G4 | Protein Coding | 38 | GC16P067277 | 1.442599773 |
| SLC39A9 | Solute Carrier Family 39 Member 9 | Protein Coding | 31 | GC14P069398 | 1.441940308 |
| CRADD | CASP2 And RIPK1 Domain Containing Adaptor With Death Domain | Protein Coding | 39 | GC12P093677 | 1.4418571 |
| NDUFA4 | NDUFA4 Mitochondrial Complex Associated | Protein Coding | 39 | GC07M010938 | 1.441697836 |
| IL6-AS1 | IL6 Antisense RNA 1 | RNA Gene | 12 | GC07M022728 | 1.441609859 |
| IQCN | IQ Motif Containing N | Protein Coding | 22 | GC19M018257 | 1.441312075 |
| MIR514B | MicroRNA 514b | RNA Gene | 12 | GC0XM147250 | 1.441311359 |
| HEATR1 | HEAT Repeat Containing 1 | Protein Coding | 32 | GC01M236549 | 1.441191196 |
| MRPS28 | Mitochondrial Ribosomal Protein S28 | Protein Coding | 36 | GC08M079940 | 1.441165924 |
| DHRS4 | Dehydrogenase/Reductase 4 | Protein Coding | 38 | GC14P023953 | 1.441111922 |
| CEPT1 | Choline/Ethanolamine Phosphotransferase 1 | Protein Coding | 36 | GC01P111139 | 1.440681696 |
| FRMD6 | FERM Domain Containing 6 | Protein Coding | 34 | GC14P051489 | 1.440587044 |
| EXOSC7 | Exosome Component 7 | Protein Coding | 33 | GC03P046363 | 1.440565705 |
| SERPINA12 | Serpin Family A Member 12 | Protein Coding | 35 | GC14M100446 | 1.440376878 |
| UPK1B | Uroplakin 1B | Protein Coding | 32 | GC03P119173 | 1.440257192 |
| GALR2 | Galanin Receptor 2 | Protein Coding | 40 | GC17P076070 | 1.440253735 |
| DDI1 | DNA Damage Inducible 1 Homolog 1 | Protein Coding | 30 | GC11P104036 | 1.440225363 |
| ANGPTL3 | Angiopoietin Like 3 | Protein Coding | 41 | GC01P062597 | 1.439847231 |
| NCKAP5L | NCK Associated Protein 5 Like | Protein Coding | 27 | GC12M049791 | 1.439465046 |
| STRN3 | Striatin 3 | Protein Coding | 35 | GC14M030893 | 1.439143658 |
| RAB3IL1 | RAB3A Interacting Protein Like 1 | Protein Coding | 34 | GC11M061897 | 1.438489676 |
| ROPN1L | Rhophilin Associated Tail Protein 1 Like | Protein Coding | 32 | GC05P010441 | 1.438489676 |
| OLAH | Oleoyl-ACP Hydrolase | Protein Coding | 30 | GC10P015032 | 1.43805778 |
| PPAN | Peter Pan Homolog | Protein Coding | 30 | GC19P010373 | 1.4375875 |
| TMEM214 | Transmembrane Protein 214 | Protein Coding | 30 | GC02P027032 | 1.436534643 |
| MIR4757 | MicroRNA 4757 | RNA Gene | 11 | GC02P019348 | 1.436385989 |
| ESYT2 | Extended Synaptotagmin 2 | Protein Coding | 32 | GC07M158730 | 1.43635416 |
| TRIM40 | Tripartite Motif Containing 40 | Protein Coding | 32 | GC06P080318 | 1.435605288 |
| MTHFSD | Methenyltetrahydrofolate Synthetase Domain Containing | Protein Coding | 31 | GC16M086530 | 1.435592651 |
| MTCO3P1 | MT-CO3 Pseudogene 1 | Pseudogene | 7 | GC06M032706 | 1.434613228 |
| AP3S1 | Adaptor Related Protein Complex 3 Subunit Sigma 1 | Protein Coding | 35 | GC05P115841 | 1.43452549 |
| GOLGA4 | Golgin A4 | Protein Coding | 33 | GC03P037243 | 1.434216261 |
| SLC31A2 | Solute Carrier Family 31 Member 2 | Protein Coding | 35 | GC09P113150 | 1.433675051 |
| TMEM158 | Transmembrane Protein 158 | Protein Coding | 28 | GC03M045241 | 1.433454037 |
| TSC22D2 | TSC22 Domain Family Member 2 | Protein Coding | 29 | GC03P150408 | 1.432765961 |
| TTL | Tubulin Tyrosine Ligase | Protein Coding | 36 | GC02P121629 | 1.432282448 |
| MYH7B | Myosin Heavy Chain 7B | Protein Coding | 34 | GC20P034956 | 1.432282448 |
| MYL12B | Myosin Light Chain 12B | Protein Coding | 32 | GC18P003261 | 1.431398392 |
| LMLN | Leishmanolysin Like Peptidase | Protein Coding | 31 | GC03P197961 | 1.431209803 |
| EIF4E2 | Eukaryotic Translation Initiation Factor 4E Family Member 2 | Protein Coding | 35 | GC02P232550 | 1.431067944 |
| EPN2 | Epsin 2 | Protein Coding | 35 | GC17P019215 | 1.430890441 |
| PPP6R3 | Protein Phosphatase 6 Regulatory Subunit 3 | Protein Coding | 32 | GC11P068460 | 1.430546641 |
| MIR646 | MicroRNA 646 | RNA Gene | 13 | GC20P060308 | 1.429965973 |
| DNAJB5 | DnaJ Heat Shock Protein Family (Hsp40) Member B5 | Protein Coding | 34 | GC09P040127 | 1.42958498 |
| SUSD4 | Sushi Domain Containing 4 | Protein Coding | 32 | GC01M223220 | 1.42942524 |
| CACNG1 | Calcium Voltage-Gated Channel Auxiliary Subunit Gamma 1 | Protein Coding | 37 | GC17P067044 | 1.429422379 |
| DDX49 | DEAD-Box Helicase 49 | Protein Coding | 31 | GC19P018919 | 1.429279327 |
| SLC35D2 | Solute Carrier Family 35 Member D2 | Protein Coding | 31 | GC09M096313 | 1.429016113 |
| LOC101928847 | Uncharacterized LOC101928847 | RNA Gene | 9 | GC11M112959 | 1.428971052 |
| LOC112163653 | Sharpr-MPRA Regulatory Region 1962 | Biological Region | 2 | GC13P032381 | 1.428971052 |
| GNL3L | G Protein Nucleolar 3 Like | Protein Coding | 31 | GC0XP054533 | 1.428801298 |
| SLC27A2 | Solute Carrier Family 27 Member 2 | Protein Coding | 40 | GC15P050182 | 1.428426504 |
| PLSCR1 | Phospholipid Scramblase 1 | Protein Coding | 39 | GC03M146515 | 1.426750422 |
| NR2E1 | Nuclear Receptor Subfamily 2 Group E Member 1 | Protein Coding | 39 | GC06P108166 | 1.426532745 |
| NTNG1 | Netrin G1 | Protein Coding | 38 | GC01P107140 | 1.426411629 |
| ZBTB10 | Zinc Finger And BTB Domain Containing 10 | Protein Coding | 31 | GC08P080485 | 1.42579937 |
| MGAT3 | Beta-1,4-Mannosyl-Glycoprotein 4-Beta-N-Acetylglucosaminyltransferase | Protein Coding | 35 | GC22P039447 | 1.425714016 |
| RPL3L | Ribosomal Protein L3 Like | Protein Coding | 35 | GC16M001943 | 1.42536211 |
| RPAP3 | RNA Polymerase II Associated Protein 3 | Protein Coding | 30 | GC12M047661 | 1.425171137 |
| ULK4 | Unc-51 Like Kinase 4 | Protein Coding | 36 | GC03M041247 | 1.425146461 |
| TOR1AIP2 | Torsin 1A Interacting Protein 2 | Protein Coding | 30 | GC01M183356 | 1.425000191 |
| DENND4C | DENN Domain Containing 4C | Protein Coding | 29 | GC09P019230 | 1.424384952 |
| EMILIN2 | Elastin Microfibril Interfacer 2 | Protein Coding | 33 | GC18P002846 | 1.424222231 |
| MIR210HG | MIR210 Host Gene | RNA Gene | 15 | GC11M000563 | 1.424173117 |
| NCCRP1 | NCCRP1, F-Box Associated Domain Containing | Protein Coding | 28 | GC19P039196 | 1.424079895 |
| MEOX2 | Mesenchyme Homeobox 2 | Protein Coding | 37 | GC07M015617 | 1.424000502 |
| SAMD7 | Sterile Alpha Motif Domain Containing 7 | Protein Coding | 30 | GC03P169911 | 1.423833489 |
| MORC4 | MORC Family CW-Type Zinc Finger 4 | Protein Coding | 28 | GC0XM106813 | 1.423707247 |
| PNPLA8 | Patatin Like Phospholipase Domain Containing 8 | Protein Coding | 39 | GC07M108470 | 1.423260212 |
| MYLK4 | Myosin Light Chain Kinase Family Member 4 | Protein Coding | 32 | GC06M002663 | 1.423140526 |
| FRYL | FRY Like Transcription Coactivator | Protein Coding | 28 | GC04M048558 | 1.42307806 |
| TSPYL5 | TSPY Like 5 | Protein Coding | 30 | GC08M097273 | 1.422580242 |
| XPOT | Exportin For TRNA | Protein Coding | 36 | GC12P064404 | 1.421591282 |
| SLITRK6 | SLIT And NTRK Like Family Member 6 | Protein Coding | 35 | GC13M085792 | 1.421484113 |
| MCMDC2 | Minichromosome Maintenance Domain Containing 2 | Protein Coding | 27 | GC08P066870 | 1.421462536 |
| TM4SF1-AS1 | TM4SF1 Antisense RNA 1 | RNA Gene | 13 | GC03P149378 | 1.421196938 |
| PRRG4 | Proline Rich And Gla Domain 4 | Protein Coding | 28 | GC11P032851 | 1.420945168 |
| USP19 | Ubiquitin Specific Peptidase 19 | Protein Coding | 35 | GC03M051017 | 1.420896173 |
| DEFA4 | Defensin Alpha 4 | Protein Coding | 32 | GC08M006935 | 1.420591116 |
| CFAP77 | Cilia And Flagella Associated Protein 77 | Protein Coding | 23 | GC09P132499 | 1.420591116 |
| NFE2L3 | NFE2 Like BZIP Transcription Factor 3 | Protein Coding | 35 | GC07P026152 | 1.42013061 |
| SCAF4 | SR-Related CTD Associated Factor 4 | Protein Coding | 31 | GC21M031796 | 1.419710875 |
| ELL3 | Elongation Factor For RNA Polymerase II 3 | Protein Coding | 29 | GC15M043785 | 1.419596553 |
| GALNT4 | Polypeptide N-Acetylgalactosaminyltransferase 4 | Protein Coding | 34 | GC12M089520 | 1.419043303 |
| ACOT13 | Acyl-CoA Thioesterase 13 | Protein Coding | 33 | GC06P024667 | 1.418910503 |
| CYTH2 | Cytohesin 2 | Protein Coding | 36 | GC19P064002 | 1.418661952 |
| TXNL1 | Thioredoxin Like 1 | Protein Coding | 36 | GC18M056597 | 1.418616891 |
| MRPL41 | Mitochondrial Ribosomal Protein L41 | Protein Coding | 31 | GC09P137551 | 1.418251395 |
| B4GALT7 | Beta-1,4-Galactosyltransferase 7 | Protein Coding | 37 | GC05P177600 | 1.417930126 |
| GLRX5 | Glutaredoxin 5 | Protein Coding | 36 | GC14P095533 | 1.417899609 |
| PCLO | Piccolo Presynaptic Cytomatrix Protein | Protein Coding | 37 | GC07M082754 | 1.417637825 |

| | | | | | |
|---|---|---|---|---|---|
| MTF2 | Metal Response Element Binding Transcription Factor 2 | Protein Coding | 34 | GC01P093079 | 1.41734457 |
| EMX2OS | EMX2 Opposite Strand/Antisense RNA | RNA Gene | 20 | GC10M117473 | 1.416864395 |
| POGLUT3 | Protein O-Glucosyltransferase 3 | Protein Coding | 25 | GC11M108473 | 1.416857243 |
| RBMS2 | RNA Binding Motif Single Stranded Interacting Protein 2 | Protein Coding | 32 | GC12P057210 | 1.416830659 |
| C11orf1 | Chromosome 11 Open Reading Frame 1 | Protein Coding | 28 | GC11P111878 | 1.416710019 |
| NINJ1 | Ninjurin 1 | Protein Coding | 36 | GC09M093121 | 1.416705132 |
| GRAMD1B | GRAM Domain Containing 1B | Protein Coding | 30 | GC11P123358 | 1.416507244 |
| ARF3 | ADP Ribosylation Factor 3 | Protein Coding | 34 | GC12M049383 | 1.41605854 |
| ARL6IP4 | ADP Ribosylation Factor Like GTPase 6 Interacting Protein 4 | Protein Coding | 30 | GC12P122980 | 1.41597867 |
| NT5C1B | 5'-Nucleotidase, Cytosolic IB | Protein Coding | 34 | GC02M018562 | 1.415873766 |
| QSOX2 | Quiescin Sulfhydryl Oxidase 2 | Protein Coding | 33 | GC09M136206 | 1.415753365 |
| MIR525 | MicroRNA 525 | RNA Gene | 17 | GC19P053697 | 1.415706158 |
| MIR329-1 | MicroRNA 329-1 | RNA Gene | 13 | GC14P109519 | 1.415621758 |
| INTS5 | Integrator Complex Subunit 5 | Protein Coding | 31 | GC11M087130 | 1.414935827 |
| PRG4 | Proteoglycan 4 | Protein Coding | 38 | GC01P186296 | 1.414778709 |
| SMIM10L2A | Small Integral Membrane Protein 10 Like 2A | Protein Coding | 14 | GC0XP135421 | 1.414631605 |
| MRPL22 | Mitochondrial Ribosomal Protein L22 | Protein Coding | 33 | GC05P154941 | 1.414609075 |
| ZNF221 | Zinc Finger Protein 221 | Protein Coding | 29 | GC19P043951 | 1.414340734 |
| COX6A1 | Cytochrome C Oxidase Subunit 6A1 | Protein Coding | 39 | GC12P120439 | 1.414310098 |
| MBNL1-AS1 | MBNL1 Antisense RNA 1 | RNA Gene | 13 | GC03M152215 | 1.414282799 |
| MOSPD3 | Motile Sperm Domain Containing 3 | Protein Coding | 30 | GC07P100985 | 1.414270163 |
| PSORS1C1 | Psoriasis Susceptibility 1 Candidate 1 | Protein Coding | 29 | GC06P031114 | 1.414067864 |
| SLC9A8 | Solute Carrier Family 9 Member A8 | Protein Coding | 35 | GC20P049813 | 1.413992882 |
| PHLDB3 | Pleckstrin Homology Like Domain Family B Member 3 | Protein Coding | 28 | GC19M043474 | 1.41390872 |
| HOXB6 | Homeobox B6 | Protein Coding | 34 | GC17M048717 | 1.413900971 |
| NOC2L | NOC2 Like Nucleolar Associated Transcriptional Repressor | Protein Coding | 34 | GC01M005507 | 1.413767576 |
| MAP7D1 | MAP7 Domain Containing 1 | Protein Coding | 29 | GC01P036155 | 1.413726091 |
| MARCO | Macrophage Receptor With Collagenous Structure | Protein Coding | 37 | GC02P118942 | 1.413397908 |
| CYP2D7 | Cytochrome P450 Family 2 Subfamily D Member 7 (Gene/Pseudogene) | Protein Coding | 19 | GC22M056124 | 1.413016915 |
| LRRC1 | Leucine Rich Repeat Containing 1 | Protein Coding | 33 | GC06P080737 | 1.412997007 |
| STX10 | Syntaxin 10 | Protein Coding | 31 | GC19M013144 | 1.412620425 |
| NPSR1 | Neuropeptide S Receptor 1 | Protein Coding | 39 | GC07P034664 | 1.412443757 |
| HMBOX1 | Homeobox Containing 1 | Protein Coding | 32 | GC08P028890 | 1.412404776 |
| KATNAL1 | Katanin Catalytic Subunit A1 Like 1 | Protein Coding | 34 | GC13M030202 | 1.412284136 |
| PNMA1 | PNMA Family Member 1 | Protein Coding | 30 | GC14M073831 | 1.41224885 |
| ARPC1A | Actin Related Protein 2/3 Complex Subunit 1A | Protein Coding | 35 | GC07P099325 | 1.411780596 |
| HMGCS2 | 3-Hydroxy-3-Methylglutaryl-CoA Synthase 2 | Protein Coding | 42 | GC01M119747 | 1.411746979 |
| MED20 | Mediator Complex Subunit 20 | Protein Coding | 31 | GC06M041905 | 1.411380768 |
| PHC2 | Polyhomeotic Homolog 2 | Protein Coding | 35 | GC01M033426 | 1.411020517 |
| ERGIC1 | Endoplasmic Reticulum-Golgi Intermediate Compartment 1 | Protein Coding | 35 | GC05P172834 | 1.410589337 |
| MIR1287 | MicroRNA 1287 | RNA Gene | 14 | GC10M098395 | 1.410114527 |
| SURF6 | Surfeit 6 | Protein Coding | 31 | GC09M133331 | 1.409381032 |
| RAB34 | RAB34, Member RAS Oncogene Family | Protein Coding | 34 | GC17M028714 | 1.409311652 |
| TAOK3 | TAO Kinase 3 | Protein Coding | 35 | GC12M118149 | 1.409109592 |
| ZNF45 | Zinc Finger Protein 45 | Protein Coding | 33 | GC19M063909 | 1.409090519 |
| ITPKA | Inositol-Trisphosphate 3-Kinase A | Protein Coding | 36 | GC15P041493 | 1.40894413 |
| ANKRD62P1-PARP4P3 | ANKRD62P1-PARP4P3 Readthrough, Transcribed Pseudogene | Pseudogene | 8 | GC22M016688 | 1.408692837 |
| CYTH3 | Cytohesin 3 | Protein Coding | 33 | GC07M006161 | 1.408652544 |
| MIR522 | MicroRNA 522 | RNA Gene | 14 | GC19P053751 | 1.408262968 |
| UBP1 | Upstream Binding Protein 1 | Protein Coding | 32 | GC03M033404 | 1.407763481 |
| WASH3P | WASP Family Homolog 3, Pseudogene | Pseudogene | 18 | GC15P101960 | 1.407721996 |
| TIMM44 | Translocase Of Inner Mitochondrial Membrane 44 | Protein Coding | 33 | GC19M007926 | 1.407496691 |
| RRP15 | Ribosomal RNA Processing 15 Homolog | Protein Coding | 30 | GC01P218285 | 1.407243252 |
| COPZ1 | COPI Coat Complex Subunit Zeta 1 | Protein Coding | 35 | GC12P054301 | 1.406921506 |
| CRYM | Crystallin Mu | Protein Coding | 39 | GC16M021238 | 1.406799316 |
| ITIH2 | Inter-Alpha-Trypsin Inhibitor Heavy Chain 2 | Protein Coding | 36 | GC10P007703 | 1.406774282 |
| EML2 | EMAP Like 2 | Protein Coding | 30 | GC19M045606 | 1.406392097 |
| LRRC34 | Leucine Rich Repeat Containing 34 | Protein Coding | 29 | GC03M169793 | 1.406289697 |
| MIR937 | MicroRNA 937 | RNA Gene | 15 | GC08M143812 | 1.405994773 |
| ADAMTS16 | ADAM Metallopeptidase With Thrombospondin Type 1 Motif 16 | Protein Coding | 35 | GC05P005140 | 1.405731559 |
| SNRNP27 | Small Nuclear Ribonucleoprotein U4/U6.U5 Subunit 27 | Protein Coding | 30 | GC02P069893 | 1.405731559 |
| LTN1 | Listerin E3 Ubiquitin Protein Ligase 1 | Protein Coding | 30 | GC21M028928 | 1.405296564 |
| KLHDC2 | Kelch Domain Containing 2 | Protein Coding | 33 | GC14P049767 | 1.4047997 |
| SUMF2 | Sulfatase Modifying Factor 2 | Protein Coding | 35 | GC07P056595 | 1.404686451 |
| RAB3IP | RAB3A Interacting Protein | Protein Coding | 34 | GC12P069738 | 1.404052615 |
| SLTM | SAFB Like Transcription Modulator | Protein Coding | 30 | GC15M058879 | 1.403703332 |
| PBLD | Phenazine Biosynthesis Like Protein Domain Containing | Protein Coding | 34 | GC10M068282 | 1.403432846 |
| TMEM109 | Transmembrane Protein 109 | Protein Coding | 27 | GC11P060914 | 1.403146029 |
| AP5Z1 | Adaptor Related Protein Complex 5 Subunit Zeta 1 | Protein Coding | 32 | GC07P004775 | 1.403094769 |
| KATNAL2 | Katanin Catalytic Subunit A1 Like 2 | Protein Coding | 32 | GC18P046917 | 1.402567387 |
| GCNA | Germ Cell Nuclear Acidic Peptidase | Protein Coding | 25 | GC0XP071578 | 1.402432919 |
| SYT8 | Synaptotagmin 8 | Protein Coding | 32 | GC11P001829 | 1.402355909 |
| MIR5702 | MicroRNA 5702 | RNA Gene | 10 | GC02M226658 | 1.401651144 |
| DBP | D-Box Binding PAR BZIP Transcription Factor | Protein Coding | 34 | GC19M048630 | 1.401634455 |
| MIR561 | MicroRNA 561 | RNA Gene | 17 | GC02P188297 | 1.401434898 |
| SRPX | Sushi Repeat Containing Protein X-Linked | Protein Coding | 35 | GC0XM038149 | 1.401377797 |
| PLD5 | Phospholipase D Family Member 5 | Protein Coding | 32 | GC01M242082 | 1.401172519 |
| TMPO-AS1 | TMPO Antisense RNA 1 | RNA Gene | 13 | GC12M098512 | 1.401081085 |
| AGPAT5 | 1-Acylglycerol-3-Phosphate O-Acyltransferase 5 | Protein Coding | 36 | GC08P006708 | 1.40100956 |
| FNBP4 | Formin Binding Protein 4 | Protein Coding | 31 | GC11M086894 | 1.400999665 |
| STARD4 | StAR Related Lipid Transfer Domain Containing 4 | Protein Coding | 35 | GC05M111496 | 1.400746584 |
| ZNF512 | Zinc Finger Protein 512 | Protein Coding | 28 | GC02P027582 | 1.400496483 |
| STK38L | Serine/Threonine Kinase 38 Like | Protein Coding | 39 | GC12P027243 | 1.400429964 |
| NOC4L | Nucleolar Complex Associated 4 Homolog | Protein Coding | 30 | GC12P132144 | 1.400284886 |
| INTS3 | Integrator Complex Subunit 3 | Protein Coding | 32 | GC01P153728 | 1.39966321 |
| AGAP3 | ArfGAP With GTPase Domain, Ankyrin Repeat And PH Domain 3 | Protein Coding | 31 | GC07P151085 | 1.399580002 |
| FBXO28 | F-Box Protein 28 | Protein Coding | 31 | GC01P224115 | 1.399577737 |
| RIMKLB | Ribosomal Modification Protein RimK Like Family Member B | Protein Coding | 30 | GC12P008681 | 1.399522305 |
| RUSC1 | RUN And SH3 Domain Containing 1 | Protein Coding | 31 | GC01P155320 | 1.399416327 |
| DMRT2 | Doublesex And Mab-3 Related Transcription Factor 2 | Protein Coding | 32 | GC09P001003 | 1.398835063 |
| CYREN | Cell Cycle Regulator Of NHEJ | Protein Coding | 25 | GC07M135130 | 1.398622751 |
| RELT | RELT TNF Receptor | Protein Coding | 37 | GC11P073376 | 1.398566127 |
| AGAP4 | ArfGAP With GTPase Domain, Ankyrin Repeat And PH Domain 4 | Protein Coding | 27 | GC10M045825 | 1.398498774 |
| EEF1E1 | Eukaryotic Translation Elongation Factor 1 Epsilon 1 | Protein Coding | 36 | GC06M008073 | 1.397719026 |
| RFXAP | Regulatory Factor X Associated Protein | Protein Coding | 33 | GC13P036819 | 1.397654176 |

| | | | | | | |
|---|---|---|---|---|---|---|
| NABP2 | Nucleic Acid Binding Protein 2 | Protein Coding | 29 | GC12P057180 | 1.397520781 |
| SNORD50A | Small Nucleolar RNA, C/D Box 50A | RNA Gene | 15 | GC06M085677 | 1.397375345 |
| MYCNUT | MYCN Upstream Transcript | RNA Gene | 13 | GC02P015920 | 1.397372365 |
| CAPZA2 | Capping Actin Protein Of Muscle Z-Line Subunit Alpha 2 | Protein Coding | 35 | GC07P116811 | 1.39731431 |
| AADAC | Arylacetamide Deacetylase | Protein Coding | 39 | GC03P151813 | 1.39721477 |
| PHKG2 | Phosphorylase Kinase Catalytic Subunit Gamma 2 | Protein Coding | 42 | GC16P040275 | 1.397214532 |
| NRON | Non-Coding Repressor Of NFAT | RNA Gene | 17 | GC09M126407 | 1.396863818 |
| LGALSL | Galectin Like | Protein Coding | 30 | GC02P064453 | 1.396721482 |
| SNX5 | Sorting Nexin 5 | Protein Coding | 37 | GC20M018125 | 1.396659851 |
| CCDC12 | Coiled-Coil Domain Containing 12 | Protein Coding | 27 | GC03M046930 | 1.396564007 |
| CLBA1 | Clathrin Binding Box Of Aftiphilin Containing 1 | Protein Coding | 22 | GC14P109291 | 1.396184206 |
| LOXL3 | Lysyl Oxidase Like 3 | Protein Coding | 38 | GC02M074532 | 1.395699501 |
| MIR4458 | MicroRNA 4458 | RNA Gene | 11 | GC05P008460 | 1.395341396 |
| NVL | Nuclear VCP Like | Protein Coding | 34 | GC01M224227 | 1.3948915 |
| OTOR | Otoraplin | Protein Coding | 34 | GC20P016748 | 1.394888401 |
| OSBP2 | Oxysterol Binding Protein 2 | Protein Coding | 35 | GC22P030693 | 1.394671559 |
| DNAL4 | Dynein Axonemal Light Chain 4 | Protein Coding | 35 | GC22M038778 | 1.394651294 |
| S100A13 | S100 Calcium Binding Protein A13 | Protein Coding | 32 | GC01M153618 | 1.393861651 |
| FFAR1 | Free Fatty Acid Receptor 1 | Protein Coding | 37 | GC19P064593 | 1.393244863 |
| ZNF768 | Zinc Finger Protein 768 | Protein Coding | 27 | GC16M030524 | 1.393225908 |
| MIR765 | MicroRNA 765 | RNA Gene | 17 | GC01M156905 | 1.392177105 |
| CHRNE | Cholinergic Receptor Nicotinic Epsilon Subunit | Protein Coding | 40 | GC17M004897 | 1.392170668 |
| CSTF2 | Cleavage Stimulation Factor Subunit 2 | Protein Coding | 33 | GC0XP100820 | 1.39205277 |
| BZW2 | Basic Leucine Zipper And W2 Domains 2 | Protein Coding | 35 | GC07P016646 | 1.391883731 |
| CDC14B | Cell Division Cycle 14B | Protein Coding | 36 | GC09M096490 | 1.391514301 |
| AAR2 | AAR2 Splicing Factor | Protein Coding | 31 | GC20P036236 | 1.391432762 |
| BPIFB1 | BPI Fold Containing Family B Member 1 | Protein Coding | 30 | GC20P033273 | 1.390615106 |
| FCGR2C | Fc Gamma Receptor IIc (Gene/Pseudogene) | Protein Coding | 30 | GC01P161683 | 1.390455127 |
| NXN | Nucleoredoxin | Protein Coding | 39 | GC17M000799 | 1.390408993 |
| ZNF232 | Zinc Finger Protein 232 | Protein Coding | 28 | GC17M005105 | 1.390275478 |
| TMEM140 | Transmembrane Protein 140 | Protein Coding | 26 | GC07P135148 | 1.390275478 |
| GMCL2 | Germ Cell-Less 2, Spermatogenesis Associated | Protein Coding | 19 | GC05M178189 | 1.390275478 |
| SWAP70 | Switching B Cell Complex Subunit SWAP70 | Protein Coding | 36 | GC11P009664 | 1.389786005 |
| CASTOR2 | Cytosolic Arginine Sensor For MTORC1 Subunit 2 | Protein Coding | 22 | GC07P075202 | 1.389693618 |
| SLC37A2 | Solute Carrier Family 37 Member 2 | Protein Coding | 32 | GC11P125063 | 1.389538884 |
| MTO1 | Mitochondrial TRNA Translation Optimization 1 | Protein Coding | 38 | GC06P073461 | 1.389167666 |
| CHAMP1 | Chromosome Alignment Maintaining Phosphoprotein 1 | Protein Coding | 35 | GC13P114314 | 1.388963461 |
| HOXC11 | Homeobox C11 | Protein Coding | 32 | GC12P053973 | 1.387706757 |
| C14orf39 | Chromosome 14 Open Reading Frame 39 | Protein Coding | 31 | GC14M060396 | 1.387424469 |
| ZSWIM4 | Zinc Finger SWIM-Type Containing 4 | Protein Coding | 27 | GC19P013795 | 1.387126803 |
| CNOT10 | CCR4-NOT Transcription Complex Subunit 10 | Protein Coding | 30 | GC03P032685 | 1.386940718 |
| NDUFB8 | NADH:Ubiquinone Oxidoreductase Subunit B8 | Protein Coding | 38 | GC10M100523 | 1.386541367 |
| TENM2 | Teneurin Transmembrane Protein 2 | Protein Coding | 31 | GC05P166979 | 1.386528492 |
| EPB41L4B | Erythrocyte Membrane Protein Band 4.1 Like 4B | Protein Coding | 33 | GC09M109171 | 1.386330366 |
| H2BC11 | H2B Clustered Histone 11 | Protein Coding | 27 | GC06M063257 | 1.386281252 |
| PDSS1 | Decaprenyl Diphosphate Synthase Subunit 1 | Protein Coding | 38 | GC10P026697 | 1.385732412 |
| SNORD116-1 | Small Nucleolar RNA, C/D Box 116-1 | RNA Gene | 15 | GC15P039157 | 1.385580182 |
| ZNF214 | Zinc Finger Protein 214 | Protein Coding | 30 | GC11M006996 | 1.385437489 |
| EIF3K | Eukaryotic Translation Initiation Factor 3 Subunit K | Protein Coding | 34 | GC19P038619 | 1.385412097 |
| MICAL3 | Microtubule Associated Monooxygenase, Calponin And LIM Domain Containing 3 | Protein Coding | 31 | GC22M017830 | 1.385252833 |
| TIMM21 | Translocase Of Inner Mitochondrial Membrane 21 | Protein Coding | 28 | GC18P074148 | 1.385151267 |
| RPL13P12 | Ribosomal Protein L13 Pseudogene 12 | Pseudogene | 10 | GC17M017383 | 1.384776115 |
| MIR663B | MicroRNA 663b | RNA Gene | 14 | GC02M133858 | 1.384690046 |
| MIR1285-1 | MicroRNA 1285-1 | RNA Gene | 11 | GC07M092204 | 1.384606361 |
| OSBPL3 | Oxysterol Binding Protein Like 3 | Protein Coding | 35 | GC07M024836 | 1.384501457 |
| SMIM38 | Small Integral Membrane Protein 38 | Protein Coding | 11 | GC11P069826 | 1.384497643 |
| RGS8 | Regulator Of G Protein Signaling 8 | Protein Coding | 34 | GC01M183386 | 1.38447237 |
| DCSTAMP | Dendrocyte Expressed Seven Transmembrane Protein | Protein Coding | 32 | GC08P104339 | 1.384063244 |
| MIR718 | MicroRNA 718 | RNA Gene | 13 | GC0XM154019 | 1.382713914 |
| CRIP1 | Cysteine Rich Protein 1 | Protein Coding | 33 | GC14P105486 | 1.381810308 |
| IL36G | Interleukin 36 Gamma | Protein Coding | 33 | GC02P112973 | 1.381453395 |
| RPP30 | Ribonuclease P/MRP Subunit P30 | Protein Coding | 34 | GC10P090871 | 1.38141942 |
| WDR55 | WD Repeat Domain 55 | Protein Coding | 28 | GC05P146095 | 1.381236792 |
| TOMM40L | Translocase Of Outer Mitochondrial Membrane 40 Like | Protein Coding | 30 | GC01P161225 | 1.381198525 |
| ZNF572 | Zinc Finger Protein 572 | Protein Coding | 27 | GC08P124973 | 1.381198525 |
| VNN2 | Vanin 2 | Protein Coding | 37 | GC06M132743 | 1.381198406 |
| PCP4 | Purkinje Cell Protein 4 | Protein Coding | 32 | GC21P039867 | 1.380775094 |
| CIDEA | Cell Death Inducing DFFA Like Effector A | Protein Coding | 36 | GC18P012254 | 1.380702257 |
| HYAL4 | Hyaluronidase 4 | Protein Coding | 35 | GC07P123784 | 1.380306721 |
| HSPA4L | Heat Shock Protein Family A (Hsp70) Member 4 Like | Protein Coding | 35 | GC04P127781 | 1.379590511 |
| TARP | TCR Gamma Alternate Reading Frame Protein | Protein Coding | 19 | GC07M039021 | 1.379480243 |
| TMED3 | Transmembrane P24 Trafficking Protein 3 | Protein Coding | 30 | GC15P079311 | 1.379453182 |
| ZNF48 | Zinc Finger Protein 48 | Protein Coding | 27 | GC16P030378 | 1.37936151 |
| PRIM2 | DNA Primase Subunit 2 | Protein Coding | 35 | GC06P057314 | 1.378567338 |
| ZRANB2 | Zinc Finger RANBP2-Type Containing 2 | Protein Coding | 31 | GC01M071063 | 1.378401041 |
| BRPF1 | Bromodomain And PHD Finger Containing 1 | Protein Coding | 36 | GC03P009731 | 1.378376126 |
| CYP51A1-AS1 | CYP51A1 Antisense RNA 1 | RNA Gene | 12 | GC07P092134 | 1.378094673 |
| COA8 | Cytochrome C Oxidase Assembly Factor 8 | Protein Coding | 27 | GC14P109321 | 1.378002882 |
| SNX2 | Sorting Nexin 2 | Protein Coding | 35 | GC05P122774 | 1.377534986 |
| MRPS15 | Mitochondrial Ribosomal Protein S15 | Protein Coding | 31 | GC01M036455 | 1.377517939 |
| KDM4E | Lysine Demethylase 4E | Protein Coding | 26 | GC11P095026 | 1.377324581 |
| RBM7 | RNA Binding Motif Protein 7 | Protein Coding | 34 | GC11P114401 | 1.37716651 |
| IDI1 | Isopentenyl-Diphosphate Delta Isomerase 1 | Protein Coding | 38 | GC10M001039 | 1.37684238 |
| INPP5J | Inositol Polyphosphate-5-Phosphatase J | Protein Coding | 36 | GC22P035013 | 1.37676096 |
| CYLC1 | Cylicin 1 | Protein Coding | 28 | GC0XP083861 | 1.376098752 |
| ACAP3 | ArfGAP With Coiled-Coil, Ankyrin Repeat And PH Domains 3 | Protein Coding | 32 | GC01M001292 | 1.376036167 |
| ITSN2 | Intersectin 2 | Protein Coding | 35 | GC02M024203 | 1.375700712 |
| PSMD5 | Proteasome 26S Subunit, Non-ATPase 5 | Protein Coding | 34 | GC09M120815 | 1.375629544 |
| MIOS | Meiosis Regulator For Oocyte Development | Protein Coding | 33 | GC07P007579 | 1.375440598 |
| NEURL1 | Neuralized E3 Ubiquitin Protein Ligase 1 | Protein Coding | 32 | GC10P103493 | 1.375387907 |
| BTN1A1 | Butyrophilin Subfamily 1 Member A1 | Protein Coding | 36 | GC06P026500 | 1.375212908 |
| PCBD2 | Pterin-4 Alpha-Carbinolamine Dehydratase 2 | Protein Coding | 33 | GC05P134904 | 1.375133276 |
| MYADM | Myeloid Associated Differentiation Marker | Protein Coding | 33 | GC19P064311 | 1.374913692 |
| WWC3 | WWC Family Member 3 | Protein Coding | 29 | GC0XP010015 | 1.374671221 |
| EIF2B3 | Eukaryotic Translation Initiation Factor 2B Subunit Gamma | Protein Coding | 37 | GC01M044850 | 1.374122739 |

| | | | | | | |
|---|---|---|---|---|---|---|
| TGS1 | Trimethylguanosine Synthase 1 | Protein Coding | 35 | GC08P055773 | 1.374005318 |
| LARP6 | La Ribonucleoprotein 6, Translational Regulator | Protein Coding | 31 | GC15M070829 | 1.373932123 |
| NIT2 | Nitrilase Family Member 2 | Protein Coding | 34 | GC03P100334 | 1.373594522 |
| DISC1 | DISC1 Scaffold Protein | Protein Coding | 40 | GC01P231626 | 1.373260975 |
| SARDH | Sarcosine Dehydrogenase | Protein Coding | 38 | GC09M133663 | 1.372967482 |
| TBCA | Tubulin Folding Cofactor A | Protein Coding | 33 | GC05M077691 | 1.372753978 |
| NOL10 | Nucleolar Protein 10 | Protein Coding | 30 | GC02M010662 | 1.372399807 |
| SMC5 | Structural Maintenance Of Chromosomes 5 | Protein Coding | 33 | GC09P070258 | 1.372236013 |
| HNRNPA1L2 | Heterogeneous Nuclear Ribonucleoprotein A1 Like 2 | Protein Coding | 28 | GC13P052617 | 1.372188568 |
| ADAMTS12 | ADAM Metallopeptidase With Thrombospondin Type 1 Motif 12 | Protein Coding | 36 | GC05M033524 | 1.372004271 |
| ZACN | Zinc Activated Ion Channel | Protein Coding | 30 | GC17P076071 | 1.371836185 |
| ZNF43 | Zinc Finger Protein 43 | Protein Coding | 31 | GC19M032587 | 1.371158361 |
| ZNF560 | Zinc Finger Protein 560 | Protein Coding | 26 | GC19M009445 | 1.371158361 |
| KLC2 | Kinesin Light Chain 2 | Protein Coding | 37 | GC11P066257 | 1.370883822 |
| HPS4 | HPS4 Biogenesis Of Lysosomal Organelles Complex 3 Subunit 2 | Protein Coding | 35 | GC22M026443 | 1.370759606 |
| RBM12 | RNA Binding Motif Protein 12 | Protein Coding | 33 | GC20M035648 | 1.370576978 |
| INPP1 | Inositol Polyphosphate-1-Phosphatase | Protein Coding | 37 | GC02P190343 | 1.370376706 |
| RDH13 | Retinol Dehydrogenase 13 | Protein Coding | 35 | GC19M055039 | 1.370233655 |
| TFB2M | Transcription Factor B2, Mitochondrial | Protein Coding | 32 | GC01M246540 | 1.370197177 |
| MLEC | Malectin | Protein Coding | 32 | GC12P120687 | 1.370019197 |
| NGLY1 | N-Glycanase 1 | Protein Coding | 39 | GC03M025718 | 1.369860888 |
| CCNP | Cyclin P | Protein Coding | 21 | GC19M064571 | 1.369809985 |
| PCBP3 | Poly(RC) Binding Protein 3 | Protein Coding | 32 | GC21P045643 | 1.369692564 |
| MMADHC | Metabolism Of Cobalamin Associated D | Protein Coding | 38 | GC02M149569 | 1.369669676 |
| ZNF473 | Zinc Finger Protein 473 | Protein Coding | 32 | GC19P064107 | 1.369590282 |
| TOMM34 | Translocase Of Outer Mitochondrial Membrane 34 | Protein Coding | 34 | GC20M044942 | 1.369303465 |
| TNRC6C | Trinucleotide Repeat Containing Adaptor 6C | Protein Coding | 31 | GC17P077959 | 1.369212389 |
| ATP5ME | ATP Synthase Membrane Subunit E | Protein Coding | 29 | GC04M001417 | 1.369142294 |
| CTF1 | Cardiotrophin 1 | Protein Coding | 34 | GC16P040280 | 1.36907649 |
| DDX51 | DEAD-Box Helicase 51 | Protein Coding | 30 | GC12M132136 | 1.3690418 |
| PROZ | Protein Z, Vitamin K Dependent Plasma Glycoprotein | Protein Coding | 38 | GC13P113158 | 1.367945671 |
| SHANK2 | SH3 And Multiple Ankyrin Repeat Domains 2 | Protein Coding | 39 | GC11M070467 | 1.367847681 |
| KLF3 | Kruppel Like Factor 3 | Protein Coding | 34 | GC04P038668 | 1.36780262 |
| METAP1 | Methionyl Aminopeptidase 1 | Protein Coding | 37 | GC04P098995 | 1.367702007 |
| CUEDC1 | CUE Domain Containing 1 | Protein Coding | 32 | GC17M057861 | 1.367686272 |
| KLHDC8A | Kelch Domain Containing 8A | Protein Coding | 31 | GC01M205305 | 1.367664576 |
| ENSG00000264545 | Novel Transcript | Protein Coding | 8 | GC09P021802 | 1.366880774 |
| NRXN2 | Neurexin 2 | Protein Coding | 36 | GC11M087198 | 1.364687681 |
| LPCAT4 | Lysophosphatidylcholine Acyltransferase 4 | Protein Coding | 34 | GC15M034358 | 1.364608645 |
| TBCD | Tubulin Folding Cofactor D | Protein Coding | 36 | GC17P082752 | 1.36410141 |
| NDUFS8 | NADH:Ubiquinone Oxidoreductase Core Subunit S8 | Protein Coding | 40 | GC11P068030 | 1.363448858 |
| RABGEF1 | RAB Guanine Nucleotide Exchange Factor 1 | Protein Coding | 38 | GC07P070017 | 1.363434911 |
| C1QTNF5 | C1q And TNF Related 5 | Protein Coding | 34 | GC11M119338 | 1.363396645 |
| CLCC1 | Chloride Channel CLIC Like 1 | Protein Coding | 35 | GC01M108881 | 1.362955332 |
| MIR577 | MicroRNA 577 | RNA Gene | 16 | GC04P114656 | 1.362652421 |
| SRR | Serine Racemase | Protein Coding | 39 | GC17P002303 | 1.362564921 |
| MRPL38 | Mitochondrial Ribosomal Protein L38 | Protein Coding | 32 | GC17M075900 | 1.362434983 |
| TIMD4 | T Cell Immunoglobulin And Mucin Domain Containing 4 | Protein Coding | 33 | GC05M156919 | 1.362165809 |
| MPHOSPH6 | M-Phase Phosphoprotein 6 | Protein Coding | 31 | GC16M082181 | 1.36183691 |
| KAZN-AS1 | KAZN Antisense RNA 1 | RNA Gene | 10 | GC01M014350 | 1.361747503 |
| MRPL9 | Mitochondrial Ribosomal Protein L9 | Protein Coding | 30 | GC01M151759 | 1.361612558 |
| ARPC5L | Actin Related Protein 2/3 Complex Subunit 5 Like | Protein Coding | 32 | GC09P124862 | 1.361345649 |
| SYT2 | Synaptotagmin 2 | Protein Coding | 40 | GC01M202559 | 1.360893846 |
| DNAJC3 | DnaJ Heat Shock Protein Family (Hsp40) Member C3 | Protein Coding | 36 | GC13P095677 | 1.360807896 |
| BAIAP2L2 | BAR/IMD Domain Containing Adaptor Protein 2 Like 2 | Protein Coding | 31 | GC22M056266 | 1.360150814 |
| CORT | Cortistatin | Protein Coding | 34 | GC01P010499 | 1.35998559 |
| GDA | Guanine Deaminase | Protein Coding | 39 | GC09P072114 | 1.359881401 |
| CHST8 | Carbohydrate Sulfotransferase 8 | Protein Coding | 36 | GC19P033621 | 1.359762192 |
| ZNF480 | Zinc Finger Protein 480 | Protein Coding | 31 | GC19P052297 | 1.359762192 |
| SPNS3 | Sphingolipid Transporter 3 (Putative) | Protein Coding | 30 | GC17P004433 | 1.359762192 |
| MS4A5 | Membrane Spanning 4-Domains A5 | Protein Coding | 28 | GC11P060447 | 1.359762192 |
| C12orf76 | Chromosome 12 Open Reading Frame 76 | Protein Coding | 27 | GC12M110022 | 1.359762192 |
| PDILT | Protein Disulfide Isomerase Like, Testis Expressed | Protein Coding | 27 | GC16M020370 | 1.359762192 |
| PLPPR2 | Phospholipid Phosphatase Related 2 | Protein Coding | 27 | GC19P011465 | 1.359762192 |
| SLC66A2 | Solute Carrier Family 66 Member 2 | Protein Coding | 27 | GC18M079903 | 1.359762192 |
| NKAP | NFKB Activating Protein | Protein Coding | 32 | GC0XM120119 | 1.359125614 |
| PELO | Pelota MRNA Surveillance And Ribosome Rescue Factor | Protein Coding | 32 | GC05P052787 | 1.359046459 |
| UBXN1 | UBX Domain Protein 1 | Protein Coding | 31 | GC11M087133 | 1.358757257 |
| DDX55 | DEAD-Box Helicase 55 | Protein Coding | 32 | GC12P123602 | 1.358342886 |
| SEPTIN5 | Septin 5 | Protein Coding | 30 | GC22P019714 | 1.357748628 |
| SOCS4 | Suppressor Of Cytokine Signaling 4 | Protein Coding | 35 | GC14P055027 | 1.357600927 |
| MIR466 | MicroRNA 466 | RNA Gene | 13 | GC03M031161 | 1.357548237 |
| INAVA | Innate Immunity Activator | Protein Coding | 27 | GC01P200892 | 1.357521296 |
| RBM22 | RNA Binding Motif Protein 22 | Protein Coding | 32 | GC05M150690 | 1.357515097 |
| SLITRK3 | SLIT And NTRK Like Family Member 3 | Protein Coding | 33 | GC03M165184 | 1.356613874 |
| CEACAM4 | CEA Cell Adhesion Molecule 4 | Protein Coding | 31 | GC19M063832 | 1.356190801 |
| DDAH1 | Dimethylarginine Dimethylaminohydrolase 1 | Protein Coding | 39 | GC01M085318 | 1.356015444 |
| GAN | Gigaxonin | Protein Coding | 35 | GC16P081319 | 1.355981827 |
| C9orf78 | Chromosome 9 Open Reading Frame 78 | Protein Coding | 29 | GC09M129827 | 1.355873108 |
| MYO5C | Myosin VC | Protein Coding | 34 | GC15M082020 | 1.355658174 |
| EMX1 | Empty Spiracles Homeobox 1 | Protein Coding | 32 | GC02P072916 | 1.355606437 |
| SYT5 | Synaptotagmin 5 | Protein Coding | 34 | GC19M064405 | 1.355374217 |
| ANAPC11 | Anaphase Promoting Complex Subunit 11 | Protein Coding | 35 | GC17P081890 | 1.35496068 |
| RPF1 | Ribosome Production Factor 1 Homolog | Protein Coding | 30 | GC01P084479 | 1.353565693 |
| SENP8 | SUMO Peptidase Family Member, NEDD8 Specific | Protein Coding | 35 | GC15P114696 | 1.352987289 |
| MIR1184-1 | MicroRNA 1184-1 | RNA Gene | 11 | GC0XM154887 | 1.352388382 |
| SYCP3 | Synaptonemal Complex Protein 3 | Protein Coding | 38 | GC12M101728 | 1.352345467 |
| GPANK1 | G-Patch Domain And Ankyrin Repeats 1 | Protein Coding | 29 | GC06M063611 | 1.352255702 |
| DISP1 | Dispatched RND Transporter Family Member 1 | Protein Coding | 35 | GC01P222814 | 1.351403356 |
| MUC22 | Mucin 22 | Protein Coding | 21 | GC06P031005 | 1.3505826 |
| PAAF1 | Proteasomal ATPase Associated Factor 1 | Protein Coding | 31 | GC11P073876 | 1.35056591 |
| ZNF639 | Zinc Finger Protein 639 | Protein Coding | 28 | GC03P179323 | 1.350197554 |
| SLITRK1 | SLIT And NTRK Like Family Member 1 | Protein Coding | 36 | GC13M083877 | 1.34980917 |
| INTS1 | Integrator Complex Subunit 1 | Protein Coding | 31 | GC07M001470 | 1.34977293 |
| IDO2 | Indoleamine 2,3-Dioxygenase 2 | Protein Coding | 33 | GC08P039949 | 1.349696875 |

| | | | | | |
|---|---|---|---|---|---|
| MTFR2 | Mitochondrial Fission Regulator 2 | Protein Coding | 28 | GC06M136231 | 1.349627733 |
| CEP135 | Centrosomal Protein 135 | Protein Coding | 35 | GC04P055948 | 1.349344969 |
| CALCOCO2 | Calcium Binding And Coiled-Coil Domain 2 | Protein Coding | 35 | GC17P053489 | 1.348887444 |
| CYB5R1 | Cytochrome B5 Reductase 1 | Protein Coding | 36 | GC01M202964 | 1.348565817 |
| MMS22L | MMS22 Like, DNA Repair Protein | Protein Coding | 29 | GC06M097142 | 1.348276377 |
| FOXJ3 | Forkhead Box J3 | Protein Coding | 31 | GC01M042176 | 1.348178506 |
| ATP5MF | ATP Synthase Membrane Subunit F | Protein Coding | 28 | GC07M099484 | 1.348100662 |
| PLS1 | Plastin 1 | Protein Coding | 38 | GC03P142596 | 1.347801328 |
| PRSS3P2 | PRSS3 Pseudogene 2 | Pseudogene | 15 | GC07Pi00773 | 1.347653508 |
| ITIH3 | Inter-Alpha-Trypsin Inhibitor Heavy Chain 3 | Protein Coding | 38 | GC03P052794 | 1.347485065 |
| MAP7D3 | MAP7 Domain Containing 3 | Protein Coding | 30 | GC0XM136213 | 1.347399831 |
| DPT | Dermatopontin | Protein Coding | 33 | GC01M168696 | 1.346903563 |
| MIR576 | MicroRNA 576 | RNA Gene | 14 | GC04P109488 | 1.346770287 |
| NRDC | Nardilysin Convertase | Protein Coding | 31 | GC01M051805 | 1.34664917 |
| PIGK | Phosphatidylinositol Glycan Anchor Biosynthesis Class K | Protein Coding | 38 | GC01M077088 | 1.346579552 |
| PIGH | Phosphatidylinositol Glycan Anchor Biosynthesis Class H | Protein Coding | 35 | GC14M067581 | 1.346410275 |
| LINC02563 | Long Intergenic Non-Protein Coding RNA 2563 | RNA Gene | 9 | GC17P065460 | 1.346181512 |
| CRIM1 | Cysteine Rich Transmembrane BMP Regulator 1 | Protein Coding | 36 | GC02P036355 | 1.346118212 |
| DGKZ | Diacylglycerol Kinase Zeta | Protein Coding | 39 | GC11P046332 | 1.345970154 |
| AMPD3 | Adenosine Monophosphate Deaminase 3 | Protein Coding | 41 | GC11P010309 | 1.345754385 |
| MDFI | MyoD Family Inhibitor | Protein Coding | 34 | GC06P080574 | 1.345410585 |
| LINC00458 | Long Intergenic Non-Protein Coding RNA 458 | RNA Gene | 14 | GC13M054115 | 1.345119238 |
| MIR1908 | MicroRNA 1908 | RNA Gene | 15 | GC11M061815 | 1.344658136 |
| MED18 | Mediator Complex Subunit 18 | Protein Coding | 30 | GC01P028340 | 1.344461441 |
| METTL18 | Methyltransferase Like 18 | Protein Coding | 27 | GC01M169792 | 1.344390869 |
| PCF11 | PCF11 Cleavage And Polyadenylation Factor Subunit | Protein Coding | 32 | GC11P083156 | 1.344127655 |
| LINC02582 | Long Intergenic Non-Protein Coding RNA 2582 | RNA Gene | 11 | GC18P073325 | 1.343632936 |
| AK1 | Adenylate Kinase 1 | Protein Coding | 42 | GC09M127866 | 1.343220472 |
| ERAP2 | Endoplasmic Reticulum Aminopeptidase 2 | Protein Coding | 36 | GC05P096875 | 1.343176126 |
| CATSPERB | Cation Channel Sperm Associated Auxiliary Subunit Beta | Protein Coding | 31 | GC14M091580 | 1.343064308 |
| BICD2 | BICD Cargo Adaptor 2 | Protein Coding | 36 | GC09M092711 | 1.342721462 |
| PPP2R3B | Protein Phosphatase 2 Regulatory Subunit B"Beta | Protein Coding | 33 | GC0XM000350 | 1.342593908 |
| ZNF713 | Zinc Finger Protein 713 | Protein Coding | 31 | GC07P055887 | 1.342048168 |
| TRIM23 | Tripartite Motif Containing 23 | Protein Coding | 35 | GC05M065589 | 1.342019081 |
| NME3 | NME/NM23 Nucleoside Diphosphate Kinase 3 | Protein Coding | 38 | GC16M001770 | 1.341789484 |
| CSTF2T | Cleavage Stimulation Factor Subunit 2 Tau Variant | Protein Coding | 36 | GC10M051695 | 1.341711044 |
| AGBL2 | AGBL Carboxypeptidase 2 | Protein Coding | 34 | GC11M086893 | 1.341367483 |
| SNX13 | Sorting Nexin 13 | Protein Coding | 33 | GC07M017798 | 1.341122985 |
| PFDN5 | Prefoldin Subunit 5 | Protein Coding | 35 | GC12P053295 | 1.341041803 |
| CYP2W1 | Cytochrome P450 Family 2 Subfamily W Member 1 | Protein Coding | 35 | GC07P000983 | 1.341017246 |
| GSDMD | Gasdermin D | Protein Coding | 35 | GC08P143553 | 1.340979815 |
| NDUFB4 | NADH:Ubiquinone Oxidoreductase Subunit B4 | Protein Coding | 35 | GC03P120596 | 1.340956926 |
| TEAD2 | TEA Domain Transcription Factor 2 | Protein Coding | 36 | GC19M064108 | 1.340778708 |
| OSBPL8 | Oxysterol Binding Protein Like 8 | Protein Coding | 34 | GC12M076354 | 1.340460777 |
| POLR2I | RNA Polymerase II Subunit I | Protein Coding | 33 | GC19M036113 | 1.3403579 |
| OMP | Olfactory Marker Protein | Protein Coding | 32 | GC11P077102 | 1.340103865 |
| CPEB3 | Cytoplasmic Polyadenylation Element Binding Protein 3 | Protein Coding | 31 | GC10M092046 | 1.340075731 |
| ATP13A1 | ATPase 13A1 | Protein Coding | 36 | GC19M019645 | 1.339688301 |
| RMDN3 | Regulator Of Microtubule Dynamics 3 | Protein Coding | 29 | GC15M040735 | 1.339430571 |
| RPL13AP3 | Ribosomal Protein L13a Pseudogene 3 | Pseudogene | 14 | GC14P055768 | 1.339191675 |
| GCNT4 | Glucosaminyl (N-Acetyl) Transferase 4 | Protein Coding | 32 | GC05M075025 | 1.338630915 |
| RADX | RPA1 Related Single Stranded DNA Binding Protein, X-Linked | Protein Coding | 25 | GC0XP106612 | 1.338630915 |
| HAUS1 | HAUS Augmin Like Complex Subunit 1 | Protein Coding | 30 | GC18P046104 | 1.337986588 |
| GPATCH1 | G-Patch Domain Containing 1 | Protein Coding | 28 | GC19P033080 | 1.337934732 |
| TMX3 | Thioredoxin Related Transmembrane Protein 3 | Protein Coding | 34 | GC18M068673 | 1.337547064 |
| SCOC | Short Coiled-Coil Protein | Protein Coding | 32 | GC04P140257 | 1.337342501 |
| ANP32C | Acidic Nuclear Phosphoprotein 32 Family Member C | Pseudogene | 23 | GC04M164197 | 1.337300777 |
| VEZF1 | Vascular Endothelial Zinc Finger 1 | Protein Coding | 32 | GC17M057971 | 1.337124109 |
| CDC40 | Cell Division Cycle 40 | Protein Coding | 35 | GC06P110180 | 1.336967707 |
| PITRM1 | Pitrilysin Metallopeptidase 1 | Protein Coding | 37 | GC10M003138 | 1.336952448 |
| ACTN3 | Actinin Alpha 3 | Protein Coding | 35 | GC11P066546 | 1.336207867 |
| RPS10P5 | Ribosomal Protein S10 Pseudogene 5 | Pseudogene | 14 | GC20M000820 | 1.336115956 |
| GPBP1L1 | GC-Rich Promoter Binding Protein 1 Like 1 | Protein Coding | 26 | GC01M045627 | 1.33611083 |
| IGLL5 | Immunoglobulin Lambda Like Polypeptide 5 | Protein Coding | 26 | GC22P034647 | 1.335722685 |
| SHLD1 | Shieldin Complex Subunit 1 | Protein Coding | 24 | GC20P005750 | 1.335585594 |
| ANKS1A | Ankyrin Repeat And Sterile Alpha Motif Domain Containing 1A | Protein Coding | 32 | GC06P080504 | 1.335506797 |
| HIVEP2 | HIVEP Zinc Finger 2 | Protein Coding | 35 | GC06M142751 | 1.334905028 |
| LOC111365141 | NOS2 5' Regulatory Region | Biological Region | 3 | GC17P027800 | 1.334717035 |
| FDXR | Ferredoxin Reductase | Protein Coding | 40 | GC17M074862 | 1.334458828 |
| PIGF | Phosphatidylinositol Glycan Anchor Biosynthesis Class F | Protein Coding | 37 | GC02M046580 | 1.334340811 |
| MIR770 | MicroRNA 770 | RNA Gene | 17 | GC14P109553 | 1.334122181 |
| TUBB8 | Tubulin Beta 8 Class VIII | Protein Coding | 35 | GC10M000048 | 1.334052086 |
| RIMBP2 | RIMS Binding Protein 2 | Protein Coding | 32 | GC12M130396 | 1.33404243 |
| AAK1 | AP2 Associated Kinase 1 | Protein Coding | 37 | GC02M069459 | 1.33357358 |
| ORMDL1 | ORMDL Sphingolipid Biosynthesis Regulator 1 | Protein Coding | 31 | GC02M189764 | 1.333135486 |
| EEA1 | Early Endosome Antigen 1 | Protein Coding | 39 | GC12M092770 | 1.332767248 |
| UBE2L6 | Ubiquitin Conjugating Enzyme E2 L6 | Protein Coding | 34 | GC11M086958 | 1.332692981 |
| DDX42 | DEAD-Box Helicase 42 | Protein Coding | 34 | GC17P063773 | 1.332661271 |
| PRPF38A | Pre-MRNA Processing Factor 38A | Protein Coding | 30 | GC01P052404 | 1.332606316 |
| CASQ2 | Calsequestrin 2 | Protein Coding | 40 | GC01M115700 | 1.331817746 |
| ELF2 | E74 Like ETS Transcription Factor 2 | Protein Coding | 36 | GC04M139028 | 1.331366181 |
| MAPK8IP2 | Mitogen-Activated Protein Kinase 8 Interacting Protein 2 | Protein Coding | 33 | GC22P050600 | 1.331282377 |
| MED22 | Mediator Complex Subunit 22 | Protein Coding | 32 | GC09M133338 | 1.331026077 |
| HSALNG0093253 | | RNA Gene | 4 | GC12M095614 | 1.331010342 |
| piR-48327-003 | | RNA Gene | 4 | GC12P095642 | 1.331010342 |
| ZFP1 | ZFP1 Zinc Finger Protein | Protein Coding | 32 | GC16P075132 | 1.330830812 |
| RBMX2 | RNA Binding Motif Protein X-Linked 2 | Protein Coding | 28 | GC0XP130401 | 1.330709934 |
| FAM184A | Family With Sequence Similarity 184 Member A | Protein Coding | 29 | GC06M118959 | 1.330554485 |
| SYNC | Syncoilin, Intermediate Filament Protein | Protein Coding | 30 | GC01M032679 | 1.329998016 |
| PPP1R16A | Protein Phosphatase 1 Regulatory Subunit 16A | Protein Coding | 30 | GC08P144477 | 1.329623938 |
| SYN1 | Synapsin I | Protein Coding | 40 | GC0XM047571 | 1.329333305 |
| LBH | LBH Regulator Of WNT Signaling Pathway | Protein Coding | 31 | GC02P030231 | 1.328995228 |
| PSMD8 | Proteasome 26S Subunit, Non-ATPase 8 | Protein Coding | 35 | GC19P038374 | 1.328935146 |
| LSM3 | LSM3 Homolog, U6 Small Nuclear RNA And MRNA Degradation Associated | Protein Coding | 34 | GC03P014180 | 1.328906894 |
| SLC24A5 | Solute Carrier Family 24 Member 5 | Protein Coding | 37 | GC15P048120 | 1.328873515 |

| | | | | | |
|---|---|---|---|---|---|
| PAPSS2 | 3'-Phosphoadenosine 5'-Phosphosulfate Synthase 2 | Protein Coding | 39 | GC10P087659 | 1.328814626 |
| YPEL4 | Yippee Like 4 | Protein Coding | 31 | GC11M057645 | 1.328635693 |
| SYNE3 | Spectrin Repeat Containing Nuclear Envelope Family Member 3 | Protein Coding | 31 | GC14M100462 | 1.328574419 |
| RPAP2 | RNA Polymerase II Associated Protein 2 | Protein Coding | 32 | GC01P092298 | 1.32839632 |
| PPP2R3A | Protein Phosphatase 2 Regulatory Subunit B"Alpha | Protein Coding | 33 | GC03P135965 | 1.328392506 |
| TRIM66 | Tripartite Motif Containing 66 | Protein Coding | 30 | GC11M008612 | 1.328309894 |
| MACO1 | Macoilin 1 | Protein Coding | 28 | GC01P025430 | 1.328044415 |
| KIR2DS1 | Killer Cell Immunoglobulin Like Receptor, Two Ig Domains And Short Cytoplasmic Tail 1 | Protein Coding | 22 | GC19Mr00063 | 1.3279984 |
| PALM | Paralemmin | Protein Coding | 31 | GC19P000708 | 1.327837229 |
| STRADB | STE20 Related Adaptor Beta | Protein Coding | 34 | GC02P201387 | 1.327797055 |
| HS3ST3A1 | Heparan Sulfate-Glucosamine 3-Sulfotransferase 3A1 | Protein Coding | 35 | GC17M013494 | 1.327463627 |
| RNF135 | Ring Finger Protein 135 | Protein Coding | 37 | GC17P052592 | 1.327455044 |
| MIR4262 | MicroRNA 4262 | RNA Gene | 11 | GC02M011836 | 1.32737422 |
| THUMPD1 | THUMP Domain Containing 1 | Protein Coding | 31 | GC16M020704 | 1.326977253 |
| NCBP2 | Nuclear Cap Binding Protein Subunit 2 | Protein Coding | 33 | GC03M196935 | 1.326818109 |
| RPPH1 | Ribonuclease P RNA Component H1 | RNA Gene | 18 | GC14M022126 | 1.326776743 |
| TARBP1 | TAR (HIV-1) RNA Binding Protein 1 | Protein Coding | 35 | GC01M234391 | 1.326533318 |
| HLA-DRB6 | Major Histocompatibility Complex, Class II, DR Beta 6 (Pseudogene) | Pseudogene | 15 | GC06M063654 | 1.3264395 |
| EEFSEC | Eukaryotic Elongation Factor, Selenocysteine-TRNA Specific | Protein Coding | 32 | GC03P128153 | 1.32640028 |
| ZRANB1 | Zinc Finger RANBP2-Type Containing 1 | Protein Coding | 32 | GC10P124941 | 1.326167464 |
| NADSYN1 | NAD Synthetase 1 | Protein Coding | 36 | GC11P071454 | 1.325584292 |
| ATP5PD | ATP Synthase Peripheral Stalk Subunit D | Protein Coding | 31 | GC17M075039 | 1.325426579 |
| ALDH6A1 | Aldehyde Dehydrogenase 6 Family Member A1 | Protein Coding | 41 | GC14M074059 | 1.325380564 |
| RAB21 | RAB21, Member RAS Oncogene Family | Protein Coding | 33 | GC12P071754 | 1.325364709 |
| CCSER1 | Coiled-Coil Serine Rich Protein 1 | Protein Coding | 27 | GC04P090127 | 1.325107336 |
| ARRDC1 | Arrestin Domain Containing 1 | Protein Coding | 30 | GC09P137605 | 1.325085521 |
| DBI | Diazepam Binding Inhibitor, Acyl-CoA Binding Protein | Protein Coding | 40 | GC02P119366 | 1.325080872 |
| NUP43 | Nucleoporin 43 | Protein Coding | 30 | GC06M149724 | 1.32506144 |
| PTPRM | Protein Tyrosine Phosphatase Receptor Type M | Protein Coding | 40 | GC18P007557 | 1.324671865 |
| SIGLEC8 | Sialic Acid Binding Ig Like Lectin 8 | Protein Coding | 36 | GC19M051450 | 1.324368596 |
| C5orf67 | Chromosome 5 Putative Open Reading Frame 67 | RNA Gene | 16 | GC05M056511 | 1.32434988 |
| ATXN2-AS | ATXN2 Antisense RNA | RNA Gene | 12 | GC12P111600 | 1.32434988 |
| HLA-K | Major Histocompatibility Complex, Class I, K (Pseudogene) | Pseudogene | 12 | GC06P080309 | 1.32434988 |
| HLA-W | Major Histocompatibility Complex, Class I, W (Pseudogene) | Pseudogene | 10 | GC06P080307 | 1.32434988 |
| ENSG00000266446 | Novel Transcript, Sense Intronic CDKN2B-AS1 | RNA Gene | 9 | GC09P021995 | 1.32434988 |
| LINC02571 | Long Intergenic Non-Protein Coding RNA 2571 | RNA Gene | 9 | GC06M063568 | 1.32434988 |
| RPS2P1 | Ribosomal Protein S2 Pseudogene 1 | Pseudogene | 6 | GC20P034122 | 1.32434988 |
| lnc-TBC1D3K-1 | | RNA Gene | 6 | GC17P052884 | 1.32434988 |
| ENSG00000237669 | HLA Complex Group 4 Pseudogene 3 | Pseudogene | 5 | GC06M064123 | 1.32434988 |
| HSALNG0039543 | | RNA Gene | 5 | GC05P001296 | 1.32434988 |
| NONHSAG045678.2-002 | | RNA Gene | 5 | GC17M037673 | 1.32434988 |
| RF00017-4261 | | RNA Gene | 5 | GC05P056584 | 1.32434988 |
| lnc-HNF1B-3 | | RNA Gene | 5 | GC17M037293 | 1.32434988 |
| lnc-ZFP36L1-9 | | RNA Gene | 5 | GC14M068397 | 1.32434988 |
| HG975427 | | RNA Gene | 4 | GC17M007670 | 1.32434988 |
| piR-46506-002 | | RNA Gene | 4 | GC05M001299 | 1.32434988 |
| PSME3IP1 | Proteasome Activator Subunit 3 Interacting Protein 1 | Protein Coding | 26 | GC16M057155 | 1.324265242 |
| NFIL3 | Nuclear Factor, Interleukin 3 Regulated | Protein Coding | 35 | GC09M091409 | 1.324254513 |
| STX18 | Syntaxin 18 | Protein Coding | 32 | GC04M004417 | 1.32409358 |
| ARSL | Arylsulfatase L | Protein Coding | 34 | GC0XM002934 | 1.323983908 |
| CDR2L | Cerebellar Degeneration Related Protein 2 Like | Protein Coding | 27 | GC17P074987 | 1.323060274 |
| PDZD8 | PDZ Domain Containing 8 | Protein Coding | 31 | GC10M117277 | 1.323039293 |
| ANP32B | Acidic Nuclear Phosphoprotein 32 Family Member B | Protein Coding | 34 | GC09P097983 | 1.322633505 |
| BRIX1 | Biogenesis Of Ribosomes BRX1 | Protein Coding | 31 | GC05P034916 | 1.322170734 |
| IFT20 | Intraflagellar Transport 20 | Protein Coding | 31 | GC17M034567 | 1.321773052 |
| GPR179 | G Protein-Coupled Receptor 179 | Protein Coding | 33 | GC17M041411 | 1.321484447 |
| QSER1 | Glutamine And Serine Rich 1 | Protein Coding | 28 | GC11P032892 | 1.320975065 |
| MCCC1 | Methylcrotonyl-CoA Carboxylase Subunit 1 | Protein Coding | 38 | GC03M183015 | 1.320364952 |
| STARD7 | StAR Related Lipid Transfer Domain Containing 7 | Protein Coding | 33 | GC02M096184 | 1.320233345 |
| PAQR4 | Progestin And AdipoQ Receptor Family Member 4 | Protein Coding | 30 | GC16P002969 | 1.320128798 |
| FXYD2 | FXYD Domain Containing Ion Transport Regulator 2 | Protein Coding | 38 | GC11M117800 | 1.318987846 |
| GTF3C3 | General Transcription Factor IIIC Subunit 3 | Protein Coding | 33 | GC02M196763 | 1.318778515 |
| ACTL7A | Actin Like 7A | Protein Coding | 31 | GC09P108862 | 1.318589926 |
| WDR81 | WD Repeat Domain 81 | Protein Coding | 33 | GC17P001716 | 1.318356276 |
| ANKRD16 | Ankyrin Repeat Domain 16 | Protein Coding | 27 | GC10M005861 | 1.318012118 |
| SNAP29 | Synaptosome Associated Protein 29 | Protein Coding | 38 | GC22P020859 | 1.31760788 |
| IQSEC2 | IQ Motif And Sec7 Domain ArfGEF 2 | Protein Coding | 35 | GC0XM053225 | 1.317156315 |
| TBC1D12 | TBC1 Domain Family Member 12 | Protein Coding | 28 | GC10P094402 | 1.316953063 |
| RSPRY1 | Ring Finger And SPRY Domain Containing 1 | Protein Coding | 33 | GC16P057187 | 1.316083193 |
| ZNF827 | Zinc Finger Protein 827 | Protein Coding | 28 | GC04M145757 | 1.315998077 |
| ZNF222 | Zinc Finger Protein 222 | Protein Coding | 27 | GC19P063795 | 1.315888405 |
| CCNJ | Cyclin J | Protein Coding | 29 | GC10P096043 | 1.315865278 |
| SENP7 | SUMO Specific Peptidase 7 | Protein Coding | 33 | GC03M101324 | 1.315841436 |
| KRT23 | Keratin 23 | Protein Coding | 32 | GC17M040922 | 1.315763593 |
| ZBTB4 | Zinc Finger And BTB Domain Containing 4 | Protein Coding | 31 | GC17M007459 | 1.315645695 |
| CAMSAP3 | Calmodulin Regulated Spectrin Associated Protein Family Member 3 | Protein Coding | 27 | GC19P007595 | 1.315487504 |
| VWCE | Von Willebrand Factor C And EGF Domains | Protein Coding | 28 | GC11M061258 | 1.315289021 |
| RGS7 | Regulator Of G Protein Signaling 7 | Protein Coding | 36 | GC01M240767 | 1.314968586 |
| FBXL17 | F-Box And Leucine Rich Repeat Protein 17 | Protein Coding | 32 | GC05M107859 | 1.314683795 |
| ADAMTS7P3 | ADAMTS7 Pseudogene 3 | Pseudogene | 7 | GC15P077976 | 1.314606428 |
| FUNDC1 | FUN14 Domain Containing 1 | Protein Coding | 30 | GC0XM044523 | 1.31433773 |
| ZNF609 | Zinc Finger Protein 609 | Protein Coding | 28 | GC15P117507 | 1.314056635 |
| NYAP1 | Neuronal Tyrosine Phosphorylated Phosphoinositide-3-Kinase Adaptor 1 | Protein Coding | 27 | GC07P100483 | 1.313766599 |
| GPM6B | Glycoprotein M6B | Protein Coding | 34 | GC0XM013789 | 1.313551426 |
| MIR365B | MicroRNA 365b | RNA Gene | 14 | GC17P052617 | 1.313490629 |
| DCP1A | Decapping MRNA 1A | Protein Coding | 33 | GC03M053301 | 1.313006639 |
| MIR329-2 | MicroRNA 329-2 | RNA Gene | 13 | GC14P109520 | 1.312949419 |
| MIR4513 | MicroRNA 4513 | RNA Gene | 14 | GC15M074788 | 1.312723041 |
| CARD6 | Caspase Recruitment Domain Family Member 6 | Protein Coding | 34 | GC05P040841 | 1.312384248 |
| HMG20B | High Mobility Group 20B | Protein Coding | 31 | GC19P003572 | 1.312260747 |
| SH3BGRL3 | SH3 Domain Binding Glutamate Rich Protein Like 3 | Protein Coding | 31 | GC01P026289 | 1.311881661 |
| GXYLT1 | Glucoside Xylosyltransferase 1 | Protein Coding | 32 | GC12M042081 | 1.311769247 |
| LINC02143 | Long Intergenic Non-Protein Coding RNA 2143 | RNA Gene | 9 | GC05M164449 | 1.310682178 |
| SLC27A3 | Solute Carrier Family 27 Member 3 | Protein Coding | 35 | GC01P153869 | 1.310488105 |

| | | | | | |
|---|---|---|---|---|---|
| MRPL19 | Mitochondrial Ribosomal Protein L19 | Protein Coding | 32 | GC02P075646 | 1.310361385 |
| MRPL35 | Mitochondrial Ribosomal Protein L35 | Protein Coding | 31 | GC02P086199 | 1.310094476 |
| HHATL | Hedgehog Acyltransferase Like | Protein Coding | 31 | GC03M042698 | 1.309837103 |
| GIPC2 | GIPC PDZ Domain Containing Family Member 2 | Protein Coding | 32 | GC01P077987 | 1.309813023 |
| RHOXF1 | Rhox Homeobox Family Member 1 | Protein Coding | 29 | GC0XM120109 | 1.309561729 |
| RTF2 | Replication Termination Factor 2 | Protein Coding | 25 | GC20P056469 | 1.309421539 |
| DCLK2 | Doublecortin Like Kinase 2 | Protein Coding | 36 | GC04P150078 | 1.30937314 |
| OPN1MW | Opsin 1, Medium Wave Sensitive | Protein Coding | 32 | GC0XP154182 | 1.309110999 |
| CHMP1B | Charged Multivesicular Body Protein 1B | Protein Coding | 34 | GC18P011851 | 1.308849335 |
| RBM47 | RNA Binding Motif Protein 47 | Protein Coding | 31 | GC04M040425 | 1.30861187 |
| EHBP1L1 | EH Domain Binding Protein 1 Like 1 | Protein Coding | 30 | GC11P069550 | 1.308579445 |
| SPC25 | SPC25 Component Of NDC80 Kinetochore Complex | Protein Coding | 29 | GC02M168834 | 1.308458209 |
| RBBP8NL | RBBP8 N-Terminal Like | Protein Coding | 25 | GC20M062410 | 1.308403969 |
| CFAP58 | Cilia And Flagella Associated Protein 58 | Protein Coding | 27 | GC10P104354 | 1.30809319 |
| TMEM98 | Transmembrane Protein 98 | Protein Coding | 34 | GC17P032927 | 1.307694912 |
| MIPEP | Mitochondrial Intermediate Peptidase | Protein Coding | 39 | GC13M023730 | 1.307449341 |
| KIRREL1 | Kirre Like Nephrin Family Adhesion Molecule 1 | Protein Coding | 31 | GC01P157994 | 1.307226419 |
| RBMXL1 | RBMX Like 1 | Protein Coding | 26 | GC01M088979 | 1.307133913 |
| CDK5R1 | Cyclin Dependent Kinase 5 Regulatory Subunit 1 | Protein Coding | 40 | GC17P052662 | 1.30692625 |
| TM9SF1 | Transmembrane 9 Superfamily Member 1 | Protein Coding | 30 | GC14M024189 | 1.306647897 |
| KCNS3 | Potassium Voltage-Gated Channel Modifier Subfamily S Member 3 | Protein Coding | 36 | GC02P017877 | 1.306298733 |
| GABARAPL3 | GABA Type A Receptor Associated Protein Like 3 Pseudogene | Pseudogene | 19 | GC15M090346 | 1.306139946 |
| MAGEB16 | MAGE Family Member B16 | Protein Coding | 25 | GC0XP035816 | 1.305855274 |
| ATP9A | ATPase Phospholipid Transporting 9A (Putative) | Protein Coding | 34 | GC20M051596 | 1.305599689 |
| GK2 | Glycerol Kinase 2 | Protein Coding | 36 | GC04M079406 | 1.305577517 |
| CC2D1A | Coiled-Coil And C2 Domain Containing 1A | Protein Coding | 36 | GC19P014146 | 1.305255175 |
| PLD6 | Phospholipase D Family Member 6 | Protein Coding | 30 | GC17M017287 | 1.304793239 |
| HOXB3 | Homeobox B3 | Protein Coding | 36 | GC17M048548 | 1.304581761 |
| ANO10 | Anoctamin 10 | Protein Coding | 36 | GC03M043355 | 1.303962231 |
| RRAGA | Ras Related GTP Binding A | Protein Coding | 34 | GC09P019049 | 1.303769827 |
| SCAF11 | SR-Related CTD Associated Factor 11 | Protein Coding | 31 | GC12M045919 | 1.303374529 |
| LINC00461 | Long Intergenic Non-Protein Coding RNA 461 | RNA Gene | 21 | GC05M088507 | 1.303109169 |
| ACSS2 | Acyl-CoA Synthetase Short Chain Family Member 2 | Protein Coding | 40 | GC20P034873 | 1.302966714 |
| RHOU | Ras Homolog Family Member U | Protein Coding | 33 | GC01P228644 | 1.302419305 |
| FKBP15 | FKBP Prolyl Isomerase Family Member 15 | Protein Coding | 31 | GC09M113161 | 1.301890492 |
| CCDC86 | Coiled-Coil Domain Containing 86 | Protein Coding | 30 | GC11P060974 | 1.301191807 |
| TMTC3 | Transmembrane O-Mannosyltransferase Targeting Cadherins 3 | Protein Coding | 32 | GC12P088142 | 1.301169276 |
| TMEM184C | Transmembrane Protein 184C | Protein Coding | 31 | GC04P147617 | 1.301100254 |
| NGDN | Neuroguidin | Protein Coding | 28 | GC14P023469 | 1.30087471 |
| NOC3L | NOC3 Like DNA Replication Regulator | Protein Coding | 31 | GC10M094333 | 1.30039525 |
| SLC41A1 | Solute Carrier Family 41 Member 1 | Protein Coding | 36 | GC01M205789 | 1.300358891 |
| CYP26C1 | Cytochrome P450 Family 26 Subfamily C Member 1 | Protein Coding | 39 | GC10P093060 | 1.300328732 |
| WIPF2 | WAS/WASL Interacting Protein Family Member 2 | Protein Coding | 32 | GC17P040219 | 1.300237894 |
| DNAJC15 | DnaJ Heat Shock Protein Family (Hsp40) Member C15 | Protein Coding | 32 | GC13P043023 | 1.300109386 |
| BPIFA3 | BPI Fold Containing Family A Member 3 | Protein Coding | 26 | GC20P033217 | 1.299796939 |
| H2BC18 | H2B Clustered Histone 18 | Protein Coding | 26 | GC01M151576 | 1.299445748 |
| SLC25A52 | Solute Carrier Family 25 Member 52 | Protein Coding | 26 | GC18M031759 | 1.299434662 |
| MSL1 | MSL Complex Subunit 1 | Protein Coding | 30 | GC17P053049 | 1.299319148 |
| GAK | Cyclin G Associated Kinase | Protein Coding | 41 | GC04M000849 | 1.299157858 |
| DUOXA1 | Dual Oxidase Maturation Factor 1 | Protein Coding | 32 | GC15M045119 | 1.299144983 |
| SLCO3A1 | Solute Carrier Organic Anion Transporter Family Member 3A1 | Protein Coding | 36 | GC15P115336 | 1.298677325 |
| MSRB3 | Methionine Sulfoxide Reductase B3 | Protein Coding | 39 | GC12P065279 | 1.298405886 |
| IFIT1 | Interferon Induced Protein With Tetratricopeptide Repeats 1 | Protein Coding | 34 | GC10P091596 | 1.298294783 |
| ASRGL1 | Asparaginase And Isoaspartyl Peptidase 1 | Protein Coding | 37 | GC11P062337 | 1.298247099 |
| SYNGR2 | Synaptogyrin 2 | Protein Coding | 34 | GC17P078168 | 1.29793644 |
| WDR61 | WD Repeat Domain 61 | Protein Coding | 34 | GC15M078277 | 1.297734261 |
| TADA2B | Transcriptional Adaptor 2B | Protein Coding | 30 | GC04P007237 | 1.297703385 |
| TTBK1 | Tau Tubulin Kinase 1 | Protein Coding | 33 | GC06P043243 | 1.296967983 |
| C16orf72 | Chromosome 16 Open Reading Frame 72 | Protein Coding | 30 | GC16P011003 | 1.296769261 |
| DEFA3 | Defensin Alpha 3 | Protein Coding | 33 | GC08M007015 | 1.29647851 |
| RTF1 | RTF1 Homolog, Paf1/RNA Polymerase II Complex Component | Protein Coding | 31 | GC15P041813 | 1.29643786 |
| ZBTB14 | Zinc Finger And BTB Domain Containing 14 | Protein Coding | 30 | GC18M005289 | 1.295846939 |
| CACNA2D4 | Calcium Voltage-Gated Channel Auxiliary Subunit Alpha2delta 4 | Protein Coding | 38 | GC12M001791 | 1.295709252 |
| SLC27A1 | Solute Carrier Family 27 Member 1 | Protein Coding | 36 | GC19P063107 | 1.295272589 |
| MED30 | Mediator Complex Subunit 30 | Protein Coding | 33 | GC08P117521 | 1.295106411 |
| HECTD1 | HECT Domain E3 Ubiquitin Protein Ligase 1 | Protein Coding | 34 | GC14M031100 | 1.294783473 |
| NDRG3 | NDRG Family Member 3 | Protein Coding | 33 | GC20M036651 | 1.294743776 |
| GTF2F2 | General Transcription Factor IIF Subunit 2 | Protein Coding | 37 | GC13P045120 | 1.294725895 |
| HAP1 | Huntingtin Associated Protein 1 | Protein Coding | 35 | GC17M041717 | 1.29427886 |
| CPB1 | Carboxypeptidase B1 | Protein Coding | 38 | GC03P148791 | 1.294004917 |
| CAAP1 | Caspase Activity And Apoptosis Inhibitor 1 | Protein Coding | 26 | GC09M026840 | 1.293851614 |
| SUCNR1 | Succinate Receptor 1 | Protein Coding | 35 | GC03P151873 | 1.293652534 |
| SIGLEC15 | Sialic Acid Binding Ig Like Lectin 15 | Protein Coding | 31 | GC18P045825 | 1.292547345 |
| CTSF | Cathepsin F | Protein Coding | 44 | GC11M087348 | 1.291971684 |
| DCAF16 | DDB1 And CUL4 Associated Factor 16 | Protein Coding | 28 | GC04M017794 | 1.291603804 |
| ATP11B | ATPase Phospholipid Transporting 11B (Putative) | Protein Coding | 36 | GC03P182793 | 1.291475534 |
| COPRS | Coordinator Of PRMT5 And Differentiation Stimulator | Protein Coding | 28 | GC17M031851 | 1.291182756 |
| DAZ1 | Deleted In Azoospermia 1 | Protein Coding | 29 | GC0YM023129 | 1.291078568 |
| PPP3CB | Protein Phosphatase 3 Catalytic Subunit Beta | Protein Coding | 40 | GC10M073436 | 1.290905476 |
| ITM2B | Integral Membrane Protein 2B | Protein Coding | 40 | GC13P048233 | 1.290669203 |
| CNIH4 | Cornichon Family AMPA Receptor Auxiliary Protein 4 | Protein Coding | 31 | GC01P224356 | 1.290345073 |
| LANCL2 | LanC Like 2 | Protein Coding | 32 | GC07P055365 | 1.289952755 |
| MCTP1 | Multiple C2 And Transmembrane Domain Containing 1 | Protein Coding | 32 | GC05M094703 | 1.289911509 |
| SLC30A6 | Solute Carrier Family 30 Member 6 | Protein Coding | 34 | GC02P032166 | 1.289887786 |
| ZC3H15 | Zinc Finger CCCH-Type Containing 15 | Protein Coding | 29 | GC02P186486 | 1.289887786 |
| CMTM3 | CKLF Like MARVEL Transmembrane Domain Containing 3 | Protein Coding | 31 | GC16P066716 | 1.289821625 |
| SNX15 | Sorting Nexin 15 | Protein Coding | 32 | GC11P065031 | 1.289448977 |
| MT-TC | Mitochondrially Encoded TRNA-Cys (UGU/C) | RNA Gene | 10 | GCMTM005763 | 1.28935647 |
| DENND2B | DENN Domain Containing 2B | Protein Coding | 28 | GC11M008739 | 1.289039135 |
| ARPC5 | Actin Related Protein 2/3 Complex Subunit 5 | Protein Coding | 34 | GC01M183621 | 1.288990617 |
| COL27A1 | Collagen Type XXVII Alpha 1 Chain | Protein Coding | 38 | GC09P118616 | 1.288349152 |
| PIH1D1 | PIH1 Domain Containing 1 | Protein Coding | 32 | GC19M064112 | 1.288036585 |
| PSRC1 | Proline And Serine Rich Coiled-Coil 1 | Protein Coding | 34 | GC01M109279 | 1.287651539 |
| SKIDA1 | SKI/DACH Domain Containing 1 | Protein Coding | 24 | GC10M021513 | 1.28761363 |
| CD180 | CD180 Molecule | Protein Coding | 34 | GC05M067181 | 1.28697145 |

| | | | | | |
|---|---|---|---|---|---|
| SMPDL3B | Sphingomyelin Phosphodiesterase Acid Like 3B | Protein Coding | 33 | GC01P028178 | 1.286860228 |
| DSCAM | DS Cell Adhesion Molecule | Protein Coding | 38 | GC21M040010 | 1.286799073 |
| MCF2 | MCF.2 Cell Line Derived Transforming Sequence | Protein Coding | 36 | GC0XM139581 | 1.286750078 |
| C12orf75 | Chromosome 12 Open Reading Frame 75 | Protein Coding | 26 | GC12P105235 | 1.286556005 |
| IFI35 | Interferon Induced Protein 35 | Protein Coding | 34 | GC17P043006 | 1.286491394 |
| ENTPD7 | Ectonucleoside Triphosphate Diphosphohydrolase 7 | Protein Coding | 34 | GC10P099659 | 1.286435843 |
| TMCO5A | Transmembrane And Coiled-Coil Domains 5A | Protein Coding | 31 | GC15P037921 | 1.286435843 |
| MKLN1-AS | MKLN1 Antisense RNA | RNA Gene | 11 | GC07M131309 | 1.286200762 |
| GPR85 | G Protein-Coupled Receptor 85 | Protein Coding | 34 | GC07M113078 | 1.286048651 |
| ELOVL2 | ELOVL Fatty Acid Elongase 2 | Protein Coding | 35 | GC06M010980 | 1.285958409 |
| BTF3L4 | Basic Transcription Factor 3 Like 4 | Protein Coding | 31 | GC01P052056 | 1.285756588 |
| FAM162A | Family With Sequence Similarity 162 Member A | Protein Coding | 31 | GC03P122384 | 1.285416722 |
| ERMP1 | Endoplasmic Reticulum Metallopeptidase 1 | Protein Coding | 32 | GC09M005749 | 1.285270452 |
| POLR3D | RNA Polymerase III Subunit D | Protein Coding | 33 | GC08P022245 | 1.28521359 |
| NME6 | NME/NM23 Nucleoside Diphosphate Kinase 6 | Protein Coding | 35 | GC03M048292 | 1.284972668 |
| C6orf47 | Chromosome 6 Open Reading Frame 47 | Protein Coding | 26 | GC06M063610 | 1.284862518 |
| RPL23AP1 | Ribosomal Protein L23a Pseudogene 1 | Pseudogene | 9 | GC06M063487 | 1.284862518 |
| CARD8 | Caspase Recruitment Domain Family Member 8 | Protein Coding | 36 | GC19M064051 | 1.284796119 |
| DAND5 | DAN Domain BMP Antagonist Family Member 5 | Protein Coding | 34 | GC19P012965 | 1.284084916 |
| RNF180 | Ring Finger Protein 180 | Protein Coding | 29 | GC05P064165 | 1.283557415 |
| CFDP1 | Craniofacial Development Protein 1 | Protein Coding | 33 | GC16M075294 | 1.283386469 |
| INTS8 | Integrator Complex Subunit 8 | Protein Coding | 34 | GC08P094813 | 1.283218265 |
| TERLR1 | TERT Regulating LncRNA 1 | RNA Gene | 12 | GC05M001168 | 1.282519698 |
| PPP2R2C | Protein Phosphatase 2 Regulatory Subunit Bgamma | Protein Coding | 38 | GC04M006322 | 1.282378554 |
| MDGA1 | MAM Domain Containing Glycosylphosphatidylinositol Anchor 1 | Protein Coding | 34 | GC06M063767 | 1.281751871 |
| PNLIP | Pancreatic Lipase | Protein Coding | 44 | GC10P116545 | 1.281431198 |
| CMTM5 | CKLF Like MARVEL Transmembrane Domain Containing 5 | Protein Coding | 32 | GC14P031745 | 1.281257391 |
| PFDN2 | Prefoldin Subunit 2 | Protein Coding | 31 | GC01M161100 | 1.281257391 |
| TXNL4A | Thioredoxin Like 4A | Protein Coding | 37 | GC18M079970 | 1.281175137 |
| AAAS | Aladin WD Repeat Nucleoporin | Protein Coding | 37 | GC12M053307 | 1.280915022 |
| CDH12 | Cadherin 12 | Protein Coding | 38 | GC05M021786 | 1.280739188 |
| SNORD138 | Small Nucleolar RNA, C/D Box 138 | RNA Gene | 12 | GC05P086703 | 1.280700684 |
| TENT2 | Terminal Nucleotidyltransferase 2 | Protein Coding | 28 | GC05P079613 | 1.280589223 |
| DCAF13 | DDB1 And CUL4 Associated Factor 13 | Protein Coding | 31 | GC08P103414 | 1.280498028 |
| ENSG00000269307 | Novel Transcript | Protein Coding | 5 | GC19P063102 | 1.280024648 |
| MCMBP | Minichromosome Maintenance Complex Binding Protein | Protein Coding | 27 | GC10M119829 | 1.279690504 |
| LINC00161 | Long Intergenic Non-Protein Coding RNA 161 | RNA Gene | 17 | GC21P028539 | 1.279553175 |
| PDCD11 | Programmed Cell Death 11 | Protein Coding | 30 | GC10P103396 | 1.278935671 |
| MRPL20-DT | MRPL20 Divergent Transcript | RNA Gene | 10 | GC01P003812 | 1.278618932 |
| ABRACL | ABRA C-Terminal Like | Protein Coding | 27 | GC06P139028 | 1.277962208 |
| NEB | Nebulin | Protein Coding | 36 | GC02M151485 | 1.277774692 |
| LMAN2L | Lectin, Mannose Binding 2 Like | Protein Coding | 34 | GC02M098025 | 1.277696013 |
| TUT7 | Terminal Uridylyl Transferase 7 | Protein Coding | 29 | GC09M092523 | 1.277653694 |
| SCARA3 | Scavenger Receptor Class A Member 3 | Protein Coding | 33 | GC08P027633 | 1.277570486 |
| SLC2A6 | Solute Carrier Family 2 Member 6 | Protein Coding | 37 | GC09M133471 | 1.277400732 |
| EXOC1 | Exocyst Complex Component 1 | Protein Coding | 31 | GC04P055853 | 1.277132034 |
| NCAPH2 | Non-SMC Condensin II Complex Subunit H2 | Protein Coding | 34 | GC22P050508 | 1.277038932 |
| ITIH1 | Inter-Alpha-Trypsin Inhibitor Heavy Chain 1 | Protein Coding | 37 | GC03P052777 | 1.276925445 |
| COLGALT1 | Collagen Beta(1-O)Galactosyltransferase 1 | Protein Coding | 34 | GC19P017555 | 1.275597334 |
| CHMP3 | Charged Multivesicular Body Protein 3 | Protein Coding | 32 | GC02M086505 | 1.275373578 |
| NUDT16L1 | Nudix Hydrolase 16 Like 1 | Protein Coding | 31 | GC16P010883 | 1.275281549 |
| SCN7A | Sodium Voltage-Gated Channel Alpha Subunit 7 | Protein Coding | 37 | GC02M166403 | 1.274587989 |
| CGB1 | Chorionic Gonadotropin Subunit Beta 1 | Protein Coding | 25 | GC19M049035 | 1.274450183 |
| ARPIN | Actin Related Protein 2/3 Complex Inhibitor | Protein Coding | 25 | GC15M089895 | 1.274105072 |
| PPP3CC | Protein Phosphatase 3 Catalytic Subunit Gamma | Protein Coding | 39 | GC08P022440 | 1.273965359 |
| LRP10 | LDL Receptor Related Protein 10 | Protein Coding | 34 | GC14P022871 | 1.273959756 |
| UTP15 | UTP15 Small Subunit Processome Component | Protein Coding | 30 | GC05P073565 | 1.273896217 |
| SLC6A6 | Solute Carrier Family 6 Member 6 | Protein Coding | 40 | GC03P014402 | 1.273769736 |
| NPLOC4 | NPL4 Homolog, Ubiquitin Recognition Factor | Protein Coding | 35 | GC17M081556 | 1.273705125 |
| PHACTR4 | Phosphatase And Actin Regulator 4 | Protein Coding | 28 | GC01P028369 | 1.273331404 |
| NMD3 | NMD3 Ribosome Export Adaptor | Protein Coding | 31 | GC03P161104 | 1.273063064 |
| MTMR11 | Myotubularin Related Protein 11 | Protein Coding | 29 | GC01M149928 | 1.272973776 |
| POP7 | POP7 Homolog, Ribonuclease P/MRP Subunit | Protein Coding | 30 | GC07P100706 | 1.272898555 |
| EXOC5 | Exocyst Complex Component 5 | Protein Coding | 36 | GC14M057200 | 1.272665739 |
| TMEM218 | Transmembrane Protein 218 | Protein Coding | 30 | GC11M125094 | 1.272488952 |
| CACTIN | Cactin, Spliceosome C Complex Subunit | Protein Coding | 26 | GC19M003610 | 1.271717548 |
| ATP8A1 | ATPase Phospholipid Transporting 8A1 | Protein Coding | 38 | GC04M042410 | 1.271456718 |
| ELOF1 | Elongation Factor 1 | Protein Coding | 31 | GC19M011551 | 1.271260977 |
| ARMC10 | Armadillo Repeat Containing 10 | Protein Coding | 30 | GC07P103074 | 1.271014214 |
| MSBP2 | Minisatellite Binding Protein 2 | Protein Coding | 5 | GC00U990214 | 1.270613194 |
| MIR1260A | MicroRNA 1260a | RNA Gene | 14 | GC14P077266 | 1.270027637 |
| TRMT61B | TRNA Methyltransferase 61B | Protein Coding | 30 | GC02M028849 | 1.269773006 |
| AFG1L | AFG1 Like ATPase | Protein Coding | 27 | GC06P108482 | 1.269630671 |
| JPH4 | Junctophilin 4 | Protein Coding | 31 | GC14M023568 | 1.269600272 |
| CSTPP1 | Centriolar Satellite-Associated Tubulin Polyglutamylase Complex Regulator 1 | Protein Coding | 25 | GC11P046938 | 1.269253492 |
| CIAO3 | Cytosolic Iron-Sulfur Assembly Component 3 | Protein Coding | 27 | GC16M006936 | 1.269139767 |
| RAB26 | RAB26, Member RAS Oncogene Family | Protein Coding | 34 | GC16P010717 | 1.269033074 |
| H1-6 | H1.6 Linker Histone, Cluster Member | Protein Coding | 29 | GC06M064153 | 1.268783092 |
| STARD3NL | STARD3 N-Terminal Like | Protein Coding | 33 | GC07P038218 | 1.268196583 |
| LDHD | Lactate Dehydrogenase D | Protein Coding | 36 | GC16M075111 | 1.26819253 |
| HAPLN3 | Hyaluronan And Proteoglycan Link Protein 3 | Protein Coding | 34 | GC15M088877 | 1.267995834 |
| SLX4IP | SLX4 Interacting Protein | Protein Coding | 24 | GC20P010435 | 1.267755747 |
| GFRA3 | GDNF Family Receptor Alpha 3 | Protein Coding | 38 | GC05M138263 | 1.26760745 |
| ZCCHC9 | Zinc Finger CCHC-Type Containing 9 | Protein Coding | 27 | GC05P081301 | 1.267496467 |
| LOC101929777 | Uncharacterized LOC101929777 | RNA Gene | 3 | GC17M046830 | 1.26739502 |
| KYAT3 | Kynurenine Aminotransferase 3 | Protein Coding | 30 | GC01M088921 | 1.267272711 |
| KDELR2 | KDEL Endoplasmic Reticulum Protein Retention Receptor 2 | Protein Coding | 36 | GC07M006447 | 1.267230272 |
| SAMD3 | Sterile Alpha Motif Domain Containing 3 | Protein Coding | 30 | GC06M130144 | 1.267222881 |
| ZNF227 | Zinc Finger Protein 227 | Protein Coding | 28 | GC19P044207 | 1.267222881 |
| AHCYL1 | Adenosylhomocysteinase Like 1 | Protein Coding | 39 | GC01P109984 | 1.267163754 |
| TM6SF2 | Transmembrane 6 Superfamily Member 2 | Protein Coding | 27 | GC19M019264 | 1.267118216 |
| BDH1 | 3-Hydroxybutyrate Dehydrogenase 1 | Protein Coding | 38 | GC03M197519 | 1.267107964 |
| KLHL8 | Kelch Like Family Member 8 | Protein Coding | 34 | GC04M087160 | 1.267094612 |
| TMEM201 | Transmembrane Protein 201 | Protein Coding | 29 | GC01P009588 | 1.267080307 |
| MIR4319 | MicroRNA 4319 | RNA Gene | 10 | GC18M044970 | 1.267050028 |

| | | | | | |
|---|---|---|---|---|---|
| ESX1 | ESX Homeobox 1 | Protein Coding | 31 | GC0XM104250 | 1.266736746 |
| SNRNP40 | Small Nuclear Ribonucleoprotein U5 Subunit 40 | Protein Coding | 31 | GC01M031259 | 1.266472936 |
| SNRPGP15 | Small Nuclear Ribonucleoprotein Polypeptide G Pseudogene 15 | Pseudogene | 13 | GC19P014489 | 1.266461849 |
| EXOC6 | Exocyst Complex Component 6 | Protein Coding | 34 | GC10P092826 | 1.265831947 |
| PCK2 | Phosphoenolpyruvate Carboxykinase 2, Mitochondrial | Protein Coding | 42 | GC14P024094 | 1.265726805 |
| PAXBP1 | PAX3 And PAX7 Binding Protein 1 | Protein Coding | 30 | GC21M032734 | 1.265461922 |
| NLRP12 | NLR Family Pyrin Domain Containing 12 | Protein Coding | 39 | GC19M053793 | 1.265416026 |
| SLC46A3 | Solute Carrier Family 46 Member 3 | Protein Coding | 32 | GC13M028700 | 1.265078783 |
| MIR1269A | MicroRNA 1269a | RNA Gene | 13 | GC04P066276 | 1.265033484 |
| HOXD1 | Homeobox D1 | Protein Coding | 34 | GC02P176238 | 1.26502037 |
| RPUSD3 | RNA Pseudouridine Synthase D3 | Protein Coding | 31 | GC03M009839 | 1.264117002 |
| GORASP2 | Golgi Reassembly Stacking Protein 2 | Protein Coding | 35 | GC02P170928 | 1.263911247 |
| RETNLB | Resistin Like Beta | Protein Coding | 32 | GC03M108743 | 1.262884736 |
| MIR1299 | MicroRNA 1299 | RNA Gene | 13 | GC09M040929 | 1.262660742 |
| SGMS2 | Sphingomyelin Synthase 2 | Protein Coding | 35 | GC04P107824 | 1.262543917 |
| ZNF202 | Zinc Finger Protein 202 | Protein Coding | 35 | GC11M123724 | 1.262315273 |
| C8orf33 | Chromosome 8 Open Reading Frame 33 | Protein Coding | 26 | GC08P145052 | 1.261867762 |
| TGM7 | Transglutaminase 7 | Protein Coding | 31 | GC15M043276 | 1.261599183 |
| MIR2355 | MicroRNA 2355 | RNA Gene | 17 | GC02M207109 | 1.26152277 |
| CNNM1 | Cyclin And CBS Domain Divalent Metal Cation Transport Mediator 1 | Protein Coding | 32 | GC10P099330 | 1.261257648 |
| NMUR1 | Neuromedin U Receptor 1 | Protein Coding | 38 | GC02M231886 | 1.260783672 |
| IFN1@ | Interferon, Type 1, Cluster | Gene Cluster | 3 | GC09U990039 | 1.260541916 |
| DXO | Decapping Exoribonuclease | Protein Coding | 30 | GC06M031969 | 1.260508299 |
| NPM3 | Nucleophosmin/Nucleoplasmin 3 | Protein Coding | 32 | GC10M101781 | 1.260478973 |
| KRT77 | Keratin 77 | Protein Coding | 32 | GC12M052689 | 1.259881973 |
| TRIM4 | Tripartite Motif Containing 4 | Protein Coding | 30 | GC07M099876 | 1.25986433 |
| MIR4516 | MicroRNA 4516 | RNA Gene | 14 | GC16P010718 | 1.259519219 |
| B3GAT3 | Beta-1,3-Glucuronyltransferase 3 | Protein Coding | 39 | GC11M087127 | 1.259013891 |
| IFNLR1 | Interferon Lambda Receptor 1 | Protein Coding | 34 | GC01M025501 | 1.258439541 |
| MYH4 | Myosin Heavy Chain 4 | Protein Coding | 35 | GC17M010443 | 1.258242488 |
| B3GNTL1 | UDP-GlcNAc:BetaGal Beta-1,3-N-Acetylglucosaminyltransferase Like 1 | Protein Coding | 30 | GC17M082942 | 1.258177638 |
| PDE10A | Phosphodiesterase 10A | Protein Coding | 42 | GC06M165327 | 1.257752657 |
| DBF4B | DBF4 Zinc Finger B | Protein Coding | 29 | GC17P044708 | 1.257736683 |
| RFX4 | Regulatory Factor X4 | Protein Coding | 31 | GC12P106583 | 1.257686138 |
| MIR593 | MicroRNA 593 | RNA Gene | 14 | GC07P128081 | 1.257429361 |
| STRN4 | Striatin 4 | Protein Coding | 34 | GC19M046719 | 1.257323742 |
| DALRD3 | DALR Anticodon Binding Domain Containing 3 | Protein Coding | 31 | GC03M049015 | 1.25707221 |
| SGPP1 | Sphingosine-1-Phosphate Phosphatase 1 | Protein Coding | 34 | GC14M063684 | 1.257060289 |
| FBXO7 | F-Box Protein 7 | Protein Coding | 39 | GC22P032474 | 1.257028103 |
| MIR769 | MicroRNA 769 | RNA Gene | 15 | GC19P046018 | 1.256936312 |
| MAP3K21 | Mitogen-Activated Protein Kinase Kinase Kinase 21 | Protein Coding | 27 | GC01P233329 | 1.256830454 |
| PGRMC2 | Progesterone Receptor Membrane Component 2 | Protein Coding | 35 | GC04M128269 | 1.256439447 |
| LDLRAD4 | Low Density Lipoprotein Receptor Class A Domain Containing 4 | Protein Coding | 28 | GC18P013217 | 1.256359816 |
| TMA16 | Translation Machinery Associated 16 Homolog | Protein Coding | 25 | GC04P163494 | 1.25628233 |
| RILP | Rab Interacting Lysosomal Protein | Protein Coding | 31 | GC17M001646 | 1.256267071 |
| SNAP25 | Synaptosome Associated Protein 25 | Protein Coding | 44 | GC20P010184 | 1.256099701 |
| ERVK-18 | Endogenous Retrovirus Group K Member 18 | Protein Coding | 6 | GC01U903332 | 1.255704403 |
| MIR589 | MicroRNA 589 | RNA Gene | 17 | GC07M005495 | 1.255297899 |
| EID3 | EP300 Interacting Inhibitor Of Differentiation 3 | Protein Coding | 27 | GC12P104303 | 1.255263209 |
| LOC109113863 | FGF2 Promoter Region | Biological Region | 2 | GC04P122916 | 1.255196333 |
| TNFSF18 | TNF Superfamily Member 18 | Protein Coding | 36 | GC01M173009 | 1.254807711 |
| ENSA | Endosulfine Alpha | Protein Coding | 36 | GC01M151638 | 1.254593015 |
| CRIP2 | Cysteine Rich Protein 2 | Protein Coding | 32 | GC14P105472 | 1.254425526 |
| HMOX2 | Heme Oxygenase 2 | Protein Coding | 42 | GC16P004474 | 1.254251838 |
| THEM4 | Thioesterase Superfamily Member 4 | Protein Coding | 35 | GC01M151870 | 1.254246116 |
| INGX | Inhibitor Of Growth Family, X-Linked (Pseudogene) | Pseudogene | 12 | GC0XM071699 | 1.254212618 |
| SLC35E2B | Solute Carrier Family 35 Member E2B | Protein Coding | 26 | GC01M001659 | 1.253704906 |
| MCHR2 | Melanin Concentrating Hormone Receptor 2 | Protein Coding | 37 | GC06M099919 | 1.253558636 |
| PPP1R14B | Protein Phosphatase 1 Regulatory Inhibitor Subunit 14B | Protein Coding | 31 | GC11M064244 | 1.253371 |
| PPP1R2P1 | Protein Phosphatase 1 Regulatory Inhibitor Subunit 2 Pseudogene 1 | Pseudogene | 18 | GC06M032876 | 1.253267527 |
| SLC22A11 | Solute Carrier Family 22 Member 11 | Protein Coding | 37 | GC11P064573 | 1.253146648 |
| RPL26L1 | Ribosomal Protein L26 Like 1 | Protein Coding | 31 | GC05P172958 | 1.253027201 |
| POLR3C | RNA Polymerase III Subunit C | Protein Coding | 34 | GC01P145911 | 1.252931714 |
| UBE2E2 | Ubiquitin Conjugating Enzyme E2 E2 | Protein Coding | 35 | GC03P023221 | 1.252808809 |
| MIR1233-1 | MicroRNA 1233-1 | RNA Gene | 10 | GC15M034382 | 1.252495408 |
| TFIP11 | Tuftelin Interacting Protein 11 | Protein Coding | 34 | GC22M026491 | 1.252287865 |
| STEEP1 | STING1 ER Exit Protein 1 | Protein Coding | 27 | GC0XM119540 | 1.252212048 |
| NPRL3 | NPR3 Like, GATOR1 Complex Subunit | Protein Coding | 33 | GC16M000084 | 1.251913548 |
| MIR515-2 | MicroRNA 515-2 | RNA Gene | 14 | GC19P053685 | 1.25153327 |
| IGES | Immunoglobulin E Concentration, Serum | Genetic Locus | 3 | GC05U990033 | 1.251503229 |
| KLF13 | Kruppel Like Factor 13 | Protein Coding | 36 | GC15P031326 | 1.251342893 |
| EPC2 | Enhancer Of Polycomb Homolog 2 | Protein Coding | 31 | GC02P148644 | 1.251238108 |
| C1orf116 | Chromosome 1 Open Reading Frame 116 | Protein Coding | 30 | GC01M207018 | 1.250908136 |
| METTL16 | Methyltransferase 16, N6-Methyladenosine | Protein Coding | 26 | GC17M002405 | 1.250844479 |
| PLP2 | Proteolipid Protein 2 | Protein Coding | 33 | GC0XP049171 | 1.250471115 |
| MRPS17 | Mitochondrial Ribosomal Protein S17 | Protein Coding | 34 | GC07P056591 | 1.250106335 |
| USP9Y | Ubiquitin Specific Peptidase 9 Y-Linked | Protein Coding | 34 | GC0YP012556 | 1.249741793 |
| DTNA | Dystrobrevin Alpha | Protein Coding | 39 | GC18P034493 | 1.249601483 |
| CES3 | Carboxylesterase 3 | Protein Coding | 36 | GC16P066963 | 1.249018431 |
| GPAA1 | Glycosylphosphatidylinositol Anchor Attachment 1 | Protein Coding | 38 | GC08P144082 | 1.248828411 |
| LRFN2 | Leucine Rich Repeat And Fibronectin Type III Domain Containing 2 | Protein Coding | 35 | GC06M063783 | 1.248735666 |
| ZFPL1 | Zinc Finger Protein Like 1 | Protein Coding | 31 | GC11P065084 | 1.248640299 |
| PSME1 | Proteasome Activator Subunit 1 | Protein Coding | 37 | GC14P024136 | 1.248417735 |
| KAZN | Kazrin, Periplakin Interacting Protein | Protein Coding | 28 | GC01P013893 | 1.248245716 |
| WDR7 | WD Repeat Domain 7 | Protein Coding | 30 | GC18P056651 | 1.248137474 |
| THEMIS | Thymocyte Selection Associated | Protein Coding | 34 | GC06M127708 | 1.248076916 |
| DCUN1D4 | Defective In Cullin Neddylation 1 Domain Containing 4 | Protein Coding | 30 | GC04P051833 | 1.247996688 |
| MRPL12 | Mitochondrial Ribosomal Protein L12 | Protein Coding | 36 | GC17P081704 | 1.247804403 |
| RPUSD1 | RNA Pseudouridine Synthase Domain Containing 1 | Protein Coding | 28 | GC16M006619 | 1.246989131 |
| C7orf50 | Chromosome 7 Open Reading Frame 50 | Protein Coding | 27 | GC07M001085 | 1.246717453 |
| CHRAC1 | Chromatin Accessibility Complex Subunit 1 | Protein Coding | 32 | GC08P140511 | 1.246512175 |
| SACM1L | SAC1 Like Phosphatidylinositide Phosphatase | Protein Coding | 34 | GC03P046375 | 1.245826721 |
| NUP155 | Nucleoporin 155 | Protein Coding | 40 | GC05M037288 | 1.245301723 |
| HOMER3 | Homer Scaffold Protein 3 | Protein Coding | 35 | GC19M018933 | 1.245159507 |
| TXNDC9 | Thioredoxin Domain Containing 9 | Protein Coding | 32 | GC02M099301 | 1.244978905 |

| | | | | | |
|---|---|---|---|---|---|
| APPL2 | Adaptor Protein, Phosphotyrosine Interacting With PH Domain And Leucine Zipper 2 | Protein Coding | 31 | GC12M105173 | 1.244849205 |
| BIN3 | Bridging Integrator 3 | Protein Coding | 32 | GC08M022620 | 1.244793415 |
| CEP72 | Centrosomal Protein 72 | Protein Coding | 34 | GC05P000612 | 1.244653583 |
| ARHGEF40 | Rho Guanine Nucleotide Exchange Factor 40 | Protein Coding | 30 | GC14P031552 | 1.244473457 |
| BHLHA9 | Basic Helix-Loop-Helix Family Member A9 | Protein Coding | 27 | GC17P001270 | 1.244384527 |
| POLR3K | RNA Polymerase III Subunit K | Protein Coding | 35 | GC16M000046 | 1.244290113 |
| ZNF746 | Zinc Finger Protein 746 | Protein Coding | 31 | GC07M149472 | 1.244214177 |
| ZNF322 | Zinc Finger Protein 322 | Protein Coding | 28 | GC06M063215 | 1.24420476 |
| MIR562 | MicroRNA 562 | RNA Gene | 15 | GC02P232172 | 1.24412775 |
| CERS6-AS1 | CERS6 Antisense RNA 1 | RNA Gene | 11 | GC02M168771 | 1.244036555 |
| DEFA6 | Defensin Alpha 6 | Protein Coding | 32 | GC08M006924 | 1.243533969 |
| TAF1L | TATA-Box Binding Protein Associated Factor 1 Like | Protein Coding | 30 | GC09M032619 | 1.243353844 |
| SFSWAP | Splicing Factor SWAP | Protein Coding | 30 | GC12P131711 | 1.243183374 |
| SIGLEC9 | Sialic Acid Binding Ig Like Lectin 9 | Protein Coding | 33 | GC19P051124 | 1.242751837 |
| HSPBP1 | HSPA (Hsp70) Binding Protein 1 | Protein Coding | 31 | GC19M055262 | 1.24160707 |
| ARHGAP18 | Rho GTPase Activating Protein 18 | Protein Coding | 33 | GC06M129576 | 1.241439819 |
| GTF3C1 | General Transcription Factor IIIC Subunit 1 | Protein Coding | 32 | GC16M027471 | 1.241402984 |
| ART3 | ADP-Ribosyltransferase 3 (Inactive) | Protein Coding | 36 | GC04P076011 | 1.24136734 |
| RCBTB2 | RCC1 And BTB Domain Containing Protein 2 | Protein Coding | 33 | GC13M048488 | 1.24109447 |
| WIZ | WIZ Zinc Finger | Protein Coding | 31 | GC19M015419 | 1.240798235 |
| RPL39P5 | Ribosomal Protein L39 Pseudogene 5 | Pseudogene | 15 | GC03M134351 | 1.240703344 |
| UHRF1BP1L | UHRF1 Binding Protein 1 Like | Protein Coding | 31 | GC12M100028 | 1.240617037 |
| YIPF5 | Yip1 Domain Family Member 5 | Protein Coding | 34 | GC05M144158 | 1.240489125 |
| H2BC13 | H2B Clustered Histone 13 | Protein Coding | 27 | GC06M063335 | 1.240319967 |
| TMEM92 | Transmembrane Protein 92 | Protein Coding | 30 | GC17P053544 | 1.24023366 |
| NFIA-AS1 | NFIA Antisense RNA 1 | RNA Gene | 14 | GC01M061248 | 1.239905 |
| CLIC5 | Chloride Intracellular Channel 5 | Protein Coding | 39 | GC06M045880 | 1.239854813 |
| HDGFL2 | HDGF Like 2 | Protein Coding | 27 | GC19P004584 | 1.239570379 |
| KRT80 | Keratin 80 | Protein Coding | 31 | GC12M052168 | 1.239318848 |
| GLRB | Glycine Receptor Beta | Protein Coding | 43 | GC04P157076 | 1.239189386 |
| NOL9 | Nucleolar Protein 9 | Protein Coding | 29 | GC01M006524 | 1.238886595 |
| SLFN5 | Schlafen Family Member 5 | Protein Coding | 28 | GC17P035243 | 1.23872602 |
| PHF23 | PHD Finger Protein 23 | Protein Coding | 28 | GC17M007237 | 1.238328457 |
| ERVK-21 | Endogenous Retrovirus Group K Member 21, Envelope | Protein Coding | 9 | GC12U901595 | 1.23831439 |
| STAMBPL1 | STAM Binding Protein Like 1 | Protein Coding | 34 | GC10P088879 | 1.238086343 |
| PROCA1 | Protein Interacting With Cyclin A1 | Protein Coding | 28 | GC17M028703 | 1.237141132 |
| DDX19A | DEAD-Box Helicase 19A | Protein Coding | 31 | GC16P070346 | 1.236910105 |
| CYP4A11 | Cytochrome P450 Family 4 Subfamily A Member 11 | Protein Coding | 39 | GC01M046929 | 1.236756921 |
| TSPAN10 | Tetraspanin 10 | Protein Coding | 29 | GC17P081637 | 1.236450672 |
| IFNL3 | Interferon Lambda 3 | Protein Coding | 32 | GC19M039243 | 1.236162543 |
| ZNF234 | Zinc Finger Protein 234 | Protein Coding | 28 | GC19P044141 | 1.236088037 |
| SLC35E2A | Solute Carrier Family 35 Member E2A | Pseudogene | 23 | GC01M005565 | 1.236088037 |
| ENSG00000284299 | Novel Protein | Protein Coding | 7 | GC01M210858 | 1.236088037 |
| SOX13 | SRY-Box Transcription Factor 13 | Protein Coding | 36 | GC01P204074 | 1.235954285 |
| CCDC167 | Coiled-Coil Domain Containing 167 | Protein Coding | 25 | GC06M037482 | 1.235784531 |
| TMEM245 | Transmembrane Protein 245 | Protein Coding | 29 | GC09M109017 | 1.235333204 |
| TOMM70 | Translocase Of Outer Mitochondrial Membrane 70 | Protein Coding | 30 | GC03M100364 | 1.235253334 |
| POU3F1 | POU Class 3 Homeobox 1 | Protein Coding | 36 | GC01M038069 | 1.235071182 |
| EPN3 | Epsin 3 | Protein Coding | 32 | GC17P050532 | 1.234701037 |
| PDIA5 | Protein Disulfide Isomerase Family A Member 5 | Protein Coding | 33 | GC03P123067 | 1.234619498 |
| ATP8A2 | ATPase Phospholipid Transporting 8A2 | Protein Coding | 36 | GC13P025386 | 1.234312057 |
| NUTM2G | NUT Family Member 2G | Protein Coding | 21 | GC09P096928 | 1.234118938 |
| UBE2R2 | Ubiquitin Conjugating Enzyme E2 R2 | Protein Coding | 38 | GC09P033817 | 1.234033942 |
| POMK | Protein O-Mannose Kinase | Protein Coding | 33 | GC08P043093 | 1.233948708 |
| MMP28 | Matrix Metallopeptidase 28 | Protein Coding | 33 | GC17M035756 | 1.233734131 |
| RRAGB | Ras Related GTP Binding B | Protein Coding | 33 | GC0XP055717 | 1.233666539 |
| WDR6 | WD Repeat Domain 6 | Protein Coding | 32 | GC03P049007 | 1.232148886 |
| SLC46A2 | Solute Carrier Family 46 Member 2 | Protein Coding | 31 | GC09M112878 | 1.231945634 |
| PAPPA2 | Pappalysin 2 | Protein Coding | 36 | GC01P176463 | 1.231938839 |
| ADCK1 | AarF Domain Containing Kinase 1 | Protein Coding | 33 | GC14P077800 | 1.231640816 |
| SNX10 | Sorting Nexin 10 | Protein Coding | 36 | GC07P026291 | 1.231113434 |
| NPM2 | Nucleophosmin/Nucleoplasmin 2 | Protein Coding | 32 | GC08P022024 | 1.231081963 |
| ANKZF1 | Ankyrin Repeat And Zinc Finger Peptidyl TRNA Hydrolase 1 | Protein Coding | 31 | GC02P219229 | 1.230866313 |
| LSM14B | LSM Family Member 14B | Protein Coding | 29 | GC20P062122 | 1.230866313 |
| PLEKHG3 | Pleckstrin Homology And RhoGEF Domain Containing G3 | Protein Coding | 31 | GC14P064703 | 1.230367661 |
| HAUS4 | HAUS Augmin Like Complex Subunit 4 | Protein Coding | 30 | GC14M022947 | 1.230294108 |
| C1R | Complement C1r | Protein Coding | 44 | GC12M007682 | 1.230111241 |
| MRPS18C | Mitochondrial Ribosomal Protein S18C | Protein Coding | 32 | GC04P083455 | 1.229921341 |
| ZNF841 | Zinc Finger Protein 841 | Protein Coding | 24 | GC19M064277 | 1.229871631 |
| CARMN | Cardiac Mesoderm Enhancer-Associated Non-Coding RNA | RNA Gene | 17 | GC05P149407 | 1.229756474 |
| QARS1 | Glutaminyl-TRNA Synthetase 1 | Protein Coding | 28 | GC03M051019 | 1.229572296 |
| BRCA1P1 | BRCA1 Pseudogene 1 | Pseudogene | 7 | GC17M043908 | 1.229450703 |
| PRR4 | Proline Rich 4 | Protein Coding | 29 | GC12M020458 | 1.228701711 |
| FAM50A | Family With Sequence Similarity 50 Member A | Protein Coding | 33 | GC0XP154445 | 1.228455782 |
| AKAP17A | A-Kinase Anchoring Protein 17A | Protein Coding | 30 | GC0XP001591 | 1.228314042 |
| RNF17 | Ring Finger Protein 17 | Protein Coding | 31 | GC13P024747 | 1.228267908 |
| DYNC1LI1 | Dynein Cytoplasmic 1 Light Intermediate Chain 1 | Protein Coding | 31 | GC03M032543 | 1.228164077 |
| GPN3 | GPN-Loop GTPase 3 | Protein Coding | 28 | GC12M110452 | 1.227827549 |
| HOXC-AS3 | HOXC Cluster Antisense RNA 3 | RNA Gene | 15 | GC12M053981 | 1.227710962 |
| PLAAT2 | Phospholipase A And Acyltransferase 2 | Protein Coding | 24 | GC11M063552 | 1.22766912 |
| NOM1 | Nucleolar Protein With MIF4G Domain 1 | Protein Coding | 33 | GC07P156949 | 1.227130175 |
| MIR616 | MicroRNA 616 | RNA Gene | 17 | GC12M057519 | 1.226741433 |
| TDRD10 | Tudor Domain Containing 10 | Protein Coding | 26 | GC01P154502 | 1.226269007 |
| RBM12B | RNA Binding Motif Protein 12B | Protein Coding | 30 | GC08M093729 | 1.226065278 |
| ZSWIM5 | Zinc Finger SWIM-Type Containing 5 | Protein Coding | 31 | GC01M045016 | 1.22588563 |
| TMEM241 | Transmembrane Protein 241 | Protein Coding | 28 | GC18M023197 | 1.22588563 |
| SNUPN | Snurportin 1 | Protein Coding | 31 | GC15M075598 | 1.225473881 |
| SUGCT | Succinyl-CoA:Glutarate-CoA Transferase | Protein Coding | 32 | GC07P040134 | 1.225409508 |
| PAM16 | Presequence Translocase Associated Motor 16 | Protein Coding | 32 | GC16M006871 | 1.225315571 |
| BBX | BBX High Mobility Group Box Domain Containing | Protein Coding | 30 | GC03P107522 | 1.225119591 |
| CGREF1 | Cell Growth Regulator With EF-Hand Domain 1 | Protein Coding | 30 | GC02M027098 | 1.225082397 |
| TASOR | Transcription Activation Suppressor | Protein Coding | 27 | GC03M056620 | 1.224817514 |
| SCFD1 | Sec1 Family Domain Containing 1 | Protein Coding | 34 | GC14P030622 | 1.224640965 |
| NPNT | Nephronectin | Protein Coding | 35 | GC04P105894 | 1.224406958 |

| | | | | | |
|---|---|---|---|---|---|
| AFMID | Arylformamidase | Protein Coding | 32 | GC17P078185 | 1.224308372 |
| SUCLG1 | Succinate-CoA Ligase GDP/ADP-Forming Subunit Alpha | Protein Coding | 41 | GC02M084423 | 1.224045396 |
| DOP1B | DOP1 Leucine Zipper Like Protein B | Protein Coding | 26 | GC21P036164 | 1.223766565 |
| PGM5P3-AS1 | PGM5P3 Antisense RNA 1 | RNA Gene | 11 | GC09P000073 | 1.222834706 |
| MPP2 | MAGUK P55 Scaffold Protein 2 | Protein Coding | 34 | GC17M043875 | 1.222755313 |
| TMCC1 | Transmembrane And Coiled-Coil Domain Family 1 | Protein Coding | 29 | GC03M129647 | 1.222729325 |
| NDUFA6 | NADH:Ubiquinone Oxidoreductase Subunit A6 | Protein Coding | 40 | GC22M042085 | 1.221809864 |
| RPP25L | Ribonuclease P/MRP Subunit P25 Like | Protein Coding | 29 | GC09M034610 | 1.221314192 |
| SGMS1 | Sphingomyelin Synthase 1 | Protein Coding | 36 | GC10M050305 | 1.221118927 |
| NREP | Neuronal Regeneration Related Protein | Protein Coding | 32 | GC05M111662 | 1.220844388 |
| MIR1285-2 | MicroRNA 1285-2 | RNA Gene | 10 | GC02M070252 | 1.220374227 |
| EOGT | EGF Domain Specific O-Linked N-Acetylglucosamine Transferase | Protein Coding | 33 | GC03M068975 | 1.220009923 |
| MPP7 | MAGUK P55 Scaffold Protein 7 | Protein Coding | 32 | GC10M028057 | 1.219458222 |
| ZNF423 | Zinc Finger Protein 423 | Protein Coding | 36 | GC16M049487 | 1.219015121 |
| LINC00908 | Long Intergenic Non-Protein Coding RNA 908 | RNA Gene | 15 | GC18P076528 | 1.218664765 |
| ELF4 | E74 Like ETS Transcription Factor 4 | Protein Coding | 35 | GC0XM130064 | 1.21863246 |
| SNX14 | Sorting Nexin 14 | Protein Coding | 34 | GC06M085505 | 1.218298674 |
| SNRPC | Small Nuclear Ribonucleoprotein Polypeptide C | Protein Coding | 33 | GC06P080502 | 1.218175054 |
| RFXANK | Regulatory Factor X Associated Ankyrin Containing Protein | Protein Coding | 36 | GC19P019192 | 1.217638731 |
| SYT14 | Synaptotagmin 14 | Protein Coding | 36 | GC01P209900 | 1.217538595 |
| RNF145 | Ring Finger Protein 145 | Protein Coding | 31 | GC05M159157 | 1.217538595 |
| CCDC30 | Coiled-Coil Domain Containing 30 | Protein Coding | 27 | GC01P042463 | 1.217538595 |
| MIR664A | MicroRNA 664a | RNA Gene | 13 | GC01M220200 | 1.217365146 |
| CASD1 | CAS1 Domain Containing 1 | Protein Coding | 31 | GC07P094509 | 1.217333436 |
| TMEM258 | Transmembrane Protein 258 | Protein Coding | 27 | GC11M061768 | 1.217313051 |
| DAGLB | Diacylglycerol Lipase Beta | Protein Coding | 35 | GC07M006416 | 1.217192769 |
| CDK16 | Cyclin Dependent Kinase 16 | Protein Coding | 38 | GC0XP047217 | 1.217108607 |
| LOC100130691 | Uncharacterized LOC100130691 | RNA Gene | 13 | GC02M177283 | 1.216924906 |
| BTNL9 | Butyrophilin Like 9 | Protein Coding | 30 | GC05P183254 | 1.216376424 |
| LINC01561 | Long Intergenic Non-Protein Coding RNA 1561 | RNA Gene | 14 | GC10P120597 | 1.216057658 |
| CDC42BPG | CDC42 Binding Protein Kinase Gamma | Protein Coding | 34 | GC11M064823 | 1.215877533 |
| RNF181 | Ring Finger Protein 181 | Protein Coding | 32 | GC02P085593 | 1.215453386 |
| FFAR2 | Free Fatty Acid Receptor 2 | Protein Coding | 38 | GC19P063450 | 1.21511209 |
| TOMM22 | Translocase Of Outer Mitochondrial Membrane 22 | Protein Coding | 31 | GC22P038681 | 1.21508348 |
| TAF1C | TATA-Box Binding Protein Associated Factor, RNA Polymerase I Subunit C | Protein Coding | 33 | GC16M084177 | 1.214792848 |
| MPHOSPH10 | M-Phase Phosphoprotein 10 | Protein Coding | 32 | GC02P071130 | 1.214621305 |
| MIR567 | MicroRNA 567 | RNA Gene | 14 | GC03P112113 | 1.214547753 |
| SGCA | Sarcoglycan Alpha | Protein Coding | 38 | GC17P050164 | 1.214390159 |
| TSKU | Tsukushi, Small Leucine Rich Proteoglycan | Protein Coding | 31 | GC11P076782 | 1.214251757 |
| SLC14A1 | Solute Carrier Family 14 Member 1 (Kidd Blood Group) | Protein Coding | 39 | GC18P045687 | 1.214071751 |
| CASP8AP2 | Caspase 8 Associated Protein 2 | Protein Coding | 32 | GC06P089829 | 1.213959455 |
| TCEA2 | Transcription Elongation Factor A2 | Protein Coding | 33 | GC20P064985 | 1.213645935 |
| MMP27 | Matrix Metallopeptidase 27 | Protein Coding | 33 | GC11M102596 | 1.213392258 |
| STK17B | Serine/Threonine Kinase 17b | Protein Coding | 38 | GC02M196133 | 1.213202357 |
| KCNA2 | Potassium Voltage-Gated Channel Subfamily A Member 2 | Protein Coding | 42 | GC01M110519 | 1.213126898 |
| SMYD4 | SET And MYND Domain Containing 4 | Protein Coding | 33 | GC17M001779 | 1.212684393 |
| TPSG1 | Tryptase Gamma 1 | Protein Coding | 36 | GC16M001221 | 1.210771322 |
| KATNIP | Katanin Interacting Protein | Protein Coding | 27 | GC16P027828 | 1.210664153 |
| LINC00494 | Long Intergenic Non-Protein Coding RNA 494 | RNA Gene | 14 | GC20P048359 | 1.210365295 |
| TMEM120A | Transmembrane Protein 120A | Protein Coding | 28 | GC07M075986 | 1.210275054 |
| EXOC8 | Exocyst Complex Component 8 | Protein Coding | 34 | GC01M231332 | 1.210223198 |
| HCFC2 | Host Cell Factor C2 | Protein Coding | 32 | GC12P104064 | 1.209693432 |
| MIR190B | MicroRNA 190b | RNA Gene | 17 | GC01M154193 | 1.209463835 |
| CABIN1 | Calcineurin Binding Protein 1 | Protein Coding | 38 | GC22P024011 | 1.209033608 |
| LLPH | LLP Homolog, Long-Term Synaptic Facilitation Factor | Protein Coding | 29 | GC12M066116 | 1.208883047 |
| ATG2B | Autophagy Related 2B | Protein Coding | 31 | GC14M100472 | 1.20857203 |
| PLEKHH3 | Pleckstrin Homology, MyTH4 And FERM Domain Containing H3 | Protein Coding | 28 | GC17M042669 | 1.208461523 |
| LRRD1 | Leucine Rich Repeats And Death Domain Containing 1 | Protein Coding | 23 | GC07M092141 | 1.208461523 |
| MRPL40 | Mitochondrial Ribosomal Protein L40 | Protein Coding | 33 | GC22P034394 | 1.208356857 |
| IFI27L2 | Interferon Alpha Inducible Protein 27 Like 2 | Protein Coding | 29 | GC14M094127 | 1.208313942 |
| CABLES2 | Cdk5 And Abl Enzyme Substrate 2 | Protein Coding | 31 | GC20M062388 | 1.207467437 |
| FAAP20 | FA Core Complex Associated Protein 20 | Protein Coding | 25 | GC01M005579 | 1.207197785 |
| NUDT19 | Nudix Hydrolase 19 | Protein Coding | 31 | GC19P063374 | 1.207171202 |
| CRB2 | Crumbs Cell Polarity Complex Component 2 | Protein Coding | 38 | GC09P123356 | 1.207125783 |
| GNPDA1 | Glucosamine-6-Phosphate Deaminase 1 | Protein Coding | 34 | GC05M141991 | 1.206846356 |
| PRSS16 | Serine Protease 16 | Protein Coding | 34 | GC06P027247 | 1.206825972 |
| CLC | Charcot-Leyden Crystal Galectin | Protein Coding | 36 | GC19M063780 | 1.206698537 |
| GK | Glycerol Kinase | Protein Coding | 42 | GC0XP030975 | 1.205793262 |
| HLA-H | Major Histocompatibility Complex, Class I, H (Pseudogene) | Pseudogene | 23 | GC06P080303 | 1.205365181 |
| KIF18B | Kinesin Family Member 18B | Protein Coding | 31 | GC17M044924 | 1.20524025 |
| CYP4F3 | Cytochrome P450 Family 4 Subfamily F Member 3 | Protein Coding | 39 | GC19P015640 | 1.205113173 |
| HSDL1 | Hydroxysteroid Dehydrogenase Like 1 | Protein Coding | 30 | GC16M084123 | 1.204525352 |
| ANKRD13A | Ankyrin Repeat Domain 13A | Protein Coding | 33 | GC12P109999 | 1.203522682 |
| ROBO3 | Roundabout Guidance Receptor 3 | Protein Coding | 40 | GC11P124865 | 1.203505754 |
| CBX6 | Chromobox 6 | Protein Coding | 32 | GC22M038861 | 1.203212976 |
| VRK3 | VRK Serine/Threonine Kinase 3 | Protein Coding | 36 | GC19M049976 | 1.203095436 |
| AVL9 | AVL9 Cell Migration Associated | Protein Coding | 30 | GC07P032495 | 1.203059435 |
| WHAMM | WASP Homolog Associated With Actin, Golgi Membranes And Microtubules | Protein Coding | 30 | GC15P115029 | 1.202692032 |
| XYLB | Xylulokinase | Protein Coding | 34 | GC03P038363 | 1.20269084 |
| SSH2 | Slingshot Protein Phosphatase 2 | Protein Coding | 35 | GC17M029625 | 1.202459097 |
| ZGRF1 | Zinc Finger GRF-Type Containing 1 | Protein Coding | 28 | GC04M112539 | 1.202427626 |
| DLD | Dihydrolipoamide Dehydrogenase | Protein Coding | 44 | GC07P107890 | 1.201740265 |
| MED26 | Mediator Complex Subunit 26 | Protein Coding | 31 | GC19M016574 | 1.20159924 |
| UBXN2A | UBX Domain Protein 2A | Protein Coding | 30 | GC02P023927 | 1.200932264 |
| KLHL38 | Kelch Like Family Member 38 | Protein Coding | 30 | GC08M123645 | 1.200356483 |
| INTS15 | Integrator Complex Subunit 15 | Protein Coding | 22 | GC07P007219 | 1.200333118 |
| PUDP | Pseudouridine 5'-Phosphatase | Protein Coding | 30 | GC0XM006668 | 1.200107813 |
| SLITRK4 | SLIT And NTRK Like Family Member 4 | Protein Coding | 31 | GC0XM143622 | 1.19995141 |
| RFESD | Rieske Fe-S Domain Containing | Protein Coding | 30 | GC05P095646 | 1.199888706 |
| TMEM131 | Transmembrane Protein 131 | Protein Coding | 30 | GC02M098062 | 1.199408054 |
| USP51 | Ubiquitin Specific Peptidase 51 | Protein Coding | 28 | GC0XM055484 | 1.19937706 |
| MIR887 | MicroRNA 887 | RNA Gene | 15 | GC05P015935 | 1.199334621 |
| ZNF566 | Zinc Finger Protein 566 | Protein Coding | 30 | GC19M065512 | 1.198836327 |
| CENPM | Centromere Protein M | Protein Coding | 31 | GC22M056121 | 1.198764086 |
| APCDD1 | APC Down-Regulated 1 | Protein Coding | 36 | GC18P010454 | 1.19876194 |

| | | | | | |
|---|---|---|---|---|---|
| DMAC1 | Distal Membrane Arm Assembly Component 1 | Protein Coding | 26 | GC09M007798 | 1.198750019 |
| CD300LF | CD300 Molecule Like Family Member F | Protein Coding | 35 | GC17M074694 | 1.198421359 |
| ACOX2 | Acyl-CoA Oxidase 2 | Protein Coding | 38 | GC03M058490 | 1.198419929 |
| SLC41A2 | Solute Carrier Family 41 Member 2 | Protein Coding | 32 | GC12M104802 | 1.198190212 |
| ANKRD46 | Ankyrin Repeat Domain 46 | Protein Coding | 30 | GC08M100510 | 1.197291136 |
| RRP8 | Ribosomal RNA Processing 8 | Protein Coding | 32 | GC11M006514 | 1.197231174 |
| PATL1 | PAT1 Homolog 1, Processing Body MRNA Decay Factor | Protein Coding | 31 | GC11M059636 | 1.197215557 |
| ACSF3 | Acyl-CoA Synthetase Family Member 3 | Protein Coding | 39 | GC16P089088 | 1.196736932 |
| ZNF607 | Zinc Finger Protein 607 | Protein Coding | 31 | GC19M037696 | 1.196582913 |
| NAA20 | N-Alpha-Acetyltransferase 20, NatB Catalytic Subunit | Protein Coding | 34 | GC20P020018 | 1.196497202 |
| TXLNG | Taxilin Gamma | Protein Coding | 29 | GC0XP016787 | 1.196494699 |
| ITGA8 | Integrin Subunit Alpha 8 | Protein Coding | 39 | GC10M015513 | 1.196320057 |
| RCOR3 | REST Corepressor 3 | Protein Coding | 31 | GC01P211258 | 1.196141481 |
| PINLYP | Phospholipase A2 Inhibitor And LY6/PLAUR Domain Containing | Protein Coding | 23 | GC19P063773 | 1.196107388 |
| SKAP2 | Src Kinase Associated Phosphoprotein 2 | Protein Coding | 36 | GC07M026654 | 1.196040511 |
| PILRB | Paired Immunoglobin Like Type 2 Receptor Beta | Protein Coding | 33 | GC07P100961 | 1.195915699 |
| ZC3H6 | Zinc Finger CCCH-Type Containing 6 | Protein Coding | 27 | GC02P121627 | 1.195915699 |
| NDUFA12 | NADH:Ubiquinone Oxidoreductase Subunit A12 | Protein Coding | 40 | GC12M094898 | 1.195823431 |
| PCYT2 | Phosphate Cytidylyltransferase 2, Ethanolamine | Protein Coding | 37 | GC17M081900 | 1.195792198 |
| USPL1 | Ubiquitin Specific Peptidase Like 1 | Protein Coding | 29 | GC13P030617 | 1.195698977 |
| CTU2 | Cytosolic Thiouridylase Subunit 2 | Protein Coding | 30 | GC16P088706 | 1.195620775 |
| GTF3C5 | General Transcription Factor IIIC Subunit 5 | Protein Coding | 31 | GC09P133030 | 1.194974422 |
| DCP1B | Decapping MRNA 1B | Protein Coding | 32 | GC12M001941 | 1.194915295 |
| S1PR4 | Sphingosine-1-Phosphate Receptor 4 | Protein Coding | 38 | GC19P003206 | 1.194843769 |
| JMY | Junction Mediating And Regulatory Protein, P53 Cofactor | Protein Coding | 32 | GC05P079236 | 1.194312572 |
| SYT1 | Synaptotagmin 1 | Protein Coding | 43 | GC12P078863 | 1.194263339 |
| MRPS14 | Mitochondrial Ribosomal Protein S14 | Protein Coding | 31 | GC01M175010 | 1.194201708 |
| COMMD9 | COMM Domain Containing 9 | Protein Coding | 30 | GC11M036293 | 1.194159985 |
| PI15 | Peptidase Inhibitor 15 | Protein Coding | 32 | GC08P074766 | 1.194144607 |
| SLC6A15 | Solute Carrier Family 6 Member 15 | Protein Coding | 36 | GC12M084859 | 1.193887949 |
| TXNDC12 | Thioredoxin Domain Containing 12 | Protein Coding | 32 | GC01M052020 | 1.193654418 |
| IGHA2 | Immunoglobulin Heavy Constant Alpha 2 (A2m Marker) | Protein Coding | 23 | GC14M112399 | 1.193648815 |
| IMP4 | IMP U3 Small Nucleolar Ribonucleoprotein 4 | Protein Coding | 31 | GC02P130342 | 1.193571806 |
| C1QL4 | Complement C1q Like 4 | Protein Coding | 30 | GC12M049332 | 1.193557262 |
| ZNF226 | Zinc Finger Protein 226 | Protein Coding | 30 | GC19P044165 | 1.193557262 |
| ZNF235 | Zinc Finger Protein 235 | Protein Coding | 30 | GC19M044228 | 1.193557262 |
| ZNF284 | Zinc Finger Protein 284 | Protein Coding | 27 | GC19P044072 | 1.193557262 |
| ZNF613 | Zinc Finger Protein 613 | Protein Coding | 27 | GC19P051927 | 1.193557262 |
| ZNF285 | Zinc Finger Protein 285 | Protein Coding | 26 | GC19M063918 | 1.193557262 |
| FAM87B | Family With Sequence Similarity 87 Member B | RNA Gene | 16 | GC01P000817 | 1.193557262 |
| HLA-J | Major Histocompatibility Complex, Class I, J (Pseudogene) | Pseudogene | 14 | GC06P080314 | 1.193557262 |
| FRY-AS1 | FRY Antisense RNA 1 | RNA Gene | 13 | GC13M036354 | 1.193557262 |
| RALY-AS1 | RALY Antisense RNA 1 | RNA Gene | 11 | GC20M033983 | 1.193557262 |
| LOC100505715 | Uncharacterized LOC100505715 | RNA Gene | 10 | GC19M063908 | 1.193557262 |
| LINC01786 | Long Intergenic Non-Protein Coding RNA 1786 | RNA Gene | 9 | GC01P003598 | 1.193557262 |
| MRPS35-DT | MRPS35 Divergent Transcript | RNA Gene | 9 | GC12M027691 | 1.193557262 |
| PCNPP1 | PEST Containing Nuclear Protein Pseudogene 1 | Pseudogene | 9 | GC12M111666 | 1.193557262 |
| ENSG00000258017 | Novel Transcript, Antisense To TUBA1B | RNA Gene | 8 | GC12P049110 | 1.193557262 |
| LINC01997 | Long Intergenic Non-Protein Coding RNA 1997 | RNA Gene | 8 | GC03P168953 | 1.193557262 |
| RPL7P8 | Ribosomal Protein L7 Pseudogene 8 | Pseudogene | 8 | GC01P109651 | 1.193557262 |
| ZNF45-AS1 | ZNF45 Antisense RNA 1 | RNA Gene | 8 | GC19P063790 | 1.193557262 |
| ENSG00000240731 | Novel Transcript | RNA Gene | 7 | GC01M005529 | 1.193557262 |
| ENSG00000248373 | Novel Transcript | RNA Gene | 7 | GC04P104653 | 1.193557262 |
| ENSG00000251259 | Novel Transcript | RNA Gene | 7 | GC04M105137 | 1.193557262 |
| ENSG00000254987 | Novel Transcript | RNA Gene | 7 | GC11M103674 | 1.193557262 |
| ENSG00000258101 | Novel Transcript, Antisense To TUBA1C | RNA Gene | 7 | GC12M049232 | 1.193557262 |
| MICC | MHC Class I Polypeptide-Related Sequence C (Pseudogene) | Pseudogene | 7 | GC06P080324 | 1.193557262 |
| RNU6-351P | RNA, U6 Small Nuclear 351, Pseudogene | Pseudogene | 7 | GC04P104974 | 1.193557262 |
| ENSG00000228650 | Novel Transcript | RNA Gene | 6 | GC05P056771 | 1.193557262 |
| ENSG00000252136 | | RNA Gene | 6 | GC04P105105 | 1.193557262 |
| ENSG00000257042 | Novel Transcript, Antisense To PTHLH | RNA Gene | 6 | GC12P027810 | 1.193557262 |
| IPO8P1 | Importin 8 Pseudogene 1 | Pseudogene | 6 | GC01M210859 | 1.193557262 |
| MARK2P12 | Microtubule Affinity Regulating Kinase 2 Pseudogene 12 | Pseudogene | 6 | GC13M073407 | 1.193557262 |
| RNU4-71P | RNA, U4 Small Nuclear 71, Pseudogene | Pseudogene | 6 | GC08M023600 | 1.193557262 |
| RPL17P25 | Ribosomal Protein L17 Pseudogene 25 | Pseudogene | 6 | GC06M080373 | 1.193557262 |
| UBQLN1P1 | Ubiquilin 1 Pseudogene 1 | Pseudogene | 6 | GC06M030358 | 1.193557262 |
| lnc-DVL1-2 | | RNA Gene | 6 | GC01M005533 | 1.193557262 |
| ENSG00000227775 | Ribosomal Protein S7 (RPS7) Pseudogene | Pseudogene | 5 | GC01P003636 | 1.193557262 |
| ENSG00000230092 | F-Box Only Protein 25 (FBXO25) Pseudogene | Pseudogene | 5 | GC01M000800 | 1.193557262 |
| ENSG00000269737 | Ribosomal Protein S7 (RPS7) Pseudogene | Pseudogene | 5 | GC01P001671 | 1.193557262 |
| ENSG00000284615 | Novel Transcript, Sense Intronic To ESR1 | RNA Gene | 5 | GC06P152112 | 1.193557262 |
| HSALNG0000137 | | RNA Gene | 5 | GC01P003607 | 1.193557262 |
| HSALNG0000138 | | RNA Gene | 5 | GC01P003603 | 1.193557262 |
| HSALNG0000139 | | RNA Gene | 5 | GC01M005675 | 1.193557262 |
| HSALNG0000142 | | RNA Gene | 5 | GC01P003744 | 1.193557262 |
| HSALNG0078328 | | RNA Gene | 5 | GC10M062510 | 1.193557262 |
| HSALNG0094037 | | RNA Gene | 5 | GC12P111432 | 1.193557262 |
| NONHSAG000093.2 | | RNA Gene | 5 | GC01P001354 | 1.193557262 |
| RF00017-1167 | | RNA Gene | 5 | GC12M111251 | 1.193557262 |
| RNY1P8 | RNY1 Pseudogene 8 | Pseudogene | 5 | GC13P073227 | 1.193557262 |
| lnc-CCDC91-1 | | RNA Gene | 5 | GC12P028081 | 1.193557262 |
| lnc-DVL1-1 | | RNA Gene | 5 | GC01M005532 | 1.193557262 |
| lnc-FAM109A-1 | | RNA Gene | 5 | GC12M111525 | 1.193557262 |
| lnc-RD3-9 | | RNA Gene | 5 | GC01M210720 | 1.193557262 |
| piR-35674-001 | | RNA Gene | 5 | GC01M005534 | 1.193557262 |
| ENSG00000251473 | Nucleoporin 43kDa (NUP43) Pseudogene | Pseudogene | 4 | GC04M105103 | 1.193557262 |
| ENSG00000270679 | Mitochondrial Ribosomal Protein S15 (MRPS15) Pseudogene | Pseudogene | 4 | GC19P063817 | 1.193557262 |
| ENSG00000274017 | Metazoan Signal Recognition Particle RNA | RNA Gene | 4 | GC12P049196 | 1.193557262 |
| HSALNG0041963 | | RNA Gene | 4 | GC05M056729 | 1.193557262 |
| HSALNG0086841 | | RNA Gene | 4 | GC11P103637 | 1.193557262 |
| HSALNG0091217 | | RNA Gene | 4 | GC12P052902 | 1.193557262 |
| HSALNG0091218 | | RNA Gene | 4 | GC12M052916 | 1.193557262 |
| HSALNG0094038 | | RNA Gene | 4 | GC12P111446 | 1.193557262 |
| HSALNG0102089 | | RNA Gene | 4 | GC14M068557 | 1.193557262 |
| HSALNG0111532 | | RNA Gene | 4 | GC16M053741 | 1.193557262 |

| | | | | | |
|---|---|---|---|---|---|
| HSALNG0129683 | | RNA Gene | 4 | GC20P034041 | 1.193557262 |
| RPSAP72 | Ribosomal Protein SA Pseudogene 72 | Pseudogene | 4 | GC06M080471 | 1.193557262 |
| lnc-FRY-1 | | RNA Gene | 4 | GC13P032297 | 1.193557262 |
| lnc-ZAR1L-2 | | RNA Gene | 4 | GC13M036388 | 1.193557262 |
| piR-36455 | | RNA Gene | 4 | GC12M111444 | 1.193557262 |
| piR-38259 | | RNA Gene | 4 | GC12M111524 | 1.193557262 |
| piR-39858-354 | | RNA Gene | 4 | GC16M053821 | 1.193557262 |
| piR-48007 | | RNA Gene | 4 | GC12M111533 | 1.193557262 |
| piR-49322-121 | | RNA Gene | 4 | GC12M028007 | 1.193557262 |
| piR-50346 | | RNA Gene | 4 | GC12M111534 | 1.193557262 |
| piR-51327 | | RNA Gene | 4 | GC12M111437 | 1.193557262 |
| piR-51449 | | RNA Gene | 4 | GC12P111448 | 1.193557262 |
| piR-56480-015 | | RNA Gene | 4 | GC12M111439 | 1.193557262 |
| piR-59412-008 | | RNA Gene | 4 | GC04P105132 | 1.193557262 |
| HSALNG0007483 | | RNA Gene | 3 | GC01M155226 | 1.193557262 |
| HSALNG0094046 | | RNA Gene | 3 | GC12M111619 | 1.193557262 |
| HSALNG0096212 | | RNA Gene | 3 | GC13P032262 | 1.193557262 |
| HSALNG0124498 | | RNA Gene | 3 | GC19M017280 | 1.193557262 |
| MK279980 | | RNA Gene | 3 | GC01M155212 | 1.193557262 |
| piR-30853-017 | | RNA Gene | 3 | GC11M103785 | 1.193557262 |
| piR-34974-004 | | RNA Gene | 3 | GC11M103837 | 1.193557262 |
| piR-41406-042 | | RNA Gene | 3 | GC20P034038 | 1.193557262 |
| piR-48820-008 | | RNA Gene | 3 | GC01M155215 | 1.193557262 |
| PPP1R15B | Protein Phosphatase 1 Regulatory Subunit 15B | Protein Coding | 33 | GC01M204495 | 1.193398356 |
| ATXN1L | Ataxin 1 Like | Protein Coding | 30 | GC16P071808 | 1.19336915 |
| CFAP20 | Cilia And Flagella Associated Protein 20 | Protein Coding | 27 | GC16M058113 | 1.193118691 |
| LOC111255642 | TNFRSF10B 5' Regulatory Region | Biological Region | 2 | GC08P023106 | 1.193055987 |
| PTPN21 | Protein Tyrosine Phosphatase Non-Receptor Type 21 | Protein Coding | 35 | GC14M088465 | 1.192941904 |
| ZNF800 | Zinc Finger Protein 800 | Protein Coding | 28 | GC07M127346 | 1.192730904 |
| MEX3C | Mex-3 RNA Binding Family Member C | Protein Coding | 34 | GC18M051174 | 1.192720413 |
| SLC24A3 | Solute Carrier Family 24 Member 3 | Protein Coding | 35 | GC20P019212 | 1.192677498 |
| RAB3D | RAB3D, Member RAS Oncogene Family | Protein Coding | 35 | GC19M011322 | 1.192503929 |
| DNASE2 | Deoxyribonuclease 2, Lysosomal | Protein Coding | 35 | GC19M012875 | 1.192292094 |
| ATP6V1G1 | ATPase H+ Transporting V1 Subunit G1 | Protein Coding | 33 | GC09P118623 | 1.191811204 |
| SIKE1 | Suppressor Of IKBKE 1 | Protein Coding | 30 | GC01M114769 | 1.19151926 |
| CERCAM | Cerebral Endothelial Cell Adhesion Molecule | Protein Coding | 30 | GC09P128405 | 1.191376686 |
| MBTD1 | Mbt Domain Containing 1 | Protein Coding | 30 | GC17M051177 | 1.191281796 |
| SNORD115-1 | Small Nucleolar RNA, C/D Box 115-1 | RNA Gene | 14 | GC15P039165 | 1.190843821 |
| OSBPL10 | Oxysterol Binding Protein Like 10 | Protein Coding | 30 | GC03M031677 | 1.190786004 |
| OR10J3 | Olfactory Receptor Family 10 Subfamily J Member 3 | Pseudogene | 26 | GC01M159313 | 1.190232515 |
| PPP1R26-AS1 | PPP1R26 Antisense RNA 1 | RNA Gene | 11 | GC09M135483 | 1.190221667 |
| NUP37 | Nucleoporin 37 | Protein Coding | 32 | GC12M102073 | 1.189982057 |
| SLC25A44 | Solute Carrier Family 25 Member 44 | Protein Coding | 31 | GC01P156195 | 1.189885139 |
| MBNL2 | Muscleblind Like Splicing Regulator 2 | Protein Coding | 33 | GC13P097141 | 1.18986702 |
| LHX9 | LIM Homeobox 9 | Protein Coding | 32 | GC01P197911 | 1.18953371 |
| TTYH1 | Tweety Family Member 1 | Protein Coding | 33 | GC19P064328 | 1.189415455 |
| REEP4 | Receptor Accessory Protein 4 | Protein Coding | 31 | GC08M022138 | 1.189375877 |
| PHPT1 | Phosphohistidine Phosphatase 1 | Protein Coding | 32 | GC09P136848 | 1.189138889 |
| NPY4R | Neuropeptide Y Receptor Y4 | Protein Coding | 36 | GC10M046461 | 1.188561201 |
| UQCC3 | Ubiquinol-Cytochrome C Reductase Complex Assembly Factor 3 | Protein Coding | 28 | GC11P062670 | 1.188474178 |
| NIPSNAP2 | Nipsnap Homolog 2 | Protein Coding | 31 | GC07P056575 | 1.188421965 |
| SEPTIN3 | Septin 3 | Protein Coding | 28 | GC22P043145 | 1.188206196 |
| SNTG2 | Syntrophin Gamma 2 | Protein Coding | 32 | GC02P000942 | 1.188178301 |
| TOR2A | Torsin Family 2 Member A | Protein Coding | 34 | GC09P127789 | 1.188148975 |
| SPDYE12 | Speedy/RINGO Cell Cycle Regulator Family Member E12 | Protein Coding | 10 | GC07M075049 | 1.187966943 |
| MIR3194 | MicroRNA 3194 | RNA Gene | 14 | GC20M051452 | 1.187779427 |
| CHRNB3 | Cholinergic Receptor Nicotinic Beta 3 Subunit | Protein Coding | 40 | GC08P042697 | 1.187506914 |
| ITLN2 | Intelectin 2 | Protein Coding | 30 | GC01M160945 | 1.18750596 |
| DDX31 | DEAD-Box Helicase 31 | Protein Coding | 34 | GC09M132594 | 1.187378764 |
| PSG1 | Pregnancy Specific Beta-1-Glycoprotein 1 | Protein Coding | 35 | GC19M042866 | 1.186555505 |
| MAP3K19 | Mitogen-Activated Protein Kinase Kinase Kinase 19 | Protein Coding | 28 | GC02M134964 | 1.186272264 |
| SNX24 | Sorting Nexin 24 | Protein Coding | 31 | GC05P122843 | 1.186060905 |
| RANBP10 | RAN Binding Protein 10 | Protein Coding | 32 | GC16M067723 | 1.185386658 |
| HIVEP1 | HIVEP Zinc Finger 1 | Protein Coding | 34 | GC06P012009 | 1.185303569 |
| SENP5 | SUMO Specific Peptidase 5 | Protein Coding | 33 | GC03P196869 | 1.185260892 |
| SCUBE1 | Signal Peptide, CUB Domain And EGF Like Domain Containing 1 | Protein Coding | 34 | GC22M043197 | 1.185150146 |
| SCAMP1 | Secretory Carrier Membrane Protein 1 | Protein Coding | 35 | GC05P078360 | 1.184467793 |
| WDR44 | WD Repeat Domain 44 | Protein Coding | 32 | GC0XP118346 | 1.184293032 |
| TPRN | Taperin | Protein Coding | 30 | GC09M137191 | 1.184261799 |
| IGHG3 | Immunoglobulin Heavy Constant Gamma 3 (G3m Marker) | Protein Coding | 26 | GC14M112408 | 1.184157848 |
| DPP8 | Dipeptidyl Peptidase 8 | Protein Coding | 35 | GC15M065442 | 1.184075832 |
| ELP5 | Elongator Acetyltransferase Complex Subunit 5 | Protein Coding | 30 | GC17P007251 | 1.183767915 |
| RPP21 | Ribonuclease P/MRP Subunit P21 | Protein Coding | 29 | GC06P030345 | 1.183253765 |
| NFYB | Nuclear Transcription Factor Y Subunit Beta | Protein Coding | 35 | GC12M104117 | 1.183237314 |
| IRGQ | Immunity Related GTPase Q | Protein Coding | 26 | GC19M043584 | 1.183205247 |
| LINC01436 | Long Intergenic Non-Protein Coding RNA 1436 | RNA Gene | 13 | GC21P036019 | 1.182973146 |
| MGRN1 | Mahogunin Ring Finger 1 | Protein Coding | 35 | GC16P010809 | 1.182720423 |
| TBL1Y | Transducin Beta Like 1 Y-Linked | Protein Coding | 29 | GC0YP006910 | 1.182692051 |
| SAMM50 | SAMM50 Sorting And Assembly Machinery Component | Protein Coding | 34 | GC22P043955 | 1.182672739 |
| CDKL3 | Cyclin Dependent Kinase Like 3 | Protein Coding | 35 | GC05M134242 | 1.182492018 |
| GBP2 | Guanylate Binding Protein 2 | Protein Coding | 34 | GC01M089106 | 1.18197608 |
| ADI1 | Acireductone Dioxygenase 1 | Protein Coding | 35 | GC02M003501 | 1.18181026 |
| ZKSCAN4 | Zinc Finger With KRAB And SCAN Domains 4 | Protein Coding | 31 | GC06M063348 | 1.181803346 |
| GCFC2 | GC-Rich Sequence DNA-Binding Factor 2 | Protein Coding | 30 | GC02M075652 | 1.181698561 |
| DCANP1 | Dendritic Cell Associated Nuclear Protein | Protein Coding | 25 | GC05M135460 | 1.181400061 |
| TBPL1 | TATA-Box Binding Protein Like 1 | Protein Coding | 35 | GC06P133952 | 1.181246877 |
| SASS6 | SAS-6 Centriolar Assembly Protein | Protein Coding | 32 | GC01M100083 | 1.181071043 |
| NUTM2D | NUT Family Member 2D | Protein Coding | 18 | GC10P087357 | 1.180960298 |
| WDR59 | WD Repeat Domain 59 | Protein Coding | 32 | GC16M074871 | 1.180934429 |
| SESN1 | Sestrin 1 | Protein Coding | 34 | GC06M108986 | 1.180925131 |
| ZBTB11 | Zinc Finger And BTB Domain Containing 11 | Protein Coding | 31 | GC03M101648 | 1.180678606 |
| RHOBTB1 | Rho Related BTB Domain Containing 1 | Protein Coding | 33 | GC10M060869 | 1.180487514 |
| HMGXB4 | HMG-Box Containing 4 | Protein Coding | 30 | GC22P035256 | 1.180309772 |
| SPIRE1 | Spire Type Actin Nucleation Factor 1 | Protein Coding | 30 | GC18M012446 | 1.180242777 |
| POLA2 | DNA Polymerase Alpha 2, Accessory Subunit | Protein Coding | 35 | GC11P069530 | 1.179913998 |

| | | | | | |
|---|---|---|---|---|---|
| TANGO2 | Transport And Golgi Organization 2 Homolog | Protein Coding | 31 | GC22P020017 | 1.179380178 |
| PSORS1C2 | Psoriasis Susceptibility 1 Candidate 2 | Protein Coding | 30 | GC06M031137 | 1.179286838 |
| MIR3065 | MicroRNA 3065 | RNA Gene | 14 | GC17P081125 | 1.179102659 |
| CMC1 | C-X9-C Motif Containing 1 | Protein Coding | 30 | GC03P028263 | 1.178810954 |
| ZMYM3 | Zinc Finger MYM-Type Containing 3 | Protein Coding | 32 | GC0XM071239 | 1.178660989 |
| KANSL3 | KAT8 Regulatory NSL Complex Subunit 3 | Protein Coding | 31 | GC02M096593 | 1.178660989 |
| B3GNT2 | UDP-GlcNAc:BetaGal Beta-1,3-N-Acetylglucosaminyltransferase 2 | Protein Coding | 35 | GC02P062196 | 1.178626657 |
| SLC52A2 | Solute Carrier Family 52 Member 2 | Protein Coding | 34 | GC08P144333 | 1.178351402 |
| PLAAT5 | Phospholipase A And Acyltransferase 5 | Protein Coding | 27 | GC11M087160 | 1.178236485 |
| MRPL2 | Mitochondrial Ribosomal Protein L2 | Protein Coding | 31 | GC06M043054 | 1.17816186 |
| LIMS2 | LIM Zinc Finger Domain Containing 2 | Protein Coding | 37 | GC02M127638 | 1.178154111 |
| MICU2 | Mitochondrial Calcium Uptake 2 | Protein Coding | 32 | GC13M021492 | 1.177923203 |
| GTPBP6 | GTP Binding Protein 6 (Putative) | Protein Coding | 25 | GC0XM000306 | 1.177642822 |
| ICE1 | Interactor Of Little Elongation Complex ELL Subunit 1 | Protein Coding | 25 | GC05P005421 | 1.177642822 |
| TMTC1 | Transmembrane O-Mannosyltransferase Targeting Cadherins 1 | Protein Coding | 32 | GC12M029500 | 1.177465677 |
| COX19 | Cytochrome C Oxidase Assembly Factor COX19 | Protein Coding | 28 | GC07M000898 | 1.177465677 |
| ETFBKMT | Electron Transfer Flavoprotein Subunit Beta Lysine Methyltransferase | Protein Coding | 27 | GC12P031651 | 1.177465677 |
| PYGO1 | Pygopus Family PHD Finger 1 | Protein Coding | 34 | GC15M055538 | 1.177031517 |
| LGI4 | Leucine Rich Repeat LGI Family Member 4 | Protein Coding | 36 | GC19M035124 | 1.176832438 |
| ERVK-7 | Endogenous Retrovirus Group K Member 7 | Protein Coding | 11 | GC01U903257 | 1.176694512 |
| TGM5 | Transglutaminase 5 | Protein Coding | 38 | GC15M043751 | 1.176692247 |
| FBXO43 | F-Box Protein 43 | Protein Coding | 31 | GC08M100133 | 1.176673532 |
| FBXW8 | F-Box And WD Repeat Domain Containing 8 | Protein Coding | 32 | GC12P116910 | 1.176645041 |
| TC2N | Tandem C2 Domains, Nuclear | Protein Coding | 31 | GC14M100414 | 1.176645041 |
| PPIL1 | Peptidylprolyl Isomerase Like 1 | Protein Coding | 38 | GC06M036854 | 1.176448345 |
| MIR513B | MicroRNA 513b | RNA Gene | 14 | GC0XM147199 | 1.176448345 |
| KIF3C | Kinesin Family Member 3C | Protein Coding | 34 | GC02M025926 | 1.175875187 |
| ARAP1-AS1 | ARAP1 Antisense RNA 1 | RNA Gene | 10 | GC11P072685 | 1.175800323 |
| TMEM88 | Transmembrane Protein 88 | Protein Coding | 28 | GC17P007854 | 1.175735235 |
| CASQ1 | Calsequestrin 1 | Protein Coding | 40 | GC01P160190 | 1.175481915 |
| JAM2 | Junctional Adhesion Molecule 2 | Protein Coding | 37 | GC21P025639 | 1.175401807 |
| TINAGL1 | Tubulointerstitial Nephritis Antigen Like 1 | Protein Coding | 34 | GC01P031576 | 1.175358534 |
| RCAN3 | RCAN Family Member 3 | Protein Coding | 33 | GC01P024502 | 1.17507875 |
| ADAMTS7 | ADAM Metallopeptidase With Thrombospondin Type 1 Motif 7 | Protein Coding | 36 | GC15M078759 | 1.174601555 |
| NRN1 | Neuritin 1 | Protein Coding | 35 | GC06M005997 | 1.174369931 |
| CYP2A7 | Cytochrome P450 Family 2 Subfamily A Member 7 | Protein Coding | 35 | GC19M040875 | 1.174309611 |
| FSD2 | Fibronectin Type III And SPRY Domain Containing 2 | Protein Coding | 31 | GC15M082755 | 1.174126148 |
| PRPF18 | Pre-MRNA Processing Factor 18 | Protein Coding | 30 | GC10P013586 | 1.174126148 |
| TESK2 | Testis Associated Actin Remodelling Kinase 2 | Protein Coding | 34 | GC01M045343 | 1.173984051 |
| POC1B | POC1 Centriolar Protein B | Protein Coding | 33 | GC12M089419 | 1.173947096 |
| MATN3 | Matrilin 3 | Protein Coding | 39 | GC02M019992 | 1.173761487 |
| ST13P4 | ST13, Hsp70 Interacting Protein Pseudogene 4 | Pseudogene | 17 | GC13P050172 | 1.173734426 |
| ZNF142 | Zinc Finger Protein 142 | Protein Coding | 31 | GC02M218637 | 1.173634529 |
| UBN2 | Ubinuclein 2 | Protein Coding | 29 | GC07P139230 | 1.17357707 |
| PDXP | Pyridoxal Phosphatase | Protein Coding | 34 | GC22P037658 | 1.173471928 |
| SLC35B2 | Solute Carrier Family 35 Member B2 | Protein Coding | 36 | GC06M044254 | 1.173227072 |
| AJAP1 | Adherens Junctions Associated Protein 1 | Protein Coding | 30 | GC01P004654 | 1.172843456 |
| ULK2 | Unc-51 Like Autophagy Activating Kinase 2 | Protein Coding | 36 | GC17M025250 | 1.172412634 |
| RNF212 | Ring Finger Protein 212 | Protein Coding | 31 | GC04M001443 | 1.171907306 |
| ERVK-19 | Endogenous Retrovirus Group K Member 19, Envelope | Protein Coding | 7 | GC19U901633 | 1.170678616 |
| SULT4A1 | Sulfotransferase Family 4A Member 1 | Protein Coding | 36 | GC22M043824 | 1.170419931 |
| IRAK2 | Interleukin 1 Receptor Associated Kinase 2 | Protein Coding | 36 | GC03P012250 | 1.170211077 |
| H3C15 | H3 Clustered Histone 15 | Protein Coding | 25 | GC01P150137 | 1.170137286 |
| LRRK1 | Leucine Rich Repeat Kinase 1 | Protein Coding | 35 | GC15P100919 | 1.169912338 |
| RHOT2 | Ras Homolog Family Member T2 | Protein Coding | 34 | GC16P010645 | 1.16987288 |
| YKT6 | YKT6 V-SNARE Homolog | Protein Coding | 35 | GC07P044200 | 1.169400454 |
| KSR2 | Kinase Suppressor Of Ras 2 | Protein Coding | 34 | GC12M117453 | 1.169396758 |
| KIF13A | Kinesin Family Member 13A | Protein Coding | 37 | GC06M017759 | 1.169273615 |
| PLXNA4 | Plexin A4 | Protein Coding | 35 | GC07M132123 | 1.169093132 |
| DR1 | Down-Regulator Of Transcription 1 | Protein Coding | 34 | GC01P093345 | 1.16862464 |
| TRHR | Thyrotropin Releasing Hormone Receptor | Protein Coding | 42 | GC08P109084 | 1.168318868 |
| MRPL39 | Mitochondrial Ribosomal Protein L39 | Protein Coding | 32 | GC21M025585 | 1.168282509 |
| SLC16A9 | Solute Carrier Family 16 Member 9 | Protein Coding | 35 | GC10M059650 | 1.168270826 |
| DCAF5 | DDB1 And CUL4 Associated Factor 5 | Protein Coding | 30 | GC14M069050 | 1.168249488 |
| MIR618 | MicroRNA 618 | RNA Gene | 14 | GC12M080935 | 1.167101622 |
| PRDM15 | PR/SET Domain 15 | Protein Coding | 30 | GC21M041798 | 1.166935205 |
| TUFT1 | Tuftelin 1 | Protein Coding | 35 | GC01P151513 | 1.166693926 |
| ZNF207 | Zinc Finger Protein 207 | Protein Coding | 32 | GC17P052658 | 1.165980816 |
| MIR1976 | MicroRNA 1976 | RNA Gene | 14 | GC01P026554 | 1.165930629 |
| FBXL6 | F-Box And Leucine Rich Repeat Protein 6 | Protein Coding | 30 | GC08M144355 | 1.165845394 |
| GPR3 | G Protein-Coupled Receptor 3 | Protein Coding | 35 | GC01P027635 | 1.165600538 |
| TTLL4 | Tubulin Tyrosine Ligase Like 4 | Protein Coding | 31 | GC02P218710 | 1.165281057 |
| SLC7A10 | Solute Carrier Family 7 Member 10 | Protein Coding | 34 | GC19M033208 | 1.165267944 |
| HHLA2 | HERV-H LTR-Associating 2 | Protein Coding | 32 | GC03P108296 | 1.165228009 |
| LINC00641 | Long Intergenic Non-Protein Coding RNA 641 | RNA Gene | 13 | GC14M022212 | 1.164476633 |
| HADH | Hydroxyacyl-CoA Dehydrogenase | Protein Coding | 42 | GC04P107989 | 1.164451122 |
| JADE2 | Jade Family PHD Finger 2 | Protein Coding | 28 | GC05P134524 | 1.163907051 |
| OPLAH | 5-Oxoprolinase, ATP-Hydrolysing | Protein Coding | 38 | GC08M144051 | 1.163904071 |
| SMAGP | Small Cell Adhesion Glycoprotein | Protein Coding | 30 | GC12M051244 | 1.163297653 |
| MGAT1 | Alpha-1,3-Mannosyl-Glycoprotein 2-Beta-N-Acetylglucosaminyltransferase | Protein Coding | 39 | GC05M181693 | 1.162916899 |
| DNAJC16 | DnaJ Heat Shock Protein Family (Hsp40) Member C16 | Protein Coding | 28 | GC01P015526 | 1.162634015 |
| IL36RN | Interleukin 36 Receptor Antagonist | Protein Coding | 36 | GC02P121640 | 1.162540317 |
| CDKL1 | Cyclin Dependent Kinase Like 1 | Protein Coding | 36 | GC14M050330 | 1.162533998 |
| GSTK1 | Glutathione S-Transferase Kappa 1 | Protein Coding | 35 | GC07P147999 | 1.162426472 |
| FBXL12 | F-Box And Leucine Rich Repeat Protein 12 | Protein Coding | 31 | GC19M009810 | 1.162127972 |
| ZMIZ1-AS1 | ZMIZ1 Antisense RNA 1 | RNA Gene | 15 | GC10M078943 | 1.162076712 |
| TRIM15 | Tripartite Motif Containing 15 | Protein Coding | 30 | GC06P080319 | 1.161823392 |
| SAT2 | Spermidine/Spermine N1-Acetyltransferase Family Member 2 | Protein Coding | 34 | GC17M007626 | 1.161757708 |
| ATP13A3 | ATPase 13A3 | Protein Coding | 35 | GC03M194402 | 1.161491752 |
| CYP39A1 | Cytochrome P450 Family 39 Subfamily A Member 1 | Protein Coding | 36 | GC06M046549 | 1.161313534 |
| ANKRD49 | Ankyrin Repeat Domain 49 | Protein Coding | 33 | GC11P094493 | 1.161196947 |
| CHMP4C | Charged Multivesicular Body Protein 4C | Protein Coding | 29 | GC08P081732 | 1.160663128 |
| EPIC1 | Epigenetically Induced MYC Interacting LncRNA 1 | RNA Gene | 10 | GC22P047632 | 1.160595417 |
| MRTFA-AS1 | MRTFA Antisense RNA 1 | RNA Gene | 11 | GC22P040523 | 1.160557985 |
| SGSM3-AS1 | SGSM3 Antisense RNA 1 | RNA Gene | 9 | GC22M057103 | 1.160557985 |

| | | | | | |
|---|---|---|---|---|---|
| SMARCE1P4 | SWI/SNF Related, Matrix Associated, Actin Dependent Regulator Of Chromatin, Subfamily E, Member 1 Pseudogene 4 | Pseudogene | 5 | GC08P037095 | 1.160557985 |
| piR-53431-462 | | RNA Gene | 5 | GC22M056947 | 1.160557985 |
| HSALNG0093256 | | RNA Gene | 4 | GC12P095624 | 1.160557985 |
| piR-42694-104 | | RNA Gene | 4 | GC22M056799 | 1.160557985 |
| IFT52 | Intraflagellar Transport 52 | Protein Coding | 31 | GC20P043590 | 1.160415411 |
| FNTA | Farnesyltransferase, CAAX Box, Alpha | Protein Coding | 39 | GC08P043034 | 1.160195947 |
| IMMP1L | Inner Mitochondrial Membrane Peptidase Subunit 1 | Protein Coding | 33 | GC11M031432 | 1.159916162 |
| ZNF503 | Zinc Finger Protein 503 | Protein Coding | 31 | GC10M075280 | 1.159819961 |
| ATP5PB | ATP Synthase Peripheral Stalk-Membrane Subunit B | Protein Coding | 28 | GC01P111449 | 1.159169912 |
| LOC102724594 | U2 Small Nuclear RNA Auxiliary Factor 1 Like 5 | Protein Coding | 5 | GC21M006484 | 1.159158945 |
| CHMP4B | Charged Multivesicular Body Protein 4B | Protein Coding | 38 | GC20P034015 | 1.158970714 |
| GZF1 | GDNF Inducible Zinc Finger Protein 1 | Protein Coding | 33 | GC20P023361 | 1.158475399 |
| BAZ2A | Bromodomain Adjacent To Zinc Finger Domain 2A | Protein Coding | 35 | GC12M056595 | 1.158225775 |
| TCEAL4 | Transcription Elongation Factor A Like 4 | Protein Coding | 29 | GC0XP103576 | 1.158185005 |
| VCX3B | Variable Charge X-Linked 3B | Protein Coding | 23 | GC0XP008464 | 1.157576919 |
| GAPVD1 | GTPase Activating Protein And VPS9 Domains 1 | Protein Coding | 36 | GC09P125261 | 1.157472014 |
| MIR3127 | MicroRNA 3127 | RNA Gene | 14 | GC02P096798 | 1.157332778 |
| GPRASP1 | G Protein-Coupled Receptor Associated Sorting Protein 1 | Protein Coding | 30 | GC0XP102651 | 1.15695858 |
| ENSG00000226380 | Novel Transcript | Uncategorized | 9 | GC07M130984 | 1.156472921 |
| CCDC59 | Coiled-Coil Domain Containing 59 | Protein Coding | 31 | GC12M082223 | 1.156310916 |
| MIR3614 | MicroRNA 3614 | RNA Gene | 13 | GC17M056891 | 1.156280279 |
| CTTNBP2NL | CTTNBP2 N-Terminal Like | Protein Coding | 30 | GC01P112396 | 1.155803204 |
| MAIP1 | Matrix AAA Peptidase Interacting Protein 1 | Protein Coding | 26 | GC02P199956 | 1.155697107 |
| ARMC6 | Armadillo Repeat Containing 6 | Protein Coding | 30 | GC19P063130 | 1.155533791 |
| MIR4665 | MicroRNA 4665 | RNA Gene | 11 | GC09P006435 | 1.155276299 |
| PLGLB1 | Plasminogen Like B1 | Protein Coding | 30 | GC02M087002 | 1.15521884 |
| SPIN2A | Spindlin Family Member 2A | Protein Coding | 26 | GC0XM057134 | 1.154955506 |
| STRIP2 | Striatin Interacting Protein 2 | Protein Coding | 30 | GC07P131689 | 1.154835463 |
| MYBPC1 | Myosin Binding Protein C1 | Protein Coding | 39 | GC12P101568 | 1.154695272 |
| RDM1 | RAD52 Motif Containing 1 | Protein Coding | 31 | GC17M035918 | 1.154659867 |
| KCTD6 | Potassium Channel Tetramerization Domain Containing 6 | Protein Coding | 30 | GC03P058494 | 1.153909922 |
| ADSS2 | Adenylosuccinate Synthase 2 | Protein Coding | 34 | GC01M244410 | 1.153838396 |
| KCNRG | Potassium Channel Regulator | Protein Coding | 31 | GC13P050015 | 1.153614759 |
| CNTD1 | Cyclin N-Terminal Domain Containing 1 | Protein Coding | 29 | GC17P042798 | 1.153270483 |
| FIGNL1 | Fidgetin Like 1 | Protein Coding | 35 | GC07M050444 | 1.15315032 |
| UQCRQ | Ubiquinol-Cytochrome C Reductase Complex III Subunit VII | Protein Coding | 36 | GC05P132866 | 1.152416229 |
| ECH1 | Enoyl-CoA Hydratase 1 | Protein Coding | 36 | GC19M038815 | 1.15240097 |
| NOL6 | Nucleolar Protein 6 | Protein Coding | 30 | GC09M033451 | 1.152117491 |
| GNPNAT1 | Glucosamine-Phosphate N-Acetyltransferase 1 | Protein Coding | 34 | GC14M052775 | 1.152090549 |
| ARHGEF15 | Rho Guanine Nucleotide Exchange Factor 15 | Protein Coding | 34 | GC17P008310 | 1.151917696 |
| DMXL1 | Dmx Like 1 | Protein Coding | 30 | GC05P119037 | 1.151706219 |
| NMUR2 | Neuromedin U Receptor 2 | Protein Coding | 36 | GC05M152391 | 1.151305676 |
| USP47 | Ubiquitin Specific Peptidase 47 | Protein Coding | 34 | GC11P011842 | 1.150757194 |
| IL18BP | Interleukin 18 Binding Protein | Protein Coding | 37 | GC11P071998 | 1.150524855 |
| RC3H1 | Ring Finger And CCCH-Type Domains 1 | Protein Coding | 32 | GC01M173931 | 1.150523067 |
| SLC28A2 | Solute Carrier Family 28 Member 2 | Protein Coding | 39 | GC15P045252 | 1.150473833 |
| FCAR | Fc Alpha Receptor | Protein Coding | 38 | GC19P064348 | 1.15032506 |
| STAMBP | STAM Binding Protein | Protein Coding | 40 | GC02P073828 | 1.15020299 |
| JTB | Jumping Translocation Breakpoint | Protein Coding | 31 | GC01M153974 | 1.149805546 |
| ZNF346 | Zinc Finger Protein 346 | Protein Coding | 31 | GC05P177022 | 1.149658918 |
| CLEC10A | C-Type Lectin Domain Containing 10A | Protein Coding | 35 | GC17M007074 | 1.149459481 |
| MIR3174 | MicroRNA 3174 | RNA Gene | 15 | GC15P090006 | 1.149412632 |
| TANC2 | Tetratricopeptide Repeat, Ankyrin Repeat And Coiled-Coil Containing 2 | Protein Coding | 33 | GC17P062967 | 1.149352789 |
| SF3B5 | Splicing Factor 3b Subunit 5 | Protein Coding | 30 | GC06M144094 | 1.149191141 |
| MRPL37 | Mitochondrial Ribosomal Protein L37 | Protein Coding | 31 | GC01P054185 | 1.149188876 |
| FBXL16 | F-Box And Leucine Rich Repeat Protein 16 | Protein Coding | 30 | GC16M006614 | 1.149013996 |
| NSFL1C | NSFL1 Cofactor | Protein Coding | 35 | GC20M001442 | 1.148947477 |
| IFIT2 | Interferon Induced Protein With Tetratricopeptide Repeats 2 | Protein Coding | 34 | GC10P091594 | 1.148940563 |
| HDHD5 | Haloacid Dehalogenase Like Hydrolase Domain Containing 5 | Protein Coding | 26 | GC22M017186 | 1.148787379 |
| PGM2 | Phosphoglucomutase 2 | Protein Coding | 34 | GC04P038038 | 1.148776412 |
| CCDC127 | Coiled-Coil Domain Containing 127 | Protein Coding | 28 | GC05M000204 | 1.148415804 |
| CUZD1 | CUB And Zona Pellucida Like Domains 1 | Protein Coding | 32 | GC10M122833 | 1.1483078 |
| SLC48A1 | Solute Carrier Family 48 Member 1 | Protein Coding | 30 | GC12P047753 | 1.148211122 |
| SH2D3C | SH2 Domain Containing 3C | Protein Coding | 34 | GC09M127738 | 1.148093581 |
| GGT5 | Gamma-Glutamyltransferase 5 | Protein Coding | 35 | GC22M024219 | 1.147927523 |
| CGN | Cingulin | Protein Coding | 34 | GC01P151483 | 1.147814393 |
| DUSP23 | Dual Specificity Phosphatase 23 | Protein Coding | 34 | GC01P159750 | 1.14773047 |
| CCDC180 | Coiled-Coil Domain Containing 180 | Protein Coding | 29 | GC09P097307 | 1.147416234 |
| PPP4R1 | Protein Phosphatase 4 Regulatory Subunit 1 | Protein Coding | 34 | GC18M009546 | 1.147408962 |
| TAF11 | TATA-Box Binding Protein Associated Factor 11 | Protein Coding | 35 | GC06M063745 | 1.14721632 |
| ADPGK-AS1 | ADPGK Antisense RNA 1 | RNA Gene | 12 | GC15P114718 | 1.147190928 |
| HSD3B7 | Hydroxy-Delta-5-Steroid Dehydrogenase, 3 Beta- And Steroid Delta-Isomerase 7 | Protein Coding | 37 | GC16P030985 | 1.147103548 |
| CPN2 | Carboxypeptidase N Subunit 2 | Protein Coding | 36 | GC03M194339 | 1.146960974 |
| MIR3156-3 | MicroRNA 3156-3 | RNA Gene | 11 | GC21M013406 | 1.146960974 |
| PPP1R36 | Protein Phosphatase 1 Regulatory Subunit 36 | Protein Coding | 28 | GC14P064549 | 1.146661758 |
| PITPNB | Phosphatidylinositol Transfer Protein Beta | Protein Coding | 36 | GC22M027851 | 1.146240473 |
| FAM201A | Family With Sequence Similarity 201 Member A | RNA Gene | 18 | GC09P038620 | 1.146001935 |
| MIR4715 | MicroRNA 4715 | RNA Gene | 13 | GC15M025848 | 1.145856857 |
| YJU2 | YJU2 Splicing Factor Homolog | Protein Coding | 23 | GC19P004253 | 1.145602465 |
| ATP5F1D | ATP Synthase F1 Subunit Delta | Protein Coding | 33 | GC19P002569 | 1.145549059 |
| URB1 | URB1 Ribosome Biogenesis Homolog | Protein Coding | 27 | GC21M032312 | 1.145549059 |
| IRGC | Immunity Related GTPase Cinema | Protein Coding | 33 | GC19P043716 | 1.145436049 |
| GLIDR | Glioblastoma Down-Regulated RNA | RNA Gene | 13 | GC09M039708 | 1.145401001 |
| ZNF521 | Zinc Finger Protein 521 | Protein Coding | 34 | GC18M025061 | 1.145354152 |
| MIR3658 | MicroRNA 3658 | RNA Gene | 9 | GC01P165907 | 1.14533186 |
| HCG4B | HLA Complex Group 4B | RNA Gene | 14 | GC06M063503 | 1.145217419 |
| MICE | MHC Class I Polypeptide-Related Sequence E (Pseudogene) | Pseudogene | 9 | GC06M063489 | 1.145217419 |
| ENSG00000285106 | Novel Transcript | Uncategorized | 8 | GC07M130793 | 1.145217419 |
| RNU6-1010P | RNA, U6 Small Nuclear 1010, Pseudogene | Pseudogene | 7 | GC07M131054 | 1.145217419 |
| PKDCC | Protein Kinase Domain Containing, Cytoplasmic | Protein Coding | 34 | GC02P042049 | 1.144923449 |
| KLHL18 | Kelch Like Family Member 18 | Protein Coding | 30 | GC03P047282 | 1.144923449 |
| GMCL1 | Germ Cell-Less 1, Spermatogenesis Associated | Protein Coding | 29 | GC02P069829 | 1.144923449 |
| CNNM3 | Cyclin And CBS Domain Divalent Metal Cation Transport Mediator 3 | Protein Coding | 34 | GC02P096815 | 1.144899011 |
| NEK4 | NIMA Related Kinase 4 | Protein Coding | 37 | GC03M052708 | 1.144278884 |

| | | | | | |
|---|---|---|---|---|---|
| APLF | Aprataxin And PNKP Like Factor | Protein Coding | 31 | GC02P068467 | 1.144237518 |
| ZFYVE21 | Zinc Finger FYVE-Type Containing 21 | Protein Coding | 31 | GC14P103715 | 1.143806458 |
| SDF2 | Stromal Cell Derived Factor 2 | Protein Coding | 33 | GC17M034577 | 1.143801689 |
| SCPEP1 | Serine Carboxypeptidase 1 | Protein Coding | 34 | GC17P056978 | 1.143576622 |
| TLE4 | TLE Family Member 4, Transcriptional Corepressor | Protein Coding | 38 | GC09P079571 | 1.143304467 |
| MYT1L | Myelin Transcription Factor 1 Like | Protein Coding | 38 | GC02M001789 | 1.142979264 |
| DDX3Y | DEAD-Box Helicase 3 Y-Linked | Protein Coding | 34 | GC0YP012903 | 1.142780304 |
| SKAP1-AS2 | SKAP1 Antisense RNA 2 | RNA Gene | 12 | GC17P053424 | 1.142662764 |
| SERPINA10 | Serpin Family A Member 10 | Protein Coding | 36 | GC14M094280 | 1.142403126 |
| TMPRSS13 | Transmembrane Serine Protease 13 | Protein Coding | 34 | GC11M117900 | 1.14239049 |
| TSEN15 | TRNA Splicing Endonuclease Subunit 15 | Protein Coding | 36 | GC01P184056 | 1.142354965 |
| SGCZ | Sarcoglycan Zeta | Protein Coding | 31 | GC08M014089 | 1.142239571 |
| FBXO9 | F-Box Protein 9 | Protein Coding | 32 | GC06P080708 | 1.141927361 |
| VPS13D | Vacuolar Protein Sorting 13 Homolog D | Protein Coding | 31 | GC01P012231 | 1.14188695 |
| FIGLA | Folliculogenesis Specific BHLH Transcription Factor | Protein Coding | 32 | GC02M070741 | 1.141785383 |
| MYL5 | Myosin Light Chain 5 | Protein Coding | 32 | GC04P000673 | 1.141177058 |
| CLEC14A | C-Type Lectin Domain Containing 14A | Protein Coding | 31 | GC14M038254 | 1.141137362 |
| SEPTIN6 | Septin 6 | Protein Coding | 30 | GC0XM119616 | 1.141048193 |
| FAM189A2 | Family With Sequence Similarity 189 Member A2 | Protein Coding | 30 | GC09P069324 | 1.140562177 |
| COL16A1 | Collagen Type XVI Alpha 1 Chain | Protein Coding | 36 | GC01M031653 | 1.140481472 |
| CDH22 | Cadherin 22 | Protein Coding | 35 | GC20M046173 | 1.140303135 |
| SKAP1 | Src Kinase Associated Phosphoprotein 1 | Protein Coding | 36 | GC17M048133 | 1.139906287 |
| P3H4 | Prolyl 3-Hydroxylase Family Member 4 (Inactive) | Protein Coding | 30 | GC17M041801 | 1.139532447 |
| SEC24C | SEC24 Homolog C, COPII Coat Complex Component | Protein Coding | 39 | GC10P073744 | 1.139427304 |
| TJP3 | Tight Junction Protein 3 | Protein Coding | 34 | GC19P003708 | 1.13942039 |
| MUC15 | Mucin 15, Cell Surface Associated | Protein Coding | 31 | GC11M026537 | 1.139304042 |
| SLC4A3 | Solute Carrier Family 4 Member 3 | Protein Coding | 37 | GC02P219627 | 1.139294147 |
| MIR3120 | MicroRNA 3120 | RNA Gene | 15 | GC01P172138 | 1.139186859 |
| FAM53A | Family With Sequence Similarity 53 Member A | Protein Coding | 27 | GC04M001610 | 1.13900423 |
| MACROD2 | Mono-ADP Ribosylhydrolase 2 | Protein Coding | 32 | GC20P013925 | 1.138014197 |
| MIR621 | MicroRNA 621 | RNA Gene | 13 | GC13P040810 | 1.137820601 |
| LOC108942766 | NANOG 5' Regulatory Region | Biological Region | 2 | GC12P020459 | 1.137688041 |
| SAMD14 | Sterile Alpha Motif Domain Containing 14 | Protein Coding | 30 | GC17M050110 | 1.137478232 |
| ETV3 | ETS Variant Transcription Factor 3 | Protein Coding | 31 | GC01M157121 | 1.137295961 |
| SPCS3 | Signal Peptidase Complex Subunit 3 | Protein Coding | 32 | GC04P176319 | 1.137082577 |
| SLC7A2 | Solute Carrier Family 7 Member 2 | Protein Coding | 38 | GC08P017497 | 1.136489511 |
| MIR2115 | MicroRNA 2115 | RNA Gene | 13 | GC03M048316 | 1.136237025 |
| ZNF608 | Zinc Finger Protein 608 | Protein Coding | 32 | GC05M124636 | 1.136117458 |
| ATP8B3 | ATPase Phospholipid Transporting 8B3 | Protein Coding | 36 | GC19M004937 | 1.136059642 |
| KCNS1 | Potassium Voltage-Gated Channel Modifier Subfamily S Member 1 | Protein Coding | 36 | GC20M045099 | 1.136028409 |
| ZNF160 | Zinc Finger Protein 160 | Protein Coding | 31 | GC19M053066 | 1.135846376 |
| GABPB2 | GA Binding Protein Transcription Factor Subunit Beta 2 | Protein Coding | 31 | GC01P151070 | 1.135807276 |
| NAE1 | NEDD8 Activating Enzyme E1 Subunit 1 | Protein Coding | 36 | GC16M066803 | 1.13577342 |
| SGF29 | SAGA Complex Associated Factor 29 | Protein Coding | 27 | GC16P040040 | 1.135694742 |
| MIR3666 | MicroRNA 3666 | RNA Gene | 11 | GC07P114653 | 1.135394812 |
| TEAD3 | TEA Domain Transcription Factor 3 | Protein Coding | 35 | GC06M063749 | 1.135320067 |
| MIR612 | MicroRNA 612 | RNA Gene | 17 | GC11P069504 | 1.135005713 |
| SCNN1D | Sodium Channel Epithelial 1 Subunit Delta | Protein Coding | 39 | GC01P001280 | 1.134716988 |
| UBXN6 | UBX Domain Protein 6 | Protein Coding | 33 | GC19M004444 | 1.134343505 |
| TMED2 | Transmembrane P24 Trafficking Protein 2 | Protein Coding | 32 | GC12P123584 | 1.133791089 |
| TTC17 | Tetratricopeptide Repeat Domain 17 | Protein Coding | 30 | GC11P043380 | 1.133791089 |
| SELENOI | Selenoprotein I | Protein Coding | 28 | GC02P026310 | 1.133306861 |
| GPR89B | G Protein-Coupled Receptor 89B | Protein Coding | 29 | GC01P147928 | 1.133281827 |
| SLC7A9 | Solute Carrier Family 7 Member 9 | Protein Coding | 40 | GC19M032830 | 1.13310802 |
| RNF121 | Ring Finger Protein 121 | Protein Coding | 28 | GC11P071928 | 1.132944345 |
| MMP24 | Matrix Metallopeptidase 24 | Protein Coding | 39 | GC20P035226 | 1.132809639 |
| RIC8A | RIC8 Guanine Nucleotide Exchange Factor A | Protein Coding | 32 | GC11P000207 | 1.132735729 |
| GPR108 | G Protein-Coupled Receptor 108 | Protein Coding | 34 | GC19M006729 | 1.131502509 |
| RAB37 | RAB37, Member RAS Oncogene Family | Protein Coding | 34 | GC17P074671 | 1.131164908 |
| ZNF776 | Zinc Finger Protein 776 | Protein Coding | 28 | GC19P057746 | 1.130699039 |
| TRIM2 | Tripartite Motif Containing 2 | Protein Coding | 37 | GC04P153152 | 1.130560279 |
| HAO2 | Hydroxyacid Oxidase 2 | Protein Coding | 34 | GC01P119368 | 1.130316496 |
| IL5RA | Interleukin 5 Receptor Subunit Alpha | Protein Coding | 41 | GC03M003066 | 1.130069971 |
| STAG1 | Stromal Antigen 1 | Protein Coding | 38 | GC03M136336 | 1.129972696 |
| WDR70 | WD Repeat Domain 70 | Protein Coding | 28 | GC05P037579 | 1.129943132 |
| CREM | CAMP Responsive Element Modulator | Protein Coding | 37 | GC10P035126 | 1.129853964 |
| GLT8D1 | Glycosyltransferase 8 Domain Containing 1 | Protein Coding | 34 | GC03M052694 | 1.129828334 |
| RASSF8 | Ras Association Domain Family Member 8 | Protein Coding | 32 | GC12P025959 | 1.129634738 |
| KIR2DS4 | Killer Cell Immunoglobulin Like Receptor, Two Ig Domains And Short Cytoplasmic Tail 4 | Protein Coding | 31 | GC19P064346 | 1.129225373 |
| VWA8 | Von Willebrand Factor A Domain Containing 8 | Protein Coding | 28 | GC13M041569 | 1.129164219 |
| B4GALT5 | Beta-1,4-Galactosyltransferase 5 | Protein Coding | 35 | GC20M049632 | 1.128869653 |
| HERC4 | HECT And RLD Domain Containing E3 Ubiquitin Protein Ligase 4 | Protein Coding | 32 | GC10M067921 | 1.128629804 |
| PPP6R1 | Protein Phosphatase 6 Regulatory Subunit 1 | Protein Coding | 31 | GC19M055229 | 1.128513336 |
| PRAG1 | PEAK1 Related, Kinase-Activating Pseudokinase 1 | Protein Coding | 27 | GC08M008318 | 1.128513336 |
| HSPA12A | Heat Shock Protein Family A (Hsp70) Member 12A | Protein Coding | 34 | GC10M116671 | 1.128461242 |
| ACTR1B | Actin Related Protein 1B | Protein Coding | 34 | GC02M097655 | 1.127881527 |
| ABI2 | Abl Interactor 2 | Protein Coding | 35 | GC02P203327 | 1.127874374 |
| P2RY6 | Pyrimidinergic Receptor P2Y6 | Protein Coding | 39 | GC11P073264 | 1.127783775 |
| DNAJC8 | DnaJ Heat Shock Protein Family (Hsp40) Member C8 | Protein Coding | 31 | GC01M028199 | 1.127775192 |
| SARAF | Store-Operated Calcium Entry Associated Regulatory Factor | Protein Coding | 28 | GC08M030063 | 1.127774954 |
| LINC02677 | Long Intergenic Non-Protein Coding RNA 2677 | RNA Gene | 9 | GC10M005598 | 1.127547979 |
| NDUFAF7 | NADH:Ubiquinone Oxidoreductase Complex Assembly Factor 7 | Protein Coding | 30 | GC02P037231 | 1.127359152 |
| C11orf21 | Chromosome 11 Open Reading Frame 21 | Protein Coding | 25 | GC11M002295 | 1.126965761 |
| CDC42SE1 | CDC42 Small Effector 1 | Protein Coding | 30 | GC01M151050 | 1.126819134 |
| CELF3 | CUGBP Elav-Like Family Member 3 | Protein Coding | 31 | GC01M151700 | 1.125860214 |
| USP34 | Ubiquitin Specific Peptidase 34 | Protein Coding | 35 | GC02M061187 | 1.125814676 |
| GPKOW | G-Patch Domain And KOW Motifs | Protein Coding | 31 | GC0XM049113 | 1.125777006 |
| FBXL14 | F-Box And Leucine Rich Repeat Protein 14 | Protein Coding | 28 | GC12M001606 | 1.124980927 |
| TAF1B | TATA-Box Binding Protein Associated Factor, RNA Polymerase I Subunit B | Protein Coding | 31 | GC02P009834 | 1.124593019 |
| NUP50 | Nucleoporin 50 | Protein Coding | 35 | GC22P045163 | 1.12458992 |
| DYNC1LI2 | Dynein Cytoplasmic 1 Light Intermediate Chain 2 | Protein Coding | 32 | GC16M066754 | 1.124163032 |
| CST2 | Cystatin SA | Protein Coding | 31 | GC20M023805 | 1.123930216 |
| INO80B | INO80 Complex Subunit B | Protein Coding | 28 | GC02P074456 | 1.123723388 |
| ACAD11 | Acyl-CoA Dehydrogenase Family Member 11 | Protein Coding | 33 | GC03M132559 | 1.123297691 |

| | | | | | |
|---|---|---|---|---|---|
| ISG20L2 | Interferon Stimulated Exonuclease Gene 20 Like 2 | Protein Coding | 32 | GC01M156723 | 1.123193979 |
| PKD3 | Polycystic Kidney Disease 3 (Autosomal Dominant) | Genetic Locus | 2 | GC02U990077 | 1.123107076 |
| TAS2R14 | Taste 2 Receptor Member 14 | Protein Coding | 31 | GC12M010937 | 1.122816086 |
| INTS4 | Integrator Complex Subunit 4 | Protein Coding | 32 | GC11M087726 | 1.122395515 |
| CISD1 | CDGSH Iron Sulfur Domain 1 | Protein Coding | 34 | GC10P058269 | 1.122240543 |
| POLR2J | RNA Polymerase II Subunit J | Protein Coding | 36 | GC07M102473 | 1.121958256 |
| PDE9A | Phosphodiesterase 9A | Protein Coding | 38 | GC21P042653 | 1.121876478 |
| SCGN | Secretagogin, EF-Hand Calcium Binding Protein | Protein Coding | 34 | GC06P025652 | 1.121644974 |
| TMEM237 | Transmembrane Protein 237 | Protein Coding | 32 | GC02M201620 | 1.121341705 |
| ANGPTL1 | Angiopoietin Like 1 | Protein Coding | 36 | GC01M178818 | 1.12103641 |
| RBM42 | RNA Binding Motif Protein 42 | Protein Coding | 29 | GC19P063453 | 1.120852113 |
| HMGXB3 | HMG-Box Containing 3 | Protein Coding | 30 | GC05P150000 | 1.120802879 |
| KCNK4 | Potassium Two Pore Domain Channel Subfamily K Member 4 | Protein Coding | 37 | GC11P064356 | 1.120778322 |
| MIR3064 | MicroRNA 3064 | RNA Gene | 13 | GC17M064500 | 1.120650053 |
| MIR762 | MicroRNA 762 | RNA Gene | 14 | GC16P030893 | 1.120294333 |
| WDR90 | WD Repeat Domain 90 | Protein Coding | 29 | GC16P010643 | 1.120089054 |
| CPXM2 | Carboxypeptidase X, M14 Family Member 2 | Protein Coding | 34 | GC10M123710 | 1.119675279 |
| YIF1A | Yip1 Interacting Factor Homolog A, Membrane Trafficking Protein | Protein Coding | 30 | GC11M087319 | 1.119501233 |
| MIR3922 | MicroRNA 3922 | RNA Gene | 12 | GC12P104591 | 1.11928761 |
| TMOD4 | Tropomodulin 4 | Protein Coding | 34 | GC01M151169 | 1.118783355 |
| VCX3A | Variable Charge X-Linked 3A | Protein Coding | 27 | GC0XM006533 | 1.118695498 |
| VCX2 | Variable Charge X-Linked 2 | Protein Coding | 26 | GC0XM008169 | 1.118695498 |
| ASH1L-AS1 | ASH1L Antisense RNA 1 | RNA Gene | 13 | GC01P155561 | 1.118603706 |
| CHST15 | Carbohydrate Sulfotransferase 15 | Protein Coding | 36 | GC10M124006 | 1.11837256 |
| ERICH6 | Glutamate Rich 6 | Protein Coding | 23 | GC03M150659 | 1.118206739 |
| DLK2 | Delta Like Non-Canonical Notch Ligand 2 | Protein Coding | 32 | GC06M043450 | 1.118076086 |
| ZNF821 | Zinc Finger Protein 821 | Protein Coding | 31 | GC16M071859 | 1.117902517 |
| SCD5 | Stearoyl-CoA Desaturase 5 | Protein Coding | 37 | GC04M082629 | 1.11747241 |
| TMEM87B | Transmembrane Protein 87B | Protein Coding | 32 | GC02P122425 | 1.117397547 |
| GAS1RR | GAS1 Adjacent Regulatory RNA | RNA Gene | 12 | GC09P086949 | 1.117380857 |
| CWF19L2 | CWF19 Like Cell Cycle Control Factor 2 | Protein Coding | 27 | GC11M107326 | 1.116883755 |
| NDUFB10 | NADH:Ubiquinone Oxidoreductase Subunit B10 | Protein Coding | 39 | GC16P010708 | 1.11665225 |
| MIR4653 | MicroRNA 4653 | RNA Gene | 12 | GC07P101159 | 1.116123438 |
| SAMD1 | Sterile Alpha Motif Domain Containing 1 | Protein Coding | 25 | GC19M014087 | 1.115616322 |
| MIR4317 | MicroRNA 4317 | RNA Gene | 11 | GC18M006374 | 1.115288854 |
| MED29 | Mediator Complex Subunit 29 | Protein Coding | 30 | GC19P063556 | 1.115190864 |
| RHOF | Ras Homolog Family Member F, Filopodia Associated | Protein Coding | 33 | GC12M121777 | 1.115035057 |
| CMTM8 | CKLF Like MARVEL Transmembrane Domain Containing 8 | Protein Coding | 33 | GC03P032238 | 1.114961863 |
| ARHGAP39 | Rho GTPase Activating Protein 39 | Protein Coding | 32 | GC08M144529 | 1.114938259 |
| LRMDA | Leucine Rich Melanocyte Differentiation Associated | Protein Coding | 29 | GC10P075432 | 1.114907622 |
| MT-TG | Mitochondrially Encoded TRNA-Gly (GGN) | RNA Gene | 11 | GCMTP009993 | 1.114839077 |
| UBL4A | Ubiquitin Like 4A | Protein Coding | 31 | GC0XM154483 | 1.114801168 |
| CSRNP1 | Cysteine And Serine Rich Nuclear Protein 1 | Protein Coding | 30 | GC03M039159 | 1.114758253 |
| LINC00536 | Long Intergenic Non-Protein Coding RNA 536 | RNA Gene | 14 | GC08M115950 | 1.114410162 |
| LINC01264 | Long Intergenic Non-Protein Coding RNA 1264 | RNA Gene | 11 | GC10M042980 | 1.114304662 |
| TRIM35 | Tripartite Motif Containing 35 | Protein Coding | 32 | GC08M027284 | 1.114245653 |
| QTRT1 | Queuine TRNA-Ribosyltransferase Catalytic Subunit 1 | Protein Coding | 34 | GC19P010701 | 1.113485932 |
| LUNAR1 | Leukemia-Associated Non-Coding IGF1R Activator RNA 1 | RNA Gene | 13 | GC15P099014 | 1.113298059 |
| BAALC-AS1 | BAALC Antisense RNA 1 | RNA Gene | 14 | GC08M103156 | 1.113143921 |
| CADM4 | Cell Adhesion Molecule 4 | Protein Coding | 33 | GC19M043622 | 1.112114906 |
| CRYGS | Crystallin Gamma S | Protein Coding | 36 | GC03M186538 | 1.111962318 |
| MITD1 | Microtubule Interacting And Trafficking Domain Containing 1 | Protein Coding | 28 | GC02M099152 | 1.11183238 |
| TAF3 | TATA-Box Binding Protein Associated Factor 3 | Protein Coding | 34 | GC10P007860 | 1.111810088 |
| BORCS6 | BLOC-1 Related Complex Subunit 6 | Protein Coding | 27 | GC17M010282 | 1.11179316 |
| MIR4717 | MicroRNA 4717 | RNA Gene | 9 | GC16P010728 | 1.11123085 |
| MIR4709 | MicroRNA 4709 | RNA Gene | 11 | GC14M074481 | 1.111215115 |
| MRPL24 | Mitochondrial Ribosomal Protein L24 | Protein Coding | 33 | GC01M156737 | 1.110971093 |
| CNOT4 | CCR4-NOT Transcription Complex Subunit 4 | Protein Coding | 35 | GC07M135361 | 1.11097002 |
| LINC02599 | Long Intergenic Non-Protein Coding RNA 2599 | RNA Gene | 11 | GC08P048556 | 1.110263109 |
| ECHS1 | Enoyl-CoA Hydratase, Short Chain 1 | Protein Coding | 42 | GC10M133362 | 1.109892249 |
| KDELR1 | KDEL Endoplasmic Reticulum Protein Retention Receptor 1 | Protein Coding | 35 | GC19M048382 | 1.109783411 |
| PALM3 | Paralemmin 3 | Protein Coding | 27 | GC19M014451 | 1.109783411 |
| IKZF2 | IKAROS Family Zinc Finger 2 | Protein Coding | 34 | GC02M213001 | 1.109755516 |
| CDH8 | Cadherin 8 | Protein Coding | 38 | GC16M061647 | 1.109280586 |
| WDR33 | WD Repeat Domain 33 | Protein Coding | 33 | GC02M127701 | 1.108588219 |
| KMO | Kynurenine 3-Monooxygenase | Protein Coding | 40 | GC01P241532 | 1.108435631 |
| GRIP2 | Glutamate Receptor Interacting Protein 2 | Protein Coding | 32 | GC03M020218 | 1.108166575 |
| ARAP2 | ArfGAP With RhoGAP Domain, Ankyrin Repeat And PH Domain 2 | Protein Coding | 31 | GC04M035950 | 1.108166575 |
| PHF11 | PHD Finger Protein 11 | Protein Coding | 32 | GC13P049495 | 1.108136535 |
| RPL22L1 | Ribosomal Protein L22 Like 1 | Protein Coding | 32 | GC03M170864 | 1.108086348 |
| EPS15L1 | Epidermal Growth Factor Receptor Pathway Substrate 15 Like 1 | Protein Coding | 35 | GC19M016333 | 1.106793165 |
| BCL2L13 | BCL2 Like 13 | Protein Coding | 34 | GC22P017628 | 1.106703401 |
| DGKQ | Diacylglycerol Kinase Theta | Protein Coding | 38 | GC04M000958 | 1.106474638 |
| KRTCAP3 | Keratinocyte Associated Protein 3 | Protein Coding | 28 | GC02P027442 | 1.106414557 |
| SCG3 | Secretogranin III | Protein Coding | 36 | GC15P051681 | 1.106410027 |
| HSD17B7P2 | Hydroxysteroid 17-Beta Dehydrogenase 7 Pseudogene 2 | Pseudogene | 14 | GC10P038353 | 1.106309414 |
| RAB14 | RAB14, Member RAS Oncogene Family | Protein Coding | 35 | GC09M121178 | 1.106167793 |
| SH3RF3 | SH3 Domain Containing Ring Finger 3 | Protein Coding | 29 | GC02P109129 | 1.106018424 |
| YIPF2 | Yip1 Domain Family Member 2 | Protein Coding | 30 | GC19M011059 | 1.105951428 |
| CTAGE3P | CTAGE Family Member 3, Pseudogene | Pseudogene | 13 | GC13M051907 | 1.105916381 |
| LOC111188152 | CSF1R Promoter E1 | Biological Region | 2 | GC05P150113 | 1.105405092 |
| UBA6 | Ubiquitin Like Modifier Activating Enzyme 6 | Protein Coding | 35 | GC04M067612 | 1.105349779 |
| METTL5 | Methyltransferase 5, N6-Adenosine | Protein Coding | 33 | GC02M169823 | 1.105328441 |
| CATSPERG | Cation Channel Sperm Associated Auxiliary Subunit Gamma | Protein Coding | 30 | GC19P063529 | 1.104979634 |
| GPR182 | G Protein-Coupled Receptor 182 | Protein Coding | 32 | GC12P057231 | 1.104802132 |
| MAF1 | MAF1 Homolog, Negative Regulator Of RNA Polymerase III | Protein Coding | 34 | GC08P144288 | 1.104779243 |
| RABGGTA | Rab Geranylgeranyltransferase Subunit Alpha | Protein Coding | 36 | GC14M024265 | 1.104711294 |
| SLC39A13 | Solute Carrier Family 39 Member 13 | Protein Coding | 37 | GC11P047407 | 1.103928566 |
| MIR920 | MicroRNA 920 | RNA Gene | 14 | GC12P024212 | 1.103398323 |
| STRA8 | Stimulated By Retinoic Acid 8 | Protein Coding | 32 | GC07P135231 | 1.103302717 |
| ATXN7L3 | Ataxin 7 Like 3 | Protein Coding | 31 | GC17M044191 | 1.102562308 |
| UBFD1 | Ubiquitin Family Domain Containing 1 | Protein Coding | 30 | GC16P023557 | 1.102495909 |
| RNR1 | RNA, Ribosomal 45S Cluster 1 | RNA Gene | 9 | GC13U990028 | 1.102256894 |
| NANS | N-Acetylneuraminate Synthase | Protein Coding | 39 | GC09P098056 | 1.102198362 |
| LINC02933 | Long Intergenic Non-Protein Coding RNA 2933 | RNA Gene | 2 | GC08U902940 | 1.101966023 |

| | | | | | |
|---|---|---|---|---|---|
| ARHGEF28 | Rho Guanine Nucleotide Exchange Factor 28 | Protein Coding | 32 | GC05P073626 | 1.101881742 |
| DMAC2 | Distal Membrane Arm Assembly Component 2 | Protein Coding | 26 | GC19M063825 | 1.101732731 |
| SLC15A2 | Solute Carrier Family 15 Member 2 | Protein Coding | 36 | GC03P121894 | 1.101438284 |
| USP29 | Ubiquitin Specific Peptidase 29 | Protein Coding | 32 | GC19P057119 | 1.101003289 |
| CISD3 | CDGSH Iron Sulfur Domain 3 | Protein Coding | 26 | GC17P038730 | 1.100999594 |
| IZUMO1 | Izumo Sperm-Egg Fusion 1 | Protein Coding | 34 | GC19M048740 | 1.100892186 |
| ZFP82 | ZFP82 Zinc Finger Protein | Protein Coding | 28 | GC19M063748 | 1.100831151 |
| TMED7 | Transmembrane P24 Trafficking Protein 7 | Protein Coding | 29 | GC05M115613 | 1.10073936 |
| SLC5A3 | Solute Carrier Family 5 Member 3 | Protein Coding | 38 | GC21P034133 | 1.100565434 |
| GINM1 | Glycosylated Integral Membrane Protein 1 | Protein Coding | 26 | GC06P149566 | 1.100276351 |
| SLF2 | SMC5-SMC6 Complex Localization Factor 2 | Protein Coding | 26 | GC10P100913 | 1.100267887 |
| CETN1 | Centrin 1 | Protein Coding | 34 | GC18P000580 | 1.099922776 |
| LOC108449897 | PATRR11 Recombination Region | Biological Region | 2 | GC11U902939 | 1.099155664 |
| ZNF512B | Zinc Finger Protein 512B | Protein Coding | 31 | GC20M063956 | 1.099096298 |
| EPS8L3 | EPS8 Like 3 | Protein Coding | 34 | GC01M109751 | 1.099069953 |
| POTEI | POTE Ankyrin Domain Family Member I | Protein Coding | 22 | GC02M130459 | 1.098664522 |
| MRPS12 | Mitochondrial Ribosomal Protein S12 | Protein Coding | 35 | GC19P038930 | 1.098497033 |
| ALDH5A1 | Aldehyde Dehydrogenase 5 Family Member A1 | Protein Coding | 43 | GC06P024494 | 1.0980829 |
| LOC113664107 | CFTR Intron 3 DNase I Hypersensitive Site | Biological Region | 2 | GC07P117509 | 1.098035693 |
| HAUS3 | HAUS Augmin Like Complex Subunit 3 | Protein Coding | 30 | GC04M002157 | 1.097897053 |
| GINS3 | GINS Complex Subunit 3 | Protein Coding | 30 | GC16P058328 | 1.097723246 |
| HCLS1 | Hematopoietic Cell-Specific Lyn Substrate 1 | Protein Coding | 37 | GC03M121631 | 1.09735775 |
| ECT2L | Epithelial Cell Transforming 2 Like | Protein Coding | 31 | GC06P138795 | 1.096946836 |
| STON1 | Stonin 1 | Protein Coding | 30 | GC02P048530 | 1.096946836 |
| CNEP1R1 | CTD Nuclear Envelope Phosphatase 1 Regulatory Subunit 1 | Protein Coding | 27 | GC16P050024 | 1.096946836 |
| CFD | Complement Factor D | Protein Coding | 40 | GC19P000859 | 1.09644568 |
| NKIRAS1 | NFKB Inhibitor Interacting Ras Like 1 | Protein Coding | 32 | GC03M024192 | 1.095995545 |
| WSB1 | WD Repeat And SOCS Box Containing 1 | Protein Coding | 36 | GC17P027294 | 1.095848322 |
| SLC25A32 | Solute Carrier Family 25 Member 32 | Protein Coding | 36 | GC08M103398 | 1.095817447 |
| MTRFR | Mitochondrial Translation Release Factor In Rescue | Protein Coding | 28 | GC12P123233 | 1.095688343 |
| GPR39 | G Protein-Coupled Receptor 39 | Protein Coding | 37 | GC02P136526 | 1.095166087 |
| ZNF101 | Zinc Finger Protein 101 | Protein Coding | 31 | GC19P063135 | 1.095002174 |
| ITGB2-AS1 | ITGB2 Antisense RNA 1 | RNA Gene | 13 | GC21P044921 | 1.094915867 |
| LRRC49 | Leucine Rich Repeat Containing 49 | Protein Coding | 28 | GC15P070853 | 1.094840288 |
| FAM114A2 | Family With Sequence Similarity 114 Member A2 | Protein Coding | 30 | GC05M153990 | 1.094605207 |
| SERPINB12 | Serpin Family B Member 12 | Protein Coding | 31 | GC18P063556 | 1.094533443 |
| EEF1AKMT2 | EEF1A Lysine Methyltransferase 2 | Protein Coding | 26 | GC10M124866 | 1.094530821 |
| FRMD4A | FERM Domain Containing 4A | Protein Coding | 34 | GC10M013643 | 1.093897104 |
| THORLNC | Testis Associated Oncogenic LncRNA | RNA Gene | 11 | GC02M118133 | 1.093803525 |
| GRPEL1 | GrpE Like 1, Mitochondrial | Protein Coding | 35 | GC04M007060 | 1.093573451 |
| NDFIP2 | Nedd4 Family Interacting Protein 2 | Protein Coding | 33 | GC13P079481 | 1.093426824 |
| SPATA5 | Spermatogenesis Associated 5 | Protein Coding | 37 | GC04P122923 | 1.093047142 |
| COG4 | Component Of Oligomeric Golgi Complex 4 | Protein Coding | 36 | GC16M070566 | 1.092838049 |
| HOXD8 | Homeobox D8 | Protein Coding | 34 | GC02P176129 | 1.091892958 |
| ZNF385A | Zinc Finger Protein 385A | Protein Coding | 30 | GC12M054369 | 1.091892958 |
| DLG4 | Discs Large MAGUK Scaffold Protein 4 | Protein Coding | 42 | GC17M007189 | 1.091732979 |
| MIR3188 | MicroRNA 3188 | RNA Gene | 14 | GC19P018282 | 1.091672897 |
| USP35 | Ubiquitin Specific Peptidase 35 | Protein Coding | 30 | GC11P078188 | 1.091019511 |
| KPNA7 | Karyopherin Subunit Alpha 7 | Protein Coding | 30 | GC07M099173 | 1.090814352 |
| WDR89 | WD Repeat Domain 89 | Protein Coding | 30 | GC14M063597 | 1.09034431 |
| NAGPA | N-Acetylglucosamine-1-Phosphodiester Alpha-N-Acetylglucosaminidase | Protein Coding | 32 | GC16M006912 | 1.089615345 |
| SPCS1 | Signal Peptidase Complex Subunit 1 | Protein Coding | 31 | GC03P053031 | 1.089362979 |
| HEXIM2 | HEXIM P-TEFb Complex Subunit 2 | Protein Coding | 31 | GC17P045159 | 1.089325428 |
| SLC38A10 | Solute Carrier Family 38 Member 10 | Protein Coding | 30 | GC17M081244 | 1.089325428 |
| RALGPS2 | Ral GEF With PH Domain And SH3 Binding Motif 2 | Protein Coding | 31 | GC01P178725 | 1.088840723 |
| PCDH18 | Protocadherin 18 | Protein Coding | 34 | GC04M137518 | 1.088332891 |
| XPO4 | Exportin 4 | Protein Coding | 34 | GC13M020777 | 1.088320494 |
| TBC1D7 | TBC1 Domain Family Member 7 | Protein Coding | 37 | GC06M013266 | 1.088164806 |
| CWF19L1 | CWF19 Like Cell Cycle Control Factor 1 | Protein Coding | 35 | GC10M100232 | 1.08801055 |
| CMTM6 | CKLF Like MARVEL Transmembrane Domain Containing 6 | Protein Coding | 32 | GC03M032499 | 1.087947607 |
| THSD7A | Thrombospondin Type 1 Domain Containing 7A | Protein Coding | 32 | GC07M011371 | 1.087701797 |
| EME2 | Essential Meiotic Structure-Specific Endonuclease Subunit 2 | Protein Coding | 30 | GC16P010700 | 1.087679386 |
| SHROOM1 | Shroom Family Member 1 | Protein Coding | 31 | GC05M132822 | 1.08740747 |
| SH2D4B | SH2 Domain Containing 4B | Protein Coding | 31 | GC10P091446 | 1.08736217 |
| PLXNA3 | Plexin A3 | Protein Coding | 34 | GC0XP154458 | 1.087258339 |
| STX2 | Syntaxin 2 | Protein Coding | 33 | GC12M130789 | 1.087242246 |
| GALNT7 | Polypeptide N-Acetylgalactosaminyltransferase 7 | Protein Coding | 34 | GC04P173168 | 1.086783648 |
| ENY2 | ENY2 Transcription And Export Complex 2 Subunit | Protein Coding | 30 | GC08P109334 | 1.08668375 |
| HTATSF1 | HIV-1 Tat Specific Factor 1 | Protein Coding | 32 | GC0XP136497 | 1.086551785 |
| GINS4 | GINS Complex Subunit 4 | Protein Coding | 31 | GC08P041529 | 1.086517811 |
| H2BC4 | H2B Clustered Histone 4 | Protein Coding | 28 | GC06M064158 | 1.086284161 |
| RUNDC3A-AS1 | RUNDC3A Antisense RNA 1 | RNA Gene | 13 | GC17M044858 | 1.086234927 |
| PCNAP1 | Proliferating Cell Nuclear Antigen Pseudogene 1 | Pseudogene | 11 | GC04M099160 | 1.086152554 |
| DCLK3 | Doublecortin Like Kinase 3 | Protein Coding | 32 | GC03M036712 | 1.085913777 |
| CACNG2 | Calcium Voltage-Gated Channel Auxiliary Subunit Gamma 2 | Protein Coding | 40 | GC22M036986 | 1.085858822 |
| ARAP1 | ArfGAP With RhoGAP Domain, Ankyrin Repeat And PH Domain 1 | Protein Coding | 37 | GC11M087543 | 1.085448384 |
| CPNE8 | Copine 8 | Protein Coding | 32 | GC12M038646 | 1.085112929 |
| PSMG3-AS1 | PSMG3 Antisense RNA 1 (Head To Head) | RNA Gene | 14 | GC07P001984 | 1.085109234 |
| JCHAIN | Joining Chain Of Multimeric IgA And IgM | Protein Coding | 32 | GC04M070655 | 1.084824085 |
| SCLT1 | Sodium Channel And Clathrin Linker 1 | Protein Coding | 30 | GC04M128864 | 1.084723711 |
| TTLL9 | Tubulin Tyrosine Ligase Like 9 | Protein Coding | 30 | GC20P031870 | 1.084587216 |
| SDF2L1 | Stromal Cell Derived Factor 2 Like 1 | Protein Coding | 32 | GC22P034526 | 1.084503412 |
| LPCAT3 | Lysophosphatidylcholine Acyltransferase 3 | Protein Coding | 31 | GC12M006976 | 1.084503412 |
| FAM83E | Family With Sequence Similarity 83 Member E | Protein Coding | 30 | GC19M064066 | 1.084191322 |
| CLTA | Clathrin Light Chain A | Protein Coding | 35 | GC09P036190 | 1.084032297 |
| PCGF1 | Polycomb Group Ring Finger 1 | Protein Coding | 32 | GC02M074505 | 1.084022522 |
| MIR3117 | MicroRNA 3117 | RNA Gene | 14 | GC01P066628 | 1.083762169 |
| LGALS12 | Galectin 12 | Protein Coding | 30 | GC11P063506 | 1.083618164 |
| SLC25A35 | Solute Carrier Family 25 Member 35 | Protein Coding | 31 | GC17M008287 | 1.08342886 |
| ZNF576 | Zinc Finger Protein 576 | Protein Coding | 26 | GC19P043596 | 1.083252549 |
| ATP6V1D | ATPase H+ Transporting V1 Subunit D | Protein Coding | 36 | GC14M067294 | 1.082776189 |
| ZC3H4 | Zinc Finger CCCH-Type Containing 4 | Protein Coding | 30 | GC19M047064 | 1.082774639 |
| TMX2 | Thioredoxin Related Transmembrane Protein 2 | Protein Coding | 33 | GC11P057713 | 1.082595825 |
| MIR1343 | MicroRNA 1343 | RNA Gene | 14 | GC11P034964 | 1.082415581 |
| SIPA1L2 | Signal Induced Proliferation Associated 1 Like 2 | Protein Coding | 34 | GC01M232397 | 1.082122087 |

| | | | | | |
|---|---|---|---|---|---|
| MTBP | MDM2 Binding Protein | Protein Coding | 29 | GC08P120426 | 1.081982851 |
| C11orf98 | Chromosome 11 Open Reading Frame 98 | Protein Coding | 17 | GC11M087132 | 1.081964731 |
| UBXN11 | UBX Domain Protein 11 | Protein Coding | 33 | GC01M026281 | 1.081757784 |
| CNOT6 | CCR4-NOT Transcription Complex Subunit 6 | Protein Coding | 33 | GC05P180494 | 1.081320763 |
| TOX4 | TOX High Mobility Group Box Family Member 4 | Protein Coding | 30 | GC14P021476 | 1.081126332 |
| ULBP1 | UL16 Binding Protein 1 | Protein Coding | 34 | GC06P149963 | 1.080846667 |
| TAF4B | TATA-Box Binding Protein Associated Factor 4b | Protein Coding | 38 | GC18P026225 | 1.080780149 |
| ATL2 | Atlastin GTPase 2 | Protein Coding | 32 | GC02M038294 | 1.080134749 |
| CLEC4G | C-Type Lectin Domain Family 4 Member G | Protein Coding | 35 | GC19M007728 | 1.079951882 |
| ANXA9 | Annexin A9 | Protein Coding | 34 | GC01P150982 | 1.079942942 |
| NUDT12 | Nudix Hydrolase 12 | Protein Coding | 34 | GC05M103548 | 1.079604387 |
| SEC61A2 | SEC61 Translocon Subunit Alpha 2 | Protein Coding | 31 | GC10P012130 | 1.078840733 |
| RPL7L1 | Ribosomal Protein L7 Like 1 | Protein Coding | 30 | GC06P080592 | 1.0787853 |
| CCDC13-AS2 | CCDC13 Antisense RNA 2 | RNA Gene | 11 | GC03P042772 | 1.078756809 |
| FBLL1 | Fibrillarin Like 1 | Protein Coding | 23 | GC05P168529 | 1.078513384 |
| TXNDC17 | Thioredoxin Domain Containing 17 | Protein Coding | 32 | GC17P006640 | 1.078192949 |
| FTMT | Ferritin Mitochondrial | Protein Coding | 34 | GC05P121851 | 1.077857971 |
| ATP6V0D1 | ATPase H+ Transporting V0 Subunit D1 | Protein Coding | 38 | GC16M067438 | 1.077831745 |
| AP3B2 | Adaptor Related Protein Complex 3 Subunit Beta 2 | Protein Coding | 36 | GC15M088932 | 1.077807426 |
| A1BG | Alpha-1-B Glycoprotein | Protein Coding | 35 | GC19M058345 | 1.077638626 |
| THOP1 | Thimet Oligopeptidase 1 | Protein Coding | 38 | GC19P002785 | 1.077435136 |
| TARS2 | Threonyl-TRNA Synthetase 2, Mitochondrial | Protein Coding | 38 | GC01P150555 | 1.077367544 |
| MYO16 | Myosin XVI | Protein Coding | 31 | GC13P108495 | 1.077280998 |
| ST3GAL6-AS1 | ST3GAL6 Antisense RNA 1 | RNA Gene | 16 | GC03M098714 | 1.077173471 |
| ARHGAP22 | Rho GTPase Activating Protein 22 | Protein Coding | 34 | GC10M048430 | 1.077110529 |
| WASH2P | WASP Family Homolog 2, Pseudogene | Pseudogene | 18 | GC02P113583 | 1.07706058 |
| KANSL2 | KAT8 Regulatory NSL Complex Subunit 2 | Protein Coding | 30 | GC12M048653 | 1.077057958 |
| MIR634 | MicroRNA 634 | RNA Gene | 14 | GC17P066787 | 1.076888561 |
| WTIP | WT1 Interacting Protein | Protein Coding | 31 | GC19P034481 | 1.076738358 |
| TALAM1 | TALAM1 Transcript, MALAT1 Antisense RNA | RNA Gene | 11 | GC11M087267 | 1.076418877 |
| CERS5 | Ceramide Synthase 5 | Protein Coding | 33 | GC12M050129 | 1.076396704 |
| MRPS16 | Mitochondrial Ribosomal Protein S16 | Protein Coding | 39 | GC10M073248 | 1.076153755 |
| MIR1245B | MicroRNA 1245b | RNA Gene | 13 | GC02M188978 | 1.07606411 |
| EMC6 | ER Membrane Protein Complex Subunit 6 | Protein Coding | 27 | GC17P003668 | 1.075993299 |
| CCDC134 | Coiled-Coil Domain Containing 134 | Protein Coding | 32 | GC22P041800 | 1.075932026 |
| ACAA1 | Acetyl-CoA Acyltransferase 1 | Protein Coding | 38 | GC03M038103 | 1.075875044 |
| XPNPEP2 | X-Prolyl Aminopeptidase 2 | Protein Coding | 40 | GC0XP129738 | 1.075469017 |
| ABI3BP | ABI Family Member 3 Binding Protein | Protein Coding | 34 | GC03M100749 | 1.075147152 |
| RADIL | Rap Associating With DIL Domain | Protein Coding | 30 | GC07M004797 | 1.075050116 |
| LRRC27 | Leucine Rich Repeat Containing 27 | Protein Coding | 26 | GC10P132332 | 1.074990273 |
| MYO3B | Myosin IIIB | Protein Coding | 37 | GC02P170178 | 1.074969292 |
| ELMOD1 | ELMO Domain Containing 1 | Protein Coding | 30 | GC11P107592 | 1.074931145 |
| CAMLG | Calcium Modulating Ligand | Protein Coding | 36 | GC05P134738 | 1.074907184 |
| TRIM17 | Tripartite Motif Containing 17 | Protein Coding | 33 | GC01M228407 | 1.07478404 |
| ACTR8 | Actin Related Protein 8 | Protein Coding | 31 | GC03M053864 | 1.074434996 |
| EVA1A | Eva-1 Homolog A, Regulator Of Programmed Cell Death | Protein Coding | 31 | GC02M075469 | 1.074325323 |
| PALS2 | Protein Associated With LIN7 2, MAGUK P55 Family Member | Protein Coding | 27 | GC07P024574 | 1.074253798 |
| C1QTNF6 | C1q And TNF Related 6 | Protein Coding | 34 | GC22M037180 | 1.074065447 |
| TMIGD2 | Transmembrane And Immunoglobulin Domain Containing 2 | Protein Coding | 28 | GC19M004292 | 1.074017763 |
| ZDHHC20 | Zinc Finger DHHC-Type Palmitoyltransferase 20 | Protein Coding | 33 | GC13M021372 | 1.073808074 |
| FAM102A | Family With Sequence Similarity 102 Member A | Protein Coding | 28 | GC09M127941 | 1.073537827 |
| HSD17B1P1 | Hydroxysteroid 17-Beta Dehydrogenase 1 Pseudogene 1 | Pseudogene | 7 | GC17P042546 | 1.07308805 |
| DYNLT3 | Dynein Light Chain Tctex-Type 3 | Protein Coding | 32 | GC0XM037836 | 1.073073268 |
| UTP25 | UTP25 Small Subunit Processome Component | Protein Coding | 26 | GC01P209828 | 1.07266736 |
| SUSD1 | Sushi Domain Containing 1 | Protein Coding | 32 | GC09M112040 | 1.072660923 |
| KRTAP10-3 | Keratin Associated Protein 10-3 | Protein Coding | 21 | GC21M044557 | 1.071714878 |
| SLC10A5 | Solute Carrier Family 10 Member 5 | Protein Coding | 30 | GC08M081694 | 1.070839643 |
| NCAPG2 | Non-SMC Condensin II Complex Subunit G2 | Protein Coding | 34 | GC07M158631 | 1.06920135 |
| MKRN3-AS1 | MKRN3 Antisense RNA 1 | RNA Gene | 6 | GC15U901326 | 1.068512678 |
| MIR761 | MicroRNA 761 | RNA Gene | 11 | GC01M051836 | 1.068437338 |
| MIR3662 | MicroRNA 3662 | RNA Gene | 13 | GC06M134979 | 1.067871571 |
| ADPGK | ADP Dependent Glucokinase | Protein Coding | 37 | GC15M072751 | 1.067543626 |
| TTC27 | Tetratricopeptide Repeat Domain 27 | Protein Coding | 28 | GC02P032628 | 1.06743145 |
| FGD6 | FYVE, RhoGEF And PH Domain Containing 6 | Protein Coding | 32 | GC12M095076 | 1.067052603 |
| CCDC14 | Coiled-Coil Domain Containing 14 | Protein Coding | 30 | GC03M123897 | 1.066790819 |
| TDH | L-Threonine Dehydrogenase (Pseudogene) | Pseudogene | 22 | GC08P011339 | 1.066790819 |
| TAF9B | TATA-Box Binding Protein Associated Factor 9b | Protein Coding | 32 | GC0XM078129 | 1.066668749 |
| LINC00240 | Long Intergenic Non-Protein Coding RNA 240 | RNA Gene | 15 | GC06P026956 | 1.066599131 |
| TMPRSS12 | Transmembrane Serine Protease 12 | Protein Coding | 31 | GC12P050842 | 1.066482306 |
| NDC1 | NDC1 Transmembrane Nucleoporin | Protein Coding | 30 | GC01M053765 | 1.066203475 |
| SPP2 | Secreted Phosphoprotein 2 | Protein Coding | 30 | GC02P234050 | 1.06604135 |
| RSRP1 | Arginine And Serine Rich Protein 1 | Protein Coding | 25 | GC01M025242 | 1.065992832 |
| ZNF233 | Zinc Finger Protein 233 | Protein Coding | 27 | GC19P044259 | 1.065635681 |
| STRIT1 | Small Transmembrane Regulator Of Ion Transport 1 | Protein Coding | 14 | GC03M155294 | 1.065635681 |
| ITGA10 | Integrin Subunit Alpha 10 | Protein Coding | 35 | GC01M145891 | 1.06561923 |
| PIP5K1B | Phosphatidylinositol-4-Phosphate 5-Kinase Type 1 Beta | Protein Coding | 39 | GC09P068705 | 1.064971209 |
| PAQR6 | Progestin And AdipoQ Receptor Family Member 6 | Protein Coding | 31 | GC01M156243 | 1.064424157 |
| CCDC57 | Coiled-Coil Domain Containing 57 | Protein Coding | 29 | GC17M082101 | 1.06400919 |
| KNDC1 | Kinase Non-Catalytic C-Lobe Domain Containing 1 | Protein Coding | 31 | GC10P133161 | 1.063945532 |
| MAP1S | Microtubule Associated Protein 1S | Protein Coding | 31 | GC19P063110 | 1.063730001 |
| CAMTA1-DT | CAMTA1 Divergent Transcript | RNA Gene | 11 | GC01M006849 | 1.063646793 |
| LAPTM5 | Lysosomal Protein Transmembrane 5 | Protein Coding | 34 | GC01M030732 | 1.063354254 |
| HABP4 | Hyaluronan Binding Protein 4 | Protein Coding | 32 | GC09P096450 | 1.063354254 |
| ZNF622 | Zinc Finger Protein 622 | Protein Coding | 31 | GC05M016451 | 1.063354254 |
| ARFIP2 | ADP Ribosylation Factor Interacting Protein 2 | Protein Coding | 35 | GC11M006476 | 1.062996387 |
| ARMC1 | Armadillo Repeat Containing 1 | Protein Coding | 31 | GC08M065602 | 1.062996387 |
| C2CD5 | C2 Calcium Dependent Domain Containing 5 | Protein Coding | 28 | GC12M022448 | 1.062972307 |
| ZNF106 | Zinc Finger Protein 106 | Protein Coding | 30 | GC15M042412 | 1.062450886 |
| OVOL2 | Ovo Like Zinc Finger 2 | Protein Coding | 32 | GC20M017956 | 1.061022043 |
| TRMT12 | TRNA Methyltransferase 12 Homolog | Protein Coding | 30 | GC08P124450 | 1.060989857 |
| MIR3184 | MicroRNA 3184 | RNA Gene | 12 | GC17M030117 | 1.060450554 |
| TNS2 | Tensin 2 | Protein Coding | 31 | GC12P053046 | 1.05971849 |
| PLEKHA1 | Pleckstrin Homology Domain Containing A1 | Protein Coding | 36 | GC10P122374 | 1.059583187 |
| TUBGCP5 | Tubulin Gamma Complex Associated Protein 5 | Protein Coding | 29 | GC15M022983 | 1.059511065 |
| CRYBB2P1 | Crystallin Beta B2 Pseudogene 1 | Pseudogene | 12 | GC22P025448 | 1.059241295 |

| | | | | | |
|---|---|---|---|---|---|
| SEH1L | SEH1 Like Nucleoporin | Protein Coding | 29 | GC18P012947 | 1.059054613 |
| OSR2 | Odd-Skipped Related Transciption Factor 2 | Protein Coding | 32 | GC08P098944 | 1.058146954 |
| SYPL1 | Synaptophysin Like 1 | Protein Coding | 34 | GC07M106090 | 1.058110952 |
| ZNF579 | Zinc Finger Protein 579 | Protein Coding | 26 | GC19M055576 | 1.058018923 |
| KCNIP4 | Potassium Voltage-Gated Channel Interacting Protein 4 | Protein Coding | 34 | GC04M020728 | 1.057922721 |
| DDX28 | DEAD-Box Helicase 28 | Protein Coding | 32 | GC16M068085 | 1.057840109 |
| HISLA | HIF1A Stabilizing Long Noncoding RNA | RNA Gene | 11 | GC14P088025 | 1.057566047 |
| ELOVL1 | ELOVL Fatty Acid Elongase 1 | Protein Coding | 36 | GC01M043363 | 1.057565689 |
| IGKV2D-29 | Immunoglobulin Kappa Variable 2D-29 | Protein Coding | 10 | GC02P091239 | 1.057181001 |
| TAPT1 | Transmembrane Anterior Posterior Transformation 1 | Protein Coding | 33 | GC04M016162 | 1.056719065 |
| ELOVL7 | ELOVL Fatty Acid Elongase 7 | Protein Coding | 32 | GC05M060751 | 1.056540251 |
| ATP5F1E | ATP Synthase F1 Subunit Epsilon | Protein Coding | 33 | GC20M059026 | 1.056449533 |
| KRTAP4-8 | Keratin Associated Protein 4-8 | Protein Coding | 24 | GC17M041096 | 1.056441545 |
| ACRV1 | Acrosomal Vesicle Protein 1 | Protein Coding | 30 | GC11M125671 | 1.055856228 |
| TMEM132E | Transmembrane Protein 132E | Protein Coding | 32 | GC17P052701 | 1.055765152 |
| CHIA | Chitinase Acidic | Protein Coding | 39 | GC01P111291 | 1.055699229 |
| CARHSP1 | Calcium Regulated Heat Stable Protein 1 | Protein Coding | 32 | GC16M008852 | 1.055699229 |
| FBXW12 | F-Box And WD Repeat Domain Containing 12 | Protein Coding | 31 | GC03P048372 | 1.055699229 |
| ZNF420 | Zinc Finger Protein 420 | Protein Coding | 32 | GC19P037007 | 1.055598259 |
| LOC105372990 | Uncharacterized LOC105372990 | RNA Gene | 6 | GC22P030432 | 1.055452466 |
| LOC101927764 | Uncharacterized LOC101927764 | RNA Gene | 3 | GC02M119721 | 1.055452466 |
| C18orf21 | Chromosome 18 Open Reading Frame 21 | Protein Coding | 25 | GC18P035973 | 1.055311561 |
| AARSD1 | Alanyl-TRNA Synthetase Domain Containing 1 | Protein Coding | 31 | GC17M042951 | 1.054670215 |
| SYAP1 | Synapse Associated Protein 1 | Protein Coding | 29 | GC0XP016719 | 1.054670215 |
| NDST1 | N-Deacetylase And N-Sulfotransferase 1 | Protein Coding | 40 | GC05P150484 | 1.054549575 |
| GPX6 | Glutathione Peroxidase 6 | Protein Coding | 32 | GC06M028503 | 1.053867698 |
| EMC4 | ER Membrane Protein Complex Subunit 4 | Protein Coding | 28 | GC15P039609 | 1.053569555 |
| IFT80 | Intraflagellar Transport 80 | Protein Coding | 32 | GC03M160256 | 1.052913904 |
| LINC02871 | Long Intergenic Non-Protein Coding RNA 2871 | RNA Gene | 14 | GC20P010997 | 1.052853703 |
| PHKA1 | Phosphorylase Kinase Regulatory Subunit Alpha 1 | Protein Coding | 38 | GC0XM072578 | 1.05261898 |
| MIR3156-1 | MicroRNA 3156-1 | RNA Gene | 11 | GC10P045164 | 1.052618504 |
| MIR3156-2 | MicroRNA 3156-2 | RNA Gene | 11 | GC18P014830 | 1.052618504 |
| GPR17 | G Protein-Coupled Receptor 17 | Protein Coding | 36 | GC02P127645 | 1.052300811 |
| PTCHD3 | Patched Domain Containing 3 (Gene/Pseudogene) | Protein Coding | 28 | GC10M027834 | 1.05211997 |
| LOC106128905 | KLK3 Upstream Enhancer/Promoter Region | Biological Region | 2 | GC19P064146 | 1.051766753 |
| MIR891B | MicroRNA 891b | RNA Gene | 11 | GC0XM146001 | 1.051452875 |
| MIR550A1 | MicroRNA 550a-1 | RNA Gene | 17 | GC07P030289 | 1.050664186 |
| ABCA8 | ATP Binding Cassette Subfamily A Member 8 | Protein Coding | 35 | GC17M068867 | 1.050542951 |
| TRIM36 | Tripartite Motif Containing 36 | Protein Coding | 34 | GC05M115124 | 1.049994946 |
| MIR564 | MicroRNA 564 | RNA Gene | 13 | GC03P046359 | 1.049854159 |
| AP3M1 | Adaptor Related Protein Complex 3 Subunit Mu 1 | Protein Coding | 34 | GC10M074120 | 1.049851418 |
| MIR3682 | MicroRNA 3682 | RNA Gene | 11 | GC02M053849 | 1.04977107 |
| STYX | Serine/Threonine/Tyrosine Interacting Protein | Protein Coding | 32 | GC14P052730 | 1.049758196 |
| GPRC5C | G Protein-Coupled Receptor Class C Group 5 Member C | Protein Coding | 33 | GC17P074424 | 1.049671888 |
| SREK1 | Splicing Regulatory Glutamic Acid And Lysine Rich Protein 1 | Protein Coding | 33 | GC05P066139 | 1.049657583 |
| PPIAP29 | Peptidylprolyl Isomerase A Pseudogene 29 | Pseudogene | 7 | GC06P024977 | 1.049600482 |
| IGSF9 | Immunoglobulin Superfamily Member 9 | Protein Coding | 33 | GC01M159927 | 1.049081802 |
| CRYZ | Crystallin Zeta | Protein Coding | 40 | GC01M074705 | 1.049023986 |
| NKX2-2 | NK2 Homeobox 2 | Protein Coding | 36 | GC20M021511 | 1.048939586 |
| NFATC2IP | Nuclear Factor Of Activated T Cells 2 Interacting Protein | Protein Coding | 31 | GC16P040107 | 1.048701525 |
| BUD31 | BUD31 Homolog | Protein Coding | 33 | GC07P099408 | 1.048283339 |
| PSME4 | Proteasome Activator Subunit 4 | Protein Coding | 35 | GC02M053864 | 1.048274755 |
| FAM172A | Family With Sequence Similarity 172 Member A | Protein Coding | 32 | GC05M093617 | 1.048073411 |
| ZNF718 | Zinc Finger Protein 718 | Protein Coding | 25 | GC04P000118 | 1.04792881 |
| SYCP2 | Synaptonemal Complex Protein 2 | Protein Coding | 34 | GC20M059863 | 1.047793508 |
| SAP30L | SAP30 Like | Protein Coding | 33 | GC05P154445 | 1.04702878 |
| WSB2 | WD Repeat And SOCS Box Containing 2 | Protein Coding | 31 | GC12M118032 | 1.046963453 |
| HEBP1 | Heme Binding Protein 1 | Protein Coding | 31 | GC12M012974 | 1.046823978 |
| MIR23AHG | MiR-23a/27a/24-2 Cluster Host Gene | RNA Gene | 14 | GC19M014476 | 1.046717048 |
| CPAMD8 | C3 And PZP Like Alpha-2-Macroglobulin Domain Containing 8 | Protein Coding | 34 | GC19M016892 | 1.046521306 |
| C11orf24 | Chromosome 11 Open Reading Frame 24 | Protein Coding | 28 | GC11M087428 | 1.046063781 |
| EHD3 | EH Domain Containing 3 | Protein Coding | 32 | GC02P031234 | 1.045101166 |
| CEP120 | Centrosomal Protein 120 | Protein Coding | 35 | GC05M123344 | 1.044954419 |
| IL26 | Interleukin 26 | Protein Coding | 31 | GC12M068201 | 1.044934988 |
| MIR19B2 | MicroRNA 19b-2 | RNA Gene | 15 | GC0XM134301 | 1.044909596 |
| PRDM13 | PR/SET Domain 13 | Protein Coding | 30 | GC06P099606 | 1.044793248 |
| ELP2 | Elongator Acetyltransferase Complex Subunit 2 | Protein Coding | 34 | GC18P036129 | 1.04450202 |
| PDCD2 | Programmed Cell Death 2 | Protein Coding | 33 | GC06M170589 | 1.044217944 |
| SPCS2 | Signal Peptidase Complex Subunit 2 | Protein Coding | 30 | GC11P077657 | 1.044086218 |
| RWDD4 | RWD Domain Containing 4 | Protein Coding | 28 | GC04M183639 | 1.043998599 |
| TYW5 | TRNA-YW Synthesizing Protein 5 | Protein Coding | 28 | GC02M199928 | 1.043859005 |
| CENPV | Centromere Protein V | Protein Coding | 28 | GC17M016342 | 1.043154359 |
| MS | Multiple Sclerosis | Genetic Locus | 3 | GC00U990205 | 1.042782784 |
| C21orf91 | Chromosome 21 Open Reading Frame 91 | Protein Coding | 31 | GC21M017788 | 1.042550802 |
| TBCC | Tubulin Folding Cofactor C | Protein Coding | 32 | GC06M042744 | 1.042497873 |
| SLC2A14 | Solute Carrier Family 2 Member 14 | Protein Coding | 30 | GC12M007812 | 1.042497873 |
| C11orf54 | Chromosome 11 Open Reading Frame 54 | Protein Coding | 29 | GC11P093741 | 1.042389035 |
| TOB2 | Transducer Of ERBB2, 2 | Protein Coding | 34 | GC22M041433 | 1.042137027 |
| MARS2 | Methionyl-TRNA Synthetase 2, Mitochondrial | Protein Coding | 37 | GC02P197705 | 1.041857362 |
| MIR597 | MicroRNA 597 | RNA Gene | 14 | GC08P009741 | 1.041808486 |
| MIR520D | MicroRNA 520d | RNA Gene | 17 | GC19P053720 | 1.041359186 |
| L3MBTL2 | L3MBTL Histone Methyl-Lysine Binding Protein 2 | Protein Coding | 32 | GC22P041205 | 1.041260719 |
| TMEM80 | Transmembrane Protein 80 | Protein Coding | 27 | GC11P000695 | 1.041045427 |
| ZSCAN21 | Zinc Finger And SCAN Domain Containing 21 | Protein Coding | 31 | GC07P100049 | 1.040439844 |
| MIR5195 | MicroRNA 5195 | RNA Gene | 9 | GC14M112510 | 1.040384293 |
| MIR4758 | MicroRNA 4758 | RNA Gene | 10 | GC20M062332 | 1.040379405 |
| HEATR5B | HEAT Repeat Containing 5B | Protein Coding | 30 | GC02M036949 | 1.040245056 |
| NTM | Neurotrimin | Protein Coding | 38 | GC11P131370 | 1.040104985 |
| SNHG29 | Small Nucleolar RNA Host Gene 29 | RNA Gene | 18 | GC17P016920 | 1.039946079 |
| POLR2D | RNA Polymerase II Subunit D | Protein Coding | 36 | GC02M128222 | 1.039686084 |
| FGD3 | FYVE, RhoGEF And PH Domain Containing 3 | Protein Coding | 35 | GC09P092947 | 1.039652348 |
| SLC39A5 | Solute Carrier Family 39 Member 5 | Protein Coding | 37 | GC12P057187 | 1.039405823 |
| P2RY4 | Pyrimidinergic Receptor P2Y4 | Protein Coding | 36 | GC0XM070258 | 1.039304972 |
| ENSG00000270055 | Novel Transcript | RNA Gene | 7 | GC15P039451 | 1.039022446 |
| MTDHP4 | Metadherin Pseudogene 4 | Pseudogene | 6 | GC19P063162 | 1.039022446 |

| | | | | | |
|---|---|---|---|---|---|
| RTKN2 | Rhotekin 2 | Protein Coding | 32 | GC10M062183 | 1.038662434 |
| LMO3 | LIM Domain Only 3 | Protein Coding | 35 | GC12M016548 | 1.038533568 |
| TMSB15B | Thymosin Beta 15B | Protein Coding | 23 | GC0XP103918 | 1.038526058 |
| IGFBPL1 | Insulin Like Growth Factor Binding Protein Like 1 | Protein Coding | 34 | GC09M039914 | 1.038079739 |
| ICA1L | Islet Cell Autoantigen 1 Like | Protein Coding | 31 | GC02M202829 | 1.038079739 |
| GOLGA7 | Golgin A7 | Protein Coding | 33 | GC08P041467 | 1.037833929 |
| OAZ3 | Ornithine Decarboxylase Antizyme 3 | Protein Coding | 32 | GC01P151762 | 1.037726283 |
| UBA3 | Ubiquitin Like Modifier Activating Enzyme 3 | Protein Coding | 36 | GC03M069054 | 1.037616611 |
| DYNLL2 | Dynein Light Chain LC8-Type 2 | Protein Coding | 34 | GC17P058083 | 1.037597179 |
| MAP9 | Microtubule Associated Protein 9 | Protein Coding | 29 | GC04M155342 | 1.037597179 |
| RBM45 | RNA Binding Motif Protein 45 | Protein Coding | 30 | GC02P178112 | 1.037287235 |
| ATP5PF | ATP Synthase Peripheral Stalk Subunit F6 | Protein Coding | 31 | GC21M025875 | 1.036505938 |
| BROX | BRO1 Domain And CAAX Motif Containing | Protein Coding | 26 | GC01P222712 | 1.036016464 |
| KNOP1 | Lysine Rich Nucleolar Protein 1 | Protein Coding | 25 | GC16M019714 | 1.035679221 |
| INSIG1 | Insulin Induced Gene 1 | Protein Coding | 36 | GC07P155297 | 1.035274744 |
| MIR552 | MicroRNA 552 | RNA Gene | 13 | GC01M034669 | 1.035226583 |
| ATOH8 | Atonal BHLH Transcription Factor 8 | Protein Coding | 31 | GC02P085751 | 1.034926414 |
| COL24A1 | Collagen Type XXIV Alpha 1 Chain | Protein Coding | 31 | GC01M085729 | 1.034817219 |
| WDR25 | WD Repeat Domain 25 | Protein Coding | 28 | GC14P109201 | 1.034762502 |
| SLC27A6 | Solute Carrier Family 27 Member 6 | Protein Coding | 37 | GC05P128538 | 1.034728527 |
| DBR1 | Debranching RNA Lariats 1 | Protein Coding | 31 | GC03M138160 | 1.034589887 |
| RPS19BP1 | Ribosomal Protein S19 Binding Protein 1 | Protein Coding | 29 | GC22M056110 | 1.033971786 |
| GREP1 | Glycine Rich Extracellular Protein 1 | Protein Coding | 10 | GC16P011086 | 1.033619523 |
| SUSD6 | Sushi Domain Containing 6 | Protein Coding | 28 | GC14P069611 | 1.033384323 |
| DNAJC17 | DnaJ Heat Shock Protein Family (Hsp40) Member C17 | Protein Coding | 32 | GC15M040765 | 1.033042908 |
| KERA | Keratocan | Protein Coding | 39 | GC12M091050 | 1.032157183 |
| KIAA2013 | KIAA2013 | Protein Coding | 28 | GC01M011919 | 1.031897902 |
| SCCPDH | Saccharopine Dehydrogenase (Putative) | Protein Coding | 32 | GC01P246724 | 1.031498432 |
| MDFIC | MyoD Family Inhibitor Domain Containing | Protein Coding | 32 | GC07P114922 | 1.031365156 |
| SRSF12 | Serine And Arginine Rich Splicing Factor 12 | Protein Coding | 28 | GC06M089095 | 1.031230569 |
| DERL3 | Derlin 3 | Protein Coding | 31 | GC22M023834 | 1.031195879 |
| MARVELD3 | MARVEL Domain Containing 3 | Protein Coding | 30 | GC16P071626 | 1.031075478 |
| MRPL48 | Mitochondrial Ribosomal Protein L48 | Protein Coding | 31 | GC11P073787 | 1.031036854 |
| ICMT-DT | ICMT Divergent Transcript | RNA Gene | 13 | GC01P006240 | 1.030355692 |
| TAF5 | TATA-Box Binding Protein Associated Factor 5 | Protein Coding | 32 | GC10P103368 | 1.030282617 |
| MIR4677 | MicroRNA 4677 | RNA Gene | 13 | GC01P243346 | 1.029690742 |
| GNL1 | G Protein Nucleolar 1 (Putative) | Protein Coding | 32 | GC06M030541 | 1.029654622 |
| NPTXR | Neuronal Pentraxin Receptor | Protein Coding | 37 | GC22M038818 | 1.029567719 |
| ZNF44 | Zinc Finger Protein 44 | Protein Coding | 33 | GC19M012224 | 1.029456019 |
| PTTG3P | Pituitary Tumor-Transforming 3, Pseudogene | Pseudogene | 17 | GC08M066767 | 1.028740406 |
| MIR4465 | MicroRNA 4465 | RNA Gene | 9 | GC06P140683 | 1.028509378 |
| GIT2 | GIT ArfGAP 2 | Protein Coding | 36 | GC12M109929 | 1.028331757 |
| ZNF516 | Zinc Finger Protein 516 | Protein Coding | 30 | GC18M076357 | 1.028306484 |
| ITM2A | Integral Membrane Protein 2A | Protein Coding | 35 | GC0XM079360 | 1.028295994 |
| MIR520E | MicroRNA 520e | RNA Gene | 13 | GC19P064295 | 1.028191805 |
| MIR766 | MicroRNA 766 | RNA Gene | 17 | GC0XM119646 | 1.028118134 |
| PPP1R26 | Protein Phosphatase 1 Regulatory Subunit 26 | Protein Coding | 28 | GC09P135479 | 1.027475834 |
| ZBTB1 | Zinc Finger And BTB Domain Containing 1 | Protein Coding | 30 | GC14P064503 | 1.02708149 |
| LOC112543491 | Linc-UFC1 | RNA Gene | 5 | GC01P161315 | 1.026798487 |
| INO80C | INO80 Complex Subunit C | Protein Coding | 28 | GC18M035452 | 1.026609182 |
| RAB44 | RAB44, Member RAS Oncogene Family | Protein Coding | 25 | GC06P080536 | 1.026451349 |
| FCGR1BP | Fc Gamma Receptor Ib, Pseudogene | Pseudogene | 26 | GC01P121215 | 1.026438951 |
| HSBP1 | Heat Shock Factor Binding Protein 1 | Protein Coding | 31 | GC16P083719 | 1.026115775 |
| ACOT9 | Acyl-CoA Thioesterase 9 | Protein Coding | 34 | GC0XM023701 | 1.02512002 |
| PDZRN3 | PDZ Domain Containing Ring Finger 3 | Protein Coding | 30 | GC03M073382 | 1.024998903 |
| IP6K1 | Inositol Hexakisphosphate Kinase 1 | Protein Coding | 35 | GC03M051055 | 1.024878502 |
| MIR4740 | MicroRNA 4740 | RNA Gene | 11 | GC17M081400 | 1.024877548 |
| MIR4704 | MicroRNA 4704 | RNA Gene | 10 | GC13P066218 | 1.024877548 |
| ANAPC15 | Anaphase Promoting Complex Subunit 15 | Protein Coding | 27 | GC11M072106 | 1.024697304 |
| GMPR | Guanosine Monophosphate Reductase | Protein Coding | 38 | GC06P016238 | 1.024423599 |
| SYNGR3 | Synaptogyrin 3 | Protein Coding | 32 | GC16P001989 | 1.023662806 |
| COQ10B | Coenzyme Q10B | Protein Coding | 31 | GC02P197453 | 1.023662806 |
| STMN4 | Stathmin 4 | Protein Coding | 31 | GC08M027235 | 1.023104906 |
| TOGARAM2 | TOG Array Regulator Of Axonemal Microtubules 2 | Protein Coding | 22 | GC02P028956 | 1.023104906 |
| SRRM5 | Serine/Arginine Repetitive Matrix 5 | Protein Coding | 21 | GC19P063774 | 1.023104906 |
| IRF1-AS1 | IRF1 Antisense RNA 1 | RNA Gene | 20 | GC05P132420 | 1.023104906 |
| MEIKIN | Meiotic Kinetochore Factor | Protein Coding | 18 | GC05M131806 | 1.023104906 |
| PTPRVP | Protein Tyrosine Phosphatase Receptor Type V, Pseudogene | Pseudogene | 15 | GC01P202168 | 1.023104906 |
| LINC01376 | Long Intergenic Non-Protein Coding RNA 1376 | RNA Gene | 13 | GC02M018986 | 1.023104906 |
| MIR548H4 | MicroRNA 548h-4 | RNA Gene | 13 | GC08M027049 | 1.023104906 |
| ERICH6-AS1 | ERICH6 Antisense RNA 1 | RNA Gene | 12 | GC03P150703 | 1.023104906 |
| TRIM31-AS1 | TRIM31 Antisense RNA 1 | RNA Gene | 12 | GC06P080317 | 1.023104906 |
| LINC01487 | Long Intergenic Non-Protein Coding RNA 1487 | RNA Gene | 11 | GC03P155240 | 1.023104906 |
| LINC01921 | Long Intergenic Non-Protein Coding RNA 1921 | RNA Gene | 11 | GC02P216859 | 1.023104906 |
| LINC02068 | Long Intergenic Non-Protein Coding RNA 2068 | RNA Gene | 11 | GC03M172562 | 1.023104906 |
| ZNF225-AS1 | ZNF225 And ZNF224 Antisense RNA 1 | RNA Gene | 11 | GC19M065507 | 1.023104906 |
| LINC01395 | Long Intergenic Non-Protein Coding RNA 1395 | RNA Gene | 10 | GC11M129593 | 1.023104906 |
| MIR7847 | MicroRNA 7847 | RNA Gene | 10 | GC11P001890 | 1.023104906 |
| RPL23AP53 | Ribosomal Protein L23a Pseudogene 53 | Pseudogene | 10 | GC08M000208 | 1.023104906 |
| SIAH2-AS1 | SIAH2 Antisense RNA 1 | RNA Gene | 10 | GC03P150761 | 1.023104906 |
| ZNF230-DT | ZNF230 Divergent Transcript | RNA Gene | 10 | GC19M065508 | 1.023104906 |
| LINC02863 | Long Intergenic Non-Protein Coding RNA 2863 | RNA Gene | 9 | GC05M132422 | 1.023104906 |
| LOC105373876 | Uncharacterized LOC105373876 | RNA Gene | 9 | GC02M216868 | 1.023104906 |
| ENSG00000202533 | Y RNA | RNA Gene | 7 | GC05P132468 | 1.023104906 |
| ENSG00000227021 | Novel Transcript | RNA Gene | 7 | GC02P216868 | 1.023104906 |
| ENSG00000231597 | Novel Transcript | RNA Gene | 7 | GC02M216854 | 1.023104906 |
| ENSG00000268601 | Novel Transcript | RNA Gene | 7 | GC19M043794 | 1.023104906 |
| ENSG00000283782 | Novel Protein | Protein Coding | 7 | GC05P132417 | 1.023104906 |
| PA2G4P3 | Proliferation-Associated 2G4 Pseudogene 3 | Pseudogene | 7 | GC18M027452 | 1.023104906 |
| RNU6-813P | RNA, U6 Small Nuclear 813, Pseudogene | Pseudogene | 7 | GC06M151615 | 1.023104906 |
| RN7SKP43 | RN7SK Pseudogene 43 | Pseudogene | 6 | GC02P217251 | 1.023104906 |
| RPS27P20 | Ribosomal Protein S27 Pseudogene 20 | Pseudogene | 6 | GC11P129537 | 1.023104906 |
| lnc-KCNN4-1 | | RNA Gene | 6 | GC19M063905 | 1.023104906 |
| ENSG00000176761 | Zinc Finger Protein 285B (Pseudogene) | Pseudogene | 5 | GC19P064561 | 1.023104906 |
| ENSG00000281386 | Novel Transcript | RNA Gene | 5 | GC11P129592 | 1.023104906 |

| | | | | | |
|---|---|---|---|---|---|
| HSALNG0054371 | | RNA Gene | 5 | GC06M151592 | 1.023104906 |
| HSALNG0068232 | | RNA Gene | 5 | GC08M123761 | 1.023104906 |
| HSALNG0111536 | | RNA Gene | 5 | GC16P053888 | 1.023104906 |
| HSALNG0126379 | | RNA Gene | 5 | GC19P043771 | 1.023104906 |
| NONHSAG007400.2 | | RNA Gene | 5 | GC11P001871 | 1.023104906 |
| SNODB1936 | | RNA Gene | 5 | GC01P202232 | 1.023104906 |
| lnc-ATM-1 | | RNA Gene | 5 | GC11P108498 | 1.023104906 |
| lnc-IRF1-1 | | RNA Gene | 5 | GC05M132473 | 1.023104906 |
| lnc-KCNS3-4 | | RNA Gene | 5 | GC02P019097 | 1.023104906 |
| lnc-OSR1-6 | | RNA Gene | 5 | GC02M018849 | 1.023104906 |
| lnc-RUFY4-6 | | RNA Gene | 5 | GC02P217509 | 1.023104906 |
| lnc-TNFSF10-3 | | RNA Gene | 5 | GC03M172552 | 1.023104906 |
| lnc-TNFSF10-4 | | RNA Gene | 5 | GC03M172553 | 1.023104906 |
| lnc-ULK3-1 | | RNA Gene | 5 | GC15M074853 | 1.023104906 |
| piR-34822-157 | | RNA Gene | 5 | GC19P065461 | 1.023104906 |
| piR-39348 | | RNA Gene | 5 | GC19M063906 | 1.023104906 |
| ENSG00000224431 | Cytoplasmic Linker Associated Protein 2 (CLASP2) Pseudogene | Pseudogene | 4 | GC05P132199 | 1.023104906 |
| HSALNG0009715 | | RNA Gene | 4 | GC01M202203 | 1.023104906 |
| HSALNG0022195 | | RNA Gene | 4 | GC02P217430 | 1.023104906 |
| HSALNG0030738 | | RNA Gene | 4 | GC03P172530 | 1.023104906 |
| HSALNG0030743 | | RNA Gene | 4 | GC03P172560 | 1.023104906 |
| HSALNG0044846 | | RNA Gene | 4 | GC05P132467 | 1.023104906 |
| HSALNG0053445 | | RNA Gene | 4 | GC06M130121 | 1.023104906 |
| HSALNG0085355 | | RNA Gene | 4 | GC11P069512 | 1.023104906 |
| HSALNG0103271 | | RNA Gene | 4 | GC14P092556 | 1.023104906 |
| HSALNG0103272 | | RNA Gene | 4 | GC14P092633 | 1.023104906 |
| LOC105375739 | Uncharacterized LOC105375739 | RNA Gene | 4 | GC08P123719 | 1.023104906 |
| MN298214 | | RNA Gene | 4 | GC05P132451 | 1.023104906 |
| NONHSAG025076.2 | | RNA Gene | 4 | GC19P065259 | 1.023104906 |
| RF00017-1680 | | RNA Gene | 4 | GC15P117462 | 1.023104906 |
| RF00026-961 | | RNA Gene | 4 | GC06M151622 | 1.023104906 |
| HSALNG0013911 | | RNA Gene | 3 | GC02P028896 | 1.023104906 |
| HSALNG0085360 | | RNA Gene | 3 | GC11P069531 | 1.023104906 |
| HSALNG0087003 | | RNA Gene | 3 | GC11P108474 | 1.023104906 |
| HSALNG0088162 | | RNA Gene | 3 | GC11M129572 | 1.023104906 |
| HSALNG0107224 | | RNA Gene | 3 | GC15P115675 | 1.023104906 |
| HSALNG0124497 | | RNA Gene | 3 | GC19P064628 | 1.023104906 |
| HSALNG0124634 | | RNA Gene | 3 | GC19P064669 | 1.023104906 |
| LOC107986528 | Uncharacterized LOC107986528 | RNA Gene | 3 | GC06M151610 | 1.023104906 |
| RF00017-1520 | | RNA Gene | 3 | GC14M101123 | 1.023104906 |
| RF00017-5476 | | RNA Gene | 3 | GC06P152138 | 1.023104906 |
| piR-36588-081 | | RNA Gene | 3 | GC11M129540 | 1.023104906 |
| piR-37972-377 | | RNA Gene | 3 | GC02P019162 | 1.023104906 |
| piR-38562-049 | | RNA Gene | 3 | GC18M027824 | 1.023104906 |
| piR-43083-065 | | RNA Gene | 3 | GC06P151553 | 1.023104906 |
| piR-44085-013 | | RNA Gene | 3 | GC02P028904 | 1.023104906 |
| piR-36756-046 | | RNA Gene | 2 | GC18M027823 | 1.023104906 |
| MTFR1 | Mitochondrial Fission Regulator 1 | Protein Coding | 33 | GC08P065643 | 1.02282095 |
| MIR1229 | MicroRNA 1229 | RNA Gene | 13 | GC05M179798 | 1.022789717 |
| ADAMTSL5 | ADAMTS Like 5 | Protein Coding | 32 | GC19M004880 | 1.022751689 |
| CENPO | Centromere Protein O | Protein Coding | 31 | GC02P024793 | 1.022661805 |
| GCM1 | Glial Cells Missing Transcription Factor 1 | Protein Coding | 34 | GC06M064010 | 1.022593021 |
| MIR3187 | MicroRNA 3187 | RNA Gene | 13 | GC19P000813 | 1.022505879 |
| RGS20 | Regulator Of G Protein Signaling 20 | Protein Coding | 36 | GC08P053851 | 1.022265434 |
| TBC1D2B | TBC1 Domain Family Member 2B | Protein Coding | 32 | GC15M077984 | 1.022265434 |
| FBXO16 | F-Box Protein 16 | Protein Coding | 27 | GC08M028348 | 1.022265434 |
| MOAP1 | Modulator Of Apoptosis 1 | Protein Coding | 32 | GC14M093182 | 1.02167511 |
| MED9 | Mediator Complex Subunit 9 | Protein Coding | 30 | GC17P017476 | 1.021109581 |
| SLBP | Stem-Loop Binding Protein | Protein Coding | 32 | GC04M001692 | 1.021073341 |
| TACR2 | Tachykinin Receptor 2 | Protein Coding | 40 | GC10M069403 | 1.020657539 |
| MIS18BP1 | MIS18 Binding Protein 1 | Protein Coding | 28 | GC14M045203 | 1.020315886 |
| TRUB1 | TruB Pseudouridine Synthase Family Member 1 | Protein Coding | 33 | GC10P114938 | 1.020125985 |
| SPC24 | SPC24 Component Of NDC80 Kinetochore Complex | Protein Coding | 30 | GC19M011131 | 1.019843698 |
| VPS50 | VPS50 Subunit Of EARP/GARPII Complex | Protein Coding | 29 | GC07P093233 | 1.019226909 |
| ABITRAM | Actin Binding Transcription Modulator | Protein Coding | 25 | GC09P108935 | 1.019226909 |
| GIP | Gastric Inhibitory Polypeptide | Protein Coding | 37 | GC17M048958 | 1.019155741 |
| PTPN18 | Protein Tyrosine Phosphatase Non-Receptor Type 18 | Protein Coding | 35 | GC02P130356 | 1.019019604 |
| SLCO5A1 | Solute Carrier Organic Anion Transporter Family Member 5A1 | Protein Coding | 31 | GC08M069667 | 1.018711805 |
| PUM3 | Pumilio RNA Binding Family Member 3 | Protein Coding | 27 | GC09M002721 | 1.018524647 |
| JOSD1 | Josephin Domain Containing 1 | Protein Coding | 32 | GC22M056100 | 1.018460751 |
| WARS2 | Tryptophanyl TRNA Synthetase 2, Mitochondrial | Protein Coding | 40 | GC01M119031 | 1.018238664 |
| MAP4K1 | Mitogen-Activated Protein Kinase Kinase Kinase Kinase 1 | Protein Coding | 39 | GC19M038587 | 1.018167496 |
| TRMT11 | TRNA Methyltransferase 11 Homolog | Protein Coding | 34 | GC06P125986 | 1.017976284 |
| NUP58 | Nucleoporin 58 | Protein Coding | 28 | GC13P025397 | 1.017976284 |
| SEMA6C | Semaphorin 6C | Protein Coding | 34 | GC01M151131 | 1.01773262 |
| COX16 | Cytochrome C Oxidase Assembly Factor COX16 | Protein Coding | 31 | GC14M070326 | 1.017688513 |
| KLHL12 | Kelch Like Family Member 12 | Protein Coding | 34 | GC01M202891 | 1.017445803 |
| TMEM115 | Transmembrane Protein 115 | Protein Coding | 32 | GC03M050354 | 1.017439961 |
| SAYSD1 | SAYSVFN Motif Domain Containing 1 | Protein Coding | 27 | GC06M063775 | 1.017320871 |
| ANKHD1-EIF4EBP3 | ANKHD1-EIF4EBP3 Readthrough | Protein Coding | 18 | GC05P145793 | 1.017320871 |
| STX18-AS1 | STX18 Antisense RNA 1 (Head To Head) | RNA Gene | 14 | GC04P004546 | 1.016974926 |
| DCDC2C | Doublecortin Domain Containing 2C | Protein Coding | 23 | GC02P003703 | 1.016962767 |
| AGAP14P | ArfGAP With GTPase Domain, Ankyrin Repeat And PH Domain 14, Pseudogene | Pseudogene | 6 | GC10P046534 | 1.016962767 |
| HOXB4 | Homeobox B4 | Protein Coding | 36 | GC17M048575 | 1.01692605 |
| COMMD4 | COMM Domain Containing 4 | Protein Coding | 31 | GC15P114790 | 1.016337395 |
| ALG2 | ALG2 Alpha-1,3/1,6-Mannosyltransferase | Protein Coding | 37 | GC09M099216 | 1.016064167 |
| HPCAL1 | Hippocalcin Like 1 | Protein Coding | 36 | GC02P010302 | 1.015712261 |
| APEX2 | Apurinic/Apyrimidinic Endodeoxyribonuclease 2 | Protein Coding | 32 | GC0XP055000 | 1.014662027 |
| FAM183BP | Family With Sequence Similarity 183 Member B, Pseudogene | Pseudogene | 18 | GC07M039041 | 1.014424801 |
| HCP5B | HLA Complex P5B | RNA Gene | 11 | GC06M064174 | 1.014424801 |
| MICD | MHC Class I Polypeptide-Related Sequence D (Pseudogene) | Pseudogene | 11 | GC06M063506 | 1.014424801 |
| ETF1P1 | Eukaryotic Translation Termination Factor 1 Pseudogene 1 | Pseudogene | 10 | GC06P080315 | 1.014424801 |
| LINC02676 | Long Intergenic Non-Protein Coding RNA 2676 | RNA Gene | 9 | GC10P008726 | 1.014424801 |
| RPL3P2 | Ribosomal Protein L3 Pseudogene 2 | Pseudogene | 9 | GC06P031280 | 1.014424801 |
| ENSG00000265194 | Novel Transcript, Antisense MTAP | RNA Gene | 7 | GC09M021860 | 1.014424801 |

| | | | | | |
|---|---|---|---|---|---|
| ENSG00000271380 | Novel Transcript, Antisense To SHC1 And PYGO2 | Uncategorized | 7 | GC01P154961 | 1.014424801 |
| PAIP1P1 | PAIP1 Pseudogene 1 | Pseudogene | 7 | GC06M030186 | 1.014424801 |
| lnc-CDKN2B-2-002 | | RNA Gene | 6 | GC09M021995 | 1.014424801 |
| ENSG00000230521 | HLA Complex Group 4 Pseudogene 7 | Pseudogene | 5 | GC06M063497 | 1.014424801 |
| ENSG00000271267 | ATP Synthase, H+ Transporting, Mitochondrial Fo Complex, Subunit F2 (ATP5J2) Pseudogene | Pseudogene | 5 | GC01M155568 | 1.014424801 |
| HSALNG0070395 | | RNA Gene | 5 | GC09M021990 | 1.014424801 |
| lnc-CDKN2B-2-001 | | RNA Gene | 5 | GC09M021989 | 1.014424801 |
| MESP2 | Mesoderm Posterior BHLH Transcription Factor 2 | Protein Coding | 34 | GC15P115254 | 1.014359355 |
| PRRC2B | Proline Rich Coiled-Coil 2B | Protein Coding | 29 | GC09P131373 | 1.013683796 |
| DTX3 | Deltex E3 Ubiquitin Ligase 3 | Protein Coding | 30 | GC12P057604 | 1.013580322 |
| TMCC3 | Transmembrane And Coiled-Coil Domain Family 3 | Protein Coding | 31 | GC12M094567 | 1.013476133 |
| NUB1 | Negative Regulator Of Ubiquitin Like Proteins 1 | Protein Coding | 34 | GC07P151341 | 1.013415933 |
| MIR3145 | MicroRNA 3145 | RNA Gene | 15 | GC06M138435 | 1.013403654 |
| CLPTM1 | CLPTM1 Regulator Of GABA Type A Receptor Forward Trafficking | Protein Coding | 34 | GC19P044954 | 1.013288379 |
| WASHC4 | WASH Complex Subunit 4 | Protein Coding | 27 | GC12P105108 | 1.013236523 |
| LIPT1 | Lipoyltransferase 1 | Protein Coding | 36 | GC02P099191 | 1.013206959 |
| GPR89A | G Protein-Coupled Receptor 89A | Protein Coding | 29 | GC01P145607 | 1.012802005 |
| COX7C | Cytochrome C Oxidase Subunit 7C | Protein Coding | 32 | GC05P086617 | 1.012645721 |
| SZT2 | SZT2 Subunit Of KICSTOR Complex | Protein Coding | 32 | GC01P043389 | 1.012526393 |
| PLEKHO2 | Pleckstrin Homology Domain Containing O2 | Protein Coding | 31 | GC15P117441 | 1.012526274 |
| MAN1A2 | Mannosidase Alpha Class 1A Member 2 | Protein Coding | 39 | GC01P117367 | 1.012508631 |
| FRA6E | Fragile Site, Aphidicolin Type, Common, Fra(6)(Q26) | Uncategorized | 5 | GC06U990032 | 1.012264848 |
| MOCS2 | Molybdenum Cofactor Synthesis 2 | Protein Coding | 37 | GC05M053095 | 1.011606574 |
| CCDC71L | Coiled-Coil Domain Containing 71 Like | Protein Coding | 25 | GC07M106656 | 1.011567116 |
| MIR4282 | MicroRNA 4282 | RNA Gene | 9 | GC06M072967 | 1.011518002 |
| ME3 | Malic Enzyme 3 | Protein Coding | 36 | GC11M087833 | 1.010330439 |
| CEP126 | Centrosomal Protein 126 | Protein Coding | 27 | GC11P101916 | 1.010330439 |
| NPIPB5 | Nuclear Pore Complex Interacting Protein Family Member B5 | Protein Coding | 21 | GC16P022479 | 1.009876847 |
| MFAP3 | Microfibril Associated Protein 3 | Protein Coding | 34 | GC05P154014 | 1.009770036 |
| KIAA2026 | KIAA2026 | Protein Coding | 25 | GC09M005861 | 1.009717345 |
| MUC20 | Mucin 20, Cell Surface Associated | Protein Coding | 34 | GC03P195720 | 1.009225249 |
| GDAP2 | Ganglioside Induced Differentiation Associated Protein 2 | Protein Coding | 32 | GC01M117863 | 1.009165287 |
| PRR18 | Proline Rich 18 | Protein Coding | 25 | GC06M166305 | 1.008834481 |
| CADM2 | Cell Adhesion Molecule 2 | Protein Coding | 35 | GC03P085008 | 1.00843215 |
| WDR83 | WD Repeat Domain 83 | Protein Coding | 32 | GC19P012666 | 1.00843215 |
| TMEM130 | Transmembrane Protein 130 | Protein Coding | 28 | GC07M098846 | 1.008369088 |
| SUN1 | Sad1 And UNC84 Domain Containing 1 | Protein Coding | 35 | GC07P000815 | 1.008276582 |
| NDUFS5 | NADH:Ubiquinone Oxidoreductase Subunit S5 | Protein Coding | 37 | GC01P039026 | 1.008032322 |
| NDUFB5 | NADH:Ubiquinone Oxidoreductase Subunit B5 | Protein Coding | 31 | GC03P179604 | 1.008032322 |
| CMTM1 | CKLF Like MARVEL Transmembrane Domain Containing 1 | Protein Coding | 31 | GC16P066566 | 1.007264137 |
| LOC102724023 | Glutamine Amidotransferase Class 1 Domain Containing 3B | Protein Coding | 5 | GC21M005132 | 1.007001162 |
| C1QTNF9 | C1q And TNF Related 9 | Protein Coding | 30 | GC13P024307 | 1.006945014 |
| MIR2467 | MicroRNA 2467 | RNA Gene | 13 | GC02M239352 | 1.006898403 |
| ZNF32 | Zinc Finger Protein 32 | Protein Coding | 29 | GC10M043643 | 1.006288886 |
| ZBTB40 | Zinc Finger And BTB Domain Containing 40 | Protein Coding | 33 | GC01P022428 | 1.006173611 |
| TTC39A | Tetratricopeptide Repeat Domain 39A | Protein Coding | 29 | GC01M051287 | 1.005800486 |
| ZDHHC11B | Zinc Finger DHHC-Type Containing 11B | Protein Coding | 23 | GC05M000710 | 1.005777121 |
| MBOAT2 | Membrane Bound O-Acyltransferase Domain Containing 2 | Protein Coding | 31 | GC02M008853 | 1.00568521 |
| EHD4 | EH Domain Containing 4 | Protein Coding | 33 | GC15M041895 | 1.005524516 |
| CCDC8 | Coiled-Coil Domain Containing 8 | Protein Coding | 35 | GC19M046410 | 1.005473137 |
| SLC35B4 | Solute Carrier Family 35 Member B4 | Protein Coding | 30 | GC07M134290 | 1.005385876 |
| H2BU1 | H2B.U Histone 1 | Protein Coding | 26 | GC01P229207 | 1.005385876 |
| VPS26B | VPS26, Retromer Complex Component B | Protein Coding | 31 | GC11P134224 | 1.005028009 |
| LOC109611593 | RUNX2 P1 Promoter Region | Biological Region | 2 | GC06P081805 | 1.004998922 |
| S100PBP | S100P Binding Protein | Protein Coding | 30 | GC01P032818 | 1.004155517 |
| MIR4675 | MicroRNA 4675 | RNA Gene | 10 | GC10P020551 | 1.003921986 |
| MIR4795 | MicroRNA 4795 | RNA Gene | 10 | GC03M087226 | 1.003921986 |
| CASKIN2 | CASK Interacting Protein 2 | Protein Coding | 29 | GC17M075500 | 1.003701925 |
| ARL6IP6 | ADP Ribosylation Factor Like GTPase 6 Interacting Protein 6 | Protein Coding | 30 | GC02P152717 | 1.003643274 |
| ZCCHC3 | Zinc Finger CCHC-Type Containing 3 | Protein Coding | 28 | GC20P000278 | 1.003445864 |
| BTG4 | BTG Anti-Proliferation Factor 4 | Protein Coding | 34 | GC11M111385 | 1.003196478 |
| CYB561D2 | Cytochrome B561 Family Member D2 | Protein Coding | 30 | GC03P050350 | 1.003163934 |
| AGAP6 | ArfGAP With GTPase Domain, Ankyrin Repeat And PH Domain 6 | Protein Coding | 23 | GC10P050175 | 1.002739787 |
| LYPLA1 | Lysophospholipase 1 | Protein Coding | 35 | GC08M054046 | 1.002488494 |
| REEP2 | Receptor Accessory Protein 2 | Protein Coding | 36 | GC05P138439 | 1.00232029 |
| ARNILA | Androgen Receptor Negatively Regulated LncRNA | RNA Gene | 7 | GC02M025370 | 1.002114415 |
| CHCHD7 | Coiled-Coil-Helix-Coiled-Coil-Helix Domain Containing 7 | Protein Coding | 31 | GC08P056211 | 1.001941323 |
| RPAIN | RPA Interacting Protein | Protein Coding | 31 | GC17P005419 | 1.0017066 |
| GUCY1B1 | Guanylate Cyclase 1 Soluble Subunit Beta 1 | Protein Coding | 33 | GC04P155759 | 1.001551628 |
| LAX1 | Lymphocyte Transmembrane Adaptor 1 | Protein Coding | 33 | GC01P203768 | 1.00149107 |
| RPL19P16 | Ribosomal Protein L19 Pseudogene 16 | Pseudogene | 6 | GC10M121133 | 1.001038551 |
| RGPD8 | RANBP2 Like And GRIP Domain Containing 8 | Protein Coding | 27 | GC02M112368 | 1.000893593 |
| ZDHHC5 | Zinc Finger DHHC-Type Palmitoyltransferase 5 | Protein Coding | 33 | GC11P057670 | 0.999980271 |
| SLC22A7 | Solute Carrier Family 22 Member 7 | Protein Coding | 38 | GC06P080596 | 0.999683619 |
| TUBGCP3 | Tubulin Gamma Complex Associated Protein 3 | Protein Coding | 34 | GC13M112485 | 0.999427319 |
| LINC01060 | Long Intergenic Non-Protein Coding RNA 1060 | RNA Gene | 13 | GC04P188372 | 0.99941951 |
| ZMYM1 | Zinc Finger MYM-Type Containing 1 | Protein Coding | 30 | GC01P035032 | 0.999155998 |
| MMGT1 | Membrane Magnesium Transporter 1 | Protein Coding | 30 | GC0XM135962 | 0.999125004 |
| WDR41 | WD Repeat Domain 41 | Protein Coding | 31 | GC05M077425 | 0.999123812 |
| SYT13 | Synaptotagmin 13 | Protein Coding | 31 | GC11M045240 | 0.99874872 |
| BEX1 | Brain Expressed X-Linked 1 | Protein Coding | 28 | GC0XM103063 | 0.99874872 |
| PDCL | Phosducin Like | Protein Coding | 36 | GC09M122798 | 0.998355985 |
| FBRS | Fibrosin | Protein Coding | 29 | GC16P040269 | 0.997929871 |
| PIDD1 | P53-Induced Death Domain Protein 1 | Protein Coding | 31 | GC11M002931 | 0.99779433 |
| SLC25A36 | Solute Carrier Family 25 Member 36 | Protein Coding | 35 | GC03P140941 | 0.997606039 |
| ZNRF2 | Zinc And Ring Finger 2 | Protein Coding | 30 | GC07P030284 | 0.997482598 |
| CMYA5 | Cardiomyopathy Associated 5 | Protein Coding | 30 | GC05P079689 | 0.997258604 |
| EFCAB7 | EF-Hand Calcium Binding Domain 7 | Protein Coding | 30 | GC01P063523 | 0.997258604 |
| OTP | Orthopedia Homeobox | Protein Coding | 32 | GC05M077628 | 0.997103691 |
| MRPL49 | Mitochondrial Ribosomal Protein L49 | Protein Coding | 31 | GC11P069510 | 0.996932626 |
| ASCL4 | Achaete-Scute Family BHLH Transcription Factor 4 | Protein Coding | 28 | GC12P107774 | 0.996826828 |
| DLX3 | Distal-Less Homeobox 3 | Protein Coding | 38 | GC17M049990 | 0.996728361 |
| ALDH1L2 | Aldehyde Dehydrogenase 1 Family Member L2 | Protein Coding | 36 | GC12M105019 | 0.996471882 |
| PLA2G4C | Phospholipase A2 Group IVC | Protein Coding | 38 | GC19M048047 | 0.996429682 |

| | | | | | |
|---|---|---|---|---|---|
| VKORC1L1 | Vitamin K Epoxide Reductase Complex Subunit 1 Like 1 | Protein Coding | 34 | GC07P069971 | 0.996052861 |
| LAYN | Layilin | Protein Coding | 32 | GC11P111697 | 0.995373607 |
| SSBP2 | Single Stranded DNA Binding Protein 2 | Protein Coding | 32 | GC05M081413 | 0.9942801 |
| GHRLOS | Ghrelin Opposite Strand/Antisense RNA | RNA Gene | 18 | GC03P012260 | 0.994102716 |
| ZSWIM6 | Zinc Finger SWIM-Type Containing 6 | Protein Coding | 32 | GC05P061332 | 0.993942499 |
| TM9SF3 | Transmembrane 9 Superfamily Member 3 | Protein Coding | 33 | GC10M096518 | 0.993932664 |
| VBP1 | VHL Binding Protein 1 | Protein Coding | 32 | GC0XP155197 | 0.993932664 |
| ARL17A | ADP Ribosylation Factor Like GTPase 17A | Protein Coding | 26 | GC17M048366 | 0.993873537 |
| NOX3 | NADPH Oxidase 3 | Protein Coding | 37 | GC06M155395 | 0.993669212 |
| FDX1 | Ferredoxin 1 | Protein Coding | 36 | GC11P110429 | 0.993578732 |
| HACD2 | 3-Hydroxyacyl-CoA Dehydratase 2 | Protein Coding | 30 | GC03M123490 | 0.992770076 |
| GBA3 | Glucosylceramidase Beta 3 (Gene/Pseudogene) | Protein Coding | 31 | GC04P022694 | 0.992208898 |
| USP42 | Ubiquitin Specific Peptidase 42 | Protein Coding | 33 | GC07P006078 | 0.992184639 |
| SLC44A2 | Solute Carrier Family 44 Member 2 | Protein Coding | 38 | GC19P010602 | 0.991659582 |
| RNF34 | Ring Finger Protein 34 | Protein Coding | 35 | GC12P121400 | 0.991416097 |
| HELB | DNA Helicase B | Protein Coding | 30 | GC12P066302 | 0.991168678 |
| PPIC | Peptidylprolyl Isomerase C | Protein Coding | 37 | GC05M123023 | 0.990968943 |
| POLE3 | DNA Polymerase Epsilon 3, Accessory Subunit | Protein Coding | 36 | GC09M113407 | 0.990844309 |
| IYD | Iodotyrosine Deiodinase | Protein Coding | 38 | GC06P150368 | 0.99075532 |
| PARP12 | Poly(ADP-Ribose) Polymerase Family Member 12 | Protein Coding | 34 | GC07M140023 | 0.990729511 |
| STAC2 | SH3 And Cysteine Rich Domain 2 | Protein Coding | 33 | GC17M041521 | 0.99012953 |
| SAPCD1 | Suppressor APC Domain Containing 1 | Protein Coding | 25 | GC06P080371 | 0.989296257 |
| CTDSP2 | CTD Small Phosphatase 2 | Protein Coding | 35 | GC12M057819 | 0.988338053 |
| UQCRH | Ubiquinol-Cytochrome C Reductase Hinge Protein | Protein Coding | 34 | GC01P046303 | 0.987463951 |
| SORBS2 | Sorbin And SH3 Domain Containing 2 | Protein Coding | 36 | GC04M185585 | 0.987321079 |
| GFUS | GDP-L-Fucose Synthase | Protein Coding | 32 | GC08M143660 | 0.986961126 |
| CFLAR-AS1 | CFLAR Antisense RNA 1 | RNA Gene | 16 | GC02M201140 | 0.986892581 |
| DYRK4 | Dual Specificity Tyrosine Phosphorylation Regulated Kinase 4 | Protein Coding | 35 | GC12P019788 | 0.986883223 |
| PRDM9 | PR/SET Domain 9 | Protein Coding | 34 | GC05P023443 | 0.986860275 |
| MIR3131 | MicroRNA 3131 | RNA Gene | 12 | GC02M219058 | 0.986624479 |
| COX18 | Cytochrome C Oxidase Assembly Factor COX18 | Protein Coding | 31 | GC04M073054 | 0.986285806 |
| JPH1 | Junctophilin 1 | Protein Coding | 35 | GC08M074234 | 0.986104369 |
| PMPCB | Peptidase, Mitochondrial Processing Subunit Beta | Protein Coding | 39 | GC07P103297 | 0.985638499 |
| MMRN1 | Multimerin 1 | Protein Coding | 36 | GC04P089879 | 0.985506654 |
| MIR519C | MicroRNA 519c | RNA Gene | 17 | GC19P053686 | 0.985055566 |
| NADK | NAD Kinase | Protein Coding | 37 | GC01M001751 | 0.984443188 |
| MIR1185-2 | MicroRNA 1185-2 | RNA Gene | 14 | GC14P109510 | 0.984319806 |
| MIR1204 | MicroRNA 1204 | RNA Gene | 13 | GC08P127795 | 0.984139681 |
| MRS2 | Magnesium Transporter MRS2 | Protein Coding | 34 | GC06P024402 | 0.984078705 |
| SYF2 | SYF2 Pre-MRNA Splicing Factor | Protein Coding | 29 | GC01M025222 | 0.984078705 |
| RABGAP1 | RAB GTPase Activating Protein 1 | Protein Coding | 32 | GC09P122932 | 0.983904183 |
| TGFBRAP1 | Transforming Growth Factor Beta Receptor Associated Protein 1 | Protein Coding | 31 | GC02M105250 | 0.983904183 |
| SDE2 | SDE2 Telomere Maintenance Homolog | Protein Coding | 24 | GC01M225982 | 0.983904183 |
| MRPL54 | Mitochondrial Ribosomal Protein L54 | Protein Coding | 31 | GC19P003762 | 0.983712435 |
| ALKAL2 | ALK And LTK Ligand 2 | Protein Coding | 22 | GC02M000279 | 0.98365891 |
| ERVK3-3 | Endogenous Retrovirus Group K3 Member 3 | Uncategorized | 3 | GC05U901567 | 0.982997239 |
| ERVK3-4 | Endogenous Retrovirus Group K3 Member 4 | Uncategorized | 3 | GC11U901879 | 0.982997239 |
| ERVK3-5 | Endogenous Retrovirus Group K3 Member 5 | Uncategorized | 3 | GC12U901587 | 0.982997239 |
| ERVK3-6 | Endogenous Retrovirus Group K3 Member 6 | Uncategorized | 3 | GC19U901553 | 0.982997239 |
| ERVK3-7 | Endogenous Retrovirus Group K3 Member 7 | Uncategorized | 3 | GC19U901557 | 0.982997239 |
| ERVK3-8 | Endogenous Retrovirus Group K3 Member 8 | Uncategorized | 3 | GC20U900687 | 0.982997239 |
| TMEM44-AS1 | TMEM44 Antisense RNA 1 | RNA Gene | 13 | GC03P194584 | 0.982458413 |
| MIR3130-2 | MicroRNA 3130-2 | RNA Gene | 13 | GC02P206783 | 0.982442856 |
| CA13 | Carbonic Anhydrase 13 | Protein Coding | 34 | GC08P085221 | 0.981755733 |
| SPOUT1 | SPOUT Domain Containing Methyltransferase 1 | Protein Coding | 28 | GC09M128820 | 0.981528997 |
| TM2D3 | TM2 Domain Containing 3 | Protein Coding | 31 | GC15M101621 | 0.981490672 |
| GPLD1 | Glycosylphosphatidylinositol Specific Phospholipase D1 | Protein Coding | 39 | GC06M024484 | 0.981202185 |
| MRPL55 | Mitochondrial Ribosomal Protein L55 | Protein Coding | 30 | GC01M228202 | 0.981121302 |
| LILRA4 | Leukocyte Immunoglobulin Like Receptor A4 | Protein Coding | 36 | GC19M054333 | 0.980800867 |
| GLTP | Glycolipid Transfer Protein | Protein Coding | 32 | GC12M109850 | 0.98052758 |
| SRSF8 | Serine And Arginine Rich Splicing Factor 8 | Protein Coding | 26 | GC11P095066 | 0.980404496 |
| RABEP2 | Rabaptin, RAB GTPase Binding Effector Protein 2 | Protein Coding | 30 | GC16M036343 | 0.980189145 |
| TCEAL2 | Transcription Elongation Factor A Like 2 | Protein Coding | 27 | GC0XP102125 | 0.980156422 |
| FBXO38 | F-Box Protein 38 | Protein Coding | 34 | GC05P148383 | 0.979713202 |
| NKIRAS2 | NFKB Inhibitor Interacting Ras Like 2 | Protein Coding | 35 | GC17P042011 | 0.979554951 |
| LINC00702 | Long Intergenic Non-Protein Coding RNA 702 | RNA Gene | 13 | GC10M004201 | 0.979457378 |
| HS6ST3 | Heparan Sulfate 6-O-Sulfotransferase 3 | Protein Coding | 32 | GC13P096090 | 0.979055285 |
| ESCO1 | Establishment Of Sister Chromatid Cohesion N-Acetyltransferase 1 | Protein Coding | 34 | GC18M021529 | 0.978832364 |
| AFD1 | Acrofacial Dysostosis 1, Nager Type | Genetic Locus | 2 | GC09U990001 | 0.978807032 |
| G2E3 | G2/M-Phase Specific E3 Ubiquitin Protein Ligase | Protein Coding | 34 | GC14P030559 | 0.978743792 |
| DENND4A | DENN Domain Containing 4A | Protein Coding | 33 | GC15M081195 | 0.978703737 |
| MICALL2 | MICAL Like 2 | Protein Coding | 31 | GC07M001613 | 0.978668392 |
| MIR520F | MicroRNA 520f | RNA Gene | 13 | GC19P053682 | 0.978668392 |
| POLR2J2 | RNA Polymerase II Subunit J2 | Protein Coding | 26 | GC07M103119 | 0.978517592 |
| CRIPT | CXXC Repeat Containing Interactor Of PDZ3 Domain | Protein Coding | 35 | GC02P046581 | 0.978487611 |
| RAMP2-AS1 | RAMP2 Antisense RNA 1 | RNA Gene | 15 | GC17M042794 | 0.978384078 |
| RNASE7 | Ribonuclease A Family Member 7 | Protein Coding | 31 | GC14P021042 | 0.978359044 |
| UBL7 | Ubiquitin Like 7 | Protein Coding | 30 | GC15M081435 | 0.978290021 |
| PPRC1 | PPARG Related Coactivator 1 | Protein Coding | 32 | GC10P102161 | 0.978208005 |
| MIR1226 | MicroRNA 1226 | RNA Gene | 15 | GC03P047849 | 0.978141665 |
| OR4D2 | Olfactory Receptor Family 4 Subfamily D Member 2 | Protein Coding | 30 | GC17P058169 | 0.978053272 |
| PUSL1 | Pseudouridine Synthase Like 1 | Protein Coding | 28 | GC01P001308 | 0.977703571 |
| TBC1D30 | TBC1 Domain Family Member 30 | Protein Coding | 28 | GC12P064789 | 0.977703571 |
| COA4 | Cytochrome C Oxidase Assembly Factor 4 Homolog | Protein Coding | 28 | GC11M087567 | 0.977644145 |
| ISY1 | ISY1 Splicing Factor Homolog | Protein Coding | 28 | GC03M129127 | 0.977200508 |
| TEX2 | Testis Expressed 2 | Protein Coding | 30 | GC17M064147 | 0.977119684 |
| PRPF39 | Pre-MRNA Processing Factor 39 | Protein Coding | 32 | GC14P045084 | 0.97694248 |
| SLC25A15 | Solute Carrier Family 25 Member 15 | Protein Coding | 39 | GC13P040789 | 0.976840079 |
| CRYBB1 | Crystallin Beta B1 | Protein Coding | 34 | GC22M026599 | 0.976442635 |
| GSC | Goosecoid Homeobox | Protein Coding | 38 | GC14M094768 | 0.975675583 |
| ADGRB2 | Adhesion G Protein-Coupled Receptor B2 | Protein Coding | 32 | GC01M031727 | 0.975513577 |
| MIR4729 | MicroRNA 4729 | RNA Gene | 12 | GC17P059366 | 0.975115418 |
| FOLH1B | Folate Hydrolase 1B | Protein Coding | 26 | GC11P089638 | 0.975055575 |
| LOC117600004 | CTSL Promoter Region | Biological Region | 2 | GC09P087724 | 0.974948347 |
| PALMD | Palmdelphin | Protein Coding | 30 | GC01P099646 | 0.974193573 |

| | | | | | |
|---|---|---|---|---|---|
| MIR4454 | MicroRNA 4454 | RNA Gene | 11 | GC04M163093 | 0.973937035 |
| SCNM1 | Sodium Channel Modifier 1 | Protein Coding | 28 | GC01P151168 | 0.973750532 |
| SORCS3 | Sortilin Related VPS10 Domain Containing Receptor 3 | Protein Coding | 32 | GC10P104642 | 0.973451257 |
| PRB1 | Proline Rich Protein BstNI Subfamily 1 | Protein Coding | 28 | GC12M020466 | 0.972635388 |
| NUFIP1 | Nuclear FMR1 Interacting Protein 1 | Protein Coding | 31 | GC13M044939 | 0.97211659 |
| CNTNAP4 | Contactin Associated Protein Family Member 4 | Protein Coding | 33 | GC16P076278 | 0.971601129 |
| TMA7 | Translation Machinery Associated 7 Homolog | Protein Coding | 24 | GC03P048440 | 0.971372068 |
| SAR1B | Secretion Associated Ras Related GTPase 1B | Protein Coding | 39 | GC05M134601 | 0.971299291 |
| IL27RA | Interleukin 27 Receptor Subunit Alpha | Protein Coding | 36 | GC19P014031 | 0.971285284 |
| SELENOM | Selenoprotein M | Protein Coding | 26 | GC22M036192 | 0.971113801 |
| TADA1 | Transcriptional Adaptor 1 | Protein Coding | 28 | GC01M166826 | 0.971089125 |
| TBCB | Tubulin Folding Cofactor B | Protein Coding | 35 | GC19P063475 | 0.97092247 |
| EEPD1 | Endonuclease/Exonuclease/Phosphatase Family Domain Containing 1 | Protein Coding | 30 | GC07P036153 | 0.970877409 |
| SLC35D1 | Solute Carrier Family 35 Member D1 | Protein Coding | 36 | GC01M066999 | 0.970375478 |
| LINC01857 | Long Intergenic Non-Protein Coding RNA 1857 | RNA Gene | 11 | GC02P207663 | 0.970158875 |
| LASTR | LncRNA Associated With SART3 Regulation Of Splicing | RNA Gene | 11 | GC10M005597 | 0.969915986 |
| CGRRF1 | Cell Growth Regulator With Ring Finger Domain 1 | Protein Coding | 31 | GC14P054509 | 0.969741106 |
| ARL4D | ADP Ribosylation Factor Like GTPase 4D | Protein Coding | 32 | GC17P043398 | 0.969651222 |
| MRM1 | Mitochondrial RRNA Methyltransferase 1 | Protein Coding | 32 | GC17P052844 | 0.969356358 |
| NRAD1 | Non-Coding RNA In The Aldehyde Dehydrogenase 1A Pathway | RNA Gene | 13 | GC13P043910 | 0.969356358 |
| LINC02862 | Long Intergenic Non-Protein Coding RNA 2862 | RNA Gene | 7 | GC02P215276 | 0.969356358 |
| SACS-AS1 | SACS Antisense RNA 1 | RNA Gene | 14 | GC13P023418 | 0.969096899 |
| RAB5B | RAB5B, Member RAS Oncogene Family | Protein Coding | 37 | GC12P055973 | 0.969073176 |
| SMOC1 | SPARC Related Modular Calcium Binding 1 | Protein Coding | 37 | GC14P069854 | 0.968961358 |
| TSNAX | Translin Associated Factor X | Protein Coding | 35 | GC01P231529 | 0.968956947 |
| KIR2DS2 | Killer Cell Immunoglobulin Like Receptor, Two Ig Domains And Short Cytoplasmic Tail 2 | Protein Coding | 24 | GC19MR00122 | 0.968956947 |
| CCS | Copper Chaperone For Superoxide Dismutase | Protein Coding | 38 | GC11P066593 | 0.968773007 |
| ADGRE2 | Adhesion G Protein-Coupled Receptor E2 | Protein Coding | 36 | GC19M014733 | 0.968508959 |
| SAMD12 | Sterile Alpha Motif Domain Containing 12 | Protein Coding | 33 | GC08M118131 | 0.968290091 |
| ATXN3L | Ataxin 3 Like | Protein Coding | 30 | GC0XM013336 | 0.968289375 |
| MIR1255B1 | MicroRNA 1255b-1 | RNA Gene | 11 | GC04M036426 | 0.967680871 |
| MIR1255B2 | MicroRNA 1255b-2 | RNA Gene | 10 | GC01P167998 | 0.967680871 |
| YJU2B | YJU2 Splicing Factor Homolog B | Protein Coding | 24 | GC19P014133 | 0.967567384 |
| EGID-7795 | Methylation Modifier For Class I HLA | Genetic Locus | 1 | GC01U901621 | 0.967489958 |
| LSM10 | LSM10, U7 Small Nuclear RNA Associated | Protein Coding | 31 | GC01M036391 | 0.967366099 |
| OTUD1 | OTU Deubiquitinase 1 | Protein Coding | 28 | GC10P023439 | 0.96716702 |
| PARD3B | Par-3 Family Cell Polarity Regulator Beta | Protein Coding | 34 | GC02P204545 | 0.96702975 |
| MARCHF5 | Membrane Associated Ring-CH-Type Finger 5 | Protein Coding | 31 | GC10P092297 | 0.966959894 |
| MIDEAS | Mitotic Deacetylase Associated SANT Domain Protein | Protein Coding | 23 | GC14M073832 | 0.966880798 |
| CLVS1 | Clavesin 1 | Protein Coding | 32 | GC08P060966 | 0.966303587 |
| ZNF665 | Zinc Finger Protein 665 | Protein Coding | 28 | GC19M053159 | 0.966303587 |
| FAM227B | Family With Sequence Similarity 227 Member B | Protein Coding | 25 | GC15M049326 | 0.966303587 |
| LINC00917 | Long Intergenic Non-Protein Coding RNA 917 | RNA Gene | 14 | GC16M086366 | 0.966303587 |
| TSPAN6 | Tetraspanin 6 | Protein Coding | 34 | GC0XM100627 | 0.966001689 |
| RTL8C | Retrotransposon Gag Like 8C | Protein Coding | 24 | GC0XP135033 | 0.965179503 |
| FLYWCH1 | FLYWCH-Type Zinc Finger 1 | Protein Coding | 28 | GC16P002911 | 0.965088725 |
| TPPP3 | Tubulin Polymerization Promoting Protein Family Member 3 | Protein Coding | 33 | GC16M067389 | 0.96471566 |
| MVB12B | Multivesicular Body Subunit 12B | Protein Coding | 30 | GC09P126326 | 0.964541376 |
| MINDY2 | MINDY Lysine 48 Deubiquitinase 2 | Protein Coding | 27 | GC15P058772 | 0.964470267 |
| SIGLEC12 | Sialic Acid Binding Ig Like Lectin 12 | Protein Coding | 35 | GC19M051491 | 0.963926733 |
| DRAM1 | DNA Damage Regulated Autophagy Modulator 1 | Protein Coding | 30 | GC12P101877 | 0.963139713 |
| GTPBP3 | GTP Binding Protein 3, Mitochondrial | Protein Coding | 36 | GC19P063105 | 0.962989807 |
| CDIN1 | CDAN1 Interacting Nuclease 1 | Protein Coding | 28 | GC15P039709 | 0.962559938 |
| RBM34 | RNA Binding Motif Protein 34 | Protein Coding | 29 | GC01M235131 | 0.962484777 |
| LYSMD3 | LysM Domain Containing 3 | Protein Coding | 28 | GC05M090515 | 0.962142467 |
| NT5DC1 | 5'-Nucleotidase Domain Containing 1 | Protein Coding | 31 | GC06P116100 | 0.961892128 |
| SHISA3 | Shisa Family Member 3 | Protein Coding | 30 | GC04P042399 | 0.961892128 |
| MIR1298 | MicroRNA 1298 | RNA Gene | 12 | GC0XP114715 | 0.961892128 |
| MIR4644 | MicroRNA 4644 | RNA Gene | 10 | GC06P170330 | 0.96174705 |
| DOK4 | Docking Protein 4 | Protein Coding | 34 | GC16M057505 | 0.961527586 |
| DCAF11 | DDB1 And CUL4 Associated Factor 11 | Protein Coding | 29 | GC14P024114 | 0.96147871 |
| MIR4756 | MicroRNA 4756 | RNA Gene | 11 | GC20M054068 | 0.961430967 |
| MIR645 | MicroRNA 645 | RNA Gene | 11 | GC20P050585 | 0.960248172 |
| MRPL36 | Mitochondrial Ribosomal Protein L36 | Protein Coding | 32 | GC05M001799 | 0.960228562 |
| ACP2 | Acid Phosphatase 2, Lysosomal | Protein Coding | 39 | GC11M086878 | 0.959808409 |
| SINHCAF | SIN3-HDAC Complex Associated Factor | Protein Coding | 26 | GC12M031283 | 0.959395111 |
| EIF3CL | Eukaryotic Translation Initiation Factor 3 Subunit C Like | Protein Coding | 24 | GC16M028384 | 0.959300935 |
| MIR1281 | MicroRNA 1281 | RNA Gene | 13 | GC22P041092 | 0.959300935 |
| ZNF740 | Zinc Finger Protein 740 | Protein Coding | 29 | GC12P053180 | 0.959068596 |
| AXDND1 | Axonemal Dynein Light Chain Domain Containing 1 | Protein Coding | 26 | GC01P179366 | 0.958077013 |
| C12orf43 | Chromosome 12 Open Reading Frame 43 | Protein Coding | 28 | GC12M121000 | 0.957558274 |
| DUSP16 | Dual Specificity Phosphatase 16 | Protein Coding | 36 | GC12M012473 | 0.957557797 |
| TSEN2 | TRNA Splicing Endonuclease Subunit 2 | Protein Coding | 36 | GC03P012484 | 0.957480311 |
| HGH1 | HGH1 Homolog | Protein Coding | 25 | GC08P144137 | 0.957362711 |
| SNED1 | Sushi, Nidogen And EGF Like Domains 1 | Protein Coding | 32 | GC02P240998 | 0.957246959 |
| NUDT2 | Nudix Hydrolase 2 | Protein Coding | 36 | GC09P034329 | 0.957126141 |
| MRPL10 | Mitochondrial Ribosomal Protein L10 | Protein Coding | 32 | GC17M048536 | 0.956992984 |
| ZNF767P | Zinc Finger Family Member 767, Pseudogene | Pseudogene | 14 | GC07M149547 | 0.956992984 |
| PLPPR5 | Phospholipid Phosphatase Related 5 | Protein Coding | 27 | GC01M098891 | 0.956720114 |
| SLC10A7 | Solute Carrier Family 10 Member 7 | Protein Coding | 34 | GC04M146253 | 0.956554294 |
| IL17RE | Interleukin 17 Receptor E | Protein Coding | 35 | GC03P012176 | 0.956220627 |
| NPC2 | NPC Intracellular Cholesterol Transporter 2 | Protein Coding | 38 | GC14M074476 | 0.956046522 |
| CD200R1 | CD200 Receptor 1 | Protein Coding | 35 | GC03M112921 | 0.955893278 |
| SUMO1P1 | SUMO1 Pseudogene 1 | Pseudogene | 15 | GC20M053874 | 0.955689907 |
| ZG16 | Zymogen Granule Protein 16 | Protein Coding | 29 | GC16P029760 | 0.955522239 |
| ZNF197 | Zinc Finger Protein 197 | Protein Coding | 28 | GC03P046346 | 0.954691768 |
| SPINT1-AS1 | SPINT1 Antisense RNA 1 | RNA Gene | 11 | GC15M040836 | 0.9546628 |
| WNK4 | WNK Lysine Deficient Protein Kinase 4 | Protein Coding | 41 | GC17P053178 | 0.954606593 |
| ZKSCAN5 | Zinc Finger With KRAB And SCAN Domains 5 | Protein Coding | 32 | GC07P099504 | 0.954082608 |
| PAMR1 | Peptidase Domain Containing Associated With Muscle Regeneration 1 | Protein Coding | 32 | GC11M035453 | 0.95372957 |
| C6orf15 | Chromosome 6 Open Reading Frame 15 | Protein Coding | 28 | GC06M031111 | 0.953646064 |
| BBOX1-AS1 | BBOX1 Antisense RNA 1 | RNA Gene | 12 | GC11M027047 | 0.953600764 |
| SNTG1 | Syntrophin Gamma 1 | Protein Coding | 32 | GC08P049909 | 0.953368425 |
| CYP2F1 | Cytochrome P450 Family 2 Subfamily F Member 1 | Protein Coding | 38 | GC19P041114 | 0.953243732 |

| | | | | | |
|---|---|---|---|---|---|
| TM4SF4 | Transmembrane 4 L Six Family Member 4 | Protein Coding | 31 | GC03P149473 | 0.952800393 |
| SIDT1 | SID1 Transmembrane Family Member 1 | Protein Coding | 31 | GC03P113532 | 0.951387644 |
| TYSND1 | Trypsin Like Peroxisomal Matrix Peptidase 1 | Protein Coding | 32 | GC10M070137 | 0.951342702 |
| NEUROG2 | Neurogenin 2 | Protein Coding | 34 | GC04M112513 | 0.95129925 |
| PISD | Phosphatidylserine Decarboxylase | Protein Coding | 42 | GC22M036242 | 0.950976968 |
| SFXN3 | Sideroflexin 3 | Protein Coding | 34 | GC10P101031 | 0.950720906 |
| EMC2 | ER Membrane Protein Complex Subunit 2 | Protein Coding | 28 | GC08P108443 | 0.950372934 |
| MIR3622A | MicroRNA 3622a | RNA Gene | 13 | GC08P027701 | 0.950372934 |
| MIR4784 | MicroRNA 4784 | RNA Gene | 10 | GC02M131491 | 0.950372934 |
| LINC00629 | Long Intergenic Non-Protein Coding RNA 629 | RNA Gene | 14 | GC0XP134549 | 0.950344324 |
| CNOT6L | CCR4-NOT Transcription Complex Subunit 6 Like | Protein Coding | 30 | GC04M077713 | 0.950239182 |
| LILRB3 | Leukocyte Immunoglobulin Like Receptor B3 | Protein Coding | 35 | GC19M054216 | 0.949751496 |
| NBEAL1 | Neurobeachin Like 1 | Protein Coding | 31 | GC02P203042 | 0.949709594 |
| RNF138 | Ring Finger Protein 138 | Protein Coding | 35 | GC18P032091 | 0.949603856 |
| ARHGEF17 | Rho Guanine Nucleotide Exchange Factor 17 | Protein Coding | 32 | GC11P073306 | 0.949603856 |
| FARS2 | Phenylalanyl-TRNA Synthetase 2, Mitochondrial | Protein Coding | 38 | GC06P005261 | 0.949569821 |
| CRELD1 | Cysteine Rich With EGF Like Domains 1 | Protein Coding | 37 | GC03P012219 | 0.94930023 |
| RABGGTB | Rab Geranylgeranyltransferase Subunit Beta | Protein Coding | 36 | GC01P075786 | 0.949249923 |
| C8orf44-SGK3 | C8orf44-SGK3 Readthrough | Protein Coding | 17 | GC08P066731 | 0.949106872 |
| ASMTL-AS1 | ASMTL Antisense RNA 1 | RNA Gene | 14 | GC0XP001400 | 0.949106872 |
| CC2D2B | Coiled-Coil And C2 Domain Containing 2B | Protein Coding | 29 | GC10P095975 | 0.949000001 |
| TLCD5 | TLC Domain Containing 5 | Protein Coding | 23 | GC11P120324 | 0.949000001 |
| MIR631 | MicroRNA 631 | RNA Gene | 15 | GC15M075353 | 0.948642492 |
| PMS2P3 | PMS1 Homolog 2, Mismatch Repair System Component Pseudogene 3 | Pseudogene | 19 | GC07M075507 | 0.94836545 |
| KCNC3 | Potassium Voltage-Gated Channel Subfamily C Member 3 | Protein Coding | 41 | GC19M064182 | 0.947863936 |
| TMEM25 | Transmembrane Protein 25 | Protein Coding | 32 | GC11P118531 | 0.947789013 |
| TMEM87A | Transmembrane Protein 87A | Protein Coding | 31 | GC15M042210 | 0.947789013 |
| MIR892B | MicroRNA 892b | RNA Gene | 11 | GC0XM146072 | 0.947789013 |
| MIR5694 | MicroRNA 5694 | RNA Gene | 9 | GC14M067441 | 0.947789013 |
| SNAI3-AS1 | SNAI3 Antisense RNA 1 | RNA Gene | 14 | GC16P088663 | 0.947586477 |
| ASB10 | Ankyrin Repeat And SOCS Box Containing 10 | Protein Coding | 34 | GC07M151175 | 0.947204053 |
| CDC42EP5 | CDC42 Effector Protein 5 | Protein Coding | 30 | GC19M054465 | 0.947104633 |
| NT5C | 5', 3'-Nucleotidase, Cytosolic | Protein Coding | 37 | GC17M075130 | 0.946498752 |
| ACAD8 | Acyl-CoA Dehydrogenase Family Member 8 | Protein Coding | 39 | GC11P134253 | 0.946432412 |
| HTR7 | 5-Hydroxytryptamine Receptor 7 | Protein Coding | 42 | GC10M090740 | 0.946288168 |
| ZDHHC6 | Zinc Finger DHHC-Type Palmitoyltransferase 6 | Protein Coding | 34 | GC10M112430 | 0.946230888 |
| ACOT8 | Acyl-CoA Thioesterase 8 | Protein Coding | 34 | GC20M045841 | 0.946152151 |
| SLC35A3 | Solute Carrier Family 35 Member A3 | Protein Coding | 38 | GC01P099968 | 0.946133673 |
| HPCA | Hippocalcin | Protein Coding | 38 | GC01P032885 | 0.946119308 |
| ZNF789 | Zinc Finger Protein 789 | Protein Coding | 26 | GC07P099472 | 0.94529891 |
| CCDC9 | Coiled-Coil Domain Containing 9 | Protein Coding | 26 | GC19P047255 | 0.945193708 |
| KLKP1 | Kallikrein Pseudogene 1 | Pseudogene | 13 | GC19M064213 | 0.944804311 |
| MTRF1 | Mitochondrial Translation Release Factor 1 | Protein Coding | 33 | GC13M041216 | 0.944447279 |
| TTYH2 | Tweety Family Member 2 | Protein Coding | 32 | GC17P074215 | 0.944287896 |
| PSTPIP2 | Proline-Serine-Threonine Phosphatase Interacting Protein 2 | Protein Coding | 32 | GC18M045983 | 0.94335556 |
| TIGD1 | Tigger Transposable Element Derived 1 | Protein Coding | 28 | GC02M232547 | 0.943144143 |
| RSRC1 | Arginine And Serine Rich Coiled-Coil 1 | Protein Coding | 35 | GC03P158105 | 0.942952096 |
| DHRS7B | Dehydrogenase/Reductase 7B | Protein Coding | 31 | GC17P052375 | 0.94284445 |
| SLC25A30 | Solute Carrier Family 25 Member 30 | Protein Coding | 31 | GC13M045393 | 0.94275558 |
| HOXC13-AS | HOXC13 Antisense RNA | RNA Gene | 15 | GC12M053935 | 0.942754328 |
| CYP2G1P | Cytochrome P450 Family 2 Subfamily G Member 1, Pseudogene | Pseudogene | 18 | GC19P040890 | 0.942446351 |
| ENSG00000228162 | Novel Transcript | RNA Gene | 7 | GC02P234438 | 0.942446351 |
| ENSG00000260586 | Novel Transcript, Intronic To THSD4 | RNA Gene | 7 | GC15P114682 | 0.942446351 |
| CCDC85C | Coiled-Coil Domain Containing 85C | Protein Coding | 29 | GC14M099500 | 0.942252517 |
| CLK4 | CDC Like Kinase 4 | Protein Coding | 36 | GC05M178602 | 0.941207707 |
| MTCH1 | Mitochondrial Carrier 1 | Protein Coding | 34 | GC06M063764 | 0.941207707 |
| MIR570 | MicroRNA 570 | RNA Gene | 16 | GC03P196191 | 0.941207707 |
| CDC42EP2 | CDC42 Effector Protein 2 | Protein Coding | 33 | GC11P069535 | 0.940938473 |
| INPP5A | Inositol Polyphosphate-5-Phosphatase A | Protein Coding | 37 | GC10P132537 | 0.940449357 |
| NSUN6 | NOP2/Sun RNA Methyltransferase 6 | Protein Coding | 34 | GC10M018540 | 0.940449357 |
| SSH1 | Slingshot Protein Phosphatase 1 | Protein Coding | 36 | GC12M108784 | 0.939983606 |
| ZNRF1 | Zinc And Ring Finger 1 | Protein Coding | 35 | GC16P075033 | 0.939946592 |
| ST3GAL5 | ST3 Beta-Galactoside Alpha-2,3-Sialyltransferase 5 | Protein Coding | 42 | GC02M085839 | 0.939738214 |
| TMEM150A | Transmembrane Protein 150A | Protein Coding | 29 | GC02M085626 | 0.939637303 |
| TMEM199 | Transmembrane Protein 199 | Protein Coding | 31 | GC17P052480 | 0.939587653 |
| CDCA4 | Cell Division Cycle Associated 4 | Protein Coding | 32 | GC14M105009 | 0.939403772 |
| SPRING1 | SREBF Pathway Regulator In Golgi 1 | Protein Coding | 25 | GC12M116712 | 0.939403772 |
| SFT2D1 | SFT2 Domain Containing 1 | Protein Coding | 28 | GC06M166319 | 0.939074278 |
| C2orf92 | Chromosome 2 Open Reading Frame 92 | Protein Coding | 11 | GC02P097739 | 0.939074278 |
| OPALIN | Oligodendrocytic Myelin Paranodal And Inner Loop Protein | Protein Coding | 30 | GC10M096343 | 0.938959837 |
| ZMYND15 | Zinc Finger MYND-Type Containing 15 | Protein Coding | 30 | GC17P004740 | 0.938959837 |
| FHIP2A | FHF Complex Subunit HOOK Interacting Protein 2A | Protein Coding | 25 | GC10P114871 | 0.938959837 |
| NRDE2 | NRDE-2, Necessary For RNA Interference, Domain Containing | Protein Coding | 25 | GC14M100409 | 0.938843071 |
| MSL3P1 | MSL Complex Subunit 3 Pseudogene 1 | Pseudogene | 18 | GC02M233866 | 0.938843071 |
| LINC01287 | Long Intergenic Non-Protein Coding RNA 1287 | RNA Gene | 11 | GC07M153399 | 0.938843071 |
| FKBP14 | FKBP Prolyl Isomerase 14 | Protein Coding | 36 | GC07M030010 | 0.938805282 |
| TCEANC | Transcription Elongation Factor A N-Terminal And Central Domain Containing | Protein Coding | 26 | GC0XP013652 | 0.938663065 |
| RGS11 | Regulator Of G Protein Signaling 11 | Protein Coding | 36 | GC16M000268 | 0.938658595 |
| GNPDA2 | Glucosamine-6-Phosphate Deaminase 2 | Protein Coding | 34 | GC04M044684 | 0.9386549 |
| DNAJC11 | DnaJ Heat Shock Protein Family (Hsp40) Member C11 | Protein Coding | 31 | GC01M006634 | 0.938119352 |
| MIR4521 | MicroRNA 4521 | RNA Gene | 13 | GC17P010919 | 0.938119352 |
| MIR2682 | MicroRNA 2682 | RNA Gene | 13 | GC01M098045 | 0.938084662 |
| MIR4735 | MicroRNA 4735 | RNA Gene | 10 | GC01M196555 | 0.938084662 |
| LRFN4 | Leucine Rich Repeat And Fibronectin Type III Domain Containing 4 | Protein Coding | 32 | GC11P066856 | 0.938004613 |
| SLC52A1 | Solute Carrier Family 52 Member 1 | Protein Coding | 35 | GC17M005032 | 0.937853038 |
| EPB41L4A | Erythrocyte Membrane Protein Band 4.1 Like 4A | Protein Coding | 31 | GC05M112142 | 0.937760532 |
| LRRC42 | Leucine Rich Repeat Containing 42 | Protein Coding | 27 | GC01P053946 | 0.937760532 |
| MIR3651 | MicroRNA 3651 | RNA Gene | 16 | GC09M092292 | 0.937760532 |
| RNF125 | Ring Finger Protein 125 | Protein Coding | 36 | GC18P032144 | 0.937468886 |
| TBC1D23 | TBC1 Domain Family Member 23 | Protein Coding | 32 | GC03P100260 | 0.937251925 |
| SNX29 | Sorting Nexin 29 | Protein Coding | 30 | GC16P011976 | 0.937251925 |
| ZNF319 | Zinc Finger Protein 319 | Protein Coding | 28 | GC16M057994 | 0.937155664 |
| PHF24 | PHD Finger Protein 24 | Protein Coding | 26 | GC09P034810 | 0.937126279 |
| HAS2-AS1 | HAS2 Antisense RNA 1 | RNA Gene | 18 | GC08P121639 | 0.936865866 |
| SOWAHC | Sosondowah Ankyrin Repeat Domain Family Member C | Protein Coding | 24 | GC02P109614 | 0.936812162 |

| | | | | | |
|---|---|---|---|---|---|
| PACRG | Parkin Coregulated | Protein Coding | 35 | GC06P162727 | 0.936491191 |
| NUTF2 | Nuclear Transport Factor 2 | Protein Coding | 34 | GC16P067846 | 0.936263144 |
| PLLP | Plasmolipin | Protein Coding | 32 | GC16M057290 | 0.936221898 |
| MAN2B2 | Mannosidase Alpha Class 2B Member 2 | Protein Coding | 35 | GC04P006575 | 0.935905159 |
| GEMIN6 | Gem Nuclear Organelle Associated Protein 6 | Protein Coding | 33 | GC02P038751 | 0.935905159 |
| RTCA | RNA 3'-Terminal Phosphate Cyclase | Protein Coding | 32 | GC01P100266 | 0.935905159 |
| IFNA17 | Interferon Alpha 17 | Protein Coding | 32 | GC09M021227 | 0.935783625 |
| OSTF1 | Osteoclast Stimulating Factor 1 | Protein Coding | 35 | GC09P075088 | 0.935632467 |
| MLXIP | MLX Interacting Protein | Protein Coding | 30 | GC12P122078 | 0.934776187 |
| ACOX3 | Acyl-CoA Oxidase 3, Pristanoyl | Protein Coding | 36 | GC04M008380 | 0.934660673 |
| GTDC1 | Glycosyltransferase Like Domain Containing 1 | Protein Coding | 32 | GC02M143938 | 0.9346205 |
| TMCO3 | Transmembrane And Coiled-Coil Domains 3 | Protein Coding | 31 | GC13P113490 | 0.934587717 |
| PDE12 | Phosphodiesterase 12 | Protein Coding | 32 | GC03P057556 | 0.934392631 |
| BSN | Bassoon Presynaptic Cytomatrix Protein | Protein Coding | 32 | GC03P049554 | 0.934333503 |
| RGPD3 | RANBP2 Like And GRIP Domain Containing 3 | Protein Coding | 26 | GC02M106373 | 0.933875084 |
| PACERR | PTGS2 Antisense NFKB1 Complex-Mediated Expression Regulator RNA | RNA Gene | 13 | GC01P186680 | 0.9338305 |
| ABCF3 | ATP Binding Cassette Subfamily F Member 3 | Protein Coding | 33 | GC03P184186 | 0.933819592 |
| AKAP11 | A-Kinase Anchoring Protein 11 | Protein Coding | 34 | GC13P042272 | 0.933093071 |
| MAMDC2 | MAM Domain Containing 2 | Protein Coding | 32 | GC09P070043 | 0.932953238 |
| EPS8L2 | EPS8 Like 2 | Protein Coding | 35 | GC11P000694 | 0.932583928 |
| DDA1 | DET1 And DDB1 Associated 1 | Protein Coding | 30 | GC19P063099 | 0.932583928 |
| CENPN | Centromere Protein N | Protein Coding | 32 | GC16P081006 | 0.932419181 |
| HAUS5 | HAUS Augmin Like Complex Subunit 5 | Protein Coding | 30 | GC19P063452 | 0.932330668 |
| MIR2116 | MicroRNA 2116 | RNA Gene | 14 | GC15M081081 | 0.932330668 |
| SPOCK3 | SPARC (Osteonectin), Cwcv And Kazal Like Domains Proteoglycan 3 | Protein Coding | 33 | GC04M166733 | 0.932076335 |
| RPL21P4 | Ribosomal Protein L21 Pseudogene 4 | Pseudogene | 6 | GC17P043079 | 0.931793332 |
| ZNF131 | Zinc Finger Protein 131 | Protein Coding | 32 | GC05P044230 | 0.931631565 |
| MIR623 | MicroRNA 623 | RNA Gene | 12 | GC13P099356 | 0.931251764 |
| CYTH1 | Cytohesin 1 | Protein Coding | 36 | GC17M078674 | 0.931238353 |
| PLEKHA5 | Pleckstrin Homology Domain Containing A5 | Protein Coding | 34 | GC12P019129 | 0.931179225 |
| MOB4 | MOB Family Member 4, Phocein | Protein Coding | 31 | GC02P197515 | 0.931179225 |
| SERAC1 | Serine Active Site Containing 1 | Protein Coding | 32 | GC06M158109 | 0.930491626 |
| HMX2 | H6 Family Homeobox 2 | Protein Coding | 30 | GC10P123142 | 0.930182219 |
| MATN2 | Matrilin 2 | Protein Coding | 35 | GC08P097868 | 0.929985106 |
| TMEM176B | Transmembrane Protein 176B | Protein Coding | 30 | GC07M150791 | 0.929372847 |
| FXYD1 | FXYD Domain Containing Ion Transport Regulator 1 | Protein Coding | 34 | GC19P063445 | 0.929368854 |
| KIF9 | Kinesin Family Member 9 | Protein Coding | 32 | GC03M047228 | 0.929034114 |
| ZSCAN9 | Zinc Finger And SCAN Domain Containing 9 | Protein Coding | 30 | GC06P028224 | 0.929034114 |
| PCDHB3 | Protocadherin Beta 3 | Protein Coding | 30 | GC05P145812 | 0.928465009 |
| UTP6 | UTP6 Small Subunit Processome Component | Protein Coding | 33 | GC17M031860 | 0.928423166 |
| TRIML2 | Tripartite Motif Family Like 2 | Protein Coding | 31 | GC04M188091 | 0.928174913 |
| BSPRY | B-Box And SPRY Domain Containing | Protein Coding | 31 | GC09P113349 | 0.928135812 |
| GEMIN7 | Gem Nuclear Organelle Associated Protein 7 | Protein Coding | 31 | GC19P045075 | 0.928135812 |
| KLC4 | Kinesin Light Chain 4 | Protein Coding | 32 | GC06P043040 | 0.927947223 |
| GORAB | Golgin, RAB6 Interacting | Protein Coding | 34 | GC01P170501 | 0.927835643 |
| KRBA1 | KRAB-A Domain Containing 1 | Protein Coding | 26 | GC07P149714 | 0.927683353 |
| AGAP5 | ArfGAP With GTPase Domain, Ankyrin Repeat And PH Domain 5 | Protein Coding | 25 | GC10M073899 | 0.927683353 |
| MIR323B | MicroRNA 323b | RNA Gene | 15 | GC14P109518 | 0.927683353 |
| ERN2 | Endoplasmic Reticulum To Nucleus Signaling 2 | Protein Coding | 34 | GC16M023690 | 0.927122593 |
| GRAMD2B | GRAM Domain Containing 2B | Protein Coding | 26 | GC05P126361 | 0.926994681 |
| BAHCC1 | BAH Domain And Coiled-Coil Containing 1 | Protein Coding | 25 | GC17P081395 | 0.926618278 |
| CHML | CHM Like Rab Escort Protein | Protein Coding | 34 | GC01M241628 | 0.926470935 |
| RABAC1 | Rab Acceptor 1 | Protein Coding | 31 | GC19M041956 | 0.926195502 |
| XPO6 | Exportin 6 | Protein Coding | 34 | GC16M028097 | 0.926005483 |
| PCYOX1L | Prenylcysteine Oxidase 1 Like | Protein Coding | 31 | GC05P149358 | 0.925943434 |
| MAN1A1 | Mannosidase Alpha Class 1A Member 1 | Protein Coding | 37 | GC06M119269 | 0.925844133 |
| FBRSL1 | Fibrosin Like 1 | Protein Coding | 28 | GC12P132489 | 0.925543129 |
| RNF39 | Ring Finger Protein 39 | Protein Coding | 31 | GC06M063507 | 0.925490737 |
| DEFB106A | Defensin Beta 106A | Protein Coding | 25 | GC08P007825 | 0.925325155 |
| DEFB106B | Defensin Beta 106B | Protein Coding | 23 | GC08M007482 | 0.925325155 |
| ZNF189 | Zinc Finger Protein 189 | Protein Coding | 32 | GC09P101398 | 0.924860477 |
| BCRP1 | BCR Pseudogene 1 | Pseudogene | 8 | GC22M036426 | 0.92426759 |
| FAM133B | Family With Sequence Similarity 133 Member B | Protein Coding | 26 | GC07M092560 | 0.923941195 |
| NAPA | NSF Attachment Protein Alpha | Protein Coding | 33 | GC19M064034 | 0.923913836 |
| SPDYA | Speedy/RINGO Cell Cycle Regulator Family Member A | Protein Coding | 31 | GC02P028782 | 0.923877895 |
| KLHL17 | Kelch Like Family Member 17 | Protein Coding | 30 | GC01P000960 | 0.923522294 |
| TRMT6 | TRNA Methyltransferase 6 Non-Catalytic Subunit | Protein Coding | 30 | GC20M005937 | 0.923454881 |
| UTP11 | UTP11 Small Subunit Processome Component | Protein Coding | 28 | GC01P038234 | 0.923122406 |
| BMF | Bcl2 Modifying Factor | Protein Coding | 34 | GC15M040087 | 0.922363043 |
| NXPH4 | Neurexophilin 4 | Protein Coding | 32 | GC12P057222 | 0.922222733 |
| KRI1 | KRI1 Homolog | Protein Coding | 29 | GC19M010553 | 0.921537161 |
| MIR633 | MicroRNA 633 | RNA Gene | 13 | GC17P062944 | 0.921537161 |
| ERVK-24 | Endogenous Retrovirus Group K Member 24 | Protein Coding | 9 | GC22U900840 | 0.921393275 |
| MIR3193 | MicroRNA 3193 | RNA Gene | 14 | GC20P031607 | 0.921291113 |
| ERVK-9 | Endogenous Retrovirus Group K Member 9 | Protein Coding | 7 | GC06U902225 | 0.920665622 |
| ANKIB1 | Ankyrin Repeat And IBR Domain Containing 1 | Protein Coding | 29 | GC07P092246 | 0.920614481 |
| PCGF3 | Polycomb Group Ring Finger 3 | Protein Coding | 30 | GC04P000734 | 0.920541346 |
| NDOR1 | NADPH Dependent Diflavin Oxidoreductase 1 | Protein Coding | 34 | GC09P137205 | 0.920508504 |
| SH3GL3 | SH3 Domain Containing GRB2 Like 3, Endophilin A3 | Protein Coding | 37 | GC15P083447 | 0.920454562 |
| BRK1 | BRICK1 Subunit Of SCAR/WAVE Actin Nucleating Complex | Protein Coding | 32 | GC03P010115 | 0.920450568 |
| PIGB | Phosphatidylinositol Glycan Anchor Biosynthesis Class B | Protein Coding | 38 | GC15P055318 | 0.920249879 |
| RRN3 | RRN3 Homolog, RNA Polymerase I Transcription Factor | Protein Coding | 33 | GC16M015060 | 0.920042336 |
| ARHGAP19 | Rho GTPase Activating Protein 19 | Protein Coding | 32 | GC10M097222 | 0.920042336 |
| PPP4R2 | Protein Phosphatase 4 Regulatory Subunit 2 | Protein Coding | 31 | GC03P072996 | 0.920042336 |
| CARD19 | Caspase Recruitment Domain Family Member 19 | Protein Coding | 28 | GC09P093097 | 0.919788659 |
| ZNF573 | Zinc Finger Protein 573 | Protein Coding | 28 | GC19M037735 | 0.919738829 |
| LOC107980440 | ABL Breakpoint Recombination Region | Biological Region | 2 | GC09P130710 | 0.91966182 |
| GAL3ST2 | Galactose-3-O-Sulfotransferase 2 | Protein Coding | 30 | GC02P241777 | 0.919610858 |
| MAPT-AS1 | MAPT Antisense RNA 1 | RNA Gene | 17 | GC17M045799 | 0.919340611 |
| LSM5 | LSM5 Homolog, U6 Small Nuclear RNA And MRNA Degradation Associated | Protein Coding | 32 | GC07M032485 | 0.919323444 |
| CES1P1 | Carboxylesterase 1 Pseudogene 1 | Pseudogene | 18 | GC16P055795 | 0.919129312 |
| GEMIN8 | Gem Nuclear Organelle Associated Protein 8 | Protein Coding | 32 | GC0XM014002 | 0.918758631 |
| CCDC66 | Coiled-Coil Domain Containing 66 | Protein Coding | 29 | GC03P056558 | 0.918663561 |
| ALDH16A1 | Aldehyde Dehydrogenase 16 Family Member A1 | Protein Coding | 31 | GC19P049453 | 0.918372869 |
| MIR4446 | MicroRNA 4446 | RNA Gene | 12 | GC03P113594 | 0.918048143 |

| | | | | | |
|---|---|---|---|---|---|
| CEP57L1 | Centrosomal Protein 57 Like 1 | Protein Coding | 30 | GC06P109095 | 0.917835593 |
| ZNF507 | Zinc Finger Protein 507 | Protein Coding | 31 | GC19P032345 | 0.917773783 |
| CCDC15 | Coiled-Coil Domain Containing 15 | Protein Coding | 27 | GC11P124954 | 0.917637944 |
| ZNF137P | Zinc Finger Protein 137, Pseudogene | Pseudogene | 17 | GC19P052588 | 0.917637944 |
| LMCD1-AS1 | LMCD1 Antisense RNA 1 | RNA Gene | 13 | GC03M007996 | 0.917637944 |
| NSL1 | NSL1 Component Of MIS12 Kinetochore Complex | Protein Coding | 32 | GC01M212726 | 0.917240858 |
| LOC110973015 | NOS3 5' Regulatory Region | Biological Region | 2 | GC07P150988 | 0.917052865 |
| GPHA2 | Glycoprotein Hormone Subunit Alpha 2 | Protein Coding | 34 | GC11M087229 | 0.916583002 |
| ASIC5 | Acid Sensing Ion Channel Subunit Family Member 5 | Protein Coding | 30 | GC04M155829 | 0.916583002 |
| ZNF37A | Zinc Finger Protein 37A | Protein Coding | 31 | GC10P038091 | 0.915976882 |
| PDE4C | Phosphodiesterase 4C | Protein Coding | 39 | GC19M018237 | 0.915752947 |
| SULT1C2 | Sulfotransferase Family 1C Member 2 | Protein Coding | 37 | GC02P108288 | 0.915748477 |
| MCOLN3 | Mucolipin TRP Cation Channel 3 | Protein Coding | 35 | GC01M085019 | 0.915748477 |
| STXBP5L | Syntaxin Binding Protein 5L | Protein Coding | 31 | GC03P120908 | 0.915748477 |
| IGLC2 | Immunoglobulin Lambda Constant 2 | Protein Coding | 18 | GC22P022900 | 0.915748477 |
| ANO2 | Anoctamin 2 | Protein Coding | 34 | GC12M005532 | 0.915743828 |
| PODXL2 | Podocalyxin Like 2 | Protein Coding | 32 | GC03P127629 | 0.915395081 |
| CCDC102A | Coiled-Coil Domain Containing 102A | Protein Coding | 29 | GC16M057512 | 0.915395081 |
| BNIPL | BCL2 Interacting Protein Like | Protein Coding | 30 | GC01P151036 | 0.91535306 |
| OPN4 | Opsin 4 | Protein Coding | 37 | GC10P091492 | 0.914735973 |
| SLC36A1 | Solute Carrier Family 36 Member 1 | Protein Coding | 35 | GC05P151413 | 0.91439271 |
| ZNF804B | Zinc Finger Protein 804B | Protein Coding | 29 | GC07P088759 | 0.914342165 |
| GPAT4 | Glycerol-3-Phosphate Acyltransferase 4 | Protein Coding | 31 | GC08P041577 | 0.914157867 |
| PADI1 | Peptidyl Arginine Deiminase 1 | Protein Coding | 34 | GC01P017205 | 0.913921297 |
| ACADSB | Acyl-CoA Dehydrogenase Short/Branched Chain | Protein Coding | 40 | GC10P123008 | 0.913288951 |
| CIAO1 | Cytosolic Iron-Sulfur Assembly Component 1 | Protein Coding | 36 | GC02P096283 | 0.912836432 |
| MIR6838 | MicroRNA 6838 | RNA Gene | 8 | GC07M044073 | 0.912723839 |
| PWWP2A | PWWP Domain Containing 2A | Protein Coding | 31 | GC05M160061 | 0.91243875 |
| BEND3 | BEN Domain Containing 3 | Protein Coding | 30 | GC06M107340 | 0.912163556 |
| MLYCD | Malonyl-CoA Decarboxylase | Protein Coding | 38 | GC16P083899 | 0.91211766 |
| GPRC5B | G Protein-Coupled Receptor Class C Group 5 Member B | Protein Coding | 34 | GC16M019856 | 0.911512852 |
| BEX3 | Brain Expressed X-Linked 3 | Protein Coding | 28 | GC0XP103377 | 0.910882354 |
| CRPPA | CDP-L-Ribitol Pyrophosphorylase A | Protein Coding | 30 | GC07M016088 | 0.91072011 |
| SLC38A5 | Solute Carrier Family 38 Member 5 | Protein Coding | 32 | GC0XM048458 | 0.910711646 |
| CELF6 | CUGBP Elav-Like Family Member 6 | Protein Coding | 30 | GC15M081355 | 0.910711646 |
| ACOT7 | Acyl-CoA Thioesterase 7 | Protein Coding | 35 | GC01M006265 | 0.910228968 |
| FETUB | Fetuin B | Protein Coding | 33 | GC03P186635 | 0.909732521 |
| ZNF446 | Zinc Finger Protein 446 | Protein Coding | 30 | GC19P058474 | 0.909732521 |
| ANKRD13D | Ankyrin Repeat Domain 13D | Protein Coding | 29 | GC11P069702 | 0.909732521 |
| ZNF584 | Zinc Finger Protein 584 | Protein Coding | 28 | GC19P064536 | 0.909732521 |
| MIR4711 | MicroRNA 4711 | RNA Gene | 11 | GC01M059733 | 0.909551859 |
| MPPE1 | Metallophosphoesterase 1 | Protein Coding | 34 | GC18M024009 | 0.90942657 |
| FMO2 | Flavin Containing Dimethylaniline Monoxygenase 2 | Protein Coding | 38 | GC01P171185 | 0.909272194 |
| CDS2 | CDP-Diacylglycerol Synthase 2 | Protein Coding | 35 | GC20P005126 | 0.90916723 |
| CLTB | Clathrin Light Chain B | Protein Coding | 35 | GC05M176392 | 0.90916723 |
| DEDD | Death Effector Domain Containing | Protein Coding | 33 | GC01M161120 | 0.90916723 |
| TMEM256 | Transmembrane Protein 256 | Protein Coding | 27 | GC17M007402 | 0.90916723 |
| CHCHD6 | Coiled-Coil-Helix-Coiled-Coil-Helix Domain Containing 6 | Protein Coding | 31 | GC03P126704 | 0.909129679 |
| HCAR3 | Hydroxycarboxylic Acid Receptor 3 | Protein Coding | 34 | GC12M122714 | 0.908830523 |
| HSD17B14 | Hydroxysteroid 17-Beta Dehydrogenase 14 | Protein Coding | 32 | GC19M048813 | 0.908830523 |
| C1orf53 | Chromosome 1 Open Reading Frame 53 | Protein Coding | 26 | GC01P197871 | 0.908625185 |
| USP43 | Ubiquitin Specific Peptidase 43 | Protein Coding | 31 | GC17P010972 | 0.908335924 |
| MIR632 | MicroRNA 632 | RNA Gene | 14 | GC17P032350 | 0.908335924 |
| BTN2A1 | Butyrophilin Subfamily 2 Member A1 | Protein Coding | 35 | GC06P026457 | 0.908270299 |
| ZNF333 | Zinc Finger Protein 333 | Protein Coding | 30 | GC19P014689 | 0.907873929 |
| RGPD6 | RANBP2 Like And GRIP Domain Containing 6 | Protein Coding | 24 | GC02M110513 | 0.907777667 |
| ARV1 | ARV1 Homolog, Fatty Acid Homeostasis Modulator | Protein Coding | 32 | GC01P230978 | 0.907405734 |
| SLC7A13 | Solute Carrier Family 7 Member 13 | Protein Coding | 32 | GC08M086214 | 0.907405734 |
| CCDC172 | Coiled-Coil Domain Containing 172 | Protein Coding | 26 | GC10P116324 | 0.907405734 |
| GMNC | Geminin Coiled-Coil Domain Containing | Protein Coding | 26 | GC03M190852 | 0.907405734 |
| RETSAT | Retinol Saturase | Protein Coding | 34 | GC02M085344 | 0.907256126 |
| BFAR | Bifunctional Apoptosis Regulator | Protein Coding | 34 | GC16P014647 | 0.906963408 |
| PTGES3L-AARSD1 | PTGES3L-AARSD1 Readthrough | Protein Coding | 22 | GC17M042950 | 0.906843424 |
| MIR3178 | MicroRNA 3178 | RNA Gene | 12 | GC16M002531 | 0.906645715 |
| HMGN3 | High Mobility Group Nucleosomal Binding Domain 3 | Protein Coding | 31 | GC06M079201 | 0.906576037 |
| CDH26 | Cadherin 26 | Protein Coding | 32 | GC20P059958 | 0.906417489 |
| KLHL26 | Kelch Like Family Member 26 | Protein Coding | 31 | GC19P063128 | 0.906417489 |
| KIR2DS3 | Killer Cell Immunoglobulin Like Receptor, Two Ig Domains And Short Cytoplasmic Tail 3 | Protein Coding | 20 | GC19ME00037 | 0.905070782 |
| ILVBL | IlvB Acetolactate Synthase Like | Protein Coding | 34 | GC19M015116 | 0.904598713 |
| LRP11 | LDL Receptor Related Protein 11 | Protein Coding | 30 | GC06M149818 | 0.904120088 |
| MAMSTR | MEF2 Activating Motif And SAP Domain Containing Transcriptional Regulator | Protein Coding | 27 | GC19M048712 | 0.904120088 |
| ZNF578 | Zinc Finger Protein 578 | Protein Coding | 26 | GC19P064223 | 0.904120088 |
| TBC1D29P | TBC1 Domain Family Member 29, Pseudogene | Pseudogene | 18 | GC17P052564 | 0.904120088 |
| RAB9BP1 | RAB9B, Member RAS Oncogene Family Pseudogene 1 | Pseudogene | 10 | GC05P105099 | 0.904120088 |
| PLEK2 | Pleckstrin 2 | Protein Coding | 33 | GC14M067386 | 0.903833508 |
| PXMP4 | Peroxisomal Membrane Protein 4 | Protein Coding | 31 | GC20M033796 | 0.903646827 |
| ELOVL3 | ELOVL Fatty Acid Elongase 3 | Protein Coding | 32 | GC10P102226 | 0.903460205 |
| SEC22A | SEC22 Homolog A, Vesicle Trafficking Protein | Protein Coding | 32 | GC03P123201 | 0.903460205 |
| SIT1 | Signaling Threshold Regulating Transmembrane Adaptor 1 | Protein Coding | 32 | GC09M035640 | 0.903460205 |
| ARL14 | ADP Ribosylation Factor Like GTPase 14 | Protein Coding | 29 | GC03P160677 | 0.903460205 |
| SLC35G2 | Solute Carrier Family 35 Member G2 | Protein Coding | 29 | GC03P136818 | 0.903460205 |
| GARIN2 | Golgi Associated RAB2 Interactor Family Member 2 | Protein Coding | 23 | GC14P067192 | 0.903460205 |
| ARHGAP5-AS1 | ARHGAP5 Antisense RNA 1 (Head To Head) | RNA Gene | 15 | GC14M032072 | 0.903460205 |
| MIR1468 | MicroRNA 1468 | RNA Gene | 14 | GC0XM063786 | 0.903460205 |
| TIMM13 | Translocase Of Inner Mitochondrial Membrane 13 | Protein Coding | 32 | GC19M002425 | 0.903449774 |
| PTGFRN | Prostaglandin F2 Receptor Inhibitor | Protein Coding | 36 | GC01P116910 | 0.903205693 |
| DDX19B | DEAD-Box Helicase 19B | Protein Coding | 33 | GC16P070289 | 0.903151214 |
| MRPL32 | Mitochondrial Ribosomal Protein L32 | Protein Coding | 32 | GC07P042933 | 0.903151214 |
| KCTD9 | Potassium Channel Tetramerization Domain Containing 9 | Protein Coding | 31 | GC08M025427 | 0.903151214 |
| MTFR1L | Mitochondrial Fission Regulator 1 Like | Protein Coding | 27 | GC01P025822 | 0.903151214 |
| H2BC17 | H2B Clustered Histone 17 | Protein Coding | 24 | GC06P079955 | 0.903151214 |
| CHST2 | Carbohydrate Sulfotransferase 2 | Protein Coding | 34 | GC03P143119 | 0.902201176 |
| MAP4K5 | Mitogen-Activated Protein Kinase Kinase Kinase Kinase 5 | Protein Coding | 39 | GC14M050418 | 0.90196681 |
| ENPP6 | Ectonucleotide Pyrophosphatase/Phosphodiesterase 6 | Protein Coding | 34 | GC04M184088 | 0.901928782 |

| | | | | | |
|---|---|---|---|---|---|
| SH3BGRL | SH3 Domain Binding Glutamate Rich Protein Like | Protein Coding | 33 | GC0XP081201 | 0.901928782 |
| EEF1AKMT1 | EEF1A Lysine Methyltransferase 1 | Protein Coding | 27 | GC13M020729 | 0.901928782 |
| KLHDC7B | Kelch Domain Containing 7B | Protein Coding | 25 | GC22P050548 | 0.901928782 |
| FNDC10 | Fibronectin Type III Domain Containing 10 | Protein Coding | 19 | GC01M005554 | 0.901928782 |
| GVINP1 | GTPase, Very Large Interferon Inducible Pseudogene 1 | Pseudogene | 17 | GC11M006713 | 0.901928782 |
| ACAT2 | Acetyl-CoA Acetyltransferase 2 | Protein Coding | 40 | GC06P160430 | 0.901636302 |
| CWC15 | CWC15 Spliceosome Associated Protein Homolog | Protein Coding | 28 | GC11M094962 | 0.901502371 |
| CLDN20 | Claudin 20 | Protein Coding | 30 | GC06P155264 | 0.900916517 |
| MIR4723 | MicroRNA 4723 | RNA Gene | 10 | GC17P028360 | 0.900907755 |
| HTR3D | 5-Hydroxytryptamine Receptor 3D | Protein Coding | 31 | GC03P184031 | 0.900855482 |
| MYO19 | Myosin XIX | Protein Coding | 29 | GC17M036495 | 0.900671482 |
| MTSS2 | MTSS I-BAR Domain Containing 2 | Protein Coding | 26 | GC16M070661 | 0.900671482 |
| MIEF1 | Mitochondrial Elongation Factor 1 | Protein Coding | 29 | GC22P039510 | 0.900596857 |
| NPSA | Novel Prostate-Specific Antigen | Protein Coding | 4 | GC00U902522 | 0.900457859 |
| SLU7 | SLU7 Homolog, Splicing Factor | Protein Coding | 32 | GC05M160401 | 0.900240183 |
| COCH | Cochlin | Protein Coding | 39 | GC14P030874 | 0.899844646 |
| LRCH1 | Leucine Rich Repeats And Calponin Homology Domain Containing 1 | Protein Coding | 31 | GC13P046553 | 0.899278879 |
| H3C13 | H3 Clustered Histone 13 | Protein Coding | 24 | GC01M151577 | 0.898898542 |
| ARHGEF25 | Rho Guanine Nucleotide Exchange Factor 25 | Protein Coding | 31 | GC12P057611 | 0.898787618 |
| CCDC90B | Coiled-Coil Domain Containing 90B | Protein Coding | 34 | GC11M083259 | 0.898328781 |
| HERC6 | HECT And RLD Domain Containing E3 Ubiquitin Protein Ligase Family Member 6 | Protein Coding | 32 | GC04P088378 | 0.898328781 |
| TPRG1-AS1 | TPRG1 Antisense RNA 1 | RNA Gene | 13 | GC03M188941 | 0.897951722 |
| HS3ST4 | Heparan Sulfate-Glucosamine 3-Sulfotransferase 4 | Protein Coding | 31 | GC16P027805 | 0.897684455 |
| ALDH3B1 | Aldehyde Dehydrogenase 3 Family Member B1 | Protein Coding | 37 | GC11P069766 | 0.897416711 |
| MEF2C-AS2 | MEF2C Antisense RNA 2 | RNA Gene | 14 | GC05P088677 | 0.896989346 |
| CSMD2-AS1 | CSMD2 Antisense RNA 1 | RNA Gene | 13 | GC01P033868 | 0.896989346 |
| MME-AS1 | MME Antisense RNA 1 | RNA Gene | 13 | GC03M155158 | 0.896989346 |
| ANKRD40 | Ankyrin Repeat Domain 40 | Protein Coding | 30 | GC17M050693 | 0.896895647 |
| MON2 | MON2 Homolog, Regulator Of Endosome-To-Golgi Trafficking | Protein Coding | 29 | GC12P062466 | 0.896885097 |
| OSBPL9 | Oxysterol Binding Protein Like 9 | Protein Coding | 34 | GC01P051577 | 0.896882892 |
| MRPL14 | Mitochondrial Ribosomal Protein L14 | Protein Coding | 31 | GC06M044113 | 0.896878898 |
| ARL5B | ADP Ribosylation Factor Like GTPase 5B | Protein Coding | 34 | GC10P018659 | 0.896463692 |
| UPF2 | UPF2 Regulator Of Nonsense Mediated MRNA Decay | Protein Coding | 36 | GC10M011920 | 0.896054029 |
| MIR3679 | MicroRNA 3679 | RNA Gene | 14 | GC02P134127 | 0.895963192 |
| LRRN4 | Leucine Rich Repeat Neuronal 4 | Protein Coding | 29 | GC20M008045 | 0.895764351 |
| LINC00886 | Long Intergenic Non-Protein Coding RNA 886 | RNA Gene | 13 | GC03M156747 | 0.895721018 |
| SLC36A4 | Solute Carrier Family 36 Member 4 | Protein Coding | 31 | GC11M095899 | 0.894670904 |
| ZNF552 | Zinc Finger Protein 552 | Protein Coding | 27 | GC19M057803 | 0.894631624 |
| AGAP11 | ArfGAP With GTPase Domain, Ankyrin Repeat And PH Domain 11 | RNA Gene | 23 | GC10P091497 | 0.894631624 |
| AGAP9 | ArfGAP With GTPase Domain, Ankyrin Repeat And PH Domain 9 | Protein Coding | 23 | GC10M047501 | 0.894631624 |
| TYRL | Tyrosinase Like (Pseudogene) | Pseudogene | 10 | GC11M086916 | 0.894631624 |
| RASSF9 | Ras Association Domain Family Member 9 | Protein Coding | 30 | GC12M085800 | 0.894464374 |
| CKS1BP7 | CDC28 Protein Kinase Regulatory Subunit 1B Pseudogene 7 | Pseudogene | 6 | GC08M080644 | 0.894003451 |
| RBPMS-AS1 | RBPMS Antisense RNA 1 | RNA Gene | 12 | GC08M030455 | 0.893430293 |
| FLRT2 | Fibronectin Leucine Rich Transmembrane Protein 2 | Protein Coding | 34 | GC14P085527 | 0.893351793 |
| ZBTB8A | Zinc Finger And BTB Domain Containing 8A | Protein Coding | 31 | GC01P032539 | 0.893351793 |
| TTC13 | Tetratricopeptide Repeat Domain 13 | Protein Coding | 28 | GC01M230906 | 0.893182695 |
| GALM | Galactose Mutarotase | Protein Coding | 40 | GC02P038665 | 0.892862797 |
| IGKV3-20 | Immunoglobulin Kappa Variable 3-20 | Protein Coding | 13 | GC02M090823 | 0.89269042 |
| MEGF11 | Multiple EGF Like Domains 11 | Protein Coding | 34 | GC15M081207 | 0.892317891 |
| RAB28 | RAB28, Member RAS Oncogene Family | Protein Coding | 37 | GC04M013361 | 0.892113209 |
| SEC14L5 | SEC14 Like Lipid Binding 5 | Protein Coding | 29 | GC16P004958 | 0.891604424 |
| NRGN | Neurogranin | Protein Coding | 35 | GC11P124739 | 0.891016483 |
| PPP1R21 | Protein Phosphatase 1 Regulatory Subunit 21 | Protein Coding | 32 | GC02P048440 | 0.890579343 |
| ARHGAP12 | Rho GTPase Activating Protein 12 | Protein Coding | 33 | GC10M032757 | 0.8903144 |
| PDCD7 | Programmed Cell Death 7 | Protein Coding | 31 | GC15M065117 | 0.8903144 |
| LOC109113860 | FGF1 Promoter C | Biological Region | 2 | GC05P145988 | 0.890258908 |
| CNPY2 | Canopy FGF Signaling Regulator 2 | Protein Coding | 31 | GC12M056309 | 0.890176058 |
| KLRF1 | Killer Cell Lectin Like Receptor F1 | Protein Coding | 32 | GC12P019952 | 0.889993906 |
| MCOLN2 | Mucolipin TRP Cation Channel 2 | Protein Coding | 32 | GC01M084925 | 0.889993906 |
| TRIM69 | Tripartite Motif Containing 69 | Protein Coding | 32 | GC15P044747 | 0.889993906 |
| XKRX | XK Related X-Linked | Protein Coding | 25 | GC0XM100950 | 0.889993906 |
| CCDC138 | Coiled-Coil Domain Containing 138 | Protein Coding | 28 | GC02P108786 | 0.889275312 |
| CHSY3 | Chondroitin Sulfate Synthase 3 | Protein Coding | 32 | GC05P129904 | 0.889178872 |
| CSAD | Cysteine Sulfinic Acid Decarboxylase | Protein Coding | 35 | GC12M053160 | 0.889129281 |
| MIR8084 | MicroRNA 8084 | RNA Gene | 8 | GC08P093029 | 0.888653159 |
| CGB8 | Chorionic Gonadotropin Subunit Beta 8 | Protein Coding | 27 | GC19M049047 | 0.888554454 |
| SNX31 | Sorting Nexin 31 | Protein Coding | 29 | GC08M100572 | 0.888356626 |
| IGSF6 | Immunoglobulin Superfamily Member 6 | Protein Coding | 32 | GC16M021639 | 0.888288617 |
| SHF | Src Homology 2 Domain Containing F | Protein Coding | 31 | GC15M045167 | 0.888288617 |
| PKHD1L1 | PKHD1 Like 1 | Protein Coding | 30 | GC08P109362 | 0.888288617 |
| MAGIX | MAGI Family Member, X-Linked | Protein Coding | 28 | GC0XP049162 | 0.888288617 |
| TAFA2 | TAFA Chemokine Like Family Member 2 | Protein Coding | 28 | GC12M061709 | 0.888288617 |
| VWA3A | Von Willebrand Factor A Domain Containing 3A | Protein Coding | 27 | GC16P022174 | 0.888288617 |
| ZIM3 | Zinc Finger Imprinted 3 | Protein Coding | 27 | GC19M057134 | 0.888288617 |
| SYCE1L | Synaptonemal Complex Central Element Protein 1 Like | Protein Coding | 26 | GC16P077200 | 0.888288617 |
| USP49 | Ubiquitin Specific Peptidase 49 | Protein Coding | 33 | GC06M063825 | 0.887563109 |
| BBOX1 | Gamma-Butyrobetaine Hydroxylase 1 | Protein Coding | 36 | GC11P027019 | 0.887300313 |
| CSDC2 | Cold Shock Domain Containing C2 | Protein Coding | 32 | GC22P041560 | 0.887249112 |
| NUDT15 | Nudix Hydrolase 15 | Protein Coding | 35 | GC13P048037 | 0.887199223 |
| KANK3 | KN Motif And Ankyrin Repeat Domains 3 | Protein Coding | 28 | GC19M008322 | 0.887157917 |
| RC3H2 | Ring Finger And CCCH-Type Domains 2 | Protein Coding | 30 | GC09M122844 | 0.886862278 |
| THAP1 | THAP Domain Containing 1 | Protein Coding | 35 | GC08M042836 | 0.886580646 |
| AADACL2 | Arylacetamide Deacetylase Like 2 | Protein Coding | 30 | GC03P151733 | 0.88650322 |
| ZNF468 | Zinc Finger Protein 468 | Protein Coding | 28 | GC19M052838 | 0.88650322 |
| PATE2 | Prostate And Testis Expressed 2 | Protein Coding | 24 | GC11M125772 | 0.88650322 |
| PATE3 | Prostate And Testis Expressed 3 | Protein Coding | 24 | GC11P125788 | 0.88650322 |
| PATE4 | Prostate And Testis Expressed 4 | Protein Coding | 23 | GC11P125833 | 0.88650322 |
| SATB1-AS1 | SATB1 Antisense RNA 1 | RNA Gene | 13 | GC03P018445 | 0.88650322 |
| H2BC19P | H2B Clustered Histone 19, Pseudogene | Pseudogene | 12 | GC01P150133 | 0.88650322 |
| SPACA6-AS1 | SPACA6 Antisense RNA 1 | RNA Gene | 12 | GC19M051682 | 0.88650322 |
| KRT8P3 | Keratin 8 Pseudogene 3 | Pseudogene | 9 | GC08P061578 | 0.88650322 |
| ENSG00000287725 | Novel Protein, TPCN2 - SMIM38 Readthrough | Protein Coding | 7 | GC11P069072 | 0.88650322 |
| KCNN1 | Potassium Calcium-Activated Channel Subfamily N Member 1 | Protein Coding | 35 | GC19P063116 | 0.886061728 |
| TMEM242 | Transmembrane Protein 242 | Protein Coding | 28 | GC06M157289 | 0.886061728 |

| | | | | | |
|---|---|---|---|---|---|
| MIR1825 | MicroRNA 1825 | RNA Gene | 14 | GC20P032237 | 0.886037886 |
| MIR509-2 | MicroRNA 509-2 | RNA Gene | 11 | GC0XM147258 | 0.886037886 |
| CHERP | Calcium Homeostasis Endoplasmic Reticulum Protein | Protein Coding | 31 | GC19M016517 | 0.885563493 |
| CYTH4 | Cytohesin 4 | Protein Coding | 34 | GC22P037282 | 0.885127425 |
| TMEM238 | Transmembrane Protein 238 | Protein Coding | 23 | GC19M055379 | 0.884681702 |
| RUNDC1 | RUN Domain Containing 1 | Protein Coding | 28 | GC17P053179 | 0.884563148 |
| GAS2L3 | Growth Arrest Specific 2 Like 3 | Protein Coding | 29 | GC12P100573 | 0.8834126 |
| MIR933 | MicroRNA 933 | RNA Gene | 13 | GC02M175167 | 0.8834126 |
| TK2 | Thymidine Kinase 2 | Protein Coding | 36 | GC16M066508 | 0.882909417 |
| KIAA1143 | KIAA1143 | Protein Coding | 27 | GC03M044737 | 0.882696748 |
| LCN1P1 | Lipocalin 1 Pseudogene 1 | Pseudogene | 11 | GC09M133224 | 0.882696748 |
| LIN54 | Lin-54 DREAM MuvB Core Complex Component | Protein Coding | 32 | GC04M082909 | 0.881846726 |
| DOCK3 | Dedicator Of Cytokinesis 3 | Protein Coding | 37 | GC03P050675 | 0.881635129 |
| LSS | Lanosterol Synthase | Protein Coding | 40 | GC21M050459 | 0.881269574 |
| INO80E | INO80 Complex Subunit E | Protein Coding | 28 | GC16P040200 | 0.881163239 |
| RARS2 | Arginyl-TRNA Synthetase 2, Mitochondrial | Protein Coding | 38 | GC06M087514 | 0.880973756 |
| MIR4656 | MicroRNA 4656 | RNA Gene | 10 | GC07M004788 | 0.880241394 |
| MIR4730 | MicroRNA 4730 | RNA Gene | 9 | GC17P080419 | 0.880241394 |
| GABRA5 | Gamma-Aminobutyric Acid Type A Receptor Subunit Alpha5 | Protein Coding | 43 | GC15P026866 | 0.88001132 |
| MIR1193 | MicroRNA 1193 | RNA Gene | 11 | GC14P109511 | 0.879983664 |
| TENM4 | Teneurin Transmembrane Protein 4 | Protein Coding | 31 | GC11M078652 | 0.879837036 |
| RAB9B | RAB9B, Member RAS Oncogene Family | Protein Coding | 31 | GC0XM103780 | 0.879129171 |
| GLULP6 | Glutamate-Ammonia Ligase Pseudogene 6 | Pseudogene | 6 | GC02M194129 | 0.878218174 |
| KRT18P27 | Keratin 18 Pseudogene 27 | Pseudogene | 6 | GC13P090230 | 0.878218174 |
| RN7SL5P | RNA, 7SL, Cytoplasmic 5, Pseudogene | Pseudogene | 6 | GC09P009441 | 0.878218174 |
| RPL9P19 | Ribosomal Protein L9 Pseudogene 19 | Pseudogene | 6 | GC07P008994 | 0.878218174 |
| RPS12P24 | Ribosomal Protein S12 Pseudogene 24 | Pseudogene | 6 | GC13M037220 | 0.878218174 |
| RPS18P6 | Ribosomal Protein S18 Pseudogene 6 | Pseudogene | 6 | GC03P095171 | 0.878218174 |
| RPS8P6 | Ribosomal Protein S8 Pseudogene 6 | Pseudogene | 5 | GC03M000309 | 0.878218174 |
| NDUFS6 | NADH:Ubiquinone Oxidoreductase Subunit S6 | Protein Coding | 39 | GC05P001801 | 0.878215253 |
| CIDEC | Cell Death Inducing DFFA Like Effector C | Protein Coding | 36 | GC03M009866 | 0.878144503 |
| LIN7A | Lin-7 Homolog A, Crumbs Cell Polarity Complex Component | Protein Coding | 35 | GC12M080792 | 0.878107667 |
| NACAD | NAC Alpha Domain Containing | Protein Coding | 27 | GC07M045080 | 0.877274513 |
| LNP1 | Leukemia NUP98 Fusion Partner 1 | Protein Coding | 26 | GC03P100401 | 0.877092481 |
| L3MBTL4 | L3MBTL Histone Methyl-Lysine Binding Protein 4 | Protein Coding | 31 | GC18M005954 | 0.876892447 |
| OTUB2 | OTU Deubiquitinase, Ubiquitin Aldehyde Binding 2 | Protein Coding | 31 | GC14P094023 | 0.876507282 |
| ZNF691 | Zinc Finger Protein 691 | Protein Coding | 28 | GC01P042847 | 0.876115441 |
| PBDC1 | Polysaccharide Biosynthesis Domain Containing 1 | Protein Coding | 26 | GC0XP076172 | 0.876115441 |
| UBE2G2 | Ubiquitin Conjugating Enzyme E2 G2 | Protein Coding | 39 | GC21M044768 | 0.876024723 |
| UNK | Unk Zinc Finger | Protein Coding | 31 | GC17P075784 | 0.875757456 |
| N4BP3 | NEDD4 Binding Protein 3 | Protein Coding | 29 | GC05P178113 | 0.875757456 |
| FAM3A | FAM3 Metabolism Regulating Signaling Molecule A | Protein Coding | 31 | GC0XM154506 | 0.875598669 |
| MORN1 | MORN Repeat Containing 1 | Protein Coding | 30 | GC01M005582 | 0.875579953 |
| MTCL1 | Microtubule Crosslinking Factor 1 | Protein Coding | 30 | GC18P008705 | 0.875463068 |
| AKR7A3 | Aldo-Keto Reductase Family 7 Member A3 | Protein Coding | 34 | GC01M019282 | 0.875167012 |
| SLC35F6 | Solute Carrier Family 35 Member F6 | Protein Coding | 31 | GC02P026764 | 0.875162959 |
| KRT222 | Keratin 222 | Protein Coding | 28 | GC17M040654 | 0.875131965 |
| MIR5582 | MicroRNA 5582 | RNA Gene | 10 | GC11M088261 | 0.874927402 |
| PLGLB2 | Plasminogen Like B2 | Protein Coding | 28 | GC02P087748 | 0.874838352 |
| R3HDM2 | R3H Domain Containing 2 | Protein Coding | 30 | GC12M057253 | 0.874699831 |
| MIR3192 | MicroRNA 3192 | RNA Gene | 11 | GC20P018494 | 0.874655962 |
| DUSP12 | Dual Specificity Phosphatase 12 | Protein Coding | 33 | GC01P161749 | 0.87403959 |
| ADAM30 | ADAM Metallopeptidase Domain 30 | Protein Coding | 32 | GC01M119893 | 0.873316109 |
| GHDC | GH3 Domain Containing | Protein Coding | 28 | GC17M042188 | 0.873316109 |
| WDR83OS | WD Repeat Domain 83 Opposite Strand | Protein Coding | 28 | GC19M012668 | 0.873316109 |
| DLGAP1 | DLG Associated Protein 1 | Protein Coding | 38 | GC18M003488 | 0.872937679 |
| TMEM168 | Transmembrane Protein 168 | Protein Coding | 30 | GC07M112762 | 0.871800065 |
| CARNS1 | Carnosine Synthase 1 | Protein Coding | 27 | GC11P067414 | 0.870947123 |
| SNORD3A | Small Nucleolar RNA, C/D Box 3A | RNA Gene | 17 | GC17P052275 | 0.870947123 |
| TSIX | TSIX Transcript, XIST Antisense RNA | RNA Gene | 16 | GC0XP073792 | 0.870375633 |
| PTDSS2 | Phosphatidylserine Synthase 2 | Protein Coding | 32 | GC11P000448 | 0.869877696 |
| RGP1 | RGP1 Homolog, RAB6A GEF Complex Partner 1 | Protein Coding | 30 | GC09P035749 | 0.869780302 |
| USP17L18 | Ubiquitin Specific Peptidase 17 Like Family Member 18 | Protein Coding | 16 | GC04P009254 | 0.869780302 |
| MIR4636 | MicroRNA 4636 | RNA Gene | 11 | GC05M009055 | 0.868867278 |
| NUP210L | Nucleoporin 210 Like | Protein Coding | 28 | GC01M153993 | 0.868726671 |
| UBE2W | Ubiquitin Conjugating Enzyme E2 W | Protein Coding | 31 | GC08M073780 | 0.868710279 |
| MMP23A | Matrix Metallopeptidase 23A (Pseudogene) | Pseudogene | 16 | GC01P001699 | 0.868710279 |
| NT5C1A | 5'-Nucleotidase, Cytosolic IA | Protein Coding | 35 | GC01M039659 | 0.868686557 |
| TMEM38B | Transmembrane Protein 38B | Protein Coding | 38 | GC09P105694 | 0.868475378 |
| UQCRFS1P1 | Ubiquinol-Cytochrome C Reductase, Rieske Iron-Sulfur Polypeptide 1 Pseudogene 1 | Pseudogene | 12 | GC22P039875 | 0.868346095 |
| MIR4316 | MicroRNA 4316 | RNA Gene | 11 | GC17M077396 | 0.868346095 |
| EIF2D | Eukaryotic Translation Initiation Factor 2D | Protein Coding | 29 | GC01M206571 | 0.867339969 |
| MIR3609 | MicroRNA 3609 | RNA Gene | 13 | GC07P098881 | 0.867185056 |
| PDE8A | Phosphodiesterase 8A | Protein Coding | 36 | GC15P115143 | 0.866580188 |
| GLT8D2 | Glycosyltransferase 8 Domain Containing 2 | Protein Coding | 32 | GC12M103988 | 0.866580188 |
| MESD | Mesoderm Development LRP Chaperone | Protein Coding | 34 | GC15M081672 | 0.866409302 |
| SLC43A3 | Solute Carrier Family 43 Member 3 | Protein Coding | 33 | GC11M086955 | 0.866375148 |
| EIF2AK1 | Eukaryotic Translation Initiation Factor 2 Alpha Kinase 1 | Protein Coding | 39 | GC07M006022 | 0.866243899 |
| TTC9B | Tetratricopeptide Repeat Domain 9B | Protein Coding | 28 | GC19M040216 | 0.866187572 |
| TTC6 | Tetratricopeptide Repeat Domain 6 | Protein Coding | 24 | GC14P037595 | 0.866075993 |
| AKR1D1 | Aldo-Keto Reductase Family 1 Member D1 | Protein Coding | 41 | GC07P138003 | 0.865945935 |
| RHOQ | Ras Homolog Family Member Q | Protein Coding | 37 | GC02P046543 | 0.865814328 |
| NPHP3-ACAD11 | NPHP3-ACAD11 Readthrough (NMD Candidate) | RNA Gene | 13 | GC03M132558 | 0.865611255 |
| RANBP6 | RAN Binding Protein 6 | Protein Coding | 32 | GC09M006002 | 0.86480087 |
| LMBR1 | Limb Development Membrane Protein 1 | Protein Coding | 35 | GC07M156668 | 0.86472851 |
| MIR1910 | MicroRNA 1910 | RNA Gene | 13 | GC16M085742 | 0.864674807 |
| MIR4472-2 | MicroRNA 4472-2 | RNA Gene | 10 | GC12M116428 | 0.864674807 |
| MIR4472-1 | MicroRNA 4472-1 | RNA Gene | 9 | GC08P142176 | 0.864674807 |
| RAB13 | RAB13, Member RAS Oncogene Family | Protein Coding | 34 | GC01M153981 | 0.864288032 |
| SRSF11 | Serine And Arginine Rich Splicing Factor 11 | Protein Coding | 33 | GC01P070206 | 0.864093006 |
| SERP1 | Stress Associated Endoplasmic Reticulum Protein 1 | Protein Coding | 30 | GC03M150541 | 0.864088953 |
| C1orf115 | Chromosome 1 Open Reading Frame 115 | Protein Coding | 27 | GC01P220689 | 0.864088953 |
| DINOL | Damage Induced Long Noncoding RNA | RNA Gene | 10 | GC06M064121 | 0.864088953 |
| AIFM3 | Apoptosis Inducing Factor Mitochondria Associated 3 | Protein Coding | 34 | GC22P020965 | 0.863773167 |
| SHFL | Shiftless Antiviral Inhibitor Of Ribosomal Frameshifting | Protein Coding | 25 | GC19P010086 | 0.863215744 |

| | | | | | |
|---|---|---|---|---|---|
| KRT38 | Keratin 38 | Protein Coding | 31 | GC17M041436 | 0.863005042 |
| STYXL1 | Serine/Threonine/Tyrosine Interacting Like 1 | Protein Coding | 31 | GC07M075995 | 0.862802446 |
| PLEKHM2 | Pleckstrin Homology And RUN Domain Containing M2 | Protein Coding | 31 | GC01P015796 | 0.862386346 |
| TMPRSS7 | Transmembrane Serine Protease 7 | Protein Coding | 28 | GC03P112034 | 0.861352444 |
| UBA6-DT | UBA6 Divergent Transcript | RNA Gene | 13 | GC04P067702 | 0.859998941 |
| NECAP1 | NECAP Endocytosis Associated 1 | Protein Coding | 35 | GC12P019883 | 0.859908164 |
| VPS29 | VPS29 Retromer Complex Component | Protein Coding | 35 | GC12M110491 | 0.859761834 |
| SLC25A17 | Solute Carrier Family 25 Member 17 | Protein Coding | 35 | GC22M056113 | 0.859605432 |
| MTERF1 | Mitochondrial Transcription Termination Factor 1 | Protein Coding | 30 | GC07M091692 | 0.859218597 |
| RNF183 | Ring Finger Protein 183 | Protein Coding | 27 | GC09M113297 | 0.85885787 |
| SARS2 | Seryl-TRNA Synthetase 2, Mitochondrial | Protein Coding | 38 | GC19M063771 | 0.858368456 |
| DLEU1-AS1 | DLEU1 Antisense RNA 1 | RNA Gene | 14 | GC13M050520 | 0.858345032 |
| LOC105377871 | Uncharacterized LOC105377871 | RNA Gene | 3 | GC06M081444 | 0.858110249 |
| LIF-AS1 | LIF Antisense RNA 1 | RNA Gene | 11 | GC22P034971 | 0.858072996 |
| EMC8 | ER Membrane Protein Complex Subunit 8 | Protein Coding | 29 | GC16M085771 | 0.857999802 |
| GAS6-DT | GAS6 Divergent Transcript | RNA Gene | 12 | GC13P113865 | 0.857999802 |
| NIPAL1 | NIPA Like Domain Containing 1 | Protein Coding | 31 | GC04P047917 | 0.857696652 |
| KREMEN2 | Kringle Containing Transmembrane Protein 2 | Protein Coding | 32 | GC16P010752 | 0.85765022 |
| TCHHL1 | Trichohyalin Like 1 | Protein Coding | 31 | GC01M152085 | 0.85758096 |
| SH3BGRL2 | SH3 Domain Binding Glutamate Rich Protein Like 2 | Protein Coding | 30 | GC06P080970 | 0.857500851 |
| HMGN4 | High Mobility Group Nucleosomal Binding Domain 4 | Protein Coding | 27 | GC06P026538 | 0.857500851 |
| HSP90AB2P | Heat Shock Protein 90 Alpha Family Class B Member 2, Pseudogene | Pseudogene | 15 | GC04P017702 | 0.857500851 |
| IRAG2 | Inositol 1,4,5-Triphosphate Receptor Associated 2 | Protein Coding | 26 | GC12P025031 | 0.85738194 |
| LOC109029529 | HSD3B1 5' Regulatory Region | Biological Region | 2 | GC01P119504 | 0.856948853 |
| MIR3129 | MicroRNA 3129 | RNA Gene | 14 | GC02M189133 | 0.856578946 |
| MIR499B | MicroRNA 499b | RNA Gene | 10 | GC20M034990 | 0.856578946 |
| LRRC20 | Leucine Rich Repeat Containing 20 | Protein Coding | 30 | GC10M070298 | 0.856491566 |
| PTPRCAP | Protein Tyrosine Phosphatase Receptor Type C Associated Protein | Protein Coding | 30 | GC11M087384 | 0.856491566 |
| GPR132 | G Protein-Coupled Receptor 132 | Protein Coding | 35 | GC14M105049 | 0.856254816 |
| GPR107 | G Protein-Coupled Receptor 107 | Protein Coding | 33 | GC09P130053 | 0.856254816 |
| XXYLT1 | Xyloside Xylosyltransferase 1 | Protein Coding | 28 | GC03M195068 | 0.856185794 |
| NLN | Neurolysin | Protein Coding | 36 | GC05P065722 | 0.856024861 |
| MRPS24 | Mitochondrial Ribosomal Protein S24 | Protein Coding | 32 | GC07M043868 | 0.856018066 |
| TRIL | TLR4 Interactor With Leucine Rich Repeats | Protein Coding | 29 | GC07M028953 | 0.855699241 |
| NOMO2 | NODAL Modulator 2 | Protein Coding | 30 | GC16M019186 | 0.855595469 |
| RPL17P39 | Ribosomal Protein L17 Pseudogene 39 | Pseudogene | 10 | GC15M071341 | 0.855227768 |
| LINC02953 | Long Intergenic Non-Protein Coding RNA 2953 | RNA Gene | 9 | GC11P069838 | 0.855227768 |
| ENSG00000272501 | Novel Transcript, Antisense To HCG27 | RNA Gene | 8 | GC06M063558 | 0.855227768 |
| ENSG00000262133 | Novel Transcript, Antisense To NXN | RNA Gene | 7 | GC17P000877 | 0.855227768 |
| RPL7P18 | Ribosomal Protein L7 Pseudogene 18 | Pseudogene | 7 | GC05M094827 | 0.855227768 |
| ENSG00000224272 | Novel Transcript | RNA Gene | 6 | GC02M241808 | 0.855227768 |
| TRANK1 | Tetratricopeptide Repeat And Ankyrin Repeat Containing 1 | Protein Coding | 29 | GC03M036845 | 0.85492301 |
| DEUP1 | Deuterosome Assembly Protein 1 | Protein Coding | 25 | GC11P093330 | 0.854453087 |
| NPEPL1 | Aminopeptidase Like 1 | Protein Coding | 30 | GC20P058689 | 0.853630304 |
| PGGHG | Protein-Glucosylgalactosylhydroxylysine Glucosidase | Protein Coding | 28 | GC11P000288 | 0.853630304 |
| LINC00174 | Long Intergenic Non-Protein Coding RNA 174 | RNA Gene | 18 | GC07M066376 | 0.853630304 |
| IGHV1-45 | Immunoglobulin Heavy Variable 1-45 | Protein Coding | 10 | GC14M112475 | 0.852986097 |
| VMA21 | Vacuolar ATPase Assembly Factor VMA21 | Protein Coding | 32 | GC0XP151396 | 0.852682531 |
| MIR1323 | MicroRNA 1323 | RNA Gene | 11 | GC19P064293 | 0.85241437 |
| LINC01013 | Long Intergenic Non-Protein Coding RNA 1013 | RNA Gene | 15 | GC06P132131 | 0.852399707 |
| KLHL25 | Kelch Like Family Member 25 | Protein Coding | 31 | GC15M089539 | 0.852197647 |
| RFX8 | Regulatory Factor X8 | Protein Coding | 27 | GC02M101394 | 0.852036655 |
| NXF5 | Nuclear RNA Export Factor 5 | Pseudogene | 27 | GC0XM101832 | 0.852007568 |
| RAB11FIP5 | RAB11 Family Interacting Protein 5 | Protein Coding | 36 | GC02M073128 | 0.85180068 |
| USP26 | Ubiquitin Specific Peptidase 26 | Protein Coding | 33 | GC0XM133024 | 0.85172832 |
| RAB11B-AS1 | RAB11B Antisense RNA 1 | RNA Gene | 13 | GC19M008374 | 0.851208448 |
| SPACDR | Sperm Acrosome Developmental Regulator | Protein Coding | 23 | GC07M101888 | 0.851019025 |
| QPRT | Quinolinate Phosphoribosyltransferase | Protein Coding | 36 | GC16P040183 | 0.850936294 |
| AFTPH | Aftiphilin | Protein Coding | 30 | GC02P064524 | 0.850936294 |
| SCML2 | Scm Polycomb Group Protein Like 2 | Protein Coding | 30 | GC0XM018167 | 0.850936294 |
| HS1BP3 | HCLS1 Binding Protein 3 | Protein Coding | 32 | GC02M020560 | 0.850831151 |
| PCDHGB3 | Protocadherin Gamma Subfamily B, 3 | Protein Coding | 28 | GC05P145832 | 0.850381851 |
| MIR3175 | MicroRNA 3175 | RNA Gene | 14 | GC15P092904 | 0.850014448 |
| MIR451B | MicroRNA 451b | RNA Gene | 13 | GC17P028861 | 0.850014448 |
| PPM1M | Protein Phosphatase, Mg2+/Mn2+ Dependent 1M | Protein Coding | 33 | GC03P052245 | 0.849291623 |
| FMO5 | Flavin Containing Dimethylaniline Monoxygenase 5 | Protein Coding | 37 | GC01M147175 | 0.849211574 |
| TM6SF1 | Transmembrane 6 Superfamily Member 1 | Protein Coding | 28 | GC15P115042 | 0.84851557 |
| A1BG-AS1 | A1BG Antisense RNA 1 | RNA Gene | 13 | GC19P058347 | 0.84851557 |
| MIR3144 | MicroRNA 3144 | RNA Gene | 11 | GC06P120015 | 0.84851557 |
| LOC109117330 | FGF4 Promoter Region | Biological Region | 2 | GC11P069788 | 0.84851557 |
| KIF1C | Kinesin Family Member 1C | Protein Coding | 38 | GC17P005009 | 0.847670436 |
| ROCR | Regulator Of Chondrogenesis RNA | RNA Gene | 11 | GC17M072023 | 0.847319961 |
| MIEF2 | Mitochondrial Elongation Factor 2 | Protein Coding | 30 | GC17P018262 | 0.84679234 |
| PLPP6 | Phospholipid Phosphatase 6 | Protein Coding | 28 | GC09P004663 | 0.846625686 |
| DCTN6 | Dynactin Subunit 6 | Protein Coding | 32 | GC08P030133 | 0.846473694 |
| TSPOAP1-AS1 | TSPOAP1, SUPT4H1 And RNF43 Antisense RNA 1 | RNA Gene | 13 | GC17P058326 | 0.846240282 |
| FAM193B | Family With Sequence Similarity 193 Member B | Protein Coding | 30 | GC05M177519 | 0.845958829 |
| PRTG | Protogenin | Protein Coding | 34 | GC15M055611 | 0.845616937 |
| C5orf38 | Chromosome 5 Open Reading Frame 38 | RNA Gene | 27 | GC05P002752 | 0.845236182 |
| ADAP2 | ArfGAP With Dual PH Domains 2 | Protein Coding | 32 | GC17P030906 | 0.845067859 |
| SAMD4A | Sterile Alpha Motif Domain Containing 4A | Protein Coding | 32 | GC14P054567 | 0.844864607 |
| LINC02273 | Long Intergenic Non-Protein Coding RNA 2273 | RNA Gene | 11 | GC04P152101 | 0.844864607 |
| MKI67P1 | Marker Of Proliferation Ki-67 Pseudogene 1 | Pseudogene | 5 | GC0XM046560 | 0.844835162 |
| KCNJ4 | Potassium Inwardly Rectifying Channel Subfamily J Member 4 | Protein Coding | 40 | GC22M038426 | 0.844769478 |
| TRIM7 | Tripartite Motif Containing 7 | Protein Coding | 30 | GC05M181193 | 0.844676852 |
| PLEKHG5 | Pleckstrin Homology And RhoGEF Domain Containing G5 | Protein Coding | 36 | GC01M006466 | 0.84441185 |
| WDFY3-AS2 | WDFY3 Antisense RNA 2 | RNA Gene | 15 | GC04P084965 | 0.844034553 |
| MIR644A | MicroRNA 644a | RNA Gene | 11 | GC20P034468 | 0.844034553 |
| ZNF347 | Zinc Finger Protein 347 | Protein Coding | 30 | GC19M053124 | 0.843972385 |
| ZNF816 | Zinc Finger Protein 816 | Protein Coding | 29 | GC19M052949 | 0.843972385 |
| IQCH | IQ Motif Containing H | Protein Coding | 28 | GC15P067254 | 0.843972385 |
| ZNF600 | Zinc Finger Protein 600 | Protein Coding | 28 | GC19M052749 | 0.843972385 |
| ZNF611 | Zinc Finger Protein 611 | Protein Coding | 28 | GC19M052702 | 0.843972385 |
| ZNF766 | Zinc Finger Protein 766 | Protein Coding | 28 | GC19P052269 | 0.843972385 |
| TMEM229B | Transmembrane Protein 229B | Protein Coding | 27 | GC14M067447 | 0.843972385 |

| | | | | | |
|---|---|---|---|---|---|
| ZNF836 | Zinc Finger Protein 836 | Protein Coding | 27 | GC19M052153 | 0.843972385 |
| ZNF808 | Zinc Finger Protein 808 | Protein Coding | 25 | GC19P064226 | 0.843972385 |
| ZNF761 | Zinc Finger Protein 761 | Protein Coding | 24 | GC19P053431 | 0.843972385 |
| ZNF525 | Zinc Finger Protein 525 | Protein Coding | 23 | GC19P064271 | 0.843972385 |
| ZNF880 | Zinc Finger Protein 880 | Protein Coding | 23 | GC19P052369 | 0.843972385 |
| HCG20 | HLA Complex Group 20 | RNA Gene | 15 | GC06P080333 | 0.843972385 |
| LINC02210-CRHR1 | LINC02210-CRHR1 Readthrough | Protein Coding | 14 | GC17P053284 | 0.843972385 |
| LINC01948 | Long Intergenic Non-Protein Coding RNA 1948 | RNA Gene | 13 | GC05M056459 | 0.843972385 |
| THRB-AS1 | THRB Antisense RNA 1 | RNA Gene | 13 | GC03P024494 | 0.843972385 |
| HCG25 | HLA Complex Group 25 | RNA Gene | 12 | GC06P080421 | 0.843972385 |
| KIF9-AS1 | KIF9 Antisense RNA 1 | RNA Gene | 12 | GC03P047164 | 0.843972385 |
| LINC01271 | Long Intergenic Non-Protein Coding RNA 1271 | RNA Gene | 12 | GC20M050310 | 0.843972385 |
| LINC00489 | Long Intergenic Non-Protein Coding RNA 489 | RNA Gene | 11 | GC20M037619 | 0.843972385 |
| LINC01416 | Long Intergenic Non-Protein Coding RNA 1416 | RNA Gene | 11 | GC18M055881 | 0.843972385 |
| LINC02188 | Long Intergenic Non-Protein Coding RNA 2188 | RNA Gene | 11 | GC16P086716 | 0.843972385 |
| LOC338694 | Uncharacterized LOC338694 | RNA Gene | 11 | GC11M069138 | 0.843972385 |
| TPM3P9 | Tropomyosin 3 Pseudogene 9 | Pseudogene | 11 | GC19P064274 | 0.843972385 |
| TYRO3P | TYRO3P Protein Tyrosine Kinase Pseudogene | Pseudogene | 11 | GC15M076258 | 0.843972385 |
| SMC2-DT | SMC2 Divergent Transcript | RNA Gene | 10 | GC09M104077 | 0.843972385 |
| TAB2-AS1 | TAB2 Antisense RNA 1 | RNA Gene | 10 | GC06M149245 | 0.843972385 |
| TFPI2-DT | TFPI2 Divergent Transcript | RNA Gene | 10 | GC07P093892 | 0.843972385 |
| BET1-AS1 | BET1 Antisense RNA 1 | RNA Gene | 9 | GC07P093967 | 0.843972385 |
| GP6-AS1 | GP6 Antisense RNA 1 | RNA Gene | 9 | GC19P064603 | 0.843972385 |
| LINC01519 | Long Intergenic Non-Protein Coding RNA 1519 | RNA Gene | 9 | GC10M085193 | 0.843972385 |
| ENSG00000224984 | Novel Transcript | RNA Gene | 8 | GC06M064034 | 0.843972385 |
| ENSG00000233340 | Novel Transcript | RNA Gene | 8 | GC10M112671 | 0.843972385 |
| IGFBP-AS1 | IGFBP5 Antisense RNA 1 | RNA Gene | 8 | GC02P216712 | 0.843972385 |
| LINC01396 | Long Intergenic Non-Protein Coding RNA 1396 | RNA Gene | 8 | GC04P004844 | 0.843972385 |
| MAPK8IP1P1 | Mitogen-Activated Protein Kinase 8 Interacting Protein 1 Pseudogene 1 | Pseudogene | 8 | GC17P053293 | 0.843972385 |
| RNY4P10 | RNY4 Pseudogene 10 | Pseudogene | 8 | GC06P033199 | 0.843972385 |
| RPL35P2 | Ribosomal Protein L35 Pseudogene 2 | Pseudogene | 8 | GC06M063741 | 0.843972385 |
| ENSG00000226647 | Novel Transcript | RNA Gene | 7 | GC10M005712 | 0.843972385 |
| ENSG00000232935 | Novel Transcript, Antisense To DOCK1 | RNA Gene | 7 | GC10M127013 | 0.843972385 |
| ENSG00000234393 | Novel Transcript | RNA Gene | 7 | GC10M113710 | 0.843972385 |
| ENSG00000236352 | Novel Transcript | RNA Gene | 7 | GC20P053940 | 0.843972385 |
| ENSG00000236471 | Novel Transcript | RNA Gene | 7 | GC21P015067 | 0.843972385 |
| ENSG00000250751 | Novel Transcript | RNA Gene | 7 | GC17P049708 | 0.843972385 |
| ENSG00000253217 | Novel Transcript | RNA Gene | 7 | GC08P100337 | 0.843972385 |
| ENSG00000253666 | Novel Transcript | RNA Gene | 7 | GC08P100475 | 0.843972385 |
| ENSG00000260160 | Novel Transcript | RNA Gene | 7 | GC19M064278 | 0.843972385 |
| ENSG00000262837 | Novel Transcript | RNA Gene | 7 | GC17P049834 | 0.843972385 |
| ENSG00000268030 | Novel Transcript, Antisense To KXD1 | RNA Gene | 7 | GC19M018557 | 0.843972385 |
| ENSG00000268938 | Novel Transcript, Antisense To FKBP8 | RNA Gene | 7 | GC19P063125 | 0.843972385 |
| ENSG00000268970 | Novel Transcript, Sense Intronic To ZNF83 | RNA Gene | 7 | GC19M052555 | 0.843972385 |
| ENSG00000269191 | Novel Transcript, Antisense To FKBP8 | RNA Gene | 7 | GC19P063126 | 0.843972385 |
| LINC02066 | Long Intergenic Non-Protein Coding RNA 2066 | RNA Gene | 7 | GC03P151629 | 0.843972385 |
| LINC02646 | Long Intergenic Non-Protein Coding RNA 2646 | RNA Gene | 7 | GC10P130215 | 0.843972385 |
| LOC107985164 | Uncharacterized LOC107985164 | RNA Gene | 7 | GC18P055894 | 0.843972385 |
| RNU1-88P | RNA, U1 Small Nuclear 88, Pseudogene | Pseudogene | 7 | GC06M063761 | 0.843972385 |
| RNU6-1213P | RNA, U6 Small Nuclear 1213, Pseudogene | Pseudogene | 7 | GC08P080405 | 0.843972385 |
| RPS15AP27 | Ribosomal Protein S15a Pseudogene 27 | Pseudogene | 7 | GC09M107963 | 0.843972385 |
| RPS15AP5 | Ribosomal Protein S15a Pseudogene 5 | Pseudogene | 7 | GC10P121713 | 0.843972385 |
| TPM3P6 | Tropomyosin 3 Pseudogene 6 | Pseudogene | 7 | GC19P053479 | 0.843972385 |
| UBE2D3P3 | Ubiquitin Conjugating Enzyme E2 D3 Pseudogene 3 | Pseudogene | 7 | GC01P150800 | 0.843972385 |
| YWHAQP6 | YWHAQ Pseudogene 6 | Pseudogene | 7 | GC03M141599 | 0.843972385 |
| CHCHD3P1 | Coiled-Coil-Helix-Coiled-Coil-Helix Domain Containing 3 Pseudogene 1 | Pseudogene | 6 | GC10P008662 | 0.843972385 |
| CHCHD4P2 | Coiled-Coil-Helix-Coiled-Coil-Helix Domain Containing 4 Pseudogene 2 | Pseudogene | 6 | GC09M108097 | 0.843972385 |
| ENSG00000202310 | Y RNA | RNA Gene | 6 | GC08P100158 | 0.843972385 |
| ENSG00000223528 | Novel Transcript | RNA Gene | 6 | GC10M126988 | 0.843972385 |
| ENSG00000260064 | Novel Transcript, Sense Intronic To CDYL2 | RNA Gene | 6 | GC16M080627 | 0.843972385 |
| ENSG00000263680 | Novel Transcript | RNA Gene | 6 | GC17P072425 | 0.843972385 |
| ENSG00000269349 | Novel Transcript, Antisense To ZNF578 | RNA Gene | 6 | GC19M052511 | 0.843972385 |
| ENSG00000271828 | Novel Transcript, Antisense To MIER3 | RNA Gene | 6 | GC05P056927 | 0.843972385 |
| ENSG00000272489 | Novel Transcript | RNA Gene | 6 | GC10P091439 | 0.843972385 |
| ENSG00000272540 | Novel Transcript, Antisense To TUBB | RNA Gene | 6 | GC06M063531 | 0.843972385 |
| ENSG00000288555 | Novel Transcript | RNA Gene | 6 | GC21M015191 | 0.843972385 |
| RF00017-4593 | | RNA Gene | 6 | GC05P146083 | 0.843972385 |
| RN7SL636P | RNA, 7SL, Cytoplasmic 636, Pseudogene | Pseudogene | 6 | GC20M050485 | 0.843972385 |
| RNA5SP299 | RNA, 5S Ribosomal Pseudogene 299 | Pseudogene | 6 | GC10M008699 | 0.843972385 |
| RNU6-342P | RNA, U6 Small Nuclear 342, Pseudogene | Pseudogene | 6 | GC03M027266 | 0.843972385 |
| RPL21P38 | Ribosomal Protein L21 Pseudogene 38 | Pseudogene | 6 | GC02P171587 | 0.843972385 |
| RPL23P5 | Ribosomal Protein L23 Pseudogene 5 | Pseudogene | 6 | GC02P221583 | 0.843972385 |
| RPS2P18 | Ribosomal Protein S2 Pseudogene 18 | Pseudogene | 6 | GC02P173297 | 0.843972385 |
| lnc-GGH-4 | | RNA Gene | 6 | GC08M061661 | 0.843972385 |
| lnc-RNF19A-8 | | RNA Gene | 6 | GC08M100287 | 0.843972385 |
| lnc-SOX9-7 | | RNA Gene | 6 | GC17P072403 | 0.843972385 |
| lnc-TBX3-5 | | RNA Gene | 6 | GC12M114685 | 0.843972385 |
| lnc-TBX5-3 | | RNA Gene | 6 | GC12M114672 | 0.843972385 |
| lnc-TBX5-4 | | RNA Gene | 6 | GC12M114677 | 0.843972385 |
| lnc-UBLCP1-11 | | RNA Gene | 6 | GC05P158847 | 0.843972385 |
| ENSG00000235286 | Keratin 8 (KRT8) Pseudogene | Pseudogene | 5 | GC11M111670 | 0.843972385 |
| ENSG00000254851 | Proprotein Convertase Subtilisin/Kexin Type 7 (PCSK7) Pseudogene | Pseudogene | 5 | GC11P117136 | 0.843972385 |
| ENSG00000269001 | Zinc Finger Protein 818, Pseudogene | Pseudogene | 5 | GC19P064261 | 0.843972385 |
| ENSG00000269825 | Novel Zinc Finger Protein | Protein Coding | 5 | GC19M052650 | 0.843972385 |
| ENSG00000285551 | Novel Transcript | RNA Gene | 5 | GC10P062520 | 0.843972385 |
| ENSG00000285837 | Novel Protein | Protein Coding | 5 | GC10P062375 | 0.843972385 |
| HSALNG0021547 | | RNA Gene | 5 | GC02P201278 | 0.843972385 |
| HSALNG0046177 | | RNA Gene | 5 | GC05P158600 | 0.843972385 |
| HSALNG0049754 | | RNA Gene | 5 | GC06P081304 | 0.843972385 |
| HSALNG0068416 | | RNA Gene | 5 | GC08M131494 | 0.843972385 |
| HSALNG0079123 | | RNA Gene | 5 | GC10P091748 | 0.843972385 |
| HSALNG0085364 | | RNA Gene | 5 | GC11M069639 | 0.843972385 |
| HSALNG0094226 | | RNA Gene | 5 | GC12M114663 | 0.843972385 |
| HSALNG0102091 | | RNA Gene | 5 | GC14M068475 | 0.843972385 |
| HSALNG0102093 | | RNA Gene | 5 | GC14M068552 | 0.843972385 |

| | | | | | |
|---|---|---|---|---|---|
| KX404985 | | RNA Gene | 5 | GC08M061681 | 0.843972385 |
| L13304-024 | | RNA Gene | 5 | GC22M056438 | 0.843972385 |
| L13715-018 | | RNA Gene | 5 | GC22M056440 | 0.843972385 |
| LC413688 | | RNA Gene | 5 | GC11P069640 | 0.843972385 |
| MK280066 | | RNA Gene | 5 | GC11M069641 | 0.843972385 |
| MN298114-183 | | RNA Gene | 5 | GC06M064670 | 0.843972385 |
| MN308994 | | RNA Gene | 5 | GC01M155220 | 0.843972385 |
| NONHSAG030244.2 | | RNA Gene | 5 | GC02P201258 | 0.843972385 |
| NONHSAG043601.2 | | RNA Gene | 5 | GC06P080426 | 0.843972385 |
| NONHSAG046700.2 | | RNA Gene | 5 | GC06P080427 | 0.843972385 |
| PPIAP39 | Peptidylprolyl Isomerase A Pseudogene 39 | Pseudogene | 5 | GC10P113484 | 0.843972385 |
| RF00017-3533 | | RNA Gene | 5 | GC22P038389 | 0.843972385 |
| RF00017-4901 | | RNA Gene | 5 | GC06M014110 | 0.843972385 |
| RF00017-5096 | | RNA Gene | 5 | GC06P083320 | 0.843972385 |
| RF00017-5098 | | RNA Gene | 5 | GC06M065483 | 0.843972385 |
| RF00017-5100 | | RNA Gene | 5 | GC06M065484 | 0.843972385 |
| RN7SKP104 | RN7SK Pseudogene 104 | Pseudogene | 5 | GC07M097607 | 0.843972385 |
| RN7SKP242 | RN7SK Pseudogene 242 | Pseudogene | 5 | GC09M092459 | 0.843972385 |
| RN7SL383P | RNA, 7SL, Cytoplasmic 383, Pseudogene | Pseudogene | 5 | GC05P044782 | 0.843972385 |
| SINHCAFP3 | SINHCAF Pseudogene 3 | Pseudogene | 5 | GC08M023635 | 0.843972385 |
| VDAC1P10 | Voltage Dependent Anion Channel 1 Pseudogene 10 | Pseudogene | 5 | GC01P215376 | 0.843972385 |
| lnc-ARG2-1 | | RNA Gene | 5 | GC14P067601 | 0.843972385 |
| lnc-BAIAP2L2-4 | | RNA Gene | 5 | GC22M056452 | 0.843972385 |
| lnc-CD83-6 | | RNA Gene | 5 | GC06P014112 | 0.843972385 |
| lnc-CDYL2-1 | | RNA Gene | 5 | GC16M080597 | 0.843972385 |
| lnc-IQCH-7 | | RNA Gene | 5 | GC15P115806 | 0.843972385 |
| lnc-NDFIP1-2 | | RNA Gene | 5 | GC05P145969 | 0.843972385 |
| lnc-NRIP1-5 | | RNA Gene | 5 | GC21M015210 | 0.843972385 |
| lnc-ORAOV1-5 | | RNA Gene | 5 | GC11M087463 | 0.843972385 |
| lnc-PXYLP1-3 | | RNA Gene | 5 | GC03P141368 | 0.843972385 |
| lnc-RNF145-5 | | RNA Gene | 5 | GC05M158698 | 0.843972385 |
| lnc-SDSL-19 | | RNA Gene | 5 | GC12P114684 | 0.843972385 |
| lnc-SPRY4-3 | | RNA Gene | 5 | GC05M142286 | 0.843972385 |
| lnc-TFDP2-12 | | RNA Gene | 5 | GC03M141385 | 0.843972385 |
| lnc-TPCN2-5 | | RNA Gene | 5 | GC11P069820 | 0.843972385 |
| lnc-ZCCHC24-7 | | RNA Gene | 5 | GC10M079298 | 0.843972385 |
| piR-41513 | | RNA Gene | 5 | GC12P114758 | 0.843972385 |
| piR-59591 | | RNA Gene | 5 | GC03P141444 | 0.843972385 |
| AB372743 | | RNA Gene | 4 | GC05M056655 | 0.843972385 |
| ENSG00000226040 | Glycine Cleavage System Protein H (Aminomethyl Carrier) (GCSH) Pseudogene | Pseudogene | 4 | GC05M141896 | 0.843972385 |
| ENSG00000259502 | Protein Kinase C, Eta (PRKCH) Pseudogene | Pseudogene | 4 | GC14P067611 | 0.843972385 |
| HSALNG0024792 | | RNA Gene | 4 | GC03P027309 | 0.843972385 |
| HSALNG0058161 | | RNA Gene | 4 | GC07M055067 | 0.843972385 |
| HSALNG0058163 | | RNA Gene | 4 | GC07M055150 | 0.843972385 |
| HSALNG0076596 | | RNA Gene | 4 | GC10P021530 | 0.843972385 |
| HSALNG0078329 | | RNA Gene | 4 | GC10P062545 | 0.843972385 |
| HSALNG0079128 | | RNA Gene | 4 | GC10P091750 | 0.843972385 |
| HSALNG0126902 | | RNA Gene | 4 | GC19P064011 | 0.843972385 |
| HSALNG0131983 | | RNA Gene | 4 | GC21P015192 | 0.843972385 |
| LOC105375751 | Uncharacterized LOC105375751 | RNA Gene | 4 | GC08P126561 | 0.843972385 |
| LOC107986820 | Uncharacterized LOC107986820 | RNA Gene | 4 | GC07P093989 | 0.843972385 |
| MK280144-578 | | RNA Gene | 4 | GC06P054915 | 0.843972385 |
| MN309188 | | RNA Gene | 4 | GC10M079292 | 0.843972385 |
| NONHSAG005377.2 | | RNA Gene | 4 | GC10P021567 | 0.843972385 |
| RF00017-4262 | | RNA Gene | 4 | GC05P056716 | 0.843972385 |
| RF01061-114 | | RNA Gene | 4 | GC03M027364 | 0.843972385 |
| RN7SKP82 | RN7SK Pseudogene 82 | Pseudogene | 4 | GC04P042896 | 0.843972385 |
| lnc-FAM104A-8 | | RNA Gene | 4 | GC17M072932 | 0.843972385 |
| lnc-FBXO43-7 | | RNA Gene | 4 | GC08M100268 | 0.843972385 |
| lnc-IFT20-7 | | RNA Gene | 4 | GC17M034812 | 0.843972385 |
| lnc-SLC4A7-1 | | RNA Gene | 4 | GC03M027309 | 0.843972385 |
| lnc-VTI1B-2 | | RNA Gene | 4 | GC14M067589 | 0.843972385 |
| piR-32331-006 | | RNA Gene | 4 | GC06P080428 | 0.843972385 |
| piR-34696-018 | | RNA Gene | 4 | GC08P100316 | 0.843972385 |
| piR-43099-059 | | RNA Gene | 4 | GC10M079279 | 0.843972385 |
| piR-48343 | | RNA Gene | 4 | GC03M150743 | 0.843972385 |
| piR-50437-058 | | RNA Gene | 4 | GC10P062524 | 0.843972385 |
| piR-52373-003 | | RNA Gene | 4 | GC06P080429 | 0.843972385 |
| ENSG00000259648 | High Mobility Group Box 1 (HMGB1) Pseudogene | Pseudogene | 3 | GC14P067624 | 0.843972385 |
| ENSG00000278172 | Metazoan Signal Recognition Particle RNA | RNA Gene | 3 | GC13M029873 | 0.843972385 |
| ENSG00000287914 | Novel Transcript, Antisense To ESR1 | RNA Gene | 3 | GC06M152091 | 0.843972385 |
| HSALNG0021548 | | RNA Gene | 3 | GC02M201279 | 0.843972385 |
| HSALNG0025763 | | RNA Gene | 3 | GC03M047346 | 0.843972385 |
| HSALNG0063996 | | RNA Gene | 3 | GC08P023576 | 0.843972385 |
| HSALNG0073384 | | RNA Gene | 3 | GC09M104079 | 0.843972385 |
| HSALNG0127326 | | RNA Gene | 3 | GC19M052515 | 0.843972385 |
| HSALNG0127327 | | RNA Gene | 3 | GC19M052527 | 0.843972385 |
| HSALNG0129010 | | RNA Gene | 3 | GC20M019493 | 0.843972385 |
| L13714-198 | | RNA Gene | 3 | GC17M034802 | 0.843972385 |
| LOC105370543 | Uncharacterized LOC105370543 | RNA Gene | 3 | GC14P067600 | 0.843972385 |
| LOC105372657 | Uncharacterized LOC105372657 | RNA Gene | 3 | GC20P050350 | 0.843972385 |
| RF00017-1273 | | RNA Gene | 3 | GC13M029882 | 0.843972385 |
| RF00017-2331 | | RNA Gene | 3 | GC17M073036 | 0.843972385 |
| RF00994-1055 | | RNA Gene | 3 | GC08M023585 | 0.843972385 |
| lnc-TFPI2-2 | | RNA Gene | 3 | GC07M093972 | 0.843972385 |
| piR-35163 | | RNA Gene | 3 | GC17M034885 | 0.843972385 |
| piR-38562-078 | | RNA Gene | 3 | GC20P019538 | 0.843972385 |
| MIR3162 | MicroRNA 3162 | RNA Gene | 9 | GC11M059595 | 0.843845844 |
| SHISA5 | Shisa Family Member 5 | Protein Coding | 32 | GC03M048468 | 0.843319178 |
| ZNF335 | Zinc Finger Protein 335 | Protein Coding | 34 | GC20M045948 | 0.842459679 |
| MTPN | Myotrophin | Protein Coding | 31 | GC07M135926 | 0.842287779 |
| ZNF136 | Zinc Finger Protein 136 | Protein Coding | 32 | GC19P012163 | 0.84152317 |
| HERC2P9 | HERC2 Pseudogene 9 | Pseudogene | 11 | GC15P039313 | 0.841427863 |
| ERVK-10 | Endogenous Retrovirus Group K Member 10 | Protein Coding | 9 | GC05U901581 | 0.841412842 |
| AKAP2 | A-Kinase Anchoring Protein 2 | Protein Coding | 16 | GC00U936898 | 0.841336429 |

| | | | | | |
|---|---|---|---|---|---|
| ZCCHC2 | Zinc Finger CCHC-Type Containing 2 | Protein Coding | 30 | GC18P062523 | 0.841247439 |
| MIR4306 | MicroRNA 4306 | RNA Gene | 12 | GC13P099643 | 0.840881705 |
| CDC42EP4 | CDC42 Effector Protein 4 | Protein Coding | 34 | GC17M073283 | 0.840609729 |
| LMAN2 | Lectin, Mannose Binding 2 | Protein Coding | 34 | GC05M177913 | 0.840609729 |
| RAD54L2 | RAD54 Like 2 | Protein Coding | 34 | GC03P051538 | 0.840609729 |
| PPWD1 | Peptidylprolyl Isomerase Domain And WD Repeat Containing 1 | Protein Coding | 31 | GC05P065563 | 0.840609729 |
| ZNF316 | Zinc Finger Protein 316 | Protein Coding | 21 | GC07P006637 | 0.840609729 |
| ZNF69 | Zinc Finger Protein 69 | Protein Coding | 27 | GC19P011887 | 0.840161681 |
| GARRE1 | Granule Associated Rac And RHOG Effector 1 | Protein Coding | 23 | GC19P063412 | 0.839811206 |
| ACSF2 | Acyl-CoA Synthetase Family Member 2 | Protein Coding | 33 | GC17P050426 | 0.839799523 |
| UBTD1 | Ubiquitin Domain Containing 1 | Protein Coding | 30 | GC10P097498 | 0.839425325 |
| FABP9 | Fatty Acid Binding Protein 9 | Protein Coding | 27 | GC08M081458 | 0.838394761 |
| MIR3908 | MicroRNA 3908 | RNA Gene | 9 | GC12P123536 | 0.838300109 |
| ZNF618 | Zinc Finger Protein 618 | Protein Coding | 31 | GC09P113876 | 0.838282943 |
| MRPL46 | Mitochondrial Ribosomal Protein L46 | Protein Coding | 34 | GC15M088491 | 0.83814013 |
| PRMT8 | Protein Arginine Methyltransferase 8 | Protein Coding | 34 | GC12P003381 | 0.837998927 |
| PRPS2 | Phosphoribosyl Pyrophosphate Synthetase 2 | Protein Coding | 38 | GC0XP012791 | 0.837703943 |
| GTF3A | General Transcription Factor IIIA | Protein Coding | 31 | GC13P027427 | 0.837402046 |
| DEFB132 | Defensin Beta 132 | Protein Coding | 27 | GC20P000257 | 0.837378979 |
| IFNL1 | Interferon Lambda 1 | Protein Coding | 32 | GC19P039296 | 0.837227881 |
| RAB40C | RAB40C, Member RAS Oncogene Family | Protein Coding | 32 | GC16P010638 | 0.837182105 |
| TMED4 | Transmembrane P24 Trafficking Protein 4 | Protein Coding | 32 | GC07M044577 | 0.836737394 |
| C1orf174 | Chromosome 1 Open Reading Frame 174 | Protein Coding | 27 | GC01M003889 | 0.836737394 |
| TRIM65 | Tripartite Motif Containing 65 | Protein Coding | 30 | GC17M075880 | 0.836707652 |
| GPR171 | G Protein-Coupled Receptor 171 | Protein Coding | 31 | GC03M151197 | 0.836629033 |
| C19orf47 | Chromosome 19 Open Reading Frame 47 | Protein Coding | 28 | GC19M063789 | 0.836206138 |
| ZDHHC3 | Zinc Finger DHHC-Type Palmitoyltransferase 3 | Protein Coding | 33 | GC03M044915 | 0.835815549 |
| BTN3A3 | Butyrophilin Subfamily 3 Member A3 | Protein Coding | 32 | GC06P079888 | 0.835815549 |
| MIR4638 | MicroRNA 4638 | RNA Gene | 11 | GC05M181719 | 0.835815549 |
| MIR4782 | MicroRNA 4782 | RNA Gene | 10 | GC02M113721 | 0.835815549 |
| TUBB7P | Tubulin Beta 7 Pseudogene | Pseudogene | 13 | GC04M189982 | 0.834850192 |
| SLC37A1 | Solute Carrier Family 37 Member 1 | Protein Coding | 34 | GC21P042566 | 0.834770441 |
| ZBTB21 | Zinc Finger And BTB Domain Containing 21 | Protein Coding | 30 | GC21M041986 | 0.834337413 |
| ZCCHC17 | Zinc Finger CCHC-Type Containing 17 | Protein Coding | 30 | GC01P031298 | 0.834337413 |
| ALKBH7 | AlkB Homolog 7 | Protein Coding | 26 | GC19P006372 | 0.834337413 |
| LAMTOR4 | Late Endosomal/Lysosomal Adaptor, MAPK And MTOR Activator 4 | Protein Coding | 26 | GC07P100944 | 0.834337413 |
| MB21D2 | Mab-21 Domain Containing 2 | Protein Coding | 25 | GC03M192796 | 0.834337413 |
| EP400P1 | EP400 Pseudogene 1 | Pseudogene | 17 | GC12P132085 | 0.834337413 |
| RACGAP1P1 | Rac GTPase Activating Protein 1 Pseudogene 1 | Pseudogene | 9 | GC12M045064 | 0.834316611 |
| MIR6736 | MicroRNA 6736 | RNA Gene | 7 | GC01M145850 | 0.834316611 |
| LOC108964933 | CYP11A1 Promoter Region | Biological Region | 2 | GC15P114753 | 0.834316611 |
| MIR519A1 | MicroRNA 519a-1 | RNA Gene | 15 | GC19P053752 | 0.834242821 |
| CTBP1-DT | CTBP1 Divergent Transcript | RNA Gene | 14 | GC04P001250 | 0.834242821 |
| SAC3D1 | SAC3 Domain Containing 1 | Protein Coding | 27 | GC11P065040 | 0.834049821 |
| ERP44 | Endoplasmic Reticulum Protein 44 | Protein Coding | 37 | GC09M099979 | 0.833302617 |
| ZNF14 | Zinc Finger Protein 14 | Protein Coding | 32 | GC19M019710 | 0.832446158 |
| LINC00944 | Long Intergenic Non-Protein Coding RNA 944 | RNA Gene | 12 | GC12M126729 | 0.831398308 |
| MIR374C | MicroRNA 374c | RNA Gene | 12 | GC0XP074218 | 0.831398308 |
| MIR5188 | MicroRNA 5188 | RNA Gene | 12 | GC12P124915 | 0.831398308 |
| SAMSN1 | SAM Domain, SH3 Domain And Nuclear Localization Signals 1 | Protein Coding | 32 | GC21M014485 | 0.831037223 |
| ZNF184 | Zinc Finger Protein 184 | Protein Coding | 32 | GC06M063279 | 0.831037223 |
| ANO3 | Anoctamin 3 | Protein Coding | 36 | GC11P026188 | 0.830745816 |
| PP7080 | Uncharacterized LOC25845 | RNA Gene | 11 | GC05M000471 | 0.829759359 |
| SNAI1P1 | Snail Family Zinc Finger 1 Pseudogene 1 | Pseudogene | 7 | GC02P209808 | 0.829750121 |
| LINC02904 | Long Intergenic Non-Protein Coding RNA 2904 | RNA Gene | 19 | GC08P143040 | 0.829618633 |
| LEKR1 | Leucine, Glutamate And Lysine Rich 1 | Protein Coding | 28 | GC03P156825 | 0.829589963 |
| ATP6V1G3 | ATPase H+ Transporting V1 Subunit G3 | Protein Coding | 38 | GC01M198492 | 0.82920599 |
| TMEM65 | Transmembrane Protein 65 | Protein Coding | 28 | GC08M124310 | 0.82920599 |
| VSIG8 | V-Set And Immunoglobulin Domain Containing 8 | Protein Coding | 28 | GC01M159856 | 0.82920599 |
| NAA35 | N-Alpha-Acetyltransferase 35, NatC Auxiliary Subunit | Protein Coding | 30 | GC09P085941 | 0.829105616 |
| H4C7 | H4 Clustered Histone 7 | Protein Coding | 21 | GC06M064171 | 0.828968048 |
| RTE1 | Repressor Of Telomerase Expression 1 | Genetic Locus | 2 | GC00U916808 | 0.828968048 |
| ZNF266 | Zinc Finger Protein 266 | Protein Coding | 32 | GC19M009412 | 0.828692257 |
| GLYATL2 | Glycine-N-Acyltransferase Like 2 | Protein Coding | 28 | GC11M086976 | 0.828692257 |
| LINC02593 | Long Intergenic Non-Protein Coding RNA 2593 | RNA Gene | 11 | GC01M005505 | 0.828623414 |
| SV2B | Synaptic Vesicle Glycoprotein 2B | Protein Coding | 36 | GC15P091099 | 0.828564882 |
| STARD9 | StAR Related Lipid Transfer Domain Containing 9 | Protein Coding | 27 | GC15P042575 | 0.828226805 |
| OR51J1 | Olfactory Receptor Family 51 Subfamily J Member 1 | Pseudogene | 18 | GC11P005403 | 0.828226805 |
| MIR3189 | MicroRNA 3189 | RNA Gene | 13 | GC19P018386 | 0.828046143 |
| MIR4768 | MicroRNA 4768 | RNA Gene | 9 | GC0XP017447 | 0.828046143 |
| TMSB4Y | Thymosin Beta 4 Y-Linked | Protein Coding | 23 | GC0YP013703 | 0.82779479 |
| ERVK-8 | Endogenous Retrovirus Group K Member 8, Envelope | Protein Coding | 8 | GC08U901574 | 0.82779479 |
| ERVK-5 | Endogenous Retrovirus Group K Member 5 | Protein Coding | 7 | GC03U901800 | 0.82779479 |
| SPATA20 | Spermatogenesis Associated 20 | Protein Coding | 30 | GC17P050543 | 0.827772915 |
| GLIPR2 | GLI Pathogenesis Related 2 | Protein Coding | 33 | GC09P040184 | 0.827772856 |
| UBLCP1 | Ubiquitin Like Domain Containing CTD Phosphatase 1 | Protein Coding | 31 | GC05P159263 | 0.827772856 |
| ZBTB45 | Zinc Finger And BTB Domain Containing 45 | Protein Coding | 31 | GC19M058513 | 0.827678442 |
| KANK4 | KN Motif And Ankyrin Repeat Domains 4 | Protein Coding | 31 | GC01M062236 | 0.827505231 |
| PSPHP1 | Phosphoserine Phosphatase Pseudogene 1 | Pseudogene | 14 | GC07P055764 | 0.827324867 |
| SLFN14 | Schlafen Family Member 14 | Protein Coding | 30 | GC17M035576 | 0.827263355 |
| DGKB | Diacylglycerol Kinase Beta | Protein Coding | 40 | GC07M014145 | 0.826830208 |
| DUSP7 | Dual Specificity Phosphatase 7 | Protein Coding | 35 | GC03M052048 | 0.826428056 |
| PLEKHD1 | Pleckstrin Homology And Coiled-Coil Domain Containing D1 | Protein Coding | 23 | GC14P069484 | 0.825717986 |
| LRRC2 | Leucine Rich Repeat Containing 2 | Protein Coding | 32 | GC03M046515 | 0.825232983 |
| PRPF40B | Pre-MRNA Processing Factor 40 Homolog B | Protein Coding | 28 | GC12P049568 | 0.825232983 |
| PPP1R35 | Protein Phosphatase 1 Regulatory Subunit 35 | Protein Coding | 27 | GC07M101753 | 0.825232983 |
| TRAPPC14 | Trafficking Protein Particle Complex Subunit 14 | Protein Coding | 27 | GC07M101889 | 0.825232983 |
| TILRLS | TCL1A Interacting LncRNA, Retroperitoneal Liposarcoma Associated | RNA Gene | 3 | GC05U903354 | 0.824617624 |
| LENEP | Lens Epithelial Protein | Protein Coding | 23 | GC01P154993 | 0.824467063 |
| CLUL1 | Clusterin Like 1 | Protein Coding | 31 | GC18P000597 | 0.824460685 |
| PYDC1 | Pyrin Domain Containing 1 | Protein Coding | 28 | GC16M036585 | 0.824144602 |
| LOC101929767 | Uncharacterized LOC101929767 | RNA Gene | 7 | GC17M043148 | 0.823823273 |
| HMGN1P29 | High Mobility Group Nucleosome Binding Domain 1 Pseudogene 29 | Pseudogene | 3 | GC17P043144 | 0.823823273 |
| B3GAT1-DT | B3GAT1 Divergent Transcript | RNA Gene | 12 | GC11P134413 | 0.823323786 |
| INSC | INSC Spindle Orientation Adaptor Protein | Protein Coding | 32 | GC11P015127 | 0.823267817 |

| | | | | | |
|---|---|---|---|---|---|
| LMBR1L | Limb Development Membrane Protein 1 Like | Protein Coding | 32 | GC12M049096 | 0.823267817 |
| RND1 | Rho Family GTPase 1 | Protein Coding | 35 | GC12M048857 | 0.823160827 |
| ITPRIP | Inositol 1,4,5-Trisphosphate Receptor Interacting Protein | Protein Coding | 33 | GC10M104309 | 0.822525203 |
| C2orf49 | Chromosome 2 Open Reading Frame 49 | Protein Coding | 27 | GC02P105337 | 0.822525203 |
| PCDHGA7 | Protocadherin Gamma Subfamily A, 7 | Protein Coding | 27 | GC05P145838 | 0.822525203 |
| LOC108410393 | NR0B1 5' Regulatory Region | Biological Region | 2 | GC0XP030309 | 0.821954489 |
| MATN1 | Matrilin 1 | Protein Coding | 36 | GC01M030711 | 0.821363688 |
| UXS1 | UDP-Glucuronate Decarboxylase 1 | Protein Coding | 35 | GC02M106094 | 0.820871115 |
| ANKRD40CL | ANKRD40 C-Terminal Like | Protein Coding | 15 | GC17M050801 | 0.820871115 |
| MIR3182 | MicroRNA 3182 | RNA Gene | 11 | GC16P083508 | 0.820871115 |
| GMIP | GEM Interacting Protein | Protein Coding | 34 | GC19M019629 | 0.820620835 |
| NDUFA7 | NADH:Ubiquinone Oxidoreductase Subunit A7 | Protein Coding | 33 | GC19M008308 | 0.820620835 |
| CYTL1 | Cytokine Like 1 | Protein Coding | 29 | GC04M005016 | 0.820620835 |
| SEPTIN8 | Septin 8 | Protein Coding | 29 | GC05M132751 | 0.820475757 |
| BORA | BORA Aurora Kinase A Activator | Protein Coding | 30 | GC13P072727 | 0.820419252 |
| C1QTNF3 | C1q And TNF Related 3 | Protein Coding | 33 | GC05M034017 | 0.820103168 |
| LINC00921 | Long Intergenic Non-Protein Coding RNA 921 | RNA Gene | 12 | GC16P010782 | 0.819709957 |
| MIR3157 | MicroRNA 3157 | RNA Gene | 13 | GC10M096064 | 0.819698989 |
| MIR4691 | MicroRNA 4691 | RNA Gene | 13 | GC11P068033 | 0.819698989 |
| MIR4772 | MicroRNA 4772 | RNA Gene | 13 | GC02P102432 | 0.819698989 |
| MIR4651 | MicroRNA 4651 | RNA Gene | 12 | GC07P075915 | 0.819698989 |
| MIR3659 | MicroRNA 3659 | RNA Gene | 11 | GC01P038089 | 0.819698989 |
| MIR4474 | MicroRNA 4474 | RNA Gene | 10 | GC09M020502 | 0.819698989 |
| CST9L | Cystatin 9 Like | Protein Coding | 32 | GC20M023564 | 0.819631338 |
| DNAJC3-DT | DNAJC3 Divergent Transcript | RNA Gene | 12 | GC13M095649 | 0.819277644 |
| PXMP2 | Peroxisomal Membrane Protein 2 | Protein Coding | 32 | GC12P132687 | 0.819165826 |
| GALNTL5 | Polypeptide N-Acetylgalactosaminyltransferase Like 5 | Protein Coding | 34 | GC07P151956 | 0.818534732 |
| DDR1-DT | DDR1 Divergent Transcript | RNA Gene | 11 | GC06M063536 | 0.818363249 |
| PRDM4 | PR/SET Domain 4 | Protein Coding | 32 | GC12M107732 | 0.818162084 |
| DNASE1L2 | Deoxyribonuclease 1 Like 2 | Protein Coding | 32 | GC16P002235 | 0.817415237 |
| RGPD5 | RANBP2 Like And GRIP Domain Containing 5 | Protein Coding | 27 | GC02P109760 | 0.817099035 |
| SLC50A1 | Solute Carrier Family 50 Member 1 | Protein Coding | 30 | GC01P155135 | 0.816697657 |
| MFSD6 | Major Facilitator Superfamily Domain Containing 6 | Protein Coding | 31 | GC02P190408 | 0.815933108 |
| SULT1B1 | Sulfotransferase Family 1B Member 1 | Protein Coding | 36 | GC04M069721 | 0.815389991 |
| TMEM248 | Transmembrane Protein 248 | Protein Coding | 28 | GC07P066921 | 0.815389991 |
| GCAT | Glycine C-Acetyltransferase | Protein Coding | 38 | GC22P037807 | 0.814920664 |
| GALNT16 | Polypeptide N-Acetylgalactosaminyltransferase 16 | Protein Coding | 31 | GC14P069259 | 0.814769864 |
| RSKR | Ribosomal Protein S6 Kinase Related | Protein Coding | 23 | GC17M035116 | 0.814561903 |
| LGALS7B | Galectin 7B | Protein Coding | 28 | GC19P063539 | 0.814167738 |
| KCNG3 | Potassium Voltage-Gated Channel Modifier Subfamily G Member 3 | Protein Coding | 35 | GC02M042442 | 0.813573956 |
| MTERF3 | Mitochondrial Transcription Termination Factor 3 | Protein Coding | 27 | GC08M096240 | 0.813573956 |
| C1orf226 | Chromosome 1 Open Reading Frame 226 | Protein Coding | 25 | GC01P162378 | 0.813573956 |
| MIR3118-1 | MicroRNA 3118-1 | RNA Gene | 10 | GC21M013644 | 0.813573956 |
| ZNF415 | Zinc Finger Protein 415 | Protein Coding | 30 | GC19M053107 | 0.813125968 |
| SLC4A8 | Solute Carrier Family 4 Member 8 | Protein Coding | 35 | GC12P051391 | 0.813012421 |
| EARS2 | Glutamyl-TRNA Synthetase 2, Mitochondrial | Protein Coding | 38 | GC16M023538 | 0.812595487 |
| RHOJ | Ras Homolog Family Member J | Protein Coding | 34 | GC14P063204 | 0.812329412 |
| GPR50 | G Protein-Coupled Receptor 50 | Protein Coding | 36 | GC0XP151176 | 0.812190771 |
| GIMAP4 | GTPase, IMAP Family Member 4 | Protein Coding | 34 | GC07P150568 | 0.811655045 |
| RNF152 | Ring Finger Protein 152 | Protein Coding | 32 | GC18M061808 | 0.811543822 |
| RAB3A | RAB3A, Member RAS Oncogene Family | Protein Coding | 38 | GC19M018196 | 0.811378837 |
| A3GALT2 | Alpha 1,3-Galactosyltransferase 2 | Protein Coding | 25 | GC01M033306 | 0.811075389 |
| MIR2110 | MicroRNA 2110 | RNA Gene | 13 | GC10M114174 | 0.810899556 |
| MIR3140 | MicroRNA 3140 | RNA Gene | 13 | GC04M152490 | 0.810621977 |
| MIR3691 | MicroRNA 3691 | RNA Gene | 11 | GC06M005265 | 0.810621977 |
| MIR3940 | MicroRNA 3940 | RNA Gene | 11 | GC19M006421 | 0.810621977 |
| MIR4674 | MicroRNA 4674 | RNA Gene | 11 | GC09M136546 | 0.810621977 |
| MIR4746 | MicroRNA 4746 | RNA Gene | 11 | GC19P004572 | 0.810621977 |
| MIR4793 | MicroRNA 4793 | RNA Gene | 11 | GC03M048644 | 0.810621977 |
| MIR4775 | MicroRNA 4775 | RNA Gene | 10 | GC02P207754 | 0.810621977 |
| MIR4635 | MicroRNA 4635 | RNA Gene | 9 | GC05M001109 | 0.810621977 |
| MIR4718 | MicroRNA 4718 | RNA Gene | 9 | GC16P012720 | 0.810621977 |
| TRBV18 | T Cell Receptor Beta Variable 18 | Protein Coding | 8 | GC07P148249 | 0.810621977 |
| SLC22A18AS | SLC22A18 Antisense RNA | RNA Gene | 26 | GC11M003100 | 0.810477734 |
| ARHGAP4 | Rho GTPase Activating Protein 4 | Protein Coding | 38 | GC0XM153907 | 0.810168028 |
| MRPL57 | Mitochondrial Ribosomal Protein L57 | Protein Coding | 26 | GC13P021176 | 0.810005546 |
| MRPL1 | Mitochondrial Ribosomal Protein L1 | Protein Coding | 33 | GC04P077862 | 0.809908688 |
| OPN1LW | Opsin 1, Long Wave Sensitive | Protein Coding | 34 | GC0XP154144 | 0.809272528 |
| CNN3 | Calponin 3 | Protein Coding | 33 | GC01M094896 | 0.808958113 |
| PRB3 | Proline Rich Protein BstNI Subfamily 3 | Protein Coding | 26 | GC12M011265 | 0.808712482 |
| GALNT8 | Polypeptide N-Acetylgalactosaminyltransferase 8 | Protein Coding | 31 | GC12P004720 | 0.80848819 |
| PYROXD2 | Pyridine Nucleotide-Disulphide Oxidoreductase Domain 2 | Protein Coding | 30 | GC10M098383 | 0.80848819 |
| IGHG4 | Immunoglobulin Heavy Constant Gamma 4 (G4m Marker) | Protein Coding | 25 | GC14M112402 | 0.80848819 |
| SHOC1 | Shortage In Chiasmata 1 | Protein Coding | 22 | GC09M111689 | 0.80848819 |
| IGLC1 | Immunoglobulin Lambda Constant 1 | Protein Coding | 21 | GC22P022895 | 0.80848819 |
| MIR559 | MicroRNA 559 | RNA Gene | 11 | GC02P047388 | 0.80848819 |
| PLPP2 | Phospholipid Phosphatase 2 | Protein Coding | 32 | GC19M004764 | 0.807769656 |
| PXYLP1 | 2-Phosphoxylose Phosphatase 1 | Protein Coding | 30 | GC03P141228 | 0.807769656 |
| LINC01094 | Long Intergenic Non-Protein Coding RNA 1094 | RNA Gene | 13 | GC04P078681 | 0.807769656 |
| PCSK5 | Proprotein Convertase Subtilisin/Kexin Type 5 | Protein Coding | 38 | GC09P075890 | 0.807227612 |
| CC2D1B | Coiled-Coil And C2 Domain Containing 1B | Protein Coding | 28 | GC01M052346 | 0.807058871 |
| GOSR2 | Golgi SNAP Receptor Complex Member 2 | Protein Coding | 38 | GC17P053365 | 0.806437612 |
| PLCL2 | Phospholipase C Like 2 | Protein Coding | 34 | GC03P016924 | 0.805843651 |
| ARHGAP25 | Rho GTPase Activating Protein 25 | Protein Coding | 31 | GC02P068679 | 0.805843651 |
| IER2 | Immediate Early Response 2 | Protein Coding | 29 | GC19P013150 | 0.805843651 |
| GML | Glycosylphosphatidylinositol Anchored Molecule Like | Protein Coding | 28 | GC08P142834 | 0.80581063 |
| MAK16 | MAK16 Homolog | Protein Coding | 29 | GC08P033486 | 0.80580461 |
| DNAAF10 | Dynein Axonemal Assembly Factor 10 | Protein Coding | 25 | GC02M068127 | 0.80580461 |
| MIR519B | MicroRNA 519b | RNA Gene | 16 | GC19P053695 | 0.80580461 |
| MIR4428 | MicroRNA 4428 | RNA Gene | 10 | GC01P237471 | 0.805356562 |
| MIR4480 | MicroRNA 4480 | RNA Gene | 9 | GC10P012578 | 0.805356562 |
| MIR6744 | MicroRNA 6744 | RNA Gene | 7 | GC11P001256 | 0.805356562 |
| C5orf24 | Chromosome 5 Open Reading Frame 24 | Protein Coding | 27 | GC05P134845 | 0.805260837 |
| GJC3 | Gap Junction Protein Gamma 3 | Protein Coding | 35 | GC07M099923 | 0.804478884 |
| NT5C3A | 5'-Nucleotidase, Cytosolic IIIA | Protein Coding | 36 | GC07M033014 | 0.80433929 |

| | | | | | | |
|---|---|---|---|---|---|---|
| MIR3944 | MicroRNA 3944 | RNA Gene | 13 | GC10M133371 | 0.804297447 |
| MIR4671 | MicroRNA 4671 | RNA Gene | 11 | GC01P234306 | 0.804297447 |
| PISRT1 | PISRT1 LncRNA | RNA Gene | 14 | GC03M139232 | 0.804232121 |
| SLC25A38 | Solute Carrier Family 25 Member 38 | Protein Coding | 34 | GC03P039845 | 0.804100633 |
| LHX1 | LIM Homeobox 1 | Protein Coding | 38 | GC17P052850 | 0.804009676 |
| DCXR | Dicarbonyl And L-Xylulose Reductase | Protein Coding | 39 | GC17M082036 | 0.803664148 |
| TNMD | Tenomodulin | Protein Coding | 32 | GC0XP100584 | 0.802969575 |
| NLRP13 | NLR Family Pyrin Domain Containing 13 | Protein Coding | 31 | GC19M064444 | 0.802969575 |
| ZBP1 | Z-DNA Binding Protein 1 | Protein Coding | 35 | GC20M057603 | 0.802519441 |
| LINC00676 | Long Intergenic Non-Protein Coding RNA 676 | RNA Gene | 12 | GC13P109728 | 0.802072406 |
| CCDC106 | Coiled-Coil Domain Containing 106 | Protein Coding | 28 | GC19P055641 | 0.801291943 |
| ERMAP | Erythroblast Membrane Associated Protein (Scianna Blood Group) | Protein Coding | 35 | GC01P042819 | 0.801191092 |
| AKAP7 | A-Kinase Anchoring Protein 7 | Protein Coding | 34 | GC06P131126 | 0.801191092 |
| TMX4 | Thioredoxin Related Transmembrane Protein 4 | Protein Coding | 33 | GC20M007977 | 0.801191092 |
| ZNF282 | Zinc Finger Protein 282 | Protein Coding | 30 | GC07P149195 | 0.801191092 |
| C9orf43 | Chromosome 9 Open Reading Frame 43 | Protein Coding | 28 | GC09P113410 | 0.801191092 |
| PHACTR2-AS1 | PHACTR2 Antisense RNA 1 | RNA Gene | 11 | GC06M143555 | 0.801191092 |
| MIR3150A | MicroRNA 3150a | RNA Gene | 13 | GC08P095072 | 0.800581813 |
| MIR4767 | MicroRNA 4767 | RNA Gene | 12 | GC0XP007148 | 0.800581813 |
| MIR4739 | MicroRNA 4739 | RNA Gene | 10 | GC17M079707 | 0.800581813 |
| JDP2 | Jun Dimerization Protein 2 | Protein Coding | 36 | GC14P075427 | 0.80048418 |
| P4HTM | Prolyl 4-Hydroxylase, Transmembrane | Protein Coding | 33 | GC03P049562 | 0.80043906 |
| KLRC3 | Killer Cell Lectin Like Receptor C3 | Protein Coding | 31 | GC12M020445 | 0.800221741 |
| HNRNPCL1 | Heterogeneous Nuclear Ribonucleoprotein C Like 1 | Protein Coding | 26 | GC01M012848 | 0.80004108 |
| ZNF652-AS1 | ZNF652 Antisense RNA 1 | RNA Gene | 10 | GC17P053514 | 0.799891472 |
| COMMD3 | COMM Domain Containing 3 | Protein Coding | 30 | GC10P022315 | 0.79935956 |
| DNAJC19 | DnaJ Heat Shock Protein Family (Hsp40) Member C19 | Protein Coding | 38 | GC03M180983 | 0.799353957 |
| OR5F1 | Olfactory Receptor Family 5 Subfamily F Member 1 | Protein Coding | 28 | GC11M086939 | 0.798805594 |
| WFDC8 | WAP Four-Disulfide Core Domain 8 | Protein Coding | 28 | GC20M045551 | 0.798805594 |
| XG | Xg Glycoprotein (Xg Blood Group) | Protein Coding | 28 | GC0XP002752 | 0.798805594 |
| FAM9A | Family With Sequence Similarity 9 Member A | Protein Coding | 25 | GC0XM008790 | 0.798805594 |
| WDR11-DT | WDR11 Divergent Transcript | RNA Gene | 13 | GC10M120757 | 0.798805594 |
| ANOS2P | Anosmin 2, Pseudogene | Pseudogene | 6 | GC0YP013705 | 0.798805594 |
| ARL6IP1 | ADP Ribosylation Factor Like GTPase 6 Interacting Protein 1 | Protein Coding | 36 | GC16M019195 | 0.798778951 |
| GALNT10 | Polypeptide N-Acetylgalactosaminyltransferase 10 | Protein Coding | 35 | GC05P154165 | 0.798395038 |
| MIR3170 | MicroRNA 3170 | RNA Gene | 13 | GC13P098208 | 0.797959685 |
| ZBTB32 | Zinc Finger And BTB Domain Containing 32 | Protein Coding | 34 | GC19P063457 | 0.797757745 |
| ZC3H3 | Zinc Finger CCCH-Type Containing 3 | Protein Coding | 30 | GC08M143437 | 0.797457397 |
| ZFP30 | ZFP30 Zinc Finger Protein | Protein Coding | 31 | GC19M037613 | 0.797407627 |
| TIGD2 | Tigger Transposable Element Derived 2 | Protein Coding | 28 | GC04P089112 | 0.797009408 |
| FAM216B | Family With Sequence Similarity 216 Member B | Protein Coding | 24 | GC13P042781 | 0.797009408 |
| CCDC169 | Coiled-Coil Domain Containing 169 | Protein Coding | 22 | GC13M036222 | 0.797009408 |
| FGF13-AS1 | FGF13 Antisense RNA 1 | RNA Gene | 12 | GC0XP138711 | 0.797009408 |
| CTDSPL2 | CTD Small Phosphatase Like 2 | Protein Coding | 32 | GC15P044427 | 0.79676652 |
| STK32A | Serine/Threonine Kinase 32A | Protein Coding | 32 | GC05P147234 | 0.79676652 |
| SLC44A1 | Solute Carrier Family 44 Member 1 | Protein Coding | 40 | GC09P105244 | 0.79661721 |
| HMCES | 5-Hydroxymethylcytosine Binding, ES Cell Specific | Protein Coding | 27 | GC03P129278 | 0.796208024 |
| LINC00243 | Long Intergenic Non-Protein Coding RNA 243 | RNA Gene | 17 | GC06M063530 | 0.795803487 |
| DEPDC4 | DEP Domain Containing 4 | Protein Coding | 26 | GC12M100203 | 0.795360565 |
| MTMR6 | Myotubularin Related Protein 6 | Protein Coding | 35 | GC13M025246 | 0.795158327 |
| SLC25A29 | Solute Carrier Family 25 Member 29 | Protein Coding | 35 | GC14M100514 | 0.794909656 |
| PCDHB7 | Protocadherin Beta 7 | Protein Coding | 31 | GC05P145816 | 0.7942518 |
| RNF112 | Ring Finger Protein 112 | Protein Coding | 31 | GC17P052295 | 0.7942518 |
| MFSD10 | Major Facilitator Superfamily Domain Containing 10 | Protein Coding | 30 | GC04M002903 | 0.7942518 |
| OR4L1 | Olfactory Receptor Family 4 Subfamily L Member 1 | Protein Coding | 30 | GC14P020060 | 0.7942518 |
| CFAP54 | Cilia And Flagella Associated Protein 54 | Protein Coding | 21 | GC12P096489 | 0.7942518 |
| UPF3B | UPF3B Regulator Of Nonsense Mediated MRNA Decay | Protein Coding | 36 | GC0XM119805 | 0.794012368 |
| CARMIL1 | Capping Protein Regulator And Myosin 1 Linker 1 | Protein Coding | 28 | GC06P025354 | 0.793669462 |
| CHCHD1 | Coiled-Coil-Helix-Coiled-Coil-Helix Domain Containing 1 | Protein Coding | 30 | GC10P073782 | 0.793476582 |
| CCNYL1 | Cyclin Y Like 1 | Protein Coding | 28 | GC02P207712 | 0.793476582 |
| TBC1D19 | TBC1 Domain Family Member 19 | Protein Coding | 28 | GC04P026585 | 0.793476582 |
| ARHGAP20 | Rho GTPase Activating Protein 20 | Protein Coding | 31 | GC11M110577 | 0.793421686 |
| IGLC3 | Immunoglobulin Lambda Constant 3 (Kern-Oz+ Marker) | Protein Coding | 15 | GC22P034649 | 0.793421686 |
| MIR550A2 | MicroRNA 550a-2 | RNA Gene | 14 | GC07P032733 | 0.793421686 |
| NHLH2 | Nescient Helix-Loop-Helix 2 | Protein Coding | 34 | GC01M115836 | 0.792703807 |
| SAA3P | Serum Amyloid A3, Pseudogene | Pseudogene | 13 | GC11M018112 | 0.792677581 |
| LRRC3 | Leucine Rich Repeat Containing 3 | Protein Coding | 27 | GC21P044455 | 0.790832698 |
| HES7 | Hes Family BHLH Transcription Factor 7 | Protein Coding | 34 | GC17M008120 | 0.790181935 |
| NAA30 | N-Alpha-Acetyltransferase 30, NatC Catalytic Subunit | Protein Coding | 32 | GC14P057390 | 0.78976357 |
| CYMP-AS1 | CYMP Antisense RNA 1 | RNA Gene | 10 | GC01M110488 | 0.789433897 |
| TTLL11 | Tubulin Tyrosine Ligase Like 11 | Protein Coding | 30 | GC09M121816 | 0.789137244 |
| POLE4 | DNA Polymerase Epsilon 4, Accessory Subunit | Protein Coding | 33 | GC02P074958 | 0.788380384 |
| ZNF593 | Zinc Finger Protein 593 | Protein Coding | 30 | GC01P026171 | 0.788380384 |
| FAM102B | Family With Sequence Similarity 102 Member B | Protein Coding | 25 | GC01P108560 | 0.788380384 |
| MIR620 | MicroRNA 620 | RNA Gene | 11 | GC12M116148 | 0.788380384 |
| MOB1B | MOB Kinase Activator 1B | Protein Coding | 32 | GC04P070902 | 0.788283467 |
| USP17L9P | Ubiquitin Specific Peptidase 17 Like Family Member 9, Pseudogene | Pseudogene | 13 | GC04P009362 | 0.788160861 |
| DEPP1 | DEPP1 Autophagy Regulator | Protein Coding | 25 | GC10M044971 | 0.787932396 |
| ZSCAN4 | Zinc Finger And SCAN Domain Containing 4 | Protein Coding | 31 | GC19P057651 | 0.787880778 |
| LINC01235 | Long Intergenic Non-Protein Coding RNA 1235 | RNA Gene | 11 | GC09M013407 | 0.787857771 |
| KRT40 | Keratin 40 | Protein Coding | 29 | GC17M040977 | 0.787803054 |
| SH3YL1 | SH3 And SYLF Domain Containing 1 | Protein Coding | 30 | GC02M000208 | 0.787704468 |
| CCDC28B | Coiled-Coil Domain Containing 28B | Protein Coding | 32 | GC01P032200 | 0.787575662 |
| COL6A5 | Collagen Type VI Alpha 5 Chain | Protein Coding | 30 | GC03P130345 | 0.787000656 |
| MISP3 | MISP Family Member 3 | Protein Coding | 17 | GC19P014072 | 0.785597682 |
| NEGR1 | Neuronal Growth Regulator 1 | Protein Coding | 35 | GC01M071395 | 0.785379529 |
| METTL17 | Methyltransferase Like 17 | Protein Coding | 28 | GC14P031553 | 0.785365582 |
| KRT34 | Keratin 34 | Protein Coding | 31 | GC17M041377 | 0.785135329 |
| GTF2A1L | General Transcription Factor IIA Subunit 1 Like | Protein Coding | 32 | GC02P048617 | 0.785074532 |
| SGIP1 | SH3GL Interacting Endocytic Adaptor 1 | Protein Coding | 32 | GC01P066533 | 0.785074532 |
| PPIL6 | Peptidylprolyl Isomerase Like 6 | Protein Coding | 31 | GC06M109390 | 0.785074532 |
| ELFN2 | Extracellular Leucine Rich Repeat And Fibronectin Type III Domain Containing 2 | Protein Coding | 30 | GC22M037344 | 0.785074532 |
| METTL6 | Methyltransferase 6, Methylcytidine | Protein Coding | 30 | GC03M020240 | 0.785074532 |
| SLC22A25 | Solute Carrier Family 22 Member 25 | Protein Coding | 30 | GC11M087157 | 0.785074532 |
| ATP5MC1 | ATP Synthase Membrane Subunit C Locus 1 | Protein Coding | 28 | GC17P053491 | 0.785074532 |

| | | | | | |
|---|---|---|---|---|---|
| KLHL34 | Kelch Like Family Member 34 | Protein Coding | 25 | GC0XM021654 | 0.785074532 |
| C1orf185 | Chromosome 1 Open Reading Frame 185 | Protein Coding | 22 | GC01P051102 | 0.785074532 |
| IGHV3-23 | Immunoglobulin Heavy Variable 3-23 | Protein Coding | 16 | GC14M106268 | 0.785074532 |
| MIR4301 | MicroRNA 4301 | RNA Gene | 11 | GC11M113451 | 0.785074532 |
| LRRC37B | Leucine Rich Repeat Containing 37B | Protein Coding | 30 | GC17P052631 | 0.784920096 |
| DEGS2 | Delta 4-Desaturase, Sphingolipid 2 | Protein Coding | 34 | GC14M100143 | 0.784869492 |
| CASP12 | Caspase 12 (Gene/Pseudogene) | Protein Coding | 30 | GC11M104885 | 0.784869492 |
| SGSM2 | Small G Protein Signaling Modulator 2 | Protein Coding | 33 | GC17P002352 | 0.784688234 |
| CCDC117 | Coiled-Coil Domain Containing 117 | Protein Coding | 27 | GC22P028772 | 0.784407377 |
| PROSER2 | Proline And Serine Rich 2 | Protein Coding | 25 | GC10P011865 | 0.784407377 |
| TPRA1 | Transmembrane Protein Adipocyte Associated 1 | Protein Coding | 30 | GC03M127573 | 0.783324122 |
| TBC1D8 | TBC1 Domain Family Member 8 | Protein Coding | 31 | GC02M101007 | 0.78324765 |
| MACIR | Macrophage Immunometabolism Regulator | Protein Coding | 24 | GC05P103259 | 0.78311044 |
| URAD | Ureidoimidazoline (2-Oxo-4-Hydroxy-4-Carboxy-5-) Decarboxylase | Protein Coding | 22 | GC13M027978 | 0.78147471 |
| SPATA4 | Spermatogenesis Associated 4 | Protein Coding | 30 | GC04M176184 | 0.780302763 |
| BCAN | Brevican | Protein Coding | 36 | GC01P156641 | 0.780220389 |
| SEPHS2 | Selenophosphate Synthetase 2 | Protein Coding | 34 | GC16M036523 | 0.780220389 |
| ARSD-AS1 | ARSD Antisense RNA 1 | RNA Gene | 9 | GC0XP002904 | 0.780220389 |
| NLRC3 | NLR Family CARD Domain Containing 3 | Protein Coding | 32 | GC16M003539 | 0.780154765 |
| CAPN13 | Calpain 13 | Protein Coding | 35 | GC02M030722 | 0.779663563 |
| TRPT1 | TRNA Phosphotransferase 1 | Protein Coding | 29 | GC11M064223 | 0.779277682 |
| YARS2 | Tyrosyl-TRNA Synthetase 2 | Protein Coding | 39 | GC12M032725 | 0.778533995 |
| ZNF777 | Zinc Finger Protein 777 | Protein Coding | 30 | GC07M149431 | 0.77834022 |
| SPRR2E | Small Proline Rich Protein 2E | Protein Coding | 25 | GC01M153093 | 0.77786392 |
| LINC00885 | Long Intergenic Non-Protein Coding RNA 885 | RNA Gene | 14 | GC03P196240 | 0.777471364 |
| NDEL1 | NudE Neurodevelopment Protein 1 Like 1 | Protein Coding | 36 | GC17P010932 | 0.7768749 |
| MIR3688-1 | MicroRNA 3688-1 | RNA Gene | 13 | GC04M159128 | 0.776812196 |
| HOXD4 | Homeobox D4 | Protein Coding | 35 | GC02P176166 | 0.776634932 |
| ADGRE3 | Adhesion G Protein-Coupled Receptor E3 | Protein Coding | 30 | GC19M014619 | 0.776634932 |
| TMEM266 | Transmembrane Protein 266 | Protein Coding | 26 | GC15P114843 | 0.776634932 |
| CPNE2 | Copine 2 | Protein Coding | 30 | GC16P057126 | 0.77656734 |
| FAM200A | Family With Sequence Similarity 200 Member A | Protein Coding | 25 | GC07M099546 | 0.77656734 |
| NTMT1 | N-Terminal Xaa-Pro-Lys N-Methyltransferase 1 | Protein Coding | 32 | GC09P129608 | 0.776225984 |
| TLL2 | Tolloid Like 2 | Protein Coding | 36 | GC10M096364 | 0.775997519 |
| NUDT13 | Nudix Hydrolase 13 | Protein Coding | 32 | GC10P073110 | 0.775997519 |
| SUN3 | Sad1 And UNC84 Domain Containing 3 | Protein Coding | 31 | GC07M047987 | 0.775997519 |
| THAP2 | THAP Domain Containing 2 | Protein Coding | 30 | GC12P071663 | 0.775997519 |
| WDR31 | WD Repeat Domain 31 | Protein Coding | 29 | GC09M113313 | 0.775997519 |
| MS4A10 | Membrane Spanning 4-Domains A10 | Protein Coding | 28 | GC11P060789 | 0.775997519 |
| ACCSL | 1-Aminocyclopropane-1-Carboxylate Synthase Homolog (Inactive) Like | Protein Coding | 27 | GC11P044027 | 0.775997519 |
| CLECL1 | C-Type Lectin Like 1 | Pseudogene | 27 | GC12M020433 | 0.775997519 |
| PP2D1 | Protein Phosphatase 2C Like Domain Containing 1 | Protein Coding | 26 | GC03M019979 | 0.775997519 |
| TEDDM1 | Transmembrane Epididymal Protein 1 | Protein Coding | 23 | GC01M182398 | 0.775997519 |
| TRIM75 | Tripartite Motif Containing 75 | Protein Coding | 14 | GC04P165169 | 0.775997519 |
| IGHV3-30 | Immunoglobulin Heavy Variable 3-30 | Protein Coding | 12 | GC14M112541 | 0.775997519 |
| IGHV3-13 | Immunoglobulin Heavy Variable 3-13 | Protein Coding | 11 | GC14M106129 | 0.775997519 |
| USP6NL-AS1 | USP6NL Antisense RNA 1 | RNA Gene | 10 | GC10P011614 | 0.775997519 |
| KPRP | Keratinocyte Proline Rich Protein | Protein Coding | 27 | GC01P152773 | 0.775514841 |
| ACAA2 | Acetyl-CoA Acyltransferase 2 | Protein Coding | 39 | GC18M049782 | 0.774852872 |
| FNTB | Farnesyltransferase, CAAX Box, Beta | Protein Coding | 39 | GC14P064986 | 0.77471453 |
| TRIM51 | Tripartite Motif-Containing 51 | Protein Coding | 25 | GC11P055883 | 0.774201572 |
| BPIFA2 | BPI Fold Containing Family A Member 2 | Protein Coding | 27 | GC20P033161 | 0.772626996 |
| ATP4B | ATPase H+/K+ Transporting Subunit Beta | Protein Coding | 37 | GC13M113648 | 0.772408247 |
| ATXN7L3B | Ataxin 7 Like 3B | Protein Coding | 27 | GC12P074537 | 0.771990478 |
| MIR1587 | MicroRNA 1587 | RNA Gene | 10 | GC0XP039837 | 0.771873236 |
| GYPE | Glycophorin E (MNS Blood Group) | Protein Coding | 27 | GC04M143870 | 0.771408319 |
| SUPV3L1 | Suv3 Like RNA Helicase | Protein Coding | 34 | GC10P069182 | 0.771284342 |
| TEX29 | Testis Expressed 29 | Protein Coding | 26 | GC13P111306 | 0.771271706 |
| ARRDC2 | Arrestin Domain Containing 2 | Protein Coding | 31 | GC19P063117 | 0.770889521 |
| ZBED9 | Zinc Finger BED-Type Containing 9 | Protein Coding | 27 | GC06M063367 | 0.770889521 |
| KDELR3 | KDEL Endoplasmic Reticulum Protein Retention Receptor 3 | Protein Coding | 32 | GC22P038468 | 0.770295024 |
| RAB19 | RAB19, Member RAS Oncogene Family | Protein Coding | 29 | GC07P140403 | 0.770295024 |
| OLFM3 | Olfactomedin 3 | Protein Coding | 34 | GC01M101802 | 0.769620419 |
| DNAH14 | Dynein Axonemal Heavy Chain 14 | Protein Coding | 27 | GC01P224896 | 0.768815815 |
| ZNF407 | Zinc Finger Protein 407 | Protein Coding | 31 | GC18P074597 | 0.768707395 |
| MIF4GD | MIF4G Domain Containing | Protein Coding | 30 | GC17M075266 | 0.768533826 |
| UBE2D4 | Ubiquitin Conjugating Enzyme E2 D4 (Putative) | Protein Coding | 34 | GC07P043926 | 0.768107295 |
| GXYLT2 | Glucoside Xylosyltransferase 2 | Protein Coding | 32 | GC03P072888 | 0.768107295 |
| ASB8 | Ankyrin Repeat And SOCS Box Containing 8 | Protein Coding | 31 | GC12M048147 | 0.768084049 |
| MRPS10 | Mitochondrial Ribosomal Protein S10 | Protein Coding | 32 | GC06M042206 | 0.767892361 |
| PHC1 | Polyhomeotic Homolog 1 | Protein Coding | 37 | GC12P008913 | 0.76689291 |
| HSF4 | Heat Shock Transcription Factor 4 | Protein Coding | 38 | GC16P067164 | 0.766122222 |
| RNF167 | Ring Finger Protein 167 | Protein Coding | 31 | GC17P004960 | 0.766100168 |
| PCOLCE2 | Procollagen C-Endopeptidase Enhancer 2 | Protein Coding | 31 | GC03M142815 | 0.765957355 |
| ZNF200 | Zinc Finger Protein 200 | Protein Coding | 31 | GC16M006829 | 0.765957355 |
| ZSWIM2 | Zinc Finger SWIM-Type Containing 2 | Protein Coding | 30 | GC02M186827 | 0.765957355 |
| DQX1 | DEAQ-Box RNA Dependent ATPase 1 | Protein Coding | 28 | GC02M074518 | 0.765957355 |
| OSTN | Osteocrin | Protein Coding | 28 | GC03P191199 | 0.765957355 |
| PRELID2 | PRELI Domain Containing 2 | Protein Coding | 28 | GC05M145471 | 0.765957355 |
| TOMM20L | Translocase Of Outer Mitochondrial Membrane 20 Like | Protein Coding | 28 | GC14P058395 | 0.765957355 |
| PDZD9 | PDZ Domain Containing 9 | Protein Coding | 27 | GC16M021957 | 0.765957355 |
| TMEM233 | Transmembrane Protein 233 | Protein Coding | 26 | GC12P119594 | 0.765957355 |
| TMEM64 | Transmembrane Protein 64 | Protein Coding | 26 | GC08M090623 | 0.765957355 |
| ZNF229 | Zinc Finger Protein 229 | Protein Coding | 26 | GC19M044417 | 0.765957355 |
| CXorf58 | Chromosome X Open Reading Frame 58 | Protein Coding | 23 | GC0XP023907 | 0.765957355 |
| C2orf78 | Chromosome 2 Open Reading Frame 78 | Protein Coding | 21 | GC02P073784 | 0.765957355 |
| MIR578 | MicroRNA 578 | RNA Gene | 13 | GC04P165386 | 0.765957355 |
| JAML | Junction Adhesion Molecule Like | Protein Coding | 28 | GC11M118195 | 0.763865948 |
| TNPO2 | Transportin 2 | Protein Coding | 34 | GC19M012699 | 0.763730466 |
| ZNF589 | Zinc Finger Protein 589 | Protein Coding | 30 | GC03P048241 | 0.763730466 |
| CCDC87 | Coiled-Coil Domain Containing 87 | Protein Coding | 26 | GC11M066590 | 0.763730466 |
| STX4 | Syntaxin 4 | Protein Coding | 37 | GC16P040286 | 0.763393164 |
| ZNF395 | Zinc Finger Protein 395 | Protein Coding | 31 | GC08M028345 | 0.763393164 |
| MRPS33 | Mitochondrial Ribosomal Protein S33 | Protein Coding | 31 | GC07M141029 | 0.763161063 |
| RFNG | RFNG O-Fucosylpeptide 3-Beta-N-Acetylglucosaminyltransferase | Protein Coding | 34 | GC17M082047 | 0.762800753 |

| | | | | | |
|---|---|---|---|---|---|
| COQ10A | Coenzyme Q10A | Protein Coding | 31 | GC12P056266 | 0.762796223 |
| ZNF213 | Zinc Finger Protein 213 | Protein Coding | 31 | GC16P010767 | 0.762796223 |
| SNORA71B | Small Nucleolar RNA, H/ACA Box 71B | RNA Gene | 15 | GC20M038646 | 0.762796223 |
| UPP2 | Uridine Phosphorylase 2 | Protein Coding | 34 | GC02P157876 | 0.762528837 |
| JMJD4 | Jumonji Domain Containing 4 | Protein Coding | 31 | GC01M227730 | 0.762126088 |
| GRINA | Glutamate Ionotropic Receptor NMDA Type Subunit Associated Protein 1 | Protein Coding | 31 | GC08P143990 | 0.761522412 |
| IL17REL | Interleukin 17 Receptor E Like | Protein Coding | 27 | GC22M056241 | 0.759855151 |
| UCN2 | Urocortin 2 | Protein Coding | 31 | GC03M048561 | 0.75965786 |
| SEC16B | SEC16 Homolog B, Endoplasmic Reticulum Export Factor | Protein Coding | 33 | GC01M177923 | 0.759637117 |
| DPH3 | Diphthamide Biosynthesis 3 | Protein Coding | 28 | GC03M016257 | 0.759245753 |
| SMYD5 | SMYD Family Member 5 | Protein Coding | 30 | GC02P073214 | 0.759104013 |
| MROH8 | Maestro Heat Like Repeat Family Member 8 | Protein Coding | 25 | GC20M037101 | 0.757729888 |
| PHOX2A | Paired Like Homeobox 2A | Protein Coding | 38 | GC11M072239 | 0.757406831 |
| MSANTD3 | Myb/SANT DNA Binding Domain Containing 3 | Protein Coding | 27 | GC09P100427 | 0.757184625 |
| SORBS3 | Sorbin And SH3 Domain Containing 3 | Protein Coding | 35 | GC08P022544 | 0.757110476 |
| ABTB1 | Ankyrin Repeat And BTB Domain Containing 1 | Protein Coding | 33 | GC03P127672 | 0.757001102 |
| LINC00994 | Long Intergenic Non-Protein Coding RNA 994 | RNA Gene | 11 | GC03M064078 | 0.756828666 |
| TMEM255B | Transmembrane Protein 255B | Protein Coding | 26 | GC13P113759 | 0.755809605 |
| AAMDC | Adipogenesis Associated Mth938 Domain Containing | Protein Coding | 27 | GC11P077824 | 0.755231738 |
| TIMM23B | Translocase Of Inner Mitochondrial Membrane 23 Homolog B | Protein Coding | 21 | GC10P049942 | 0.755231738 |
| SPINDOC | Spindlin Interactor And Repressor Of Chromatin Binding | Protein Coding | 24 | GC11P063814 | 0.755130947 |
| NECAB3 | N-Terminal EF-Hand Calcium Binding Protein 3 | Protein Coding | 31 | GC20M033659 | 0.754944742 |
| H2BC1 | H2B Clustered Histone 1 | Protein Coding | 26 | GC06P081036 | 0.754889727 |
| ATG2A | Autophagy Related 2A | Protein Coding | 32 | GC11M087225 | 0.75408411 |
| TSPYL6 | TSPY Like 6 | Protein Coding | 30 | GC02M054253 | 0.753426194 |
| TMC5 | Transmembrane Channel Like 5 | Protein Coding | 30 | GC16P019422 | 0.753248692 |
| TOMM7 | Translocase Of Outer Mitochondrial Membrane 7 | Protein Coding | 32 | GC07M022816 | 0.753017545 |
| IGFL1 | IGF Like Family Member 1 | Protein Coding | 26 | GC19P046229 | 0.752756059 |
| MIR668 | MicroRNA 668 | RNA Gene | 10 | GC14P109551 | 0.752756059 |
| IGLC6 | Immunoglobulin Lambda Constant 6 | Pseudogene | 9 | GC22P022919 | 0.752756059 |
| MPP4 | MAGUK P55 Scaffold Protein 4 | Protein Coding | 32 | GC02M201644 | 0.752456069 |
| CXXC1 | CXXC Finger Protein 1 | Protein Coding | 36 | GC18M050282 | 0.752240062 |
| PPP1R3B | Protein Phosphatase 1 Regulatory Subunit 3B | Protein Coding | 34 | GC08M009136 | 0.751139045 |
| SAA2 | Serum Amyloid A2 | Protein Coding | 31 | GC11M018238 | 0.750891984 |
| MIR591 | MicroRNA 591 | RNA Gene | 11 | GC07M096219 | 0.749883056 |
| ZBTB43 | Zinc Finger And BTB Domain Containing 43 | Protein Coding | 34 | GC09P126805 | 0.749531507 |
| DRAP1 | DR1 Associated Protein 1 | Protein Coding | 33 | GC11P069573 | 0.749531507 |
| SIDT2 | SID1 Transmembrane Family Member 2 | Protein Coding | 32 | GC11P117178 | 0.749531507 |
| MRGPRF | MAS Related GPR Family Member F | Protein Coding | 31 | GC11M069004 | 0.749531507 |
| C6orf136 | Chromosome 6 Open Reading Frame 136 | Protein Coding | 26 | GC06P080329 | 0.749531507 |
| SNX25 | Sorting Nexin 25 | Protein Coding | 31 | GC04P185210 | 0.749234915 |
| SPRYD7 | SPRY Domain Containing 7 | Protein Coding | 28 | GC13M049912 | 0.748502731 |
| NKX2-1-AS1 | NKX2-1 Antisense RNA 1 | RNA Gene | 15 | GC14P036519 | 0.748502731 |
| ZNF398 | Zinc Finger Protein 398 | Protein Coding | 32 | GC07P149126 | 0.74792546 |
| SOX2-SRR2 | SRR2 Enhancer Downstream Of SOX2 | Biological Region | 1 | GC03P181716 | 0.747812152 |
| MRPL33 | Mitochondrial Ribosomal Protein L33 | Protein Coding | 29 | GC02P027918 | 0.747599959 |
| SSU72 | SSU72 Homolog, RNA Polymerase II CTD Phosphatase | Protein Coding | 31 | GC01M001541 | 0.747462332 |
| DZIP3 | DAZ Interacting Zinc Finger Protein 3 | Protein Coding | 32 | GC03P108589 | 0.747052491 |
| HSPB7 | Heat Shock Protein Family B (Small) Member 7 | Protein Coding | 34 | GC01M016014 | 0.746525347 |
| KIAA1191 | KIAA1191 | Protein Coding | 30 | GC05M176346 | 0.746046424 |
| ECE2 | Endothelin Converting Enzyme 2 | Protein Coding | 36 | GC03P184276 | 0.74598217 |
| ECM2 | Extracellular Matrix Protein 2 | Protein Coding | 35 | GC09M092493 | 0.745627165 |
| KRT79 | Keratin 79 | Protein Coding | 32 | GC12M052821 | 0.745102465 |
| LOC111188156 | CSF1R Promoter/Intronic Regulatory Region | Biological Region | 2 | GC05P150081 | 0.74480176 |
| PHOSPHO2 | Phosphatase, Orphan 2 | Protein Coding | 32 | GC02P169695 | 0.744670391 |
| RNF208 | Ring Finger Protein 208 | Protein Coding | 28 | GC09M137220 | 0.744660318 |
| TSR3 | TSR3 Ribosome Maturation Factor | Protein Coding | 28 | GC16M006642 | 0.744641662 |
| TMUB2 | Transmembrane And Ubiquitin Like Domain Containing 2 | Protein Coding | 31 | GC17P044186 | 0.744043946 |
| MEIS3 | Meis Homeobox 3 | Protein Coding | 32 | GC19M047403 | 0.74333334 |
| HOMER2 | Homer Scaffold Protein 2 | Protein Coding | 38 | GC15M089127 | 0.743307829 |
| HOMER1 | Homer Scaffold Protein 1 | Protein Coding | 36 | GC05M079372 | 0.743300319 |
| CYB561 | Cytochrome B561 | Protein Coding | 37 | GC17M063432 | 0.743252218 |
| PIGP | Phosphatidylinositol Glycan Anchor Biosynthesis Class P | Protein Coding | 36 | GC21M037059 | 0.743011534 |
| SUCO | SUN Domain Containing Ossification Factor | Protein Coding | 30 | GC01P172532 | 0.742580652 |
| INPP5F | Inositol Polyphosphate-5-Phosphatase F | Protein Coding | 34 | GC10P119704 | 0.742338657 |
| PCIF1 | Phosphorylated CTD Interacting Factor 1 | Protein Coding | 28 | GC20P045934 | 0.742338657 |
| DEFB104A | Defensin Beta 104A | Protein Coding | 28 | GC08P007836 | 0.742039323 |
| DUS1L | Dihydrouridine Synthase 1 Like | Protein Coding | 32 | GC17M082057 | 0.741762161 |
| ZNF385B | Zinc Finger Protein 385B | Protein Coding | 32 | GC02M179441 | 0.741762161 |
| SLC25A34 | Solute Carrier Family 25 Member 34 | Protein Coding | 30 | GC01P015804 | 0.741762161 |
| SNX33 | Sorting Nexin 33 | Protein Coding | 30 | GC15P114826 | 0.741762161 |
| SAMD13 | Sterile Alpha Motif Domain Containing 13 | Protein Coding | 26 | GC01P084298 | 0.741762161 |
| MIR1267 | MicroRNA 1267 | RNA Gene | 12 | GC13M107531 | 0.741359949 |
| LOC110599575 | CYP2B6 Promoter-Proximal Regulatory Region | Biological Region | 2 | GC19P040989 | 0.741359949 |
| IGFL3 | IGF Like Family Member 3 | Protein Coding | 28 | GC19M063976 | 0.741098344 |
| ALKBH2 | AlkB Homolog 2, Alpha-Ketoglutarate Dependent Dioxygenase | Protein Coding | 31 | GC12M109088 | 0.740238488 |
| PDRG1 | P53 And DNA Damage Regulated 1 | Protein Coding | 30 | GC20M031944 | 0.740238488 |
| TIMM8A | Translocase Of Inner Mitochondrial Membrane 8A | Protein Coding | 38 | GC0XM101345 | 0.739982724 |
| TRIM52 | Tripartite Motif Containing 52 | Protein Coding | 28 | GC05M181726 | 0.739866674 |
| GLB1L | Galactosidase Beta 1 Like | Protein Coding | 33 | GC02M219237 | 0.739217281 |
| ATP13A5 | ATPase 13A5 | Protein Coding | 31 | GC03M193274 | 0.739217281 |
| GTF3C6 | General Transcription Factor IIIC Subunit 6 | Protein Coding | 28 | GC06P110958 | 0.739115179 |
| MIR4713 | MicroRNA 4713 | RNA Gene | 12 | GC15P051242 | 0.738960028 |
| PLEKHO1 | Pleckstrin Homology Domain Containing O1 | Protein Coding | 30 | GC01P150150 | 0.738398433 |
| GDPD1 | Glycerophosphodiester Phosphodiesterase Domain Containing 1 | Protein Coding | 33 | GC17P059220 | 0.738059044 |
| IFNA21 | Interferon Alpha 21 | Protein Coding | 35 | GC09M021165 | 0.737809479 |
| PLXNB3 | Plexin B3 | Protein Coding | 35 | GC0XP153764 | 0.737771988 |
| C3orf56 | Chromosome 3 Open Reading Frame 56 | Protein Coding | 22 | GC03P127193 | 0.737771988 |
| RPL10AP9 | Ribosomal Protein L10a Pseudogene 9 | Pseudogene | 6 | GC05M086884 | 0.737771988 |
| CLPS | Colipase | Protein Coding | 36 | GC06M063755 | 0.737398446 |
| STH | Saitohin | Protein Coding | 28 | GC17P045999 | 0.73727417 |
| MIR4786 | MicroRNA 4786 | RNA Gene | 13 | GC02M239943 | 0.73618108 |
| MIR3677 | MicroRNA 3677 | RNA Gene | 12 | GC16P002270 | 0.73618108 |
| S100G | S100 Calcium Binding Protein G | Protein Coding | 31 | GC0XP016668 | 0.735913754 |
| MICU3 | Mitochondrial Calcium Uptake Family Member 3 | Protein Coding | 28 | GC08P017027 | 0.735622168 |

| | | | | | |
|---|---|---|---|---|---|
| VPS26A | VPS26, Retromer Complex Component A | Protein Coding | 35 | GC10P069123 | 0.735551476 |
| MRM2 | Mitochondrial RRNA Methyltransferase 2 | Protein Coding | 31 | GC07M002234 | 0.735442162 |
| VSIG10L | V-Set And Immunoglobulin Domain Containing 10 Like | Protein Coding | 27 | GC19M064235 | 0.735249758 |
| MYH2 | Myosin Heavy Chain 2 | Protein Coding | 42 | GC17M010521 | 0.734351754 |
| ZNF3 | Zinc Finger Protein 3 | Protein Coding | 32 | GC07M100063 | 0.733415008 |
| FBXW9 | F-Box And WD Repeat Domain Containing 9 | Protein Coding | 27 | GC19M012688 | 0.733415008 |
| MINDY4 | MINDY Lysine 48 Deubiquitinase 4 | Protein Coding | 25 | GC07P030771 | 0.733415008 |
| FOXL2NB | FOXL2 Neighbor | Protein Coding | 21 | GC03P138947 | 0.733415008 |
| SLX9 | SLX9 Ribosome Biogenesis Factor | Protein Coding | 23 | GC21P044939 | 0.733324766 |
| NUCB1 | Nucleobindin 1 | Protein Coding | 37 | GC19P048900 | 0.733142257 |
| POLR3F | RNA Polymerase III Subunit F | Protein Coding | 35 | GC20P018492 | 0.7320683 |
| KIR2DL5A | Killer Cell Immunoglobulin Like Receptor, Two Ig Domains And Long Cytoplasmic Tail 5A | Protein Coding | 22 | GC19MR00046 | 0.7320683 |
| FAM156B | Family With Sequence Similarity 156 Member B | Protein Coding | 23 | GC0XP052892 | 0.731755555 |
| POM121 | POM121 Transmembrane Nucleoporin | Protein Coding | 31 | GC07P072879 | 0.731735051 |
| PHB1P1 | PHB1 Pseudogene 1 | Pseudogene | 6 | GC06P150047 | 0.730510771 |
| PVRIG | PVR Related Immunoglobulin Domain Containing | Protein Coding | 30 | GC07P100218 | 0.73026669 |
| ORMDL2 | ORMDL Sphingolipid Biosynthesis Regulator 2 | Protein Coding | 32 | GC12P055867 | 0.730038166 |
| TMEM50B | Transmembrane Protein 50B | Protein Coding | 30 | GC21M033432 | 0.730038166 |
| CDKN2AIPNL | CDKN2A Interacting Protein N-Terminal Like | Protein Coding | 28 | GC05M134402 | 0.730038166 |
| MIR7704 | MicroRNA 7704 | RNA Gene | 10 | GC02P176239 | 0.730038166 |
| LY86-AS1 | LY86 Antisense RNA 1 | RNA Gene | 17 | GC06M006349 | 0.729846179 |
| MIR99AHG | Mir-99a-Let-7c Cluster Host Gene | RNA Gene | 20 | GC21P015928 | 0.729426265 |
| ECSIT | ECSIT Signaling Integrator | Protein Coding | 35 | GC19M011505 | 0.729068041 |
| COMMD6 | COMM Domain Containing 6 | Protein Coding | 29 | GC13M075525 | 0.728598118 |
| PUS3 | Pseudouridine Synthase 3 | Protein Coding | 34 | GC11M125893 | 0.728035629 |
| MLNR | Motilin Receptor | Protein Coding | 36 | GC13P049220 | 0.727912426 |
| MIR4485 | MicroRNA 4485 | RNA Gene | 12 | GC11M011030 | 0.727842093 |
| REM1 | RRAD And GEM Like GTPase 1 | Protein Coding | 30 | GC20P031475 | 0.727655292 |
| NAIF1 | Nuclear Apoptosis Inducing Factor 1 | Protein Coding | 29 | GC09M128061 | 0.727429807 |
| SLC35G6 | Solute Carrier Family 35 Member G6 | Protein Coding | 22 | GC17P010878 | 0.726375818 |
| MIR29B2CHG | MIR29B2 And MIR29C Host Gene | RNA Gene | 14 | GC01M207761 | 0.726375818 |
| OTUD6B-AS1 | OTUD6B Antisense RNA 1 (Head To Head) | RNA Gene | 11 | GC08M091059 | 0.726375818 |
| ALPQTL4 | Alkaline Phosphatase, Plasma Level Of, QTL4 | Genetic Locus | 2 | GC10U901094 | 0.726375818 |
| MIR3121 | MicroRNA 3121 | RNA Gene | 13 | GC01M180438 | 0.726140916 |
| CHCHD4 | Coiled-Coil-Helix-Coiled-Coil-Helix Domain Containing 4 | Protein Coding | 32 | GC03M020207 | 0.725943267 |
| TEDC1 | Tubulin Epsilon And Delta Complex 1 | Protein Coding | 25 | GC14P109311 | 0.725943267 |
| CRISPLD1 | Cysteine Rich Secretory Protein LCCL Domain Containing 1 | Protein Coding | 29 | GC08P074984 | 0.725553215 |
| IGHV3-48 | Immunoglobulin Heavy Variable 3-48 | Protein Coding | 14 | GC14M112480 | 0.725553215 |
| FHIP1A | FHF Complex Subunit HOOK Interacting Protein 1A | Protein Coding | 22 | GC04P151411 | 0.725292921 |
| NGFG | Nerve Growth Factor, Gamma Subunit | Genetic Locus | 3 | GC00U935700 | 0.725292921 |
| WIPI1 | WD Repeat Domain, Phosphoinositide Interacting 1 | Protein Coding | 34 | GC17M068420 | 0.725144148 |
| ITCH-IT1 | ITCH Intronic Transcript 1 | RNA Gene | 9 | GC20P034450 | 0.725005507 |
| TTC7B | Tetratricopeptide Repeat Domain 7B | Protein Coding | 30 | GC14M090524 | 0.724901021 |
| NEK11 | NIMA Related Kinase 11 | Protein Coding | 35 | GC03P131026 | 0.724856615 |
| CHMP6 | Charged Multivesicular Body Protein 6 | Protein Coding | 30 | GC17P080991 | 0.724337995 |
| OR2J3 | Olfactory Receptor Family 2 Subfamily J Member 3 | Protein Coding | 30 | GC06P080275 | 0.724337995 |
| GPATCH2L | G-Patch Domain Containing 2 Like | Protein Coding | 26 | GC14P076151 | 0.724337995 |
| YJEFN3 | YjeF N-Terminal Domain Containing 3 | Protein Coding | 26 | GC19P019528 | 0.724337995 |
| LRRC37BP1 | Leucine Rich Repeat Containing 37B Pseudogene 1 | Pseudogene | 12 | GC17P030629 | 0.724337995 |
| C11orf52 | Chromosome 11 Open Reading Frame 52 | Protein Coding | 25 | GC11P111918 | 0.724298894 |
| TTC31 | Tetratricopeptide Repeat Domain 31 | Protein Coding | 25 | GC02P074483 | 0.724063158 |
| VSX1 | Visual System Homeobox 1 | Protein Coding | 34 | GC20M025070 | 0.723745465 |
| USE1 | Unconventional SNARE In The ER 1 | Protein Coding | 31 | GC19P063098 | 0.723477721 |
| PPFIA3 | PTPRF Interacting Protein Alpha 3 | Protein Coding | 32 | GC19P049119 | 0.723263144 |
| KIF13B | Kinesin Family Member 13B | Protein Coding | 35 | GC08M029067 | 0.722748041 |
| EBPL | EBP Like | Protein Coding | 30 | GC13M049660 | 0.721950769 |
| UPK3B | Uroplakin 3B | Protein Coding | 28 | GC07P076510 | 0.720900774 |
| FBXO47 | F-Box Protein 47 | Protein Coding | 27 | GC17M038936 | 0.720836401 |
| MIR507 | MicroRNA 507 | RNA Gene | 13 | GC0XM147246 | 0.720639706 |
| ADRA2B | Adrenoceptor Alpha 2B | Protein Coding | 40 | GC02M096112 | 0.720519066 |
| SENP6 | SUMO Specific Peptidase 6 | Protein Coding | 34 | GC06P075601 | 0.720178664 |
| MIR890 | MicroRNA 890 | RNA Gene | 11 | GC0XM145994 | 0.719998002 |
| TJAP1 | Tight Junction Associated Protein 1 | Protein Coding | 31 | GC06P043477 | 0.719670951 |
| NDUFB6 | NADH:Ubiquinone Oxidoreductase Subunit B6 | Protein Coding | 35 | GC09M032553 | 0.718559444 |
| TMEM9 | Transmembrane Protein 9 | Protein Coding | 34 | GC01M201134 | 0.718483329 |
| ABLIM3 | Actin Binding LIM Protein Family Member 3 | Protein Coding | 32 | GC05P149141 | 0.717668295 |
| PDLIM3 | PDZ And LIM Domain 3 | Protein Coding | 34 | GC04M185500 | 0.716963887 |
| STOML3 | Stomatin Like 3 | Protein Coding | 30 | GC13M038965 | 0.716792464 |
| TRIM45 | Tripartite Motif Containing 45 | Protein Coding | 30 | GC01M117111 | 0.716531277 |
| LNPK | Lunapark, ER Junction Formation Factor | Protein Coding | 26 | GC02M175924 | 0.716173172 |
| PDZD11 | PDZ Domain Containing 11 | Protein Coding | 31 | GC0XM070286 | 0.715951741 |
| ZNF16 | Zinc Finger Protein 16 | Protein Coding | 31 | GC08M144930 | 0.715951741 |
| RBFA | Ribosome Binding Factor A | Protein Coding | 27 | GC18P080065 | 0.715951741 |
| SULT1A4 | Sulfotransferase Family 1A Member 4 | Protein Coding | 27 | GC16P040161 | 0.715951741 |
| TMEM30A-DT | TMEM30A Divergent Transcript | RNA Gene | 11 | GC06P080928 | 0.715951741 |
| ATP1B3 | ATPase Na+/K+ Transporting Subunit Beta 3 | Protein Coding | 38 | GC03P141876 | 0.715808868 |
| MIR4787 | MicroRNA 4787 | RNA Gene | 14 | GC03P050676 | 0.714744806 |
| MIR3177 | MicroRNA 3177 | RNA Gene | 13 | GC16P001734 | 0.714744806 |
| MIR4690 | MicroRNA 4690 | RNA Gene | 13 | GC11P065636 | 0.714744806 |
| MIR4761 | MicroRNA 4761 | RNA Gene | 13 | GC22P034419 | 0.714744806 |
| MIR4670 | MicroRNA 4670 | RNA Gene | 12 | GC09M092527 | 0.714744806 |
| MIR4796 | MicroRNA 4796 | RNA Gene | 12 | GC03M114743 | 0.714744806 |
| MIR4639 | MicroRNA 4639 | RNA Gene | 11 | GC06P016438 | 0.714744806 |
| MIR4649 | MicroRNA 4649 | RNA Gene | 11 | GC07P044112 | 0.714744806 |
| MIR4655 | MicroRNA 4655 | RNA Gene | 11 | GC07M001844 | 0.714744806 |
| MIR4800 | MicroRNA 4800 | RNA Gene | 11 | GC04M002408 | 0.714744806 |
| AK6 | Adenylate Kinase 6 | Protein Coding | 25 | GC05M069350 | 0.71435225 |
| SLC14A2 | Solute Carrier Family 14 Member 2 | Protein Coding | 38 | GC18P045167 | 0.714297831 |
| CD300A | CD300a Molecule | Protein Coding | 36 | GC17P074466 | 0.714297831 |
| GPBP1 | GC-Rich Promoter Binding Protein 1 | Protein Coding | 31 | GC05P057173 | 0.714297831 |
| RBMS3-AS3 | RBMS3 Antisense RNA 3 | RNA Gene | 13 | GC03M029038 | 0.714297831 |
| TAT-AS1 | TAT Antisense RNA 1 | RNA Gene | 13 | GC16P071565 | 0.714297831 |
| LINC01977 | Long Intergenic Non-Protein Coding RNA 1977 | RNA Gene | 10 | GC17P079826 | 0.714297831 |
| STAG3L5P | Stromal Antigen 3-Like 5 Pseudogene | Pseudogene | 9 | GC07P100960 | 0.714297831 |

| | | | | | |
|---|---|---|---|---|---|
| SGTB | Small Glutamine Rich Tetratricopeptide Repeat Co-Chaperone Beta | Protein Coding | 30 | GC05M065665 | 0.713984787 |
| RXYLT1 | Ribitol Xylosyltransferase 1 | Protein Coding | 29 | GC12P063779 | 0.713810146 |
| SREK1IP1 | SREK1 Interacting Protein 1 | Protein Coding | 28 | GC05M064718 | 0.713497758 |
| DIP2A | Disco Interacting Protein 2 Homolog A | Protein Coding | 35 | GC21P046458 | 0.713106453 |
| NXT2 | Nuclear Transport Factor 2 Like Export Factor 2 | Protein Coding | 30 | GC0XP109535 | 0.713050961 |
| ABR | ABR Activator Of RhoGEF And GTPase | Protein Coding | 36 | GC17M001003 | 0.712976575 |
| SRGAP2B | SLIT-ROBO Rho GTPase Activating Protein 2B | Protein Coding | 18 | GC01M144887 | 0.712926507 |
| HSPA12B | Heat Shock Protein Family A (Hsp70) Member 12B | Protein Coding | 31 | GC20P003997 | 0.712575734 |
| ASNSD1 | Asparagine Synthetase Domain Containing 1 | Protein Coding | 29 | GC02P189661 | 0.712575734 |
| NETO2 | Neuropilin And Tolloid Like 2 | Protein Coding | 34 | GC16M047077 | 0.712275624 |
| LOC108254682 | PAX5 Promoter Region | Biological Region | 2 | GC09P037034 | 0.712258458 |
| MRPL27 | Mitochondrial Ribosomal Protein L27 | Protein Coding | 30 | GC17M050367 | 0.711970925 |
| ASB16-AS1 | ASB16 Antisense RNA 1 | RNA Gene | 19 | GC17M044175 | 0.711970925 |
| RAB17 | RAB17, Member RAS Oncogene Family | Protein Coding | 34 | GC02M237574 | 0.711227477 |
| TRAFD1 | TRAF-Type Zinc Finger Domain Containing 1 | Protein Coding | 31 | GC12P112125 | 0.711129189 |
| PPM1A | Protein Phosphatase, Mg2+/Mn2+ Dependent 1A | Protein Coding | 39 | GC14P060245 | 0.709626079 |
| GPR151 | G Protein-Coupled Receptor 151 | Protein Coding | 34 | GC05M146513 | 0.708601892 |
| ABCA9 | ATP Binding Cassette Subfamily A Member 9 | Protein Coding | 33 | GC17M068974 | 0.708601892 |
| B4GALNT4 | Beta-1,4-N-Acetyl-Galactosaminyltransferase 4 | Protein Coding | 29 | GC11P000369 | 0.708601892 |
| PABPC5 | Poly(A) Binding Protein Cytoplasmic 5 | Protein Coding | 28 | GC0XP091434 | 0.708601892 |
| BTBD1 | BTB Domain Containing 1 | Protein Coding | 34 | GC15M089144 | 0.708435655 |
| POM121C | POM121 Transmembrane Nucleoporin C | Protein Coding | 26 | GC07M075416 | 0.707681537 |
| IFNA5 | Interferon Alpha 5 | Protein Coding | 34 | GC09M021304 | 0.706874728 |
| SNRNP48 | Small Nuclear Ribonucleoprotein U11/U12 Subunit 48 | Protein Coding | 30 | GC06P007590 | 0.706874728 |
| OSTM1 | Osteoclastogenesis Associated Transmembrane Protein 1 | Protein Coding | 36 | GC06M108041 | 0.706634939 |
| TADA2A | Transcriptional Adaptor 2A | Protein Coding | 33 | GC17P037406 | 0.705551147 |
| ENPP5 | Ectonucleotide Pyrophosphatase/Phosphodiesterase Family Member 5 | Protein Coding | 34 | GC06M046159 | 0.705069065 |
| VPS37B | VPS37B Subunit Of ESCRT-I | Protein Coding | 30 | GC12M122865 | 0.705069065 |
| PPIAP10 | Peptidylprolyl Isomerase A Pseudogene 10 | Pseudogene | 6 | GC20P053421 | 0.704719484 |
| SLC17A3 | Solute Carrier Family 17 Member 3 | Protein Coding | 36 | GC06M025833 | 0.704495609 |
| CEMP1 | Cementum Protein 1 | Protein Coding | 25 | GC16M006735 | 0.704413414 |
| LINC01152 | Long Intergenic Non-Protein Coding RNA 1152 | RNA Gene | 14 | GC17P072030 | 0.703940451 |
| KIF4B | Kinesin Family Member 4B | Protein Coding | 30 | GC05P155013 | 0.702901721 |
| NYAP2 | Neuronal Tyrosine-Phosphorylated Phosphoinositide-3-Kinase Adaptor 2 | Protein Coding | 28 | GC02P225400 | 0.702901721 |
| C22orf31 | Chromosome 22 Open Reading Frame 31 | Protein Coding | 26 | GC22M029058 | 0.702901721 |
| CIPC | CLOCK Interacting Pacemaker | Protein Coding | 26 | GC14P077099 | 0.702901721 |
| SEC1P | Secretory Blood Group 1, Pseudogene | Pseudogene | 12 | GC19P048638 | 0.702901721 |
| TMEM92-AS1 | TMEM92 Antisense RNA 1 | RNA Gene | 12 | GC17M050281 | 0.702901721 |
| MIR2277 | MicroRNA 2277 | RNA Gene | 15 | GC05M093620 | 0.702738583 |
| DCAF4L1 | DDB1 And CUL4 Associated Factor 4 Like 1 | Protein Coding | 26 | GC04P041983 | 0.702129483 |
| IGKV2D-40 | Immunoglobulin Kappa Variable 2D-40 | Protein Coding | 11 | GC02P089853 | 0.702129483 |
| CSGALNACT2 | Chondroitin Sulfate N-Acetylgalactosaminyltransferase 2 | Protein Coding | 35 | GC10P043138 | 0.701179624 |
| SGMS1-AS1 | SGMS1 Antisense RNA 1 | RNA Gene | 12 | GC10P050624 | 0.701179624 |
| SDHAP3 | SDHA Pseudogene 3 | Pseudogene | 12 | GC05M001573 | 0.700639248 |
| H2BC15 | H2B Clustered Histone 15 | Protein Coding | 26 | GC06P079948 | 0.69988811 |
| SEPTIN10 | Septin 10 | Protein Coding | 27 | GC02M109543 | 0.699515164 |
| KLHL24 | Kelch Like Family Member 24 | Protein Coding | 34 | GC03P183635 | 0.698134184 |
| CHPF2 | Chondroitin Polymerizing Factor 2 | Protein Coding | 32 | GC07P151232 | 0.697771966 |
| MRPL21 | Mitochondrial Ribosomal Protein L21 | Protein Coding | 31 | GC11M087445 | 0.697771966 |
| LINC00632 | Long Intergenic Non-Protein Coding RNA 632 | RNA Gene | 15 | GC0XP140709 | 0.697589993 |
| STATH | Statherin | Protein Coding | 28 | GC04P069995 | 0.696944416 |
| PCDHA6 | Protocadherin Alpha 6 | Protein Coding | 32 | GC05P145804 | 0.696834564 |
| FBXO34 | F-Box Protein 34 | Protein Coding | 28 | GC14P055271 | 0.696834564 |
| OR5AC2 | Olfactory Receptor Family 5 Subfamily AC Member 2 | Protein Coding | 28 | GC03P098087 | 0.696834564 |
| EFCAB11 | EF-Hand Calcium Binding Domain 11 | Protein Coding | 27 | GC14M100524 | 0.696834564 |
| CYSRT1 | Cysteine Rich Tail 1 | Protein Coding | 23 | GC09P137224 | 0.696834564 |
| TRIM64C | Tripartite Motif Containing 64C | Protein Coding | 20 | GC11M086911 | 0.696834564 |
| PIPSL | PIP5K1A And PSMD4 Like (Pseudogene) | Pseudogene | 15 | GC10M093958 | 0.696834564 |
| MIR580 | MicroRNA 580 | RNA Gene | 12 | GC05M036184 | 0.696834564 |
| HCCS | Holocytochrome C Synthase | Protein Coding | 38 | GC0XP011111 | 0.696767211 |
| MRGPRX3 | MAS Related GPR Family Member X3 | Protein Coding | 32 | GC11P018141 | 0.695800066 |
| MTUS2 | Microtubule Associated Scaffold Protein 2 | Protein Coding | 31 | GC13P028820 | 0.69577843 |
| ZC3H8 | Zinc Finger CCCH-Type Containing 8 | Protein Coding | 30 | GC02M112211 | 0.695776701 |
| ADGRG2 | Adhesion G Protein-Coupled Receptor G2 | Protein Coding | 31 | GC0XM018989 | 0.695470333 |
| BHLHE40-AS1 | BHLHE40 Antisense RNA 1 | RNA Gene | 13 | GC03M004897 | 0.695083976 |
| MIR6855 | MicroRNA 6855 | RNA Gene | 9 | GC09P129869 | 0.695083976 |
| SEC24A | SEC24 Homolog A, COPII Coat Complex Component | Protein Coding | 34 | GC05P134647 | 0.694431245 |
| TEDC2 | Tubulin Epsilon And Delta Complex 2 | Protein Coding | 23 | GC16P010733 | 0.694393456 |
| LHFPL6 | LHFPL Tetraspan Subfamily Member 6 | Protein Coding | 26 | GC13M039209 | 0.694261432 |
| MIR2278 | MicroRNA 2278 | RNA Gene | 15 | GC09P094809 | 0.693661571 |
| MIR2276 | MicroRNA 2276 | RNA Gene | 12 | GC13P024162 | 0.693661571 |
| EIF4EBP3 | Eukaryotic Translation Initiation Factor 4E Binding Protein 3 | Protein Coding | 31 | GC05P145797 | 0.693082631 |
| PLPBP | Pyridoxal Phosphate Binding Protein | Protein Coding | 32 | GC08P037763 | 0.692801952 |
| MRPL43 | Mitochondrial Ribosomal Protein L43 | Protein Coding | 33 | GC10M100969 | 0.692645431 |
| TRIAP1 | TP53 Regulated Inhibitor Of Apoptosis 1 | Protein Coding | 31 | GC12M120443 | 0.691839457 |
| NRIP3 | Nuclear Receptor Interacting Protein 3 | Protein Coding | 32 | GC11M009001 | 0.69060427 |
| ZNF449 | Zinc Finger Protein 449 | Protein Coding | 28 | GC0XP135344 | 0.690002561 |
| ANGEL1 | Angel Homolog 1 | Protein Coding | 31 | GC14M077018 | 0.689383864 |
| FAM117A | Family With Sequence Similarity 117 Member A | Protein Coding | 26 | GC17M049710 | 0.689383864 |
| TEPSIN | TEPSIN Adaptor Related Protein Complex 4 Accessory Protein | Protein Coding | 23 | GC17M081229 | 0.689383864 |
| RPS17P11 | Ribosomal Protein S17 Pseudogene 11 | Pseudogene | 6 | GC05P052282 | 0.689383864 |
| BIVM | Basic, Immunoglobulin-Like Variable Motif Containing | Protein Coding | 30 | GC13P102799 | 0.689261258 |
| ZSCAN12 | Zinc Finger And SCAN Domain Containing 12 | Protein Coding | 28 | GC06M063352 | 0.688908756 |
| GRIN3B | Glutamate Ionotropic Receptor NMDA Type Subunit 3B | Protein Coding | 37 | GC19P001000 | 0.688381851 |
| UTP14C | UTP14C Small Subunit Processome Component | Protein Coding | 28 | GC13P052024 | 0.687671661 |
| ZNF396 | Zinc Finger Protein 396 | Protein Coding | 28 | GC18M035366 | 0.687661707 |
| GIMAP3P | GTPase, IMAP Family Member 3 Pseudogene | Pseudogene | 10 | GC07M150746 | 0.687661707 |
| ZSWIM8 | Zinc Finger SWIM-Type Containing 8 | Protein Coding | 28 | GC10P073785 | 0.68762064 |
| MSC-AS1 | MSC Antisense RNA 1 | RNA Gene | 14 | GC08P071828 | 0.687561512 |
| PAN3 | Poly(A) Specific Ribonuclease Subunit PAN3 | Protein Coding | 31 | GC13P028136 | 0.687148273 |
| DSCR9 | Down Syndrome Critical Region 9 | RNA Gene | 21 | GC21P037208 | 0.687145472 |
| HACL1 | 2-Hydroxyacyl-CoA Lyase 1 | Protein Coding | 35 | GC03M020250 | 0.68703723 |
| GADL1 | Glutamate Decarboxylase Like 1 | Protein Coding | 32 | GC03M030743 | 0.6868788 |
| BSX | Brain Specific Homeobox | Protein Coding | 31 | GC11M122977 | 0.686303139 |
| PRELP | Proline And Arginine Rich End Leucine Rich Repeat Protein | Protein Coding | 38 | GC01P203475 | 0.685643315 |

| | | | | | |
|---|---|---|---|---|---|
| DUSP11 | Dual Specificity Phosphatase 11 | Protein Coding | 31 | GC02M073762 | 0.685438454 |
| FAM110A | Family With Sequence Similarity 110 Member A | Protein Coding | 28 | GC20P000833 | 0.685438454 |
| SMIM4 | Small Integral Membrane Protein 4 | Protein Coding | 23 | GC03P052534 | 0.685438454 |
| DPY19L2P1 | DPY19L2 Pseudogene 1 | Pseudogene | 16 | GC07M035080 | 0.685438454 |
| ELOVL2-AS1 | ELOVL2 Antisense RNA 1 | RNA Gene | 14 | GC06P011074 | 0.685438454 |
| CCDC28A-AS1 | CCDC28A Antisense RNA 1 | RNA Gene | 13 | GC06M138730 | 0.685438454 |
| RAB20 | RAB20, Member RAS Oncogene Family | Protein Coding | 31 | GC13M110523 | 0.684862971 |
| GAPDHS | Glyceraldehyde-3-Phosphate Dehydrogenase, Spermatogenic | Protein Coding | 38 | GC19P063451 | 0.684518099 |
| ADGRA2 | Adhesion G Protein-Coupled Receptor A2 | Protein Coding | 32 | GC08P037785 | 0.684518099 |
| CRYL1 | Crystallin Lambda 1 | Protein Coding | 32 | GC13M020403 | 0.683802068 |
| ABHD2 | Abhydrolase Domain Containing 2, Acylglycerol Lipase | Protein Coding | 35 | GC15P089087 | 0.682855546 |
| ABRAXAS2 | Abraxas 2, BRISC Complex Subunit | Protein Coding | 28 | GC10P124802 | 0.682855546 |
| STX1B | Syntaxin 1B | Protein Coding | 36 | GC16M030989 | 0.682806134 |
| PDE2A | Phosphodiesterase 2A | Protein Coding | 43 | GC11M072576 | 0.682743728 |
| CCNQP1 | CCNQ Pseudogene 1 | Pseudogene | 9 | GC01P200219 | 0.682723105 |
| HEBP2 | Heme Binding Protein 2 | Protein Coding | 32 | GC06P138403 | 0.682325721 |
| CCDC122 | Coiled-Coil Domain Containing 122 | Protein Coding | 28 | GC13M043823 | 0.682119846 |
| OVCH2 | Ovochymase 2 | Protein Coding | 26 | GC11M007671 | 0.681961358 |
| VASN | Vasorin | Protein Coding | 33 | GC16P010807 | 0.68177098 |
| ZFAND5 | Zinc Finger AN1-Type Containing 5 | Protein Coding | 34 | GC09M072351 | 0.681655407 |
| HES3 | Hes Family BHLH Transcription Factor 3 | Protein Coding | 26 | GC01P006244 | 0.68158263 |
| RASSF7 | Ras Association Domain Family Member 7 | Protein Coding | 30 | GC11P000560 | 0.681332946 |
| KLC3 | Kinesin Light Chain 3 | Protein Coding | 35 | GC19P045333 | 0.680986941 |
| LHX2 | LIM Homeobox 2 | Protein Coding | 35 | GC09P124001 | 0.680902243 |
| AP4S1 | Adaptor Related Protein Complex 4 Subunit Sigma 1 | Protein Coding | 34 | GC14P031025 | 0.680353522 |
| KCNJ12 | Potassium Inwardly Rectifying Channel Subfamily J Member 12 | Protein Coding | 38 | GC17P052377 | 0.679935277 |
| EFHC2 | EF-Hand Domain Containing 2 | Protein Coding | 31 | GC0XM044146 | 0.679935277 |
| LIN52 | Lin-52 DREAM MuvB Core Complex Component | Protein Coding | 29 | GC14P074084 | 0.679785907 |
| TIMMDC1 | Translocase Of Inner Mitochondrial Membrane Domain Containing 1 | Protein Coding | 33 | GC03P119498 | 0.678207934 |
| OMD | Osteomodulin | Protein Coding | 36 | GC09M092750 | 0.677650034 |
| CA3 | Carbonic Anhydrase 3 | Protein Coding | 37 | GC08P085373 | 0.677464724 |
| NRAP | Nebulin Related Anchoring Protein | Protein Coding | 32 | GC10M113588 | 0.677126467 |
| ATXN7L1 | Ataxin 7 Like 1 | Protein Coding | 28 | GC07M105604 | 0.676923633 |
| CTSW | Cathepsin W | Protein Coding | 34 | GC11P065879 | 0.676721513 |
| ZNF181 | Zinc Finger Protein 181 | Protein Coding | 29 | GC19P034734 | 0.676170945 |
| PARP10 | Poly(ADP-Ribose) Polymerase Family Member 10 | Protein Coding | 34 | GC08M143977 | 0.675224185 |
| SNORD74 | Small Nucleolar RNA, C/D Box 74 | RNA Gene | 13 | GC01M174520 | 0.675101995 |
| SNORD80 | Small Nucleolar RNA, C/D Box 80 | RNA Gene | 13 | GC01M174518 | 0.675101995 |
| LNX2 | Ligand Of Numb-Protein X 2 | Protein Coding | 33 | GC13M027545 | 0.67459482 |
| GLB1L2 | Galactosidase Beta 1 Like 2 | Protein Coding | 31 | GC11P134331 | 0.674045086 |
| GRIK5 | Glutamate Ionotropic Receptor Kainate Type Subunit 5 | Protein Coding | 39 | GC19M041998 | 0.673963606 |
| PHACTR2 | Phosphatase And Actin Regulator 2 | Protein Coding | 31 | GC06P143536 | 0.673727036 |
| BPNT2 | 3'(2'), 5'-Bisphosphate Nucleotidase 2 | Protein Coding | 32 | GC08M056958 | 0.673675716 |
| COLEC12 | Collectin Subfamily Member 12 | Protein Coding | 36 | GC18M000318 | 0.673021436 |
| MTMR9 | Myotubularin Related Protein 9 | Protein Coding | 35 | GC08P011284 | 0.672806501 |
| AP1G2 | Adaptor Related Protein Complex 1 Subunit Gamma 2 | Protein Coding | 32 | GC14M023559 | 0.672560334 |
| PRPSAP2 | Phosphoribosyl Pyrophosphate Synthetase Associated Protein 2 | Protein Coding | 31 | GC17P052268 | 0.672116578 |
| FFAR3 | Free Fatty Acid Receptor 3 | Protein Coding | 34 | GC19P064594 | 0.671920538 |
| ATP5MC3 | ATP Synthase Membrane Subunit C Locus 3 | Protein Coding | 30 | GC02M175180 | 0.671920538 |
| ZFP62 | ZFP62 Zinc Finger Protein | Protein Coding | 28 | GC05M181663 | 0.671920538 |
| MROH1 | Maestro Heat Like Repeat Family Member 1 | Protein Coding | 27 | GC08P144300 | 0.671920538 |
| C6orf132 | Chromosome 6 Open Reading Frame 132 | Protein Coding | 24 | GC06M042101 | 0.671920538 |
| SLC66A3 | Solute Carrier Family 66 Member 3 | Protein Coding | 24 | GC02P011156 | 0.671920538 |
| LINC00299 | Long Intergenic Non-Protein Coding RNA 299 | RNA Gene | 20 | GC02M007988 | 0.671920538 |
| MIR1276 | MicroRNA 1276 | RNA Gene | 16 | GC15M089540 | 0.671920538 |
| IGHV1-46 | Immunoglobulin Heavy Variable 1-46 | Protein Coding | 13 | GC14M112476 | 0.671920538 |
| IGHV3-33 | Immunoglobulin Heavy Variable 3-33 | Protein Coding | 13 | GC14M112544 | 0.671920538 |
| MIR1255A | MicroRNA 1255a | RNA Gene | 13 | GC04M101330 | 0.671920538 |
| MIR1471 | MicroRNA 1471 | RNA Gene | 11 | GC02M231892 | 0.671920538 |
| IGKV1-12 | Immunoglobulin Kappa Variable 1-12 | Protein Coding | 10 | GC02M089040 | 0.671920538 |
| LINC02989 | Long Intergenic Non-Protein Coding RNA 2989 | Uncategorized | 2 | GC00U936997 | 0.671920538 |
| ECHDC3 | Enoyl-CoA Hydratase Domain Containing 3 | Protein Coding | 30 | GC10P011742 | 0.671766996 |
| DPH2 | Diphthamide Biosynthesis 2 | Protein Coding | 34 | GC01P043969 | 0.671338201 |
| SERINC3 | Serine Incorporator 3 | Protein Coding | 35 | GC20M044496 | 0.670229495 |
| ZNF483 | Zinc Finger Protein 483 | Protein Coding | 31 | GC09P111525 | 0.670167685 |
| SLFN12L | Schlafen Family Member 12 Like | Protein Coding | 30 | GC17M035464 | 0.670044839 |
| MFSD4B | Major Facilitator Superfamily Domain Containing 4B | Protein Coding | 28 | GC06P111426 | 0.670044839 |
| RRN3P2 | RRN3 Pseudogene 2 | Pseudogene | 15 | GC16P029074 | 0.670044839 |
| TEX53 | Testis Expressed 53 | Protein Coding | 11 | GC09M115229 | 0.670044839 |
| LOC441666 | Zinc Finger Protein 91 Pseudogene | Pseudogene | 8 | GC10M042331 | 0.670044839 |
| RTN2 | Reticulon 2 | Protein Coding | 38 | GC19M045485 | 0.66942215 |
| PLPPR3 | Phospholipid Phosphatase Related 3 | Protein Coding | 27 | GC19M004817 | 0.669050813 |
| LOC111255645 | TNFRSF10A 5' Regulatory Region | Biological Region | 2 | GC08P023229 | 0.66893512 |
| DZIP1 | DAZ Interacting Zinc Finger Protein 1 | Protein Coding | 34 | GC13M095578 | 0.668750167 |
| EBP | EBP Cholestenol Delta-Isomerase | Protein Coding | 39 | GC0XP048521 | 0.668181479 |
| RUBCN | Rubicon Autophagy Regulator | Protein Coding | 32 | GC03M198019 | 0.667725086 |
| CASS4 | Cas Scaffold Protein Family Member 4 | Protein Coding | 30 | GC20P056411 | 0.666713834 |
| MIR3200 | MicroRNA 3200 | RNA Gene | 15 | GC22P030731 | 0.666443646 |
| MBOAT4 | Membrane Bound O-Acyltransferase Domain Containing 4 | Protein Coding | 29 | GC08M030131 | 0.666403234 |
| FSIP2 | Fibrous Sheath Interacting Protein 2 | Protein Coding | 29 | GC02P185738 | 0.666232944 |
| ADM2 | Adrenomedullin 2 | Protein Coding | 31 | GC22P050481 | 0.665725172 |
| FAR2 | Fatty Acyl-CoA Reductase 2 | Protein Coding | 35 | GC12P029145 | 0.665614188 |
| P2RX5 | Purinergic Receptor P2X 5 | Protein Coding | 38 | GC17M003672 | 0.664859593 |
| MIR1261 | MicroRNA 1261 | RNA Gene | 10 | GC11M090869 | 0.663470924 |
| NALF1 | NALCN Channel Auxiliary Factor 1 | Protein Coding | 24 | GC13M107164 | 0.663426697 |
| ZNF236 | Zinc Finger Protein 236 | Protein Coding | 29 | GC18P076822 | 0.663401723 |
| CCDC73 | Coiled-Coil Domain Containing 73 | Protein Coding | 28 | GC11M032603 | 0.662614167 |
| FHDC1 | FH2 Domain Containing 1 | Protein Coding | 28 | GC04P152935 | 0.66253829 |
| TMIGD1 | Transmembrane And Immunoglobulin Domain Containing 1 | Protein Coding | 28 | GC17M030316 | 0.66253829 |
| ZNF699 | Zinc Finger Protein 699 | Protein Coding | 34 | GC19M009294 | 0.662060022 |
| SYPL2 | Synaptophysin Like 2 | Protein Coding | 32 | GC01P109466 | 0.661152482 |
| LGSN | Lengsin, Lens Protein With Glutamine Synthetase Domain | Protein Coding | 30 | GC06M063275 | 0.660137057 |
| MIR4779 | MicroRNA 4779 | RNA Gene | 10 | GC02M086193 | 0.659806848 |
| HCAR2 | Hydroxycarboxylic Acid Receptor 2 | Protein Coding | 35 | GC12M122701 | 0.659288168 |
| PLA2G15 | Phospholipase A2 Group XV | Protein Coding | 34 | GC16P068245 | 0.659016371 |

| | | | | | |
|---|---|---|---|---|---|
| TRAM2 | Translocation Associated Membrane Protein 2 | Protein Coding | 32 | GC06M063974 | 0.658974111 |
| MCUR1 | Mitochondrial Calcium Uniporter Regulator 1 | Protein Coding | 31 | GC06M013788 | 0.658974111 |
| MID1IP1 | MID1 Interacting Protein 1 | Protein Coding | 35 | GC0XP038802 | 0.65801686 |
| MEGF9 | Multiple EGF Like Domains 9 | Protein Coding | 32 | GC09M120600 | 0.656213999 |
| AASDHPPT | Aminoadipate-Semialdehyde Dehydrogenase-Phosphopantetheinyl Transferase | Protein Coding | 34 | GC11P106075 | 0.656174421 |
| DIRAS1 | DIRAS Family GTPase 1 | Protein Coding | 32 | GC19M002714 | 0.656174421 |
| SERPINA11 | Serpin Family A Member 11 | Protein Coding | 30 | GC14M094442 | 0.65559727 |
| RMND5A | Required For Meiotic Nuclear Division 5 Homolog A | Protein Coding | 33 | GC02P086721 | 0.655207694 |
| RRP36 | Ribosomal RNA Processing 36 | Protein Coding | 28 | GC06P043021 | 0.655144393 |
| PGLYRP1 | Peptidoglycan Recognition Protein 1 | Protein Coding | 37 | GC19M063972 | 0.654761493 |
| CHST1 | Carbohydrate Sulfotransferase 1 | Protein Coding | 36 | GC11M045647 | 0.65430367 |
| GALNT7-DT | GALNT7 Divergent Transcript | RNA Gene | 11 | GC04M173093 | 0.65430367 |
| TRIM39-RPP21 | TRIM39-RPP21 Readthrough | Protein Coding | 17 | GC06P080323 | 0.6541273 |
| COQ3 | Coenzyme Q3, Methyltransferase | Protein Coding | 35 | GC06M099369 | 0.654059052 |
| ABLIM2 | Actin Binding LIM Protein Family Member 2 | Protein Coding | 30 | GC04M007967 | 0.653261185 |
| SPRR2B | Small Proline Rich Protein 2B | Protein Coding | 25 | GC01M153070 | 0.652438462 |
| PIP4K2C | Phosphatidylinositol-5-Phosphate 4-Kinase Type 2 Gamma | Protein Coding | 34 | GC12P057591 | 0.651925921 |
| CPA5 | Carboxypeptidase A5 | Protein Coding | 35 | GC07P130344 | 0.651719213 |
| ABHD17B | Abhydrolase Domain Containing 17B, Depalmitoylase | Protein Coding | 27 | GC09M071862 | 0.65114212 |
| IQCC | IQ Motif Containing C | Protein Coding | 27 | GC01P032205 | 0.65114212 |
| CCDC125 | Coiled-Coil Domain Containing 125 | Protein Coding | 26 | GC05M069273 | 0.65114212 |
| TMEM128 | Transmembrane Protein 128 | Protein Coding | 27 | GC04M004285 | 0.651112318 |
| RBIS | Ribosomal Biogenesis Factor | Protein Coding | 25 | GC08M085215 | 0.650660634 |
| TULP4 | TUB Like Protein 4 | Protein Coding | 30 | GC06P158232 | 0.650064647 |
| LOC119266102 | IFNGR2 Promoter Region | Biological Region | 2 | GC21P033403 | 0.649961293 |
| RWDD1 | RWD Domain Containing 1 | Protein Coding | 30 | GC06P116571 | 0.64977473 |
| PLBD2 | Phospholipase B Domain Containing 2 | Protein Coding | 30 | GC12P113358 | 0.648260653 |
| PILRA | Paired Immunoglobin Like Type 2 Receptor Alpha | Protein Coding | 32 | GC07P100367 | 0.64799732 |
| ZSCAN25 | Zinc Finger And SCAN Domain Containing 25 | Protein Coding | 31 | GC07P099697 | 0.64799732 |
| TEX41 | Testis Expressed 41 | RNA Gene | 16 | GC02P144667 | 0.64799732 |
| PEAR1 | Platelet Endothelial Aggregation Receptor 1 | Protein Coding | 34 | GC01P156893 | 0.647854924 |
| ENOPH1 | Enolase-Phosphatase 1 | Protein Coding | 34 | GC04P082430 | 0.647366881 |
| HSP90AB4P | Heat Shock Protein 90 Alpha Family Class B Member 4, Pseudogene | Pseudogene | 13 | GC15M058690 | 0.647189379 |
| AQP7P3 | Aquaporin 7 Pseudogene 3 | Pseudogene | 13 | GC09P065877 | 0.647007167 |
| RNF122 | Ring Finger Protein 122 | Protein Coding | 30 | GC08M033524 | 0.646793008 |
| CDC37L1 | Cell Division Cycle 37 Like 1 | Protein Coding | 30 | GC09P004671 | 0.646696687 |
| FBXO21 | F-Box Protein 21 | Protein Coding | 31 | GC12M117141 | 0.646484554 |
| RAB43 | RAB43, Member RAS Oncogene Family | Protein Coding | 30 | GC03M130183 | 0.646484554 |
| PROX1-AS1 | PROX1 Antisense RNA 1 | RNA Gene | 15 | GC01M213817 | 0.646377861 |
| FAM110C | Family With Sequence Similarity 110 Member C | Protein Coding | 27 | GC02M000039 | 0.646312654 |
| PMM1 | Phosphomannomutase 1 | Protein Coding | 35 | GC22M041576 | 0.645557106 |
| TAF1A | TATA-Box Binding Protein Associated Factor, RNA Polymerase I Subunit A | Protein Coding | 33 | GC01M222557 | 0.64481777 |
| S100A7A | S100 Calcium Binding Protein A7A | Protein Coding | 31 | GC01P153416 | 0.644799054 |
| WDFY2 | WD Repeat And FYVE Domain Containing 2 | Protein Coding | 30 | GC13P051584 | 0.644736171 |
| MRPL50 | Mitochondrial Ribosomal Protein L50 | Protein Coding | 30 | GC09M101387 | 0.644561648 |
| MIR4652 | MicroRNA 4652 | RNA Gene | 10 | GC07P093716 | 0.644475341 |
| GRIN2C | Glutamate Ionotropic Receptor NMDA Type Subunit 2C | Protein Coding | 40 | GC17M074842 | 0.644347906 |
| ZNF121 | Zinc Finger Protein 121 | Protein Coding | 26 | GC19M009561 | 0.644239545 |
| TTI2 | TELO2 Interacting Protein 2 | Protein Coding | 32 | GC08M033473 | 0.644018888 |
| ZMAT2 | Zinc Finger Matrin-Type 2 | Protein Coding | 28 | GC05P146096 | 0.643663108 |
| MIR600HG | MIR600 Host Gene | RNA Gene | 19 | GC09M123109 | 0.643663108 |
| OXGR1 | Oxoglutarate Receptor 1 | Protein Coding | 36 | GC13M096985 | 0.643099725 |
| BCAP29 | B Cell Receptor Associated Protein 29 | Protein Coding | 32 | GC07P107579 | 0.642947674 |
| TIMM10 | Translocase Of Inner Mitochondrial Membrane 10 | Protein Coding | 32 | GC11M086957 | 0.641957819 |
| ACTR3C | Actin Related Protein 3C | Protein Coding | 27 | GC07M149887 | 0.641242385 |
| MIR4673 | MicroRNA 4673 | RNA Gene | 11 | GC09M137430 | 0.640507698 |
| PLEKHG2 | Pleckstrin Homology And RhoGEF Domain Containing G2 | Protein Coding | 34 | GC19P039412 | 0.640009224 |
| PIH1D2 | PIH1 Domain Containing 2 | Protein Coding | 30 | GC11M112410 | 0.639856935 |
| GCHFR | GTP Cyclohydrolase I Feedback Regulator | Protein Coding | 35 | GC15P041220 | 0.639716208 |
| NCKAP5 | NCK Associated Protein 5 | Protein Coding | 30 | GC02M132671 | 0.639367461 |
| ARHGAP21 | Rho GTPase Activating Protein 21 | Protein Coding | 34 | GC10M024583 | 0.638829827 |
| ISCA2 | Iron-Sulfur Cluster Assembly 2 | Protein Coding | 35 | GC14P074493 | 0.638760626 |
| IL17D | Interleukin 17D | Protein Coding | 33 | GC13P020702 | 0.638760626 |
| NAPRT | Nicotinate Phosphoribosyltransferase | Protein Coding | 34 | GC08M143574 | 0.638060451 |
| IFFO1 | Intermediate Filament Family Orphan 1 | Protein Coding | 29 | GC12M006538 | 0.637924492 |
| SLC9A6 | Solute Carrier Family 9 Member A6 | Protein Coding | 42 | GC0XP135985 | 0.637681723 |
| KHNYN | KH And NYN Domain Containing | Protein Coding | 30 | GC14P031764 | 0.637624264 |
| C10orf71 | Chromosome 10 Open Reading Frame 71 | Protein Coding | 28 | GC10P049297 | 0.637624264 |
| TMEM9B | TMEM9 Domain Family Member B | Protein Coding | 28 | GC11M008947 | 0.637624264 |
| DPH5 | Diphthamide Biosynthesis 5 | Protein Coding | 32 | GC01M100989 | 0.637326717 |
| RAET1E | Retinoic Acid Early Transcript 1E | Protein Coding | 32 | GC06M149883 | 0.636723936 |
| AMY2B | Amylase Alpha 2B | Protein Coding | 36 | GC01P103554 | 0.63636595 |
| SHROOM4 | Shroom Family Member 4 | Protein Coding | 31 | GC0XM050576 | 0.636259556 |
| SRGAP2C | SLIT-ROBO Rho GTPase Activating Protein 2C | Protein Coding | 22 | GC01P121184 | 0.6351192 |
| FAM107B | Family With Sequence Similarity 107 Member B | Protein Coding | 29 | GC10M014519 | 0.633041143 |
| NT5M | 5',3'-Nucleotidase, Mitochondrial | Protein Coding | 34 | GC17P017303 | 0.632497787 |
| EIF1AY | Eukaryotic Translation Initiation Factor 1A Y-Linked | Protein Coding | 30 | GC0YP020576 | 0.631657124 |
| ZNF79 | Zinc Finger Protein 79 | Protein Coding | 34 | GC09P127424 | 0.631640017 |
| MIR4731 | MicroRNA 4731 | RNA Gene | 11 | GC17M015251 | 0.631274045 |
| MIR4658 | MicroRNA 4658 | RNA Gene | 10 | GC07M100156 | 0.631274045 |
| TTC1 | Tetratricopeptide Repeat Domain 1 | Protein Coding | 31 | GC05P160009 | 0.630835116 |
| SEZ6 | Seizure Related 6 Homolog | Protein Coding | 32 | GC17M028954 | 0.630561709 |
| SERF1A | Small EDRK-Rich Factor 1A | Protein Coding | 27 | GC05P070900 | 0.630561709 |
| ZNF362 | Zinc Finger Protein 362 | Protein Coding | 26 | GC01P033256 | 0.630103946 |
| WDR76 | WD Repeat Domain 76 | Protein Coding | 30 | GC15P043826 | 0.62990886 |
| RCBTB1 | RCC1 And BTB Domain Containing Protein 1 | Protein Coding | 36 | GC13M049531 | 0.62930268 |
| OSBPL7 | Oxysterol Binding Protein Like 7 | Protein Coding | 32 | GC17M047807 | 0.628596604 |
| TRMT44 | TRNA Methyltransferase 44 Homolog | Protein Coding | 28 | GC04P008439 | 0.628596604 |
| LRRTM1 | Leucine Rich Repeat Transmembrane Neuronal 1 | Protein Coding | 35 | GC02M080264 | 0.628460824 |
| NR2C2AP | Nuclear Receptor 2C2 Associated Protein | Protein Coding | 30 | GC19M019201 | 0.628460824 |
| ZSCAN26 | Zinc Finger And SCAN Domain Containing 26 | Protein Coding | 28 | GC06P079974 | 0.628460824 |
| OBI1 | ORC Ubiquitin Ligase 1 | Protein Coding | 26 | GC13M078615 | 0.628261983 |
| EXTL3-AS1 | EXTL3 Antisense RNA 1 | RNA Gene | 14 | GC08M028679 | 0.627829194 |
| EVI5L | Ecotropic Viral Integration Site 5 Like | Protein Coding | 30 | GC19P007830 | 0.627419233 |
| PAQR5 | Progestin And AdipoQ Receptor Family Member 5 | Protein Coding | 32 | GC15P114664 | 0.62665391 |

| | | | | | |
|---|---|---|---|---|---|
| MIR519A2 | MicroRNA 519a-2 | RNA Gene | 13 | GC19P053762 | 0.626039863 |
| IFNL2 | Interferon Lambda 2 | Protein Coding | 31 | GC19P039268 | 0.62577647 |
| ANKRD30BL | Ankyrin Repeat Domain 30B Like | Protein Coding | 19 | GC02M133874 | 0.625682652 |
| RIDA | Reactive Intermediate Imine Deaminase A Homolog | Protein Coding | 29 | GC08M098103 | 0.625020027 |
| RPE | Ribulose-5-Phosphate-3-Epimerase | Protein Coding | 36 | GC02P210002 | 0.624980807 |
| RUFY4 | RUN And FYVE Domain Containing 4 | Protein Coding | 26 | GC02P218034 | 0.624961972 |
| PPP2R2D | Protein Phosphatase 2 Regulatory Subunit Bdelta | Protein Coding | 31 | GC10P131900 | 0.624824941 |
| RILPL2 | Rab Interacting Lysosomal Protein Like 2 | Protein Coding | 31 | GC12M123410 | 0.624728441 |
| ZNF358 | Zinc Finger Protein 358 | Protein Coding | 29 | GC19P007515 | 0.624728441 |
| PHB1P3 | PHB1 Pseudogene 3 | Pseudogene | 5 | GC01P059001 | 0.624631107 |
| MIR1291 | MicroRNA 1291 | RNA Gene | 13 | GC12M048654 | 0.624338686 |
| MIR4721 | MicroRNA 4721 | RNA Gene | 11 | GC16M036336 | 0.622926891 |
| MAP4K2 | Mitogen-Activated Protein Kinase Kinase Kinase Kinase 2 | Protein Coding | 41 | GC11M087217 | 0.622665644 |
| RFX7 | Regulatory Factor X7 | Protein Coding | 29 | GC15M056087 | 0.622487962 |
| ASIC2 | Acid Sensing Ion Channel Subunit 2 | Protein Coding | 35 | GC17M033013 | 0.622235298 |
| ARL1 | ADP Ribosylation Factor Like GTPase 1 | Protein Coding | 36 | GC12M101393 | 0.621792912 |
| NARF | Nuclear Prelamin A Recognition Factor | Protein Coding | 34 | GC17P082458 | 0.621531785 |
| ANGPTL7 | Angiopoietin Like 7 | Protein Coding | 35 | GC01P011189 | 0.621251822 |
| RDH8 | Retinol Dehydrogenase 8 | Protein Coding | 33 | GC19P010316 | 0.62079978 |
| CGNL1 | Cingulin Like 1 | Protein Coding | 34 | GC15P057375 | 0.620505452 |
| ZNF280C | Zinc Finger Protein 280C | Protein Coding | 30 | GC0XM130202 | 0.62024945 |
| WDR91 | WD Repeat Domain 91 | Protein Coding | 29 | GC07M135183 | 0.62024945 |
| MON1B | MON1 Homolog B, Secretory Trafficking Associated | Protein Coding | 31 | GC16P077190 | 0.620007396 |
| TLCD1 | TLC Domain Containing 1 | Protein Coding | 26 | GC17M028724 | 0.620007396 |
| FAM120AOS | Family With Sequence Similarity 120A Opposite Strand | Protein Coding | 26 | GC09M093431 | 0.619219422 |
| ZBTB47 | Zinc Finger And BTB Domain Containing 47 | Protein Coding | 28 | GC03P042653 | 0.618865967 |
| LRIF1 | Ligand Dependent Nuclear Receptor Interacting Factor 1 | Protein Coding | 31 | GC01M110877 | 0.618078947 |
| CMAHP | Cytidine Monophospho-N-Acetylneuraminic Acid Hydroxylase, Pseudogene | Pseudogene | 21 | GC06M025082 | 0.618078947 |
| PITPNC1 | Phosphatidylinositol Transfer Protein Cytoplasmic 1 | Protein Coding | 32 | GC17P067377 | 0.617828727 |
| VPS36 | Vacuolar Protein Sorting 36 Homolog | Protein Coding | 31 | GC13M052412 | 0.617763281 |
| CENPS-CORT | CENPS-CORT Readthrough | Protein Coding | 19 | GC01P010466 | 0.617549777 |
| MARCHF11 | Membrane Associated Ring-CH-Type Finger 11 | Protein Coding | 23 | GC05M016069 | 0.616573811 |
| CYP1B1-AS1 | CYP1B1 Antisense RNA 1 | RNA Gene | 19 | GC02P038075 | 0.616573811 |
| PRDM11 | PR/SET Domain 11 | Protein Coding | 28 | GC11P045072 | 0.616393149 |
| NPM1P13 | Nucleophosmin 1 Pseudogene 13 | Pseudogene | 8 | GC07M018949 | 0.615999699 |
| CRELD2 | Cysteine Rich With EGF Like Domains 2 | Protein Coding | 36 | GC22P049918 | 0.615688801 |
| RNF38 | Ring Finger Protein 38 | Protein Coding | 31 | GC09M036336 | 0.615666389 |
| MRPL42 | Mitochondrial Ribosomal Protein L42 | Protein Coding | 34 | GC12P093467 | 0.615416706 |
| TBC1D10B | TBC1 Domain Family Member 10B | Protein Coding | 30 | GC16M030357 | 0.615416706 |
| PTPDC1 | Protein Tyrosine Phosphatase Domain Containing 1 | Protein Coding | 29 | GC09P094030 | 0.615416706 |
| GAGE13 | G Antigen 13 | Protein Coding | 16 | GC0XP050551 | 0.615416706 |
| FAM186B | Family With Sequence Similarity 186 Member B | Protein Coding | 28 | GC12M049582 | 0.614942908 |
| KICS2 | KICSTOR Subunit 2 | Protein Coding | 23 | GC12M064187 | 0.614382148 |
| OSGEPL1 | O-Sialoglycoprotein Endopeptidase Like 1 | Protein Coding | 33 | GC02M189746 | 0.613854647 |
| MIR3126 | MicroRNA 3126 | RNA Gene | 13 | GC02P069103 | 0.613849878 |
| MIR4641 | MicroRNA 4641 | RNA Gene | 10 | GC06P041598 | 0.613849878 |
| MIR4725 | MicroRNA 4725 | RNA Gene | 10 | GC17P031575 | 0.613849878 |
| RHOV | Ras Homolog Family Member V | Protein Coding | 31 | GC15M040872 | 0.612547815 |
| GRIK4 | Glutamate Ionotropic Receptor Kainate Type Subunit 4 | Protein Coding | 39 | GC11P120512 | 0.611296237 |
| ZBTB18 | Zinc Finger And BTB Domain Containing 18 | Protein Coding | 35 | GC01P244048 | 0.611083806 |
| PSD2 | Pleckstrin And Sec7 Domain Containing 2 | Protein Coding | 30 | GC05P139721 | 0.61091429 |
| KCND1 | Potassium Voltage-Gated Channel Subfamily D Member 1 | Protein Coding | 36 | GC0XM049169 | 0.610781789 |
| SLC25A33 | Solute Carrier Family 25 Member 33 | Protein Coding | 32 | GC01P009539 | 0.609993935 |
| HOMEZ | Homeobox And Leucine Zipper Encoding | Protein Coding | 32 | GC14M023439 | 0.609877706 |
| TBCEL | Tubulin Folding Cofactor E Like | Protein Coding | 31 | GC11P121024 | 0.609628022 |
| MAST3 | Microtubule Associated Serine/Threonine Kinase 3 | Protein Coding | 35 | GC19P063122 | 0.608796716 |
| LOC106128906 | KLK2 Upstream Enhancer/Promoter Region | Biological Region | 2 | GC19P050866 | 0.608796716 |
| NPY2R | Neuropeptide Y Receptor Y2 | Protein Coding | 42 | GC04P155173 | 0.608674109 |
| TCTE1 | T-Complex-Associated-Testis-Expressed 1 | Protein Coding | 31 | GC06M044278 | 0.608581483 |
| MTCO2P12 | MT-CO2 Pseudogene 12 | Pseudogene | 7 | GC01P003573 | 0.607719839 |
| SFR1 | SWI5 Dependent Homologous Recombination Repair Protein 1 | Protein Coding | 27 | GC10P104120 | 0.606396735 |
| MFSD13A | Major Facilitator Superfamily Domain Containing 13A | Protein Coding | 25 | GC10P102462 | 0.606170654 |
| IQCA1 | IQ Motif Containing With AAA Domain 1 | Protein Coding | 30 | GC02M236324 | 0.605466485 |
| TSPAN16 | Tetraspanin 16 | Protein Coding | 27 | GC19P011296 | 0.605466485 |
| CD300LG | CD300 Molecule Like Family Member G | Protein Coding | 32 | GC17P053184 | 0.604603589 |
| OBP2B | Odorant Binding Protein 2B | Protein Coding | 27 | GC09M133205 | 0.604603589 |
| LOC284379 | Solute Carrier Family 7 Member 3 Pseudogene | Pseudogene | 10 | GC19M053599 | 0.604603589 |
| UBE2G1 | Ubiquitin Conjugating Enzyme E2 G1 | Protein Coding | 38 | GC17M004357 | 0.60386169 |
| MIR3913-1 | MicroRNA 3913-1 | RNA Gene | 14 | GC12M069584 | 0.603809714 |
| MIR3124 | MicroRNA 3124 | RNA Gene | 13 | GC01P248826 | 0.603809714 |
| MIR3152 | MicroRNA 3152 | RNA Gene | 13 | GC09P018573 | 0.603809714 |
| MIR3158-1 | MicroRNA 3158-1 | RNA Gene | 13 | GC10P101601 | 0.603809714 |
| MIR3180-1 | MicroRNA 3180-1 | RNA Gene | 13 | GC16P017269 | 0.603809714 |
| MIR3911 | MicroRNA 3911 | RNA Gene | 13 | GC09M127690 | 0.603809714 |
| MIR3179-1 | MicroRNA 3179-1 | RNA Gene | 12 | GC16P017266 | 0.603809714 |
| MIR3914-1 | MicroRNA 3914-1 | RNA Gene | 12 | GC07M071307 | 0.603809714 |
| MIR3529 | MicroRNA 3529 | RNA Gene | 11 | GC15M088611 | 0.603809714 |
| MIR4469 | MicroRNA 4469 | RNA Gene | 11 | GC08M042896 | 0.603809714 |
| MIR4700 | MicroRNA 4700 | RNA Gene | 11 | GC12P120723 | 0.603809714 |
| MIR3914-2 | MicroRNA 3914-2 | RNA Gene | 10 | GC07P071307 | 0.603809714 |
| MIR3925 | MicroRNA 3925 | RNA Gene | 10 | GC06M063760 | 0.603809714 |
| MIR3926-2 | MicroRNA 3926-2 | RNA Gene | 10 | GC08P012727 | 0.603809714 |
| SH3RF1 | SH3 Domain Containing Ring Finger 1 | Protein Coding | 34 | GC04M169094 | 0.603008032 |
| MOB2 | MOB Kinase Activator 2 | Protein Coding | 30 | GC11M002995 | 0.602812886 |
| SLC22A8 | Solute Carrier Family 22 Member 8 | Protein Coding | 38 | GC11M087151 | 0.602671206 |
| SPACA5 | Sperm Acrosome Associated 5 | Protein Coding | 26 | GC0XP048005 | 0.602399468 |
| SPACA5B | Sperm Acrosome Associated 5B | Protein Coding | 22 | GC0XP048130 | 0.602399468 |
| CBWD5 | COBW Domain Containing 5 | Protein Coding | 23 | GC09P066918 | 0.601665437 |
| R3HCC1 | R3H Domain And Coiled-Coil Containing 1 | Protein Coding | 27 | GC08P023270 | 0.601132274 |
| CENPX | Centromere Protein X | Protein Coding | 22 | GC17M082020 | 0.600911558 |
| SEPHS1 | Selenophosphate Synthetase 1 | Protein Coding | 38 | GC10M013317 | 0.600711584 |
| TRAPPC11 | Trafficking Protein Particle Complex Subunit 11 | Protein Coding | 31 | GC04P183659 | 0.599900126 |
| DOLK | Dolichol Kinase | Protein Coding | 35 | GC09M128945 | 0.598450303 |
| SPTBN5 | Spectrin Beta, Non-Erythrocytic 5 | Protein Coding | 31 | GC15M041848 | 0.598137558 |
| ODF2L | Outer Dense Fiber Of Sperm Tails 2 Like | Protein Coding | 31 | GC01M086346 | 0.59733969 |

| | | | | | |
|---|---|---|---|---|---|
| SEC14L4 | SEC14 Like Lipid Binding 4 | Protein Coding | 32 | GC22M030488 | 0.596610665 |
| ZNF625 | Zinc Finger Protein 625 | Protein Coding | 27 | GC19M012294 | 0.596610665 |
| TSGA10IP | Testis Specific 10 Interacting Protein | Protein Coding | 26 | GC11P069577 | 0.596610665 |
| OXCT2 | 3-Oxoacid CoA-Transferase 2 | Protein Coding | 31 | GC01M039776 | 0.596469939 |
| TMEM123 | Transmembrane Protein 123 | Protein Coding | 31 | GC11M102396 | 0.596437812 |
| ADGRG5 | Adhesion G Protein-Coupled Receptor G5 | Protein Coding | 30 | GC16P057529 | 0.596237779 |
| PEPC | Peptidase C | Protein Coding | 6 | GC01U990121 | 0.595327854 |
| NGRN | Neugrin, Neurite Outgrowth Associated | Protein Coding | 31 | GC15P090265 | 0.59502244 |
| HSF2BP | Heat Shock Transcription Factor 2 Binding Protein | Protein Coding | 32 | GC21M043453 | 0.592564404 |
| LIN37 | Lin-37 DREAM MuvB Core Complex Component | Protein Coding | 27 | GC19P063462 | 0.592491508 |
| MIR3173 | MicroRNA 3173 | RNA Gene | 17 | GC14M095137 | 0.592413604 |
| MIR3143 | MicroRNA 3143 | RNA Gene | 14 | GC06P079912 | 0.592413604 |
| MIR3198-1 | MicroRNA 3198-1 | RNA Gene | 14 | GC22M017764 | 0.592413604 |
| MIR4659A | MicroRNA 4659a | RNA Gene | 14 | GC08P006745 | 0.592413604 |
| MIR219B | MicroRNA 219b | RNA Gene | 13 | GC09P128877 | 0.592413604 |
| MIR3136 | MicroRNA 3136 | RNA Gene | 13 | GC03M069048 | 0.592413604 |
| MIR3146 | MicroRNA 3146 | RNA Gene | 13 | GC07M019744 | 0.592413604 |
| MIR3165 | MicroRNA 3165 | RNA Gene | 13 | GC11M072072 | 0.592413604 |
| MIR3179-2 | MicroRNA 3179-2 | RNA Gene | 13 | GC16P017332 | 0.592413604 |
| MIR3180-2 | MicroRNA 3180-2 | RNA Gene | 13 | GC16P016309 | 0.592413604 |
| MIR3183 | MicroRNA 3183 | RNA Gene | 13 | GC17M001022 | 0.592413604 |
| MIR3591 | MicroRNA 3591 | RNA Gene | 13 | GC18M058451 | 0.592413604 |
| MIR3661 | MicroRNA 3661 | RNA Gene | 13 | GC05P134225 | 0.592413604 |
| MIR3936 | MicroRNA 3936 | RNA Gene | 13 | GC05M132365 | 0.592413604 |
| MIR4524A | MicroRNA 4524a | RNA Gene | 13 | GC17M069099 | 0.592413604 |
| MIR4526 | MicroRNA 4526 | RNA Gene | 13 | GC18P017134 | 0.592413604 |
| MIR4632 | MicroRNA 4632 | RNA Gene | 13 | GC01P012191 | 0.592413604 |
| MIR4659B | MicroRNA 4659b | RNA Gene | 13 | GC08M006747 | 0.592413604 |
| MIR4687 | MicroRNA 4687 | RNA Gene | 13 | GC11P003856 | 0.592413604 |
| MIR4707 | MicroRNA 4707 | RNA Gene | 13 | GC14M022961 | 0.592413604 |
| MIR4708 | MicroRNA 4708 | RNA Gene | 13 | GC14M065335 | 0.592413604 |
| MIR4738 | MicroRNA 4738 | RNA Gene | 13 | GC17M075784 | 0.592413604 |
| MIR4754 | MicroRNA 4754 | RNA Gene | 13 | GC19M058386 | 0.592413604 |
| MIR4791 | MicroRNA 4791 | RNA Gene | 13 | GC03M019314 | 0.592413604 |
| MIR4803 | MicroRNA 4803 | RNA Gene | 13 | GC05P072169 | 0.592413604 |
| MIR3176 | MicroRNA 3176 | RNA Gene | 12 | GC16P000543 | 0.592413604 |
| MIR4529 | MicroRNA 4529 | RNA Gene | 12 | GC18P055479 | 0.592413604 |
| MIR4640 | MicroRNA 4640 | RNA Gene | 12 | GC06P030890 | 0.592413604 |
| MIR4664 | MicroRNA 4664 | RNA Gene | 12 | GC08M143733 | 0.592413604 |
| MIR4724 | MicroRNA 4724 | RNA Gene | 12 | GC17P031534 | 0.592413604 |
| MIR4742 | MicroRNA 4742 | RNA Gene | 12 | GC01M224398 | 0.592413604 |
| MIR4745 | MicroRNA 4745 | RNA Gene | 12 | GC19P002479 | 0.592413604 |
| MIR4755 | MicroRNA 4755 | RNA Gene | 12 | GC20P034049 | 0.592413604 |
| MIR4759 | MicroRNA 4759 | RNA Gene | 12 | GC21P026953 | 0.592413604 |
| MIR4785 | MicroRNA 4785 | RNA Gene | 12 | GC02M160408 | 0.592413604 |
| MIR2681 | MicroRNA 2681 | RNA Gene | 11 | GC13M101967 | 0.592413604 |
| MIR3115 | MicroRNA 3115 | RNA Gene | 11 | GC01P023044 | 0.592413604 |
| MIR3128 | MicroRNA 3128 | RNA Gene | 11 | GC02M177255 | 0.592413604 |
| MIR3160-1 | MicroRNA 3160-1 | RNA Gene | 11 | GC11M086863 | 0.592413604 |
| MIR3160-2 | MicroRNA 3160-2 | RNA Gene | 11 | GC11P046451 | 0.592413604 |
| MIR3179-3 | MicroRNA 3179-3 | RNA Gene | 11 | GC16M019166 | 0.592413604 |
| MIR3198-2 | MicroRNA 3198-2 | RNA Gene | 11 | GC12M054231 | 0.592413604 |
| MIR3660 | MicroRNA 3660 | RNA Gene | 11 | GC05M090016 | 0.592413604 |
| MIR3688-2 | MicroRNA 3688-2 | RNA Gene | 11 | GC04P159128 | 0.592413604 |
| MIR4422 | MicroRNA 4422 | RNA Gene | 11 | GC01P055225 | 0.592413604 |
| MIR4489 | MicroRNA 4489 | RNA Gene | 11 | GC11P069552 | 0.592413604 |
| MIR4520-1 | MicroRNA 4520-1 | RNA Gene | 11 | GC17M006655 | 0.592413604 |
| MIR4520-2 | MicroRNA 4520-2 | RNA Gene | 11 | GC17P006655 | 0.592413604 |
| MIR4637 | MicroRNA 4637 | RNA Gene | 11 | GC05M014829 | 0.592413604 |
| MIR4645 | MicroRNA 4645 | RNA Gene | 11 | GC06M002879 | 0.592413604 |
| MIR4646 | MicroRNA 4646 | RNA Gene | 11 | GC06M063616 | 0.592413604 |
| MIR4648 | MicroRNA 4648 | RNA Gene | 11 | GC07P003115 | 0.592413604 |
| MIR4657 | MicroRNA 4657 | RNA Gene | 11 | GC07M044880 | 0.592413604 |
| MIR4660 | MicroRNA 4660 | RNA Gene | 11 | GC08P009048 | 0.592413604 |
| MIR4661 | MicroRNA 4661 | RNA Gene | 11 | GC08P091205 | 0.592413604 |
| MIR4662A | MicroRNA 4662a | RNA Gene | 11 | GC08P124821 | 0.592413604 |
| MIR4668 | MicroRNA 4668 | RNA Gene | 11 | GC09P111932 | 0.592413604 |
| MIR4669 | MicroRNA 4669 | RNA Gene | 11 | GC09P134381 | 0.592413604 |
| MIR4672 | MicroRNA 4672 | RNA Gene | 11 | GC09M127869 | 0.592413604 |
| MIR4680 | MicroRNA 4680 | RNA Gene | 11 | GC10P110898 | 0.592413604 |
| MIR4681 | MicroRNA 4681 | RNA Gene | 11 | GC10P119377 | 0.592413604 |
| MIR4683 | MicroRNA 4683 | RNA Gene | 11 | GC10M035641 | 0.592413604 |
| MIR4684 | MicroRNA 4684 | RNA Gene | 11 | GC01P022719 | 0.592413604 |
| MIR4692 | MicroRNA 4692 | RNA Gene | 11 | GC11P072783 | 0.592413604 |
| MIR4701 | MicroRNA 4701 | RNA Gene | 11 | GC12M048840 | 0.592413604 |
| MIR4703 | MicroRNA 4703 | RNA Gene | 11 | GC13P051552 | 0.592413604 |
| MIR4706 | MicroRNA 4706 | RNA Gene | 11 | GC14P065044 | 0.592413604 |
| MIR4710 | MicroRNA 4710 | RNA Gene | 11 | GC14M104677 | 0.592413604 |
| MIR4733 | MicroRNA 4733 | RNA Gene | 11 | GC17M031094 | 0.592413604 |
| MIR4734 | MicroRNA 4734 | RNA Gene | 11 | GC17M038702 | 0.592413604 |
| MIR4736 | MicroRNA 4736 | RNA Gene | 11 | GC17M058335 | 0.592413604 |
| MIR4741 | MicroRNA 4741 | RNA Gene | 11 | GC18P022933 | 0.592413604 |
| MIR4744 | MicroRNA 4744 | RNA Gene | 11 | GC18M049049 | 0.592413604 |
| MIR4747 | MicroRNA 4747 | RNA Gene | 11 | GC19P004936 | 0.592413604 |
| MIR4748 | MicroRNA 4748 | RNA Gene | 11 | GC19P010780 | 0.592413604 |
| MIR4749 | MicroRNA 4749 | RNA Gene | 11 | GC19P049854 | 0.592413604 |
| MIR4750 | MicroRNA 4750 | RNA Gene | 11 | GC19P049888 | 0.592413604 |
| MIR4752 | MicroRNA 4752 | RNA Gene | 11 | GC19P064326 | 0.592413604 |
| MIR4763 | MicroRNA 4763 | RNA Gene | 11 | GC22P046113 | 0.592413604 |
| MIR4774 | MicroRNA 4774 | RNA Gene | 11 | GC02P168582 | 0.592413604 |
| MIR4776-1 | MicroRNA 4776-1 | RNA Gene | 11 | GC02P212926 | 0.592413604 |
| MIR4776-2 | MicroRNA 4776-2 | RNA Gene | 11 | GC02M212926 | 0.592413604 |
| MIR4780 | MicroRNA 4780 | RNA Gene | 11 | GC02M088082 | 0.592413604 |
| MIR4783 | MicroRNA 4783 | RNA Gene | 11 | GC02M127423 | 0.592413604 |
| MIR4788 | MicroRNA 4788 | RNA Gene | 11 | GC03P134438 | 0.592413604 |

| | | | | | |
|---|---|---|---|---|---|
| MIR4789 | MicroRNA 4789 | RNA Gene | 11 | GC03P175369 | 0.592413604 |
| MIR4797 | MicroRNA 4797 | RNA Gene | 11 | GC03M197293 | 0.592413604 |
| MIR4798 | MicroRNA 4798 | RNA Gene | 11 | GC04P007317 | 0.592413604 |
| MIR4802 | MicroRNA 4802 | RNA Gene | 11 | GC04M040507 | 0.592413604 |
| MIR4804 | MicroRNA 4804 | RNA Gene | 11 | GC05P072879 | 0.592413604 |
| MIR4436A | MicroRNA 4436a | RNA Gene | 10 | GC02P088812 | 0.592413604 |
| MIR4436B1 | MicroRNA 4436b-1 | RNA Gene | 10 | GC02M110086 | 0.592413604 |
| MIR4467 | MicroRNA 4467 | RNA Gene | 10 | GC07P102471 | 0.592413604 |
| MIR4471 | MicroRNA 4471 | RNA Gene | 10 | GC08P100382 | 0.592413604 |
| MIR4633 | MicroRNA 4633 | RNA Gene | 10 | GC05P129097 | 0.592413604 |
| MIR4634 | MicroRNA 4634 | RNA Gene | 10 | GC05P174752 | 0.592413604 |
| MIR4642 | MicroRNA 4642 | RNA Gene | 10 | GC06P044435 | 0.592413604 |
| MIR4647 | MicroRNA 4647 | RNA Gene | 10 | GC06M063881 | 0.592413604 |
| MIR4650-1 | MicroRNA 4650-1 | RNA Gene | 10 | GC07M067115 | 0.592413604 |
| MIR4650-2 | MicroRNA 4650-2 | RNA Gene | 10 | GC07P072697 | 0.592413604 |
| MIR4662B | MicroRNA 4662b | RNA Gene | 10 | GC08M124821 | 0.592413604 |
| MIR4667 | MicroRNA 4667 | RNA Gene | 10 | GC09P040154 | 0.592413604 |
| MIR4676 | MicroRNA 4676 | RNA Gene | 10 | GC10P072721 | 0.592413604 |
| MIR4678 | MicroRNA 4678 | RNA Gene | 10 | GC10P087503 | 0.592413604 |
| MIR4679-2 | MicroRNA 4679-2 | RNA Gene | 10 | GC10M089063 | 0.592413604 |
| MIR4685 | MicroRNA 4685 | RNA Gene | 10 | GC10M098431 | 0.592413604 |
| MIR4686 | MicroRNA 4686 | RNA Gene | 10 | GC11P002173 | 0.592413604 |
| MIR4688 | MicroRNA 4688 | RNA Gene | 10 | GC11P046376 | 0.592413604 |
| MIR4689 | MicroRNA 4689 | RNA Gene | 10 | GC01M005862 | 0.592413604 |
| MIR4694 | MicroRNA 4694 | RNA Gene | 10 | GC11M019784 | 0.592413604 |
| MIR4696 | MicroRNA 4696 | RNA Gene | 10 | GC11M074720 | 0.592413604 |
| MIR4697 | MicroRNA 4697 | RNA Gene | 10 | GC11M133898 | 0.592413604 |
| MIR4705 | MicroRNA 4705 | RNA Gene | 10 | GC13M102045 | 0.592413604 |
| MIR4714 | MicroRNA 4714 | RNA Gene | 10 | GC15P098784 | 0.592413604 |
| MIR4716 | MicroRNA 4716 | RNA Gene | 10 | GC15M049169 | 0.592413604 |
| MIR4719 | MicroRNA 4719 | RNA Gene | 10 | GC16P076868 | 0.592413604 |
| MIR4720 | MicroRNA 4720 | RNA Gene | 10 | GC16P081391 | 0.592413604 |
| MIR4722 | MicroRNA 4722 | RNA Gene | 10 | GC16M088716 | 0.592413604 |
| MIR4726 | MicroRNA 4726 | RNA Gene | 10 | GC17P052990 | 0.592413604 |
| MIR4727 | MicroRNA 4727 | RNA Gene | 10 | GC17P038825 | 0.592413604 |
| MIR4743 | MicroRNA 4743 | RNA Gene | 10 | GC18P048670 | 0.592413604 |
| MIR4751 | MicroRNA 4751 | RNA Gene | 10 | GC19P049933 | 0.592413604 |
| MIR4765 | MicroRNA 4765 | RNA Gene | 10 | GC02P032635 | 0.592413604 |
| MIR4769 | MicroRNA 4769 | RNA Gene | 10 | GC0XP047587 | 0.592413604 |
| MIR4770 | MicroRNA 4770 | RNA Gene | 10 | GC0XM006302 | 0.592413604 |
| MIR4773-1 | MicroRNA 4773-1 | RNA Gene | 10 | GC02P151368 | 0.592413604 |
| MIR4773-2 | MicroRNA 4773-2 | RNA Gene | 10 | GC02M151368 | 0.592413604 |
| MIR4777 | MicroRNA 4777 | RNA Gene | 10 | GC02P231602 | 0.592413604 |
| MIR4781 | MicroRNA 4781 | RNA Gene | 10 | GC01P054054 | 0.592413604 |
| MIR4790 | MicroRNA 4790 | RNA Gene | 10 | GC03M005250 | 0.592413604 |
| MIR4794 | MicroRNA 4794 | RNA Gene | 10 | GC01P064579 | 0.592413604 |
| MIR4799 | MicroRNA 4799 | RNA Gene | 10 | GC04P147782 | 0.592413604 |
| MIR3179-4 | MicroRNA 3179-4 | RNA Gene | 9 | GC16M018494 | 0.592413604 |
| MIR3942 | MicroRNA 3942 | RNA Gene | 9 | GC15M035372 | 0.592413604 |
| MIR4643 | MicroRNA 4643 | RNA Gene | 9 | GC06P091522 | 0.592413604 |
| MIR4663 | MicroRNA 4663 | RNA Gene | 9 | GC08M123215 | 0.592413604 |
| MIR4666A | MicroRNA 4666a | RNA Gene | 9 | GC01P228462 | 0.592413604 |
| MIR4679-1 | MicroRNA 4679-1 | RNA Gene | 9 | GC10P089063 | 0.592413604 |
| MIR4682 | MicroRNA 4682 | RNA Gene | 9 | GC10P119958 | 0.592413604 |
| MIR4699 | MicroRNA 4699 | RNA Gene | 9 | GC12P081158 | 0.592413604 |
| MIR4737 | MicroRNA 4737 | RNA Gene | 9 | GC17M060048 | 0.592413604 |
| MIR4762 | MicroRNA 4762 | RNA Gene | 9 | GC22P045760 | 0.592413604 |
| MIR4764 | MicroRNA 4764 | RNA Gene | 9 | GC22M033436 | 0.592413604 |
| MIR4771-1 | MicroRNA 4771-1 | RNA Gene | 9 | GC02P087194 | 0.592413604 |
| MIR4771-2 | MicroRNA 4771-2 | RNA Gene | 9 | GC02M111771 | 0.592413604 |
| MIR4778 | MicroRNA 4778 | RNA Gene | 9 | GC02M066358 | 0.592413604 |
| MIR4801 | MicroRNA 4801 | RNA Gene | 9 | GC04M037245 | 0.592413604 |
| MIR4436B2 | MicroRNA 4436b-2 | RNA Gene | 8 | GC02P110284 | 0.592413604 |
| MIR4760 | MicroRNA 4760 | RNA Gene | 8 | GC21M040212 | 0.592413604 |
| ZBTB44 | Zinc Finger And BTB Domain Containing 44 | Protein Coding | 30 | GC11M130226 | 0.5907197 |
| MTARC1 | Mitochondrial Amidoxime Reducing Component 1 | Protein Coding | 27 | GC01P220788 | 0.5907197 |
| NPVF | Neuropeptide VF Precursor | Protein Coding | 31 | GC07M025224 | 0.590707362 |
| MIR1256 | MicroRNA 1256 | RNA Gene | 13 | GC01M020988 | 0.590524614 |
| NOVA2 | NOVA Alternative Splicing Regulator 2 | Protein Coding | 34 | GC19M045933 | 0.590417385 |
| CTRB1 | Chymotrypsinogen B1 | Protein Coding | 34 | GC16P075218 | 0.589736164 |
| C3orf33 | Chromosome 3 Open Reading Frame 33 | Protein Coding | 27 | GC03M155764 | 0.589608192 |
| TMEM259 | Transmembrane Protein 259 | Protein Coding | 27 | GC19M004847 | 0.589608192 |
| SCIRT | Stem Cell Inhibitory RNA Transcript | RNA Gene | 10 | GC06M065552 | 0.589282572 |
| PRAMEF20 | PRAME Family Member 20 | Protein Coding | 23 | GC01P013410 | 0.588097334 |
| AGO3 | Argonaute RISC Catalytic Component 3 | Protein Coding | 29 | GC01P035996 | 0.58694452 |
| C1orf94 | Chromosome 1 Open Reading Frame 94 | Protein Coding | 26 | GC01P034166 | 0.58694452 |
| UHRF1BP1 | UHRF1 Binding Protein 1 | Protein Coding | 32 | GC06P080503 | 0.586475551 |
| ZNF141 | Zinc Finger Protein 141 | Protein Coding | 32 | GC04P000331 | 0.58638227 |
| SLC38A7 | Solute Carrier Family 38 Member 7 | Protein Coding | 32 | GC16M058665 | 0.585350335 |
| IRX6 | Iroquois Homeobox 6 | Protein Coding | 31 | GC16P055323 | 0.585157752 |
| CACNA1I | Calcium Voltage-Gated Channel Subunit Alpha1 I | Protein Coding | 41 | GC22P039570 | 0.584872901 |
| HES4 | Hes Family BHLH Transcription Factor 4 | Protein Coding | 29 | GC01M005513 | 0.584831953 |
| TXNDC11 | Thioredoxin Domain Containing 11 | Protein Coding | 31 | GC16M011679 | 0.584155202 |
| RILPL1 | Rab Interacting Lysosomal Protein Like 1 | Protein Coding | 31 | GC12M123470 | 0.583873391 |
| ATP8B4 | ATPase Phospholipid Transporting 8B4 (Putative) | Protein Coding | 35 | GC15M049858 | 0.582891464 |
| QRFP | Pyroglutamylated RFamide Peptide | Protein Coding | 28 | GC09M130892 | 0.582128465 |
| BTBD10 | BTB Domain Containing 10 | Protein Coding | 34 | GC11M013387 | 0.581896067 |
| CCSAP | Centriole, Cilia And Spindle Associated Protein | Protein Coding | 27 | GC01M229408 | 0.580338001 |
| DUSP8 | Dual Specificity Phosphatase 8 | Protein Coding | 32 | GC11M002998 | 0.580203235 |
| RHOBTB3 | Rho Related BTB Domain Containing 3 | Protein Coding | 32 | GC05P095713 | 0.579955935 |
| LURAP1 | Leucine Rich Adaptor Protein 1 | Protein Coding | 27 | GC01P046203 | 0.579795182 |
| GRAP2 | GRB2 Related Adaptor Protein 2 | Protein Coding | 38 | GC22P039901 | 0.579127014 |
| VTRNA1-1 | Vault RNA 1-1 | RNA Gene | 11 | GC05P146092 | 0.577772141 |
| AGPAT4 | 1-Acylglycerol-3-Phosphate O-Acyltransferase 4 | Protein Coding | 34 | GC06M161129 | 0.577253401 |
| NUP54 | Nucleoporin 54 | Protein Coding | 32 | GC04M076114 | 0.577183902 |

| | | | | | |
|---|---|---|---|---|---|
| NKTR | Natural Killer Cell Triggering Receptor | Protein Coding | 32 | GC03P042600 | 0.576801836 |
| EML5 | EMAP Like 5 | Protein Coding | 31 | GC14M100525 | 0.576701283 |
| RNF10 | Ring Finger Protein 10 | Protein Coding | 35 | GC12P120564 | 0.576576233 |
| HIRIP3 | HIRA Interacting Protein 3 | Protein Coding | 32 | GC16M029992 | 0.576452076 |
| RAX2 | Retina And Anterior Neural Fold Homeobox 2 | Protein Coding | 31 | GC19M003769 | 0.575045645 |
| AADACL3 | Arylacetamide Deacetylase Like 3 | Protein Coding | 26 | GC01P012716 | 0.574999213 |
| RAD17P1 | RAD17 Pseudogene 1 | Pseudogene | 8 | GC07M016863 | 0.573468804 |
| PCMTD2 | Protein-L-Isoaspartate (D-Aspartate) O-Methyltransferase Domain Containing 2 | Protein Coding | 30 | GC20P064255 | 0.573447227 |
| SPATA32 | Spermatogenesis Associated 32 | Protein Coding | 24 | GC17M045254 | 0.573447227 |
| GTF2A2 | General Transcription Factor IIA Subunit 2 | Protein Coding | 31 | GC15M059638 | 0.573251426 |
| BTN3A1 | Butyrophilin Subfamily 3 Member A1 | Protein Coding | 37 | GC06P026402 | 0.572099984 |
| H2BC9 | H2B Clustered Histone 9 | Protein Coding | 27 | GC06P081041 | 0.572099984 |
| TMEM150B | Transmembrane Protein 150B | Protein Coding | 27 | GC19M055308 | 0.571026206 |
| GTPBP8 | GTP Binding Protein 8 (Putative) | Protein Coding | 28 | GC03P112990 | 0.569956303 |
| OCM2 | Oncomodulin 2 | Protein Coding | 26 | GC07M097984 | 0.569956303 |
| C9 | Complement C9 | Protein Coding | 40 | GC05M039320 | 0.569925725 |
| TTC4P1 | Tetratricopeptide Repeat Domain 4 Pseudogene 1 | Pseudogene | 9 | GC07M045999 | 0.569500804 |
| FBXW4 | F-Box And WD Repeat Domain Containing 4 | Protein Coding | 32 | GC10M101610 | 0.569056571 |
| ZNF84 | Zinc Finger Protein 84 | Protein Coding | 27 | GC12P133037 | 0.56723541 |
| CMTM4 | CKLF Like MARVEL Transmembrane Domain Containing 4 | Protein Coding | 30 | GC16M066598 | 0.566864192 |
| CACNG6 | Calcium Voltage-Gated Channel Auxiliary Subunit Gamma 6 | Protein Coding | 35 | GC19P053991 | 0.566050649 |
| ADAM21 | ADAM Metallopeptidase Domain 21 | Protein Coding | 32 | GC14P070418 | 0.566050649 |
| MYO7B | Myosin VIIB | Protein Coding | 31 | GC02P127535 | 0.566050649 |
| ASPDH | Aspartate Dehydrogenase Domain Containing | Protein Coding | 28 | GC19M050511 | 0.566050649 |
| CDRT15 | CMT1A Duplicated Region Transcript 15 | Protein Coding | 25 | GC17M014235 | 0.566050649 |
| RNF213-AS1 | RNF213 Antisense RNA 1 | RNA Gene | 13 | GC17M080335 | 0.564152002 |
| PCED1A | PC-Esterase Domain Containing 1A | Protein Coding | 27 | GC20M002835 | 0.563789606 |
| PLEKHH2 | Pleckstrin Homology, MyTH4 And FERM Domain Containing H2 | Protein Coding | 34 | GC02P043600 | 0.563183367 |
| RIMS3 | Regulating Synaptic Membrane Exocytosis 3 | Protein Coding | 34 | GC01M040621 | 0.563183367 |
| ZDHHC17 | Zinc Finger DHHC-Type Palmitoyltransferase 17 | Protein Coding | 34 | GC12P076763 | 0.563183367 |
| GCSAML | Germinal Center Associated Signaling And Motility Like | Protein Coding | 26 | GC01P247507 | 0.563183367 |
| UBTFL1 | Upstream Binding Transcription Factor Like 1 | Protein Coding | 22 | GC11P090085 | 0.563183367 |
| C2CD2 | C2 Calcium Dependent Domain Containing 2 | Protein Coding | 29 | GC21M041885 | 0.562186897 |
| VPS28 | VPS28 Subunit Of ESCRT-I | Protein Coding | 34 | GC08M145339 | 0.562157452 |
| YAF2 | YY1 Associated Factor 2 | Protein Coding | 33 | GC12M042157 | 0.561462104 |
| MISP | Mitotic Spindle Positioning | Protein Coding | 28 | GC19P002465 | 0.561462104 |
| SNHG9 | Small Nucleolar RNA Host Gene 9 | RNA Gene | 16 | GC16P010709 | 0.560963094 |
| ADAT1 | Adenosine Deaminase TRNA Specific 1 | Protein Coding | 35 | GC16M075596 | 0.560459733 |
| GALNT13 | Polypeptide N-Acetylgalactosaminyltransferase 13 | Protein Coding | 35 | GC02P153871 | 0.560190439 |
| ASZ1 | Ankyrin Repeat, SAM And Basic Leucine Zipper Domain Containing 1 | Protein Coding | 33 | GC07M117363 | 0.560140371 |
| SH2B2 | SH2B Adaptor Protein 2 | Protein Coding | 32 | GC07P102286 | 0.560056567 |
| H2BC6 | H2B Clustered Histone 6 | Protein Coding | 26 | GC06P081039 | 0.560056567 |
| FKBP11 | FKBP Prolyl Isomerase 11 | Protein Coding | 31 | GC12M049386 | 0.559788585 |
| MXD4 | MAX Dimerization Protein 4 | Protein Coding | 32 | GC04M002411 | 0.559679389 |
| AKR7A2 | Aldo-Keto Reductase Family 7 Member A2 | Protein Coding | 37 | GC01M019302 | 0.55923605 |
| GTF2IRD2B | GTF2I Repeat Domain Containing 2B | Protein Coding | 27 | GC07P075092 | 0.558901727 |
| TVP23A | Trans-Golgi Network Vesicle Protein 23 Homolog A | Protein Coding | 27 | GC16M011872 | 0.558901727 |
| TEX22 | Testis Expressed 22 | Protein Coding | 23 | GC14P105398 | 0.558901727 |
| GGA2 | Golgi Associated, Gamma Adaptin Ear Containing, ARF Binding Protein 2 | Protein Coding | 34 | GC16M023464 | 0.558460593 |
| SDHAF4 | Succinate Dehydrogenase Complex Assembly Factor 4 | Protein Coding | 24 | GC06P070566 | 0.556245446 |
| THTPA | Thiamine Triphosphatase | Protein Coding | 32 | GC14P031747 | 0.55609411 |
| RASA3 | RAS P21 Protein Activator 3 | Protein Coding | 34 | GC13M113977 | 0.555876672 |
| ISOC1 | Isochorismatase Domain Containing 1 | Protein Coding | 29 | GC05P129094 | 0.555876672 |
| SNORD33 | Small Nucleolar RNA, C/D Box 33 | RNA Gene | 15 | GC19P064063 | 0.55585134 |
| TPD52L3 | TPD52 Like 3 | Protein Coding | 31 | GC09P006328 | 0.554891288 |
| NAPB | NSF Attachment Protein Beta | Protein Coding | 34 | GC20M023374 | 0.554860294 |
| CACNG4 | Calcium Voltage-Gated Channel Auxiliary Subunit Gamma 4 | Protein Coding | 37 | GC17P066964 | 0.554795265 |
| NAPEPLD | N-Acyl Phosphatidylethanolamine Phospholipase D | Protein Coding | 37 | GC07M103099 | 0.554795265 |
| CD300C | CD300c Molecule | Protein Coding | 33 | GC17M074544 | 0.554795265 |
| SPIC | Spi-C Transcription Factor | Protein Coding | 31 | GC12P101475 | 0.554795265 |
| PKD1L3 | Polycystin 1 Like 3, Transient Receptor Potential Channel Interacting | Protein Coding | 27 | GC16M071962 | 0.554795265 |
| PPP1R27 | Protein Phosphatase 1 Regulatory Subunit 27 | Protein Coding | 27 | GC17M081833 | 0.554795265 |
| SAP25 | Sin3A Associated Protein 25 | Protein Coding | 26 | GC07M101765 | 0.554795265 |
| SEC14L6 | SEC14 Like Lipid Binding 6 | Protein Coding | 26 | GC22M030522 | 0.554795265 |
| LRRC74A | Leucine Rich Repeat Containing 74A | Protein Coding | 25 | GC14P076826 | 0.554795265 |
| REX1BD | Required For Excision 1-B Domain Containing | Protein Coding | 23 | GC19P066081 | 0.554795265 |
| DPY19L2P2 | DPY19L2 Pseudogene 2 | Pseudogene | 19 | GC07M103175 | 0.554795265 |
| SPATA41 | Spermatogenesis Associated 41 | RNA Gene | 15 | GC15M116018 | 0.554795265 |
| CHRM3-AS2 | CHRM3 Antisense RNA 2 | RNA Gene | 14 | GC01M239703 | 0.554795265 |
| FGF10-AS1 | FGF10 Antisense RNA 1 | RNA Gene | 14 | GC05P044389 | 0.554795265 |
| LINC01090 | Long Intergenic Non-Protein Coding RNA 1090 | RNA Gene | 14 | GC02M187712 | 0.554795265 |
| MIR548AA1 | MicroRNA 548aa-1 | RNA Gene | 14 | GC08P123348 | 0.554795265 |
| IPO9-AS1 | IPO9 Antisense RNA 1 | RNA Gene | 13 | GC01M201658 | 0.554795265 |
| LINC01254 | Long Intergenic Non-Protein Coding RNA 1254 | RNA Gene | 12 | GC18M010410 | 0.554795265 |
| LINC01394 | Long Intergenic Non-Protein Coding RNA 1394 | RNA Gene | 10 | GC06M001208 | 0.554795265 |
| ZNF205 | Zinc Finger Protein 205 | Protein Coding | 32 | GC16P010763 | 0.553839684 |
| ZNF17 | Zinc Finger Protein 17 | Protein Coding | 30 | GC19P057411 | 0.553839684 |
| HAGHL | Hydroxyacylglutathione Hydrolase Like | Protein Coding | 28 | GC16P010650 | 0.553839684 |
| GOLGA2P5 | GOLGA2 Pseudogene 5 | Pseudogene | 15 | GC12M100156 | 0.553839684 |
| MIR1245A | MicroRNA 1245a | RNA Gene | 12 | GC02P188978 | 0.553391695 |
| FTSJ1 | FtsJ RNA 2'-O-Methyltransferase 1 | Protein Coding | 38 | GC0XP048476 | 0.552875221 |
| ANKRD10 | Ankyrin Repeat Domain 10 | Protein Coding | 29 | GC13M110878 | 0.552491605 |
| PCDH9 | Protocadherin 9 | Protein Coding | 36 | GC13M066302 | 0.552263558 |
| LOC111240475 | HSD17B1 Promoter | Biological Region | 2 | GC17P042552 | 0.552020192 |
| GGT3P | Gamma-Glutamyltransferase 3 Pseudogene | Pseudogene | 18 | GC22M018773 | 0.551489711 |
| PABIR2 | PABIR Family Member 2 | Protein Coding | 24 | GC0XM134824 | 0.55138272 |
| CHRM4 | Cholinergic Receptor Muscarinic 4 | Protein Coding | 40 | GC11M086859 | 0.550634146 |
| COL6A6 | Collagen Type VI Alpha 6 Chain | Protein Coding | 30 | GC03P133851 | 0.55047524 |
| FBXO44 | F-Box Protein 44 | Protein Coding | 29 | GC01P011654 | 0.550151408 |
| BATF3 | Basic Leucine Zipper ATF-Like Transcription Factor 3 | Protein Coding | 32 | GC01M212686 | 0.550087988 |
| TBC1D16 | TBC1 Domain Family Member 16 | Protein Coding | 30 | GC17M079932 | 0.550087988 |
| MDP1 | Magnesium Dependent Phosphatase 1 | Protein Coding | 27 | GC14M024223 | 0.550087988 |
| LOC107963951 | BCR-ABL Minor-Breakpoint Cluster Region | Biological Region | 2 | GC22P035344 | 0.549474597 |
| COA6 | Cytochrome C Oxidase Assembly Factor 6 | Protein Coding | 32 | GC01P234374 | 0.549102902 |
| DENND6A | DENN Domain Containing 6A | Protein Coding | 28 | GC03M057612 | 0.548960328 |

| | | | | | |
|---|---|---|---|---|---|
| LRRC57 | Leucine Rich Repeat Containing 57 | Protein Coding | 31 | GC15M042566 | 0.547898293 |
| BRME1 | Break Repair Meiotic Recombinase Recruitment Factor 1 | Protein Coding | 23 | GC19M014466 | 0.547496796 |
| YIPF6 | Yip1 Domain Family Member 6 | Protein Coding | 30 | GC0XP068498 | 0.54599154 |
| FAM20B | FAM20B Glycosaminoglycan Xylosylkinase | Protein Coding | 34 | GC01P179025 | 0.545566499 |
| APOLD1 | Apolipoprotein L Domain Containing 1 | Protein Coding | 30 | GC12P012725 | 0.545566499 |
| ZNF280A | Zinc Finger Protein 280A | Protein Coding | 27 | GC22M022513 | 0.545566499 |
| ARL4A | ADP Ribosylation Factor Like GTPase 4A | Protein Coding | 33 | GC07P012712 | 0.544990063 |
| PRR5-ARHGAP8 | PRR5-ARHGAP8 Readthrough | Protein Coding | 21 | GC22P044702 | 0.544271231 |
| COL28A1 | Collagen Type XXVIII Alpha 1 Chain | Protein Coding | 30 | GC07M007338 | 0.543573439 |
| FBXL19 | F-Box And Leucine Rich Repeat Protein 19 | Protein Coding | 31 | GC16P040283 | 0.542908192 |
| SIGLEC10 | Sialic Acid Binding Ig Like Lectin 10 | Protein Coding | 36 | GC19M051410 | 0.542750418 |
| RRAGD | Ras Related GTP Binding D | Protein Coding | 32 | GC06M089364 | 0.542557061 |
| IGKV2-40 | Immunoglobulin Kappa Variable 2-40 | Protein Coding | 9 | GC02M090838 | 0.541456819 |
| WWC2 | WW And C2 Domain Containing 2 | Protein Coding | 31 | GC04P183099 | 0.541070521 |
| ERVMER34-1 | Endogenous Retrovirus Group MER34 Member 1, Envelope | Protein Coding | 25 | GC04M052722 | 0.539841652 |
| SCEL | Sciellin | Protein Coding | 33 | GC13P077535 | 0.539746165 |
| NIF3L1 | NGG1 Interacting Factor 3 Like 1 | Protein Coding | 35 | GC02P200889 | 0.538743794 |
| LOC107988021 | IKBKG Recombination Region | Biological Region | 2 | GC0XP154556 | 0.538632274 |
| LOC107988022 | IKBKG Downstream Recombination Region | Biological Region | 2 | GC0XP154568 | 0.538632274 |
| LOC107988024 | IKBKGP1 Recombination Region | Biological Region | 2 | GC0XP154646 | 0.538632274 |
| LOC107988025 | IKBKGP1 Upstream Recombination Region | Biological Region | 2 | GC0XP154634 | 0.538632274 |
| NUS1P3 | NUS1 Pseudogene 3 | Pseudogene | 7 | GC13P024327 | 0.538589239 |
| MTND6P4 | MT-ND6 Pseudogene 4 | Pseudogene | 6 | GC05P134924 | 0.538589239 |
| KLHL29 | Kelch Like Family Member 29 | Protein Coding | 28 | GC02P023348 | 0.53827858 |
| PABIR1 | PP2A Aalpha (PPP2R1A) And B55A (PPP2R2A) Interacting Phosphatase Regulator 1 | Protein Coding | 24 | GC09P068781 | 0.53827858 |
| CCDC116 | Coiled-Coil Domain Containing 116 | Protein Coding | 28 | GC22P035108 | 0.537805855 |
| METTL7A | Methyltransferase Like 7A | Protein Coding | 32 | GC12P050923 | 0.537799656 |
| KCNH7 | Potassium Voltage-Gated Channel Subfamily H Member 7 | Protein Coding | 38 | GC02M162371 | 0.536642909 |
| VPS25 | Vacuolar Protein Sorting 25 Homolog | Protein Coding | 35 | GC17P042773 | 0.536642909 |
| CPNE5 | Copine 5 | Protein Coding | 31 | GC06M063762 | 0.536642909 |
| OR5V1 | Olfactory Receptor Family 5 Subfamily V Member 1 | Protein Coding | 29 | GC06M029353 | 0.536642909 |
| MOB3C | MOB Kinase Activator 3C | Protein Coding | 28 | GC01M046607 | 0.536642909 |
| PRR3 | Proline Rich 3 | Protein Coding | 28 | GC06P080327 | 0.536642909 |
| DLEU7 | Deleted In Lymphocytic Leukemia 7 | Protein Coding | 25 | GC13M050519 | 0.536642909 |
| MPZL2 | Myelin Protein Zero Like 2 | Protein Coding | 34 | GC11M118253 | 0.536602736 |
| FIBCD1 | Fibrinogen C Domain Containing 1 | Protein Coding | 31 | GC09M130902 | 0.534798205 |
| BOLA2 | BolA Family Member 2 | Protein Coding | 28 | GC16M036397 | 0.534798205 |
| PRKRIP1 | PRKR Interacting Protein 1 | Protein Coding | 28 | GC07P102905 | 0.534798205 |
| SPDL1 | Spindle Apparatus Coiled-Coil Protein 1 | Protein Coding | 28 | GC05P169583 | 0.534798205 |
| FBXL18 | F-Box And Leucine Rich Repeat Protein 18 | Protein Coding | 30 | GC07M005431 | 0.534051597 |
| EVI2B | Ecotropic Viral Integration Site 2B | Protein Coding | 30 | GC17M034676 | 0.533672214 |
| UBQLN3 | Ubiquilin 3 | Protein Coding | 31 | GC11M006344 | 0.532682657 |
| DBX2 | Developing Brain Homeobox 2 | Protein Coding | 32 | GC12M045014 | 0.53134346 |
| PRLH | Prolactin Releasing Hormone | Protein Coding | 30 | GC02P237566 | 0.530559659 |
| SNORD108 | Small Nucleolar RNA, C/D Box 108 | RNA Gene | 14 | GC15P039155 | 0.530559659 |
| OR2T1 | Olfactory Receptor Family 2 Subfamily T Member 1 | Protein Coding | 27 | GC01P248405 | 0.52987498 |
| SHLD2 | Shieldin Complex Subunit 2 | Protein Coding | 26 | GC10P091519 | 0.529814959 |
| TBC1D17 | TBC1 Domain Family Member 17 | Protein Coding | 30 | GC19P049877 | 0.529666781 |
| ZNF506 | Zinc Finger Protein 506 | Protein Coding | 29 | GC19M019785 | 0.529666781 |
| RAB39A | RAB39A, Member RAS Oncogene Family | Protein Coding | 29 | GC11P107928 | 0.529387951 |
| RELL1 | RELT Like 1 | Protein Coding | 27 | GC04M037592 | 0.529387951 |
| FBXO25 | F-Box Protein 25 | Protein Coding | 31 | GC08P000406 | 0.528781116 |
| TMEM132A | Transmembrane Protein 132A | Protein Coding | 30 | GC11P060924 | 0.528781116 |
| RBM18 | RNA Binding Motif Protein 18 | Protein Coding | 27 | GC09M122237 | 0.528781116 |
| LSMEM2 | Leucine Rich Single-Pass Membrane Protein 2 | Protein Coding | 23 | GC03P050325 | 0.528781116 |
| MYG1-AS1 | MYG1 Antisense RNA 1 | RNA Gene | 9 | GC12M053299 | 0.528781116 |
| TSACC | TSSK6 Activating Cochaperone | Protein Coding | 28 | GC01P156337 | 0.528253257 |
| DET1 | DET1 Partner Of COP1 E3 Ubiquitin Ligase | Protein Coding | 29 | GC15M088527 | 0.528238773 |
| C1orf105 | Chromosome 1 Open Reading Frame 105 | Protein Coding | 28 | GC01P172419 | 0.527565897 |
| ZNF706 | Zinc Finger Protein 706 | Protein Coding | 28 | GC08M101178 | 0.527565897 |
| DENND4B | DENN Domain Containing 4B | Protein Coding | 28 | GC01M153901 | 0.527108908 |
| FLYWCH2 | FLYWCH Family Member 2 | Protein Coding | 26 | GC16P002882 | 0.526626766 |
| TSPAN17 | Tetraspanin 17 | Protein Coding | 31 | GC05P176647 | 0.525812984 |
| GGT7 | Gamma-Glutamyltransferase 7 | Protein Coding | 36 | GC20M034844 | 0.524403036 |
| ANKRD9 | Ankyrin Repeat Domain 9 | Protein Coding | 32 | GC14M102501 | 0.521763325 |
| GPR183 | G Protein-Coupled Receptor 183 | Protein Coding | 35 | GC13M099347 | 0.521687627 |
| ZBTB39 | Zinc Finger And BTB Domain Containing 39 | Protein Coding | 28 | GC12M056998 | 0.521687627 |
| ZSWIM7 | Zinc Finger SWIM-Type Containing 7 | Protein Coding | 27 | GC17M015977 | 0.521331906 |
| ENKD1 | Enkurin Domain Containing 1 | Protein Coding | 26 | GC16M067662 | 0.521331906 |
| PITPNA | Phosphatidylinositol Transfer Protein Alpha | Protein Coding | 35 | GC17M002838 | 0.520797968 |
| STAG3L3 | Stromal Antigen 3-Like 3 (Pseudogene) | Pseudogene | 17 | GC07M072969 | 0.519626617 |
| PHF21B | PHD Finger Protein 21B | Protein Coding | 33 | GC22M044881 | 0.51958698 |
| DNAJC4 | DnaJ Heat Shock Protein Family (Hsp40) Member C4 | Protein Coding | 30 | GC11P064230 | 0.51917851 |
| GPCPD1 | Glycerophosphocholine Phosphodiesterase 1 | Protein Coding | 32 | GC20M008043 | 0.518911123 |
| PRELID1 | PRELI Domain Containing 1 | Protein Coding | 32 | GC05P177305 | 0.518911123 |
| DCUN1D2 | Defective In Cullin Neddylation 1 Domain Containing 2 | Protein Coding | 30 | GC13M113455 | 0.518483698 |
| PIGC | Phosphatidylinositol Glycan Anchor Biosynthesis Class C | Protein Coding | 36 | GC01M172339 | 0.517525733 |
| OLIG3 | Oligodendrocyte Transcription Factor 3 | Protein Coding | 34 | GC06M137492 | 0.517525733 |
| ZCWPW1 | Zinc Finger CW-Type And PWWP Domain Containing 1 | Protein Coding | 30 | GC07M101748 | 0.517525733 |
| CACFD1 | Calcium Channel Flower Domain Containing 1 | Protein Coding | 29 | GC09P133459 | 0.517525733 |
| CCDC157 | Coiled-Coil Domain Containing 157 | Protein Coding | 27 | GC22P030356 | 0.517525733 |
| ZNF391 | Zinc Finger Protein 391 | Protein Coding | 26 | GC06P027374 | 0.517525733 |
| TMEM30CP | Transmembrane Protein 30C, Pseudogene | Pseudogene | 17 | GC03P100186 | 0.517525733 |
| NR1D2 | Nuclear Receptor Subfamily 1 Group D Member 2 | Protein Coding | 39 | GC03P023945 | 0.516498625 |
| TOP1MT | DNA Topoisomerase I Mitochondrial | Protein Coding | 38 | GC08M143304 | 0.516447902 |
| IL20RB | Interleukin 20 Receptor Subunit Beta | Protein Coding | 35 | GC03P136946 | 0.515598059 |
| SGCG | Sarcoglycan Gamma | Protein Coding | 38 | GC13P023160 | 0.514452517 |
| N4BP1 | NEDD4 Binding Protein 1 | Protein Coding | 32 | GC16M048538 | 0.514034748 |
| ZNHIT3 | Zinc Finger HIT-Type Containing 3 | Protein Coding | 31 | GC17P036486 | 0.514034748 |
| TMEM184A | Transmembrane Protein 184A | Protein Coding | 29 | GC07M001542 | 0.514034748 |
| THUMPD3-AS1 | THUMPD3 Antisense RNA 1 | RNA Gene | 14 | GC03M009349 | 0.514034748 |
| PCED1B-AS1 | PCED1B Antisense RNA 1 | RNA Gene | 13 | GC12M047205 | 0.514034748 |
| FAM86JP | Family With Sequence Similarity 86 Member J, Pseudogene | Pseudogene | 11 | GC03P125916 | 0.514034748 |
| MC3R | Melanocortin 3 Receptor | Protein Coding | 40 | GC20P056248 | 0.513572276 |

| | | | | | |
|---|---|---|---|---|---|
| KCNJ9 | Potassium Inwardly Rectifying Channel Subfamily J Member 9 | Protein Coding | 38 | GC01P160079 | 0.511557817 |
| SNF8 | SNF8 Subunit Of ESCRT-II | Protein Coding | 33 | GC17M048929 | 0.511082768 |
| KRT33A | Keratin 33A | Protein Coding | 31 | GC17M041349 | 0.510757804 |
| PROSER1 | Proline And Serine Rich 1 | Protein Coding | 26 | GC13M039009 | 0.510714352 |
| GPR12 | G Protein-Coupled Receptor 12 | Protein Coding | 35 | GC13M026755 | 0.510709882 |
| FAIM | Fas Apoptotic Inhibitory Molecule | Protein Coding | 34 | GC03P138608 | 0.510709882 |
| ZKSCAN7 | Zinc Finger With KRAB And SCAN Domains 7 | Protein Coding | 29 | GC03P044556 | 0.510709882 |
| RASL11A | RAS Like Family 11 Member A | Protein Coding | 27 | GC13P027270 | 0.509558499 |
| ATP5F1EP2 | ATP Synthase F1 Subunit Epsilon Pseudogene 2 | Pseudogene | 14 | GC13P027946 | 0.509266853 |
| DHRS7 | Dehydrogenase/Reductase 7 | Protein Coding | 32 | GC14M060144 | 0.509214044 |
| ZC3HAV1L | Zinc Finger CCCH-Type Containing, Antiviral 1 Like | Protein Coding | 27 | GC07M139025 | 0.509214044 |
| DTX2P1-UPK3BP1-PMS2P11 | DTX2P1-UPK3BP1-PMS2P11 Readthrough, Transcribed Pseudogene | Pseudogene | 10 | GC07P076959 | 0.509214044 |
| ZNF138 | Zinc Finger Protein 138 | Protein Coding | 29 | GC07P064794 | 0.508296967 |
| NAA60 | N-Alpha-Acetyltransferase 60, NatF Catalytic Subunit | Protein Coding | 31 | GC16P003443 | 0.508230507 |
| SDR39U1 | Short Chain Dehydrogenase/Reductase Family 39U Member 1 | Protein Coding | 31 | GC14M024439 | 0.508230507 |
| SCRN3 | Secernin 3 | Protein Coding | 30 | GC02P174395 | 0.508230507 |
| LRRC37A3 | Leucine Rich Repeat Containing 37 Member A3 | Protein Coding | 27 | GC17M064854 | 0.508230507 |
| DENND10 | DENN Domain Containing 10 | Protein Coding | 23 | GC10P119105 | 0.508230507 |
| BNIP2 | BCL2 Interacting Protein 2 | Protein Coding | 37 | GC15M059659 | 0.506265402 |
| GATC | Glutamyl-TRNA Amidotransferase Subunit C | Protein Coding | 34 | GC12P120446 | 0.506265402 |
| DTWD2 | DTW Domain Containing 2 | Protein Coding | 28 | GC05M118836 | 0.506265402 |
| C1orf198 | Chromosome 1 Open Reading Frame 198 | Protein Coding | 27 | GC01M230837 | 0.506265402 |
| ABHD14A-ACY1 | ABHD14A-ACY1 Readthrough | Protein Coding | 17 | GC03P051974 | 0.506265402 |
| LINC00708 | Long Intergenic Non-Protein Coding RNA 708 | RNA Gene | 12 | GC10M008259 | 0.506253183 |
| FAM120B | Family With Sequence Similarity 120B | Protein Coding | 32 | GC06P170290 | 0.506129622 |
| FBXL13 | F-Box And Leucine Rich Repeat Protein 13 | Protein Coding | 32 | GC07M103165 | 0.506129622 |
| UBXN8 | UBX Domain Protein 8 | Protein Coding | 28 | GC08P030723 | 0.506129622 |
| CCDC17 | Coiled-Coil Domain Containing 17 | Protein Coding | 27 | GC01M045620 | 0.506129622 |
| MUCL3 | Mucin Like 3 | Protein Coding | 27 | GC06P081941 | 0.506129622 |
| H2BC20P | H2B Clustered Histone 20, Pseudogene | Pseudogene | 14 | GC01M151583 | 0.506129622 |
| ZSCAN16-AS1 | ZSCAN16 Antisense RNA 1 | RNA Gene | 13 | GC06M063346 | 0.506129622 |
| LINC02183 | Long Intergenic Non-Protein Coding RNA 2183 | RNA Gene | 9 | GC16M054542 | 0.506129622 |
| COG6 | Component Of Oligomeric Golgi Complex 6 | Protein Coding | 35 | GC13P039655 | 0.505914867 |
| HINT3 | Histidine Triad Nucleotide Binding Protein 3 | Protein Coding | 28 | GC06P125956 | 0.504693925 |
| RPS6KA2-AS1 | RPS6KA2 Antisense RNA 1 | RNA Gene | 13 | GC06P166903 | 0.504693925 |
| DYRK3 | Dual Specificity Tyrosine Phosphorylation Regulated Kinase 3 | Protein Coding | 37 | GC01P206636 | 0.504322708 |
| KBTBD7 | Kelch Repeat And BTB Domain Containing 7 | Protein Coding | 32 | GC13M041189 | 0.504260659 |
| MIR569 | MicroRNA 569 | RNA Gene | 13 | GC03M171106 | 0.504194379 |
| CCDC186 | Coiled-Coil Domain Containing 186 | Protein Coding | 25 | GC10M114120 | 0.50387466 |
| ATP6V1E2 | ATPase H+ Transporting V1 Subunit E2 | Protein Coding | 34 | GC02M046489 | 0.50386709 |
| HEPHL1 | Hephaestin Like 1 | Protein Coding | 34 | GC11P094021 | 0.50386709 |
| GPR157 | G Protein-Coupled Receptor 157 | Protein Coding | 31 | GC01M009100 | 0.50386709 |
| PLAC9 | Placenta Associated 9 | Protein Coding | 27 | GC10P080131 | 0.50386709 |
| C6orf201 | Chromosome 6 Open Reading Frame 201 | Pseudogene | 25 | GC06P004079 | 0.50386709 |
| SAMD8 | Sterile Alpha Motif Domain Containing 8 | Protein Coding | 34 | GC10P075099 | 0.503035665 |
| GANC | Glucosidase Alpha, Neutral C | Protein Coding | 32 | GC15P042273 | 0.503035665 |
| TMEM38A | Transmembrane Protein 38A | Protein Coding | 32 | GC19P016661 | 0.503035665 |
| MANSC1 | MANSC Domain Containing 1 | Protein Coding | 31 | GC12M012326 | 0.503035665 |
| LRP5L | LDL Receptor Related Protein 5 Like | Pseudogene | 23 | GC22M025351 | 0.503035665 |
| OR10G6 | Olfactory Receptor Family 10 Subfamily G Member 6 | Protein Coding | 19 | GC11M123994 | 0.503035665 |
| TIMM8B | Translocase Of Inner Mitochondrial Membrane 8 Homolog B | Protein Coding | 34 | GC11M112084 | 0.502617419 |
| FIZ1 | FLT3 Interacting Zinc Finger 1 | Protein Coding | 30 | GC19M055591 | 0.502617419 |
| VTRNA1-2 | Vault RNA 1-2 | RNA Gene | 11 | GC05P146093 | 0.501712799 |
| VTRNA1-3 | Vault RNA 1-3 | RNA Gene | 11 | GC05P146094 | 0.501712799 |
| ANO4 | Anoctamin 4 | Protein Coding | 31 | GC12P100717 | 0.501685679 |
| CLGN | Calmegin | Protein Coding | 33 | GC04M140388 | 0.501107216 |
| COMMD10 | COMM Domain Containing 10 | Protein Coding | 29 | GC05P116084 | 0.498421609 |
| VPS8 | VPS8 Subunit Of CORVET Complex | Protein Coding | 32 | GC03P184812 | 0.497918189 |
| HERC1 | HECT And RLD Domain Containing E3 Ubiquitin Protein Ligase Family Member 1 | Protein Coding | 37 | GC15M063608 | 0.496491313 |
| MGST3 | Microsomal Glutathione S-Transferase 3 | Protein Coding | 36 | GC01P165632 | 0.495747715 |
| MRPL53 | Mitochondrial Ribosomal Protein L53 | Protein Coding | 28 | GC02M074471 | 0.495747715 |
| ZCCHC7 | Zinc Finger CCHC-Type Containing 7 | Protein Coding | 32 | GC09P037120 | 0.495320261 |
| CENPI | Centromere Protein I | Protein Coding | 31 | GC0XP101098 | 0.493335158 |
| SLC24A2 | Solute Carrier Family 24 Member 2 | Protein Coding | 36 | GC09M019507 | 0.492611706 |
| ASB13 | Ankyrin Repeat And SOCS Box Containing 13 | Protein Coding | 34 | GC10M005639 | 0.492611706 |
| GPR61 | G Protein-Coupled Receptor 61 | Protein Coding | 32 | GC01P109539 | 0.492611706 |
| NAT9 | N-Acetyltransferase 9 (Putative) | Protein Coding | 32 | GC17M074770 | 0.492611706 |
| DUSP18 | Dual Specificity Phosphatase 18 | Protein Coding | 31 | GC22M030652 | 0.492611706 |
| PHTF1 | Putative Homeodomain Transcription Factor 1 | Protein Coding | 31 | GC01M113696 | 0.492611706 |
| ZNF490 | Zinc Finger Protein 490 | Protein Coding | 31 | GC19M012577 | 0.492611706 |
| C6orf89 | Chromosome 6 Open Reading Frame 89 | Protein Coding | 30 | GC06P080538 | 0.492611706 |
| SHISA4 | Shisa Family Member 4 | Protein Coding | 30 | GC01P201888 | 0.492611706 |
| TMEM69 | Transmembrane Protein 69 | Protein Coding | 28 | GC01P045687 | 0.492611706 |
| ZNF564 | Zinc Finger Protein 564 | Protein Coding | 28 | GC19M012536 | 0.492611706 |
| ARL16 | ADP Ribosylation Factor Like GTPase 16 | Protein Coding | 27 | GC17M081681 | 0.492611706 |
| OR14J1 | Olfactory Receptor Family 14 Subfamily J Member 1 | Protein Coding | 27 | GC06P029301 | 0.492611706 |
| ZSCAN23 | Zinc Finger And SCAN Domain Containing 23 | Protein Coding | 26 | GC06M028425 | 0.492611706 |
| KRTAP5-6 | Keratin Associated Protein 5-6 | Protein Coding | 25 | GC11P001726 | 0.492611706 |
| MBLAC1 | Metallo-Beta-Lactamase Domain Containing 1 | Protein Coding | 24 | GC07P100126 | 0.492611706 |
| C22orf46 | Chromosome 22 Putative Open Reading Frame 46 | Pseudogene | 23 | GC22P041688 | 0.492611706 |
| KRTAP4-7 | Keratin Associated Protein 4-7 | Protein Coding | 23 | GC17P041084 | 0.492611706 |
| NKAPD1 | NKAP Domain Containing 1 | Protein Coding | 23 | GC11P112075 | 0.492611706 |
| LINC00862 | Long Intergenic Non-Protein Coding RNA 862 | RNA Gene | 17 | GC01M200253 | 0.492611706 |
| OR2B8P | Olfactory Receptor Family 2 Subfamily B Member 8 Pseudogene | Protein Coding | 16 | GC06M028053 | 0.492611706 |
| SERTAD4-AS1 | SERTAD4 Antisense RNA 1 | RNA Gene | 16 | GC01M210231 | 0.492611706 |
| FAM99B | Family With Sequence Similarity 99 Member B | RNA Gene | 14 | GC11M003005 | 0.492611706 |
| TMEM225B | Transmembrane Protein 225B | Protein Coding | 14 | GC07P099572 | 0.492611706 |
| INMT-MINDY4 | INMT-MINDY4 Readthrough (NMD Candidate) | RNA Gene | 13 | GC07P030752 | 0.492611706 |
| WAKMAR2 | Wound And Keratinocyte Migration Associated LncRNA 2 | RNA Gene | 13 | GC06M137820 | 0.492611706 |
| ZKSCAN8P1 | ZKSCAN8 Pseudogene 1 | Pseudogene | 13 | GC06P083464 | 0.492611706 |
| HORMAD2-AS1 | HORMAD2 And MTMR3 Antisense RNA 1 | RNA Gene | 12 | GC22M030008 | 0.492611706 |
| LINC01170 | Long Intergenic Non-Protein Coding RNA 1170 | RNA Gene | 12 | GC05M124059 | 0.492611706 |
| LINC01924 | Long Intergenic Non-Protein Coding RNA 1924 | RNA Gene | 11 | GC18P064041 | 0.492611706 |
| MTCO2P1 | MT-CO2 Pseudogene 1 | Pseudogene | 7 | GC20M060414 | 0.492611706 |
| FAM226B | Family With Sequence Similarity 226 Member B | RNA Gene | 13 | GC0XP072817 | 0.491857111 |

| | | | | | |
|---|---|---|---|---|---|
| TEX28P2 | TEX28 Pseudogene 2 | Pseudogene | 8 | GC0XM154159 | 0.491857111 |
| RAB40A | RAB40A, Member RAS Oncogene Family | Protein Coding | 31 | GC0XM103498 | 0.49057436 |
| PGS1 | Phosphatidylglycerophosphate Synthase 1 | Protein Coding | 32 | GC17P078378 | 0.489646494 |
| C8orf82 | Chromosome 8 Open Reading Frame 82 | Protein Coding | 23 | GC08M144525 | 0.488841176 |
| SLC41A3 | Solute Carrier Family 41 Member 3 | Protein Coding | 30 | GC03M126006 | 0.488450587 |
| UBE2H | Ubiquitin Conjugating Enzyme E2 H | Protein Coding | 37 | GC07M129830 | 0.487592876 |
| CARF | Calcium Responsive Transcription Factor | Protein Coding | 30 | GC02P202912 | 0.487550914 |
| LSM6 | LSM6 Homolog, U6 Small Nuclear RNA And MRNA Degradation Associated | Protein Coding | 32 | GC04P146177 | 0.487546444 |
| CPZ | Carboxypeptidase Z | Protein Coding | 35 | GC04P008583 | 0.487454861 |
| KLRG1 | Killer Cell Lectin Like Receptor G1 | Protein Coding | 34 | GC12P008950 | 0.487445861 |
| DPPA3 | Developmental Pluripotency Associated 3 | Protein Coding | 30 | GC12P019867 | 0.486699373 |
| ISLR2 | Immunoglobulin Superfamily Containing Leucine Rich Repeat 2 | Protein Coding | 32 | GC15P074100 | 0.486250252 |
| C3orf18 | Chromosome 3 Open Reading Frame 18 | Protein Coding | 29 | GC03M051117 | 0.486250252 |
| NT5C3B | 5'-Nucleotidase, Cytosolic IIIB | Protein Coding | 28 | GC17M041825 | 0.486250252 |
| REP15 | RAB15 Effector Protein | Protein Coding | 26 | GC12P027696 | 0.486250252 |
| RNF224 | Ring Finger Protein 224 | Protein Coding | 21 | GC09P137227 | 0.486250252 |
| BAK1P1 | BCL2 Antagonist/Killer 1 Pseudogene 1 | Pseudogene | 9 | GC20M032688 | 0.486250252 |
| VDAC1P1 | Voltage Dependent Anion Channel 1 Pseudogene 1 | Pseudogene | 7 | GC0XP080929 | 0.486250252 |
| TMEM134 | Transmembrane Protein 134 | Protein Coding | 28 | GC11M067461 | 0.485843807 |
| AQP10 | Aquaporin 10 | Protein Coding | 35 | GC01P154321 | 0.485722423 |
| TRMT61A | TRNA Methyltransferase 61A | Protein Coding | 30 | GC14P103529 | 0.484883308 |
| CADPS2 | Calcium Dependent Secretion Activator 2 | Protein Coding | 35 | GC07M122318 | 0.482751429 |
| DNAJA4 | DnaJ Heat Shock Protein Family (Hsp40) Member A4 | Protein Coding | 31 | GC15P078264 | 0.481530488 |
| HDGFL3 | HDGF Like 3 | Protein Coding | 27 | GC15M089162 | 0.481530488 |
| LINC00839 | Long Intergenic Non-Protein Coding RNA 839 | RNA Gene | 16 | GC10P042475 | 0.481530488 |
| TSPOAP1 | TSPO Associated Protein 1 | Protein Coding | 28 | GC17M058302 | 0.480681211 |
| GATD1 | Glutamine Amidotransferase Class 1 Domain Containing 1 | Protein Coding | 23 | GC11M000767 | 0.480681211 |
| ANKFY1 | Ankyrin Repeat And FYVE Domain Containing 1 | Protein Coding | 34 | GC17M004163 | 0.479203731 |
| GPR119 | G Protein-Coupled Receptor 119 | Protein Coding | 34 | GC0XM130384 | 0.479203731 |
| METTL7B | Methyltransferase Like 7B | Protein Coding | 31 | GC12P055681 | 0.477658153 |
| CCDC185 | Coiled-Coil Domain Containing 185 | Protein Coding | 23 | GC01P223393 | 0.477582008 |
| GOLGA8A | Golgin A8 Family Member A | Protein Coding | 29 | GC15M035222 | 0.477228343 |
| CENPK | Centromere Protein K | Protein Coding | 32 | GC05M065517 | 0.476954937 |
| PIK3R6 | Phosphoinositide-3-Kinase Regulatory Subunit 6 | Protein Coding | 27 | GC17M008802 | 0.476954937 |
| DPYSL4 | Dihydropyrimidinase Like 4 | Protein Coding | 34 | GC10P132185 | 0.475258172 |
| GPR88 | G Protein-Coupled Receptor 88 | Protein Coding | 33 | GC01P100538 | 0.474994868 |
| GK5 | Glycerol Kinase 5 | Protein Coding | 30 | GC03M142158 | 0.474994868 |
| ZNF19 | Zinc Finger Protein 19 | Protein Coding | 30 | GC16M071593 | 0.474994868 |
| ZNF791 | Zinc Finger Protein 791 | Protein Coding | 30 | GC19P012610 | 0.474994868 |
| KLHDC7A | Kelch Domain Containing 7A | Protein Coding | 28 | GC01P018480 | 0.474994868 |
| TMEM167A | Transmembrane Protein 167A | Protein Coding | 28 | GC05M083052 | 0.474994868 |
| SELENOT | Selenoprotein T | Protein Coding | 27 | GC03P150603 | 0.474994868 |
| ZNF443 | Zinc Finger Protein 443 | Protein Coding | 27 | GC19M012441 | 0.474994868 |
| SPINK8 | Serine Peptidase Inhibitor Kazal Type 8 (Putative) | Protein Coding | 26 | GC03M048306 | 0.474994868 |
| ATP5MGL | ATP Synthase Membrane Subunit G Like | Protein Coding | 25 | GC22M056127 | 0.474994868 |
| POM121L2 | POM121 Transmembrane Nucleoporin Like 2 | Protein Coding | 25 | GC06M027285 | 0.474994868 |
| PRSS45P | Serine Protease 45, Pseudogene | Pseudogene | 22 | GC03M046746 | 0.474994868 |
| HOATZ | HOATZ Cilia And Flagella Associated Protein | Protein Coding | 21 | GC11P111708 | 0.474994868 |
| BLACE | B Cell Acute Lymphoblastic Leukemia Expressed | RNA Gene | 18 | GC07M155356 | 0.474994868 |
| OR2J1 | Olfactory Receptor Family 2 Subfamily J Member 1 | Protein Coding | 18 | GC06P080274 | 0.474994868 |
| CCDC163 | Coiled-Coil Domain Containing 163 | Protein Coding | 15 | GC01M045495 | 0.474994868 |
| FOXO3B | Forkhead Box O3B | Protein Coding | 15 | GC17M025206 | 0.474994868 |
| LINC01556 | Long Intergenic Non-Protein Coding RNA 1556 | RNA Gene | 15 | GC06P028943 | 0.474994868 |
| NUTM2B-AS1 | NUTM2B Antisense RNA 1 | RNA Gene | 15 | GC10M081655 | 0.474994868 |
| MIR3199-2 | MicroRNA 3199-2 | RNA Gene | 14 | GC22P034870 | 0.474994868 |
| MIR640 | MicroRNA 640 | RNA Gene | 13 | GC19P019435 | 0.474994868 |
| MIR646HG | MIR646 Host Gene | RNA Gene | 13 | GC20P060088 | 0.474994868 |
| P3R3URF | PIK3R3 Upstream Open Reading Frame | Protein Coding | 12 | GC01M046241 | 0.474994868 |
| LINC02242 | Long Intergenic Non-Protein Coding RNA 2242 | RNA Gene | 10 | GC05M067614 | 0.474994868 |
| LINC02428 | Long Intergenic Non-Protein Coding RNA 2428 | RNA Gene | 10 | GC04P103224 | 0.474994868 |
| MIR3921 | MicroRNA 3921 | RNA Gene | 10 | GC03M099964 | 0.474994868 |
| CRYZP1 | Crystallin Zeta Pseudogene 1 | Pseudogene | 9 | GC07M103087 | 0.474994868 |
| RANP1 | RAN Pseudogene 1 | Pseudogene | 9 | GC06P080325 | 0.474994868 |
| RPL34P26 | Ribosomal Protein L34 Pseudogene 26 | Pseudogene | 7 | GC13M050361 | 0.474994868 |
| RPSAP18 | Ribosomal Protein SA Pseudogene 18 | Pseudogene | 7 | GC01M160266 | 0.474994868 |
| ENSG00000284895 | Novel Protein | Protein Coding | 6 | GC01M041585 | 0.474994868 |
| RPL35AP35 | Ribosomal Protein L35a Pseudogene 35 | Pseudogene | 6 | GC17M029340 | 0.474994868 |
| ENSG00000273088 | Novel Protein | Protein Coding | 5 | GC01M155169 | 0.474994868 |
| ENSG00000288644 | Novel Protein | Protein Coding | 5 | GC01P203803 | 0.474994868 |
| ATP6V1F | ATPase H+ Transporting V1 Subunit F | Protein Coding | 36 | GC07P128862 | 0.472334057 |
| ALDH3B2 | Aldehyde Dehydrogenase 3 Family Member B2 | Protein Coding | 35 | GC11M067662 | 0.472334057 |
| KLHL23 | Kelch Like Family Member 23 | Protein Coding | 29 | GC02P169710 | 0.472334057 |
| ZBTB41 | Zinc Finger And BTB Domain Containing 41 | Protein Coding | 28 | GC01M197153 | 0.472334057 |
| INIP | INTS3 And NABP Interacting Protein | Protein Coding | 27 | GC09M112683 | 0.472334057 |
| ARL14EPL | ADP Ribosylation Factor Like GTPase 14 Effector Protein Like | Protein Coding | 20 | GC05P116051 | 0.472334057 |
| MYO15B | Myosin XVB | Protein Coding | 19 | GC17P075588 | 0.472334057 |
| LOXHD1 | Lipoxygenase Homology PLAT Domains 1 | Protein Coding | 32 | GC18M046476 | 0.471032143 |
| TGDS | TDP-Glucose 4,6-Dehydratase | Protein Coding | 34 | GC13M094574 | 0.470587671 |
| CLYBL | Citramalyl-CoA Lyase | Protein Coding | 34 | GC13P099606 | 0.470126718 |
| SEMG2 | Semenogelin 2 | Protein Coding | 31 | GC20P045221 | 0.469559252 |
| WSCD1 | WSC Domain Containing 1 | Protein Coding | 31 | GC17P006057 | 0.469061822 |
| CHID1 | Chitinase Domain Containing 1 | Protein Coding | 32 | GC11M000867 | 0.468693644 |
| ZNF382 | Zinc Finger Protein 382 | Protein Coding | 31 | GC19P063480 | 0.468693644 |
| CCDC174 | Coiled-Coil Domain Containing 174 | Protein Coding | 27 | GC03P014657 | 0.467404902 |
| OR3A3 | Olfactory Receptor Family 3 Subfamily A Member 3 | Protein Coding | 31 | GC17P004233 | 0.467371762 |
| MIR518E | MicroRNA 518e | RNA Gene | 17 | GC19P053729 | 0.467371762 |
| LINC01432 | Long Intergenic Non-Protein Coding RNA 1432 | RNA Gene | 11 | GC20P022054 | 0.467371762 |
| CLIP4 | CAP-Gly Domain Containing Linker Protein Family Member 4 | Protein Coding | 30 | GC02P029097 | 0.465766132 |
| MFSD5 | Major Facilitator Superfamily Domain Containing 5 | Protein Coding | 28 | GC12P053251 | 0.465766132 |
| PCDHGB5 | Protocadherin Gamma Subfamily B, 5 | Protein Coding | 27 | GC05P145841 | 0.465766132 |
| TMEM117 | Transmembrane Protein 117 | Protein Coding | 27 | GC12P043795 | 0.465766132 |
| CA14 | Carbonic Anhydrase 14 | Protein Coding | 39 | GC01P150258 | 0.465151966 |
| BET1L | Bet1 Golgi Vesicular Membrane Trafficking Protein Like | Protein Coding | 32 | GC11M002844 | 0.463257045 |
| ZFHX2 | Zinc Finger Homeobox 2 | Protein Coding | 30 | GC14M023520 | 0.463257045 |
| RIC8B | RIC8 Guanine Nucleotide Exchange Factor B | Protein Coding | 28 | GC12P106774 | 0.463257045 |

| | | | | | |
|---|---|---|---|---|---|
| TBC1D3H | TBC1 Domain Family Member 3H | Protein Coding | 19 | GC17M041251 | 0.463257045 |
| ZFYVE27 | Zinc Finger FYVE-Type Containing 27 | Protein Coding | 32 | GC10P097739 | 0.462668061 |
| UQCR10 | Ubiquinol-Cytochrome C Reductase, Complex III Subunit X | Protein Coding | 34 | GC22P029767 | 0.461791843 |
| ZNF219 | Zinc Finger Protein 219 | Protein Coding | 31 | GC14M021090 | 0.461791843 |
| FAM156A | Family With Sequence Similarity 156 Member A | Protein Coding | 26 | GC0XM052926 | 0.461791843 |
| C13orf42 | Chromosome 13 Open Reading Frame 42 | Protein Coding | 13 | GC13M051158 | 0.460086554 |
| SHLD3 | Shieldin Complex Subunit 3 | Protein Coding | 13 | GC05P065626 | 0.459402323 |
| FOXI3 | Forkhead Box I3 | Protein Coding | 28 | GC02M088446 | 0.459231377 |
| BPESC1 | Blepharophimosis, Epicanthus Inversus And Ptosis Candidate 1 | RNA Gene | 19 | GC03P139104 | 0.459231377 |
| RNF113B | Ring Finger Protein 113B | Protein Coding | 28 | GC13M098175 | 0.458515912 |
| MIR5590 | MicroRNA 5590 | RNA Gene | 7 | GC02P134857 | 0.456664413 |
| MZT1 | Mitotic Spindle Organizing Protein 1 | Protein Coding | 29 | GC13M072708 | 0.454494715 |
| SLF1 | SMC5-SMC6 Complex Localization Factor 1 | Protein Coding | 28 | GC05P094679 | 0.454494715 |
| MIR1303 | MicroRNA 1303 | RNA Gene | 14 | GC05P154685 | 0.454494715 |
| MRPL51 | Mitochondrial Ribosomal Protein L51 | Protein Coding | 30 | GC12M006491 | 0.454370022 |
| LRRIQ3 | Leucine Rich Repeats And IQ Motif Containing 3 | Protein Coding | 27 | GC01M074026 | 0.454370022 |
| CCDC183 | Coiled-Coil Domain Containing 183 | Protein Coding | 26 | GC09P136796 | 0.454370022 |
| GTF2H2C_2 | GTF2H2 Family Member C, Copy 2 | Protein Coding | 15 | GC05Mi00965 | 0.454370022 |
| OSBPL1A | Oxysterol Binding Protein Like 1A | Protein Coding | 34 | GC18M024162 | 0.453216881 |
| DUSP15 | Dual Specificity Phosphatase 15 | Protein Coding | 33 | GC20M031848 | 0.453216881 |
| NMRAL1 | NmrA Like Redox Sensor 1 | Protein Coding | 31 | GC16M004461 | 0.453216881 |
| NRSN2-AS1 | NRSN2 Antisense RNA 1 | RNA Gene | 13 | GC20M000321 | 0.453216881 |
| SAMD12-AS1 | SAMD12 Antisense RNA 1 | RNA Gene | 13 | GC08P118621 | 0.453216881 |
| ZNF853 | Zinc Finger Protein 853 | Protein Coding | 23 | GC07P006615 | 0.452668011 |
| WIPF3 | WAS/WASL Interacting Protein Family Member 3 | Protein Coding | 28 | GC07P029842 | 0.452464581 |
| SETDB2 | SET Domain Bifurcated Histone Lysine Methyltransferase 2 | Protein Coding | 32 | GC13P049444 | 0.44967401 |
| LINC01483 | Long Intergenic Non-Protein Coding RNA 1483 | RNA Gene | 12 | GC17P069577 | 0.449490458 |
| TYW3 | TRNA-YW Synthesizing Protein 3 Homolog | Protein Coding | 32 | GC01P074733 | 0.447797269 |
| HTN1 | Histatin 1 | Protein Coding | 28 | GC04P070053 | 0.447640836 |
| PYCR3 | Pyrroline-5-Carboxylate Reductase 3 | Protein Coding | 31 | GC08M143604 | 0.447594464 |
| ASB1 | Ankyrin Repeat And SOCS Box Containing 1 | Protein Coding | 32 | GC02P238426 | 0.447083831 |
| EFCAB5 | EF-Hand Calcium Binding Domain 5 | Protein Coding | 27 | GC17P029929 | 0.446727723 |
| C10orf88 | Chromosome 10 Open Reading Frame 88 | Protein Coding | 28 | GC10M122930 | 0.445121109 |
| P2RY11 | Purinergic Receptor P2Y11 | Protein Coding | 39 | GC19P010375 | 0.444470286 |
| UNC80 | Unc-80 Homolog, NALCN Channel Complex Subunit | Protein Coding | 30 | GC02P209771 | 0.443414658 |
| POFUT2 | Protein O-Fucosyltransferase 2 | Protein Coding | 34 | GC21M045263 | 0.443191558 |
| HTRA4 | HtrA Serine Peptidase 4 | Protein Coding | 32 | GC08P038976 | 0.443109453 |
| CLCA3P | Chloride Channel Accessory 3, Pseudogene | Pseudogene | 18 | GC01P086634 | 0.443109453 |
| NAT8 | N-Acetyltransferase 8 (Putative) | Protein Coding | 34 | GC02M073640 | 0.442897379 |
| FARSA-AS1 | FARSA Antisense RNA 1 | RNA Gene | 10 | GC19P013691 | 0.441820771 |
| LINC01001 | Long Intergenic Non-Protein Coding RNA 1001 | RNA Gene | 10 | GC11M002841 | 0.441820771 |
| LINC02591 | Long Intergenic Non-Protein Coding RNA 2591 | RNA Gene | 10 | GC01P143989 | 0.441820771 |
| CRYZL2P | Crystallin Zeta Like 2, Pseudogene | Pseudogene | 9 | GC01M178006 | 0.441820771 |
| LOC100132287 | Uncharacterized LOC100132287 | RNA Gene | 8 | GC01M005372 | 0.441820771 |
| PI4K2B | Phosphatidylinositol 4-Kinase Type 2 Beta | Protein Coding | 35 | GC04P025167 | 0.440852135 |
| SNX11 | Sorting Nexin 11 | Protein Coding | 31 | GC17P048103 | 0.440852135 |
| SOGA3 | SOGA Family Member 3 | Protein Coding | 27 | GC06M127438 | 0.440852135 |
| CNTNAP3B | Contactin Associated Protein Family Member 3B | Protein Coding | 26 | GC09M041890 | 0.440852135 |
| ADGB | Androglobin | Protein Coding | 25 | GC06P146598 | 0.440852135 |
| ZNF385C | Zinc Finger Protein 385C | Protein Coding | 25 | GC17M042026 | 0.440852135 |
| C6orf163 | Chromosome 6 Open Reading Frame 163 | Protein Coding | 24 | GC06P087343 | 0.440852135 |
| CDPF1 | Cysteine Rich DPF Motif Domain Containing 1 | Protein Coding | 23 | GC22M046244 | 0.440852135 |
| CMBL | Carboxymethylenebutenolidase Homolog | Protein Coding | 34 | GC05M010275 | 0.438378155 |
| POLR3E | RNA Polymerase III Subunit E | Protein Coding | 31 | GC16P022298 | 0.438378155 |
| MIR4295 | MicroRNA 4295 | RNA Gene | 10 | GC10P112634 | 0.438378155 |
| NM | Neutrophil Migration | Genetic Locus | 5 | GC07U990060 | 0.438378155 |
| HTR3E | 5-Hydroxytryptamine Receptor 3E | Protein Coding | 35 | GC03P184098 | 0.438369036 |
| VAMP5 | Vesicle Associated Membrane Protein 5 | Protein Coding | 34 | GC02P085784 | 0.437016517 |
| LCE2B | Late Cornified Envelope 2B | Protein Coding | 25 | GC01P152686 | 0.435172558 |
| METTL25B | Methyltransferase Like 25B | Protein Coding | 23 | GC01P156731 | 0.435051143 |
| MIR570HG | MIR570 Host Gene | RNA Gene | 11 | GC03P196215 | 0.433209836 |
| ZFAND2A | Zinc Finger AN1-Type Containing 2A | Protein Coding | 31 | GC07M001600 | 0.432529092 |
| C1orf43 | Chromosome 1 Open Reading Frame 43 | Protein Coding | 29 | GC01M154206 | 0.432529092 |
| C19orf25 | Chromosome 19 Open Reading Frame 25 | Protein Coding | 27 | GC19M004879 | 0.432529092 |
| PAQR7 | Progestin And AdipoQ Receptor Family Member 7 | Protein Coding | 32 | GC01M025861 | 0.429204226 |
| APOBEC3A_B | APOBEC3A And APOBEC3B Deletion Hybrid | Protein Coding | 7 | GC22Pi00068 | 0.429204226 |
| DHRS1 | Dehydrogenase/Reductase 1 | Protein Coding | 34 | GC14M024290 | 0.429158449 |
| DHRS13 | Dehydrogenase/Reductase 13 | Protein Coding | 29 | GC17M028897 | 0.423235297 |
| ASB15 | Ankyrin Repeat And SOCS Box Containing 15 | Protein Coding | 28 | GC07P123567 | 0.423235297 |
| OR1L8 | Olfactory Receptor Family 1 Subfamily L Member 8 | Protein Coding | 27 | GC09M122567 | 0.423235297 |
| ZNF626 | Zinc Finger Protein 626 | Protein Coding | 26 | GC19M020619 | 0.423235297 |
| CCDC162P | Coiled-Coil Domain Containing 162, Pseudogene | Pseudogene | 15 | GC06P109170 | 0.423235297 |
| RTL10 | Retrotransposon Gag Like 10 | Protein Coding | 22 | GC22M019846 | 0.423188269 |
| MAD2L1BP | MAD2L1 Binding Protein | Protein Coding | 30 | GC06P043629 | 0.422817022 |
| TMEM272 | Transmembrane Protein 272 | Protein Coding | 14 | GC13M051814 | 0.422510237 |
| LOC107197952 | NUP98-HOXA9 Recombination Region | Biological Region | 2 | GC07P027686 | 0.422510237 |
| BEND6 | BEN Domain Containing 6 | Protein Coding | 30 | GC06P056954 | 0.422040015 |
| ZYG11A | Zyg-11 Family Member A, Cell Cycle Regulator | Protein Coding | 28 | GC01P052842 | 0.419260979 |
| DHX34 | DExH-Box Helicase 34 | Protein Coding | 34 | GC19P047349 | 0.418051124 |
| PGAM4 | Phosphoglycerate Mutase Family Member 4 | Protein Coding | 31 | GC0XM077967 | 0.418051124 |
| CAPS | Calcyphosine | Protein Coding | 34 | GC19P005911 | 0.41755569 |
| MTIF3 | Mitochondrial Translational Initiation Factor 3 | Protein Coding | 32 | GC13M027436 | 0.416915953 |
| CDKL4 | Cyclin Dependent Kinase Like 4 | Protein Coding | 31 | GC02M039174 | 0.416915953 |
| TIFAB | TIFA Inhibitor | Protein Coding | 25 | GC05M135461 | 0.416700512 |
| MBS2 | Moebius Syndrome 2 | Genetic Locus | 2 | GC03U990092 | 0.416700512 |
| MBS3 | Moebius Syndrome 3 | Genetic Locus | 2 | GC10U990074 | 0.416700512 |
| OMS | Otitis Media, Susceptibility To | Genetic Locus | 2 | GC00U921706 | 0.416700512 |
| ZNF568 | Zinc Finger Protein 568 | Protein Coding | 29 | GC19P036916 | 0.416412532 |
| KRTAP3-4P | Keratin Associated Protein 3-4, Pseudogene | Pseudogene | 6 | GC17M041004 | 0.415608406 |
| TRIM54 | Tripartite Motif Containing 54 | Protein Coding | 32 | GC02P027289 | 0.41400969 |
| JRKL | JRK Like | Protein Coding | 30 | GC11P096389 | 0.410697758 |
| SERGEF | Secretion Regulating Guanine Nucleotide Exchange Factor | Protein Coding | 30 | GC11M017767 | 0.410686016 |
| CHCHD5 | Coiled-Coil-Helix-Coiled-Coil-Helix Domain Containing 5 | Protein Coding | 28 | GC02P121633 | 0.410351396 |
| LOC392196 | Ubiquitin Specific Peptidase 17 Like Family Member 2 Pseudogene | Pseudogene | 6 | GC08M012127 | 0.409370184 |
| YIPF1 | Yip1 Domain Family Member 1 | Protein Coding | 32 | GC01M053851 | 0.40733552 |

| | | | | | | |
|---|---|---|---|---|---|---|
| THUMPD3 | THUMP Domain Containing 3 | Protein Coding | 31 | GC03P009362 | 0.40733552 |
| LINS1 | Lines Homolog 1 | Protein Coding | 30 | GC15M116021 | 0.40733552 |
| BMERB1 | BMERB Domain Containing 1 | Protein Coding | 26 | GC16P015434 | 0.40733552 |
| SPDYE16 | Speedy/RINGO Cell Cycle Regulator Family Member E16 | Protein Coding | 14 | GC07M076734 | 0.40733552 |
| TTC9-DT | TTC9 Divergent Transcript | RNA Gene | 11 | GC14M070631 | 0.406278729 |
| TM7SF2 | Transmembrane 7 Superfamily Member 2 | Protein Coding | 35 | GC11P065111 | 0.404533237 |
| IQCK | IQ Motif Containing K | Protein Coding | 30 | GC16P019727 | 0.404533237 |
| ANKRD23 | Ankyrin Repeat Domain 23 | Protein Coding | 29 | GC02M098027 | 0.404533237 |
| THSD1 | Thrombospondin Type 1 Domain Containing 1 | Protein Coding | 36 | GC13M052377 | 0.403449595 |
| FCHO2 | FCH And Mu Domain Containing Endocytic Adaptor 2 | Protein Coding | 32 | GC05P072955 | 0.403449595 |
| ZFAND2B | Zinc Finger AN1-Type Containing 2B | Protein Coding | 31 | GC02P219195 | 0.403449595 |
| GPRIN2 | G Protein Regulated Inducer Of Neurite Outgrowth 2 | Protein Coding | 30 | GC10M046543 | 0.403449595 |
| GNG12-AS1 | GNG12, DIRAS3 And WLS Antisense RNA 1 | RNA Gene | 14 | GC01P067832 | 0.400857627 |
| LOC106804547 | Origin Of Replication Adjacent To Lamin B2 | Biological Region | 2 | GC19P002426 | 0.400624275 |
| MKRN2 | Makorin Ring Finger Protein 2 | Protein Coding | 35 | GC03P012561 | 0.400024801 |
| ZNF629 | Zinc Finger Protein 629 | Protein Coding | 28 | GC16M036561 | 0.400024801 |
| INSM2 | INSM Transcriptional Repressor 2 | Protein Coding | 31 | GC14P035534 | 0.397413015 |
| ZNF12 | Zinc Finger Protein 12 | Protein Coding | 32 | GC07M008042 | 0.397295356 |
| PRR5L | Proline Rich 5 Like | Protein Coding | 30 | GC11P036297 | 0.397295356 |
| SANBR | SANT And BTB Domain Regulator Of CSR | Protein Coding | 24 | GC02P061068 | 0.397295356 |
| RPL10P17 | Ribosomal Protein L10 Pseudogene 17 | Pseudogene | 5 | GC01M012220 | 0.397295356 |
| ALG5 | ALG5 Dolichyl-Phosphate Beta-Glucosyltransferase | Protein Coding | 34 | GC13M036949 | 0.396152496 |
| POGLUT2 | Protein O-Glucosyltransferase 2 | Protein Coding | 26 | GC13M102785 | 0.396152496 |
| NME2P1 | NME2 Pseudogene 1 | Pseudogene | 18 | GC12P120282 | 0.396152496 |
| USP17L22 | Ubiquitin Specific Peptidase 17 Like Family Member 22 | Protein Coding | 16 | GC04P009272 | 0.394640535 |
| MEA1 | Male-Enhanced Antigen 1 | Protein Coding | 30 | GC06M042980 | 0.393752486 |
| YBEY | YbeY Metalloendoribonuclease | Protein Coding | 30 | GC21P046286 | 0.393752486 |
| ZBED4 | Zinc Finger BED-Type Containing 4 | Protein Coding | 30 | GC22P049853 | 0.393752486 |
| SLITRK5 | SLIT And NTRK Like Family Member 5 | Protein Coding | 35 | GC13P087671 | 0.390156776 |
| FNDC3A | Fibronectin Type III Domain Containing 3A | Protein Coding | 31 | GC13P048975 | 0.38838309 |
| CHAC2 | ChaC Glutathione Specific Gamma-Glutamylcyclotransferase 2 | Protein Coding | 28 | GC02P053767 | 0.38838309 |
| SPATA5L1 | Spermatogenesis Associated 5 Like 1 | Protein Coding | 31 | GC15P045402 | 0.388100803 |
| DDI2 | DNA Damage Inducible 1 Homolog 2 | Protein Coding | 31 | GC01P015617 | 0.387187958 |
| FCRL6 | Fc Receptor Like 6 | Protein Coding | 31 | GC01P159800 | 0.387187958 |
| GDPD3 | Glycerophosphodiester Phosphodiesterase Domain Containing 3 | Protein Coding | 30 | GC16M036470 | 0.387187958 |
| DENND3 | DENN Domain Containing 3 | Protein Coding | 34 | GC08P141117 | 0.386916399 |
| COQ7 | Coenzyme Q7, Hydroxylase | Protein Coding | 38 | GC16P019067 | 0.385899246 |
| CTDNEP1 | CTD Nuclear Envelope Phosphatase 1 | Protein Coding | 31 | GC17M007243 | 0.385899246 |
| MFSD11 | Major Facilitator Superfamily Domain Containing 11 | Protein Coding | 31 | GC17P076735 | 0.385899246 |
| SLC10A3 | Solute Carrier Family 10 Member 3 | Protein Coding | 30 | GC0XM154488 | 0.385899246 |
| SNX22 | Sorting Nexin 22 | Protein Coding | 29 | GC15P064151 | 0.385899246 |
| ARMCX6 | Armadillo Repeat Containing X-Linked 6 | Protein Coding | 28 | GC0XM101616 | 0.385899246 |
| R3HDM4 | R3H Domain Containing 4 | Protein Coding | 27 | GC19M000896 | 0.385899246 |
| TM2D2 | TM2 Domain Containing 2 | Protein Coding | 27 | GC08M038988 | 0.385899246 |
| ACP7 | Acid Phosphatase 7, Tartrate Resistant (Putative) | Protein Coding | 25 | GC19P063550 | 0.385899246 |
| INSYN2A | Inhibitory Synaptic Factor 2A | Protein Coding | 23 | GC10M127137 | 0.385899246 |
| ISY1-RAB43 | ISY1-RAB43 Readthrough | Protein Coding | 21 | GC03M129087 | 0.385899246 |
| PEDS1-UBE2V1 | PEDS1-UBE2V1 Readthrough | Protein Coding | 21 | GC20M050085 | 0.385899246 |
| SNHG21 | Small Nucleolar RNA Host Gene 21 | RNA Gene | 13 | GC15P082750 | 0.385899246 |
| CSPG4P10 | Chondroitin Sulfate Proteoglycan 4 Pseudogene 10 | Pseudogene | 7 | GC15P114992 | 0.385899246 |
| CKS1BP3 | CDC28 Protein Kinase Regulatory Subunit 1B Pseudogene 3 | Pseudogene | 6 | GC05M062511 | 0.385899246 |
| ZMYM5 | Zinc Finger MYM-Type Containing 5 | Protein Coding | 30 | GC13M019823 | 0.383490145 |
| FAM180A | Family With Sequence Similarity 180 Member A | Protein Coding | 26 | GC07M135728 | 0.383266956 |
| BRSK2 | BR Serine/Threonine Kinase 2 | Protein Coding | 38 | GC11P001389 | 0.380286157 |
| NAXD | NAD(P)HX Dehydratase | Protein Coding | 32 | GC13P110682 | 0.380286157 |
| GCC1 | GRIP And Coiled-Coil Domain Containing 1 | Protein Coding | 32 | GC07M127580 | 0.380035937 |
| CD302 | CD302 Molecule | Protein Coding | 31 | GC02M159769 | 0.380035937 |
| SKOR2 | SKI Family Transcriptional Corepressor 2 | Protein Coding | 28 | GC18M047206 | 0.379509151 |
| TEKT2 | Tektin 2 | Protein Coding | 33 | GC01P036084 | 0.376030564 |
| RPL41 | Ribosomal Protein L41 | Protein Coding | 26 | GC12P057173 | 0.374172091 |
| RABEPK | Rab9 Effector Protein With Kelch Motifs | Protein Coding | 35 | GC09P125200 | 0.372989029 |
| TBPL2 | TATA-Box Binding Protein Like 2 | Protein Coding | 30 | GC14M055443 | 0.372989029 |
| SS18L2 | SS18 Like 2 | Protein Coding | 28 | GC03P042581 | 0.372989029 |
| TMEM52B | Transmembrane Protein 52B | Protein Coding | 25 | GC12P010170 | 0.372989029 |
| RFX3 | Regulatory Factor X3 | Protein Coding | 36 | GC09M003214 | 0.37238282 |
| VTRNA3-1P | Vault RNA 3-1, Pseudogene | Pseudogene | 5 | GC0XP054438 | 0.372381389 |
| RWDD2B | RWD Domain Containing 2B | Protein Coding | 30 | GC21M029004 | 0.370958924 |
| USP17L3 | Ubiquitin Specific Peptidase 17 Like Family Member 3 | Protein Coding | 23 | GC08M007976 | 0.369964778 |
| USP17L5 | Ubiquitin Specific Peptidase 17 Like Family Member 5 | Protein Coding | 21 | GC04P009342 | 0.369964778 |
| USP17L4 | Ubiquitin Specific Peptidase 17 Like Family Member 4 | Protein Coding | 20 | GC08P007337 | 0.369964778 |
| USP17L1 | Ubiquitin Specific Peptidase 17 Like Family Member 1 | Protein Coding | 19 | GC08P007482 | 0.369964778 |
| USP17L7 | Ubiquitin Specific Peptidase 17 Like Family Member 7 | Protein Coding | 19 | GC08M012132 | 0.369964778 |
| USP17L8 | Ubiquitin Specific Peptidase 17 Like Family Member 8 | Protein Coding | 19 | GC08M007971 | 0.369964778 |
| USP17L6P | Ubiquitin Specific Peptidase 17 Like Family Member 6, Pseudogene | Pseudogene | 14 | GC04P009371 | 0.369964778 |
| STARD5 | StAR Related Lipid Transfer Domain Containing 5 | Protein Coding | 34 | GC15M081309 | 0.368034869 |
| SPDYE2 | Speedy/RINGO Cell Cycle Regulator Family Member E2 | Protein Coding | 27 | GC07P102551 | 0.367379874 |
| SERPINB8 | Serpin Family B Member 8 | Protein Coding | 38 | GC18P063969 | 0.365219653 |
| PLD4 | Phospholipase D Family Member 4 | Protein Coding | 35 | GC14P104924 | 0.365219653 |
| GRTP1 | Growth Hormone Regulated TBC Protein 1 | Protein Coding | 31 | GC13M113324 | 0.365219653 |
| KAT14 | Lysine Acetyltransferase 14 | Protein Coding | 31 | GC20P018138 | 0.365219653 |
| UBL5 | Ubiquitin Like 5 | Protein Coding | 31 | GC19P009827 | 0.365219653 |
| TRAPPC13 | Trafficking Protein Particle Complex Subunit 13 | Protein Coding | 27 | GC05P065625 | 0.365219653 |
| RAMAC | RNA Guanine-7 Methyltransferase Activating Subunit | Protein Coding | 25 | GC15P115035 | 0.365219653 |
| RAB6C-AS1 | RAB6C Antisense RNA 1 | RNA Gene | 11 | GC02M129966 | 0.365219653 |
| MEDAG | Mesenteric Estrogen Dependent Adipogenesis | Protein Coding | 27 | GC13P030906 | 0.364613473 |
| LIPT2 | Lipoyl(Octanoyl) Transferase 2 | Protein Coding | 31 | GC11M087600 | 0.36290729 |
| MED31 | Mediator Complex Subunit 31 | Protein Coding | 30 | GC17M006643 | 0.36266765 |
| USP17L24 | Ubiquitin Specific Peptidase 17 Like Family Member 24 | Protein Coding | 19 | GC04P009327 | 0.36266765 |
| USP17L25 | Ubiquitin Specific Peptidase 17 Like Family Member 25 | Protein Coding | 18 | GC04P009333 | 0.36266765 |
| USP17L28 | Ubiquitin Specific Peptidase 17 Like Family Member 28 | Protein Coding | 18 | GC04P009352 | 0.36266765 |
| USP17L29 | Ubiquitin Specific Peptidase 17 Like Family Member 29 | Protein Coding | 18 | GC04P009357 | 0.36266765 |
| USP17L26 | Ubiquitin Specific Peptidase 17 Like Family Member 26 | Protein Coding | 17 | GC04P009338 | 0.36266765 |
| USP17L27 | Ubiquitin Specific Peptidase 17 Like Family Member 27 | Protein Coding | 17 | GC04P009347 | 0.36266765 |
| USP17L30 | Ubiquitin Specific Peptidase 17 Like Family Member 30 | Protein Coding | 17 | GC04P009372 | 0.36266765 |
| USP17L12 | Ubiquitin Specific Peptidase 17 Like Family Member 12 | Protein Coding | 16 | GC04P009220 | 0.36266765 |

| | | | | | |
|---|---|---|---|---|---|
| USP17L17 | Ubiquitin Specific Peptidase 17 Like Family Member 17 | Protein Coding | 16 | GC04P009253 | 0.36266765 |
| USP17L20 | Ubiquitin Specific Peptidase 17 Like Family Member 20 | Protein Coding | 16 | GC04P009260 | 0.36266765 |
| USP17L11 | Ubiquitin Specific Peptidase 17 Like Family Member 11 | Protein Coding | 15 | GC04P009219 | 0.36266765 |
| USP17L19 | Ubiquitin Specific Peptidase 17 Like Family Member 19 | Protein Coding | 15 | GC04P009256 | 0.36266765 |
| USP17L21 | Ubiquitin Specific Peptidase 17 Like Family Member 21 | Protein Coding | 15 | GC04P009265 | 0.36266765 |
| PHB1P4 | PHB1 Pseudogene 4 | Pseudogene | 5 | GC02P154288 | 0.360191971 |
| PHB1P2 | PHB1 Pseudogene 2 | Pseudogene | 3 | GC11P043769 | 0.360191971 |
| ELSPBP1 | Epididymal Sperm Binding Protein 1 | Protein Coding | 29 | GC19P047994 | 0.356872499 |
| SLC16A14 | Solute Carrier Family 16 Member 14 | Protein Coding | 29 | GC02M230034 | 0.356872499 |
| ATP5MJ | ATP Synthase Membrane Subunit J | Protein Coding | 26 | GC14M103917 | 0.356872499 |
| GPALPP1 | GPALPP Motifs Containing 1 | Protein Coding | 23 | GC13P044989 | 0.356872499 |
| MIR3648-1 | MicroRNA 3648-1 | RNA Gene | 8 | GC21P011130 | 0.356872499 |
| SMCP | Sperm Mitochondria Associated Cysteine Rich Protein | Protein Coding | 29 | GC01P152878 | 0.35651654 |
| NANOS1 | Nanos C2HC-Type Zinc Finger 1 | Protein Coding | 34 | GC10P119029 | 0.35626632 |
| HIGD1B | HIG1 Hypoxia Inducible Domain Family Member 1B | Protein Coding | 26 | GC17P053244 | 0.35549286 |
| USP17L10 | Ubiquitin Specific Peptidase 17 Like Family Member 10 | Protein Coding | 17 | GC04P009215 | 0.354898274 |
| USP17L13 | Ubiquitin Specific Peptidase 17 Like Family Member 13 | Protein Coding | 16 | GC04P009230 | 0.354898274 |
| USP17L15 | Ubiquitin Specific Peptidase 17 Like Family Member 15 | Protein Coding | 15 | GC04P009237 | 0.354898274 |
| LOC649352 | Ubiquitin Carboxyl-Terminal Hydrolase 17-Like Protein 2-Like | Pseudogene | 4 | GC08M012658 | 0.354898274 |
| USP17L14P | Ubiquitin Specific Peptidase 17 Like Family Member 14, Pseudogene | Pseudogene | 4 | GC04P009232 | 0.354898274 |
| USP17L16P | Ubiquitin Specific Peptidase 17 Like Family Member 16, Pseudogene | Pseudogene | 4 | GC04P009251 | 0.354898274 |
| LOC100421094 | Ubiquitin Specific Peptidase 17 Like Family Member 2 Pseudogene | Pseudogene | 3 | GC08M012374 | 0.354898274 |
| LOC392187 | Ubiquitin Carboxyl-Terminal Hydrolase 17-Like Protein 2-Like | Pseudogene | 3 | GC08M007967 | 0.354898274 |
| LOC402329 | Ubiquitin Carboxyl-Terminal Hydrolase 17-Like Protein 2-Like | Pseudogene | 2 | GC08P007486 | 0.354898274 |
| EVI2A | Ecotropic Viral Integration Site 2A | Protein Coding | 31 | GC17M031316 | 0.354516983 |
| SPOPL | Speckle Type BTB/POZ Protein Like | Protein Coding | 31 | GC02P138501 | 0.354516983 |
| ABCA13 | ATP Binding Cassette Subfamily A Member 13 | Protein Coding | 34 | GC07P048171 | 0.353861988 |
| APOOL | Apolipoprotein O Like | Protein Coding | 30 | GC0XP085003 | 0.353861988 |
| CSNK1G2-AS1 | CSNK1G2 Antisense RNA 1 | RNA Gene | 18 | GC19M004945 | 0.353861988 |
| CRYZL1 | Crystallin Zeta Like 1 | Protein Coding | 32 | GC21M033589 | 0.352867097 |
| CFAP100 | Cilia And Flagella Associated Protein 100 | Protein Coding | 23 | GC03P126395 | 0.34952265 |
| EOLA2-DT | EOLA2 Divergent Transcript | RNA Gene | 13 | GC0XP149932 | 0.348256469 |
| MRPL3 | Mitochondrial Ribosomal Protein L3 | Protein Coding | 38 | GC03M131462 | 0.347795486 |
| APBB1IP | Amyloid Beta Precursor Protein Binding Family B Member 1 Interacting Protein | Protein Coding | 35 | GC10P026462 | 0.347795486 |
| ZSCAN29 | Zinc Finger And SCAN Domain Containing 29 | Protein Coding | 30 | GC15M043358 | 0.347795486 |
| ATXN7L2 | Ataxin 7 Like 2 | Protein Coding | 28 | GC01P109483 | 0.347795486 |
| PRXL2B | Peroxiredoxin Like 2B | Protein Coding | 25 | GC01P003673 | 0.347795486 |
| TPTE2P1 | Transmembrane Phosphoinositide 3-Phosphatase And Tensin Homolog 2 Pseudogene 1 | Pseudogene | 15 | GC13M024924 | 0.347795486 |
| MIR3654 | MicroRNA 3654 | RNA Gene | 10 | GC07M133053 | 0.347795486 |
| MIR3648-2 | MicroRNA 3648-2 | RNA Gene | 8 | GC21P008986 | 0.347795486 |
| IGHV3-69-1 | Immunoglobulin Heavy Variable 3-69-1 (Pseudogene) | Pseudogene | 6 | GC14M112552 | 0.347795486 |
| SLC22A6 | Solute Carrier Family 22 Member 6 | Protein Coding | 40 | GC11M087150 | 0.347189307 |
| CCDC168 | Coiled-Coil Domain Containing 168 | Protein Coding | 24 | GC13M102729 | 0.347189307 |
| SNAI3 | Snail Family Transcriptional Repressor 3 | Protein Coding | 32 | GC16M088677 | 0.346814781 |
| MIR193BHG | MIR193b-365a Host Gene | RNA Gene | 12 | GC16P014302 | 0.345941067 |
| CCDC182 | Coiled-Coil Domain Containing 182 | Protein Coding | 21 | GC17M057744 | 0.34468472 |
| CSHL1 | Chorionic Somatomammotropin Hormone Like 1 | Protein Coding | 31 | GC17M063909 | 0.344385535 |
| BTN2A2 | Butyrophilin Subfamily 2 Member A2 | Protein Coding | 34 | GC06P026382 | 0.342662275 |
| GPIHBP1 | Glycosylphosphatidylinositol Anchored High Density Lipoprotein Binding Protein 1 | Protein Coding | 35 | GC08P143213 | 0.341521353 |
| MSRB1 | Methionine Sulfoxide Reductase B1 | Protein Coding | 35 | GC16M006675 | 0.339485526 |
| CDADC1 | Cytidine And DCMP Deaminase Domain Containing 1 | Protein Coding | 30 | GC13P049247 | 0.337755322 |
| NXPH2 | Neurexophilin 2 | Protein Coding | 30 | GC02M138669 | 0.336900145 |
| SV2C | Synaptic Vesicle Glycoprotein 2C | Protein Coding | 35 | GC05P075847 | 0.33624512 |
| ESYT3 | Extended Synaptotagmin 3 | Protein Coding | 30 | GC03P138434 | 0.33624512 |
| CASTOR1 | Cytosolic Arginine Sensor For MTORC1 Subunit 1 | Protein Coding | 23 | GC22M036143 | 0.336104333 |
| IGFL4 | IGF Like Family Member 4 | Protein Coding | 27 | GC19M046039 | 0.336004794 |
| LETM2 | Leucine Zipper And EF-Hand Containing Transmembrane Protein 2 | Protein Coding | 31 | GC08P038433 | 0.329008758 |
| MCTS2 | MCTS Family Member 2 | Protein Coding | 13 | GC20P031774 | 0.327433944 |
| DTD1 | D-Aminoacyl-TRNA Deacylase 1 | Protein Coding | 31 | GC20P018587 | 0.326359212 |
| LCE1C | Late Cornified Envelope 1C | Protein Coding | 23 | GC01M152778 | 0.326359212 |
| MIR6754 | MicroRNA 6754 | RNA Gene | 7 | GC11P071473 | 0.326359212 |
| MYOZ1 | Myozenin 1 | Protein Coding | 32 | GC10M073631 | 0.325753033 |
| MGAT4EP | MGAT4 Family Member E, Pseudogene | Pseudogene | 12 | GC01P202820 | 0.324846476 |
| PNRC1 | Proline Rich Nuclear Receptor Coactivator 1 | Protein Coding | 31 | GC06P089080 | 0.3217116 |
| ATAD2B | ATPase Family AAA Domain Containing 2B | Protein Coding | 28 | GC02M023711 | 0.318387926 |
| MYPOP | Myb Related Transcription Factor, Partner Of Profilin | Protein Coding | 26 | GC19M045890 | 0.312841326 |
| MIR1206 | MicroRNA 1206 | RNA Gene | 12 | GC08P128008 | 0.312747091 |
| PGP | Phosphoglycolate Phosphatase | Protein Coding | 34 | GC16M006701 | 0.312441945 |
| SH3D21 | SH3 Domain Containing 21 | Protein Coding | 26 | GC01P036306 | 0.312441945 |
| ZPBP | Zona Pellucida Binding Protein | Protein Coding | 35 | GC07M049850 | 0.30559507 |
| KLHL1 | Kelch Like Family Member 1 | Protein Coding | 34 | GC13M069700 | 0.30559507 |
| SERP2 | Stress Associated Endoplasmic Reticulum Protein Family Member 2 | Protein Coding | 26 | GC13P044373 | 0.30559507 |
| WHSC1L2P | Wolf-Hirschhorn Syndrome Candidate 1-Like 2, Pseudogene | Pseudogene | 9 | GC17M043718 | 0.30559507 |
| MAPK10-AS1 | MAPK10 Antisense RNA 1 | RNA Gene | 10 | GC04P086079 | 0.302519947 |
| UGT2A2 | UDP Glucuronosyltransferase Family 2 Member A2 | Protein Coding | 26 | GC04M069589 | 0.300583959 |
| FPGT-TNNI3K | FPGT-TNNI3K Readthrough | Protein Coding | 19 | GC01P074198 | 0.299708784 |
| PMS2P5 | PMS1 Homolog 2, Mismatch Repair System Component Pseudogene 5 | Pseudogene | 17 | GC07P074890 | 0.298975617 |
| HMGA1P7 | High Mobility Group AT-Hook 1 Pseudogene 7 | Pseudogene | 10 | GC06M134114 | 0.298975617 |
| LOC730100 | Uncharacterized LOC730100 | RNA Gene | 10 | GC02P051033 | 0.297382116 |
| ANIB1 | Aneurysm, Intracranial Berry 1 | Genetic Locus | 4 | GC07U901527 | 0.297382116 |
| UGT2A1 | UDP Glucuronosyltransferase Family 2 Member A1 Complex Locus | Protein Coding | 30 | GC04M069588 | 0.295224458 |
| MAP3K9-DT | MAP3K9 Divergent Transcript | RNA Gene | 10 | GC14P070808 | 0.295224458 |
| PSG4 | Pregnancy Specific Beta-1-Glycoprotein 4 | Protein Coding | 32 | GC19M063880 | 0.293714285 |
| DEFB4B | Defensin Beta 4B | Protein Coding | 25 | GC08M007414 | 0.293714285 |
| ARMH3 | Armadillo Like Helical Domain Containing 3 | Protein Coding | 24 | GC10M101846 | 0.293714285 |
| FBXW10B | F-Box And WD Repeat Domain Containing 10B | Protein Coding | 22 | GC17M015567 | 0.293714285 |
| GUSBP4 | GUSB Pseudogene 4 | Pseudogene | 14 | GC06M064080 | 0.293714285 |
| CORO7 | Coronin 7 | Protein Coding | 31 | GC16M006874 | 0.289648354 |
| DHRS12 | Dehydrogenase/Reductase 12 | Protein Coding | 27 | GC13M051767 | 0.289071679 |
| OR2K2 | Olfactory Receptor Family 2 Subfamily K Member 2 | Protein Coding | 30 | GC09M111327 | 0.288312674 |
| ASPHD1 | Aspartate Beta-Hydroxylase Domain Containing 1 | Protein Coding | 28 | GC16P040197 | 0.288312674 |
| CCDC85A | Coiled-Coil Domain Containing 85A | Protein Coding | 27 | GC02P056184 | 0.288312674 |
| ZNF654 | Zinc Finger Protein 654 | Protein Coding | 27 | GC03P088059 | 0.288312674 |
| STARD4-AS1 | STARD4 Antisense RNA 1 | RNA Gene | 12 | GC05P111514 | 0.288312674 |

| | | | | | |
|---|---|---|---|---|---|
| ZNF20 | Zinc Finger Protein 20 | Protein Coding | 34 | GC19M012265 | 0.286477864 |
| LINC01359 | Long Intergenic Non-Protein Coding RNA 1359 | RNA Gene | 11 | GC01M064972 | 0.285986006 |
| LINC02026 | Long Intergenic Non-Protein Coding RNA 2026 | RNA Gene | 11 | GC03M193958 | 0.285986006 |
| LOC101927066 | Uncharacterized LOC101927066 | RNA Gene | 7 | GC08M096951 | 0.285986006 |
| LOC109461473 | BMP7 Promoter Region | Biological Region | 2 | GC20P057268 | 0.285986006 |
| AZGP1P2 | AZGP1 Pseudogene 2 | Pseudogene | 9 | GC07M101287 | 0.284903079 |
| ANKRD54 | Ankyrin Repeat Domain 54 | Protein Coding | 32 | GC22M056265 | 0.283909112 |
| NDUFB7 | NADH:Ubiquinone Oxidoreductase Subunit B7 | Protein Coding | 35 | GC19M014566 | 0.282431632 |
| UGGT2 | UDP-Glucose Glycoprotein Glucosyltransferase 2 | Protein Coding | 34 | GC13M095801 | 0.282431632 |
| LRRC10 | Leucine Rich Repeat Containing 10 | Protein Coding | 30 | GC12M069608 | 0.282431632 |
| IGLC7 | Immunoglobulin Lambda Constant 7 | Protein Coding | 15 | GC22P034652 | 0.27960819 |
| ERVK-11 | Endogenous Retrovirus Group K Member 11 | Protein Coding | 6 | GC03U901891 | 0.27935648 |
| MSRB2 | Methionine Sulfoxide Reductase B2 | Protein Coding | 36 | GC10P023095 | 0.279337823 |
| ZNF117 | Zinc Finger Protein 117 | Protein Coding | 28 | GC07M064971 | 0.275081754 |
| SSC4D | Scavenger Receptor Cysteine Rich Family Member With 4 Domains | Protein Coding | 27 | GC07M076727 | 0.275081754 |
| ZBTB9 | Zinc Finger And BTB Domain Containing 9 | Protein Coding | 28 | GC06P033453 | 0.274597406 |
| DERA | Deoxyribose-Phosphate Aldolase | Protein Coding | 34 | GC12P015911 | 0.27335462 |
| RNF32 | Ring Finger Protein 32 | Protein Coding | 32 | GC07P156640 | 0.27335462 |
| ZNF337 | Zinc Finger Protein 337 | Protein Coding | 29 | GC20M025814 | 0.27335462 |
| CFAP206 | Cilia And Flagella Associated Protein 206 | Protein Coding | 23 | GC06P087412 | 0.27335462 |
| GAL3ST3 | Galactose-3-O-Sulfotransferase 3 | Protein Coding | 31 | GC11M066040 | 0.27246812 |
| SYCP2L | Synaptonemal Complex Protein 2 Like | Protein Coding | 27 | GC06P010887 | 0.272254825 |
| TEX26 | Testis Expressed 26 | Protein Coding | 23 | GC13P030932 | 0.272254825 |
| FCAMR | Fc Alpha And Mu Receptor | Protein Coding | 31 | GC01M206957 | 0.26821208 |
| PLK5 | Polo Like Kinase 5 (Inactive) | Protein Coding | 26 | GC19P002618 | 0.266484916 |
| MCRIP1 | MAPK Regulated Corepressor Interacting Protein 1 | Protein Coding | 25 | GC17M081823 | 0.266484916 |
| ATP6V0E1 | ATPase H+ Transporting V0 Subunit E1 | Protein Coding | 36 | GC05P172983 | 0.263314426 |
| ATP6V0E2 | ATPase H+ Transporting V0 Subunit E2 | Protein Coding | 35 | GC07P149873 | 0.263314426 |
| PDXDC2P-NPIPB14P | PDXDC2P-NPIPB14P Readthrough | Pseudogene | 13 | GC16M070570 | 0.257177949 |
| MIR3667HG | MIR3667 Host Gene | RNA Gene | 12 | GC22M056215 | 0.257177949 |
| PTPRK-AS1 | PTPRK Antisense RNA 1 | RNA Gene | 10 | GC06P128029 | 0.257177949 |
| MIR4305 | MicroRNA 4305 | RNA Gene | 9 | GC13M039664 | 0.257177949 |
| APOBEC3D | Apolipoprotein B MRNA Editing Enzyme Catalytic Subunit 3D | Protein Coding | 32 | GC22P039021 | 0.256444752 |
| CCDC70 | Coiled-Coil Domain Containing 70 | Protein Coding | 27 | GC13P051861 | 0.255994767 |
| FCF1 | FCF1 RRNA-Processing Protein | Protein Coding | 29 | GC14P074713 | 0.254694194 |
| LINC01213 | Long Intergenic Non-Protein Coding RNA 1213 | RNA Gene | 11 | GC03P150238 | 0.254694194 |
| ANKRD20A9P | Ankyrin Repeat Domain 20 Family Member A9, Pseudogene | Pseudogene | 9 | GC13M020256 | 0.254694194 |
| LOC102724159 | Periodic Tryptophan Protein 2 Homolog | Protein Coding | 8 | GC21M005130 | 0.254694194 |
| CHRNA10 | Cholinergic Receptor Nicotinic Alpha 10 Subunit | Protein Coding | 38 | GC11M003781 | 0.251918316 |
| NLRP10 | NLR Family Pyrin Domain Containing 10 | Protein Coding | 34 | GC11M007959 | 0.251918316 |
| MED12L | Mediator Complex Subunit 12L | Protein Coding | 33 | GC03P151085 | 0.251918316 |
| DYDC1 | DPY30 Domain Containing 1 | Protein Coding | 31 | GC10M080336 | 0.251918316 |
| LOC107325936 | Origin Of Replication At Fragile Site FRA3B | Biological Region | 3 | GC03P060475 | 0.251918316 |
| LOC111162621 | DeltaNp63 Promoter Of Tumor Protein P63 | Biological Region | 2 | GC03P189786 | 0.248348981 |
| PLA2G4B | Phospholipase A2 Group IVB | Protein Coding | 35 | GC15P041837 | 0.245048642 |
| HMGB4 | High Mobility Group Box 4 | Protein Coding | 30 | GC01P033860 | 0.245048642 |
| AIDA | Axin Interactor, Dorsalization Associated | Protein Coding | 29 | GC01M223735 | 0.245048642 |
| PRG1 | P53-Responsive Gene 1 | RNA Gene | 11 | GC19P043349 | 0.245048642 |
| FAM89B | Family With Sequence Similarity 89 Member B | Protein Coding | 28 | GC11P065572 | 0.243947029 |
| LMNTD1 | Lamin Tail Domain Containing 1 | Protein Coding | 27 | GC12M025499 | 0.243947029 |
| ZYG11B | Zyg-11 Family Member B, Cell Cycle Regulator | Protein Coding | 29 | GC01P052726 | 0.238400429 |
| C1QTNF9B | C1q And TNF Related 9B | Protein Coding | 28 | GC13M023891 | 0.238400429 |
| LRRC63 | Leucine Rich Repeat Containing 63 | Protein Coding | 27 | GC13P046211 | 0.238400429 |
| THEGL | Theg Spermatid Protein Like | Protein Coding | 23 | GC04P056530 | 0.238400429 |
| PALD1 | Phosphatase Domain Containing Paladin 1 | Protein Coding | 27 | GC10P070835 | 0.232432127 |
| PCDHA1 | Protocadherin Alpha 1 | Protein Coding | 32 | GC05P145799 | 0.231530756 |
| CR1L | Complement C3b/C4b Receptor 1 Like | Protein Coding | 31 | GC01P207646 | 0.231530756 |
| TAS2R5 | Taste 2 Receptor Member 5 | Protein Coding | 30 | GC07P148230 | 0.231530756 |
| OR2A4 | Olfactory Receptor Family 2 Subfamily A Member 4 | Protein Coding | 29 | GC06M131700 | 0.231530756 |
| PCDHGA11 | Protocadherin Gamma Subfamily A, 11 | Protein Coding | 28 | GC05P145845 | 0.231530756 |
| PSG11 | Pregnancy Specific Beta-1-Glycoprotein 11 | Protein Coding | 28 | GC19M063874 | 0.231530756 |
| NUP62CL | Nucleoporin 62 C-Terminal Like | Protein Coding | 27 | GC0XM107123 | 0.231530756 |
| ZFP69 | ZFP69 Zinc Finger Protein | Protein Coding | 27 | GC01P040477 | 0.231530756 |
| FAM163B | Family With Sequence Similarity 163 Member B | Protein Coding | 25 | GC09M133577 | 0.231530756 |
| NPIPA7 | Nuclear Pore Complex Interacting Protein Family Member A7 | Protein Coding | 20 | GC16P017336 | 0.231530756 |
| OBSCN-AS1 | OBSCN Antisense RNA 1 | RNA Gene | 15 | GC01M228205 | 0.231530756 |
| WASH8P | WAS Protein Family Homolog 8, Pseudogene | Pseudogene | 9 | GC12M000015 | 0.231530756 |
| RPSAP11 | Ribosomal Protein SA Pseudogene 11 | Pseudogene | 7 | GC03P032350 | 0.223434985 |
| FAM161B | FAM161 Centrosomal Protein B | Protein Coding | 30 | GC14M073931 | 0.220783591 |
| FAM32A | Family With Sequence Similarity 32 Member A | Protein Coding | 29 | GC19P063080 | 0.219640434 |
| FMO1 | Flavin Containing Dimethylaniline Monoxygenase 1 | Protein Coding | 39 | GC01P171248 | 0.217154294 |
| WBP1 | WW Domain Binding Protein 1 | Protein Coding | 30 | GC02P074458 | 0.216021895 |
| FN3KRP | Fructosamine 3 Kinase Related Protein | Protein Coding | 34 | GC17P082716 | 0.215057373 |
| FAT3 | FAT Atypical Cadherin 3 | Protein Coding | 31 | GC11P092224 | 0.215057373 |
| ZNF182 | Zinc Finger Protein 182 | Protein Coding | 32 | GC0XM047974 | 0.213913903 |
| TAC4 | Tachykinin Precursor 4 | Protein Coding | 29 | GC17M049838 | 0.213913903 |
| PCDHGA6 | Protocadherin Gamma Subfamily A, 6 | Protein Coding | 28 | GC05P145837 | 0.213913903 |
| ZNF554 | Zinc Finger Protein 554 | Protein Coding | 28 | GC19P002821 | 0.213913903 |
| ASB14 | Ankyrin Repeat And SOCS Box Containing 14 | Protein Coding | 27 | GC03M057278 | 0.213913903 |
| METTL15P1 | Methyltransferase Like 15 Pseudogene 1 | Pseudogene | 13 | GC03M156711 | 0.213913903 |
| GLUD1P3 | Glutamate Dehydrogenase 1 Pseudogene 3 | Pseudogene | 11 | GC10P073730 | 0.213913903 |
| MMADHC-DT | MMADHC Divergent Transcript | RNA Gene | 10 | GC02P149590 | 0.213913903 |
| RPL21P19 | Ribosomal Protein L21 Pseudogene 19 | Pseudogene | 10 | GC01M204491 | 0.213913903 |
| GTF2IP20 | General Transcription Factor IIi Pseudogene 20 | Pseudogene | 9 | GC01M224614 | 0.213913903 |
| DUSP8P5 | Dual Specificity Phosphatase 8 Pseudogene 5 | Pseudogene | 8 | GC10P073731 | 0.213913903 |
| RPL28P1 | Ribosomal Protein L28 Pseudogene 1 | Pseudogene | 5 | GC03M170653 | 0.213913903 |
| PCSK4 | Proprotein Convertase Subtilisin/Kexin Type 4 | Protein Coding | 35 | GC19M001481 | 0.206677511 |
| C17orf64 | Chromosome 17 Open Reading Frame 64 | Protein Coding | 27 | GC17P060393 | 0.205818132 |
| RPSAP12 | Ribosomal Protein SA Pseudogene 12 | Pseudogene | 10 | GC12M068553 | 0.205818132 |
| FRA11B | Fragile Site, Folic Acid Type, Rare, Fra(11)(Q23.3) | Biological Region | 4 | GC11P120079 | 0.205818132 |
| RPSAP52 | Ribosomal Protein SA Pseudogene 52 | Pseudogene | 14 | GC12M065758 | 0.200523257 |
| ZPBP2 | Zona Pellucida Binding Protein 2 | Protein Coding | 31 | GC17P053042 | 0.196904719 |
| ATP23 | ATP23 Metallopeptidase And ATP Synthase Assembly Factor Homolog | Protein Coding | 27 | GC12P057942 | 0.191960752 |
| LOC110467516 | CYP1A2 5' Regulatory Region | Biological Region | 2 | GC15P074744 | 0.191960752 |
| SWI5 | SWI5 Homologous Recombination Repair Protein | Protein Coding | 27 | GC09P128275 | 0.191611007 |

| | | | | | |
|---|---|---|---|---|---|
| LCMT1 | Leucine Carboxyl Methyltransferase 1 | Protein Coding | 32 | GC16P027801 | 0.190415859 |
| SIAH3 | Siah E3 Ubiquitin Protein Ligase Family Member 3 | Protein Coding | 28 | GC13M045777 | 0.190415859 |
| MORF4 | Mortality Factor 4 (Pseudogene) | Pseudogene | 14 | GC04M173615 | 0.189127147 |
| LOC111162620 | TAp63 Promoter Of Tumor Protein P63 | Biological Region | 2 | GC03P189630 | 0.189127147 |
| SLAIN1 | SLAIN Motif Family Member 1 | Protein Coding | 32 | GC13P077697 | 0.183514059 |
| ZNF124 | Zinc Finger Protein 124 | Protein Coding | 30 | GC01M247121 | 0.183514059 |
| EPOP | Elongin BC And Polycomb Repressive Complex 2 Associated Protein | Protein Coding | 21 | GC17M041425 | 0.183514059 |
| SNORD111B | Small Nucleolar RNA, C/D Box 111B | RNA Gene | 15 | GC16P071455 | 0.183514059 |
| GPR18 | G Protein-Coupled Receptor 18 | Protein Coding | 35 | GC13M099254 | 0.183263838 |
| SPATA13 | Spermatogenesis Associated 13 | Protein Coding | 33 | GC13P024016 | 0.183263838 |
| NAA16 | N-Alpha-Acetyltransferase 16, NatA Auxiliary Subunit | Protein Coding | 28 | GC13P041311 | 0.183263838 |
| SERPINE3 | Serpin Family E Member 3 | Protein Coding | 26 | GC13P051336 | 0.183263838 |
| PRR20A | Proline Rich 20A | Protein Coding | 21 | GC13P057140 | 0.183263838 |
| PRR20C | Proline Rich 20C | Protein Coding | 19 | GC13P057141 | 0.183263838 |
| LETM1P1 | Leucine Zipper And EF-Hand Containing Transmembrane Protein 1 Pseudogene 1 | Pseudogene | 7 | GC15P114656 | 0.183263838 |
| NATP | N-Acetyltransferase Pseudogene | Pseudogene | 7 | GC08P018369 | 0.183263838 |
| MPEG1 | Macrophage Expressed 1 | Protein Coding | 31 | GC11M059208 | 0.181920588 |
| IGHV4-31 | Immunoglobulin Heavy Variable 4-31 | Protein Coding | 15 | GC14M112561 | 0.17621693 |
| PDE1A | Phosphodiesterase 1A | Protein Coding | 38 | GC02M182140 | 0.175609261 |
| CLEC17A | C-Type Lectin Domain Containing 17A | Protein Coding | 27 | GC19P014582 | 0.175609261 |
| HTR5BP | 5-Hydroxytryptamine Receptor 5B, Pseudogene | Pseudogene | 11 | GC02P117859 | 0.175609261 |
| LOC107880064 | IVL Promoter Region | Biological Region | 2 | GC01P152906 | 0.175609261 |
| SLC13A1 | Solute Carrier Family 13 Member 1 | Protein Coding | 35 | GC07M123113 | 0.174186826 |
| JMJD7 | Jumonji Domain Containing 7 | Protein Coding | 29 | GC15P041829 | 0.174186826 |
| ZNF177 | Zinc Finger Protein 177 | Protein Coding | 28 | GC19P009363 | 0.174186826 |
| CLEC2B | C-Type Lectin Domain Family 2 Member B | Protein Coding | 32 | GC12M020436 | 0.170524478 |
| NUDT16 | Nudix Hydrolase 16 | Protein Coding | 30 | GC03P131381 | 0.168447554 |
| QTRT2 | Queuine TRNA-Ribosyltransferase Accessory Subunit 2 | Protein Coding | 27 | GC03P114006 | 0.168447554 |
| LNCTAM34A | Long Non Coding Transcriptional Activator Of MiR34a | RNA Gene | 11 | GC01P009183 | 0.168447554 |
| NUDT3 | Nudix Hydrolase 3 | Protein Coding | 33 | GC06M063742 | 0.164146662 |
| ABHD13 | Abhydrolase Domain Containing 13 | Protein Coding | 31 | GC13P108218 | 0.164146662 |
| ADPRHL1 | ADP-Ribosylhydrolase Like 1 | Protein Coding | 31 | GC13M113421 | 0.164146662 |
| TEX30 | Testis Expressed 30 | Protein Coding | 29 | GC13M102765 | 0.164146662 |
| ZNF747 | Zinc Finger Protein 747 | Protein Coding | 28 | GC16M030530 | 0.164146662 |
| KAAG1 | Kidney Associated DCDC2 Antisense RNA 1 | RNA Gene | 25 | GC06P024356 | 0.164146662 |
| S100A7L2 | S100 Calcium Binding Protein A7 Like 2 | Pseudogene | 23 | GC01M153436 | 0.164146662 |
| PRR20E | Proline Rich 20E | Protein Coding | 18 | GC13P057167 | 0.164146662 |
| PRR20B | Proline Rich 20B | Protein Coding | 17 | GC13P057147 | 0.164146662 |
| PRR20D | Proline Rich 20D | Protein Coding | 17 | GC13P057160 | 0.164146662 |
| PRDX2P1 | Peroxiredoxin 2 Pseudogene 1 | Pseudogene | 7 | GC13P030296 | 0.164146662 |
| FKBP2 | FKBP Prolyl Isomerase 2 | Protein Coding | 35 | GC11P064354 | 0.1601004 |
| SLC44A5 | Solute Carrier Family 44 Member 5 | Protein Coding | 31 | GC01M075202 | 0.1601004 |
| SHISA2 | Shisa Family Member 2 | Protein Coding | 30 | GC13M026044 | 0.1601004 |
| TRNAU1AP | TRNA Selenocysteine 1 Associated Protein 1 | Protein Coding | 30 | GC01P028553 | 0.1601004 |
| C9orf64 | Chromosome 9 Open Reading Frame 64 | Protein Coding | 28 | GC09M083938 | 0.1601004 |
| GGACT | Gamma-Glutamylamine Cyclotransferase | Protein Coding | 28 | GC13M100530 | 0.1601004 |
| KCTD4 | Potassium Channel Tetramerization Domain Containing 4 | Protein Coding | 28 | GC13M045192 | 0.1601004 |
| TTC9C | Tetratricopeptide Repeat Domain 9C | Protein Coding | 27 | GC11P062728 | 0.1601004 |
| C16orf87 | Chromosome 16 Open Reading Frame 87 | Protein Coding | 25 | GC16M046796 | 0.1601004 |
| LOC122394733 | Thymosin Beta 15B-Like | Protein Coding | 4 | GC0XM104072 | 0.1601004 |
| MIR1470 | MicroRNA 1470 | RNA Gene | 10 | GC19P015779 | 0.157992393 |
| IGKV2-26 | Immunoglobulin Kappa Variable 2-26 (Pseudogene) | Pseudogene | 7 | GC02M090828 | 0.157992393 |
| LOC284685 | EWS RNA Binding Protein 1 Pseudogene | Pseudogene | 4 | GC01M166276 | 0.157992393 |
| RNF19B | Ring Finger Protein 19B | Protein Coding | 31 | GC01M033153 | 0.152750552 |
| KBTBD6 | Kelch Repeat And BTB Domain Containing 6 | Protein Coding | 28 | GC13M041127 | 0.152750552 |
| PCDHGA4 | Protocadherin Gamma Subfamily A, 4 | Protein Coding | 27 | GC05P145833 | 0.152750552 |
| GOLGA8DP | Golgin A8 Family Member D, Pseudogene | Pseudogene | 12 | GC15P039766 | 0.152750552 |
| CEBPG | CCAAT Enhancer Binding Protein Gamma | Protein Coding | 32 | GC19P033373 | 0.151023388 |
| ANGEL2 | Angel Homolog 2 | Protein Coding | 31 | GC01M212992 | 0.151023388 |
| ETNPPL | Ethanolamine-Phosphate Phospho-Lyase | Protein Coding | 31 | GC04M108742 | 0.151023388 |
| FAM124A | Family With Sequence Similarity 124 Member A | Protein Coding | 31 | GC13P051222 | 0.151023388 |
| PLGRKT | Plasminogen Receptor With A C-Terminal Lysine | Protein Coding | 28 | GC09M005357 | 0.151023388 |
| RIBC2 | RIB43A Domain With Coiled-Coils 2 | Protein Coding | 27 | GC22P045413 | 0.151023388 |
| CCDC24 | Coiled-Coil Domain Containing 24 | Protein Coding | 26 | GC01P043991 | 0.151023388 |
| CBY2 | Chibby Family Member 2 | Protein Coding | 25 | GC13P045703 | 0.151023388 |
| CLHC1 | Clathrin Heavy Chain Linker Domain Containing 1 | Protein Coding | 25 | GC02M055172 | 0.151023388 |
| PPP1R17 | Protein Phosphatase 1 Regulatory Subunit 17 | Protein Coding | 33 | GC07P031726 | 0.140983209 |
| ASB6 | Ankyrin Repeat And SOCS Box Containing 6 | Protein Coding | 31 | GC09M129942 | 0.140983209 |
| IGFN1 | Immunoglobulin Like And Fibronectin Type III Domain Containing 1 | Protein Coding | 31 | GC01P201159 | 0.140983209 |
| METTL21C | Methyltransferase 21C, AARS1 Lysine | Protein Coding | 29 | GC13M102685 | 0.140983209 |
| NOL12 | Nucleolar Protein 12 | Protein Coding | 28 | GC22P038342 | 0.140983209 |
| ANKMY2 | Ankyrin Repeat And MYND Domain Containing 2 | Protein Coding | 27 | GC07M016599 | 0.140983209 |
| TIGD5 | Tigger Transposable Element Derived 5 | Protein Coding | 26 | GC08P143597 | 0.140983209 |
| SMIM2 | Small Integral Membrane Protein 2 | Protein Coding | 20 | GC13M044143 | 0.140983209 |
| EEF1DP3 | Eukaryotic Translation Elongation Factor 1 Delta Pseudogene 3 | Pseudogene | 17 | GC13P031846 | 0.140983209 |
| SDHDP3 | Succinate Dehydrogenase Complex Subunit D Pseudogene 3 | Pseudogene | 6 | GC03P169709 | 0.140983209 |
| PGM5 | Phosphoglucomutase 5 | Protein Coding | 34 | GC09P068328 | 0.139389738 |
| PAQR9 | Progestin And AdipoQ Receptor Family Member 9 | Protein Coding | 28 | GC03M142949 | 0.139232665 |
| RELL2 | RELT Like 2 | Protein Coding | 28 | GC05P141636 | 0.139232665 |
| TMEM169 | Transmembrane Protein 169 | Protein Coding | 28 | GC02P216081 | 0.139232665 |
| C14orf119 | Chromosome 14 Open Reading Frame 119 | Protein Coding | 27 | GC14P031858 | 0.139232665 |
| IFI27L1 | Interferon Alpha Inducible Protein 27 Like 1 | Protein Coding | 26 | GC14P094081 | 0.139232665 |
| IGFL2 | IGF Like Family Member 2 | Protein Coding | 26 | GC19P046078 | 0.139232665 |
| LINC00479 | Long Intergenic Non-Protein Coding RNA 479 | RNA Gene | 16 | GC21M041711 | 0.139232665 |
| N4BP2L2-IT2 | N4BPL2 Intronic Transcript 2 | RNA Gene | 11 | GC13M036307 | 0.139232665 |
| TSSC2 | Tumor Suppressing Subtransferable Candidate 2 (Pseudogene) | Pseudogene | 11 | GC11P003650 | 0.139232665 |
| PFN1P2 | Profilin 1 Pseudogene 2 | Pseudogene | 10 | GC01M120432 | 0.139232665 |
| RPL23AP5 | Ribosomal Protein L23a Pseudogene 5 | Pseudogene | 10 | GC16P010632 | 0.139232665 |
| RPS17P16 | Ribosomal Protein S17 Pseudogene 16 | Pseudogene | 10 | GC22P032039 | 0.139232665 |
| ATF4P3 | Activating Transcription Factor 4 Pseudogene 3 | Pseudogene | 9 | GC17M076225 | 0.139232665 |
| RHEBP1 | RHEB Pseudogene 1 | Pseudogene | 8 | GC10M046634 | 0.139232665 |
| ATF4P4 | Activating Transcription Factor 4 Pseudogene 4 | Pseudogene | 7 | GC11P113939 | 0.139232665 |
| IGFL1P1 | IGF Like Family Member 1 Pseudogene 1 | Pseudogene | 7 | GC19P046196 | 0.139232665 |
| IGFL1P2 | IGF Like Family Member 1 Pseudogene 2 | Pseudogene | 6 | GC19M046260 | 0.139232665 |
| MICOS10P1 | MICOS10 Pseudogene 1 | Pseudogene | 6 | GC13M036393 | 0.139232665 |

| | | | | | |
|---|---|---|---|---|---|
| ACOT2 | Acyl-CoA Thioesterase 2 | Protein Coding | 32 | GC14P073567 | 0.129587099 |
| ZNF707 | Zinc Finger Protein 707 | Protein Coding | 30 | GC08P143684 | 0.129587099 |
| BPIFB4 | BPI Fold Containing Family B Member 4 | Protein Coding | 28 | GC20P033079 | 0.129587099 |
| CTAGE9 | CTAGE Family Member 9 | Protein Coding | 20 | GC06M131708 | 0.129587099 |
| NACA4P | NACA Family Member 4, Pseudogene | Pseudogene | 14 | GC08P101362 | 0.129587099 |
| CSPG5 | Chondroitin Sulfate Proteoglycan 5 | Protein Coding | 35 | GC03M047562 | 0.121615797 |
| AAVS1 | Adeno-Associated Virus Integration Site 1 | Genetic Locus | 6 | GC19U990001 | 0.121615797 |
| MS4A3 | Membrane Spanning 4-Domains A3 | Protein Coding | 34 | GC11P060057 | 0.116069213 |
| ACTR6 | Actin Related Protein 6 | Protein Coding | 30 | GC12P100200 | 0.116069213 |
| NHLRC3 | NHL Repeat Containing 3 | Protein Coding | 28 | GC13P039038 | 0.116069213 |
| ACOD1 | Aconitate Decarboxylase 1 | Protein Coding | 25 | GC13P076949 | 0.116069213 |
| SERTM1 | Serine Rich And Transmembrane Domain Containing 1 | Protein Coding | 25 | GC13P036673 | 0.116069213 |
| ATP11AUN | ATP11A Upstream Neighbor LncRNA | RNA Gene | 20 | GC13P112647 | 0.116069213 |
| SPART-AS1 | SPART Antisense RNA 1 | RNA Gene | 14 | GC13P036347 | 0.116069213 |
| C13orf46 | Chromosome 13 Open Reading Frame 46 | Protein Coding | 13 | GC13M113954 | 0.116069213 |
| METTL21EP | Methyltransferase Like 21E, Pseudogene | Pseudogene | 13 | GC13P102880 | 0.116069213 |
| UBE2L5 | Ubiquitin Conjugating Enzyme E2 L5 | Protein Coding | 13 | GC13P030534 | 0.116069213 |
| CFAP97D2 | CFAP97 Domain Containing 2 | Protein Coding | 11 | GC13P114172 | 0.116069213 |
| LOC647264 | Keratin-Associated Protein 21-1 | Protein Coding | 11 | GC13M063833 | 0.116069213 |
| LINC01149 | Long Intergenic Non-Protein Coding RNA 1149 | RNA Gene | 10 | GC06P080346 | 0.116069213 |
| ENSG00000273167 | Novel Transcript | Protein Coding | 7 | GC13P023979 | 0.116069213 |
| ENSG00000281883 | Novel Transcript | Protein Coding | 7 | GC13P043883 | 0.116069213 |
| LOC100129307 | Putative UPF0607 Protein ENSP00000383144 | Pseudogene | 7 | GC13M077661 | 0.116069213 |
| LOC101928697 | Uncharacterized LOC101928697 | RNA Gene | 7 | GC13P019182 | 0.116069213 |
| LOC112267897 | Keratin-Associated Protein 21-1-Like | Protein Coding | 7 | GC13P063747 | 0.116069213 |
| ENSG00000283208 | Novel Protein | Protein Coding | 6 | GC13P076992 | 0.116069213 |
| ENSG00000285444 | Novel Transcript | Protein Coding | 6 | GC13M051763 | 0.116069213 |
| ZNF962P | Zinc Finger Protein 962, Pseudogene | Pseudogene | 5 | GC13M018467 | 0.116069213 |
| FRA2H | Fragile Site, Aphidicolin Type, Common, Fra(2)(Q32.1) | Uncategorized | 4 | GC02U990036 | 0.116069213 |
| CCT5-2P | Chaperonin Containing TCP1, Subunit 5 (Epsilon) Pseudogene 2 | Pseudogene | 3 | GC13M078910 | 0.116069213 |
| LOC107105350 | NUP98-HOXC11 Recombination Region | Biological Region | 2 | GC12P053974 | 0.116069213 |
| HERC3 | HECT And RLD Domain Containing E3 Ubiquitin Protein Ligase 3 | Protein Coding | 34 | GC04P088560 | 0.098452359 |
| RFPL2 | Ret Finger Protein Like 2 | Protein Coding | 30 | GC22M032190 | 0.098452359 |
| ZSCAN30 | Zinc Finger And SCAN Domain Containing 30 | Protein Coding | 25 | GC18M035251 | 0.098452359 |
| CFAP299 | Cilia And Flagella Associated Protein 299 | Protein Coding | 24 | GC04P080336 | 0.098452359 |
| TMEM121B | Transmembrane Protein 121B | Protein Coding | 23 | GC22M017119 | 0.098452359 |
| NPIPB13 | Nuclear Pore Complex Interacting Protein Family, Member B13 | Protein Coding | 15 | GC16M030222 | 0.098452359 |
| FAM224A | Family With Sequence Similarity 224 Member A | RNA Gene | 11 | GC0YP018326 | 0.098452359 |
| INO80B-WBP1 | INO80B-WBP1 Readthrough (NMD Candidate) | RNA Gene | 11 | GC02P074455 | 0.098452359 |
| MIR3134 | MicroRNA 3134 | RNA Gene | 10 | GC03M015697 | 0.098452359 |
| MIR3650 | MicroRNA 3650 | RNA Gene | 9 | GC05M038558 | 0.098452359 |
| MIR5587 | MicroRNA 5587 | RNA Gene | 9 | GC16P000535 | 0.098452359 |
| PTP4A2P1 | PTP4A2 Pseudogene 1 | Pseudogene | 7 | GC17P042533 | 0.098452359 |
| DEPDC1P2 | DEP Domain Containing 1 Pseudogene 2 | Pseudogene | 6 | GC09P105758 | 0.098452359 |
| S100A7P1 | S100 Calcium Binding Protein A7 Pseudogene 1 | Pseudogene | 6 | GC01M153400 | 0.098452359 |
| S100A7P2 | S100 Calcium Binding Protein A7 Pseudogene 2 | Pseudogene | 4 | GC01P153444 | 0.098452359 |
| LOC111089942 | Pleiotrophin Promoter | Biological Region | 2 | GC07P137343 | 0.098452359 |
| MENAQ3 | Menarche, Age At, QTL3 | Genetic Locus | 2 | GC09U901344 | 0.098452359 |
