# Supplementary material for: Exploring the potential of Huangqin Tang in breast cancer treatment using network pharmacological analysis and experimental verification
Source: BMC Complement Med Ther. 2024 Jun 7;24:221. doi: 10.1186/s12906-024-04523-0 (PMC11161988; doi:10.1186/s12906-024-04523-0)
Supplement: Supplementary file 4 — Supplementary Material 4 [file 12906_2024_4523_MOESM4_ESM.pdf]

HQT-BC intersection targets

MMP2	CRP	ADIPOQ	AKR1C3	IGF2	CASP3	RXRB	CHEK1
XDH	FN1	NOS2	F3	NPEPPS	FOSL2	BCL2L1	SELE
CYP1A1	NR1I3	CCNB1	NR1I2	IL2	ELK1	CAV1	THBD
HTR3A	TNF	PRKCB	PYGM	ABAT	IFNG	CXCL11	MAPK14
CHRM1	IL1A	BCL2	AR	HSF1	CACNA1S	RASSF1	STAT3
GSTP1	SPP1	F7	FOSL1	ACP3	SLC2A4	ICAM1	SOAT1
ADRA1A	PTGS2	MAPK10	CD40LG	VCAM1	MAP2	TOP2A	ACHE
LDLR	ADH1C	AHR	CES1	PTGS1	NOS3	CASP8	PTGER3
NCF1	HAS2	NFE2L2	CYP3A4	BAX	MTTP	CTRB1	IRF1
CCNA2	CYP2C9	CHRM4	DCAF5	MMP9	CXCL8	SLC6A4	RXRA
MT-ND6	RUNX1T1	OLR1	PCOLCE	GOT1	CHUK	CLDN4	NFATC1
GSR	ESR1	VEGFA	PPP3CA	BIRC5	SLPI	KCNH2	PRKCD
IL10	PDE3A	MYC	ADRA2A	PSMD3	ADRB2	E2F1	MPO
EGFR	STAT1	GSK3B	ADRB1	PRKCA	CTSD	APOB	EGLN1
NKX3-1	MAPK1	MMP1	EGF	SCN5A	ADRA2B	PPARG	INSR
ERBB2	PLAT	PPARD	CTNNB1	TOP1	HK2	PKIA	MAPK3
PGR	IKBKB	MMP3	CCND1	ATP5F1B	CASP7	TP53	OPRM1
RUNX2	HSD3B2	CXCL2	POR	AKT1	MCL1	NCOA2	SERPINE1
ODC1	APOD	NCOA1	CD14	CYP2B6	IL6	IL1B	HSPB1
E2F2	MAPK8	CHEK2	HMGCR	CCL2	FASLG	MMP10	CYCS
RASA1	CASP9	PDE10A	NR3C2	KDR	CYP1A2	ESR2	CACNA2D1
CHRM3	AKR1C1	CDC37	HMOX1	PPARA	AHSA1	CYP1B1	
IGFBP3	CAT	CXCL10	TEP1	RAF1	ERBB3	PLAU	
SLC6A2	ABCC1	CDKN1A	GSTM1	ADRA1B	OPRD1	GJA1	
EIF6	TDRD7	DIO1	IL4	HSD3B1	SLC6A3	FASN	
ACACA	SREBF1	NFKBIA	NOX5	HIF1A	LTA4H	LBP	
PON1	GSTM2	SOAT2	RB1	RELA	ADRA2C	DUOX2	
