# Supplementary material for: Exploring the potential of Huangqin Tang in breast cancer treatment using network pharmacological analysis and experimental verification
Source: BMC Complement Med Ther. 2024 Jun 7;24:221. doi: 10.1186/s12906-024-04523-0 (PMC11161988; doi:10.1186/s12906-024-04523-0)

Fig.5 (D)

MCF-7

HIF-1α (93 kDa)

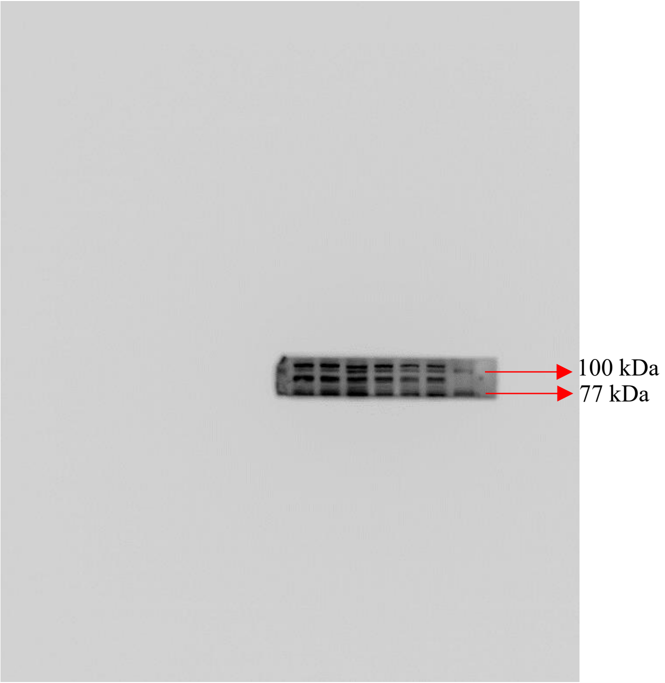

GAPDH (36 kDa)

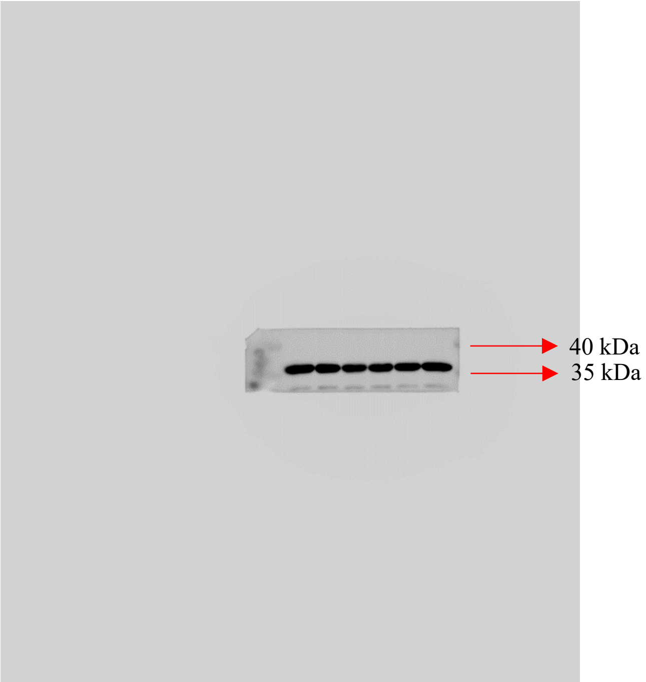

MDA-MB-231

HIF-1α (93 kDa)

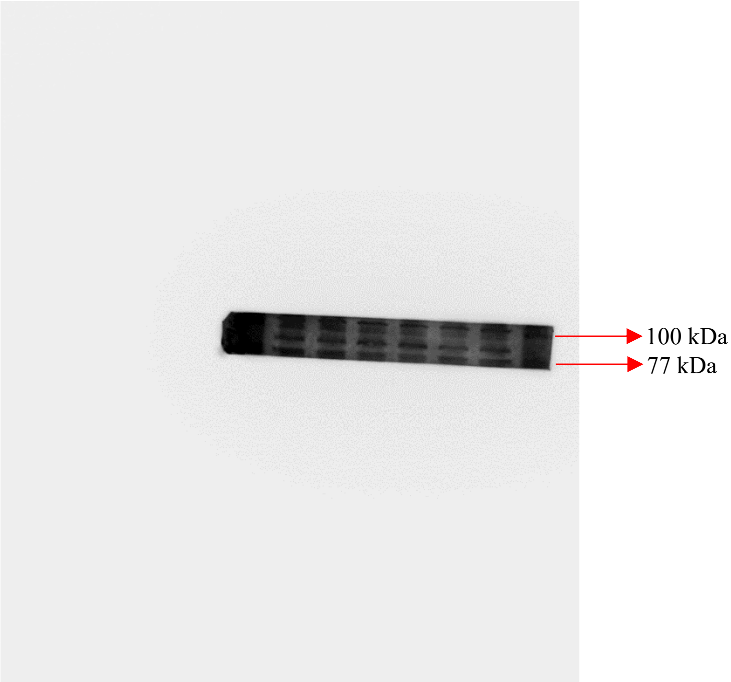

GAPDH (36 kDa)

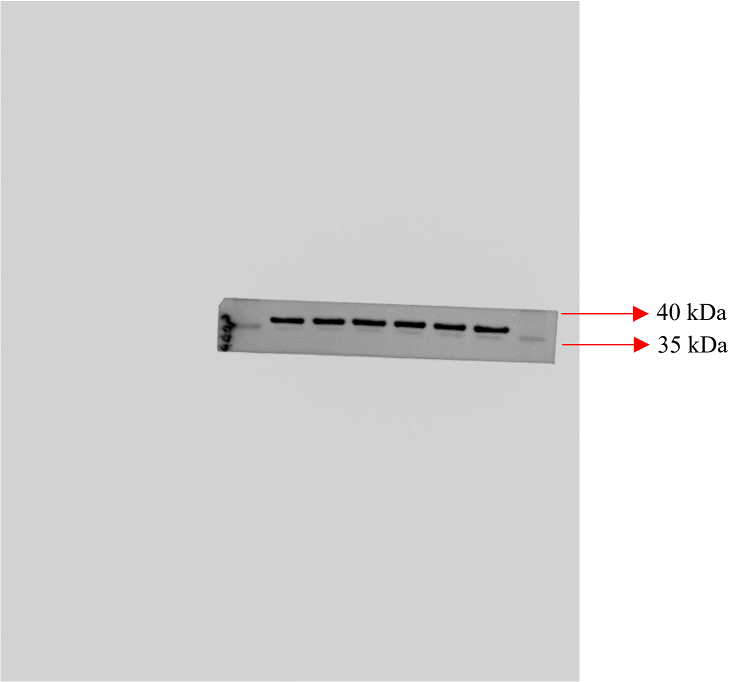

Fig.6

(C)

MCF-7

CDK1 (34 kDa)

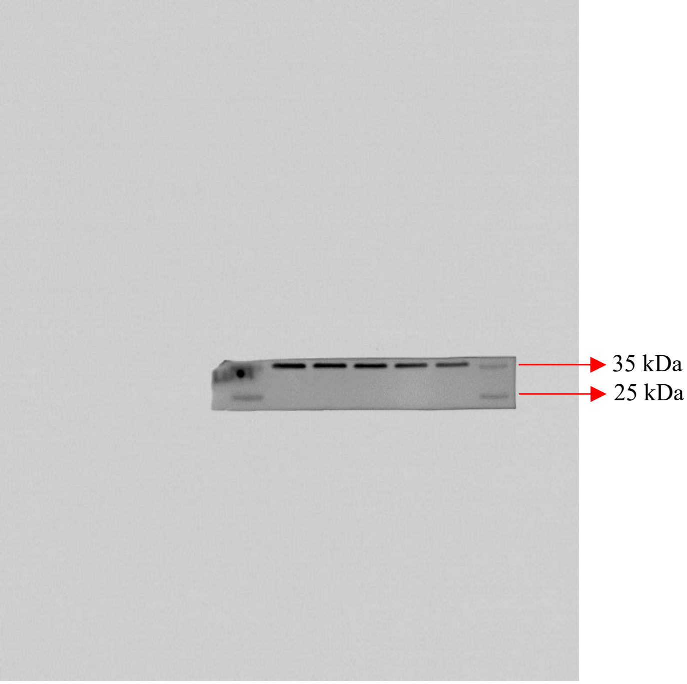

Cyclin B1 (60 kDa)

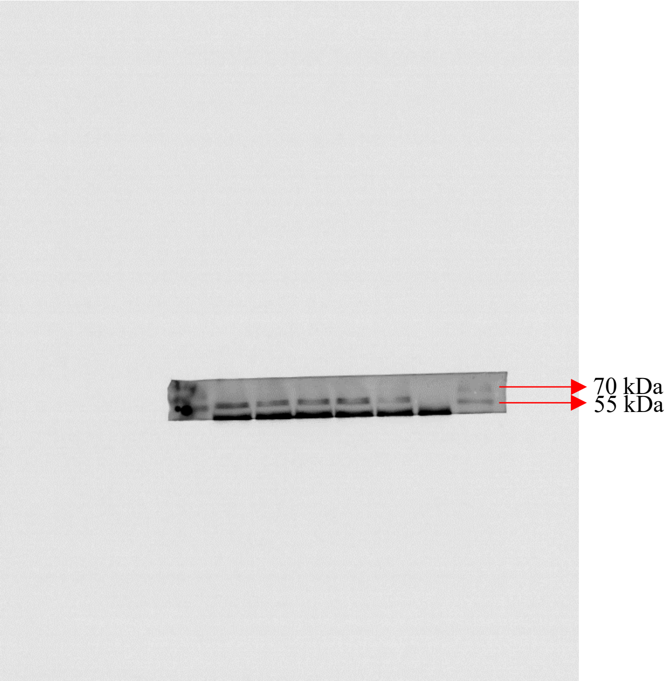

CDK2 (34 kDa)

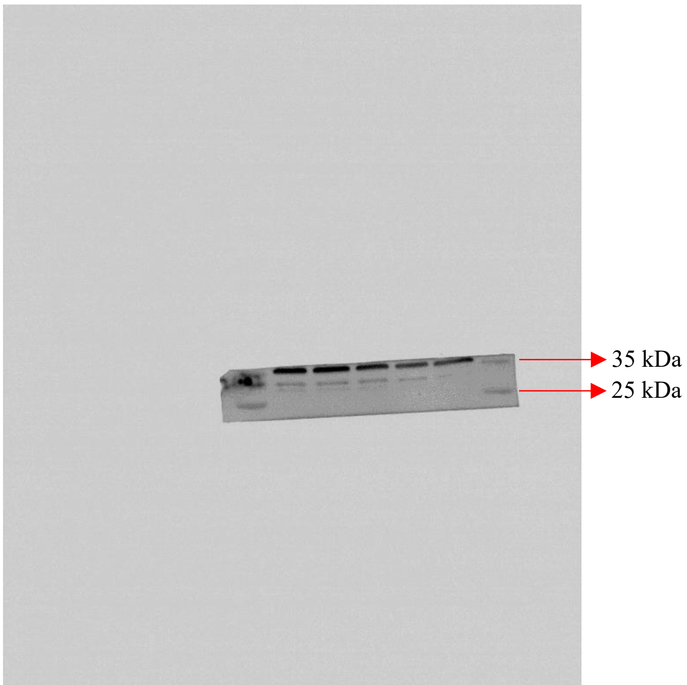

Cyclin E (50 kDa)

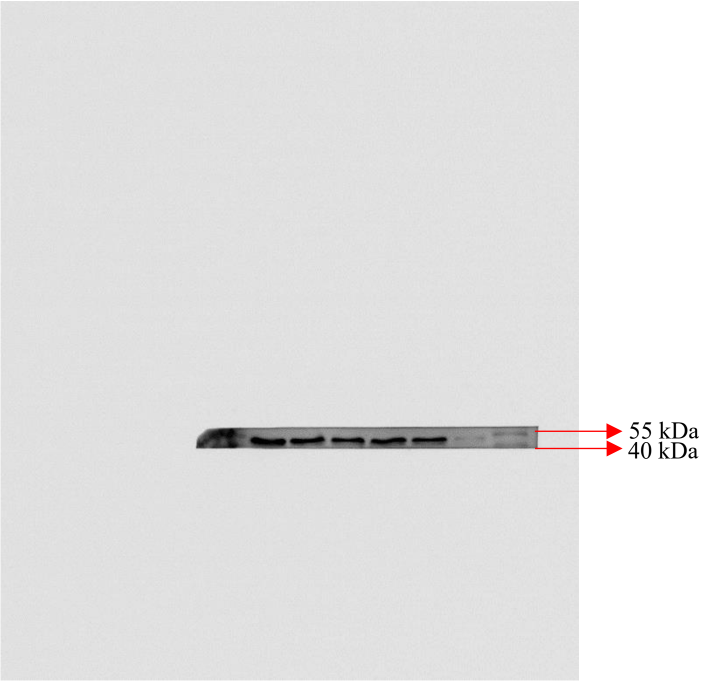

β-actin (43 kDa)

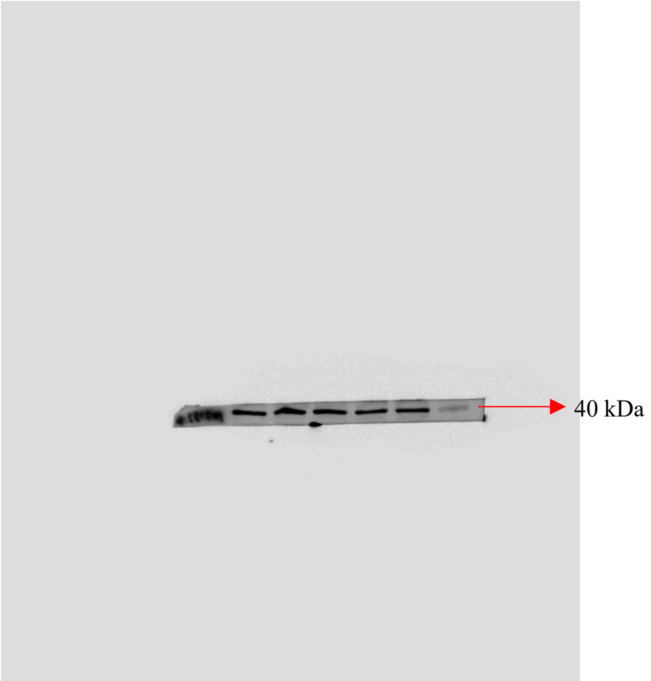

(D)

MDA-MB-231

CDK1 (34 kDa)

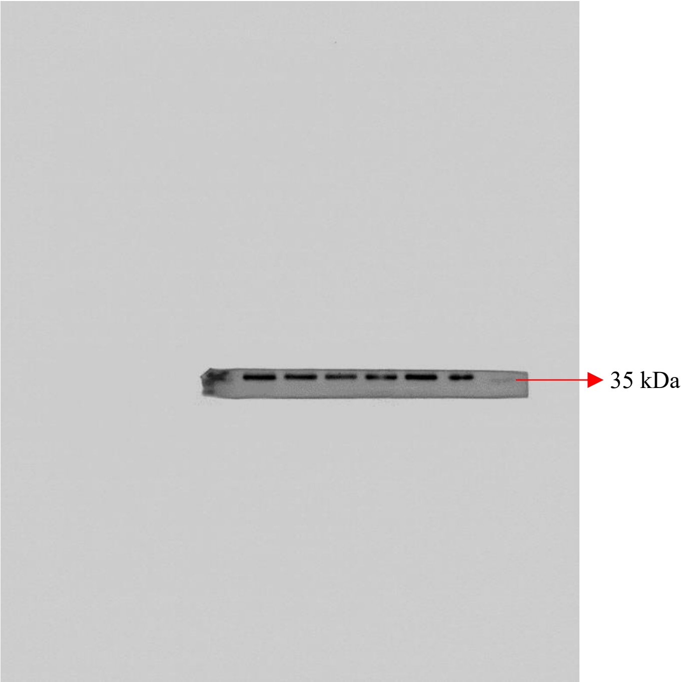

Cyclin B1 (60 kDa)

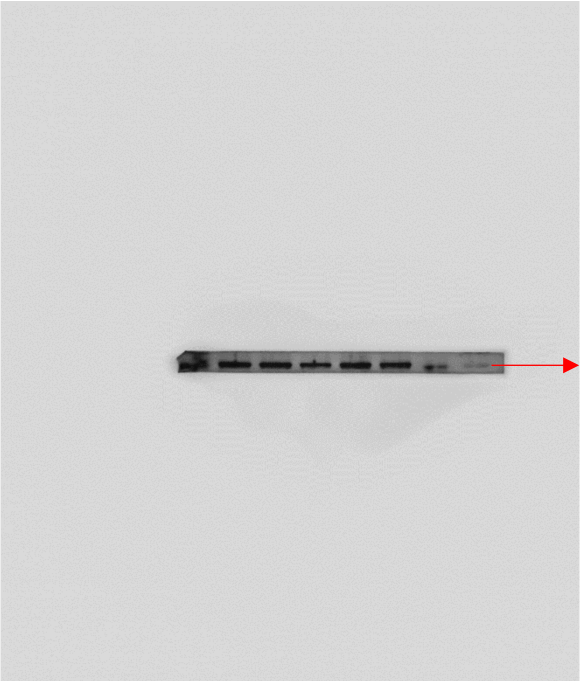

55 kDa

CDK2 (34 kDa)

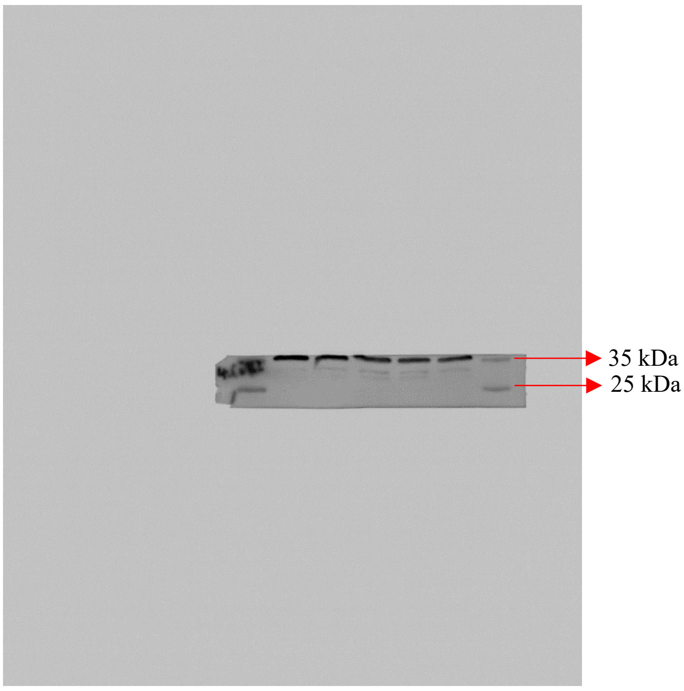

35 kDa
25 kDa

Cyclin E (50 kDa)

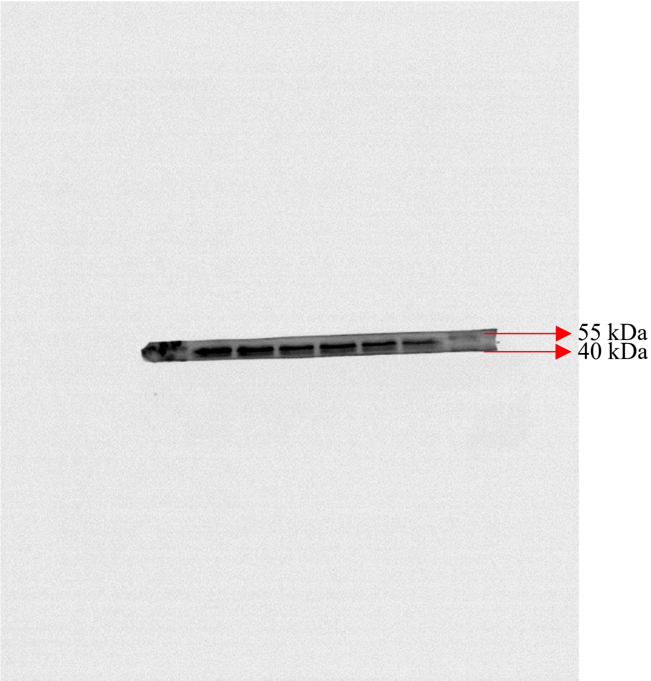

β-actin (43 kDa)

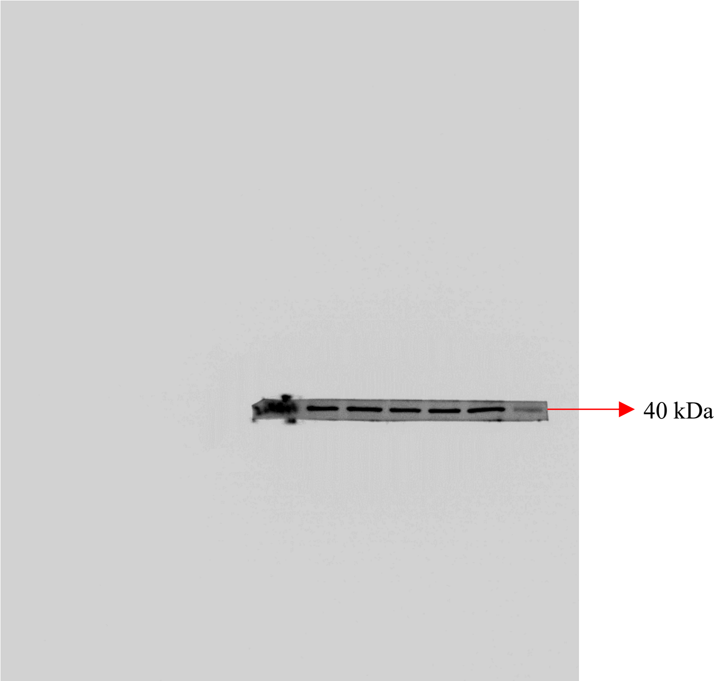

Fig.7

(C)

MCF-7

Bak (25 kDa)

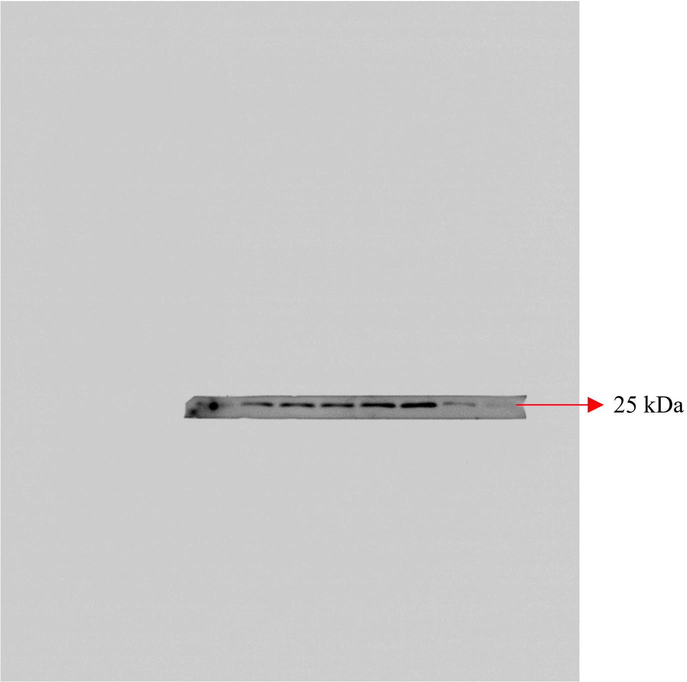

Bcl-2 (26 kDa)

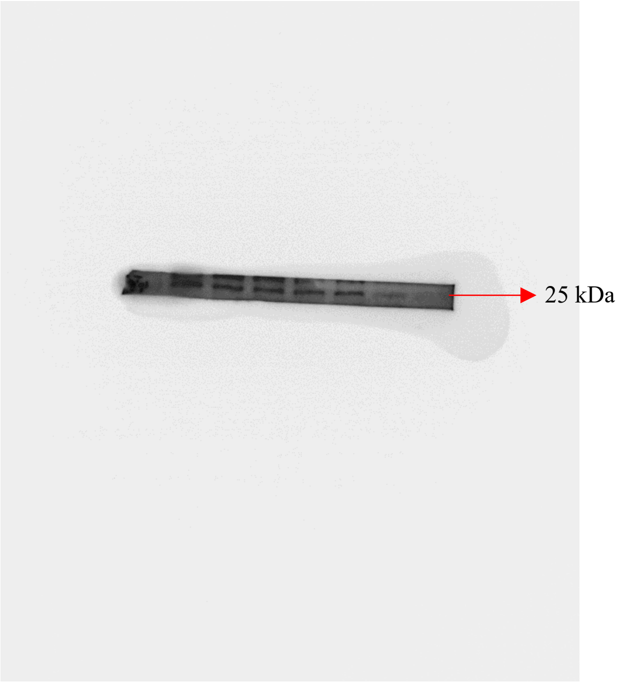

CC-3 (17 kDa)

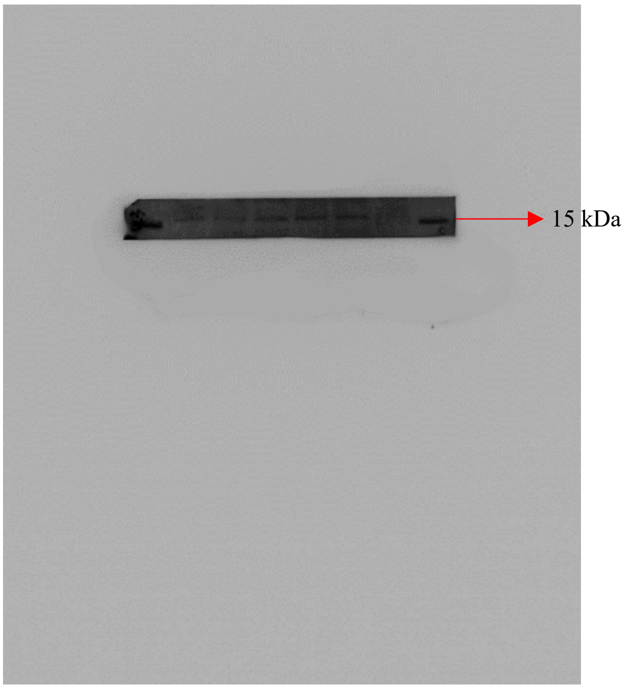

β-actin (43 kDa)

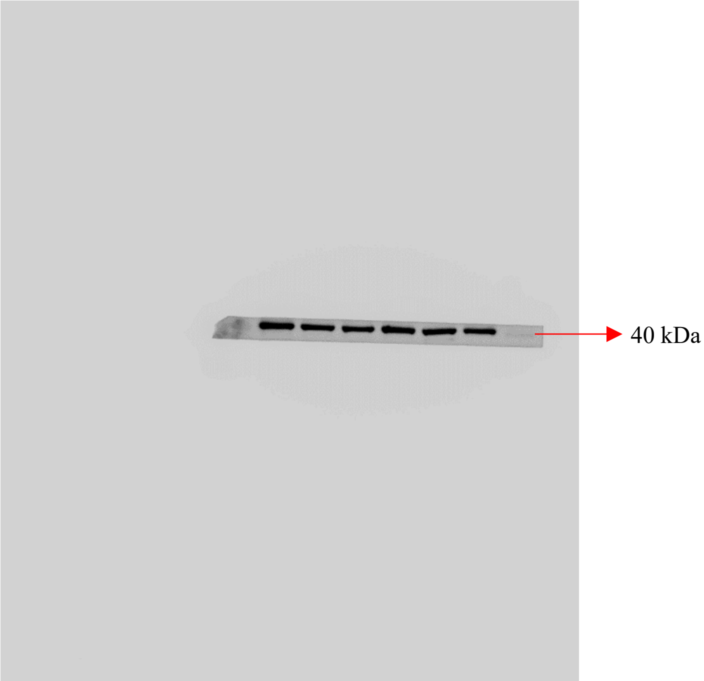

(D)

MDA-MB-231

Bak (25 kDa)

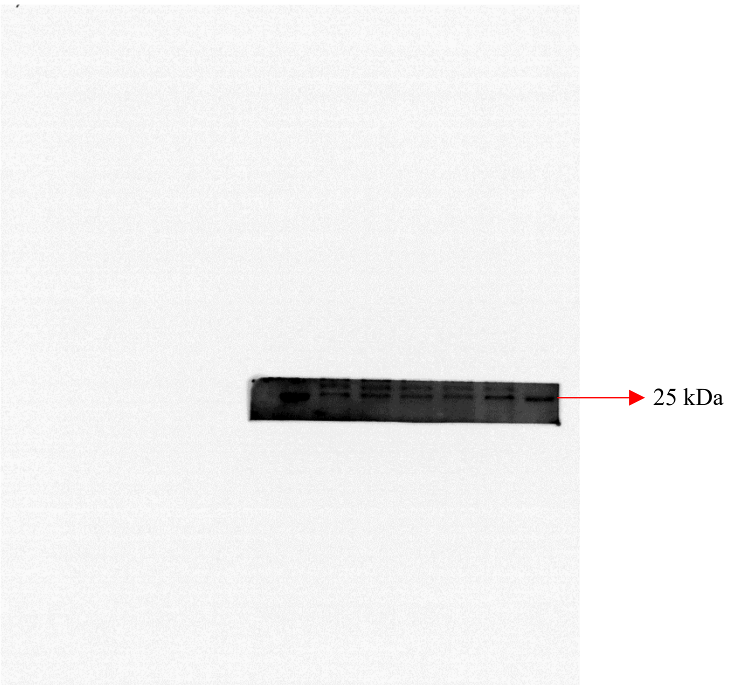

Bcl-2 (26 kDa)

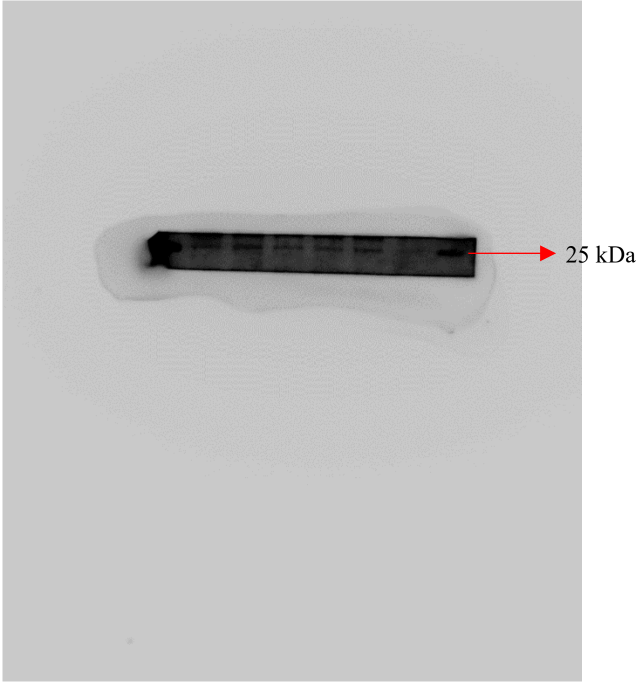

CC-3 (17 kDa)

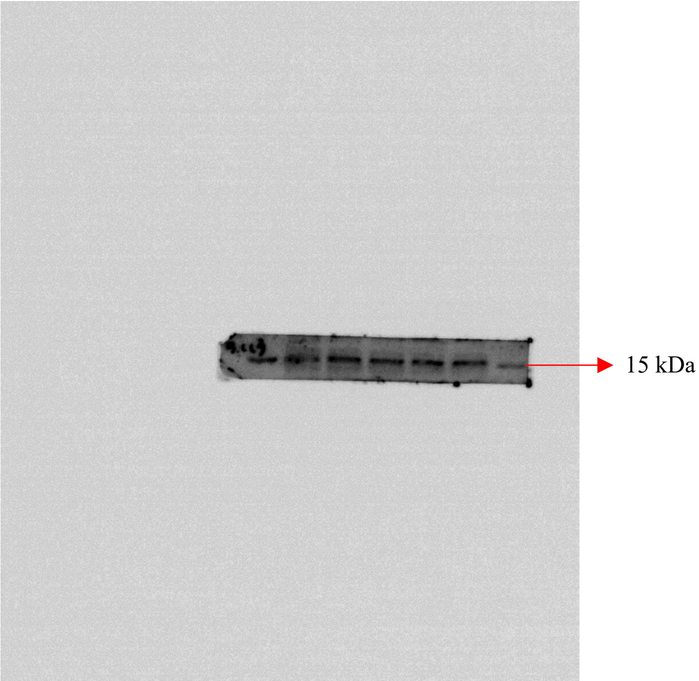

β-actin (43 kDa)

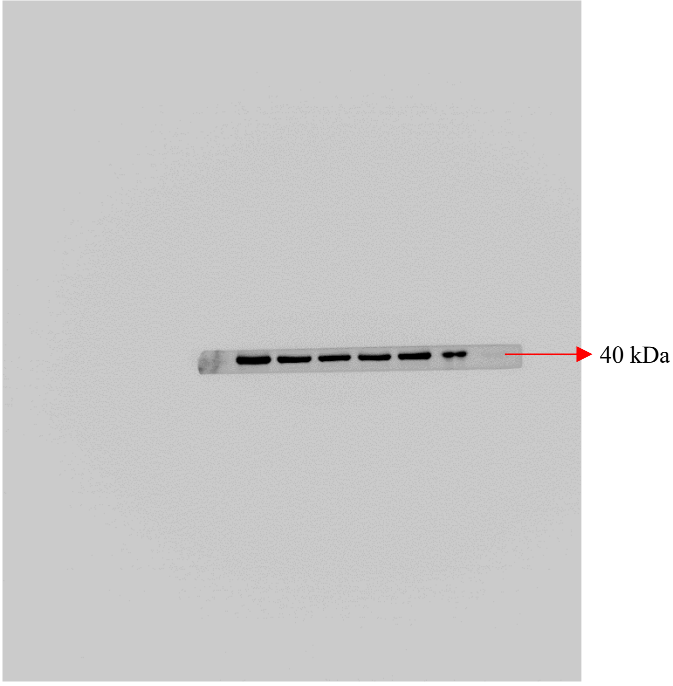

Supplement: Supplementary file 5 — Supplementary Material 5 [file 12906_2024_4523_MOESM5_ESM.pdf]
